# In Silico Genome-Wide Analysis of Respiratory Burst Oxidase Homolog (RBOH) Family Genes in Five Fruit-Producing Trees, and Potential Functional Analysis on Lignification of Stone Cells in Chinese White Pear

**DOI:** 10.3390/cells8060520

**Published:** 2019-05-29

**Authors:** Xi Cheng, Guohui Li, Muhammad Aamir Manzoor, Han Wang, Muhammad Abdullah, Xueqiang Su, Jingyun Zhang, Taoshan Jiang, Qing Jin, Yongping Cai, Yi Lin

**Affiliations:** 1 School of Life Science, Anhui Agricultural University, No. 130, Changjiang West Road, Hefei 230036, China; chengxi90@ahau.edu.cn (X.C.); xh16720349@ahau.edu.cn (G.L.); alamsher@ahau.edu.cn (M.A.M.); skywh@ahau.edu.cn (H.W.); abdullahpadana@ahau.edu.cn (M.A.); 15003011@ahau.edu.cn (X.S.); jts2015@ahau.edu.cn (T.J.); qingjin@ahau.edu.cn (Q.J.); 2 Horticultural Institute, Anhui Academy of Agricultural Sciences, Hefei, Anhui 230031, China; 16112378@ahau.edu.cn

**Keywords:** pear, respiratory burst oxidase homolog family, ROS, lignification, phylogenetic and expression analysis, subcellular localization

## Abstract

The accumulation of lignin in fruit has a significant negative impact on the quality of fruit-producing trees, and in particular the lignin formation stimulates the development of stone cells in pear fruit. Reactive oxygen species (ROS) are essential for lignin polymerization. However, knowledge of the *RBOH* family, a key enzyme in ROS metabolism, remains unknown in most fruit trees. In this study, a total of 40 *RBOH*s were identified from five fruit-producing trees (*Pyrus bretschneideri*, *Prunus persica*, *Citrus sinensis*, *Vitis vinifera*, and *Prunus mume*), and 10 of these sequences came from *Pyrus bretschneideri*. Multiple sequence alignments revealed that all 10 PbRBOHs contained the NADPH_Ox domain and the six alpha-helical transmembrane domains (TM-I to TM-VI). Chromosome localization and interspecies phylogenetic tree analysis showed that 10 PbRBOHs irregularly distributed on 8 chromosomes and 3 PbRBOHs (PbRBOHA, PbRBOHB, and PbRBOHD) are closely related to known lignification-related RBOHs. Furthermore, hormone response pattern analysis showed that the transcription of *PbRBOH*s is regulated by SA, ABA and MeJA. Reverse transcription-quantitative real-time polymerase chain reaction (qRT-PCR) and transcriptome sequencing analysis showed that *PbRBOHA*, *PbRBOHB*, and *PbRBOHD* accumulated high transcript abundance in pear fruit, and the transcriptional trends of *PbRBOHA* and *PbRBOHD* was consistent with the change of stone cell content during fruit development. In addition, subcellular localization revealed that PbRBOHA and PbRBOHD are distributed on the plasma membrane. Combining the changes of apoplastic superoxide (O_2_.^−^) content and spatio-temporal expression analysis, these results indicate that *PbRBOHA* and *PbRBOHD*, which are candidate genes, may play an important role in ROS metabolism during the lignification of pear stone cells. This study not only provided insight into the molecular characteristics of the *RBOH* family in fruit-producing trees, but also lays the foundation for studying the role of ROS in plant lignification.

## 1. Introduction

*Pyrus* spp. is one of the world’s most important deciduous fruit trees. Asiatic pears or Oriental pears are mainly distributed in East Asia, and the main cultivars include *P. bretschneideri*, *P. sinkiangensis*, *P. sinkiangensis*, *P. ussuriensis* and *P. pyrifolia* [1,2,3]. Among them, *Pyrus bretschneideri* cv. Dangshan Su is the largest diploid (2*n* = 34) pear species in China. However, ‘Dangshan Su’ has the defect of a high content of large-diameter stone cell cluster in the fruit, which seriously affects its quality and economic value [4,5,6]. In addition, the large diameter and high content of stone cell clusters are also the “common problems” of some *P. ussuriensis* and *P. pyrifolia* varieties [1]. Therefore, reducing the size and content of the stone cell cluster of pear fruit and improving the fruit quality is an urgent problem to be solved in the pear industry.

Stone cells (also known as sclereids) exist in the xylem, phloem, leaves, fruits and seeds of trees, with support and protection functions [7,8]. The stone cells of pears accumulate specifically in the fruit, which is formed by the thickening and lignification of the secondary cell wall of the parenchyma cells [9,10,11]. The multiple aggregated stone cells are composed of stone cell clusters. At present, a large number of studies have shown that the size, content and density of stone cell clusters not only have a significant negative impact on the texture of pear fruit, but also have a close relationship with its sucrose content [6,12,13,14].

The average content of lignin in pear stone cells is approximately 37%, and the highest is approximately 44% [15]. Pear stone cells can not only undergo lignin-specific color reaction with phloroglucinol-HCl (Wiesner staining), but also have autofluorescence [16,17]. Transmission electron microscopy (TEM) observations revealed a large amount of lignin deposition in the cell corners, compound middle lamellas and secondary cell walls of pear stone cells [18,19]. It can be seen that lignin is one of the key components of pear stone cells, and the biosynthesis of lignin is closely related to stone cell development [20,21]. It is worth noting that lignin synthesis not only has a negative impact on the texture of pear fruit, but also has a significant effect on the intrinsic quality of peach (*Prunus persica*), loquat (*Eriobotrya japonica*), pomegranate (*Punica granatum*), sweet orange (*Citrus sinensis*), and other fruit trees [22,23,24,25,26,27]. Therefore, studying lignin metabolism is important for fruit-producing trees.

The polymerization mechanism of lignin monomers is still unclear. Previous studies have speculated that lignin monomer-coupled polymeric proteins mainly include dirigent (DIR), peroxidase (POD), laccase (LAC), superoxide dismutase (SOD) and nicotinamide adenine dinucleotide phosphate (NADPH) oxidases [8]. Notably, reactive oxygen species (ROS) play an important role in the lignification of plant cells [8,28,29,30,31]. Plant NADPH oxidase, also known as respiratory burst oxidase homolog (RBOH) [28], is a homolog of the mammalian macrophage NADPH oxidase catalytic subunit gp91^phox^ [8,32]. It catalyzes the production of O_2_.^−^, NADP^+^ and H^+^ by O_2_ and NADPH. Subsequent O_2_.^−^ can be catalyzed as a substrate for SOD (EC 1.15.1.1) to form hydrogen peroxide (H_2_O_2_) and molecular oxygen (O_2_). H_2_O_2_ and O_2_ participate in the polymerization of lignin monomers as a second substrate for POD and LAC, respectively [8,28,29]. Finally, POD and LAC activate the lignin monomer radicals, resulting in a terminal polymerization of the free radical oligo/monomers with the extension units, and finally the polymerization is coupled to form a long chain of lignin polymer [8,28,33]. Currently, although the functional study of RBOH in biotic and abiotic stress is not uncommon in the field of stress biology, the role of RBOH in plant lignin synthesis and cell wall development has not been well-investigated until now. In addition, the *DIR*, *POD*, *LAC*, and *SOD* families related to lignin polymerization in pears have been identified [4,34,35,36], but the study of the *RBOH* family of pears has not been reported.

To this end, we identified the *RBOH* family at the genome-wide level of five fruit-producing trees, *Pyrus bretschneideri*, *Prunus persica*, *Citrus sinensis*, *Vitis vinifera*, and *Prunus mume*, and systematically analyzed molecular evolution, chromosomal localization, microsynteny, conserved domains, gene structures and other aspects. Secondly, through phylogenetic tree clustering, gene expression profiling and determination of reactive oxygen species, the lignification-related RBOHs in pears were screened and subcellular localization analysis was performed. This study not only clarified the molecular characteristics of the five fruit-producing tree *RBOH* families, but also laid the foundation for the later study of lignification of pear stone cells and improvement of fruit texture. 

## 2. Materials and Methods

### 2.1. Collection and Identification of Respiratory Burst Oxidase Homologs (RBOHs) in Five Fruit-Producing Trees

*Pyrus bretschneideri* (Chinese white pear) genome files were downloaded from the GigaDB data (http://gigadb.org/dataset/100083). The genome databases of *Prunus persica* (peach) and *Vitis vinifera* (common grape vine) were obtained from the Phytozome database (https://phytozome.jgi.doe.gov/pz/portal.html). *Prunus mume* (mei) genome files were downloaded from the Genome Database for Rosaceae (GDR) (http://www.rosaceae.org/). *Citrus sinensis* (sweet orange) genome files were downloaded from the *Citrus sinensis* Annotation Project (CAP) (http://citrus.hzau.edu.cn/orange/download/index.php).

Using the amino acid sequences of ten Arabidopsis *RBOH* family members [37] as the query sequences, candidate *RBOH*s were screened in the genome database using the BLASTp program (*p*-value = 10^−10^) in BioEdit v7.0.9.0 [38]. After removing repeating and redundant sequences, Pfam protein families database 32.0 (http://pfam.sanger.ac.uk/search) [39] and the Simple Modular Architecture Research Tool (http://smart.embl-heidelberg.de/) [40] were used to identify whether the candidate sequence contained the NADPH_Ox (PF08414) domain. The properties of the sequence of each RBOH [including amino acid sequence length (aa), molecular weight (MW), isoelectric point (pI) and subcellular localization] were completed by the ExPASy-Compute pI/Mw tool (http://web.expasy.org/compute_pi/) and WoLF PSORT (http://www.genscript.com/wolf-psort.html). Amino acid multiple sequence alignments were performed using BioEdit v7.0.9.0. The three-dimensional structures of RBOHs were confirmed using the PHYRE2 Protein Fold Recognition Server [41].

### 2.2. Conserved Motif, Gene Structure and Evolution Analysis

The conserved motifs and gene structures of the *RBOH* families were performed using Gene Structure Display Server (http://gsds.cbi.pku.edu.cn/) and Multiple Em for Motif Elicitation (http://meme-suite.org/), respectively [42,43,44,45].

The species phylogenetic tree was obtained from Common Taxonomy Tree (https://www.ncbi.nlm.nih.gov/Taxonomy/CommonTree/wwwcmt.cgi). All phylogenetic trees in this study were completed using MEGA v. 5.1 software. The construction parameters are as follows: Statistical Method: Neighbor-joining; Test of phylogeny: Bootstrap method; No. of Bootstrap Replications: 1000; Model: *p*-distance; Gaps: Pairwise deletion.

### 2.3. RBOH Family Genes’ Physical Localization and Gene Duplications

Visualization of the chromosomal location of the *RBOH*s was done using MapInspect v.1 software [46,47]. The identification of tandem duplication and segmental duplication events is based on the method used as previously described [16]. 

### 2.4. Microsynteny and Cis-Acting Elements Analysis of RBOH Family Genes

Microsynteny analysis was completed by using the Plant Genome Duplication Database and Circos v. 0.69 software (http://circos.ca/software/download/circos/) [4,48].

A sequence of 1500 bp upstream of the start codon of each *PbRBOH* coding region (CDS) was extracted from the pear genome as a putative promoter. The identification of the type and distribution of *cis*-acting elements was carried out using the software PLANT CARE v. 1 (http://bioinformatics.psb.ugent.be/webtools/plantcare/html/).

### 2.5. Plant Materials and Treatments

The annual stem segments, buds, flowers, mature leaves and fruits were sampled from 40-year-old *Pyrus bretschneideri* cv. Dangshan Su trees were grown in the orchard of Dangshan County, Anhui Province, China. We collected fruits at eight developmental stages: 15 days after flowering (DAF), 39 DAF, 47 DAF, 63 DAF, 79 DAF, 102 DAF and 145 DAF (mature period). For exogenous hormone treatment of pear fruit, pear trees with no pests and diseases, with the same age and plant height, were selected. Hormones were sprayed on the entire surface of the pear fruit using a sprayer at 39 DAF. The concentration of the hormone treatment [0.5 mmol/L abscisic acid (ABA), 0.5 mmol/L methyl jasmonate (MeJA), or 0.2 mmol/L salicylic acid (SA)] was determined according to Cheng et al. (2018 and 2019) [4,5]. Samples were collected at 0, 1, 2, and 3 h post-treatment (HPT), and pear fruits of uniform size were harvested at each time point and immediately frozen in liquid nitrogen. 

### 2.6. Determination of Apoplastic Superoxide (O_2_.^−^) Content in Pear Fruit

Histochemical staining with nitroblue tetrazolium (NBT) was used to analyse the in situ accumulation of O_2_.^−^ according to Huang et al. (2011) [49]. Apoplastic superoxide was measured using superoxide anion free radical detection kit (sulfonamide colorimetry) following the manufacturer’s instructions (Beijing Leagene Biotech Co. Ltd., Beijing, China).

### 2.7. RNA Extraction and Reverse Transcription-Quantitative Real-Time Polymerase Chain Reaction (RT-qPCR) Analysis

RNA extraction and reverse transcription were performed according to the instructions in the Plant RNA maxi kit (Biomiga, San Diego, CA, USA) and the EasyScript One-Step gDNA Removal and cDNA Synthesis SuperMix (TransGen Biotech Co., Beijing, China), respectively. Reverse transcription-quantitative real-time polymerase chain reaction (RT-qPCR) analysis was performed using TransStart Probe qPCR SuperMix (TransGen Biotech Co.) and detected using a CFX96 Touch™ Real-Time PCR Detection System (Bio-rad Laboratories, Inc., Singapore, Singapore). Relative expression levels were calculated using the 2^−△△Ct^ method [50]. All RT-qPCR primers are listed in Table S1. In this study, *Tubulin* (accession No. AB239680.1) was used as an internal reference [4]. The transcriptome sequencing data have been deposited in the Sequence Read Archive (https://www.ncbi.nlm.nih.gov/sra) under accession numbers SUB2967341 [6].

### 2.8. Subcellular Localization of PbRBOHs

The specific primers (Table S1) were designed to amplify the *PbRBOH* (*PbRBOHA* and *PbRBOHD*) CDS, and two restriction sites (*Xba* I, *Sma* I) were introduced at both ends according to the information provided for the eukaryotic expression vector pCAMBIA1305-GFP. By enzyme digestion and ligation, the plant expression vectors pCAMBIA1304-*PbRBOHA/D*-*GFP* were obtained. The vector was transformed into *Agrobacterium tumefaciens* EHA105 by electroporation, and 4-6-week-old *Nicotiana benthamiana* were used for the subcellular localization analysis. The method of subcellular localization was based on Cheng et al. (2017) [16]. 

## 3. Results

### 3.1. Identification, Characterization and Genomic Distribution of RBOH Family Genes in Five Fruit-Producing Trees

BLASTp was performed in the genome database of five fruit-producing trees using the amino acid sequence of AtRBOHA~J as the query sequence [51]. After deleting the repeat sequence and the redundant sequence, it was finally determined that there were 10 *RBOH*s in Chinese white pear (*PbRBOH*), and 7 *RBOH*s in peach (*PpRBOH*), 8 *RBOH*s in mei (*PmRBOH*), 7 *RBOH*s in common grape vine (*VvRBOH*), and 8 *RBOH*s in sweet orange (*CsRBOH*). We named the 40 RBOHs separately and analyzed their amino acid properties (Table 1). The 40 predicted full-length RBOH proteins varied from 116 (PmRBOHA) to 1067 (PmRBOHE) amino acid residues and the relative molecular mass ranged from 13.74 (PmRBOHA) to 121.09 (PmRBOHE) kDa, with isoelectric points in the range of 4.58–9.37.

Across *PbRBOH* family members, the MW varied from 79.09 to 111.34 kDa (average MW is 100.41 kDa), and the theoretical pI of these members ranged from 8.07 to 9.37 (average theoretical pI is 8.86). For PpRBOHs, VvRBOHs, PmRBOHs, and CsRBOHs, the average MW are 102.76 kDa, 99.64 kDa, 93.39 kDa, and 93.20 kDa, and the average theoretical pI are 9.00, 8.82, 8.38, and 8.75 respectively. Subcellular localization predictions indicated that 40 RBOHs derived from pear, peach, mei, grape and sweet orange were mainly located in chloroplast (22.5%), cytoplasmic (30%), nucleus (15%) and plasmodesmata (32.5%) (Table 1).

**Figure 1:**
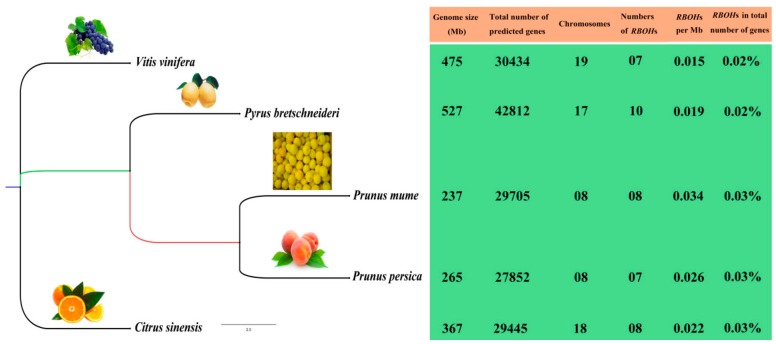
A summary of the evolutionary relationships, genome size, and *RBOH* distribution of the five fruit-producing trees.

To expand knowledge of the *RBOH* family of the five fruit-producing trees, we compared the genome size, chromosome number and *RBOH* family distribution of the five fruit-producing trees from the common hexaploid ancestor [26]. We constructed a species phylogenetic tree and performed statistics on the number of *RBOH*s (Figure 1). The results showed that the range of the number (7–10) of *RBOH* family members among the five species was small. The percentage of putative *RBOH*s in the total number of predicted genes in five fruit-producing trees was also similar. The number of genes per megabase (Mb) ranged from 0.015 (common grape vine) to 0.034 (mei). This indicates that the number of *RBOH* family members has not increased due to the size of the species genome. Therefore, the expansion of these five fruit-producing tree *RBOH* families was not associated with an increase in the genome size and in the total number of predicted genes.

### 3.2. Chromosome Distribution and Duplication Events of RBOHs in Five Fruit-Producing Trees

In order to clarify the distribution of *RBOH* on the chromosomes of five fruit trees, we used MapInspect v.1 software to map the location of *RBOH* family members (Figure S1). The *RBOH* family members of the five fruit trees showed an irregular distribution on the chromosome and did not form a large number of gene clusters. According to the phylogenetic tree clustering of each *RBOH* family (Figure S2) and the rule of determining gene duplication events [16], only two segmental duplication events were identified in the pear *RBOH* family. The segmental duplicated gene pairs are *PbRBOHA*/*PbRBOHB* and *PbRBOHH*/*PbRBOHG*, respectively (Figure S1).

### 3.3. Phylogenetic Relationship, Conserved Motifs and Intron/Exon Structures of RBOHs in Five Fruit-Producing Trees

**Figure 2:**
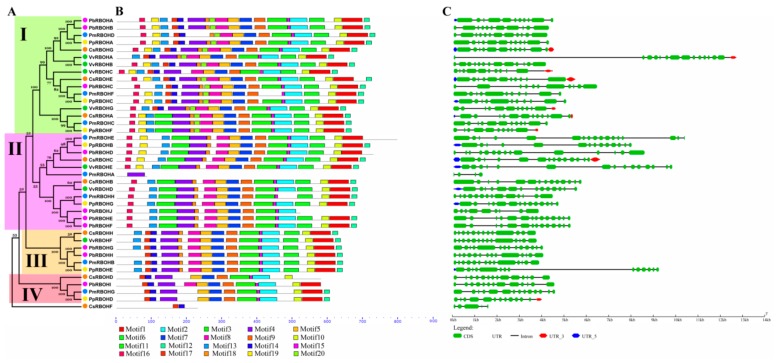
The gene structures and conserved motifs of *RBOH* family members of five fruit-producing trees based on the evolutionary relationship. (**A**) Phylogenetic tree of *RBOH* family members of five fruit-producing trees. (**B**) Conserved motifs of RBOHs of five fruit-producing trees. (**C**) The exon-intron structure of *PbRBOH*s of five fruit-producing trees.

Through systematic phylogenetic tree analysis, 40 non-repetitive *RBOHs* derived from five fruit-producing trees can be divided into four subfamilies (I, II, III and IV). In addition, *CsRBOF* forms an independent clade. Except for subfamily IV, members of subfamily I–III are composed of *RBOHs* of five fruit-producing trees. Eleven gene pairs were formed among *RBOH*s with higher bootstrap values (≥99), except for the lower bootstrap values of two pairs: *CsRBOHB*/*VvRBOHD* and *CsRBOHH*/*VvRBOHF* (Figure 2A). 

To investigate the structural diversity of RBOHs, a total of 20 conserved motifs in the *RBOHs* were captured by MEME v. 5.05 software (Figure 2B and Table S2). It is worth noting that the type and distribution of the C-terminal domains (motifs 3, 15, 2, 6, 10, 1, and 12) of most RBOHs are similar. However, the type and distribution of the N-terminal domains exhibit a certain subfamily specificity. For example, motif 16 is only present in members of subfamilies I and II. Members of subfamily IV do not contain motif 13.

In addition, the N-terminal domain of the members of subfamily I is generally composed of motifs 16, 19, and 13. Members of subfamily II can have two major clades, one of which has the same N-terminal domain composition as the members of subfamily I, and the N-terminal domain of another clade member consists only of motifs 16 and 13. The N-terminal domain of the subfamily IV members contains motifs 17 and 14, which lack a motif 13 compared to members of subfamily III (Figure 2B). These results suggest that differences in the composition of the N-terminal domain may confer different biological functions to members of each subfamily.

Gene structure analysis showed that the number of introns of *RBOH*s varied from 3 to 16 (Figure 2C). The exons of the *RBOH*s of the five fruit trees are all shorter, which may be characteristic of the *RBOH* family. Generally, most of *RBOH* family members in the same subfamilies showed similar motif characteristic and exon-intron structure, which supports their close evolutionary relationship and the classification of subfamilies. 

### 3.4. Amino Acid Sequence and Characteristic Domain Analysis of PbRBOHs

**Figure 3:**
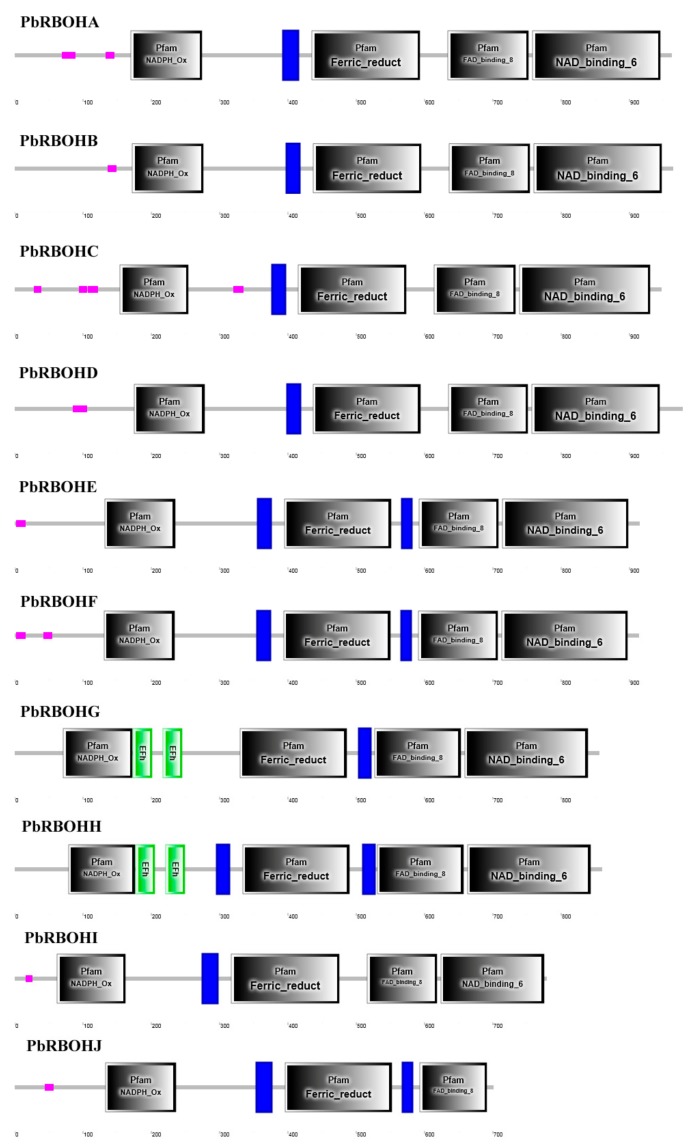
Depiction of various domains in *PbRBOHs*.

We further analyzed the analysis of the characteristic domains of the *RBOH* family of pear (Figure 3). The results showed that the characteristic domains of the *PbRBOH* family members mainly include: NADPH_Ox (PF08414), EFh (IPR002048), Ferric_reduct (PF01794), FAD_binding_8 (PF08022), and NAD_binding_6 (PF08030). As can be seen from Figure 3, NADPH_Ox and EFh are mainly distributed at the N-terminus, while Ferric_reduct, FAD_binding_8, and NAD_binding_6 are distributed at the C-terminus. According to the annotated results of SMART (http://smart.embl-heidelberg.de/smart/show_motifs.pl), Ferric_reduct domain is a common region in the transmembrane proteins mammalian cytochrome B-245 heavy chain (gp91-phox). Plant *RBOH* encode homologs of the mammalian gp91^phox^ [52], therefore, each PbRBOH contains this domain. NADPH_Ox is a characteristic domain of plant respiratory burst NADPH oxidase proteins, and PbRBOHA-J all have this domain, further demonstrating that the *PbRBOH* family members we identified are reliable. Interestingly, only PbRBOHJ lacks the NAD_binding_6 domain, which may cause changes in its function.

**Figure 4:**
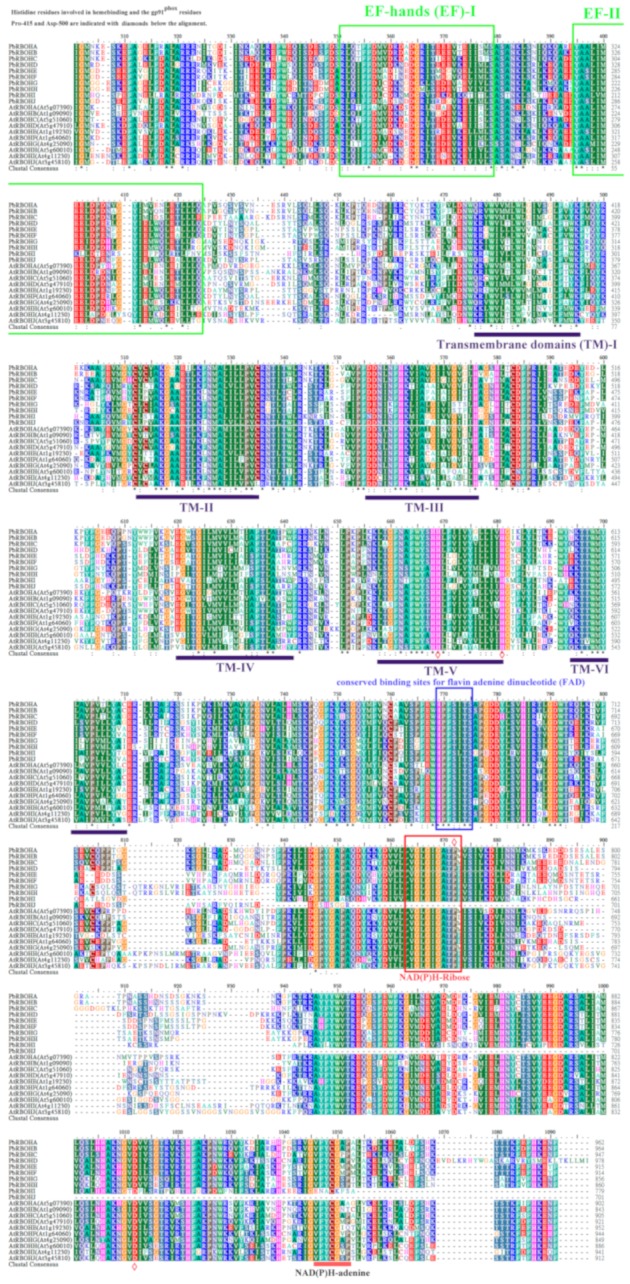
Protein sequence multi-alignment and domain structure of RBOHs from pear and *Arabidopsis*. Conservative residues are highlighted by black asterisks. Histidine residues involved in heme binding and the gp91^phox^ residues Pro-415 and Asp-500 are indicated with diamonds. EF-hands and conserved binding sites for FAD, NAD(P)H-adenine, and NAD(P)H-ribose are represented by straight lines or boxes. Transmembrane domains are indicated by straight lines.

In addition, multiple sequence alignments of 10 PbRBOHs and 10 AtRBOHs were performed according to previously reported methods [32,53] (Figure 4). It can be found that the amino acid sequences of *AtRBOH* family members and *PbRBOH* family members are highly conserved, especially the typical domain of the *RBOH* family, such as two calcium-binding motifs (EF-hands) and six α-helical transmembrane domains (TM-I to TM-VI), and flavin adenine dinucleotide (FAD), nicotinamide adenine dinucleotide phosphate [NAD(P)H]-ribose, and NAD(P)H-adenine conserved binding sites in the C-terminal region. Notably, two amino acid residues (Pro-415 and Asp-500) in gp91^phox^ are critical for catalytic activity [53]. However, PbRBOHJ lacks these two sites and the tertiary structure changes significantly, suggesting that the catalytic activity of this member will be affected (Figure 4 and Figure S3). 

### 3.5. Microsynteny Analysis of PbRBOHs

**Figure 5:**
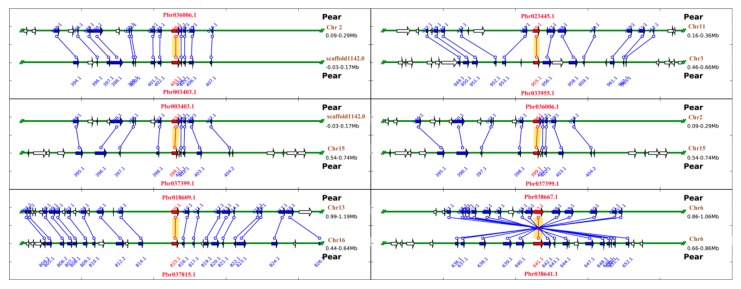
Microsynteny maps of *PbRBOH*s and their flanking genes within syntenic chromosomal intervals. The relative positions of all flanking protein-coding genes were defined by the anchored *RBOH*s, highlighted in red. The chromosome segments are shown as horizontal lines, with arrows corresponding to individual genes and their transcriptional orientations. Conserved gene pairs among the segments are connected with lines.

To explore the evolutionary relationship of *RBOH*s, we performed microsynteny analysis of *PbRBOH*s and the coding genes in their surrounding chromosomal regions (Figure 5). It was found that there are 6 pairs of collinear gene pairs in the pear genome, including Pbr003403.1/Pbr036006.1, Pbr003403.1/Pbr037399.1, Pbr018609.1/Pbr037815.1, Pbr023445.1/Pbr033955.1, Pbr036006.1/ Pbr037399.1, and Pbr038641.1/Pbr038667.1. These 6 pairs of collinear gene pairs did not undergo chromosomal inversion during duplication (Figure 5).

Pear, sweet orange, mei, peach and grape share a common ancestor [4,26]. To further clarify the evolutionary origin and orthologous relationship of the *RBOH* family among the five fruit-producing trees, we identified the interspecific collinear relationship of these five species (Spreadsheets S1). Our results suggest that 18 collinear gene pairs (7 collinear gene pairs between pear and grape, 1 pair between pear and sweet orange, 3 pairs between sweet orange and grape, 3 pairs between mei and grape, 3 pairs between peach and grape, 1 gene pair is within the peach) were found among pear, sweet orange, mei, peach and grape (Spreadsheets S1). Overall, the degree of interspecies collinearity of *CsRBOH*s, *PpRBOH*s, *VvRBOH*s, and *PmRBOH*s is lower than intraspecies collinearity. These results indicate that the *RBOH* family of four species is relatively independent in evolution, and that chromosome duplication, deletion and rearrangement are later than species differentiation.

### 3.6. Comparative Phylogenetic Analysis and Functional Prediction of PbRBOHs

**Figure 6:**
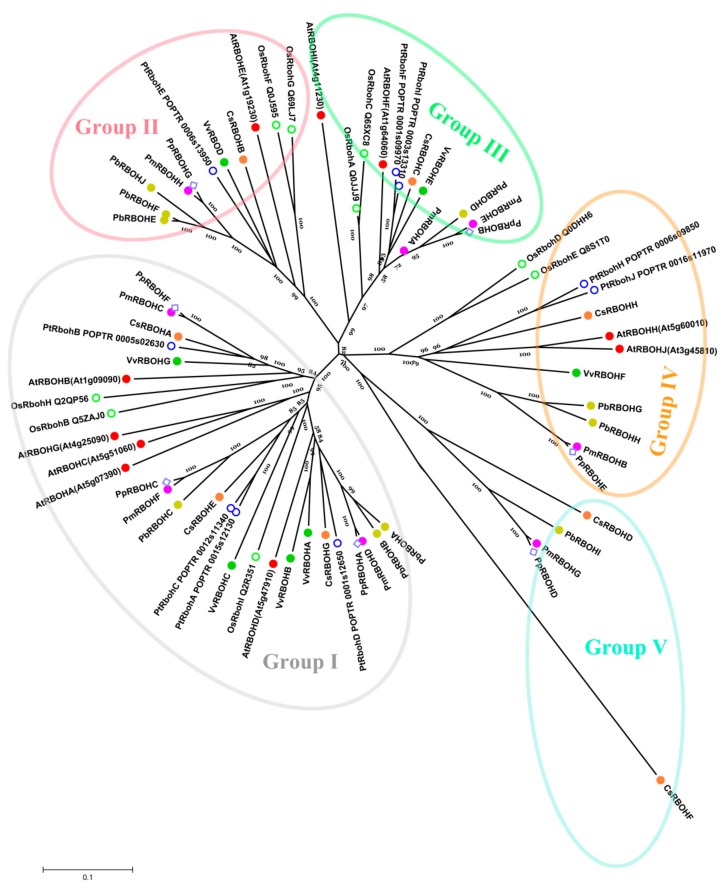
Phylogenetic analysis of RBOHs from five fruit-producing trees (pear, sweet orange, mei, peach and grape) and other known RBOHs. Full-length polypeptide sequences were used to make the interspecific phylogenetic tree.

To characterize the evolutionary relationship and possible functions between RBOHs from five fruit trees and other known RBOHs from *Arabidopsis*, rice and poplar, a neighbor-joining tree was created (Figure 6). 

The results showed that the 70 RBOHs from eight species (pear, peach, grape, mei, sweet orange, Arabidopsis, rice and poplar) could be assigned to 5 phylogenetic groups (I-V). With the exception of Group V, the other four phylogenetic groups are composed of RBOHs from eight species. Ten PbRBOHs were distributed in five phylogenetic groups, suggesting the existence of a diversified *RBOH* family in pear with diverse functions. Generally, RBOHs from pear have closer relationships with the PpRBOHs or PmRBOHs than that from VvRBOHs and CsRBOHs, which is in accordance with the current understanding of plant evolutionary history. In addition, evolutionary analysis also identified some closely related orthologous RBOHs between peach and mei, indicating that an ancestral set of *RBOH*s existed prior to the divergence of peach and mei.

For members of Group I and III, AtRBOHD and AtRBOHF are considered to be closely related to lignification, and they are also involved in the plant’s stress response process, which has a significant impact on the production of ROS in plants [8,28,31,54,55]. Phylogenetic tree clustering results indicated that RBOHA, RBOHB, and RBOHD in pears belonged to the same clades as AtRBOHD and AtRBOHF, suggesting that they may have similar biological functions (Figure 6).

It has now been demonstrated that *AtRBOHC* mutations result in inhibition of *Arabidopsis* root and root hair growth, and the level of ROS in plants is significantly reduced [54]. In addition, *AtRBOHA*, *-B*, and *-G* are all specifically expressed in the root [37]. It is speculated that RBOHs that are closely related to them may play a role mainly in the roots.

The functions of members in Group II and IV are currently poorly understood. *AtRBOHH* and *-J* are specifically expressed in pollen, *AtRBOHE* and *OsRbohD* are expressed in root and seeds [37]. Therefore, whether PbRBOHs of the same group has similar expression patterns and functions remains to be further verified. Group V contains only 5 RBOHs, while other groups contain at least 11 RBOHs. The five RBOHs in this group are derived from pear, sweet orange, mei and peach, respectively. It is speculated that these five RBOHs (CsRBOHD, CsRBOHF, PbRBOHI, PmRBOHG, and PpRBOHD) are a unique class of these four species, which may have new unknown functions.

### 3.7. Analysis of Cis-Acting Elements in Putative PbRBOH Promoters

Previous studies have shown that RBOH plays an important role in plant stress response, and their expression is regulated by a variety of plant hormones [37,56,57]. To this end, *cis*-acting elements of 10 *PbRBOH* promoter regions were identified in this study to explore their possible expression regulation mechanisms (Spreadsheets S2).

Analysis of the results showed that except for *PbRBOHG*, the other nine members contained G-box elements, which could respond to a variety of hormones, including ABA and MeJA [58]. In addition, W-box elements that can respond to SA, MeJA and ethylene were identified in the promoter regions of *PbRBOHG*, *PbRBOHA*, *PbRBOHH*, and *PbRBOHC* [5]. We found that 70% of the *PbRBOH* family members have ABRE (ABA-responsive) in their promoters and 50% of members contain TGACG-motif (MeJA-responsive). In addition to the hormone response elements, we also identified some abiotic stress response elements in the *PbRBOH* promoter region, such as LTR (low-temperature responsiveness), MBS (drought-inducibility), and ARE (anaerobic induction). Therefore, the transcription of *PbRBOH* is likely to be multi-regulated by hormones and the environment.

### 3.8. Differentially Expressed PbRBOHs under Hormonal Treatments

Based on the analysis of the *cis*-acting elements in the promoters of the *PbRBOH* family members, we found that most of the promoters of *PbRBOH*s contain a variety of plant hormone response-related elements (Spreadsheets S2). Therefore, we used three exogenous hormone (SA, ABA, and MeJA)-treated pear fruits as materials to analyze the effect of hormone treatment on *PbRBOH* expression patterns.

**Figure 7:**
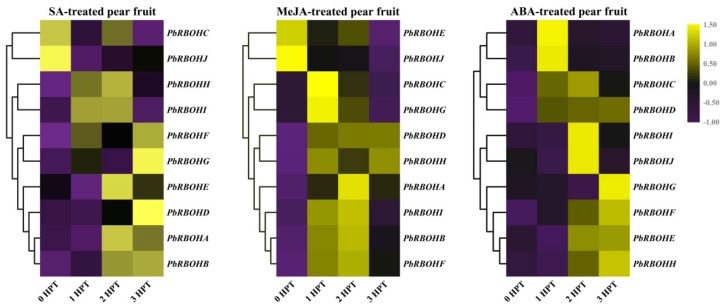
Heatmap representation of *PbRBOH*s expression in response to salicylic acid (SA), methyl jasmonate (MeJA) and abscisic acid (ABA) treatments. The gene expression data of *PbRBOH*s at 0 h post-treatment (HPT), 1 HPT, 2 HPT and 3 HPT were obtained through reverse transcription-quantitative real-time polymerase chain reaction (qRT-PCR). The expression data of each gene at 0 HPT was used as a control sample to show the relative expression level. Purple and yellow boxes indicate lower or higher transcript levels, respectively. Each qRT-PCR analysis was performed in triplicate.

For SA-treated pear fruits, it was found that the transcription levels of *PbRBOHC* and *PbRBOHJ* were inhibited, and the remaining members peaked at 1 to 3 HPT. Among them, *PbRBOHA*, *PbRBOHE*, *PbRBOHH* and *PbRBOHI* showed a rise–fall tendency. *PbRBOHB*, *PbRBOHF*, *PbRBOHG* and *PbRBOHD* peaked at 3 HPT (Figure 7).

In the MeJA-treated pear fruits, the transcription levels of *PbRBOHE* and *PbRBOHJ* were inhibited, and the expression of other members was induced to varying degrees. Moreover, the transcriptional abundance of most members peaked at 1 HPT and 2 HPT, and decreased at 3 HPT (Figure 7).

ABA plays an inducing role in the transcription of 10 *PbRBOH*s. Among them, the expression levels of *PbRBOHA* and *PbRBOHB* reached a peak at 1 HPT, and then basically returned to the pre-treatment level. *PbRBOHC*, *PbRBOHI* and *PbRBOJ* have the highest expression at 2 HPT. The inducing effect of ABA on *PbRBOHD* lasted for a long time, and the transcript abundance of PbRBOHD from 1 to 3HPT was maintained at a high level. In addition, the transcript levels of *PbRBOHG*, *PbRBOHF*, *PbRBOHE*, and *PbRBOHH* were gradually increased after ABA treatment, reaching a peak at 3 HPT (Figure 7).

### 3.9. Histochemical Staining and Determination of Apoplastic Superoxide (O_2_.^−^) in Pear Fruit

**Figure 8:**
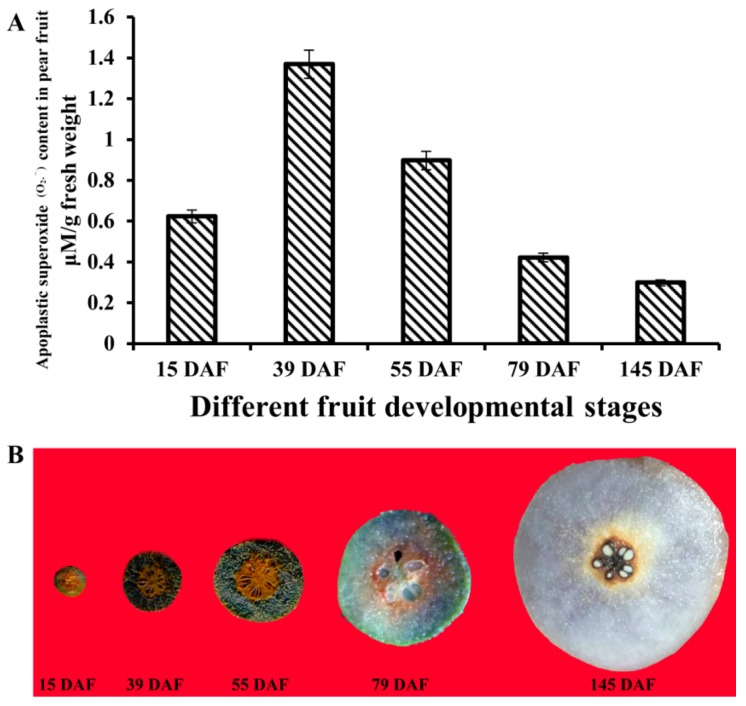
Analysis of apoplastic superoxide content in pear fruit at different developmental stages. (**A**) The content of O_2_.^−^ in pear fruits at different developmental stages. (**B**) Specific histochemical staining of apoplastic superoxide in pear pulp.

To obtain some clues on the roles of PbRBOHs in the development of pear stone cells, this study determined the content of RBOH product (O_2_.^−^) in different developmental stages of pear fruit. The accumulation of O_2_.^−^ in pear fruit was also analyzed by NBT staining. It can be seen from Figure 8 that the content of O_2_.^−^ firstly increased and then decreased during different developmental stages of pear fruit, and reached a peak at 39 DAF. NBT-specific staining results also showed that O_2_.^−^ accumulated at high levels in 39 DAF and 55 DAF pear fruits, and lower in 79 DAF and mature fruits. Previous studies have shown that high-density stone cells are distributed in pear flesh at 39 DAF and 55 DAF, and stone cell development and lignification have basically stopped at 79 DAF [10,16]. This indicates that the O_2_.^−^ content change pattern is consistent with the development of stone cells and lignification, further confirming that the secondary cell wall lignification of stone cells requires the participation of O_2_.^−^.

### 3.10. Tissue Specificity and Temporal Expression Patterns in Pear Fruit during Development

**Figure 9:**
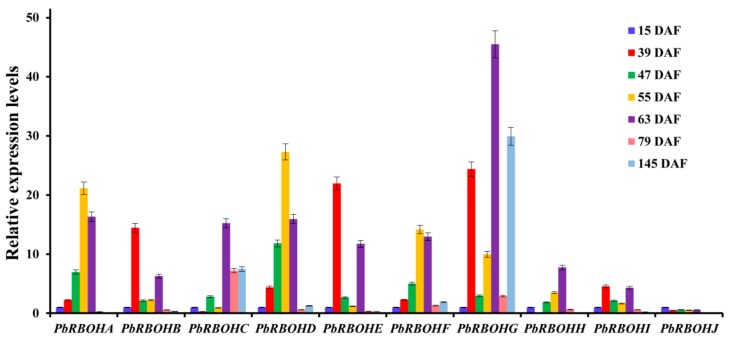
Expression profiles of pear *RBOH*s across different developmental stages. The expression level of 10 *PbRBOH*s in pear fruits at eight developmental stages (15 DAF, 39 DAF, 47 DAF, 63 DAF, 79 DAF, and 145 DAF). The relative expression level was calculated by setting the expression value of *PbRBOH*s in fruit (15 DAF) of pear at 1.

Our initial interest in PbRBOHs came from the proposed role of *PbRBOH*s in cell wall lignification. In order to screen out RBOHs involved in the production of reactive oxygen species during the lignification of stone cells, we analyzed the expression patterns of 10 *PbRBOH*s in different developmental stages of pear fruit. As can be seen from Figure 9, the transcriptional levels of *PbRBOHA*, *PbRBOHD*, and *PbRBOHF* showed a rise-fall tendency, consistent with changes in the content of stone cells and O_2_.^−^. This suggests that they may be involved in the production of ROS during the lignification of stone cells. 

**Figure 10:**
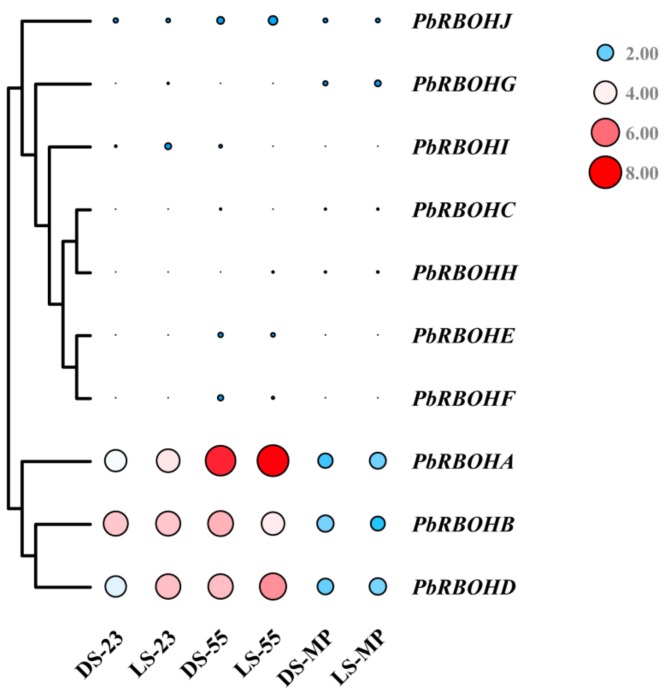
Expression profiles of *PbRBOH*s in different pear varieties at different developmental stages. FPKM values were obtained by RNA-seq analysis and presented as a heatmap. The colour scale and circle size are provided with the heat map to indicate the differential pattern of expression. DS: *Pyrus bretschneideri* cv. Dangshan Su; LS: *Pyrus bretschneideri* cv. Lianglizaosu; 23: 23 DAF; 55: 55 DAF; MP: mature period. The heatmap is drawn using the TBTools v. 0.6644 software (http://www.tbtools.com/).

Subsequently, we used the fruit transcriptome database of two Chinese white pear (*Pyrus bretschneideri*) cultivars that were previously completed [35] to analyze the transcript abundance of 10 *PbRBOH*s in different developmental stages of pear fruit (Figure 10). The fragments per kilobase of exon per million fragments mapped (FPKM) values for each *PbRBOH* are listed in Table S3. The results showed that although the expression changes of some members in pear fruit were consistent with the content of stone cells and O_2_.^−^, only *PbRBOHA*, *PbRBOHB*, and *PbRBOHD* had higher transcript abundance in fruits. The remaining members are weakly expressed in the fruit and may not play a major role in the fruit, such as *PbRBOHF.*

Therefore, combined with the results of phylogenetic tree clustering, trend of expression changes and transcript abundance, this study speculated that PbRBOHA and PbRBOHD are mainly responsible for the supply of ROS during the lignification of pear stone cells.

**Figure 11:**
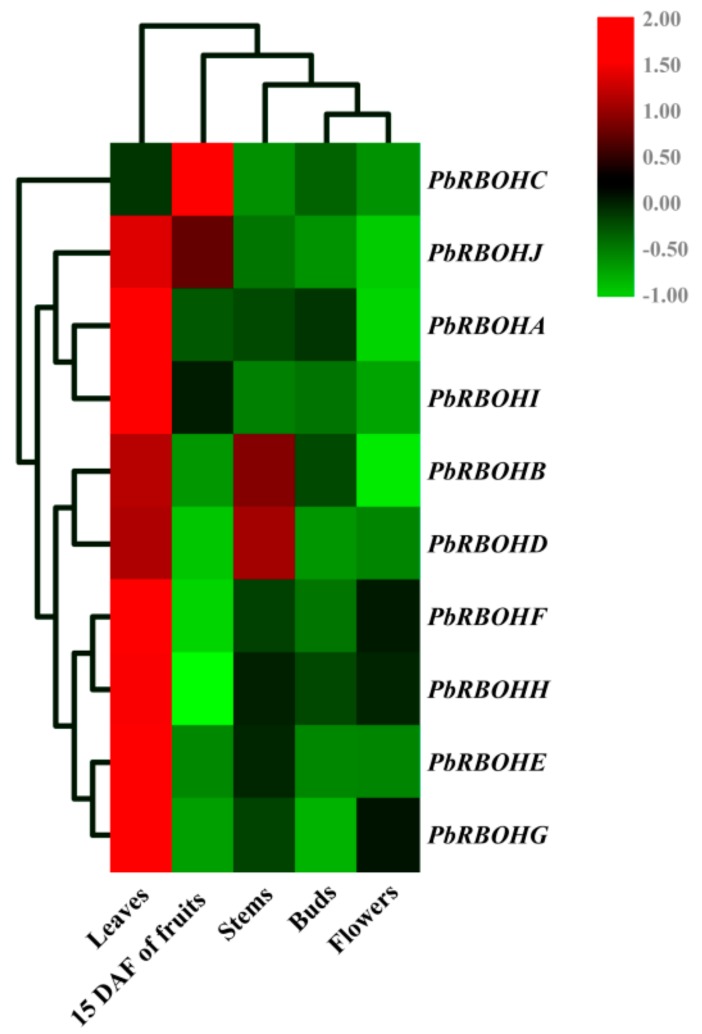
Expression profiles of pear *RBOH*s across different tissues. The transcript level of the 10 *PbRBOH*s in different tissues (fruits, leaves, stems, buds, and flowers). The expression data of each gene in fruit (at 15 DAF) of pear was used as a control sample to show the relative expression level. Each qRT-PCR analysis was performed in triplicate. The heatmap is drawn using the TBTools v. 0.6644 software (https://github.com/CJ-Chen/TBtools/releases).

For buds, stems, leaves and flowers, except for *PbRBOHC*, the other 9 *PbRBOH*s expressed the highest in leaves. *PbRBOHB* and *PbRBOHD* also have higher levels of transcription in stems (Figure 11).

### 3.11. Subcellular Localization Analysis of Candidate PbRBOHs for Pear Stone Cell Lignification

**Figure 12:**
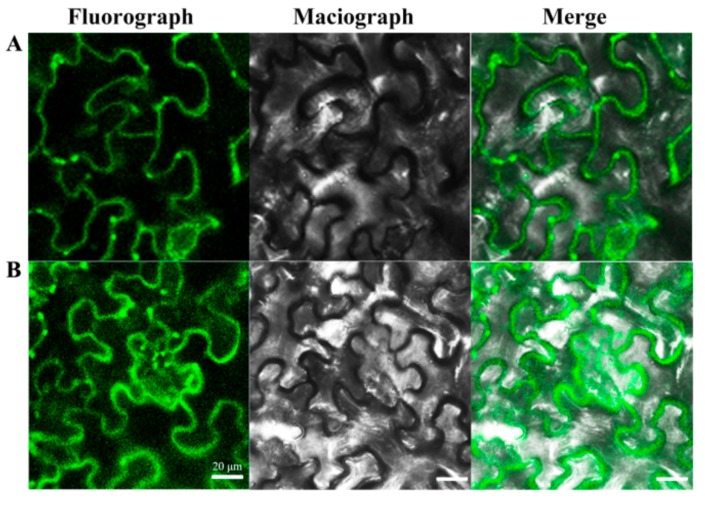
Subcellular localization of PbRBOHA-GFP and PbRBOHD-GFP. (**A**) Subcellular localization of PbRBOHA-GFP. (**B**) Subcellular localization of PbRBOHD-GFP.

Previous studies have shown that the *RBOH* is mainly localized on the plasma membrane and is responsible for catalyzing the formation of O_2_.^−^ and thus participating in the polymerization of lignin monomers [8,37]. In order to clarify the subcellular distribution of the candidate genes, the eukaryotic expression vectors of *PbRBOHA* and *PbRBOHD* were constructed and transiently transformed into tobacco leaves. Green fluorescent protein (GFP) fluorescence was observed by laser scanning confocal microscopy (LSCM) (Olympus, Tokyo, Japan). As can be seen from Figure 12, both PbRBOHA-GFP and PbRBOHD-GFP are localized on the plasma membrane.

## 4. Discussion

There are five main types of lignified cells currently known in plants, including tracheary elements (TEs), sclerenchyma cells, endodermal cells, and seed coat cells [8]. Lignified cells are critical to plants, including resistance to external environmental conditions, seed protection, mechanical support, water and nutrient transport [7,8]. However, excessive lignification of the cells can have many negative effects on the fruit quality of trees [18,23,25,59]. Therefore, studying the molecular mechanism of lignification is of great significance for regulating fruit quality. 

Numerous studies have shown that ROS play an important role in the lignification process [28,31]. However, the research on the *RBOH* family of catalytically lignification-related ROS production in fruit trees has rarely been reported. For this reason, 40 *RBOH*s were screened from the genome database of five fruit trees. Among them, the number of *PbRBOH* family members was the highest, and the number of *PpRBOH* family members was the least (Table 1). Phylogenetic tree analysis showed that the *RBOH* family members of pear, mei and peach were closely related, but they were far from sweet orange and grape, probably because the first three species belonged to Rosaceae (Figure 2). In particular, pear, mei, peach and sweet orange all differentiated into a single group (Group V) *RBOH* family members. The members of this subfamily are very distantly related to the *RBOH* members of poplar, rice, grape and Arabidopsis (Figure 6). It may already have completely different biological functions.

The formation of pear stone cells is a typical lignification process [19]. It provides a good model for the formation of lignification-related ROS. Therefore, this study focused on the content of O_2_.^−^ in pear fruit and the expression pattern of *PbRBOH*s. The distribution of stone cell clusters in different developmental stages of pear fruit has been revealed in our previous studies [16]. The results show that in the fruit of 39 DAF to 55 DAF, the stone cell cluster accumulates in the pulp between the fruit core and the peels. As expected, NBT staining is the deepest in the pear fruit at this stage of development, suggesting that a large amount of O_2_.^−^ is produced at this time to promote stone cell development and lignification (Figure 8). Peroxidase and laccase catalyzed the formation of monolignol radicals must have H_2_O_2_ and O_2_ as additional substrates, while O_2_.^−^ is H_2_O_2_ and O_2_ direct precursor [8,29], so the content of O_2_.^−^ directly affects the lignification polymerization process.

Analysis of *cis*-acting elements revealed that most of the *PbRBOH* promoters contained a large number of elements related to hormones and stress response (Spreadsheets S2). This is consistent with previous studies [57]. We selected three hormones (SA, ABA, and MeJA) to treat pear fruit and found that it has a significant effect on the expression of *PbRBOH*s, and the transcription of most *PbRBOH*s is induced in a short time (Figure 7). Notably, not all of the 10 *PbRBOH* promoters have elements that respond to the three hormones. For example, the SA response element has not been found in the *PbRBOHI* promoter. However, the expression level of *PbRBOHI* still changed in the fruits treated with SA (Spreadsheets S2 and Figure 7). This may be due to interactions between various plant hormones in plants, which can promote synergy and induce one another [60]. Therefore, spraying a hormone is likely to induce an increase in the content of another hormone, thereby affecting the transcription of the gene. In addition, photoresponsive elements such as Box4, GATA-motif, GT1-motif are also present in the *RBOH* promoters. This suggests that bagging treatment changes the lignin and stone cell content of pear fruit may be related to the transcription of *RBOH* [61,62].

Importantly, in order to identify the RBOHs responsible for lignification-related ROS synthesis in pear fruit, we compared the expression patterns of 10 *PbRBOH*s in pear fruit at 7 developmental stages (Figure 9). Phylogenetic tree clustering indicated that *PbRBOHA*, *PbRBOHB*, and *PbRBOHD* were most likely related to lignification, and transcriptome sequencing also showed that the transcript abundance of *PbRBOHA*, *PbRBOHB* and *PbRBOHD* was the highest among the 10 members in different pear varieties (Figure 10). It is indicated that these three *RBOH*s play a role in pear fruit. However, the expression level of *PbRBOHB* showed a downward trend during the large accumulation period of stone cell (47 DAF and 55 DAF) (Figure 9) [10,16], and the expression level was up-regulated after the stone cell development was basically stopped (63 DAF) [10], which was inconsistent with the process of stone cell development. It is speculated that although this gene has a high expression level in fruits, it is not the major gene involved in the synthesis of ROS during the development of stone cells. In addition, the expression levels of *PbRBOHA*, *PbRBOHD* and *PbRBOHF* in pear fruit showed a rise-fall tendency, which was consistent with the trend of stone cell content (Figure 9) [10,61,63]. However, transcriptome sequencing showed that *PbRBOHF* has a weak transcription level in fruits (Figure 10), so it is speculated that this gene has little correlation with stone cell development. Therefore, *PbRBOHA* and *PbRBOHD* are considered to be lignification-related *RBOH* in pears. Notably, the up-regulation of *PbRBOHA* is most significant at 55 DAF (Figure 10 and Table S3), suggesting that the gene may be the major gene responsible for lignification-related ROS production. 

**Figure 13:**
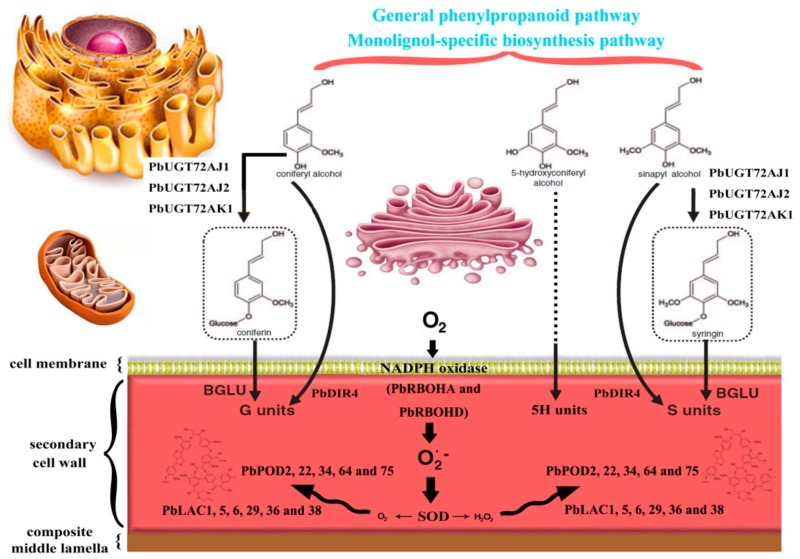
A model of reactive oxygen species (ROS) metabolism and lignin monomer polymerization in pear fruit.

More strikingly, we summarized the results of this study with the reported pear uridine diphosphate-glycosyltransferases (UGTs), PODs, LACs and DIRs in an ideograph of the polymerization of lignin monomer in pear fruit (Figure 13) [4,19,35,36]. The monolignols in pear fruit may be catalyzed by PbUGT72AJ1, PbUGT72AJ2 and PbUGT72AK1 to form lignin glycoside, which is then transported across the membrane to cell wall deposition. Subsequently, the lignin oligomers are formed by the catalysis of PbDIR4. Finally, POD and LAC use ROS and O_2_ produced by RBOH (PbRBOHA and PbRBOHD) and SOD to catalyze the elongation of lignin polymer chains. 

## 5. Conclusions

In this study, we identified 10, 7, 8, 8 and 7 members of the *RBOH* family from the pear, peach, mei, sweet orange and grape genomes, respectively. RBOHs derived from fruit-producing trees can be divided into five subfamilies, and the distribution of conserved motifs and gene structures have a certain subfamily specificity. The 10 *RBOH* family members of the pear all have six α-helical transmembrane domains, and there are a large number of *cis*-acting elements related to biotic and abiotic stress in the promoter. Hormone treatment revealed that the transcription of most *PbRBOH*s was induced by SA, ABA and MeJA. Analysis of spatio-temporal expression patterns showed that *PbRBOHA*, *PbRBOHB*, and *PbRBOHD* had higher transcript abundance in pear fruit, and the expression trends of *PbRBOHA* and *PbRBOHD* were consistent with the content of stone cells and O_2_.^−^. Combined with the results of phylogenetic tree clustering and expression pattern analysis, it is speculated that *PbRBOHA* and *PbRBOHD* participate in the production of reactive oxygen species during the formation of stone cells. Subcellular localization analysis indicated that both RBOHs were localized to the plasma membrane. 

## Figures and Tables

**Table 1:** Sequence information of the *RBOH* family genes of five fruit-producing trees.

Species	Sequence ID	Name	Chr	Protein			
				Length (aa)	MW (kDa)	pI	Subcellular Localization Predicted
Pear	Pbr018609.1	*PbRBOHA*	13	962	108.07	9.02	Plas
	Pbr037815.1	*PbRBOHB*	16	964	108.42	9.11	Plas
	Pbr038667.1	*PbRBOHC*	6	947	106.18	8.07	Plas
	Pbr006277.1	*PbRBOHD*	6	978	111.34	9.37	Cyto
	Pbr036006.1	*PbRBOHE*	2	915	103.56	8.59	Cyto
	Pbr003403.1	*PbRBOHF*	/	914	103.11	8.59	Cyto
	Pbr023445.1	*PbRBOHG*	11	856	97.65	8.66	Cyto
	Pbr033955.1	*PbRBOHH*	3	860	97.56	8.79	Cyto
	Pbr007212.1	*PbRBOHI*	14	779	89.07	9.06	Plas
	Pbr037399.1	*PbRBOHJ*	15	701	79.09	9.29	Chlo
Peach	Prupe.1G211000	*PpRBOHA*	1	971	108.87	9.18	Chlo
	Prupe.5G107400	*PpRBOHB*	5	964	109.47	9.32	Chlo
	Prupe.5G138300	*PpRBOHC*	5	941	105.22	8.97	Plas
	Prupe.5G204900	*PpRBOHD*	5	810	92.48	8.85	Plas
	Prupe.6G088800	*PpRBOHE*	6	859	98.48	8.88	Nucl
	Prupe.6G321500	*PpRBOHF*	6	893	101.94	8.87	Nucl
	Prupe.7G193000	*PpRBOHG*	7	906	102.84	8.93	Cyto
Mei	Pm000665	*PmRBOHA*	1	116	13.74	4.58	Cyto
	Pm000754	*PmRBOHB*	1	860	98.62	8.83	Cyto
	Pm003305	*PmRBOHC*	1	893	102.01	9.00	Nucl
	Pm007134	*PmRBOHD*	2	982	110.11	9.02	Nucl
	Pm023955	*PmRBOHE*	7	1067	121.09	9.29	Chlo
	Pm024297	*PmRBOHF*	7	940	105.50	8.73	Chlo
	Pm024959	*PmRBOHG*	7	810	92.32	8.81	Plas
	Pm027130	*PmRBOHH*	8	914	103.69	8.74	Plas
Grape	GSVIVT01001122001	*VvRBOHA*	1	827	94.13	9.16	Chlo
	GSVIVT01001123001	*VvRBOHB*	1	906	102.80	9.13	Nucl
	GSVIVT01014350001	*VvRBOHC*	19	840	95.40	6.47	Plas
	GSVIVT01015025001	*VvRBOHD*	11	917	103.81	9.26	Chlo
	GSVIVT01019429001	*VvRBOHE*	2	922	104.75	9.26	Cyto
	GSVIVT01025074001	*VvRBOHF*	6	852	97.14	9.12	Cyto
	GSVIVT01031128001	*VvRBOHG*	14	873	99.44	9.34	Cyto
Sweet orange	Cs3g14240.1	*CsRBOHA*	3	889	101.04	9.14	Cyto
	Cs4g06920.1	*CsRBOHB*	4	910	103.34	9.19	Chlo
	Cs5g02940.1	*CsRBOHC*	5	946	107.50	9.37	Chlo
	Cs5g11890.1	*CsRBOHD*	5	777	88.70	8.57	Plas
	Cs7g19320.2	*CsRBOHE*	7	970	109.69	8.78	Nucl
	Cs7g19380.1	*CsRBOHF*	7	308	35.23	6.72	Plas
	Cs8g12000.1	*CsRBOHG*	8	915	103.52	9.11	Plas
	Cs8g17640.1	*CsRBOHH*	8	842	96.61	9.11	Plas

“/” indicates that the specific chromosome is not known. Chloroplast (Chlo); Cytoplasmic (Cyto); Nucl (Nucleus); Plasmodesmata (Plas).

## Supplementary Materials

The following are available online at https://www.mdpi.com/2073-4409/8/6/520/s1.
