# Supplementary material for: In Silico Genome-Wide Analysis of Respiratory Burst Oxidase Homolog (RBOH) Family Genes in Five Fruit-Producing Trees, and Potential Functional Analysis on Lignification of Stone Cells in Chinese White Pear
Source: Cells. 2019 May 29;8(6):520. doi: 10.3390/cells8060520 (PMC6627160; doi:10.3390/cells8060520)
Supplement: Supplementary file 1 [file cells-08-00520-s001.zip › Supplementary Material/Supplementary Tables.docx]

***Supplementary Material***

**Supplementary Table 1 Primers for qRT-PCR and vector construction.**

| **Gene ID** | **Sense primer sequence (5’-3’)** | **Anti-sense primer sequence (5’-3’)** |
| --- | --- | --- |
| **PbRBOHA** | GGACTTTTCACACAAGACGACCACA | CCCCCATCTTTAATCCATATACTTT |
| **PbRBOHB** | GGATAGCCGAGTGGGGGTGTTTTAC | CTCCCCTTTTTAATGACGCTACTTA |
| **PbRBOHC** | TTCTCTCACAAAACCTCCACCCGAT | ATGTGGTGGACAGACTTGTGTTATC |
| **PbRBOHD** | AAACTTCCTCCAAACACTGTAATG | ACTAGCTCCCCAATAATGACGTTTT |
| **PbRBOHE** | GACATCCTATCTGGCACCCGAGTAA | TCGCTGCTTAGGATTGTGATGATGG |
| **PbRBOHF** | TCAAGGTGCTCTCACATGAACTCAG | TGGTCTTCTAGCTGTAGAAATACTC |
| **PbRBOHG** | GGGAGCTCTGCCTAGAATTTAGTCT | CAGAAAGGACTGCATCTGCACTGTG |
| **PbRBOHH** | CAGGAAGCTCTGCCTAGAATTTAGT | TCTGCACTGTGGAACAGCTGGTCTA |
| **PbRBOHI** | AATAAGCACTATACAAGCGTTGC | GCAATCTTTTATGCTGAGAATTTAC |
| **PbRBOHJ** | CCCATTTGAATGGCATCCATTTTCC | TATGGCTCCCCTCATCCAGATTTCG |
| ***Tubulin*** | AGAACAAGAACTCGTCCTAC | GAACTGCTCGCTCACTCTCC |
| ***PbRBOHA*-ZH** | GCTCTAGAATGAACGGTCATCACGATGAGGAAG | TCCCCCGGGAAAATTCTCTTTGTGGAACTCGAAC |
| ***PbRBOHD*-ZH** | GCTCTAGAATGAGGGATCTTCCGAGGCACGAGC | TCCCCCGGGAATCATTAGCAATTTGGTCAACTTC |

**Supplementary Table 2 Details of 20 conserved motifs in the PbRBOHs.**

| Motif | Width | Best Possible Match | Domain |
| --- | --- | --- | --- |
| 1 | 39 | FQVMGYCLCTAKGAAETLKFNMALILLPVCRNTJTWLRS | * |
| 2 | 50 | KLGVFVPFDDNJNFHKVIAVGIAVGVIJHAGNHLACDFPRLIHASEEKYE | Ferric_reduct |
| 3 | 50 | VIVYVLLIIHGYFLYLTKKWYKKTTWMYJAVPVLLYAGERLJRAFRSGIY | Low complexity region |
| 4 | 36 | HPFSITSAPGDDYLSVHIRTLGDWTRZLKTVFSEVC | FAD_binding_8 |
| 5 | 44 | EMHNYLTSVYEEGDARSALITMVQALNHAKNGVDIVSGTRVKTH | NAD_binding_6 |
| 6 | 41 | PKLLIDGPYGAPAQDYKKYDVLLLVGLGIGATPFISILKDI | NAD_binding_6 |
| 7 | 41 | LKVAVYPGNVLALHMSKPQGFKYKSGQYMFVQCPAVSPFEW | FAD_binding_8 |
| 8 | 41 | EVKEIIMLSASANKLSNJKEQAEEYAALIMEELDPDNLGYI | * |
| 9 | 41 | KITKEELREFWEQISDZSFDSRLQIFFDMVDKNADGRITEE | NADPH_Ox |
| 10 | 34 | TRAYFYWVTREQGSFEWFKGVMBEVAEMDQKGVI | NAD_binding_6 |
| 11 | 41 | NWRKVFKKJASKHPNSRVGVFYCGAPALTKELKQLCLEFSH | NAD_binding_6 |
| 12 | 41 | SSKTKYFLQDNWKRVWVVLLWLGIMAGLFTWKFIQYRNKAA | transmembrane region |
| 13 | 29 | PWFRRNKVNLPKPLKKLTGFNAFWYSHHL | * |
| 14 | 21 | KDSKEFAGELFDALARRRRIK | * |
| 15 | 15 | GILMVVLMAIAFTLA | * |
| 16 | 15 | BGLLPRSDFGECIGM | * |
| 17 | 29 | RRPRARLDRTKSAAARALKGLKFISKTTG | * |
| 18 | 11 | STKFEFHKENF | * |
| 19 | 15 | PPTYWDLVKGVEGVT | * |
| 20 | 20 | BDDZDYVEVTLDLRDDSVAV | * |

Note: ‘*’ indicates that the motif has no specific annotation information.

**Table S3 FPKM values of *PbRBOH*s in pear fruit at various developmental stages.**

| Gene ID | Gene name | FPKM values | | | | | |
| --- | --- | --- | --- | --- | --- | --- | --- |
|  |  | DS-23 | LS-23 | DS-55 | LS-55 | DS-MP | LS-MP |
| Pbr018609 | *PbRBOHA* | 11.74624 | 16.95834 | 127.65 | 192.5325 | 2.182326 | 3.221277 |
| Pbr037815 | *PbRBOHB* | 24.0533 | 24.0339 | 29.8897 | 16.1717 | 3.44108 | 1.96922 |
| Pbr038667 | *PbRBOHC* | 0 | 0 | 0.017822 | 0 | 0.016493 | 0.018703 |
| Pbr006277 | *PbRBOHD* | 9.545156 | 26.19174 | 26.35151 | 43.51613 | 2.991633 | 3.600557 |
| Pbr036006 | *PbRBOHE* | 0 | 0 | 0.128704 | 0.093559 | 0 | 0 |
| Pbr003403 | *PbRBOHF* | 0 | 0 | 0.182652 | 0.037219 | 0 | 0 |
| Pbr023445 | *PbRBOHG* | 0 | 0.018509 | 0 | 0 | 0.117542 | 0.225214 |
| Pbr033955 | *PbRBOHH* | 0 | 0 | 0 | 0.020697 | 0.019423 | 0.022288 |
| Pbr007212 | *PbRBOHI* | 0.023546 | 0.238255 | 0.053854 | 0 | 0 | 0 |
| Pbr037399 | *PbRBOHJ* | 0.123882 | 0.094528 | 0.31618 | 0.536125 | 0.10405 | 1.00E-01 |

Note: DS: *Pyrus bretschneideri* cv. Dangshan Su; LS: *Pyrus bretschneideri* cv. Lianglizaosu; 23: 23 DAF; 55: 55 DAF; MP: mature period.
