# Supplementary material for: In Silico Genome-Wide Analysis of Respiratory Burst Oxidase Homolog (RBOH) Family Genes in Five Fruit-Producing Trees, and Potential Functional Analysis on Lignification of Stone Cells in Chinese White Pear
Source: Cells. 2019 May 29;8(6):520. doi: 10.3390/cells8060520 (PMC6627160; doi:10.3390/cells8060520)
Supplement: Supplementary file 1 [file cells-08-00520-s001.zip › Supplementary Material/Supplementary Figures.docx]

***Supplementary Material***

**Supplementary Figures**

**
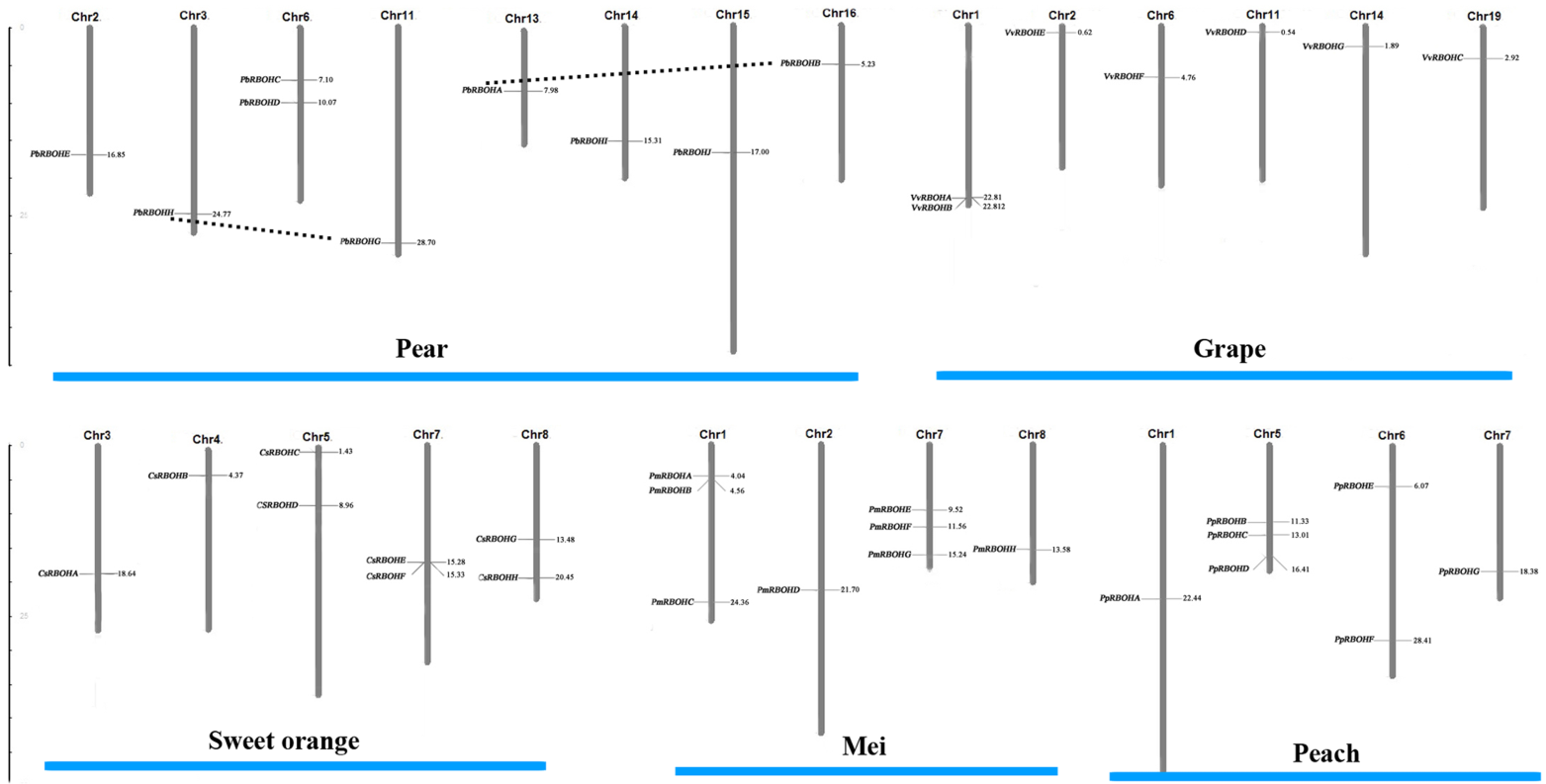
**

**Supplementary Fig. 1 Chromosome map of *RBOH*s** **of the five fruit-producing trees.** The segmental duplicated genes are represented by black lines.

**
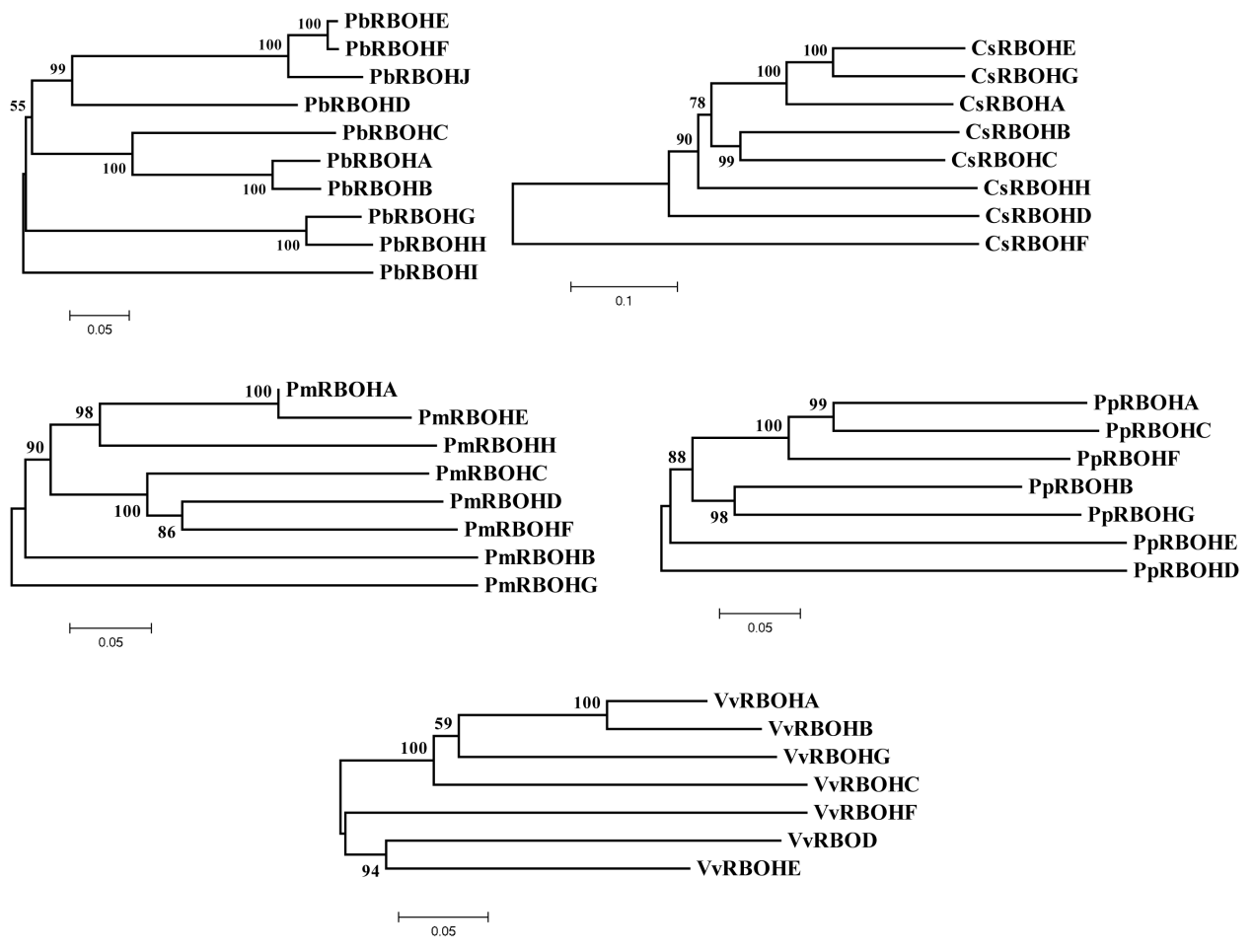
**

**Supplementary Fig. 2 Neighbor-joining tree of *RBOH* family members in five fruit-producing trees.**

**
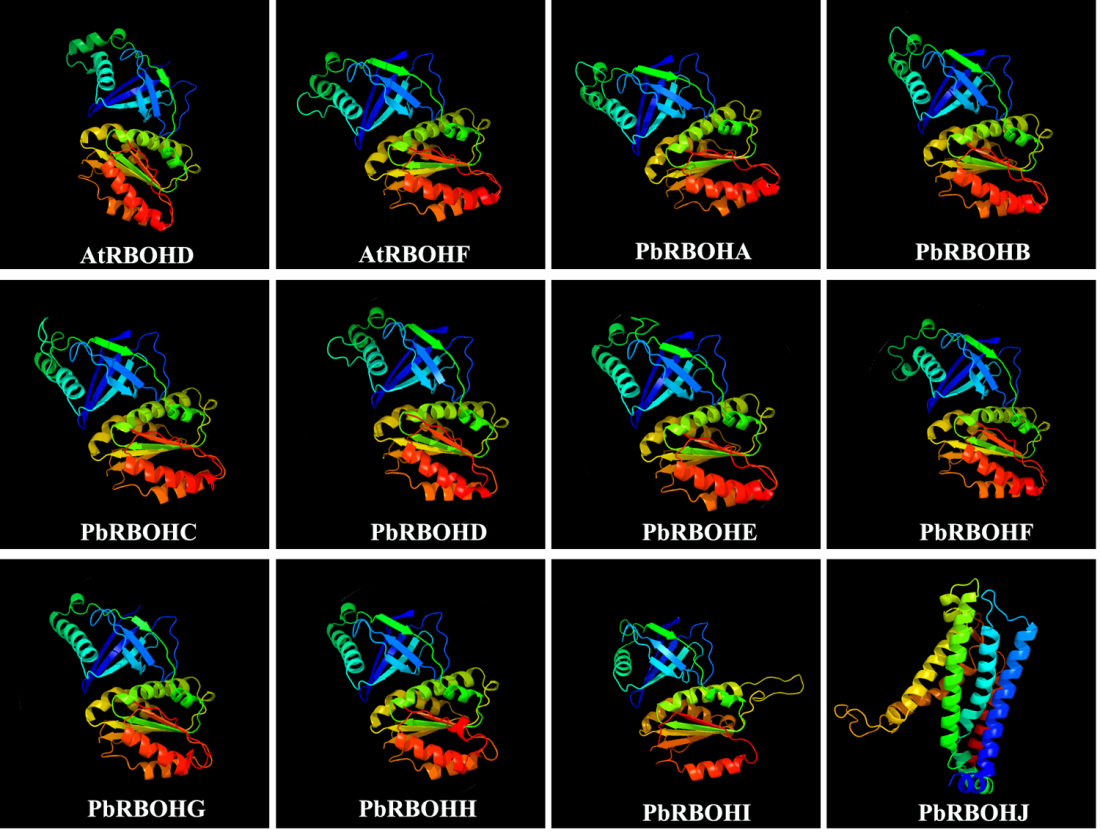
**

**Supplementary Fig. 3 Protein tertiary structures of RBOHs from different species.** AtRBOHD and AtRBOHF are known to be involved in lignification.
