# Supplementary material for: In Silico Genome-Wide Analysis of Respiratory Burst Oxidase Homolog (RBOH) Family Genes in Five Fruit-Producing Trees, and Potential Functional Analysis on Lignification of Stone Cells in Chinese White Pear
Source: Cells. 2019 May 29;8(6):520. doi: 10.3390/cells8060520 (PMC6627160; doi:10.3390/cells8060520)
Supplement: Supplementary file 1 [file cells-08-00520-s001.zip › Supplementary Material/Spreadsheets S1. Interspecies microsynteny of RBOHs.zip!/Pbr018649.1 GSVIVT01028874001.pdf]

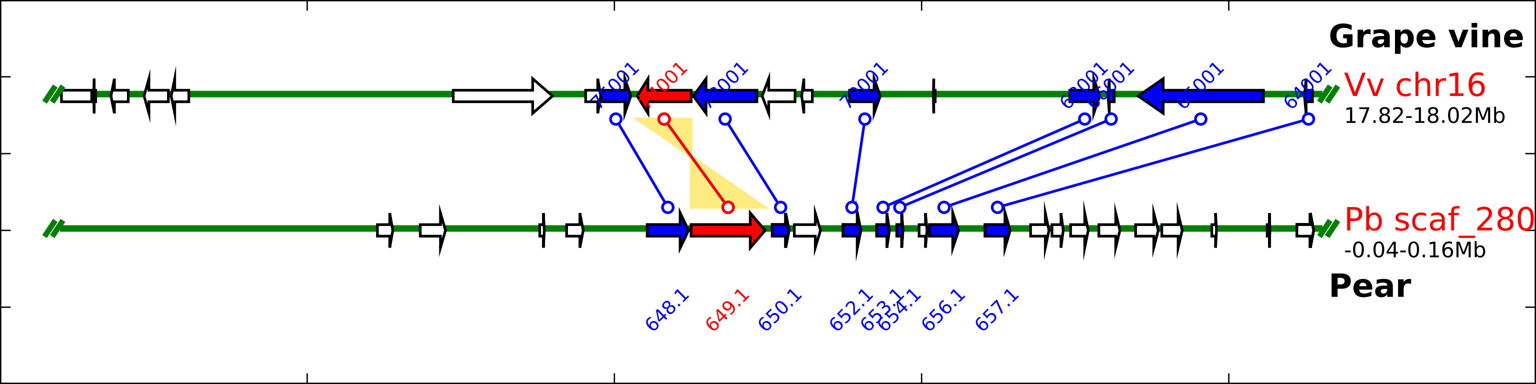
Grape vine
Vv chr16
17.82-18.02Mb
Pb scaf_280
-0.04-0.16Mb
Pear
648.1
649.1
650.1
652.1
653.1
654.1
656.1
657.1
