# Supplementary material for: In Silico Genome-Wide Analysis of Respiratory Burst Oxidase Homolog (RBOH) Family Genes in Five Fruit-Producing Trees, and Potential Functional Analysis on Lignification of Stone Cells in Chinese White Pear
Source: Cells. 2019 May 29;8(6):520. doi: 10.3390/cells8060520 (PMC6627160; doi:10.3390/cells8060520)
Supplement: Supplementary file 1 [file cells-08-00520-s001.zip › Supplementary Material/Spreadsheets S1. Interspecies microsynteny of RBOHs.zip!/Pbr021509.1 GSVIVT01028874001.pdf]

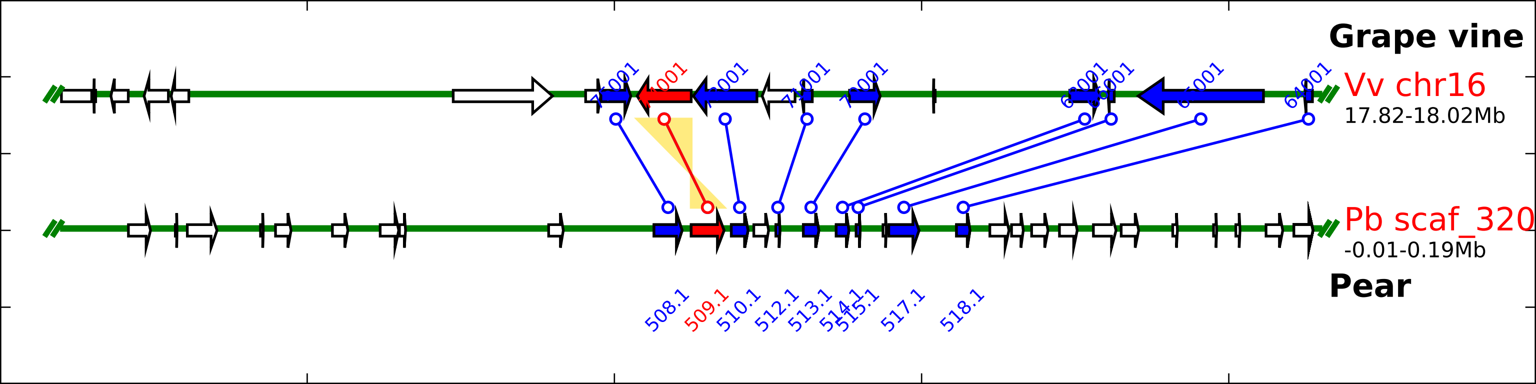
Grape vine
Vv chr16
17.82-18.02Mb
Pb scaf_320
-0.01-0.19Mb
Pear
508.1
509.1
510.1
512.1
513.1
514.1
515.1
517.1
518.1
