# Supplementary material for: In Silico Genome-Wide Analysis of Respiratory Burst Oxidase Homolog (RBOH) Family Genes in Five Fruit-Producing Trees, and Potential Functional Analysis on Lignification of Stone Cells in Chinese White Pear
Source: Cells. 2019 May 29;8(6):520. doi: 10.3390/cells8060520 (PMC6627160; doi:10.3390/cells8060520)
Supplement: Supplementary file 1 [file cells-08-00520-s001.zip › Supplementary Material/Spreadsheets S1. Interspecies microsynteny of RBOHs.zip!/Pbr021576.1 GSVIVT01028873001.pdf]

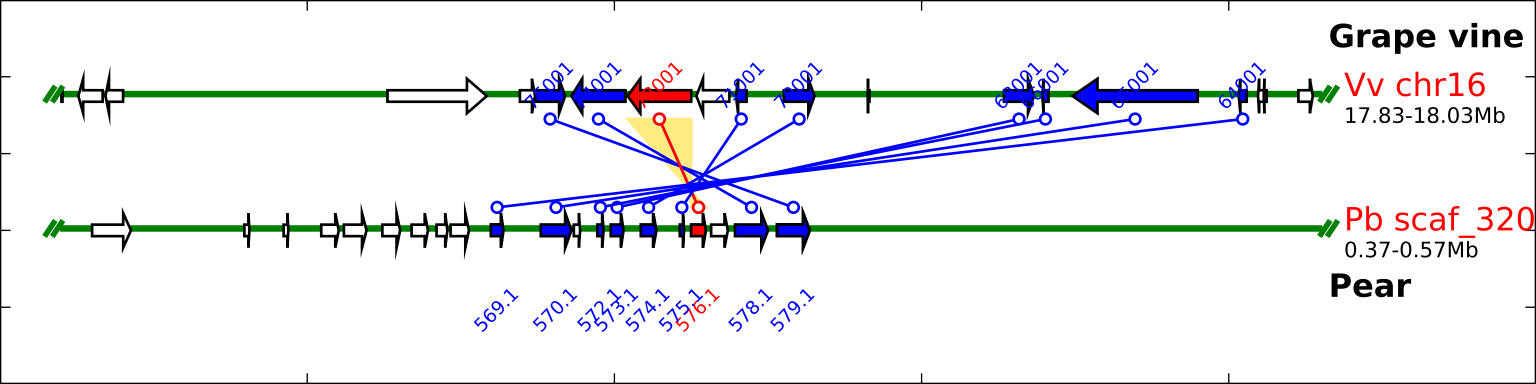
Grape vine
Vv chr16
17.83-18.03Mb
Pb scaf_320
0.37-0.57Mb
Pear
569.1
570.1
572.1
573.1
574.1
575.1
576.1
578.1
579.1
