# Supplementary material for: In Silico Genome-Wide Analysis of Respiratory Burst Oxidase Homolog (RBOH) Family Genes in Five Fruit-Producing Trees, and Potential Functional Analysis on Lignification of Stone Cells in Chinese White Pear
Source: Cells. 2019 May 29;8(6):520. doi: 10.3390/cells8060520 (PMC6627160; doi:10.3390/cells8060520)
Supplement: Supplementary file 1 [file cells-08-00520-s001.zip › Supplementary Material/Spreadsheets S1. Interspecies microsynteny of RBOHs.zip!/orange1.1g002830m Pbr037399.1.pdf]

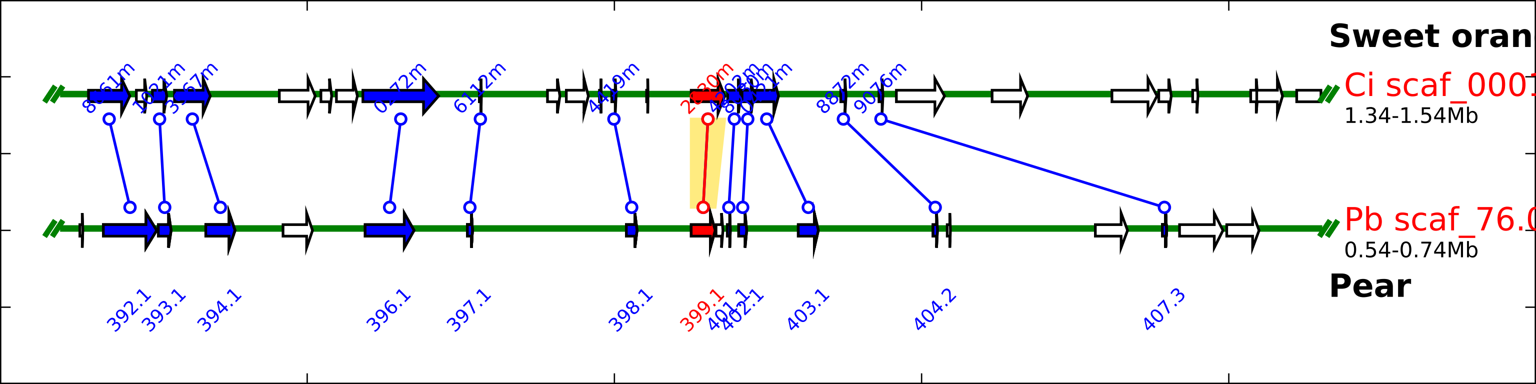
Sweet oran
Ci scaf_0001
1.34-1.54Mb
Pb scaf_76.0
0.54-0.74Mb
Pear
6112m
4419m
8872m
9076m
392.1
393.1
394.1
396.1
397.1
398.1
399.1
401.1
402.1
403.1
404.2
407.3
