# Supplementary material for: In Silico Genome-Wide Analysis of Respiratory Burst Oxidase Homolog (RBOH) Family Genes in Five Fruit-Producing Trees, and Potential Functional Analysis on Lignification of Stone Cells in Chinese White Pear
Source: Cells. 2019 May 29;8(6):520. doi: 10.3390/cells8060520 (PMC6627160; doi:10.3390/cells8060520)
Supplement: Supplementary file 1 [file cells-08-00520-s001.zip › Supplementary Material/Spreadsheets S1. Interspecies microsynteny of RBOHs.zip!/orange1.1g048244mú¿Cs9g19350.1ú⌐ GSVIVT01028873001.pdf]

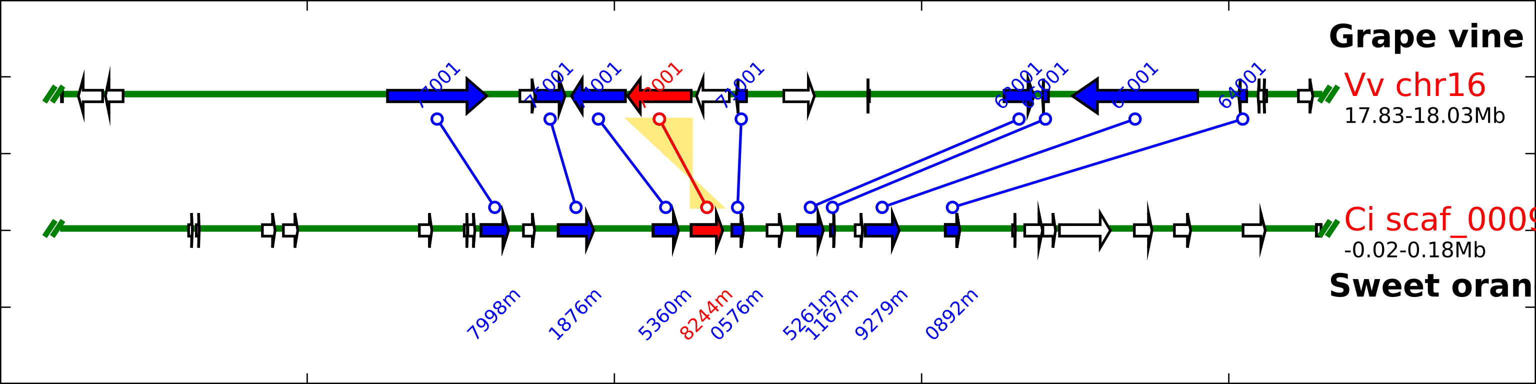
Grape vine
Vv chr16
17.83-18.03Mb
-0.02-0.18Mb
7998m
1876m
5360m
8244m
0576m
5261m
1167m
9279m
0892m
64001
