# Supplementary material for: In Silico Genome-Wide Analysis of Respiratory Burst Oxidase Homolog (RBOH) Family Genes in Five Fruit-Producing Trees, and Potential Functional Analysis on Lignification of Stone Cells in Chinese White Pear
Source: Cells. 2019 May 29;8(6):520. doi: 10.3390/cells8060520 (PMC6627160; doi:10.3390/cells8060520)
Supplement: Supplementary file 1 [file cells-08-00520-s001.zip › Supplementary Material/Spreadsheets S1. Interspecies microsynteny of RBOHs.zip!/ppa000883mú¿Prupe.1G211000.1ú⌐ppa000984mú¿Prupe.5G138300.1ú⌐.pdf]

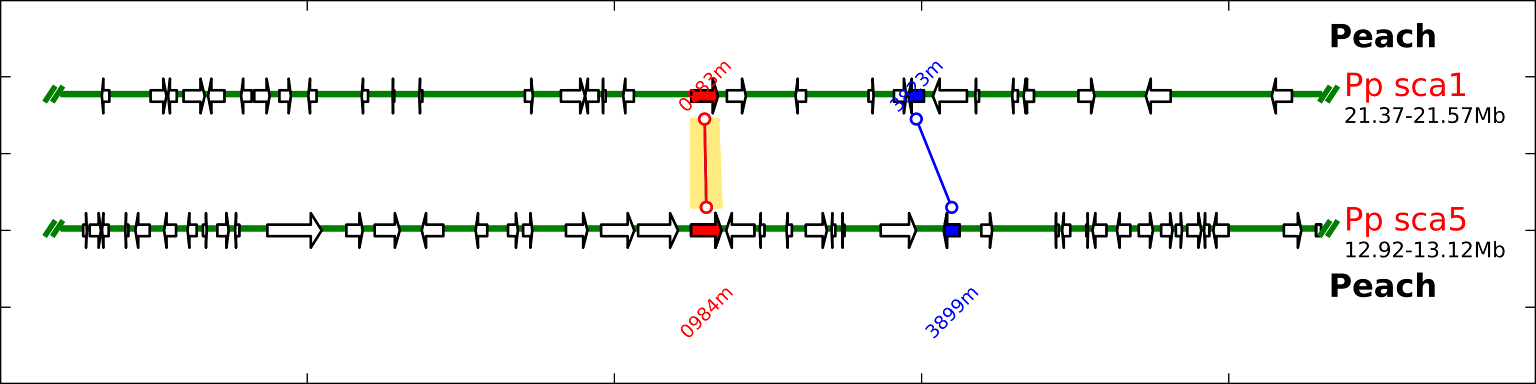

Peach
Pp sca1
21.37-21.57Mb
0983m
3823m
Pp sca5
12.92-13.12Mb
Peach
0984m
3899m
