# Supplementary material for: In Silico Genome-Wide Analysis of Respiratory Burst Oxidase Homolog (RBOH) Family Genes in Five Fruit-Producing Trees, and Potential Functional Analysis on Lignification of Stone Cells in Chinese White Pear
Source: Cells. 2019 May 29;8(6):520. doi: 10.3390/cells8060520 (PMC6627160; doi:10.3390/cells8060520)
Supplement: Supplementary file 1 [file cells-08-00520-s001.zip › Supplementary Material/Spreadsheets S1. Interspecies microsynteny of RBOHs.zip!/ppa005651mú¿Prupe.2G299900.1ú⌐ GSVIVT01028873001.pdf]

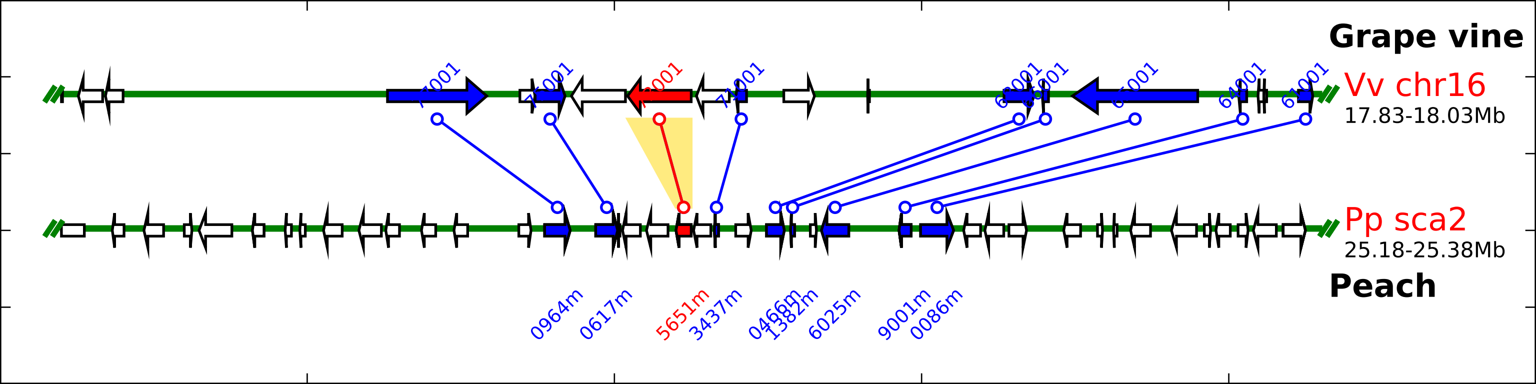

Grape vine
Vv chr16
17.83-18.03Mb
64001
61001
Pp sca2
25.18-25.38Mb
Peach
0964m
0617m
5651m
3437m
0466m
1382m
6025m
9001m
0086m
