# Supplementary material for: In Silico Genome-Wide Analysis of Respiratory Burst Oxidase Homolog (RBOH) Family Genes in Five Fruit-Producing Trees, and Potential Functional Analysis on Lignification of Stone Cells in Chinese White Pear
Source: Cells. 2019 May 29;8(6):520. doi: 10.3390/cells8060520 (PMC6627160; doi:10.3390/cells8060520)
Supplement: Supplementary file 1 [file cells-08-00520-s001.zip › Supplementary Material/Spreadsheets S1. Interspecies microsynteny of RBOHs.zip!/ppa023645mú¿Prupe.6G088800.1ú⌐GSVIVT01025074001.pdf]

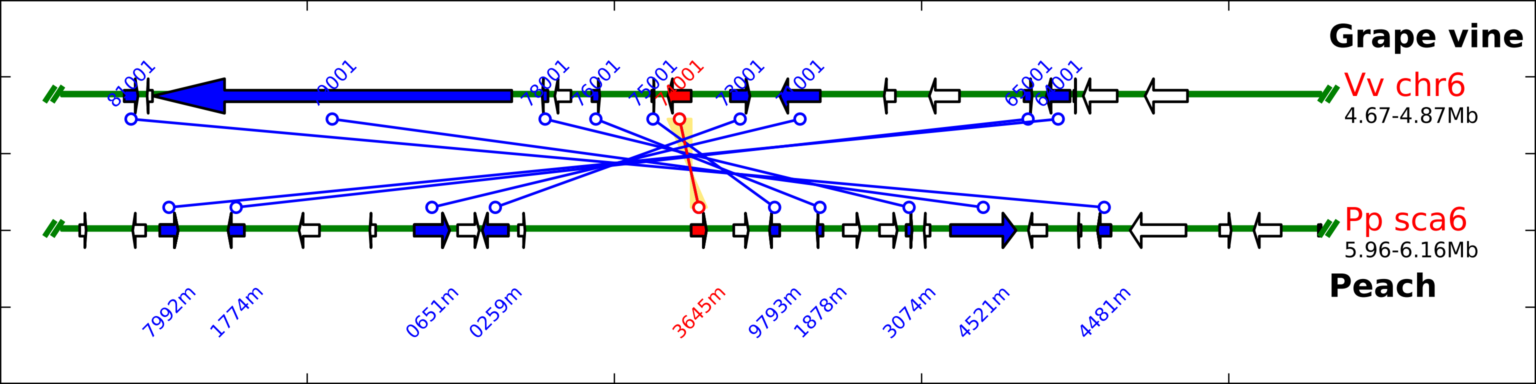

Grape vine
Vv chr6
4.67-4.87Mb
81001
78001
76001
75001
74001
73001
65001
64001
Pp sca6
5.96-6.16Mb
Peach
7992m
1774m
0651m
0259m
3645m
9793m
1878m
3074m
4521m
4481m
