# Supplementary material for: In Silico Genome-Wide Analysis of Respiratory Burst Oxidase Homolog (RBOH) Family Genes in Five Fruit-Producing Trees, and Potential Functional Analysis on Lignification of Stone Cells in Chinese White Pear
Source: Cells. 2019 May 29;8(6):520. doi: 10.3390/cells8060520 (PMC6627160; doi:10.3390/cells8060520)
Supplement: Supplementary file 1 [file cells-08-00520-s001.zip › Supplementary Material/Spreadsheets S2. Numbers of cis-elements in promoter region of PbRBOHs.tar.zip!/PbRBOHF.html]

Content-Type: text/html; charset=ISO-8859-1


PlantCARE


Webmaster Firefox specific output  
To save the result:
click on the frame with the right mouse button and save the source code as a text file with extension .html  
REFERENCE:PlantCARE: a database of plant cis-acting regulatory elements and a portal to tools for in silico analysis of promoter sequences.  
Lescot, M., Déhais, P., Moreau, Y., De Moor, B., Rouzé ,P.,and Rombauts, S.  
Nucleic Acids Res., Database issue(2002), 30(1):325-327.   


---

>003403   
+ TAAAAATAGT GAAAGAACCA CCGGCCGGAT AAACAAGAGT AAAGCTGTCT GAAAATAAAC CCTACTCACA   
  
  
+ CACATGGTAT TTCTCATATT TTATTTTATA GTCAATTATT AAGATAATAA GCATTCTAAC CATTTATGTA   
  
  
+ TACATGTTCA TACCGCGAGC GTTGCTCGGA GGAACACATT TTTGTATCAA GTGATGTTGC AAAATGATGC   
  
  
+ ATACAATCAG TACACATCAC TCTAGCTACT TACTCCATCT TGTAGTATTG CAATGCAGTA AAATATAGCA   
  
  
+ CTAGTCCCAG ATTAATTATT ATGGTTCTCC TTTCAAACAC ATAGTTAATG GTTATAATGA TATACTTAAG   
  
  
+ GGCAAAATAT ATTACTTATA CAAAATAACT CGATGGGTTA CGTAAAATAC ATGAAGAATT CAGTTTAATA   
  
  
+ AGTTTAGTAT ATGCTTGCAT GAGTATGATA CAATATATAT GTGACAAAAT AAAATACAAG TCTAAATTTA   
  
  
+ CGACACCACC ACCCCCTTAT ATAGAGTTAA AACGTGAGAT TCAAGAGTGT AGTTGGTGGA AAAAGATAAA   
  
  
+ TTGTAGGGTT ATTGTCTTGT AGAAATACAT ATACATAAAT ATTTATCTGT ACAAGATGTG CGTATGTACA   
  
  
+ ATAATCATTG ACAATTAACC TAAACATTGC AATTAACATA AACTTTGTTT CTTCTTAAAT TTTAGATGGT   
  
  
+ AAGTTCCTCC TATAAAATCA TTACAAAATC ATAAAATCCC AATAACACAC TTTATCTATA CGCAAAAAAA   
  
  
+ TTTGCCGGGG AAAATATAGT ACGTGGAGGT ATATATAGTA CGTACTTTCT CCTTTGCTTT CAAAGATCAT   
  
  
+ CAAATTTTAA TTAGTTTATA TTATTTTATA TATACACACA CACACACACA CACAAAGTGT GTGAGACATT   
  
  
+ TTTTTTATTT TTGTTTTTGA AATAGAAGGA GAGAGGAAGA GAGTGAATTA GATTTTTTTT TTTTTTTAAT   
  
  
+ TAAAGATATG TTAAGATTAC ATGTAGGTGA GATTTTGAAA AAAAAAACAG CCAAATTTAG TTATGTGAAA   
  
  
+ TTACATTTTT GCCCCGTATT TATTATTCAT ATTTTGTTTT AATTAGATGG TTAAAGTGGT AATTTGATAG   
  
  
+ GATTTTAGTT TACAATGAGT GTTTTATTAA TTAGTAGATA TATATTAAGT ATGTATTTTC AATTAAAAAT   
  
  
+ AAAGAAAAAA AAGCAGCATC TAGCATGGCA AGCACAACAC AAGCAGATAT ATATATATAT ATATATATAT   
  
  
+ ATAATTGGAA AAGTATTGTT GTCGTGGAGA CAAATTTAGG TTCTTGAGCA AAAACTCAAC TACTCTCTAG   
  
  
+ AGAGAGAGAG AGAGAGAGGT AGCAGTAACA CGACTTGTGT AGAAATTGTG AGGGACGAAT CGGTTAAGAA   
  
  
+ GTGCATGTCT TGTATCTCTC TCTAGAGCTA GAAAAATCCT AAGTGTTTCA TTTTTTTTGG AACAAACACA   
  
  
+ GAAAACCCCA AGAATTATAA TAATTAAAC  

- ATTTTTATCA CTTTCTTGGT GGCCGGCCTA TTTGTTCTCA TTTCGACAGA CTTTTATTTG GGATGAGTGT   
  
  
- GTGTACCATA AAGAGTATAA AATAAAATAT CAGTTAATAA TTCTATTATT CGTAAGATTG GTAAATACAT   
  
  
- ATGTACAAGT ATGGCGCTCG CAACGAGCCT CCTTGTGTAA AAACATAGTT CACTACAACG TTTTACTACG   
  
  
- TATGTTAGTC ATGTGTAGTG AGATCGATGA ATGAGGTAGA ACATCATAAC GTTACGTCAT TTTATATCGT   
  
  
- GATCAGGGTC TAATTAATAA TACCAAGAGG AAAGTTTGTG TATCAATTAC CAATATTACT ATATGAATTC   
  
  
- CCGTTTTATA TAATGAATAT GTTTTATTGA GCTACCCAAT GCATTTTATG TACTTCTTAA GTCAAATTAT   
  
  
- TCAAATCATA TACGAACGTA CTCATACTAT GTTATATATA CACTGTTTTA TTTTATGTTC AGATTTAAAT   
  
  
- GCTGTGGTGG TGGGGGAATA TATCTCAATT TTGCACTCTA AGTTCTCACA TCAACCACCT TTTTCTATTT   
  
  
- AACATCCCAA TAACAGAACA TCTTTATGTA TATGTATTTA TAAATAGACA TGTTCTACAC GCATACATGT   
  
  
- TATTAGTAAC TGTTAATTGG ATTTGTAACG TTAATTGTAT TTGAAACAAA GAAGAATTTA AAATCTACCA   
  
  
- TTCAAGGAGG ATATTTTAGT AATGTTTTAG TATTTTAGGG TTATTGTGTG AAATAGATAT GCGTTTTTTT   
  
  
- AAACGGCCCC TTTTATATCA TGCACCTCCA TATATATCAT GCATGAAAGA GGAAACGAAA GTTTCTAGTA   
  
  
- GTTTAAAATT AATCAAATAT AATAAAATAT ATATGTGTGT GTGTGTGTGT GTGTTTCACA CACTCTGTAA   
  
  
- AAAAAATAAA AACAAAAACT TTATCTTCCT CTCTCCTTCT CTCACTTAAT CTAAAAAAAA AAAAAAATTA   
  
  
- ATTTCTATAC AATTCTAATG TACATCCACT CTAAAACTTT TTTTTTTGTC GGTTTAAATC AATACACTTT   
  
  
- AATGTAAAAA CGGGGCATAA ATAATAAGTA TAAAACAAAA TTAATCTACC AATTTCACCA TTAAACTATC   
  
  
- CTAAAATCAA ATGTTACTCA CAAAATAATT AATCATCTAT ATATAATTCA TACATAAAAG TTAATTTTTA   
  
  
- TTTCTTTTTT TTCGTCGTAG ATCGTACCGT TCGTGTTGTG TTCGTCTATA TATATATATA TATATATATA   
  
  
- TATTAACCTT TTCATAACAA CAGCACCTCT GTTTAAATCC AAGAACTCGT TTTTGAGTTG ATGAGAGATC   
  
  
- TCTCTCTCTC TCTCTCTCCA TCGTCATTGT GCTGAACACA TCTTTAACAC TCCCTGCTTA GCCAATTCTT   
  
  
- CACGTACAGA ACATAGAGAG AGATCTCGAT CTTTTTAGGA TTCACAAAGT AAAAAAAACC TTGTTTGTGT   
  
  
- CTTTTGGGGT TCTTAATATT ATTAATTTG

  
  
Motifs Found  

+   

| Site Name | Organism | Position | Strand | Matrix score. | sequence | function |
| --- | --- | --- | --- | --- | --- | --- |
|  | organism | 144 | + | 4 | motif\_sequence | short\_function |
|  | organism | 946 | - | 4 | motif\_sequence | short\_function |
|  | organism | 616 | + | 4 | motif\_sequence | short\_function |
|  | organism | 1154 | - | 4 | motif\_sequence | short\_function |
|  | organism | 607 | + | 4 | motif\_sequence | short\_function |
|  | organism | 938 | - | 4 | motif\_sequence | short\_function |
|  | organism | 624 | + | 4 | motif\_sequence | short\_function |
|  | organism | 1233 | - | 4 | motif\_sequence | short\_function |

>003403   
+ TAAAAATAGT GAAAGAACCA CCGGCCGGAT AAACAAGAGT AAAGCTGTCT GAAAATAAAC CCTACTCACA   
  
  
+ CACATGGTAT TTCTCATATT TTATTTTATA GTCAATTATT AAGATAATAA GCATTCTAAC CATTTATGTA   
  
  
+ TACATGTTCA TACCGCGAGC GTTGCTCGGA GGAACACATT TTTGTATCAA GTGATGTTGC AAAATGATGC   
  
  
+ ATACAATCAG TACACATCAC TCTAGCTACT TACTCCATCT TGTAGTATTG CAATGCAGTA AAATATAGCA   
  
  
+ CTAGTCCCAG ATTAATTATT ATGGTTCTCC TTTCAAACAC ATAGTTAATG GTTATAATGA TATACTTAAG   
  
  
+ GGCAAAATAT ATTACTTATA CAAAATAACT CGATGGGTTA CGTAAAATAC ATGAAGAATT CAGTTTAATA   
  
  
+ AGTTTAGTAT ATGCTTGCAT GAGTATGATA CAATATATAT GTGACAAAAT AAAATACAAG TCTAAATTTA   
  
  
+ CGACACCACC ACCCCCTTAT ATAGAGTTAA AACGTGAGAT TCAAGAGTGT AGTTGGTGGA AAAAGATAAA   
  
  
+ TTGTAGGGTT ATTGTCTTGT AGAAATACAT ATACATAAAT ATTTATCTGT ACAAGATGTG CGTATGTACA   
  
  
+ ATAATCATTG ACAATTAACC TAAACATTGC AATTAACATA AACTTTGTTT CTTCTTAAAT TTTAGATGGT   
  
  
+ AAGTTCCTCC TATAAAATCA TTACAAAATC ATAAAATCCC AATAACACAC TTTATCTATA CGCAAAAAAA   
  
  
+ TTTGCCGGGG AAAATATAGT ACGTGGAGGT ATATATAGTA CGTACTTTCT CCTTTGCTTT CAAAGATCAT   
  
  
+ CAAATTTTAA TTAGTTTATA TTATTTTATA TATACACACA CACACACACA CACAAAGTGT GTGAGACATT   
  
  
+ TTTTTTATTT TTGTTTTTGA AATAGAAGGA GAGAGGAAGA GAGTGAATTA GATTTTTTTT TTTTTTTAAT   
  
  
+ TAAAGATATG TTAAGATTAC ATGTAGGTGA GATTTTGAAA AAAAAAACAG CCAAATTTAG TTATGTGAAA   
  
  
+ TTACATTTTT GCCCCGTATT TATTATTCAT ATTTTGTTTT AATTAGATGG TTAAAGTGGT AATTTGATAG   
  
  
+ GATTTTAGTT TACAATGAGT GTTTTATTAA TTAGTAGATA TATATTAAGT ATGTATTTTC AATTAAAAAT   
  
  
+ AAAGAAAAAA AAGCAGCATC TAGCATGGCA AGCACAACAC AAGCAGATAT ATATATATAT ATATATATAT   
  
  
+ ATAATTGGAA AAGTATTGTT GTCGTGGAGA CAAATTTAGG TTCTTGAGCA AAAACTCAAC TACTCTCTAG   
  
  
+ AGAGAGAGAG AGAGAGAGGT AGCAGTAACA CGACTTGTGT AGAAATTGTG AGGGACGAAT CGGTTAAGAA   
  
  
+ GTGCATGTCT TGTATCTCTC TCTAGAGCTA GAAAAATCCT AAGTGTTTCA TTTTTTTTGG AACAAACACA   
  
  
+ GAAAACCCCA AGAATTATAA TAATTAAAC  

- ATTTTTATCA CTTTCTTGGT GGCCGGCCTA TTTGTTCTCA TTTCGACAGA CTTTTATTTG GGATGAGTGT   
  
  
- GTGTACCATA AAGAGTATAA AATAAAATAT CAGTTAATAA TTCTATTATT CGTAAGATTG GTAAATACAT   
  
  
- ATGTACAAGT ATGGCGCTCG CAACGAGCCT CCTTGTGTAA AAACATAGTT CACTACAACG TTTTACTACG   
  
  
- TATGTTAGTC ATGTGTAGTG AGATCGATGA ATGAGGTAGA ACATCATAAC GTTACGTCAT TTTATATCGT   
  
  
- GATCAGGGTC TAATTAATAA TACCAAGAGG AAAGTTTGTG TATCAATTAC CAATATTACT ATATGAATTC   
  
  
- CCGTTTTATA TAATGAATAT GTTTTATTGA GCTACCCAAT GCATTTTATG TACTTCTTAA GTCAAATTAT   
  
  
- TCAAATCATA TACGAACGTA CTCATACTAT GTTATATATA CACTGTTTTA TTTTATGTTC AGATTTAAAT   
  
  
- GCTGTGGTGG TGGGGGAATA TATCTCAATT TTGCACTCTA AGTTCTCACA TCAACCACCT TTTTCTATTT   
  
  
- AACATCCCAA TAACAGAACA TCTTTATGTA TATGTATTTA TAAATAGACA TGTTCTACAC GCATACATGT   
  
  
- TATTAGTAAC TGTTAATTGG ATTTGTAACG TTAATTGTAT TTGAAACAAA GAAGAATTTA AAATCTACCA   
  
  
- TTCAAGGAGG ATATTTTAGT AATGTTTTAG TATTTTAGGG TTATTGTGTG AAATAGATAT GCGTTTTTTT   
  
  
- AAACGGCCCC TTTTATATCA TGCACCTCCA TATATATCAT GCATGAAAGA GGAAACGAAA GTTTCTAGTA   
  
  
- GTTTAAAATT AATCAAATAT AATAAAATAT ATATGTGTGT GTGTGTGTGT GTGTTTCACA CACTCTGTAA   
  
  
- AAAAAATAAA AACAAAAACT TTATCTTCCT CTCTCCTTCT CTCACTTAAT CTAAAAAAAA AAAAAAATTA   
  
  
- ATTTCTATAC AATTCTAATG TACATCCACT CTAAAACTTT TTTTTTTGTC GGTTTAAATC AATACACTTT   
  
  
- AATGTAAAAA CGGGGCATAA ATAATAAGTA TAAAACAAAA TTAATCTACC AATTTCACCA TTAAACTATC   
  
  
- CTAAAATCAA ATGTTACTCA CAAAATAATT AATCATCTAT ATATAATTCA TACATAAAAG TTAATTTTTA   
  
  
- TTTCTTTTTT TTCGTCGTAG ATCGTACCGT TCGTGTTGTG TTCGTCTATA TATATATATA TATATATATA   
  
  
- TATTAACCTT TTCATAACAA CAGCACCTCT GTTTAAATCC AAGAACTCGT TTTTGAGTTG ATGAGAGATC   
  
  
- TCTCTCTCTC TCTCTCTCCA TCGTCATTGT GCTGAACACA TCTTTAACAC TCCCTGCTTA GCCAATTCTT   
  
  
- CACGTACAGA ACATAGAGAG AGATCTCGAT CTTTTTAGGA TTCACAAAGT AAAAAAAACC TTGTTTGTGT   
  
  
- CTTTTGGGGT TCTTAATATT ATTAATTTG

+     AAGAA-motif

| Site Name | Organism | Position | Strand | Matrix score. | sequence | function |
| --- | --- | --- | --- | --- | --- | --- |
| AAGAA-motif | Avena sativa | 11 | + | 7 | GAAAGAA |  |

>003403   
+ TAAAAATAGT GAAAGAACCA CCGGCCGGAT AAACAAGAGT AAAGCTGTCT GAAAATAAAC CCTACTCACA   
  
  
+ CACATGGTAT TTCTCATATT TTATTTTATA GTCAATTATT AAGATAATAA GCATTCTAAC CATTTATGTA   
  
  
+ TACATGTTCA TACCGCGAGC GTTGCTCGGA GGAACACATT TTTGTATCAA GTGATGTTGC AAAATGATGC   
  
  
+ ATACAATCAG TACACATCAC TCTAGCTACT TACTCCATCT TGTAGTATTG CAATGCAGTA AAATATAGCA   
  
  
+ CTAGTCCCAG ATTAATTATT ATGGTTCTCC TTTCAAACAC ATAGTTAATG GTTATAATGA TATACTTAAG   
  
  
+ GGCAAAATAT ATTACTTATA CAAAATAACT CGATGGGTTA CGTAAAATAC ATGAAGAATT CAGTTTAATA   
  
  
+ AGTTTAGTAT ATGCTTGCAT GAGTATGATA CAATATATAT GTGACAAAAT AAAATACAAG TCTAAATTTA   
  
  
+ CGACACCACC ACCCCCTTAT ATAGAGTTAA AACGTGAGAT TCAAGAGTGT AGTTGGTGGA AAAAGATAAA   
  
  
+ TTGTAGGGTT ATTGTCTTGT AGAAATACAT ATACATAAAT ATTTATCTGT ACAAGATGTG CGTATGTACA   
  
  
+ ATAATCATTG ACAATTAACC TAAACATTGC AATTAACATA AACTTTGTTT CTTCTTAAAT TTTAGATGGT   
  
  
+ AAGTTCCTCC TATAAAATCA TTACAAAATC ATAAAATCCC AATAACACAC TTTATCTATA CGCAAAAAAA   
  
  
+ TTTGCCGGGG AAAATATAGT ACGTGGAGGT ATATATAGTA CGTACTTTCT CCTTTGCTTT CAAAGATCAT   
  
  
+ CAAATTTTAA TTAGTTTATA TTATTTTATA TATACACACA CACACACACA CACAAAGTGT GTGAGACATT   
  
  
+ TTTTTTATTT TTGTTTTTGA AATAGAAGGA GAGAGGAAGA GAGTGAATTA GATTTTTTTT TTTTTTTAAT   
  
  
+ TAAAGATATG TTAAGATTAC ATGTAGGTGA GATTTTGAAA AAAAAAACAG CCAAATTTAG TTATGTGAAA   
  
  
+ TTACATTTTT GCCCCGTATT TATTATTCAT ATTTTGTTTT AATTAGATGG TTAAAGTGGT AATTTGATAG   
  
  
+ GATTTTAGTT TACAATGAGT GTTTTATTAA TTAGTAGATA TATATTAAGT ATGTATTTTC AATTAAAAAT   
  
  
+ AAAGAAAAAA AAGCAGCATC TAGCATGGCA AGCACAACAC AAGCAGATAT ATATATATAT ATATATATAT   
  
  
+ ATAATTGGAA AAGTATTGTT GTCGTGGAGA CAAATTTAGG TTCTTGAGCA AAAACTCAAC TACTCTCTAG   
  
  
+ AGAGAGAGAG AGAGAGAGGT AGCAGTAACA CGACTTGTGT AGAAATTGTG AGGGACGAAT CGGTTAAGAA   
  
  
+ GTGCATGTCT TGTATCTCTC TCTAGAGCTA GAAAAATCCT AAGTGTTTCA TTTTTTTTGG AACAAACACA   
  
  
+ GAAAACCCCA AGAATTATAA TAATTAAAC  

- ATTTTTATCA CTTTCTTGGT GGCCGGCCTA TTTGTTCTCA TTTCGACAGA CTTTTATTTG GGATGAGTGT   
  
  
- GTGTACCATA AAGAGTATAA AATAAAATAT CAGTTAATAA TTCTATTATT CGTAAGATTG GTAAATACAT   
  
  
- ATGTACAAGT ATGGCGCTCG CAACGAGCCT CCTTGTGTAA AAACATAGTT CACTACAACG TTTTACTACG   
  
  
- TATGTTAGTC ATGTGTAGTG AGATCGATGA ATGAGGTAGA ACATCATAAC GTTACGTCAT TTTATATCGT   
  
  
- GATCAGGGTC TAATTAATAA TACCAAGAGG AAAGTTTGTG TATCAATTAC CAATATTACT ATATGAATTC   
  
  
- CCGTTTTATA TAATGAATAT GTTTTATTGA GCTACCCAAT GCATTTTATG TACTTCTTAA GTCAAATTAT   
  
  
- TCAAATCATA TACGAACGTA CTCATACTAT GTTATATATA CACTGTTTTA TTTTATGTTC AGATTTAAAT   
  
  
- GCTGTGGTGG TGGGGGAATA TATCTCAATT TTGCACTCTA AGTTCTCACA TCAACCACCT TTTTCTATTT   
  
  
- AACATCCCAA TAACAGAACA TCTTTATGTA TATGTATTTA TAAATAGACA TGTTCTACAC GCATACATGT   
  
  
- TATTAGTAAC TGTTAATTGG ATTTGTAACG TTAATTGTAT TTGAAACAAA GAAGAATTTA AAATCTACCA   
  
  
- TTCAAGGAGG ATATTTTAGT AATGTTTTAG TATTTTAGGG TTATTGTGTG AAATAGATAT GCGTTTTTTT   
  
  
- AAACGGCCCC TTTTATATCA TGCACCTCCA TATATATCAT GCATGAAAGA GGAAACGAAA GTTTCTAGTA   
  
  
- GTTTAAAATT AATCAAATAT AATAAAATAT ATATGTGTGT GTGTGTGTGT GTGTTTCACA CACTCTGTAA   
  
  
- AAAAAATAAA AACAAAAACT TTATCTTCCT CTCTCCTTCT CTCACTTAAT CTAAAAAAAA AAAAAAATTA   
  
  
- ATTTCTATAC AATTCTAATG TACATCCACT CTAAAACTTT TTTTTTTGTC GGTTTAAATC AATACACTTT   
  
  
- AATGTAAAAA CGGGGCATAA ATAATAAGTA TAAAACAAAA TTAATCTACC AATTTCACCA TTAAACTATC   
  
  
- CTAAAATCAA ATGTTACTCA CAAAATAATT AATCATCTAT ATATAATTCA TACATAAAAG TTAATTTTTA   
  
  
- TTTCTTTTTT TTCGTCGTAG ATCGTACCGT TCGTGTTGTG TTCGTCTATA TATATATATA TATATATATA   
  
  
- TATTAACCTT TTCATAACAA CAGCACCTCT GTTTAAATCC AAGAACTCGT TTTTGAGTTG ATGAGAGATC   
  
  
- TCTCTCTCTC TCTCTCTCCA TCGTCATTGT GCTGAACACA TCTTTAACAC TCCCTGCTTA GCCAATTCTT   
  
  
- CACGTACAGA ACATAGAGAG AGATCTCGAT CTTTTTAGGA TTCACAAAGT AAAAAAAACC TTGTTTGTGT   
  
  
- CTTTTGGGGT TCTTAATATT ATTAATTTG

+     ABRE

| Site Name | Organism | Position | Strand | Matrix score. | sequence | function |
| --- | --- | --- | --- | --- | --- | --- |
| ABRE | Arabidopsis thaliana | 522 | + | 5 | ACGTG | cis-acting element involved in the abscisic acid responsiveness |
| ABRE | Arabidopsis thaliana | 791 | + | 5 | ACGTG | cis-acting element involved in the abscisic acid responsiveness |

>003403   
+ TAAAAATAGT GAAAGAACCA CCGGCCGGAT AAACAAGAGT AAAGCTGTCT GAAAATAAAC CCTACTCACA   
  
  
+ CACATGGTAT TTCTCATATT TTATTTTATA GTCAATTATT AAGATAATAA GCATTCTAAC CATTTATGTA   
  
  
+ TACATGTTCA TACCGCGAGC GTTGCTCGGA GGAACACATT TTTGTATCAA GTGATGTTGC AAAATGATGC   
  
  
+ ATACAATCAG TACACATCAC TCTAGCTACT TACTCCATCT TGTAGTATTG CAATGCAGTA AAATATAGCA   
  
  
+ CTAGTCCCAG ATTAATTATT ATGGTTCTCC TTTCAAACAC ATAGTTAATG GTTATAATGA TATACTTAAG   
  
  
+ GGCAAAATAT ATTACTTATA CAAAATAACT CGATGGGTTA CGTAAAATAC ATGAAGAATT CAGTTTAATA   
  
  
+ AGTTTAGTAT ATGCTTGCAT GAGTATGATA CAATATATAT GTGACAAAAT AAAATACAAG TCTAAATTTA   
  
  
+ CGACACCACC ACCCCCTTAT ATAGAGTTAA AACGTGAGAT TCAAGAGTGT AGTTGGTGGA AAAAGATAAA   
  
  
+ TTGTAGGGTT ATTGTCTTGT AGAAATACAT ATACATAAAT ATTTATCTGT ACAAGATGTG CGTATGTACA   
  
  
+ ATAATCATTG ACAATTAACC TAAACATTGC AATTAACATA AACTTTGTTT CTTCTTAAAT TTTAGATGGT   
  
  
+ AAGTTCCTCC TATAAAATCA TTACAAAATC ATAAAATCCC AATAACACAC TTTATCTATA CGCAAAAAAA   
  
  
+ TTTGCCGGGG AAAATATAGT ACGTGGAGGT ATATATAGTA CGTACTTTCT CCTTTGCTTT CAAAGATCAT   
  
  
+ CAAATTTTAA TTAGTTTATA TTATTTTATA TATACACACA CACACACACA CACAAAGTGT GTGAGACATT   
  
  
+ TTTTTTATTT TTGTTTTTGA AATAGAAGGA GAGAGGAAGA GAGTGAATTA GATTTTTTTT TTTTTTTAAT   
  
  
+ TAAAGATATG TTAAGATTAC ATGTAGGTGA GATTTTGAAA AAAAAAACAG CCAAATTTAG TTATGTGAAA   
  
  
+ TTACATTTTT GCCCCGTATT TATTATTCAT ATTTTGTTTT AATTAGATGG TTAAAGTGGT AATTTGATAG   
  
  
+ GATTTTAGTT TACAATGAGT GTTTTATTAA TTAGTAGATA TATATTAAGT ATGTATTTTC AATTAAAAAT   
  
  
+ AAAGAAAAAA AAGCAGCATC TAGCATGGCA AGCACAACAC AAGCAGATAT ATATATATAT ATATATATAT   
  
  
+ ATAATTGGAA AAGTATTGTT GTCGTGGAGA CAAATTTAGG TTCTTGAGCA AAAACTCAAC TACTCTCTAG   
  
  
+ AGAGAGAGAG AGAGAGAGGT AGCAGTAACA CGACTTGTGT AGAAATTGTG AGGGACGAAT CGGTTAAGAA   
  
  
+ GTGCATGTCT TGTATCTCTC TCTAGAGCTA GAAAAATCCT AAGTGTTTCA TTTTTTTTGG AACAAACACA   
  
  
+ GAAAACCCCA AGAATTATAA TAATTAAAC  

- ATTTTTATCA CTTTCTTGGT GGCCGGCCTA TTTGTTCTCA TTTCGACAGA CTTTTATTTG GGATGAGTGT   
  
  
- GTGTACCATA AAGAGTATAA AATAAAATAT CAGTTAATAA TTCTATTATT CGTAAGATTG GTAAATACAT   
  
  
- ATGTACAAGT ATGGCGCTCG CAACGAGCCT CCTTGTGTAA AAACATAGTT CACTACAACG TTTTACTACG   
  
  
- TATGTTAGTC ATGTGTAGTG AGATCGATGA ATGAGGTAGA ACATCATAAC GTTACGTCAT TTTATATCGT   
  
  
- GATCAGGGTC TAATTAATAA TACCAAGAGG AAAGTTTGTG TATCAATTAC CAATATTACT ATATGAATTC   
  
  
- CCGTTTTATA TAATGAATAT GTTTTATTGA GCTACCCAAT GCATTTTATG TACTTCTTAA GTCAAATTAT   
  
  
- TCAAATCATA TACGAACGTA CTCATACTAT GTTATATATA CACTGTTTTA TTTTATGTTC AGATTTAAAT   
  
  
- GCTGTGGTGG TGGGGGAATA TATCTCAATT TTGCACTCTA AGTTCTCACA TCAACCACCT TTTTCTATTT   
  
  
- AACATCCCAA TAACAGAACA TCTTTATGTA TATGTATTTA TAAATAGACA TGTTCTACAC GCATACATGT   
  
  
- TATTAGTAAC TGTTAATTGG ATTTGTAACG TTAATTGTAT TTGAAACAAA GAAGAATTTA AAATCTACCA   
  
  
- TTCAAGGAGG ATATTTTAGT AATGTTTTAG TATTTTAGGG TTATTGTGTG AAATAGATAT GCGTTTTTTT   
  
  
- AAACGGCCCC TTTTATATCA TGCACCTCCA TATATATCAT GCATGAAAGA GGAAACGAAA GTTTCTAGTA   
  
  
- GTTTAAAATT AATCAAATAT AATAAAATAT ATATGTGTGT GTGTGTGTGT GTGTTTCACA CACTCTGTAA   
  
  
- AAAAAATAAA AACAAAAACT TTATCTTCCT CTCTCCTTCT CTCACTTAAT CTAAAAAAAA AAAAAAATTA   
  
  
- ATTTCTATAC AATTCTAATG TACATCCACT CTAAAACTTT TTTTTTTGTC GGTTTAAATC AATACACTTT   
  
  
- AATGTAAAAA CGGGGCATAA ATAATAAGTA TAAAACAAAA TTAATCTACC AATTTCACCA TTAAACTATC   
  
  
- CTAAAATCAA ATGTTACTCA CAAAATAATT AATCATCTAT ATATAATTCA TACATAAAAG TTAATTTTTA   
  
  
- TTTCTTTTTT TTCGTCGTAG ATCGTACCGT TCGTGTTGTG TTCGTCTATA TATATATATA TATATATATA   
  
  
- TATTAACCTT TTCATAACAA CAGCACCTCT GTTTAAATCC AAGAACTCGT TTTTGAGTTG ATGAGAGATC   
  
  
- TCTCTCTCTC TCTCTCTCCA TCGTCATTGT GCTGAACACA TCTTTAACAC TCCCTGCTTA GCCAATTCTT   
  
  
- CACGTACAGA ACATAGAGAG AGATCTCGAT CTTTTTAGGA TTCACAAAGT AAAAAAAACC TTGTTTGTGT   
  
  
- CTTTTGGGGT TCTTAATATT ATTAATTTG

+     ABRE3a

| Site Name | Organism | Position | Strand | Matrix score. | sequence | function |
| --- | --- | --- | --- | --- | --- | --- |
| ABRE3a | Zea mays | 790 | + | 6 | TACGTG |  |

>003403   
+ TAAAAATAGT GAAAGAACCA CCGGCCGGAT AAACAAGAGT AAAGCTGTCT GAAAATAAAC CCTACTCACA   
  
  
+ CACATGGTAT TTCTCATATT TTATTTTATA GTCAATTATT AAGATAATAA GCATTCTAAC CATTTATGTA   
  
  
+ TACATGTTCA TACCGCGAGC GTTGCTCGGA GGAACACATT TTTGTATCAA GTGATGTTGC AAAATGATGC   
  
  
+ ATACAATCAG TACACATCAC TCTAGCTACT TACTCCATCT TGTAGTATTG CAATGCAGTA AAATATAGCA   
  
  
+ CTAGTCCCAG ATTAATTATT ATGGTTCTCC TTTCAAACAC ATAGTTAATG GTTATAATGA TATACTTAAG   
  
  
+ GGCAAAATAT ATTACTTATA CAAAATAACT CGATGGGTTA CGTAAAATAC ATGAAGAATT CAGTTTAATA   
  
  
+ AGTTTAGTAT ATGCTTGCAT GAGTATGATA CAATATATAT GTGACAAAAT AAAATACAAG TCTAAATTTA   
  
  
+ CGACACCACC ACCCCCTTAT ATAGAGTTAA AACGTGAGAT TCAAGAGTGT AGTTGGTGGA AAAAGATAAA   
  
  
+ TTGTAGGGTT ATTGTCTTGT AGAAATACAT ATACATAAAT ATTTATCTGT ACAAGATGTG CGTATGTACA   
  
  
+ ATAATCATTG ACAATTAACC TAAACATTGC AATTAACATA AACTTTGTTT CTTCTTAAAT TTTAGATGGT   
  
  
+ AAGTTCCTCC TATAAAATCA TTACAAAATC ATAAAATCCC AATAACACAC TTTATCTATA CGCAAAAAAA   
  
  
+ TTTGCCGGGG AAAATATAGT ACGTGGAGGT ATATATAGTA CGTACTTTCT CCTTTGCTTT CAAAGATCAT   
  
  
+ CAAATTTTAA TTAGTTTATA TTATTTTATA TATACACACA CACACACACA CACAAAGTGT GTGAGACATT   
  
  
+ TTTTTTATTT TTGTTTTTGA AATAGAAGGA GAGAGGAAGA GAGTGAATTA GATTTTTTTT TTTTTTTAAT   
  
  
+ TAAAGATATG TTAAGATTAC ATGTAGGTGA GATTTTGAAA AAAAAAACAG CCAAATTTAG TTATGTGAAA   
  
  
+ TTACATTTTT GCCCCGTATT TATTATTCAT ATTTTGTTTT AATTAGATGG TTAAAGTGGT AATTTGATAG   
  
  
+ GATTTTAGTT TACAATGAGT GTTTTATTAA TTAGTAGATA TATATTAAGT ATGTATTTTC AATTAAAAAT   
  
  
+ AAAGAAAAAA AAGCAGCATC TAGCATGGCA AGCACAACAC AAGCAGATAT ATATATATAT ATATATATAT   
  
  
+ ATAATTGGAA AAGTATTGTT GTCGTGGAGA CAAATTTAGG TTCTTGAGCA AAAACTCAAC TACTCTCTAG   
  
  
+ AGAGAGAGAG AGAGAGAGGT AGCAGTAACA CGACTTGTGT AGAAATTGTG AGGGACGAAT CGGTTAAGAA   
  
  
+ GTGCATGTCT TGTATCTCTC TCTAGAGCTA GAAAAATCCT AAGTGTTTCA TTTTTTTTGG AACAAACACA   
  
  
+ GAAAACCCCA AGAATTATAA TAATTAAAC  

- ATTTTTATCA CTTTCTTGGT GGCCGGCCTA TTTGTTCTCA TTTCGACAGA CTTTTATTTG GGATGAGTGT   
  
  
- GTGTACCATA AAGAGTATAA AATAAAATAT CAGTTAATAA TTCTATTATT CGTAAGATTG GTAAATACAT   
  
  
- ATGTACAAGT ATGGCGCTCG CAACGAGCCT CCTTGTGTAA AAACATAGTT CACTACAACG TTTTACTACG   
  
  
- TATGTTAGTC ATGTGTAGTG AGATCGATGA ATGAGGTAGA ACATCATAAC GTTACGTCAT TTTATATCGT   
  
  
- GATCAGGGTC TAATTAATAA TACCAAGAGG AAAGTTTGTG TATCAATTAC CAATATTACT ATATGAATTC   
  
  
- CCGTTTTATA TAATGAATAT GTTTTATTGA GCTACCCAAT GCATTTTATG TACTTCTTAA GTCAAATTAT   
  
  
- TCAAATCATA TACGAACGTA CTCATACTAT GTTATATATA CACTGTTTTA TTTTATGTTC AGATTTAAAT   
  
  
- GCTGTGGTGG TGGGGGAATA TATCTCAATT TTGCACTCTA AGTTCTCACA TCAACCACCT TTTTCTATTT   
  
  
- AACATCCCAA TAACAGAACA TCTTTATGTA TATGTATTTA TAAATAGACA TGTTCTACAC GCATACATGT   
  
  
- TATTAGTAAC TGTTAATTGG ATTTGTAACG TTAATTGTAT TTGAAACAAA GAAGAATTTA AAATCTACCA   
  
  
- TTCAAGGAGG ATATTTTAGT AATGTTTTAG TATTTTAGGG TTATTGTGTG AAATAGATAT GCGTTTTTTT   
  
  
- AAACGGCCCC TTTTATATCA TGCACCTCCA TATATATCAT GCATGAAAGA GGAAACGAAA GTTTCTAGTA   
  
  
- GTTTAAAATT AATCAAATAT AATAAAATAT ATATGTGTGT GTGTGTGTGT GTGTTTCACA CACTCTGTAA   
  
  
- AAAAAATAAA AACAAAAACT TTATCTTCCT CTCTCCTTCT CTCACTTAAT CTAAAAAAAA AAAAAAATTA   
  
  
- ATTTCTATAC AATTCTAATG TACATCCACT CTAAAACTTT TTTTTTTGTC GGTTTAAATC AATACACTTT   
  
  
- AATGTAAAAA CGGGGCATAA ATAATAAGTA TAAAACAAAA TTAATCTACC AATTTCACCA TTAAACTATC   
  
  
- CTAAAATCAA ATGTTACTCA CAAAATAATT AATCATCTAT ATATAATTCA TACATAAAAG TTAATTTTTA   
  
  
- TTTCTTTTTT TTCGTCGTAG ATCGTACCGT TCGTGTTGTG TTCGTCTATA TATATATATA TATATATATA   
  
  
- TATTAACCTT TTCATAACAA CAGCACCTCT GTTTAAATCC AAGAACTCGT TTTTGAGTTG ATGAGAGATC   
  
  
- TCTCTCTCTC TCTCTCTCCA TCGTCATTGT GCTGAACACA TCTTTAACAC TCCCTGCTTA GCCAATTCTT   
  
  
- CACGTACAGA ACATAGAGAG AGATCTCGAT CTTTTTAGGA TTCACAAAGT AAAAAAAACC TTGTTTGTGT   
  
  
- CTTTTGGGGT TCTTAATATT ATTAATTTG

+     ABRE4

| Site Name | Organism | Position | Strand | Matrix score. | sequence | function |
| --- | --- | --- | --- | --- | --- | --- |
| ABRE4 | Zea mays | 790 | - | 6 | CACGTA |  |

>003403   
+ TAAAAATAGT GAAAGAACCA CCGGCCGGAT AAACAAGAGT AAAGCTGTCT GAAAATAAAC CCTACTCACA   
  
  
+ CACATGGTAT TTCTCATATT TTATTTTATA GTCAATTATT AAGATAATAA GCATTCTAAC CATTTATGTA   
  
  
+ TACATGTTCA TACCGCGAGC GTTGCTCGGA GGAACACATT TTTGTATCAA GTGATGTTGC AAAATGATGC   
  
  
+ ATACAATCAG TACACATCAC TCTAGCTACT TACTCCATCT TGTAGTATTG CAATGCAGTA AAATATAGCA   
  
  
+ CTAGTCCCAG ATTAATTATT ATGGTTCTCC TTTCAAACAC ATAGTTAATG GTTATAATGA TATACTTAAG   
  
  
+ GGCAAAATAT ATTACTTATA CAAAATAACT CGATGGGTTA CGTAAAATAC ATGAAGAATT CAGTTTAATA   
  
  
+ AGTTTAGTAT ATGCTTGCAT GAGTATGATA CAATATATAT GTGACAAAAT AAAATACAAG TCTAAATTTA   
  
  
+ CGACACCACC ACCCCCTTAT ATAGAGTTAA AACGTGAGAT TCAAGAGTGT AGTTGGTGGA AAAAGATAAA   
  
  
+ TTGTAGGGTT ATTGTCTTGT AGAAATACAT ATACATAAAT ATTTATCTGT ACAAGATGTG CGTATGTACA   
  
  
+ ATAATCATTG ACAATTAACC TAAACATTGC AATTAACATA AACTTTGTTT CTTCTTAAAT TTTAGATGGT   
  
  
+ AAGTTCCTCC TATAAAATCA TTACAAAATC ATAAAATCCC AATAACACAC TTTATCTATA CGCAAAAAAA   
  
  
+ TTTGCCGGGG AAAATATAGT ACGTGGAGGT ATATATAGTA CGTACTTTCT CCTTTGCTTT CAAAGATCAT   
  
  
+ CAAATTTTAA TTAGTTTATA TTATTTTATA TATACACACA CACACACACA CACAAAGTGT GTGAGACATT   
  
  
+ TTTTTTATTT TTGTTTTTGA AATAGAAGGA GAGAGGAAGA GAGTGAATTA GATTTTTTTT TTTTTTTAAT   
  
  
+ TAAAGATATG TTAAGATTAC ATGTAGGTGA GATTTTGAAA AAAAAAACAG CCAAATTTAG TTATGTGAAA   
  
  
+ TTACATTTTT GCCCCGTATT TATTATTCAT ATTTTGTTTT AATTAGATGG TTAAAGTGGT AATTTGATAG   
  
  
+ GATTTTAGTT TACAATGAGT GTTTTATTAA TTAGTAGATA TATATTAAGT ATGTATTTTC AATTAAAAAT   
  
  
+ AAAGAAAAAA AAGCAGCATC TAGCATGGCA AGCACAACAC AAGCAGATAT ATATATATAT ATATATATAT   
  
  
+ ATAATTGGAA AAGTATTGTT GTCGTGGAGA CAAATTTAGG TTCTTGAGCA AAAACTCAAC TACTCTCTAG   
  
  
+ AGAGAGAGAG AGAGAGAGGT AGCAGTAACA CGACTTGTGT AGAAATTGTG AGGGACGAAT CGGTTAAGAA   
  
  
+ GTGCATGTCT TGTATCTCTC TCTAGAGCTA GAAAAATCCT AAGTGTTTCA TTTTTTTTGG AACAAACACA   
  
  
+ GAAAACCCCA AGAATTATAA TAATTAAAC  

- ATTTTTATCA CTTTCTTGGT GGCCGGCCTA TTTGTTCTCA TTTCGACAGA CTTTTATTTG GGATGAGTGT   
  
  
- GTGTACCATA AAGAGTATAA AATAAAATAT CAGTTAATAA TTCTATTATT CGTAAGATTG GTAAATACAT   
  
  
- ATGTACAAGT ATGGCGCTCG CAACGAGCCT CCTTGTGTAA AAACATAGTT CACTACAACG TTTTACTACG   
  
  
- TATGTTAGTC ATGTGTAGTG AGATCGATGA ATGAGGTAGA ACATCATAAC GTTACGTCAT TTTATATCGT   
  
  
- GATCAGGGTC TAATTAATAA TACCAAGAGG AAAGTTTGTG TATCAATTAC CAATATTACT ATATGAATTC   
  
  
- CCGTTTTATA TAATGAATAT GTTTTATTGA GCTACCCAAT GCATTTTATG TACTTCTTAA GTCAAATTAT   
  
  
- TCAAATCATA TACGAACGTA CTCATACTAT GTTATATATA CACTGTTTTA TTTTATGTTC AGATTTAAAT   
  
  
- GCTGTGGTGG TGGGGGAATA TATCTCAATT TTGCACTCTA AGTTCTCACA TCAACCACCT TTTTCTATTT   
  
  
- AACATCCCAA TAACAGAACA TCTTTATGTA TATGTATTTA TAAATAGACA TGTTCTACAC GCATACATGT   
  
  
- TATTAGTAAC TGTTAATTGG ATTTGTAACG TTAATTGTAT TTGAAACAAA GAAGAATTTA AAATCTACCA   
  
  
- TTCAAGGAGG ATATTTTAGT AATGTTTTAG TATTTTAGGG TTATTGTGTG AAATAGATAT GCGTTTTTTT   
  
  
- AAACGGCCCC TTTTATATCA TGCACCTCCA TATATATCAT GCATGAAAGA GGAAACGAAA GTTTCTAGTA   
  
  
- GTTTAAAATT AATCAAATAT AATAAAATAT ATATGTGTGT GTGTGTGTGT GTGTTTCACA CACTCTGTAA   
  
  
- AAAAAATAAA AACAAAAACT TTATCTTCCT CTCTCCTTCT CTCACTTAAT CTAAAAAAAA AAAAAAATTA   
  
  
- ATTTCTATAC AATTCTAATG TACATCCACT CTAAAACTTT TTTTTTTGTC GGTTTAAATC AATACACTTT   
  
  
- AATGTAAAAA CGGGGCATAA ATAATAAGTA TAAAACAAAA TTAATCTACC AATTTCACCA TTAAACTATC   
  
  
- CTAAAATCAA ATGTTACTCA CAAAATAATT AATCATCTAT ATATAATTCA TACATAAAAG TTAATTTTTA   
  
  
- TTTCTTTTTT TTCGTCGTAG ATCGTACCGT TCGTGTTGTG TTCGTCTATA TATATATATA TATATATATA   
  
  
- TATTAACCTT TTCATAACAA CAGCACCTCT GTTTAAATCC AAGAACTCGT TTTTGAGTTG ATGAGAGATC   
  
  
- TCTCTCTCTC TCTCTCTCCA TCGTCATTGT GCTGAACACA TCTTTAACAC TCCCTGCTTA GCCAATTCTT   
  
  
- CACGTACAGA ACATAGAGAG AGATCTCGAT CTTTTTAGGA TTCACAAAGT AAAAAAAACC TTGTTTGTGT   
  
  
- CTTTTGGGGT TCTTAATATT ATTAATTTG

+     AE-box

| Site Name | Organism | Position | Strand | Matrix score. | sequence | function |
| --- | --- | --- | --- | --- | --- | --- |
| AE-box | Arabidopsis thaliana | 675 | - | 8 | AGAAACAA | part of a module for light response |

>003403   
+ TAAAAATAGT GAAAGAACCA CCGGCCGGAT AAACAAGAGT AAAGCTGTCT GAAAATAAAC CCTACTCACA   
  
  
+ CACATGGTAT TTCTCATATT TTATTTTATA GTCAATTATT AAGATAATAA GCATTCTAAC CATTTATGTA   
  
  
+ TACATGTTCA TACCGCGAGC GTTGCTCGGA GGAACACATT TTTGTATCAA GTGATGTTGC AAAATGATGC   
  
  
+ ATACAATCAG TACACATCAC TCTAGCTACT TACTCCATCT TGTAGTATTG CAATGCAGTA AAATATAGCA   
  
  
+ CTAGTCCCAG ATTAATTATT ATGGTTCTCC TTTCAAACAC ATAGTTAATG GTTATAATGA TATACTTAAG   
  
  
+ GGCAAAATAT ATTACTTATA CAAAATAACT CGATGGGTTA CGTAAAATAC ATGAAGAATT CAGTTTAATA   
  
  
+ AGTTTAGTAT ATGCTTGCAT GAGTATGATA CAATATATAT GTGACAAAAT AAAATACAAG TCTAAATTTA   
  
  
+ CGACACCACC ACCCCCTTAT ATAGAGTTAA AACGTGAGAT TCAAGAGTGT AGTTGGTGGA AAAAGATAAA   
  
  
+ TTGTAGGGTT ATTGTCTTGT AGAAATACAT ATACATAAAT ATTTATCTGT ACAAGATGTG CGTATGTACA   
  
  
+ ATAATCATTG ACAATTAACC TAAACATTGC AATTAACATA AACTTTGTTT CTTCTTAAAT TTTAGATGGT   
  
  
+ AAGTTCCTCC TATAAAATCA TTACAAAATC ATAAAATCCC AATAACACAC TTTATCTATA CGCAAAAAAA   
  
  
+ TTTGCCGGGG AAAATATAGT ACGTGGAGGT ATATATAGTA CGTACTTTCT CCTTTGCTTT CAAAGATCAT   
  
  
+ CAAATTTTAA TTAGTTTATA TTATTTTATA TATACACACA CACACACACA CACAAAGTGT GTGAGACATT   
  
  
+ TTTTTTATTT TTGTTTTTGA AATAGAAGGA GAGAGGAAGA GAGTGAATTA GATTTTTTTT TTTTTTTAAT   
  
  
+ TAAAGATATG TTAAGATTAC ATGTAGGTGA GATTTTGAAA AAAAAAACAG CCAAATTTAG TTATGTGAAA   
  
  
+ TTACATTTTT GCCCCGTATT TATTATTCAT ATTTTGTTTT AATTAGATGG TTAAAGTGGT AATTTGATAG   
  
  
+ GATTTTAGTT TACAATGAGT GTTTTATTAA TTAGTAGATA TATATTAAGT ATGTATTTTC AATTAAAAAT   
  
  
+ AAAGAAAAAA AAGCAGCATC TAGCATGGCA AGCACAACAC AAGCAGATAT ATATATATAT ATATATATAT   
  
  
+ ATAATTGGAA AAGTATTGTT GTCGTGGAGA CAAATTTAGG TTCTTGAGCA AAAACTCAAC TACTCTCTAG   
  
  
+ AGAGAGAGAG AGAGAGAGGT AGCAGTAACA CGACTTGTGT AGAAATTGTG AGGGACGAAT CGGTTAAGAA   
  
  
+ GTGCATGTCT TGTATCTCTC TCTAGAGCTA GAAAAATCCT AAGTGTTTCA TTTTTTTTGG AACAAACACA   
  
  
+ GAAAACCCCA AGAATTATAA TAATTAAAC  

- ATTTTTATCA CTTTCTTGGT GGCCGGCCTA TTTGTTCTCA TTTCGACAGA CTTTTATTTG GGATGAGTGT   
  
  
- GTGTACCATA AAGAGTATAA AATAAAATAT CAGTTAATAA TTCTATTATT CGTAAGATTG GTAAATACAT   
  
  
- ATGTACAAGT ATGGCGCTCG CAACGAGCCT CCTTGTGTAA AAACATAGTT CACTACAACG TTTTACTACG   
  
  
- TATGTTAGTC ATGTGTAGTG AGATCGATGA ATGAGGTAGA ACATCATAAC GTTACGTCAT TTTATATCGT   
  
  
- GATCAGGGTC TAATTAATAA TACCAAGAGG AAAGTTTGTG TATCAATTAC CAATATTACT ATATGAATTC   
  
  
- CCGTTTTATA TAATGAATAT GTTTTATTGA GCTACCCAAT GCATTTTATG TACTTCTTAA GTCAAATTAT   
  
  
- TCAAATCATA TACGAACGTA CTCATACTAT GTTATATATA CACTGTTTTA TTTTATGTTC AGATTTAAAT   
  
  
- GCTGTGGTGG TGGGGGAATA TATCTCAATT TTGCACTCTA AGTTCTCACA TCAACCACCT TTTTCTATTT   
  
  
- AACATCCCAA TAACAGAACA TCTTTATGTA TATGTATTTA TAAATAGACA TGTTCTACAC GCATACATGT   
  
  
- TATTAGTAAC TGTTAATTGG ATTTGTAACG TTAATTGTAT TTGAAACAAA GAAGAATTTA AAATCTACCA   
  
  
- TTCAAGGAGG ATATTTTAGT AATGTTTTAG TATTTTAGGG TTATTGTGTG AAATAGATAT GCGTTTTTTT   
  
  
- AAACGGCCCC TTTTATATCA TGCACCTCCA TATATATCAT GCATGAAAGA GGAAACGAAA GTTTCTAGTA   
  
  
- GTTTAAAATT AATCAAATAT AATAAAATAT ATATGTGTGT GTGTGTGTGT GTGTTTCACA CACTCTGTAA   
  
  
- AAAAAATAAA AACAAAAACT TTATCTTCCT CTCTCCTTCT CTCACTTAAT CTAAAAAAAA AAAAAAATTA   
  
  
- ATTTCTATAC AATTCTAATG TACATCCACT CTAAAACTTT TTTTTTTGTC GGTTTAAATC AATACACTTT   
  
  
- AATGTAAAAA CGGGGCATAA ATAATAAGTA TAAAACAAAA TTAATCTACC AATTTCACCA TTAAACTATC   
  
  
- CTAAAATCAA ATGTTACTCA CAAAATAATT AATCATCTAT ATATAATTCA TACATAAAAG TTAATTTTTA   
  
  
- TTTCTTTTTT TTCGTCGTAG ATCGTACCGT TCGTGTTGTG TTCGTCTATA TATATATATA TATATATATA   
  
  
- TATTAACCTT TTCATAACAA CAGCACCTCT GTTTAAATCC AAGAACTCGT TTTTGAGTTG ATGAGAGATC   
  
  
- TCTCTCTCTC TCTCTCTCCA TCGTCATTGT GCTGAACACA TCTTTAACAC TCCCTGCTTA GCCAATTCTT   
  
  
- CACGTACAGA ACATAGAGAG AGATCTCGAT CTTTTTAGGA TTCACAAAGT AAAAAAAACC TTGTTTGTGT   
  
  
- CTTTTGGGGT TCTTAATATT ATTAATTTG

+     ATCT-motif

| Site Name | Organism | Position | Strand | Matrix score. | sequence | function |
| --- | --- | --- | --- | --- | --- | --- |
| ATCT-motif | Pisum sativum | 955 | - | 9 | AATCTAATCC | part of a conserved DNA module involved in light responsiveness |

>003403   
+ TAAAAATAGT GAAAGAACCA CCGGCCGGAT AAACAAGAGT AAAGCTGTCT GAAAATAAAC CCTACTCACA   
  
  
+ CACATGGTAT TTCTCATATT TTATTTTATA GTCAATTATT AAGATAATAA GCATTCTAAC CATTTATGTA   
  
  
+ TACATGTTCA TACCGCGAGC GTTGCTCGGA GGAACACATT TTTGTATCAA GTGATGTTGC AAAATGATGC   
  
  
+ ATACAATCAG TACACATCAC TCTAGCTACT TACTCCATCT TGTAGTATTG CAATGCAGTA AAATATAGCA   
  
  
+ CTAGTCCCAG ATTAATTATT ATGGTTCTCC TTTCAAACAC ATAGTTAATG GTTATAATGA TATACTTAAG   
  
  
+ GGCAAAATAT ATTACTTATA CAAAATAACT CGATGGGTTA CGTAAAATAC ATGAAGAATT CAGTTTAATA   
  
  
+ AGTTTAGTAT ATGCTTGCAT GAGTATGATA CAATATATAT GTGACAAAAT AAAATACAAG TCTAAATTTA   
  
  
+ CGACACCACC ACCCCCTTAT ATAGAGTTAA AACGTGAGAT TCAAGAGTGT AGTTGGTGGA AAAAGATAAA   
  
  
+ TTGTAGGGTT ATTGTCTTGT AGAAATACAT ATACATAAAT ATTTATCTGT ACAAGATGTG CGTATGTACA   
  
  
+ ATAATCATTG ACAATTAACC TAAACATTGC AATTAACATA AACTTTGTTT CTTCTTAAAT TTTAGATGGT   
  
  
+ AAGTTCCTCC TATAAAATCA TTACAAAATC ATAAAATCCC AATAACACAC TTTATCTATA CGCAAAAAAA   
  
  
+ TTTGCCGGGG AAAATATAGT ACGTGGAGGT ATATATAGTA CGTACTTTCT CCTTTGCTTT CAAAGATCAT   
  
  
+ CAAATTTTAA TTAGTTTATA TTATTTTATA TATACACACA CACACACACA CACAAAGTGT GTGAGACATT   
  
  
+ TTTTTTATTT TTGTTTTTGA AATAGAAGGA GAGAGGAAGA GAGTGAATTA GATTTTTTTT TTTTTTTAAT   
  
  
+ TAAAGATATG TTAAGATTAC ATGTAGGTGA GATTTTGAAA AAAAAAACAG CCAAATTTAG TTATGTGAAA   
  
  
+ TTACATTTTT GCCCCGTATT TATTATTCAT ATTTTGTTTT AATTAGATGG TTAAAGTGGT AATTTGATAG   
  
  
+ GATTTTAGTT TACAATGAGT GTTTTATTAA TTAGTAGATA TATATTAAGT ATGTATTTTC AATTAAAAAT   
  
  
+ AAAGAAAAAA AAGCAGCATC TAGCATGGCA AGCACAACAC AAGCAGATAT ATATATATAT ATATATATAT   
  
  
+ ATAATTGGAA AAGTATTGTT GTCGTGGAGA CAAATTTAGG TTCTTGAGCA AAAACTCAAC TACTCTCTAG   
  
  
+ AGAGAGAGAG AGAGAGAGGT AGCAGTAACA CGACTTGTGT AGAAATTGTG AGGGACGAAT CGGTTAAGAA   
  
  
+ GTGCATGTCT TGTATCTCTC TCTAGAGCTA GAAAAATCCT AAGTGTTTCA TTTTTTTTGG AACAAACACA   
  
  
+ GAAAACCCCA AGAATTATAA TAATTAAAC  

- ATTTTTATCA CTTTCTTGGT GGCCGGCCTA TTTGTTCTCA TTTCGACAGA CTTTTATTTG GGATGAGTGT   
  
  
- GTGTACCATA AAGAGTATAA AATAAAATAT CAGTTAATAA TTCTATTATT CGTAAGATTG GTAAATACAT   
  
  
- ATGTACAAGT ATGGCGCTCG CAACGAGCCT CCTTGTGTAA AAACATAGTT CACTACAACG TTTTACTACG   
  
  
- TATGTTAGTC ATGTGTAGTG AGATCGATGA ATGAGGTAGA ACATCATAAC GTTACGTCAT TTTATATCGT   
  
  
- GATCAGGGTC TAATTAATAA TACCAAGAGG AAAGTTTGTG TATCAATTAC CAATATTACT ATATGAATTC   
  
  
- CCGTTTTATA TAATGAATAT GTTTTATTGA GCTACCCAAT GCATTTTATG TACTTCTTAA GTCAAATTAT   
  
  
- TCAAATCATA TACGAACGTA CTCATACTAT GTTATATATA CACTGTTTTA TTTTATGTTC AGATTTAAAT   
  
  
- GCTGTGGTGG TGGGGGAATA TATCTCAATT TTGCACTCTA AGTTCTCACA TCAACCACCT TTTTCTATTT   
  
  
- AACATCCCAA TAACAGAACA TCTTTATGTA TATGTATTTA TAAATAGACA TGTTCTACAC GCATACATGT   
  
  
- TATTAGTAAC TGTTAATTGG ATTTGTAACG TTAATTGTAT TTGAAACAAA GAAGAATTTA AAATCTACCA   
  
  
- TTCAAGGAGG ATATTTTAGT AATGTTTTAG TATTTTAGGG TTATTGTGTG AAATAGATAT GCGTTTTTTT   
  
  
- AAACGGCCCC TTTTATATCA TGCACCTCCA TATATATCAT GCATGAAAGA GGAAACGAAA GTTTCTAGTA   
  
  
- GTTTAAAATT AATCAAATAT AATAAAATAT ATATGTGTGT GTGTGTGTGT GTGTTTCACA CACTCTGTAA   
  
  
- AAAAAATAAA AACAAAAACT TTATCTTCCT CTCTCCTTCT CTCACTTAAT CTAAAAAAAA AAAAAAATTA   
  
  
- ATTTCTATAC AATTCTAATG TACATCCACT CTAAAACTTT TTTTTTTGTC GGTTTAAATC AATACACTTT   
  
  
- AATGTAAAAA CGGGGCATAA ATAATAAGTA TAAAACAAAA TTAATCTACC AATTTCACCA TTAAACTATC   
  
  
- CTAAAATCAA ATGTTACTCA CAAAATAATT AATCATCTAT ATATAATTCA TACATAAAAG TTAATTTTTA   
  
  
- TTTCTTTTTT TTCGTCGTAG ATCGTACCGT TCGTGTTGTG TTCGTCTATA TATATATATA TATATATATA   
  
  
- TATTAACCTT TTCATAACAA CAGCACCTCT GTTTAAATCC AAGAACTCGT TTTTGAGTTG ATGAGAGATC   
  
  
- TCTCTCTCTC TCTCTCTCCA TCGTCATTGT GCTGAACACA TCTTTAACAC TCCCTGCTTA GCCAATTCTT   
  
  
- CACGTACAGA ACATAGAGAG AGATCTCGAT CTTTTTAGGA TTCACAAAGT AAAAAAAACC TTGTTTGTGT   
  
  
- CTTTTGGGGT TCTTAATATT ATTAATTTG

+     AT~TATA-box

| Site Name | Organism | Position | Strand | Matrix score. | sequence | function |
| --- | --- | --- | --- | --- | --- | --- |
| AT~TATA-box | Arabidopsis thaliana | 508 | + | 6 | TATATA |  |
| AT~TATA-box | Arabidopsis thaliana | 1242 | - | 6 | TATATA |  |
| AT~TATA-box | Arabidopsis thaliana | 1159 | - | 6 | TATATA |  |
| AT~TATA-box | Arabidopsis thaliana | 1254 | - | 6 | TATATA |  |
| AT~TATA-box | Arabidopsis thaliana | 865 | - | 8 | TATATAAA |  |
| AT~TATA-box | Arabidopsis thaliana | 1250 | - | 6 | TATATA |  |
| AT~TATA-box | Arabidopsis thaliana | 1238 | - | 6 | TATATA |  |
| AT~TATA-box | Arabidopsis thaliana | 1258 | - | 6 | TATATA |  |
| AT~TATA-box | Arabidopsis thaliana | 800 | - | 6 | TATATA |  |
| AT~TATA-box | Arabidopsis thaliana | 1246 | - | 6 | TATATA |  |
| AT~TATA-box | Arabidopsis thaliana | 869 | - | 6 | TATATA |  |
| AT~TATA-box | Arabidopsis thaliana | 1256 | - | 6 | TATATA |  |
| AT~TATA-box | Arabidopsis thaliana | 867 | - | 6 | TATATA |  |
| AT~TATA-box | Arabidopsis thaliana | 1252 | - | 6 | TATATA |  |
| AT~TATA-box | Arabidopsis thaliana | 1240 | - | 6 | TATATA |  |
| AT~TATA-box | Arabidopsis thaliana | 1244 | - | 6 | TATATA |  |
| AT~TATA-box | Arabidopsis thaliana | 454 | + | 6 | TATATA |  |
| AT~TATA-box | Arabidopsis thaliana | 1248 | - | 6 | TATATA |  |
| AT~TATA-box | Arabidopsis thaliana | 802 | - | 6 | TATATA |  |

>003403   
+ TAAAAATAGT GAAAGAACCA CCGGCCGGAT AAACAAGAGT AAAGCTGTCT GAAAATAAAC CCTACTCACA   
  
  
+ CACATGGTAT TTCTCATATT TTATTTTATA GTCAATTATT AAGATAATAA GCATTCTAAC CATTTATGTA   
  
  
+ TACATGTTCA TACCGCGAGC GTTGCTCGGA GGAACACATT TTTGTATCAA GTGATGTTGC AAAATGATGC   
  
  
+ ATACAATCAG TACACATCAC TCTAGCTACT TACTCCATCT TGTAGTATTG CAATGCAGTA AAATATAGCA   
  
  
+ CTAGTCCCAG ATTAATTATT ATGGTTCTCC TTTCAAACAC ATAGTTAATG GTTATAATGA TATACTTAAG   
  
  
+ GGCAAAATAT ATTACTTATA CAAAATAACT CGATGGGTTA CGTAAAATAC ATGAAGAATT CAGTTTAATA   
  
  
+ AGTTTAGTAT ATGCTTGCAT GAGTATGATA CAATATATAT GTGACAAAAT AAAATACAAG TCTAAATTTA   
  
  
+ CGACACCACC ACCCCCTTAT ATAGAGTTAA AACGTGAGAT TCAAGAGTGT AGTTGGTGGA AAAAGATAAA   
  
  
+ TTGTAGGGTT ATTGTCTTGT AGAAATACAT ATACATAAAT ATTTATCTGT ACAAGATGTG CGTATGTACA   
  
  
+ ATAATCATTG ACAATTAACC TAAACATTGC AATTAACATA AACTTTGTTT CTTCTTAAAT TTTAGATGGT   
  
  
+ AAGTTCCTCC TATAAAATCA TTACAAAATC ATAAAATCCC AATAACACAC TTTATCTATA CGCAAAAAAA   
  
  
+ TTTGCCGGGG AAAATATAGT ACGTGGAGGT ATATATAGTA CGTACTTTCT CCTTTGCTTT CAAAGATCAT   
  
  
+ CAAATTTTAA TTAGTTTATA TTATTTTATA TATACACACA CACACACACA CACAAAGTGT GTGAGACATT   
  
  
+ TTTTTTATTT TTGTTTTTGA AATAGAAGGA GAGAGGAAGA GAGTGAATTA GATTTTTTTT TTTTTTTAAT   
  
  
+ TAAAGATATG TTAAGATTAC ATGTAGGTGA GATTTTGAAA AAAAAAACAG CCAAATTTAG TTATGTGAAA   
  
  
+ TTACATTTTT GCCCCGTATT TATTATTCAT ATTTTGTTTT AATTAGATGG TTAAAGTGGT AATTTGATAG   
  
  
+ GATTTTAGTT TACAATGAGT GTTTTATTAA TTAGTAGATA TATATTAAGT ATGTATTTTC AATTAAAAAT   
  
  
+ AAAGAAAAAA AAGCAGCATC TAGCATGGCA AGCACAACAC AAGCAGATAT ATATATATAT ATATATATAT   
  
  
+ ATAATTGGAA AAGTATTGTT GTCGTGGAGA CAAATTTAGG TTCTTGAGCA AAAACTCAAC TACTCTCTAG   
  
  
+ AGAGAGAGAG AGAGAGAGGT AGCAGTAACA CGACTTGTGT AGAAATTGTG AGGGACGAAT CGGTTAAGAA   
  
  
+ GTGCATGTCT TGTATCTCTC TCTAGAGCTA GAAAAATCCT AAGTGTTTCA TTTTTTTTGG AACAAACACA   
  
  
+ GAAAACCCCA AGAATTATAA TAATTAAAC  

- ATTTTTATCA CTTTCTTGGT GGCCGGCCTA TTTGTTCTCA TTTCGACAGA CTTTTATTTG GGATGAGTGT   
  
  
- GTGTACCATA AAGAGTATAA AATAAAATAT CAGTTAATAA TTCTATTATT CGTAAGATTG GTAAATACAT   
  
  
- ATGTACAAGT ATGGCGCTCG CAACGAGCCT CCTTGTGTAA AAACATAGTT CACTACAACG TTTTACTACG   
  
  
- TATGTTAGTC ATGTGTAGTG AGATCGATGA ATGAGGTAGA ACATCATAAC GTTACGTCAT TTTATATCGT   
  
  
- GATCAGGGTC TAATTAATAA TACCAAGAGG AAAGTTTGTG TATCAATTAC CAATATTACT ATATGAATTC   
  
  
- CCGTTTTATA TAATGAATAT GTTTTATTGA GCTACCCAAT GCATTTTATG TACTTCTTAA GTCAAATTAT   
  
  
- TCAAATCATA TACGAACGTA CTCATACTAT GTTATATATA CACTGTTTTA TTTTATGTTC AGATTTAAAT   
  
  
- GCTGTGGTGG TGGGGGAATA TATCTCAATT TTGCACTCTA AGTTCTCACA TCAACCACCT TTTTCTATTT   
  
  
- AACATCCCAA TAACAGAACA TCTTTATGTA TATGTATTTA TAAATAGACA TGTTCTACAC GCATACATGT   
  
  
- TATTAGTAAC TGTTAATTGG ATTTGTAACG TTAATTGTAT TTGAAACAAA GAAGAATTTA AAATCTACCA   
  
  
- TTCAAGGAGG ATATTTTAGT AATGTTTTAG TATTTTAGGG TTATTGTGTG AAATAGATAT GCGTTTTTTT   
  
  
- AAACGGCCCC TTTTATATCA TGCACCTCCA TATATATCAT GCATGAAAGA GGAAACGAAA GTTTCTAGTA   
  
  
- GTTTAAAATT AATCAAATAT AATAAAATAT ATATGTGTGT GTGTGTGTGT GTGTTTCACA CACTCTGTAA   
  
  
- AAAAAATAAA AACAAAAACT TTATCTTCCT CTCTCCTTCT CTCACTTAAT CTAAAAAAAA AAAAAAATTA   
  
  
- ATTTCTATAC AATTCTAATG TACATCCACT CTAAAACTTT TTTTTTTGTC GGTTTAAATC AATACACTTT   
  
  
- AATGTAAAAA CGGGGCATAA ATAATAAGTA TAAAACAAAA TTAATCTACC AATTTCACCA TTAAACTATC   
  
  
- CTAAAATCAA ATGTTACTCA CAAAATAATT AATCATCTAT ATATAATTCA TACATAAAAG TTAATTTTTA   
  
  
- TTTCTTTTTT TTCGTCGTAG ATCGTACCGT TCGTGTTGTG TTCGTCTATA TATATATATA TATATATATA   
  
  
- TATTAACCTT TTCATAACAA CAGCACCTCT GTTTAAATCC AAGAACTCGT TTTTGAGTTG ATGAGAGATC   
  
  
- TCTCTCTCTC TCTCTCTCCA TCGTCATTGT GCTGAACACA TCTTTAACAC TCCCTGCTTA GCCAATTCTT   
  
  
- CACGTACAGA ACATAGAGAG AGATCTCGAT CTTTTTAGGA TTCACAAAGT AAAAAAAACC TTGTTTGTGT   
  
  
- CTTTTGGGGT TCTTAATATT ATTAATTTG

+     Box 4

| Site Name | Organism | Position | Strand | Matrix score. | sequence | function |
| --- | --- | --- | --- | --- | --- | --- |
| Box 4 | Petroselinum crispum | 291 | + | 6 | ATTAAT | part of a conserved DNA module involved in light responsiveness |
| Box 4 | Petroselinum crispum | 1146 | - | 6 | ATTAAT | part of a conserved DNA module involved in light responsiveness |

>003403   
+ TAAAAATAGT GAAAGAACCA CCGGCCGGAT AAACAAGAGT AAAGCTGTCT GAAAATAAAC CCTACTCACA   
  
  
+ CACATGGTAT TTCTCATATT TTATTTTATA GTCAATTATT AAGATAATAA GCATTCTAAC CATTTATGTA   
  
  
+ TACATGTTCA TACCGCGAGC GTTGCTCGGA GGAACACATT TTTGTATCAA GTGATGTTGC AAAATGATGC   
  
  
+ ATACAATCAG TACACATCAC TCTAGCTACT TACTCCATCT TGTAGTATTG CAATGCAGTA AAATATAGCA   
  
  
+ CTAGTCCCAG ATTAATTATT ATGGTTCTCC TTTCAAACAC ATAGTTAATG GTTATAATGA TATACTTAAG   
  
  
+ GGCAAAATAT ATTACTTATA CAAAATAACT CGATGGGTTA CGTAAAATAC ATGAAGAATT CAGTTTAATA   
  
  
+ AGTTTAGTAT ATGCTTGCAT GAGTATGATA CAATATATAT GTGACAAAAT AAAATACAAG TCTAAATTTA   
  
  
+ CGACACCACC ACCCCCTTAT ATAGAGTTAA AACGTGAGAT TCAAGAGTGT AGTTGGTGGA AAAAGATAAA   
  
  
+ TTGTAGGGTT ATTGTCTTGT AGAAATACAT ATACATAAAT ATTTATCTGT ACAAGATGTG CGTATGTACA   
  
  
+ ATAATCATTG ACAATTAACC TAAACATTGC AATTAACATA AACTTTGTTT CTTCTTAAAT TTTAGATGGT   
  
  
+ AAGTTCCTCC TATAAAATCA TTACAAAATC ATAAAATCCC AATAACACAC TTTATCTATA CGCAAAAAAA   
  
  
+ TTTGCCGGGG AAAATATAGT ACGTGGAGGT ATATATAGTA CGTACTTTCT CCTTTGCTTT CAAAGATCAT   
  
  
+ CAAATTTTAA TTAGTTTATA TTATTTTATA TATACACACA CACACACACA CACAAAGTGT GTGAGACATT   
  
  
+ TTTTTTATTT TTGTTTTTGA AATAGAAGGA GAGAGGAAGA GAGTGAATTA GATTTTTTTT TTTTTTTAAT   
  
  
+ TAAAGATATG TTAAGATTAC ATGTAGGTGA GATTTTGAAA AAAAAAACAG CCAAATTTAG TTATGTGAAA   
  
  
+ TTACATTTTT GCCCCGTATT TATTATTCAT ATTTTGTTTT AATTAGATGG TTAAAGTGGT AATTTGATAG   
  
  
+ GATTTTAGTT TACAATGAGT GTTTTATTAA TTAGTAGATA TATATTAAGT ATGTATTTTC AATTAAAAAT   
  
  
+ AAAGAAAAAA AAGCAGCATC TAGCATGGCA AGCACAACAC AAGCAGATAT ATATATATAT ATATATATAT   
  
  
+ ATAATTGGAA AAGTATTGTT GTCGTGGAGA CAAATTTAGG TTCTTGAGCA AAAACTCAAC TACTCTCTAG   
  
  
+ AGAGAGAGAG AGAGAGAGGT AGCAGTAACA CGACTTGTGT AGAAATTGTG AGGGACGAAT CGGTTAAGAA   
  
  
+ GTGCATGTCT TGTATCTCTC TCTAGAGCTA GAAAAATCCT AAGTGTTTCA TTTTTTTTGG AACAAACACA   
  
  
+ GAAAACCCCA AGAATTATAA TAATTAAAC  

- ATTTTTATCA CTTTCTTGGT GGCCGGCCTA TTTGTTCTCA TTTCGACAGA CTTTTATTTG GGATGAGTGT   
  
  
- GTGTACCATA AAGAGTATAA AATAAAATAT CAGTTAATAA TTCTATTATT CGTAAGATTG GTAAATACAT   
  
  
- ATGTACAAGT ATGGCGCTCG CAACGAGCCT CCTTGTGTAA AAACATAGTT CACTACAACG TTTTACTACG   
  
  
- TATGTTAGTC ATGTGTAGTG AGATCGATGA ATGAGGTAGA ACATCATAAC GTTACGTCAT TTTATATCGT   
  
  
- GATCAGGGTC TAATTAATAA TACCAAGAGG AAAGTTTGTG TATCAATTAC CAATATTACT ATATGAATTC   
  
  
- CCGTTTTATA TAATGAATAT GTTTTATTGA GCTACCCAAT GCATTTTATG TACTTCTTAA GTCAAATTAT   
  
  
- TCAAATCATA TACGAACGTA CTCATACTAT GTTATATATA CACTGTTTTA TTTTATGTTC AGATTTAAAT   
  
  
- GCTGTGGTGG TGGGGGAATA TATCTCAATT TTGCACTCTA AGTTCTCACA TCAACCACCT TTTTCTATTT   
  
  
- AACATCCCAA TAACAGAACA TCTTTATGTA TATGTATTTA TAAATAGACA TGTTCTACAC GCATACATGT   
  
  
- TATTAGTAAC TGTTAATTGG ATTTGTAACG TTAATTGTAT TTGAAACAAA GAAGAATTTA AAATCTACCA   
  
  
- TTCAAGGAGG ATATTTTAGT AATGTTTTAG TATTTTAGGG TTATTGTGTG AAATAGATAT GCGTTTTTTT   
  
  
- AAACGGCCCC TTTTATATCA TGCACCTCCA TATATATCAT GCATGAAAGA GGAAACGAAA GTTTCTAGTA   
  
  
- GTTTAAAATT AATCAAATAT AATAAAATAT ATATGTGTGT GTGTGTGTGT GTGTTTCACA CACTCTGTAA   
  
  
- AAAAAATAAA AACAAAAACT TTATCTTCCT CTCTCCTTCT CTCACTTAAT CTAAAAAAAA AAAAAAATTA   
  
  
- ATTTCTATAC AATTCTAATG TACATCCACT CTAAAACTTT TTTTTTTGTC GGTTTAAATC AATACACTTT   
  
  
- AATGTAAAAA CGGGGCATAA ATAATAAGTA TAAAACAAAA TTAATCTACC AATTTCACCA TTAAACTATC   
  
  
- CTAAAATCAA ATGTTACTCA CAAAATAATT AATCATCTAT ATATAATTCA TACATAAAAG TTAATTTTTA   
  
  
- TTTCTTTTTT TTCGTCGTAG ATCGTACCGT TCGTGTTGTG TTCGTCTATA TATATATATA TATATATATA   
  
  
- TATTAACCTT TTCATAACAA CAGCACCTCT GTTTAAATCC AAGAACTCGT TTTTGAGTTG ATGAGAGATC   
  
  
- TCTCTCTCTC TCTCTCTCCA TCGTCATTGT GCTGAACACA TCTTTAACAC TCCCTGCTTA GCCAATTCTT   
  
  
- CACGTACAGA ACATAGAGAG AGATCTCGAT CTTTTTAGGA TTCACAAAGT AAAAAAAACC TTGTTTGTGT   
  
  
- CTTTTGGGGT TCTTAATATT ATTAATTTG

+     CAAT-box

| Site Name | Organism | Position | Strand | Matrix score. | sequence | function |
| --- | --- | --- | --- | --- | --- | --- |
| CAAT-box | Nicotiana glutinosa | 103 | + | 4 | CAAT |  |
| CAAT-box | Nicotiana glutinosa | 214 | + | 4 | CAAT |  |
| CAAT-box | Nicotiana glutinosa | 257 | - | 4 | CAAT |  |
| CAAT-box | Nicotiana glutinosa | 261 | + | 4 | CAAT |  |
| CAAT-box | Nicotiana glutinosa | 451 | + | 4 | CAAT |  |
| CAAT-box | Nicotiana glutinosa | 560 | - | 4 | CAAT |  |
| CAAT-box | Nicotiana glutinosa | 571 | - | 4 | CAAT |  |
| CAAT-box | Nicotiana glutinosa | 629 | + | 4 | CAAT |  |
| CAAT-box | Nicotiana glutinosa | 637 | - | 4 | CAAT |  |
| CAAT-box | Nicotiana glutinosa | 642 | + | 4 | CAAT |  |
| CAAT-box | Nicotiana glutinosa | 656 | - | 4 | CAAT |  |
| CAAT-box | Nicotiana glutinosa | 660 | + | 4 | CAAT |  |
| CAAT-box | Arabidopsis thaliana | 739 | + | 5 | CCAAT | common cis-acting element in promoter and enhancer regions |
| CAAT-box | Nicotiana glutinosa | 740 | + | 4 | CAAT |  |
| CAAT-box | Pisum sativum | 770 | - | 5 | CAAAT | common cis-acting element in promoter and enhancer regions |
| CAAT-box | Pisum sativum | 841 | + | 5 | CAAAT | common cis-acting element in promoter and enhancer regions |
| CAAT-box | Pisum sativum | 1032 | + | 5 | CAAAT | common cis-acting element in promoter and enhancer regions |
| CAAT-box | Pisum sativum | 1112 | - | 5 | CAAAT | common cis-acting element in promoter and enhancer regions |
| CAAT-box | Nicotiana glutinosa | 1133 | + | 4 | CAAT |  |
| CAAT-box | Nicotiana glutinosa | 1180 | + | 4 | CAAT |  |
| CAAT-box | Arabidopsis thaliana | 1264 | - | 5 | CCAAT | common cis-acting element in promoter and enhancer regions |
| CAAT-box | Nicotiana glutinosa | 1275 | - | 4 | CAAT |  |
| CAAT-box | Pisum sativum | 1291 | + | 5 | CAAAT | common cis-acting element in promoter and enhancer regions |
| CAAT-box | Nicotiana glutinosa | 1375 | - | 4 | CAAT |  |

>003403   
+ TAAAAATAGT GAAAGAACCA CCGGCCGGAT AAACAAGAGT AAAGCTGTCT GAAAATAAAC CCTACTCACA   
  
  
+ CACATGGTAT TTCTCATATT TTATTTTATA GTCAATTATT AAGATAATAA GCATTCTAAC CATTTATGTA   
  
  
+ TACATGTTCA TACCGCGAGC GTTGCTCGGA GGAACACATT TTTGTATCAA GTGATGTTGC AAAATGATGC   
  
  
+ ATACAATCAG TACACATCAC TCTAGCTACT TACTCCATCT TGTAGTATTG CAATGCAGTA AAATATAGCA   
  
  
+ CTAGTCCCAG ATTAATTATT ATGGTTCTCC TTTCAAACAC ATAGTTAATG GTTATAATGA TATACTTAAG   
  
  
+ GGCAAAATAT ATTACTTATA CAAAATAACT CGATGGGTTA CGTAAAATAC ATGAAGAATT CAGTTTAATA   
  
  
+ AGTTTAGTAT ATGCTTGCAT GAGTATGATA CAATATATAT GTGACAAAAT AAAATACAAG TCTAAATTTA   
  
  
+ CGACACCACC ACCCCCTTAT ATAGAGTTAA AACGTGAGAT TCAAGAGTGT AGTTGGTGGA AAAAGATAAA   
  
  
+ TTGTAGGGTT ATTGTCTTGT AGAAATACAT ATACATAAAT ATTTATCTGT ACAAGATGTG CGTATGTACA   
  
  
+ ATAATCATTG ACAATTAACC TAAACATTGC AATTAACATA AACTTTGTTT CTTCTTAAAT TTTAGATGGT   
  
  
+ AAGTTCCTCC TATAAAATCA TTACAAAATC ATAAAATCCC AATAACACAC TTTATCTATA CGCAAAAAAA   
  
  
+ TTTGCCGGGG AAAATATAGT ACGTGGAGGT ATATATAGTA CGTACTTTCT CCTTTGCTTT CAAAGATCAT   
  
  
+ CAAATTTTAA TTAGTTTATA TTATTTTATA TATACACACA CACACACACA CACAAAGTGT GTGAGACATT   
  
  
+ TTTTTTATTT TTGTTTTTGA AATAGAAGGA GAGAGGAAGA GAGTGAATTA GATTTTTTTT TTTTTTTAAT   
  
  
+ TAAAGATATG TTAAGATTAC ATGTAGGTGA GATTTTGAAA AAAAAAACAG CCAAATTTAG TTATGTGAAA   
  
  
+ TTACATTTTT GCCCCGTATT TATTATTCAT ATTTTGTTTT AATTAGATGG TTAAAGTGGT AATTTGATAG   
  
  
+ GATTTTAGTT TACAATGAGT GTTTTATTAA TTAGTAGATA TATATTAAGT ATGTATTTTC AATTAAAAAT   
  
  
+ AAAGAAAAAA AAGCAGCATC TAGCATGGCA AGCACAACAC AAGCAGATAT ATATATATAT ATATATATAT   
  
  
+ ATAATTGGAA AAGTATTGTT GTCGTGGAGA CAAATTTAGG TTCTTGAGCA AAAACTCAAC TACTCTCTAG   
  
  
+ AGAGAGAGAG AGAGAGAGGT AGCAGTAACA CGACTTGTGT AGAAATTGTG AGGGACGAAT CGGTTAAGAA   
  
  
+ GTGCATGTCT TGTATCTCTC TCTAGAGCTA GAAAAATCCT AAGTGTTTCA TTTTTTTTGG AACAAACACA   
  
  
+ GAAAACCCCA AGAATTATAA TAATTAAAC  

- ATTTTTATCA CTTTCTTGGT GGCCGGCCTA TTTGTTCTCA TTTCGACAGA CTTTTATTTG GGATGAGTGT   
  
  
- GTGTACCATA AAGAGTATAA AATAAAATAT CAGTTAATAA TTCTATTATT CGTAAGATTG GTAAATACAT   
  
  
- ATGTACAAGT ATGGCGCTCG CAACGAGCCT CCTTGTGTAA AAACATAGTT CACTACAACG TTTTACTACG   
  
  
- TATGTTAGTC ATGTGTAGTG AGATCGATGA ATGAGGTAGA ACATCATAAC GTTACGTCAT TTTATATCGT   
  
  
- GATCAGGGTC TAATTAATAA TACCAAGAGG AAAGTTTGTG TATCAATTAC CAATATTACT ATATGAATTC   
  
  
- CCGTTTTATA TAATGAATAT GTTTTATTGA GCTACCCAAT GCATTTTATG TACTTCTTAA GTCAAATTAT   
  
  
- TCAAATCATA TACGAACGTA CTCATACTAT GTTATATATA CACTGTTTTA TTTTATGTTC AGATTTAAAT   
  
  
- GCTGTGGTGG TGGGGGAATA TATCTCAATT TTGCACTCTA AGTTCTCACA TCAACCACCT TTTTCTATTT   
  
  
- AACATCCCAA TAACAGAACA TCTTTATGTA TATGTATTTA TAAATAGACA TGTTCTACAC GCATACATGT   
  
  
- TATTAGTAAC TGTTAATTGG ATTTGTAACG TTAATTGTAT TTGAAACAAA GAAGAATTTA AAATCTACCA   
  
  
- TTCAAGGAGG ATATTTTAGT AATGTTTTAG TATTTTAGGG TTATTGTGTG AAATAGATAT GCGTTTTTTT   
  
  
- AAACGGCCCC TTTTATATCA TGCACCTCCA TATATATCAT GCATGAAAGA GGAAACGAAA GTTTCTAGTA   
  
  
- GTTTAAAATT AATCAAATAT AATAAAATAT ATATGTGTGT GTGTGTGTGT GTGTTTCACA CACTCTGTAA   
  
  
- AAAAAATAAA AACAAAAACT TTATCTTCCT CTCTCCTTCT CTCACTTAAT CTAAAAAAAA AAAAAAATTA   
  
  
- ATTTCTATAC AATTCTAATG TACATCCACT CTAAAACTTT TTTTTTTGTC GGTTTAAATC AATACACTTT   
  
  
- AATGTAAAAA CGGGGCATAA ATAATAAGTA TAAAACAAAA TTAATCTACC AATTTCACCA TTAAACTATC   
  
  
- CTAAAATCAA ATGTTACTCA CAAAATAATT AATCATCTAT ATATAATTCA TACATAAAAG TTAATTTTTA   
  
  
- TTTCTTTTTT TTCGTCGTAG ATCGTACCGT TCGTGTTGTG TTCGTCTATA TATATATATA TATATATATA   
  
  
- TATTAACCTT TTCATAACAA CAGCACCTCT GTTTAAATCC AAGAACTCGT TTTTGAGTTG ATGAGAGATC   
  
  
- TCTCTCTCTC TCTCTCTCCA TCGTCATTGT GCTGAACACA TCTTTAACAC TCCCTGCTTA GCCAATTCTT   
  
  
- CACGTACAGA ACATAGAGAG AGATCTCGAT CTTTTTAGGA TTCACAAAGT AAAAAAAACC TTGTTTGTGT   
  
  
- CTTTTGGGGT TCTTAATATT ATTAATTTG

+     G-Box

| Site Name | Organism | Position | Strand | Matrix score. | sequence | function |
| --- | --- | --- | --- | --- | --- | --- |
| G-Box | Pisum sativum | 521 | - | 6 | CACGTT | cis-acting regulatory element involved in light responsiveness |

>003403   
+ TAAAAATAGT GAAAGAACCA CCGGCCGGAT AAACAAGAGT AAAGCTGTCT GAAAATAAAC CCTACTCACA   
  
  
+ CACATGGTAT TTCTCATATT TTATTTTATA GTCAATTATT AAGATAATAA GCATTCTAAC CATTTATGTA   
  
  
+ TACATGTTCA TACCGCGAGC GTTGCTCGGA GGAACACATT TTTGTATCAA GTGATGTTGC AAAATGATGC   
  
  
+ ATACAATCAG TACACATCAC TCTAGCTACT TACTCCATCT TGTAGTATTG CAATGCAGTA AAATATAGCA   
  
  
+ CTAGTCCCAG ATTAATTATT ATGGTTCTCC TTTCAAACAC ATAGTTAATG GTTATAATGA TATACTTAAG   
  
  
+ GGCAAAATAT ATTACTTATA CAAAATAACT CGATGGGTTA CGTAAAATAC ATGAAGAATT CAGTTTAATA   
  
  
+ AGTTTAGTAT ATGCTTGCAT GAGTATGATA CAATATATAT GTGACAAAAT AAAATACAAG TCTAAATTTA   
  
  
+ CGACACCACC ACCCCCTTAT ATAGAGTTAA AACGTGAGAT TCAAGAGTGT AGTTGGTGGA AAAAGATAAA   
  
  
+ TTGTAGGGTT ATTGTCTTGT AGAAATACAT ATACATAAAT ATTTATCTGT ACAAGATGTG CGTATGTACA   
  
  
+ ATAATCATTG ACAATTAACC TAAACATTGC AATTAACATA AACTTTGTTT CTTCTTAAAT TTTAGATGGT   
  
  
+ AAGTTCCTCC TATAAAATCA TTACAAAATC ATAAAATCCC AATAACACAC TTTATCTATA CGCAAAAAAA   
  
  
+ TTTGCCGGGG AAAATATAGT ACGTGGAGGT ATATATAGTA CGTACTTTCT CCTTTGCTTT CAAAGATCAT   
  
  
+ CAAATTTTAA TTAGTTTATA TTATTTTATA TATACACACA CACACACACA CACAAAGTGT GTGAGACATT   
  
  
+ TTTTTTATTT TTGTTTTTGA AATAGAAGGA GAGAGGAAGA GAGTGAATTA GATTTTTTTT TTTTTTTAAT   
  
  
+ TAAAGATATG TTAAGATTAC ATGTAGGTGA GATTTTGAAA AAAAAAACAG CCAAATTTAG TTATGTGAAA   
  
  
+ TTACATTTTT GCCCCGTATT TATTATTCAT ATTTTGTTTT AATTAGATGG TTAAAGTGGT AATTTGATAG   
  
  
+ GATTTTAGTT TACAATGAGT GTTTTATTAA TTAGTAGATA TATATTAAGT ATGTATTTTC AATTAAAAAT   
  
  
+ AAAGAAAAAA AAGCAGCATC TAGCATGGCA AGCACAACAC AAGCAGATAT ATATATATAT ATATATATAT   
  
  
+ ATAATTGGAA AAGTATTGTT GTCGTGGAGA CAAATTTAGG TTCTTGAGCA AAAACTCAAC TACTCTCTAG   
  
  
+ AGAGAGAGAG AGAGAGAGGT AGCAGTAACA CGACTTGTGT AGAAATTGTG AGGGACGAAT CGGTTAAGAA   
  
  
+ GTGCATGTCT TGTATCTCTC TCTAGAGCTA GAAAAATCCT AAGTGTTTCA TTTTTTTTGG AACAAACACA   
  
  
+ GAAAACCCCA AGAATTATAA TAATTAAAC  

- ATTTTTATCA CTTTCTTGGT GGCCGGCCTA TTTGTTCTCA TTTCGACAGA CTTTTATTTG GGATGAGTGT   
  
  
- GTGTACCATA AAGAGTATAA AATAAAATAT CAGTTAATAA TTCTATTATT CGTAAGATTG GTAAATACAT   
  
  
- ATGTACAAGT ATGGCGCTCG CAACGAGCCT CCTTGTGTAA AAACATAGTT CACTACAACG TTTTACTACG   
  
  
- TATGTTAGTC ATGTGTAGTG AGATCGATGA ATGAGGTAGA ACATCATAAC GTTACGTCAT TTTATATCGT   
  
  
- GATCAGGGTC TAATTAATAA TACCAAGAGG AAAGTTTGTG TATCAATTAC CAATATTACT ATATGAATTC   
  
  
- CCGTTTTATA TAATGAATAT GTTTTATTGA GCTACCCAAT GCATTTTATG TACTTCTTAA GTCAAATTAT   
  
  
- TCAAATCATA TACGAACGTA CTCATACTAT GTTATATATA CACTGTTTTA TTTTATGTTC AGATTTAAAT   
  
  
- GCTGTGGTGG TGGGGGAATA TATCTCAATT TTGCACTCTA AGTTCTCACA TCAACCACCT TTTTCTATTT   
  
  
- AACATCCCAA TAACAGAACA TCTTTATGTA TATGTATTTA TAAATAGACA TGTTCTACAC GCATACATGT   
  
  
- TATTAGTAAC TGTTAATTGG ATTTGTAACG TTAATTGTAT TTGAAACAAA GAAGAATTTA AAATCTACCA   
  
  
- TTCAAGGAGG ATATTTTAGT AATGTTTTAG TATTTTAGGG TTATTGTGTG AAATAGATAT GCGTTTTTTT   
  
  
- AAACGGCCCC TTTTATATCA TGCACCTCCA TATATATCAT GCATGAAAGA GGAAACGAAA GTTTCTAGTA   
  
  
- GTTTAAAATT AATCAAATAT AATAAAATAT ATATGTGTGT GTGTGTGTGT GTGTTTCACA CACTCTGTAA   
  
  
- AAAAAATAAA AACAAAAACT TTATCTTCCT CTCTCCTTCT CTCACTTAAT CTAAAAAAAA AAAAAAATTA   
  
  
- ATTTCTATAC AATTCTAATG TACATCCACT CTAAAACTTT TTTTTTTGTC GGTTTAAATC AATACACTTT   
  
  
- AATGTAAAAA CGGGGCATAA ATAATAAGTA TAAAACAAAA TTAATCTACC AATTTCACCA TTAAACTATC   
  
  
- CTAAAATCAA ATGTTACTCA CAAAATAATT AATCATCTAT ATATAATTCA TACATAAAAG TTAATTTTTA   
  
  
- TTTCTTTTTT TTCGTCGTAG ATCGTACCGT TCGTGTTGTG TTCGTCTATA TATATATATA TATATATATA   
  
  
- TATTAACCTT TTCATAACAA CAGCACCTCT GTTTAAATCC AAGAACTCGT TTTTGAGTTG ATGAGAGATC   
  
  
- TCTCTCTCTC TCTCTCTCCA TCGTCATTGT GCTGAACACA TCTTTAACAC TCCCTGCTTA GCCAATTCTT   
  
  
- CACGTACAGA ACATAGAGAG AGATCTCGAT CTTTTTAGGA TTCACAAAGT AAAAAAAACC TTGTTTGTGT   
  
  
- CTTTTGGGGT TCTTAATATT ATTAATTTG

+     G-box

| Site Name | Organism | Position | Strand | Matrix score. | sequence | function |
| --- | --- | --- | --- | --- | --- | --- |
| G-box | Arabidopsis thaliana | 790 | + | 6 | TACGTG | cis-acting regulatory element involved in light responsiveness |
| G-box | Zea mays | 1359 | + | 6 | CACGAC | cis-acting regulatory element involved in light responsiveness |
| G-box | Zea mays | 1281 | - | 6 | CACGAC | cis-acting regulatory element involved in light responsiveness |

>003403   
+ TAAAAATAGT GAAAGAACCA CCGGCCGGAT AAACAAGAGT AAAGCTGTCT GAAAATAAAC CCTACTCACA   
  
  
+ CACATGGTAT TTCTCATATT TTATTTTATA GTCAATTATT AAGATAATAA GCATTCTAAC CATTTATGTA   
  
  
+ TACATGTTCA TACCGCGAGC GTTGCTCGGA GGAACACATT TTTGTATCAA GTGATGTTGC AAAATGATGC   
  
  
+ ATACAATCAG TACACATCAC TCTAGCTACT TACTCCATCT TGTAGTATTG CAATGCAGTA AAATATAGCA   
  
  
+ CTAGTCCCAG ATTAATTATT ATGGTTCTCC TTTCAAACAC ATAGTTAATG GTTATAATGA TATACTTAAG   
  
  
+ GGCAAAATAT ATTACTTATA CAAAATAACT CGATGGGTTA CGTAAAATAC ATGAAGAATT CAGTTTAATA   
  
  
+ AGTTTAGTAT ATGCTTGCAT GAGTATGATA CAATATATAT GTGACAAAAT AAAATACAAG TCTAAATTTA   
  
  
+ CGACACCACC ACCCCCTTAT ATAGAGTTAA AACGTGAGAT TCAAGAGTGT AGTTGGTGGA AAAAGATAAA   
  
  
+ TTGTAGGGTT ATTGTCTTGT AGAAATACAT ATACATAAAT ATTTATCTGT ACAAGATGTG CGTATGTACA   
  
  
+ ATAATCATTG ACAATTAACC TAAACATTGC AATTAACATA AACTTTGTTT CTTCTTAAAT TTTAGATGGT   
  
  
+ AAGTTCCTCC TATAAAATCA TTACAAAATC ATAAAATCCC AATAACACAC TTTATCTATA CGCAAAAAAA   
  
  
+ TTTGCCGGGG AAAATATAGT ACGTGGAGGT ATATATAGTA CGTACTTTCT CCTTTGCTTT CAAAGATCAT   
  
  
+ CAAATTTTAA TTAGTTTATA TTATTTTATA TATACACACA CACACACACA CACAAAGTGT GTGAGACATT   
  
  
+ TTTTTTATTT TTGTTTTTGA AATAGAAGGA GAGAGGAAGA GAGTGAATTA GATTTTTTTT TTTTTTTAAT   
  
  
+ TAAAGATATG TTAAGATTAC ATGTAGGTGA GATTTTGAAA AAAAAAACAG CCAAATTTAG TTATGTGAAA   
  
  
+ TTACATTTTT GCCCCGTATT TATTATTCAT ATTTTGTTTT AATTAGATGG TTAAAGTGGT AATTTGATAG   
  
  
+ GATTTTAGTT TACAATGAGT GTTTTATTAA TTAGTAGATA TATATTAAGT ATGTATTTTC AATTAAAAAT   
  
  
+ AAAGAAAAAA AAGCAGCATC TAGCATGGCA AGCACAACAC AAGCAGATAT ATATATATAT ATATATATAT   
  
  
+ ATAATTGGAA AAGTATTGTT GTCGTGGAGA CAAATTTAGG TTCTTGAGCA AAAACTCAAC TACTCTCTAG   
  
  
+ AGAGAGAGAG AGAGAGAGGT AGCAGTAACA CGACTTGTGT AGAAATTGTG AGGGACGAAT CGGTTAAGAA   
  
  
+ GTGCATGTCT TGTATCTCTC TCTAGAGCTA GAAAAATCCT AAGTGTTTCA TTTTTTTTGG AACAAACACA   
  
  
+ GAAAACCCCA AGAATTATAA TAATTAAAC  

- ATTTTTATCA CTTTCTTGGT GGCCGGCCTA TTTGTTCTCA TTTCGACAGA CTTTTATTTG GGATGAGTGT   
  
  
- GTGTACCATA AAGAGTATAA AATAAAATAT CAGTTAATAA TTCTATTATT CGTAAGATTG GTAAATACAT   
  
  
- ATGTACAAGT ATGGCGCTCG CAACGAGCCT CCTTGTGTAA AAACATAGTT CACTACAACG TTTTACTACG   
  
  
- TATGTTAGTC ATGTGTAGTG AGATCGATGA ATGAGGTAGA ACATCATAAC GTTACGTCAT TTTATATCGT   
  
  
- GATCAGGGTC TAATTAATAA TACCAAGAGG AAAGTTTGTG TATCAATTAC CAATATTACT ATATGAATTC   
  
  
- CCGTTTTATA TAATGAATAT GTTTTATTGA GCTACCCAAT GCATTTTATG TACTTCTTAA GTCAAATTAT   
  
  
- TCAAATCATA TACGAACGTA CTCATACTAT GTTATATATA CACTGTTTTA TTTTATGTTC AGATTTAAAT   
  
  
- GCTGTGGTGG TGGGGGAATA TATCTCAATT TTGCACTCTA AGTTCTCACA TCAACCACCT TTTTCTATTT   
  
  
- AACATCCCAA TAACAGAACA TCTTTATGTA TATGTATTTA TAAATAGACA TGTTCTACAC GCATACATGT   
  
  
- TATTAGTAAC TGTTAATTGG ATTTGTAACG TTAATTGTAT TTGAAACAAA GAAGAATTTA AAATCTACCA   
  
  
- TTCAAGGAGG ATATTTTAGT AATGTTTTAG TATTTTAGGG TTATTGTGTG AAATAGATAT GCGTTTTTTT   
  
  
- AAACGGCCCC TTTTATATCA TGCACCTCCA TATATATCAT GCATGAAAGA GGAAACGAAA GTTTCTAGTA   
  
  
- GTTTAAAATT AATCAAATAT AATAAAATAT ATATGTGTGT GTGTGTGTGT GTGTTTCACA CACTCTGTAA   
  
  
- AAAAAATAAA AACAAAAACT TTATCTTCCT CTCTCCTTCT CTCACTTAAT CTAAAAAAAA AAAAAAATTA   
  
  
- ATTTCTATAC AATTCTAATG TACATCCACT CTAAAACTTT TTTTTTTGTC GGTTTAAATC AATACACTTT   
  
  
- AATGTAAAAA CGGGGCATAA ATAATAAGTA TAAAACAAAA TTAATCTACC AATTTCACCA TTAAACTATC   
  
  
- CTAAAATCAA ATGTTACTCA CAAAATAATT AATCATCTAT ATATAATTCA TACATAAAAG TTAATTTTTA   
  
  
- TTTCTTTTTT TTCGTCGTAG ATCGTACCGT TCGTGTTGTG TTCGTCTATA TATATATATA TATATATATA   
  
  
- TATTAACCTT TTCATAACAA CAGCACCTCT GTTTAAATCC AAGAACTCGT TTTTGAGTTG ATGAGAGATC   
  
  
- TCTCTCTCTC TCTCTCTCCA TCGTCATTGT GCTGAACACA TCTTTAACAC TCCCTGCTTA GCCAATTCTT   
  
  
- CACGTACAGA ACATAGAGAG AGATCTCGAT CTTTTTAGGA TTCACAAAGT AAAAAAAACC TTGTTTGTGT   
  
  
- CTTTTGGGGT TCTTAATATT ATTAATTTG

+     GA-motif

| Site Name | Organism | Position | Strand | Matrix score. | sequence | function |
| --- | --- | --- | --- | --- | --- | --- |
| GA-motif | Arabidopsis thaliana | 752 | - | 8 | ATAGATAA | part of a light responsive element |

>003403   
+ TAAAAATAGT GAAAGAACCA CCGGCCGGAT AAACAAGAGT AAAGCTGTCT GAAAATAAAC CCTACTCACA   
  
  
+ CACATGGTAT TTCTCATATT TTATTTTATA GTCAATTATT AAGATAATAA GCATTCTAAC CATTTATGTA   
  
  
+ TACATGTTCA TACCGCGAGC GTTGCTCGGA GGAACACATT TTTGTATCAA GTGATGTTGC AAAATGATGC   
  
  
+ ATACAATCAG TACACATCAC TCTAGCTACT TACTCCATCT TGTAGTATTG CAATGCAGTA AAATATAGCA   
  
  
+ CTAGTCCCAG ATTAATTATT ATGGTTCTCC TTTCAAACAC ATAGTTAATG GTTATAATGA TATACTTAAG   
  
  
+ GGCAAAATAT ATTACTTATA CAAAATAACT CGATGGGTTA CGTAAAATAC ATGAAGAATT CAGTTTAATA   
  
  
+ AGTTTAGTAT ATGCTTGCAT GAGTATGATA CAATATATAT GTGACAAAAT AAAATACAAG TCTAAATTTA   
  
  
+ CGACACCACC ACCCCCTTAT ATAGAGTTAA AACGTGAGAT TCAAGAGTGT AGTTGGTGGA AAAAGATAAA   
  
  
+ TTGTAGGGTT ATTGTCTTGT AGAAATACAT ATACATAAAT ATTTATCTGT ACAAGATGTG CGTATGTACA   
  
  
+ ATAATCATTG ACAATTAACC TAAACATTGC AATTAACATA AACTTTGTTT CTTCTTAAAT TTTAGATGGT   
  
  
+ AAGTTCCTCC TATAAAATCA TTACAAAATC ATAAAATCCC AATAACACAC TTTATCTATA CGCAAAAAAA   
  
  
+ TTTGCCGGGG AAAATATAGT ACGTGGAGGT ATATATAGTA CGTACTTTCT CCTTTGCTTT CAAAGATCAT   
  
  
+ CAAATTTTAA TTAGTTTATA TTATTTTATA TATACACACA CACACACACA CACAAAGTGT GTGAGACATT   
  
  
+ TTTTTTATTT TTGTTTTTGA AATAGAAGGA GAGAGGAAGA GAGTGAATTA GATTTTTTTT TTTTTTTAAT   
  
  
+ TAAAGATATG TTAAGATTAC ATGTAGGTGA GATTTTGAAA AAAAAAACAG CCAAATTTAG TTATGTGAAA   
  
  
+ TTACATTTTT GCCCCGTATT TATTATTCAT ATTTTGTTTT AATTAGATGG TTAAAGTGGT AATTTGATAG   
  
  
+ GATTTTAGTT TACAATGAGT GTTTTATTAA TTAGTAGATA TATATTAAGT ATGTATTTTC AATTAAAAAT   
  
  
+ AAAGAAAAAA AAGCAGCATC TAGCATGGCA AGCACAACAC AAGCAGATAT ATATATATAT ATATATATAT   
  
  
+ ATAATTGGAA AAGTATTGTT GTCGTGGAGA CAAATTTAGG TTCTTGAGCA AAAACTCAAC TACTCTCTAG   
  
  
+ AGAGAGAGAG AGAGAGAGGT AGCAGTAACA CGACTTGTGT AGAAATTGTG AGGGACGAAT CGGTTAAGAA   
  
  
+ GTGCATGTCT TGTATCTCTC TCTAGAGCTA GAAAAATCCT AAGTGTTTCA TTTTTTTTGG AACAAACACA   
  
  
+ GAAAACCCCA AGAATTATAA TAATTAAAC  

- ATTTTTATCA CTTTCTTGGT GGCCGGCCTA TTTGTTCTCA TTTCGACAGA CTTTTATTTG GGATGAGTGT   
  
  
- GTGTACCATA AAGAGTATAA AATAAAATAT CAGTTAATAA TTCTATTATT CGTAAGATTG GTAAATACAT   
  
  
- ATGTACAAGT ATGGCGCTCG CAACGAGCCT CCTTGTGTAA AAACATAGTT CACTACAACG TTTTACTACG   
  
  
- TATGTTAGTC ATGTGTAGTG AGATCGATGA ATGAGGTAGA ACATCATAAC GTTACGTCAT TTTATATCGT   
  
  
- GATCAGGGTC TAATTAATAA TACCAAGAGG AAAGTTTGTG TATCAATTAC CAATATTACT ATATGAATTC   
  
  
- CCGTTTTATA TAATGAATAT GTTTTATTGA GCTACCCAAT GCATTTTATG TACTTCTTAA GTCAAATTAT   
  
  
- TCAAATCATA TACGAACGTA CTCATACTAT GTTATATATA CACTGTTTTA TTTTATGTTC AGATTTAAAT   
  
  
- GCTGTGGTGG TGGGGGAATA TATCTCAATT TTGCACTCTA AGTTCTCACA TCAACCACCT TTTTCTATTT   
  
  
- AACATCCCAA TAACAGAACA TCTTTATGTA TATGTATTTA TAAATAGACA TGTTCTACAC GCATACATGT   
  
  
- TATTAGTAAC TGTTAATTGG ATTTGTAACG TTAATTGTAT TTGAAACAAA GAAGAATTTA AAATCTACCA   
  
  
- TTCAAGGAGG ATATTTTAGT AATGTTTTAG TATTTTAGGG TTATTGTGTG AAATAGATAT GCGTTTTTTT   
  
  
- AAACGGCCCC TTTTATATCA TGCACCTCCA TATATATCAT GCATGAAAGA GGAAACGAAA GTTTCTAGTA   
  
  
- GTTTAAAATT AATCAAATAT AATAAAATAT ATATGTGTGT GTGTGTGTGT GTGTTTCACA CACTCTGTAA   
  
  
- AAAAAATAAA AACAAAAACT TTATCTTCCT CTCTCCTTCT CTCACTTAAT CTAAAAAAAA AAAAAAATTA   
  
  
- ATTTCTATAC AATTCTAATG TACATCCACT CTAAAACTTT TTTTTTTGTC GGTTTAAATC AATACACTTT   
  
  
- AATGTAAAAA CGGGGCATAA ATAATAAGTA TAAAACAAAA TTAATCTACC AATTTCACCA TTAAACTATC   
  
  
- CTAAAATCAA ATGTTACTCA CAAAATAATT AATCATCTAT ATATAATTCA TACATAAAAG TTAATTTTTA   
  
  
- TTTCTTTTTT TTCGTCGTAG ATCGTACCGT TCGTGTTGTG TTCGTCTATA TATATATATA TATATATATA   
  
  
- TATTAACCTT TTCATAACAA CAGCACCTCT GTTTAAATCC AAGAACTCGT TTTTGAGTTG ATGAGAGATC   
  
  
- TCTCTCTCTC TCTCTCTCCA TCGTCATTGT GCTGAACACA TCTTTAACAC TCCCTGCTTA GCCAATTCTT   
  
  
- CACGTACAGA ACATAGAGAG AGATCTCGAT CTTTTTAGGA TTCACAAAGT AAAAAAAACC TTGTTTGTGT   
  
  
- CTTTTGGGGT TCTTAATATT ATTAATTTG

+     GATA-motif

| Site Name | Organism | Position | Strand | Matrix score. | sequence | function |
| --- | --- | --- | --- | --- | --- | --- |
| GATA-motif | Arabidopsis thaliana | 1116 | + | 7 | GATAGGA | part of a light responsive element |

>003403   
+ TAAAAATAGT GAAAGAACCA CCGGCCGGAT AAACAAGAGT AAAGCTGTCT GAAAATAAAC CCTACTCACA   
  
  
+ CACATGGTAT TTCTCATATT TTATTTTATA GTCAATTATT AAGATAATAA GCATTCTAAC CATTTATGTA   
  
  
+ TACATGTTCA TACCGCGAGC GTTGCTCGGA GGAACACATT TTTGTATCAA GTGATGTTGC AAAATGATGC   
  
  
+ ATACAATCAG TACACATCAC TCTAGCTACT TACTCCATCT TGTAGTATTG CAATGCAGTA AAATATAGCA   
  
  
+ CTAGTCCCAG ATTAATTATT ATGGTTCTCC TTTCAAACAC ATAGTTAATG GTTATAATGA TATACTTAAG   
  
  
+ GGCAAAATAT ATTACTTATA CAAAATAACT CGATGGGTTA CGTAAAATAC ATGAAGAATT CAGTTTAATA   
  
  
+ AGTTTAGTAT ATGCTTGCAT GAGTATGATA CAATATATAT GTGACAAAAT AAAATACAAG TCTAAATTTA   
  
  
+ CGACACCACC ACCCCCTTAT ATAGAGTTAA AACGTGAGAT TCAAGAGTGT AGTTGGTGGA AAAAGATAAA   
  
  
+ TTGTAGGGTT ATTGTCTTGT AGAAATACAT ATACATAAAT ATTTATCTGT ACAAGATGTG CGTATGTACA   
  
  
+ ATAATCATTG ACAATTAACC TAAACATTGC AATTAACATA AACTTTGTTT CTTCTTAAAT TTTAGATGGT   
  
  
+ AAGTTCCTCC TATAAAATCA TTACAAAATC ATAAAATCCC AATAACACAC TTTATCTATA CGCAAAAAAA   
  
  
+ TTTGCCGGGG AAAATATAGT ACGTGGAGGT ATATATAGTA CGTACTTTCT CCTTTGCTTT CAAAGATCAT   
  
  
+ CAAATTTTAA TTAGTTTATA TTATTTTATA TATACACACA CACACACACA CACAAAGTGT GTGAGACATT   
  
  
+ TTTTTTATTT TTGTTTTTGA AATAGAAGGA GAGAGGAAGA GAGTGAATTA GATTTTTTTT TTTTTTTAAT   
  
  
+ TAAAGATATG TTAAGATTAC ATGTAGGTGA GATTTTGAAA AAAAAAACAG CCAAATTTAG TTATGTGAAA   
  
  
+ TTACATTTTT GCCCCGTATT TATTATTCAT ATTTTGTTTT AATTAGATGG TTAAAGTGGT AATTTGATAG   
  
  
+ GATTTTAGTT TACAATGAGT GTTTTATTAA TTAGTAGATA TATATTAAGT ATGTATTTTC AATTAAAAAT   
  
  
+ AAAGAAAAAA AAGCAGCATC TAGCATGGCA AGCACAACAC AAGCAGATAT ATATATATAT ATATATATAT   
  
  
+ ATAATTGGAA AAGTATTGTT GTCGTGGAGA CAAATTTAGG TTCTTGAGCA AAAACTCAAC TACTCTCTAG   
  
  
+ AGAGAGAGAG AGAGAGAGGT AGCAGTAACA CGACTTGTGT AGAAATTGTG AGGGACGAAT CGGTTAAGAA   
  
  
+ GTGCATGTCT TGTATCTCTC TCTAGAGCTA GAAAAATCCT AAGTGTTTCA TTTTTTTTGG AACAAACACA   
  
  
+ GAAAACCCCA AGAATTATAA TAATTAAAC  

- ATTTTTATCA CTTTCTTGGT GGCCGGCCTA TTTGTTCTCA TTTCGACAGA CTTTTATTTG GGATGAGTGT   
  
  
- GTGTACCATA AAGAGTATAA AATAAAATAT CAGTTAATAA TTCTATTATT CGTAAGATTG GTAAATACAT   
  
  
- ATGTACAAGT ATGGCGCTCG CAACGAGCCT CCTTGTGTAA AAACATAGTT CACTACAACG TTTTACTACG   
  
  
- TATGTTAGTC ATGTGTAGTG AGATCGATGA ATGAGGTAGA ACATCATAAC GTTACGTCAT TTTATATCGT   
  
  
- GATCAGGGTC TAATTAATAA TACCAAGAGG AAAGTTTGTG TATCAATTAC CAATATTACT ATATGAATTC   
  
  
- CCGTTTTATA TAATGAATAT GTTTTATTGA GCTACCCAAT GCATTTTATG TACTTCTTAA GTCAAATTAT   
  
  
- TCAAATCATA TACGAACGTA CTCATACTAT GTTATATATA CACTGTTTTA TTTTATGTTC AGATTTAAAT   
  
  
- GCTGTGGTGG TGGGGGAATA TATCTCAATT TTGCACTCTA AGTTCTCACA TCAACCACCT TTTTCTATTT   
  
  
- AACATCCCAA TAACAGAACA TCTTTATGTA TATGTATTTA TAAATAGACA TGTTCTACAC GCATACATGT   
  
  
- TATTAGTAAC TGTTAATTGG ATTTGTAACG TTAATTGTAT TTGAAACAAA GAAGAATTTA AAATCTACCA   
  
  
- TTCAAGGAGG ATATTTTAGT AATGTTTTAG TATTTTAGGG TTATTGTGTG AAATAGATAT GCGTTTTTTT   
  
  
- AAACGGCCCC TTTTATATCA TGCACCTCCA TATATATCAT GCATGAAAGA GGAAACGAAA GTTTCTAGTA   
  
  
- GTTTAAAATT AATCAAATAT AATAAAATAT ATATGTGTGT GTGTGTGTGT GTGTTTCACA CACTCTGTAA   
  
  
- AAAAAATAAA AACAAAAACT TTATCTTCCT CTCTCCTTCT CTCACTTAAT CTAAAAAAAA AAAAAAATTA   
  
  
- ATTTCTATAC AATTCTAATG TACATCCACT CTAAAACTTT TTTTTTTGTC GGTTTAAATC AATACACTTT   
  
  
- AATGTAAAAA CGGGGCATAA ATAATAAGTA TAAAACAAAA TTAATCTACC AATTTCACCA TTAAACTATC   
  
  
- CTAAAATCAA ATGTTACTCA CAAAATAATT AATCATCTAT ATATAATTCA TACATAAAAG TTAATTTTTA   
  
  
- TTTCTTTTTT TTCGTCGTAG ATCGTACCGT TCGTGTTGTG TTCGTCTATA TATATATATA TATATATATA   
  
  
- TATTAACCTT TTCATAACAA CAGCACCTCT GTTTAAATCC AAGAACTCGT TTTTGAGTTG ATGAGAGATC   
  
  
- TCTCTCTCTC TCTCTCTCCA TCGTCATTGT GCTGAACACA TCTTTAACAC TCCCTGCTTA GCCAATTCTT   
  
  
- CACGTACAGA ACATAGAGAG AGATCTCGAT CTTTTTAGGA TTCACAAAGT AAAAAAAACC TTGTTTGTGT   
  
  
- CTTTTGGGGT TCTTAATATT ATTAATTTG

+     GT1-motif

| Site Name | Organism | Position | Strand | Matrix score. | sequence | function |
| --- | --- | --- | --- | --- | --- | --- |
| GT1-motif | Avena sativa | 644 | - | 7 | GGTTAAT | light responsive element |
| GT1-motif | Arabidopsis thaliana | 1099 | + | 6 | GGTTAA | light responsive element |
| GT1-motif | Arabidopsis thaliana | 645 | - | 6 | GGTTAA | light responsive element |
| GT1-motif | Arabidopsis thaliana | 1392 | + | 6 | GGTTAA | light responsive element |

>003403   
+ TAAAAATAGT GAAAGAACCA CCGGCCGGAT AAACAAGAGT AAAGCTGTCT GAAAATAAAC CCTACTCACA   
  
  
+ CACATGGTAT TTCTCATATT TTATTTTATA GTCAATTATT AAGATAATAA GCATTCTAAC CATTTATGTA   
  
  
+ TACATGTTCA TACCGCGAGC GTTGCTCGGA GGAACACATT TTTGTATCAA GTGATGTTGC AAAATGATGC   
  
  
+ ATACAATCAG TACACATCAC TCTAGCTACT TACTCCATCT TGTAGTATTG CAATGCAGTA AAATATAGCA   
  
  
+ CTAGTCCCAG ATTAATTATT ATGGTTCTCC TTTCAAACAC ATAGTTAATG GTTATAATGA TATACTTAAG   
  
  
+ GGCAAAATAT ATTACTTATA CAAAATAACT CGATGGGTTA CGTAAAATAC ATGAAGAATT CAGTTTAATA   
  
  
+ AGTTTAGTAT ATGCTTGCAT GAGTATGATA CAATATATAT GTGACAAAAT AAAATACAAG TCTAAATTTA   
  
  
+ CGACACCACC ACCCCCTTAT ATAGAGTTAA AACGTGAGAT TCAAGAGTGT AGTTGGTGGA AAAAGATAAA   
  
  
+ TTGTAGGGTT ATTGTCTTGT AGAAATACAT ATACATAAAT ATTTATCTGT ACAAGATGTG CGTATGTACA   
  
  
+ ATAATCATTG ACAATTAACC TAAACATTGC AATTAACATA AACTTTGTTT CTTCTTAAAT TTTAGATGGT   
  
  
+ AAGTTCCTCC TATAAAATCA TTACAAAATC ATAAAATCCC AATAACACAC TTTATCTATA CGCAAAAAAA   
  
  
+ TTTGCCGGGG AAAATATAGT ACGTGGAGGT ATATATAGTA CGTACTTTCT CCTTTGCTTT CAAAGATCAT   
  
  
+ CAAATTTTAA TTAGTTTATA TTATTTTATA TATACACACA CACACACACA CACAAAGTGT GTGAGACATT   
  
  
+ TTTTTTATTT TTGTTTTTGA AATAGAAGGA GAGAGGAAGA GAGTGAATTA GATTTTTTTT TTTTTTTAAT   
  
  
+ TAAAGATATG TTAAGATTAC ATGTAGGTGA GATTTTGAAA AAAAAAACAG CCAAATTTAG TTATGTGAAA   
  
  
+ TTACATTTTT GCCCCGTATT TATTATTCAT ATTTTGTTTT AATTAGATGG TTAAAGTGGT AATTTGATAG   
  
  
+ GATTTTAGTT TACAATGAGT GTTTTATTAA TTAGTAGATA TATATTAAGT ATGTATTTTC AATTAAAAAT   
  
  
+ AAAGAAAAAA AAGCAGCATC TAGCATGGCA AGCACAACAC AAGCAGATAT ATATATATAT ATATATATAT   
  
  
+ ATAATTGGAA AAGTATTGTT GTCGTGGAGA CAAATTTAGG TTCTTGAGCA AAAACTCAAC TACTCTCTAG   
  
  
+ AGAGAGAGAG AGAGAGAGGT AGCAGTAACA CGACTTGTGT AGAAATTGTG AGGGACGAAT CGGTTAAGAA   
  
  
+ GTGCATGTCT TGTATCTCTC TCTAGAGCTA GAAAAATCCT AAGTGTTTCA TTTTTTTTGG AACAAACACA   
  
  
+ GAAAACCCCA AGAATTATAA TAATTAAAC  

- ATTTTTATCA CTTTCTTGGT GGCCGGCCTA TTTGTTCTCA TTTCGACAGA CTTTTATTTG GGATGAGTGT   
  
  
- GTGTACCATA AAGAGTATAA AATAAAATAT CAGTTAATAA TTCTATTATT CGTAAGATTG GTAAATACAT   
  
  
- ATGTACAAGT ATGGCGCTCG CAACGAGCCT CCTTGTGTAA AAACATAGTT CACTACAACG TTTTACTACG   
  
  
- TATGTTAGTC ATGTGTAGTG AGATCGATGA ATGAGGTAGA ACATCATAAC GTTACGTCAT TTTATATCGT   
  
  
- GATCAGGGTC TAATTAATAA TACCAAGAGG AAAGTTTGTG TATCAATTAC CAATATTACT ATATGAATTC   
  
  
- CCGTTTTATA TAATGAATAT GTTTTATTGA GCTACCCAAT GCATTTTATG TACTTCTTAA GTCAAATTAT   
  
  
- TCAAATCATA TACGAACGTA CTCATACTAT GTTATATATA CACTGTTTTA TTTTATGTTC AGATTTAAAT   
  
  
- GCTGTGGTGG TGGGGGAATA TATCTCAATT TTGCACTCTA AGTTCTCACA TCAACCACCT TTTTCTATTT   
  
  
- AACATCCCAA TAACAGAACA TCTTTATGTA TATGTATTTA TAAATAGACA TGTTCTACAC GCATACATGT   
  
  
- TATTAGTAAC TGTTAATTGG ATTTGTAACG TTAATTGTAT TTGAAACAAA GAAGAATTTA AAATCTACCA   
  
  
- TTCAAGGAGG ATATTTTAGT AATGTTTTAG TATTTTAGGG TTATTGTGTG AAATAGATAT GCGTTTTTTT   
  
  
- AAACGGCCCC TTTTATATCA TGCACCTCCA TATATATCAT GCATGAAAGA GGAAACGAAA GTTTCTAGTA   
  
  
- GTTTAAAATT AATCAAATAT AATAAAATAT ATATGTGTGT GTGTGTGTGT GTGTTTCACA CACTCTGTAA   
  
  
- AAAAAATAAA AACAAAAACT TTATCTTCCT CTCTCCTTCT CTCACTTAAT CTAAAAAAAA AAAAAAATTA   
  
  
- ATTTCTATAC AATTCTAATG TACATCCACT CTAAAACTTT TTTTTTTGTC GGTTTAAATC AATACACTTT   
  
  
- AATGTAAAAA CGGGGCATAA ATAATAAGTA TAAAACAAAA TTAATCTACC AATTTCACCA TTAAACTATC   
  
  
- CTAAAATCAA ATGTTACTCA CAAAATAATT AATCATCTAT ATATAATTCA TACATAAAAG TTAATTTTTA   
  
  
- TTTCTTTTTT TTCGTCGTAG ATCGTACCGT TCGTGTTGTG TTCGTCTATA TATATATATA TATATATATA   
  
  
- TATTAACCTT TTCATAACAA CAGCACCTCT GTTTAAATCC AAGAACTCGT TTTTGAGTTG ATGAGAGATC   
  
  
- TCTCTCTCTC TCTCTCTCCA TCGTCATTGT GCTGAACACA TCTTTAACAC TCCCTGCTTA GCCAATTCTT   
  
  
- CACGTACAGA ACATAGAGAG AGATCTCGAT CTTTTTAGGA TTCACAAAGT AAAAAAAACC TTGTTTGTGT   
  
  
- CTTTTGGGGT TCTTAATATT ATTAATTTG

+     L-box

| Site Name | Organism | Position | Strand | Matrix score. | sequence | function |
| --- | --- | --- | --- | --- | --- | --- |
| L-box | Petroselinum crispum | 1003 | - | 10 | ATCCCACCTAC | part of a light responsive element |

>003403   
+ TAAAAATAGT GAAAGAACCA CCGGCCGGAT AAACAAGAGT AAAGCTGTCT GAAAATAAAC CCTACTCACA   
  
  
+ CACATGGTAT TTCTCATATT TTATTTTATA GTCAATTATT AAGATAATAA GCATTCTAAC CATTTATGTA   
  
  
+ TACATGTTCA TACCGCGAGC GTTGCTCGGA GGAACACATT TTTGTATCAA GTGATGTTGC AAAATGATGC   
  
  
+ ATACAATCAG TACACATCAC TCTAGCTACT TACTCCATCT TGTAGTATTG CAATGCAGTA AAATATAGCA   
  
  
+ CTAGTCCCAG ATTAATTATT ATGGTTCTCC TTTCAAACAC ATAGTTAATG GTTATAATGA TATACTTAAG   
  
  
+ GGCAAAATAT ATTACTTATA CAAAATAACT CGATGGGTTA CGTAAAATAC ATGAAGAATT CAGTTTAATA   
  
  
+ AGTTTAGTAT ATGCTTGCAT GAGTATGATA CAATATATAT GTGACAAAAT AAAATACAAG TCTAAATTTA   
  
  
+ CGACACCACC ACCCCCTTAT ATAGAGTTAA AACGTGAGAT TCAAGAGTGT AGTTGGTGGA AAAAGATAAA   
  
  
+ TTGTAGGGTT ATTGTCTTGT AGAAATACAT ATACATAAAT ATTTATCTGT ACAAGATGTG CGTATGTACA   
  
  
+ ATAATCATTG ACAATTAACC TAAACATTGC AATTAACATA AACTTTGTTT CTTCTTAAAT TTTAGATGGT   
  
  
+ AAGTTCCTCC TATAAAATCA TTACAAAATC ATAAAATCCC AATAACACAC TTTATCTATA CGCAAAAAAA   
  
  
+ TTTGCCGGGG AAAATATAGT ACGTGGAGGT ATATATAGTA CGTACTTTCT CCTTTGCTTT CAAAGATCAT   
  
  
+ CAAATTTTAA TTAGTTTATA TTATTTTATA TATACACACA CACACACACA CACAAAGTGT GTGAGACATT   
  
  
+ TTTTTTATTT TTGTTTTTGA AATAGAAGGA GAGAGGAAGA GAGTGAATTA GATTTTTTTT TTTTTTTAAT   
  
  
+ TAAAGATATG TTAAGATTAC ATGTAGGTGA GATTTTGAAA AAAAAAACAG CCAAATTTAG TTATGTGAAA   
  
  
+ TTACATTTTT GCCCCGTATT TATTATTCAT ATTTTGTTTT AATTAGATGG TTAAAGTGGT AATTTGATAG   
  
  
+ GATTTTAGTT TACAATGAGT GTTTTATTAA TTAGTAGATA TATATTAAGT ATGTATTTTC AATTAAAAAT   
  
  
+ AAAGAAAAAA AAGCAGCATC TAGCATGGCA AGCACAACAC AAGCAGATAT ATATATATAT ATATATATAT   
  
  
+ ATAATTGGAA AAGTATTGTT GTCGTGGAGA CAAATTTAGG TTCTTGAGCA AAAACTCAAC TACTCTCTAG   
  
  
+ AGAGAGAGAG AGAGAGAGGT AGCAGTAACA CGACTTGTGT AGAAATTGTG AGGGACGAAT CGGTTAAGAA   
  
  
+ GTGCATGTCT TGTATCTCTC TCTAGAGCTA GAAAAATCCT AAGTGTTTCA TTTTTTTTGG AACAAACACA   
  
  
+ GAAAACCCCA AGAATTATAA TAATTAAAC  

- ATTTTTATCA CTTTCTTGGT GGCCGGCCTA TTTGTTCTCA TTTCGACAGA CTTTTATTTG GGATGAGTGT   
  
  
- GTGTACCATA AAGAGTATAA AATAAAATAT CAGTTAATAA TTCTATTATT CGTAAGATTG GTAAATACAT   
  
  
- ATGTACAAGT ATGGCGCTCG CAACGAGCCT CCTTGTGTAA AAACATAGTT CACTACAACG TTTTACTACG   
  
  
- TATGTTAGTC ATGTGTAGTG AGATCGATGA ATGAGGTAGA ACATCATAAC GTTACGTCAT TTTATATCGT   
  
  
- GATCAGGGTC TAATTAATAA TACCAAGAGG AAAGTTTGTG TATCAATTAC CAATATTACT ATATGAATTC   
  
  
- CCGTTTTATA TAATGAATAT GTTTTATTGA GCTACCCAAT GCATTTTATG TACTTCTTAA GTCAAATTAT   
  
  
- TCAAATCATA TACGAACGTA CTCATACTAT GTTATATATA CACTGTTTTA TTTTATGTTC AGATTTAAAT   
  
  
- GCTGTGGTGG TGGGGGAATA TATCTCAATT TTGCACTCTA AGTTCTCACA TCAACCACCT TTTTCTATTT   
  
  
- AACATCCCAA TAACAGAACA TCTTTATGTA TATGTATTTA TAAATAGACA TGTTCTACAC GCATACATGT   
  
  
- TATTAGTAAC TGTTAATTGG ATTTGTAACG TTAATTGTAT TTGAAACAAA GAAGAATTTA AAATCTACCA   
  
  
- TTCAAGGAGG ATATTTTAGT AATGTTTTAG TATTTTAGGG TTATTGTGTG AAATAGATAT GCGTTTTTTT   
  
  
- AAACGGCCCC TTTTATATCA TGCACCTCCA TATATATCAT GCATGAAAGA GGAAACGAAA GTTTCTAGTA   
  
  
- GTTTAAAATT AATCAAATAT AATAAAATAT ATATGTGTGT GTGTGTGTGT GTGTTTCACA CACTCTGTAA   
  
  
- AAAAAATAAA AACAAAAACT TTATCTTCCT CTCTCCTTCT CTCACTTAAT CTAAAAAAAA AAAAAAATTA   
  
  
- ATTTCTATAC AATTCTAATG TACATCCACT CTAAAACTTT TTTTTTTGTC GGTTTAAATC AATACACTTT   
  
  
- AATGTAAAAA CGGGGCATAA ATAATAAGTA TAAAACAAAA TTAATCTACC AATTTCACCA TTAAACTATC   
  
  
- CTAAAATCAA ATGTTACTCA CAAAATAATT AATCATCTAT ATATAATTCA TACATAAAAG TTAATTTTTA   
  
  
- TTTCTTTTTT TTCGTCGTAG ATCGTACCGT TCGTGTTGTG TTCGTCTATA TATATATATA TATATATATA   
  
  
- TATTAACCTT TTCATAACAA CAGCACCTCT GTTTAAATCC AAGAACTCGT TTTTGAGTTG ATGAGAGATC   
  
  
- TCTCTCTCTC TCTCTCTCCA TCGTCATTGT GCTGAACACA TCTTTAACAC TCCCTGCTTA GCCAATTCTT   
  
  
- CACGTACAGA ACATAGAGAG AGATCTCGAT CTTTTTAGGA TTCACAAAGT AAAAAAAACC TTGTTTGTGT   
  
  
- CTTTTGGGGT TCTTAATATT ATTAATTTG

+     MRE

| Site Name | Organism | Position | Strand | Matrix score. | sequence | function |
| --- | --- | --- | --- | --- | --- | --- |
| MRE | Petroselinum crispum | 647 | + | 7 | AACCTAA | MYB binding site involved in light responsiveness |
| MRE | Petroselinum crispum | 1296 | - | 7 | AACCTAA | MYB binding site involved in light responsiveness |

>003403   
+ TAAAAATAGT GAAAGAACCA CCGGCCGGAT AAACAAGAGT AAAGCTGTCT GAAAATAAAC CCTACTCACA   
  
  
+ CACATGGTAT TTCTCATATT TTATTTTATA GTCAATTATT AAGATAATAA GCATTCTAAC CATTTATGTA   
  
  
+ TACATGTTCA TACCGCGAGC GTTGCTCGGA GGAACACATT TTTGTATCAA GTGATGTTGC AAAATGATGC   
  
  
+ ATACAATCAG TACACATCAC TCTAGCTACT TACTCCATCT TGTAGTATTG CAATGCAGTA AAATATAGCA   
  
  
+ CTAGTCCCAG ATTAATTATT ATGGTTCTCC TTTCAAACAC ATAGTTAATG GTTATAATGA TATACTTAAG   
  
  
+ GGCAAAATAT ATTACTTATA CAAAATAACT CGATGGGTTA CGTAAAATAC ATGAAGAATT CAGTTTAATA   
  
  
+ AGTTTAGTAT ATGCTTGCAT GAGTATGATA CAATATATAT GTGACAAAAT AAAATACAAG TCTAAATTTA   
  
  
+ CGACACCACC ACCCCCTTAT ATAGAGTTAA AACGTGAGAT TCAAGAGTGT AGTTGGTGGA AAAAGATAAA   
  
  
+ TTGTAGGGTT ATTGTCTTGT AGAAATACAT ATACATAAAT ATTTATCTGT ACAAGATGTG CGTATGTACA   
  
  
+ ATAATCATTG ACAATTAACC TAAACATTGC AATTAACATA AACTTTGTTT CTTCTTAAAT TTTAGATGGT   
  
  
+ AAGTTCCTCC TATAAAATCA TTACAAAATC ATAAAATCCC AATAACACAC TTTATCTATA CGCAAAAAAA   
  
  
+ TTTGCCGGGG AAAATATAGT ACGTGGAGGT ATATATAGTA CGTACTTTCT CCTTTGCTTT CAAAGATCAT   
  
  
+ CAAATTTTAA TTAGTTTATA TTATTTTATA TATACACACA CACACACACA CACAAAGTGT GTGAGACATT   
  
  
+ TTTTTTATTT TTGTTTTTGA AATAGAAGGA GAGAGGAAGA GAGTGAATTA GATTTTTTTT TTTTTTTAAT   
  
  
+ TAAAGATATG TTAAGATTAC ATGTAGGTGA GATTTTGAAA AAAAAAACAG CCAAATTTAG TTATGTGAAA   
  
  
+ TTACATTTTT GCCCCGTATT TATTATTCAT ATTTTGTTTT AATTAGATGG TTAAAGTGGT AATTTGATAG   
  
  
+ GATTTTAGTT TACAATGAGT GTTTTATTAA TTAGTAGATA TATATTAAGT ATGTATTTTC AATTAAAAAT   
  
  
+ AAAGAAAAAA AAGCAGCATC TAGCATGGCA AGCACAACAC AAGCAGATAT ATATATATAT ATATATATAT   
  
  
+ ATAATTGGAA AAGTATTGTT GTCGTGGAGA CAAATTTAGG TTCTTGAGCA AAAACTCAAC TACTCTCTAG   
  
  
+ AGAGAGAGAG AGAGAGAGGT AGCAGTAACA CGACTTGTGT AGAAATTGTG AGGGACGAAT CGGTTAAGAA   
  
  
+ GTGCATGTCT TGTATCTCTC TCTAGAGCTA GAAAAATCCT AAGTGTTTCA TTTTTTTTGG AACAAACACA   
  
  
+ GAAAACCCCA AGAATTATAA TAATTAAAC  

- ATTTTTATCA CTTTCTTGGT GGCCGGCCTA TTTGTTCTCA TTTCGACAGA CTTTTATTTG GGATGAGTGT   
  
  
- GTGTACCATA AAGAGTATAA AATAAAATAT CAGTTAATAA TTCTATTATT CGTAAGATTG GTAAATACAT   
  
  
- ATGTACAAGT ATGGCGCTCG CAACGAGCCT CCTTGTGTAA AAACATAGTT CACTACAACG TTTTACTACG   
  
  
- TATGTTAGTC ATGTGTAGTG AGATCGATGA ATGAGGTAGA ACATCATAAC GTTACGTCAT TTTATATCGT   
  
  
- GATCAGGGTC TAATTAATAA TACCAAGAGG AAAGTTTGTG TATCAATTAC CAATATTACT ATATGAATTC   
  
  
- CCGTTTTATA TAATGAATAT GTTTTATTGA GCTACCCAAT GCATTTTATG TACTTCTTAA GTCAAATTAT   
  
  
- TCAAATCATA TACGAACGTA CTCATACTAT GTTATATATA CACTGTTTTA TTTTATGTTC AGATTTAAAT   
  
  
- GCTGTGGTGG TGGGGGAATA TATCTCAATT TTGCACTCTA AGTTCTCACA TCAACCACCT TTTTCTATTT   
  
  
- AACATCCCAA TAACAGAACA TCTTTATGTA TATGTATTTA TAAATAGACA TGTTCTACAC GCATACATGT   
  
  
- TATTAGTAAC TGTTAATTGG ATTTGTAACG TTAATTGTAT TTGAAACAAA GAAGAATTTA AAATCTACCA   
  
  
- TTCAAGGAGG ATATTTTAGT AATGTTTTAG TATTTTAGGG TTATTGTGTG AAATAGATAT GCGTTTTTTT   
  
  
- AAACGGCCCC TTTTATATCA TGCACCTCCA TATATATCAT GCATGAAAGA GGAAACGAAA GTTTCTAGTA   
  
  
- GTTTAAAATT AATCAAATAT AATAAAATAT ATATGTGTGT GTGTGTGTGT GTGTTTCACA CACTCTGTAA   
  
  
- AAAAAATAAA AACAAAAACT TTATCTTCCT CTCTCCTTCT CTCACTTAAT CTAAAAAAAA AAAAAAATTA   
  
  
- ATTTCTATAC AATTCTAATG TACATCCACT CTAAAACTTT TTTTTTTGTC GGTTTAAATC AATACACTTT   
  
  
- AATGTAAAAA CGGGGCATAA ATAATAAGTA TAAAACAAAA TTAATCTACC AATTTCACCA TTAAACTATC   
  
  
- CTAAAATCAA ATGTTACTCA CAAAATAATT AATCATCTAT ATATAATTCA TACATAAAAG TTAATTTTTA   
  
  
- TTTCTTTTTT TTCGTCGTAG ATCGTACCGT TCGTGTTGTG TTCGTCTATA TATATATATA TATATATATA   
  
  
- TATTAACCTT TTCATAACAA CAGCACCTCT GTTTAAATCC AAGAACTCGT TTTTGAGTTG ATGAGAGATC   
  
  
- TCTCTCTCTC TCTCTCTCCA TCGTCATTGT GCTGAACACA TCTTTAACAC TCCCTGCTTA GCCAATTCTT   
  
  
- CACGTACAGA ACATAGAGAG AGATCTCGAT CTTTTTAGGA TTCACAAAGT AAAAAAAACC TTGTTTGTGT   
  
  
- CTTTTGGGGT TCTTAATATT ATTAATTTG

+     MYB

| Site Name | Organism | Position | Strand | Matrix score. | sequence | function |
| --- | --- | --- | --- | --- | --- | --- |
| MYB | Arabidopsis thaliana | 127 | + | 6 | TAACCA |  |
| MYB | Arabidopsis thaliana | 1098 | - | 6 | TAACCA |  |
| MYB | Arabidopsis thaliana | 329 | - | 6 | TAACCA |  |

>003403   
+ TAAAAATAGT GAAAGAACCA CCGGCCGGAT AAACAAGAGT AAAGCTGTCT GAAAATAAAC CCTACTCACA   
  
  
+ CACATGGTAT TTCTCATATT TTATTTTATA GTCAATTATT AAGATAATAA GCATTCTAAC CATTTATGTA   
  
  
+ TACATGTTCA TACCGCGAGC GTTGCTCGGA GGAACACATT TTTGTATCAA GTGATGTTGC AAAATGATGC   
  
  
+ ATACAATCAG TACACATCAC TCTAGCTACT TACTCCATCT TGTAGTATTG CAATGCAGTA AAATATAGCA   
  
  
+ CTAGTCCCAG ATTAATTATT ATGGTTCTCC TTTCAAACAC ATAGTTAATG GTTATAATGA TATACTTAAG   
  
  
+ GGCAAAATAT ATTACTTATA CAAAATAACT CGATGGGTTA CGTAAAATAC ATGAAGAATT CAGTTTAATA   
  
  
+ AGTTTAGTAT ATGCTTGCAT GAGTATGATA CAATATATAT GTGACAAAAT AAAATACAAG TCTAAATTTA   
  
  
+ CGACACCACC ACCCCCTTAT ATAGAGTTAA AACGTGAGAT TCAAGAGTGT AGTTGGTGGA AAAAGATAAA   
  
  
+ TTGTAGGGTT ATTGTCTTGT AGAAATACAT ATACATAAAT ATTTATCTGT ACAAGATGTG CGTATGTACA   
  
  
+ ATAATCATTG ACAATTAACC TAAACATTGC AATTAACATA AACTTTGTTT CTTCTTAAAT TTTAGATGGT   
  
  
+ AAGTTCCTCC TATAAAATCA TTACAAAATC ATAAAATCCC AATAACACAC TTTATCTATA CGCAAAAAAA   
  
  
+ TTTGCCGGGG AAAATATAGT ACGTGGAGGT ATATATAGTA CGTACTTTCT CCTTTGCTTT CAAAGATCAT   
  
  
+ CAAATTTTAA TTAGTTTATA TTATTTTATA TATACACACA CACACACACA CACAAAGTGT GTGAGACATT   
  
  
+ TTTTTTATTT TTGTTTTTGA AATAGAAGGA GAGAGGAAGA GAGTGAATTA GATTTTTTTT TTTTTTTAAT   
  
  
+ TAAAGATATG TTAAGATTAC ATGTAGGTGA GATTTTGAAA AAAAAAACAG CCAAATTTAG TTATGTGAAA   
  
  
+ TTACATTTTT GCCCCGTATT TATTATTCAT ATTTTGTTTT AATTAGATGG TTAAAGTGGT AATTTGATAG   
  
  
+ GATTTTAGTT TACAATGAGT GTTTTATTAA TTAGTAGATA TATATTAAGT ATGTATTTTC AATTAAAAAT   
  
  
+ AAAGAAAAAA AAGCAGCATC TAGCATGGCA AGCACAACAC AAGCAGATAT ATATATATAT ATATATATAT   
  
  
+ ATAATTGGAA AAGTATTGTT GTCGTGGAGA CAAATTTAGG TTCTTGAGCA AAAACTCAAC TACTCTCTAG   
  
  
+ AGAGAGAGAG AGAGAGAGGT AGCAGTAACA CGACTTGTGT AGAAATTGTG AGGGACGAAT CGGTTAAGAA   
  
  
+ GTGCATGTCT TGTATCTCTC TCTAGAGCTA GAAAAATCCT AAGTGTTTCA TTTTTTTTGG AACAAACACA   
  
  
+ GAAAACCCCA AGAATTATAA TAATTAAAC  

- ATTTTTATCA CTTTCTTGGT GGCCGGCCTA TTTGTTCTCA TTTCGACAGA CTTTTATTTG GGATGAGTGT   
  
  
- GTGTACCATA AAGAGTATAA AATAAAATAT CAGTTAATAA TTCTATTATT CGTAAGATTG GTAAATACAT   
  
  
- ATGTACAAGT ATGGCGCTCG CAACGAGCCT CCTTGTGTAA AAACATAGTT CACTACAACG TTTTACTACG   
  
  
- TATGTTAGTC ATGTGTAGTG AGATCGATGA ATGAGGTAGA ACATCATAAC GTTACGTCAT TTTATATCGT   
  
  
- GATCAGGGTC TAATTAATAA TACCAAGAGG AAAGTTTGTG TATCAATTAC CAATATTACT ATATGAATTC   
  
  
- CCGTTTTATA TAATGAATAT GTTTTATTGA GCTACCCAAT GCATTTTATG TACTTCTTAA GTCAAATTAT   
  
  
- TCAAATCATA TACGAACGTA CTCATACTAT GTTATATATA CACTGTTTTA TTTTATGTTC AGATTTAAAT   
  
  
- GCTGTGGTGG TGGGGGAATA TATCTCAATT TTGCACTCTA AGTTCTCACA TCAACCACCT TTTTCTATTT   
  
  
- AACATCCCAA TAACAGAACA TCTTTATGTA TATGTATTTA TAAATAGACA TGTTCTACAC GCATACATGT   
  
  
- TATTAGTAAC TGTTAATTGG ATTTGTAACG TTAATTGTAT TTGAAACAAA GAAGAATTTA AAATCTACCA   
  
  
- TTCAAGGAGG ATATTTTAGT AATGTTTTAG TATTTTAGGG TTATTGTGTG AAATAGATAT GCGTTTTTTT   
  
  
- AAACGGCCCC TTTTATATCA TGCACCTCCA TATATATCAT GCATGAAAGA GGAAACGAAA GTTTCTAGTA   
  
  
- GTTTAAAATT AATCAAATAT AATAAAATAT ATATGTGTGT GTGTGTGTGT GTGTTTCACA CACTCTGTAA   
  
  
- AAAAAATAAA AACAAAAACT TTATCTTCCT CTCTCCTTCT CTCACTTAAT CTAAAAAAAA AAAAAAATTA   
  
  
- ATTTCTATAC AATTCTAATG TACATCCACT CTAAAACTTT TTTTTTTGTC GGTTTAAATC AATACACTTT   
  
  
- AATGTAAAAA CGGGGCATAA ATAATAAGTA TAAAACAAAA TTAATCTACC AATTTCACCA TTAAACTATC   
  
  
- CTAAAATCAA ATGTTACTCA CAAAATAATT AATCATCTAT ATATAATTCA TACATAAAAG TTAATTTTTA   
  
  
- TTTCTTTTTT TTCGTCGTAG ATCGTACCGT TCGTGTTGTG TTCGTCTATA TATATATATA TATATATATA   
  
  
- TATTAACCTT TTCATAACAA CAGCACCTCT GTTTAAATCC AAGAACTCGT TTTTGAGTTG ATGAGAGATC   
  
  
- TCTCTCTCTC TCTCTCTCCA TCGTCATTGT GCTGAACACA TCTTTAACAC TCCCTGCTTA GCCAATTCTT   
  
  
- CACGTACAGA ACATAGAGAG AGATCTCGAT CTTTTTAGGA TTCACAAAGT AAAAAAAACC TTGTTTGTGT   
  
  
- CTTTTGGGGT TCTTAATATT ATTAATTTG

+     MYB-like sequence

| Site Name | Organism | Position | Strand | Matrix score. | sequence | function |
| --- | --- | --- | --- | --- | --- | --- |
| MYB-like sequence | Arabidopsis thaliana | 127 | + | 6 | TAACCA |  |
| MYB-like sequence | Arabidopsis thaliana | 1098 | - | 6 | TAACCA |  |
| MYB-like sequence | Arabidopsis thaliana | 329 | - | 6 | TAACCA |  |

>003403   
+ TAAAAATAGT GAAAGAACCA CCGGCCGGAT AAACAAGAGT AAAGCTGTCT GAAAATAAAC CCTACTCACA   
  
  
+ CACATGGTAT TTCTCATATT TTATTTTATA GTCAATTATT AAGATAATAA GCATTCTAAC CATTTATGTA   
  
  
+ TACATGTTCA TACCGCGAGC GTTGCTCGGA GGAACACATT TTTGTATCAA GTGATGTTGC AAAATGATGC   
  
  
+ ATACAATCAG TACACATCAC TCTAGCTACT TACTCCATCT TGTAGTATTG CAATGCAGTA AAATATAGCA   
  
  
+ CTAGTCCCAG ATTAATTATT ATGGTTCTCC TTTCAAACAC ATAGTTAATG GTTATAATGA TATACTTAAG   
  
  
+ GGCAAAATAT ATTACTTATA CAAAATAACT CGATGGGTTA CGTAAAATAC ATGAAGAATT CAGTTTAATA   
  
  
+ AGTTTAGTAT ATGCTTGCAT GAGTATGATA CAATATATAT GTGACAAAAT AAAATACAAG TCTAAATTTA   
  
  
+ CGACACCACC ACCCCCTTAT ATAGAGTTAA AACGTGAGAT TCAAGAGTGT AGTTGGTGGA AAAAGATAAA   
  
  
+ TTGTAGGGTT ATTGTCTTGT AGAAATACAT ATACATAAAT ATTTATCTGT ACAAGATGTG CGTATGTACA   
  
  
+ ATAATCATTG ACAATTAACC TAAACATTGC AATTAACATA AACTTTGTTT CTTCTTAAAT TTTAGATGGT   
  
  
+ AAGTTCCTCC TATAAAATCA TTACAAAATC ATAAAATCCC AATAACACAC TTTATCTATA CGCAAAAAAA   
  
  
+ TTTGCCGGGG AAAATATAGT ACGTGGAGGT ATATATAGTA CGTACTTTCT CCTTTGCTTT CAAAGATCAT   
  
  
+ CAAATTTTAA TTAGTTTATA TTATTTTATA TATACACACA CACACACACA CACAAAGTGT GTGAGACATT   
  
  
+ TTTTTTATTT TTGTTTTTGA AATAGAAGGA GAGAGGAAGA GAGTGAATTA GATTTTTTTT TTTTTTTAAT   
  
  
+ TAAAGATATG TTAAGATTAC ATGTAGGTGA GATTTTGAAA AAAAAAACAG CCAAATTTAG TTATGTGAAA   
  
  
+ TTACATTTTT GCCCCGTATT TATTATTCAT ATTTTGTTTT AATTAGATGG TTAAAGTGGT AATTTGATAG   
  
  
+ GATTTTAGTT TACAATGAGT GTTTTATTAA TTAGTAGATA TATATTAAGT ATGTATTTTC AATTAAAAAT   
  
  
+ AAAGAAAAAA AAGCAGCATC TAGCATGGCA AGCACAACAC AAGCAGATAT ATATATATAT ATATATATAT   
  
  
+ ATAATTGGAA AAGTATTGTT GTCGTGGAGA CAAATTTAGG TTCTTGAGCA AAAACTCAAC TACTCTCTAG   
  
  
+ AGAGAGAGAG AGAGAGAGGT AGCAGTAACA CGACTTGTGT AGAAATTGTG AGGGACGAAT CGGTTAAGAA   
  
  
+ GTGCATGTCT TGTATCTCTC TCTAGAGCTA GAAAAATCCT AAGTGTTTCA TTTTTTTTGG AACAAACACA   
  
  
+ GAAAACCCCA AGAATTATAA TAATTAAAC  

- ATTTTTATCA CTTTCTTGGT GGCCGGCCTA TTTGTTCTCA TTTCGACAGA CTTTTATTTG GGATGAGTGT   
  
  
- GTGTACCATA AAGAGTATAA AATAAAATAT CAGTTAATAA TTCTATTATT CGTAAGATTG GTAAATACAT   
  
  
- ATGTACAAGT ATGGCGCTCG CAACGAGCCT CCTTGTGTAA AAACATAGTT CACTACAACG TTTTACTACG   
  
  
- TATGTTAGTC ATGTGTAGTG AGATCGATGA ATGAGGTAGA ACATCATAAC GTTACGTCAT TTTATATCGT   
  
  
- GATCAGGGTC TAATTAATAA TACCAAGAGG AAAGTTTGTG TATCAATTAC CAATATTACT ATATGAATTC   
  
  
- CCGTTTTATA TAATGAATAT GTTTTATTGA GCTACCCAAT GCATTTTATG TACTTCTTAA GTCAAATTAT   
  
  
- TCAAATCATA TACGAACGTA CTCATACTAT GTTATATATA CACTGTTTTA TTTTATGTTC AGATTTAAAT   
  
  
- GCTGTGGTGG TGGGGGAATA TATCTCAATT TTGCACTCTA AGTTCTCACA TCAACCACCT TTTTCTATTT   
  
  
- AACATCCCAA TAACAGAACA TCTTTATGTA TATGTATTTA TAAATAGACA TGTTCTACAC GCATACATGT   
  
  
- TATTAGTAAC TGTTAATTGG ATTTGTAACG TTAATTGTAT TTGAAACAAA GAAGAATTTA AAATCTACCA   
  
  
- TTCAAGGAGG ATATTTTAGT AATGTTTTAG TATTTTAGGG TTATTGTGTG AAATAGATAT GCGTTTTTTT   
  
  
- AAACGGCCCC TTTTATATCA TGCACCTCCA TATATATCAT GCATGAAAGA GGAAACGAAA GTTTCTAGTA   
  
  
- GTTTAAAATT AATCAAATAT AATAAAATAT ATATGTGTGT GTGTGTGTGT GTGTTTCACA CACTCTGTAA   
  
  
- AAAAAATAAA AACAAAAACT TTATCTTCCT CTCTCCTTCT CTCACTTAAT CTAAAAAAAA AAAAAAATTA   
  
  
- ATTTCTATAC AATTCTAATG TACATCCACT CTAAAACTTT TTTTTTTGTC GGTTTAAATC AATACACTTT   
  
  
- AATGTAAAAA CGGGGCATAA ATAATAAGTA TAAAACAAAA TTAATCTACC AATTTCACCA TTAAACTATC   
  
  
- CTAAAATCAA ATGTTACTCA CAAAATAATT AATCATCTAT ATATAATTCA TACATAAAAG TTAATTTTTA   
  
  
- TTTCTTTTTT TTCGTCGTAG ATCGTACCGT TCGTGTTGTG TTCGTCTATA TATATATATA TATATATATA   
  
  
- TATTAACCTT TTCATAACAA CAGCACCTCT GTTTAAATCC AAGAACTCGT TTTTGAGTTG ATGAGAGATC   
  
  
- TCTCTCTCTC TCTCTCTCCA TCGTCATTGT GCTGAACACA TCTTTAACAC TCCCTGCTTA GCCAATTCTT   
  
  
- CACGTACAGA ACATAGAGAG AGATCTCGAT CTTTTTAGGA TTCACAAAGT AAAAAAAACC TTGTTTGTGT   
  
  
- CTTTTGGGGT TCTTAATATT ATTAATTTG

+     MYC

| Site Name | Organism | Position | Strand | Matrix score. | sequence | function |
| --- | --- | --- | --- | --- | --- | --- |
| MYC | Arabidopsis thaliana | 71 | - | 6 | CATGTG |  |

>003403   
+ TAAAAATAGT GAAAGAACCA CCGGCCGGAT AAACAAGAGT AAAGCTGTCT GAAAATAAAC CCTACTCACA   
  
  
+ CACATGGTAT TTCTCATATT TTATTTTATA GTCAATTATT AAGATAATAA GCATTCTAAC CATTTATGTA   
  
  
+ TACATGTTCA TACCGCGAGC GTTGCTCGGA GGAACACATT TTTGTATCAA GTGATGTTGC AAAATGATGC   
  
  
+ ATACAATCAG TACACATCAC TCTAGCTACT TACTCCATCT TGTAGTATTG CAATGCAGTA AAATATAGCA   
  
  
+ CTAGTCCCAG ATTAATTATT ATGGTTCTCC TTTCAAACAC ATAGTTAATG GTTATAATGA TATACTTAAG   
  
  
+ GGCAAAATAT ATTACTTATA CAAAATAACT CGATGGGTTA CGTAAAATAC ATGAAGAATT CAGTTTAATA   
  
  
+ AGTTTAGTAT ATGCTTGCAT GAGTATGATA CAATATATAT GTGACAAAAT AAAATACAAG TCTAAATTTA   
  
  
+ CGACACCACC ACCCCCTTAT ATAGAGTTAA AACGTGAGAT TCAAGAGTGT AGTTGGTGGA AAAAGATAAA   
  
  
+ TTGTAGGGTT ATTGTCTTGT AGAAATACAT ATACATAAAT ATTTATCTGT ACAAGATGTG CGTATGTACA   
  
  
+ ATAATCATTG ACAATTAACC TAAACATTGC AATTAACATA AACTTTGTTT CTTCTTAAAT TTTAGATGGT   
  
  
+ AAGTTCCTCC TATAAAATCA TTACAAAATC ATAAAATCCC AATAACACAC TTTATCTATA CGCAAAAAAA   
  
  
+ TTTGCCGGGG AAAATATAGT ACGTGGAGGT ATATATAGTA CGTACTTTCT CCTTTGCTTT CAAAGATCAT   
  
  
+ CAAATTTTAA TTAGTTTATA TTATTTTATA TATACACACA CACACACACA CACAAAGTGT GTGAGACATT   
  
  
+ TTTTTTATTT TTGTTTTTGA AATAGAAGGA GAGAGGAAGA GAGTGAATTA GATTTTTTTT TTTTTTTAAT   
  
  
+ TAAAGATATG TTAAGATTAC ATGTAGGTGA GATTTTGAAA AAAAAAACAG CCAAATTTAG TTATGTGAAA   
  
  
+ TTACATTTTT GCCCCGTATT TATTATTCAT ATTTTGTTTT AATTAGATGG TTAAAGTGGT AATTTGATAG   
  
  
+ GATTTTAGTT TACAATGAGT GTTTTATTAA TTAGTAGATA TATATTAAGT ATGTATTTTC AATTAAAAAT   
  
  
+ AAAGAAAAAA AAGCAGCATC TAGCATGGCA AGCACAACAC AAGCAGATAT ATATATATAT ATATATATAT   
  
  
+ ATAATTGGAA AAGTATTGTT GTCGTGGAGA CAAATTTAGG TTCTTGAGCA AAAACTCAAC TACTCTCTAG   
  
  
+ AGAGAGAGAG AGAGAGAGGT AGCAGTAACA CGACTTGTGT AGAAATTGTG AGGGACGAAT CGGTTAAGAA   
  
  
+ GTGCATGTCT TGTATCTCTC TCTAGAGCTA GAAAAATCCT AAGTGTTTCA TTTTTTTTGG AACAAACACA   
  
  
+ GAAAACCCCA AGAATTATAA TAATTAAAC  

- ATTTTTATCA CTTTCTTGGT GGCCGGCCTA TTTGTTCTCA TTTCGACAGA CTTTTATTTG GGATGAGTGT   
  
  
- GTGTACCATA AAGAGTATAA AATAAAATAT CAGTTAATAA TTCTATTATT CGTAAGATTG GTAAATACAT   
  
  
- ATGTACAAGT ATGGCGCTCG CAACGAGCCT CCTTGTGTAA AAACATAGTT CACTACAACG TTTTACTACG   
  
  
- TATGTTAGTC ATGTGTAGTG AGATCGATGA ATGAGGTAGA ACATCATAAC GTTACGTCAT TTTATATCGT   
  
  
- GATCAGGGTC TAATTAATAA TACCAAGAGG AAAGTTTGTG TATCAATTAC CAATATTACT ATATGAATTC   
  
  
- CCGTTTTATA TAATGAATAT GTTTTATTGA GCTACCCAAT GCATTTTATG TACTTCTTAA GTCAAATTAT   
  
  
- TCAAATCATA TACGAACGTA CTCATACTAT GTTATATATA CACTGTTTTA TTTTATGTTC AGATTTAAAT   
  
  
- GCTGTGGTGG TGGGGGAATA TATCTCAATT TTGCACTCTA AGTTCTCACA TCAACCACCT TTTTCTATTT   
  
  
- AACATCCCAA TAACAGAACA TCTTTATGTA TATGTATTTA TAAATAGACA TGTTCTACAC GCATACATGT   
  
  
- TATTAGTAAC TGTTAATTGG ATTTGTAACG TTAATTGTAT TTGAAACAAA GAAGAATTTA AAATCTACCA   
  
  
- TTCAAGGAGG ATATTTTAGT AATGTTTTAG TATTTTAGGG TTATTGTGTG AAATAGATAT GCGTTTTTTT   
  
  
- AAACGGCCCC TTTTATATCA TGCACCTCCA TATATATCAT GCATGAAAGA GGAAACGAAA GTTTCTAGTA   
  
  
- GTTTAAAATT AATCAAATAT AATAAAATAT ATATGTGTGT GTGTGTGTGT GTGTTTCACA CACTCTGTAA   
  
  
- AAAAAATAAA AACAAAAACT TTATCTTCCT CTCTCCTTCT CTCACTTAAT CTAAAAAAAA AAAAAAATTA   
  
  
- ATTTCTATAC AATTCTAATG TACATCCACT CTAAAACTTT TTTTTTTGTC GGTTTAAATC AATACACTTT   
  
  
- AATGTAAAAA CGGGGCATAA ATAATAAGTA TAAAACAAAA TTAATCTACC AATTTCACCA TTAAACTATC   
  
  
- CTAAAATCAA ATGTTACTCA CAAAATAATT AATCATCTAT ATATAATTCA TACATAAAAG TTAATTTTTA   
  
  
- TTTCTTTTTT TTCGTCGTAG ATCGTACCGT TCGTGTTGTG TTCGTCTATA TATATATATA TATATATATA   
  
  
- TATTAACCTT TTCATAACAA CAGCACCTCT GTTTAAATCC AAGAACTCGT TTTTGAGTTG ATGAGAGATC   
  
  
- TCTCTCTCTC TCTCTCTCCA TCGTCATTGT GCTGAACACA TCTTTAACAC TCCCTGCTTA GCCAATTCTT   
  
  
- CACGTACAGA ACATAGAGAG AGATCTCGAT CTTTTTAGGA TTCACAAAGT AAAAAAAACC TTGTTTGTGT   
  
  
- CTTTTGGGGT TCTTAATATT ATTAATTTG

+     STRE

| Site Name | Organism | Position | Strand | Matrix score. | sequence | function |
| --- | --- | --- | --- | --- | --- | --- |
| STRE | Arabidopsis thaliana | 503 | - | 5 | AGGGG |  |

>003403   
+ TAAAAATAGT GAAAGAACCA CCGGCCGGAT AAACAAGAGT AAAGCTGTCT GAAAATAAAC CCTACTCACA   
  
  
+ CACATGGTAT TTCTCATATT TTATTTTATA GTCAATTATT AAGATAATAA GCATTCTAAC CATTTATGTA   
  
  
+ TACATGTTCA TACCGCGAGC GTTGCTCGGA GGAACACATT TTTGTATCAA GTGATGTTGC AAAATGATGC   
  
  
+ ATACAATCAG TACACATCAC TCTAGCTACT TACTCCATCT TGTAGTATTG CAATGCAGTA AAATATAGCA   
  
  
+ CTAGTCCCAG ATTAATTATT ATGGTTCTCC TTTCAAACAC ATAGTTAATG GTTATAATGA TATACTTAAG   
  
  
+ GGCAAAATAT ATTACTTATA CAAAATAACT CGATGGGTTA CGTAAAATAC ATGAAGAATT CAGTTTAATA   
  
  
+ AGTTTAGTAT ATGCTTGCAT GAGTATGATA CAATATATAT GTGACAAAAT AAAATACAAG TCTAAATTTA   
  
  
+ CGACACCACC ACCCCCTTAT ATAGAGTTAA AACGTGAGAT TCAAGAGTGT AGTTGGTGGA AAAAGATAAA   
  
  
+ TTGTAGGGTT ATTGTCTTGT AGAAATACAT ATACATAAAT ATTTATCTGT ACAAGATGTG CGTATGTACA   
  
  
+ ATAATCATTG ACAATTAACC TAAACATTGC AATTAACATA AACTTTGTTT CTTCTTAAAT TTTAGATGGT   
  
  
+ AAGTTCCTCC TATAAAATCA TTACAAAATC ATAAAATCCC AATAACACAC TTTATCTATA CGCAAAAAAA   
  
  
+ TTTGCCGGGG AAAATATAGT ACGTGGAGGT ATATATAGTA CGTACTTTCT CCTTTGCTTT CAAAGATCAT   
  
  
+ CAAATTTTAA TTAGTTTATA TTATTTTATA TATACACACA CACACACACA CACAAAGTGT GTGAGACATT   
  
  
+ TTTTTTATTT TTGTTTTTGA AATAGAAGGA GAGAGGAAGA GAGTGAATTA GATTTTTTTT TTTTTTTAAT   
  
  
+ TAAAGATATG TTAAGATTAC ATGTAGGTGA GATTTTGAAA AAAAAAACAG CCAAATTTAG TTATGTGAAA   
  
  
+ TTACATTTTT GCCCCGTATT TATTATTCAT ATTTTGTTTT AATTAGATGG TTAAAGTGGT AATTTGATAG   
  
  
+ GATTTTAGTT TACAATGAGT GTTTTATTAA TTAGTAGATA TATATTAAGT ATGTATTTTC AATTAAAAAT   
  
  
+ AAAGAAAAAA AAGCAGCATC TAGCATGGCA AGCACAACAC AAGCAGATAT ATATATATAT ATATATATAT   
  
  
+ ATAATTGGAA AAGTATTGTT GTCGTGGAGA CAAATTTAGG TTCTTGAGCA AAAACTCAAC TACTCTCTAG   
  
  
+ AGAGAGAGAG AGAGAGAGGT AGCAGTAACA CGACTTGTGT AGAAATTGTG AGGGACGAAT CGGTTAAGAA   
  
  
+ GTGCATGTCT TGTATCTCTC TCTAGAGCTA GAAAAATCCT AAGTGTTTCA TTTTTTTTGG AACAAACACA   
  
  
+ GAAAACCCCA AGAATTATAA TAATTAAAC  

- ATTTTTATCA CTTTCTTGGT GGCCGGCCTA TTTGTTCTCA TTTCGACAGA CTTTTATTTG GGATGAGTGT   
  
  
- GTGTACCATA AAGAGTATAA AATAAAATAT CAGTTAATAA TTCTATTATT CGTAAGATTG GTAAATACAT   
  
  
- ATGTACAAGT ATGGCGCTCG CAACGAGCCT CCTTGTGTAA AAACATAGTT CACTACAACG TTTTACTACG   
  
  
- TATGTTAGTC ATGTGTAGTG AGATCGATGA ATGAGGTAGA ACATCATAAC GTTACGTCAT TTTATATCGT   
  
  
- GATCAGGGTC TAATTAATAA TACCAAGAGG AAAGTTTGTG TATCAATTAC CAATATTACT ATATGAATTC   
  
  
- CCGTTTTATA TAATGAATAT GTTTTATTGA GCTACCCAAT GCATTTTATG TACTTCTTAA GTCAAATTAT   
  
  
- TCAAATCATA TACGAACGTA CTCATACTAT GTTATATATA CACTGTTTTA TTTTATGTTC AGATTTAAAT   
  
  
- GCTGTGGTGG TGGGGGAATA TATCTCAATT TTGCACTCTA AGTTCTCACA TCAACCACCT TTTTCTATTT   
  
  
- AACATCCCAA TAACAGAACA TCTTTATGTA TATGTATTTA TAAATAGACA TGTTCTACAC GCATACATGT   
  
  
- TATTAGTAAC TGTTAATTGG ATTTGTAACG TTAATTGTAT TTGAAACAAA GAAGAATTTA AAATCTACCA   
  
  
- TTCAAGGAGG ATATTTTAGT AATGTTTTAG TATTTTAGGG TTATTGTGTG AAATAGATAT GCGTTTTTTT   
  
  
- AAACGGCCCC TTTTATATCA TGCACCTCCA TATATATCAT GCATGAAAGA GGAAACGAAA GTTTCTAGTA   
  
  
- GTTTAAAATT AATCAAATAT AATAAAATAT ATATGTGTGT GTGTGTGTGT GTGTTTCACA CACTCTGTAA   
  
  
- AAAAAATAAA AACAAAAACT TTATCTTCCT CTCTCCTTCT CTCACTTAAT CTAAAAAAAA AAAAAAATTA   
  
  
- ATTTCTATAC AATTCTAATG TACATCCACT CTAAAACTTT TTTTTTTGTC GGTTTAAATC AATACACTTT   
  
  
- AATGTAAAAA CGGGGCATAA ATAATAAGTA TAAAACAAAA TTAATCTACC AATTTCACCA TTAAACTATC   
  
  
- CTAAAATCAA ATGTTACTCA CAAAATAATT AATCATCTAT ATATAATTCA TACATAAAAG TTAATTTTTA   
  
  
- TTTCTTTTTT TTCGTCGTAG ATCGTACCGT TCGTGTTGTG TTCGTCTATA TATATATATA TATATATATA   
  
  
- TATTAACCTT TTCATAACAA CAGCACCTCT GTTTAAATCC AAGAACTCGT TTTTGAGTTG ATGAGAGATC   
  
  
- TCTCTCTCTC TCTCTCTCCA TCGTCATTGT GCTGAACACA TCTTTAACAC TCCCTGCTTA GCCAATTCTT   
  
  
- CACGTACAGA ACATAGAGAG AGATCTCGAT CTTTTTAGGA TTCACAAAGT AAAAAAAACC TTGTTTGTGT   
  
  
- CTTTTGGGGT TCTTAATATT ATTAATTTG

+     TATA

| Site Name | Organism | Position | Strand | Matrix score. | sequence | function |
| --- | --- | --- | --- | --- | --- | --- |
| TATA | Arabidopsis thaliana | 93 | - | 8 | TATAAAAT |  |
| TATA | Arabidopsis thaliana | 863 | - | 8 | TATAAAAT |  |
| TATA | Arabidopsis thaliana | 711 | + | 8 | TATAAAAT |  |

>003403   
+ TAAAAATAGT GAAAGAACCA CCGGCCGGAT AAACAAGAGT AAAGCTGTCT GAAAATAAAC CCTACTCACA   
  
  
+ CACATGGTAT TTCTCATATT TTATTTTATA GTCAATTATT AAGATAATAA GCATTCTAAC CATTTATGTA   
  
  
+ TACATGTTCA TACCGCGAGC GTTGCTCGGA GGAACACATT TTTGTATCAA GTGATGTTGC AAAATGATGC   
  
  
+ ATACAATCAG TACACATCAC TCTAGCTACT TACTCCATCT TGTAGTATTG CAATGCAGTA AAATATAGCA   
  
  
+ CTAGTCCCAG ATTAATTATT ATGGTTCTCC TTTCAAACAC ATAGTTAATG GTTATAATGA TATACTTAAG   
  
  
+ GGCAAAATAT ATTACTTATA CAAAATAACT CGATGGGTTA CGTAAAATAC ATGAAGAATT CAGTTTAATA   
  
  
+ AGTTTAGTAT ATGCTTGCAT GAGTATGATA CAATATATAT GTGACAAAAT AAAATACAAG TCTAAATTTA   
  
  
+ CGACACCACC ACCCCCTTAT ATAGAGTTAA AACGTGAGAT TCAAGAGTGT AGTTGGTGGA AAAAGATAAA   
  
  
+ TTGTAGGGTT ATTGTCTTGT AGAAATACAT ATACATAAAT ATTTATCTGT ACAAGATGTG CGTATGTACA   
  
  
+ ATAATCATTG ACAATTAACC TAAACATTGC AATTAACATA AACTTTGTTT CTTCTTAAAT TTTAGATGGT   
  
  
+ AAGTTCCTCC TATAAAATCA TTACAAAATC ATAAAATCCC AATAACACAC TTTATCTATA CGCAAAAAAA   
  
  
+ TTTGCCGGGG AAAATATAGT ACGTGGAGGT ATATATAGTA CGTACTTTCT CCTTTGCTTT CAAAGATCAT   
  
  
+ CAAATTTTAA TTAGTTTATA TTATTTTATA TATACACACA CACACACACA CACAAAGTGT GTGAGACATT   
  
  
+ TTTTTTATTT TTGTTTTTGA AATAGAAGGA GAGAGGAAGA GAGTGAATTA GATTTTTTTT TTTTTTTAAT   
  
  
+ TAAAGATATG TTAAGATTAC ATGTAGGTGA GATTTTGAAA AAAAAAACAG CCAAATTTAG TTATGTGAAA   
  
  
+ TTACATTTTT GCCCCGTATT TATTATTCAT ATTTTGTTTT AATTAGATGG TTAAAGTGGT AATTTGATAG   
  
  
+ GATTTTAGTT TACAATGAGT GTTTTATTAA TTAGTAGATA TATATTAAGT ATGTATTTTC AATTAAAAAT   
  
  
+ AAAGAAAAAA AAGCAGCATC TAGCATGGCA AGCACAACAC AAGCAGATAT ATATATATAT ATATATATAT   
  
  
+ ATAATTGGAA AAGTATTGTT GTCGTGGAGA CAAATTTAGG TTCTTGAGCA AAAACTCAAC TACTCTCTAG   
  
  
+ AGAGAGAGAG AGAGAGAGGT AGCAGTAACA CGACTTGTGT AGAAATTGTG AGGGACGAAT CGGTTAAGAA   
  
  
+ GTGCATGTCT TGTATCTCTC TCTAGAGCTA GAAAAATCCT AAGTGTTTCA TTTTTTTTGG AACAAACACA   
  
  
+ GAAAACCCCA AGAATTATAA TAATTAAAC  

- ATTTTTATCA CTTTCTTGGT GGCCGGCCTA TTTGTTCTCA TTTCGACAGA CTTTTATTTG GGATGAGTGT   
  
  
- GTGTACCATA AAGAGTATAA AATAAAATAT CAGTTAATAA TTCTATTATT CGTAAGATTG GTAAATACAT   
  
  
- ATGTACAAGT ATGGCGCTCG CAACGAGCCT CCTTGTGTAA AAACATAGTT CACTACAACG TTTTACTACG   
  
  
- TATGTTAGTC ATGTGTAGTG AGATCGATGA ATGAGGTAGA ACATCATAAC GTTACGTCAT TTTATATCGT   
  
  
- GATCAGGGTC TAATTAATAA TACCAAGAGG AAAGTTTGTG TATCAATTAC CAATATTACT ATATGAATTC   
  
  
- CCGTTTTATA TAATGAATAT GTTTTATTGA GCTACCCAAT GCATTTTATG TACTTCTTAA GTCAAATTAT   
  
  
- TCAAATCATA TACGAACGTA CTCATACTAT GTTATATATA CACTGTTTTA TTTTATGTTC AGATTTAAAT   
  
  
- GCTGTGGTGG TGGGGGAATA TATCTCAATT TTGCACTCTA AGTTCTCACA TCAACCACCT TTTTCTATTT   
  
  
- AACATCCCAA TAACAGAACA TCTTTATGTA TATGTATTTA TAAATAGACA TGTTCTACAC GCATACATGT   
  
  
- TATTAGTAAC TGTTAATTGG ATTTGTAACG TTAATTGTAT TTGAAACAAA GAAGAATTTA AAATCTACCA   
  
  
- TTCAAGGAGG ATATTTTAGT AATGTTTTAG TATTTTAGGG TTATTGTGTG AAATAGATAT GCGTTTTTTT   
  
  
- AAACGGCCCC TTTTATATCA TGCACCTCCA TATATATCAT GCATGAAAGA GGAAACGAAA GTTTCTAGTA   
  
  
- GTTTAAAATT AATCAAATAT AATAAAATAT ATATGTGTGT GTGTGTGTGT GTGTTTCACA CACTCTGTAA   
  
  
- AAAAAATAAA AACAAAAACT TTATCTTCCT CTCTCCTTCT CTCACTTAAT CTAAAAAAAA AAAAAAATTA   
  
  
- ATTTCTATAC AATTCTAATG TACATCCACT CTAAAACTTT TTTTTTTGTC GGTTTAAATC AATACACTTT   
  
  
- AATGTAAAAA CGGGGCATAA ATAATAAGTA TAAAACAAAA TTAATCTACC AATTTCACCA TTAAACTATC   
  
  
- CTAAAATCAA ATGTTACTCA CAAAATAATT AATCATCTAT ATATAATTCA TACATAAAAG TTAATTTTTA   
  
  
- TTTCTTTTTT TTCGTCGTAG ATCGTACCGT TCGTGTTGTG TTCGTCTATA TATATATATA TATATATATA   
  
  
- TATTAACCTT TTCATAACAA CAGCACCTCT GTTTAAATCC AAGAACTCGT TTTTGAGTTG ATGAGAGATC   
  
  
- TCTCTCTCTC TCTCTCTCCA TCGTCATTGT GCTGAACACA TCTTTAACAC TCCCTGCTTA GCCAATTCTT   
  
  
- CACGTACAGA ACATAGAGAG AGATCTCGAT CTTTTTAGGA TTCACAAAGT AAAAAAAACC TTGTTTGTGT   
  
  
- CTTTTGGGGT TCTTAATATT ATTAATTTG

+     TATA-box

| Site Name | Organism | Position | Strand | Matrix score. | sequence | function |
| --- | --- | --- | --- | --- | --- | --- |
| TATA-box | Pisum sativum | 94 | - | 7 | TATAAAA | core promoter element around -30 of transcription start |
| TATA-box | Arabidopsis thaliana | 590 | + | 4 | TATA | core promoter element around -30 of transcription start |
| TATA-box | Helianthus annuus | 95 | - | 6 | TATAAA | core promoter element around -30 of transcription start |
| TATA-box | Arabidopsis thaliana | 510 | + | 4 | TATA | core promoter element around -30 of transcription start |
| TATA-box | Oryza sativa | 592 | + | 8 | TACATAAA | core promoter element around -30 of transcription start |
| TATA-box | Arabidopsis thaliana | 96 | - | 5 | TATAA | core promoter element around -30 of transcription start |
| TATA-box | Arabidopsis thaliana | 709 | + | 9 | ccTATAAAaa | core promoter element around -30 of transcription start |
| TATA-box | Arabidopsis thaliana | 97 | + | 4 | TATA | core promoter element around -30 of transcription start |
| TATA-box | Oryza sativa | 133 | - | 8 | TACATAAA | core promoter element around -30 of transcription start |
| TATA-box | Helianthus annuus | 137 | - | 6 | TATACA | core promoter element around -30 of transcription start |
| TATA-box | Arabidopsis thaliana | 139 | + | 4 | TATA | core promoter element around -30 of transcription start |
| TATA-box | Oryza sativa | 180 | - | 7 | TACAAAA | core promoter element around -30 of transcription start |
| TATA-box | Arabidopsis thaliana | 274 | + | 4 | TATA | core promoter element around -30 of transcription start |
| TATA-box | Arabidopsis thaliana | 332 | - | 5 | TATAA | core promoter element around -30 of transcription start |
| TATA-box | Arabidopsis thaliana | 333 | + | 4 | TATA | core promoter element around -30 of transcription start |
| TATA-box | Arabidopsis thaliana | 341 | + | 4 | TATA | core promoter element around -30 of transcription start |
| TATA-box | Brassica napus | 357 | + | 6 | ATATAT | core promoter element around -30 of transcription start |
| TATA-box | Arabidopsis thaliana | 358 | + | 4 | TATA | core promoter element around -30 of transcription start |
| TATA-box | Arabidopsis thaliana | 366 | - | 5 | TATAA | core promoter element around -30 of transcription start |
| TATA-box | Arabidopsis thaliana | 367 | + | 4 | TATA | core promoter element around -30 of transcription start |
| TATA-box | Oryza sativa | 369 | + | 7 | TACAAAA | core promoter element around -30 of transcription start |
| TATA-box | Arabidopsis thaliana | 428 | + | 4 | TATA | core promoter element around -30 of transcription start |
| TATA-box | Brassica napus | 453 | + | 6 | ATATAT | core promoter element around -30 of transcription start |
| TATA-box | Arabidopsis thaliana | 454 | + | 6 | TATATA | core promoter element around -30 of transcription start |
| TATA-box | Brassica napus | 455 | + | 6 | ATATAT | core promoter element around -30 of transcription start |
| TATA-box | Arabidopsis thaliana | 456 | + | 4 | TATA | core promoter element around -30 of transcription start |
| TATA-box | Arabidopsis thaliana | 507 | - | 7 | TATATAA | core promoter element around -30 of transcription start |
| TATA-box | Arabidopsis thaliana | 711 | + | 4 | TATA | core promoter element around -30 of transcription start |
| TATA-box | Arabidopsis thaliana | 508 | + | 6 | TATATA | core promoter element around -30 of transcription start |
| TATA-box | Oryza sativa | 722 | + | 7 | TACAAAA | core promoter element around -30 of transcription start |
| TATA-box | Arabidopsis thaliana | 757 | - | 4 | TATA | core promoter element around -30 of transcription start |
| TATA-box | Arabidopsis thaliana | 785 | - | 4 | TATA | core promoter element around -30 of transcription start |
| TATA-box | Arabidopsis thaliana | 800 | - | 6 | TATATA | core promoter element around -30 of transcription start |
| TATA-box | Brassica napus | 801 | - | 6 | ATATAT | core promoter element around -30 of transcription start |
| TATA-box | Arabidopsis thaliana | 802 | - | 6 | TATATA | core promoter element around -30 of transcription start |
| TATA-box | Arabidopsis thaliana | 804 | - | 4 | TATA | core promoter element around -30 of transcription start |
| TATA-box | Helianthus annuus | 855 | - | 6 | TATAAA | core promoter element around -30 of transcription start |
| TATA-box | Arabidopsis thaliana | 856 | - | 5 | TATAA | core promoter element around -30 of transcription start |
| TATA-box | Arabidopsis thaliana | 857 | - | 4 | TATA | core promoter element around -30 of transcription start |
| TATA-box | Pisum sativum | 864 | - | 7 | TATAAAA | core promoter element around -30 of transcription start |
| TATA-box | Helianthus annuus | 865 | - | 6 | TATAAA | core promoter element around -30 of transcription start |
| TATA-box | Arabidopsis thaliana | 866 | - | 7 | TATATAA | core promoter element around -30 of transcription start |
| TATA-box | Arabidopsis thaliana | 867 | - | 6 | TATATA | core promoter element around -30 of transcription start |
| TATA-box | Brassica napus | 868 | - | 6 | ATATAT | core promoter element around -30 of transcription start |
| TATA-box | Arabidopsis thaliana | 869 | - | 6 | TATATA | core promoter element around -30 of transcription start |
| TATA-box | Arabidopsis thaliana | 871 | - | 4 | TATA | core promoter element around -30 of transcription start |
| TATA-box | Brassica napus | 1158 | - | 6 | ATATAT | core promoter element around -30 of transcription start |
| TATA-box | Arabidopsis thaliana | 1159 | - | 6 | TATATA | core promoter element around -30 of transcription start |
| TATA-box | Brassica napus | 1160 | - | 6 | ATATAT | core promoter element around -30 of transcription start |
| TATA-box | Arabidopsis thaliana | 1161 | - | 4 | TATA | core promoter element around -30 of transcription start |
| TATA-box | Arabidopsis thaliana | 1236 | - | 9 | taTATAAAtc | core promoter element around -30 of transcription start |
| TATA-box | Brassica napus | 1237 | - | 6 | ATATAT | core promoter element around -30 of transcription start |
| TATA-box | Arabidopsis thaliana | 1238 | - | 6 | TATATA | core promoter element around -30 of transcription start |
| TATA-box | Brassica napus | 1239 | - | 6 | ATATAT | core promoter element around -30 of transcription start |
| TATA-box | Arabidopsis thaliana | 1240 | - | 6 | TATATA | core promoter element around -30 of transcription start |
| TATA-box | Brassica napus | 1241 | - | 6 | ATATAT | core promoter element around -30 of transcription start |
| TATA-box | Arabidopsis thaliana | 1242 | - | 6 | TATATA | core promoter element around -30 of transcription start |
| TATA-box | Brassica napus | 1243 | - | 6 | ATATAT | core promoter element around -30 of transcription start |
| TATA-box | Arabidopsis thaliana | 1244 | - | 6 | TATATA | core promoter element around -30 of transcription start |
| TATA-box | Brassica napus | 1245 | - | 6 | ATATAT | core promoter element around -30 of transcription start |
| TATA-box | Arabidopsis thaliana | 1246 | - | 6 | TATATA | core promoter element around -30 of transcription start |
| TATA-box | Brassica napus | 1247 | - | 6 | ATATAT | core promoter element around -30 of transcription start |
| TATA-box | Arabidopsis thaliana | 1248 | - | 6 | TATATA | core promoter element around -30 of transcription start |
| TATA-box | Brassica napus | 1249 | - | 6 | ATATAT | core promoter element around -30 of transcription start |
| TATA-box | Arabidopsis thaliana | 1250 | - | 6 | TATATA | core promoter element around -30 of transcription start |
| TATA-box | Brassica napus | 1251 | - | 6 | ATATAT | core promoter element around -30 of transcription start |
| TATA-box | Arabidopsis thaliana | 1252 | - | 6 | TATATA | core promoter element around -30 of transcription start |
| TATA-box | Brassica napus | 1253 | - | 6 | ATATAT | core promoter element around -30 of transcription start |
| TATA-box | Arabidopsis thaliana | 1254 | - | 6 | TATATA | core promoter element around -30 of transcription start |
| TATA-box | Brassica napus | 1255 | - | 6 | ATATAT | core promoter element around -30 of transcription start |
| TATA-box | Arabidopsis thaliana | 1256 | - | 6 | TATATA | core promoter element around -30 of transcription start |
| TATA-box | Brassica napus | 1257 | - | 6 | ATATAT | core promoter element around -30 of transcription start |
| TATA-box | Arabidopsis thaliana | 1258 | - | 6 | TATATA | core promoter element around -30 of transcription start |
| TATA-box | Brassica oleracea | 1259 | + | 6 | ATATAA | core promoter element around -30 of transcription start |
| TATA-box | Arabidopsis thaliana | 1260 | - | 4 | TATA | core promoter element around -30 of transcription start |
| TATA-box | Brassica napus | 1484 | + | 6 | ATTATA | core promoter element around -30 of transcription start |
| TATA-box | Arabidopsis thaliana | 1485 | - | 5 | TATAA | core promoter element around -30 of transcription start |
| TATA-box | Arabidopsis thaliana | 1486 | - | 4 | TATA | core promoter element around -30 of transcription start |

>003403   
+ TAAAAATAGT GAAAGAACCA CCGGCCGGAT AAACAAGAGT AAAGCTGTCT GAAAATAAAC CCTACTCACA   
  
  
+ CACATGGTAT TTCTCATATT TTATTTTATA GTCAATTATT AAGATAATAA GCATTCTAAC CATTTATGTA   
  
  
+ TACATGTTCA TACCGCGAGC GTTGCTCGGA GGAACACATT TTTGTATCAA GTGATGTTGC AAAATGATGC   
  
  
+ ATACAATCAG TACACATCAC TCTAGCTACT TACTCCATCT TGTAGTATTG CAATGCAGTA AAATATAGCA   
  
  
+ CTAGTCCCAG ATTAATTATT ATGGTTCTCC TTTCAAACAC ATAGTTAATG GTTATAATGA TATACTTAAG   
  
  
+ GGCAAAATAT ATTACTTATA CAAAATAACT CGATGGGTTA CGTAAAATAC ATGAAGAATT CAGTTTAATA   
  
  
+ AGTTTAGTAT ATGCTTGCAT GAGTATGATA CAATATATAT GTGACAAAAT AAAATACAAG TCTAAATTTA   
  
  
+ CGACACCACC ACCCCCTTAT ATAGAGTTAA AACGTGAGAT TCAAGAGTGT AGTTGGTGGA AAAAGATAAA   
  
  
+ TTGTAGGGTT ATTGTCTTGT AGAAATACAT ATACATAAAT ATTTATCTGT ACAAGATGTG CGTATGTACA   
  
  
+ ATAATCATTG ACAATTAACC TAAACATTGC AATTAACATA AACTTTGTTT CTTCTTAAAT TTTAGATGGT   
  
  
+ AAGTTCCTCC TATAAAATCA TTACAAAATC ATAAAATCCC AATAACACAC TTTATCTATA CGCAAAAAAA   
  
  
+ TTTGCCGGGG AAAATATAGT ACGTGGAGGT ATATATAGTA CGTACTTTCT CCTTTGCTTT CAAAGATCAT   
  
  
+ CAAATTTTAA TTAGTTTATA TTATTTTATA TATACACACA CACACACACA CACAAAGTGT GTGAGACATT   
  
  
+ TTTTTTATTT TTGTTTTTGA AATAGAAGGA GAGAGGAAGA GAGTGAATTA GATTTTTTTT TTTTTTTAAT   
  
  
+ TAAAGATATG TTAAGATTAC ATGTAGGTGA GATTTTGAAA AAAAAAACAG CCAAATTTAG TTATGTGAAA   
  
  
+ TTACATTTTT GCCCCGTATT TATTATTCAT ATTTTGTTTT AATTAGATGG TTAAAGTGGT AATTTGATAG   
  
  
+ GATTTTAGTT TACAATGAGT GTTTTATTAA TTAGTAGATA TATATTAAGT ATGTATTTTC AATTAAAAAT   
  
  
+ AAAGAAAAAA AAGCAGCATC TAGCATGGCA AGCACAACAC AAGCAGATAT ATATATATAT ATATATATAT   
  
  
+ ATAATTGGAA AAGTATTGTT GTCGTGGAGA CAAATTTAGG TTCTTGAGCA AAAACTCAAC TACTCTCTAG   
  
  
+ AGAGAGAGAG AGAGAGAGGT AGCAGTAACA CGACTTGTGT AGAAATTGTG AGGGACGAAT CGGTTAAGAA   
  
  
+ GTGCATGTCT TGTATCTCTC TCTAGAGCTA GAAAAATCCT AAGTGTTTCA TTTTTTTTGG AACAAACACA   
  
  
+ GAAAACCCCA AGAATTATAA TAATTAAAC  

- ATTTTTATCA CTTTCTTGGT GGCCGGCCTA TTTGTTCTCA TTTCGACAGA CTTTTATTTG GGATGAGTGT   
  
  
- GTGTACCATA AAGAGTATAA AATAAAATAT CAGTTAATAA TTCTATTATT CGTAAGATTG GTAAATACAT   
  
  
- ATGTACAAGT ATGGCGCTCG CAACGAGCCT CCTTGTGTAA AAACATAGTT CACTACAACG TTTTACTACG   
  
  
- TATGTTAGTC ATGTGTAGTG AGATCGATGA ATGAGGTAGA ACATCATAAC GTTACGTCAT TTTATATCGT   
  
  
- GATCAGGGTC TAATTAATAA TACCAAGAGG AAAGTTTGTG TATCAATTAC CAATATTACT ATATGAATTC   
  
  
- CCGTTTTATA TAATGAATAT GTTTTATTGA GCTACCCAAT GCATTTTATG TACTTCTTAA GTCAAATTAT   
  
  
- TCAAATCATA TACGAACGTA CTCATACTAT GTTATATATA CACTGTTTTA TTTTATGTTC AGATTTAAAT   
  
  
- GCTGTGGTGG TGGGGGAATA TATCTCAATT TTGCACTCTA AGTTCTCACA TCAACCACCT TTTTCTATTT   
  
  
- AACATCCCAA TAACAGAACA TCTTTATGTA TATGTATTTA TAAATAGACA TGTTCTACAC GCATACATGT   
  
  
- TATTAGTAAC TGTTAATTGG ATTTGTAACG TTAATTGTAT TTGAAACAAA GAAGAATTTA AAATCTACCA   
  
  
- TTCAAGGAGG ATATTTTAGT AATGTTTTAG TATTTTAGGG TTATTGTGTG AAATAGATAT GCGTTTTTTT   
  
  
- AAACGGCCCC TTTTATATCA TGCACCTCCA TATATATCAT GCATGAAAGA GGAAACGAAA GTTTCTAGTA   
  
  
- GTTTAAAATT AATCAAATAT AATAAAATAT ATATGTGTGT GTGTGTGTGT GTGTTTCACA CACTCTGTAA   
  
  
- AAAAAATAAA AACAAAAACT TTATCTTCCT CTCTCCTTCT CTCACTTAAT CTAAAAAAAA AAAAAAATTA   
  
  
- ATTTCTATAC AATTCTAATG TACATCCACT CTAAAACTTT TTTTTTTGTC GGTTTAAATC AATACACTTT   
  
  
- AATGTAAAAA CGGGGCATAA ATAATAAGTA TAAAACAAAA TTAATCTACC AATTTCACCA TTAAACTATC   
  
  
- CTAAAATCAA ATGTTACTCA CAAAATAATT AATCATCTAT ATATAATTCA TACATAAAAG TTAATTTTTA   
  
  
- TTTCTTTTTT TTCGTCGTAG ATCGTACCGT TCGTGTTGTG TTCGTCTATA TATATATATA TATATATATA   
  
  
- TATTAACCTT TTCATAACAA CAGCACCTCT GTTTAAATCC AAGAACTCGT TTTTGAGTTG ATGAGAGATC   
  
  
- TCTCTCTCTC TCTCTCTCCA TCGTCATTGT GCTGAACACA TCTTTAACAC TCCCTGCTTA GCCAATTCTT   
  
  
- CACGTACAGA ACATAGAGAG AGATCTCGAT CTTTTTAGGA TTCACAAAGT AAAAAAAACC TTGTTTGTGT   
  
  
- CTTTTGGGGT TCTTAATATT ATTAATTTG

+     Unnamed\_\_1

| Site Name | Organism | Position | Strand | Matrix score. | sequence | function |
| --- | --- | --- | --- | --- | --- | --- |
| Unnamed\_\_1 | Zea mays | 792 | + | 5 | CGTGG |  |
| Unnamed\_\_1 | Zea mays | 1283 | + | 5 | CGTGG |  |

>003403   
+ TAAAAATAGT GAAAGAACCA CCGGCCGGAT AAACAAGAGT AAAGCTGTCT GAAAATAAAC CCTACTCACA   
  
  
+ CACATGGTAT TTCTCATATT TTATTTTATA GTCAATTATT AAGATAATAA GCATTCTAAC CATTTATGTA   
  
  
+ TACATGTTCA TACCGCGAGC GTTGCTCGGA GGAACACATT TTTGTATCAA GTGATGTTGC AAAATGATGC   
  
  
+ ATACAATCAG TACACATCAC TCTAGCTACT TACTCCATCT TGTAGTATTG CAATGCAGTA AAATATAGCA   
  
  
+ CTAGTCCCAG ATTAATTATT ATGGTTCTCC TTTCAAACAC ATAGTTAATG GTTATAATGA TATACTTAAG   
  
  
+ GGCAAAATAT ATTACTTATA CAAAATAACT CGATGGGTTA CGTAAAATAC ATGAAGAATT CAGTTTAATA   
  
  
+ AGTTTAGTAT ATGCTTGCAT GAGTATGATA CAATATATAT GTGACAAAAT AAAATACAAG TCTAAATTTA   
  
  
+ CGACACCACC ACCCCCTTAT ATAGAGTTAA AACGTGAGAT TCAAGAGTGT AGTTGGTGGA AAAAGATAAA   
  
  
+ TTGTAGGGTT ATTGTCTTGT AGAAATACAT ATACATAAAT ATTTATCTGT ACAAGATGTG CGTATGTACA   
  
  
+ ATAATCATTG ACAATTAACC TAAACATTGC AATTAACATA AACTTTGTTT CTTCTTAAAT TTTAGATGGT   
  
  
+ AAGTTCCTCC TATAAAATCA TTACAAAATC ATAAAATCCC AATAACACAC TTTATCTATA CGCAAAAAAA   
  
  
+ TTTGCCGGGG AAAATATAGT ACGTGGAGGT ATATATAGTA CGTACTTTCT CCTTTGCTTT CAAAGATCAT   
  
  
+ CAAATTTTAA TTAGTTTATA TTATTTTATA TATACACACA CACACACACA CACAAAGTGT GTGAGACATT   
  
  
+ TTTTTTATTT TTGTTTTTGA AATAGAAGGA GAGAGGAAGA GAGTGAATTA GATTTTTTTT TTTTTTTAAT   
  
  
+ TAAAGATATG TTAAGATTAC ATGTAGGTGA GATTTTGAAA AAAAAAACAG CCAAATTTAG TTATGTGAAA   
  
  
+ TTACATTTTT GCCCCGTATT TATTATTCAT ATTTTGTTTT AATTAGATGG TTAAAGTGGT AATTTGATAG   
  
  
+ GATTTTAGTT TACAATGAGT GTTTTATTAA TTAGTAGATA TATATTAAGT ATGTATTTTC AATTAAAAAT   
  
  
+ AAAGAAAAAA AAGCAGCATC TAGCATGGCA AGCACAACAC AAGCAGATAT ATATATATAT ATATATATAT   
  
  
+ ATAATTGGAA AAGTATTGTT GTCGTGGAGA CAAATTTAGG TTCTTGAGCA AAAACTCAAC TACTCTCTAG   
  
  
+ AGAGAGAGAG AGAGAGAGGT AGCAGTAACA CGACTTGTGT AGAAATTGTG AGGGACGAAT CGGTTAAGAA   
  
  
+ GTGCATGTCT TGTATCTCTC TCTAGAGCTA GAAAAATCCT AAGTGTTTCA TTTTTTTTGG AACAAACACA   
  
  
+ GAAAACCCCA AGAATTATAA TAATTAAAC  

- ATTTTTATCA CTTTCTTGGT GGCCGGCCTA TTTGTTCTCA TTTCGACAGA CTTTTATTTG GGATGAGTGT   
  
  
- GTGTACCATA AAGAGTATAA AATAAAATAT CAGTTAATAA TTCTATTATT CGTAAGATTG GTAAATACAT   
  
  
- ATGTACAAGT ATGGCGCTCG CAACGAGCCT CCTTGTGTAA AAACATAGTT CACTACAACG TTTTACTACG   
  
  
- TATGTTAGTC ATGTGTAGTG AGATCGATGA ATGAGGTAGA ACATCATAAC GTTACGTCAT TTTATATCGT   
  
  
- GATCAGGGTC TAATTAATAA TACCAAGAGG AAAGTTTGTG TATCAATTAC CAATATTACT ATATGAATTC   
  
  
- CCGTTTTATA TAATGAATAT GTTTTATTGA GCTACCCAAT GCATTTTATG TACTTCTTAA GTCAAATTAT   
  
  
- TCAAATCATA TACGAACGTA CTCATACTAT GTTATATATA CACTGTTTTA TTTTATGTTC AGATTTAAAT   
  
  
- GCTGTGGTGG TGGGGGAATA TATCTCAATT TTGCACTCTA AGTTCTCACA TCAACCACCT TTTTCTATTT   
  
  
- AACATCCCAA TAACAGAACA TCTTTATGTA TATGTATTTA TAAATAGACA TGTTCTACAC GCATACATGT   
  
  
- TATTAGTAAC TGTTAATTGG ATTTGTAACG TTAATTGTAT TTGAAACAAA GAAGAATTTA AAATCTACCA   
  
  
- TTCAAGGAGG ATATTTTAGT AATGTTTTAG TATTTTAGGG TTATTGTGTG AAATAGATAT GCGTTTTTTT   
  
  
- AAACGGCCCC TTTTATATCA TGCACCTCCA TATATATCAT GCATGAAAGA GGAAACGAAA GTTTCTAGTA   
  
  
- GTTTAAAATT AATCAAATAT AATAAAATAT ATATGTGTGT GTGTGTGTGT GTGTTTCACA CACTCTGTAA   
  
  
- AAAAAATAAA AACAAAAACT TTATCTTCCT CTCTCCTTCT CTCACTTAAT CTAAAAAAAA AAAAAAATTA   
  
  
- ATTTCTATAC AATTCTAATG TACATCCACT CTAAAACTTT TTTTTTTGTC GGTTTAAATC AATACACTTT   
  
  
- AATGTAAAAA CGGGGCATAA ATAATAAGTA TAAAACAAAA TTAATCTACC AATTTCACCA TTAAACTATC   
  
  
- CTAAAATCAA ATGTTACTCA CAAAATAATT AATCATCTAT ATATAATTCA TACATAAAAG TTAATTTTTA   
  
  
- TTTCTTTTTT TTCGTCGTAG ATCGTACCGT TCGTGTTGTG TTCGTCTATA TATATATATA TATATATATA   
  
  
- TATTAACCTT TTCATAACAA CAGCACCTCT GTTTAAATCC AAGAACTCGT TTTTGAGTTG ATGAGAGATC   
  
  
- TCTCTCTCTC TCTCTCTCCA TCGTCATTGT GCTGAACACA TCTTTAACAC TCCCTGCTTA GCCAATTCTT   
  
  
- CACGTACAGA ACATAGAGAG AGATCTCGAT CTTTTTAGGA TTCACAAAGT AAAAAAAACC TTGTTTGTGT   
  
  
- CTTTTGGGGT TCTTAATATT ATTAATTTG

+     Unnamed\_\_2

| Site Name | Organism | Position | Strand | Matrix score. | sequence | function |
| --- | --- | --- | --- | --- | --- | --- |
| Unnamed\_\_2 | Zea mays | 775 | - | 6 | CCCCGG |  |

>003403   
+ TAAAAATAGT GAAAGAACCA CCGGCCGGAT AAACAAGAGT AAAGCTGTCT GAAAATAAAC CCTACTCACA   
  
  
+ CACATGGTAT TTCTCATATT TTATTTTATA GTCAATTATT AAGATAATAA GCATTCTAAC CATTTATGTA   
  
  
+ TACATGTTCA TACCGCGAGC GTTGCTCGGA GGAACACATT TTTGTATCAA GTGATGTTGC AAAATGATGC   
  
  
+ ATACAATCAG TACACATCAC TCTAGCTACT TACTCCATCT TGTAGTATTG CAATGCAGTA AAATATAGCA   
  
  
+ CTAGTCCCAG ATTAATTATT ATGGTTCTCC TTTCAAACAC ATAGTTAATG GTTATAATGA TATACTTAAG   
  
  
+ GGCAAAATAT ATTACTTATA CAAAATAACT CGATGGGTTA CGTAAAATAC ATGAAGAATT CAGTTTAATA   
  
  
+ AGTTTAGTAT ATGCTTGCAT GAGTATGATA CAATATATAT GTGACAAAAT AAAATACAAG TCTAAATTTA   
  
  
+ CGACACCACC ACCCCCTTAT ATAGAGTTAA AACGTGAGAT TCAAGAGTGT AGTTGGTGGA AAAAGATAAA   
  
  
+ TTGTAGGGTT ATTGTCTTGT AGAAATACAT ATACATAAAT ATTTATCTGT ACAAGATGTG CGTATGTACA   
  
  
+ ATAATCATTG ACAATTAACC TAAACATTGC AATTAACATA AACTTTGTTT CTTCTTAAAT TTTAGATGGT   
  
  
+ AAGTTCCTCC TATAAAATCA TTACAAAATC ATAAAATCCC AATAACACAC TTTATCTATA CGCAAAAAAA   
  
  
+ TTTGCCGGGG AAAATATAGT ACGTGGAGGT ATATATAGTA CGTACTTTCT CCTTTGCTTT CAAAGATCAT   
  
  
+ CAAATTTTAA TTAGTTTATA TTATTTTATA TATACACACA CACACACACA CACAAAGTGT GTGAGACATT   
  
  
+ TTTTTTATTT TTGTTTTTGA AATAGAAGGA GAGAGGAAGA GAGTGAATTA GATTTTTTTT TTTTTTTAAT   
  
  
+ TAAAGATATG TTAAGATTAC ATGTAGGTGA GATTTTGAAA AAAAAAACAG CCAAATTTAG TTATGTGAAA   
  
  
+ TTACATTTTT GCCCCGTATT TATTATTCAT ATTTTGTTTT AATTAGATGG TTAAAGTGGT AATTTGATAG   
  
  
+ GATTTTAGTT TACAATGAGT GTTTTATTAA TTAGTAGATA TATATTAAGT ATGTATTTTC AATTAAAAAT   
  
  
+ AAAGAAAAAA AAGCAGCATC TAGCATGGCA AGCACAACAC AAGCAGATAT ATATATATAT ATATATATAT   
  
  
+ ATAATTGGAA AAGTATTGTT GTCGTGGAGA CAAATTTAGG TTCTTGAGCA AAAACTCAAC TACTCTCTAG   
  
  
+ AGAGAGAGAG AGAGAGAGGT AGCAGTAACA CGACTTGTGT AGAAATTGTG AGGGACGAAT CGGTTAAGAA   
  
  
+ GTGCATGTCT TGTATCTCTC TCTAGAGCTA GAAAAATCCT AAGTGTTTCA TTTTTTTTGG AACAAACACA   
  
  
+ GAAAACCCCA AGAATTATAA TAATTAAAC  

- ATTTTTATCA CTTTCTTGGT GGCCGGCCTA TTTGTTCTCA TTTCGACAGA CTTTTATTTG GGATGAGTGT   
  
  
- GTGTACCATA AAGAGTATAA AATAAAATAT CAGTTAATAA TTCTATTATT CGTAAGATTG GTAAATACAT   
  
  
- ATGTACAAGT ATGGCGCTCG CAACGAGCCT CCTTGTGTAA AAACATAGTT CACTACAACG TTTTACTACG   
  
  
- TATGTTAGTC ATGTGTAGTG AGATCGATGA ATGAGGTAGA ACATCATAAC GTTACGTCAT TTTATATCGT   
  
  
- GATCAGGGTC TAATTAATAA TACCAAGAGG AAAGTTTGTG TATCAATTAC CAATATTACT ATATGAATTC   
  
  
- CCGTTTTATA TAATGAATAT GTTTTATTGA GCTACCCAAT GCATTTTATG TACTTCTTAA GTCAAATTAT   
  
  
- TCAAATCATA TACGAACGTA CTCATACTAT GTTATATATA CACTGTTTTA TTTTATGTTC AGATTTAAAT   
  
  
- GCTGTGGTGG TGGGGGAATA TATCTCAATT TTGCACTCTA AGTTCTCACA TCAACCACCT TTTTCTATTT   
  
  
- AACATCCCAA TAACAGAACA TCTTTATGTA TATGTATTTA TAAATAGACA TGTTCTACAC GCATACATGT   
  
  
- TATTAGTAAC TGTTAATTGG ATTTGTAACG TTAATTGTAT TTGAAACAAA GAAGAATTTA AAATCTACCA   
  
  
- TTCAAGGAGG ATATTTTAGT AATGTTTTAG TATTTTAGGG TTATTGTGTG AAATAGATAT GCGTTTTTTT   
  
  
- AAACGGCCCC TTTTATATCA TGCACCTCCA TATATATCAT GCATGAAAGA GGAAACGAAA GTTTCTAGTA   
  
  
- GTTTAAAATT AATCAAATAT AATAAAATAT ATATGTGTGT GTGTGTGTGT GTGTTTCACA CACTCTGTAA   
  
  
- AAAAAATAAA AACAAAAACT TTATCTTCCT CTCTCCTTCT CTCACTTAAT CTAAAAAAAA AAAAAAATTA   
  
  
- ATTTCTATAC AATTCTAATG TACATCCACT CTAAAACTTT TTTTTTTGTC GGTTTAAATC AATACACTTT   
  
  
- AATGTAAAAA CGGGGCATAA ATAATAAGTA TAAAACAAAA TTAATCTACC AATTTCACCA TTAAACTATC   
  
  
- CTAAAATCAA ATGTTACTCA CAAAATAATT AATCATCTAT ATATAATTCA TACATAAAAG TTAATTTTTA   
  
  
- TTTCTTTTTT TTCGTCGTAG ATCGTACCGT TCGTGTTGTG TTCGTCTATA TATATATATA TATATATATA   
  
  
- TATTAACCTT TTCATAACAA CAGCACCTCT GTTTAAATCC AAGAACTCGT TTTTGAGTTG ATGAGAGATC   
  
  
- TCTCTCTCTC TCTCTCTCCA TCGTCATTGT GCTGAACACA TCTTTAACAC TCCCTGCTTA GCCAATTCTT   
  
  
- CACGTACAGA ACATAGAGAG AGATCTCGAT CTTTTTAGGA TTCACAAAGT AAAAAAAACC TTGTTTGTGT   
  
  
- CTTTTGGGGT TCTTAATATT ATTAATTTG

+     Unnamed\_\_4

| Site Name | Organism | Position | Strand | Matrix score. | sequence | function |
| --- | --- | --- | --- | --- | --- | --- |
| Unnamed\_\_4 | Petroselinum hortense | 168 | - | 4 | CTCC |  |
| Unnamed\_\_4 | Petroselinum hortense | 795 | - | 4 | CTCC |  |
| Unnamed\_\_4 | Petroselinum hortense | 307 | + | 4 | CTCC |  |
| Unnamed\_\_4 | Petroselinum hortense | 938 | - | 4 | CTCC |  |
| Unnamed\_\_4 | Petroselinum hortense | 243 | + | 4 | CTCC |  |
| Unnamed\_\_4 | Petroselinum hortense | 819 | + | 4 | CTCC |  |
| Unnamed\_\_4 | Petroselinum hortense | 707 | + | 4 | CTCC |  |
| Unnamed\_\_4 | Petroselinum hortense | 1286 | - | 4 | CTCC |  |

>003403   
+ TAAAAATAGT GAAAGAACCA CCGGCCGGAT AAACAAGAGT AAAGCTGTCT GAAAATAAAC CCTACTCACA   
  
  
+ CACATGGTAT TTCTCATATT TTATTTTATA GTCAATTATT AAGATAATAA GCATTCTAAC CATTTATGTA   
  
  
+ TACATGTTCA TACCGCGAGC GTTGCTCGGA GGAACACATT TTTGTATCAA GTGATGTTGC AAAATGATGC   
  
  
+ ATACAATCAG TACACATCAC TCTAGCTACT TACTCCATCT TGTAGTATTG CAATGCAGTA AAATATAGCA   
  
  
+ CTAGTCCCAG ATTAATTATT ATGGTTCTCC TTTCAAACAC ATAGTTAATG GTTATAATGA TATACTTAAG   
  
  
+ GGCAAAATAT ATTACTTATA CAAAATAACT CGATGGGTTA CGTAAAATAC ATGAAGAATT CAGTTTAATA   
  
  
+ AGTTTAGTAT ATGCTTGCAT GAGTATGATA CAATATATAT GTGACAAAAT AAAATACAAG TCTAAATTTA   
  
  
+ CGACACCACC ACCCCCTTAT ATAGAGTTAA AACGTGAGAT TCAAGAGTGT AGTTGGTGGA AAAAGATAAA   
  
  
+ TTGTAGGGTT ATTGTCTTGT AGAAATACAT ATACATAAAT ATTTATCTGT ACAAGATGTG CGTATGTACA   
  
  
+ ATAATCATTG ACAATTAACC TAAACATTGC AATTAACATA AACTTTGTTT CTTCTTAAAT TTTAGATGGT   
  
  
+ AAGTTCCTCC TATAAAATCA TTACAAAATC ATAAAATCCC AATAACACAC TTTATCTATA CGCAAAAAAA   
  
  
+ TTTGCCGGGG AAAATATAGT ACGTGGAGGT ATATATAGTA CGTACTTTCT CCTTTGCTTT CAAAGATCAT   
  
  
+ CAAATTTTAA TTAGTTTATA TTATTTTATA TATACACACA CACACACACA CACAAAGTGT GTGAGACATT   
  
  
+ TTTTTTATTT TTGTTTTTGA AATAGAAGGA GAGAGGAAGA GAGTGAATTA GATTTTTTTT TTTTTTTAAT   
  
  
+ TAAAGATATG TTAAGATTAC ATGTAGGTGA GATTTTGAAA AAAAAAACAG CCAAATTTAG TTATGTGAAA   
  
  
+ TTACATTTTT GCCCCGTATT TATTATTCAT ATTTTGTTTT AATTAGATGG TTAAAGTGGT AATTTGATAG   
  
  
+ GATTTTAGTT TACAATGAGT GTTTTATTAA TTAGTAGATA TATATTAAGT ATGTATTTTC AATTAAAAAT   
  
  
+ AAAGAAAAAA AAGCAGCATC TAGCATGGCA AGCACAACAC AAGCAGATAT ATATATATAT ATATATATAT   
  
  
+ ATAATTGGAA AAGTATTGTT GTCGTGGAGA CAAATTTAGG TTCTTGAGCA AAAACTCAAC TACTCTCTAG   
  
  
+ AGAGAGAGAG AGAGAGAGGT AGCAGTAACA CGACTTGTGT AGAAATTGTG AGGGACGAAT CGGTTAAGAA   
  
  
+ GTGCATGTCT TGTATCTCTC TCTAGAGCTA GAAAAATCCT AAGTGTTTCA TTTTTTTTGG AACAAACACA   
  
  
+ GAAAACCCCA AGAATTATAA TAATTAAAC  

- ATTTTTATCA CTTTCTTGGT GGCCGGCCTA TTTGTTCTCA TTTCGACAGA CTTTTATTTG GGATGAGTGT   
  
  
- GTGTACCATA AAGAGTATAA AATAAAATAT CAGTTAATAA TTCTATTATT CGTAAGATTG GTAAATACAT   
  
  
- ATGTACAAGT ATGGCGCTCG CAACGAGCCT CCTTGTGTAA AAACATAGTT CACTACAACG TTTTACTACG   
  
  
- TATGTTAGTC ATGTGTAGTG AGATCGATGA ATGAGGTAGA ACATCATAAC GTTACGTCAT TTTATATCGT   
  
  
- GATCAGGGTC TAATTAATAA TACCAAGAGG AAAGTTTGTG TATCAATTAC CAATATTACT ATATGAATTC   
  
  
- CCGTTTTATA TAATGAATAT GTTTTATTGA GCTACCCAAT GCATTTTATG TACTTCTTAA GTCAAATTAT   
  
  
- TCAAATCATA TACGAACGTA CTCATACTAT GTTATATATA CACTGTTTTA TTTTATGTTC AGATTTAAAT   
  
  
- GCTGTGGTGG TGGGGGAATA TATCTCAATT TTGCACTCTA AGTTCTCACA TCAACCACCT TTTTCTATTT   
  
  
- AACATCCCAA TAACAGAACA TCTTTATGTA TATGTATTTA TAAATAGACA TGTTCTACAC GCATACATGT   
  
  
- TATTAGTAAC TGTTAATTGG ATTTGTAACG TTAATTGTAT TTGAAACAAA GAAGAATTTA AAATCTACCA   
  
  
- TTCAAGGAGG ATATTTTAGT AATGTTTTAG TATTTTAGGG TTATTGTGTG AAATAGATAT GCGTTTTTTT   
  
  
- AAACGGCCCC TTTTATATCA TGCACCTCCA TATATATCAT GCATGAAAGA GGAAACGAAA GTTTCTAGTA   
  
  
- GTTTAAAATT AATCAAATAT AATAAAATAT ATATGTGTGT GTGTGTGTGT GTGTTTCACA CACTCTGTAA   
  
  
- AAAAAATAAA AACAAAAACT TTATCTTCCT CTCTCCTTCT CTCACTTAAT CTAAAAAAAA AAAAAAATTA   
  
  
- ATTTCTATAC AATTCTAATG TACATCCACT CTAAAACTTT TTTTTTTGTC GGTTTAAATC AATACACTTT   
  
  
- AATGTAAAAA CGGGGCATAA ATAATAAGTA TAAAACAAAA TTAATCTACC AATTTCACCA TTAAACTATC   
  
  
- CTAAAATCAA ATGTTACTCA CAAAATAATT AATCATCTAT ATATAATTCA TACATAAAAG TTAATTTTTA   
  
  
- TTTCTTTTTT TTCGTCGTAG ATCGTACCGT TCGTGTTGTG TTCGTCTATA TATATATATA TATATATATA   
  
  
- TATTAACCTT TTCATAACAA CAGCACCTCT GTTTAAATCC AAGAACTCGT TTTTGAGTTG ATGAGAGATC   
  
  
- TCTCTCTCTC TCTCTCTCCA TCGTCATTGT GCTGAACACA TCTTTAACAC TCCCTGCTTA GCCAATTCTT   
  
  
- CACGTACAGA ACATAGAGAG AGATCTCGAT CTTTTTAGGA TTCACAAAGT AAAAAAAACC TTGTTTGTGT   
  
  
- CTTTTGGGGT TCTTAATATT ATTAATTTG

+     Unnamed\_\_6

| Site Name | Organism | Position | Strand | Matrix score. | sequence | function |
| --- | --- | --- | --- | --- | --- | --- |
| Unnamed\_\_6 | Zea mays | 1235 | - | 10 | taTAAATATct |  |

>003403   
+ TAAAAATAGT GAAAGAACCA CCGGCCGGAT AAACAAGAGT AAAGCTGTCT GAAAATAAAC CCTACTCACA   
  
  
+ CACATGGTAT TTCTCATATT TTATTTTATA GTCAATTATT AAGATAATAA GCATTCTAAC CATTTATGTA   
  
  
+ TACATGTTCA TACCGCGAGC GTTGCTCGGA GGAACACATT TTTGTATCAA GTGATGTTGC AAAATGATGC   
  
  
+ ATACAATCAG TACACATCAC TCTAGCTACT TACTCCATCT TGTAGTATTG CAATGCAGTA AAATATAGCA   
  
  
+ CTAGTCCCAG ATTAATTATT ATGGTTCTCC TTTCAAACAC ATAGTTAATG GTTATAATGA TATACTTAAG   
  
  
+ GGCAAAATAT ATTACTTATA CAAAATAACT CGATGGGTTA CGTAAAATAC ATGAAGAATT CAGTTTAATA   
  
  
+ AGTTTAGTAT ATGCTTGCAT GAGTATGATA CAATATATAT GTGACAAAAT AAAATACAAG TCTAAATTTA   
  
  
+ CGACACCACC ACCCCCTTAT ATAGAGTTAA AACGTGAGAT TCAAGAGTGT AGTTGGTGGA AAAAGATAAA   
  
  
+ TTGTAGGGTT ATTGTCTTGT AGAAATACAT ATACATAAAT ATTTATCTGT ACAAGATGTG CGTATGTACA   
  
  
+ ATAATCATTG ACAATTAACC TAAACATTGC AATTAACATA AACTTTGTTT CTTCTTAAAT TTTAGATGGT   
  
  
+ AAGTTCCTCC TATAAAATCA TTACAAAATC ATAAAATCCC AATAACACAC TTTATCTATA CGCAAAAAAA   
  
  
+ TTTGCCGGGG AAAATATAGT ACGTGGAGGT ATATATAGTA CGTACTTTCT CCTTTGCTTT CAAAGATCAT   
  
  
+ CAAATTTTAA TTAGTTTATA TTATTTTATA TATACACACA CACACACACA CACAAAGTGT GTGAGACATT   
  
  
+ TTTTTTATTT TTGTTTTTGA AATAGAAGGA GAGAGGAAGA GAGTGAATTA GATTTTTTTT TTTTTTTAAT   
  
  
+ TAAAGATATG TTAAGATTAC ATGTAGGTGA GATTTTGAAA AAAAAAACAG CCAAATTTAG TTATGTGAAA   
  
  
+ TTACATTTTT GCCCCGTATT TATTATTCAT ATTTTGTTTT AATTAGATGG TTAAAGTGGT AATTTGATAG   
  
  
+ GATTTTAGTT TACAATGAGT GTTTTATTAA TTAGTAGATA TATATTAAGT ATGTATTTTC AATTAAAAAT   
  
  
+ AAAGAAAAAA AAGCAGCATC TAGCATGGCA AGCACAACAC AAGCAGATAT ATATATATAT ATATATATAT   
  
  
+ ATAATTGGAA AAGTATTGTT GTCGTGGAGA CAAATTTAGG TTCTTGAGCA AAAACTCAAC TACTCTCTAG   
  
  
+ AGAGAGAGAG AGAGAGAGGT AGCAGTAACA CGACTTGTGT AGAAATTGTG AGGGACGAAT CGGTTAAGAA   
  
  
+ GTGCATGTCT TGTATCTCTC TCTAGAGCTA GAAAAATCCT AAGTGTTTCA TTTTTTTTGG AACAAACACA   
  
  
+ GAAAACCCCA AGAATTATAA TAATTAAAC  

- ATTTTTATCA CTTTCTTGGT GGCCGGCCTA TTTGTTCTCA TTTCGACAGA CTTTTATTTG GGATGAGTGT   
  
  
- GTGTACCATA AAGAGTATAA AATAAAATAT CAGTTAATAA TTCTATTATT CGTAAGATTG GTAAATACAT   
  
  
- ATGTACAAGT ATGGCGCTCG CAACGAGCCT CCTTGTGTAA AAACATAGTT CACTACAACG TTTTACTACG   
  
  
- TATGTTAGTC ATGTGTAGTG AGATCGATGA ATGAGGTAGA ACATCATAAC GTTACGTCAT TTTATATCGT   
  
  
- GATCAGGGTC TAATTAATAA TACCAAGAGG AAAGTTTGTG TATCAATTAC CAATATTACT ATATGAATTC   
  
  
- CCGTTTTATA TAATGAATAT GTTTTATTGA GCTACCCAAT GCATTTTATG TACTTCTTAA GTCAAATTAT   
  
  
- TCAAATCATA TACGAACGTA CTCATACTAT GTTATATATA CACTGTTTTA TTTTATGTTC AGATTTAAAT   
  
  
- GCTGTGGTGG TGGGGGAATA TATCTCAATT TTGCACTCTA AGTTCTCACA TCAACCACCT TTTTCTATTT   
  
  
- AACATCCCAA TAACAGAACA TCTTTATGTA TATGTATTTA TAAATAGACA TGTTCTACAC GCATACATGT   
  
  
- TATTAGTAAC TGTTAATTGG ATTTGTAACG TTAATTGTAT TTGAAACAAA GAAGAATTTA AAATCTACCA   
  
  
- TTCAAGGAGG ATATTTTAGT AATGTTTTAG TATTTTAGGG TTATTGTGTG AAATAGATAT GCGTTTTTTT   
  
  
- AAACGGCCCC TTTTATATCA TGCACCTCCA TATATATCAT GCATGAAAGA GGAAACGAAA GTTTCTAGTA   
  
  
- GTTTAAAATT AATCAAATAT AATAAAATAT ATATGTGTGT GTGTGTGTGT GTGTTTCACA CACTCTGTAA   
  
  
- AAAAAATAAA AACAAAAACT TTATCTTCCT CTCTCCTTCT CTCACTTAAT CTAAAAAAAA AAAAAAATTA   
  
  
- ATTTCTATAC AATTCTAATG TACATCCACT CTAAAACTTT TTTTTTTGTC GGTTTAAATC AATACACTTT   
  
  
- AATGTAAAAA CGGGGCATAA ATAATAAGTA TAAAACAAAA TTAATCTACC AATTTCACCA TTAAACTATC   
  
  
- CTAAAATCAA ATGTTACTCA CAAAATAATT AATCATCTAT ATATAATTCA TACATAAAAG TTAATTTTTA   
  
  
- TTTCTTTTTT TTCGTCGTAG ATCGTACCGT TCGTGTTGTG TTCGTCTATA TATATATATA TATATATATA   
  
  
- TATTAACCTT TTCATAACAA CAGCACCTCT GTTTAAATCC AAGAACTCGT TTTTGAGTTG ATGAGAGATC   
  
  
- TCTCTCTCTC TCTCTCTCCA TCGTCATTGT GCTGAACACA TCTTTAACAC TCCCTGCTTA GCCAATTCTT   
  
  
- CACGTACAGA ACATAGAGAG AGATCTCGAT CTTTTTAGGA TTCACAAAGT AAAAAAAACC TTGTTTGTGT   
  
  
- CTTTTGGGGT TCTTAATATT ATTAATTTG

+     chs-CMA2a

| Site Name | Organism | Position | Strand | Matrix score. | sequence | function |
| --- | --- | --- | --- | --- | --- | --- |
| chs-CMA2a | Petroselinum crispum | 187 | - | 8 | TCACTTGA | part of a light responsive element |

>003403   
+ TAAAAATAGT GAAAGAACCA CCGGCCGGAT AAACAAGAGT AAAGCTGTCT GAAAATAAAC CCTACTCACA   
  
  
+ CACATGGTAT TTCTCATATT TTATTTTATA GTCAATTATT AAGATAATAA GCATTCTAAC CATTTATGTA   
  
  
+ TACATGTTCA TACCGCGAGC GTTGCTCGGA GGAACACATT TTTGTATCAA GTGATGTTGC AAAATGATGC   
  
  
+ ATACAATCAG TACACATCAC TCTAGCTACT TACTCCATCT TGTAGTATTG CAATGCAGTA AAATATAGCA   
  
  
+ CTAGTCCCAG ATTAATTATT ATGGTTCTCC TTTCAAACAC ATAGTTAATG GTTATAATGA TATACTTAAG   
  
  
+ GGCAAAATAT ATTACTTATA CAAAATAACT CGATGGGTTA CGTAAAATAC ATGAAGAATT CAGTTTAATA   
  
  
+ AGTTTAGTAT ATGCTTGCAT GAGTATGATA CAATATATAT GTGACAAAAT AAAATACAAG TCTAAATTTA   
  
  
+ CGACACCACC ACCCCCTTAT ATAGAGTTAA AACGTGAGAT TCAAGAGTGT AGTTGGTGGA AAAAGATAAA   
  
  
+ TTGTAGGGTT ATTGTCTTGT AGAAATACAT ATACATAAAT ATTTATCTGT ACAAGATGTG CGTATGTACA   
  
  
+ ATAATCATTG ACAATTAACC TAAACATTGC AATTAACATA AACTTTGTTT CTTCTTAAAT TTTAGATGGT   
  
  
+ AAGTTCCTCC TATAAAATCA TTACAAAATC ATAAAATCCC AATAACACAC TTTATCTATA CGCAAAAAAA   
  
  
+ TTTGCCGGGG AAAATATAGT ACGTGGAGGT ATATATAGTA CGTACTTTCT CCTTTGCTTT CAAAGATCAT   
  
  
+ CAAATTTTAA TTAGTTTATA TTATTTTATA TATACACACA CACACACACA CACAAAGTGT GTGAGACATT   
  
  
+ TTTTTTATTT TTGTTTTTGA AATAGAAGGA GAGAGGAAGA GAGTGAATTA GATTTTTTTT TTTTTTTAAT   
  
  
+ TAAAGATATG TTAAGATTAC ATGTAGGTGA GATTTTGAAA AAAAAAACAG CCAAATTTAG TTATGTGAAA   
  
  
+ TTACATTTTT GCCCCGTATT TATTATTCAT ATTTTGTTTT AATTAGATGG TTAAAGTGGT AATTTGATAG   
  
  
+ GATTTTAGTT TACAATGAGT GTTTTATTAA TTAGTAGATA TATATTAAGT ATGTATTTTC AATTAAAAAT   
  
  
+ AAAGAAAAAA AAGCAGCATC TAGCATGGCA AGCACAACAC AAGCAGATAT ATATATATAT ATATATATAT   
  
  
+ ATAATTGGAA AAGTATTGTT GTCGTGGAGA CAAATTTAGG TTCTTGAGCA AAAACTCAAC TACTCTCTAG   
  
  
+ AGAGAGAGAG AGAGAGAGGT AGCAGTAACA CGACTTGTGT AGAAATTGTG AGGGACGAAT CGGTTAAGAA   
  
  
+ GTGCATGTCT TGTATCTCTC TCTAGAGCTA GAAAAATCCT AAGTGTTTCA TTTTTTTTGG AACAAACACA   
  
  
+ GAAAACCCCA AGAATTATAA TAATTAAAC  

- ATTTTTATCA CTTTCTTGGT GGCCGGCCTA TTTGTTCTCA TTTCGACAGA CTTTTATTTG GGATGAGTGT   
  
  
- GTGTACCATA AAGAGTATAA AATAAAATAT CAGTTAATAA TTCTATTATT CGTAAGATTG GTAAATACAT   
  
  
- ATGTACAAGT ATGGCGCTCG CAACGAGCCT CCTTGTGTAA AAACATAGTT CACTACAACG TTTTACTACG   
  
  
- TATGTTAGTC ATGTGTAGTG AGATCGATGA ATGAGGTAGA ACATCATAAC GTTACGTCAT TTTATATCGT   
  
  
- GATCAGGGTC TAATTAATAA TACCAAGAGG AAAGTTTGTG TATCAATTAC CAATATTACT ATATGAATTC   
  
  
- CCGTTTTATA TAATGAATAT GTTTTATTGA GCTACCCAAT GCATTTTATG TACTTCTTAA GTCAAATTAT   
  
  
- TCAAATCATA TACGAACGTA CTCATACTAT GTTATATATA CACTGTTTTA TTTTATGTTC AGATTTAAAT   
  
  
- GCTGTGGTGG TGGGGGAATA TATCTCAATT TTGCACTCTA AGTTCTCACA TCAACCACCT TTTTCTATTT   
  
  
- AACATCCCAA TAACAGAACA TCTTTATGTA TATGTATTTA TAAATAGACA TGTTCTACAC GCATACATGT   
  
  
- TATTAGTAAC TGTTAATTGG ATTTGTAACG TTAATTGTAT TTGAAACAAA GAAGAATTTA AAATCTACCA   
  
  
- TTCAAGGAGG ATATTTTAGT AATGTTTTAG TATTTTAGGG TTATTGTGTG AAATAGATAT GCGTTTTTTT   
  
  
- AAACGGCCCC TTTTATATCA TGCACCTCCA TATATATCAT GCATGAAAGA GGAAACGAAA GTTTCTAGTA   
  
  
- GTTTAAAATT AATCAAATAT AATAAAATAT ATATGTGTGT GTGTGTGTGT GTGTTTCACA CACTCTGTAA   
  
  
- AAAAAATAAA AACAAAAACT TTATCTTCCT CTCTCCTTCT CTCACTTAAT CTAAAAAAAA AAAAAAATTA   
  
  
- ATTTCTATAC AATTCTAATG TACATCCACT CTAAAACTTT TTTTTTTGTC GGTTTAAATC AATACACTTT   
  
  
- AATGTAAAAA CGGGGCATAA ATAATAAGTA TAAAACAAAA TTAATCTACC AATTTCACCA TTAAACTATC   
  
  
- CTAAAATCAA ATGTTACTCA CAAAATAATT AATCATCTAT ATATAATTCA TACATAAAAG TTAATTTTTA   
  
  
- TTTCTTTTTT TTCGTCGTAG ATCGTACCGT TCGTGTTGTG TTCGTCTATA TATATATATA TATATATATA   
  
  
- TATTAACCTT TTCATAACAA CAGCACCTCT GTTTAAATCC AAGAACTCGT TTTTGAGTTG ATGAGAGATC   
  
  
- TCTCTCTCTC TCTCTCTCCA TCGTCATTGT GCTGAACACA TCTTTAACAC TCCCTGCTTA GCCAATTCTT   
  
  
- CACGTACAGA ACATAGAGAG AGATCTCGAT CTTTTTAGGA TTCACAAAGT AAAAAAAACC TTGTTTGTGT   
  
  
- CTTTTGGGGT TCTTAATATT ATTAATTTG
