# Supplementary material for: In Silico Genome-Wide Analysis of Respiratory Burst Oxidase Homolog (RBOH) Family Genes in Five Fruit-Producing Trees, and Potential Functional Analysis on Lignification of Stone Cells in Chinese White Pear
Source: Cells. 2019 May 29;8(6):520. doi: 10.3390/cells8060520 (PMC6627160; doi:10.3390/cells8060520)
Supplement: Supplementary file 1 [file cells-08-00520-s001.zip › Supplementary Material/Spreadsheets S2. Numbers of cis-elements in promoter region of PbRBOHs.tar.zip!/PbRBOHD.html]

Content-Type: text/html; charset=ISO-8859-1


PlantCARE


Webmaster Firefox specific output  
To save the result:
click on the frame with the right mouse button and save the source code as a text file with extension .html  
REFERENCE:PlantCARE: a database of plant cis-acting regulatory elements and a portal to tools for in silico analysis of promoter sequences.  
Lescot, M., Déhais, P., Moreau, Y., De Moor, B., Rouzé ,P.,and Rombauts, S.  
Nucleic Acids Res., Database issue(2002), 30(1):325-327.   


---

>006277   
+ TGATGAATAA AACAGAATGA TAATTAACAA ACAGAAACAG TTGTGTATAA TTTAAAAATG AATGGAGGGA   
  
  
+ GGTGGTGGTG TTTATAAGCG TTGTAAAAAT GCAGAGGTGA GGAGAGTGAT GAGTGAGTTT TGTTTTCTTC   
  
  
+ TATTCTTTTT GTGAAAAGAG AGTGAATTGG TCGTGGAACG CGGTTTGTTC GGAATCTCAG GGAAAAGACA   
  
  
+ TGTACGCGGA ACGCAGCATT AGACAGAGAA AAAAGAGAGA AGCAGACAAA GCCTTGTATT CAGAGAAGGA   
  
  
+ AGAAAGCTAA AAGTACTCAA TTATCTCGTG GGGAGCATAC AATAAAACCA CCACAATTAT ATTATACATG   
  
  
+ TACATATTTA TGTGTTATAT ATGTACGTTT ATTTATTTAT TTTATGTAAA TTGATATTTG TTATGAAATT   
  
  
+ ATATGCGGAT GTGGTTACGG TTTGTTTGAA TGTGTTACAC GCAAGGATTC AAAAGGTGCT AGGCACTGGT   
  
  
+ TGGGCGACGG ATATGTGTCT AGGTGGGAGT TTAGGCAGAG CCTAGGTGGG GGTTTAGGCA GTTTTTTTTT   
  
  
+ TTTAATTTTA ATTGAATTTA TTATATAACA TATAAATAAC TGCCTACTTG TACTTAAAAA AATACATCAT   
  
  
+ TGTATTAAAA CGCTTGAATT GCAAAATAGA ATGATATATT GATTATAAAA TATTCAAACA TATTGAAAAT   
  
  
+ ATGAGAAACA ACATACAATG AGTGCTCATC CAAACATTCA AGAAATCTCG TACAATTTAA TGAAAAAATA   
  
  
+ AAATGCAAGA TGGAAGTTAT CTTTCTTAAG TAAGAATTTC GACCTAAGTG GGTGGACAGG TCTAGGTGGG   
  
  
+ CGCCTAGGTA GGCTAGGTGC CCTTTCTTAA TTTTAAATGC TTAGGGACTA ATCATGTCGG TAACCAGCCG   
  
  
+ CCTAGCACTA GGCAGAGATT TTTAGAACAC TAGTTACATG CATTATTTGT AATGGTGACT TATTATTTGA   
  
  
+ ATGTCAAGCT TTTATGCATT ATCAATTGTG TTTTTATATC CCGCAAACTG AGAATTTAGC TTGAGTAGTT   
  
  
+ ATTTTAGTTT TATTCGTCAT CTAACAGTGT TGAGAAATTT GGAAAAATAA CTAAATTTTA AATCCCGTAT   
  
  
+ AGAATGTTAA CGATCATTTT AATTTTATGT AATTATGTAA TTATAATCAA TACTTGATGT AAAAGTATTA   
  
  
+ ATATACCCTT AATATTAGGA TTTCTGACAA CAACCAAAAC ACAACCTTTA AACTACCTTA AAATGAGGAA   
  
  
+ ATAATCTATA TCACTTTTTA GTTTTCATTA TTCTCTTCAA CTTTTGAACT ACTTTCTGCA ACCTTTCGGA   
  
  
+ TTTTTGCAAA ACGATACAAC AGCCTTATTG TTACCTTTGA CTGCACTTTC ATTCTTCAAT CACATCAATT   
  
  
+ TTAAAATCTT ATTGGAAGTA CCGAGATACC TAGCAATTTA ATCTCGAAGA CTTTGTTTTT TTTATTCTTT   
  
  
+ TTTGTTTCAA TTCAGGCTTT CATTATGGT  

- ACTACTTATT TTGTCTTACT ATTAATTGTT TGTCTTTGTC AACACATATT AAATTTTTAC TTACCTCCCT   
  
  
- CCACCACCAC AAATATTCGC AACATTTTTA CGTCTCCACT CCTCTCACTA CTCACTCAAA ACAAAAGAAG   
  
  
- ATAAGAAAAA CACTTTTCTC TCACTTAACC AGCACCTTGC GCCAAACAAG CCTTAGAGTC CCTTTTCTGT   
  
  
- ACATGCGCCT TGCGTCGTAA TCTGTCTCTT TTTTCTCTCT TCGTCTGTTT CGGAACATAA GTCTCTTCCT   
  
  
- TCTTTCGATT TTCATGAGTT AATAGAGCAC CCCTCGTATG TTATTTTGGT GGTGTTAATA TAATATGTAC   
  
  
- ATGTATAAAT ACACAATATA TACATGCAAA TAAATAAATA AAATACATTT AACTATAAAC AATACTTTAA   
  
  
- TATACGCCTA CACCAATGCC AAACAAACTT ACACAATGTG CGTTCCTAAG TTTTCCACGA TCCGTGACCA   
  
  
- ACCCGCTGCC TATACACAGA TCCACCCTCA AATCCGTCTC GGATCCACCC CCAAATCCGT CAAAAAAAAA   
  
  
- AAATTAAAAT TAACTTAAAT AATATATTGT ATATTTATTG ACGGATGAAC ATGAATTTTT TTATGTAGTA   
  
  
- ACATAATTTT GCGAACTTAA CGTTTTATCT TACTATATAA CTAATATTTT ATAAGTTTGT ATAACTTTTA   
  
  
- TACTCTTTGT TGTATGTTAC TCACGAGTAG GTTTGTAAGT TCTTTAGAGC ATGTTAAATT ACTTTTTTAT   
  
  
- TTTACGTTCT ACCTTCAATA GAAAGAATTC ATTCTTAAAG CTGGATTCAC CCACCTGTCC AGATCCACCC   
  
  
- GCGGATCCAT CCGATCCACG GGAAAGAATT AAAATTTACG AATCCCTGAT TAGTACAGCC ATTGGTCGGC   
  
  
- GGATCGTGAT CCGTCTCTAA AAATCTTGTG ATCAATGTAC GTAATAAACA TTACCACTGA ATAATAAACT   
  
  
- TACAGTTCGA AAATACGTAA TAGTTAACAC AAAAATATAG GGCGTTTGAC TCTTAAATCG AACTCATCAA   
  
  
- TAAAATCAAA ATAAGCAGTA GATTGTCACA ACTCTTTAAA CCTTTTTATT GATTTAAAAT TTAGGGCATA   
  
  
- TCTTACAATT GCTAGTAAAA TTAAAATACA TTAATACATT AATATTAGTT ATGAACTACA TTTTCATAAT   
  
  
- TATATGGGAA TTATAATCCT AAAGACTGTT GTTGGTTTTG TGTTGGAAAT TTGATGGAAT TTTACTCCTT   
  
  
- TATTAGATAT AGTGAAAAAT CAAAAGTAAT AAGAGAAGTT GAAAACTTGA TGAAAGACGT TGGAAAGCCT   
  
  
- AAAAACGTTT TGCTATGTTG TCGGAATAAC AATGGAAACT GACGTGAAAG TAAGAAGTTA GTGTAGTTAA   
  
  
- AATTTTAGAA TAACCTTCAT GGCTCTATGG ATCGTTAAAT TAGAGCTTCT GAAACAAAAA AAATAAGAAA   
  
  
- AAACAAAGTT AAGTCCGAAA GTAATACCA

  
  
Motifs Found  

+   

| Site Name | Organism | Position | Strand | Matrix score. | sequence | function |
| --- | --- | --- | --- | --- | --- | --- |
|  | organism | 525 | - | 4 | motif\_sequence | short\_function |
|  | organism | 101 | - | 4 | motif\_sequence | short\_function |
|  | organism | 350 | - | 4 | motif\_sequence | short\_function |
|  | organism | 824 | - | 4 | motif\_sequence | short\_function |
|  | organism | 348 | + | 4 | motif\_sequence | short\_function |
|  | organism | 922 | - | 4 | motif\_sequence | short\_function |
|  | organism | 1293 | + | 4 | motif\_sequence | short\_function |
|  | organism | 371 | + | 4 | motif\_sequence | short\_function |
|  | organism | 210 | + | 4 | motif\_sequence | short\_function |
|  | organism | 111 | - | 4 | motif\_sequence | short\_function |

>006277   
+ TGATGAATAA AACAGAATGA TAATTAACAA ACAGAAACAG TTGTGTATAA TTTAAAAATG AATGGAGGGA   
  
  
+ GGTGGTGGTG TTTATAAGCG TTGTAAAAAT GCAGAGGTGA GGAGAGTGAT GAGTGAGTTT TGTTTTCTTC   
  
  
+ TATTCTTTTT GTGAAAAGAG AGTGAATTGG TCGTGGAACG CGGTTTGTTC GGAATCTCAG GGAAAAGACA   
  
  
+ TGTACGCGGA ACGCAGCATT AGACAGAGAA AAAAGAGAGA AGCAGACAAA GCCTTGTATT CAGAGAAGGA   
  
  
+ AGAAAGCTAA AAGTACTCAA TTATCTCGTG GGGAGCATAC AATAAAACCA CCACAATTAT ATTATACATG   
  
  
+ TACATATTTA TGTGTTATAT ATGTACGTTT ATTTATTTAT TTTATGTAAA TTGATATTTG TTATGAAATT   
  
  
+ ATATGCGGAT GTGGTTACGG TTTGTTTGAA TGTGTTACAC GCAAGGATTC AAAAGGTGCT AGGCACTGGT   
  
  
+ TGGGCGACGG ATATGTGTCT AGGTGGGAGT TTAGGCAGAG CCTAGGTGGG GGTTTAGGCA GTTTTTTTTT   
  
  
+ TTTAATTTTA ATTGAATTTA TTATATAACA TATAAATAAC TGCCTACTTG TACTTAAAAA AATACATCAT   
  
  
+ TGTATTAAAA CGCTTGAATT GCAAAATAGA ATGATATATT GATTATAAAA TATTCAAACA TATTGAAAAT   
  
  
+ ATGAGAAACA ACATACAATG AGTGCTCATC CAAACATTCA AGAAATCTCG TACAATTTAA TGAAAAAATA   
  
  
+ AAATGCAAGA TGGAAGTTAT CTTTCTTAAG TAAGAATTTC GACCTAAGTG GGTGGACAGG TCTAGGTGGG   
  
  
+ CGCCTAGGTA GGCTAGGTGC CCTTTCTTAA TTTTAAATGC TTAGGGACTA ATCATGTCGG TAACCAGCCG   
  
  
+ CCTAGCACTA GGCAGAGATT TTTAGAACAC TAGTTACATG CATTATTTGT AATGGTGACT TATTATTTGA   
  
  
+ ATGTCAAGCT TTTATGCATT ATCAATTGTG TTTTTATATC CCGCAAACTG AGAATTTAGC TTGAGTAGTT   
  
  
+ ATTTTAGTTT TATTCGTCAT CTAACAGTGT TGAGAAATTT GGAAAAATAA CTAAATTTTA AATCCCGTAT   
  
  
+ AGAATGTTAA CGATCATTTT AATTTTATGT AATTATGTAA TTATAATCAA TACTTGATGT AAAAGTATTA   
  
  
+ ATATACCCTT AATATTAGGA TTTCTGACAA CAACCAAAAC ACAACCTTTA AACTACCTTA AAATGAGGAA   
  
  
+ ATAATCTATA TCACTTTTTA GTTTTCATTA TTCTCTTCAA CTTTTGAACT ACTTTCTGCA ACCTTTCGGA   
  
  
+ TTTTTGCAAA ACGATACAAC AGCCTTATTG TTACCTTTGA CTGCACTTTC ATTCTTCAAT CACATCAATT   
  
  
+ TTAAAATCTT ATTGGAAGTA CCGAGATACC TAGCAATTTA ATCTCGAAGA CTTTGTTTTT TTTATTCTTT   
  
  
+ TTTGTTTCAA TTCAGGCTTT CATTATGGT  

- ACTACTTATT TTGTCTTACT ATTAATTGTT TGTCTTTGTC AACACATATT AAATTTTTAC TTACCTCCCT   
  
  
- CCACCACCAC AAATATTCGC AACATTTTTA CGTCTCCACT CCTCTCACTA CTCACTCAAA ACAAAAGAAG   
  
  
- ATAAGAAAAA CACTTTTCTC TCACTTAACC AGCACCTTGC GCCAAACAAG CCTTAGAGTC CCTTTTCTGT   
  
  
- ACATGCGCCT TGCGTCGTAA TCTGTCTCTT TTTTCTCTCT TCGTCTGTTT CGGAACATAA GTCTCTTCCT   
  
  
- TCTTTCGATT TTCATGAGTT AATAGAGCAC CCCTCGTATG TTATTTTGGT GGTGTTAATA TAATATGTAC   
  
  
- ATGTATAAAT ACACAATATA TACATGCAAA TAAATAAATA AAATACATTT AACTATAAAC AATACTTTAA   
  
  
- TATACGCCTA CACCAATGCC AAACAAACTT ACACAATGTG CGTTCCTAAG TTTTCCACGA TCCGTGACCA   
  
  
- ACCCGCTGCC TATACACAGA TCCACCCTCA AATCCGTCTC GGATCCACCC CCAAATCCGT CAAAAAAAAA   
  
  
- AAATTAAAAT TAACTTAAAT AATATATTGT ATATTTATTG ACGGATGAAC ATGAATTTTT TTATGTAGTA   
  
  
- ACATAATTTT GCGAACTTAA CGTTTTATCT TACTATATAA CTAATATTTT ATAAGTTTGT ATAACTTTTA   
  
  
- TACTCTTTGT TGTATGTTAC TCACGAGTAG GTTTGTAAGT TCTTTAGAGC ATGTTAAATT ACTTTTTTAT   
  
  
- TTTACGTTCT ACCTTCAATA GAAAGAATTC ATTCTTAAAG CTGGATTCAC CCACCTGTCC AGATCCACCC   
  
  
- GCGGATCCAT CCGATCCACG GGAAAGAATT AAAATTTACG AATCCCTGAT TAGTACAGCC ATTGGTCGGC   
  
  
- GGATCGTGAT CCGTCTCTAA AAATCTTGTG ATCAATGTAC GTAATAAACA TTACCACTGA ATAATAAACT   
  
  
- TACAGTTCGA AAATACGTAA TAGTTAACAC AAAAATATAG GGCGTTTGAC TCTTAAATCG AACTCATCAA   
  
  
- TAAAATCAAA ATAAGCAGTA GATTGTCACA ACTCTTTAAA CCTTTTTATT GATTTAAAAT TTAGGGCATA   
  
  
- TCTTACAATT GCTAGTAAAA TTAAAATACA TTAATACATT AATATTAGTT ATGAACTACA TTTTCATAAT   
  
  
- TATATGGGAA TTATAATCCT AAAGACTGTT GTTGGTTTTG TGTTGGAAAT TTGATGGAAT TTTACTCCTT   
  
  
- TATTAGATAT AGTGAAAAAT CAAAAGTAAT AAGAGAAGTT GAAAACTTGA TGAAAGACGT TGGAAAGCCT   
  
  
- AAAAACGTTT TGCTATGTTG TCGGAATAAC AATGGAAACT GACGTGAAAG TAAGAAGTTA GTGTAGTTAA   
  
  
- AATTTTAGAA TAACCTTCAT GGCTCTATGG ATCGTTAAAT TAGAGCTTCT GAAACAAAAA AAATAAGAAA   
  
  
- AAACAAAGTT AAGTCCGAAA GTAATACCA

+     AE-box

| Site Name | Organism | Position | Strand | Matrix score. | sequence | function |
| --- | --- | --- | --- | --- | --- | --- |
| AE-box | Arabidopsis thaliana | 704 | + | 8 | AGAAACAA | part of a module for light response |

>006277   
+ TGATGAATAA AACAGAATGA TAATTAACAA ACAGAAACAG TTGTGTATAA TTTAAAAATG AATGGAGGGA   
  
  
+ GGTGGTGGTG TTTATAAGCG TTGTAAAAAT GCAGAGGTGA GGAGAGTGAT GAGTGAGTTT TGTTTTCTTC   
  
  
+ TATTCTTTTT GTGAAAAGAG AGTGAATTGG TCGTGGAACG CGGTTTGTTC GGAATCTCAG GGAAAAGACA   
  
  
+ TGTACGCGGA ACGCAGCATT AGACAGAGAA AAAAGAGAGA AGCAGACAAA GCCTTGTATT CAGAGAAGGA   
  
  
+ AGAAAGCTAA AAGTACTCAA TTATCTCGTG GGGAGCATAC AATAAAACCA CCACAATTAT ATTATACATG   
  
  
+ TACATATTTA TGTGTTATAT ATGTACGTTT ATTTATTTAT TTTATGTAAA TTGATATTTG TTATGAAATT   
  
  
+ ATATGCGGAT GTGGTTACGG TTTGTTTGAA TGTGTTACAC GCAAGGATTC AAAAGGTGCT AGGCACTGGT   
  
  
+ TGGGCGACGG ATATGTGTCT AGGTGGGAGT TTAGGCAGAG CCTAGGTGGG GGTTTAGGCA GTTTTTTTTT   
  
  
+ TTTAATTTTA ATTGAATTTA TTATATAACA TATAAATAAC TGCCTACTTG TACTTAAAAA AATACATCAT   
  
  
+ TGTATTAAAA CGCTTGAATT GCAAAATAGA ATGATATATT GATTATAAAA TATTCAAACA TATTGAAAAT   
  
  
+ ATGAGAAACA ACATACAATG AGTGCTCATC CAAACATTCA AGAAATCTCG TACAATTTAA TGAAAAAATA   
  
  
+ AAATGCAAGA TGGAAGTTAT CTTTCTTAAG TAAGAATTTC GACCTAAGTG GGTGGACAGG TCTAGGTGGG   
  
  
+ CGCCTAGGTA GGCTAGGTGC CCTTTCTTAA TTTTAAATGC TTAGGGACTA ATCATGTCGG TAACCAGCCG   
  
  
+ CCTAGCACTA GGCAGAGATT TTTAGAACAC TAGTTACATG CATTATTTGT AATGGTGACT TATTATTTGA   
  
  
+ ATGTCAAGCT TTTATGCATT ATCAATTGTG TTTTTATATC CCGCAAACTG AGAATTTAGC TTGAGTAGTT   
  
  
+ ATTTTAGTTT TATTCGTCAT CTAACAGTGT TGAGAAATTT GGAAAAATAA CTAAATTTTA AATCCCGTAT   
  
  
+ AGAATGTTAA CGATCATTTT AATTTTATGT AATTATGTAA TTATAATCAA TACTTGATGT AAAAGTATTA   
  
  
+ ATATACCCTT AATATTAGGA TTTCTGACAA CAACCAAAAC ACAACCTTTA AACTACCTTA AAATGAGGAA   
  
  
+ ATAATCTATA TCACTTTTTA GTTTTCATTA TTCTCTTCAA CTTTTGAACT ACTTTCTGCA ACCTTTCGGA   
  
  
+ TTTTTGCAAA ACGATACAAC AGCCTTATTG TTACCTTTGA CTGCACTTTC ATTCTTCAAT CACATCAATT   
  
  
+ TTAAAATCTT ATTGGAAGTA CCGAGATACC TAGCAATTTA ATCTCGAAGA CTTTGTTTTT TTTATTCTTT   
  
  
+ TTTGTTTCAA TTCAGGCTTT CATTATGGT  

- ACTACTTATT TTGTCTTACT ATTAATTGTT TGTCTTTGTC AACACATATT AAATTTTTAC TTACCTCCCT   
  
  
- CCACCACCAC AAATATTCGC AACATTTTTA CGTCTCCACT CCTCTCACTA CTCACTCAAA ACAAAAGAAG   
  
  
- ATAAGAAAAA CACTTTTCTC TCACTTAACC AGCACCTTGC GCCAAACAAG CCTTAGAGTC CCTTTTCTGT   
  
  
- ACATGCGCCT TGCGTCGTAA TCTGTCTCTT TTTTCTCTCT TCGTCTGTTT CGGAACATAA GTCTCTTCCT   
  
  
- TCTTTCGATT TTCATGAGTT AATAGAGCAC CCCTCGTATG TTATTTTGGT GGTGTTAATA TAATATGTAC   
  
  
- ATGTATAAAT ACACAATATA TACATGCAAA TAAATAAATA AAATACATTT AACTATAAAC AATACTTTAA   
  
  
- TATACGCCTA CACCAATGCC AAACAAACTT ACACAATGTG CGTTCCTAAG TTTTCCACGA TCCGTGACCA   
  
  
- ACCCGCTGCC TATACACAGA TCCACCCTCA AATCCGTCTC GGATCCACCC CCAAATCCGT CAAAAAAAAA   
  
  
- AAATTAAAAT TAACTTAAAT AATATATTGT ATATTTATTG ACGGATGAAC ATGAATTTTT TTATGTAGTA   
  
  
- ACATAATTTT GCGAACTTAA CGTTTTATCT TACTATATAA CTAATATTTT ATAAGTTTGT ATAACTTTTA   
  
  
- TACTCTTTGT TGTATGTTAC TCACGAGTAG GTTTGTAAGT TCTTTAGAGC ATGTTAAATT ACTTTTTTAT   
  
  
- TTTACGTTCT ACCTTCAATA GAAAGAATTC ATTCTTAAAG CTGGATTCAC CCACCTGTCC AGATCCACCC   
  
  
- GCGGATCCAT CCGATCCACG GGAAAGAATT AAAATTTACG AATCCCTGAT TAGTACAGCC ATTGGTCGGC   
  
  
- GGATCGTGAT CCGTCTCTAA AAATCTTGTG ATCAATGTAC GTAATAAACA TTACCACTGA ATAATAAACT   
  
  
- TACAGTTCGA AAATACGTAA TAGTTAACAC AAAAATATAG GGCGTTTGAC TCTTAAATCG AACTCATCAA   
  
  
- TAAAATCAAA ATAAGCAGTA GATTGTCACA ACTCTTTAAA CCTTTTTATT GATTTAAAAT TTAGGGCATA   
  
  
- TCTTACAATT GCTAGTAAAA TTAAAATACA TTAATACATT AATATTAGTT ATGAACTACA TTTTCATAAT   
  
  
- TATATGGGAA TTATAATCCT AAAGACTGTT GTTGGTTTTG TGTTGGAAAT TTGATGGAAT TTTACTCCTT   
  
  
- TATTAGATAT AGTGAAAAAT CAAAAGTAAT AAGAGAAGTT GAAAACTTGA TGAAAGACGT TGGAAAGCCT   
  
  
- AAAAACGTTT TGCTATGTTG TCGGAATAAC AATGGAAACT GACGTGAAAG TAAGAAGTTA GTGTAGTTAA   
  
  
- AATTTTAGAA TAACCTTCAT GGCTCTATGG ATCGTTAAAT TAGAGCTTCT GAAACAAAAA AAATAAGAAA   
  
  
- AAACAAAGTT AAGTCCGAAA GTAATACCA

+     ARE

| Site Name | Organism | Position | Strand | Matrix score. | sequence | function |
| --- | --- | --- | --- | --- | --- | --- |
| ARE | Zea mays | 325 | + | 6 | AAACCA | cis-acting regulatory element essential for the anaerobic induction |

>006277   
+ TGATGAATAA AACAGAATGA TAATTAACAA ACAGAAACAG TTGTGTATAA TTTAAAAATG AATGGAGGGA   
  
  
+ GGTGGTGGTG TTTATAAGCG TTGTAAAAAT GCAGAGGTGA GGAGAGTGAT GAGTGAGTTT TGTTTTCTTC   
  
  
+ TATTCTTTTT GTGAAAAGAG AGTGAATTGG TCGTGGAACG CGGTTTGTTC GGAATCTCAG GGAAAAGACA   
  
  
+ TGTACGCGGA ACGCAGCATT AGACAGAGAA AAAAGAGAGA AGCAGACAAA GCCTTGTATT CAGAGAAGGA   
  
  
+ AGAAAGCTAA AAGTACTCAA TTATCTCGTG GGGAGCATAC AATAAAACCA CCACAATTAT ATTATACATG   
  
  
+ TACATATTTA TGTGTTATAT ATGTACGTTT ATTTATTTAT TTTATGTAAA TTGATATTTG TTATGAAATT   
  
  
+ ATATGCGGAT GTGGTTACGG TTTGTTTGAA TGTGTTACAC GCAAGGATTC AAAAGGTGCT AGGCACTGGT   
  
  
+ TGGGCGACGG ATATGTGTCT AGGTGGGAGT TTAGGCAGAG CCTAGGTGGG GGTTTAGGCA GTTTTTTTTT   
  
  
+ TTTAATTTTA ATTGAATTTA TTATATAACA TATAAATAAC TGCCTACTTG TACTTAAAAA AATACATCAT   
  
  
+ TGTATTAAAA CGCTTGAATT GCAAAATAGA ATGATATATT GATTATAAAA TATTCAAACA TATTGAAAAT   
  
  
+ ATGAGAAACA ACATACAATG AGTGCTCATC CAAACATTCA AGAAATCTCG TACAATTTAA TGAAAAAATA   
  
  
+ AAATGCAAGA TGGAAGTTAT CTTTCTTAAG TAAGAATTTC GACCTAAGTG GGTGGACAGG TCTAGGTGGG   
  
  
+ CGCCTAGGTA GGCTAGGTGC CCTTTCTTAA TTTTAAATGC TTAGGGACTA ATCATGTCGG TAACCAGCCG   
  
  
+ CCTAGCACTA GGCAGAGATT TTTAGAACAC TAGTTACATG CATTATTTGT AATGGTGACT TATTATTTGA   
  
  
+ ATGTCAAGCT TTTATGCATT ATCAATTGTG TTTTTATATC CCGCAAACTG AGAATTTAGC TTGAGTAGTT   
  
  
+ ATTTTAGTTT TATTCGTCAT CTAACAGTGT TGAGAAATTT GGAAAAATAA CTAAATTTTA AATCCCGTAT   
  
  
+ AGAATGTTAA CGATCATTTT AATTTTATGT AATTATGTAA TTATAATCAA TACTTGATGT AAAAGTATTA   
  
  
+ ATATACCCTT AATATTAGGA TTTCTGACAA CAACCAAAAC ACAACCTTTA AACTACCTTA AAATGAGGAA   
  
  
+ ATAATCTATA TCACTTTTTA GTTTTCATTA TTCTCTTCAA CTTTTGAACT ACTTTCTGCA ACCTTTCGGA   
  
  
+ TTTTTGCAAA ACGATACAAC AGCCTTATTG TTACCTTTGA CTGCACTTTC ATTCTTCAAT CACATCAATT   
  
  
+ TTAAAATCTT ATTGGAAGTA CCGAGATACC TAGCAATTTA ATCTCGAAGA CTTTGTTTTT TTTATTCTTT   
  
  
+ TTTGTTTCAA TTCAGGCTTT CATTATGGT  

- ACTACTTATT TTGTCTTACT ATTAATTGTT TGTCTTTGTC AACACATATT AAATTTTTAC TTACCTCCCT   
  
  
- CCACCACCAC AAATATTCGC AACATTTTTA CGTCTCCACT CCTCTCACTA CTCACTCAAA ACAAAAGAAG   
  
  
- ATAAGAAAAA CACTTTTCTC TCACTTAACC AGCACCTTGC GCCAAACAAG CCTTAGAGTC CCTTTTCTGT   
  
  
- ACATGCGCCT TGCGTCGTAA TCTGTCTCTT TTTTCTCTCT TCGTCTGTTT CGGAACATAA GTCTCTTCCT   
  
  
- TCTTTCGATT TTCATGAGTT AATAGAGCAC CCCTCGTATG TTATTTTGGT GGTGTTAATA TAATATGTAC   
  
  
- ATGTATAAAT ACACAATATA TACATGCAAA TAAATAAATA AAATACATTT AACTATAAAC AATACTTTAA   
  
  
- TATACGCCTA CACCAATGCC AAACAAACTT ACACAATGTG CGTTCCTAAG TTTTCCACGA TCCGTGACCA   
  
  
- ACCCGCTGCC TATACACAGA TCCACCCTCA AATCCGTCTC GGATCCACCC CCAAATCCGT CAAAAAAAAA   
  
  
- AAATTAAAAT TAACTTAAAT AATATATTGT ATATTTATTG ACGGATGAAC ATGAATTTTT TTATGTAGTA   
  
  
- ACATAATTTT GCGAACTTAA CGTTTTATCT TACTATATAA CTAATATTTT ATAAGTTTGT ATAACTTTTA   
  
  
- TACTCTTTGT TGTATGTTAC TCACGAGTAG GTTTGTAAGT TCTTTAGAGC ATGTTAAATT ACTTTTTTAT   
  
  
- TTTACGTTCT ACCTTCAATA GAAAGAATTC ATTCTTAAAG CTGGATTCAC CCACCTGTCC AGATCCACCC   
  
  
- GCGGATCCAT CCGATCCACG GGAAAGAATT AAAATTTACG AATCCCTGAT TAGTACAGCC ATTGGTCGGC   
  
  
- GGATCGTGAT CCGTCTCTAA AAATCTTGTG ATCAATGTAC GTAATAAACA TTACCACTGA ATAATAAACT   
  
  
- TACAGTTCGA AAATACGTAA TAGTTAACAC AAAAATATAG GGCGTTTGAC TCTTAAATCG AACTCATCAA   
  
  
- TAAAATCAAA ATAAGCAGTA GATTGTCACA ACTCTTTAAA CCTTTTTATT GATTTAAAAT TTAGGGCATA   
  
  
- TCTTACAATT GCTAGTAAAA TTAAAATACA TTAATACATT AATATTAGTT ATGAACTACA TTTTCATAAT   
  
  
- TATATGGGAA TTATAATCCT AAAGACTGTT GTTGGTTTTG TGTTGGAAAT TTGATGGAAT TTTACTCCTT   
  
  
- TATTAGATAT AGTGAAAAAT CAAAAGTAAT AAGAGAAGTT GAAAACTTGA TGAAAGACGT TGGAAAGCCT   
  
  
- AAAAACGTTT TGCTATGTTG TCGGAATAAC AATGGAAACT GACGTGAAAG TAAGAAGTTA GTGTAGTTAA   
  
  
- AATTTTAGAA TAACCTTCAT GGCTCTATGG ATCGTTAAAT TAGAGCTTCT GAAACAAAAA AAATAAGAAA   
  
  
- AAACAAAGTT AAGTCCGAAA GTAATACCA

+     AT~TATA-box

| Site Name | Organism | Position | Strand | Matrix score. | sequence | function |
| --- | --- | --- | --- | --- | --- | --- |
| AT~TATA-box | Arabidopsis thaliana | 582 | + | 6 | TATATA |  |
| AT~TATA-box | Arabidopsis thaliana | 366 | + | 6 | TATATA |  |

>006277   
+ TGATGAATAA AACAGAATGA TAATTAACAA ACAGAAACAG TTGTGTATAA TTTAAAAATG AATGGAGGGA   
  
  
+ GGTGGTGGTG TTTATAAGCG TTGTAAAAAT GCAGAGGTGA GGAGAGTGAT GAGTGAGTTT TGTTTTCTTC   
  
  
+ TATTCTTTTT GTGAAAAGAG AGTGAATTGG TCGTGGAACG CGGTTTGTTC GGAATCTCAG GGAAAAGACA   
  
  
+ TGTACGCGGA ACGCAGCATT AGACAGAGAA AAAAGAGAGA AGCAGACAAA GCCTTGTATT CAGAGAAGGA   
  
  
+ AGAAAGCTAA AAGTACTCAA TTATCTCGTG GGGAGCATAC AATAAAACCA CCACAATTAT ATTATACATG   
  
  
+ TACATATTTA TGTGTTATAT ATGTACGTTT ATTTATTTAT TTTATGTAAA TTGATATTTG TTATGAAATT   
  
  
+ ATATGCGGAT GTGGTTACGG TTTGTTTGAA TGTGTTACAC GCAAGGATTC AAAAGGTGCT AGGCACTGGT   
  
  
+ TGGGCGACGG ATATGTGTCT AGGTGGGAGT TTAGGCAGAG CCTAGGTGGG GGTTTAGGCA GTTTTTTTTT   
  
  
+ TTTAATTTTA ATTGAATTTA TTATATAACA TATAAATAAC TGCCTACTTG TACTTAAAAA AATACATCAT   
  
  
+ TGTATTAAAA CGCTTGAATT GCAAAATAGA ATGATATATT GATTATAAAA TATTCAAACA TATTGAAAAT   
  
  
+ ATGAGAAACA ACATACAATG AGTGCTCATC CAAACATTCA AGAAATCTCG TACAATTTAA TGAAAAAATA   
  
  
+ AAATGCAAGA TGGAAGTTAT CTTTCTTAAG TAAGAATTTC GACCTAAGTG GGTGGACAGG TCTAGGTGGG   
  
  
+ CGCCTAGGTA GGCTAGGTGC CCTTTCTTAA TTTTAAATGC TTAGGGACTA ATCATGTCGG TAACCAGCCG   
  
  
+ CCTAGCACTA GGCAGAGATT TTTAGAACAC TAGTTACATG CATTATTTGT AATGGTGACT TATTATTTGA   
  
  
+ ATGTCAAGCT TTTATGCATT ATCAATTGTG TTTTTATATC CCGCAAACTG AGAATTTAGC TTGAGTAGTT   
  
  
+ ATTTTAGTTT TATTCGTCAT CTAACAGTGT TGAGAAATTT GGAAAAATAA CTAAATTTTA AATCCCGTAT   
  
  
+ AGAATGTTAA CGATCATTTT AATTTTATGT AATTATGTAA TTATAATCAA TACTTGATGT AAAAGTATTA   
  
  
+ ATATACCCTT AATATTAGGA TTTCTGACAA CAACCAAAAC ACAACCTTTA AACTACCTTA AAATGAGGAA   
  
  
+ ATAATCTATA TCACTTTTTA GTTTTCATTA TTCTCTTCAA CTTTTGAACT ACTTTCTGCA ACCTTTCGGA   
  
  
+ TTTTTGCAAA ACGATACAAC AGCCTTATTG TTACCTTTGA CTGCACTTTC ATTCTTCAAT CACATCAATT   
  
  
+ TTAAAATCTT ATTGGAAGTA CCGAGATACC TAGCAATTTA ATCTCGAAGA CTTTGTTTTT TTTATTCTTT   
  
  
+ TTTGTTTCAA TTCAGGCTTT CATTATGGT  

- ACTACTTATT TTGTCTTACT ATTAATTGTT TGTCTTTGTC AACACATATT AAATTTTTAC TTACCTCCCT   
  
  
- CCACCACCAC AAATATTCGC AACATTTTTA CGTCTCCACT CCTCTCACTA CTCACTCAAA ACAAAAGAAG   
  
  
- ATAAGAAAAA CACTTTTCTC TCACTTAACC AGCACCTTGC GCCAAACAAG CCTTAGAGTC CCTTTTCTGT   
  
  
- ACATGCGCCT TGCGTCGTAA TCTGTCTCTT TTTTCTCTCT TCGTCTGTTT CGGAACATAA GTCTCTTCCT   
  
  
- TCTTTCGATT TTCATGAGTT AATAGAGCAC CCCTCGTATG TTATTTTGGT GGTGTTAATA TAATATGTAC   
  
  
- ATGTATAAAT ACACAATATA TACATGCAAA TAAATAAATA AAATACATTT AACTATAAAC AATACTTTAA   
  
  
- TATACGCCTA CACCAATGCC AAACAAACTT ACACAATGTG CGTTCCTAAG TTTTCCACGA TCCGTGACCA   
  
  
- ACCCGCTGCC TATACACAGA TCCACCCTCA AATCCGTCTC GGATCCACCC CCAAATCCGT CAAAAAAAAA   
  
  
- AAATTAAAAT TAACTTAAAT AATATATTGT ATATTTATTG ACGGATGAAC ATGAATTTTT TTATGTAGTA   
  
  
- ACATAATTTT GCGAACTTAA CGTTTTATCT TACTATATAA CTAATATTTT ATAAGTTTGT ATAACTTTTA   
  
  
- TACTCTTTGT TGTATGTTAC TCACGAGTAG GTTTGTAAGT TCTTTAGAGC ATGTTAAATT ACTTTTTTAT   
  
  
- TTTACGTTCT ACCTTCAATA GAAAGAATTC ATTCTTAAAG CTGGATTCAC CCACCTGTCC AGATCCACCC   
  
  
- GCGGATCCAT CCGATCCACG GGAAAGAATT AAAATTTACG AATCCCTGAT TAGTACAGCC ATTGGTCGGC   
  
  
- GGATCGTGAT CCGTCTCTAA AAATCTTGTG ATCAATGTAC GTAATAAACA TTACCACTGA ATAATAAACT   
  
  
- TACAGTTCGA AAATACGTAA TAGTTAACAC AAAAATATAG GGCGTTTGAC TCTTAAATCG AACTCATCAA   
  
  
- TAAAATCAAA ATAAGCAGTA GATTGTCACA ACTCTTTAAA CCTTTTTATT GATTTAAAAT TTAGGGCATA   
  
  
- TCTTACAATT GCTAGTAAAA TTAAAATACA TTAATACATT AATATTAGTT ATGAACTACA TTTTCATAAT   
  
  
- TATATGGGAA TTATAATCCT AAAGACTGTT GTTGGTTTTG TGTTGGAAAT TTGATGGAAT TTTACTCCTT   
  
  
- TATTAGATAT AGTGAAAAAT CAAAAGTAAT AAGAGAAGTT GAAAACTTGA TGAAAGACGT TGGAAAGCCT   
  
  
- AAAAACGTTT TGCTATGTTG TCGGAATAAC AATGGAAACT GACGTGAAAG TAAGAAGTTA GTGTAGTTAA   
  
  
- AATTTTAGAA TAACCTTCAT GGCTCTATGG ATCGTTAAAT TAGAGCTTCT GAAACAAAAA AAATAAGAAA   
  
  
- AAACAAAGTT AAGTCCGAAA GTAATACCA

+     Box 4

| Site Name | Organism | Position | Strand | Matrix score. | sequence | function |
| --- | --- | --- | --- | --- | --- | --- |
| Box 4 | Petroselinum crispum | 1187 | - | 6 | ATTAAT | part of a conserved DNA module involved in light responsiveness |

>006277   
+ TGATGAATAA AACAGAATGA TAATTAACAA ACAGAAACAG TTGTGTATAA TTTAAAAATG AATGGAGGGA   
  
  
+ GGTGGTGGTG TTTATAAGCG TTGTAAAAAT GCAGAGGTGA GGAGAGTGAT GAGTGAGTTT TGTTTTCTTC   
  
  
+ TATTCTTTTT GTGAAAAGAG AGTGAATTGG TCGTGGAACG CGGTTTGTTC GGAATCTCAG GGAAAAGACA   
  
  
+ TGTACGCGGA ACGCAGCATT AGACAGAGAA AAAAGAGAGA AGCAGACAAA GCCTTGTATT CAGAGAAGGA   
  
  
+ AGAAAGCTAA AAGTACTCAA TTATCTCGTG GGGAGCATAC AATAAAACCA CCACAATTAT ATTATACATG   
  
  
+ TACATATTTA TGTGTTATAT ATGTACGTTT ATTTATTTAT TTTATGTAAA TTGATATTTG TTATGAAATT   
  
  
+ ATATGCGGAT GTGGTTACGG TTTGTTTGAA TGTGTTACAC GCAAGGATTC AAAAGGTGCT AGGCACTGGT   
  
  
+ TGGGCGACGG ATATGTGTCT AGGTGGGAGT TTAGGCAGAG CCTAGGTGGG GGTTTAGGCA GTTTTTTTTT   
  
  
+ TTTAATTTTA ATTGAATTTA TTATATAACA TATAAATAAC TGCCTACTTG TACTTAAAAA AATACATCAT   
  
  
+ TGTATTAAAA CGCTTGAATT GCAAAATAGA ATGATATATT GATTATAAAA TATTCAAACA TATTGAAAAT   
  
  
+ ATGAGAAACA ACATACAATG AGTGCTCATC CAAACATTCA AGAAATCTCG TACAATTTAA TGAAAAAATA   
  
  
+ AAATGCAAGA TGGAAGTTAT CTTTCTTAAG TAAGAATTTC GACCTAAGTG GGTGGACAGG TCTAGGTGGG   
  
  
+ CGCCTAGGTA GGCTAGGTGC CCTTTCTTAA TTTTAAATGC TTAGGGACTA ATCATGTCGG TAACCAGCCG   
  
  
+ CCTAGCACTA GGCAGAGATT TTTAGAACAC TAGTTACATG CATTATTTGT AATGGTGACT TATTATTTGA   
  
  
+ ATGTCAAGCT TTTATGCATT ATCAATTGTG TTTTTATATC CCGCAAACTG AGAATTTAGC TTGAGTAGTT   
  
  
+ ATTTTAGTTT TATTCGTCAT CTAACAGTGT TGAGAAATTT GGAAAAATAA CTAAATTTTA AATCCCGTAT   
  
  
+ AGAATGTTAA CGATCATTTT AATTTTATGT AATTATGTAA TTATAATCAA TACTTGATGT AAAAGTATTA   
  
  
+ ATATACCCTT AATATTAGGA TTTCTGACAA CAACCAAAAC ACAACCTTTA AACTACCTTA AAATGAGGAA   
  
  
+ ATAATCTATA TCACTTTTTA GTTTTCATTA TTCTCTTCAA CTTTTGAACT ACTTTCTGCA ACCTTTCGGA   
  
  
+ TTTTTGCAAA ACGATACAAC AGCCTTATTG TTACCTTTGA CTGCACTTTC ATTCTTCAAT CACATCAATT   
  
  
+ TTAAAATCTT ATTGGAAGTA CCGAGATACC TAGCAATTTA ATCTCGAAGA CTTTGTTTTT TTTATTCTTT   
  
  
+ TTTGTTTCAA TTCAGGCTTT CATTATGGT  

- ACTACTTATT TTGTCTTACT ATTAATTGTT TGTCTTTGTC AACACATATT AAATTTTTAC TTACCTCCCT   
  
  
- CCACCACCAC AAATATTCGC AACATTTTTA CGTCTCCACT CCTCTCACTA CTCACTCAAA ACAAAAGAAG   
  
  
- ATAAGAAAAA CACTTTTCTC TCACTTAACC AGCACCTTGC GCCAAACAAG CCTTAGAGTC CCTTTTCTGT   
  
  
- ACATGCGCCT TGCGTCGTAA TCTGTCTCTT TTTTCTCTCT TCGTCTGTTT CGGAACATAA GTCTCTTCCT   
  
  
- TCTTTCGATT TTCATGAGTT AATAGAGCAC CCCTCGTATG TTATTTTGGT GGTGTTAATA TAATATGTAC   
  
  
- ATGTATAAAT ACACAATATA TACATGCAAA TAAATAAATA AAATACATTT AACTATAAAC AATACTTTAA   
  
  
- TATACGCCTA CACCAATGCC AAACAAACTT ACACAATGTG CGTTCCTAAG TTTTCCACGA TCCGTGACCA   
  
  
- ACCCGCTGCC TATACACAGA TCCACCCTCA AATCCGTCTC GGATCCACCC CCAAATCCGT CAAAAAAAAA   
  
  
- AAATTAAAAT TAACTTAAAT AATATATTGT ATATTTATTG ACGGATGAAC ATGAATTTTT TTATGTAGTA   
  
  
- ACATAATTTT GCGAACTTAA CGTTTTATCT TACTATATAA CTAATATTTT ATAAGTTTGT ATAACTTTTA   
  
  
- TACTCTTTGT TGTATGTTAC TCACGAGTAG GTTTGTAAGT TCTTTAGAGC ATGTTAAATT ACTTTTTTAT   
  
  
- TTTACGTTCT ACCTTCAATA GAAAGAATTC ATTCTTAAAG CTGGATTCAC CCACCTGTCC AGATCCACCC   
  
  
- GCGGATCCAT CCGATCCACG GGAAAGAATT AAAATTTACG AATCCCTGAT TAGTACAGCC ATTGGTCGGC   
  
  
- GGATCGTGAT CCGTCTCTAA AAATCTTGTG ATCAATGTAC GTAATAAACA TTACCACTGA ATAATAAACT   
  
  
- TACAGTTCGA AAATACGTAA TAGTTAACAC AAAAATATAG GGCGTTTGAC TCTTAAATCG AACTCATCAA   
  
  
- TAAAATCAAA ATAAGCAGTA GATTGTCACA ACTCTTTAAA CCTTTTTATT GATTTAAAAT TTAGGGCATA   
  
  
- TCTTACAATT GCTAGTAAAA TTAAAATACA TTAATACATT AATATTAGTT ATGAACTACA TTTTCATAAT   
  
  
- TATATGGGAA TTATAATCCT AAAGACTGTT GTTGGTTTTG TGTTGGAAAT TTGATGGAAT TTTACTCCTT   
  
  
- TATTAGATAT AGTGAAAAAT CAAAAGTAAT AAGAGAAGTT GAAAACTTGA TGAAAGACGT TGGAAAGCCT   
  
  
- AAAAACGTTT TGCTATGTTG TCGGAATAAC AATGGAAACT GACGTGAAAG TAAGAAGTTA GTGTAGTTAA   
  
  
- AATTTTAGAA TAACCTTCAT GGCTCTATGG ATCGTTAAAT TAGAGCTTCT GAAACAAAAA AAATAAGAAA   
  
  
- AAACAAAGTT AAGTCCGAAA GTAATACCA

+     CAAT-box

| Site Name | Organism | Position | Strand | Matrix score. | sequence | function |
| --- | --- | --- | --- | --- | --- | --- |
| CAAT-box | Nicotiana glutinosa | 1478 | + | 4 | CAAT |  |
| CAAT-box | Arabidopsis thaliana | 1411 | - | 5 | CCAAT | common cis-acting element in promoter and enhancer regions |
| CAAT-box | Nicotiana glutinosa | 1434 | + | 4 | CAAT |  |
| CAAT-box | Nicotiana glutinosa | 1387 | + | 4 | CAAT |  |
| CAAT-box | Nicotiana glutinosa | 1396 | + | 4 | CAAT |  |
| CAAT-box | Nicotiana glutinosa | 1005 | - | 4 | CAAT |  |
| CAAT-box | Pisum sativum | 1087 | - | 5 | CAAAT | common cis-acting element in promoter and enhancer regions |
| CAAT-box | Pisum sativum | 975 | - | 5 | CAAAT | common cis-acting element in promoter and enhancer regions |
| CAAT-box | Nicotiana glutinosa | 1003 | + | 4 | CAAT |  |
| CAAT-box | Nicotiana glutinosa | 1168 | + | 4 | CAAT |  |
| CAAT-box | Nicotiana glutinosa | 1357 | - | 4 | CAAT |  |
| CAAT-box | Pisum sativum | 955 | - | 5 | CAAAT | common cis-acting element in promoter and enhancer regions |
| CAAT-box | Nicotiana glutinosa | 320 | + | 4 | CAAT |  |
| CAAT-box | Nicotiana glutinosa | 753 | + | 4 | CAAT |  |
| CAAT-box | Nicotiana glutinosa | 716 | + | 4 | CAAT |  |
| CAAT-box | Nicotiana glutinosa | 692 | - | 4 | CAAT |  |
| CAAT-box | Nicotiana glutinosa | 648 | - | 4 | CAAT |  |
| CAAT-box | Nicotiana glutinosa | 668 | - | 4 | CAAT |  |
| CAAT-box | Arabidopsis thaliana | 166 | - | 5 | CCAAT | common cis-acting element in promoter and enhancer regions |
| CAAT-box | Pisum sativum | 406 | - | 5 | CAAAT | common cis-acting element in promoter and enhancer regions |
| CAAT-box | Nicotiana glutinosa | 400 | - | 4 | CAAT |  |
| CAAT-box | Nicotiana glutinosa | 571 | - | 4 | CAAT |  |
| CAAT-box | Nicotiana glutinosa | 298 | + | 4 | CAAT |  |
| CAAT-box | Nicotiana glutinosa | 629 | - | 4 | CAAT |  |
| CAAT-box | Nicotiana glutinosa | 334 | + | 4 | CAAT |  |

>006277   
+ TGATGAATAA AACAGAATGA TAATTAACAA ACAGAAACAG TTGTGTATAA TTTAAAAATG AATGGAGGGA   
  
  
+ GGTGGTGGTG TTTATAAGCG TTGTAAAAAT GCAGAGGTGA GGAGAGTGAT GAGTGAGTTT TGTTTTCTTC   
  
  
+ TATTCTTTTT GTGAAAAGAG AGTGAATTGG TCGTGGAACG CGGTTTGTTC GGAATCTCAG GGAAAAGACA   
  
  
+ TGTACGCGGA ACGCAGCATT AGACAGAGAA AAAAGAGAGA AGCAGACAAA GCCTTGTATT CAGAGAAGGA   
  
  
+ AGAAAGCTAA AAGTACTCAA TTATCTCGTG GGGAGCATAC AATAAAACCA CCACAATTAT ATTATACATG   
  
  
+ TACATATTTA TGTGTTATAT ATGTACGTTT ATTTATTTAT TTTATGTAAA TTGATATTTG TTATGAAATT   
  
  
+ ATATGCGGAT GTGGTTACGG TTTGTTTGAA TGTGTTACAC GCAAGGATTC AAAAGGTGCT AGGCACTGGT   
  
  
+ TGGGCGACGG ATATGTGTCT AGGTGGGAGT TTAGGCAGAG CCTAGGTGGG GGTTTAGGCA GTTTTTTTTT   
  
  
+ TTTAATTTTA ATTGAATTTA TTATATAACA TATAAATAAC TGCCTACTTG TACTTAAAAA AATACATCAT   
  
  
+ TGTATTAAAA CGCTTGAATT GCAAAATAGA ATGATATATT GATTATAAAA TATTCAAACA TATTGAAAAT   
  
  
+ ATGAGAAACA ACATACAATG AGTGCTCATC CAAACATTCA AGAAATCTCG TACAATTTAA TGAAAAAATA   
  
  
+ AAATGCAAGA TGGAAGTTAT CTTTCTTAAG TAAGAATTTC GACCTAAGTG GGTGGACAGG TCTAGGTGGG   
  
  
+ CGCCTAGGTA GGCTAGGTGC CCTTTCTTAA TTTTAAATGC TTAGGGACTA ATCATGTCGG TAACCAGCCG   
  
  
+ CCTAGCACTA GGCAGAGATT TTTAGAACAC TAGTTACATG CATTATTTGT AATGGTGACT TATTATTTGA   
  
  
+ ATGTCAAGCT TTTATGCATT ATCAATTGTG TTTTTATATC CCGCAAACTG AGAATTTAGC TTGAGTAGTT   
  
  
+ ATTTTAGTTT TATTCGTCAT CTAACAGTGT TGAGAAATTT GGAAAAATAA CTAAATTTTA AATCCCGTAT   
  
  
+ AGAATGTTAA CGATCATTTT AATTTTATGT AATTATGTAA TTATAATCAA TACTTGATGT AAAAGTATTA   
  
  
+ ATATACCCTT AATATTAGGA TTTCTGACAA CAACCAAAAC ACAACCTTTA AACTACCTTA AAATGAGGAA   
  
  
+ ATAATCTATA TCACTTTTTA GTTTTCATTA TTCTCTTCAA CTTTTGAACT ACTTTCTGCA ACCTTTCGGA   
  
  
+ TTTTTGCAAA ACGATACAAC AGCCTTATTG TTACCTTTGA CTGCACTTTC ATTCTTCAAT CACATCAATT   
  
  
+ TTAAAATCTT ATTGGAAGTA CCGAGATACC TAGCAATTTA ATCTCGAAGA CTTTGTTTTT TTTATTCTTT   
  
  
+ TTTGTTTCAA TTCAGGCTTT CATTATGGT  

- ACTACTTATT TTGTCTTACT ATTAATTGTT TGTCTTTGTC AACACATATT AAATTTTTAC TTACCTCCCT   
  
  
- CCACCACCAC AAATATTCGC AACATTTTTA CGTCTCCACT CCTCTCACTA CTCACTCAAA ACAAAAGAAG   
  
  
- ATAAGAAAAA CACTTTTCTC TCACTTAACC AGCACCTTGC GCCAAACAAG CCTTAGAGTC CCTTTTCTGT   
  
  
- ACATGCGCCT TGCGTCGTAA TCTGTCTCTT TTTTCTCTCT TCGTCTGTTT CGGAACATAA GTCTCTTCCT   
  
  
- TCTTTCGATT TTCATGAGTT AATAGAGCAC CCCTCGTATG TTATTTTGGT GGTGTTAATA TAATATGTAC   
  
  
- ATGTATAAAT ACACAATATA TACATGCAAA TAAATAAATA AAATACATTT AACTATAAAC AATACTTTAA   
  
  
- TATACGCCTA CACCAATGCC AAACAAACTT ACACAATGTG CGTTCCTAAG TTTTCCACGA TCCGTGACCA   
  
  
- ACCCGCTGCC TATACACAGA TCCACCCTCA AATCCGTCTC GGATCCACCC CCAAATCCGT CAAAAAAAAA   
  
  
- AAATTAAAAT TAACTTAAAT AATATATTGT ATATTTATTG ACGGATGAAC ATGAATTTTT TTATGTAGTA   
  
  
- ACATAATTTT GCGAACTTAA CGTTTTATCT TACTATATAA CTAATATTTT ATAAGTTTGT ATAACTTTTA   
  
  
- TACTCTTTGT TGTATGTTAC TCACGAGTAG GTTTGTAAGT TCTTTAGAGC ATGTTAAATT ACTTTTTTAT   
  
  
- TTTACGTTCT ACCTTCAATA GAAAGAATTC ATTCTTAAAG CTGGATTCAC CCACCTGTCC AGATCCACCC   
  
  
- GCGGATCCAT CCGATCCACG GGAAAGAATT AAAATTTACG AATCCCTGAT TAGTACAGCC ATTGGTCGGC   
  
  
- GGATCGTGAT CCGTCTCTAA AAATCTTGTG ATCAATGTAC GTAATAAACA TTACCACTGA ATAATAAACT   
  
  
- TACAGTTCGA AAATACGTAA TAGTTAACAC AAAAATATAG GGCGTTTGAC TCTTAAATCG AACTCATCAA   
  
  
- TAAAATCAAA ATAAGCAGTA GATTGTCACA ACTCTTTAAA CCTTTTTATT GATTTAAAAT TTAGGGCATA   
  
  
- TCTTACAATT GCTAGTAAAA TTAAAATACA TTAATACATT AATATTAGTT ATGAACTACA TTTTCATAAT   
  
  
- TATATGGGAA TTATAATCCT AAAGACTGTT GTTGGTTTTG TGTTGGAAAT TTGATGGAAT TTTACTCCTT   
  
  
- TATTAGATAT AGTGAAAAAT CAAAAGTAAT AAGAGAAGTT GAAAACTTGA TGAAAGACGT TGGAAAGCCT   
  
  
- AAAAACGTTT TGCTATGTTG TCGGAATAAC AATGGAAACT GACGTGAAAG TAAGAAGTTA GTGTAGTTAA   
  
  
- AATTTTAGAA TAACCTTCAT GGCTCTATGG ATCGTTAAAT TAGAGCTTCT GAAACAAAAA AAATAAGAAA   
  
  
- AAACAAAGTT AAGTCCGAAA GTAATACCA

+     CGTCA-motif

| Site Name | Organism | Position | Strand | Matrix score. | sequence | function |
| --- | --- | --- | --- | --- | --- | --- |
| CGTCA-motif | Hordeum vulgare | 1065 | + | 5 | CGTCA | cis-acting regulatory element involved in the MeJA-responsiveness |

>006277   
+ TGATGAATAA AACAGAATGA TAATTAACAA ACAGAAACAG TTGTGTATAA TTTAAAAATG AATGGAGGGA   
  
  
+ GGTGGTGGTG TTTATAAGCG TTGTAAAAAT GCAGAGGTGA GGAGAGTGAT GAGTGAGTTT TGTTTTCTTC   
  
  
+ TATTCTTTTT GTGAAAAGAG AGTGAATTGG TCGTGGAACG CGGTTTGTTC GGAATCTCAG GGAAAAGACA   
  
  
+ TGTACGCGGA ACGCAGCATT AGACAGAGAA AAAAGAGAGA AGCAGACAAA GCCTTGTATT CAGAGAAGGA   
  
  
+ AGAAAGCTAA AAGTACTCAA TTATCTCGTG GGGAGCATAC AATAAAACCA CCACAATTAT ATTATACATG   
  
  
+ TACATATTTA TGTGTTATAT ATGTACGTTT ATTTATTTAT TTTATGTAAA TTGATATTTG TTATGAAATT   
  
  
+ ATATGCGGAT GTGGTTACGG TTTGTTTGAA TGTGTTACAC GCAAGGATTC AAAAGGTGCT AGGCACTGGT   
  
  
+ TGGGCGACGG ATATGTGTCT AGGTGGGAGT TTAGGCAGAG CCTAGGTGGG GGTTTAGGCA GTTTTTTTTT   
  
  
+ TTTAATTTTA ATTGAATTTA TTATATAACA TATAAATAAC TGCCTACTTG TACTTAAAAA AATACATCAT   
  
  
+ TGTATTAAAA CGCTTGAATT GCAAAATAGA ATGATATATT GATTATAAAA TATTCAAACA TATTGAAAAT   
  
  
+ ATGAGAAACA ACATACAATG AGTGCTCATC CAAACATTCA AGAAATCTCG TACAATTTAA TGAAAAAATA   
  
  
+ AAATGCAAGA TGGAAGTTAT CTTTCTTAAG TAAGAATTTC GACCTAAGTG GGTGGACAGG TCTAGGTGGG   
  
  
+ CGCCTAGGTA GGCTAGGTGC CCTTTCTTAA TTTTAAATGC TTAGGGACTA ATCATGTCGG TAACCAGCCG   
  
  
+ CCTAGCACTA GGCAGAGATT TTTAGAACAC TAGTTACATG CATTATTTGT AATGGTGACT TATTATTTGA   
  
  
+ ATGTCAAGCT TTTATGCATT ATCAATTGTG TTTTTATATC CCGCAAACTG AGAATTTAGC TTGAGTAGTT   
  
  
+ ATTTTAGTTT TATTCGTCAT CTAACAGTGT TGAGAAATTT GGAAAAATAA CTAAATTTTA AATCCCGTAT   
  
  
+ AGAATGTTAA CGATCATTTT AATTTTATGT AATTATGTAA TTATAATCAA TACTTGATGT AAAAGTATTA   
  
  
+ ATATACCCTT AATATTAGGA TTTCTGACAA CAACCAAAAC ACAACCTTTA AACTACCTTA AAATGAGGAA   
  
  
+ ATAATCTATA TCACTTTTTA GTTTTCATTA TTCTCTTCAA CTTTTGAACT ACTTTCTGCA ACCTTTCGGA   
  
  
+ TTTTTGCAAA ACGATACAAC AGCCTTATTG TTACCTTTGA CTGCACTTTC ATTCTTCAAT CACATCAATT   
  
  
+ TTAAAATCTT ATTGGAAGTA CCGAGATACC TAGCAATTTA ATCTCGAAGA CTTTGTTTTT TTTATTCTTT   
  
  
+ TTTGTTTCAA TTCAGGCTTT CATTATGGT  

- ACTACTTATT TTGTCTTACT ATTAATTGTT TGTCTTTGTC AACACATATT AAATTTTTAC TTACCTCCCT   
  
  
- CCACCACCAC AAATATTCGC AACATTTTTA CGTCTCCACT CCTCTCACTA CTCACTCAAA ACAAAAGAAG   
  
  
- ATAAGAAAAA CACTTTTCTC TCACTTAACC AGCACCTTGC GCCAAACAAG CCTTAGAGTC CCTTTTCTGT   
  
  
- ACATGCGCCT TGCGTCGTAA TCTGTCTCTT TTTTCTCTCT TCGTCTGTTT CGGAACATAA GTCTCTTCCT   
  
  
- TCTTTCGATT TTCATGAGTT AATAGAGCAC CCCTCGTATG TTATTTTGGT GGTGTTAATA TAATATGTAC   
  
  
- ATGTATAAAT ACACAATATA TACATGCAAA TAAATAAATA AAATACATTT AACTATAAAC AATACTTTAA   
  
  
- TATACGCCTA CACCAATGCC AAACAAACTT ACACAATGTG CGTTCCTAAG TTTTCCACGA TCCGTGACCA   
  
  
- ACCCGCTGCC TATACACAGA TCCACCCTCA AATCCGTCTC GGATCCACCC CCAAATCCGT CAAAAAAAAA   
  
  
- AAATTAAAAT TAACTTAAAT AATATATTGT ATATTTATTG ACGGATGAAC ATGAATTTTT TTATGTAGTA   
  
  
- ACATAATTTT GCGAACTTAA CGTTTTATCT TACTATATAA CTAATATTTT ATAAGTTTGT ATAACTTTTA   
  
  
- TACTCTTTGT TGTATGTTAC TCACGAGTAG GTTTGTAAGT TCTTTAGAGC ATGTTAAATT ACTTTTTTAT   
  
  
- TTTACGTTCT ACCTTCAATA GAAAGAATTC ATTCTTAAAG CTGGATTCAC CCACCTGTCC AGATCCACCC   
  
  
- GCGGATCCAT CCGATCCACG GGAAAGAATT AAAATTTACG AATCCCTGAT TAGTACAGCC ATTGGTCGGC   
  
  
- GGATCGTGAT CCGTCTCTAA AAATCTTGTG ATCAATGTAC GTAATAAACA TTACCACTGA ATAATAAACT   
  
  
- TACAGTTCGA AAATACGTAA TAGTTAACAC AAAAATATAG GGCGTTTGAC TCTTAAATCG AACTCATCAA   
  
  
- TAAAATCAAA ATAAGCAGTA GATTGTCACA ACTCTTTAAA CCTTTTTATT GATTTAAAAT TTAGGGCATA   
  
  
- TCTTACAATT GCTAGTAAAA TTAAAATACA TTAATACATT AATATTAGTT ATGAACTACA TTTTCATAAT   
  
  
- TATATGGGAA TTATAATCCT AAAGACTGTT GTTGGTTTTG TGTTGGAAAT TTGATGGAAT TTTACTCCTT   
  
  
- TATTAGATAT AGTGAAAAAT CAAAAGTAAT AAGAGAAGTT GAAAACTTGA TGAAAGACGT TGGAAAGCCT   
  
  
- AAAAACGTTT TGCTATGTTG TCGGAATAAC AATGGAAACT GACGTGAAAG TAAGAAGTTA GTGTAGTTAA   
  
  
- AATTTTAGAA TAACCTTCAT GGCTCTATGG ATCGTTAAAT TAGAGCTTCT GAAACAAAAA AAATAAGAAA   
  
  
- AAACAAAGTT AAGTCCGAAA GTAATACCA

+     DRE

| Site Name | Organism | Position | Strand | Matrix score. | sequence | function |
| --- | --- | --- | --- | --- | --- | --- |
| DRE | Arabidopsis thaliana | 894 | - | 9 | TACCGACAT | "cis-acting element involved in dehydration, low-temp, salt stresses |

>006277   
+ TGATGAATAA AACAGAATGA TAATTAACAA ACAGAAACAG TTGTGTATAA TTTAAAAATG AATGGAGGGA   
  
  
+ GGTGGTGGTG TTTATAAGCG TTGTAAAAAT GCAGAGGTGA GGAGAGTGAT GAGTGAGTTT TGTTTTCTTC   
  
  
+ TATTCTTTTT GTGAAAAGAG AGTGAATTGG TCGTGGAACG CGGTTTGTTC GGAATCTCAG GGAAAAGACA   
  
  
+ TGTACGCGGA ACGCAGCATT AGACAGAGAA AAAAGAGAGA AGCAGACAAA GCCTTGTATT CAGAGAAGGA   
  
  
+ AGAAAGCTAA AAGTACTCAA TTATCTCGTG GGGAGCATAC AATAAAACCA CCACAATTAT ATTATACATG   
  
  
+ TACATATTTA TGTGTTATAT ATGTACGTTT ATTTATTTAT TTTATGTAAA TTGATATTTG TTATGAAATT   
  
  
+ ATATGCGGAT GTGGTTACGG TTTGTTTGAA TGTGTTACAC GCAAGGATTC AAAAGGTGCT AGGCACTGGT   
  
  
+ TGGGCGACGG ATATGTGTCT AGGTGGGAGT TTAGGCAGAG CCTAGGTGGG GGTTTAGGCA GTTTTTTTTT   
  
  
+ TTTAATTTTA ATTGAATTTA TTATATAACA TATAAATAAC TGCCTACTTG TACTTAAAAA AATACATCAT   
  
  
+ TGTATTAAAA CGCTTGAATT GCAAAATAGA ATGATATATT GATTATAAAA TATTCAAACA TATTGAAAAT   
  
  
+ ATGAGAAACA ACATACAATG AGTGCTCATC CAAACATTCA AGAAATCTCG TACAATTTAA TGAAAAAATA   
  
  
+ AAATGCAAGA TGGAAGTTAT CTTTCTTAAG TAAGAATTTC GACCTAAGTG GGTGGACAGG TCTAGGTGGG   
  
  
+ CGCCTAGGTA GGCTAGGTGC CCTTTCTTAA TTTTAAATGC TTAGGGACTA ATCATGTCGG TAACCAGCCG   
  
  
+ CCTAGCACTA GGCAGAGATT TTTAGAACAC TAGTTACATG CATTATTTGT AATGGTGACT TATTATTTGA   
  
  
+ ATGTCAAGCT TTTATGCATT ATCAATTGTG TTTTTATATC CCGCAAACTG AGAATTTAGC TTGAGTAGTT   
  
  
+ ATTTTAGTTT TATTCGTCAT CTAACAGTGT TGAGAAATTT GGAAAAATAA CTAAATTTTA AATCCCGTAT   
  
  
+ AGAATGTTAA CGATCATTTT AATTTTATGT AATTATGTAA TTATAATCAA TACTTGATGT AAAAGTATTA   
  
  
+ ATATACCCTT AATATTAGGA TTTCTGACAA CAACCAAAAC ACAACCTTTA AACTACCTTA AAATGAGGAA   
  
  
+ ATAATCTATA TCACTTTTTA GTTTTCATTA TTCTCTTCAA CTTTTGAACT ACTTTCTGCA ACCTTTCGGA   
  
  
+ TTTTTGCAAA ACGATACAAC AGCCTTATTG TTACCTTTGA CTGCACTTTC ATTCTTCAAT CACATCAATT   
  
  
+ TTAAAATCTT ATTGGAAGTA CCGAGATACC TAGCAATTTA ATCTCGAAGA CTTTGTTTTT TTTATTCTTT   
  
  
+ TTTGTTTCAA TTCAGGCTTT CATTATGGT  

- ACTACTTATT TTGTCTTACT ATTAATTGTT TGTCTTTGTC AACACATATT AAATTTTTAC TTACCTCCCT   
  
  
- CCACCACCAC AAATATTCGC AACATTTTTA CGTCTCCACT CCTCTCACTA CTCACTCAAA ACAAAAGAAG   
  
  
- ATAAGAAAAA CACTTTTCTC TCACTTAACC AGCACCTTGC GCCAAACAAG CCTTAGAGTC CCTTTTCTGT   
  
  
- ACATGCGCCT TGCGTCGTAA TCTGTCTCTT TTTTCTCTCT TCGTCTGTTT CGGAACATAA GTCTCTTCCT   
  
  
- TCTTTCGATT TTCATGAGTT AATAGAGCAC CCCTCGTATG TTATTTTGGT GGTGTTAATA TAATATGTAC   
  
  
- ATGTATAAAT ACACAATATA TACATGCAAA TAAATAAATA AAATACATTT AACTATAAAC AATACTTTAA   
  
  
- TATACGCCTA CACCAATGCC AAACAAACTT ACACAATGTG CGTTCCTAAG TTTTCCACGA TCCGTGACCA   
  
  
- ACCCGCTGCC TATACACAGA TCCACCCTCA AATCCGTCTC GGATCCACCC CCAAATCCGT CAAAAAAAAA   
  
  
- AAATTAAAAT TAACTTAAAT AATATATTGT ATATTTATTG ACGGATGAAC ATGAATTTTT TTATGTAGTA   
  
  
- ACATAATTTT GCGAACTTAA CGTTTTATCT TACTATATAA CTAATATTTT ATAAGTTTGT ATAACTTTTA   
  
  
- TACTCTTTGT TGTATGTTAC TCACGAGTAG GTTTGTAAGT TCTTTAGAGC ATGTTAAATT ACTTTTTTAT   
  
  
- TTTACGTTCT ACCTTCAATA GAAAGAATTC ATTCTTAAAG CTGGATTCAC CCACCTGTCC AGATCCACCC   
  
  
- GCGGATCCAT CCGATCCACG GGAAAGAATT AAAATTTACG AATCCCTGAT TAGTACAGCC ATTGGTCGGC   
  
  
- GGATCGTGAT CCGTCTCTAA AAATCTTGTG ATCAATGTAC GTAATAAACA TTACCACTGA ATAATAAACT   
  
  
- TACAGTTCGA AAATACGTAA TAGTTAACAC AAAAATATAG GGCGTTTGAC TCTTAAATCG AACTCATCAA   
  
  
- TAAAATCAAA ATAAGCAGTA GATTGTCACA ACTCTTTAAA CCTTTTTATT GATTTAAAAT TTAGGGCATA   
  
  
- TCTTACAATT GCTAGTAAAA TTAAAATACA TTAATACATT AATATTAGTT ATGAACTACA TTTTCATAAT   
  
  
- TATATGGGAA TTATAATCCT AAAGACTGTT GTTGGTTTTG TGTTGGAAAT TTGATGGAAT TTTACTCCTT   
  
  
- TATTAGATAT AGTGAAAAAT CAAAAGTAAT AAGAGAAGTT GAAAACTTGA TGAAAGACGT TGGAAAGCCT   
  
  
- AAAAACGTTT TGCTATGTTG TCGGAATAAC AATGGAAACT GACGTGAAAG TAAGAAGTTA GTGTAGTTAA   
  
  
- AATTTTAGAA TAACCTTCAT GGCTCTATGG ATCGTTAAAT TAGAGCTTCT GAAACAAAAA AAATAAGAAA   
  
  
- AAACAAAGTT AAGTCCGAAA GTAATACCA

+     DRE1

| Site Name | Organism | Position | Strand | Matrix score. | sequence | function |
| --- | --- | --- | --- | --- | --- | --- |
| DRE1 | Zea mays | 1420 | + | 7 | ACCGAGA |  |

>006277   
+ TGATGAATAA AACAGAATGA TAATTAACAA ACAGAAACAG TTGTGTATAA TTTAAAAATG AATGGAGGGA   
  
  
+ GGTGGTGGTG TTTATAAGCG TTGTAAAAAT GCAGAGGTGA GGAGAGTGAT GAGTGAGTTT TGTTTTCTTC   
  
  
+ TATTCTTTTT GTGAAAAGAG AGTGAATTGG TCGTGGAACG CGGTTTGTTC GGAATCTCAG GGAAAAGACA   
  
  
+ TGTACGCGGA ACGCAGCATT AGACAGAGAA AAAAGAGAGA AGCAGACAAA GCCTTGTATT CAGAGAAGGA   
  
  
+ AGAAAGCTAA AAGTACTCAA TTATCTCGTG GGGAGCATAC AATAAAACCA CCACAATTAT ATTATACATG   
  
  
+ TACATATTTA TGTGTTATAT ATGTACGTTT ATTTATTTAT TTTATGTAAA TTGATATTTG TTATGAAATT   
  
  
+ ATATGCGGAT GTGGTTACGG TTTGTTTGAA TGTGTTACAC GCAAGGATTC AAAAGGTGCT AGGCACTGGT   
  
  
+ TGGGCGACGG ATATGTGTCT AGGTGGGAGT TTAGGCAGAG CCTAGGTGGG GGTTTAGGCA GTTTTTTTTT   
  
  
+ TTTAATTTTA ATTGAATTTA TTATATAACA TATAAATAAC TGCCTACTTG TACTTAAAAA AATACATCAT   
  
  
+ TGTATTAAAA CGCTTGAATT GCAAAATAGA ATGATATATT GATTATAAAA TATTCAAACA TATTGAAAAT   
  
  
+ ATGAGAAACA ACATACAATG AGTGCTCATC CAAACATTCA AGAAATCTCG TACAATTTAA TGAAAAAATA   
  
  
+ AAATGCAAGA TGGAAGTTAT CTTTCTTAAG TAAGAATTTC GACCTAAGTG GGTGGACAGG TCTAGGTGGG   
  
  
+ CGCCTAGGTA GGCTAGGTGC CCTTTCTTAA TTTTAAATGC TTAGGGACTA ATCATGTCGG TAACCAGCCG   
  
  
+ CCTAGCACTA GGCAGAGATT TTTAGAACAC TAGTTACATG CATTATTTGT AATGGTGACT TATTATTTGA   
  
  
+ ATGTCAAGCT TTTATGCATT ATCAATTGTG TTTTTATATC CCGCAAACTG AGAATTTAGC TTGAGTAGTT   
  
  
+ ATTTTAGTTT TATTCGTCAT CTAACAGTGT TGAGAAATTT GGAAAAATAA CTAAATTTTA AATCCCGTAT   
  
  
+ AGAATGTTAA CGATCATTTT AATTTTATGT AATTATGTAA TTATAATCAA TACTTGATGT AAAAGTATTA   
  
  
+ ATATACCCTT AATATTAGGA TTTCTGACAA CAACCAAAAC ACAACCTTTA AACTACCTTA AAATGAGGAA   
  
  
+ ATAATCTATA TCACTTTTTA GTTTTCATTA TTCTCTTCAA CTTTTGAACT ACTTTCTGCA ACCTTTCGGA   
  
  
+ TTTTTGCAAA ACGATACAAC AGCCTTATTG TTACCTTTGA CTGCACTTTC ATTCTTCAAT CACATCAATT   
  
  
+ TTAAAATCTT ATTGGAAGTA CCGAGATACC TAGCAATTTA ATCTCGAAGA CTTTGTTTTT TTTATTCTTT   
  
  
+ TTTGTTTCAA TTCAGGCTTT CATTATGGT  

- ACTACTTATT TTGTCTTACT ATTAATTGTT TGTCTTTGTC AACACATATT AAATTTTTAC TTACCTCCCT   
  
  
- CCACCACCAC AAATATTCGC AACATTTTTA CGTCTCCACT CCTCTCACTA CTCACTCAAA ACAAAAGAAG   
  
  
- ATAAGAAAAA CACTTTTCTC TCACTTAACC AGCACCTTGC GCCAAACAAG CCTTAGAGTC CCTTTTCTGT   
  
  
- ACATGCGCCT TGCGTCGTAA TCTGTCTCTT TTTTCTCTCT TCGTCTGTTT CGGAACATAA GTCTCTTCCT   
  
  
- TCTTTCGATT TTCATGAGTT AATAGAGCAC CCCTCGTATG TTATTTTGGT GGTGTTAATA TAATATGTAC   
  
  
- ATGTATAAAT ACACAATATA TACATGCAAA TAAATAAATA AAATACATTT AACTATAAAC AATACTTTAA   
  
  
- TATACGCCTA CACCAATGCC AAACAAACTT ACACAATGTG CGTTCCTAAG TTTTCCACGA TCCGTGACCA   
  
  
- ACCCGCTGCC TATACACAGA TCCACCCTCA AATCCGTCTC GGATCCACCC CCAAATCCGT CAAAAAAAAA   
  
  
- AAATTAAAAT TAACTTAAAT AATATATTGT ATATTTATTG ACGGATGAAC ATGAATTTTT TTATGTAGTA   
  
  
- ACATAATTTT GCGAACTTAA CGTTTTATCT TACTATATAA CTAATATTTT ATAAGTTTGT ATAACTTTTA   
  
  
- TACTCTTTGT TGTATGTTAC TCACGAGTAG GTTTGTAAGT TCTTTAGAGC ATGTTAAATT ACTTTTTTAT   
  
  
- TTTACGTTCT ACCTTCAATA GAAAGAATTC ATTCTTAAAG CTGGATTCAC CCACCTGTCC AGATCCACCC   
  
  
- GCGGATCCAT CCGATCCACG GGAAAGAATT AAAATTTACG AATCCCTGAT TAGTACAGCC ATTGGTCGGC   
  
  
- GGATCGTGAT CCGTCTCTAA AAATCTTGTG ATCAATGTAC GTAATAAACA TTACCACTGA ATAATAAACT   
  
  
- TACAGTTCGA AAATACGTAA TAGTTAACAC AAAAATATAG GGCGTTTGAC TCTTAAATCG AACTCATCAA   
  
  
- TAAAATCAAA ATAAGCAGTA GATTGTCACA ACTCTTTAAA CCTTTTTATT GATTTAAAAT TTAGGGCATA   
  
  
- TCTTACAATT GCTAGTAAAA TTAAAATACA TTAATACATT AATATTAGTT ATGAACTACA TTTTCATAAT   
  
  
- TATATGGGAA TTATAATCCT AAAGACTGTT GTTGGTTTTG TGTTGGAAAT TTGATGGAAT TTTACTCCTT   
  
  
- TATTAGATAT AGTGAAAAAT CAAAAGTAAT AAGAGAAGTT GAAAACTTGA TGAAAGACGT TGGAAAGCCT   
  
  
- AAAAACGTTT TGCTATGTTG TCGGAATAAC AATGGAAACT GACGTGAAAG TAAGAAGTTA GTGTAGTTAA   
  
  
- AATTTTAGAA TAACCTTCAT GGCTCTATGG ATCGTTAAAT TAGAGCTTCT GAAACAAAAA AAATAAGAAA   
  
  
- AAACAAAGTT AAGTCCGAAA GTAATACCA

+     ERE

| Site Name | Organism | Position | Strand | Matrix score. | sequence | function |
| --- | --- | --- | --- | --- | --- | --- |
| ERE | Nicotiana glutinos | 1398 | + | 8 | ATTTTAAA |  |
| ERE | Nicotiana glutinos | 1105 | + | 8 | ATTTTAAA |  |
| ERE | Nicotiana glutinos | 1400 | - | 8 | ATTTTAAA |  |
| ERE | Nicotiana glutinos | 412 | - | 8 | ATTTCATA |  |
| ERE | Nicotiana glutinos | 870 | + | 8 | ATTTTAAA |  |

>006277   
+ TGATGAATAA AACAGAATGA TAATTAACAA ACAGAAACAG TTGTGTATAA TTTAAAAATG AATGGAGGGA   
  
  
+ GGTGGTGGTG TTTATAAGCG TTGTAAAAAT GCAGAGGTGA GGAGAGTGAT GAGTGAGTTT TGTTTTCTTC   
  
  
+ TATTCTTTTT GTGAAAAGAG AGTGAATTGG TCGTGGAACG CGGTTTGTTC GGAATCTCAG GGAAAAGACA   
  
  
+ TGTACGCGGA ACGCAGCATT AGACAGAGAA AAAAGAGAGA AGCAGACAAA GCCTTGTATT CAGAGAAGGA   
  
  
+ AGAAAGCTAA AAGTACTCAA TTATCTCGTG GGGAGCATAC AATAAAACCA CCACAATTAT ATTATACATG   
  
  
+ TACATATTTA TGTGTTATAT ATGTACGTTT ATTTATTTAT TTTATGTAAA TTGATATTTG TTATGAAATT   
  
  
+ ATATGCGGAT GTGGTTACGG TTTGTTTGAA TGTGTTACAC GCAAGGATTC AAAAGGTGCT AGGCACTGGT   
  
  
+ TGGGCGACGG ATATGTGTCT AGGTGGGAGT TTAGGCAGAG CCTAGGTGGG GGTTTAGGCA GTTTTTTTTT   
  
  
+ TTTAATTTTA ATTGAATTTA TTATATAACA TATAAATAAC TGCCTACTTG TACTTAAAAA AATACATCAT   
  
  
+ TGTATTAAAA CGCTTGAATT GCAAAATAGA ATGATATATT GATTATAAAA TATTCAAACA TATTGAAAAT   
  
  
+ ATGAGAAACA ACATACAATG AGTGCTCATC CAAACATTCA AGAAATCTCG TACAATTTAA TGAAAAAATA   
  
  
+ AAATGCAAGA TGGAAGTTAT CTTTCTTAAG TAAGAATTTC GACCTAAGTG GGTGGACAGG TCTAGGTGGG   
  
  
+ CGCCTAGGTA GGCTAGGTGC CCTTTCTTAA TTTTAAATGC TTAGGGACTA ATCATGTCGG TAACCAGCCG   
  
  
+ CCTAGCACTA GGCAGAGATT TTTAGAACAC TAGTTACATG CATTATTTGT AATGGTGACT TATTATTTGA   
  
  
+ ATGTCAAGCT TTTATGCATT ATCAATTGTG TTTTTATATC CCGCAAACTG AGAATTTAGC TTGAGTAGTT   
  
  
+ ATTTTAGTTT TATTCGTCAT CTAACAGTGT TGAGAAATTT GGAAAAATAA CTAAATTTTA AATCCCGTAT   
  
  
+ AGAATGTTAA CGATCATTTT AATTTTATGT AATTATGTAA TTATAATCAA TACTTGATGT AAAAGTATTA   
  
  
+ ATATACCCTT AATATTAGGA TTTCTGACAA CAACCAAAAC ACAACCTTTA AACTACCTTA AAATGAGGAA   
  
  
+ ATAATCTATA TCACTTTTTA GTTTTCATTA TTCTCTTCAA CTTTTGAACT ACTTTCTGCA ACCTTTCGGA   
  
  
+ TTTTTGCAAA ACGATACAAC AGCCTTATTG TTACCTTTGA CTGCACTTTC ATTCTTCAAT CACATCAATT   
  
  
+ TTAAAATCTT ATTGGAAGTA CCGAGATACC TAGCAATTTA ATCTCGAAGA CTTTGTTTTT TTTATTCTTT   
  
  
+ TTTGTTTCAA TTCAGGCTTT CATTATGGT  

- ACTACTTATT TTGTCTTACT ATTAATTGTT TGTCTTTGTC AACACATATT AAATTTTTAC TTACCTCCCT   
  
  
- CCACCACCAC AAATATTCGC AACATTTTTA CGTCTCCACT CCTCTCACTA CTCACTCAAA ACAAAAGAAG   
  
  
- ATAAGAAAAA CACTTTTCTC TCACTTAACC AGCACCTTGC GCCAAACAAG CCTTAGAGTC CCTTTTCTGT   
  
  
- ACATGCGCCT TGCGTCGTAA TCTGTCTCTT TTTTCTCTCT TCGTCTGTTT CGGAACATAA GTCTCTTCCT   
  
  
- TCTTTCGATT TTCATGAGTT AATAGAGCAC CCCTCGTATG TTATTTTGGT GGTGTTAATA TAATATGTAC   
  
  
- ATGTATAAAT ACACAATATA TACATGCAAA TAAATAAATA AAATACATTT AACTATAAAC AATACTTTAA   
  
  
- TATACGCCTA CACCAATGCC AAACAAACTT ACACAATGTG CGTTCCTAAG TTTTCCACGA TCCGTGACCA   
  
  
- ACCCGCTGCC TATACACAGA TCCACCCTCA AATCCGTCTC GGATCCACCC CCAAATCCGT CAAAAAAAAA   
  
  
- AAATTAAAAT TAACTTAAAT AATATATTGT ATATTTATTG ACGGATGAAC ATGAATTTTT TTATGTAGTA   
  
  
- ACATAATTTT GCGAACTTAA CGTTTTATCT TACTATATAA CTAATATTTT ATAAGTTTGT ATAACTTTTA   
  
  
- TACTCTTTGT TGTATGTTAC TCACGAGTAG GTTTGTAAGT TCTTTAGAGC ATGTTAAATT ACTTTTTTAT   
  
  
- TTTACGTTCT ACCTTCAATA GAAAGAATTC ATTCTTAAAG CTGGATTCAC CCACCTGTCC AGATCCACCC   
  
  
- GCGGATCCAT CCGATCCACG GGAAAGAATT AAAATTTACG AATCCCTGAT TAGTACAGCC ATTGGTCGGC   
  
  
- GGATCGTGAT CCGTCTCTAA AAATCTTGTG ATCAATGTAC GTAATAAACA TTACCACTGA ATAATAAACT   
  
  
- TACAGTTCGA AAATACGTAA TAGTTAACAC AAAAATATAG GGCGTTTGAC TCTTAAATCG AACTCATCAA   
  
  
- TAAAATCAAA ATAAGCAGTA GATTGTCACA ACTCTTTAAA CCTTTTTATT GATTTAAAAT TTAGGGCATA   
  
  
- TCTTACAATT GCTAGTAAAA TTAAAATACA TTAATACATT AATATTAGTT ATGAACTACA TTTTCATAAT   
  
  
- TATATGGGAA TTATAATCCT AAAGACTGTT GTTGGTTTTG TGTTGGAAAT TTGATGGAAT TTTACTCCTT   
  
  
- TATTAGATAT AGTGAAAAAT CAAAAGTAAT AAGAGAAGTT GAAAACTTGA TGAAAGACGT TGGAAAGCCT   
  
  
- AAAAACGTTT TGCTATGTTG TCGGAATAAC AATGGAAACT GACGTGAAAG TAAGAAGTTA GTGTAGTTAA   
  
  
- AATTTTAGAA TAACCTTCAT GGCTCTATGG ATCGTTAAAT TAGAGCTTCT GAAACAAAAA AAATAAGAAA   
  
  
- AAACAAAGTT AAGTCCGAAA GTAATACCA

+     G-box

| Site Name | Organism | Position | Strand | Matrix score. | sequence | function |
| --- | --- | --- | --- | --- | --- | --- |
| G-box | Zea mays | 170 | - | 6 | CACGAC | cis-acting regulatory element involved in light responsiveness |

>006277   
+ TGATGAATAA AACAGAATGA TAATTAACAA ACAGAAACAG TTGTGTATAA TTTAAAAATG AATGGAGGGA   
  
  
+ GGTGGTGGTG TTTATAAGCG TTGTAAAAAT GCAGAGGTGA GGAGAGTGAT GAGTGAGTTT TGTTTTCTTC   
  
  
+ TATTCTTTTT GTGAAAAGAG AGTGAATTGG TCGTGGAACG CGGTTTGTTC GGAATCTCAG GGAAAAGACA   
  
  
+ TGTACGCGGA ACGCAGCATT AGACAGAGAA AAAAGAGAGA AGCAGACAAA GCCTTGTATT CAGAGAAGGA   
  
  
+ AGAAAGCTAA AAGTACTCAA TTATCTCGTG GGGAGCATAC AATAAAACCA CCACAATTAT ATTATACATG   
  
  
+ TACATATTTA TGTGTTATAT ATGTACGTTT ATTTATTTAT TTTATGTAAA TTGATATTTG TTATGAAATT   
  
  
+ ATATGCGGAT GTGGTTACGG TTTGTTTGAA TGTGTTACAC GCAAGGATTC AAAAGGTGCT AGGCACTGGT   
  
  
+ TGGGCGACGG ATATGTGTCT AGGTGGGAGT TTAGGCAGAG CCTAGGTGGG GGTTTAGGCA GTTTTTTTTT   
  
  
+ TTTAATTTTA ATTGAATTTA TTATATAACA TATAAATAAC TGCCTACTTG TACTTAAAAA AATACATCAT   
  
  
+ TGTATTAAAA CGCTTGAATT GCAAAATAGA ATGATATATT GATTATAAAA TATTCAAACA TATTGAAAAT   
  
  
+ ATGAGAAACA ACATACAATG AGTGCTCATC CAAACATTCA AGAAATCTCG TACAATTTAA TGAAAAAATA   
  
  
+ AAATGCAAGA TGGAAGTTAT CTTTCTTAAG TAAGAATTTC GACCTAAGTG GGTGGACAGG TCTAGGTGGG   
  
  
+ CGCCTAGGTA GGCTAGGTGC CCTTTCTTAA TTTTAAATGC TTAGGGACTA ATCATGTCGG TAACCAGCCG   
  
  
+ CCTAGCACTA GGCAGAGATT TTTAGAACAC TAGTTACATG CATTATTTGT AATGGTGACT TATTATTTGA   
  
  
+ ATGTCAAGCT TTTATGCATT ATCAATTGTG TTTTTATATC CCGCAAACTG AGAATTTAGC TTGAGTAGTT   
  
  
+ ATTTTAGTTT TATTCGTCAT CTAACAGTGT TGAGAAATTT GGAAAAATAA CTAAATTTTA AATCCCGTAT   
  
  
+ AGAATGTTAA CGATCATTTT AATTTTATGT AATTATGTAA TTATAATCAA TACTTGATGT AAAAGTATTA   
  
  
+ ATATACCCTT AATATTAGGA TTTCTGACAA CAACCAAAAC ACAACCTTTA AACTACCTTA AAATGAGGAA   
  
  
+ ATAATCTATA TCACTTTTTA GTTTTCATTA TTCTCTTCAA CTTTTGAACT ACTTTCTGCA ACCTTTCGGA   
  
  
+ TTTTTGCAAA ACGATACAAC AGCCTTATTG TTACCTTTGA CTGCACTTTC ATTCTTCAAT CACATCAATT   
  
  
+ TTAAAATCTT ATTGGAAGTA CCGAGATACC TAGCAATTTA ATCTCGAAGA CTTTGTTTTT TTTATTCTTT   
  
  
+ TTTGTTTCAA TTCAGGCTTT CATTATGGT  

- ACTACTTATT TTGTCTTACT ATTAATTGTT TGTCTTTGTC AACACATATT AAATTTTTAC TTACCTCCCT   
  
  
- CCACCACCAC AAATATTCGC AACATTTTTA CGTCTCCACT CCTCTCACTA CTCACTCAAA ACAAAAGAAG   
  
  
- ATAAGAAAAA CACTTTTCTC TCACTTAACC AGCACCTTGC GCCAAACAAG CCTTAGAGTC CCTTTTCTGT   
  
  
- ACATGCGCCT TGCGTCGTAA TCTGTCTCTT TTTTCTCTCT TCGTCTGTTT CGGAACATAA GTCTCTTCCT   
  
  
- TCTTTCGATT TTCATGAGTT AATAGAGCAC CCCTCGTATG TTATTTTGGT GGTGTTAATA TAATATGTAC   
  
  
- ATGTATAAAT ACACAATATA TACATGCAAA TAAATAAATA AAATACATTT AACTATAAAC AATACTTTAA   
  
  
- TATACGCCTA CACCAATGCC AAACAAACTT ACACAATGTG CGTTCCTAAG TTTTCCACGA TCCGTGACCA   
  
  
- ACCCGCTGCC TATACACAGA TCCACCCTCA AATCCGTCTC GGATCCACCC CCAAATCCGT CAAAAAAAAA   
  
  
- AAATTAAAAT TAACTTAAAT AATATATTGT ATATTTATTG ACGGATGAAC ATGAATTTTT TTATGTAGTA   
  
  
- ACATAATTTT GCGAACTTAA CGTTTTATCT TACTATATAA CTAATATTTT ATAAGTTTGT ATAACTTTTA   
  
  
- TACTCTTTGT TGTATGTTAC TCACGAGTAG GTTTGTAAGT TCTTTAGAGC ATGTTAAATT ACTTTTTTAT   
  
  
- TTTACGTTCT ACCTTCAATA GAAAGAATTC ATTCTTAAAG CTGGATTCAC CCACCTGTCC AGATCCACCC   
  
  
- GCGGATCCAT CCGATCCACG GGAAAGAATT AAAATTTACG AATCCCTGAT TAGTACAGCC ATTGGTCGGC   
  
  
- GGATCGTGAT CCGTCTCTAA AAATCTTGTG ATCAATGTAC GTAATAAACA TTACCACTGA ATAATAAACT   
  
  
- TACAGTTCGA AAATACGTAA TAGTTAACAC AAAAATATAG GGCGTTTGAC TCTTAAATCG AACTCATCAA   
  
  
- TAAAATCAAA ATAAGCAGTA GATTGTCACA ACTCTTTAAA CCTTTTTATT GATTTAAAAT TTAGGGCATA   
  
  
- TCTTACAATT GCTAGTAAAA TTAAAATACA TTAATACATT AATATTAGTT ATGAACTACA TTTTCATAAT   
  
  
- TATATGGGAA TTATAATCCT AAAGACTGTT GTTGGTTTTG TGTTGGAAAT TTGATGGAAT TTTACTCCTT   
  
  
- TATTAGATAT AGTGAAAAAT CAAAAGTAAT AAGAGAAGTT GAAAACTTGA TGAAAGACGT TGGAAAGCCT   
  
  
- AAAAACGTTT TGCTATGTTG TCGGAATAAC AATGGAAACT GACGTGAAAG TAAGAAGTTA GTGTAGTTAA   
  
  
- AATTTTAGAA TAACCTTCAT GGCTCTATGG ATCGTTAAAT TAGAGCTTCT GAAACAAAAA AAATAAGAAA   
  
  
- AAACAAAGTT AAGTCCGAAA GTAATACCA

+     LTR

| Site Name | Organism | Position | Strand | Matrix score. | sequence | function |
| --- | --- | --- | --- | --- | --- | --- |
| LTR | Hordeum vulgare | 1324 | - | 6 | CCGAAA | cis-acting element involved in low-temperature responsiveness |

>006277   
+ TGATGAATAA AACAGAATGA TAATTAACAA ACAGAAACAG TTGTGTATAA TTTAAAAATG AATGGAGGGA   
  
  
+ GGTGGTGGTG TTTATAAGCG TTGTAAAAAT GCAGAGGTGA GGAGAGTGAT GAGTGAGTTT TGTTTTCTTC   
  
  
+ TATTCTTTTT GTGAAAAGAG AGTGAATTGG TCGTGGAACG CGGTTTGTTC GGAATCTCAG GGAAAAGACA   
  
  
+ TGTACGCGGA ACGCAGCATT AGACAGAGAA AAAAGAGAGA AGCAGACAAA GCCTTGTATT CAGAGAAGGA   
  
  
+ AGAAAGCTAA AAGTACTCAA TTATCTCGTG GGGAGCATAC AATAAAACCA CCACAATTAT ATTATACATG   
  
  
+ TACATATTTA TGTGTTATAT ATGTACGTTT ATTTATTTAT TTTATGTAAA TTGATATTTG TTATGAAATT   
  
  
+ ATATGCGGAT GTGGTTACGG TTTGTTTGAA TGTGTTACAC GCAAGGATTC AAAAGGTGCT AGGCACTGGT   
  
  
+ TGGGCGACGG ATATGTGTCT AGGTGGGAGT TTAGGCAGAG CCTAGGTGGG GGTTTAGGCA GTTTTTTTTT   
  
  
+ TTTAATTTTA ATTGAATTTA TTATATAACA TATAAATAAC TGCCTACTTG TACTTAAAAA AATACATCAT   
  
  
+ TGTATTAAAA CGCTTGAATT GCAAAATAGA ATGATATATT GATTATAAAA TATTCAAACA TATTGAAAAT   
  
  
+ ATGAGAAACA ACATACAATG AGTGCTCATC CAAACATTCA AGAAATCTCG TACAATTTAA TGAAAAAATA   
  
  
+ AAATGCAAGA TGGAAGTTAT CTTTCTTAAG TAAGAATTTC GACCTAAGTG GGTGGACAGG TCTAGGTGGG   
  
  
+ CGCCTAGGTA GGCTAGGTGC CCTTTCTTAA TTTTAAATGC TTAGGGACTA ATCATGTCGG TAACCAGCCG   
  
  
+ CCTAGCACTA GGCAGAGATT TTTAGAACAC TAGTTACATG CATTATTTGT AATGGTGACT TATTATTTGA   
  
  
+ ATGTCAAGCT TTTATGCATT ATCAATTGTG TTTTTATATC CCGCAAACTG AGAATTTAGC TTGAGTAGTT   
  
  
+ ATTTTAGTTT TATTCGTCAT CTAACAGTGT TGAGAAATTT GGAAAAATAA CTAAATTTTA AATCCCGTAT   
  
  
+ AGAATGTTAA CGATCATTTT AATTTTATGT AATTATGTAA TTATAATCAA TACTTGATGT AAAAGTATTA   
  
  
+ ATATACCCTT AATATTAGGA TTTCTGACAA CAACCAAAAC ACAACCTTTA AACTACCTTA AAATGAGGAA   
  
  
+ ATAATCTATA TCACTTTTTA GTTTTCATTA TTCTCTTCAA CTTTTGAACT ACTTTCTGCA ACCTTTCGGA   
  
  
+ TTTTTGCAAA ACGATACAAC AGCCTTATTG TTACCTTTGA CTGCACTTTC ATTCTTCAAT CACATCAATT   
  
  
+ TTAAAATCTT ATTGGAAGTA CCGAGATACC TAGCAATTTA ATCTCGAAGA CTTTGTTTTT TTTATTCTTT   
  
  
+ TTTGTTTCAA TTCAGGCTTT CATTATGGT  

- ACTACTTATT TTGTCTTACT ATTAATTGTT TGTCTTTGTC AACACATATT AAATTTTTAC TTACCTCCCT   
  
  
- CCACCACCAC AAATATTCGC AACATTTTTA CGTCTCCACT CCTCTCACTA CTCACTCAAA ACAAAAGAAG   
  
  
- ATAAGAAAAA CACTTTTCTC TCACTTAACC AGCACCTTGC GCCAAACAAG CCTTAGAGTC CCTTTTCTGT   
  
  
- ACATGCGCCT TGCGTCGTAA TCTGTCTCTT TTTTCTCTCT TCGTCTGTTT CGGAACATAA GTCTCTTCCT   
  
  
- TCTTTCGATT TTCATGAGTT AATAGAGCAC CCCTCGTATG TTATTTTGGT GGTGTTAATA TAATATGTAC   
  
  
- ATGTATAAAT ACACAATATA TACATGCAAA TAAATAAATA AAATACATTT AACTATAAAC AATACTTTAA   
  
  
- TATACGCCTA CACCAATGCC AAACAAACTT ACACAATGTG CGTTCCTAAG TTTTCCACGA TCCGTGACCA   
  
  
- ACCCGCTGCC TATACACAGA TCCACCCTCA AATCCGTCTC GGATCCACCC CCAAATCCGT CAAAAAAAAA   
  
  
- AAATTAAAAT TAACTTAAAT AATATATTGT ATATTTATTG ACGGATGAAC ATGAATTTTT TTATGTAGTA   
  
  
- ACATAATTTT GCGAACTTAA CGTTTTATCT TACTATATAA CTAATATTTT ATAAGTTTGT ATAACTTTTA   
  
  
- TACTCTTTGT TGTATGTTAC TCACGAGTAG GTTTGTAAGT TCTTTAGAGC ATGTTAAATT ACTTTTTTAT   
  
  
- TTTACGTTCT ACCTTCAATA GAAAGAATTC ATTCTTAAAG CTGGATTCAC CCACCTGTCC AGATCCACCC   
  
  
- GCGGATCCAT CCGATCCACG GGAAAGAATT AAAATTTACG AATCCCTGAT TAGTACAGCC ATTGGTCGGC   
  
  
- GGATCGTGAT CCGTCTCTAA AAATCTTGTG ATCAATGTAC GTAATAAACA TTACCACTGA ATAATAAACT   
  
  
- TACAGTTCGA AAATACGTAA TAGTTAACAC AAAAATATAG GGCGTTTGAC TCTTAAATCG AACTCATCAA   
  
  
- TAAAATCAAA ATAAGCAGTA GATTGTCACA ACTCTTTAAA CCTTTTTATT GATTTAAAAT TTAGGGCATA   
  
  
- TCTTACAATT GCTAGTAAAA TTAAAATACA TTAATACATT AATATTAGTT ATGAACTACA TTTTCATAAT   
  
  
- TATATGGGAA TTATAATCCT AAAGACTGTT GTTGGTTTTG TGTTGGAAAT TTGATGGAAT TTTACTCCTT   
  
  
- TATTAGATAT AGTGAAAAAT CAAAAGTAAT AAGAGAAGTT GAAAACTTGA TGAAAGACGT TGGAAAGCCT   
  
  
- AAAAACGTTT TGCTATGTTG TCGGAATAAC AATGGAAACT GACGTGAAAG TAAGAAGTTA GTGTAGTTAA   
  
  
- AATTTTAGAA TAACCTTCAT GGCTCTATGG ATCGTTAAAT TAGAGCTTCT GAAACAAAAA AAATAAGAAA   
  
  
- AAACAAAGTT AAGTCCGAAA GTAATACCA

+     MBS

| Site Name | Organism | Position | Strand | Matrix score. | sequence | function |
| --- | --- | --- | --- | --- | --- | --- |
| MBS | Arabidopsis thaliana | 38 | - | 6 | CAACTG | MYB binding site involved in drought-inducibility |

>006277   
+ TGATGAATAA AACAGAATGA TAATTAACAA ACAGAAACAG TTGTGTATAA TTTAAAAATG AATGGAGGGA   
  
  
+ GGTGGTGGTG TTTATAAGCG TTGTAAAAAT GCAGAGGTGA GGAGAGTGAT GAGTGAGTTT TGTTTTCTTC   
  
  
+ TATTCTTTTT GTGAAAAGAG AGTGAATTGG TCGTGGAACG CGGTTTGTTC GGAATCTCAG GGAAAAGACA   
  
  
+ TGTACGCGGA ACGCAGCATT AGACAGAGAA AAAAGAGAGA AGCAGACAAA GCCTTGTATT CAGAGAAGGA   
  
  
+ AGAAAGCTAA AAGTACTCAA TTATCTCGTG GGGAGCATAC AATAAAACCA CCACAATTAT ATTATACATG   
  
  
+ TACATATTTA TGTGTTATAT ATGTACGTTT ATTTATTTAT TTTATGTAAA TTGATATTTG TTATGAAATT   
  
  
+ ATATGCGGAT GTGGTTACGG TTTGTTTGAA TGTGTTACAC GCAAGGATTC AAAAGGTGCT AGGCACTGGT   
  
  
+ TGGGCGACGG ATATGTGTCT AGGTGGGAGT TTAGGCAGAG CCTAGGTGGG GGTTTAGGCA GTTTTTTTTT   
  
  
+ TTTAATTTTA ATTGAATTTA TTATATAACA TATAAATAAC TGCCTACTTG TACTTAAAAA AATACATCAT   
  
  
+ TGTATTAAAA CGCTTGAATT GCAAAATAGA ATGATATATT GATTATAAAA TATTCAAACA TATTGAAAAT   
  
  
+ ATGAGAAACA ACATACAATG AGTGCTCATC CAAACATTCA AGAAATCTCG TACAATTTAA TGAAAAAATA   
  
  
+ AAATGCAAGA TGGAAGTTAT CTTTCTTAAG TAAGAATTTC GACCTAAGTG GGTGGACAGG TCTAGGTGGG   
  
  
+ CGCCTAGGTA GGCTAGGTGC CCTTTCTTAA TTTTAAATGC TTAGGGACTA ATCATGTCGG TAACCAGCCG   
  
  
+ CCTAGCACTA GGCAGAGATT TTTAGAACAC TAGTTACATG CATTATTTGT AATGGTGACT TATTATTTGA   
  
  
+ ATGTCAAGCT TTTATGCATT ATCAATTGTG TTTTTATATC CCGCAAACTG AGAATTTAGC TTGAGTAGTT   
  
  
+ ATTTTAGTTT TATTCGTCAT CTAACAGTGT TGAGAAATTT GGAAAAATAA CTAAATTTTA AATCCCGTAT   
  
  
+ AGAATGTTAA CGATCATTTT AATTTTATGT AATTATGTAA TTATAATCAA TACTTGATGT AAAAGTATTA   
  
  
+ ATATACCCTT AATATTAGGA TTTCTGACAA CAACCAAAAC ACAACCTTTA AACTACCTTA AAATGAGGAA   
  
  
+ ATAATCTATA TCACTTTTTA GTTTTCATTA TTCTCTTCAA CTTTTGAACT ACTTTCTGCA ACCTTTCGGA   
  
  
+ TTTTTGCAAA ACGATACAAC AGCCTTATTG TTACCTTTGA CTGCACTTTC ATTCTTCAAT CACATCAATT   
  
  
+ TTAAAATCTT ATTGGAAGTA CCGAGATACC TAGCAATTTA ATCTCGAAGA CTTTGTTTTT TTTATTCTTT   
  
  
+ TTTGTTTCAA TTCAGGCTTT CATTATGGT  

- ACTACTTATT TTGTCTTACT ATTAATTGTT TGTCTTTGTC AACACATATT AAATTTTTAC TTACCTCCCT   
  
  
- CCACCACCAC AAATATTCGC AACATTTTTA CGTCTCCACT CCTCTCACTA CTCACTCAAA ACAAAAGAAG   
  
  
- ATAAGAAAAA CACTTTTCTC TCACTTAACC AGCACCTTGC GCCAAACAAG CCTTAGAGTC CCTTTTCTGT   
  
  
- ACATGCGCCT TGCGTCGTAA TCTGTCTCTT TTTTCTCTCT TCGTCTGTTT CGGAACATAA GTCTCTTCCT   
  
  
- TCTTTCGATT TTCATGAGTT AATAGAGCAC CCCTCGTATG TTATTTTGGT GGTGTTAATA TAATATGTAC   
  
  
- ATGTATAAAT ACACAATATA TACATGCAAA TAAATAAATA AAATACATTT AACTATAAAC AATACTTTAA   
  
  
- TATACGCCTA CACCAATGCC AAACAAACTT ACACAATGTG CGTTCCTAAG TTTTCCACGA TCCGTGACCA   
  
  
- ACCCGCTGCC TATACACAGA TCCACCCTCA AATCCGTCTC GGATCCACCC CCAAATCCGT CAAAAAAAAA   
  
  
- AAATTAAAAT TAACTTAAAT AATATATTGT ATATTTATTG ACGGATGAAC ATGAATTTTT TTATGTAGTA   
  
  
- ACATAATTTT GCGAACTTAA CGTTTTATCT TACTATATAA CTAATATTTT ATAAGTTTGT ATAACTTTTA   
  
  
- TACTCTTTGT TGTATGTTAC TCACGAGTAG GTTTGTAAGT TCTTTAGAGC ATGTTAAATT ACTTTTTTAT   
  
  
- TTTACGTTCT ACCTTCAATA GAAAGAATTC ATTCTTAAAG CTGGATTCAC CCACCTGTCC AGATCCACCC   
  
  
- GCGGATCCAT CCGATCCACG GGAAAGAATT AAAATTTACG AATCCCTGAT TAGTACAGCC ATTGGTCGGC   
  
  
- GGATCGTGAT CCGTCTCTAA AAATCTTGTG ATCAATGTAC GTAATAAACA TTACCACTGA ATAATAAACT   
  
  
- TACAGTTCGA AAATACGTAA TAGTTAACAC AAAAATATAG GGCGTTTGAC TCTTAAATCG AACTCATCAA   
  
  
- TAAAATCAAA ATAAGCAGTA GATTGTCACA ACTCTTTAAA CCTTTTTATT GATTTAAAAT TTAGGGCATA   
  
  
- TCTTACAATT GCTAGTAAAA TTAAAATACA TTAATACATT AATATTAGTT ATGAACTACA TTTTCATAAT   
  
  
- TATATGGGAA TTATAATCCT AAAGACTGTT GTTGGTTTTG TGTTGGAAAT TTGATGGAAT TTTACTCCTT   
  
  
- TATTAGATAT AGTGAAAAAT CAAAAGTAAT AAGAGAAGTT GAAAACTTGA TGAAAGACGT TGGAAAGCCT   
  
  
- AAAAACGTTT TGCTATGTTG TCGGAATAAC AATGGAAACT GACGTGAAAG TAAGAAGTTA GTGTAGTTAA   
  
  
- AATTTTAGAA TAACCTTCAT GGCTCTATGG ATCGTTAAAT TAGAGCTTCT GAAACAAAAA AAATAAGAAA   
  
  
- AAACAAAGTT AAGTCCGAAA GTAATACCA

+     MYB

| Site Name | Organism | Position | Strand | Matrix score. | sequence | function |
| --- | --- | --- | --- | --- | --- | --- |
| MYB | Arabidopsis thaliana | 1347 | + | 6 | CAACAG |  |
| MYB | Arabidopsis thaliana | 901 | + | 6 | TAACCA |  |
| MYB | Arabidopsis thaliana | 1221 | + | 6 | CAACCA |  |
| MYB | Arabidopsis thaliana | 432 | - | 6 | TAACCA |  |
| MYB | Arabidopsis thaliana | 487 | - | 6 | CAACCA |  |

>006277   
+ TGATGAATAA AACAGAATGA TAATTAACAA ACAGAAACAG TTGTGTATAA TTTAAAAATG AATGGAGGGA   
  
  
+ GGTGGTGGTG TTTATAAGCG TTGTAAAAAT GCAGAGGTGA GGAGAGTGAT GAGTGAGTTT TGTTTTCTTC   
  
  
+ TATTCTTTTT GTGAAAAGAG AGTGAATTGG TCGTGGAACG CGGTTTGTTC GGAATCTCAG GGAAAAGACA   
  
  
+ TGTACGCGGA ACGCAGCATT AGACAGAGAA AAAAGAGAGA AGCAGACAAA GCCTTGTATT CAGAGAAGGA   
  
  
+ AGAAAGCTAA AAGTACTCAA TTATCTCGTG GGGAGCATAC AATAAAACCA CCACAATTAT ATTATACATG   
  
  
+ TACATATTTA TGTGTTATAT ATGTACGTTT ATTTATTTAT TTTATGTAAA TTGATATTTG TTATGAAATT   
  
  
+ ATATGCGGAT GTGGTTACGG TTTGTTTGAA TGTGTTACAC GCAAGGATTC AAAAGGTGCT AGGCACTGGT   
  
  
+ TGGGCGACGG ATATGTGTCT AGGTGGGAGT TTAGGCAGAG CCTAGGTGGG GGTTTAGGCA GTTTTTTTTT   
  
  
+ TTTAATTTTA ATTGAATTTA TTATATAACA TATAAATAAC TGCCTACTTG TACTTAAAAA AATACATCAT   
  
  
+ TGTATTAAAA CGCTTGAATT GCAAAATAGA ATGATATATT GATTATAAAA TATTCAAACA TATTGAAAAT   
  
  
+ ATGAGAAACA ACATACAATG AGTGCTCATC CAAACATTCA AGAAATCTCG TACAATTTAA TGAAAAAATA   
  
  
+ AAATGCAAGA TGGAAGTTAT CTTTCTTAAG TAAGAATTTC GACCTAAGTG GGTGGACAGG TCTAGGTGGG   
  
  
+ CGCCTAGGTA GGCTAGGTGC CCTTTCTTAA TTTTAAATGC TTAGGGACTA ATCATGTCGG TAACCAGCCG   
  
  
+ CCTAGCACTA GGCAGAGATT TTTAGAACAC TAGTTACATG CATTATTTGT AATGGTGACT TATTATTTGA   
  
  
+ ATGTCAAGCT TTTATGCATT ATCAATTGTG TTTTTATATC CCGCAAACTG AGAATTTAGC TTGAGTAGTT   
  
  
+ ATTTTAGTTT TATTCGTCAT CTAACAGTGT TGAGAAATTT GGAAAAATAA CTAAATTTTA AATCCCGTAT   
  
  
+ AGAATGTTAA CGATCATTTT AATTTTATGT AATTATGTAA TTATAATCAA TACTTGATGT AAAAGTATTA   
  
  
+ ATATACCCTT AATATTAGGA TTTCTGACAA CAACCAAAAC ACAACCTTTA AACTACCTTA AAATGAGGAA   
  
  
+ ATAATCTATA TCACTTTTTA GTTTTCATTA TTCTCTTCAA CTTTTGAACT ACTTTCTGCA ACCTTTCGGA   
  
  
+ TTTTTGCAAA ACGATACAAC AGCCTTATTG TTACCTTTGA CTGCACTTTC ATTCTTCAAT CACATCAATT   
  
  
+ TTAAAATCTT ATTGGAAGTA CCGAGATACC TAGCAATTTA ATCTCGAAGA CTTTGTTTTT TTTATTCTTT   
  
  
+ TTTGTTTCAA TTCAGGCTTT CATTATGGT  

- ACTACTTATT TTGTCTTACT ATTAATTGTT TGTCTTTGTC AACACATATT AAATTTTTAC TTACCTCCCT   
  
  
- CCACCACCAC AAATATTCGC AACATTTTTA CGTCTCCACT CCTCTCACTA CTCACTCAAA ACAAAAGAAG   
  
  
- ATAAGAAAAA CACTTTTCTC TCACTTAACC AGCACCTTGC GCCAAACAAG CCTTAGAGTC CCTTTTCTGT   
  
  
- ACATGCGCCT TGCGTCGTAA TCTGTCTCTT TTTTCTCTCT TCGTCTGTTT CGGAACATAA GTCTCTTCCT   
  
  
- TCTTTCGATT TTCATGAGTT AATAGAGCAC CCCTCGTATG TTATTTTGGT GGTGTTAATA TAATATGTAC   
  
  
- ATGTATAAAT ACACAATATA TACATGCAAA TAAATAAATA AAATACATTT AACTATAAAC AATACTTTAA   
  
  
- TATACGCCTA CACCAATGCC AAACAAACTT ACACAATGTG CGTTCCTAAG TTTTCCACGA TCCGTGACCA   
  
  
- ACCCGCTGCC TATACACAGA TCCACCCTCA AATCCGTCTC GGATCCACCC CCAAATCCGT CAAAAAAAAA   
  
  
- AAATTAAAAT TAACTTAAAT AATATATTGT ATATTTATTG ACGGATGAAC ATGAATTTTT TTATGTAGTA   
  
  
- ACATAATTTT GCGAACTTAA CGTTTTATCT TACTATATAA CTAATATTTT ATAAGTTTGT ATAACTTTTA   
  
  
- TACTCTTTGT TGTATGTTAC TCACGAGTAG GTTTGTAAGT TCTTTAGAGC ATGTTAAATT ACTTTTTTAT   
  
  
- TTTACGTTCT ACCTTCAATA GAAAGAATTC ATTCTTAAAG CTGGATTCAC CCACCTGTCC AGATCCACCC   
  
  
- GCGGATCCAT CCGATCCACG GGAAAGAATT AAAATTTACG AATCCCTGAT TAGTACAGCC ATTGGTCGGC   
  
  
- GGATCGTGAT CCGTCTCTAA AAATCTTGTG ATCAATGTAC GTAATAAACA TTACCACTGA ATAATAAACT   
  
  
- TACAGTTCGA AAATACGTAA TAGTTAACAC AAAAATATAG GGCGTTTGAC TCTTAAATCG AACTCATCAA   
  
  
- TAAAATCAAA ATAAGCAGTA GATTGTCACA ACTCTTTAAA CCTTTTTATT GATTTAAAAT TTAGGGCATA   
  
  
- TCTTACAATT GCTAGTAAAA TTAAAATACA TTAATACATT AATATTAGTT ATGAACTACA TTTTCATAAT   
  
  
- TATATGGGAA TTATAATCCT AAAGACTGTT GTTGGTTTTG TGTTGGAAAT TTGATGGAAT TTTACTCCTT   
  
  
- TATTAGATAT AGTGAAAAAT CAAAAGTAAT AAGAGAAGTT GAAAACTTGA TGAAAGACGT TGGAAAGCCT   
  
  
- AAAAACGTTT TGCTATGTTG TCGGAATAAC AATGGAAACT GACGTGAAAG TAAGAAGTTA GTGTAGTTAA   
  
  
- AATTTTAGAA TAACCTTCAT GGCTCTATGG ATCGTTAAAT TAGAGCTTCT GAAACAAAAA AAATAAGAAA   
  
  
- AAACAAAGTT AAGTCCGAAA GTAATACCA

+     MYB-like sequence

| Site Name | Organism | Position | Strand | Matrix score. | sequence | function |
| --- | --- | --- | --- | --- | --- | --- |
| MYB-like sequence | Arabidopsis thaliana | 432 | - | 6 | TAACCA |  |
| MYB-like sequence | Arabidopsis thaliana | 901 | + | 6 | TAACCA |  |

>006277   
+ TGATGAATAA AACAGAATGA TAATTAACAA ACAGAAACAG TTGTGTATAA TTTAAAAATG AATGGAGGGA   
  
  
+ GGTGGTGGTG TTTATAAGCG TTGTAAAAAT GCAGAGGTGA GGAGAGTGAT GAGTGAGTTT TGTTTTCTTC   
  
  
+ TATTCTTTTT GTGAAAAGAG AGTGAATTGG TCGTGGAACG CGGTTTGTTC GGAATCTCAG GGAAAAGACA   
  
  
+ TGTACGCGGA ACGCAGCATT AGACAGAGAA AAAAGAGAGA AGCAGACAAA GCCTTGTATT CAGAGAAGGA   
  
  
+ AGAAAGCTAA AAGTACTCAA TTATCTCGTG GGGAGCATAC AATAAAACCA CCACAATTAT ATTATACATG   
  
  
+ TACATATTTA TGTGTTATAT ATGTACGTTT ATTTATTTAT TTTATGTAAA TTGATATTTG TTATGAAATT   
  
  
+ ATATGCGGAT GTGGTTACGG TTTGTTTGAA TGTGTTACAC GCAAGGATTC AAAAGGTGCT AGGCACTGGT   
  
  
+ TGGGCGACGG ATATGTGTCT AGGTGGGAGT TTAGGCAGAG CCTAGGTGGG GGTTTAGGCA GTTTTTTTTT   
  
  
+ TTTAATTTTA ATTGAATTTA TTATATAACA TATAAATAAC TGCCTACTTG TACTTAAAAA AATACATCAT   
  
  
+ TGTATTAAAA CGCTTGAATT GCAAAATAGA ATGATATATT GATTATAAAA TATTCAAACA TATTGAAAAT   
  
  
+ ATGAGAAACA ACATACAATG AGTGCTCATC CAAACATTCA AGAAATCTCG TACAATTTAA TGAAAAAATA   
  
  
+ AAATGCAAGA TGGAAGTTAT CTTTCTTAAG TAAGAATTTC GACCTAAGTG GGTGGACAGG TCTAGGTGGG   
  
  
+ CGCCTAGGTA GGCTAGGTGC CCTTTCTTAA TTTTAAATGC TTAGGGACTA ATCATGTCGG TAACCAGCCG   
  
  
+ CCTAGCACTA GGCAGAGATT TTTAGAACAC TAGTTACATG CATTATTTGT AATGGTGACT TATTATTTGA   
  
  
+ ATGTCAAGCT TTTATGCATT ATCAATTGTG TTTTTATATC CCGCAAACTG AGAATTTAGC TTGAGTAGTT   
  
  
+ ATTTTAGTTT TATTCGTCAT CTAACAGTGT TGAGAAATTT GGAAAAATAA CTAAATTTTA AATCCCGTAT   
  
  
+ AGAATGTTAA CGATCATTTT AATTTTATGT AATTATGTAA TTATAATCAA TACTTGATGT AAAAGTATTA   
  
  
+ ATATACCCTT AATATTAGGA TTTCTGACAA CAACCAAAAC ACAACCTTTA AACTACCTTA AAATGAGGAA   
  
  
+ ATAATCTATA TCACTTTTTA GTTTTCATTA TTCTCTTCAA CTTTTGAACT ACTTTCTGCA ACCTTTCGGA   
  
  
+ TTTTTGCAAA ACGATACAAC AGCCTTATTG TTACCTTTGA CTGCACTTTC ATTCTTCAAT CACATCAATT   
  
  
+ TTAAAATCTT ATTGGAAGTA CCGAGATACC TAGCAATTTA ATCTCGAAGA CTTTGTTTTT TTTATTCTTT   
  
  
+ TTTGTTTCAA TTCAGGCTTT CATTATGGT  

- ACTACTTATT TTGTCTTACT ATTAATTGTT TGTCTTTGTC AACACATATT AAATTTTTAC TTACCTCCCT   
  
  
- CCACCACCAC AAATATTCGC AACATTTTTA CGTCTCCACT CCTCTCACTA CTCACTCAAA ACAAAAGAAG   
  
  
- ATAAGAAAAA CACTTTTCTC TCACTTAACC AGCACCTTGC GCCAAACAAG CCTTAGAGTC CCTTTTCTGT   
  
  
- ACATGCGCCT TGCGTCGTAA TCTGTCTCTT TTTTCTCTCT TCGTCTGTTT CGGAACATAA GTCTCTTCCT   
  
  
- TCTTTCGATT TTCATGAGTT AATAGAGCAC CCCTCGTATG TTATTTTGGT GGTGTTAATA TAATATGTAC   
  
  
- ATGTATAAAT ACACAATATA TACATGCAAA TAAATAAATA AAATACATTT AACTATAAAC AATACTTTAA   
  
  
- TATACGCCTA CACCAATGCC AAACAAACTT ACACAATGTG CGTTCCTAAG TTTTCCACGA TCCGTGACCA   
  
  
- ACCCGCTGCC TATACACAGA TCCACCCTCA AATCCGTCTC GGATCCACCC CCAAATCCGT CAAAAAAAAA   
  
  
- AAATTAAAAT TAACTTAAAT AATATATTGT ATATTTATTG ACGGATGAAC ATGAATTTTT TTATGTAGTA   
  
  
- ACATAATTTT GCGAACTTAA CGTTTTATCT TACTATATAA CTAATATTTT ATAAGTTTGT ATAACTTTTA   
  
  
- TACTCTTTGT TGTATGTTAC TCACGAGTAG GTTTGTAAGT TCTTTAGAGC ATGTTAAATT ACTTTTTTAT   
  
  
- TTTACGTTCT ACCTTCAATA GAAAGAATTC ATTCTTAAAG CTGGATTCAC CCACCTGTCC AGATCCACCC   
  
  
- GCGGATCCAT CCGATCCACG GGAAAGAATT AAAATTTACG AATCCCTGAT TAGTACAGCC ATTGGTCGGC   
  
  
- GGATCGTGAT CCGTCTCTAA AAATCTTGTG ATCAATGTAC GTAATAAACA TTACCACTGA ATAATAAACT   
  
  
- TACAGTTCGA AAATACGTAA TAGTTAACAC AAAAATATAG GGCGTTTGAC TCTTAAATCG AACTCATCAA   
  
  
- TAAAATCAAA ATAAGCAGTA GATTGTCACA ACTCTTTAAA CCTTTTTATT GATTTAAAAT TTAGGGCATA   
  
  
- TCTTACAATT GCTAGTAAAA TTAAAATACA TTAATACATT AATATTAGTT ATGAACTACA TTTTCATAAT   
  
  
- TATATGGGAA TTATAATCCT AAAGACTGTT GTTGGTTTTG TGTTGGAAAT TTGATGGAAT TTTACTCCTT   
  
  
- TATTAGATAT AGTGAAAAAT CAAAAGTAAT AAGAGAAGTT GAAAACTTGA TGAAAGACGT TGGAAAGCCT   
  
  
- AAAAACGTTT TGCTATGTTG TCGGAATAAC AATGGAAACT GACGTGAAAG TAAGAAGTTA GTGTAGTTAA   
  
  
- AATTTTAGAA TAACCTTCAT GGCTCTATGG ATCGTTAAAT TAGAGCTTCT GAAACAAAAA AAATAAGAAA   
  
  
- AAACAAAGTT AAGTCCGAAA GTAATACCA

+     MYC

| Site Name | Organism | Position | Strand | Matrix score. | sequence | function |
| --- | --- | --- | --- | --- | --- | --- |
| MYC | Arabidopsis thaliana | 1003 | - | 6 | CAATTG |  |

>006277   
+ TGATGAATAA AACAGAATGA TAATTAACAA ACAGAAACAG TTGTGTATAA TTTAAAAATG AATGGAGGGA   
  
  
+ GGTGGTGGTG TTTATAAGCG TTGTAAAAAT GCAGAGGTGA GGAGAGTGAT GAGTGAGTTT TGTTTTCTTC   
  
  
+ TATTCTTTTT GTGAAAAGAG AGTGAATTGG TCGTGGAACG CGGTTTGTTC GGAATCTCAG GGAAAAGACA   
  
  
+ TGTACGCGGA ACGCAGCATT AGACAGAGAA AAAAGAGAGA AGCAGACAAA GCCTTGTATT CAGAGAAGGA   
  
  
+ AGAAAGCTAA AAGTACTCAA TTATCTCGTG GGGAGCATAC AATAAAACCA CCACAATTAT ATTATACATG   
  
  
+ TACATATTTA TGTGTTATAT ATGTACGTTT ATTTATTTAT TTTATGTAAA TTGATATTTG TTATGAAATT   
  
  
+ ATATGCGGAT GTGGTTACGG TTTGTTTGAA TGTGTTACAC GCAAGGATTC AAAAGGTGCT AGGCACTGGT   
  
  
+ TGGGCGACGG ATATGTGTCT AGGTGGGAGT TTAGGCAGAG CCTAGGTGGG GGTTTAGGCA GTTTTTTTTT   
  
  
+ TTTAATTTTA ATTGAATTTA TTATATAACA TATAAATAAC TGCCTACTTG TACTTAAAAA AATACATCAT   
  
  
+ TGTATTAAAA CGCTTGAATT GCAAAATAGA ATGATATATT GATTATAAAA TATTCAAACA TATTGAAAAT   
  
  
+ ATGAGAAACA ACATACAATG AGTGCTCATC CAAACATTCA AGAAATCTCG TACAATTTAA TGAAAAAATA   
  
  
+ AAATGCAAGA TGGAAGTTAT CTTTCTTAAG TAAGAATTTC GACCTAAGTG GGTGGACAGG TCTAGGTGGG   
  
  
+ CGCCTAGGTA GGCTAGGTGC CCTTTCTTAA TTTTAAATGC TTAGGGACTA ATCATGTCGG TAACCAGCCG   
  
  
+ CCTAGCACTA GGCAGAGATT TTTAGAACAC TAGTTACATG CATTATTTGT AATGGTGACT TATTATTTGA   
  
  
+ ATGTCAAGCT TTTATGCATT ATCAATTGTG TTTTTATATC CCGCAAACTG AGAATTTAGC TTGAGTAGTT   
  
  
+ ATTTTAGTTT TATTCGTCAT CTAACAGTGT TGAGAAATTT GGAAAAATAA CTAAATTTTA AATCCCGTAT   
  
  
+ AGAATGTTAA CGATCATTTT AATTTTATGT AATTATGTAA TTATAATCAA TACTTGATGT AAAAGTATTA   
  
  
+ ATATACCCTT AATATTAGGA TTTCTGACAA CAACCAAAAC ACAACCTTTA AACTACCTTA AAATGAGGAA   
  
  
+ ATAATCTATA TCACTTTTTA GTTTTCATTA TTCTCTTCAA CTTTTGAACT ACTTTCTGCA ACCTTTCGGA   
  
  
+ TTTTTGCAAA ACGATACAAC AGCCTTATTG TTACCTTTGA CTGCACTTTC ATTCTTCAAT CACATCAATT   
  
  
+ TTAAAATCTT ATTGGAAGTA CCGAGATACC TAGCAATTTA ATCTCGAAGA CTTTGTTTTT TTTATTCTTT   
  
  
+ TTTGTTTCAA TTCAGGCTTT CATTATGGT  

- ACTACTTATT TTGTCTTACT ATTAATTGTT TGTCTTTGTC AACACATATT AAATTTTTAC TTACCTCCCT   
  
  
- CCACCACCAC AAATATTCGC AACATTTTTA CGTCTCCACT CCTCTCACTA CTCACTCAAA ACAAAAGAAG   
  
  
- ATAAGAAAAA CACTTTTCTC TCACTTAACC AGCACCTTGC GCCAAACAAG CCTTAGAGTC CCTTTTCTGT   
  
  
- ACATGCGCCT TGCGTCGTAA TCTGTCTCTT TTTTCTCTCT TCGTCTGTTT CGGAACATAA GTCTCTTCCT   
  
  
- TCTTTCGATT TTCATGAGTT AATAGAGCAC CCCTCGTATG TTATTTTGGT GGTGTTAATA TAATATGTAC   
  
  
- ATGTATAAAT ACACAATATA TACATGCAAA TAAATAAATA AAATACATTT AACTATAAAC AATACTTTAA   
  
  
- TATACGCCTA CACCAATGCC AAACAAACTT ACACAATGTG CGTTCCTAAG TTTTCCACGA TCCGTGACCA   
  
  
- ACCCGCTGCC TATACACAGA TCCACCCTCA AATCCGTCTC GGATCCACCC CCAAATCCGT CAAAAAAAAA   
  
  
- AAATTAAAAT TAACTTAAAT AATATATTGT ATATTTATTG ACGGATGAAC ATGAATTTTT TTATGTAGTA   
  
  
- ACATAATTTT GCGAACTTAA CGTTTTATCT TACTATATAA CTAATATTTT ATAAGTTTGT ATAACTTTTA   
  
  
- TACTCTTTGT TGTATGTTAC TCACGAGTAG GTTTGTAAGT TCTTTAGAGC ATGTTAAATT ACTTTTTTAT   
  
  
- TTTACGTTCT ACCTTCAATA GAAAGAATTC ATTCTTAAAG CTGGATTCAC CCACCTGTCC AGATCCACCC   
  
  
- GCGGATCCAT CCGATCCACG GGAAAGAATT AAAATTTACG AATCCCTGAT TAGTACAGCC ATTGGTCGGC   
  
  
- GGATCGTGAT CCGTCTCTAA AAATCTTGTG ATCAATGTAC GTAATAAACA TTACCACTGA ATAATAAACT   
  
  
- TACAGTTCGA AAATACGTAA TAGTTAACAC AAAAATATAG GGCGTTTGAC TCTTAAATCG AACTCATCAA   
  
  
- TAAAATCAAA ATAAGCAGTA GATTGTCACA ACTCTTTAAA CCTTTTTATT GATTTAAAAT TTAGGGCATA   
  
  
- TCTTACAATT GCTAGTAAAA TTAAAATACA TTAATACATT AATATTAGTT ATGAACTACA TTTTCATAAT   
  
  
- TATATGGGAA TTATAATCCT AAAGACTGTT GTTGGTTTTG TGTTGGAAAT TTGATGGAAT TTTACTCCTT   
  
  
- TATTAGATAT AGTGAAAAAT CAAAAGTAAT AAGAGAAGTT GAAAACTTGA TGAAAGACGT TGGAAAGCCT   
  
  
- AAAAACGTTT TGCTATGTTG TCGGAATAAC AATGGAAACT GACGTGAAAG TAAGAAGTTA GTGTAGTTAA   
  
  
- AATTTTAGAA TAACCTTCAT GGCTCTATGG ATCGTTAAAT TAGAGCTTCT GAAACAAAAA AAATAAGAAA   
  
  
- AAACAAAGTT AAGTCCGAAA GTAATACCA

+     Myb

| Site Name | Organism | Position | Strand | Matrix score. | sequence | function |
| --- | --- | --- | --- | --- | --- | --- |
| Myb | Arabidopsis thaliana | 597 | + | 6 | TAACTG |  |
| Myb | Arabidopsis thaliana | 38 | - | 6 | CAACTG |  |

>006277   
+ TGATGAATAA AACAGAATGA TAATTAACAA ACAGAAACAG TTGTGTATAA TTTAAAAATG AATGGAGGGA   
  
  
+ GGTGGTGGTG TTTATAAGCG TTGTAAAAAT GCAGAGGTGA GGAGAGTGAT GAGTGAGTTT TGTTTTCTTC   
  
  
+ TATTCTTTTT GTGAAAAGAG AGTGAATTGG TCGTGGAACG CGGTTTGTTC GGAATCTCAG GGAAAAGACA   
  
  
+ TGTACGCGGA ACGCAGCATT AGACAGAGAA AAAAGAGAGA AGCAGACAAA GCCTTGTATT CAGAGAAGGA   
  
  
+ AGAAAGCTAA AAGTACTCAA TTATCTCGTG GGGAGCATAC AATAAAACCA CCACAATTAT ATTATACATG   
  
  
+ TACATATTTA TGTGTTATAT ATGTACGTTT ATTTATTTAT TTTATGTAAA TTGATATTTG TTATGAAATT   
  
  
+ ATATGCGGAT GTGGTTACGG TTTGTTTGAA TGTGTTACAC GCAAGGATTC AAAAGGTGCT AGGCACTGGT   
  
  
+ TGGGCGACGG ATATGTGTCT AGGTGGGAGT TTAGGCAGAG CCTAGGTGGG GGTTTAGGCA GTTTTTTTTT   
  
  
+ TTTAATTTTA ATTGAATTTA TTATATAACA TATAAATAAC TGCCTACTTG TACTTAAAAA AATACATCAT   
  
  
+ TGTATTAAAA CGCTTGAATT GCAAAATAGA ATGATATATT GATTATAAAA TATTCAAACA TATTGAAAAT   
  
  
+ ATGAGAAACA ACATACAATG AGTGCTCATC CAAACATTCA AGAAATCTCG TACAATTTAA TGAAAAAATA   
  
  
+ AAATGCAAGA TGGAAGTTAT CTTTCTTAAG TAAGAATTTC GACCTAAGTG GGTGGACAGG TCTAGGTGGG   
  
  
+ CGCCTAGGTA GGCTAGGTGC CCTTTCTTAA TTTTAAATGC TTAGGGACTA ATCATGTCGG TAACCAGCCG   
  
  
+ CCTAGCACTA GGCAGAGATT TTTAGAACAC TAGTTACATG CATTATTTGT AATGGTGACT TATTATTTGA   
  
  
+ ATGTCAAGCT TTTATGCATT ATCAATTGTG TTTTTATATC CCGCAAACTG AGAATTTAGC TTGAGTAGTT   
  
  
+ ATTTTAGTTT TATTCGTCAT CTAACAGTGT TGAGAAATTT GGAAAAATAA CTAAATTTTA AATCCCGTAT   
  
  
+ AGAATGTTAA CGATCATTTT AATTTTATGT AATTATGTAA TTATAATCAA TACTTGATGT AAAAGTATTA   
  
  
+ ATATACCCTT AATATTAGGA TTTCTGACAA CAACCAAAAC ACAACCTTTA AACTACCTTA AAATGAGGAA   
  
  
+ ATAATCTATA TCACTTTTTA GTTTTCATTA TTCTCTTCAA CTTTTGAACT ACTTTCTGCA ACCTTTCGGA   
  
  
+ TTTTTGCAAA ACGATACAAC AGCCTTATTG TTACCTTTGA CTGCACTTTC ATTCTTCAAT CACATCAATT   
  
  
+ TTAAAATCTT ATTGGAAGTA CCGAGATACC TAGCAATTTA ATCTCGAAGA CTTTGTTTTT TTTATTCTTT   
  
  
+ TTTGTTTCAA TTCAGGCTTT CATTATGGT  

- ACTACTTATT TTGTCTTACT ATTAATTGTT TGTCTTTGTC AACACATATT AAATTTTTAC TTACCTCCCT   
  
  
- CCACCACCAC AAATATTCGC AACATTTTTA CGTCTCCACT CCTCTCACTA CTCACTCAAA ACAAAAGAAG   
  
  
- ATAAGAAAAA CACTTTTCTC TCACTTAACC AGCACCTTGC GCCAAACAAG CCTTAGAGTC CCTTTTCTGT   
  
  
- ACATGCGCCT TGCGTCGTAA TCTGTCTCTT TTTTCTCTCT TCGTCTGTTT CGGAACATAA GTCTCTTCCT   
  
  
- TCTTTCGATT TTCATGAGTT AATAGAGCAC CCCTCGTATG TTATTTTGGT GGTGTTAATA TAATATGTAC   
  
  
- ATGTATAAAT ACACAATATA TACATGCAAA TAAATAAATA AAATACATTT AACTATAAAC AATACTTTAA   
  
  
- TATACGCCTA CACCAATGCC AAACAAACTT ACACAATGTG CGTTCCTAAG TTTTCCACGA TCCGTGACCA   
  
  
- ACCCGCTGCC TATACACAGA TCCACCCTCA AATCCGTCTC GGATCCACCC CCAAATCCGT CAAAAAAAAA   
  
  
- AAATTAAAAT TAACTTAAAT AATATATTGT ATATTTATTG ACGGATGAAC ATGAATTTTT TTATGTAGTA   
  
  
- ACATAATTTT GCGAACTTAA CGTTTTATCT TACTATATAA CTAATATTTT ATAAGTTTGT ATAACTTTTA   
  
  
- TACTCTTTGT TGTATGTTAC TCACGAGTAG GTTTGTAAGT TCTTTAGAGC ATGTTAAATT ACTTTTTTAT   
  
  
- TTTACGTTCT ACCTTCAATA GAAAGAATTC ATTCTTAAAG CTGGATTCAC CCACCTGTCC AGATCCACCC   
  
  
- GCGGATCCAT CCGATCCACG GGAAAGAATT AAAATTTACG AATCCCTGAT TAGTACAGCC ATTGGTCGGC   
  
  
- GGATCGTGAT CCGTCTCTAA AAATCTTGTG ATCAATGTAC GTAATAAACA TTACCACTGA ATAATAAACT   
  
  
- TACAGTTCGA AAATACGTAA TAGTTAACAC AAAAATATAG GGCGTTTGAC TCTTAAATCG AACTCATCAA   
  
  
- TAAAATCAAA ATAAGCAGTA GATTGTCACA ACTCTTTAAA CCTTTTTATT GATTTAAAAT TTAGGGCATA   
  
  
- TCTTACAATT GCTAGTAAAA TTAAAATACA TTAATACATT AATATTAGTT ATGAACTACA TTTTCATAAT   
  
  
- TATATGGGAA TTATAATCCT AAAGACTGTT GTTGGTTTTG TGTTGGAAAT TTGATGGAAT TTTACTCCTT   
  
  
- TATTAGATAT AGTGAAAAAT CAAAAGTAAT AAGAGAAGTT GAAAACTTGA TGAAAGACGT TGGAAAGCCT   
  
  
- AAAAACGTTT TGCTATGTTG TCGGAATAAC AATGGAAACT GACGTGAAAG TAAGAAGTTA GTGTAGTTAA   
  
  
- AATTTTAGAA TAACCTTCAT GGCTCTATGG ATCGTTAAAT TAGAGCTTCT GAAACAAAAA AAATAAGAAA   
  
  
- AAACAAAGTT AAGTCCGAAA GTAATACCA

+     Myb-binding site

| Site Name | Organism | Position | Strand | Matrix score. | sequence | function |
| --- | --- | --- | --- | --- | --- | --- |
| Myb-binding site | Nicotiana tabacum | 1347 | + | 6 | CAACAG |  |

>006277   
+ TGATGAATAA AACAGAATGA TAATTAACAA ACAGAAACAG TTGTGTATAA TTTAAAAATG AATGGAGGGA   
  
  
+ GGTGGTGGTG TTTATAAGCG TTGTAAAAAT GCAGAGGTGA GGAGAGTGAT GAGTGAGTTT TGTTTTCTTC   
  
  
+ TATTCTTTTT GTGAAAAGAG AGTGAATTGG TCGTGGAACG CGGTTTGTTC GGAATCTCAG GGAAAAGACA   
  
  
+ TGTACGCGGA ACGCAGCATT AGACAGAGAA AAAAGAGAGA AGCAGACAAA GCCTTGTATT CAGAGAAGGA   
  
  
+ AGAAAGCTAA AAGTACTCAA TTATCTCGTG GGGAGCATAC AATAAAACCA CCACAATTAT ATTATACATG   
  
  
+ TACATATTTA TGTGTTATAT ATGTACGTTT ATTTATTTAT TTTATGTAAA TTGATATTTG TTATGAAATT   
  
  
+ ATATGCGGAT GTGGTTACGG TTTGTTTGAA TGTGTTACAC GCAAGGATTC AAAAGGTGCT AGGCACTGGT   
  
  
+ TGGGCGACGG ATATGTGTCT AGGTGGGAGT TTAGGCAGAG CCTAGGTGGG GGTTTAGGCA GTTTTTTTTT   
  
  
+ TTTAATTTTA ATTGAATTTA TTATATAACA TATAAATAAC TGCCTACTTG TACTTAAAAA AATACATCAT   
  
  
+ TGTATTAAAA CGCTTGAATT GCAAAATAGA ATGATATATT GATTATAAAA TATTCAAACA TATTGAAAAT   
  
  
+ ATGAGAAACA ACATACAATG AGTGCTCATC CAAACATTCA AGAAATCTCG TACAATTTAA TGAAAAAATA   
  
  
+ AAATGCAAGA TGGAAGTTAT CTTTCTTAAG TAAGAATTTC GACCTAAGTG GGTGGACAGG TCTAGGTGGG   
  
  
+ CGCCTAGGTA GGCTAGGTGC CCTTTCTTAA TTTTAAATGC TTAGGGACTA ATCATGTCGG TAACCAGCCG   
  
  
+ CCTAGCACTA GGCAGAGATT TTTAGAACAC TAGTTACATG CATTATTTGT AATGGTGACT TATTATTTGA   
  
  
+ ATGTCAAGCT TTTATGCATT ATCAATTGTG TTTTTATATC CCGCAAACTG AGAATTTAGC TTGAGTAGTT   
  
  
+ ATTTTAGTTT TATTCGTCAT CTAACAGTGT TGAGAAATTT GGAAAAATAA CTAAATTTTA AATCCCGTAT   
  
  
+ AGAATGTTAA CGATCATTTT AATTTTATGT AATTATGTAA TTATAATCAA TACTTGATGT AAAAGTATTA   
  
  
+ ATATACCCTT AATATTAGGA TTTCTGACAA CAACCAAAAC ACAACCTTTA AACTACCTTA AAATGAGGAA   
  
  
+ ATAATCTATA TCACTTTTTA GTTTTCATTA TTCTCTTCAA CTTTTGAACT ACTTTCTGCA ACCTTTCGGA   
  
  
+ TTTTTGCAAA ACGATACAAC AGCCTTATTG TTACCTTTGA CTGCACTTTC ATTCTTCAAT CACATCAATT   
  
  
+ TTAAAATCTT ATTGGAAGTA CCGAGATACC TAGCAATTTA ATCTCGAAGA CTTTGTTTTT TTTATTCTTT   
  
  
+ TTTGTTTCAA TTCAGGCTTT CATTATGGT  

- ACTACTTATT TTGTCTTACT ATTAATTGTT TGTCTTTGTC AACACATATT AAATTTTTAC TTACCTCCCT   
  
  
- CCACCACCAC AAATATTCGC AACATTTTTA CGTCTCCACT CCTCTCACTA CTCACTCAAA ACAAAAGAAG   
  
  
- ATAAGAAAAA CACTTTTCTC TCACTTAACC AGCACCTTGC GCCAAACAAG CCTTAGAGTC CCTTTTCTGT   
  
  
- ACATGCGCCT TGCGTCGTAA TCTGTCTCTT TTTTCTCTCT TCGTCTGTTT CGGAACATAA GTCTCTTCCT   
  
  
- TCTTTCGATT TTCATGAGTT AATAGAGCAC CCCTCGTATG TTATTTTGGT GGTGTTAATA TAATATGTAC   
  
  
- ATGTATAAAT ACACAATATA TACATGCAAA TAAATAAATA AAATACATTT AACTATAAAC AATACTTTAA   
  
  
- TATACGCCTA CACCAATGCC AAACAAACTT ACACAATGTG CGTTCCTAAG TTTTCCACGA TCCGTGACCA   
  
  
- ACCCGCTGCC TATACACAGA TCCACCCTCA AATCCGTCTC GGATCCACCC CCAAATCCGT CAAAAAAAAA   
  
  
- AAATTAAAAT TAACTTAAAT AATATATTGT ATATTTATTG ACGGATGAAC ATGAATTTTT TTATGTAGTA   
  
  
- ACATAATTTT GCGAACTTAA CGTTTTATCT TACTATATAA CTAATATTTT ATAAGTTTGT ATAACTTTTA   
  
  
- TACTCTTTGT TGTATGTTAC TCACGAGTAG GTTTGTAAGT TCTTTAGAGC ATGTTAAATT ACTTTTTTAT   
  
  
- TTTACGTTCT ACCTTCAATA GAAAGAATTC ATTCTTAAAG CTGGATTCAC CCACCTGTCC AGATCCACCC   
  
  
- GCGGATCCAT CCGATCCACG GGAAAGAATT AAAATTTACG AATCCCTGAT TAGTACAGCC ATTGGTCGGC   
  
  
- GGATCGTGAT CCGTCTCTAA AAATCTTGTG ATCAATGTAC GTAATAAACA TTACCACTGA ATAATAAACT   
  
  
- TACAGTTCGA AAATACGTAA TAGTTAACAC AAAAATATAG GGCGTTTGAC TCTTAAATCG AACTCATCAA   
  
  
- TAAAATCAAA ATAAGCAGTA GATTGTCACA ACTCTTTAAA CCTTTTTATT GATTTAAAAT TTAGGGCATA   
  
  
- TCTTACAATT GCTAGTAAAA TTAAAATACA TTAATACATT AATATTAGTT ATGAACTACA TTTTCATAAT   
  
  
- TATATGGGAA TTATAATCCT AAAGACTGTT GTTGGTTTTG TGTTGGAAAT TTGATGGAAT TTTACTCCTT   
  
  
- TATTAGATAT AGTGAAAAAT CAAAAGTAAT AAGAGAAGTT GAAAACTTGA TGAAAGACGT TGGAAAGCCT   
  
  
- AAAAACGTTT TGCTATGTTG TCGGAATAAC AATGGAAACT GACGTGAAAG TAAGAAGTTA GTGTAGTTAA   
  
  
- AATTTTAGAA TAACCTTCAT GGCTCTATGG ATCGTTAAAT TAGAGCTTCT GAAACAAAAA AAATAAGAAA   
  
  
- AAACAAAGTT AAGTCCGAAA GTAATACCA

+     P-box

| Site Name | Organism | Position | Strand | Matrix score. | sequence | function |
| --- | --- | --- | --- | --- | --- | --- |
| P-box | Oryza sativa | 470 | - | 7 | CCTTTTG | gibberellin-responsive element |

>006277   
+ TGATGAATAA AACAGAATGA TAATTAACAA ACAGAAACAG TTGTGTATAA TTTAAAAATG AATGGAGGGA   
  
  
+ GGTGGTGGTG TTTATAAGCG TTGTAAAAAT GCAGAGGTGA GGAGAGTGAT GAGTGAGTTT TGTTTTCTTC   
  
  
+ TATTCTTTTT GTGAAAAGAG AGTGAATTGG TCGTGGAACG CGGTTTGTTC GGAATCTCAG GGAAAAGACA   
  
  
+ TGTACGCGGA ACGCAGCATT AGACAGAGAA AAAAGAGAGA AGCAGACAAA GCCTTGTATT CAGAGAAGGA   
  
  
+ AGAAAGCTAA AAGTACTCAA TTATCTCGTG GGGAGCATAC AATAAAACCA CCACAATTAT ATTATACATG   
  
  
+ TACATATTTA TGTGTTATAT ATGTACGTTT ATTTATTTAT TTTATGTAAA TTGATATTTG TTATGAAATT   
  
  
+ ATATGCGGAT GTGGTTACGG TTTGTTTGAA TGTGTTACAC GCAAGGATTC AAAAGGTGCT AGGCACTGGT   
  
  
+ TGGGCGACGG ATATGTGTCT AGGTGGGAGT TTAGGCAGAG CCTAGGTGGG GGTTTAGGCA GTTTTTTTTT   
  
  
+ TTTAATTTTA ATTGAATTTA TTATATAACA TATAAATAAC TGCCTACTTG TACTTAAAAA AATACATCAT   
  
  
+ TGTATTAAAA CGCTTGAATT GCAAAATAGA ATGATATATT GATTATAAAA TATTCAAACA TATTGAAAAT   
  
  
+ ATGAGAAACA ACATACAATG AGTGCTCATC CAAACATTCA AGAAATCTCG TACAATTTAA TGAAAAAATA   
  
  
+ AAATGCAAGA TGGAAGTTAT CTTTCTTAAG TAAGAATTTC GACCTAAGTG GGTGGACAGG TCTAGGTGGG   
  
  
+ CGCCTAGGTA GGCTAGGTGC CCTTTCTTAA TTTTAAATGC TTAGGGACTA ATCATGTCGG TAACCAGCCG   
  
  
+ CCTAGCACTA GGCAGAGATT TTTAGAACAC TAGTTACATG CATTATTTGT AATGGTGACT TATTATTTGA   
  
  
+ ATGTCAAGCT TTTATGCATT ATCAATTGTG TTTTTATATC CCGCAAACTG AGAATTTAGC TTGAGTAGTT   
  
  
+ ATTTTAGTTT TATTCGTCAT CTAACAGTGT TGAGAAATTT GGAAAAATAA CTAAATTTTA AATCCCGTAT   
  
  
+ AGAATGTTAA CGATCATTTT AATTTTATGT AATTATGTAA TTATAATCAA TACTTGATGT AAAAGTATTA   
  
  
+ ATATACCCTT AATATTAGGA TTTCTGACAA CAACCAAAAC ACAACCTTTA AACTACCTTA AAATGAGGAA   
  
  
+ ATAATCTATA TCACTTTTTA GTTTTCATTA TTCTCTTCAA CTTTTGAACT ACTTTCTGCA ACCTTTCGGA   
  
  
+ TTTTTGCAAA ACGATACAAC AGCCTTATTG TTACCTTTGA CTGCACTTTC ATTCTTCAAT CACATCAATT   
  
  
+ TTAAAATCTT ATTGGAAGTA CCGAGATACC TAGCAATTTA ATCTCGAAGA CTTTGTTTTT TTTATTCTTT   
  
  
+ TTTGTTTCAA TTCAGGCTTT CATTATGGT  

- ACTACTTATT TTGTCTTACT ATTAATTGTT TGTCTTTGTC AACACATATT AAATTTTTAC TTACCTCCCT   
  
  
- CCACCACCAC AAATATTCGC AACATTTTTA CGTCTCCACT CCTCTCACTA CTCACTCAAA ACAAAAGAAG   
  
  
- ATAAGAAAAA CACTTTTCTC TCACTTAACC AGCACCTTGC GCCAAACAAG CCTTAGAGTC CCTTTTCTGT   
  
  
- ACATGCGCCT TGCGTCGTAA TCTGTCTCTT TTTTCTCTCT TCGTCTGTTT CGGAACATAA GTCTCTTCCT   
  
  
- TCTTTCGATT TTCATGAGTT AATAGAGCAC CCCTCGTATG TTATTTTGGT GGTGTTAATA TAATATGTAC   
  
  
- ATGTATAAAT ACACAATATA TACATGCAAA TAAATAAATA AAATACATTT AACTATAAAC AATACTTTAA   
  
  
- TATACGCCTA CACCAATGCC AAACAAACTT ACACAATGTG CGTTCCTAAG TTTTCCACGA TCCGTGACCA   
  
  
- ACCCGCTGCC TATACACAGA TCCACCCTCA AATCCGTCTC GGATCCACCC CCAAATCCGT CAAAAAAAAA   
  
  
- AAATTAAAAT TAACTTAAAT AATATATTGT ATATTTATTG ACGGATGAAC ATGAATTTTT TTATGTAGTA   
  
  
- ACATAATTTT GCGAACTTAA CGTTTTATCT TACTATATAA CTAATATTTT ATAAGTTTGT ATAACTTTTA   
  
  
- TACTCTTTGT TGTATGTTAC TCACGAGTAG GTTTGTAAGT TCTTTAGAGC ATGTTAAATT ACTTTTTTAT   
  
  
- TTTACGTTCT ACCTTCAATA GAAAGAATTC ATTCTTAAAG CTGGATTCAC CCACCTGTCC AGATCCACCC   
  
  
- GCGGATCCAT CCGATCCACG GGAAAGAATT AAAATTTACG AATCCCTGAT TAGTACAGCC ATTGGTCGGC   
  
  
- GGATCGTGAT CCGTCTCTAA AAATCTTGTG ATCAATGTAC GTAATAAACA TTACCACTGA ATAATAAACT   
  
  
- TACAGTTCGA AAATACGTAA TAGTTAACAC AAAAATATAG GGCGTTTGAC TCTTAAATCG AACTCATCAA   
  
  
- TAAAATCAAA ATAAGCAGTA GATTGTCACA ACTCTTTAAA CCTTTTTATT GATTTAAAAT TTAGGGCATA   
  
  
- TCTTACAATT GCTAGTAAAA TTAAAATACA TTAATACATT AATATTAGTT ATGAACTACA TTTTCATAAT   
  
  
- TATATGGGAA TTATAATCCT AAAGACTGTT GTTGGTTTTG TGTTGGAAAT TTGATGGAAT TTTACTCCTT   
  
  
- TATTAGATAT AGTGAAAAAT CAAAAGTAAT AAGAGAAGTT GAAAACTTGA TGAAAGACGT TGGAAAGCCT   
  
  
- AAAAACGTTT TGCTATGTTG TCGGAATAAC AATGGAAACT GACGTGAAAG TAAGAAGTTA GTGTAGTTAA   
  
  
- AATTTTAGAA TAACCTTCAT GGCTCTATGG ATCGTTAAAT TAGAGCTTCT GAAACAAAAA AAATAAGAAA   
  
  
- AAACAAAGTT AAGTCCGAAA GTAATACCA

+     TATA

| Site Name | Organism | Position | Strand | Matrix score. | sequence | function |
| --- | --- | --- | --- | --- | --- | --- |
| TATA | Arabidopsis thaliana | 674 | + | 8 | TATAAAAT |  |

>006277   
+ TGATGAATAA AACAGAATGA TAATTAACAA ACAGAAACAG TTGTGTATAA TTTAAAAATG AATGGAGGGA   
  
  
+ GGTGGTGGTG TTTATAAGCG TTGTAAAAAT GCAGAGGTGA GGAGAGTGAT GAGTGAGTTT TGTTTTCTTC   
  
  
+ TATTCTTTTT GTGAAAAGAG AGTGAATTGG TCGTGGAACG CGGTTTGTTC GGAATCTCAG GGAAAAGACA   
  
  
+ TGTACGCGGA ACGCAGCATT AGACAGAGAA AAAAGAGAGA AGCAGACAAA GCCTTGTATT CAGAGAAGGA   
  
  
+ AGAAAGCTAA AAGTACTCAA TTATCTCGTG GGGAGCATAC AATAAAACCA CCACAATTAT ATTATACATG   
  
  
+ TACATATTTA TGTGTTATAT ATGTACGTTT ATTTATTTAT TTTATGTAAA TTGATATTTG TTATGAAATT   
  
  
+ ATATGCGGAT GTGGTTACGG TTTGTTTGAA TGTGTTACAC GCAAGGATTC AAAAGGTGCT AGGCACTGGT   
  
  
+ TGGGCGACGG ATATGTGTCT AGGTGGGAGT TTAGGCAGAG CCTAGGTGGG GGTTTAGGCA GTTTTTTTTT   
  
  
+ TTTAATTTTA ATTGAATTTA TTATATAACA TATAAATAAC TGCCTACTTG TACTTAAAAA AATACATCAT   
  
  
+ TGTATTAAAA CGCTTGAATT GCAAAATAGA ATGATATATT GATTATAAAA TATTCAAACA TATTGAAAAT   
  
  
+ ATGAGAAACA ACATACAATG AGTGCTCATC CAAACATTCA AGAAATCTCG TACAATTTAA TGAAAAAATA   
  
  
+ AAATGCAAGA TGGAAGTTAT CTTTCTTAAG TAAGAATTTC GACCTAAGTG GGTGGACAGG TCTAGGTGGG   
  
  
+ CGCCTAGGTA GGCTAGGTGC CCTTTCTTAA TTTTAAATGC TTAGGGACTA ATCATGTCGG TAACCAGCCG   
  
  
+ CCTAGCACTA GGCAGAGATT TTTAGAACAC TAGTTACATG CATTATTTGT AATGGTGACT TATTATTTGA   
  
  
+ ATGTCAAGCT TTTATGCATT ATCAATTGTG TTTTTATATC CCGCAAACTG AGAATTTAGC TTGAGTAGTT   
  
  
+ ATTTTAGTTT TATTCGTCAT CTAACAGTGT TGAGAAATTT GGAAAAATAA CTAAATTTTA AATCCCGTAT   
  
  
+ AGAATGTTAA CGATCATTTT AATTTTATGT AATTATGTAA TTATAATCAA TACTTGATGT AAAAGTATTA   
  
  
+ ATATACCCTT AATATTAGGA TTTCTGACAA CAACCAAAAC ACAACCTTTA AACTACCTTA AAATGAGGAA   
  
  
+ ATAATCTATA TCACTTTTTA GTTTTCATTA TTCTCTTCAA CTTTTGAACT ACTTTCTGCA ACCTTTCGGA   
  
  
+ TTTTTGCAAA ACGATACAAC AGCCTTATTG TTACCTTTGA CTGCACTTTC ATTCTTCAAT CACATCAATT   
  
  
+ TTAAAATCTT ATTGGAAGTA CCGAGATACC TAGCAATTTA ATCTCGAAGA CTTTGTTTTT TTTATTCTTT   
  
  
+ TTTGTTTCAA TTCAGGCTTT CATTATGGT  

- ACTACTTATT TTGTCTTACT ATTAATTGTT TGTCTTTGTC AACACATATT AAATTTTTAC TTACCTCCCT   
  
  
- CCACCACCAC AAATATTCGC AACATTTTTA CGTCTCCACT CCTCTCACTA CTCACTCAAA ACAAAAGAAG   
  
  
- ATAAGAAAAA CACTTTTCTC TCACTTAACC AGCACCTTGC GCCAAACAAG CCTTAGAGTC CCTTTTCTGT   
  
  
- ACATGCGCCT TGCGTCGTAA TCTGTCTCTT TTTTCTCTCT TCGTCTGTTT CGGAACATAA GTCTCTTCCT   
  
  
- TCTTTCGATT TTCATGAGTT AATAGAGCAC CCCTCGTATG TTATTTTGGT GGTGTTAATA TAATATGTAC   
  
  
- ATGTATAAAT ACACAATATA TACATGCAAA TAAATAAATA AAATACATTT AACTATAAAC AATACTTTAA   
  
  
- TATACGCCTA CACCAATGCC AAACAAACTT ACACAATGTG CGTTCCTAAG TTTTCCACGA TCCGTGACCA   
  
  
- ACCCGCTGCC TATACACAGA TCCACCCTCA AATCCGTCTC GGATCCACCC CCAAATCCGT CAAAAAAAAA   
  
  
- AAATTAAAAT TAACTTAAAT AATATATTGT ATATTTATTG ACGGATGAAC ATGAATTTTT TTATGTAGTA   
  
  
- ACATAATTTT GCGAACTTAA CGTTTTATCT TACTATATAA CTAATATTTT ATAAGTTTGT ATAACTTTTA   
  
  
- TACTCTTTGT TGTATGTTAC TCACGAGTAG GTTTGTAAGT TCTTTAGAGC ATGTTAAATT ACTTTTTTAT   
  
  
- TTTACGTTCT ACCTTCAATA GAAAGAATTC ATTCTTAAAG CTGGATTCAC CCACCTGTCC AGATCCACCC   
  
  
- GCGGATCCAT CCGATCCACG GGAAAGAATT AAAATTTACG AATCCCTGAT TAGTACAGCC ATTGGTCGGC   
  
  
- GGATCGTGAT CCGTCTCTAA AAATCTTGTG ATCAATGTAC GTAATAAACA TTACCACTGA ATAATAAACT   
  
  
- TACAGTTCGA AAATACGTAA TAGTTAACAC AAAAATATAG GGCGTTTGAC TCTTAAATCG AACTCATCAA   
  
  
- TAAAATCAAA ATAAGCAGTA GATTGTCACA ACTCTTTAAA CCTTTTTATT GATTTAAAAT TTAGGGCATA   
  
  
- TCTTACAATT GCTAGTAAAA TTAAAATACA TTAATACATT AATATTAGTT ATGAACTACA TTTTCATAAT   
  
  
- TATATGGGAA TTATAATCCT AAAGACTGTT GTTGGTTTTG TGTTGGAAAT TTGATGGAAT TTTACTCCTT   
  
  
- TATTAGATAT AGTGAAAAAT CAAAAGTAAT AAGAGAAGTT GAAAACTTGA TGAAAGACGT TGGAAAGCCT   
  
  
- AAAAACGTTT TGCTATGTTG TCGGAATAAC AATGGAAACT GACGTGAAAG TAAGAAGTTA GTGTAGTTAA   
  
  
- AATTTTAGAA TAACCTTCAT GGCTCTATGG ATCGTTAAAT TAGAGCTTCT GAAACAAAAA AAATAAGAAA   
  
  
- AAACAAAGTT AAGTCCGAAA GTAATACCA

+     TATA-box

| Site Name | Organism | Position | Strand | Matrix score. | sequence | function |
| --- | --- | --- | --- | --- | --- | --- |
| TATA-box | Arabidopsis thaliana | 1267 | - | 4 | TATA | core promoter element around -30 of transcription start |
| TATA-box | Arabidopsis thaliana | 1192 | - | 4 | TATA | core promoter element around -30 of transcription start |
| TATA-box | Arabidopsis thaliana | 1162 | - | 4 | TATA | core promoter element around -30 of transcription start |
| TATA-box | Arabidopsis thaliana | 1161 | - | 5 | TATAA | core promoter element around -30 of transcription start |
| TATA-box | Arabidopsis thaliana | 581 | - | 7 | TATATAA | core promoter element around -30 of transcription start |
| TATA-box | Brassica napus | 664 | + | 6 | ATATAT | core promoter element around -30 of transcription start |
| TATA-box | Arabidopsis thaliana | 1015 | - | 4 | TATA | core promoter element around -30 of transcription start |
| TATA-box | Arabidopsis thaliana | 591 | + | 4 | TATA | core promoter element around -30 of transcription start |
| TATA-box | Arabidopsis thaliana | 1118 | - | 4 | TATA | core promoter element around -30 of transcription start |
| TATA-box | Oryza sativa | 1144 | - | 8 | TACATAAA | core promoter element around -30 of transcription start |
| TATA-box | Brassica napus | 1160 | + | 6 | ATTATA | core promoter element around -30 of transcription start |
| TATA-box | Arabidopsis thaliana | 1014 | - | 5 | TATAA | core promoter element around -30 of transcription start |
| TATA-box | Helianthus annuus | 1013 | - | 6 | TATAAA | core promoter element around -30 of transcription start |
| TATA-box | Arabidopsis thaliana | 582 | + | 6 | TATATA | core promoter element around -30 of transcription start |
| TATA-box | Brassica napus | 580 | + | 6 | ATTATA | core promoter element around -30 of transcription start |
| TATA-box | Arabidopsis thaliana | 420 | + | 4 | TATA | core promoter element around -30 of transcription start |
| TATA-box | Arabidopsis thaliana | 665 | + | 4 | TATA | core promoter element around -30 of transcription start |
| TATA-box | Brassica oleracea | 590 | + | 6 | ATATAA | core promoter element around -30 of transcription start |
| TATA-box | Arabidopsis thaliana | 584 | + | 4 | TATA | core promoter element around -30 of transcription start |
| TATA-box | Brassica oleracea | 583 | + | 6 | ATATAA | core promoter element around -30 of transcription start |
| TATA-box | Arabidopsis thaliana | 674 | + | 4 | TATA | core promoter element around -30 of transcription start |
| TATA-box | Brassica napus | 672 | + | 6 | ATTATA | core promoter element around -30 of transcription start |
| TATA-box | Pisum sativum | 1012 | - | 7 | TATAAAA | core promoter element around -30 of transcription start |
| TATA-box | Arabidopsis thaliana | 673 | - | 5 | TATAA | core promoter element around -30 of transcription start |
| TATA-box | Arabidopsis thaliana | 368 | + | 4 | TATA | core promoter element around -30 of transcription start |
| TATA-box | Oryza sativa | 391 | - | 8 | TACATAAA | core promoter element around -30 of transcription start |
| TATA-box | Brassica napus | 418 | + | 6 | ATTATA | core promoter element around -30 of transcription start |
| TATA-box | Arabidopsis thaliana | 419 | - | 5 | TATAA | core promoter element around -30 of transcription start |
| TATA-box | Brassica napus | 367 | + | 6 | ATATAT | core promoter element around -30 of transcription start |
| TATA-box | Arabidopsis thaliana | 366 | + | 6 | TATATA | core promoter element around -30 of transcription start |
| TATA-box | Arabidopsis thaliana | 343 | + | 4 | TATA | core promoter element around -30 of transcription start |
| TATA-box | Arabidopsis thaliana | 82 | - | 5 | TATAA | core promoter element around -30 of transcription start |
| TATA-box | Arabidopsis thaliana | 365 | - | 7 | TATATAA | core promoter element around -30 of transcription start |
| TATA-box | Arabidopsis thaliana | 342 | - | 5 | TATAA | core promoter element around -30 of transcription start |
| TATA-box | Brassica napus | 341 | + | 6 | ATTATA | core promoter element around -30 of transcription start |
| TATA-box | Arabidopsis thaliana | 338 | + | 4 | TATA | core promoter element around -30 of transcription start |
| TATA-box | Arabidopsis thaliana | 337 | - | 5 | TATAA | core promoter element around -30 of transcription start |
| TATA-box | Brassica napus | 336 | + | 6 | ATTATA | core promoter element around -30 of transcription start |
| TATA-box | Arabidopsis thaliana | 83 | + | 4 | TATA | core promoter element around -30 of transcription start |
| TATA-box | Arabidopsis thaliana | 46 | + | 4 | TATA | core promoter element around -30 of transcription start |
| TATA-box | Helianthus annuus | 81 | - | 6 | TATAAA | core promoter element around -30 of transcription start |
| TATA-box | Helianthus annuus | 44 | - | 6 | TATACA | core promoter element around -30 of transcription start |

>006277   
+ TGATGAATAA AACAGAATGA TAATTAACAA ACAGAAACAG TTGTGTATAA TTTAAAAATG AATGGAGGGA   
  
  
+ GGTGGTGGTG TTTATAAGCG TTGTAAAAAT GCAGAGGTGA GGAGAGTGAT GAGTGAGTTT TGTTTTCTTC   
  
  
+ TATTCTTTTT GTGAAAAGAG AGTGAATTGG TCGTGGAACG CGGTTTGTTC GGAATCTCAG GGAAAAGACA   
  
  
+ TGTACGCGGA ACGCAGCATT AGACAGAGAA AAAAGAGAGA AGCAGACAAA GCCTTGTATT CAGAGAAGGA   
  
  
+ AGAAAGCTAA AAGTACTCAA TTATCTCGTG GGGAGCATAC AATAAAACCA CCACAATTAT ATTATACATG   
  
  
+ TACATATTTA TGTGTTATAT ATGTACGTTT ATTTATTTAT TTTATGTAAA TTGATATTTG TTATGAAATT   
  
  
+ ATATGCGGAT GTGGTTACGG TTTGTTTGAA TGTGTTACAC GCAAGGATTC AAAAGGTGCT AGGCACTGGT   
  
  
+ TGGGCGACGG ATATGTGTCT AGGTGGGAGT TTAGGCAGAG CCTAGGTGGG GGTTTAGGCA GTTTTTTTTT   
  
  
+ TTTAATTTTA ATTGAATTTA TTATATAACA TATAAATAAC TGCCTACTTG TACTTAAAAA AATACATCAT   
  
  
+ TGTATTAAAA CGCTTGAATT GCAAAATAGA ATGATATATT GATTATAAAA TATTCAAACA TATTGAAAAT   
  
  
+ ATGAGAAACA ACATACAATG AGTGCTCATC CAAACATTCA AGAAATCTCG TACAATTTAA TGAAAAAATA   
  
  
+ AAATGCAAGA TGGAAGTTAT CTTTCTTAAG TAAGAATTTC GACCTAAGTG GGTGGACAGG TCTAGGTGGG   
  
  
+ CGCCTAGGTA GGCTAGGTGC CCTTTCTTAA TTTTAAATGC TTAGGGACTA ATCATGTCGG TAACCAGCCG   
  
  
+ CCTAGCACTA GGCAGAGATT TTTAGAACAC TAGTTACATG CATTATTTGT AATGGTGACT TATTATTTGA   
  
  
+ ATGTCAAGCT TTTATGCATT ATCAATTGTG TTTTTATATC CCGCAAACTG AGAATTTAGC TTGAGTAGTT   
  
  
+ ATTTTAGTTT TATTCGTCAT CTAACAGTGT TGAGAAATTT GGAAAAATAA CTAAATTTTA AATCCCGTAT   
  
  
+ AGAATGTTAA CGATCATTTT AATTTTATGT AATTATGTAA TTATAATCAA TACTTGATGT AAAAGTATTA   
  
  
+ ATATACCCTT AATATTAGGA TTTCTGACAA CAACCAAAAC ACAACCTTTA AACTACCTTA AAATGAGGAA   
  
  
+ ATAATCTATA TCACTTTTTA GTTTTCATTA TTCTCTTCAA CTTTTGAACT ACTTTCTGCA ACCTTTCGGA   
  
  
+ TTTTTGCAAA ACGATACAAC AGCCTTATTG TTACCTTTGA CTGCACTTTC ATTCTTCAAT CACATCAATT   
  
  
+ TTAAAATCTT ATTGGAAGTA CCGAGATACC TAGCAATTTA ATCTCGAAGA CTTTGTTTTT TTTATTCTTT   
  
  
+ TTTGTTTCAA TTCAGGCTTT CATTATGGT  

- ACTACTTATT TTGTCTTACT ATTAATTGTT TGTCTTTGTC AACACATATT AAATTTTTAC TTACCTCCCT   
  
  
- CCACCACCAC AAATATTCGC AACATTTTTA CGTCTCCACT CCTCTCACTA CTCACTCAAA ACAAAAGAAG   
  
  
- ATAAGAAAAA CACTTTTCTC TCACTTAACC AGCACCTTGC GCCAAACAAG CCTTAGAGTC CCTTTTCTGT   
  
  
- ACATGCGCCT TGCGTCGTAA TCTGTCTCTT TTTTCTCTCT TCGTCTGTTT CGGAACATAA GTCTCTTCCT   
  
  
- TCTTTCGATT TTCATGAGTT AATAGAGCAC CCCTCGTATG TTATTTTGGT GGTGTTAATA TAATATGTAC   
  
  
- ATGTATAAAT ACACAATATA TACATGCAAA TAAATAAATA AAATACATTT AACTATAAAC AATACTTTAA   
  
  
- TATACGCCTA CACCAATGCC AAACAAACTT ACACAATGTG CGTTCCTAAG TTTTCCACGA TCCGTGACCA   
  
  
- ACCCGCTGCC TATACACAGA TCCACCCTCA AATCCGTCTC GGATCCACCC CCAAATCCGT CAAAAAAAAA   
  
  
- AAATTAAAAT TAACTTAAAT AATATATTGT ATATTTATTG ACGGATGAAC ATGAATTTTT TTATGTAGTA   
  
  
- ACATAATTTT GCGAACTTAA CGTTTTATCT TACTATATAA CTAATATTTT ATAAGTTTGT ATAACTTTTA   
  
  
- TACTCTTTGT TGTATGTTAC TCACGAGTAG GTTTGTAAGT TCTTTAGAGC ATGTTAAATT ACTTTTTTAT   
  
  
- TTTACGTTCT ACCTTCAATA GAAAGAATTC ATTCTTAAAG CTGGATTCAC CCACCTGTCC AGATCCACCC   
  
  
- GCGGATCCAT CCGATCCACG GGAAAGAATT AAAATTTACG AATCCCTGAT TAGTACAGCC ATTGGTCGGC   
  
  
- GGATCGTGAT CCGTCTCTAA AAATCTTGTG ATCAATGTAC GTAATAAACA TTACCACTGA ATAATAAACT   
  
  
- TACAGTTCGA AAATACGTAA TAGTTAACAC AAAAATATAG GGCGTTTGAC TCTTAAATCG AACTCATCAA   
  
  
- TAAAATCAAA ATAAGCAGTA GATTGTCACA ACTCTTTAAA CCTTTTTATT GATTTAAAAT TTAGGGCATA   
  
  
- TCTTACAATT GCTAGTAAAA TTAAAATACA TTAATACATT AATATTAGTT ATGAACTACA TTTTCATAAT   
  
  
- TATATGGGAA TTATAATCCT AAAGACTGTT GTTGGTTTTG TGTTGGAAAT TTGATGGAAT TTTACTCCTT   
  
  
- TATTAGATAT AGTGAAAAAT CAAAAGTAAT AAGAGAAGTT GAAAACTTGA TGAAAGACGT TGGAAAGCCT   
  
  
- AAAAACGTTT TGCTATGTTG TCGGAATAAC AATGGAAACT GACGTGAAAG TAAGAAGTTA GTGTAGTTAA   
  
  
- AATTTTAGAA TAACCTTCAT GGCTCTATGG ATCGTTAAAT TAGAGCTTCT GAAACAAAAA AAATAAGAAA   
  
  
- AAACAAAGTT AAGTCCGAAA GTAATACCA

+     TCT-motif

| Site Name | Organism | Position | Strand | Matrix score. | sequence | function |
| --- | --- | --- | --- | --- | --- | --- |
| TCT-motif | Arabidopsis thaliana | 800 | - | 6 | TCTTAC | part of a light responsive element |

>006277   
+ TGATGAATAA AACAGAATGA TAATTAACAA ACAGAAACAG TTGTGTATAA TTTAAAAATG AATGGAGGGA   
  
  
+ GGTGGTGGTG TTTATAAGCG TTGTAAAAAT GCAGAGGTGA GGAGAGTGAT GAGTGAGTTT TGTTTTCTTC   
  
  
+ TATTCTTTTT GTGAAAAGAG AGTGAATTGG TCGTGGAACG CGGTTTGTTC GGAATCTCAG GGAAAAGACA   
  
  
+ TGTACGCGGA ACGCAGCATT AGACAGAGAA AAAAGAGAGA AGCAGACAAA GCCTTGTATT CAGAGAAGGA   
  
  
+ AGAAAGCTAA AAGTACTCAA TTATCTCGTG GGGAGCATAC AATAAAACCA CCACAATTAT ATTATACATG   
  
  
+ TACATATTTA TGTGTTATAT ATGTACGTTT ATTTATTTAT TTTATGTAAA TTGATATTTG TTATGAAATT   
  
  
+ ATATGCGGAT GTGGTTACGG TTTGTTTGAA TGTGTTACAC GCAAGGATTC AAAAGGTGCT AGGCACTGGT   
  
  
+ TGGGCGACGG ATATGTGTCT AGGTGGGAGT TTAGGCAGAG CCTAGGTGGG GGTTTAGGCA GTTTTTTTTT   
  
  
+ TTTAATTTTA ATTGAATTTA TTATATAACA TATAAATAAC TGCCTACTTG TACTTAAAAA AATACATCAT   
  
  
+ TGTATTAAAA CGCTTGAATT GCAAAATAGA ATGATATATT GATTATAAAA TATTCAAACA TATTGAAAAT   
  
  
+ ATGAGAAACA ACATACAATG AGTGCTCATC CAAACATTCA AGAAATCTCG TACAATTTAA TGAAAAAATA   
  
  
+ AAATGCAAGA TGGAAGTTAT CTTTCTTAAG TAAGAATTTC GACCTAAGTG GGTGGACAGG TCTAGGTGGG   
  
  
+ CGCCTAGGTA GGCTAGGTGC CCTTTCTTAA TTTTAAATGC TTAGGGACTA ATCATGTCGG TAACCAGCCG   
  
  
+ CCTAGCACTA GGCAGAGATT TTTAGAACAC TAGTTACATG CATTATTTGT AATGGTGACT TATTATTTGA   
  
  
+ ATGTCAAGCT TTTATGCATT ATCAATTGTG TTTTTATATC CCGCAAACTG AGAATTTAGC TTGAGTAGTT   
  
  
+ ATTTTAGTTT TATTCGTCAT CTAACAGTGT TGAGAAATTT GGAAAAATAA CTAAATTTTA AATCCCGTAT   
  
  
+ AGAATGTTAA CGATCATTTT AATTTTATGT AATTATGTAA TTATAATCAA TACTTGATGT AAAAGTATTA   
  
  
+ ATATACCCTT AATATTAGGA TTTCTGACAA CAACCAAAAC ACAACCTTTA AACTACCTTA AAATGAGGAA   
  
  
+ ATAATCTATA TCACTTTTTA GTTTTCATTA TTCTCTTCAA CTTTTGAACT ACTTTCTGCA ACCTTTCGGA   
  
  
+ TTTTTGCAAA ACGATACAAC AGCCTTATTG TTACCTTTGA CTGCACTTTC ATTCTTCAAT CACATCAATT   
  
  
+ TTAAAATCTT ATTGGAAGTA CCGAGATACC TAGCAATTTA ATCTCGAAGA CTTTGTTTTT TTTATTCTTT   
  
  
+ TTTGTTTCAA TTCAGGCTTT CATTATGGT  

- ACTACTTATT TTGTCTTACT ATTAATTGTT TGTCTTTGTC AACACATATT AAATTTTTAC TTACCTCCCT   
  
  
- CCACCACCAC AAATATTCGC AACATTTTTA CGTCTCCACT CCTCTCACTA CTCACTCAAA ACAAAAGAAG   
  
  
- ATAAGAAAAA CACTTTTCTC TCACTTAACC AGCACCTTGC GCCAAACAAG CCTTAGAGTC CCTTTTCTGT   
  
  
- ACATGCGCCT TGCGTCGTAA TCTGTCTCTT TTTTCTCTCT TCGTCTGTTT CGGAACATAA GTCTCTTCCT   
  
  
- TCTTTCGATT TTCATGAGTT AATAGAGCAC CCCTCGTATG TTATTTTGGT GGTGTTAATA TAATATGTAC   
  
  
- ATGTATAAAT ACACAATATA TACATGCAAA TAAATAAATA AAATACATTT AACTATAAAC AATACTTTAA   
  
  
- TATACGCCTA CACCAATGCC AAACAAACTT ACACAATGTG CGTTCCTAAG TTTTCCACGA TCCGTGACCA   
  
  
- ACCCGCTGCC TATACACAGA TCCACCCTCA AATCCGTCTC GGATCCACCC CCAAATCCGT CAAAAAAAAA   
  
  
- AAATTAAAAT TAACTTAAAT AATATATTGT ATATTTATTG ACGGATGAAC ATGAATTTTT TTATGTAGTA   
  
  
- ACATAATTTT GCGAACTTAA CGTTTTATCT TACTATATAA CTAATATTTT ATAAGTTTGT ATAACTTTTA   
  
  
- TACTCTTTGT TGTATGTTAC TCACGAGTAG GTTTGTAAGT TCTTTAGAGC ATGTTAAATT ACTTTTTTAT   
  
  
- TTTACGTTCT ACCTTCAATA GAAAGAATTC ATTCTTAAAG CTGGATTCAC CCACCTGTCC AGATCCACCC   
  
  
- GCGGATCCAT CCGATCCACG GGAAAGAATT AAAATTTACG AATCCCTGAT TAGTACAGCC ATTGGTCGGC   
  
  
- GGATCGTGAT CCGTCTCTAA AAATCTTGTG ATCAATGTAC GTAATAAACA TTACCACTGA ATAATAAACT   
  
  
- TACAGTTCGA AAATACGTAA TAGTTAACAC AAAAATATAG GGCGTTTGAC TCTTAAATCG AACTCATCAA   
  
  
- TAAAATCAAA ATAAGCAGTA GATTGTCACA ACTCTTTAAA CCTTTTTATT GATTTAAAAT TTAGGGCATA   
  
  
- TCTTACAATT GCTAGTAAAA TTAAAATACA TTAATACATT AATATTAGTT ATGAACTACA TTTTCATAAT   
  
  
- TATATGGGAA TTATAATCCT AAAGACTGTT GTTGGTTTTG TGTTGGAAAT TTGATGGAAT TTTACTCCTT   
  
  
- TATTAGATAT AGTGAAAAAT CAAAAGTAAT AAGAGAAGTT GAAAACTTGA TGAAAGACGT TGGAAAGCCT   
  
  
- AAAAACGTTT TGCTATGTTG TCGGAATAAC AATGGAAACT GACGTGAAAG TAAGAAGTTA GTGTAGTTAA   
  
  
- AATTTTAGAA TAACCTTCAT GGCTCTATGG ATCGTTAAAT TAGAGCTTCT GAAACAAAAA AAATAAGAAA   
  
  
- AAACAAAGTT AAGTCCGAAA GTAATACCA

+     TGACG-motif

| Site Name | Organism | Position | Strand | Matrix score. | sequence | function |
| --- | --- | --- | --- | --- | --- | --- |
| TGACG-motif | Hordeum vulgare | 1065 | - | 5 | TGACG | cis-acting regulatory element involved in the MeJA-responsiveness |

>006277   
+ TGATGAATAA AACAGAATGA TAATTAACAA ACAGAAACAG TTGTGTATAA TTTAAAAATG AATGGAGGGA   
  
  
+ GGTGGTGGTG TTTATAAGCG TTGTAAAAAT GCAGAGGTGA GGAGAGTGAT GAGTGAGTTT TGTTTTCTTC   
  
  
+ TATTCTTTTT GTGAAAAGAG AGTGAATTGG TCGTGGAACG CGGTTTGTTC GGAATCTCAG GGAAAAGACA   
  
  
+ TGTACGCGGA ACGCAGCATT AGACAGAGAA AAAAGAGAGA AGCAGACAAA GCCTTGTATT CAGAGAAGGA   
  
  
+ AGAAAGCTAA AAGTACTCAA TTATCTCGTG GGGAGCATAC AATAAAACCA CCACAATTAT ATTATACATG   
  
  
+ TACATATTTA TGTGTTATAT ATGTACGTTT ATTTATTTAT TTTATGTAAA TTGATATTTG TTATGAAATT   
  
  
+ ATATGCGGAT GTGGTTACGG TTTGTTTGAA TGTGTTACAC GCAAGGATTC AAAAGGTGCT AGGCACTGGT   
  
  
+ TGGGCGACGG ATATGTGTCT AGGTGGGAGT TTAGGCAGAG CCTAGGTGGG GGTTTAGGCA GTTTTTTTTT   
  
  
+ TTTAATTTTA ATTGAATTTA TTATATAACA TATAAATAAC TGCCTACTTG TACTTAAAAA AATACATCAT   
  
  
+ TGTATTAAAA CGCTTGAATT GCAAAATAGA ATGATATATT GATTATAAAA TATTCAAACA TATTGAAAAT   
  
  
+ ATGAGAAACA ACATACAATG AGTGCTCATC CAAACATTCA AGAAATCTCG TACAATTTAA TGAAAAAATA   
  
  
+ AAATGCAAGA TGGAAGTTAT CTTTCTTAAG TAAGAATTTC GACCTAAGTG GGTGGACAGG TCTAGGTGGG   
  
  
+ CGCCTAGGTA GGCTAGGTGC CCTTTCTTAA TTTTAAATGC TTAGGGACTA ATCATGTCGG TAACCAGCCG   
  
  
+ CCTAGCACTA GGCAGAGATT TTTAGAACAC TAGTTACATG CATTATTTGT AATGGTGACT TATTATTTGA   
  
  
+ ATGTCAAGCT TTTATGCATT ATCAATTGTG TTTTTATATC CCGCAAACTG AGAATTTAGC TTGAGTAGTT   
  
  
+ ATTTTAGTTT TATTCGTCAT CTAACAGTGT TGAGAAATTT GGAAAAATAA CTAAATTTTA AATCCCGTAT   
  
  
+ AGAATGTTAA CGATCATTTT AATTTTATGT AATTATGTAA TTATAATCAA TACTTGATGT AAAAGTATTA   
  
  
+ ATATACCCTT AATATTAGGA TTTCTGACAA CAACCAAAAC ACAACCTTTA AACTACCTTA AAATGAGGAA   
  
  
+ ATAATCTATA TCACTTTTTA GTTTTCATTA TTCTCTTCAA CTTTTGAACT ACTTTCTGCA ACCTTTCGGA   
  
  
+ TTTTTGCAAA ACGATACAAC AGCCTTATTG TTACCTTTGA CTGCACTTTC ATTCTTCAAT CACATCAATT   
  
  
+ TTAAAATCTT ATTGGAAGTA CCGAGATACC TAGCAATTTA ATCTCGAAGA CTTTGTTTTT TTTATTCTTT   
  
  
+ TTTGTTTCAA TTCAGGCTTT CATTATGGT  

- ACTACTTATT TTGTCTTACT ATTAATTGTT TGTCTTTGTC AACACATATT AAATTTTTAC TTACCTCCCT   
  
  
- CCACCACCAC AAATATTCGC AACATTTTTA CGTCTCCACT CCTCTCACTA CTCACTCAAA ACAAAAGAAG   
  
  
- ATAAGAAAAA CACTTTTCTC TCACTTAACC AGCACCTTGC GCCAAACAAG CCTTAGAGTC CCTTTTCTGT   
  
  
- ACATGCGCCT TGCGTCGTAA TCTGTCTCTT TTTTCTCTCT TCGTCTGTTT CGGAACATAA GTCTCTTCCT   
  
  
- TCTTTCGATT TTCATGAGTT AATAGAGCAC CCCTCGTATG TTATTTTGGT GGTGTTAATA TAATATGTAC   
  
  
- ATGTATAAAT ACACAATATA TACATGCAAA TAAATAAATA AAATACATTT AACTATAAAC AATACTTTAA   
  
  
- TATACGCCTA CACCAATGCC AAACAAACTT ACACAATGTG CGTTCCTAAG TTTTCCACGA TCCGTGACCA   
  
  
- ACCCGCTGCC TATACACAGA TCCACCCTCA AATCCGTCTC GGATCCACCC CCAAATCCGT CAAAAAAAAA   
  
  
- AAATTAAAAT TAACTTAAAT AATATATTGT ATATTTATTG ACGGATGAAC ATGAATTTTT TTATGTAGTA   
  
  
- ACATAATTTT GCGAACTTAA CGTTTTATCT TACTATATAA CTAATATTTT ATAAGTTTGT ATAACTTTTA   
  
  
- TACTCTTTGT TGTATGTTAC TCACGAGTAG GTTTGTAAGT TCTTTAGAGC ATGTTAAATT ACTTTTTTAT   
  
  
- TTTACGTTCT ACCTTCAATA GAAAGAATTC ATTCTTAAAG CTGGATTCAC CCACCTGTCC AGATCCACCC   
  
  
- GCGGATCCAT CCGATCCACG GGAAAGAATT AAAATTTACG AATCCCTGAT TAGTACAGCC ATTGGTCGGC   
  
  
- GGATCGTGAT CCGTCTCTAA AAATCTTGTG ATCAATGTAC GTAATAAACA TTACCACTGA ATAATAAACT   
  
  
- TACAGTTCGA AAATACGTAA TAGTTAACAC AAAAATATAG GGCGTTTGAC TCTTAAATCG AACTCATCAA   
  
  
- TAAAATCAAA ATAAGCAGTA GATTGTCACA ACTCTTTAAA CCTTTTTATT GATTTAAAAT TTAGGGCATA   
  
  
- TCTTACAATT GCTAGTAAAA TTAAAATACA TTAATACATT AATATTAGTT ATGAACTACA TTTTCATAAT   
  
  
- TATATGGGAA TTATAATCCT AAAGACTGTT GTTGGTTTTG TGTTGGAAAT TTGATGGAAT TTTACTCCTT   
  
  
- TATTAGATAT AGTGAAAAAT CAAAAGTAAT AAGAGAAGTT GAAAACTTGA TGAAAGACGT TGGAAAGCCT   
  
  
- AAAAACGTTT TGCTATGTTG TCGGAATAAC AATGGAAACT GACGTGAAAG TAAGAAGTTA GTGTAGTTAA   
  
  
- AATTTTAGAA TAACCTTCAT GGCTCTATGG ATCGTTAAAT TAGAGCTTCT GAAACAAAAA AAATAAGAAA   
  
  
- AAACAAAGTT AAGTCCGAAA GTAATACCA

+     Unnamed\_\_1

| Site Name | Organism | Position | Strand | Matrix score. | sequence | function |
| --- | --- | --- | --- | --- | --- | --- |
| Unnamed\_\_1 | Zea mays | 172 | + | 5 | CGTGG |  |
| Unnamed\_\_1 | Zea mays | 307 | + | 5 | CGTGG |  |

>006277   
+ TGATGAATAA AACAGAATGA TAATTAACAA ACAGAAACAG TTGTGTATAA TTTAAAAATG AATGGAGGGA   
  
  
+ GGTGGTGGTG TTTATAAGCG TTGTAAAAAT GCAGAGGTGA GGAGAGTGAT GAGTGAGTTT TGTTTTCTTC   
  
  
+ TATTCTTTTT GTGAAAAGAG AGTGAATTGG TCGTGGAACG CGGTTTGTTC GGAATCTCAG GGAAAAGACA   
  
  
+ TGTACGCGGA ACGCAGCATT AGACAGAGAA AAAAGAGAGA AGCAGACAAA GCCTTGTATT CAGAGAAGGA   
  
  
+ AGAAAGCTAA AAGTACTCAA TTATCTCGTG GGGAGCATAC AATAAAACCA CCACAATTAT ATTATACATG   
  
  
+ TACATATTTA TGTGTTATAT ATGTACGTTT ATTTATTTAT TTTATGTAAA TTGATATTTG TTATGAAATT   
  
  
+ ATATGCGGAT GTGGTTACGG TTTGTTTGAA TGTGTTACAC GCAAGGATTC AAAAGGTGCT AGGCACTGGT   
  
  
+ TGGGCGACGG ATATGTGTCT AGGTGGGAGT TTAGGCAGAG CCTAGGTGGG GGTTTAGGCA GTTTTTTTTT   
  
  
+ TTTAATTTTA ATTGAATTTA TTATATAACA TATAAATAAC TGCCTACTTG TACTTAAAAA AATACATCAT   
  
  
+ TGTATTAAAA CGCTTGAATT GCAAAATAGA ATGATATATT GATTATAAAA TATTCAAACA TATTGAAAAT   
  
  
+ ATGAGAAACA ACATACAATG AGTGCTCATC CAAACATTCA AGAAATCTCG TACAATTTAA TGAAAAAATA   
  
  
+ AAATGCAAGA TGGAAGTTAT CTTTCTTAAG TAAGAATTTC GACCTAAGTG GGTGGACAGG TCTAGGTGGG   
  
  
+ CGCCTAGGTA GGCTAGGTGC CCTTTCTTAA TTTTAAATGC TTAGGGACTA ATCATGTCGG TAACCAGCCG   
  
  
+ CCTAGCACTA GGCAGAGATT TTTAGAACAC TAGTTACATG CATTATTTGT AATGGTGACT TATTATTTGA   
  
  
+ ATGTCAAGCT TTTATGCATT ATCAATTGTG TTTTTATATC CCGCAAACTG AGAATTTAGC TTGAGTAGTT   
  
  
+ ATTTTAGTTT TATTCGTCAT CTAACAGTGT TGAGAAATTT GGAAAAATAA CTAAATTTTA AATCCCGTAT   
  
  
+ AGAATGTTAA CGATCATTTT AATTTTATGT AATTATGTAA TTATAATCAA TACTTGATGT AAAAGTATTA   
  
  
+ ATATACCCTT AATATTAGGA TTTCTGACAA CAACCAAAAC ACAACCTTTA AACTACCTTA AAATGAGGAA   
  
  
+ ATAATCTATA TCACTTTTTA GTTTTCATTA TTCTCTTCAA CTTTTGAACT ACTTTCTGCA ACCTTTCGGA   
  
  
+ TTTTTGCAAA ACGATACAAC AGCCTTATTG TTACCTTTGA CTGCACTTTC ATTCTTCAAT CACATCAATT   
  
  
+ TTAAAATCTT ATTGGAAGTA CCGAGATACC TAGCAATTTA ATCTCGAAGA CTTTGTTTTT TTTATTCTTT   
  
  
+ TTTGTTTCAA TTCAGGCTTT CATTATGGT  

- ACTACTTATT TTGTCTTACT ATTAATTGTT TGTCTTTGTC AACACATATT AAATTTTTAC TTACCTCCCT   
  
  
- CCACCACCAC AAATATTCGC AACATTTTTA CGTCTCCACT CCTCTCACTA CTCACTCAAA ACAAAAGAAG   
  
  
- ATAAGAAAAA CACTTTTCTC TCACTTAACC AGCACCTTGC GCCAAACAAG CCTTAGAGTC CCTTTTCTGT   
  
  
- ACATGCGCCT TGCGTCGTAA TCTGTCTCTT TTTTCTCTCT TCGTCTGTTT CGGAACATAA GTCTCTTCCT   
  
  
- TCTTTCGATT TTCATGAGTT AATAGAGCAC CCCTCGTATG TTATTTTGGT GGTGTTAATA TAATATGTAC   
  
  
- ATGTATAAAT ACACAATATA TACATGCAAA TAAATAAATA AAATACATTT AACTATAAAC AATACTTTAA   
  
  
- TATACGCCTA CACCAATGCC AAACAAACTT ACACAATGTG CGTTCCTAAG TTTTCCACGA TCCGTGACCA   
  
  
- ACCCGCTGCC TATACACAGA TCCACCCTCA AATCCGTCTC GGATCCACCC CCAAATCCGT CAAAAAAAAA   
  
  
- AAATTAAAAT TAACTTAAAT AATATATTGT ATATTTATTG ACGGATGAAC ATGAATTTTT TTATGTAGTA   
  
  
- ACATAATTTT GCGAACTTAA CGTTTTATCT TACTATATAA CTAATATTTT ATAAGTTTGT ATAACTTTTA   
  
  
- TACTCTTTGT TGTATGTTAC TCACGAGTAG GTTTGTAAGT TCTTTAGAGC ATGTTAAATT ACTTTTTTAT   
  
  
- TTTACGTTCT ACCTTCAATA GAAAGAATTC ATTCTTAAAG CTGGATTCAC CCACCTGTCC AGATCCACCC   
  
  
- GCGGATCCAT CCGATCCACG GGAAAGAATT AAAATTTACG AATCCCTGAT TAGTACAGCC ATTGGTCGGC   
  
  
- GGATCGTGAT CCGTCTCTAA AAATCTTGTG ATCAATGTAC GTAATAAACA TTACCACTGA ATAATAAACT   
  
  
- TACAGTTCGA AAATACGTAA TAGTTAACAC AAAAATATAG GGCGTTTGAC TCTTAAATCG AACTCATCAA   
  
  
- TAAAATCAAA ATAAGCAGTA GATTGTCACA ACTCTTTAAA CCTTTTTATT GATTTAAAAT TTAGGGCATA   
  
  
- TCTTACAATT GCTAGTAAAA TTAAAATACA TTAATACATT AATATTAGTT ATGAACTACA TTTTCATAAT   
  
  
- TATATGGGAA TTATAATCCT AAAGACTGTT GTTGGTTTTG TGTTGGAAAT TTGATGGAAT TTTACTCCTT   
  
  
- TATTAGATAT AGTGAAAAAT CAAAAGTAAT AAGAGAAGTT GAAAACTTGA TGAAAGACGT TGGAAAGCCT   
  
  
- AAAAACGTTT TGCTATGTTG TCGGAATAAC AATGGAAACT GACGTGAAAG TAAGAAGTTA GTGTAGTTAA   
  
  
- AATTTTAGAA TAACCTTCAT GGCTCTATGG ATCGTTAAAT TAGAGCTTCT GAAACAAAAA AAATAAGAAA   
  
  
- AAACAAAGTT AAGTCCGAAA GTAATACCA

+     Unnamed\_\_4

| Site Name | Organism | Position | Strand | Matrix score. | sequence | function |
| --- | --- | --- | --- | --- | --- | --- |
| Unnamed\_\_4 | Petroselinum hortense | 516 | - | 4 | CTCC |  |
| Unnamed\_\_4 | Petroselinum hortense | 312 | - | 4 | CTCC |  |
| Unnamed\_\_4 | Petroselinum hortense | 111 | - | 4 | CTCC |  |
| Unnamed\_\_4 | Petroselinum hortense | 64 | - | 4 | CTCC |  |
| Unnamed\_\_4 | Petroselinum hortense | 68 | - | 4 | CTCC |  |

>006277   
+ TGATGAATAA AACAGAATGA TAATTAACAA ACAGAAACAG TTGTGTATAA TTTAAAAATG AATGGAGGGA   
  
  
+ GGTGGTGGTG TTTATAAGCG TTGTAAAAAT GCAGAGGTGA GGAGAGTGAT GAGTGAGTTT TGTTTTCTTC   
  
  
+ TATTCTTTTT GTGAAAAGAG AGTGAATTGG TCGTGGAACG CGGTTTGTTC GGAATCTCAG GGAAAAGACA   
  
  
+ TGTACGCGGA ACGCAGCATT AGACAGAGAA AAAAGAGAGA AGCAGACAAA GCCTTGTATT CAGAGAAGGA   
  
  
+ AGAAAGCTAA AAGTACTCAA TTATCTCGTG GGGAGCATAC AATAAAACCA CCACAATTAT ATTATACATG   
  
  
+ TACATATTTA TGTGTTATAT ATGTACGTTT ATTTATTTAT TTTATGTAAA TTGATATTTG TTATGAAATT   
  
  
+ ATATGCGGAT GTGGTTACGG TTTGTTTGAA TGTGTTACAC GCAAGGATTC AAAAGGTGCT AGGCACTGGT   
  
  
+ TGGGCGACGG ATATGTGTCT AGGTGGGAGT TTAGGCAGAG CCTAGGTGGG GGTTTAGGCA GTTTTTTTTT   
  
  
+ TTTAATTTTA ATTGAATTTA TTATATAACA TATAAATAAC TGCCTACTTG TACTTAAAAA AATACATCAT   
  
  
+ TGTATTAAAA CGCTTGAATT GCAAAATAGA ATGATATATT GATTATAAAA TATTCAAACA TATTGAAAAT   
  
  
+ ATGAGAAACA ACATACAATG AGTGCTCATC CAAACATTCA AGAAATCTCG TACAATTTAA TGAAAAAATA   
  
  
+ AAATGCAAGA TGGAAGTTAT CTTTCTTAAG TAAGAATTTC GACCTAAGTG GGTGGACAGG TCTAGGTGGG   
  
  
+ CGCCTAGGTA GGCTAGGTGC CCTTTCTTAA TTTTAAATGC TTAGGGACTA ATCATGTCGG TAACCAGCCG   
  
  
+ CCTAGCACTA GGCAGAGATT TTTAGAACAC TAGTTACATG CATTATTTGT AATGGTGACT TATTATTTGA   
  
  
+ ATGTCAAGCT TTTATGCATT ATCAATTGTG TTTTTATATC CCGCAAACTG AGAATTTAGC TTGAGTAGTT   
  
  
+ ATTTTAGTTT TATTCGTCAT CTAACAGTGT TGAGAAATTT GGAAAAATAA CTAAATTTTA AATCCCGTAT   
  
  
+ AGAATGTTAA CGATCATTTT AATTTTATGT AATTATGTAA TTATAATCAA TACTTGATGT AAAAGTATTA   
  
  
+ ATATACCCTT AATATTAGGA TTTCTGACAA CAACCAAAAC ACAACCTTTA AACTACCTTA AAATGAGGAA   
  
  
+ ATAATCTATA TCACTTTTTA GTTTTCATTA TTCTCTTCAA CTTTTGAACT ACTTTCTGCA ACCTTTCGGA   
  
  
+ TTTTTGCAAA ACGATACAAC AGCCTTATTG TTACCTTTGA CTGCACTTTC ATTCTTCAAT CACATCAATT   
  
  
+ TTAAAATCTT ATTGGAAGTA CCGAGATACC TAGCAATTTA ATCTCGAAGA CTTTGTTTTT TTTATTCTTT   
  
  
+ TTTGTTTCAA TTCAGGCTTT CATTATGGT  

- ACTACTTATT TTGTCTTACT ATTAATTGTT TGTCTTTGTC AACACATATT AAATTTTTAC TTACCTCCCT   
  
  
- CCACCACCAC AAATATTCGC AACATTTTTA CGTCTCCACT CCTCTCACTA CTCACTCAAA ACAAAAGAAG   
  
  
- ATAAGAAAAA CACTTTTCTC TCACTTAACC AGCACCTTGC GCCAAACAAG CCTTAGAGTC CCTTTTCTGT   
  
  
- ACATGCGCCT TGCGTCGTAA TCTGTCTCTT TTTTCTCTCT TCGTCTGTTT CGGAACATAA GTCTCTTCCT   
  
  
- TCTTTCGATT TTCATGAGTT AATAGAGCAC CCCTCGTATG TTATTTTGGT GGTGTTAATA TAATATGTAC   
  
  
- ATGTATAAAT ACACAATATA TACATGCAAA TAAATAAATA AAATACATTT AACTATAAAC AATACTTTAA   
  
  
- TATACGCCTA CACCAATGCC AAACAAACTT ACACAATGTG CGTTCCTAAG TTTTCCACGA TCCGTGACCA   
  
  
- ACCCGCTGCC TATACACAGA TCCACCCTCA AATCCGTCTC GGATCCACCC CCAAATCCGT CAAAAAAAAA   
  
  
- AAATTAAAAT TAACTTAAAT AATATATTGT ATATTTATTG ACGGATGAAC ATGAATTTTT TTATGTAGTA   
  
  
- ACATAATTTT GCGAACTTAA CGTTTTATCT TACTATATAA CTAATATTTT ATAAGTTTGT ATAACTTTTA   
  
  
- TACTCTTTGT TGTATGTTAC TCACGAGTAG GTTTGTAAGT TCTTTAGAGC ATGTTAAATT ACTTTTTTAT   
  
  
- TTTACGTTCT ACCTTCAATA GAAAGAATTC ATTCTTAAAG CTGGATTCAC CCACCTGTCC AGATCCACCC   
  
  
- GCGGATCCAT CCGATCCACG GGAAAGAATT AAAATTTACG AATCCCTGAT TAGTACAGCC ATTGGTCGGC   
  
  
- GGATCGTGAT CCGTCTCTAA AAATCTTGTG ATCAATGTAC GTAATAAACA TTACCACTGA ATAATAAACT   
  
  
- TACAGTTCGA AAATACGTAA TAGTTAACAC AAAAATATAG GGCGTTTGAC TCTTAAATCG AACTCATCAA   
  
  
- TAAAATCAAA ATAAGCAGTA GATTGTCACA ACTCTTTAAA CCTTTTTATT GATTTAAAAT TTAGGGCATA   
  
  
- TCTTACAATT GCTAGTAAAA TTAAAATACA TTAATACATT AATATTAGTT ATGAACTACA TTTTCATAAT   
  
  
- TATATGGGAA TTATAATCCT AAAGACTGTT GTTGGTTTTG TGTTGGAAAT TTGATGGAAT TTTACTCCTT   
  
  
- TATTAGATAT AGTGAAAAAT CAAAAGTAAT AAGAGAAGTT GAAAACTTGA TGAAAGACGT TGGAAAGCCT   
  
  
- AAAAACGTTT TGCTATGTTG TCGGAATAAC AATGGAAACT GACGTGAAAG TAAGAAGTTA GTGTAGTTAA   
  
  
- AATTTTAGAA TAACCTTCAT GGCTCTATGG ATCGTTAAAT TAGAGCTTCT GAAACAAAAA AAATAAGAAA   
  
  
- AAACAAAGTT AAGTCCGAAA GTAATACCA

+     Unnamed\_\_6

| Site Name | Organism | Position | Strand | Matrix score. | sequence | function |
| --- | --- | --- | --- | --- | --- | --- |
| Unnamed\_\_6 | Zea mays | 591 | + | 10 | taTAAATATct |  |

>006277   
+ TGATGAATAA AACAGAATGA TAATTAACAA ACAGAAACAG TTGTGTATAA TTTAAAAATG AATGGAGGGA   
  
  
+ GGTGGTGGTG TTTATAAGCG TTGTAAAAAT GCAGAGGTGA GGAGAGTGAT GAGTGAGTTT TGTTTTCTTC   
  
  
+ TATTCTTTTT GTGAAAAGAG AGTGAATTGG TCGTGGAACG CGGTTTGTTC GGAATCTCAG GGAAAAGACA   
  
  
+ TGTACGCGGA ACGCAGCATT AGACAGAGAA AAAAGAGAGA AGCAGACAAA GCCTTGTATT CAGAGAAGGA   
  
  
+ AGAAAGCTAA AAGTACTCAA TTATCTCGTG GGGAGCATAC AATAAAACCA CCACAATTAT ATTATACATG   
  
  
+ TACATATTTA TGTGTTATAT ATGTACGTTT ATTTATTTAT TTTATGTAAA TTGATATTTG TTATGAAATT   
  
  
+ ATATGCGGAT GTGGTTACGG TTTGTTTGAA TGTGTTACAC GCAAGGATTC AAAAGGTGCT AGGCACTGGT   
  
  
+ TGGGCGACGG ATATGTGTCT AGGTGGGAGT TTAGGCAGAG CCTAGGTGGG GGTTTAGGCA GTTTTTTTTT   
  
  
+ TTTAATTTTA ATTGAATTTA TTATATAACA TATAAATAAC TGCCTACTTG TACTTAAAAA AATACATCAT   
  
  
+ TGTATTAAAA CGCTTGAATT GCAAAATAGA ATGATATATT GATTATAAAA TATTCAAACA TATTGAAAAT   
  
  
+ ATGAGAAACA ACATACAATG AGTGCTCATC CAAACATTCA AGAAATCTCG TACAATTTAA TGAAAAAATA   
  
  
+ AAATGCAAGA TGGAAGTTAT CTTTCTTAAG TAAGAATTTC GACCTAAGTG GGTGGACAGG TCTAGGTGGG   
  
  
+ CGCCTAGGTA GGCTAGGTGC CCTTTCTTAA TTTTAAATGC TTAGGGACTA ATCATGTCGG TAACCAGCCG   
  
  
+ CCTAGCACTA GGCAGAGATT TTTAGAACAC TAGTTACATG CATTATTTGT AATGGTGACT TATTATTTGA   
  
  
+ ATGTCAAGCT TTTATGCATT ATCAATTGTG TTTTTATATC CCGCAAACTG AGAATTTAGC TTGAGTAGTT   
  
  
+ ATTTTAGTTT TATTCGTCAT CTAACAGTGT TGAGAAATTT GGAAAAATAA CTAAATTTTA AATCCCGTAT   
  
  
+ AGAATGTTAA CGATCATTTT AATTTTATGT AATTATGTAA TTATAATCAA TACTTGATGT AAAAGTATTA   
  
  
+ ATATACCCTT AATATTAGGA TTTCTGACAA CAACCAAAAC ACAACCTTTA AACTACCTTA AAATGAGGAA   
  
  
+ ATAATCTATA TCACTTTTTA GTTTTCATTA TTCTCTTCAA CTTTTGAACT ACTTTCTGCA ACCTTTCGGA   
  
  
+ TTTTTGCAAA ACGATACAAC AGCCTTATTG TTACCTTTGA CTGCACTTTC ATTCTTCAAT CACATCAATT   
  
  
+ TTAAAATCTT ATTGGAAGTA CCGAGATACC TAGCAATTTA ATCTCGAAGA CTTTGTTTTT TTTATTCTTT   
  
  
+ TTTGTTTCAA TTCAGGCTTT CATTATGGT  

- ACTACTTATT TTGTCTTACT ATTAATTGTT TGTCTTTGTC AACACATATT AAATTTTTAC TTACCTCCCT   
  
  
- CCACCACCAC AAATATTCGC AACATTTTTA CGTCTCCACT CCTCTCACTA CTCACTCAAA ACAAAAGAAG   
  
  
- ATAAGAAAAA CACTTTTCTC TCACTTAACC AGCACCTTGC GCCAAACAAG CCTTAGAGTC CCTTTTCTGT   
  
  
- ACATGCGCCT TGCGTCGTAA TCTGTCTCTT TTTTCTCTCT TCGTCTGTTT CGGAACATAA GTCTCTTCCT   
  
  
- TCTTTCGATT TTCATGAGTT AATAGAGCAC CCCTCGTATG TTATTTTGGT GGTGTTAATA TAATATGTAC   
  
  
- ATGTATAAAT ACACAATATA TACATGCAAA TAAATAAATA AAATACATTT AACTATAAAC AATACTTTAA   
  
  
- TATACGCCTA CACCAATGCC AAACAAACTT ACACAATGTG CGTTCCTAAG TTTTCCACGA TCCGTGACCA   
  
  
- ACCCGCTGCC TATACACAGA TCCACCCTCA AATCCGTCTC GGATCCACCC CCAAATCCGT CAAAAAAAAA   
  
  
- AAATTAAAAT TAACTTAAAT AATATATTGT ATATTTATTG ACGGATGAAC ATGAATTTTT TTATGTAGTA   
  
  
- ACATAATTTT GCGAACTTAA CGTTTTATCT TACTATATAA CTAATATTTT ATAAGTTTGT ATAACTTTTA   
  
  
- TACTCTTTGT TGTATGTTAC TCACGAGTAG GTTTGTAAGT TCTTTAGAGC ATGTTAAATT ACTTTTTTAT   
  
  
- TTTACGTTCT ACCTTCAATA GAAAGAATTC ATTCTTAAAG CTGGATTCAC CCACCTGTCC AGATCCACCC   
  
  
- GCGGATCCAT CCGATCCACG GGAAAGAATT AAAATTTACG AATCCCTGAT TAGTACAGCC ATTGGTCGGC   
  
  
- GGATCGTGAT CCGTCTCTAA AAATCTTGTG ATCAATGTAC GTAATAAACA TTACCACTGA ATAATAAACT   
  
  
- TACAGTTCGA AAATACGTAA TAGTTAACAC AAAAATATAG GGCGTTTGAC TCTTAAATCG AACTCATCAA   
  
  
- TAAAATCAAA ATAAGCAGTA GATTGTCACA ACTCTTTAAA CCTTTTTATT GATTTAAAAT TTAGGGCATA   
  
  
- TCTTACAATT GCTAGTAAAA TTAAAATACA TTAATACATT AATATTAGTT ATGAACTACA TTTTCATAAT   
  
  
- TATATGGGAA TTATAATCCT AAAGACTGTT GTTGGTTTTG TGTTGGAAAT TTGATGGAAT TTTACTCCTT   
  
  
- TATTAGATAT AGTGAAAAAT CAAAAGTAAT AAGAGAAGTT GAAAACTTGA TGAAAGACGT TGGAAAGCCT   
  
  
- AAAAACGTTT TGCTATGTTG TCGGAATAAC AATGGAAACT GACGTGAAAG TAAGAAGTTA GTGTAGTTAA   
  
  
- AATTTTAGAA TAACCTTCAT GGCTCTATGG ATCGTTAAAT TAGAGCTTCT GAAACAAAAA AAATAAGAAA   
  
  
- AAACAAAGTT AAGTCCGAAA GTAATACCA

+     WRE3

| Site Name | Organism | Position | Strand | Matrix score. | sequence | function |
| --- | --- | --- | --- | --- | --- | --- |
| WRE3 | Pisum sativum | 834 | - | 6 | CCACCT |  |
| WRE3 | Pisum sativum | 534 | - | 6 | CCACCT |  |
| WRE3 | Pisum sativum | 70 | - | 6 | CCACCT |  |
| WRE3 | Pisum sativum | 511 | - | 6 | CCACCT |  |

>006277   
+ TGATGAATAA AACAGAATGA TAATTAACAA ACAGAAACAG TTGTGTATAA TTTAAAAATG AATGGAGGGA   
  
  
+ GGTGGTGGTG TTTATAAGCG TTGTAAAAAT GCAGAGGTGA GGAGAGTGAT GAGTGAGTTT TGTTTTCTTC   
  
  
+ TATTCTTTTT GTGAAAAGAG AGTGAATTGG TCGTGGAACG CGGTTTGTTC GGAATCTCAG GGAAAAGACA   
  
  
+ TGTACGCGGA ACGCAGCATT AGACAGAGAA AAAAGAGAGA AGCAGACAAA GCCTTGTATT CAGAGAAGGA   
  
  
+ AGAAAGCTAA AAGTACTCAA TTATCTCGTG GGGAGCATAC AATAAAACCA CCACAATTAT ATTATACATG   
  
  
+ TACATATTTA TGTGTTATAT ATGTACGTTT ATTTATTTAT TTTATGTAAA TTGATATTTG TTATGAAATT   
  
  
+ ATATGCGGAT GTGGTTACGG TTTGTTTGAA TGTGTTACAC GCAAGGATTC AAAAGGTGCT AGGCACTGGT   
  
  
+ TGGGCGACGG ATATGTGTCT AGGTGGGAGT TTAGGCAGAG CCTAGGTGGG GGTTTAGGCA GTTTTTTTTT   
  
  
+ TTTAATTTTA ATTGAATTTA TTATATAACA TATAAATAAC TGCCTACTTG TACTTAAAAA AATACATCAT   
  
  
+ TGTATTAAAA CGCTTGAATT GCAAAATAGA ATGATATATT GATTATAAAA TATTCAAACA TATTGAAAAT   
  
  
+ ATGAGAAACA ACATACAATG AGTGCTCATC CAAACATTCA AGAAATCTCG TACAATTTAA TGAAAAAATA   
  
  
+ AAATGCAAGA TGGAAGTTAT CTTTCTTAAG TAAGAATTTC GACCTAAGTG GGTGGACAGG TCTAGGTGGG   
  
  
+ CGCCTAGGTA GGCTAGGTGC CCTTTCTTAA TTTTAAATGC TTAGGGACTA ATCATGTCGG TAACCAGCCG   
  
  
+ CCTAGCACTA GGCAGAGATT TTTAGAACAC TAGTTACATG CATTATTTGT AATGGTGACT TATTATTTGA   
  
  
+ ATGTCAAGCT TTTATGCATT ATCAATTGTG TTTTTATATC CCGCAAACTG AGAATTTAGC TTGAGTAGTT   
  
  
+ ATTTTAGTTT TATTCGTCAT CTAACAGTGT TGAGAAATTT GGAAAAATAA CTAAATTTTA AATCCCGTAT   
  
  
+ AGAATGTTAA CGATCATTTT AATTTTATGT AATTATGTAA TTATAATCAA TACTTGATGT AAAAGTATTA   
  
  
+ ATATACCCTT AATATTAGGA TTTCTGACAA CAACCAAAAC ACAACCTTTA AACTACCTTA AAATGAGGAA   
  
  
+ ATAATCTATA TCACTTTTTA GTTTTCATTA TTCTCTTCAA CTTTTGAACT ACTTTCTGCA ACCTTTCGGA   
  
  
+ TTTTTGCAAA ACGATACAAC AGCCTTATTG TTACCTTTGA CTGCACTTTC ATTCTTCAAT CACATCAATT   
  
  
+ TTAAAATCTT ATTGGAAGTA CCGAGATACC TAGCAATTTA ATCTCGAAGA CTTTGTTTTT TTTATTCTTT   
  
  
+ TTTGTTTCAA TTCAGGCTTT CATTATGGT  

- ACTACTTATT TTGTCTTACT ATTAATTGTT TGTCTTTGTC AACACATATT AAATTTTTAC TTACCTCCCT   
  
  
- CCACCACCAC AAATATTCGC AACATTTTTA CGTCTCCACT CCTCTCACTA CTCACTCAAA ACAAAAGAAG   
  
  
- ATAAGAAAAA CACTTTTCTC TCACTTAACC AGCACCTTGC GCCAAACAAG CCTTAGAGTC CCTTTTCTGT   
  
  
- ACATGCGCCT TGCGTCGTAA TCTGTCTCTT TTTTCTCTCT TCGTCTGTTT CGGAACATAA GTCTCTTCCT   
  
  
- TCTTTCGATT TTCATGAGTT AATAGAGCAC CCCTCGTATG TTATTTTGGT GGTGTTAATA TAATATGTAC   
  
  
- ATGTATAAAT ACACAATATA TACATGCAAA TAAATAAATA AAATACATTT AACTATAAAC AATACTTTAA   
  
  
- TATACGCCTA CACCAATGCC AAACAAACTT ACACAATGTG CGTTCCTAAG TTTTCCACGA TCCGTGACCA   
  
  
- ACCCGCTGCC TATACACAGA TCCACCCTCA AATCCGTCTC GGATCCACCC CCAAATCCGT CAAAAAAAAA   
  
  
- AAATTAAAAT TAACTTAAAT AATATATTGT ATATTTATTG ACGGATGAAC ATGAATTTTT TTATGTAGTA   
  
  
- ACATAATTTT GCGAACTTAA CGTTTTATCT TACTATATAA CTAATATTTT ATAAGTTTGT ATAACTTTTA   
  
  
- TACTCTTTGT TGTATGTTAC TCACGAGTAG GTTTGTAAGT TCTTTAGAGC ATGTTAAATT ACTTTTTTAT   
  
  
- TTTACGTTCT ACCTTCAATA GAAAGAATTC ATTCTTAAAG CTGGATTCAC CCACCTGTCC AGATCCACCC   
  
  
- GCGGATCCAT CCGATCCACG GGAAAGAATT AAAATTTACG AATCCCTGAT TAGTACAGCC ATTGGTCGGC   
  
  
- GGATCGTGAT CCGTCTCTAA AAATCTTGTG ATCAATGTAC GTAATAAACA TTACCACTGA ATAATAAACT   
  
  
- TACAGTTCGA AAATACGTAA TAGTTAACAC AAAAATATAG GGCGTTTGAC TCTTAAATCG AACTCATCAA   
  
  
- TAAAATCAAA ATAAGCAGTA GATTGTCACA ACTCTTTAAA CCTTTTTATT GATTTAAAAT TTAGGGCATA   
  
  
- TCTTACAATT GCTAGTAAAA TTAAAATACA TTAATACATT AATATTAGTT ATGAACTACA TTTTCATAAT   
  
  
- TATATGGGAA TTATAATCCT AAAGACTGTT GTTGGTTTTG TGTTGGAAAT TTGATGGAAT TTTACTCCTT   
  
  
- TATTAGATAT AGTGAAAAAT CAAAAGTAAT AAGAGAAGTT GAAAACTTGA TGAAAGACGT TGGAAAGCCT   
  
  
- AAAAACGTTT TGCTATGTTG TCGGAATAAC AATGGAAACT GACGTGAAAG TAAGAAGTTA GTGTAGTTAA   
  
  
- AATTTTAGAA TAACCTTCAT GGCTCTATGG ATCGTTAAAT TAGAGCTTCT GAAACAAAAA AAATAAGAAA   
  
  
- AAACAAAGTT AAGTCCGAAA GTAATACCA

+     as-1

| Site Name | Organism | Position | Strand | Matrix score. | sequence | function |
| --- | --- | --- | --- | --- | --- | --- |
| as-1 | Arabidopsis thaliana | 1065 | - | 5 | TGACG |  |

>006277   
+ TGATGAATAA AACAGAATGA TAATTAACAA ACAGAAACAG TTGTGTATAA TTTAAAAATG AATGGAGGGA   
  
  
+ GGTGGTGGTG TTTATAAGCG TTGTAAAAAT GCAGAGGTGA GGAGAGTGAT GAGTGAGTTT TGTTTTCTTC   
  
  
+ TATTCTTTTT GTGAAAAGAG AGTGAATTGG TCGTGGAACG CGGTTTGTTC GGAATCTCAG GGAAAAGACA   
  
  
+ TGTACGCGGA ACGCAGCATT AGACAGAGAA AAAAGAGAGA AGCAGACAAA GCCTTGTATT CAGAGAAGGA   
  
  
+ AGAAAGCTAA AAGTACTCAA TTATCTCGTG GGGAGCATAC AATAAAACCA CCACAATTAT ATTATACATG   
  
  
+ TACATATTTA TGTGTTATAT ATGTACGTTT ATTTATTTAT TTTATGTAAA TTGATATTTG TTATGAAATT   
  
  
+ ATATGCGGAT GTGGTTACGG TTTGTTTGAA TGTGTTACAC GCAAGGATTC AAAAGGTGCT AGGCACTGGT   
  
  
+ TGGGCGACGG ATATGTGTCT AGGTGGGAGT TTAGGCAGAG CCTAGGTGGG GGTTTAGGCA GTTTTTTTTT   
  
  
+ TTTAATTTTA ATTGAATTTA TTATATAACA TATAAATAAC TGCCTACTTG TACTTAAAAA AATACATCAT   
  
  
+ TGTATTAAAA CGCTTGAATT GCAAAATAGA ATGATATATT GATTATAAAA TATTCAAACA TATTGAAAAT   
  
  
+ ATGAGAAACA ACATACAATG AGTGCTCATC CAAACATTCA AGAAATCTCG TACAATTTAA TGAAAAAATA   
  
  
+ AAATGCAAGA TGGAAGTTAT CTTTCTTAAG TAAGAATTTC GACCTAAGTG GGTGGACAGG TCTAGGTGGG   
  
  
+ CGCCTAGGTA GGCTAGGTGC CCTTTCTTAA TTTTAAATGC TTAGGGACTA ATCATGTCGG TAACCAGCCG   
  
  
+ CCTAGCACTA GGCAGAGATT TTTAGAACAC TAGTTACATG CATTATTTGT AATGGTGACT TATTATTTGA   
  
  
+ ATGTCAAGCT TTTATGCATT ATCAATTGTG TTTTTATATC CCGCAAACTG AGAATTTAGC TTGAGTAGTT   
  
  
+ ATTTTAGTTT TATTCGTCAT CTAACAGTGT TGAGAAATTT GGAAAAATAA CTAAATTTTA AATCCCGTAT   
  
  
+ AGAATGTTAA CGATCATTTT AATTTTATGT AATTATGTAA TTATAATCAA TACTTGATGT AAAAGTATTA   
  
  
+ ATATACCCTT AATATTAGGA TTTCTGACAA CAACCAAAAC ACAACCTTTA AACTACCTTA AAATGAGGAA   
  
  
+ ATAATCTATA TCACTTTTTA GTTTTCATTA TTCTCTTCAA CTTTTGAACT ACTTTCTGCA ACCTTTCGGA   
  
  
+ TTTTTGCAAA ACGATACAAC AGCCTTATTG TTACCTTTGA CTGCACTTTC ATTCTTCAAT CACATCAATT   
  
  
+ TTAAAATCTT ATTGGAAGTA CCGAGATACC TAGCAATTTA ATCTCGAAGA CTTTGTTTTT TTTATTCTTT   
  
  
+ TTTGTTTCAA TTCAGGCTTT CATTATGGT  

- ACTACTTATT TTGTCTTACT ATTAATTGTT TGTCTTTGTC AACACATATT AAATTTTTAC TTACCTCCCT   
  
  
- CCACCACCAC AAATATTCGC AACATTTTTA CGTCTCCACT CCTCTCACTA CTCACTCAAA ACAAAAGAAG   
  
  
- ATAAGAAAAA CACTTTTCTC TCACTTAACC AGCACCTTGC GCCAAACAAG CCTTAGAGTC CCTTTTCTGT   
  
  
- ACATGCGCCT TGCGTCGTAA TCTGTCTCTT TTTTCTCTCT TCGTCTGTTT CGGAACATAA GTCTCTTCCT   
  
  
- TCTTTCGATT TTCATGAGTT AATAGAGCAC CCCTCGTATG TTATTTTGGT GGTGTTAATA TAATATGTAC   
  
  
- ATGTATAAAT ACACAATATA TACATGCAAA TAAATAAATA AAATACATTT AACTATAAAC AATACTTTAA   
  
  
- TATACGCCTA CACCAATGCC AAACAAACTT ACACAATGTG CGTTCCTAAG TTTTCCACGA TCCGTGACCA   
  
  
- ACCCGCTGCC TATACACAGA TCCACCCTCA AATCCGTCTC GGATCCACCC CCAAATCCGT CAAAAAAAAA   
  
  
- AAATTAAAAT TAACTTAAAT AATATATTGT ATATTTATTG ACGGATGAAC ATGAATTTTT TTATGTAGTA   
  
  
- ACATAATTTT GCGAACTTAA CGTTTTATCT TACTATATAA CTAATATTTT ATAAGTTTGT ATAACTTTTA   
  
  
- TACTCTTTGT TGTATGTTAC TCACGAGTAG GTTTGTAAGT TCTTTAGAGC ATGTTAAATT ACTTTTTTAT   
  
  
- TTTACGTTCT ACCTTCAATA GAAAGAATTC ATTCTTAAAG CTGGATTCAC CCACCTGTCC AGATCCACCC   
  
  
- GCGGATCCAT CCGATCCACG GGAAAGAATT AAAATTTACG AATCCCTGAT TAGTACAGCC ATTGGTCGGC   
  
  
- GGATCGTGAT CCGTCTCTAA AAATCTTGTG ATCAATGTAC GTAATAAACA TTACCACTGA ATAATAAACT   
  
  
- TACAGTTCGA AAATACGTAA TAGTTAACAC AAAAATATAG GGCGTTTGAC TCTTAAATCG AACTCATCAA   
  
  
- TAAAATCAAA ATAAGCAGTA GATTGTCACA ACTCTTTAAA CCTTTTTATT GATTTAAAAT TTAGGGCATA   
  
  
- TCTTACAATT GCTAGTAAAA TTAAAATACA TTAATACATT AATATTAGTT ATGAACTACA TTTTCATAAT   
  
  
- TATATGGGAA TTATAATCCT AAAGACTGTT GTTGGTTTTG TGTTGGAAAT TTGATGGAAT TTTACTCCTT   
  
  
- TATTAGATAT AGTGAAAAAT CAAAAGTAAT AAGAGAAGTT GAAAACTTGA TGAAAGACGT TGGAAAGCCT   
  
  
- AAAAACGTTT TGCTATGTTG TCGGAATAAC AATGGAAACT GACGTGAAAG TAAGAAGTTA GTGTAGTTAA   
  
  
- AATTTTAGAA TAACCTTCAT GGCTCTATGG ATCGTTAAAT TAGAGCTTCT GAAACAAAAA AAATAAGAAA   
  
  
- AAACAAAGTT AAGTCCGAAA GTAATACCA

+     chs-CMA1a

| Site Name | Organism | Position | Strand | Matrix score. | sequence | function |
| --- | --- | --- | --- | --- | --- | --- |
| chs-CMA1a | Daucus carota | 796 | - | 8 | TTACTTAA | part of a light responsive element |

>006277   
+ TGATGAATAA AACAGAATGA TAATTAACAA ACAGAAACAG TTGTGTATAA TTTAAAAATG AATGGAGGGA   
  
  
+ GGTGGTGGTG TTTATAAGCG TTGTAAAAAT GCAGAGGTGA GGAGAGTGAT GAGTGAGTTT TGTTTTCTTC   
  
  
+ TATTCTTTTT GTGAAAAGAG AGTGAATTGG TCGTGGAACG CGGTTTGTTC GGAATCTCAG GGAAAAGACA   
  
  
+ TGTACGCGGA ACGCAGCATT AGACAGAGAA AAAAGAGAGA AGCAGACAAA GCCTTGTATT CAGAGAAGGA   
  
  
+ AGAAAGCTAA AAGTACTCAA TTATCTCGTG GGGAGCATAC AATAAAACCA CCACAATTAT ATTATACATG   
  
  
+ TACATATTTA TGTGTTATAT ATGTACGTTT ATTTATTTAT TTTATGTAAA TTGATATTTG TTATGAAATT   
  
  
+ ATATGCGGAT GTGGTTACGG TTTGTTTGAA TGTGTTACAC GCAAGGATTC AAAAGGTGCT AGGCACTGGT   
  
  
+ TGGGCGACGG ATATGTGTCT AGGTGGGAGT TTAGGCAGAG CCTAGGTGGG GGTTTAGGCA GTTTTTTTTT   
  
  
+ TTTAATTTTA ATTGAATTTA TTATATAACA TATAAATAAC TGCCTACTTG TACTTAAAAA AATACATCAT   
  
  
+ TGTATTAAAA CGCTTGAATT GCAAAATAGA ATGATATATT GATTATAAAA TATTCAAACA TATTGAAAAT   
  
  
+ ATGAGAAACA ACATACAATG AGTGCTCATC CAAACATTCA AGAAATCTCG TACAATTTAA TGAAAAAATA   
  
  
+ AAATGCAAGA TGGAAGTTAT CTTTCTTAAG TAAGAATTTC GACCTAAGTG GGTGGACAGG TCTAGGTGGG   
  
  
+ CGCCTAGGTA GGCTAGGTGC CCTTTCTTAA TTTTAAATGC TTAGGGACTA ATCATGTCGG TAACCAGCCG   
  
  
+ CCTAGCACTA GGCAGAGATT TTTAGAACAC TAGTTACATG CATTATTTGT AATGGTGACT TATTATTTGA   
  
  
+ ATGTCAAGCT TTTATGCATT ATCAATTGTG TTTTTATATC CCGCAAACTG AGAATTTAGC TTGAGTAGTT   
  
  
+ ATTTTAGTTT TATTCGTCAT CTAACAGTGT TGAGAAATTT GGAAAAATAA CTAAATTTTA AATCCCGTAT   
  
  
+ AGAATGTTAA CGATCATTTT AATTTTATGT AATTATGTAA TTATAATCAA TACTTGATGT AAAAGTATTA   
  
  
+ ATATACCCTT AATATTAGGA TTTCTGACAA CAACCAAAAC ACAACCTTTA AACTACCTTA AAATGAGGAA   
  
  
+ ATAATCTATA TCACTTTTTA GTTTTCATTA TTCTCTTCAA CTTTTGAACT ACTTTCTGCA ACCTTTCGGA   
  
  
+ TTTTTGCAAA ACGATACAAC AGCCTTATTG TTACCTTTGA CTGCACTTTC ATTCTTCAAT CACATCAATT   
  
  
+ TTAAAATCTT ATTGGAAGTA CCGAGATACC TAGCAATTTA ATCTCGAAGA CTTTGTTTTT TTTATTCTTT   
  
  
+ TTTGTTTCAA TTCAGGCTTT CATTATGGT  

- ACTACTTATT TTGTCTTACT ATTAATTGTT TGTCTTTGTC AACACATATT AAATTTTTAC TTACCTCCCT   
  
  
- CCACCACCAC AAATATTCGC AACATTTTTA CGTCTCCACT CCTCTCACTA CTCACTCAAA ACAAAAGAAG   
  
  
- ATAAGAAAAA CACTTTTCTC TCACTTAACC AGCACCTTGC GCCAAACAAG CCTTAGAGTC CCTTTTCTGT   
  
  
- ACATGCGCCT TGCGTCGTAA TCTGTCTCTT TTTTCTCTCT TCGTCTGTTT CGGAACATAA GTCTCTTCCT   
  
  
- TCTTTCGATT TTCATGAGTT AATAGAGCAC CCCTCGTATG TTATTTTGGT GGTGTTAATA TAATATGTAC   
  
  
- ATGTATAAAT ACACAATATA TACATGCAAA TAAATAAATA AAATACATTT AACTATAAAC AATACTTTAA   
  
  
- TATACGCCTA CACCAATGCC AAACAAACTT ACACAATGTG CGTTCCTAAG TTTTCCACGA TCCGTGACCA   
  
  
- ACCCGCTGCC TATACACAGA TCCACCCTCA AATCCGTCTC GGATCCACCC CCAAATCCGT CAAAAAAAAA   
  
  
- AAATTAAAAT TAACTTAAAT AATATATTGT ATATTTATTG ACGGATGAAC ATGAATTTTT TTATGTAGTA   
  
  
- ACATAATTTT GCGAACTTAA CGTTTTATCT TACTATATAA CTAATATTTT ATAAGTTTGT ATAACTTTTA   
  
  
- TACTCTTTGT TGTATGTTAC TCACGAGTAG GTTTGTAAGT TCTTTAGAGC ATGTTAAATT ACTTTTTTAT   
  
  
- TTTACGTTCT ACCTTCAATA GAAAGAATTC ATTCTTAAAG CTGGATTCAC CCACCTGTCC AGATCCACCC   
  
  
- GCGGATCCAT CCGATCCACG GGAAAGAATT AAAATTTACG AATCCCTGAT TAGTACAGCC ATTGGTCGGC   
  
  
- GGATCGTGAT CCGTCTCTAA AAATCTTGTG ATCAATGTAC GTAATAAACA TTACCACTGA ATAATAAACT   
  
  
- TACAGTTCGA AAATACGTAA TAGTTAACAC AAAAATATAG GGCGTTTGAC TCTTAAATCG AACTCATCAA   
  
  
- TAAAATCAAA ATAAGCAGTA GATTGTCACA ACTCTTTAAA CCTTTTTATT GATTTAAAAT TTAGGGCATA   
  
  
- TCTTACAATT GCTAGTAAAA TTAAAATACA TTAATACATT AATATTAGTT ATGAACTACA TTTTCATAAT   
  
  
- TATATGGGAA TTATAATCCT AAAGACTGTT GTTGGTTTTG TGTTGGAAAT TTGATGGAAT TTTACTCCTT   
  
  
- TATTAGATAT AGTGAAAAAT CAAAAGTAAT AAGAGAAGTT GAAAACTTGA TGAAAGACGT TGGAAAGCCT   
  
  
- AAAAACGTTT TGCTATGTTG TCGGAATAAC AATGGAAACT GACGTGAAAG TAAGAAGTTA GTGTAGTTAA   
  
  
- AATTTTAGAA TAACCTTCAT GGCTCTATGG ATCGTTAAAT TAGAGCTTCT GAAACAAAAA AAATAAGAAA   
  
  
- AAACAAAGTT AAGTCCGAAA GTAATACCA
