# Supplementary material for: In Silico Genome-Wide Analysis of Respiratory Burst Oxidase Homolog (RBOH) Family Genes in Five Fruit-Producing Trees, and Potential Functional Analysis on Lignification of Stone Cells in Chinese White Pear
Source: Cells. 2019 May 29;8(6):520. doi: 10.3390/cells8060520 (PMC6627160; doi:10.3390/cells8060520)
Supplement: Supplementary file 1 [file cells-08-00520-s001.zip › Supplementary Material/Spreadsheets S2. Numbers of cis-elements in promoter region of PbRBOHs.tar.zip!/PbRBOHI.html]

Content-Type: text/html; charset=ISO-8859-1


PlantCARE


Webmaster Firefox specific output  
To save the result:
click on the frame with the right mouse button and save the source code as a text file with extension .html  
REFERENCE:PlantCARE: a database of plant cis-acting regulatory elements and a portal to tools for in silico analysis of promoter sequences.  
Lescot, M., Déhais, P., Moreau, Y., De Moor, B., Rouzé ,P.,and Rombauts, S.  
Nucleic Acids Res., Database issue(2002), 30(1):325-327.   


---

>007212   
+ TGGTGAGTAT TTCTTTTTTC CGGTGTAGAA GCAGGTCGAA GCACGAGAAA ACCAAAAGAA ACTAGCAAAT   
  
  
+ CACCACCCTA TTACGATCCC TTCTAGCGAT ATCATTCTCG AGGATACAAG AAATATAAGA AGGAGCATTA   
  
  
+ GCGAACTGCG AACATATGGA CACCGAAAGA GAGGTTATAG CCCGAAATAG CTAAAGGTGA TCAAAGTTTT   
  
  
+ GATGAGAAGA AAGAGATGTG GACTGCTGTG TGAGGTGGGA GAGTTTTAGT TTTTTTTTTT CTGGAAGGAA   
  
  
+ ATGAAGATCG ATGGGTGGAG TGCTTATAAC TTGTTTGAAA GGTGTAAGTT TTTTGGGATG GAAGGAGAAG   
  
  
+ AGAGTGGCCT CATTTATTTT GTTTTGTTTG GTTTGTACAA GCTAATCAGC TAAGAATTCT TGTTTAACTG   
  
  
+ GTTTTGTGTT CTAGTTTTTG GTTTCTTGGA ACTTTGGTGG TTGTTGAGTG AGAAAGTTGA GCAAGTGAGA   
  
  
+ GAGAGAGAGA GGTTTTGTTA GGGGTAGAAA GGTTAGGAGC GAAAAGGAAG TGAGAGAGAG AAAGAGCAGG   
  
  
+ TAAGACTAGT GAAAAGGTTG TGATATGAGA GAGAGAGAGA GAGAGGGAAT AAGGTTAAAT TTCACATCAA   
  
  
+ CCTTATTCGA ATAAGAGAGA CTATTTAGTA GGATAACTTA ATTCGGTCAC AAAATATGCA ATGGTGTGTG   
  
  
+ TATTGAGAGT TGTTTTTACG TGAGGTCGGA TAATAATCAA ACTTAATGGA AATCAATTTT TTATGAAAGT   
  
  
+ GTACGATCGT CTTCTCTATC CACTGTGCGA GTTTTTCTCA CCAAATACAT ATGAAAATGG CATGATTCAC   
  
  
+ CATGCAAGAA AGGGATTTGG AGAATAATTG GTTATAACAG GTCATGTTTG CCTAGCAGCT TCCTTCTACT   
  
  
+ CCATTAATTT GGACTTGTGC ATTGCATGCC TTATATGGGT TGTATTTTGT GGATAAAGGA AAAGGACAAA   
  
  
+ GGGTGGGCTG GTTACGTAGG CTTTTTAGTT TTAATGAAAC ATTTCCGTTA CTGTTCATTT TTAACAAAAA   
  
  
+ TAACATTTTT ACTCTAAAAA AATCACTCCT CGTACTATTC ATTTACAACA CATTTTTGTC ATTTTTATTA   
  
  
+ AAACTCAAAG TTTTCAAGTT TTTTTCATTA ATTTTCCTTT TTATTTATTT ATTTTCAGAA TTAGGCAAAC   
  
  
+ AATTAGCCAC ATTACTTGAT CAATCAACTA TATGACATTG ATAGAATTCT ACTTTGCACT TTGCAACATC   
  
  
+ AAGTTTCCAC TTCAACCAAC CAGATGGGAT TTGCTAATTT TAGTCATTAA CTAAAGATTA TTCGATGTTG   
  
  
+ AGTATAAGGT TTGTCGCTTA AATGATTAAG AGCATTTATT AGTGCACCTA ATTGTTGCAC TACTTGAAGT   
  
  
+ CGTGTATTAG ACCAAGATCA TGCTCATATA ACTCATGGTA GATTAGGATG TGACTTAATT GTAGTTTGTT   
  
  
+ CATTCAAAAT ATCTATTTTC TTTTGGGTG  

- ACCACTCATA AAGAAAAAAG GCCACATCTT CGTCCAGCTT CGTGCTCTTT TGGTTTTCTT TGATCGTTTA   
  
  
- GTGGTGGGAT AATGCTAGGG AAGATCGCTA TAGTAAGAGC TCCTATGTTC TTTATATTCT TCCTCGTAAT   
  
  
- CGCTTGACGC TTGTATACCT GTGGCTTTCT CTCCAATATC GGGCTTTATC GATTTCCACT AGTTTCAAAA   
  
  
- CTACTCTTCT TTCTCTACAC CTGACGACAC ACTCCACCCT CTCAAAATCA AAAAAAAAAA GACCTTCCTT   
  
  
- TACTTCTAGC TACCCACCTC ACGAATATTG AACAAACTTT CCACATTCAA AAAACCCTAC CTTCCTCTTC   
  
  
- TCTCACCGGA GTAAATAAAA CAAAACAAAC CAAACATGTT CGATTAGTCG ATTCTTAAGA ACAAATTGAC   
  
  
- CAAAACACAA GATCAAAAAC CAAAGAACCT TGAAACCACC AACAACTCAC TCTTTCAACT CGTTCACTCT   
  
  
- CTCTCTCTCT CCAAAACAAT CCCCATCTTT CCAATCCTCG CTTTTCCTTC ACTCTCTCTC TTTCTCGTCC   
  
  
- ATTCTGATCA CTTTTCCAAC ACTATACTCT CTCTCTCTCT CTCTCCCTTA TTCCAATTTA AAGTGTAGTT   
  
  
- GGAATAAGCT TATTCTCTCT GATAAATCAT CCTATTGAAT TAAGCCAGTG TTTTATACGT TACCACACAC   
  
  
- ATAACTCTCA ACAAAAATGC ACTCCAGCCT ATTATTAGTT TGAATTACCT TTAGTTAAAA AATACTTTCA   
  
  
- CATGCTAGCA GAAGAGATAG GTGACACGCT CAAAAAGAGT GGTTTATGTA TACTTTTACC GTACTAAGTG   
  
  
- GTACGTTCTT TCCCTAAACC TCTTATTAAC CAATATTGTC CAGTACAAAC GGATCGTCGA AGGAAGATGA   
  
  
- GGTAATTAAA CCTGAACACG TAACGTACGG AATATACCCA ACATAAAACA CCTATTTCCT TTTCCTGTTT   
  
  
- CCCACCCGAC CAATGCATCC GAAAAATCAA AATTACTTTG TAAAGGCAAT GACAAGTAAA AATTGTTTTT   
  
  
- ATTGTAAAAA TGAGATTTTT TTAGTGAGGA GCATGATAAG TAAATGTTGT GTAAAAACAG TAAAAATAAT   
  
  
- TTTGAGTTTC AAAAGTTCAA AAAAAGTAAT TAAAAGGAAA AATAAATAAA TAAAAGTCTT AATCCGTTTG   
  
  
- TTAATCGGTG TAATGAACTA GTTAGTTGAT ATACTGTAAC TATCTTAAGA TGAAACGTGA AACGTTGTAG   
  
  
- TTCAAAGGTG AAGTTGGTTG GTCTACCCTA AACGATTAAA ATCAGTAATT GATTTCTAAT AAGCTACAAC   
  
  
- TCATATTCCA AACAGCGAAT TTACTAATTC TCGTAAATAA TCACGTGGAT TAACAACGTG ATGAACTTCA   
  
  
- GCACATAATC TGGTTCTAGT ACGAGTATAT TGAGTACCAT CTAATCCTAC ACTGAATTAA CATCAAACAA   
  
  
- GTAAGTTTTA TAGATAAAAG AAAACCCAC

  
  
Motifs Found  

+   

| Site Name | Organism | Position | Strand | Matrix score. | sequence | function |
| --- | --- | --- | --- | --- | --- | --- |
|  | organism | 150 | - | 4 | motif\_sequence | short\_function |
|  | organism | 347 | - | 4 | motif\_sequence | short\_function |
|  | organism | 1438 | - | 4 | motif\_sequence | short\_function |
|  | organism | 1031 | + | 4 | motif\_sequence | short\_function |
|  | organism | 793 | + | 4 | motif\_sequence | short\_function |
|  | organism | 283 | - | 4 | motif\_sequence | short\_function |
|  | organism | 248 | - | 4 | motif\_sequence | short\_function |

>007212   
+ TGGTGAGTAT TTCTTTTTTC CGGTGTAGAA GCAGGTCGAA GCACGAGAAA ACCAAAAGAA ACTAGCAAAT   
  
  
+ CACCACCCTA TTACGATCCC TTCTAGCGAT ATCATTCTCG AGGATACAAG AAATATAAGA AGGAGCATTA   
  
  
+ GCGAACTGCG AACATATGGA CACCGAAAGA GAGGTTATAG CCCGAAATAG CTAAAGGTGA TCAAAGTTTT   
  
  
+ GATGAGAAGA AAGAGATGTG GACTGCTGTG TGAGGTGGGA GAGTTTTAGT TTTTTTTTTT CTGGAAGGAA   
  
  
+ ATGAAGATCG ATGGGTGGAG TGCTTATAAC TTGTTTGAAA GGTGTAAGTT TTTTGGGATG GAAGGAGAAG   
  
  
+ AGAGTGGCCT CATTTATTTT GTTTTGTTTG GTTTGTACAA GCTAATCAGC TAAGAATTCT TGTTTAACTG   
  
  
+ GTTTTGTGTT CTAGTTTTTG GTTTCTTGGA ACTTTGGTGG TTGTTGAGTG AGAAAGTTGA GCAAGTGAGA   
  
  
+ GAGAGAGAGA GGTTTTGTTA GGGGTAGAAA GGTTAGGAGC GAAAAGGAAG TGAGAGAGAG AAAGAGCAGG   
  
  
+ TAAGACTAGT GAAAAGGTTG TGATATGAGA GAGAGAGAGA GAGAGGGAAT AAGGTTAAAT TTCACATCAA   
  
  
+ CCTTATTCGA ATAAGAGAGA CTATTTAGTA GGATAACTTA ATTCGGTCAC AAAATATGCA ATGGTGTGTG   
  
  
+ TATTGAGAGT TGTTTTTACG TGAGGTCGGA TAATAATCAA ACTTAATGGA AATCAATTTT TTATGAAAGT   
  
  
+ GTACGATCGT CTTCTCTATC CACTGTGCGA GTTTTTCTCA CCAAATACAT ATGAAAATGG CATGATTCAC   
  
  
+ CATGCAAGAA AGGGATTTGG AGAATAATTG GTTATAACAG GTCATGTTTG CCTAGCAGCT TCCTTCTACT   
  
  
+ CCATTAATTT GGACTTGTGC ATTGCATGCC TTATATGGGT TGTATTTTGT GGATAAAGGA AAAGGACAAA   
  
  
+ GGGTGGGCTG GTTACGTAGG CTTTTTAGTT TTAATGAAAC ATTTCCGTTA CTGTTCATTT TTAACAAAAA   
  
  
+ TAACATTTTT ACTCTAAAAA AATCACTCCT CGTACTATTC ATTTACAACA CATTTTTGTC ATTTTTATTA   
  
  
+ AAACTCAAAG TTTTCAAGTT TTTTTCATTA ATTTTCCTTT TTATTTATTT ATTTTCAGAA TTAGGCAAAC   
  
  
+ AATTAGCCAC ATTACTTGAT CAATCAACTA TATGACATTG ATAGAATTCT ACTTTGCACT TTGCAACATC   
  
  
+ AAGTTTCCAC TTCAACCAAC CAGATGGGAT TTGCTAATTT TAGTCATTAA CTAAAGATTA TTCGATGTTG   
  
  
+ AGTATAAGGT TTGTCGCTTA AATGATTAAG AGCATTTATT AGTGCACCTA ATTGTTGCAC TACTTGAAGT   
  
  
+ CGTGTATTAG ACCAAGATCA TGCTCATATA ACTCATGGTA GATTAGGATG TGACTTAATT GTAGTTTGTT   
  
  
+ CATTCAAAAT ATCTATTTTC TTTTGGGTG  

- ACCACTCATA AAGAAAAAAG GCCACATCTT CGTCCAGCTT CGTGCTCTTT TGGTTTTCTT TGATCGTTTA   
  
  
- GTGGTGGGAT AATGCTAGGG AAGATCGCTA TAGTAAGAGC TCCTATGTTC TTTATATTCT TCCTCGTAAT   
  
  
- CGCTTGACGC TTGTATACCT GTGGCTTTCT CTCCAATATC GGGCTTTATC GATTTCCACT AGTTTCAAAA   
  
  
- CTACTCTTCT TTCTCTACAC CTGACGACAC ACTCCACCCT CTCAAAATCA AAAAAAAAAA GACCTTCCTT   
  
  
- TACTTCTAGC TACCCACCTC ACGAATATTG AACAAACTTT CCACATTCAA AAAACCCTAC CTTCCTCTTC   
  
  
- TCTCACCGGA GTAAATAAAA CAAAACAAAC CAAACATGTT CGATTAGTCG ATTCTTAAGA ACAAATTGAC   
  
  
- CAAAACACAA GATCAAAAAC CAAAGAACCT TGAAACCACC AACAACTCAC TCTTTCAACT CGTTCACTCT   
  
  
- CTCTCTCTCT CCAAAACAAT CCCCATCTTT CCAATCCTCG CTTTTCCTTC ACTCTCTCTC TTTCTCGTCC   
  
  
- ATTCTGATCA CTTTTCCAAC ACTATACTCT CTCTCTCTCT CTCTCCCTTA TTCCAATTTA AAGTGTAGTT   
  
  
- GGAATAAGCT TATTCTCTCT GATAAATCAT CCTATTGAAT TAAGCCAGTG TTTTATACGT TACCACACAC   
  
  
- ATAACTCTCA ACAAAAATGC ACTCCAGCCT ATTATTAGTT TGAATTACCT TTAGTTAAAA AATACTTTCA   
  
  
- CATGCTAGCA GAAGAGATAG GTGACACGCT CAAAAAGAGT GGTTTATGTA TACTTTTACC GTACTAAGTG   
  
  
- GTACGTTCTT TCCCTAAACC TCTTATTAAC CAATATTGTC CAGTACAAAC GGATCGTCGA AGGAAGATGA   
  
  
- GGTAATTAAA CCTGAACACG TAACGTACGG AATATACCCA ACATAAAACA CCTATTTCCT TTTCCTGTTT   
  
  
- CCCACCCGAC CAATGCATCC GAAAAATCAA AATTACTTTG TAAAGGCAAT GACAAGTAAA AATTGTTTTT   
  
  
- ATTGTAAAAA TGAGATTTTT TTAGTGAGGA GCATGATAAG TAAATGTTGT GTAAAAACAG TAAAAATAAT   
  
  
- TTTGAGTTTC AAAAGTTCAA AAAAAGTAAT TAAAAGGAAA AATAAATAAA TAAAAGTCTT AATCCGTTTG   
  
  
- TTAATCGGTG TAATGAACTA GTTAGTTGAT ATACTGTAAC TATCTTAAGA TGAAACGTGA AACGTTGTAG   
  
  
- TTCAAAGGTG AAGTTGGTTG GTCTACCCTA AACGATTAAA ATCAGTAATT GATTTCTAAT AAGCTACAAC   
  
  
- TCATATTCCA AACAGCGAAT TTACTAATTC TCGTAAATAA TCACGTGGAT TAACAACGTG ATGAACTTCA   
  
  
- GCACATAATC TGGTTCTAGT ACGAGTATAT TGAGTACCAT CTAATCCTAC ACTGAATTAA CATCAAACAA   
  
  
- GTAAGTTTTA TAGATAAAAG AAAACCCAC

+     ABRE

| Site Name | Organism | Position | Strand | Matrix score. | sequence | function |
| --- | --- | --- | --- | --- | --- | --- |
| ABRE | Arabidopsis thaliana | 718 | + | 5 | ACGTG | cis-acting element involved in the abscisic acid responsiveness |

>007212   
+ TGGTGAGTAT TTCTTTTTTC CGGTGTAGAA GCAGGTCGAA GCACGAGAAA ACCAAAAGAA ACTAGCAAAT   
  
  
+ CACCACCCTA TTACGATCCC TTCTAGCGAT ATCATTCTCG AGGATACAAG AAATATAAGA AGGAGCATTA   
  
  
+ GCGAACTGCG AACATATGGA CACCGAAAGA GAGGTTATAG CCCGAAATAG CTAAAGGTGA TCAAAGTTTT   
  
  
+ GATGAGAAGA AAGAGATGTG GACTGCTGTG TGAGGTGGGA GAGTTTTAGT TTTTTTTTTT CTGGAAGGAA   
  
  
+ ATGAAGATCG ATGGGTGGAG TGCTTATAAC TTGTTTGAAA GGTGTAAGTT TTTTGGGATG GAAGGAGAAG   
  
  
+ AGAGTGGCCT CATTTATTTT GTTTTGTTTG GTTTGTACAA GCTAATCAGC TAAGAATTCT TGTTTAACTG   
  
  
+ GTTTTGTGTT CTAGTTTTTG GTTTCTTGGA ACTTTGGTGG TTGTTGAGTG AGAAAGTTGA GCAAGTGAGA   
  
  
+ GAGAGAGAGA GGTTTTGTTA GGGGTAGAAA GGTTAGGAGC GAAAAGGAAG TGAGAGAGAG AAAGAGCAGG   
  
  
+ TAAGACTAGT GAAAAGGTTG TGATATGAGA GAGAGAGAGA GAGAGGGAAT AAGGTTAAAT TTCACATCAA   
  
  
+ CCTTATTCGA ATAAGAGAGA CTATTTAGTA GGATAACTTA ATTCGGTCAC AAAATATGCA ATGGTGTGTG   
  
  
+ TATTGAGAGT TGTTTTTACG TGAGGTCGGA TAATAATCAA ACTTAATGGA AATCAATTTT TTATGAAAGT   
  
  
+ GTACGATCGT CTTCTCTATC CACTGTGCGA GTTTTTCTCA CCAAATACAT ATGAAAATGG CATGATTCAC   
  
  
+ CATGCAAGAA AGGGATTTGG AGAATAATTG GTTATAACAG GTCATGTTTG CCTAGCAGCT TCCTTCTACT   
  
  
+ CCATTAATTT GGACTTGTGC ATTGCATGCC TTATATGGGT TGTATTTTGT GGATAAAGGA AAAGGACAAA   
  
  
+ GGGTGGGCTG GTTACGTAGG CTTTTTAGTT TTAATGAAAC ATTTCCGTTA CTGTTCATTT TTAACAAAAA   
  
  
+ TAACATTTTT ACTCTAAAAA AATCACTCCT CGTACTATTC ATTTACAACA CATTTTTGTC ATTTTTATTA   
  
  
+ AAACTCAAAG TTTTCAAGTT TTTTTCATTA ATTTTCCTTT TTATTTATTT ATTTTCAGAA TTAGGCAAAC   
  
  
+ AATTAGCCAC ATTACTTGAT CAATCAACTA TATGACATTG ATAGAATTCT ACTTTGCACT TTGCAACATC   
  
  
+ AAGTTTCCAC TTCAACCAAC CAGATGGGAT TTGCTAATTT TAGTCATTAA CTAAAGATTA TTCGATGTTG   
  
  
+ AGTATAAGGT TTGTCGCTTA AATGATTAAG AGCATTTATT AGTGCACCTA ATTGTTGCAC TACTTGAAGT   
  
  
+ CGTGTATTAG ACCAAGATCA TGCTCATATA ACTCATGGTA GATTAGGATG TGACTTAATT GTAGTTTGTT   
  
  
+ CATTCAAAAT ATCTATTTTC TTTTGGGTG  

- ACCACTCATA AAGAAAAAAG GCCACATCTT CGTCCAGCTT CGTGCTCTTT TGGTTTTCTT TGATCGTTTA   
  
  
- GTGGTGGGAT AATGCTAGGG AAGATCGCTA TAGTAAGAGC TCCTATGTTC TTTATATTCT TCCTCGTAAT   
  
  
- CGCTTGACGC TTGTATACCT GTGGCTTTCT CTCCAATATC GGGCTTTATC GATTTCCACT AGTTTCAAAA   
  
  
- CTACTCTTCT TTCTCTACAC CTGACGACAC ACTCCACCCT CTCAAAATCA AAAAAAAAAA GACCTTCCTT   
  
  
- TACTTCTAGC TACCCACCTC ACGAATATTG AACAAACTTT CCACATTCAA AAAACCCTAC CTTCCTCTTC   
  
  
- TCTCACCGGA GTAAATAAAA CAAAACAAAC CAAACATGTT CGATTAGTCG ATTCTTAAGA ACAAATTGAC   
  
  
- CAAAACACAA GATCAAAAAC CAAAGAACCT TGAAACCACC AACAACTCAC TCTTTCAACT CGTTCACTCT   
  
  
- CTCTCTCTCT CCAAAACAAT CCCCATCTTT CCAATCCTCG CTTTTCCTTC ACTCTCTCTC TTTCTCGTCC   
  
  
- ATTCTGATCA CTTTTCCAAC ACTATACTCT CTCTCTCTCT CTCTCCCTTA TTCCAATTTA AAGTGTAGTT   
  
  
- GGAATAAGCT TATTCTCTCT GATAAATCAT CCTATTGAAT TAAGCCAGTG TTTTATACGT TACCACACAC   
  
  
- ATAACTCTCA ACAAAAATGC ACTCCAGCCT ATTATTAGTT TGAATTACCT TTAGTTAAAA AATACTTTCA   
  
  
- CATGCTAGCA GAAGAGATAG GTGACACGCT CAAAAAGAGT GGTTTATGTA TACTTTTACC GTACTAAGTG   
  
  
- GTACGTTCTT TCCCTAAACC TCTTATTAAC CAATATTGTC CAGTACAAAC GGATCGTCGA AGGAAGATGA   
  
  
- GGTAATTAAA CCTGAACACG TAACGTACGG AATATACCCA ACATAAAACA CCTATTTCCT TTTCCTGTTT   
  
  
- CCCACCCGAC CAATGCATCC GAAAAATCAA AATTACTTTG TAAAGGCAAT GACAAGTAAA AATTGTTTTT   
  
  
- ATTGTAAAAA TGAGATTTTT TTAGTGAGGA GCATGATAAG TAAATGTTGT GTAAAAACAG TAAAAATAAT   
  
  
- TTTGAGTTTC AAAAGTTCAA AAAAAGTAAT TAAAAGGAAA AATAAATAAA TAAAAGTCTT AATCCGTTTG   
  
  
- TTAATCGGTG TAATGAACTA GTTAGTTGAT ATACTGTAAC TATCTTAAGA TGAAACGTGA AACGTTGTAG   
  
  
- TTCAAAGGTG AAGTTGGTTG GTCTACCCTA AACGATTAAA ATCAGTAATT GATTTCTAAT AAGCTACAAC   
  
  
- TCATATTCCA AACAGCGAAT TTACTAATTC TCGTAAATAA TCACGTGGAT TAACAACGTG ATGAACTTCA   
  
  
- GCACATAATC TGGTTCTAGT ACGAGTATAT TGAGTACCAT CTAATCCTAC ACTGAATTAA CATCAAACAA   
  
  
- GTAAGTTTTA TAGATAAAAG AAAACCCAC

+     ABRE3a

| Site Name | Organism | Position | Strand | Matrix score. | sequence | function |
| --- | --- | --- | --- | --- | --- | --- |
| ABRE3a | Zea mays | 717 | + | 6 | TACGTG |  |

>007212   
+ TGGTGAGTAT TTCTTTTTTC CGGTGTAGAA GCAGGTCGAA GCACGAGAAA ACCAAAAGAA ACTAGCAAAT   
  
  
+ CACCACCCTA TTACGATCCC TTCTAGCGAT ATCATTCTCG AGGATACAAG AAATATAAGA AGGAGCATTA   
  
  
+ GCGAACTGCG AACATATGGA CACCGAAAGA GAGGTTATAG CCCGAAATAG CTAAAGGTGA TCAAAGTTTT   
  
  
+ GATGAGAAGA AAGAGATGTG GACTGCTGTG TGAGGTGGGA GAGTTTTAGT TTTTTTTTTT CTGGAAGGAA   
  
  
+ ATGAAGATCG ATGGGTGGAG TGCTTATAAC TTGTTTGAAA GGTGTAAGTT TTTTGGGATG GAAGGAGAAG   
  
  
+ AGAGTGGCCT CATTTATTTT GTTTTGTTTG GTTTGTACAA GCTAATCAGC TAAGAATTCT TGTTTAACTG   
  
  
+ GTTTTGTGTT CTAGTTTTTG GTTTCTTGGA ACTTTGGTGG TTGTTGAGTG AGAAAGTTGA GCAAGTGAGA   
  
  
+ GAGAGAGAGA GGTTTTGTTA GGGGTAGAAA GGTTAGGAGC GAAAAGGAAG TGAGAGAGAG AAAGAGCAGG   
  
  
+ TAAGACTAGT GAAAAGGTTG TGATATGAGA GAGAGAGAGA GAGAGGGAAT AAGGTTAAAT TTCACATCAA   
  
  
+ CCTTATTCGA ATAAGAGAGA CTATTTAGTA GGATAACTTA ATTCGGTCAC AAAATATGCA ATGGTGTGTG   
  
  
+ TATTGAGAGT TGTTTTTACG TGAGGTCGGA TAATAATCAA ACTTAATGGA AATCAATTTT TTATGAAAGT   
  
  
+ GTACGATCGT CTTCTCTATC CACTGTGCGA GTTTTTCTCA CCAAATACAT ATGAAAATGG CATGATTCAC   
  
  
+ CATGCAAGAA AGGGATTTGG AGAATAATTG GTTATAACAG GTCATGTTTG CCTAGCAGCT TCCTTCTACT   
  
  
+ CCATTAATTT GGACTTGTGC ATTGCATGCC TTATATGGGT TGTATTTTGT GGATAAAGGA AAAGGACAAA   
  
  
+ GGGTGGGCTG GTTACGTAGG CTTTTTAGTT TTAATGAAAC ATTTCCGTTA CTGTTCATTT TTAACAAAAA   
  
  
+ TAACATTTTT ACTCTAAAAA AATCACTCCT CGTACTATTC ATTTACAACA CATTTTTGTC ATTTTTATTA   
  
  
+ AAACTCAAAG TTTTCAAGTT TTTTTCATTA ATTTTCCTTT TTATTTATTT ATTTTCAGAA TTAGGCAAAC   
  
  
+ AATTAGCCAC ATTACTTGAT CAATCAACTA TATGACATTG ATAGAATTCT ACTTTGCACT TTGCAACATC   
  
  
+ AAGTTTCCAC TTCAACCAAC CAGATGGGAT TTGCTAATTT TAGTCATTAA CTAAAGATTA TTCGATGTTG   
  
  
+ AGTATAAGGT TTGTCGCTTA AATGATTAAG AGCATTTATT AGTGCACCTA ATTGTTGCAC TACTTGAAGT   
  
  
+ CGTGTATTAG ACCAAGATCA TGCTCATATA ACTCATGGTA GATTAGGATG TGACTTAATT GTAGTTTGTT   
  
  
+ CATTCAAAAT ATCTATTTTC TTTTGGGTG  

- ACCACTCATA AAGAAAAAAG GCCACATCTT CGTCCAGCTT CGTGCTCTTT TGGTTTTCTT TGATCGTTTA   
  
  
- GTGGTGGGAT AATGCTAGGG AAGATCGCTA TAGTAAGAGC TCCTATGTTC TTTATATTCT TCCTCGTAAT   
  
  
- CGCTTGACGC TTGTATACCT GTGGCTTTCT CTCCAATATC GGGCTTTATC GATTTCCACT AGTTTCAAAA   
  
  
- CTACTCTTCT TTCTCTACAC CTGACGACAC ACTCCACCCT CTCAAAATCA AAAAAAAAAA GACCTTCCTT   
  
  
- TACTTCTAGC TACCCACCTC ACGAATATTG AACAAACTTT CCACATTCAA AAAACCCTAC CTTCCTCTTC   
  
  
- TCTCACCGGA GTAAATAAAA CAAAACAAAC CAAACATGTT CGATTAGTCG ATTCTTAAGA ACAAATTGAC   
  
  
- CAAAACACAA GATCAAAAAC CAAAGAACCT TGAAACCACC AACAACTCAC TCTTTCAACT CGTTCACTCT   
  
  
- CTCTCTCTCT CCAAAACAAT CCCCATCTTT CCAATCCTCG CTTTTCCTTC ACTCTCTCTC TTTCTCGTCC   
  
  
- ATTCTGATCA CTTTTCCAAC ACTATACTCT CTCTCTCTCT CTCTCCCTTA TTCCAATTTA AAGTGTAGTT   
  
  
- GGAATAAGCT TATTCTCTCT GATAAATCAT CCTATTGAAT TAAGCCAGTG TTTTATACGT TACCACACAC   
  
  
- ATAACTCTCA ACAAAAATGC ACTCCAGCCT ATTATTAGTT TGAATTACCT TTAGTTAAAA AATACTTTCA   
  
  
- CATGCTAGCA GAAGAGATAG GTGACACGCT CAAAAAGAGT GGTTTATGTA TACTTTTACC GTACTAAGTG   
  
  
- GTACGTTCTT TCCCTAAACC TCTTATTAAC CAATATTGTC CAGTACAAAC GGATCGTCGA AGGAAGATGA   
  
  
- GGTAATTAAA CCTGAACACG TAACGTACGG AATATACCCA ACATAAAACA CCTATTTCCT TTTCCTGTTT   
  
  
- CCCACCCGAC CAATGCATCC GAAAAATCAA AATTACTTTG TAAAGGCAAT GACAAGTAAA AATTGTTTTT   
  
  
- ATTGTAAAAA TGAGATTTTT TTAGTGAGGA GCATGATAAG TAAATGTTGT GTAAAAACAG TAAAAATAAT   
  
  
- TTTGAGTTTC AAAAGTTCAA AAAAAGTAAT TAAAAGGAAA AATAAATAAA TAAAAGTCTT AATCCGTTTG   
  
  
- TTAATCGGTG TAATGAACTA GTTAGTTGAT ATACTGTAAC TATCTTAAGA TGAAACGTGA AACGTTGTAG   
  
  
- TTCAAAGGTG AAGTTGGTTG GTCTACCCTA AACGATTAAA ATCAGTAATT GATTTCTAAT AAGCTACAAC   
  
  
- TCATATTCCA AACAGCGAAT TTACTAATTC TCGTAAATAA TCACGTGGAT TAACAACGTG ATGAACTTCA   
  
  
- GCACATAATC TGGTTCTAGT ACGAGTATAT TGAGTACCAT CTAATCCTAC ACTGAATTAA CATCAAACAA   
  
  
- GTAAGTTTTA TAGATAAAAG AAAACCCAC

+     ABRE4

| Site Name | Organism | Position | Strand | Matrix score. | sequence | function |
| --- | --- | --- | --- | --- | --- | --- |
| ABRE4 | Zea mays | 717 | - | 6 | CACGTA |  |

>007212   
+ TGGTGAGTAT TTCTTTTTTC CGGTGTAGAA GCAGGTCGAA GCACGAGAAA ACCAAAAGAA ACTAGCAAAT   
  
  
+ CACCACCCTA TTACGATCCC TTCTAGCGAT ATCATTCTCG AGGATACAAG AAATATAAGA AGGAGCATTA   
  
  
+ GCGAACTGCG AACATATGGA CACCGAAAGA GAGGTTATAG CCCGAAATAG CTAAAGGTGA TCAAAGTTTT   
  
  
+ GATGAGAAGA AAGAGATGTG GACTGCTGTG TGAGGTGGGA GAGTTTTAGT TTTTTTTTTT CTGGAAGGAA   
  
  
+ ATGAAGATCG ATGGGTGGAG TGCTTATAAC TTGTTTGAAA GGTGTAAGTT TTTTGGGATG GAAGGAGAAG   
  
  
+ AGAGTGGCCT CATTTATTTT GTTTTGTTTG GTTTGTACAA GCTAATCAGC TAAGAATTCT TGTTTAACTG   
  
  
+ GTTTTGTGTT CTAGTTTTTG GTTTCTTGGA ACTTTGGTGG TTGTTGAGTG AGAAAGTTGA GCAAGTGAGA   
  
  
+ GAGAGAGAGA GGTTTTGTTA GGGGTAGAAA GGTTAGGAGC GAAAAGGAAG TGAGAGAGAG AAAGAGCAGG   
  
  
+ TAAGACTAGT GAAAAGGTTG TGATATGAGA GAGAGAGAGA GAGAGGGAAT AAGGTTAAAT TTCACATCAA   
  
  
+ CCTTATTCGA ATAAGAGAGA CTATTTAGTA GGATAACTTA ATTCGGTCAC AAAATATGCA ATGGTGTGTG   
  
  
+ TATTGAGAGT TGTTTTTACG TGAGGTCGGA TAATAATCAA ACTTAATGGA AATCAATTTT TTATGAAAGT   
  
  
+ GTACGATCGT CTTCTCTATC CACTGTGCGA GTTTTTCTCA CCAAATACAT ATGAAAATGG CATGATTCAC   
  
  
+ CATGCAAGAA AGGGATTTGG AGAATAATTG GTTATAACAG GTCATGTTTG CCTAGCAGCT TCCTTCTACT   
  
  
+ CCATTAATTT GGACTTGTGC ATTGCATGCC TTATATGGGT TGTATTTTGT GGATAAAGGA AAAGGACAAA   
  
  
+ GGGTGGGCTG GTTACGTAGG CTTTTTAGTT TTAATGAAAC ATTTCCGTTA CTGTTCATTT TTAACAAAAA   
  
  
+ TAACATTTTT ACTCTAAAAA AATCACTCCT CGTACTATTC ATTTACAACA CATTTTTGTC ATTTTTATTA   
  
  
+ AAACTCAAAG TTTTCAAGTT TTTTTCATTA ATTTTCCTTT TTATTTATTT ATTTTCAGAA TTAGGCAAAC   
  
  
+ AATTAGCCAC ATTACTTGAT CAATCAACTA TATGACATTG ATAGAATTCT ACTTTGCACT TTGCAACATC   
  
  
+ AAGTTTCCAC TTCAACCAAC CAGATGGGAT TTGCTAATTT TAGTCATTAA CTAAAGATTA TTCGATGTTG   
  
  
+ AGTATAAGGT TTGTCGCTTA AATGATTAAG AGCATTTATT AGTGCACCTA ATTGTTGCAC TACTTGAAGT   
  
  
+ CGTGTATTAG ACCAAGATCA TGCTCATATA ACTCATGGTA GATTAGGATG TGACTTAATT GTAGTTTGTT   
  
  
+ CATTCAAAAT ATCTATTTTC TTTTGGGTG  

- ACCACTCATA AAGAAAAAAG GCCACATCTT CGTCCAGCTT CGTGCTCTTT TGGTTTTCTT TGATCGTTTA   
  
  
- GTGGTGGGAT AATGCTAGGG AAGATCGCTA TAGTAAGAGC TCCTATGTTC TTTATATTCT TCCTCGTAAT   
  
  
- CGCTTGACGC TTGTATACCT GTGGCTTTCT CTCCAATATC GGGCTTTATC GATTTCCACT AGTTTCAAAA   
  
  
- CTACTCTTCT TTCTCTACAC CTGACGACAC ACTCCACCCT CTCAAAATCA AAAAAAAAAA GACCTTCCTT   
  
  
- TACTTCTAGC TACCCACCTC ACGAATATTG AACAAACTTT CCACATTCAA AAAACCCTAC CTTCCTCTTC   
  
  
- TCTCACCGGA GTAAATAAAA CAAAACAAAC CAAACATGTT CGATTAGTCG ATTCTTAAGA ACAAATTGAC   
  
  
- CAAAACACAA GATCAAAAAC CAAAGAACCT TGAAACCACC AACAACTCAC TCTTTCAACT CGTTCACTCT   
  
  
- CTCTCTCTCT CCAAAACAAT CCCCATCTTT CCAATCCTCG CTTTTCCTTC ACTCTCTCTC TTTCTCGTCC   
  
  
- ATTCTGATCA CTTTTCCAAC ACTATACTCT CTCTCTCTCT CTCTCCCTTA TTCCAATTTA AAGTGTAGTT   
  
  
- GGAATAAGCT TATTCTCTCT GATAAATCAT CCTATTGAAT TAAGCCAGTG TTTTATACGT TACCACACAC   
  
  
- ATAACTCTCA ACAAAAATGC ACTCCAGCCT ATTATTAGTT TGAATTACCT TTAGTTAAAA AATACTTTCA   
  
  
- CATGCTAGCA GAAGAGATAG GTGACACGCT CAAAAAGAGT GGTTTATGTA TACTTTTACC GTACTAAGTG   
  
  
- GTACGTTCTT TCCCTAAACC TCTTATTAAC CAATATTGTC CAGTACAAAC GGATCGTCGA AGGAAGATGA   
  
  
- GGTAATTAAA CCTGAACACG TAACGTACGG AATATACCCA ACATAAAACA CCTATTTCCT TTTCCTGTTT   
  
  
- CCCACCCGAC CAATGCATCC GAAAAATCAA AATTACTTTG TAAAGGCAAT GACAAGTAAA AATTGTTTTT   
  
  
- ATTGTAAAAA TGAGATTTTT TTAGTGAGGA GCATGATAAG TAAATGTTGT GTAAAAACAG TAAAAATAAT   
  
  
- TTTGAGTTTC AAAAGTTCAA AAAAAGTAAT TAAAAGGAAA AATAAATAAA TAAAAGTCTT AATCCGTTTG   
  
  
- TTAATCGGTG TAATGAACTA GTTAGTTGAT ATACTGTAAC TATCTTAAGA TGAAACGTGA AACGTTGTAG   
  
  
- TTCAAAGGTG AAGTTGGTTG GTCTACCCTA AACGATTAAA ATCAGTAATT GATTTCTAAT AAGCTACAAC   
  
  
- TCATATTCCA AACAGCGAAT TTACTAATTC TCGTAAATAA TCACGTGGAT TAACAACGTG ATGAACTTCA   
  
  
- GCACATAATC TGGTTCTAGT ACGAGTATAT TGAGTACCAT CTAATCCTAC ACTGAATTAA CATCAAACAA   
  
  
- GTAAGTTTTA TAGATAAAAG AAAACCCAC

+     ARE

| Site Name | Organism | Position | Strand | Matrix score. | sequence | function |
| --- | --- | --- | --- | --- | --- | --- |
| ARE | Zea mays | 379 | - | 6 | AAACCA | cis-acting regulatory element essential for the anaerobic induction |
| ARE | Zea mays | 49 | + | 6 | AAACCA | cis-acting regulatory element essential for the anaerobic induction |
| ARE | Zea mays | 439 | - | 6 | AAACCA | cis-acting regulatory element essential for the anaerobic induction |
| ARE | Zea mays | 419 | - | 6 | AAACCA | cis-acting regulatory element essential for the anaerobic induction |

>007212   
+ TGGTGAGTAT TTCTTTTTTC CGGTGTAGAA GCAGGTCGAA GCACGAGAAA ACCAAAAGAA ACTAGCAAAT   
  
  
+ CACCACCCTA TTACGATCCC TTCTAGCGAT ATCATTCTCG AGGATACAAG AAATATAAGA AGGAGCATTA   
  
  
+ GCGAACTGCG AACATATGGA CACCGAAAGA GAGGTTATAG CCCGAAATAG CTAAAGGTGA TCAAAGTTTT   
  
  
+ GATGAGAAGA AAGAGATGTG GACTGCTGTG TGAGGTGGGA GAGTTTTAGT TTTTTTTTTT CTGGAAGGAA   
  
  
+ ATGAAGATCG ATGGGTGGAG TGCTTATAAC TTGTTTGAAA GGTGTAAGTT TTTTGGGATG GAAGGAGAAG   
  
  
+ AGAGTGGCCT CATTTATTTT GTTTTGTTTG GTTTGTACAA GCTAATCAGC TAAGAATTCT TGTTTAACTG   
  
  
+ GTTTTGTGTT CTAGTTTTTG GTTTCTTGGA ACTTTGGTGG TTGTTGAGTG AGAAAGTTGA GCAAGTGAGA   
  
  
+ GAGAGAGAGA GGTTTTGTTA GGGGTAGAAA GGTTAGGAGC GAAAAGGAAG TGAGAGAGAG AAAGAGCAGG   
  
  
+ TAAGACTAGT GAAAAGGTTG TGATATGAGA GAGAGAGAGA GAGAGGGAAT AAGGTTAAAT TTCACATCAA   
  
  
+ CCTTATTCGA ATAAGAGAGA CTATTTAGTA GGATAACTTA ATTCGGTCAC AAAATATGCA ATGGTGTGTG   
  
  
+ TATTGAGAGT TGTTTTTACG TGAGGTCGGA TAATAATCAA ACTTAATGGA AATCAATTTT TTATGAAAGT   
  
  
+ GTACGATCGT CTTCTCTATC CACTGTGCGA GTTTTTCTCA CCAAATACAT ATGAAAATGG CATGATTCAC   
  
  
+ CATGCAAGAA AGGGATTTGG AGAATAATTG GTTATAACAG GTCATGTTTG CCTAGCAGCT TCCTTCTACT   
  
  
+ CCATTAATTT GGACTTGTGC ATTGCATGCC TTATATGGGT TGTATTTTGT GGATAAAGGA AAAGGACAAA   
  
  
+ GGGTGGGCTG GTTACGTAGG CTTTTTAGTT TTAATGAAAC ATTTCCGTTA CTGTTCATTT TTAACAAAAA   
  
  
+ TAACATTTTT ACTCTAAAAA AATCACTCCT CGTACTATTC ATTTACAACA CATTTTTGTC ATTTTTATTA   
  
  
+ AAACTCAAAG TTTTCAAGTT TTTTTCATTA ATTTTCCTTT TTATTTATTT ATTTTCAGAA TTAGGCAAAC   
  
  
+ AATTAGCCAC ATTACTTGAT CAATCAACTA TATGACATTG ATAGAATTCT ACTTTGCACT TTGCAACATC   
  
  
+ AAGTTTCCAC TTCAACCAAC CAGATGGGAT TTGCTAATTT TAGTCATTAA CTAAAGATTA TTCGATGTTG   
  
  
+ AGTATAAGGT TTGTCGCTTA AATGATTAAG AGCATTTATT AGTGCACCTA ATTGTTGCAC TACTTGAAGT   
  
  
+ CGTGTATTAG ACCAAGATCA TGCTCATATA ACTCATGGTA GATTAGGATG TGACTTAATT GTAGTTTGTT   
  
  
+ CATTCAAAAT ATCTATTTTC TTTTGGGTG  

- ACCACTCATA AAGAAAAAAG GCCACATCTT CGTCCAGCTT CGTGCTCTTT TGGTTTTCTT TGATCGTTTA   
  
  
- GTGGTGGGAT AATGCTAGGG AAGATCGCTA TAGTAAGAGC TCCTATGTTC TTTATATTCT TCCTCGTAAT   
  
  
- CGCTTGACGC TTGTATACCT GTGGCTTTCT CTCCAATATC GGGCTTTATC GATTTCCACT AGTTTCAAAA   
  
  
- CTACTCTTCT TTCTCTACAC CTGACGACAC ACTCCACCCT CTCAAAATCA AAAAAAAAAA GACCTTCCTT   
  
  
- TACTTCTAGC TACCCACCTC ACGAATATTG AACAAACTTT CCACATTCAA AAAACCCTAC CTTCCTCTTC   
  
  
- TCTCACCGGA GTAAATAAAA CAAAACAAAC CAAACATGTT CGATTAGTCG ATTCTTAAGA ACAAATTGAC   
  
  
- CAAAACACAA GATCAAAAAC CAAAGAACCT TGAAACCACC AACAACTCAC TCTTTCAACT CGTTCACTCT   
  
  
- CTCTCTCTCT CCAAAACAAT CCCCATCTTT CCAATCCTCG CTTTTCCTTC ACTCTCTCTC TTTCTCGTCC   
  
  
- ATTCTGATCA CTTTTCCAAC ACTATACTCT CTCTCTCTCT CTCTCCCTTA TTCCAATTTA AAGTGTAGTT   
  
  
- GGAATAAGCT TATTCTCTCT GATAAATCAT CCTATTGAAT TAAGCCAGTG TTTTATACGT TACCACACAC   
  
  
- ATAACTCTCA ACAAAAATGC ACTCCAGCCT ATTATTAGTT TGAATTACCT TTAGTTAAAA AATACTTTCA   
  
  
- CATGCTAGCA GAAGAGATAG GTGACACGCT CAAAAAGAGT GGTTTATGTA TACTTTTACC GTACTAAGTG   
  
  
- GTACGTTCTT TCCCTAAACC TCTTATTAAC CAATATTGTC CAGTACAAAC GGATCGTCGA AGGAAGATGA   
  
  
- GGTAATTAAA CCTGAACACG TAACGTACGG AATATACCCA ACATAAAACA CCTATTTCCT TTTCCTGTTT   
  
  
- CCCACCCGAC CAATGCATCC GAAAAATCAA AATTACTTTG TAAAGGCAAT GACAAGTAAA AATTGTTTTT   
  
  
- ATTGTAAAAA TGAGATTTTT TTAGTGAGGA GCATGATAAG TAAATGTTGT GTAAAAACAG TAAAAATAAT   
  
  
- TTTGAGTTTC AAAAGTTCAA AAAAAGTAAT TAAAAGGAAA AATAAATAAA TAAAAGTCTT AATCCGTTTG   
  
  
- TTAATCGGTG TAATGAACTA GTTAGTTGAT ATACTGTAAC TATCTTAAGA TGAAACGTGA AACGTTGTAG   
  
  
- TTCAAAGGTG AAGTTGGTTG GTCTACCCTA AACGATTAAA ATCAGTAATT GATTTCTAAT AAGCTACAAC   
  
  
- TCATATTCCA AACAGCGAAT TTACTAATTC TCGTAAATAA TCACGTGGAT TAACAACGTG ATGAACTTCA   
  
  
- GCACATAATC TGGTTCTAGT ACGAGTATAT TGAGTACCAT CTAATCCTAC ACTGAATTAA CATCAAACAA   
  
  
- GTAAGTTTTA TAGATAAAAG AAAACCCAC

+     AT-rich element

| Site Name | Organism | Position | Strand | Matrix score. | sequence | function |
| --- | --- | --- | --- | --- | --- | --- |
| AT-rich element | Glycine max | 746 | + | 10 | ATAGAAATCAA | binding site of AT-rich DNA binding protein (ATBP-1) |

>007212   
+ TGGTGAGTAT TTCTTTTTTC CGGTGTAGAA GCAGGTCGAA GCACGAGAAA ACCAAAAGAA ACTAGCAAAT   
  
  
+ CACCACCCTA TTACGATCCC TTCTAGCGAT ATCATTCTCG AGGATACAAG AAATATAAGA AGGAGCATTA   
  
  
+ GCGAACTGCG AACATATGGA CACCGAAAGA GAGGTTATAG CCCGAAATAG CTAAAGGTGA TCAAAGTTTT   
  
  
+ GATGAGAAGA AAGAGATGTG GACTGCTGTG TGAGGTGGGA GAGTTTTAGT TTTTTTTTTT CTGGAAGGAA   
  
  
+ ATGAAGATCG ATGGGTGGAG TGCTTATAAC TTGTTTGAAA GGTGTAAGTT TTTTGGGATG GAAGGAGAAG   
  
  
+ AGAGTGGCCT CATTTATTTT GTTTTGTTTG GTTTGTACAA GCTAATCAGC TAAGAATTCT TGTTTAACTG   
  
  
+ GTTTTGTGTT CTAGTTTTTG GTTTCTTGGA ACTTTGGTGG TTGTTGAGTG AGAAAGTTGA GCAAGTGAGA   
  
  
+ GAGAGAGAGA GGTTTTGTTA GGGGTAGAAA GGTTAGGAGC GAAAAGGAAG TGAGAGAGAG AAAGAGCAGG   
  
  
+ TAAGACTAGT GAAAAGGTTG TGATATGAGA GAGAGAGAGA GAGAGGGAAT AAGGTTAAAT TTCACATCAA   
  
  
+ CCTTATTCGA ATAAGAGAGA CTATTTAGTA GGATAACTTA ATTCGGTCAC AAAATATGCA ATGGTGTGTG   
  
  
+ TATTGAGAGT TGTTTTTACG TGAGGTCGGA TAATAATCAA ACTTAATGGA AATCAATTTT TTATGAAAGT   
  
  
+ GTACGATCGT CTTCTCTATC CACTGTGCGA GTTTTTCTCA CCAAATACAT ATGAAAATGG CATGATTCAC   
  
  
+ CATGCAAGAA AGGGATTTGG AGAATAATTG GTTATAACAG GTCATGTTTG CCTAGCAGCT TCCTTCTACT   
  
  
+ CCATTAATTT GGACTTGTGC ATTGCATGCC TTATATGGGT TGTATTTTGT GGATAAAGGA AAAGGACAAA   
  
  
+ GGGTGGGCTG GTTACGTAGG CTTTTTAGTT TTAATGAAAC ATTTCCGTTA CTGTTCATTT TTAACAAAAA   
  
  
+ TAACATTTTT ACTCTAAAAA AATCACTCCT CGTACTATTC ATTTACAACA CATTTTTGTC ATTTTTATTA   
  
  
+ AAACTCAAAG TTTTCAAGTT TTTTTCATTA ATTTTCCTTT TTATTTATTT ATTTTCAGAA TTAGGCAAAC   
  
  
+ AATTAGCCAC ATTACTTGAT CAATCAACTA TATGACATTG ATAGAATTCT ACTTTGCACT TTGCAACATC   
  
  
+ AAGTTTCCAC TTCAACCAAC CAGATGGGAT TTGCTAATTT TAGTCATTAA CTAAAGATTA TTCGATGTTG   
  
  
+ AGTATAAGGT TTGTCGCTTA AATGATTAAG AGCATTTATT AGTGCACCTA ATTGTTGCAC TACTTGAAGT   
  
  
+ CGTGTATTAG ACCAAGATCA TGCTCATATA ACTCATGGTA GATTAGGATG TGACTTAATT GTAGTTTGTT   
  
  
+ CATTCAAAAT ATCTATTTTC TTTTGGGTG  

- ACCACTCATA AAGAAAAAAG GCCACATCTT CGTCCAGCTT CGTGCTCTTT TGGTTTTCTT TGATCGTTTA   
  
  
- GTGGTGGGAT AATGCTAGGG AAGATCGCTA TAGTAAGAGC TCCTATGTTC TTTATATTCT TCCTCGTAAT   
  
  
- CGCTTGACGC TTGTATACCT GTGGCTTTCT CTCCAATATC GGGCTTTATC GATTTCCACT AGTTTCAAAA   
  
  
- CTACTCTTCT TTCTCTACAC CTGACGACAC ACTCCACCCT CTCAAAATCA AAAAAAAAAA GACCTTCCTT   
  
  
- TACTTCTAGC TACCCACCTC ACGAATATTG AACAAACTTT CCACATTCAA AAAACCCTAC CTTCCTCTTC   
  
  
- TCTCACCGGA GTAAATAAAA CAAAACAAAC CAAACATGTT CGATTAGTCG ATTCTTAAGA ACAAATTGAC   
  
  
- CAAAACACAA GATCAAAAAC CAAAGAACCT TGAAACCACC AACAACTCAC TCTTTCAACT CGTTCACTCT   
  
  
- CTCTCTCTCT CCAAAACAAT CCCCATCTTT CCAATCCTCG CTTTTCCTTC ACTCTCTCTC TTTCTCGTCC   
  
  
- ATTCTGATCA CTTTTCCAAC ACTATACTCT CTCTCTCTCT CTCTCCCTTA TTCCAATTTA AAGTGTAGTT   
  
  
- GGAATAAGCT TATTCTCTCT GATAAATCAT CCTATTGAAT TAAGCCAGTG TTTTATACGT TACCACACAC   
  
  
- ATAACTCTCA ACAAAAATGC ACTCCAGCCT ATTATTAGTT TGAATTACCT TTAGTTAAAA AATACTTTCA   
  
  
- CATGCTAGCA GAAGAGATAG GTGACACGCT CAAAAAGAGT GGTTTATGTA TACTTTTACC GTACTAAGTG   
  
  
- GTACGTTCTT TCCCTAAACC TCTTATTAAC CAATATTGTC CAGTACAAAC GGATCGTCGA AGGAAGATGA   
  
  
- GGTAATTAAA CCTGAACACG TAACGTACGG AATATACCCA ACATAAAACA CCTATTTCCT TTTCCTGTTT   
  
  
- CCCACCCGAC CAATGCATCC GAAAAATCAA AATTACTTTG TAAAGGCAAT GACAAGTAAA AATTGTTTTT   
  
  
- ATTGTAAAAA TGAGATTTTT TTAGTGAGGA GCATGATAAG TAAATGTTGT GTAAAAACAG TAAAAATAAT   
  
  
- TTTGAGTTTC AAAAGTTCAA AAAAAGTAAT TAAAAGGAAA AATAAATAAA TAAAAGTCTT AATCCGTTTG   
  
  
- TTAATCGGTG TAATGAACTA GTTAGTTGAT ATACTGTAAC TATCTTAAGA TGAAACGTGA AACGTTGTAG   
  
  
- TTCAAAGGTG AAGTTGGTTG GTCTACCCTA AACGATTAAA ATCAGTAATT GATTTCTAAT AAGCTACAAC   
  
  
- TCATATTCCA AACAGCGAAT TTACTAATTC TCGTAAATAA TCACGTGGAT TAACAACGTG ATGAACTTCA   
  
  
- GCACATAATC TGGTTCTAGT ACGAGTATAT TGAGTACCAT CTAATCCTAC ACTGAATTAA CATCAAACAA   
  
  
- GTAAGTTTTA TAGATAAAAG AAAACCCAC

+     Box 4

| Site Name | Organism | Position | Strand | Matrix score. | sequence | function |
| --- | --- | --- | --- | --- | --- | --- |
| Box 4 | Petroselinum crispum | 913 | - | 6 | ATTAAT | part of a conserved DNA module involved in light responsiveness |
| Box 4 | Petroselinum crispum | 1147 | - | 6 | ATTAAT | part of a conserved DNA module involved in light responsiveness |

>007212   
+ TGGTGAGTAT TTCTTTTTTC CGGTGTAGAA GCAGGTCGAA GCACGAGAAA ACCAAAAGAA ACTAGCAAAT   
  
  
+ CACCACCCTA TTACGATCCC TTCTAGCGAT ATCATTCTCG AGGATACAAG AAATATAAGA AGGAGCATTA   
  
  
+ GCGAACTGCG AACATATGGA CACCGAAAGA GAGGTTATAG CCCGAAATAG CTAAAGGTGA TCAAAGTTTT   
  
  
+ GATGAGAAGA AAGAGATGTG GACTGCTGTG TGAGGTGGGA GAGTTTTAGT TTTTTTTTTT CTGGAAGGAA   
  
  
+ ATGAAGATCG ATGGGTGGAG TGCTTATAAC TTGTTTGAAA GGTGTAAGTT TTTTGGGATG GAAGGAGAAG   
  
  
+ AGAGTGGCCT CATTTATTTT GTTTTGTTTG GTTTGTACAA GCTAATCAGC TAAGAATTCT TGTTTAACTG   
  
  
+ GTTTTGTGTT CTAGTTTTTG GTTTCTTGGA ACTTTGGTGG TTGTTGAGTG AGAAAGTTGA GCAAGTGAGA   
  
  
+ GAGAGAGAGA GGTTTTGTTA GGGGTAGAAA GGTTAGGAGC GAAAAGGAAG TGAGAGAGAG AAAGAGCAGG   
  
  
+ TAAGACTAGT GAAAAGGTTG TGATATGAGA GAGAGAGAGA GAGAGGGAAT AAGGTTAAAT TTCACATCAA   
  
  
+ CCTTATTCGA ATAAGAGAGA CTATTTAGTA GGATAACTTA ATTCGGTCAC AAAATATGCA ATGGTGTGTG   
  
  
+ TATTGAGAGT TGTTTTTACG TGAGGTCGGA TAATAATCAA ACTTAATGGA AATCAATTTT TTATGAAAGT   
  
  
+ GTACGATCGT CTTCTCTATC CACTGTGCGA GTTTTTCTCA CCAAATACAT ATGAAAATGG CATGATTCAC   
  
  
+ CATGCAAGAA AGGGATTTGG AGAATAATTG GTTATAACAG GTCATGTTTG CCTAGCAGCT TCCTTCTACT   
  
  
+ CCATTAATTT GGACTTGTGC ATTGCATGCC TTATATGGGT TGTATTTTGT GGATAAAGGA AAAGGACAAA   
  
  
+ GGGTGGGCTG GTTACGTAGG CTTTTTAGTT TTAATGAAAC ATTTCCGTTA CTGTTCATTT TTAACAAAAA   
  
  
+ TAACATTTTT ACTCTAAAAA AATCACTCCT CGTACTATTC ATTTACAACA CATTTTTGTC ATTTTTATTA   
  
  
+ AAACTCAAAG TTTTCAAGTT TTTTTCATTA ATTTTCCTTT TTATTTATTT ATTTTCAGAA TTAGGCAAAC   
  
  
+ AATTAGCCAC ATTACTTGAT CAATCAACTA TATGACATTG ATAGAATTCT ACTTTGCACT TTGCAACATC   
  
  
+ AAGTTTCCAC TTCAACCAAC CAGATGGGAT TTGCTAATTT TAGTCATTAA CTAAAGATTA TTCGATGTTG   
  
  
+ AGTATAAGGT TTGTCGCTTA AATGATTAAG AGCATTTATT AGTGCACCTA ATTGTTGCAC TACTTGAAGT   
  
  
+ CGTGTATTAG ACCAAGATCA TGCTCATATA ACTCATGGTA GATTAGGATG TGACTTAATT GTAGTTTGTT   
  
  
+ CATTCAAAAT ATCTATTTTC TTTTGGGTG  

- ACCACTCATA AAGAAAAAAG GCCACATCTT CGTCCAGCTT CGTGCTCTTT TGGTTTTCTT TGATCGTTTA   
  
  
- GTGGTGGGAT AATGCTAGGG AAGATCGCTA TAGTAAGAGC TCCTATGTTC TTTATATTCT TCCTCGTAAT   
  
  
- CGCTTGACGC TTGTATACCT GTGGCTTTCT CTCCAATATC GGGCTTTATC GATTTCCACT AGTTTCAAAA   
  
  
- CTACTCTTCT TTCTCTACAC CTGACGACAC ACTCCACCCT CTCAAAATCA AAAAAAAAAA GACCTTCCTT   
  
  
- TACTTCTAGC TACCCACCTC ACGAATATTG AACAAACTTT CCACATTCAA AAAACCCTAC CTTCCTCTTC   
  
  
- TCTCACCGGA GTAAATAAAA CAAAACAAAC CAAACATGTT CGATTAGTCG ATTCTTAAGA ACAAATTGAC   
  
  
- CAAAACACAA GATCAAAAAC CAAAGAACCT TGAAACCACC AACAACTCAC TCTTTCAACT CGTTCACTCT   
  
  
- CTCTCTCTCT CCAAAACAAT CCCCATCTTT CCAATCCTCG CTTTTCCTTC ACTCTCTCTC TTTCTCGTCC   
  
  
- ATTCTGATCA CTTTTCCAAC ACTATACTCT CTCTCTCTCT CTCTCCCTTA TTCCAATTTA AAGTGTAGTT   
  
  
- GGAATAAGCT TATTCTCTCT GATAAATCAT CCTATTGAAT TAAGCCAGTG TTTTATACGT TACCACACAC   
  
  
- ATAACTCTCA ACAAAAATGC ACTCCAGCCT ATTATTAGTT TGAATTACCT TTAGTTAAAA AATACTTTCA   
  
  
- CATGCTAGCA GAAGAGATAG GTGACACGCT CAAAAAGAGT GGTTTATGTA TACTTTTACC GTACTAAGTG   
  
  
- GTACGTTCTT TCCCTAAACC TCTTATTAAC CAATATTGTC CAGTACAAAC GGATCGTCGA AGGAAGATGA   
  
  
- GGTAATTAAA CCTGAACACG TAACGTACGG AATATACCCA ACATAAAACA CCTATTTCCT TTTCCTGTTT   
  
  
- CCCACCCGAC CAATGCATCC GAAAAATCAA AATTACTTTG TAAAGGCAAT GACAAGTAAA AATTGTTTTT   
  
  
- ATTGTAAAAA TGAGATTTTT TTAGTGAGGA GCATGATAAG TAAATGTTGT GTAAAAACAG TAAAAATAAT   
  
  
- TTTGAGTTTC AAAAGTTCAA AAAAAGTAAT TAAAAGGAAA AATAAATAAA TAAAAGTCTT AATCCGTTTG   
  
  
- TTAATCGGTG TAATGAACTA GTTAGTTGAT ATACTGTAAC TATCTTAAGA TGAAACGTGA AACGTTGTAG   
  
  
- TTCAAAGGTG AAGTTGGTTG GTCTACCCTA AACGATTAAA ATCAGTAATT GATTTCTAAT AAGCTACAAC   
  
  
- TCATATTCCA AACAGCGAAT TTACTAATTC TCGTAAATAA TCACGTGGAT TAACAACGTG ATGAACTTCA   
  
  
- GCACATAATC TGGTTCTAGT ACGAGTATAT TGAGTACCAT CTAATCCTAC ACTGAATTAA CATCAAACAA   
  
  
- GTAAGTTTTA TAGATAAAAG AAAACCCAC

+     CAAT-box

| Site Name | Organism | Position | Strand | Matrix score. | sequence | function |
| --- | --- | --- | --- | --- | --- | --- |
| CAAT-box | Nicotiana glutinosa | 1458 | - | 4 | CAAT |  |
| CAAT-box | Pisum sativum | 1289 | - | 5 | CAAAT | common cis-acting element in promoter and enhancer regions |
| CAAT-box | Nicotiana glutinosa | 1381 | - | 4 | CAAT |  |
| CAAT-box | Nicotiana glutinosa | 1227 | - | 4 | CAAT |  |
| CAAT-box | Nicotiana glutinosa | 702 | - | 4 | CAAT |  |
| CAAT-box | Nicotiana glutinosa | 1211 | + | 4 | CAAT |  |
| CAAT-box | Nicotiana glutinosa | 1190 | + | 4 | CAAT |  |
| CAAT-box | Nicotiana glutinosa | 931 | - | 4 | CAAT |  |
| CAAT-box | Nicotiana glutinosa | 754 | + | 4 | CAAT |  |
| CAAT-box | Pisum sativum | 66 | + | 5 | CAAAT | common cis-acting element in promoter and enhancer regions |
| CAAT-box | Nicotiana glutinosa | 689 | + | 4 | CAAT |  |
| CAAT-box | Arabidopsis thaliana | 867 | - | 5 | CCAAT | common cis-acting element in promoter and enhancer regions |
| CAAT-box | Pisum sativum | 917 | - | 5 | CAAAT | common cis-acting element in promoter and enhancer regions |
| CAAT-box | Pisum sativum | 855 | - | 5 | CAAAT | common cis-acting element in promoter and enhancer regions |
| CAAT-box | Pisum sativum | 812 | + | 5 | CAAAT | common cis-acting element in promoter and enhancer regions |

>007212   
+ TGGTGAGTAT TTCTTTTTTC CGGTGTAGAA GCAGGTCGAA GCACGAGAAA ACCAAAAGAA ACTAGCAAAT   
  
  
+ CACCACCCTA TTACGATCCC TTCTAGCGAT ATCATTCTCG AGGATACAAG AAATATAAGA AGGAGCATTA   
  
  
+ GCGAACTGCG AACATATGGA CACCGAAAGA GAGGTTATAG CCCGAAATAG CTAAAGGTGA TCAAAGTTTT   
  
  
+ GATGAGAAGA AAGAGATGTG GACTGCTGTG TGAGGTGGGA GAGTTTTAGT TTTTTTTTTT CTGGAAGGAA   
  
  
+ ATGAAGATCG ATGGGTGGAG TGCTTATAAC TTGTTTGAAA GGTGTAAGTT TTTTGGGATG GAAGGAGAAG   
  
  
+ AGAGTGGCCT CATTTATTTT GTTTTGTTTG GTTTGTACAA GCTAATCAGC TAAGAATTCT TGTTTAACTG   
  
  
+ GTTTTGTGTT CTAGTTTTTG GTTTCTTGGA ACTTTGGTGG TTGTTGAGTG AGAAAGTTGA GCAAGTGAGA   
  
  
+ GAGAGAGAGA GGTTTTGTTA GGGGTAGAAA GGTTAGGAGC GAAAAGGAAG TGAGAGAGAG AAAGAGCAGG   
  
  
+ TAAGACTAGT GAAAAGGTTG TGATATGAGA GAGAGAGAGA GAGAGGGAAT AAGGTTAAAT TTCACATCAA   
  
  
+ CCTTATTCGA ATAAGAGAGA CTATTTAGTA GGATAACTTA ATTCGGTCAC AAAATATGCA ATGGTGTGTG   
  
  
+ TATTGAGAGT TGTTTTTACG TGAGGTCGGA TAATAATCAA ACTTAATGGA AATCAATTTT TTATGAAAGT   
  
  
+ GTACGATCGT CTTCTCTATC CACTGTGCGA GTTTTTCTCA CCAAATACAT ATGAAAATGG CATGATTCAC   
  
  
+ CATGCAAGAA AGGGATTTGG AGAATAATTG GTTATAACAG GTCATGTTTG CCTAGCAGCT TCCTTCTACT   
  
  
+ CCATTAATTT GGACTTGTGC ATTGCATGCC TTATATGGGT TGTATTTTGT GGATAAAGGA AAAGGACAAA   
  
  
+ GGGTGGGCTG GTTACGTAGG CTTTTTAGTT TTAATGAAAC ATTTCCGTTA CTGTTCATTT TTAACAAAAA   
  
  
+ TAACATTTTT ACTCTAAAAA AATCACTCCT CGTACTATTC ATTTACAACA CATTTTTGTC ATTTTTATTA   
  
  
+ AAACTCAAAG TTTTCAAGTT TTTTTCATTA ATTTTCCTTT TTATTTATTT ATTTTCAGAA TTAGGCAAAC   
  
  
+ AATTAGCCAC ATTACTTGAT CAATCAACTA TATGACATTG ATAGAATTCT ACTTTGCACT TTGCAACATC   
  
  
+ AAGTTTCCAC TTCAACCAAC CAGATGGGAT TTGCTAATTT TAGTCATTAA CTAAAGATTA TTCGATGTTG   
  
  
+ AGTATAAGGT TTGTCGCTTA AATGATTAAG AGCATTTATT AGTGCACCTA ATTGTTGCAC TACTTGAAGT   
  
  
+ CGTGTATTAG ACCAAGATCA TGCTCATATA ACTCATGGTA GATTAGGATG TGACTTAATT GTAGTTTGTT   
  
  
+ CATTCAAAAT ATCTATTTTC TTTTGGGTG  

- ACCACTCATA AAGAAAAAAG GCCACATCTT CGTCCAGCTT CGTGCTCTTT TGGTTTTCTT TGATCGTTTA   
  
  
- GTGGTGGGAT AATGCTAGGG AAGATCGCTA TAGTAAGAGC TCCTATGTTC TTTATATTCT TCCTCGTAAT   
  
  
- CGCTTGACGC TTGTATACCT GTGGCTTTCT CTCCAATATC GGGCTTTATC GATTTCCACT AGTTTCAAAA   
  
  
- CTACTCTTCT TTCTCTACAC CTGACGACAC ACTCCACCCT CTCAAAATCA AAAAAAAAAA GACCTTCCTT   
  
  
- TACTTCTAGC TACCCACCTC ACGAATATTG AACAAACTTT CCACATTCAA AAAACCCTAC CTTCCTCTTC   
  
  
- TCTCACCGGA GTAAATAAAA CAAAACAAAC CAAACATGTT CGATTAGTCG ATTCTTAAGA ACAAATTGAC   
  
  
- CAAAACACAA GATCAAAAAC CAAAGAACCT TGAAACCACC AACAACTCAC TCTTTCAACT CGTTCACTCT   
  
  
- CTCTCTCTCT CCAAAACAAT CCCCATCTTT CCAATCCTCG CTTTTCCTTC ACTCTCTCTC TTTCTCGTCC   
  
  
- ATTCTGATCA CTTTTCCAAC ACTATACTCT CTCTCTCTCT CTCTCCCTTA TTCCAATTTA AAGTGTAGTT   
  
  
- GGAATAAGCT TATTCTCTCT GATAAATCAT CCTATTGAAT TAAGCCAGTG TTTTATACGT TACCACACAC   
  
  
- ATAACTCTCA ACAAAAATGC ACTCCAGCCT ATTATTAGTT TGAATTACCT TTAGTTAAAA AATACTTTCA   
  
  
- CATGCTAGCA GAAGAGATAG GTGACACGCT CAAAAAGAGT GGTTTATGTA TACTTTTACC GTACTAAGTG   
  
  
- GTACGTTCTT TCCCTAAACC TCTTATTAAC CAATATTGTC CAGTACAAAC GGATCGTCGA AGGAAGATGA   
  
  
- GGTAATTAAA CCTGAACACG TAACGTACGG AATATACCCA ACATAAAACA CCTATTTCCT TTTCCTGTTT   
  
  
- CCCACCCGAC CAATGCATCC GAAAAATCAA AATTACTTTG TAAAGGCAAT GACAAGTAAA AATTGTTTTT   
  
  
- ATTGTAAAAA TGAGATTTTT TTAGTGAGGA GCATGATAAG TAAATGTTGT GTAAAAACAG TAAAAATAAT   
  
  
- TTTGAGTTTC AAAAGTTCAA AAAAAGTAAT TAAAAGGAAA AATAAATAAA TAAAAGTCTT AATCCGTTTG   
  
  
- TTAATCGGTG TAATGAACTA GTTAGTTGAT ATACTGTAAC TATCTTAAGA TGAAACGTGA AACGTTGTAG   
  
  
- TTCAAAGGTG AAGTTGGTTG GTCTACCCTA AACGATTAAA ATCAGTAATT GATTTCTAAT AAGCTACAAC   
  
  
- TCATATTCCA AACAGCGAAT TTACTAATTC TCGTAAATAA TCACGTGGAT TAACAACGTG ATGAACTTCA   
  
  
- GCACATAATC TGGTTCTAGT ACGAGTATAT TGAGTACCAT CTAATCCTAC ACTGAATTAA CATCAAACAA   
  
  
- GTAAGTTTTA TAGATAAAAG AAAACCCAC

+     CAT-box

| Site Name | Organism | Position | Strand | Matrix score. | sequence | function |
| --- | --- | --- | --- | --- | --- | --- |
| CAT-box | Arabidopsis thaliana | 353 | - | 6 | GCCACT | cis-acting regulatory element related to meristem expression |

>007212   
+ TGGTGAGTAT TTCTTTTTTC CGGTGTAGAA GCAGGTCGAA GCACGAGAAA ACCAAAAGAA ACTAGCAAAT   
  
  
+ CACCACCCTA TTACGATCCC TTCTAGCGAT ATCATTCTCG AGGATACAAG AAATATAAGA AGGAGCATTA   
  
  
+ GCGAACTGCG AACATATGGA CACCGAAAGA GAGGTTATAG CCCGAAATAG CTAAAGGTGA TCAAAGTTTT   
  
  
+ GATGAGAAGA AAGAGATGTG GACTGCTGTG TGAGGTGGGA GAGTTTTAGT TTTTTTTTTT CTGGAAGGAA   
  
  
+ ATGAAGATCG ATGGGTGGAG TGCTTATAAC TTGTTTGAAA GGTGTAAGTT TTTTGGGATG GAAGGAGAAG   
  
  
+ AGAGTGGCCT CATTTATTTT GTTTTGTTTG GTTTGTACAA GCTAATCAGC TAAGAATTCT TGTTTAACTG   
  
  
+ GTTTTGTGTT CTAGTTTTTG GTTTCTTGGA ACTTTGGTGG TTGTTGAGTG AGAAAGTTGA GCAAGTGAGA   
  
  
+ GAGAGAGAGA GGTTTTGTTA GGGGTAGAAA GGTTAGGAGC GAAAAGGAAG TGAGAGAGAG AAAGAGCAGG   
  
  
+ TAAGACTAGT GAAAAGGTTG TGATATGAGA GAGAGAGAGA GAGAGGGAAT AAGGTTAAAT TTCACATCAA   
  
  
+ CCTTATTCGA ATAAGAGAGA CTATTTAGTA GGATAACTTA ATTCGGTCAC AAAATATGCA ATGGTGTGTG   
  
  
+ TATTGAGAGT TGTTTTTACG TGAGGTCGGA TAATAATCAA ACTTAATGGA AATCAATTTT TTATGAAAGT   
  
  
+ GTACGATCGT CTTCTCTATC CACTGTGCGA GTTTTTCTCA CCAAATACAT ATGAAAATGG CATGATTCAC   
  
  
+ CATGCAAGAA AGGGATTTGG AGAATAATTG GTTATAACAG GTCATGTTTG CCTAGCAGCT TCCTTCTACT   
  
  
+ CCATTAATTT GGACTTGTGC ATTGCATGCC TTATATGGGT TGTATTTTGT GGATAAAGGA AAAGGACAAA   
  
  
+ GGGTGGGCTG GTTACGTAGG CTTTTTAGTT TTAATGAAAC ATTTCCGTTA CTGTTCATTT TTAACAAAAA   
  
  
+ TAACATTTTT ACTCTAAAAA AATCACTCCT CGTACTATTC ATTTACAACA CATTTTTGTC ATTTTTATTA   
  
  
+ AAACTCAAAG TTTTCAAGTT TTTTTCATTA ATTTTCCTTT TTATTTATTT ATTTTCAGAA TTAGGCAAAC   
  
  
+ AATTAGCCAC ATTACTTGAT CAATCAACTA TATGACATTG ATAGAATTCT ACTTTGCACT TTGCAACATC   
  
  
+ AAGTTTCCAC TTCAACCAAC CAGATGGGAT TTGCTAATTT TAGTCATTAA CTAAAGATTA TTCGATGTTG   
  
  
+ AGTATAAGGT TTGTCGCTTA AATGATTAAG AGCATTTATT AGTGCACCTA ATTGTTGCAC TACTTGAAGT   
  
  
+ CGTGTATTAG ACCAAGATCA TGCTCATATA ACTCATGGTA GATTAGGATG TGACTTAATT GTAGTTTGTT   
  
  
+ CATTCAAAAT ATCTATTTTC TTTTGGGTG  

- ACCACTCATA AAGAAAAAAG GCCACATCTT CGTCCAGCTT CGTGCTCTTT TGGTTTTCTT TGATCGTTTA   
  
  
- GTGGTGGGAT AATGCTAGGG AAGATCGCTA TAGTAAGAGC TCCTATGTTC TTTATATTCT TCCTCGTAAT   
  
  
- CGCTTGACGC TTGTATACCT GTGGCTTTCT CTCCAATATC GGGCTTTATC GATTTCCACT AGTTTCAAAA   
  
  
- CTACTCTTCT TTCTCTACAC CTGACGACAC ACTCCACCCT CTCAAAATCA AAAAAAAAAA GACCTTCCTT   
  
  
- TACTTCTAGC TACCCACCTC ACGAATATTG AACAAACTTT CCACATTCAA AAAACCCTAC CTTCCTCTTC   
  
  
- TCTCACCGGA GTAAATAAAA CAAAACAAAC CAAACATGTT CGATTAGTCG ATTCTTAAGA ACAAATTGAC   
  
  
- CAAAACACAA GATCAAAAAC CAAAGAACCT TGAAACCACC AACAACTCAC TCTTTCAACT CGTTCACTCT   
  
  
- CTCTCTCTCT CCAAAACAAT CCCCATCTTT CCAATCCTCG CTTTTCCTTC ACTCTCTCTC TTTCTCGTCC   
  
  
- ATTCTGATCA CTTTTCCAAC ACTATACTCT CTCTCTCTCT CTCTCCCTTA TTCCAATTTA AAGTGTAGTT   
  
  
- GGAATAAGCT TATTCTCTCT GATAAATCAT CCTATTGAAT TAAGCCAGTG TTTTATACGT TACCACACAC   
  
  
- ATAACTCTCA ACAAAAATGC ACTCCAGCCT ATTATTAGTT TGAATTACCT TTAGTTAAAA AATACTTTCA   
  
  
- CATGCTAGCA GAAGAGATAG GTGACACGCT CAAAAAGAGT GGTTTATGTA TACTTTTACC GTACTAAGTG   
  
  
- GTACGTTCTT TCCCTAAACC TCTTATTAAC CAATATTGTC CAGTACAAAC GGATCGTCGA AGGAAGATGA   
  
  
- GGTAATTAAA CCTGAACACG TAACGTACGG AATATACCCA ACATAAAACA CCTATTTCCT TTTCCTGTTT   
  
  
- CCCACCCGAC CAATGCATCC GAAAAATCAA AATTACTTTG TAAAGGCAAT GACAAGTAAA AATTGTTTTT   
  
  
- ATTGTAAAAA TGAGATTTTT TTAGTGAGGA GCATGATAAG TAAATGTTGT GTAAAAACAG TAAAAATAAT   
  
  
- TTTGAGTTTC AAAAGTTCAA AAAAAGTAAT TAAAAGGAAA AATAAATAAA TAAAAGTCTT AATCCGTTTG   
  
  
- TTAATCGGTG TAATGAACTA GTTAGTTGAT ATACTGTAAC TATCTTAAGA TGAAACGTGA AACGTTGTAG   
  
  
- TTCAAAGGTG AAGTTGGTTG GTCTACCCTA AACGATTAAA ATCAGTAATT GATTTCTAAT AAGCTACAAC   
  
  
- TCATATTCCA AACAGCGAAT TTACTAATTC TCGTAAATAA TCACGTGGAT TAACAACGTG ATGAACTTCA   
  
  
- GCACATAATC TGGTTCTAGT ACGAGTATAT TGAGTACCAT CTAATCCTAC ACTGAATTAA CATCAAACAA   
  
  
- GTAAGTTTTA TAGATAAAAG AAAACCCAC

+     G-box

| Site Name | Organism | Position | Strand | Matrix score. | sequence | function |
| --- | --- | --- | --- | --- | --- | --- |
| G-box | Zea mays | 1399 | - | 6 | CACGAC | cis-acting regulatory element involved in light responsiveness |
| G-box | Arabidopsis thaliana | 717 | + | 6 | TACGTG | cis-acting regulatory element involved in light responsiveness |

>007212   
+ TGGTGAGTAT TTCTTTTTTC CGGTGTAGAA GCAGGTCGAA GCACGAGAAA ACCAAAAGAA ACTAGCAAAT   
  
  
+ CACCACCCTA TTACGATCCC TTCTAGCGAT ATCATTCTCG AGGATACAAG AAATATAAGA AGGAGCATTA   
  
  
+ GCGAACTGCG AACATATGGA CACCGAAAGA GAGGTTATAG CCCGAAATAG CTAAAGGTGA TCAAAGTTTT   
  
  
+ GATGAGAAGA AAGAGATGTG GACTGCTGTG TGAGGTGGGA GAGTTTTAGT TTTTTTTTTT CTGGAAGGAA   
  
  
+ ATGAAGATCG ATGGGTGGAG TGCTTATAAC TTGTTTGAAA GGTGTAAGTT TTTTGGGATG GAAGGAGAAG   
  
  
+ AGAGTGGCCT CATTTATTTT GTTTTGTTTG GTTTGTACAA GCTAATCAGC TAAGAATTCT TGTTTAACTG   
  
  
+ GTTTTGTGTT CTAGTTTTTG GTTTCTTGGA ACTTTGGTGG TTGTTGAGTG AGAAAGTTGA GCAAGTGAGA   
  
  
+ GAGAGAGAGA GGTTTTGTTA GGGGTAGAAA GGTTAGGAGC GAAAAGGAAG TGAGAGAGAG AAAGAGCAGG   
  
  
+ TAAGACTAGT GAAAAGGTTG TGATATGAGA GAGAGAGAGA GAGAGGGAAT AAGGTTAAAT TTCACATCAA   
  
  
+ CCTTATTCGA ATAAGAGAGA CTATTTAGTA GGATAACTTA ATTCGGTCAC AAAATATGCA ATGGTGTGTG   
  
  
+ TATTGAGAGT TGTTTTTACG TGAGGTCGGA TAATAATCAA ACTTAATGGA AATCAATTTT TTATGAAAGT   
  
  
+ GTACGATCGT CTTCTCTATC CACTGTGCGA GTTTTTCTCA CCAAATACAT ATGAAAATGG CATGATTCAC   
  
  
+ CATGCAAGAA AGGGATTTGG AGAATAATTG GTTATAACAG GTCATGTTTG CCTAGCAGCT TCCTTCTACT   
  
  
+ CCATTAATTT GGACTTGTGC ATTGCATGCC TTATATGGGT TGTATTTTGT GGATAAAGGA AAAGGACAAA   
  
  
+ GGGTGGGCTG GTTACGTAGG CTTTTTAGTT TTAATGAAAC ATTTCCGTTA CTGTTCATTT TTAACAAAAA   
  
  
+ TAACATTTTT ACTCTAAAAA AATCACTCCT CGTACTATTC ATTTACAACA CATTTTTGTC ATTTTTATTA   
  
  
+ AAACTCAAAG TTTTCAAGTT TTTTTCATTA ATTTTCCTTT TTATTTATTT ATTTTCAGAA TTAGGCAAAC   
  
  
+ AATTAGCCAC ATTACTTGAT CAATCAACTA TATGACATTG ATAGAATTCT ACTTTGCACT TTGCAACATC   
  
  
+ AAGTTTCCAC TTCAACCAAC CAGATGGGAT TTGCTAATTT TAGTCATTAA CTAAAGATTA TTCGATGTTG   
  
  
+ AGTATAAGGT TTGTCGCTTA AATGATTAAG AGCATTTATT AGTGCACCTA ATTGTTGCAC TACTTGAAGT   
  
  
+ CGTGTATTAG ACCAAGATCA TGCTCATATA ACTCATGGTA GATTAGGATG TGACTTAATT GTAGTTTGTT   
  
  
+ CATTCAAAAT ATCTATTTTC TTTTGGGTG  

- ACCACTCATA AAGAAAAAAG GCCACATCTT CGTCCAGCTT CGTGCTCTTT TGGTTTTCTT TGATCGTTTA   
  
  
- GTGGTGGGAT AATGCTAGGG AAGATCGCTA TAGTAAGAGC TCCTATGTTC TTTATATTCT TCCTCGTAAT   
  
  
- CGCTTGACGC TTGTATACCT GTGGCTTTCT CTCCAATATC GGGCTTTATC GATTTCCACT AGTTTCAAAA   
  
  
- CTACTCTTCT TTCTCTACAC CTGACGACAC ACTCCACCCT CTCAAAATCA AAAAAAAAAA GACCTTCCTT   
  
  
- TACTTCTAGC TACCCACCTC ACGAATATTG AACAAACTTT CCACATTCAA AAAACCCTAC CTTCCTCTTC   
  
  
- TCTCACCGGA GTAAATAAAA CAAAACAAAC CAAACATGTT CGATTAGTCG ATTCTTAAGA ACAAATTGAC   
  
  
- CAAAACACAA GATCAAAAAC CAAAGAACCT TGAAACCACC AACAACTCAC TCTTTCAACT CGTTCACTCT   
  
  
- CTCTCTCTCT CCAAAACAAT CCCCATCTTT CCAATCCTCG CTTTTCCTTC ACTCTCTCTC TTTCTCGTCC   
  
  
- ATTCTGATCA CTTTTCCAAC ACTATACTCT CTCTCTCTCT CTCTCCCTTA TTCCAATTTA AAGTGTAGTT   
  
  
- GGAATAAGCT TATTCTCTCT GATAAATCAT CCTATTGAAT TAAGCCAGTG TTTTATACGT TACCACACAC   
  
  
- ATAACTCTCA ACAAAAATGC ACTCCAGCCT ATTATTAGTT TGAATTACCT TTAGTTAAAA AATACTTTCA   
  
  
- CATGCTAGCA GAAGAGATAG GTGACACGCT CAAAAAGAGT GGTTTATGTA TACTTTTACC GTACTAAGTG   
  
  
- GTACGTTCTT TCCCTAAACC TCTTATTAAC CAATATTGTC CAGTACAAAC GGATCGTCGA AGGAAGATGA   
  
  
- GGTAATTAAA CCTGAACACG TAACGTACGG AATATACCCA ACATAAAACA CCTATTTCCT TTTCCTGTTT   
  
  
- CCCACCCGAC CAATGCATCC GAAAAATCAA AATTACTTTG TAAAGGCAAT GACAAGTAAA AATTGTTTTT   
  
  
- ATTGTAAAAA TGAGATTTTT TTAGTGAGGA GCATGATAAG TAAATGTTGT GTAAAAACAG TAAAAATAAT   
  
  
- TTTGAGTTTC AAAAGTTCAA AAAAAGTAAT TAAAAGGAAA AATAAATAAA TAAAAGTCTT AATCCGTTTG   
  
  
- TTAATCGGTG TAATGAACTA GTTAGTTGAT ATACTGTAAC TATCTTAAGA TGAAACGTGA AACGTTGTAG   
  
  
- TTCAAAGGTG AAGTTGGTTG GTCTACCCTA AACGATTAAA ATCAGTAATT GATTTCTAAT AAGCTACAAC   
  
  
- TCATATTCCA AACAGCGAAT TTACTAATTC TCGTAAATAA TCACGTGGAT TAACAACGTG ATGAACTTCA   
  
  
- GCACATAATC TGGTTCTAGT ACGAGTATAT TGAGTACCAT CTAATCCTAC ACTGAATTAA CATCAAACAA   
  
  
- GTAAGTTTTA TAGATAAAAG AAAACCCAC

+     GATA-motif

| Site Name | Organism | Position | Strand | Matrix score. | sequence | function |
| --- | --- | --- | --- | --- | --- | --- |
| GATA-motif | Solanum tuberosum | 966 | + | 9 | AAGGATAAGG | part of a light responsive element |

>007212   
+ TGGTGAGTAT TTCTTTTTTC CGGTGTAGAA GCAGGTCGAA GCACGAGAAA ACCAAAAGAA ACTAGCAAAT   
  
  
+ CACCACCCTA TTACGATCCC TTCTAGCGAT ATCATTCTCG AGGATACAAG AAATATAAGA AGGAGCATTA   
  
  
+ GCGAACTGCG AACATATGGA CACCGAAAGA GAGGTTATAG CCCGAAATAG CTAAAGGTGA TCAAAGTTTT   
  
  
+ GATGAGAAGA AAGAGATGTG GACTGCTGTG TGAGGTGGGA GAGTTTTAGT TTTTTTTTTT CTGGAAGGAA   
  
  
+ ATGAAGATCG ATGGGTGGAG TGCTTATAAC TTGTTTGAAA GGTGTAAGTT TTTTGGGATG GAAGGAGAAG   
  
  
+ AGAGTGGCCT CATTTATTTT GTTTTGTTTG GTTTGTACAA GCTAATCAGC TAAGAATTCT TGTTTAACTG   
  
  
+ GTTTTGTGTT CTAGTTTTTG GTTTCTTGGA ACTTTGGTGG TTGTTGAGTG AGAAAGTTGA GCAAGTGAGA   
  
  
+ GAGAGAGAGA GGTTTTGTTA GGGGTAGAAA GGTTAGGAGC GAAAAGGAAG TGAGAGAGAG AAAGAGCAGG   
  
  
+ TAAGACTAGT GAAAAGGTTG TGATATGAGA GAGAGAGAGA GAGAGGGAAT AAGGTTAAAT TTCACATCAA   
  
  
+ CCTTATTCGA ATAAGAGAGA CTATTTAGTA GGATAACTTA ATTCGGTCAC AAAATATGCA ATGGTGTGTG   
  
  
+ TATTGAGAGT TGTTTTTACG TGAGGTCGGA TAATAATCAA ACTTAATGGA AATCAATTTT TTATGAAAGT   
  
  
+ GTACGATCGT CTTCTCTATC CACTGTGCGA GTTTTTCTCA CCAAATACAT ATGAAAATGG CATGATTCAC   
  
  
+ CATGCAAGAA AGGGATTTGG AGAATAATTG GTTATAACAG GTCATGTTTG CCTAGCAGCT TCCTTCTACT   
  
  
+ CCATTAATTT GGACTTGTGC ATTGCATGCC TTATATGGGT TGTATTTTGT GGATAAAGGA AAAGGACAAA   
  
  
+ GGGTGGGCTG GTTACGTAGG CTTTTTAGTT TTAATGAAAC ATTTCCGTTA CTGTTCATTT TTAACAAAAA   
  
  
+ TAACATTTTT ACTCTAAAAA AATCACTCCT CGTACTATTC ATTTACAACA CATTTTTGTC ATTTTTATTA   
  
  
+ AAACTCAAAG TTTTCAAGTT TTTTTCATTA ATTTTCCTTT TTATTTATTT ATTTTCAGAA TTAGGCAAAC   
  
  
+ AATTAGCCAC ATTACTTGAT CAATCAACTA TATGACATTG ATAGAATTCT ACTTTGCACT TTGCAACATC   
  
  
+ AAGTTTCCAC TTCAACCAAC CAGATGGGAT TTGCTAATTT TAGTCATTAA CTAAAGATTA TTCGATGTTG   
  
  
+ AGTATAAGGT TTGTCGCTTA AATGATTAAG AGCATTTATT AGTGCACCTA ATTGTTGCAC TACTTGAAGT   
  
  
+ CGTGTATTAG ACCAAGATCA TGCTCATATA ACTCATGGTA GATTAGGATG TGACTTAATT GTAGTTTGTT   
  
  
+ CATTCAAAAT ATCTATTTTC TTTTGGGTG  

- ACCACTCATA AAGAAAAAAG GCCACATCTT CGTCCAGCTT CGTGCTCTTT TGGTTTTCTT TGATCGTTTA   
  
  
- GTGGTGGGAT AATGCTAGGG AAGATCGCTA TAGTAAGAGC TCCTATGTTC TTTATATTCT TCCTCGTAAT   
  
  
- CGCTTGACGC TTGTATACCT GTGGCTTTCT CTCCAATATC GGGCTTTATC GATTTCCACT AGTTTCAAAA   
  
  
- CTACTCTTCT TTCTCTACAC CTGACGACAC ACTCCACCCT CTCAAAATCA AAAAAAAAAA GACCTTCCTT   
  
  
- TACTTCTAGC TACCCACCTC ACGAATATTG AACAAACTTT CCACATTCAA AAAACCCTAC CTTCCTCTTC   
  
  
- TCTCACCGGA GTAAATAAAA CAAAACAAAC CAAACATGTT CGATTAGTCG ATTCTTAAGA ACAAATTGAC   
  
  
- CAAAACACAA GATCAAAAAC CAAAGAACCT TGAAACCACC AACAACTCAC TCTTTCAACT CGTTCACTCT   
  
  
- CTCTCTCTCT CCAAAACAAT CCCCATCTTT CCAATCCTCG CTTTTCCTTC ACTCTCTCTC TTTCTCGTCC   
  
  
- ATTCTGATCA CTTTTCCAAC ACTATACTCT CTCTCTCTCT CTCTCCCTTA TTCCAATTTA AAGTGTAGTT   
  
  
- GGAATAAGCT TATTCTCTCT GATAAATCAT CCTATTGAAT TAAGCCAGTG TTTTATACGT TACCACACAC   
  
  
- ATAACTCTCA ACAAAAATGC ACTCCAGCCT ATTATTAGTT TGAATTACCT TTAGTTAAAA AATACTTTCA   
  
  
- CATGCTAGCA GAAGAGATAG GTGACACGCT CAAAAAGAGT GGTTTATGTA TACTTTTACC GTACTAAGTG   
  
  
- GTACGTTCTT TCCCTAAACC TCTTATTAAC CAATATTGTC CAGTACAAAC GGATCGTCGA AGGAAGATGA   
  
  
- GGTAATTAAA CCTGAACACG TAACGTACGG AATATACCCA ACATAAAACA CCTATTTCCT TTTCCTGTTT   
  
  
- CCCACCCGAC CAATGCATCC GAAAAATCAA AATTACTTTG TAAAGGCAAT GACAAGTAAA AATTGTTTTT   
  
  
- ATTGTAAAAA TGAGATTTTT TTAGTGAGGA GCATGATAAG TAAATGTTGT GTAAAAACAG TAAAAATAAT   
  
  
- TTTGAGTTTC AAAAGTTCAA AAAAAGTAAT TAAAAGGAAA AATAAATAAA TAAAAGTCTT AATCCGTTTG   
  
  
- TTAATCGGTG TAATGAACTA GTTAGTTGAT ATACTGTAAC TATCTTAAGA TGAAACGTGA AACGTTGTAG   
  
  
- TTCAAAGGTG AAGTTGGTTG GTCTACCCTA AACGATTAAA ATCAGTAATT GATTTCTAAT AAGCTACAAC   
  
  
- TCATATTCCA AACAGCGAAT TTACTAATTC TCGTAAATAA TCACGTGGAT TAACAACGTG ATGAACTTCA   
  
  
- GCACATAATC TGGTTCTAGT ACGAGTATAT TGAGTACCAT CTAATCCTAC ACTGAATTAA CATCAAACAA   
  
  
- GTAAGTTTTA TAGATAAAAG AAAACCCAC

+     GT1-motif

| Site Name | Organism | Position | Strand | Matrix score. | sequence | function |
| --- | --- | --- | --- | --- | --- | --- |
| GT1-motif | Arabidopsis thaliana | 613 | + | 6 | GGTTAA | light responsive element |

>007212   
+ TGGTGAGTAT TTCTTTTTTC CGGTGTAGAA GCAGGTCGAA GCACGAGAAA ACCAAAAGAA ACTAGCAAAT   
  
  
+ CACCACCCTA TTACGATCCC TTCTAGCGAT ATCATTCTCG AGGATACAAG AAATATAAGA AGGAGCATTA   
  
  
+ GCGAACTGCG AACATATGGA CACCGAAAGA GAGGTTATAG CCCGAAATAG CTAAAGGTGA TCAAAGTTTT   
  
  
+ GATGAGAAGA AAGAGATGTG GACTGCTGTG TGAGGTGGGA GAGTTTTAGT TTTTTTTTTT CTGGAAGGAA   
  
  
+ ATGAAGATCG ATGGGTGGAG TGCTTATAAC TTGTTTGAAA GGTGTAAGTT TTTTGGGATG GAAGGAGAAG   
  
  
+ AGAGTGGCCT CATTTATTTT GTTTTGTTTG GTTTGTACAA GCTAATCAGC TAAGAATTCT TGTTTAACTG   
  
  
+ GTTTTGTGTT CTAGTTTTTG GTTTCTTGGA ACTTTGGTGG TTGTTGAGTG AGAAAGTTGA GCAAGTGAGA   
  
  
+ GAGAGAGAGA GGTTTTGTTA GGGGTAGAAA GGTTAGGAGC GAAAAGGAAG TGAGAGAGAG AAAGAGCAGG   
  
  
+ TAAGACTAGT GAAAAGGTTG TGATATGAGA GAGAGAGAGA GAGAGGGAAT AAGGTTAAAT TTCACATCAA   
  
  
+ CCTTATTCGA ATAAGAGAGA CTATTTAGTA GGATAACTTA ATTCGGTCAC AAAATATGCA ATGGTGTGTG   
  
  
+ TATTGAGAGT TGTTTTTACG TGAGGTCGGA TAATAATCAA ACTTAATGGA AATCAATTTT TTATGAAAGT   
  
  
+ GTACGATCGT CTTCTCTATC CACTGTGCGA GTTTTTCTCA CCAAATACAT ATGAAAATGG CATGATTCAC   
  
  
+ CATGCAAGAA AGGGATTTGG AGAATAATTG GTTATAACAG GTCATGTTTG CCTAGCAGCT TCCTTCTACT   
  
  
+ CCATTAATTT GGACTTGTGC ATTGCATGCC TTATATGGGT TGTATTTTGT GGATAAAGGA AAAGGACAAA   
  
  
+ GGGTGGGCTG GTTACGTAGG CTTTTTAGTT TTAATGAAAC ATTTCCGTTA CTGTTCATTT TTAACAAAAA   
  
  
+ TAACATTTTT ACTCTAAAAA AATCACTCCT CGTACTATTC ATTTACAACA CATTTTTGTC ATTTTTATTA   
  
  
+ AAACTCAAAG TTTTCAAGTT TTTTTCATTA ATTTTCCTTT TTATTTATTT ATTTTCAGAA TTAGGCAAAC   
  
  
+ AATTAGCCAC ATTACTTGAT CAATCAACTA TATGACATTG ATAGAATTCT ACTTTGCACT TTGCAACATC   
  
  
+ AAGTTTCCAC TTCAACCAAC CAGATGGGAT TTGCTAATTT TAGTCATTAA CTAAAGATTA TTCGATGTTG   
  
  
+ AGTATAAGGT TTGTCGCTTA AATGATTAAG AGCATTTATT AGTGCACCTA ATTGTTGCAC TACTTGAAGT   
  
  
+ CGTGTATTAG ACCAAGATCA TGCTCATATA ACTCATGGTA GATTAGGATG TGACTTAATT GTAGTTTGTT   
  
  
+ CATTCAAAAT ATCTATTTTC TTTTGGGTG  

- ACCACTCATA AAGAAAAAAG GCCACATCTT CGTCCAGCTT CGTGCTCTTT TGGTTTTCTT TGATCGTTTA   
  
  
- GTGGTGGGAT AATGCTAGGG AAGATCGCTA TAGTAAGAGC TCCTATGTTC TTTATATTCT TCCTCGTAAT   
  
  
- CGCTTGACGC TTGTATACCT GTGGCTTTCT CTCCAATATC GGGCTTTATC GATTTCCACT AGTTTCAAAA   
  
  
- CTACTCTTCT TTCTCTACAC CTGACGACAC ACTCCACCCT CTCAAAATCA AAAAAAAAAA GACCTTCCTT   
  
  
- TACTTCTAGC TACCCACCTC ACGAATATTG AACAAACTTT CCACATTCAA AAAACCCTAC CTTCCTCTTC   
  
  
- TCTCACCGGA GTAAATAAAA CAAAACAAAC CAAACATGTT CGATTAGTCG ATTCTTAAGA ACAAATTGAC   
  
  
- CAAAACACAA GATCAAAAAC CAAAGAACCT TGAAACCACC AACAACTCAC TCTTTCAACT CGTTCACTCT   
  
  
- CTCTCTCTCT CCAAAACAAT CCCCATCTTT CCAATCCTCG CTTTTCCTTC ACTCTCTCTC TTTCTCGTCC   
  
  
- ATTCTGATCA CTTTTCCAAC ACTATACTCT CTCTCTCTCT CTCTCCCTTA TTCCAATTTA AAGTGTAGTT   
  
  
- GGAATAAGCT TATTCTCTCT GATAAATCAT CCTATTGAAT TAAGCCAGTG TTTTATACGT TACCACACAC   
  
  
- ATAACTCTCA ACAAAAATGC ACTCCAGCCT ATTATTAGTT TGAATTACCT TTAGTTAAAA AATACTTTCA   
  
  
- CATGCTAGCA GAAGAGATAG GTGACACGCT CAAAAAGAGT GGTTTATGTA TACTTTTACC GTACTAAGTG   
  
  
- GTACGTTCTT TCCCTAAACC TCTTATTAAC CAATATTGTC CAGTACAAAC GGATCGTCGA AGGAAGATGA   
  
  
- GGTAATTAAA CCTGAACACG TAACGTACGG AATATACCCA ACATAAAACA CCTATTTCCT TTTCCTGTTT   
  
  
- CCCACCCGAC CAATGCATCC GAAAAATCAA AATTACTTTG TAAAGGCAAT GACAAGTAAA AATTGTTTTT   
  
  
- ATTGTAAAAA TGAGATTTTT TTAGTGAGGA GCATGATAAG TAAATGTTGT GTAAAAACAG TAAAAATAAT   
  
  
- TTTGAGTTTC AAAAGTTCAA AAAAAGTAAT TAAAAGGAAA AATAAATAAA TAAAAGTCTT AATCCGTTTG   
  
  
- TTAATCGGTG TAATGAACTA GTTAGTTGAT ATACTGTAAC TATCTTAAGA TGAAACGTGA AACGTTGTAG   
  
  
- TTCAAAGGTG AAGTTGGTTG GTCTACCCTA AACGATTAAA ATCAGTAATT GATTTCTAAT AAGCTACAAC   
  
  
- TCATATTCCA AACAGCGAAT TTACTAATTC TCGTAAATAA TCACGTGGAT TAACAACGTG ATGAACTTCA   
  
  
- GCACATAATC TGGTTCTAGT ACGAGTATAT TGAGTACCAT CTAATCCTAC ACTGAATTAA CATCAAACAA   
  
  
- GTAAGTTTTA TAGATAAAAG AAAACCCAC

+     LTR

| Site Name | Organism | Position | Strand | Matrix score. | sequence | function |
| --- | --- | --- | --- | --- | --- | --- |
| LTR | Hordeum vulgare | 182 | + | 6 | CCGAAA | cis-acting element involved in low-temperature responsiveness |
| LTR | Hordeum vulgare | 163 | + | 6 | CCGAAA | cis-acting element involved in low-temperature responsiveness |

>007212   
+ TGGTGAGTAT TTCTTTTTTC CGGTGTAGAA GCAGGTCGAA GCACGAGAAA ACCAAAAGAA ACTAGCAAAT   
  
  
+ CACCACCCTA TTACGATCCC TTCTAGCGAT ATCATTCTCG AGGATACAAG AAATATAAGA AGGAGCATTA   
  
  
+ GCGAACTGCG AACATATGGA CACCGAAAGA GAGGTTATAG CCCGAAATAG CTAAAGGTGA TCAAAGTTTT   
  
  
+ GATGAGAAGA AAGAGATGTG GACTGCTGTG TGAGGTGGGA GAGTTTTAGT TTTTTTTTTT CTGGAAGGAA   
  
  
+ ATGAAGATCG ATGGGTGGAG TGCTTATAAC TTGTTTGAAA GGTGTAAGTT TTTTGGGATG GAAGGAGAAG   
  
  
+ AGAGTGGCCT CATTTATTTT GTTTTGTTTG GTTTGTACAA GCTAATCAGC TAAGAATTCT TGTTTAACTG   
  
  
+ GTTTTGTGTT CTAGTTTTTG GTTTCTTGGA ACTTTGGTGG TTGTTGAGTG AGAAAGTTGA GCAAGTGAGA   
  
  
+ GAGAGAGAGA GGTTTTGTTA GGGGTAGAAA GGTTAGGAGC GAAAAGGAAG TGAGAGAGAG AAAGAGCAGG   
  
  
+ TAAGACTAGT GAAAAGGTTG TGATATGAGA GAGAGAGAGA GAGAGGGAAT AAGGTTAAAT TTCACATCAA   
  
  
+ CCTTATTCGA ATAAGAGAGA CTATTTAGTA GGATAACTTA ATTCGGTCAC AAAATATGCA ATGGTGTGTG   
  
  
+ TATTGAGAGT TGTTTTTACG TGAGGTCGGA TAATAATCAA ACTTAATGGA AATCAATTTT TTATGAAAGT   
  
  
+ GTACGATCGT CTTCTCTATC CACTGTGCGA GTTTTTCTCA CCAAATACAT ATGAAAATGG CATGATTCAC   
  
  
+ CATGCAAGAA AGGGATTTGG AGAATAATTG GTTATAACAG GTCATGTTTG CCTAGCAGCT TCCTTCTACT   
  
  
+ CCATTAATTT GGACTTGTGC ATTGCATGCC TTATATGGGT TGTATTTTGT GGATAAAGGA AAAGGACAAA   
  
  
+ GGGTGGGCTG GTTACGTAGG CTTTTTAGTT TTAATGAAAC ATTTCCGTTA CTGTTCATTT TTAACAAAAA   
  
  
+ TAACATTTTT ACTCTAAAAA AATCACTCCT CGTACTATTC ATTTACAACA CATTTTTGTC ATTTTTATTA   
  
  
+ AAACTCAAAG TTTTCAAGTT TTTTTCATTA ATTTTCCTTT TTATTTATTT ATTTTCAGAA TTAGGCAAAC   
  
  
+ AATTAGCCAC ATTACTTGAT CAATCAACTA TATGACATTG ATAGAATTCT ACTTTGCACT TTGCAACATC   
  
  
+ AAGTTTCCAC TTCAACCAAC CAGATGGGAT TTGCTAATTT TAGTCATTAA CTAAAGATTA TTCGATGTTG   
  
  
+ AGTATAAGGT TTGTCGCTTA AATGATTAAG AGCATTTATT AGTGCACCTA ATTGTTGCAC TACTTGAAGT   
  
  
+ CGTGTATTAG ACCAAGATCA TGCTCATATA ACTCATGGTA GATTAGGATG TGACTTAATT GTAGTTTGTT   
  
  
+ CATTCAAAAT ATCTATTTTC TTTTGGGTG  

- ACCACTCATA AAGAAAAAAG GCCACATCTT CGTCCAGCTT CGTGCTCTTT TGGTTTTCTT TGATCGTTTA   
  
  
- GTGGTGGGAT AATGCTAGGG AAGATCGCTA TAGTAAGAGC TCCTATGTTC TTTATATTCT TCCTCGTAAT   
  
  
- CGCTTGACGC TTGTATACCT GTGGCTTTCT CTCCAATATC GGGCTTTATC GATTTCCACT AGTTTCAAAA   
  
  
- CTACTCTTCT TTCTCTACAC CTGACGACAC ACTCCACCCT CTCAAAATCA AAAAAAAAAA GACCTTCCTT   
  
  
- TACTTCTAGC TACCCACCTC ACGAATATTG AACAAACTTT CCACATTCAA AAAACCCTAC CTTCCTCTTC   
  
  
- TCTCACCGGA GTAAATAAAA CAAAACAAAC CAAACATGTT CGATTAGTCG ATTCTTAAGA ACAAATTGAC   
  
  
- CAAAACACAA GATCAAAAAC CAAAGAACCT TGAAACCACC AACAACTCAC TCTTTCAACT CGTTCACTCT   
  
  
- CTCTCTCTCT CCAAAACAAT CCCCATCTTT CCAATCCTCG CTTTTCCTTC ACTCTCTCTC TTTCTCGTCC   
  
  
- ATTCTGATCA CTTTTCCAAC ACTATACTCT CTCTCTCTCT CTCTCCCTTA TTCCAATTTA AAGTGTAGTT   
  
  
- GGAATAAGCT TATTCTCTCT GATAAATCAT CCTATTGAAT TAAGCCAGTG TTTTATACGT TACCACACAC   
  
  
- ATAACTCTCA ACAAAAATGC ACTCCAGCCT ATTATTAGTT TGAATTACCT TTAGTTAAAA AATACTTTCA   
  
  
- CATGCTAGCA GAAGAGATAG GTGACACGCT CAAAAAGAGT GGTTTATGTA TACTTTTACC GTACTAAGTG   
  
  
- GTACGTTCTT TCCCTAAACC TCTTATTAAC CAATATTGTC CAGTACAAAC GGATCGTCGA AGGAAGATGA   
  
  
- GGTAATTAAA CCTGAACACG TAACGTACGG AATATACCCA ACATAAAACA CCTATTTCCT TTTCCTGTTT   
  
  
- CCCACCCGAC CAATGCATCC GAAAAATCAA AATTACTTTG TAAAGGCAAT GACAAGTAAA AATTGTTTTT   
  
  
- ATTGTAAAAA TGAGATTTTT TTAGTGAGGA GCATGATAAG TAAATGTTGT GTAAAAACAG TAAAAATAAT   
  
  
- TTTGAGTTTC AAAAGTTCAA AAAAAGTAAT TAAAAGGAAA AATAAATAAA TAAAAGTCTT AATCCGTTTG   
  
  
- TTAATCGGTG TAATGAACTA GTTAGTTGAT ATACTGTAAC TATCTTAAGA TGAAACGTGA AACGTTGTAG   
  
  
- TTCAAAGGTG AAGTTGGTTG GTCTACCCTA AACGATTAAA ATCAGTAATT GATTTCTAAT AAGCTACAAC   
  
  
- TCATATTCCA AACAGCGAAT TTACTAATTC TCGTAAATAA TCACGTGGAT TAACAACGTG ATGAACTTCA   
  
  
- GCACATAATC TGGTTCTAGT ACGAGTATAT TGAGTACCAT CTAATCCTAC ACTGAATTAA CATCAAACAA   
  
  
- GTAAGTTTTA TAGATAAAAG AAAACCCAC

+     MYB

| Site Name | Organism | Position | Strand | Matrix score. | sequence | function |
| --- | --- | --- | --- | --- | --- | --- |
| MYB | Arabidopsis thaliana | 1277 | + | 6 | CAACCA |  |
| MYB | Arabidopsis thaliana | 1273 | + | 6 | CAACCA |  |
| MYB | Arabidopsis thaliana | 989 | - | 6 | TAACCA |  |
| MYB | Arabidopsis thaliana | 869 | - | 6 | TAACCA |  |
| MYB | Arabidopsis thaliana | 458 | - | 6 | CAACCA |  |

>007212   
+ TGGTGAGTAT TTCTTTTTTC CGGTGTAGAA GCAGGTCGAA GCACGAGAAA ACCAAAAGAA ACTAGCAAAT   
  
  
+ CACCACCCTA TTACGATCCC TTCTAGCGAT ATCATTCTCG AGGATACAAG AAATATAAGA AGGAGCATTA   
  
  
+ GCGAACTGCG AACATATGGA CACCGAAAGA GAGGTTATAG CCCGAAATAG CTAAAGGTGA TCAAAGTTTT   
  
  
+ GATGAGAAGA AAGAGATGTG GACTGCTGTG TGAGGTGGGA GAGTTTTAGT TTTTTTTTTT CTGGAAGGAA   
  
  
+ ATGAAGATCG ATGGGTGGAG TGCTTATAAC TTGTTTGAAA GGTGTAAGTT TTTTGGGATG GAAGGAGAAG   
  
  
+ AGAGTGGCCT CATTTATTTT GTTTTGTTTG GTTTGTACAA GCTAATCAGC TAAGAATTCT TGTTTAACTG   
  
  
+ GTTTTGTGTT CTAGTTTTTG GTTTCTTGGA ACTTTGGTGG TTGTTGAGTG AGAAAGTTGA GCAAGTGAGA   
  
  
+ GAGAGAGAGA GGTTTTGTTA GGGGTAGAAA GGTTAGGAGC GAAAAGGAAG TGAGAGAGAG AAAGAGCAGG   
  
  
+ TAAGACTAGT GAAAAGGTTG TGATATGAGA GAGAGAGAGA GAGAGGGAAT AAGGTTAAAT TTCACATCAA   
  
  
+ CCTTATTCGA ATAAGAGAGA CTATTTAGTA GGATAACTTA ATTCGGTCAC AAAATATGCA ATGGTGTGTG   
  
  
+ TATTGAGAGT TGTTTTTACG TGAGGTCGGA TAATAATCAA ACTTAATGGA AATCAATTTT TTATGAAAGT   
  
  
+ GTACGATCGT CTTCTCTATC CACTGTGCGA GTTTTTCTCA CCAAATACAT ATGAAAATGG CATGATTCAC   
  
  
+ CATGCAAGAA AGGGATTTGG AGAATAATTG GTTATAACAG GTCATGTTTG CCTAGCAGCT TCCTTCTACT   
  
  
+ CCATTAATTT GGACTTGTGC ATTGCATGCC TTATATGGGT TGTATTTTGT GGATAAAGGA AAAGGACAAA   
  
  
+ GGGTGGGCTG GTTACGTAGG CTTTTTAGTT TTAATGAAAC ATTTCCGTTA CTGTTCATTT TTAACAAAAA   
  
  
+ TAACATTTTT ACTCTAAAAA AATCACTCCT CGTACTATTC ATTTACAACA CATTTTTGTC ATTTTTATTA   
  
  
+ AAACTCAAAG TTTTCAAGTT TTTTTCATTA ATTTTCCTTT TTATTTATTT ATTTTCAGAA TTAGGCAAAC   
  
  
+ AATTAGCCAC ATTACTTGAT CAATCAACTA TATGACATTG ATAGAATTCT ACTTTGCACT TTGCAACATC   
  
  
+ AAGTTTCCAC TTCAACCAAC CAGATGGGAT TTGCTAATTT TAGTCATTAA CTAAAGATTA TTCGATGTTG   
  
  
+ AGTATAAGGT TTGTCGCTTA AATGATTAAG AGCATTTATT AGTGCACCTA ATTGTTGCAC TACTTGAAGT   
  
  
+ CGTGTATTAG ACCAAGATCA TGCTCATATA ACTCATGGTA GATTAGGATG TGACTTAATT GTAGTTTGTT   
  
  
+ CATTCAAAAT ATCTATTTTC TTTTGGGTG  

- ACCACTCATA AAGAAAAAAG GCCACATCTT CGTCCAGCTT CGTGCTCTTT TGGTTTTCTT TGATCGTTTA   
  
  
- GTGGTGGGAT AATGCTAGGG AAGATCGCTA TAGTAAGAGC TCCTATGTTC TTTATATTCT TCCTCGTAAT   
  
  
- CGCTTGACGC TTGTATACCT GTGGCTTTCT CTCCAATATC GGGCTTTATC GATTTCCACT AGTTTCAAAA   
  
  
- CTACTCTTCT TTCTCTACAC CTGACGACAC ACTCCACCCT CTCAAAATCA AAAAAAAAAA GACCTTCCTT   
  
  
- TACTTCTAGC TACCCACCTC ACGAATATTG AACAAACTTT CCACATTCAA AAAACCCTAC CTTCCTCTTC   
  
  
- TCTCACCGGA GTAAATAAAA CAAAACAAAC CAAACATGTT CGATTAGTCG ATTCTTAAGA ACAAATTGAC   
  
  
- CAAAACACAA GATCAAAAAC CAAAGAACCT TGAAACCACC AACAACTCAC TCTTTCAACT CGTTCACTCT   
  
  
- CTCTCTCTCT CCAAAACAAT CCCCATCTTT CCAATCCTCG CTTTTCCTTC ACTCTCTCTC TTTCTCGTCC   
  
  
- ATTCTGATCA CTTTTCCAAC ACTATACTCT CTCTCTCTCT CTCTCCCTTA TTCCAATTTA AAGTGTAGTT   
  
  
- GGAATAAGCT TATTCTCTCT GATAAATCAT CCTATTGAAT TAAGCCAGTG TTTTATACGT TACCACACAC   
  
  
- ATAACTCTCA ACAAAAATGC ACTCCAGCCT ATTATTAGTT TGAATTACCT TTAGTTAAAA AATACTTTCA   
  
  
- CATGCTAGCA GAAGAGATAG GTGACACGCT CAAAAAGAGT GGTTTATGTA TACTTTTACC GTACTAAGTG   
  
  
- GTACGTTCTT TCCCTAAACC TCTTATTAAC CAATATTGTC CAGTACAAAC GGATCGTCGA AGGAAGATGA   
  
  
- GGTAATTAAA CCTGAACACG TAACGTACGG AATATACCCA ACATAAAACA CCTATTTCCT TTTCCTGTTT   
  
  
- CCCACCCGAC CAATGCATCC GAAAAATCAA AATTACTTTG TAAAGGCAAT GACAAGTAAA AATTGTTTTT   
  
  
- ATTGTAAAAA TGAGATTTTT TTAGTGAGGA GCATGATAAG TAAATGTTGT GTAAAAACAG TAAAAATAAT   
  
  
- TTTGAGTTTC AAAAGTTCAA AAAAAGTAAT TAAAAGGAAA AATAAATAAA TAAAAGTCTT AATCCGTTTG   
  
  
- TTAATCGGTG TAATGAACTA GTTAGTTGAT ATACTGTAAC TATCTTAAGA TGAAACGTGA AACGTTGTAG   
  
  
- TTCAAAGGTG AAGTTGGTTG GTCTACCCTA AACGATTAAA ATCAGTAATT GATTTCTAAT AAGCTACAAC   
  
  
- TCATATTCCA AACAGCGAAT TTACTAATTC TCGTAAATAA TCACGTGGAT TAACAACGTG ATGAACTTCA   
  
  
- GCACATAATC TGGTTCTAGT ACGAGTATAT TGAGTACCAT CTAATCCTAC ACTGAATTAA CATCAAACAA   
  
  
- GTAAGTTTTA TAGATAAAAG AAAACCCAC

+     MYB-like sequence

| Site Name | Organism | Position | Strand | Matrix score. | sequence | function |
| --- | --- | --- | --- | --- | --- | --- |
| MYB-like sequence | Arabidopsis thaliana | 989 | - | 6 | TAACCA |  |
| MYB-like sequence | Arabidopsis thaliana | 869 | - | 6 | TAACCA |  |

>007212   
+ TGGTGAGTAT TTCTTTTTTC CGGTGTAGAA GCAGGTCGAA GCACGAGAAA ACCAAAAGAA ACTAGCAAAT   
  
  
+ CACCACCCTA TTACGATCCC TTCTAGCGAT ATCATTCTCG AGGATACAAG AAATATAAGA AGGAGCATTA   
  
  
+ GCGAACTGCG AACATATGGA CACCGAAAGA GAGGTTATAG CCCGAAATAG CTAAAGGTGA TCAAAGTTTT   
  
  
+ GATGAGAAGA AAGAGATGTG GACTGCTGTG TGAGGTGGGA GAGTTTTAGT TTTTTTTTTT CTGGAAGGAA   
  
  
+ ATGAAGATCG ATGGGTGGAG TGCTTATAAC TTGTTTGAAA GGTGTAAGTT TTTTGGGATG GAAGGAGAAG   
  
  
+ AGAGTGGCCT CATTTATTTT GTTTTGTTTG GTTTGTACAA GCTAATCAGC TAAGAATTCT TGTTTAACTG   
  
  
+ GTTTTGTGTT CTAGTTTTTG GTTTCTTGGA ACTTTGGTGG TTGTTGAGTG AGAAAGTTGA GCAAGTGAGA   
  
  
+ GAGAGAGAGA GGTTTTGTTA GGGGTAGAAA GGTTAGGAGC GAAAAGGAAG TGAGAGAGAG AAAGAGCAGG   
  
  
+ TAAGACTAGT GAAAAGGTTG TGATATGAGA GAGAGAGAGA GAGAGGGAAT AAGGTTAAAT TTCACATCAA   
  
  
+ CCTTATTCGA ATAAGAGAGA CTATTTAGTA GGATAACTTA ATTCGGTCAC AAAATATGCA ATGGTGTGTG   
  
  
+ TATTGAGAGT TGTTTTTACG TGAGGTCGGA TAATAATCAA ACTTAATGGA AATCAATTTT TTATGAAAGT   
  
  
+ GTACGATCGT CTTCTCTATC CACTGTGCGA GTTTTTCTCA CCAAATACAT ATGAAAATGG CATGATTCAC   
  
  
+ CATGCAAGAA AGGGATTTGG AGAATAATTG GTTATAACAG GTCATGTTTG CCTAGCAGCT TCCTTCTACT   
  
  
+ CCATTAATTT GGACTTGTGC ATTGCATGCC TTATATGGGT TGTATTTTGT GGATAAAGGA AAAGGACAAA   
  
  
+ GGGTGGGCTG GTTACGTAGG CTTTTTAGTT TTAATGAAAC ATTTCCGTTA CTGTTCATTT TTAACAAAAA   
  
  
+ TAACATTTTT ACTCTAAAAA AATCACTCCT CGTACTATTC ATTTACAACA CATTTTTGTC ATTTTTATTA   
  
  
+ AAACTCAAAG TTTTCAAGTT TTTTTCATTA ATTTTCCTTT TTATTTATTT ATTTTCAGAA TTAGGCAAAC   
  
  
+ AATTAGCCAC ATTACTTGAT CAATCAACTA TATGACATTG ATAGAATTCT ACTTTGCACT TTGCAACATC   
  
  
+ AAGTTTCCAC TTCAACCAAC CAGATGGGAT TTGCTAATTT TAGTCATTAA CTAAAGATTA TTCGATGTTG   
  
  
+ AGTATAAGGT TTGTCGCTTA AATGATTAAG AGCATTTATT AGTGCACCTA ATTGTTGCAC TACTTGAAGT   
  
  
+ CGTGTATTAG ACCAAGATCA TGCTCATATA ACTCATGGTA GATTAGGATG TGACTTAATT GTAGTTTGTT   
  
  
+ CATTCAAAAT ATCTATTTTC TTTTGGGTG  

- ACCACTCATA AAGAAAAAAG GCCACATCTT CGTCCAGCTT CGTGCTCTTT TGGTTTTCTT TGATCGTTTA   
  
  
- GTGGTGGGAT AATGCTAGGG AAGATCGCTA TAGTAAGAGC TCCTATGTTC TTTATATTCT TCCTCGTAAT   
  
  
- CGCTTGACGC TTGTATACCT GTGGCTTTCT CTCCAATATC GGGCTTTATC GATTTCCACT AGTTTCAAAA   
  
  
- CTACTCTTCT TTCTCTACAC CTGACGACAC ACTCCACCCT CTCAAAATCA AAAAAAAAAA GACCTTCCTT   
  
  
- TACTTCTAGC TACCCACCTC ACGAATATTG AACAAACTTT CCACATTCAA AAAACCCTAC CTTCCTCTTC   
  
  
- TCTCACCGGA GTAAATAAAA CAAAACAAAC CAAACATGTT CGATTAGTCG ATTCTTAAGA ACAAATTGAC   
  
  
- CAAAACACAA GATCAAAAAC CAAAGAACCT TGAAACCACC AACAACTCAC TCTTTCAACT CGTTCACTCT   
  
  
- CTCTCTCTCT CCAAAACAAT CCCCATCTTT CCAATCCTCG CTTTTCCTTC ACTCTCTCTC TTTCTCGTCC   
  
  
- ATTCTGATCA CTTTTCCAAC ACTATACTCT CTCTCTCTCT CTCTCCCTTA TTCCAATTTA AAGTGTAGTT   
  
  
- GGAATAAGCT TATTCTCTCT GATAAATCAT CCTATTGAAT TAAGCCAGTG TTTTATACGT TACCACACAC   
  
  
- ATAACTCTCA ACAAAAATGC ACTCCAGCCT ATTATTAGTT TGAATTACCT TTAGTTAAAA AATACTTTCA   
  
  
- CATGCTAGCA GAAGAGATAG GTGACACGCT CAAAAAGAGT GGTTTATGTA TACTTTTACC GTACTAAGTG   
  
  
- GTACGTTCTT TCCCTAAACC TCTTATTAAC CAATATTGTC CAGTACAAAC GGATCGTCGA AGGAAGATGA   
  
  
- GGTAATTAAA CCTGAACACG TAACGTACGG AATATACCCA ACATAAAACA CCTATTTCCT TTTCCTGTTT   
  
  
- CCCACCCGAC CAATGCATCC GAAAAATCAA AATTACTTTG TAAAGGCAAT GACAAGTAAA AATTGTTTTT   
  
  
- ATTGTAAAAA TGAGATTTTT TTAGTGAGGA GCATGATAAG TAAATGTTGT GTAAAAACAG TAAAAATAAT   
  
  
- TTTGAGTTTC AAAAGTTCAA AAAAAGTAAT TAAAAGGAAA AATAAATAAA TAAAAGTCTT AATCCGTTTG   
  
  
- TTAATCGGTG TAATGAACTA GTTAGTTGAT ATACTGTAAC TATCTTAAGA TGAAACGTGA AACGTTGTAG   
  
  
- TTCAAAGGTG AAGTTGGTTG GTCTACCCTA AACGATTAAA ATCAGTAATT GATTTCTAAT AAGCTACAAC   
  
  
- TCATATTCCA AACAGCGAAT TTACTAATTC TCGTAAATAA TCACGTGGAT TAACAACGTG ATGAACTTCA   
  
  
- GCACATAATC TGGTTCTAGT ACGAGTATAT TGAGTACCAT CTAATCCTAC ACTGAATTAA CATCAAACAA   
  
  
- GTAAGTTTTA TAGATAAAAG AAAACCCAC

+     Myb

| Site Name | Organism | Position | Strand | Matrix score. | sequence | function |
| --- | --- | --- | --- | --- | --- | --- |
| Myb | Arabidopsis thaliana | 415 | + | 6 | TAACTG |  |

>007212   
+ TGGTGAGTAT TTCTTTTTTC CGGTGTAGAA GCAGGTCGAA GCACGAGAAA ACCAAAAGAA ACTAGCAAAT   
  
  
+ CACCACCCTA TTACGATCCC TTCTAGCGAT ATCATTCTCG AGGATACAAG AAATATAAGA AGGAGCATTA   
  
  
+ GCGAACTGCG AACATATGGA CACCGAAAGA GAGGTTATAG CCCGAAATAG CTAAAGGTGA TCAAAGTTTT   
  
  
+ GATGAGAAGA AAGAGATGTG GACTGCTGTG TGAGGTGGGA GAGTTTTAGT TTTTTTTTTT CTGGAAGGAA   
  
  
+ ATGAAGATCG ATGGGTGGAG TGCTTATAAC TTGTTTGAAA GGTGTAAGTT TTTTGGGATG GAAGGAGAAG   
  
  
+ AGAGTGGCCT CATTTATTTT GTTTTGTTTG GTTTGTACAA GCTAATCAGC TAAGAATTCT TGTTTAACTG   
  
  
+ GTTTTGTGTT CTAGTTTTTG GTTTCTTGGA ACTTTGGTGG TTGTTGAGTG AGAAAGTTGA GCAAGTGAGA   
  
  
+ GAGAGAGAGA GGTTTTGTTA GGGGTAGAAA GGTTAGGAGC GAAAAGGAAG TGAGAGAGAG AAAGAGCAGG   
  
  
+ TAAGACTAGT GAAAAGGTTG TGATATGAGA GAGAGAGAGA GAGAGGGAAT AAGGTTAAAT TTCACATCAA   
  
  
+ CCTTATTCGA ATAAGAGAGA CTATTTAGTA GGATAACTTA ATTCGGTCAC AAAATATGCA ATGGTGTGTG   
  
  
+ TATTGAGAGT TGTTTTTACG TGAGGTCGGA TAATAATCAA ACTTAATGGA AATCAATTTT TTATGAAAGT   
  
  
+ GTACGATCGT CTTCTCTATC CACTGTGCGA GTTTTTCTCA CCAAATACAT ATGAAAATGG CATGATTCAC   
  
  
+ CATGCAAGAA AGGGATTTGG AGAATAATTG GTTATAACAG GTCATGTTTG CCTAGCAGCT TCCTTCTACT   
  
  
+ CCATTAATTT GGACTTGTGC ATTGCATGCC TTATATGGGT TGTATTTTGT GGATAAAGGA AAAGGACAAA   
  
  
+ GGGTGGGCTG GTTACGTAGG CTTTTTAGTT TTAATGAAAC ATTTCCGTTA CTGTTCATTT TTAACAAAAA   
  
  
+ TAACATTTTT ACTCTAAAAA AATCACTCCT CGTACTATTC ATTTACAACA CATTTTTGTC ATTTTTATTA   
  
  
+ AAACTCAAAG TTTTCAAGTT TTTTTCATTA ATTTTCCTTT TTATTTATTT ATTTTCAGAA TTAGGCAAAC   
  
  
+ AATTAGCCAC ATTACTTGAT CAATCAACTA TATGACATTG ATAGAATTCT ACTTTGCACT TTGCAACATC   
  
  
+ AAGTTTCCAC TTCAACCAAC CAGATGGGAT TTGCTAATTT TAGTCATTAA CTAAAGATTA TTCGATGTTG   
  
  
+ AGTATAAGGT TTGTCGCTTA AATGATTAAG AGCATTTATT AGTGCACCTA ATTGTTGCAC TACTTGAAGT   
  
  
+ CGTGTATTAG ACCAAGATCA TGCTCATATA ACTCATGGTA GATTAGGATG TGACTTAATT GTAGTTTGTT   
  
  
+ CATTCAAAAT ATCTATTTTC TTTTGGGTG  

- ACCACTCATA AAGAAAAAAG GCCACATCTT CGTCCAGCTT CGTGCTCTTT TGGTTTTCTT TGATCGTTTA   
  
  
- GTGGTGGGAT AATGCTAGGG AAGATCGCTA TAGTAAGAGC TCCTATGTTC TTTATATTCT TCCTCGTAAT   
  
  
- CGCTTGACGC TTGTATACCT GTGGCTTTCT CTCCAATATC GGGCTTTATC GATTTCCACT AGTTTCAAAA   
  
  
- CTACTCTTCT TTCTCTACAC CTGACGACAC ACTCCACCCT CTCAAAATCA AAAAAAAAAA GACCTTCCTT   
  
  
- TACTTCTAGC TACCCACCTC ACGAATATTG AACAAACTTT CCACATTCAA AAAACCCTAC CTTCCTCTTC   
  
  
- TCTCACCGGA GTAAATAAAA CAAAACAAAC CAAACATGTT CGATTAGTCG ATTCTTAAGA ACAAATTGAC   
  
  
- CAAAACACAA GATCAAAAAC CAAAGAACCT TGAAACCACC AACAACTCAC TCTTTCAACT CGTTCACTCT   
  
  
- CTCTCTCTCT CCAAAACAAT CCCCATCTTT CCAATCCTCG CTTTTCCTTC ACTCTCTCTC TTTCTCGTCC   
  
  
- ATTCTGATCA CTTTTCCAAC ACTATACTCT CTCTCTCTCT CTCTCCCTTA TTCCAATTTA AAGTGTAGTT   
  
  
- GGAATAAGCT TATTCTCTCT GATAAATCAT CCTATTGAAT TAAGCCAGTG TTTTATACGT TACCACACAC   
  
  
- ATAACTCTCA ACAAAAATGC ACTCCAGCCT ATTATTAGTT TGAATTACCT TTAGTTAAAA AATACTTTCA   
  
  
- CATGCTAGCA GAAGAGATAG GTGACACGCT CAAAAAGAGT GGTTTATGTA TACTTTTACC GTACTAAGTG   
  
  
- GTACGTTCTT TCCCTAAACC TCTTATTAAC CAATATTGTC CAGTACAAAC GGATCGTCGA AGGAAGATGA   
  
  
- GGTAATTAAA CCTGAACACG TAACGTACGG AATATACCCA ACATAAAACA CCTATTTCCT TTTCCTGTTT   
  
  
- CCCACCCGAC CAATGCATCC GAAAAATCAA AATTACTTTG TAAAGGCAAT GACAAGTAAA AATTGTTTTT   
  
  
- ATTGTAAAAA TGAGATTTTT TTAGTGAGGA GCATGATAAG TAAATGTTGT GTAAAAACAG TAAAAATAAT   
  
  
- TTTGAGTTTC AAAAGTTCAA AAAAAGTAAT TAAAAGGAAA AATAAATAAA TAAAAGTCTT AATCCGTTTG   
  
  
- TTAATCGGTG TAATGAACTA GTTAGTTGAT ATACTGTAAC TATCTTAAGA TGAAACGTGA AACGTTGTAG   
  
  
- TTCAAAGGTG AAGTTGGTTG GTCTACCCTA AACGATTAAA ATCAGTAATT GATTTCTAAT AAGCTACAAC   
  
  
- TCATATTCCA AACAGCGAAT TTACTAATTC TCGTAAATAA TCACGTGGAT TAACAACGTG ATGAACTTCA   
  
  
- GCACATAATC TGGTTCTAGT ACGAGTATAT TGAGTACCAT CTAATCCTAC ACTGAATTAA CATCAAACAA   
  
  
- GTAAGTTTTA TAGATAAAAG AAAACCCAC

+     Myc

| Site Name | Organism | Position | Strand | Matrix score. | sequence | function |
| --- | --- | --- | --- | --- | --- | --- |
| Myc | Arabidopsis thaliana | 642 | - | 7 | TCTCTTA |  |

>007212   
+ TGGTGAGTAT TTCTTTTTTC CGGTGTAGAA GCAGGTCGAA GCACGAGAAA ACCAAAAGAA ACTAGCAAAT   
  
  
+ CACCACCCTA TTACGATCCC TTCTAGCGAT ATCATTCTCG AGGATACAAG AAATATAAGA AGGAGCATTA   
  
  
+ GCGAACTGCG AACATATGGA CACCGAAAGA GAGGTTATAG CCCGAAATAG CTAAAGGTGA TCAAAGTTTT   
  
  
+ GATGAGAAGA AAGAGATGTG GACTGCTGTG TGAGGTGGGA GAGTTTTAGT TTTTTTTTTT CTGGAAGGAA   
  
  
+ ATGAAGATCG ATGGGTGGAG TGCTTATAAC TTGTTTGAAA GGTGTAAGTT TTTTGGGATG GAAGGAGAAG   
  
  
+ AGAGTGGCCT CATTTATTTT GTTTTGTTTG GTTTGTACAA GCTAATCAGC TAAGAATTCT TGTTTAACTG   
  
  
+ GTTTTGTGTT CTAGTTTTTG GTTTCTTGGA ACTTTGGTGG TTGTTGAGTG AGAAAGTTGA GCAAGTGAGA   
  
  
+ GAGAGAGAGA GGTTTTGTTA GGGGTAGAAA GGTTAGGAGC GAAAAGGAAG TGAGAGAGAG AAAGAGCAGG   
  
  
+ TAAGACTAGT GAAAAGGTTG TGATATGAGA GAGAGAGAGA GAGAGGGAAT AAGGTTAAAT TTCACATCAA   
  
  
+ CCTTATTCGA ATAAGAGAGA CTATTTAGTA GGATAACTTA ATTCGGTCAC AAAATATGCA ATGGTGTGTG   
  
  
+ TATTGAGAGT TGTTTTTACG TGAGGTCGGA TAATAATCAA ACTTAATGGA AATCAATTTT TTATGAAAGT   
  
  
+ GTACGATCGT CTTCTCTATC CACTGTGCGA GTTTTTCTCA CCAAATACAT ATGAAAATGG CATGATTCAC   
  
  
+ CATGCAAGAA AGGGATTTGG AGAATAATTG GTTATAACAG GTCATGTTTG CCTAGCAGCT TCCTTCTACT   
  
  
+ CCATTAATTT GGACTTGTGC ATTGCATGCC TTATATGGGT TGTATTTTGT GGATAAAGGA AAAGGACAAA   
  
  
+ GGGTGGGCTG GTTACGTAGG CTTTTTAGTT TTAATGAAAC ATTTCCGTTA CTGTTCATTT TTAACAAAAA   
  
  
+ TAACATTTTT ACTCTAAAAA AATCACTCCT CGTACTATTC ATTTACAACA CATTTTTGTC ATTTTTATTA   
  
  
+ AAACTCAAAG TTTTCAAGTT TTTTTCATTA ATTTTCCTTT TTATTTATTT ATTTTCAGAA TTAGGCAAAC   
  
  
+ AATTAGCCAC ATTACTTGAT CAATCAACTA TATGACATTG ATAGAATTCT ACTTTGCACT TTGCAACATC   
  
  
+ AAGTTTCCAC TTCAACCAAC CAGATGGGAT TTGCTAATTT TAGTCATTAA CTAAAGATTA TTCGATGTTG   
  
  
+ AGTATAAGGT TTGTCGCTTA AATGATTAAG AGCATTTATT AGTGCACCTA ATTGTTGCAC TACTTGAAGT   
  
  
+ CGTGTATTAG ACCAAGATCA TGCTCATATA ACTCATGGTA GATTAGGATG TGACTTAATT GTAGTTTGTT   
  
  
+ CATTCAAAAT ATCTATTTTC TTTTGGGTG  

- ACCACTCATA AAGAAAAAAG GCCACATCTT CGTCCAGCTT CGTGCTCTTT TGGTTTTCTT TGATCGTTTA   
  
  
- GTGGTGGGAT AATGCTAGGG AAGATCGCTA TAGTAAGAGC TCCTATGTTC TTTATATTCT TCCTCGTAAT   
  
  
- CGCTTGACGC TTGTATACCT GTGGCTTTCT CTCCAATATC GGGCTTTATC GATTTCCACT AGTTTCAAAA   
  
  
- CTACTCTTCT TTCTCTACAC CTGACGACAC ACTCCACCCT CTCAAAATCA AAAAAAAAAA GACCTTCCTT   
  
  
- TACTTCTAGC TACCCACCTC ACGAATATTG AACAAACTTT CCACATTCAA AAAACCCTAC CTTCCTCTTC   
  
  
- TCTCACCGGA GTAAATAAAA CAAAACAAAC CAAACATGTT CGATTAGTCG ATTCTTAAGA ACAAATTGAC   
  
  
- CAAAACACAA GATCAAAAAC CAAAGAACCT TGAAACCACC AACAACTCAC TCTTTCAACT CGTTCACTCT   
  
  
- CTCTCTCTCT CCAAAACAAT CCCCATCTTT CCAATCCTCG CTTTTCCTTC ACTCTCTCTC TTTCTCGTCC   
  
  
- ATTCTGATCA CTTTTCCAAC ACTATACTCT CTCTCTCTCT CTCTCCCTTA TTCCAATTTA AAGTGTAGTT   
  
  
- GGAATAAGCT TATTCTCTCT GATAAATCAT CCTATTGAAT TAAGCCAGTG TTTTATACGT TACCACACAC   
  
  
- ATAACTCTCA ACAAAAATGC ACTCCAGCCT ATTATTAGTT TGAATTACCT TTAGTTAAAA AATACTTTCA   
  
  
- CATGCTAGCA GAAGAGATAG GTGACACGCT CAAAAAGAGT GGTTTATGTA TACTTTTACC GTACTAAGTG   
  
  
- GTACGTTCTT TCCCTAAACC TCTTATTAAC CAATATTGTC CAGTACAAAC GGATCGTCGA AGGAAGATGA   
  
  
- GGTAATTAAA CCTGAACACG TAACGTACGG AATATACCCA ACATAAAACA CCTATTTCCT TTTCCTGTTT   
  
  
- CCCACCCGAC CAATGCATCC GAAAAATCAA AATTACTTTG TAAAGGCAAT GACAAGTAAA AATTGTTTTT   
  
  
- ATTGTAAAAA TGAGATTTTT TTAGTGAGGA GCATGATAAG TAAATGTTGT GTAAAAACAG TAAAAATAAT   
  
  
- TTTGAGTTTC AAAAGTTCAA AAAAAGTAAT TAAAAGGAAA AATAAATAAA TAAAAGTCTT AATCCGTTTG   
  
  
- TTAATCGGTG TAATGAACTA GTTAGTTGAT ATACTGTAAC TATCTTAAGA TGAAACGTGA AACGTTGTAG   
  
  
- TTCAAAGGTG AAGTTGGTTG GTCTACCCTA AACGATTAAA ATCAGTAATT GATTTCTAAT AAGCTACAAC   
  
  
- TCATATTCCA AACAGCGAAT TTACTAATTC TCGTAAATAA TCACGTGGAT TAACAACGTG ATGAACTTCA   
  
  
- GCACATAATC TGGTTCTAGT ACGAGTATAT TGAGTACCAT CTAATCCTAC ACTGAATTAA CATCAAACAA   
  
  
- GTAAGTTTTA TAGATAAAAG AAAACCCAC

+     O2-site

| Site Name | Organism | Position | Strand | Matrix score. | sequence | function |
| --- | --- | --- | --- | --- | --- | --- |
| O2-site | Zea mays | 622 | - | 9 | GTTGACGTGA | cis-acting regulatory element involved in zein metabolism regulation |

>007212   
+ TGGTGAGTAT TTCTTTTTTC CGGTGTAGAA GCAGGTCGAA GCACGAGAAA ACCAAAAGAA ACTAGCAAAT   
  
  
+ CACCACCCTA TTACGATCCC TTCTAGCGAT ATCATTCTCG AGGATACAAG AAATATAAGA AGGAGCATTA   
  
  
+ GCGAACTGCG AACATATGGA CACCGAAAGA GAGGTTATAG CCCGAAATAG CTAAAGGTGA TCAAAGTTTT   
  
  
+ GATGAGAAGA AAGAGATGTG GACTGCTGTG TGAGGTGGGA GAGTTTTAGT TTTTTTTTTT CTGGAAGGAA   
  
  
+ ATGAAGATCG ATGGGTGGAG TGCTTATAAC TTGTTTGAAA GGTGTAAGTT TTTTGGGATG GAAGGAGAAG   
  
  
+ AGAGTGGCCT CATTTATTTT GTTTTGTTTG GTTTGTACAA GCTAATCAGC TAAGAATTCT TGTTTAACTG   
  
  
+ GTTTTGTGTT CTAGTTTTTG GTTTCTTGGA ACTTTGGTGG TTGTTGAGTG AGAAAGTTGA GCAAGTGAGA   
  
  
+ GAGAGAGAGA GGTTTTGTTA GGGGTAGAAA GGTTAGGAGC GAAAAGGAAG TGAGAGAGAG AAAGAGCAGG   
  
  
+ TAAGACTAGT GAAAAGGTTG TGATATGAGA GAGAGAGAGA GAGAGGGAAT AAGGTTAAAT TTCACATCAA   
  
  
+ CCTTATTCGA ATAAGAGAGA CTATTTAGTA GGATAACTTA ATTCGGTCAC AAAATATGCA ATGGTGTGTG   
  
  
+ TATTGAGAGT TGTTTTTACG TGAGGTCGGA TAATAATCAA ACTTAATGGA AATCAATTTT TTATGAAAGT   
  
  
+ GTACGATCGT CTTCTCTATC CACTGTGCGA GTTTTTCTCA CCAAATACAT ATGAAAATGG CATGATTCAC   
  
  
+ CATGCAAGAA AGGGATTTGG AGAATAATTG GTTATAACAG GTCATGTTTG CCTAGCAGCT TCCTTCTACT   
  
  
+ CCATTAATTT GGACTTGTGC ATTGCATGCC TTATATGGGT TGTATTTTGT GGATAAAGGA AAAGGACAAA   
  
  
+ GGGTGGGCTG GTTACGTAGG CTTTTTAGTT TTAATGAAAC ATTTCCGTTA CTGTTCATTT TTAACAAAAA   
  
  
+ TAACATTTTT ACTCTAAAAA AATCACTCCT CGTACTATTC ATTTACAACA CATTTTTGTC ATTTTTATTA   
  
  
+ AAACTCAAAG TTTTCAAGTT TTTTTCATTA ATTTTCCTTT TTATTTATTT ATTTTCAGAA TTAGGCAAAC   
  
  
+ AATTAGCCAC ATTACTTGAT CAATCAACTA TATGACATTG ATAGAATTCT ACTTTGCACT TTGCAACATC   
  
  
+ AAGTTTCCAC TTCAACCAAC CAGATGGGAT TTGCTAATTT TAGTCATTAA CTAAAGATTA TTCGATGTTG   
  
  
+ AGTATAAGGT TTGTCGCTTA AATGATTAAG AGCATTTATT AGTGCACCTA ATTGTTGCAC TACTTGAAGT   
  
  
+ CGTGTATTAG ACCAAGATCA TGCTCATATA ACTCATGGTA GATTAGGATG TGACTTAATT GTAGTTTGTT   
  
  
+ CATTCAAAAT ATCTATTTTC TTTTGGGTG  

- ACCACTCATA AAGAAAAAAG GCCACATCTT CGTCCAGCTT CGTGCTCTTT TGGTTTTCTT TGATCGTTTA   
  
  
- GTGGTGGGAT AATGCTAGGG AAGATCGCTA TAGTAAGAGC TCCTATGTTC TTTATATTCT TCCTCGTAAT   
  
  
- CGCTTGACGC TTGTATACCT GTGGCTTTCT CTCCAATATC GGGCTTTATC GATTTCCACT AGTTTCAAAA   
  
  
- CTACTCTTCT TTCTCTACAC CTGACGACAC ACTCCACCCT CTCAAAATCA AAAAAAAAAA GACCTTCCTT   
  
  
- TACTTCTAGC TACCCACCTC ACGAATATTG AACAAACTTT CCACATTCAA AAAACCCTAC CTTCCTCTTC   
  
  
- TCTCACCGGA GTAAATAAAA CAAAACAAAC CAAACATGTT CGATTAGTCG ATTCTTAAGA ACAAATTGAC   
  
  
- CAAAACACAA GATCAAAAAC CAAAGAACCT TGAAACCACC AACAACTCAC TCTTTCAACT CGTTCACTCT   
  
  
- CTCTCTCTCT CCAAAACAAT CCCCATCTTT CCAATCCTCG CTTTTCCTTC ACTCTCTCTC TTTCTCGTCC   
  
  
- ATTCTGATCA CTTTTCCAAC ACTATACTCT CTCTCTCTCT CTCTCCCTTA TTCCAATTTA AAGTGTAGTT   
  
  
- GGAATAAGCT TATTCTCTCT GATAAATCAT CCTATTGAAT TAAGCCAGTG TTTTATACGT TACCACACAC   
  
  
- ATAACTCTCA ACAAAAATGC ACTCCAGCCT ATTATTAGTT TGAATTACCT TTAGTTAAAA AATACTTTCA   
  
  
- CATGCTAGCA GAAGAGATAG GTGACACGCT CAAAAAGAGT GGTTTATGTA TACTTTTACC GTACTAAGTG   
  
  
- GTACGTTCTT TCCCTAAACC TCTTATTAAC CAATATTGTC CAGTACAAAC GGATCGTCGA AGGAAGATGA   
  
  
- GGTAATTAAA CCTGAACACG TAACGTACGG AATATACCCA ACATAAAACA CCTATTTCCT TTTCCTGTTT   
  
  
- CCCACCCGAC CAATGCATCC GAAAAATCAA AATTACTTTG TAAAGGCAAT GACAAGTAAA AATTGTTTTT   
  
  
- ATTGTAAAAA TGAGATTTTT TTAGTGAGGA GCATGATAAG TAAATGTTGT GTAAAAACAG TAAAAATAAT   
  
  
- TTTGAGTTTC AAAAGTTCAA AAAAAGTAAT TAAAAGGAAA AATAAATAAA TAAAAGTCTT AATCCGTTTG   
  
  
- TTAATCGGTG TAATGAACTA GTTAGTTGAT ATACTGTAAC TATCTTAAGA TGAAACGTGA AACGTTGTAG   
  
  
- TTCAAAGGTG AAGTTGGTTG GTCTACCCTA AACGATTAAA ATCAGTAATT GATTTCTAAT AAGCTACAAC   
  
  
- TCATATTCCA AACAGCGAAT TTACTAATTC TCGTAAATAA TCACGTGGAT TAACAACGTG ATGAACTTCA   
  
  
- GCACATAATC TGGTTCTAGT ACGAGTATAT TGAGTACCAT CTAATCCTAC ACTGAATTAA CATCAAACAA   
  
  
- GTAAGTTTTA TAGATAAAAG AAAACCCAC

+     STRE

| Site Name | Organism | Position | Strand | Matrix score. | sequence | function |
| --- | --- | --- | --- | --- | --- | --- |
| STRE | Arabidopsis thaliana | 510 | + | 5 | AGGGG |  |

>007212   
+ TGGTGAGTAT TTCTTTTTTC CGGTGTAGAA GCAGGTCGAA GCACGAGAAA ACCAAAAGAA ACTAGCAAAT   
  
  
+ CACCACCCTA TTACGATCCC TTCTAGCGAT ATCATTCTCG AGGATACAAG AAATATAAGA AGGAGCATTA   
  
  
+ GCGAACTGCG AACATATGGA CACCGAAAGA GAGGTTATAG CCCGAAATAG CTAAAGGTGA TCAAAGTTTT   
  
  
+ GATGAGAAGA AAGAGATGTG GACTGCTGTG TGAGGTGGGA GAGTTTTAGT TTTTTTTTTT CTGGAAGGAA   
  
  
+ ATGAAGATCG ATGGGTGGAG TGCTTATAAC TTGTTTGAAA GGTGTAAGTT TTTTGGGATG GAAGGAGAAG   
  
  
+ AGAGTGGCCT CATTTATTTT GTTTTGTTTG GTTTGTACAA GCTAATCAGC TAAGAATTCT TGTTTAACTG   
  
  
+ GTTTTGTGTT CTAGTTTTTG GTTTCTTGGA ACTTTGGTGG TTGTTGAGTG AGAAAGTTGA GCAAGTGAGA   
  
  
+ GAGAGAGAGA GGTTTTGTTA GGGGTAGAAA GGTTAGGAGC GAAAAGGAAG TGAGAGAGAG AAAGAGCAGG   
  
  
+ TAAGACTAGT GAAAAGGTTG TGATATGAGA GAGAGAGAGA GAGAGGGAAT AAGGTTAAAT TTCACATCAA   
  
  
+ CCTTATTCGA ATAAGAGAGA CTATTTAGTA GGATAACTTA ATTCGGTCAC AAAATATGCA ATGGTGTGTG   
  
  
+ TATTGAGAGT TGTTTTTACG TGAGGTCGGA TAATAATCAA ACTTAATGGA AATCAATTTT TTATGAAAGT   
  
  
+ GTACGATCGT CTTCTCTATC CACTGTGCGA GTTTTTCTCA CCAAATACAT ATGAAAATGG CATGATTCAC   
  
  
+ CATGCAAGAA AGGGATTTGG AGAATAATTG GTTATAACAG GTCATGTTTG CCTAGCAGCT TCCTTCTACT   
  
  
+ CCATTAATTT GGACTTGTGC ATTGCATGCC TTATATGGGT TGTATTTTGT GGATAAAGGA AAAGGACAAA   
  
  
+ GGGTGGGCTG GTTACGTAGG CTTTTTAGTT TTAATGAAAC ATTTCCGTTA CTGTTCATTT TTAACAAAAA   
  
  
+ TAACATTTTT ACTCTAAAAA AATCACTCCT CGTACTATTC ATTTACAACA CATTTTTGTC ATTTTTATTA   
  
  
+ AAACTCAAAG TTTTCAAGTT TTTTTCATTA ATTTTCCTTT TTATTTATTT ATTTTCAGAA TTAGGCAAAC   
  
  
+ AATTAGCCAC ATTACTTGAT CAATCAACTA TATGACATTG ATAGAATTCT ACTTTGCACT TTGCAACATC   
  
  
+ AAGTTTCCAC TTCAACCAAC CAGATGGGAT TTGCTAATTT TAGTCATTAA CTAAAGATTA TTCGATGTTG   
  
  
+ AGTATAAGGT TTGTCGCTTA AATGATTAAG AGCATTTATT AGTGCACCTA ATTGTTGCAC TACTTGAAGT   
  
  
+ CGTGTATTAG ACCAAGATCA TGCTCATATA ACTCATGGTA GATTAGGATG TGACTTAATT GTAGTTTGTT   
  
  
+ CATTCAAAAT ATCTATTTTC TTTTGGGTG  

- ACCACTCATA AAGAAAAAAG GCCACATCTT CGTCCAGCTT CGTGCTCTTT TGGTTTTCTT TGATCGTTTA   
  
  
- GTGGTGGGAT AATGCTAGGG AAGATCGCTA TAGTAAGAGC TCCTATGTTC TTTATATTCT TCCTCGTAAT   
  
  
- CGCTTGACGC TTGTATACCT GTGGCTTTCT CTCCAATATC GGGCTTTATC GATTTCCACT AGTTTCAAAA   
  
  
- CTACTCTTCT TTCTCTACAC CTGACGACAC ACTCCACCCT CTCAAAATCA AAAAAAAAAA GACCTTCCTT   
  
  
- TACTTCTAGC TACCCACCTC ACGAATATTG AACAAACTTT CCACATTCAA AAAACCCTAC CTTCCTCTTC   
  
  
- TCTCACCGGA GTAAATAAAA CAAAACAAAC CAAACATGTT CGATTAGTCG ATTCTTAAGA ACAAATTGAC   
  
  
- CAAAACACAA GATCAAAAAC CAAAGAACCT TGAAACCACC AACAACTCAC TCTTTCAACT CGTTCACTCT   
  
  
- CTCTCTCTCT CCAAAACAAT CCCCATCTTT CCAATCCTCG CTTTTCCTTC ACTCTCTCTC TTTCTCGTCC   
  
  
- ATTCTGATCA CTTTTCCAAC ACTATACTCT CTCTCTCTCT CTCTCCCTTA TTCCAATTTA AAGTGTAGTT   
  
  
- GGAATAAGCT TATTCTCTCT GATAAATCAT CCTATTGAAT TAAGCCAGTG TTTTATACGT TACCACACAC   
  
  
- ATAACTCTCA ACAAAAATGC ACTCCAGCCT ATTATTAGTT TGAATTACCT TTAGTTAAAA AATACTTTCA   
  
  
- CATGCTAGCA GAAGAGATAG GTGACACGCT CAAAAAGAGT GGTTTATGTA TACTTTTACC GTACTAAGTG   
  
  
- GTACGTTCTT TCCCTAAACC TCTTATTAAC CAATATTGTC CAGTACAAAC GGATCGTCGA AGGAAGATGA   
  
  
- GGTAATTAAA CCTGAACACG TAACGTACGG AATATACCCA ACATAAAACA CCTATTTCCT TTTCCTGTTT   
  
  
- CCCACCCGAC CAATGCATCC GAAAAATCAA AATTACTTTG TAAAGGCAAT GACAAGTAAA AATTGTTTTT   
  
  
- ATTGTAAAAA TGAGATTTTT TTAGTGAGGA GCATGATAAG TAAATGTTGT GTAAAAACAG TAAAAATAAT   
  
  
- TTTGAGTTTC AAAAGTTCAA AAAAAGTAAT TAAAAGGAAA AATAAATAAA TAAAAGTCTT AATCCGTTTG   
  
  
- TTAATCGGTG TAATGAACTA GTTAGTTGAT ATACTGTAAC TATCTTAAGA TGAAACGTGA AACGTTGTAG   
  
  
- TTCAAAGGTG AAGTTGGTTG GTCTACCCTA AACGATTAAA ATCAGTAATT GATTTCTAAT AAGCTACAAC   
  
  
- TCATATTCCA AACAGCGAAT TTACTAATTC TCGTAAATAA TCACGTGGAT TAACAACGTG ATGAACTTCA   
  
  
- GCACATAATC TGGTTCTAGT ACGAGTATAT TGAGTACCAT CTAATCCTAC ACTGAATTAA CATCAAACAA   
  
  
- GTAAGTTTTA TAGATAAAAG AAAACCCAC

+     TATA-box

| Site Name | Organism | Position | Strand | Matrix score. | sequence | function |
| --- | --- | --- | --- | --- | --- | --- |
| TATA-box | Arabidopsis thaliana | 1427 | - | 4 | TATA | core promoter element around -30 of transcription start |
| TATA-box | Arabidopsis thaliana | 1312 | + | 8 | TAAAGATT | core promoter element around -30 of transcription start |
| TATA-box | Brassica oleracea | 1426 | + | 6 | ATATAA | core promoter element around -30 of transcription start |
| TATA-box | Arabidopsis thaliana | 1333 | - | 4 | TATA | core promoter element around -30 of transcription start |
| TATA-box | Arabidopsis thaliana | 1219 | - | 4 | TATA | core promoter element around -30 of transcription start |
| TATA-box | Arabidopsis thaliana | 873 | - | 4 | TATA | core promoter element around -30 of transcription start |
| TATA-box | Arabidopsis thaliana | 942 | - | 4 | TATA | core promoter element around -30 of transcription start |
| TATA-box | Arabidopsis thaliana | 941 | - | 5 | TATAA | core promoter element around -30 of transcription start |
| TATA-box | Arabidopsis thaliana | 872 | - | 5 | TATAA | core promoter element around -30 of transcription start |
| TATA-box | Arabidopsis thaliana | 305 | + | 4 | TATA | core promoter element around -30 of transcription start |
| TATA-box | Arabidopsis thaliana | 176 | + | 4 | TATA | core promoter element around -30 of transcription start |
| TATA-box | Arabidopsis thaliana | 304 | - | 5 | TATAA | core promoter element around -30 of transcription start |
| TATA-box | Arabidopsis thaliana | 175 | - | 5 | TATAA | core promoter element around -30 of transcription start |
| TATA-box | Arabidopsis thaliana | 124 | + | 4 | TATA | core promoter element around -30 of transcription start |
| TATA-box | Brassica oleracea | 123 | + | 6 | ATATAA | core promoter element around -30 of transcription start |

>007212   
+ TGGTGAGTAT TTCTTTTTTC CGGTGTAGAA GCAGGTCGAA GCACGAGAAA ACCAAAAGAA ACTAGCAAAT   
  
  
+ CACCACCCTA TTACGATCCC TTCTAGCGAT ATCATTCTCG AGGATACAAG AAATATAAGA AGGAGCATTA   
  
  
+ GCGAACTGCG AACATATGGA CACCGAAAGA GAGGTTATAG CCCGAAATAG CTAAAGGTGA TCAAAGTTTT   
  
  
+ GATGAGAAGA AAGAGATGTG GACTGCTGTG TGAGGTGGGA GAGTTTTAGT TTTTTTTTTT CTGGAAGGAA   
  
  
+ ATGAAGATCG ATGGGTGGAG TGCTTATAAC TTGTTTGAAA GGTGTAAGTT TTTTGGGATG GAAGGAGAAG   
  
  
+ AGAGTGGCCT CATTTATTTT GTTTTGTTTG GTTTGTACAA GCTAATCAGC TAAGAATTCT TGTTTAACTG   
  
  
+ GTTTTGTGTT CTAGTTTTTG GTTTCTTGGA ACTTTGGTGG TTGTTGAGTG AGAAAGTTGA GCAAGTGAGA   
  
  
+ GAGAGAGAGA GGTTTTGTTA GGGGTAGAAA GGTTAGGAGC GAAAAGGAAG TGAGAGAGAG AAAGAGCAGG   
  
  
+ TAAGACTAGT GAAAAGGTTG TGATATGAGA GAGAGAGAGA GAGAGGGAAT AAGGTTAAAT TTCACATCAA   
  
  
+ CCTTATTCGA ATAAGAGAGA CTATTTAGTA GGATAACTTA ATTCGGTCAC AAAATATGCA ATGGTGTGTG   
  
  
+ TATTGAGAGT TGTTTTTACG TGAGGTCGGA TAATAATCAA ACTTAATGGA AATCAATTTT TTATGAAAGT   
  
  
+ GTACGATCGT CTTCTCTATC CACTGTGCGA GTTTTTCTCA CCAAATACAT ATGAAAATGG CATGATTCAC   
  
  
+ CATGCAAGAA AGGGATTTGG AGAATAATTG GTTATAACAG GTCATGTTTG CCTAGCAGCT TCCTTCTACT   
  
  
+ CCATTAATTT GGACTTGTGC ATTGCATGCC TTATATGGGT TGTATTTTGT GGATAAAGGA AAAGGACAAA   
  
  
+ GGGTGGGCTG GTTACGTAGG CTTTTTAGTT TTAATGAAAC ATTTCCGTTA CTGTTCATTT TTAACAAAAA   
  
  
+ TAACATTTTT ACTCTAAAAA AATCACTCCT CGTACTATTC ATTTACAACA CATTTTTGTC ATTTTTATTA   
  
  
+ AAACTCAAAG TTTTCAAGTT TTTTTCATTA ATTTTCCTTT TTATTTATTT ATTTTCAGAA TTAGGCAAAC   
  
  
+ AATTAGCCAC ATTACTTGAT CAATCAACTA TATGACATTG ATAGAATTCT ACTTTGCACT TTGCAACATC   
  
  
+ AAGTTTCCAC TTCAACCAAC CAGATGGGAT TTGCTAATTT TAGTCATTAA CTAAAGATTA TTCGATGTTG   
  
  
+ AGTATAAGGT TTGTCGCTTA AATGATTAAG AGCATTTATT AGTGCACCTA ATTGTTGCAC TACTTGAAGT   
  
  
+ CGTGTATTAG ACCAAGATCA TGCTCATATA ACTCATGGTA GATTAGGATG TGACTTAATT GTAGTTTGTT   
  
  
+ CATTCAAAAT ATCTATTTTC TTTTGGGTG  

- ACCACTCATA AAGAAAAAAG GCCACATCTT CGTCCAGCTT CGTGCTCTTT TGGTTTTCTT TGATCGTTTA   
  
  
- GTGGTGGGAT AATGCTAGGG AAGATCGCTA TAGTAAGAGC TCCTATGTTC TTTATATTCT TCCTCGTAAT   
  
  
- CGCTTGACGC TTGTATACCT GTGGCTTTCT CTCCAATATC GGGCTTTATC GATTTCCACT AGTTTCAAAA   
  
  
- CTACTCTTCT TTCTCTACAC CTGACGACAC ACTCCACCCT CTCAAAATCA AAAAAAAAAA GACCTTCCTT   
  
  
- TACTTCTAGC TACCCACCTC ACGAATATTG AACAAACTTT CCACATTCAA AAAACCCTAC CTTCCTCTTC   
  
  
- TCTCACCGGA GTAAATAAAA CAAAACAAAC CAAACATGTT CGATTAGTCG ATTCTTAAGA ACAAATTGAC   
  
  
- CAAAACACAA GATCAAAAAC CAAAGAACCT TGAAACCACC AACAACTCAC TCTTTCAACT CGTTCACTCT   
  
  
- CTCTCTCTCT CCAAAACAAT CCCCATCTTT CCAATCCTCG CTTTTCCTTC ACTCTCTCTC TTTCTCGTCC   
  
  
- ATTCTGATCA CTTTTCCAAC ACTATACTCT CTCTCTCTCT CTCTCCCTTA TTCCAATTTA AAGTGTAGTT   
  
  
- GGAATAAGCT TATTCTCTCT GATAAATCAT CCTATTGAAT TAAGCCAGTG TTTTATACGT TACCACACAC   
  
  
- ATAACTCTCA ACAAAAATGC ACTCCAGCCT ATTATTAGTT TGAATTACCT TTAGTTAAAA AATACTTTCA   
  
  
- CATGCTAGCA GAAGAGATAG GTGACACGCT CAAAAAGAGT GGTTTATGTA TACTTTTACC GTACTAAGTG   
  
  
- GTACGTTCTT TCCCTAAACC TCTTATTAAC CAATATTGTC CAGTACAAAC GGATCGTCGA AGGAAGATGA   
  
  
- GGTAATTAAA CCTGAACACG TAACGTACGG AATATACCCA ACATAAAACA CCTATTTCCT TTTCCTGTTT   
  
  
- CCCACCCGAC CAATGCATCC GAAAAATCAA AATTACTTTG TAAAGGCAAT GACAAGTAAA AATTGTTTTT   
  
  
- ATTGTAAAAA TGAGATTTTT TTAGTGAGGA GCATGATAAG TAAATGTTGT GTAAAAACAG TAAAAATAAT   
  
  
- TTTGAGTTTC AAAAGTTCAA AAAAAGTAAT TAAAAGGAAA AATAAATAAA TAAAAGTCTT AATCCGTTTG   
  
  
- TTAATCGGTG TAATGAACTA GTTAGTTGAT ATACTGTAAC TATCTTAAGA TGAAACGTGA AACGTTGTAG   
  
  
- TTCAAAGGTG AAGTTGGTTG GTCTACCCTA AACGATTAAA ATCAGTAATT GATTTCTAAT AAGCTACAAC   
  
  
- TCATATTCCA AACAGCGAAT TTACTAATTC TCGTAAATAA TCACGTGGAT TAACAACGTG ATGAACTTCA   
  
  
- GCACATAATC TGGTTCTAGT ACGAGTATAT TGAGTACCAT CTAATCCTAC ACTGAATTAA CATCAAACAA   
  
  
- GTAAGTTTTA TAGATAAAAG AAAACCCAC

+     TCT-motif

| Site Name | Organism | Position | Strand | Matrix score. | sequence | function |
| --- | --- | --- | --- | --- | --- | --- |
| TCT-motif | Arabidopsis thaliana | 560 | - | 6 | TCTTAC | part of a light responsive element |

>007212   
+ TGGTGAGTAT TTCTTTTTTC CGGTGTAGAA GCAGGTCGAA GCACGAGAAA ACCAAAAGAA ACTAGCAAAT   
  
  
+ CACCACCCTA TTACGATCCC TTCTAGCGAT ATCATTCTCG AGGATACAAG AAATATAAGA AGGAGCATTA   
  
  
+ GCGAACTGCG AACATATGGA CACCGAAAGA GAGGTTATAG CCCGAAATAG CTAAAGGTGA TCAAAGTTTT   
  
  
+ GATGAGAAGA AAGAGATGTG GACTGCTGTG TGAGGTGGGA GAGTTTTAGT TTTTTTTTTT CTGGAAGGAA   
  
  
+ ATGAAGATCG ATGGGTGGAG TGCTTATAAC TTGTTTGAAA GGTGTAAGTT TTTTGGGATG GAAGGAGAAG   
  
  
+ AGAGTGGCCT CATTTATTTT GTTTTGTTTG GTTTGTACAA GCTAATCAGC TAAGAATTCT TGTTTAACTG   
  
  
+ GTTTTGTGTT CTAGTTTTTG GTTTCTTGGA ACTTTGGTGG TTGTTGAGTG AGAAAGTTGA GCAAGTGAGA   
  
  
+ GAGAGAGAGA GGTTTTGTTA GGGGTAGAAA GGTTAGGAGC GAAAAGGAAG TGAGAGAGAG AAAGAGCAGG   
  
  
+ TAAGACTAGT GAAAAGGTTG TGATATGAGA GAGAGAGAGA GAGAGGGAAT AAGGTTAAAT TTCACATCAA   
  
  
+ CCTTATTCGA ATAAGAGAGA CTATTTAGTA GGATAACTTA ATTCGGTCAC AAAATATGCA ATGGTGTGTG   
  
  
+ TATTGAGAGT TGTTTTTACG TGAGGTCGGA TAATAATCAA ACTTAATGGA AATCAATTTT TTATGAAAGT   
  
  
+ GTACGATCGT CTTCTCTATC CACTGTGCGA GTTTTTCTCA CCAAATACAT ATGAAAATGG CATGATTCAC   
  
  
+ CATGCAAGAA AGGGATTTGG AGAATAATTG GTTATAACAG GTCATGTTTG CCTAGCAGCT TCCTTCTACT   
  
  
+ CCATTAATTT GGACTTGTGC ATTGCATGCC TTATATGGGT TGTATTTTGT GGATAAAGGA AAAGGACAAA   
  
  
+ GGGTGGGCTG GTTACGTAGG CTTTTTAGTT TTAATGAAAC ATTTCCGTTA CTGTTCATTT TTAACAAAAA   
  
  
+ TAACATTTTT ACTCTAAAAA AATCACTCCT CGTACTATTC ATTTACAACA CATTTTTGTC ATTTTTATTA   
  
  
+ AAACTCAAAG TTTTCAAGTT TTTTTCATTA ATTTTCCTTT TTATTTATTT ATTTTCAGAA TTAGGCAAAC   
  
  
+ AATTAGCCAC ATTACTTGAT CAATCAACTA TATGACATTG ATAGAATTCT ACTTTGCACT TTGCAACATC   
  
  
+ AAGTTTCCAC TTCAACCAAC CAGATGGGAT TTGCTAATTT TAGTCATTAA CTAAAGATTA TTCGATGTTG   
  
  
+ AGTATAAGGT TTGTCGCTTA AATGATTAAG AGCATTTATT AGTGCACCTA ATTGTTGCAC TACTTGAAGT   
  
  
+ CGTGTATTAG ACCAAGATCA TGCTCATATA ACTCATGGTA GATTAGGATG TGACTTAATT GTAGTTTGTT   
  
  
+ CATTCAAAAT ATCTATTTTC TTTTGGGTG  

- ACCACTCATA AAGAAAAAAG GCCACATCTT CGTCCAGCTT CGTGCTCTTT TGGTTTTCTT TGATCGTTTA   
  
  
- GTGGTGGGAT AATGCTAGGG AAGATCGCTA TAGTAAGAGC TCCTATGTTC TTTATATTCT TCCTCGTAAT   
  
  
- CGCTTGACGC TTGTATACCT GTGGCTTTCT CTCCAATATC GGGCTTTATC GATTTCCACT AGTTTCAAAA   
  
  
- CTACTCTTCT TTCTCTACAC CTGACGACAC ACTCCACCCT CTCAAAATCA AAAAAAAAAA GACCTTCCTT   
  
  
- TACTTCTAGC TACCCACCTC ACGAATATTG AACAAACTTT CCACATTCAA AAAACCCTAC CTTCCTCTTC   
  
  
- TCTCACCGGA GTAAATAAAA CAAAACAAAC CAAACATGTT CGATTAGTCG ATTCTTAAGA ACAAATTGAC   
  
  
- CAAAACACAA GATCAAAAAC CAAAGAACCT TGAAACCACC AACAACTCAC TCTTTCAACT CGTTCACTCT   
  
  
- CTCTCTCTCT CCAAAACAAT CCCCATCTTT CCAATCCTCG CTTTTCCTTC ACTCTCTCTC TTTCTCGTCC   
  
  
- ATTCTGATCA CTTTTCCAAC ACTATACTCT CTCTCTCTCT CTCTCCCTTA TTCCAATTTA AAGTGTAGTT   
  
  
- GGAATAAGCT TATTCTCTCT GATAAATCAT CCTATTGAAT TAAGCCAGTG TTTTATACGT TACCACACAC   
  
  
- ATAACTCTCA ACAAAAATGC ACTCCAGCCT ATTATTAGTT TGAATTACCT TTAGTTAAAA AATACTTTCA   
  
  
- CATGCTAGCA GAAGAGATAG GTGACACGCT CAAAAAGAGT GGTTTATGTA TACTTTTACC GTACTAAGTG   
  
  
- GTACGTTCTT TCCCTAAACC TCTTATTAAC CAATATTGTC CAGTACAAAC GGATCGTCGA AGGAAGATGA   
  
  
- GGTAATTAAA CCTGAACACG TAACGTACGG AATATACCCA ACATAAAACA CCTATTTCCT TTTCCTGTTT   
  
  
- CCCACCCGAC CAATGCATCC GAAAAATCAA AATTACTTTG TAAAGGCAAT GACAAGTAAA AATTGTTTTT   
  
  
- ATTGTAAAAA TGAGATTTTT TTAGTGAGGA GCATGATAAG TAAATGTTGT GTAAAAACAG TAAAAATAAT   
  
  
- TTTGAGTTTC AAAAGTTCAA AAAAAGTAAT TAAAAGGAAA AATAAATAAA TAAAAGTCTT AATCCGTTTG   
  
  
- TTAATCGGTG TAATGAACTA GTTAGTTGAT ATACTGTAAC TATCTTAAGA TGAAACGTGA AACGTTGTAG   
  
  
- TTCAAAGGTG AAGTTGGTTG GTCTACCCTA AACGATTAAA ATCAGTAATT GATTTCTAAT AAGCTACAAC   
  
  
- TCATATTCCA AACAGCGAAT TTACTAATTC TCGTAAATAA TCACGTGGAT TAACAACGTG ATGAACTTCA   
  
  
- GCACATAATC TGGTTCTAGT ACGAGTATAT TGAGTACCAT CTAATCCTAC ACTGAATTAA CATCAAACAA   
  
  
- GTAAGTTTTA TAGATAAAAG AAAACCCAC

+     Unnamed\_\_4

| Site Name | Organism | Position | Strand | Matrix score. | sequence | function |
| --- | --- | --- | --- | --- | --- | --- |
| Unnamed\_\_4 | Petroselinum hortense | 248 | - | 4 | CTCC |  |
| Unnamed\_\_4 | Petroselinum hortense | 1076 | + | 4 | CTCC |  |
| Unnamed\_\_4 | Petroselinum hortense | 909 | + | 4 | CTCC |  |
| Unnamed\_\_4 | Petroselinum hortense | 859 | - | 4 | CTCC |  |
| Unnamed\_\_4 | Petroselinum hortense | 132 | - | 4 | CTCC |  |
| Unnamed\_\_4 | Petroselinum hortense | 526 | - | 4 | CTCC |  |
| Unnamed\_\_4 | Petroselinum hortense | 344 | - | 4 | CTCC |  |
| Unnamed\_\_4 | Petroselinum hortense | 297 | - | 4 | CTCC |  |

>007212   
+ TGGTGAGTAT TTCTTTTTTC CGGTGTAGAA GCAGGTCGAA GCACGAGAAA ACCAAAAGAA ACTAGCAAAT   
  
  
+ CACCACCCTA TTACGATCCC TTCTAGCGAT ATCATTCTCG AGGATACAAG AAATATAAGA AGGAGCATTA   
  
  
+ GCGAACTGCG AACATATGGA CACCGAAAGA GAGGTTATAG CCCGAAATAG CTAAAGGTGA TCAAAGTTTT   
  
  
+ GATGAGAAGA AAGAGATGTG GACTGCTGTG TGAGGTGGGA GAGTTTTAGT TTTTTTTTTT CTGGAAGGAA   
  
  
+ ATGAAGATCG ATGGGTGGAG TGCTTATAAC TTGTTTGAAA GGTGTAAGTT TTTTGGGATG GAAGGAGAAG   
  
  
+ AGAGTGGCCT CATTTATTTT GTTTTGTTTG GTTTGTACAA GCTAATCAGC TAAGAATTCT TGTTTAACTG   
  
  
+ GTTTTGTGTT CTAGTTTTTG GTTTCTTGGA ACTTTGGTGG TTGTTGAGTG AGAAAGTTGA GCAAGTGAGA   
  
  
+ GAGAGAGAGA GGTTTTGTTA GGGGTAGAAA GGTTAGGAGC GAAAAGGAAG TGAGAGAGAG AAAGAGCAGG   
  
  
+ TAAGACTAGT GAAAAGGTTG TGATATGAGA GAGAGAGAGA GAGAGGGAAT AAGGTTAAAT TTCACATCAA   
  
  
+ CCTTATTCGA ATAAGAGAGA CTATTTAGTA GGATAACTTA ATTCGGTCAC AAAATATGCA ATGGTGTGTG   
  
  
+ TATTGAGAGT TGTTTTTACG TGAGGTCGGA TAATAATCAA ACTTAATGGA AATCAATTTT TTATGAAAGT   
  
  
+ GTACGATCGT CTTCTCTATC CACTGTGCGA GTTTTTCTCA CCAAATACAT ATGAAAATGG CATGATTCAC   
  
  
+ CATGCAAGAA AGGGATTTGG AGAATAATTG GTTATAACAG GTCATGTTTG CCTAGCAGCT TCCTTCTACT   
  
  
+ CCATTAATTT GGACTTGTGC ATTGCATGCC TTATATGGGT TGTATTTTGT GGATAAAGGA AAAGGACAAA   
  
  
+ GGGTGGGCTG GTTACGTAGG CTTTTTAGTT TTAATGAAAC ATTTCCGTTA CTGTTCATTT TTAACAAAAA   
  
  
+ TAACATTTTT ACTCTAAAAA AATCACTCCT CGTACTATTC ATTTACAACA CATTTTTGTC ATTTTTATTA   
  
  
+ AAACTCAAAG TTTTCAAGTT TTTTTCATTA ATTTTCCTTT TTATTTATTT ATTTTCAGAA TTAGGCAAAC   
  
  
+ AATTAGCCAC ATTACTTGAT CAATCAACTA TATGACATTG ATAGAATTCT ACTTTGCACT TTGCAACATC   
  
  
+ AAGTTTCCAC TTCAACCAAC CAGATGGGAT TTGCTAATTT TAGTCATTAA CTAAAGATTA TTCGATGTTG   
  
  
+ AGTATAAGGT TTGTCGCTTA AATGATTAAG AGCATTTATT AGTGCACCTA ATTGTTGCAC TACTTGAAGT   
  
  
+ CGTGTATTAG ACCAAGATCA TGCTCATATA ACTCATGGTA GATTAGGATG TGACTTAATT GTAGTTTGTT   
  
  
+ CATTCAAAAT ATCTATTTTC TTTTGGGTG  

- ACCACTCATA AAGAAAAAAG GCCACATCTT CGTCCAGCTT CGTGCTCTTT TGGTTTTCTT TGATCGTTTA   
  
  
- GTGGTGGGAT AATGCTAGGG AAGATCGCTA TAGTAAGAGC TCCTATGTTC TTTATATTCT TCCTCGTAAT   
  
  
- CGCTTGACGC TTGTATACCT GTGGCTTTCT CTCCAATATC GGGCTTTATC GATTTCCACT AGTTTCAAAA   
  
  
- CTACTCTTCT TTCTCTACAC CTGACGACAC ACTCCACCCT CTCAAAATCA AAAAAAAAAA GACCTTCCTT   
  
  
- TACTTCTAGC TACCCACCTC ACGAATATTG AACAAACTTT CCACATTCAA AAAACCCTAC CTTCCTCTTC   
  
  
- TCTCACCGGA GTAAATAAAA CAAAACAAAC CAAACATGTT CGATTAGTCG ATTCTTAAGA ACAAATTGAC   
  
  
- CAAAACACAA GATCAAAAAC CAAAGAACCT TGAAACCACC AACAACTCAC TCTTTCAACT CGTTCACTCT   
  
  
- CTCTCTCTCT CCAAAACAAT CCCCATCTTT CCAATCCTCG CTTTTCCTTC ACTCTCTCTC TTTCTCGTCC   
  
  
- ATTCTGATCA CTTTTCCAAC ACTATACTCT CTCTCTCTCT CTCTCCCTTA TTCCAATTTA AAGTGTAGTT   
  
  
- GGAATAAGCT TATTCTCTCT GATAAATCAT CCTATTGAAT TAAGCCAGTG TTTTATACGT TACCACACAC   
  
  
- ATAACTCTCA ACAAAAATGC ACTCCAGCCT ATTATTAGTT TGAATTACCT TTAGTTAAAA AATACTTTCA   
  
  
- CATGCTAGCA GAAGAGATAG GTGACACGCT CAAAAAGAGT GGTTTATGTA TACTTTTACC GTACTAAGTG   
  
  
- GTACGTTCTT TCCCTAAACC TCTTATTAAC CAATATTGTC CAGTACAAAC GGATCGTCGA AGGAAGATGA   
  
  
- GGTAATTAAA CCTGAACACG TAACGTACGG AATATACCCA ACATAAAACA CCTATTTCCT TTTCCTGTTT   
  
  
- CCCACCCGAC CAATGCATCC GAAAAATCAA AATTACTTTG TAAAGGCAAT GACAAGTAAA AATTGTTTTT   
  
  
- ATTGTAAAAA TGAGATTTTT TTAGTGAGGA GCATGATAAG TAAATGTTGT GTAAAAACAG TAAAAATAAT   
  
  
- TTTGAGTTTC AAAAGTTCAA AAAAAGTAAT TAAAAGGAAA AATAAATAAA TAAAAGTCTT AATCCGTTTG   
  
  
- TTAATCGGTG TAATGAACTA GTTAGTTGAT ATACTGTAAC TATCTTAAGA TGAAACGTGA AACGTTGTAG   
  
  
- TTCAAAGGTG AAGTTGGTTG GTCTACCCTA AACGATTAAA ATCAGTAATT GATTTCTAAT AAGCTACAAC   
  
  
- TCATATTCCA AACAGCGAAT TTACTAATTC TCGTAAATAA TCACGTGGAT TAACAACGTG ATGAACTTCA   
  
  
- GCACATAATC TGGTTCTAGT ACGAGTATAT TGAGTACCAT CTAATCCTAC ACTGAATTAA CATCAAACAA   
  
  
- GTAAGTTTTA TAGATAAAAG AAAACCCAC

+     WRE3

| Site Name | Organism | Position | Strand | Matrix score. | sequence | function |
| --- | --- | --- | --- | --- | --- | --- |
| WRE3 | Pisum sativum | 243 | - | 6 | CCACCT |  |

>007212   
+ TGGTGAGTAT TTCTTTTTTC CGGTGTAGAA GCAGGTCGAA GCACGAGAAA ACCAAAAGAA ACTAGCAAAT   
  
  
+ CACCACCCTA TTACGATCCC TTCTAGCGAT ATCATTCTCG AGGATACAAG AAATATAAGA AGGAGCATTA   
  
  
+ GCGAACTGCG AACATATGGA CACCGAAAGA GAGGTTATAG CCCGAAATAG CTAAAGGTGA TCAAAGTTTT   
  
  
+ GATGAGAAGA AAGAGATGTG GACTGCTGTG TGAGGTGGGA GAGTTTTAGT TTTTTTTTTT CTGGAAGGAA   
  
  
+ ATGAAGATCG ATGGGTGGAG TGCTTATAAC TTGTTTGAAA GGTGTAAGTT TTTTGGGATG GAAGGAGAAG   
  
  
+ AGAGTGGCCT CATTTATTTT GTTTTGTTTG GTTTGTACAA GCTAATCAGC TAAGAATTCT TGTTTAACTG   
  
  
+ GTTTTGTGTT CTAGTTTTTG GTTTCTTGGA ACTTTGGTGG TTGTTGAGTG AGAAAGTTGA GCAAGTGAGA   
  
  
+ GAGAGAGAGA GGTTTTGTTA GGGGTAGAAA GGTTAGGAGC GAAAAGGAAG TGAGAGAGAG AAAGAGCAGG   
  
  
+ TAAGACTAGT GAAAAGGTTG TGATATGAGA GAGAGAGAGA GAGAGGGAAT AAGGTTAAAT TTCACATCAA   
  
  
+ CCTTATTCGA ATAAGAGAGA CTATTTAGTA GGATAACTTA ATTCGGTCAC AAAATATGCA ATGGTGTGTG   
  
  
+ TATTGAGAGT TGTTTTTACG TGAGGTCGGA TAATAATCAA ACTTAATGGA AATCAATTTT TTATGAAAGT   
  
  
+ GTACGATCGT CTTCTCTATC CACTGTGCGA GTTTTTCTCA CCAAATACAT ATGAAAATGG CATGATTCAC   
  
  
+ CATGCAAGAA AGGGATTTGG AGAATAATTG GTTATAACAG GTCATGTTTG CCTAGCAGCT TCCTTCTACT   
  
  
+ CCATTAATTT GGACTTGTGC ATTGCATGCC TTATATGGGT TGTATTTTGT GGATAAAGGA AAAGGACAAA   
  
  
+ GGGTGGGCTG GTTACGTAGG CTTTTTAGTT TTAATGAAAC ATTTCCGTTA CTGTTCATTT TTAACAAAAA   
  
  
+ TAACATTTTT ACTCTAAAAA AATCACTCCT CGTACTATTC ATTTACAACA CATTTTTGTC ATTTTTATTA   
  
  
+ AAACTCAAAG TTTTCAAGTT TTTTTCATTA ATTTTCCTTT TTATTTATTT ATTTTCAGAA TTAGGCAAAC   
  
  
+ AATTAGCCAC ATTACTTGAT CAATCAACTA TATGACATTG ATAGAATTCT ACTTTGCACT TTGCAACATC   
  
  
+ AAGTTTCCAC TTCAACCAAC CAGATGGGAT TTGCTAATTT TAGTCATTAA CTAAAGATTA TTCGATGTTG   
  
  
+ AGTATAAGGT TTGTCGCTTA AATGATTAAG AGCATTTATT AGTGCACCTA ATTGTTGCAC TACTTGAAGT   
  
  
+ CGTGTATTAG ACCAAGATCA TGCTCATATA ACTCATGGTA GATTAGGATG TGACTTAATT GTAGTTTGTT   
  
  
+ CATTCAAAAT ATCTATTTTC TTTTGGGTG  

- ACCACTCATA AAGAAAAAAG GCCACATCTT CGTCCAGCTT CGTGCTCTTT TGGTTTTCTT TGATCGTTTA   
  
  
- GTGGTGGGAT AATGCTAGGG AAGATCGCTA TAGTAAGAGC TCCTATGTTC TTTATATTCT TCCTCGTAAT   
  
  
- CGCTTGACGC TTGTATACCT GTGGCTTTCT CTCCAATATC GGGCTTTATC GATTTCCACT AGTTTCAAAA   
  
  
- CTACTCTTCT TTCTCTACAC CTGACGACAC ACTCCACCCT CTCAAAATCA AAAAAAAAAA GACCTTCCTT   
  
  
- TACTTCTAGC TACCCACCTC ACGAATATTG AACAAACTTT CCACATTCAA AAAACCCTAC CTTCCTCTTC   
  
  
- TCTCACCGGA GTAAATAAAA CAAAACAAAC CAAACATGTT CGATTAGTCG ATTCTTAAGA ACAAATTGAC   
  
  
- CAAAACACAA GATCAAAAAC CAAAGAACCT TGAAACCACC AACAACTCAC TCTTTCAACT CGTTCACTCT   
  
  
- CTCTCTCTCT CCAAAACAAT CCCCATCTTT CCAATCCTCG CTTTTCCTTC ACTCTCTCTC TTTCTCGTCC   
  
  
- ATTCTGATCA CTTTTCCAAC ACTATACTCT CTCTCTCTCT CTCTCCCTTA TTCCAATTTA AAGTGTAGTT   
  
  
- GGAATAAGCT TATTCTCTCT GATAAATCAT CCTATTGAAT TAAGCCAGTG TTTTATACGT TACCACACAC   
  
  
- ATAACTCTCA ACAAAAATGC ACTCCAGCCT ATTATTAGTT TGAATTACCT TTAGTTAAAA AATACTTTCA   
  
  
- CATGCTAGCA GAAGAGATAG GTGACACGCT CAAAAAGAGT GGTTTATGTA TACTTTTACC GTACTAAGTG   
  
  
- GTACGTTCTT TCCCTAAACC TCTTATTAAC CAATATTGTC CAGTACAAAC GGATCGTCGA AGGAAGATGA   
  
  
- GGTAATTAAA CCTGAACACG TAACGTACGG AATATACCCA ACATAAAACA CCTATTTCCT TTTCCTGTTT   
  
  
- CCCACCCGAC CAATGCATCC GAAAAATCAA AATTACTTTG TAAAGGCAAT GACAAGTAAA AATTGTTTTT   
  
  
- ATTGTAAAAA TGAGATTTTT TTAGTGAGGA GCATGATAAG TAAATGTTGT GTAAAAACAG TAAAAATAAT   
  
  
- TTTGAGTTTC AAAAGTTCAA AAAAAGTAAT TAAAAGGAAA AATAAATAAA TAAAAGTCTT AATCCGTTTG   
  
  
- TTAATCGGTG TAATGAACTA GTTAGTTGAT ATACTGTAAC TATCTTAAGA TGAAACGTGA AACGTTGTAG   
  
  
- TTCAAAGGTG AAGTTGGTTG GTCTACCCTA AACGATTAAA ATCAGTAATT GATTTCTAAT AAGCTACAAC   
  
  
- TCATATTCCA AACAGCGAAT TTACTAATTC TCGTAAATAA TCACGTGGAT TAACAACGTG ATGAACTTCA   
  
  
- GCACATAATC TGGTTCTAGT ACGAGTATAT TGAGTACCAT CTAATCCTAC ACTGAATTAA CATCAAACAA   
  
  
- GTAAGTTTTA TAGATAAAAG AAAACCCAC
