# Supplementary material for: In Silico Genome-Wide Analysis of Respiratory Burst Oxidase Homolog (RBOH) Family Genes in Five Fruit-Producing Trees, and Potential Functional Analysis on Lignification of Stone Cells in Chinese White Pear
Source: Cells. 2019 May 29;8(6):520. doi: 10.3390/cells8060520 (PMC6627160; doi:10.3390/cells8060520)
Supplement: Supplementary file 1 [file cells-08-00520-s001.zip › Supplementary Material/Spreadsheets S2. Numbers of cis-elements in promoter region of PbRBOHs.tar.zip!/PbRBOHG.html]

Content-Type: text/html; charset=ISO-8859-1


PlantCARE


Webmaster Firefox specific output  
To save the result:
click on the frame with the right mouse button and save the source code as a text file with extension .html  
REFERENCE:PlantCARE: a database of plant cis-acting regulatory elements and a portal to tools for in silico analysis of promoter sequences.  
Lescot, M., Déhais, P., Moreau, Y., De Moor, B., Rouzé ,P.,and Rombauts, S.  
Nucleic Acids Res., Database issue(2002), 30(1):325-327.   


---

>023445   
+ TGTAAGTGGT GAATTACCCT GTAAATAAGG CTTTCTTCTT CAACCTACGG TGTCCCGGGC TCAAACCTCC   
  
  
+ GCTCTCCATA CTTGTAGGAT AAATGAATTT AGCGTAAAAA TAAAAAGAGA AAACAAAATT GTTAAAAAAC   
  
  
+ AACTTAATAT CTAAAATACC AAAATAAATA ACTATACTGG GGTGGAAATT TTTACCAAAT TACCGTACCA   
  
  
+ ACCGCCTTAC CAATCATACC ATACCAAATT ATTACCAAAA ATTTGTACCA ATCGGTAATG GTACGGTATT   
  
  
+ TGTACCATAC CATACTATTT TGATACGGTA CTGGTATCCA AAACCAGTAC CGGTGGTATA CCATACCTAC   
  
  
+ CAATATTATT AATATTATAT TATGTATTTA TTCATATAAA TATTTGAGGG AAAAATTAAT AAAATTAATA   
  
  
+ ATAATTATTG TAGAAGTGTA AAAAATGTAA AAAAAAGTAT ACATTCACTC ATAGTGACAA GTTTTACAAC   
  
  
+ TTTCATGAAA ATGTCAGCTT CAAAATCCAA CGCTATAATC CATAAACCTT AAATTTATAT TTAACCGTCG   
  
  
+ ATTGTCATTT ACGCTTTATT CTTCTTATTG AATTTCATTT TTAAAATTTT CTTTTTTGAA AACATGATCA   
  
  
+ TTTGAAGGAT GTAAGAAGAT GAATGGTTTA GATCTTTGAT ATCATGTTTC GATATTTACG GTTAACTGAA   
  
  
+ ATATGAGTCG ATGTCATATA GTTTGTAGAA ACTTTATAAA CATCAAGCAA TATTTTTATT AACCATAAAA   
  
  
+ CTTTGAACAT AATATCAACG ATCCAAATCA TTCATCTTCG TACATCCTCT AATAGATCAT GTTTTCAAAA   
  
  
+ AAGAAAATCT GAAAAAAAAC AAAATTTGAT GAAAAAAATG AAACGTAAAT GTAAACAACA ATCAACGATT   
  
  
+ AAATTTTGAT CTATGATTTA TATGATTATA ATGGCGAATT TCAAAGTTAA GCTCCTCACA AAAGTGGTAA   
  
  
+ AGCTTGTCAT TACAAGCGTT TATAAATTTT TTCTATATTT TTAACACCTC TATATTAATT AAAAAATATT   
  
  
+ AGATTTTGGG CAAACTAAGT GGCGTAAAAC AAAGTGACAA TTACCATTGG CATACCATAA CAACCAAACT   
  
  
+ ATTTGGTATA TTGATATTTA GTATACCGAA AGTTTGGTAC GGTAATGATA ATAGATTTTG CCGTATCAAA   
  
  
+ TATTTGGTAT GGTAATTGGT AGAGAGGTTT TGGTACGGTA ATGTACCAAA ACCACCCCTG TATGCATAGC   
  
  
+ TAAAAACAAA AATAATGAAA TACAAGTAGC TAGTGATATT GTTCTCCTAT TATTAGACAA AGAACCACCA   
  
  
+ AACTAACTGA GCCTTCCATT GCTCTTCCCT CCTTCCACAC ACATTTGACC CTTTCAGCTT TGTCTTGTCT   
  
  
+ CCACAACCCT AACTCCGCCC TTCTTTCTCT ATTTTCCACA TCCATCTTTC CCTTCACCAT GACATGTAAA   
  
  
+ TTTCAGAGGT AAAAAAATTT CTTGGTGGC  

- ACATTCACCA CTTAATGGGA CATTTATTCC GAAAGAAGAA GTTGGATGCC ACAGGGCCCG AGTTTGGAGG   
  
  
- CGAGAGGTAT GAACATCCTA TTTACTTAAA TCGCATTTTT ATTTTTCTCT TTTGTTTTAA CAATTTTTTG   
  
  
- TTGAATTATA GATTTTATGG TTTTATTTAT TGATATGACC CCACCTTTAA AAATGGTTTA ATGGCATGGT   
  
  
- TGGCGGAATG GTTAGTATGG TATGGTTTAA TAATGGTTTT TAAACATGGT TAGCCATTAC CATGCCATAA   
  
  
- ACATGGTATG GTATGATAAA ACTATGCCAT GACCATAGGT TTTGGTCATG GCCACCATAT GGTATGGATG   
  
  
- GTTATAATAA TTATAATATA ATACATAAAT AAGTATATTT ATAAACTCCC TTTTTAATTA TTTTAATTAT   
  
  
- TATTAATAAC ATCTTCACAT TTTTTACATT TTTTTTCATA TGTAAGTGAG TATCACTGTT CAAAATGTTG   
  
  
- AAAGTACTTT TACAGTCGAA GTTTTAGGTT GCGATATTAG GTATTTGGAA TTTAAATATA AATTGGCAGC   
  
  
- TAACAGTAAA TGCGAAATAA GAAGAATAAC TTAAAGTAAA AATTTTAAAA GAAAAAACTT TTGTACTAGT   
  
  
- AAACTTCCTA CATTCTTCTA CTTACCAAAT CTAGAAACTA TAGTACAAAG CTATAAATGC CAATTGACTT   
  
  
- TATACTCAGC TACAGTATAT CAAACATCTT TGAAATATTT GTAGTTCGTT ATAAAAATAA TTGGTATTTT   
  
  
- GAAACTTGTA TTATAGTTGC TAGGTTTAGT AAGTAGAAGC ATGTAGGAGA TTATCTAGTA CAAAAGTTTT   
  
  
- TTCTTTTAGA CTTTTTTTTG TTTTAAACTA CTTTTTTTAC TTTGCATTTA CATTTGTTGT TAGTTGCTAA   
  
  
- TTTAAAACTA GATACTAAAT ATACTAATAT TACCGCTTAA AGTTTCAATT CGAGGAGTGT TTTCACCATT   
  
  
- TCGAACAGTA ATGTTCGCAA ATATTTAAAA AAGATATAAA AATTGTGGAG ATATAATTAA TTTTTTATAA   
  
  
- TCTAAAACCC GTTTGATTCA CCGCATTTTG TTTCACTGTT AATGGTAACC GTATGGTATT GTTGGTTTGA   
  
  
- TAAACCATAT AACTATAAAT CATATGGCTT TCAAACCATG CCATTACTAT TATCTAAAAC GGCATAGTTT   
  
  
- ATAAACCATA CCATTAACCA TCTCTCCAAA ACCATGCCAT TACATGGTTT TGGTGGGGAC ATACGTATCG   
  
  
- ATTTTTGTTT TTATTACTTT ATGTTCATCG ATCACTATAA CAAGAGGATA ATAATCTGTT TCTTGGTGGT   
  
  
- TTGATTGACT CGGAAGGTAA CGAGAAGGGA GGAAGGTGTG TGTAAACTGG GAAAGTCGAA ACAGAACAGA   
  
  
- GGTGTTGGGA TTGAGGCGGG AAGAAAGAGA TAAAAGGTGT AGGTAGAAAG GGAAGTGGTA CTGTACATTT   
  
  
- AAAGTCTCCA TTTTTTTAAA GAACCACCG

  
  
Motifs Found  

+   

| Site Name | Organism | Position | Strand | Matrix score. | sequence | function |
| --- | --- | --- | --- | --- | --- | --- |
|  | organism | 775 | - | 4 | motif\_sequence | short\_function |
|  | organism | 1231 | + | 4 | motif\_sequence | short\_function |
|  | organism | 810 | - | 4 | motif\_sequence | short\_function |
|  | organism | 804 | + | 4 | motif\_sequence | short\_function |
|  | organism | 72 | + | 4 | motif\_sequence | short\_function |
|  | organism | 645 | - | 4 | motif\_sequence | short\_function |
|  | organism | 1209 | - | 4 | motif\_sequence | short\_function |
|  | organism | 1352 | + | 4 | motif\_sequence | short\_function |

>023445   
+ TGTAAGTGGT GAATTACCCT GTAAATAAGG CTTTCTTCTT CAACCTACGG TGTCCCGGGC TCAAACCTCC   
  
  
+ GCTCTCCATA CTTGTAGGAT AAATGAATTT AGCGTAAAAA TAAAAAGAGA AAACAAAATT GTTAAAAAAC   
  
  
+ AACTTAATAT CTAAAATACC AAAATAAATA ACTATACTGG GGTGGAAATT TTTACCAAAT TACCGTACCA   
  
  
+ ACCGCCTTAC CAATCATACC ATACCAAATT ATTACCAAAA ATTTGTACCA ATCGGTAATG GTACGGTATT   
  
  
+ TGTACCATAC CATACTATTT TGATACGGTA CTGGTATCCA AAACCAGTAC CGGTGGTATA CCATACCTAC   
  
  
+ CAATATTATT AATATTATAT TATGTATTTA TTCATATAAA TATTTGAGGG AAAAATTAAT AAAATTAATA   
  
  
+ ATAATTATTG TAGAAGTGTA AAAAATGTAA AAAAAAGTAT ACATTCACTC ATAGTGACAA GTTTTACAAC   
  
  
+ TTTCATGAAA ATGTCAGCTT CAAAATCCAA CGCTATAATC CATAAACCTT AAATTTATAT TTAACCGTCG   
  
  
+ ATTGTCATTT ACGCTTTATT CTTCTTATTG AATTTCATTT TTAAAATTTT CTTTTTTGAA AACATGATCA   
  
  
+ TTTGAAGGAT GTAAGAAGAT GAATGGTTTA GATCTTTGAT ATCATGTTTC GATATTTACG GTTAACTGAA   
  
  
+ ATATGAGTCG ATGTCATATA GTTTGTAGAA ACTTTATAAA CATCAAGCAA TATTTTTATT AACCATAAAA   
  
  
+ CTTTGAACAT AATATCAACG ATCCAAATCA TTCATCTTCG TACATCCTCT AATAGATCAT GTTTTCAAAA   
  
  
+ AAGAAAATCT GAAAAAAAAC AAAATTTGAT GAAAAAAATG AAACGTAAAT GTAAACAACA ATCAACGATT   
  
  
+ AAATTTTGAT CTATGATTTA TATGATTATA ATGGCGAATT TCAAAGTTAA GCTCCTCACA AAAGTGGTAA   
  
  
+ AGCTTGTCAT TACAAGCGTT TATAAATTTT TTCTATATTT TTAACACCTC TATATTAATT AAAAAATATT   
  
  
+ AGATTTTGGG CAAACTAAGT GGCGTAAAAC AAAGTGACAA TTACCATTGG CATACCATAA CAACCAAACT   
  
  
+ ATTTGGTATA TTGATATTTA GTATACCGAA AGTTTGGTAC GGTAATGATA ATAGATTTTG CCGTATCAAA   
  
  
+ TATTTGGTAT GGTAATTGGT AGAGAGGTTT TGGTACGGTA ATGTACCAAA ACCACCCCTG TATGCATAGC   
  
  
+ TAAAAACAAA AATAATGAAA TACAAGTAGC TAGTGATATT GTTCTCCTAT TATTAGACAA AGAACCACCA   
  
  
+ AACTAACTGA GCCTTCCATT GCTCTTCCCT CCTTCCACAC ACATTTGACC CTTTCAGCTT TGTCTTGTCT   
  
  
+ CCACAACCCT AACTCCGCCC TTCTTTCTCT ATTTTCCACA TCCATCTTTC CCTTCACCAT GACATGTAAA   
  
  
+ TTTCAGAGGT AAAAAAATTT CTTGGTGGC  

- ACATTCACCA CTTAATGGGA CATTTATTCC GAAAGAAGAA GTTGGATGCC ACAGGGCCCG AGTTTGGAGG   
  
  
- CGAGAGGTAT GAACATCCTA TTTACTTAAA TCGCATTTTT ATTTTTCTCT TTTGTTTTAA CAATTTTTTG   
  
  
- TTGAATTATA GATTTTATGG TTTTATTTAT TGATATGACC CCACCTTTAA AAATGGTTTA ATGGCATGGT   
  
  
- TGGCGGAATG GTTAGTATGG TATGGTTTAA TAATGGTTTT TAAACATGGT TAGCCATTAC CATGCCATAA   
  
  
- ACATGGTATG GTATGATAAA ACTATGCCAT GACCATAGGT TTTGGTCATG GCCACCATAT GGTATGGATG   
  
  
- GTTATAATAA TTATAATATA ATACATAAAT AAGTATATTT ATAAACTCCC TTTTTAATTA TTTTAATTAT   
  
  
- TATTAATAAC ATCTTCACAT TTTTTACATT TTTTTTCATA TGTAAGTGAG TATCACTGTT CAAAATGTTG   
  
  
- AAAGTACTTT TACAGTCGAA GTTTTAGGTT GCGATATTAG GTATTTGGAA TTTAAATATA AATTGGCAGC   
  
  
- TAACAGTAAA TGCGAAATAA GAAGAATAAC TTAAAGTAAA AATTTTAAAA GAAAAAACTT TTGTACTAGT   
  
  
- AAACTTCCTA CATTCTTCTA CTTACCAAAT CTAGAAACTA TAGTACAAAG CTATAAATGC CAATTGACTT   
  
  
- TATACTCAGC TACAGTATAT CAAACATCTT TGAAATATTT GTAGTTCGTT ATAAAAATAA TTGGTATTTT   
  
  
- GAAACTTGTA TTATAGTTGC TAGGTTTAGT AAGTAGAAGC ATGTAGGAGA TTATCTAGTA CAAAAGTTTT   
  
  
- TTCTTTTAGA CTTTTTTTTG TTTTAAACTA CTTTTTTTAC TTTGCATTTA CATTTGTTGT TAGTTGCTAA   
  
  
- TTTAAAACTA GATACTAAAT ATACTAATAT TACCGCTTAA AGTTTCAATT CGAGGAGTGT TTTCACCATT   
  
  
- TCGAACAGTA ATGTTCGCAA ATATTTAAAA AAGATATAAA AATTGTGGAG ATATAATTAA TTTTTTATAA   
  
  
- TCTAAAACCC GTTTGATTCA CCGCATTTTG TTTCACTGTT AATGGTAACC GTATGGTATT GTTGGTTTGA   
  
  
- TAAACCATAT AACTATAAAT CATATGGCTT TCAAACCATG CCATTACTAT TATCTAAAAC GGCATAGTTT   
  
  
- ATAAACCATA CCATTAACCA TCTCTCCAAA ACCATGCCAT TACATGGTTT TGGTGGGGAC ATACGTATCG   
  
  
- ATTTTTGTTT TTATTACTTT ATGTTCATCG ATCACTATAA CAAGAGGATA ATAATCTGTT TCTTGGTGGT   
  
  
- TTGATTGACT CGGAAGGTAA CGAGAAGGGA GGAAGGTGTG TGTAAACTGG GAAAGTCGAA ACAGAACAGA   
  
  
- GGTGTTGGGA TTGAGGCGGG AAGAAAGAGA TAAAAGGTGT AGGTAGAAAG GGAAGTGGTA CTGTACATTT   
  
  
- AAAGTCTCCA TTTTTTTAAA GAACCACCG

+     AAGAA-motif

| Site Name | Organism | Position | Strand | Matrix score. | sequence | function |
| --- | --- | --- | --- | --- | --- | --- |
| AAGAA-motif | Avena sativa | 1421 | - | 7 | GAAAGAA |  |
| AAGAA-motif | Avena sativa | 1478 | + | 9 | gGTAAAGAAA |  |

>023445   
+ TGTAAGTGGT GAATTACCCT GTAAATAAGG CTTTCTTCTT CAACCTACGG TGTCCCGGGC TCAAACCTCC   
  
  
+ GCTCTCCATA CTTGTAGGAT AAATGAATTT AGCGTAAAAA TAAAAAGAGA AAACAAAATT GTTAAAAAAC   
  
  
+ AACTTAATAT CTAAAATACC AAAATAAATA ACTATACTGG GGTGGAAATT TTTACCAAAT TACCGTACCA   
  
  
+ ACCGCCTTAC CAATCATACC ATACCAAATT ATTACCAAAA ATTTGTACCA ATCGGTAATG GTACGGTATT   
  
  
+ TGTACCATAC CATACTATTT TGATACGGTA CTGGTATCCA AAACCAGTAC CGGTGGTATA CCATACCTAC   
  
  
+ CAATATTATT AATATTATAT TATGTATTTA TTCATATAAA TATTTGAGGG AAAAATTAAT AAAATTAATA   
  
  
+ ATAATTATTG TAGAAGTGTA AAAAATGTAA AAAAAAGTAT ACATTCACTC ATAGTGACAA GTTTTACAAC   
  
  
+ TTTCATGAAA ATGTCAGCTT CAAAATCCAA CGCTATAATC CATAAACCTT AAATTTATAT TTAACCGTCG   
  
  
+ ATTGTCATTT ACGCTTTATT CTTCTTATTG AATTTCATTT TTAAAATTTT CTTTTTTGAA AACATGATCA   
  
  
+ TTTGAAGGAT GTAAGAAGAT GAATGGTTTA GATCTTTGAT ATCATGTTTC GATATTTACG GTTAACTGAA   
  
  
+ ATATGAGTCG ATGTCATATA GTTTGTAGAA ACTTTATAAA CATCAAGCAA TATTTTTATT AACCATAAAA   
  
  
+ CTTTGAACAT AATATCAACG ATCCAAATCA TTCATCTTCG TACATCCTCT AATAGATCAT GTTTTCAAAA   
  
  
+ AAGAAAATCT GAAAAAAAAC AAAATTTGAT GAAAAAAATG AAACGTAAAT GTAAACAACA ATCAACGATT   
  
  
+ AAATTTTGAT CTATGATTTA TATGATTATA ATGGCGAATT TCAAAGTTAA GCTCCTCACA AAAGTGGTAA   
  
  
+ AGCTTGTCAT TACAAGCGTT TATAAATTTT TTCTATATTT TTAACACCTC TATATTAATT AAAAAATATT   
  
  
+ AGATTTTGGG CAAACTAAGT GGCGTAAAAC AAAGTGACAA TTACCATTGG CATACCATAA CAACCAAACT   
  
  
+ ATTTGGTATA TTGATATTTA GTATACCGAA AGTTTGGTAC GGTAATGATA ATAGATTTTG CCGTATCAAA   
  
  
+ TATTTGGTAT GGTAATTGGT AGAGAGGTTT TGGTACGGTA ATGTACCAAA ACCACCCCTG TATGCATAGC   
  
  
+ TAAAAACAAA AATAATGAAA TACAAGTAGC TAGTGATATT GTTCTCCTAT TATTAGACAA AGAACCACCA   
  
  
+ AACTAACTGA GCCTTCCATT GCTCTTCCCT CCTTCCACAC ACATTTGACC CTTTCAGCTT TGTCTTGTCT   
  
  
+ CCACAACCCT AACTCCGCCC TTCTTTCTCT ATTTTCCACA TCCATCTTTC CCTTCACCAT GACATGTAAA   
  
  
+ TTTCAGAGGT AAAAAAATTT CTTGGTGGC  

- ACATTCACCA CTTAATGGGA CATTTATTCC GAAAGAAGAA GTTGGATGCC ACAGGGCCCG AGTTTGGAGG   
  
  
- CGAGAGGTAT GAACATCCTA TTTACTTAAA TCGCATTTTT ATTTTTCTCT TTTGTTTTAA CAATTTTTTG   
  
  
- TTGAATTATA GATTTTATGG TTTTATTTAT TGATATGACC CCACCTTTAA AAATGGTTTA ATGGCATGGT   
  
  
- TGGCGGAATG GTTAGTATGG TATGGTTTAA TAATGGTTTT TAAACATGGT TAGCCATTAC CATGCCATAA   
  
  
- ACATGGTATG GTATGATAAA ACTATGCCAT GACCATAGGT TTTGGTCATG GCCACCATAT GGTATGGATG   
  
  
- GTTATAATAA TTATAATATA ATACATAAAT AAGTATATTT ATAAACTCCC TTTTTAATTA TTTTAATTAT   
  
  
- TATTAATAAC ATCTTCACAT TTTTTACATT TTTTTTCATA TGTAAGTGAG TATCACTGTT CAAAATGTTG   
  
  
- AAAGTACTTT TACAGTCGAA GTTTTAGGTT GCGATATTAG GTATTTGGAA TTTAAATATA AATTGGCAGC   
  
  
- TAACAGTAAA TGCGAAATAA GAAGAATAAC TTAAAGTAAA AATTTTAAAA GAAAAAACTT TTGTACTAGT   
  
  
- AAACTTCCTA CATTCTTCTA CTTACCAAAT CTAGAAACTA TAGTACAAAG CTATAAATGC CAATTGACTT   
  
  
- TATACTCAGC TACAGTATAT CAAACATCTT TGAAATATTT GTAGTTCGTT ATAAAAATAA TTGGTATTTT   
  
  
- GAAACTTGTA TTATAGTTGC TAGGTTTAGT AAGTAGAAGC ATGTAGGAGA TTATCTAGTA CAAAAGTTTT   
  
  
- TTCTTTTAGA CTTTTTTTTG TTTTAAACTA CTTTTTTTAC TTTGCATTTA CATTTGTTGT TAGTTGCTAA   
  
  
- TTTAAAACTA GATACTAAAT ATACTAATAT TACCGCTTAA AGTTTCAATT CGAGGAGTGT TTTCACCATT   
  
  
- TCGAACAGTA ATGTTCGCAA ATATTTAAAA AAGATATAAA AATTGTGGAG ATATAATTAA TTTTTTATAA   
  
  
- TCTAAAACCC GTTTGATTCA CCGCATTTTG TTTCACTGTT AATGGTAACC GTATGGTATT GTTGGTTTGA   
  
  
- TAAACCATAT AACTATAAAT CATATGGCTT TCAAACCATG CCATTACTAT TATCTAAAAC GGCATAGTTT   
  
  
- ATAAACCATA CCATTAACCA TCTCTCCAAA ACCATGCCAT TACATGGTTT TGGTGGGGAC ATACGTATCG   
  
  
- ATTTTTGTTT TTATTACTTT ATGTTCATCG ATCACTATAA CAAGAGGATA ATAATCTGTT TCTTGGTGGT   
  
  
- TTGATTGACT CGGAAGGTAA CGAGAAGGGA GGAAGGTGTG TGTAAACTGG GAAAGTCGAA ACAGAACAGA   
  
  
- GGTGTTGGGA TTGAGGCGGG AAGAAAGAGA TAAAAGGTGT AGGTAGAAAG GGAAGTGGTA CTGTACATTT   
  
  
- AAAGTCTCCA TTTTTTTAAA GAACCACCG

+     AC-I

| Site Name | Organism | Position | Strand | Matrix score. | sequence | function |
| --- | --- | --- | --- | --- | --- | --- |
| AC-I | Phaseolus vulgaris | 341 | + | 8.5 | (T/C)C(T/C)(C/T)ACC(T/C)ACC |  |

>023445   
+ TGTAAGTGGT GAATTACCCT GTAAATAAGG CTTTCTTCTT CAACCTACGG TGTCCCGGGC TCAAACCTCC   
  
  
+ GCTCTCCATA CTTGTAGGAT AAATGAATTT AGCGTAAAAA TAAAAAGAGA AAACAAAATT GTTAAAAAAC   
  
  
+ AACTTAATAT CTAAAATACC AAAATAAATA ACTATACTGG GGTGGAAATT TTTACCAAAT TACCGTACCA   
  
  
+ ACCGCCTTAC CAATCATACC ATACCAAATT ATTACCAAAA ATTTGTACCA ATCGGTAATG GTACGGTATT   
  
  
+ TGTACCATAC CATACTATTT TGATACGGTA CTGGTATCCA AAACCAGTAC CGGTGGTATA CCATACCTAC   
  
  
+ CAATATTATT AATATTATAT TATGTATTTA TTCATATAAA TATTTGAGGG AAAAATTAAT AAAATTAATA   
  
  
+ ATAATTATTG TAGAAGTGTA AAAAATGTAA AAAAAAGTAT ACATTCACTC ATAGTGACAA GTTTTACAAC   
  
  
+ TTTCATGAAA ATGTCAGCTT CAAAATCCAA CGCTATAATC CATAAACCTT AAATTTATAT TTAACCGTCG   
  
  
+ ATTGTCATTT ACGCTTTATT CTTCTTATTG AATTTCATTT TTAAAATTTT CTTTTTTGAA AACATGATCA   
  
  
+ TTTGAAGGAT GTAAGAAGAT GAATGGTTTA GATCTTTGAT ATCATGTTTC GATATTTACG GTTAACTGAA   
  
  
+ ATATGAGTCG ATGTCATATA GTTTGTAGAA ACTTTATAAA CATCAAGCAA TATTTTTATT AACCATAAAA   
  
  
+ CTTTGAACAT AATATCAACG ATCCAAATCA TTCATCTTCG TACATCCTCT AATAGATCAT GTTTTCAAAA   
  
  
+ AAGAAAATCT GAAAAAAAAC AAAATTTGAT GAAAAAAATG AAACGTAAAT GTAAACAACA ATCAACGATT   
  
  
+ AAATTTTGAT CTATGATTTA TATGATTATA ATGGCGAATT TCAAAGTTAA GCTCCTCACA AAAGTGGTAA   
  
  
+ AGCTTGTCAT TACAAGCGTT TATAAATTTT TTCTATATTT TTAACACCTC TATATTAATT AAAAAATATT   
  
  
+ AGATTTTGGG CAAACTAAGT GGCGTAAAAC AAAGTGACAA TTACCATTGG CATACCATAA CAACCAAACT   
  
  
+ ATTTGGTATA TTGATATTTA GTATACCGAA AGTTTGGTAC GGTAATGATA ATAGATTTTG CCGTATCAAA   
  
  
+ TATTTGGTAT GGTAATTGGT AGAGAGGTTT TGGTACGGTA ATGTACCAAA ACCACCCCTG TATGCATAGC   
  
  
+ TAAAAACAAA AATAATGAAA TACAAGTAGC TAGTGATATT GTTCTCCTAT TATTAGACAA AGAACCACCA   
  
  
+ AACTAACTGA GCCTTCCATT GCTCTTCCCT CCTTCCACAC ACATTTGACC CTTTCAGCTT TGTCTTGTCT   
  
  
+ CCACAACCCT AACTCCGCCC TTCTTTCTCT ATTTTCCACA TCCATCTTTC CCTTCACCAT GACATGTAAA   
  
  
+ TTTCAGAGGT AAAAAAATTT CTTGGTGGC  

- ACATTCACCA CTTAATGGGA CATTTATTCC GAAAGAAGAA GTTGGATGCC ACAGGGCCCG AGTTTGGAGG   
  
  
- CGAGAGGTAT GAACATCCTA TTTACTTAAA TCGCATTTTT ATTTTTCTCT TTTGTTTTAA CAATTTTTTG   
  
  
- TTGAATTATA GATTTTATGG TTTTATTTAT TGATATGACC CCACCTTTAA AAATGGTTTA ATGGCATGGT   
  
  
- TGGCGGAATG GTTAGTATGG TATGGTTTAA TAATGGTTTT TAAACATGGT TAGCCATTAC CATGCCATAA   
  
  
- ACATGGTATG GTATGATAAA ACTATGCCAT GACCATAGGT TTTGGTCATG GCCACCATAT GGTATGGATG   
  
  
- GTTATAATAA TTATAATATA ATACATAAAT AAGTATATTT ATAAACTCCC TTTTTAATTA TTTTAATTAT   
  
  
- TATTAATAAC ATCTTCACAT TTTTTACATT TTTTTTCATA TGTAAGTGAG TATCACTGTT CAAAATGTTG   
  
  
- AAAGTACTTT TACAGTCGAA GTTTTAGGTT GCGATATTAG GTATTTGGAA TTTAAATATA AATTGGCAGC   
  
  
- TAACAGTAAA TGCGAAATAA GAAGAATAAC TTAAAGTAAA AATTTTAAAA GAAAAAACTT TTGTACTAGT   
  
  
- AAACTTCCTA CATTCTTCTA CTTACCAAAT CTAGAAACTA TAGTACAAAG CTATAAATGC CAATTGACTT   
  
  
- TATACTCAGC TACAGTATAT CAAACATCTT TGAAATATTT GTAGTTCGTT ATAAAAATAA TTGGTATTTT   
  
  
- GAAACTTGTA TTATAGTTGC TAGGTTTAGT AAGTAGAAGC ATGTAGGAGA TTATCTAGTA CAAAAGTTTT   
  
  
- TTCTTTTAGA CTTTTTTTTG TTTTAAACTA CTTTTTTTAC TTTGCATTTA CATTTGTTGT TAGTTGCTAA   
  
  
- TTTAAAACTA GATACTAAAT ATACTAATAT TACCGCTTAA AGTTTCAATT CGAGGAGTGT TTTCACCATT   
  
  
- TCGAACAGTA ATGTTCGCAA ATATTTAAAA AAGATATAAA AATTGTGGAG ATATAATTAA TTTTTTATAA   
  
  
- TCTAAAACCC GTTTGATTCA CCGCATTTTG TTTCACTGTT AATGGTAACC GTATGGTATT GTTGGTTTGA   
  
  
- TAAACCATAT AACTATAAAT CATATGGCTT TCAAACCATG CCATTACTAT TATCTAAAAC GGCATAGTTT   
  
  
- ATAAACCATA CCATTAACCA TCTCTCCAAA ACCATGCCAT TACATGGTTT TGGTGGGGAC ATACGTATCG   
  
  
- ATTTTTGTTT TTATTACTTT ATGTTCATCG ATCACTATAA CAAGAGGATA ATAATCTGTT TCTTGGTGGT   
  
  
- TTGATTGACT CGGAAGGTAA CGAGAAGGGA GGAAGGTGTG TGTAAACTGG GAAAGTCGAA ACAGAACAGA   
  
  
- GGTGTTGGGA TTGAGGCGGG AAGAAAGAGA TAAAAGGTGT AGGTAGAAAG GGAAGTGGTA CTGTACATTT   
  
  
- AAAGTCTCCA TTTTTTTAAA GAACCACCG

+     AE-box

| Site Name | Organism | Position | Strand | Matrix score. | sequence | function |
| --- | --- | --- | --- | --- | --- | --- |
| AE-box | Arabidopsis thaliana | 727 | + | 8 | AGAAACTT | part of a module for light response |

>023445   
+ TGTAAGTGGT GAATTACCCT GTAAATAAGG CTTTCTTCTT CAACCTACGG TGTCCCGGGC TCAAACCTCC   
  
  
+ GCTCTCCATA CTTGTAGGAT AAATGAATTT AGCGTAAAAA TAAAAAGAGA AAACAAAATT GTTAAAAAAC   
  
  
+ AACTTAATAT CTAAAATACC AAAATAAATA ACTATACTGG GGTGGAAATT TTTACCAAAT TACCGTACCA   
  
  
+ ACCGCCTTAC CAATCATACC ATACCAAATT ATTACCAAAA ATTTGTACCA ATCGGTAATG GTACGGTATT   
  
  
+ TGTACCATAC CATACTATTT TGATACGGTA CTGGTATCCA AAACCAGTAC CGGTGGTATA CCATACCTAC   
  
  
+ CAATATTATT AATATTATAT TATGTATTTA TTCATATAAA TATTTGAGGG AAAAATTAAT AAAATTAATA   
  
  
+ ATAATTATTG TAGAAGTGTA AAAAATGTAA AAAAAAGTAT ACATTCACTC ATAGTGACAA GTTTTACAAC   
  
  
+ TTTCATGAAA ATGTCAGCTT CAAAATCCAA CGCTATAATC CATAAACCTT AAATTTATAT TTAACCGTCG   
  
  
+ ATTGTCATTT ACGCTTTATT CTTCTTATTG AATTTCATTT TTAAAATTTT CTTTTTTGAA AACATGATCA   
  
  
+ TTTGAAGGAT GTAAGAAGAT GAATGGTTTA GATCTTTGAT ATCATGTTTC GATATTTACG GTTAACTGAA   
  
  
+ ATATGAGTCG ATGTCATATA GTTTGTAGAA ACTTTATAAA CATCAAGCAA TATTTTTATT AACCATAAAA   
  
  
+ CTTTGAACAT AATATCAACG ATCCAAATCA TTCATCTTCG TACATCCTCT AATAGATCAT GTTTTCAAAA   
  
  
+ AAGAAAATCT GAAAAAAAAC AAAATTTGAT GAAAAAAATG AAACGTAAAT GTAAACAACA ATCAACGATT   
  
  
+ AAATTTTGAT CTATGATTTA TATGATTATA ATGGCGAATT TCAAAGTTAA GCTCCTCACA AAAGTGGTAA   
  
  
+ AGCTTGTCAT TACAAGCGTT TATAAATTTT TTCTATATTT TTAACACCTC TATATTAATT AAAAAATATT   
  
  
+ AGATTTTGGG CAAACTAAGT GGCGTAAAAC AAAGTGACAA TTACCATTGG CATACCATAA CAACCAAACT   
  
  
+ ATTTGGTATA TTGATATTTA GTATACCGAA AGTTTGGTAC GGTAATGATA ATAGATTTTG CCGTATCAAA   
  
  
+ TATTTGGTAT GGTAATTGGT AGAGAGGTTT TGGTACGGTA ATGTACCAAA ACCACCCCTG TATGCATAGC   
  
  
+ TAAAAACAAA AATAATGAAA TACAAGTAGC TAGTGATATT GTTCTCCTAT TATTAGACAA AGAACCACCA   
  
  
+ AACTAACTGA GCCTTCCATT GCTCTTCCCT CCTTCCACAC ACATTTGACC CTTTCAGCTT TGTCTTGTCT   
  
  
+ CCACAACCCT AACTCCGCCC TTCTTTCTCT ATTTTCCACA TCCATCTTTC CCTTCACCAT GACATGTAAA   
  
  
+ TTTCAGAGGT AAAAAAATTT CTTGGTGGC  

- ACATTCACCA CTTAATGGGA CATTTATTCC GAAAGAAGAA GTTGGATGCC ACAGGGCCCG AGTTTGGAGG   
  
  
- CGAGAGGTAT GAACATCCTA TTTACTTAAA TCGCATTTTT ATTTTTCTCT TTTGTTTTAA CAATTTTTTG   
  
  
- TTGAATTATA GATTTTATGG TTTTATTTAT TGATATGACC CCACCTTTAA AAATGGTTTA ATGGCATGGT   
  
  
- TGGCGGAATG GTTAGTATGG TATGGTTTAA TAATGGTTTT TAAACATGGT TAGCCATTAC CATGCCATAA   
  
  
- ACATGGTATG GTATGATAAA ACTATGCCAT GACCATAGGT TTTGGTCATG GCCACCATAT GGTATGGATG   
  
  
- GTTATAATAA TTATAATATA ATACATAAAT AAGTATATTT ATAAACTCCC TTTTTAATTA TTTTAATTAT   
  
  
- TATTAATAAC ATCTTCACAT TTTTTACATT TTTTTTCATA TGTAAGTGAG TATCACTGTT CAAAATGTTG   
  
  
- AAAGTACTTT TACAGTCGAA GTTTTAGGTT GCGATATTAG GTATTTGGAA TTTAAATATA AATTGGCAGC   
  
  
- TAACAGTAAA TGCGAAATAA GAAGAATAAC TTAAAGTAAA AATTTTAAAA GAAAAAACTT TTGTACTAGT   
  
  
- AAACTTCCTA CATTCTTCTA CTTACCAAAT CTAGAAACTA TAGTACAAAG CTATAAATGC CAATTGACTT   
  
  
- TATACTCAGC TACAGTATAT CAAACATCTT TGAAATATTT GTAGTTCGTT ATAAAAATAA TTGGTATTTT   
  
  
- GAAACTTGTA TTATAGTTGC TAGGTTTAGT AAGTAGAAGC ATGTAGGAGA TTATCTAGTA CAAAAGTTTT   
  
  
- TTCTTTTAGA CTTTTTTTTG TTTTAAACTA CTTTTTTTAC TTTGCATTTA CATTTGTTGT TAGTTGCTAA   
  
  
- TTTAAAACTA GATACTAAAT ATACTAATAT TACCGCTTAA AGTTTCAATT CGAGGAGTGT TTTCACCATT   
  
  
- TCGAACAGTA ATGTTCGCAA ATATTTAAAA AAGATATAAA AATTGTGGAG ATATAATTAA TTTTTTATAA   
  
  
- TCTAAAACCC GTTTGATTCA CCGCATTTTG TTTCACTGTT AATGGTAACC GTATGGTATT GTTGGTTTGA   
  
  
- TAAACCATAT AACTATAAAT CATATGGCTT TCAAACCATG CCATTACTAT TATCTAAAAC GGCATAGTTT   
  
  
- ATAAACCATA CCATTAACCA TCTCTCCAAA ACCATGCCAT TACATGGTTT TGGTGGGGAC ATACGTATCG   
  
  
- ATTTTTGTTT TTATTACTTT ATGTTCATCG ATCACTATAA CAAGAGGATA ATAATCTGTT TCTTGGTGGT   
  
  
- TTGATTGACT CGGAAGGTAA CGAGAAGGGA GGAAGGTGTG TGTAAACTGG GAAAGTCGAA ACAGAACAGA   
  
  
- GGTGTTGGGA TTGAGGCGGG AAGAAAGAGA TAAAAGGTGT AGGTAGAAAG GGAAGTGGTA CTGTACATTT   
  
  
- AAAGTCTCCA TTTTTTTAAA GAACCACCG

+     ARE

| Site Name | Organism | Position | Strand | Matrix score. | sequence | function |
| --- | --- | --- | --- | --- | --- | --- |
| ARE | Zea mays | 654 | - | 6 | AAACCA | cis-acting regulatory element essential for the anaerobic induction |
| ARE | Zea mays | 1239 | + | 6 | AAACCA | cis-acting regulatory element essential for the anaerobic induction |
| ARE | Zea mays | 321 | + | 6 | AAACCA | cis-acting regulatory element essential for the anaerobic induction |

>023445   
+ TGTAAGTGGT GAATTACCCT GTAAATAAGG CTTTCTTCTT CAACCTACGG TGTCCCGGGC TCAAACCTCC   
  
  
+ GCTCTCCATA CTTGTAGGAT AAATGAATTT AGCGTAAAAA TAAAAAGAGA AAACAAAATT GTTAAAAAAC   
  
  
+ AACTTAATAT CTAAAATACC AAAATAAATA ACTATACTGG GGTGGAAATT TTTACCAAAT TACCGTACCA   
  
  
+ ACCGCCTTAC CAATCATACC ATACCAAATT ATTACCAAAA ATTTGTACCA ATCGGTAATG GTACGGTATT   
  
  
+ TGTACCATAC CATACTATTT TGATACGGTA CTGGTATCCA AAACCAGTAC CGGTGGTATA CCATACCTAC   
  
  
+ CAATATTATT AATATTATAT TATGTATTTA TTCATATAAA TATTTGAGGG AAAAATTAAT AAAATTAATA   
  
  
+ ATAATTATTG TAGAAGTGTA AAAAATGTAA AAAAAAGTAT ACATTCACTC ATAGTGACAA GTTTTACAAC   
  
  
+ TTTCATGAAA ATGTCAGCTT CAAAATCCAA CGCTATAATC CATAAACCTT AAATTTATAT TTAACCGTCG   
  
  
+ ATTGTCATTT ACGCTTTATT CTTCTTATTG AATTTCATTT TTAAAATTTT CTTTTTTGAA AACATGATCA   
  
  
+ TTTGAAGGAT GTAAGAAGAT GAATGGTTTA GATCTTTGAT ATCATGTTTC GATATTTACG GTTAACTGAA   
  
  
+ ATATGAGTCG ATGTCATATA GTTTGTAGAA ACTTTATAAA CATCAAGCAA TATTTTTATT AACCATAAAA   
  
  
+ CTTTGAACAT AATATCAACG ATCCAAATCA TTCATCTTCG TACATCCTCT AATAGATCAT GTTTTCAAAA   
  
  
+ AAGAAAATCT GAAAAAAAAC AAAATTTGAT GAAAAAAATG AAACGTAAAT GTAAACAACA ATCAACGATT   
  
  
+ AAATTTTGAT CTATGATTTA TATGATTATA ATGGCGAATT TCAAAGTTAA GCTCCTCACA AAAGTGGTAA   
  
  
+ AGCTTGTCAT TACAAGCGTT TATAAATTTT TTCTATATTT TTAACACCTC TATATTAATT AAAAAATATT   
  
  
+ AGATTTTGGG CAAACTAAGT GGCGTAAAAC AAAGTGACAA TTACCATTGG CATACCATAA CAACCAAACT   
  
  
+ ATTTGGTATA TTGATATTTA GTATACCGAA AGTTTGGTAC GGTAATGATA ATAGATTTTG CCGTATCAAA   
  
  
+ TATTTGGTAT GGTAATTGGT AGAGAGGTTT TGGTACGGTA ATGTACCAAA ACCACCCCTG TATGCATAGC   
  
  
+ TAAAAACAAA AATAATGAAA TACAAGTAGC TAGTGATATT GTTCTCCTAT TATTAGACAA AGAACCACCA   
  
  
+ AACTAACTGA GCCTTCCATT GCTCTTCCCT CCTTCCACAC ACATTTGACC CTTTCAGCTT TGTCTTGTCT   
  
  
+ CCACAACCCT AACTCCGCCC TTCTTTCTCT ATTTTCCACA TCCATCTTTC CCTTCACCAT GACATGTAAA   
  
  
+ TTTCAGAGGT AAAAAAATTT CTTGGTGGC  

- ACATTCACCA CTTAATGGGA CATTTATTCC GAAAGAAGAA GTTGGATGCC ACAGGGCCCG AGTTTGGAGG   
  
  
- CGAGAGGTAT GAACATCCTA TTTACTTAAA TCGCATTTTT ATTTTTCTCT TTTGTTTTAA CAATTTTTTG   
  
  
- TTGAATTATA GATTTTATGG TTTTATTTAT TGATATGACC CCACCTTTAA AAATGGTTTA ATGGCATGGT   
  
  
- TGGCGGAATG GTTAGTATGG TATGGTTTAA TAATGGTTTT TAAACATGGT TAGCCATTAC CATGCCATAA   
  
  
- ACATGGTATG GTATGATAAA ACTATGCCAT GACCATAGGT TTTGGTCATG GCCACCATAT GGTATGGATG   
  
  
- GTTATAATAA TTATAATATA ATACATAAAT AAGTATATTT ATAAACTCCC TTTTTAATTA TTTTAATTAT   
  
  
- TATTAATAAC ATCTTCACAT TTTTTACATT TTTTTTCATA TGTAAGTGAG TATCACTGTT CAAAATGTTG   
  
  
- AAAGTACTTT TACAGTCGAA GTTTTAGGTT GCGATATTAG GTATTTGGAA TTTAAATATA AATTGGCAGC   
  
  
- TAACAGTAAA TGCGAAATAA GAAGAATAAC TTAAAGTAAA AATTTTAAAA GAAAAAACTT TTGTACTAGT   
  
  
- AAACTTCCTA CATTCTTCTA CTTACCAAAT CTAGAAACTA TAGTACAAAG CTATAAATGC CAATTGACTT   
  
  
- TATACTCAGC TACAGTATAT CAAACATCTT TGAAATATTT GTAGTTCGTT ATAAAAATAA TTGGTATTTT   
  
  
- GAAACTTGTA TTATAGTTGC TAGGTTTAGT AAGTAGAAGC ATGTAGGAGA TTATCTAGTA CAAAAGTTTT   
  
  
- TTCTTTTAGA CTTTTTTTTG TTTTAAACTA CTTTTTTTAC TTTGCATTTA CATTTGTTGT TAGTTGCTAA   
  
  
- TTTAAAACTA GATACTAAAT ATACTAATAT TACCGCTTAA AGTTTCAATT CGAGGAGTGT TTTCACCATT   
  
  
- TCGAACAGTA ATGTTCGCAA ATATTTAAAA AAGATATAAA AATTGTGGAG ATATAATTAA TTTTTTATAA   
  
  
- TCTAAAACCC GTTTGATTCA CCGCATTTTG TTTCACTGTT AATGGTAACC GTATGGTATT GTTGGTTTGA   
  
  
- TAAACCATAT AACTATAAAT CATATGGCTT TCAAACCATG CCATTACTAT TATCTAAAAC GGCATAGTTT   
  
  
- ATAAACCATA CCATTAACCA TCTCTCCAAA ACCATGCCAT TACATGGTTT TGGTGGGGAC ATACGTATCG   
  
  
- ATTTTTGTTT TTATTACTTT ATGTTCATCG ATCACTATAA CAAGAGGATA ATAATCTGTT TCTTGGTGGT   
  
  
- TTGATTGACT CGGAAGGTAA CGAGAAGGGA GGAAGGTGTG TGTAAACTGG GAAAGTCGAA ACAGAACAGA   
  
  
- GGTGTTGGGA TTGAGGCGGG AAGAAAGAGA TAAAAGGTGT AGGTAGAAAG GGAAGTGGTA CTGTACATTT   
  
  
- AAAGTCTCCA TTTTTTTAAA GAACCACCG

+     Box 4

| Site Name | Organism | Position | Strand | Matrix score. | sequence | function |
| --- | --- | --- | --- | --- | --- | --- |
| Box 4 | Petroselinum crispum | 414 | + | 6 | ATTAAT | part of a conserved DNA module involved in light responsiveness |
| Box 4 | Petroselinum crispum | 405 | + | 6 | ATTAAT | part of a conserved DNA module involved in light responsiveness |
| Box 4 | Petroselinum crispum | 1034 | - | 6 | ATTAAT | part of a conserved DNA module involved in light responsiveness |
| Box 4 | Petroselinum crispum | 358 | + | 6 | ATTAAT | part of a conserved DNA module involved in light responsiveness |

>023445   
+ TGTAAGTGGT GAATTACCCT GTAAATAAGG CTTTCTTCTT CAACCTACGG TGTCCCGGGC TCAAACCTCC   
  
  
+ GCTCTCCATA CTTGTAGGAT AAATGAATTT AGCGTAAAAA TAAAAAGAGA AAACAAAATT GTTAAAAAAC   
  
  
+ AACTTAATAT CTAAAATACC AAAATAAATA ACTATACTGG GGTGGAAATT TTTACCAAAT TACCGTACCA   
  
  
+ ACCGCCTTAC CAATCATACC ATACCAAATT ATTACCAAAA ATTTGTACCA ATCGGTAATG GTACGGTATT   
  
  
+ TGTACCATAC CATACTATTT TGATACGGTA CTGGTATCCA AAACCAGTAC CGGTGGTATA CCATACCTAC   
  
  
+ CAATATTATT AATATTATAT TATGTATTTA TTCATATAAA TATTTGAGGG AAAAATTAAT AAAATTAATA   
  
  
+ ATAATTATTG TAGAAGTGTA AAAAATGTAA AAAAAAGTAT ACATTCACTC ATAGTGACAA GTTTTACAAC   
  
  
+ TTTCATGAAA ATGTCAGCTT CAAAATCCAA CGCTATAATC CATAAACCTT AAATTTATAT TTAACCGTCG   
  
  
+ ATTGTCATTT ACGCTTTATT CTTCTTATTG AATTTCATTT TTAAAATTTT CTTTTTTGAA AACATGATCA   
  
  
+ TTTGAAGGAT GTAAGAAGAT GAATGGTTTA GATCTTTGAT ATCATGTTTC GATATTTACG GTTAACTGAA   
  
  
+ ATATGAGTCG ATGTCATATA GTTTGTAGAA ACTTTATAAA CATCAAGCAA TATTTTTATT AACCATAAAA   
  
  
+ CTTTGAACAT AATATCAACG ATCCAAATCA TTCATCTTCG TACATCCTCT AATAGATCAT GTTTTCAAAA   
  
  
+ AAGAAAATCT GAAAAAAAAC AAAATTTGAT GAAAAAAATG AAACGTAAAT GTAAACAACA ATCAACGATT   
  
  
+ AAATTTTGAT CTATGATTTA TATGATTATA ATGGCGAATT TCAAAGTTAA GCTCCTCACA AAAGTGGTAA   
  
  
+ AGCTTGTCAT TACAAGCGTT TATAAATTTT TTCTATATTT TTAACACCTC TATATTAATT AAAAAATATT   
  
  
+ AGATTTTGGG CAAACTAAGT GGCGTAAAAC AAAGTGACAA TTACCATTGG CATACCATAA CAACCAAACT   
  
  
+ ATTTGGTATA TTGATATTTA GTATACCGAA AGTTTGGTAC GGTAATGATA ATAGATTTTG CCGTATCAAA   
  
  
+ TATTTGGTAT GGTAATTGGT AGAGAGGTTT TGGTACGGTA ATGTACCAAA ACCACCCCTG TATGCATAGC   
  
  
+ TAAAAACAAA AATAATGAAA TACAAGTAGC TAGTGATATT GTTCTCCTAT TATTAGACAA AGAACCACCA   
  
  
+ AACTAACTGA GCCTTCCATT GCTCTTCCCT CCTTCCACAC ACATTTGACC CTTTCAGCTT TGTCTTGTCT   
  
  
+ CCACAACCCT AACTCCGCCC TTCTTTCTCT ATTTTCCACA TCCATCTTTC CCTTCACCAT GACATGTAAA   
  
  
+ TTTCAGAGGT AAAAAAATTT CTTGGTGGC  

- ACATTCACCA CTTAATGGGA CATTTATTCC GAAAGAAGAA GTTGGATGCC ACAGGGCCCG AGTTTGGAGG   
  
  
- CGAGAGGTAT GAACATCCTA TTTACTTAAA TCGCATTTTT ATTTTTCTCT TTTGTTTTAA CAATTTTTTG   
  
  
- TTGAATTATA GATTTTATGG TTTTATTTAT TGATATGACC CCACCTTTAA AAATGGTTTA ATGGCATGGT   
  
  
- TGGCGGAATG GTTAGTATGG TATGGTTTAA TAATGGTTTT TAAACATGGT TAGCCATTAC CATGCCATAA   
  
  
- ACATGGTATG GTATGATAAA ACTATGCCAT GACCATAGGT TTTGGTCATG GCCACCATAT GGTATGGATG   
  
  
- GTTATAATAA TTATAATATA ATACATAAAT AAGTATATTT ATAAACTCCC TTTTTAATTA TTTTAATTAT   
  
  
- TATTAATAAC ATCTTCACAT TTTTTACATT TTTTTTCATA TGTAAGTGAG TATCACTGTT CAAAATGTTG   
  
  
- AAAGTACTTT TACAGTCGAA GTTTTAGGTT GCGATATTAG GTATTTGGAA TTTAAATATA AATTGGCAGC   
  
  
- TAACAGTAAA TGCGAAATAA GAAGAATAAC TTAAAGTAAA AATTTTAAAA GAAAAAACTT TTGTACTAGT   
  
  
- AAACTTCCTA CATTCTTCTA CTTACCAAAT CTAGAAACTA TAGTACAAAG CTATAAATGC CAATTGACTT   
  
  
- TATACTCAGC TACAGTATAT CAAACATCTT TGAAATATTT GTAGTTCGTT ATAAAAATAA TTGGTATTTT   
  
  
- GAAACTTGTA TTATAGTTGC TAGGTTTAGT AAGTAGAAGC ATGTAGGAGA TTATCTAGTA CAAAAGTTTT   
  
  
- TTCTTTTAGA CTTTTTTTTG TTTTAAACTA CTTTTTTTAC TTTGCATTTA CATTTGTTGT TAGTTGCTAA   
  
  
- TTTAAAACTA GATACTAAAT ATACTAATAT TACCGCTTAA AGTTTCAATT CGAGGAGTGT TTTCACCATT   
  
  
- TCGAACAGTA ATGTTCGCAA ATATTTAAAA AAGATATAAA AATTGTGGAG ATATAATTAA TTTTTTATAA   
  
  
- TCTAAAACCC GTTTGATTCA CCGCATTTTG TTTCACTGTT AATGGTAACC GTATGGTATT GTTGGTTTGA   
  
  
- TAAACCATAT AACTATAAAT CATATGGCTT TCAAACCATG CCATTACTAT TATCTAAAAC GGCATAGTTT   
  
  
- ATAAACCATA CCATTAACCA TCTCTCCAAA ACCATGCCAT TACATGGTTT TGGTGGGGAC ATACGTATCG   
  
  
- ATTTTTGTTT TTATTACTTT ATGTTCATCG ATCACTATAA CAAGAGGATA ATAATCTGTT TCTTGGTGGT   
  
  
- TTGATTGACT CGGAAGGTAA CGAGAAGGGA GGAAGGTGTG TGTAAACTGG GAAAGTCGAA ACAGAACAGA   
  
  
- GGTGTTGGGA TTGAGGCGGG AAGAAAGAGA TAAAAGGTGT AGGTAGAAAG GGAAGTGGTA CTGTACATTT   
  
  
- AAAGTCTCCA TTTTTTTAAA GAACCACCG

+     Box II

| Site Name | Organism | Position | Strand | Matrix score. | sequence | function |
| --- | --- | --- | --- | --- | --- | --- |
| Box II | Solanum tuberosum | 239 | - | 9 | TGGTAATAA | part of a light responsive element |

>023445   
+ TGTAAGTGGT GAATTACCCT GTAAATAAGG CTTTCTTCTT CAACCTACGG TGTCCCGGGC TCAAACCTCC   
  
  
+ GCTCTCCATA CTTGTAGGAT AAATGAATTT AGCGTAAAAA TAAAAAGAGA AAACAAAATT GTTAAAAAAC   
  
  
+ AACTTAATAT CTAAAATACC AAAATAAATA ACTATACTGG GGTGGAAATT TTTACCAAAT TACCGTACCA   
  
  
+ ACCGCCTTAC CAATCATACC ATACCAAATT ATTACCAAAA ATTTGTACCA ATCGGTAATG GTACGGTATT   
  
  
+ TGTACCATAC CATACTATTT TGATACGGTA CTGGTATCCA AAACCAGTAC CGGTGGTATA CCATACCTAC   
  
  
+ CAATATTATT AATATTATAT TATGTATTTA TTCATATAAA TATTTGAGGG AAAAATTAAT AAAATTAATA   
  
  
+ ATAATTATTG TAGAAGTGTA AAAAATGTAA AAAAAAGTAT ACATTCACTC ATAGTGACAA GTTTTACAAC   
  
  
+ TTTCATGAAA ATGTCAGCTT CAAAATCCAA CGCTATAATC CATAAACCTT AAATTTATAT TTAACCGTCG   
  
  
+ ATTGTCATTT ACGCTTTATT CTTCTTATTG AATTTCATTT TTAAAATTTT CTTTTTTGAA AACATGATCA   
  
  
+ TTTGAAGGAT GTAAGAAGAT GAATGGTTTA GATCTTTGAT ATCATGTTTC GATATTTACG GTTAACTGAA   
  
  
+ ATATGAGTCG ATGTCATATA GTTTGTAGAA ACTTTATAAA CATCAAGCAA TATTTTTATT AACCATAAAA   
  
  
+ CTTTGAACAT AATATCAACG ATCCAAATCA TTCATCTTCG TACATCCTCT AATAGATCAT GTTTTCAAAA   
  
  
+ AAGAAAATCT GAAAAAAAAC AAAATTTGAT GAAAAAAATG AAACGTAAAT GTAAACAACA ATCAACGATT   
  
  
+ AAATTTTGAT CTATGATTTA TATGATTATA ATGGCGAATT TCAAAGTTAA GCTCCTCACA AAAGTGGTAA   
  
  
+ AGCTTGTCAT TACAAGCGTT TATAAATTTT TTCTATATTT TTAACACCTC TATATTAATT AAAAAATATT   
  
  
+ AGATTTTGGG CAAACTAAGT GGCGTAAAAC AAAGTGACAA TTACCATTGG CATACCATAA CAACCAAACT   
  
  
+ ATTTGGTATA TTGATATTTA GTATACCGAA AGTTTGGTAC GGTAATGATA ATAGATTTTG CCGTATCAAA   
  
  
+ TATTTGGTAT GGTAATTGGT AGAGAGGTTT TGGTACGGTA ATGTACCAAA ACCACCCCTG TATGCATAGC   
  
  
+ TAAAAACAAA AATAATGAAA TACAAGTAGC TAGTGATATT GTTCTCCTAT TATTAGACAA AGAACCACCA   
  
  
+ AACTAACTGA GCCTTCCATT GCTCTTCCCT CCTTCCACAC ACATTTGACC CTTTCAGCTT TGTCTTGTCT   
  
  
+ CCACAACCCT AACTCCGCCC TTCTTTCTCT ATTTTCCACA TCCATCTTTC CCTTCACCAT GACATGTAAA   
  
  
+ TTTCAGAGGT AAAAAAATTT CTTGGTGGC  

- ACATTCACCA CTTAATGGGA CATTTATTCC GAAAGAAGAA GTTGGATGCC ACAGGGCCCG AGTTTGGAGG   
  
  
- CGAGAGGTAT GAACATCCTA TTTACTTAAA TCGCATTTTT ATTTTTCTCT TTTGTTTTAA CAATTTTTTG   
  
  
- TTGAATTATA GATTTTATGG TTTTATTTAT TGATATGACC CCACCTTTAA AAATGGTTTA ATGGCATGGT   
  
  
- TGGCGGAATG GTTAGTATGG TATGGTTTAA TAATGGTTTT TAAACATGGT TAGCCATTAC CATGCCATAA   
  
  
- ACATGGTATG GTATGATAAA ACTATGCCAT GACCATAGGT TTTGGTCATG GCCACCATAT GGTATGGATG   
  
  
- GTTATAATAA TTATAATATA ATACATAAAT AAGTATATTT ATAAACTCCC TTTTTAATTA TTTTAATTAT   
  
  
- TATTAATAAC ATCTTCACAT TTTTTACATT TTTTTTCATA TGTAAGTGAG TATCACTGTT CAAAATGTTG   
  
  
- AAAGTACTTT TACAGTCGAA GTTTTAGGTT GCGATATTAG GTATTTGGAA TTTAAATATA AATTGGCAGC   
  
  
- TAACAGTAAA TGCGAAATAA GAAGAATAAC TTAAAGTAAA AATTTTAAAA GAAAAAACTT TTGTACTAGT   
  
  
- AAACTTCCTA CATTCTTCTA CTTACCAAAT CTAGAAACTA TAGTACAAAG CTATAAATGC CAATTGACTT   
  
  
- TATACTCAGC TACAGTATAT CAAACATCTT TGAAATATTT GTAGTTCGTT ATAAAAATAA TTGGTATTTT   
  
  
- GAAACTTGTA TTATAGTTGC TAGGTTTAGT AAGTAGAAGC ATGTAGGAGA TTATCTAGTA CAAAAGTTTT   
  
  
- TTCTTTTAGA CTTTTTTTTG TTTTAAACTA CTTTTTTTAC TTTGCATTTA CATTTGTTGT TAGTTGCTAA   
  
  
- TTTAAAACTA GATACTAAAT ATACTAATAT TACCGCTTAA AGTTTCAATT CGAGGAGTGT TTTCACCATT   
  
  
- TCGAACAGTA ATGTTCGCAA ATATTTAAAA AAGATATAAA AATTGTGGAG ATATAATTAA TTTTTTATAA   
  
  
- TCTAAAACCC GTTTGATTCA CCGCATTTTG TTTCACTGTT AATGGTAACC GTATGGTATT GTTGGTTTGA   
  
  
- TAAACCATAT AACTATAAAT CATATGGCTT TCAAACCATG CCATTACTAT TATCTAAAAC GGCATAGTTT   
  
  
- ATAAACCATA CCATTAACCA TCTCTCCAAA ACCATGCCAT TACATGGTTT TGGTGGGGAC ATACGTATCG   
  
  
- ATTTTTGTTT TTATTACTTT ATGTTCATCG ATCACTATAA CAAGAGGATA ATAATCTGTT TCTTGGTGGT   
  
  
- TTGATTGACT CGGAAGGTAA CGAGAAGGGA GGAAGGTGTG TGTAAACTGG GAAAGTCGAA ACAGAACAGA   
  
  
- GGTGTTGGGA TTGAGGCGGG AAGAAAGAGA TAAAAGGTGT AGGTAGAAAG GGAAGTGGTA CTGTACATTT   
  
  
- AAAGTCTCCA TTTTTTTAAA GAACCACCG

+     CAAT-box

| Site Name | Organism | Position | Strand | Matrix score. | sequence | function |
| --- | --- | --- | --- | --- | --- | --- |
| CAAT-box | Nicotiana glutinosa | 748 | + | 4 | CAAT |  |
| CAAT-box | Pisum sativum | 794 | + | 5 | CAAAT | common cis-acting element in promoter and enhancer regions |
| CAAT-box | Pisum sativum | 864 | - | 5 | CAAAT | common cis-acting element in promoter and enhancer regions |
| CAAT-box | Nicotiana glutinosa | 899 | + | 4 | CAAT |  |
| CAAT-box | Pisum sativum | 630 | - | 5 | CAAAT | common cis-acting element in promoter and enhancer regions |
| CAAT-box | Nicotiana glutinosa | 1130 | - | 4 | CAAT |  |
| CAAT-box | Pisum sativum | 1121 | - | 5 | CAAAT | common cis-acting element in promoter and enhancer regions |
| CAAT-box | Pisum sativum | 1373 | - | 5 | CAAAT | common cis-acting element in promoter and enhancer regions |
| CAAT-box | Nicotiana glutinosa | 1348 | - | 4 | CAAT |  |
| CAAT-box | Arabidopsis thaliana | 1205 | - | 5 | CCAAT | common cis-acting element in promoter and enhancer regions |
| CAAT-box | Nicotiana glutinosa | 587 | - | 4 | CAAT |  |
| CAAT-box | Nicotiana glutinosa | 1298 | - | 4 | CAAT |  |
| CAAT-box | Pisum sativum | 1187 | + | 5 | CAAAT | common cis-acting element in promoter and enhancer regions |
| CAAT-box | Pisum sativum | 1192 | - | 5 | CAAAT | common cis-acting element in promoter and enhancer regions |
| CAAT-box | Nicotiana glutinosa | 351 | + | 4 | CAAT |  |
| CAAT-box | Nicotiana glutinosa | 427 | - | 4 | CAAT |  |
| CAAT-box | Arabidopsis thaliana | 1096 | - | 5 | CCAAT | common cis-acting element in promoter and enhancer regions |
| CAAT-box | Nicotiana glutinosa | 1088 | + | 4 | CAAT |  |
| CAAT-box | Nicotiana glutinosa | 561 | - | 4 | CAAT |  |
| CAAT-box | Pisum sativum | 392 | - | 5 | CAAAT | common cis-acting element in promoter and enhancer regions |
| CAAT-box | Pisum sativum | 278 | - | 5 | CAAAT | common cis-acting element in promoter and enhancer regions |
| CAAT-box | Arabidopsis thaliana | 350 | + | 5 | CCAAT | common cis-acting element in promoter and enhancer regions |
| CAAT-box | Arabidopsis thaliana | 258 | + | 5 | CCAAT | common cis-acting element in promoter and enhancer regions |
| CAAT-box | Pisum sativum | 235 | + | 5 | CAAAT | common cis-acting element in promoter and enhancer regions |
| CAAT-box | Nicotiana glutinosa | 259 | + | 4 | CAAT |  |
| CAAT-box | Pisum sativum | 251 | - | 5 | CAAAT | common cis-acting element in promoter and enhancer regions |
| CAAT-box | Nicotiana glutinosa | 221 | + | 4 | CAAT |  |
| CAAT-box | Pisum sativum | 196 | + | 5 | CAAAT | common cis-acting element in promoter and enhancer regions |
| CAAT-box | Arabidopsis thaliana | 220 | + | 5 | CCAAT | common cis-acting element in promoter and enhancer regions |
| CAAT-box | Nicotiana glutinosa | 128 | - | 4 | CAAT |  |

>023445   
+ TGTAAGTGGT GAATTACCCT GTAAATAAGG CTTTCTTCTT CAACCTACGG TGTCCCGGGC TCAAACCTCC   
  
  
+ GCTCTCCATA CTTGTAGGAT AAATGAATTT AGCGTAAAAA TAAAAAGAGA AAACAAAATT GTTAAAAAAC   
  
  
+ AACTTAATAT CTAAAATACC AAAATAAATA ACTATACTGG GGTGGAAATT TTTACCAAAT TACCGTACCA   
  
  
+ ACCGCCTTAC CAATCATACC ATACCAAATT ATTACCAAAA ATTTGTACCA ATCGGTAATG GTACGGTATT   
  
  
+ TGTACCATAC CATACTATTT TGATACGGTA CTGGTATCCA AAACCAGTAC CGGTGGTATA CCATACCTAC   
  
  
+ CAATATTATT AATATTATAT TATGTATTTA TTCATATAAA TATTTGAGGG AAAAATTAAT AAAATTAATA   
  
  
+ ATAATTATTG TAGAAGTGTA AAAAATGTAA AAAAAAGTAT ACATTCACTC ATAGTGACAA GTTTTACAAC   
  
  
+ TTTCATGAAA ATGTCAGCTT CAAAATCCAA CGCTATAATC CATAAACCTT AAATTTATAT TTAACCGTCG   
  
  
+ ATTGTCATTT ACGCTTTATT CTTCTTATTG AATTTCATTT TTAAAATTTT CTTTTTTGAA AACATGATCA   
  
  
+ TTTGAAGGAT GTAAGAAGAT GAATGGTTTA GATCTTTGAT ATCATGTTTC GATATTTACG GTTAACTGAA   
  
  
+ ATATGAGTCG ATGTCATATA GTTTGTAGAA ACTTTATAAA CATCAAGCAA TATTTTTATT AACCATAAAA   
  
  
+ CTTTGAACAT AATATCAACG ATCCAAATCA TTCATCTTCG TACATCCTCT AATAGATCAT GTTTTCAAAA   
  
  
+ AAGAAAATCT GAAAAAAAAC AAAATTTGAT GAAAAAAATG AAACGTAAAT GTAAACAACA ATCAACGATT   
  
  
+ AAATTTTGAT CTATGATTTA TATGATTATA ATGGCGAATT TCAAAGTTAA GCTCCTCACA AAAGTGGTAA   
  
  
+ AGCTTGTCAT TACAAGCGTT TATAAATTTT TTCTATATTT TTAACACCTC TATATTAATT AAAAAATATT   
  
  
+ AGATTTTGGG CAAACTAAGT GGCGTAAAAC AAAGTGACAA TTACCATTGG CATACCATAA CAACCAAACT   
  
  
+ ATTTGGTATA TTGATATTTA GTATACCGAA AGTTTGGTAC GGTAATGATA ATAGATTTTG CCGTATCAAA   
  
  
+ TATTTGGTAT GGTAATTGGT AGAGAGGTTT TGGTACGGTA ATGTACCAAA ACCACCCCTG TATGCATAGC   
  
  
+ TAAAAACAAA AATAATGAAA TACAAGTAGC TAGTGATATT GTTCTCCTAT TATTAGACAA AGAACCACCA   
  
  
+ AACTAACTGA GCCTTCCATT GCTCTTCCCT CCTTCCACAC ACATTTGACC CTTTCAGCTT TGTCTTGTCT   
  
  
+ CCACAACCCT AACTCCGCCC TTCTTTCTCT ATTTTCCACA TCCATCTTTC CCTTCACCAT GACATGTAAA   
  
  
+ TTTCAGAGGT AAAAAAATTT CTTGGTGGC  

- ACATTCACCA CTTAATGGGA CATTTATTCC GAAAGAAGAA GTTGGATGCC ACAGGGCCCG AGTTTGGAGG   
  
  
- CGAGAGGTAT GAACATCCTA TTTACTTAAA TCGCATTTTT ATTTTTCTCT TTTGTTTTAA CAATTTTTTG   
  
  
- TTGAATTATA GATTTTATGG TTTTATTTAT TGATATGACC CCACCTTTAA AAATGGTTTA ATGGCATGGT   
  
  
- TGGCGGAATG GTTAGTATGG TATGGTTTAA TAATGGTTTT TAAACATGGT TAGCCATTAC CATGCCATAA   
  
  
- ACATGGTATG GTATGATAAA ACTATGCCAT GACCATAGGT TTTGGTCATG GCCACCATAT GGTATGGATG   
  
  
- GTTATAATAA TTATAATATA ATACATAAAT AAGTATATTT ATAAACTCCC TTTTTAATTA TTTTAATTAT   
  
  
- TATTAATAAC ATCTTCACAT TTTTTACATT TTTTTTCATA TGTAAGTGAG TATCACTGTT CAAAATGTTG   
  
  
- AAAGTACTTT TACAGTCGAA GTTTTAGGTT GCGATATTAG GTATTTGGAA TTTAAATATA AATTGGCAGC   
  
  
- TAACAGTAAA TGCGAAATAA GAAGAATAAC TTAAAGTAAA AATTTTAAAA GAAAAAACTT TTGTACTAGT   
  
  
- AAACTTCCTA CATTCTTCTA CTTACCAAAT CTAGAAACTA TAGTACAAAG CTATAAATGC CAATTGACTT   
  
  
- TATACTCAGC TACAGTATAT CAAACATCTT TGAAATATTT GTAGTTCGTT ATAAAAATAA TTGGTATTTT   
  
  
- GAAACTTGTA TTATAGTTGC TAGGTTTAGT AAGTAGAAGC ATGTAGGAGA TTATCTAGTA CAAAAGTTTT   
  
  
- TTCTTTTAGA CTTTTTTTTG TTTTAAACTA CTTTTTTTAC TTTGCATTTA CATTTGTTGT TAGTTGCTAA   
  
  
- TTTAAAACTA GATACTAAAT ATACTAATAT TACCGCTTAA AGTTTCAATT CGAGGAGTGT TTTCACCATT   
  
  
- TCGAACAGTA ATGTTCGCAA ATATTTAAAA AAGATATAAA AATTGTGGAG ATATAATTAA TTTTTTATAA   
  
  
- TCTAAAACCC GTTTGATTCA CCGCATTTTG TTTCACTGTT AATGGTAACC GTATGGTATT GTTGGTTTGA   
  
  
- TAAACCATAT AACTATAAAT CATATGGCTT TCAAACCATG CCATTACTAT TATCTAAAAC GGCATAGTTT   
  
  
- ATAAACCATA CCATTAACCA TCTCTCCAAA ACCATGCCAT TACATGGTTT TGGTGGGGAC ATACGTATCG   
  
  
- ATTTTTGTTT TTATTACTTT ATGTTCATCG ATCACTATAA CAAGAGGATA ATAATCTGTT TCTTGGTGGT   
  
  
- TTGATTGACT CGGAAGGTAA CGAGAAGGGA GGAAGGTGTG TGTAAACTGG GAAAGTCGAA ACAGAACAGA   
  
  
- GGTGTTGGGA TTGAGGCGGG AAGAAAGAGA TAAAAGGTGT AGGTAGAAAG GGAAGTGGTA CTGTACATTT   
  
  
- AAAGTCTCCA TTTTTTTAAA GAACCACCG

+     CAT-box

| Site Name | Organism | Position | Strand | Matrix score. | sequence | function |
| --- | --- | --- | --- | --- | --- | --- |
| CAT-box | Arabidopsis thaliana | 1068 | - | 6 | GCCACT | cis-acting regulatory element related to meristem expression |

>023445   
+ TGTAAGTGGT GAATTACCCT GTAAATAAGG CTTTCTTCTT CAACCTACGG TGTCCCGGGC TCAAACCTCC   
  
  
+ GCTCTCCATA CTTGTAGGAT AAATGAATTT AGCGTAAAAA TAAAAAGAGA AAACAAAATT GTTAAAAAAC   
  
  
+ AACTTAATAT CTAAAATACC AAAATAAATA ACTATACTGG GGTGGAAATT TTTACCAAAT TACCGTACCA   
  
  
+ ACCGCCTTAC CAATCATACC ATACCAAATT ATTACCAAAA ATTTGTACCA ATCGGTAATG GTACGGTATT   
  
  
+ TGTACCATAC CATACTATTT TGATACGGTA CTGGTATCCA AAACCAGTAC CGGTGGTATA CCATACCTAC   
  
  
+ CAATATTATT AATATTATAT TATGTATTTA TTCATATAAA TATTTGAGGG AAAAATTAAT AAAATTAATA   
  
  
+ ATAATTATTG TAGAAGTGTA AAAAATGTAA AAAAAAGTAT ACATTCACTC ATAGTGACAA GTTTTACAAC   
  
  
+ TTTCATGAAA ATGTCAGCTT CAAAATCCAA CGCTATAATC CATAAACCTT AAATTTATAT TTAACCGTCG   
  
  
+ ATTGTCATTT ACGCTTTATT CTTCTTATTG AATTTCATTT TTAAAATTTT CTTTTTTGAA AACATGATCA   
  
  
+ TTTGAAGGAT GTAAGAAGAT GAATGGTTTA GATCTTTGAT ATCATGTTTC GATATTTACG GTTAACTGAA   
  
  
+ ATATGAGTCG ATGTCATATA GTTTGTAGAA ACTTTATAAA CATCAAGCAA TATTTTTATT AACCATAAAA   
  
  
+ CTTTGAACAT AATATCAACG ATCCAAATCA TTCATCTTCG TACATCCTCT AATAGATCAT GTTTTCAAAA   
  
  
+ AAGAAAATCT GAAAAAAAAC AAAATTTGAT GAAAAAAATG AAACGTAAAT GTAAACAACA ATCAACGATT   
  
  
+ AAATTTTGAT CTATGATTTA TATGATTATA ATGGCGAATT TCAAAGTTAA GCTCCTCACA AAAGTGGTAA   
  
  
+ AGCTTGTCAT TACAAGCGTT TATAAATTTT TTCTATATTT TTAACACCTC TATATTAATT AAAAAATATT   
  
  
+ AGATTTTGGG CAAACTAAGT GGCGTAAAAC AAAGTGACAA TTACCATTGG CATACCATAA CAACCAAACT   
  
  
+ ATTTGGTATA TTGATATTTA GTATACCGAA AGTTTGGTAC GGTAATGATA ATAGATTTTG CCGTATCAAA   
  
  
+ TATTTGGTAT GGTAATTGGT AGAGAGGTTT TGGTACGGTA ATGTACCAAA ACCACCCCTG TATGCATAGC   
  
  
+ TAAAAACAAA AATAATGAAA TACAAGTAGC TAGTGATATT GTTCTCCTAT TATTAGACAA AGAACCACCA   
  
  
+ AACTAACTGA GCCTTCCATT GCTCTTCCCT CCTTCCACAC ACATTTGACC CTTTCAGCTT TGTCTTGTCT   
  
  
+ CCACAACCCT AACTCCGCCC TTCTTTCTCT ATTTTCCACA TCCATCTTTC CCTTCACCAT GACATGTAAA   
  
  
+ TTTCAGAGGT AAAAAAATTT CTTGGTGGC  

- ACATTCACCA CTTAATGGGA CATTTATTCC GAAAGAAGAA GTTGGATGCC ACAGGGCCCG AGTTTGGAGG   
  
  
- CGAGAGGTAT GAACATCCTA TTTACTTAAA TCGCATTTTT ATTTTTCTCT TTTGTTTTAA CAATTTTTTG   
  
  
- TTGAATTATA GATTTTATGG TTTTATTTAT TGATATGACC CCACCTTTAA AAATGGTTTA ATGGCATGGT   
  
  
- TGGCGGAATG GTTAGTATGG TATGGTTTAA TAATGGTTTT TAAACATGGT TAGCCATTAC CATGCCATAA   
  
  
- ACATGGTATG GTATGATAAA ACTATGCCAT GACCATAGGT TTTGGTCATG GCCACCATAT GGTATGGATG   
  
  
- GTTATAATAA TTATAATATA ATACATAAAT AAGTATATTT ATAAACTCCC TTTTTAATTA TTTTAATTAT   
  
  
- TATTAATAAC ATCTTCACAT TTTTTACATT TTTTTTCATA TGTAAGTGAG TATCACTGTT CAAAATGTTG   
  
  
- AAAGTACTTT TACAGTCGAA GTTTTAGGTT GCGATATTAG GTATTTGGAA TTTAAATATA AATTGGCAGC   
  
  
- TAACAGTAAA TGCGAAATAA GAAGAATAAC TTAAAGTAAA AATTTTAAAA GAAAAAACTT TTGTACTAGT   
  
  
- AAACTTCCTA CATTCTTCTA CTTACCAAAT CTAGAAACTA TAGTACAAAG CTATAAATGC CAATTGACTT   
  
  
- TATACTCAGC TACAGTATAT CAAACATCTT TGAAATATTT GTAGTTCGTT ATAAAAATAA TTGGTATTTT   
  
  
- GAAACTTGTA TTATAGTTGC TAGGTTTAGT AAGTAGAAGC ATGTAGGAGA TTATCTAGTA CAAAAGTTTT   
  
  
- TTCTTTTAGA CTTTTTTTTG TTTTAAACTA CTTTTTTTAC TTTGCATTTA CATTTGTTGT TAGTTGCTAA   
  
  
- TTTAAAACTA GATACTAAAT ATACTAATAT TACCGCTTAA AGTTTCAATT CGAGGAGTGT TTTCACCATT   
  
  
- TCGAACAGTA ATGTTCGCAA ATATTTAAAA AAGATATAAA AATTGTGGAG ATATAATTAA TTTTTTATAA   
  
  
- TCTAAAACCC GTTTGATTCA CCGCATTTTG TTTCACTGTT AATGGTAACC GTATGGTATT GTTGGTTTGA   
  
  
- TAAACCATAT AACTATAAAT CATATGGCTT TCAAACCATG CCATTACTAT TATCTAAAAC GGCATAGTTT   
  
  
- ATAAACCATA CCATTAACCA TCTCTCCAAA ACCATGCCAT TACATGGTTT TGGTGGGGAC ATACGTATCG   
  
  
- ATTTTTGTTT TTATTACTTT ATGTTCATCG ATCACTATAA CAAGAGGATA ATAATCTGTT TCTTGGTGGT   
  
  
- TTGATTGACT CGGAAGGTAA CGAGAAGGGA GGAAGGTGTG TGTAAACTGG GAAAGTCGAA ACAGAACAGA   
  
  
- GGTGTTGGGA TTGAGGCGGG AAGAAAGAGA TAAAAGGTGT AGGTAGAAAG GGAAGTGGTA CTGTACATTT   
  
  
- AAAGTCTCCA TTTTTTTAAA GAACCACCG

+     ERE

| Site Name | Organism | Position | Strand | Matrix score. | sequence | function |
| --- | --- | --- | --- | --- | --- | --- |
| ERE | Nicotiana glutinos | 600 | - | 8 | ATTTTAAA |  |

>023445   
+ TGTAAGTGGT GAATTACCCT GTAAATAAGG CTTTCTTCTT CAACCTACGG TGTCCCGGGC TCAAACCTCC   
  
  
+ GCTCTCCATA CTTGTAGGAT AAATGAATTT AGCGTAAAAA TAAAAAGAGA AAACAAAATT GTTAAAAAAC   
  
  
+ AACTTAATAT CTAAAATACC AAAATAAATA ACTATACTGG GGTGGAAATT TTTACCAAAT TACCGTACCA   
  
  
+ ACCGCCTTAC CAATCATACC ATACCAAATT ATTACCAAAA ATTTGTACCA ATCGGTAATG GTACGGTATT   
  
  
+ TGTACCATAC CATACTATTT TGATACGGTA CTGGTATCCA AAACCAGTAC CGGTGGTATA CCATACCTAC   
  
  
+ CAATATTATT AATATTATAT TATGTATTTA TTCATATAAA TATTTGAGGG AAAAATTAAT AAAATTAATA   
  
  
+ ATAATTATTG TAGAAGTGTA AAAAATGTAA AAAAAAGTAT ACATTCACTC ATAGTGACAA GTTTTACAAC   
  
  
+ TTTCATGAAA ATGTCAGCTT CAAAATCCAA CGCTATAATC CATAAACCTT AAATTTATAT TTAACCGTCG   
  
  
+ ATTGTCATTT ACGCTTTATT CTTCTTATTG AATTTCATTT TTAAAATTTT CTTTTTTGAA AACATGATCA   
  
  
+ TTTGAAGGAT GTAAGAAGAT GAATGGTTTA GATCTTTGAT ATCATGTTTC GATATTTACG GTTAACTGAA   
  
  
+ ATATGAGTCG ATGTCATATA GTTTGTAGAA ACTTTATAAA CATCAAGCAA TATTTTTATT AACCATAAAA   
  
  
+ CTTTGAACAT AATATCAACG ATCCAAATCA TTCATCTTCG TACATCCTCT AATAGATCAT GTTTTCAAAA   
  
  
+ AAGAAAATCT GAAAAAAAAC AAAATTTGAT GAAAAAAATG AAACGTAAAT GTAAACAACA ATCAACGATT   
  
  
+ AAATTTTGAT CTATGATTTA TATGATTATA ATGGCGAATT TCAAAGTTAA GCTCCTCACA AAAGTGGTAA   
  
  
+ AGCTTGTCAT TACAAGCGTT TATAAATTTT TTCTATATTT TTAACACCTC TATATTAATT AAAAAATATT   
  
  
+ AGATTTTGGG CAAACTAAGT GGCGTAAAAC AAAGTGACAA TTACCATTGG CATACCATAA CAACCAAACT   
  
  
+ ATTTGGTATA TTGATATTTA GTATACCGAA AGTTTGGTAC GGTAATGATA ATAGATTTTG CCGTATCAAA   
  
  
+ TATTTGGTAT GGTAATTGGT AGAGAGGTTT TGGTACGGTA ATGTACCAAA ACCACCCCTG TATGCATAGC   
  
  
+ TAAAAACAAA AATAATGAAA TACAAGTAGC TAGTGATATT GTTCTCCTAT TATTAGACAA AGAACCACCA   
  
  
+ AACTAACTGA GCCTTCCATT GCTCTTCCCT CCTTCCACAC ACATTTGACC CTTTCAGCTT TGTCTTGTCT   
  
  
+ CCACAACCCT AACTCCGCCC TTCTTTCTCT ATTTTCCACA TCCATCTTTC CCTTCACCAT GACATGTAAA   
  
  
+ TTTCAGAGGT AAAAAAATTT CTTGGTGGC  

- ACATTCACCA CTTAATGGGA CATTTATTCC GAAAGAAGAA GTTGGATGCC ACAGGGCCCG AGTTTGGAGG   
  
  
- CGAGAGGTAT GAACATCCTA TTTACTTAAA TCGCATTTTT ATTTTTCTCT TTTGTTTTAA CAATTTTTTG   
  
  
- TTGAATTATA GATTTTATGG TTTTATTTAT TGATATGACC CCACCTTTAA AAATGGTTTA ATGGCATGGT   
  
  
- TGGCGGAATG GTTAGTATGG TATGGTTTAA TAATGGTTTT TAAACATGGT TAGCCATTAC CATGCCATAA   
  
  
- ACATGGTATG GTATGATAAA ACTATGCCAT GACCATAGGT TTTGGTCATG GCCACCATAT GGTATGGATG   
  
  
- GTTATAATAA TTATAATATA ATACATAAAT AAGTATATTT ATAAACTCCC TTTTTAATTA TTTTAATTAT   
  
  
- TATTAATAAC ATCTTCACAT TTTTTACATT TTTTTTCATA TGTAAGTGAG TATCACTGTT CAAAATGTTG   
  
  
- AAAGTACTTT TACAGTCGAA GTTTTAGGTT GCGATATTAG GTATTTGGAA TTTAAATATA AATTGGCAGC   
  
  
- TAACAGTAAA TGCGAAATAA GAAGAATAAC TTAAAGTAAA AATTTTAAAA GAAAAAACTT TTGTACTAGT   
  
  
- AAACTTCCTA CATTCTTCTA CTTACCAAAT CTAGAAACTA TAGTACAAAG CTATAAATGC CAATTGACTT   
  
  
- TATACTCAGC TACAGTATAT CAAACATCTT TGAAATATTT GTAGTTCGTT ATAAAAATAA TTGGTATTTT   
  
  
- GAAACTTGTA TTATAGTTGC TAGGTTTAGT AAGTAGAAGC ATGTAGGAGA TTATCTAGTA CAAAAGTTTT   
  
  
- TTCTTTTAGA CTTTTTTTTG TTTTAAACTA CTTTTTTTAC TTTGCATTTA CATTTGTTGT TAGTTGCTAA   
  
  
- TTTAAAACTA GATACTAAAT ATACTAATAT TACCGCTTAA AGTTTCAATT CGAGGAGTGT TTTCACCATT   
  
  
- TCGAACAGTA ATGTTCGCAA ATATTTAAAA AAGATATAAA AATTGTGGAG ATATAATTAA TTTTTTATAA   
  
  
- TCTAAAACCC GTTTGATTCA CCGCATTTTG TTTCACTGTT AATGGTAACC GTATGGTATT GTTGGTTTGA   
  
  
- TAAACCATAT AACTATAAAT CATATGGCTT TCAAACCATG CCATTACTAT TATCTAAAAC GGCATAGTTT   
  
  
- ATAAACCATA CCATTAACCA TCTCTCCAAA ACCATGCCAT TACATGGTTT TGGTGGGGAC ATACGTATCG   
  
  
- ATTTTTGTTT TTATTACTTT ATGTTCATCG ATCACTATAA CAAGAGGATA ATAATCTGTT TCTTGGTGGT   
  
  
- TTGATTGACT CGGAAGGTAA CGAGAAGGGA GGAAGGTGTG TGTAAACTGG GAAAGTCGAA ACAGAACAGA   
  
  
- GGTGTTGGGA TTGAGGCGGG AAGAAAGAGA TAAAAGGTGT AGGTAGAAAG GGAAGTGGTA CTGTACATTT   
  
  
- AAAGTCTCCA TTTTTTTAAA GAACCACCG

+     F-box

| Site Name | Organism | Position | Strand | Matrix score. | sequence | function |
| --- | --- | --- | --- | --- | --- | --- |
| F-box | Lycopersicon esculentum | 1164 | - | 10 | CTATTCTCATT |  |

>023445   
+ TGTAAGTGGT GAATTACCCT GTAAATAAGG CTTTCTTCTT CAACCTACGG TGTCCCGGGC TCAAACCTCC   
  
  
+ GCTCTCCATA CTTGTAGGAT AAATGAATTT AGCGTAAAAA TAAAAAGAGA AAACAAAATT GTTAAAAAAC   
  
  
+ AACTTAATAT CTAAAATACC AAAATAAATA ACTATACTGG GGTGGAAATT TTTACCAAAT TACCGTACCA   
  
  
+ ACCGCCTTAC CAATCATACC ATACCAAATT ATTACCAAAA ATTTGTACCA ATCGGTAATG GTACGGTATT   
  
  
+ TGTACCATAC CATACTATTT TGATACGGTA CTGGTATCCA AAACCAGTAC CGGTGGTATA CCATACCTAC   
  
  
+ CAATATTATT AATATTATAT TATGTATTTA TTCATATAAA TATTTGAGGG AAAAATTAAT AAAATTAATA   
  
  
+ ATAATTATTG TAGAAGTGTA AAAAATGTAA AAAAAAGTAT ACATTCACTC ATAGTGACAA GTTTTACAAC   
  
  
+ TTTCATGAAA ATGTCAGCTT CAAAATCCAA CGCTATAATC CATAAACCTT AAATTTATAT TTAACCGTCG   
  
  
+ ATTGTCATTT ACGCTTTATT CTTCTTATTG AATTTCATTT TTAAAATTTT CTTTTTTGAA AACATGATCA   
  
  
+ TTTGAAGGAT GTAAGAAGAT GAATGGTTTA GATCTTTGAT ATCATGTTTC GATATTTACG GTTAACTGAA   
  
  
+ ATATGAGTCG ATGTCATATA GTTTGTAGAA ACTTTATAAA CATCAAGCAA TATTTTTATT AACCATAAAA   
  
  
+ CTTTGAACAT AATATCAACG ATCCAAATCA TTCATCTTCG TACATCCTCT AATAGATCAT GTTTTCAAAA   
  
  
+ AAGAAAATCT GAAAAAAAAC AAAATTTGAT GAAAAAAATG AAACGTAAAT GTAAACAACA ATCAACGATT   
  
  
+ AAATTTTGAT CTATGATTTA TATGATTATA ATGGCGAATT TCAAAGTTAA GCTCCTCACA AAAGTGGTAA   
  
  
+ AGCTTGTCAT TACAAGCGTT TATAAATTTT TTCTATATTT TTAACACCTC TATATTAATT AAAAAATATT   
  
  
+ AGATTTTGGG CAAACTAAGT GGCGTAAAAC AAAGTGACAA TTACCATTGG CATACCATAA CAACCAAACT   
  
  
+ ATTTGGTATA TTGATATTTA GTATACCGAA AGTTTGGTAC GGTAATGATA ATAGATTTTG CCGTATCAAA   
  
  
+ TATTTGGTAT GGTAATTGGT AGAGAGGTTT TGGTACGGTA ATGTACCAAA ACCACCCCTG TATGCATAGC   
  
  
+ TAAAAACAAA AATAATGAAA TACAAGTAGC TAGTGATATT GTTCTCCTAT TATTAGACAA AGAACCACCA   
  
  
+ AACTAACTGA GCCTTCCATT GCTCTTCCCT CCTTCCACAC ACATTTGACC CTTTCAGCTT TGTCTTGTCT   
  
  
+ CCACAACCCT AACTCCGCCC TTCTTTCTCT ATTTTCCACA TCCATCTTTC CCTTCACCAT GACATGTAAA   
  
  
+ TTTCAGAGGT AAAAAAATTT CTTGGTGGC  

- ACATTCACCA CTTAATGGGA CATTTATTCC GAAAGAAGAA GTTGGATGCC ACAGGGCCCG AGTTTGGAGG   
  
  
- CGAGAGGTAT GAACATCCTA TTTACTTAAA TCGCATTTTT ATTTTTCTCT TTTGTTTTAA CAATTTTTTG   
  
  
- TTGAATTATA GATTTTATGG TTTTATTTAT TGATATGACC CCACCTTTAA AAATGGTTTA ATGGCATGGT   
  
  
- TGGCGGAATG GTTAGTATGG TATGGTTTAA TAATGGTTTT TAAACATGGT TAGCCATTAC CATGCCATAA   
  
  
- ACATGGTATG GTATGATAAA ACTATGCCAT GACCATAGGT TTTGGTCATG GCCACCATAT GGTATGGATG   
  
  
- GTTATAATAA TTATAATATA ATACATAAAT AAGTATATTT ATAAACTCCC TTTTTAATTA TTTTAATTAT   
  
  
- TATTAATAAC ATCTTCACAT TTTTTACATT TTTTTTCATA TGTAAGTGAG TATCACTGTT CAAAATGTTG   
  
  
- AAAGTACTTT TACAGTCGAA GTTTTAGGTT GCGATATTAG GTATTTGGAA TTTAAATATA AATTGGCAGC   
  
  
- TAACAGTAAA TGCGAAATAA GAAGAATAAC TTAAAGTAAA AATTTTAAAA GAAAAAACTT TTGTACTAGT   
  
  
- AAACTTCCTA CATTCTTCTA CTTACCAAAT CTAGAAACTA TAGTACAAAG CTATAAATGC CAATTGACTT   
  
  
- TATACTCAGC TACAGTATAT CAAACATCTT TGAAATATTT GTAGTTCGTT ATAAAAATAA TTGGTATTTT   
  
  
- GAAACTTGTA TTATAGTTGC TAGGTTTAGT AAGTAGAAGC ATGTAGGAGA TTATCTAGTA CAAAAGTTTT   
  
  
- TTCTTTTAGA CTTTTTTTTG TTTTAAACTA CTTTTTTTAC TTTGCATTTA CATTTGTTGT TAGTTGCTAA   
  
  
- TTTAAAACTA GATACTAAAT ATACTAATAT TACCGCTTAA AGTTTCAATT CGAGGAGTGT TTTCACCATT   
  
  
- TCGAACAGTA ATGTTCGCAA ATATTTAAAA AAGATATAAA AATTGTGGAG ATATAATTAA TTTTTTATAA   
  
  
- TCTAAAACCC GTTTGATTCA CCGCATTTTG TTTCACTGTT AATGGTAACC GTATGGTATT GTTGGTTTGA   
  
  
- TAAACCATAT AACTATAAAT CATATGGCTT TCAAACCATG CCATTACTAT TATCTAAAAC GGCATAGTTT   
  
  
- ATAAACCATA CCATTAACCA TCTCTCCAAA ACCATGCCAT TACATGGTTT TGGTGGGGAC ATACGTATCG   
  
  
- ATTTTTGTTT TTATTACTTT ATGTTCATCG ATCACTATAA CAAGAGGATA ATAATCTGTT TCTTGGTGGT   
  
  
- TTGATTGACT CGGAAGGTAA CGAGAAGGGA GGAAGGTGTG TGTAAACTGG GAAAGTCGAA ACAGAACAGA   
  
  
- GGTGTTGGGA TTGAGGCGGG AAGAAAGAGA TAAAAGGTGT AGGTAGAAAG GGAAGTGGTA CTGTACATTT   
  
  
- AAAGTCTCCA TTTTTTTAAA GAACCACCG

+     GT1-motif

| Site Name | Organism | Position | Strand | Matrix score. | sequence | function |
| --- | --- | --- | --- | --- | --- | --- |
| GT1-motif | Arabidopsis thaliana | 759 | - | 6 | GGTTAA | light responsive element |
| GT1-motif | Avena sativa | 758 | - | 7 | GGTTAAT | light responsive element |
| GT1-motif | Arabidopsis thaliana | 551 | - | 6 | GGTTAA | light responsive element |
| GT1-motif | Arabidopsis thaliana | 690 | + | 6 | GGTTAA | light responsive element |

>023445   
+ TGTAAGTGGT GAATTACCCT GTAAATAAGG CTTTCTTCTT CAACCTACGG TGTCCCGGGC TCAAACCTCC   
  
  
+ GCTCTCCATA CTTGTAGGAT AAATGAATTT AGCGTAAAAA TAAAAAGAGA AAACAAAATT GTTAAAAAAC   
  
  
+ AACTTAATAT CTAAAATACC AAAATAAATA ACTATACTGG GGTGGAAATT TTTACCAAAT TACCGTACCA   
  
  
+ ACCGCCTTAC CAATCATACC ATACCAAATT ATTACCAAAA ATTTGTACCA ATCGGTAATG GTACGGTATT   
  
  
+ TGTACCATAC CATACTATTT TGATACGGTA CTGGTATCCA AAACCAGTAC CGGTGGTATA CCATACCTAC   
  
  
+ CAATATTATT AATATTATAT TATGTATTTA TTCATATAAA TATTTGAGGG AAAAATTAAT AAAATTAATA   
  
  
+ ATAATTATTG TAGAAGTGTA AAAAATGTAA AAAAAAGTAT ACATTCACTC ATAGTGACAA GTTTTACAAC   
  
  
+ TTTCATGAAA ATGTCAGCTT CAAAATCCAA CGCTATAATC CATAAACCTT AAATTTATAT TTAACCGTCG   
  
  
+ ATTGTCATTT ACGCTTTATT CTTCTTATTG AATTTCATTT TTAAAATTTT CTTTTTTGAA AACATGATCA   
  
  
+ TTTGAAGGAT GTAAGAAGAT GAATGGTTTA GATCTTTGAT ATCATGTTTC GATATTTACG GTTAACTGAA   
  
  
+ ATATGAGTCG ATGTCATATA GTTTGTAGAA ACTTTATAAA CATCAAGCAA TATTTTTATT AACCATAAAA   
  
  
+ CTTTGAACAT AATATCAACG ATCCAAATCA TTCATCTTCG TACATCCTCT AATAGATCAT GTTTTCAAAA   
  
  
+ AAGAAAATCT GAAAAAAAAC AAAATTTGAT GAAAAAAATG AAACGTAAAT GTAAACAACA ATCAACGATT   
  
  
+ AAATTTTGAT CTATGATTTA TATGATTATA ATGGCGAATT TCAAAGTTAA GCTCCTCACA AAAGTGGTAA   
  
  
+ AGCTTGTCAT TACAAGCGTT TATAAATTTT TTCTATATTT TTAACACCTC TATATTAATT AAAAAATATT   
  
  
+ AGATTTTGGG CAAACTAAGT GGCGTAAAAC AAAGTGACAA TTACCATTGG CATACCATAA CAACCAAACT   
  
  
+ ATTTGGTATA TTGATATTTA GTATACCGAA AGTTTGGTAC GGTAATGATA ATAGATTTTG CCGTATCAAA   
  
  
+ TATTTGGTAT GGTAATTGGT AGAGAGGTTT TGGTACGGTA ATGTACCAAA ACCACCCCTG TATGCATAGC   
  
  
+ TAAAAACAAA AATAATGAAA TACAAGTAGC TAGTGATATT GTTCTCCTAT TATTAGACAA AGAACCACCA   
  
  
+ AACTAACTGA GCCTTCCATT GCTCTTCCCT CCTTCCACAC ACATTTGACC CTTTCAGCTT TGTCTTGTCT   
  
  
+ CCACAACCCT AACTCCGCCC TTCTTTCTCT ATTTTCCACA TCCATCTTTC CCTTCACCAT GACATGTAAA   
  
  
+ TTTCAGAGGT AAAAAAATTT CTTGGTGGC  

- ACATTCACCA CTTAATGGGA CATTTATTCC GAAAGAAGAA GTTGGATGCC ACAGGGCCCG AGTTTGGAGG   
  
  
- CGAGAGGTAT GAACATCCTA TTTACTTAAA TCGCATTTTT ATTTTTCTCT TTTGTTTTAA CAATTTTTTG   
  
  
- TTGAATTATA GATTTTATGG TTTTATTTAT TGATATGACC CCACCTTTAA AAATGGTTTA ATGGCATGGT   
  
  
- TGGCGGAATG GTTAGTATGG TATGGTTTAA TAATGGTTTT TAAACATGGT TAGCCATTAC CATGCCATAA   
  
  
- ACATGGTATG GTATGATAAA ACTATGCCAT GACCATAGGT TTTGGTCATG GCCACCATAT GGTATGGATG   
  
  
- GTTATAATAA TTATAATATA ATACATAAAT AAGTATATTT ATAAACTCCC TTTTTAATTA TTTTAATTAT   
  
  
- TATTAATAAC ATCTTCACAT TTTTTACATT TTTTTTCATA TGTAAGTGAG TATCACTGTT CAAAATGTTG   
  
  
- AAAGTACTTT TACAGTCGAA GTTTTAGGTT GCGATATTAG GTATTTGGAA TTTAAATATA AATTGGCAGC   
  
  
- TAACAGTAAA TGCGAAATAA GAAGAATAAC TTAAAGTAAA AATTTTAAAA GAAAAAACTT TTGTACTAGT   
  
  
- AAACTTCCTA CATTCTTCTA CTTACCAAAT CTAGAAACTA TAGTACAAAG CTATAAATGC CAATTGACTT   
  
  
- TATACTCAGC TACAGTATAT CAAACATCTT TGAAATATTT GTAGTTCGTT ATAAAAATAA TTGGTATTTT   
  
  
- GAAACTTGTA TTATAGTTGC TAGGTTTAGT AAGTAGAAGC ATGTAGGAGA TTATCTAGTA CAAAAGTTTT   
  
  
- TTCTTTTAGA CTTTTTTTTG TTTTAAACTA CTTTTTTTAC TTTGCATTTA CATTTGTTGT TAGTTGCTAA   
  
  
- TTTAAAACTA GATACTAAAT ATACTAATAT TACCGCTTAA AGTTTCAATT CGAGGAGTGT TTTCACCATT   
  
  
- TCGAACAGTA ATGTTCGCAA ATATTTAAAA AAGATATAAA AATTGTGGAG ATATAATTAA TTTTTTATAA   
  
  
- TCTAAAACCC GTTTGATTCA CCGCATTTTG TTTCACTGTT AATGGTAACC GTATGGTATT GTTGGTTTGA   
  
  
- TAAACCATAT AACTATAAAT CATATGGCTT TCAAACCATG CCATTACTAT TATCTAAAAC GGCATAGTTT   
  
  
- ATAAACCATA CCATTAACCA TCTCTCCAAA ACCATGCCAT TACATGGTTT TGGTGGGGAC ATACGTATCG   
  
  
- ATTTTTGTTT TTATTACTTT ATGTTCATCG ATCACTATAA CAAGAGGATA ATAATCTGTT TCTTGGTGGT   
  
  
- TTGATTGACT CGGAAGGTAA CGAGAAGGGA GGAAGGTGTG TGTAAACTGG GAAAGTCGAA ACAGAACAGA   
  
  
- GGTGTTGGGA TTGAGGCGGG AAGAAAGAGA TAAAAGGTGT AGGTAGAAAG GGAAGTGGTA CTGTACATTT   
  
  
- AAAGTCTCCA TTTTTTTAAA GAACCACCG

+     LTR

| Site Name | Organism | Position | Strand | Matrix score. | sequence | function |
| --- | --- | --- | --- | --- | --- | --- |
| LTR | Hordeum vulgare | 1146 | + | 6 | CCGAAA | cis-acting element involved in low-temperature responsiveness |

>023445   
+ TGTAAGTGGT GAATTACCCT GTAAATAAGG CTTTCTTCTT CAACCTACGG TGTCCCGGGC TCAAACCTCC   
  
  
+ GCTCTCCATA CTTGTAGGAT AAATGAATTT AGCGTAAAAA TAAAAAGAGA AAACAAAATT GTTAAAAAAC   
  
  
+ AACTTAATAT CTAAAATACC AAAATAAATA ACTATACTGG GGTGGAAATT TTTACCAAAT TACCGTACCA   
  
  
+ ACCGCCTTAC CAATCATACC ATACCAAATT ATTACCAAAA ATTTGTACCA ATCGGTAATG GTACGGTATT   
  
  
+ TGTACCATAC CATACTATTT TGATACGGTA CTGGTATCCA AAACCAGTAC CGGTGGTATA CCATACCTAC   
  
  
+ CAATATTATT AATATTATAT TATGTATTTA TTCATATAAA TATTTGAGGG AAAAATTAAT AAAATTAATA   
  
  
+ ATAATTATTG TAGAAGTGTA AAAAATGTAA AAAAAAGTAT ACATTCACTC ATAGTGACAA GTTTTACAAC   
  
  
+ TTTCATGAAA ATGTCAGCTT CAAAATCCAA CGCTATAATC CATAAACCTT AAATTTATAT TTAACCGTCG   
  
  
+ ATTGTCATTT ACGCTTTATT CTTCTTATTG AATTTCATTT TTAAAATTTT CTTTTTTGAA AACATGATCA   
  
  
+ TTTGAAGGAT GTAAGAAGAT GAATGGTTTA GATCTTTGAT ATCATGTTTC GATATTTACG GTTAACTGAA   
  
  
+ ATATGAGTCG ATGTCATATA GTTTGTAGAA ACTTTATAAA CATCAAGCAA TATTTTTATT AACCATAAAA   
  
  
+ CTTTGAACAT AATATCAACG ATCCAAATCA TTCATCTTCG TACATCCTCT AATAGATCAT GTTTTCAAAA   
  
  
+ AAGAAAATCT GAAAAAAAAC AAAATTTGAT GAAAAAAATG AAACGTAAAT GTAAACAACA ATCAACGATT   
  
  
+ AAATTTTGAT CTATGATTTA TATGATTATA ATGGCGAATT TCAAAGTTAA GCTCCTCACA AAAGTGGTAA   
  
  
+ AGCTTGTCAT TACAAGCGTT TATAAATTTT TTCTATATTT TTAACACCTC TATATTAATT AAAAAATATT   
  
  
+ AGATTTTGGG CAAACTAAGT GGCGTAAAAC AAAGTGACAA TTACCATTGG CATACCATAA CAACCAAACT   
  
  
+ ATTTGGTATA TTGATATTTA GTATACCGAA AGTTTGGTAC GGTAATGATA ATAGATTTTG CCGTATCAAA   
  
  
+ TATTTGGTAT GGTAATTGGT AGAGAGGTTT TGGTACGGTA ATGTACCAAA ACCACCCCTG TATGCATAGC   
  
  
+ TAAAAACAAA AATAATGAAA TACAAGTAGC TAGTGATATT GTTCTCCTAT TATTAGACAA AGAACCACCA   
  
  
+ AACTAACTGA GCCTTCCATT GCTCTTCCCT CCTTCCACAC ACATTTGACC CTTTCAGCTT TGTCTTGTCT   
  
  
+ CCACAACCCT AACTCCGCCC TTCTTTCTCT ATTTTCCACA TCCATCTTTC CCTTCACCAT GACATGTAAA   
  
  
+ TTTCAGAGGT AAAAAAATTT CTTGGTGGC  

- ACATTCACCA CTTAATGGGA CATTTATTCC GAAAGAAGAA GTTGGATGCC ACAGGGCCCG AGTTTGGAGG   
  
  
- CGAGAGGTAT GAACATCCTA TTTACTTAAA TCGCATTTTT ATTTTTCTCT TTTGTTTTAA CAATTTTTTG   
  
  
- TTGAATTATA GATTTTATGG TTTTATTTAT TGATATGACC CCACCTTTAA AAATGGTTTA ATGGCATGGT   
  
  
- TGGCGGAATG GTTAGTATGG TATGGTTTAA TAATGGTTTT TAAACATGGT TAGCCATTAC CATGCCATAA   
  
  
- ACATGGTATG GTATGATAAA ACTATGCCAT GACCATAGGT TTTGGTCATG GCCACCATAT GGTATGGATG   
  
  
- GTTATAATAA TTATAATATA ATACATAAAT AAGTATATTT ATAAACTCCC TTTTTAATTA TTTTAATTAT   
  
  
- TATTAATAAC ATCTTCACAT TTTTTACATT TTTTTTCATA TGTAAGTGAG TATCACTGTT CAAAATGTTG   
  
  
- AAAGTACTTT TACAGTCGAA GTTTTAGGTT GCGATATTAG GTATTTGGAA TTTAAATATA AATTGGCAGC   
  
  
- TAACAGTAAA TGCGAAATAA GAAGAATAAC TTAAAGTAAA AATTTTAAAA GAAAAAACTT TTGTACTAGT   
  
  
- AAACTTCCTA CATTCTTCTA CTTACCAAAT CTAGAAACTA TAGTACAAAG CTATAAATGC CAATTGACTT   
  
  
- TATACTCAGC TACAGTATAT CAAACATCTT TGAAATATTT GTAGTTCGTT ATAAAAATAA TTGGTATTTT   
  
  
- GAAACTTGTA TTATAGTTGC TAGGTTTAGT AAGTAGAAGC ATGTAGGAGA TTATCTAGTA CAAAAGTTTT   
  
  
- TTCTTTTAGA CTTTTTTTTG TTTTAAACTA CTTTTTTTAC TTTGCATTTA CATTTGTTGT TAGTTGCTAA   
  
  
- TTTAAAACTA GATACTAAAT ATACTAATAT TACCGCTTAA AGTTTCAATT CGAGGAGTGT TTTCACCATT   
  
  
- TCGAACAGTA ATGTTCGCAA ATATTTAAAA AAGATATAAA AATTGTGGAG ATATAATTAA TTTTTTATAA   
  
  
- TCTAAAACCC GTTTGATTCA CCGCATTTTG TTTCACTGTT AATGGTAACC GTATGGTATT GTTGGTTTGA   
  
  
- TAAACCATAT AACTATAAAT CATATGGCTT TCAAACCATG CCATTACTAT TATCTAAAAC GGCATAGTTT   
  
  
- ATAAACCATA CCATTAACCA TCTCTCCAAA ACCATGCCAT TACATGGTTT TGGTGGGGAC ATACGTATCG   
  
  
- ATTTTTGTTT TTATTACTTT ATGTTCATCG ATCACTATAA CAAGAGGATA ATAATCTGTT TCTTGGTGGT   
  
  
- TTGATTGACT CGGAAGGTAA CGAGAAGGGA GGAAGGTGTG TGTAAACTGG GAAAGTCGAA ACAGAACAGA   
  
  
- GGTGTTGGGA TTGAGGCGGG AAGAAAGAGA TAAAAGGTGT AGGTAGAAAG GGAAGTGGTA CTGTACATTT   
  
  
- AAAGTCTCCA TTTTTTTAAA GAACCACCG

+     MBSI

| Site Name | Organism | Position | Strand | Matrix score. | sequence | function |
| --- | --- | --- | --- | --- | --- | --- |
| MBSI | Petunia hybrida | 683 | + | 11 | TTTTTACGGTTA | MYB binding site involved in flavonoid biosynthetic genes regulation |

>023445   
+ TGTAAGTGGT GAATTACCCT GTAAATAAGG CTTTCTTCTT CAACCTACGG TGTCCCGGGC TCAAACCTCC   
  
  
+ GCTCTCCATA CTTGTAGGAT AAATGAATTT AGCGTAAAAA TAAAAAGAGA AAACAAAATT GTTAAAAAAC   
  
  
+ AACTTAATAT CTAAAATACC AAAATAAATA ACTATACTGG GGTGGAAATT TTTACCAAAT TACCGTACCA   
  
  
+ ACCGCCTTAC CAATCATACC ATACCAAATT ATTACCAAAA ATTTGTACCA ATCGGTAATG GTACGGTATT   
  
  
+ TGTACCATAC CATACTATTT TGATACGGTA CTGGTATCCA AAACCAGTAC CGGTGGTATA CCATACCTAC   
  
  
+ CAATATTATT AATATTATAT TATGTATTTA TTCATATAAA TATTTGAGGG AAAAATTAAT AAAATTAATA   
  
  
+ ATAATTATTG TAGAAGTGTA AAAAATGTAA AAAAAAGTAT ACATTCACTC ATAGTGACAA GTTTTACAAC   
  
  
+ TTTCATGAAA ATGTCAGCTT CAAAATCCAA CGCTATAATC CATAAACCTT AAATTTATAT TTAACCGTCG   
  
  
+ ATTGTCATTT ACGCTTTATT CTTCTTATTG AATTTCATTT TTAAAATTTT CTTTTTTGAA AACATGATCA   
  
  
+ TTTGAAGGAT GTAAGAAGAT GAATGGTTTA GATCTTTGAT ATCATGTTTC GATATTTACG GTTAACTGAA   
  
  
+ ATATGAGTCG ATGTCATATA GTTTGTAGAA ACTTTATAAA CATCAAGCAA TATTTTTATT AACCATAAAA   
  
  
+ CTTTGAACAT AATATCAACG ATCCAAATCA TTCATCTTCG TACATCCTCT AATAGATCAT GTTTTCAAAA   
  
  
+ AAGAAAATCT GAAAAAAAAC AAAATTTGAT GAAAAAAATG AAACGTAAAT GTAAACAACA ATCAACGATT   
  
  
+ AAATTTTGAT CTATGATTTA TATGATTATA ATGGCGAATT TCAAAGTTAA GCTCCTCACA AAAGTGGTAA   
  
  
+ AGCTTGTCAT TACAAGCGTT TATAAATTTT TTCTATATTT TTAACACCTC TATATTAATT AAAAAATATT   
  
  
+ AGATTTTGGG CAAACTAAGT GGCGTAAAAC AAAGTGACAA TTACCATTGG CATACCATAA CAACCAAACT   
  
  
+ ATTTGGTATA TTGATATTTA GTATACCGAA AGTTTGGTAC GGTAATGATA ATAGATTTTG CCGTATCAAA   
  
  
+ TATTTGGTAT GGTAATTGGT AGAGAGGTTT TGGTACGGTA ATGTACCAAA ACCACCCCTG TATGCATAGC   
  
  
+ TAAAAACAAA AATAATGAAA TACAAGTAGC TAGTGATATT GTTCTCCTAT TATTAGACAA AGAACCACCA   
  
  
+ AACTAACTGA GCCTTCCATT GCTCTTCCCT CCTTCCACAC ACATTTGACC CTTTCAGCTT TGTCTTGTCT   
  
  
+ CCACAACCCT AACTCCGCCC TTCTTTCTCT ATTTTCCACA TCCATCTTTC CCTTCACCAT GACATGTAAA   
  
  
+ TTTCAGAGGT AAAAAAATTT CTTGGTGGC  

- ACATTCACCA CTTAATGGGA CATTTATTCC GAAAGAAGAA GTTGGATGCC ACAGGGCCCG AGTTTGGAGG   
  
  
- CGAGAGGTAT GAACATCCTA TTTACTTAAA TCGCATTTTT ATTTTTCTCT TTTGTTTTAA CAATTTTTTG   
  
  
- TTGAATTATA GATTTTATGG TTTTATTTAT TGATATGACC CCACCTTTAA AAATGGTTTA ATGGCATGGT   
  
  
- TGGCGGAATG GTTAGTATGG TATGGTTTAA TAATGGTTTT TAAACATGGT TAGCCATTAC CATGCCATAA   
  
  
- ACATGGTATG GTATGATAAA ACTATGCCAT GACCATAGGT TTTGGTCATG GCCACCATAT GGTATGGATG   
  
  
- GTTATAATAA TTATAATATA ATACATAAAT AAGTATATTT ATAAACTCCC TTTTTAATTA TTTTAATTAT   
  
  
- TATTAATAAC ATCTTCACAT TTTTTACATT TTTTTTCATA TGTAAGTGAG TATCACTGTT CAAAATGTTG   
  
  
- AAAGTACTTT TACAGTCGAA GTTTTAGGTT GCGATATTAG GTATTTGGAA TTTAAATATA AATTGGCAGC   
  
  
- TAACAGTAAA TGCGAAATAA GAAGAATAAC TTAAAGTAAA AATTTTAAAA GAAAAAACTT TTGTACTAGT   
  
  
- AAACTTCCTA CATTCTTCTA CTTACCAAAT CTAGAAACTA TAGTACAAAG CTATAAATGC CAATTGACTT   
  
  
- TATACTCAGC TACAGTATAT CAAACATCTT TGAAATATTT GTAGTTCGTT ATAAAAATAA TTGGTATTTT   
  
  
- GAAACTTGTA TTATAGTTGC TAGGTTTAGT AAGTAGAAGC ATGTAGGAGA TTATCTAGTA CAAAAGTTTT   
  
  
- TTCTTTTAGA CTTTTTTTTG TTTTAAACTA CTTTTTTTAC TTTGCATTTA CATTTGTTGT TAGTTGCTAA   
  
  
- TTTAAAACTA GATACTAAAT ATACTAATAT TACCGCTTAA AGTTTCAATT CGAGGAGTGT TTTCACCATT   
  
  
- TCGAACAGTA ATGTTCGCAA ATATTTAAAA AAGATATAAA AATTGTGGAG ATATAATTAA TTTTTTATAA   
  
  
- TCTAAAACCC GTTTGATTCA CCGCATTTTG TTTCACTGTT AATGGTAACC GTATGGTATT GTTGGTTTGA   
  
  
- TAAACCATAT AACTATAAAT CATATGGCTT TCAAACCATG CCATTACTAT TATCTAAAAC GGCATAGTTT   
  
  
- ATAAACCATA CCATTAACCA TCTCTCCAAA ACCATGCCAT TACATGGTTT TGGTGGGGAC ATACGTATCG   
  
  
- ATTTTTGTTT TTATTACTTT ATGTTCATCG ATCACTATAA CAAGAGGATA ATAATCTGTT TCTTGGTGGT   
  
  
- TTGATTGACT CGGAAGGTAA CGAGAAGGGA GGAAGGTGTG TGTAAACTGG GAAAGTCGAA ACAGAACAGA   
  
  
- GGTGTTGGGA TTGAGGCGGG AAGAAAGAGA TAAAAGGTGT AGGTAGAAAG GGAAGTGGTA CTGTACATTT   
  
  
- AAAGTCTCCA TTTTTTTAAA GAACCACCG

+     MYB

| Site Name | Organism | Position | Strand | Matrix score. | sequence | function |
| --- | --- | --- | --- | --- | --- | --- |
| MYB | Arabidopsis thaliana | 1111 | + | 6 | CAACCA |  |
| MYB | Arabidopsis thaliana | 760 | + | 6 | TAACCA |  |

>023445   
+ TGTAAGTGGT GAATTACCCT GTAAATAAGG CTTTCTTCTT CAACCTACGG TGTCCCGGGC TCAAACCTCC   
  
  
+ GCTCTCCATA CTTGTAGGAT AAATGAATTT AGCGTAAAAA TAAAAAGAGA AAACAAAATT GTTAAAAAAC   
  
  
+ AACTTAATAT CTAAAATACC AAAATAAATA ACTATACTGG GGTGGAAATT TTTACCAAAT TACCGTACCA   
  
  
+ ACCGCCTTAC CAATCATACC ATACCAAATT ATTACCAAAA ATTTGTACCA ATCGGTAATG GTACGGTATT   
  
  
+ TGTACCATAC CATACTATTT TGATACGGTA CTGGTATCCA AAACCAGTAC CGGTGGTATA CCATACCTAC   
  
  
+ CAATATTATT AATATTATAT TATGTATTTA TTCATATAAA TATTTGAGGG AAAAATTAAT AAAATTAATA   
  
  
+ ATAATTATTG TAGAAGTGTA AAAAATGTAA AAAAAAGTAT ACATTCACTC ATAGTGACAA GTTTTACAAC   
  
  
+ TTTCATGAAA ATGTCAGCTT CAAAATCCAA CGCTATAATC CATAAACCTT AAATTTATAT TTAACCGTCG   
  
  
+ ATTGTCATTT ACGCTTTATT CTTCTTATTG AATTTCATTT TTAAAATTTT CTTTTTTGAA AACATGATCA   
  
  
+ TTTGAAGGAT GTAAGAAGAT GAATGGTTTA GATCTTTGAT ATCATGTTTC GATATTTACG GTTAACTGAA   
  
  
+ ATATGAGTCG ATGTCATATA GTTTGTAGAA ACTTTATAAA CATCAAGCAA TATTTTTATT AACCATAAAA   
  
  
+ CTTTGAACAT AATATCAACG ATCCAAATCA TTCATCTTCG TACATCCTCT AATAGATCAT GTTTTCAAAA   
  
  
+ AAGAAAATCT GAAAAAAAAC AAAATTTGAT GAAAAAAATG AAACGTAAAT GTAAACAACA ATCAACGATT   
  
  
+ AAATTTTGAT CTATGATTTA TATGATTATA ATGGCGAATT TCAAAGTTAA GCTCCTCACA AAAGTGGTAA   
  
  
+ AGCTTGTCAT TACAAGCGTT TATAAATTTT TTCTATATTT TTAACACCTC TATATTAATT AAAAAATATT   
  
  
+ AGATTTTGGG CAAACTAAGT GGCGTAAAAC AAAGTGACAA TTACCATTGG CATACCATAA CAACCAAACT   
  
  
+ ATTTGGTATA TTGATATTTA GTATACCGAA AGTTTGGTAC GGTAATGATA ATAGATTTTG CCGTATCAAA   
  
  
+ TATTTGGTAT GGTAATTGGT AGAGAGGTTT TGGTACGGTA ATGTACCAAA ACCACCCCTG TATGCATAGC   
  
  
+ TAAAAACAAA AATAATGAAA TACAAGTAGC TAGTGATATT GTTCTCCTAT TATTAGACAA AGAACCACCA   
  
  
+ AACTAACTGA GCCTTCCATT GCTCTTCCCT CCTTCCACAC ACATTTGACC CTTTCAGCTT TGTCTTGTCT   
  
  
+ CCACAACCCT AACTCCGCCC TTCTTTCTCT ATTTTCCACA TCCATCTTTC CCTTCACCAT GACATGTAAA   
  
  
+ TTTCAGAGGT AAAAAAATTT CTTGGTGGC  

- ACATTCACCA CTTAATGGGA CATTTATTCC GAAAGAAGAA GTTGGATGCC ACAGGGCCCG AGTTTGGAGG   
  
  
- CGAGAGGTAT GAACATCCTA TTTACTTAAA TCGCATTTTT ATTTTTCTCT TTTGTTTTAA CAATTTTTTG   
  
  
- TTGAATTATA GATTTTATGG TTTTATTTAT TGATATGACC CCACCTTTAA AAATGGTTTA ATGGCATGGT   
  
  
- TGGCGGAATG GTTAGTATGG TATGGTTTAA TAATGGTTTT TAAACATGGT TAGCCATTAC CATGCCATAA   
  
  
- ACATGGTATG GTATGATAAA ACTATGCCAT GACCATAGGT TTTGGTCATG GCCACCATAT GGTATGGATG   
  
  
- GTTATAATAA TTATAATATA ATACATAAAT AAGTATATTT ATAAACTCCC TTTTTAATTA TTTTAATTAT   
  
  
- TATTAATAAC ATCTTCACAT TTTTTACATT TTTTTTCATA TGTAAGTGAG TATCACTGTT CAAAATGTTG   
  
  
- AAAGTACTTT TACAGTCGAA GTTTTAGGTT GCGATATTAG GTATTTGGAA TTTAAATATA AATTGGCAGC   
  
  
- TAACAGTAAA TGCGAAATAA GAAGAATAAC TTAAAGTAAA AATTTTAAAA GAAAAAACTT TTGTACTAGT   
  
  
- AAACTTCCTA CATTCTTCTA CTTACCAAAT CTAGAAACTA TAGTACAAAG CTATAAATGC CAATTGACTT   
  
  
- TATACTCAGC TACAGTATAT CAAACATCTT TGAAATATTT GTAGTTCGTT ATAAAAATAA TTGGTATTTT   
  
  
- GAAACTTGTA TTATAGTTGC TAGGTTTAGT AAGTAGAAGC ATGTAGGAGA TTATCTAGTA CAAAAGTTTT   
  
  
- TTCTTTTAGA CTTTTTTTTG TTTTAAACTA CTTTTTTTAC TTTGCATTTA CATTTGTTGT TAGTTGCTAA   
  
  
- TTTAAAACTA GATACTAAAT ATACTAATAT TACCGCTTAA AGTTTCAATT CGAGGAGTGT TTTCACCATT   
  
  
- TCGAACAGTA ATGTTCGCAA ATATTTAAAA AAGATATAAA AATTGTGGAG ATATAATTAA TTTTTTATAA   
  
  
- TCTAAAACCC GTTTGATTCA CCGCATTTTG TTTCACTGTT AATGGTAACC GTATGGTATT GTTGGTTTGA   
  
  
- TAAACCATAT AACTATAAAT CATATGGCTT TCAAACCATG CCATTACTAT TATCTAAAAC GGCATAGTTT   
  
  
- ATAAACCATA CCATTAACCA TCTCTCCAAA ACCATGCCAT TACATGGTTT TGGTGGGGAC ATACGTATCG   
  
  
- ATTTTTGTTT TTATTACTTT ATGTTCATCG ATCACTATAA CAAGAGGATA ATAATCTGTT TCTTGGTGGT   
  
  
- TTGATTGACT CGGAAGGTAA CGAGAAGGGA GGAAGGTGTG TGTAAACTGG GAAAGTCGAA ACAGAACAGA   
  
  
- GGTGTTGGGA TTGAGGCGGG AAGAAAGAGA TAAAAGGTGT AGGTAGAAAG GGAAGTGGTA CTGTACATTT   
  
  
- AAAGTCTCCA TTTTTTTAAA GAACCACCG

+     MYB-like sequence

| Site Name | Organism | Position | Strand | Matrix score. | sequence | function |
| --- | --- | --- | --- | --- | --- | --- |
| MYB-like sequence | Arabidopsis thaliana | 760 | + | 6 | TAACCA |  |

>023445   
+ TGTAAGTGGT GAATTACCCT GTAAATAAGG CTTTCTTCTT CAACCTACGG TGTCCCGGGC TCAAACCTCC   
  
  
+ GCTCTCCATA CTTGTAGGAT AAATGAATTT AGCGTAAAAA TAAAAAGAGA AAACAAAATT GTTAAAAAAC   
  
  
+ AACTTAATAT CTAAAATACC AAAATAAATA ACTATACTGG GGTGGAAATT TTTACCAAAT TACCGTACCA   
  
  
+ ACCGCCTTAC CAATCATACC ATACCAAATT ATTACCAAAA ATTTGTACCA ATCGGTAATG GTACGGTATT   
  
  
+ TGTACCATAC CATACTATTT TGATACGGTA CTGGTATCCA AAACCAGTAC CGGTGGTATA CCATACCTAC   
  
  
+ CAATATTATT AATATTATAT TATGTATTTA TTCATATAAA TATTTGAGGG AAAAATTAAT AAAATTAATA   
  
  
+ ATAATTATTG TAGAAGTGTA AAAAATGTAA AAAAAAGTAT ACATTCACTC ATAGTGACAA GTTTTACAAC   
  
  
+ TTTCATGAAA ATGTCAGCTT CAAAATCCAA CGCTATAATC CATAAACCTT AAATTTATAT TTAACCGTCG   
  
  
+ ATTGTCATTT ACGCTTTATT CTTCTTATTG AATTTCATTT TTAAAATTTT CTTTTTTGAA AACATGATCA   
  
  
+ TTTGAAGGAT GTAAGAAGAT GAATGGTTTA GATCTTTGAT ATCATGTTTC GATATTTACG GTTAACTGAA   
  
  
+ ATATGAGTCG ATGTCATATA GTTTGTAGAA ACTTTATAAA CATCAAGCAA TATTTTTATT AACCATAAAA   
  
  
+ CTTTGAACAT AATATCAACG ATCCAAATCA TTCATCTTCG TACATCCTCT AATAGATCAT GTTTTCAAAA   
  
  
+ AAGAAAATCT GAAAAAAAAC AAAATTTGAT GAAAAAAATG AAACGTAAAT GTAAACAACA ATCAACGATT   
  
  
+ AAATTTTGAT CTATGATTTA TATGATTATA ATGGCGAATT TCAAAGTTAA GCTCCTCACA AAAGTGGTAA   
  
  
+ AGCTTGTCAT TACAAGCGTT TATAAATTTT TTCTATATTT TTAACACCTC TATATTAATT AAAAAATATT   
  
  
+ AGATTTTGGG CAAACTAAGT GGCGTAAAAC AAAGTGACAA TTACCATTGG CATACCATAA CAACCAAACT   
  
  
+ ATTTGGTATA TTGATATTTA GTATACCGAA AGTTTGGTAC GGTAATGATA ATAGATTTTG CCGTATCAAA   
  
  
+ TATTTGGTAT GGTAATTGGT AGAGAGGTTT TGGTACGGTA ATGTACCAAA ACCACCCCTG TATGCATAGC   
  
  
+ TAAAAACAAA AATAATGAAA TACAAGTAGC TAGTGATATT GTTCTCCTAT TATTAGACAA AGAACCACCA   
  
  
+ AACTAACTGA GCCTTCCATT GCTCTTCCCT CCTTCCACAC ACATTTGACC CTTTCAGCTT TGTCTTGTCT   
  
  
+ CCACAACCCT AACTCCGCCC TTCTTTCTCT ATTTTCCACA TCCATCTTTC CCTTCACCAT GACATGTAAA   
  
  
+ TTTCAGAGGT AAAAAAATTT CTTGGTGGC  

- ACATTCACCA CTTAATGGGA CATTTATTCC GAAAGAAGAA GTTGGATGCC ACAGGGCCCG AGTTTGGAGG   
  
  
- CGAGAGGTAT GAACATCCTA TTTACTTAAA TCGCATTTTT ATTTTTCTCT TTTGTTTTAA CAATTTTTTG   
  
  
- TTGAATTATA GATTTTATGG TTTTATTTAT TGATATGACC CCACCTTTAA AAATGGTTTA ATGGCATGGT   
  
  
- TGGCGGAATG GTTAGTATGG TATGGTTTAA TAATGGTTTT TAAACATGGT TAGCCATTAC CATGCCATAA   
  
  
- ACATGGTATG GTATGATAAA ACTATGCCAT GACCATAGGT TTTGGTCATG GCCACCATAT GGTATGGATG   
  
  
- GTTATAATAA TTATAATATA ATACATAAAT AAGTATATTT ATAAACTCCC TTTTTAATTA TTTTAATTAT   
  
  
- TATTAATAAC ATCTTCACAT TTTTTACATT TTTTTTCATA TGTAAGTGAG TATCACTGTT CAAAATGTTG   
  
  
- AAAGTACTTT TACAGTCGAA GTTTTAGGTT GCGATATTAG GTATTTGGAA TTTAAATATA AATTGGCAGC   
  
  
- TAACAGTAAA TGCGAAATAA GAAGAATAAC TTAAAGTAAA AATTTTAAAA GAAAAAACTT TTGTACTAGT   
  
  
- AAACTTCCTA CATTCTTCTA CTTACCAAAT CTAGAAACTA TAGTACAAAG CTATAAATGC CAATTGACTT   
  
  
- TATACTCAGC TACAGTATAT CAAACATCTT TGAAATATTT GTAGTTCGTT ATAAAAATAA TTGGTATTTT   
  
  
- GAAACTTGTA TTATAGTTGC TAGGTTTAGT AAGTAGAAGC ATGTAGGAGA TTATCTAGTA CAAAAGTTTT   
  
  
- TTCTTTTAGA CTTTTTTTTG TTTTAAACTA CTTTTTTTAC TTTGCATTTA CATTTGTTGT TAGTTGCTAA   
  
  
- TTTAAAACTA GATACTAAAT ATACTAATAT TACCGCTTAA AGTTTCAATT CGAGGAGTGT TTTCACCATT   
  
  
- TCGAACAGTA ATGTTCGCAA ATATTTAAAA AAGATATAAA AATTGTGGAG ATATAATTAA TTTTTTATAA   
  
  
- TCTAAAACCC GTTTGATTCA CCGCATTTTG TTTCACTGTT AATGGTAACC GTATGGTATT GTTGGTTTGA   
  
  
- TAAACCATAT AACTATAAAT CATATGGCTT TCAAACCATG CCATTACTAT TATCTAAAAC GGCATAGTTT   
  
  
- ATAAACCATA CCATTAACCA TCTCTCCAAA ACCATGCCAT TACATGGTTT TGGTGGGGAC ATACGTATCG   
  
  
- ATTTTTGTTT TTATTACTTT ATGTTCATCG ATCACTATAA CAAGAGGATA ATAATCTGTT TCTTGGTGGT   
  
  
- TTGATTGACT CGGAAGGTAA CGAGAAGGGA GGAAGGTGTG TGTAAACTGG GAAAGTCGAA ACAGAACAGA   
  
  
- GGTGTTGGGA TTGAGGCGGG AAGAAAGAGA TAAAAGGTGT AGGTAGAAAG GGAAGTGGTA CTGTACATTT   
  
  
- AAAGTCTCCA TTTTTTTAAA GAACCACCG

+     MYC

| Site Name | Organism | Position | Strand | Matrix score. | sequence | function |
| --- | --- | --- | --- | --- | --- | --- |
| MYC | Arabidopsis thaliana | 1372 | + | 6 | CATTTG |  |
| MYC | Arabidopsis thaliana | 629 | + | 6 | CATTTG |  |

>023445   
+ TGTAAGTGGT GAATTACCCT GTAAATAAGG CTTTCTTCTT CAACCTACGG TGTCCCGGGC TCAAACCTCC   
  
  
+ GCTCTCCATA CTTGTAGGAT AAATGAATTT AGCGTAAAAA TAAAAAGAGA AAACAAAATT GTTAAAAAAC   
  
  
+ AACTTAATAT CTAAAATACC AAAATAAATA ACTATACTGG GGTGGAAATT TTTACCAAAT TACCGTACCA   
  
  
+ ACCGCCTTAC CAATCATACC ATACCAAATT ATTACCAAAA ATTTGTACCA ATCGGTAATG GTACGGTATT   
  
  
+ TGTACCATAC CATACTATTT TGATACGGTA CTGGTATCCA AAACCAGTAC CGGTGGTATA CCATACCTAC   
  
  
+ CAATATTATT AATATTATAT TATGTATTTA TTCATATAAA TATTTGAGGG AAAAATTAAT AAAATTAATA   
  
  
+ ATAATTATTG TAGAAGTGTA AAAAATGTAA AAAAAAGTAT ACATTCACTC ATAGTGACAA GTTTTACAAC   
  
  
+ TTTCATGAAA ATGTCAGCTT CAAAATCCAA CGCTATAATC CATAAACCTT AAATTTATAT TTAACCGTCG   
  
  
+ ATTGTCATTT ACGCTTTATT CTTCTTATTG AATTTCATTT TTAAAATTTT CTTTTTTGAA AACATGATCA   
  
  
+ TTTGAAGGAT GTAAGAAGAT GAATGGTTTA GATCTTTGAT ATCATGTTTC GATATTTACG GTTAACTGAA   
  
  
+ ATATGAGTCG ATGTCATATA GTTTGTAGAA ACTTTATAAA CATCAAGCAA TATTTTTATT AACCATAAAA   
  
  
+ CTTTGAACAT AATATCAACG ATCCAAATCA TTCATCTTCG TACATCCTCT AATAGATCAT GTTTTCAAAA   
  
  
+ AAGAAAATCT GAAAAAAAAC AAAATTTGAT GAAAAAAATG AAACGTAAAT GTAAACAACA ATCAACGATT   
  
  
+ AAATTTTGAT CTATGATTTA TATGATTATA ATGGCGAATT TCAAAGTTAA GCTCCTCACA AAAGTGGTAA   
  
  
+ AGCTTGTCAT TACAAGCGTT TATAAATTTT TTCTATATTT TTAACACCTC TATATTAATT AAAAAATATT   
  
  
+ AGATTTTGGG CAAACTAAGT GGCGTAAAAC AAAGTGACAA TTACCATTGG CATACCATAA CAACCAAACT   
  
  
+ ATTTGGTATA TTGATATTTA GTATACCGAA AGTTTGGTAC GGTAATGATA ATAGATTTTG CCGTATCAAA   
  
  
+ TATTTGGTAT GGTAATTGGT AGAGAGGTTT TGGTACGGTA ATGTACCAAA ACCACCCCTG TATGCATAGC   
  
  
+ TAAAAACAAA AATAATGAAA TACAAGTAGC TAGTGATATT GTTCTCCTAT TATTAGACAA AGAACCACCA   
  
  
+ AACTAACTGA GCCTTCCATT GCTCTTCCCT CCTTCCACAC ACATTTGACC CTTTCAGCTT TGTCTTGTCT   
  
  
+ CCACAACCCT AACTCCGCCC TTCTTTCTCT ATTTTCCACA TCCATCTTTC CCTTCACCAT GACATGTAAA   
  
  
+ TTTCAGAGGT AAAAAAATTT CTTGGTGGC  

- ACATTCACCA CTTAATGGGA CATTTATTCC GAAAGAAGAA GTTGGATGCC ACAGGGCCCG AGTTTGGAGG   
  
  
- CGAGAGGTAT GAACATCCTA TTTACTTAAA TCGCATTTTT ATTTTTCTCT TTTGTTTTAA CAATTTTTTG   
  
  
- TTGAATTATA GATTTTATGG TTTTATTTAT TGATATGACC CCACCTTTAA AAATGGTTTA ATGGCATGGT   
  
  
- TGGCGGAATG GTTAGTATGG TATGGTTTAA TAATGGTTTT TAAACATGGT TAGCCATTAC CATGCCATAA   
  
  
- ACATGGTATG GTATGATAAA ACTATGCCAT GACCATAGGT TTTGGTCATG GCCACCATAT GGTATGGATG   
  
  
- GTTATAATAA TTATAATATA ATACATAAAT AAGTATATTT ATAAACTCCC TTTTTAATTA TTTTAATTAT   
  
  
- TATTAATAAC ATCTTCACAT TTTTTACATT TTTTTTCATA TGTAAGTGAG TATCACTGTT CAAAATGTTG   
  
  
- AAAGTACTTT TACAGTCGAA GTTTTAGGTT GCGATATTAG GTATTTGGAA TTTAAATATA AATTGGCAGC   
  
  
- TAACAGTAAA TGCGAAATAA GAAGAATAAC TTAAAGTAAA AATTTTAAAA GAAAAAACTT TTGTACTAGT   
  
  
- AAACTTCCTA CATTCTTCTA CTTACCAAAT CTAGAAACTA TAGTACAAAG CTATAAATGC CAATTGACTT   
  
  
- TATACTCAGC TACAGTATAT CAAACATCTT TGAAATATTT GTAGTTCGTT ATAAAAATAA TTGGTATTTT   
  
  
- GAAACTTGTA TTATAGTTGC TAGGTTTAGT AAGTAGAAGC ATGTAGGAGA TTATCTAGTA CAAAAGTTTT   
  
  
- TTCTTTTAGA CTTTTTTTTG TTTTAAACTA CTTTTTTTAC TTTGCATTTA CATTTGTTGT TAGTTGCTAA   
  
  
- TTTAAAACTA GATACTAAAT ATACTAATAT TACCGCTTAA AGTTTCAATT CGAGGAGTGT TTTCACCATT   
  
  
- TCGAACAGTA ATGTTCGCAA ATATTTAAAA AAGATATAAA AATTGTGGAG ATATAATTAA TTTTTTATAA   
  
  
- TCTAAAACCC GTTTGATTCA CCGCATTTTG TTTCACTGTT AATGGTAACC GTATGGTATT GTTGGTTTGA   
  
  
- TAAACCATAT AACTATAAAT CATATGGCTT TCAAACCATG CCATTACTAT TATCTAAAAC GGCATAGTTT   
  
  
- ATAAACCATA CCATTAACCA TCTCTCCAAA ACCATGCCAT TACATGGTTT TGGTGGGGAC ATACGTATCG   
  
  
- ATTTTTGTTT TTATTACTTT ATGTTCATCG ATCACTATAA CAAGAGGATA ATAATCTGTT TCTTGGTGGT   
  
  
- TTGATTGACT CGGAAGGTAA CGAGAAGGGA GGAAGGTGTG TGTAAACTGG GAAAGTCGAA ACAGAACAGA   
  
  
- GGTGTTGGGA TTGAGGCGGG AAGAAAGAGA TAAAAGGTGT AGGTAGAAAG GGAAGTGGTA CTGTACATTT   
  
  
- AAAGTCTCCA TTTTTTTAAA GAACCACCG

+     Myb

| Site Name | Organism | Position | Strand | Matrix score. | sequence | function |
| --- | --- | --- | --- | --- | --- | --- |
| Myb | Arabidopsis thaliana | 693 | + | 6 | TAACTG |  |
| Myb | Arabidopsis thaliana | 1334 | + | 6 | TAACTG |  |

>023445   
+ TGTAAGTGGT GAATTACCCT GTAAATAAGG CTTTCTTCTT CAACCTACGG TGTCCCGGGC TCAAACCTCC   
  
  
+ GCTCTCCATA CTTGTAGGAT AAATGAATTT AGCGTAAAAA TAAAAAGAGA AAACAAAATT GTTAAAAAAC   
  
  
+ AACTTAATAT CTAAAATACC AAAATAAATA ACTATACTGG GGTGGAAATT TTTACCAAAT TACCGTACCA   
  
  
+ ACCGCCTTAC CAATCATACC ATACCAAATT ATTACCAAAA ATTTGTACCA ATCGGTAATG GTACGGTATT   
  
  
+ TGTACCATAC CATACTATTT TGATACGGTA CTGGTATCCA AAACCAGTAC CGGTGGTATA CCATACCTAC   
  
  
+ CAATATTATT AATATTATAT TATGTATTTA TTCATATAAA TATTTGAGGG AAAAATTAAT AAAATTAATA   
  
  
+ ATAATTATTG TAGAAGTGTA AAAAATGTAA AAAAAAGTAT ACATTCACTC ATAGTGACAA GTTTTACAAC   
  
  
+ TTTCATGAAA ATGTCAGCTT CAAAATCCAA CGCTATAATC CATAAACCTT AAATTTATAT TTAACCGTCG   
  
  
+ ATTGTCATTT ACGCTTTATT CTTCTTATTG AATTTCATTT TTAAAATTTT CTTTTTTGAA AACATGATCA   
  
  
+ TTTGAAGGAT GTAAGAAGAT GAATGGTTTA GATCTTTGAT ATCATGTTTC GATATTTACG GTTAACTGAA   
  
  
+ ATATGAGTCG ATGTCATATA GTTTGTAGAA ACTTTATAAA CATCAAGCAA TATTTTTATT AACCATAAAA   
  
  
+ CTTTGAACAT AATATCAACG ATCCAAATCA TTCATCTTCG TACATCCTCT AATAGATCAT GTTTTCAAAA   
  
  
+ AAGAAAATCT GAAAAAAAAC AAAATTTGAT GAAAAAAATG AAACGTAAAT GTAAACAACA ATCAACGATT   
  
  
+ AAATTTTGAT CTATGATTTA TATGATTATA ATGGCGAATT TCAAAGTTAA GCTCCTCACA AAAGTGGTAA   
  
  
+ AGCTTGTCAT TACAAGCGTT TATAAATTTT TTCTATATTT TTAACACCTC TATATTAATT AAAAAATATT   
  
  
+ AGATTTTGGG CAAACTAAGT GGCGTAAAAC AAAGTGACAA TTACCATTGG CATACCATAA CAACCAAACT   
  
  
+ ATTTGGTATA TTGATATTTA GTATACCGAA AGTTTGGTAC GGTAATGATA ATAGATTTTG CCGTATCAAA   
  
  
+ TATTTGGTAT GGTAATTGGT AGAGAGGTTT TGGTACGGTA ATGTACCAAA ACCACCCCTG TATGCATAGC   
  
  
+ TAAAAACAAA AATAATGAAA TACAAGTAGC TAGTGATATT GTTCTCCTAT TATTAGACAA AGAACCACCA   
  
  
+ AACTAACTGA GCCTTCCATT GCTCTTCCCT CCTTCCACAC ACATTTGACC CTTTCAGCTT TGTCTTGTCT   
  
  
+ CCACAACCCT AACTCCGCCC TTCTTTCTCT ATTTTCCACA TCCATCTTTC CCTTCACCAT GACATGTAAA   
  
  
+ TTTCAGAGGT AAAAAAATTT CTTGGTGGC  

- ACATTCACCA CTTAATGGGA CATTTATTCC GAAAGAAGAA GTTGGATGCC ACAGGGCCCG AGTTTGGAGG   
  
  
- CGAGAGGTAT GAACATCCTA TTTACTTAAA TCGCATTTTT ATTTTTCTCT TTTGTTTTAA CAATTTTTTG   
  
  
- TTGAATTATA GATTTTATGG TTTTATTTAT TGATATGACC CCACCTTTAA AAATGGTTTA ATGGCATGGT   
  
  
- TGGCGGAATG GTTAGTATGG TATGGTTTAA TAATGGTTTT TAAACATGGT TAGCCATTAC CATGCCATAA   
  
  
- ACATGGTATG GTATGATAAA ACTATGCCAT GACCATAGGT TTTGGTCATG GCCACCATAT GGTATGGATG   
  
  
- GTTATAATAA TTATAATATA ATACATAAAT AAGTATATTT ATAAACTCCC TTTTTAATTA TTTTAATTAT   
  
  
- TATTAATAAC ATCTTCACAT TTTTTACATT TTTTTTCATA TGTAAGTGAG TATCACTGTT CAAAATGTTG   
  
  
- AAAGTACTTT TACAGTCGAA GTTTTAGGTT GCGATATTAG GTATTTGGAA TTTAAATATA AATTGGCAGC   
  
  
- TAACAGTAAA TGCGAAATAA GAAGAATAAC TTAAAGTAAA AATTTTAAAA GAAAAAACTT TTGTACTAGT   
  
  
- AAACTTCCTA CATTCTTCTA CTTACCAAAT CTAGAAACTA TAGTACAAAG CTATAAATGC CAATTGACTT   
  
  
- TATACTCAGC TACAGTATAT CAAACATCTT TGAAATATTT GTAGTTCGTT ATAAAAATAA TTGGTATTTT   
  
  
- GAAACTTGTA TTATAGTTGC TAGGTTTAGT AAGTAGAAGC ATGTAGGAGA TTATCTAGTA CAAAAGTTTT   
  
  
- TTCTTTTAGA CTTTTTTTTG TTTTAAACTA CTTTTTTTAC TTTGCATTTA CATTTGTTGT TAGTTGCTAA   
  
  
- TTTAAAACTA GATACTAAAT ATACTAATAT TACCGCTTAA AGTTTCAATT CGAGGAGTGT TTTCACCATT   
  
  
- TCGAACAGTA ATGTTCGCAA ATATTTAAAA AAGATATAAA AATTGTGGAG ATATAATTAA TTTTTTATAA   
  
  
- TCTAAAACCC GTTTGATTCA CCGCATTTTG TTTCACTGTT AATGGTAACC GTATGGTATT GTTGGTTTGA   
  
  
- TAAACCATAT AACTATAAAT CATATGGCTT TCAAACCATG CCATTACTAT TATCTAAAAC GGCATAGTTT   
  
  
- ATAAACCATA CCATTAACCA TCTCTCCAAA ACCATGCCAT TACATGGTTT TGGTGGGGAC ATACGTATCG   
  
  
- ATTTTTGTTT TTATTACTTT ATGTTCATCG ATCACTATAA CAAGAGGATA ATAATCTGTT TCTTGGTGGT   
  
  
- TTGATTGACT CGGAAGGTAA CGAGAAGGGA GGAAGGTGTG TGTAAACTGG GAAAGTCGAA ACAGAACAGA   
  
  
- GGTGTTGGGA TTGAGGCGGG AAGAAAGAGA TAAAAGGTGT AGGTAGAAAG GGAAGTGGTA CTGTACATTT   
  
  
- AAAGTCTCCA TTTTTTTAAA GAACCACCG

+     STRE

| Site Name | Organism | Position | Strand | Matrix score. | sequence | function |
| --- | --- | --- | --- | --- | --- | --- |
| STRE | Arabidopsis thaliana | 1245 | - | 5 | AGGGG |  |

>023445   
+ TGTAAGTGGT GAATTACCCT GTAAATAAGG CTTTCTTCTT CAACCTACGG TGTCCCGGGC TCAAACCTCC   
  
  
+ GCTCTCCATA CTTGTAGGAT AAATGAATTT AGCGTAAAAA TAAAAAGAGA AAACAAAATT GTTAAAAAAC   
  
  
+ AACTTAATAT CTAAAATACC AAAATAAATA ACTATACTGG GGTGGAAATT TTTACCAAAT TACCGTACCA   
  
  
+ ACCGCCTTAC CAATCATACC ATACCAAATT ATTACCAAAA ATTTGTACCA ATCGGTAATG GTACGGTATT   
  
  
+ TGTACCATAC CATACTATTT TGATACGGTA CTGGTATCCA AAACCAGTAC CGGTGGTATA CCATACCTAC   
  
  
+ CAATATTATT AATATTATAT TATGTATTTA TTCATATAAA TATTTGAGGG AAAAATTAAT AAAATTAATA   
  
  
+ ATAATTATTG TAGAAGTGTA AAAAATGTAA AAAAAAGTAT ACATTCACTC ATAGTGACAA GTTTTACAAC   
  
  
+ TTTCATGAAA ATGTCAGCTT CAAAATCCAA CGCTATAATC CATAAACCTT AAATTTATAT TTAACCGTCG   
  
  
+ ATTGTCATTT ACGCTTTATT CTTCTTATTG AATTTCATTT TTAAAATTTT CTTTTTTGAA AACATGATCA   
  
  
+ TTTGAAGGAT GTAAGAAGAT GAATGGTTTA GATCTTTGAT ATCATGTTTC GATATTTACG GTTAACTGAA   
  
  
+ ATATGAGTCG ATGTCATATA GTTTGTAGAA ACTTTATAAA CATCAAGCAA TATTTTTATT AACCATAAAA   
  
  
+ CTTTGAACAT AATATCAACG ATCCAAATCA TTCATCTTCG TACATCCTCT AATAGATCAT GTTTTCAAAA   
  
  
+ AAGAAAATCT GAAAAAAAAC AAAATTTGAT GAAAAAAATG AAACGTAAAT GTAAACAACA ATCAACGATT   
  
  
+ AAATTTTGAT CTATGATTTA TATGATTATA ATGGCGAATT TCAAAGTTAA GCTCCTCACA AAAGTGGTAA   
  
  
+ AGCTTGTCAT TACAAGCGTT TATAAATTTT TTCTATATTT TTAACACCTC TATATTAATT AAAAAATATT   
  
  
+ AGATTTTGGG CAAACTAAGT GGCGTAAAAC AAAGTGACAA TTACCATTGG CATACCATAA CAACCAAACT   
  
  
+ ATTTGGTATA TTGATATTTA GTATACCGAA AGTTTGGTAC GGTAATGATA ATAGATTTTG CCGTATCAAA   
  
  
+ TATTTGGTAT GGTAATTGGT AGAGAGGTTT TGGTACGGTA ATGTACCAAA ACCACCCCTG TATGCATAGC   
  
  
+ TAAAAACAAA AATAATGAAA TACAAGTAGC TAGTGATATT GTTCTCCTAT TATTAGACAA AGAACCACCA   
  
  
+ AACTAACTGA GCCTTCCATT GCTCTTCCCT CCTTCCACAC ACATTTGACC CTTTCAGCTT TGTCTTGTCT   
  
  
+ CCACAACCCT AACTCCGCCC TTCTTTCTCT ATTTTCCACA TCCATCTTTC CCTTCACCAT GACATGTAAA   
  
  
+ TTTCAGAGGT AAAAAAATTT CTTGGTGGC  

- ACATTCACCA CTTAATGGGA CATTTATTCC GAAAGAAGAA GTTGGATGCC ACAGGGCCCG AGTTTGGAGG   
  
  
- CGAGAGGTAT GAACATCCTA TTTACTTAAA TCGCATTTTT ATTTTTCTCT TTTGTTTTAA CAATTTTTTG   
  
  
- TTGAATTATA GATTTTATGG TTTTATTTAT TGATATGACC CCACCTTTAA AAATGGTTTA ATGGCATGGT   
  
  
- TGGCGGAATG GTTAGTATGG TATGGTTTAA TAATGGTTTT TAAACATGGT TAGCCATTAC CATGCCATAA   
  
  
- ACATGGTATG GTATGATAAA ACTATGCCAT GACCATAGGT TTTGGTCATG GCCACCATAT GGTATGGATG   
  
  
- GTTATAATAA TTATAATATA ATACATAAAT AAGTATATTT ATAAACTCCC TTTTTAATTA TTTTAATTAT   
  
  
- TATTAATAAC ATCTTCACAT TTTTTACATT TTTTTTCATA TGTAAGTGAG TATCACTGTT CAAAATGTTG   
  
  
- AAAGTACTTT TACAGTCGAA GTTTTAGGTT GCGATATTAG GTATTTGGAA TTTAAATATA AATTGGCAGC   
  
  
- TAACAGTAAA TGCGAAATAA GAAGAATAAC TTAAAGTAAA AATTTTAAAA GAAAAAACTT TTGTACTAGT   
  
  
- AAACTTCCTA CATTCTTCTA CTTACCAAAT CTAGAAACTA TAGTACAAAG CTATAAATGC CAATTGACTT   
  
  
- TATACTCAGC TACAGTATAT CAAACATCTT TGAAATATTT GTAGTTCGTT ATAAAAATAA TTGGTATTTT   
  
  
- GAAACTTGTA TTATAGTTGC TAGGTTTAGT AAGTAGAAGC ATGTAGGAGA TTATCTAGTA CAAAAGTTTT   
  
  
- TTCTTTTAGA CTTTTTTTTG TTTTAAACTA CTTTTTTTAC TTTGCATTTA CATTTGTTGT TAGTTGCTAA   
  
  
- TTTAAAACTA GATACTAAAT ATACTAATAT TACCGCTTAA AGTTTCAATT CGAGGAGTGT TTTCACCATT   
  
  
- TCGAACAGTA ATGTTCGCAA ATATTTAAAA AAGATATAAA AATTGTGGAG ATATAATTAA TTTTTTATAA   
  
  
- TCTAAAACCC GTTTGATTCA CCGCATTTTG TTTCACTGTT AATGGTAACC GTATGGTATT GTTGGTTTGA   
  
  
- TAAACCATAT AACTATAAAT CATATGGCTT TCAAACCATG CCATTACTAT TATCTAAAAC GGCATAGTTT   
  
  
- ATAAACCATA CCATTAACCA TCTCTCCAAA ACCATGCCAT TACATGGTTT TGGTGGGGAC ATACGTATCG   
  
  
- ATTTTTGTTT TTATTACTTT ATGTTCATCG ATCACTATAA CAAGAGGATA ATAATCTGTT TCTTGGTGGT   
  
  
- TTGATTGACT CGGAAGGTAA CGAGAAGGGA GGAAGGTGTG TGTAAACTGG GAAAGTCGAA ACAGAACAGA   
  
  
- GGTGTTGGGA TTGAGGCGGG AAGAAAGAGA TAAAAGGTGT AGGTAGAAAG GGAAGTGGTA CTGTACATTT   
  
  
- AAAGTCTCCA TTTTTTTAAA GAACCACCG

+     Sp1

| Site Name | Organism | Position | Strand | Matrix score. | sequence | function |
| --- | --- | --- | --- | --- | --- | --- |
| Sp1 | Oryza sativa | 1415 | - | 6 | GGGCGG | light responsive element |

>023445   
+ TGTAAGTGGT GAATTACCCT GTAAATAAGG CTTTCTTCTT CAACCTACGG TGTCCCGGGC TCAAACCTCC   
  
  
+ GCTCTCCATA CTTGTAGGAT AAATGAATTT AGCGTAAAAA TAAAAAGAGA AAACAAAATT GTTAAAAAAC   
  
  
+ AACTTAATAT CTAAAATACC AAAATAAATA ACTATACTGG GGTGGAAATT TTTACCAAAT TACCGTACCA   
  
  
+ ACCGCCTTAC CAATCATACC ATACCAAATT ATTACCAAAA ATTTGTACCA ATCGGTAATG GTACGGTATT   
  
  
+ TGTACCATAC CATACTATTT TGATACGGTA CTGGTATCCA AAACCAGTAC CGGTGGTATA CCATACCTAC   
  
  
+ CAATATTATT AATATTATAT TATGTATTTA TTCATATAAA TATTTGAGGG AAAAATTAAT AAAATTAATA   
  
  
+ ATAATTATTG TAGAAGTGTA AAAAATGTAA AAAAAAGTAT ACATTCACTC ATAGTGACAA GTTTTACAAC   
  
  
+ TTTCATGAAA ATGTCAGCTT CAAAATCCAA CGCTATAATC CATAAACCTT AAATTTATAT TTAACCGTCG   
  
  
+ ATTGTCATTT ACGCTTTATT CTTCTTATTG AATTTCATTT TTAAAATTTT CTTTTTTGAA AACATGATCA   
  
  
+ TTTGAAGGAT GTAAGAAGAT GAATGGTTTA GATCTTTGAT ATCATGTTTC GATATTTACG GTTAACTGAA   
  
  
+ ATATGAGTCG ATGTCATATA GTTTGTAGAA ACTTTATAAA CATCAAGCAA TATTTTTATT AACCATAAAA   
  
  
+ CTTTGAACAT AATATCAACG ATCCAAATCA TTCATCTTCG TACATCCTCT AATAGATCAT GTTTTCAAAA   
  
  
+ AAGAAAATCT GAAAAAAAAC AAAATTTGAT GAAAAAAATG AAACGTAAAT GTAAACAACA ATCAACGATT   
  
  
+ AAATTTTGAT CTATGATTTA TATGATTATA ATGGCGAATT TCAAAGTTAA GCTCCTCACA AAAGTGGTAA   
  
  
+ AGCTTGTCAT TACAAGCGTT TATAAATTTT TTCTATATTT TTAACACCTC TATATTAATT AAAAAATATT   
  
  
+ AGATTTTGGG CAAACTAAGT GGCGTAAAAC AAAGTGACAA TTACCATTGG CATACCATAA CAACCAAACT   
  
  
+ ATTTGGTATA TTGATATTTA GTATACCGAA AGTTTGGTAC GGTAATGATA ATAGATTTTG CCGTATCAAA   
  
  
+ TATTTGGTAT GGTAATTGGT AGAGAGGTTT TGGTACGGTA ATGTACCAAA ACCACCCCTG TATGCATAGC   
  
  
+ TAAAAACAAA AATAATGAAA TACAAGTAGC TAGTGATATT GTTCTCCTAT TATTAGACAA AGAACCACCA   
  
  
+ AACTAACTGA GCCTTCCATT GCTCTTCCCT CCTTCCACAC ACATTTGACC CTTTCAGCTT TGTCTTGTCT   
  
  
+ CCACAACCCT AACTCCGCCC TTCTTTCTCT ATTTTCCACA TCCATCTTTC CCTTCACCAT GACATGTAAA   
  
  
+ TTTCAGAGGT AAAAAAATTT CTTGGTGGC  

- ACATTCACCA CTTAATGGGA CATTTATTCC GAAAGAAGAA GTTGGATGCC ACAGGGCCCG AGTTTGGAGG   
  
  
- CGAGAGGTAT GAACATCCTA TTTACTTAAA TCGCATTTTT ATTTTTCTCT TTTGTTTTAA CAATTTTTTG   
  
  
- TTGAATTATA GATTTTATGG TTTTATTTAT TGATATGACC CCACCTTTAA AAATGGTTTA ATGGCATGGT   
  
  
- TGGCGGAATG GTTAGTATGG TATGGTTTAA TAATGGTTTT TAAACATGGT TAGCCATTAC CATGCCATAA   
  
  
- ACATGGTATG GTATGATAAA ACTATGCCAT GACCATAGGT TTTGGTCATG GCCACCATAT GGTATGGATG   
  
  
- GTTATAATAA TTATAATATA ATACATAAAT AAGTATATTT ATAAACTCCC TTTTTAATTA TTTTAATTAT   
  
  
- TATTAATAAC ATCTTCACAT TTTTTACATT TTTTTTCATA TGTAAGTGAG TATCACTGTT CAAAATGTTG   
  
  
- AAAGTACTTT TACAGTCGAA GTTTTAGGTT GCGATATTAG GTATTTGGAA TTTAAATATA AATTGGCAGC   
  
  
- TAACAGTAAA TGCGAAATAA GAAGAATAAC TTAAAGTAAA AATTTTAAAA GAAAAAACTT TTGTACTAGT   
  
  
- AAACTTCCTA CATTCTTCTA CTTACCAAAT CTAGAAACTA TAGTACAAAG CTATAAATGC CAATTGACTT   
  
  
- TATACTCAGC TACAGTATAT CAAACATCTT TGAAATATTT GTAGTTCGTT ATAAAAATAA TTGGTATTTT   
  
  
- GAAACTTGTA TTATAGTTGC TAGGTTTAGT AAGTAGAAGC ATGTAGGAGA TTATCTAGTA CAAAAGTTTT   
  
  
- TTCTTTTAGA CTTTTTTTTG TTTTAAACTA CTTTTTTTAC TTTGCATTTA CATTTGTTGT TAGTTGCTAA   
  
  
- TTTAAAACTA GATACTAAAT ATACTAATAT TACCGCTTAA AGTTTCAATT CGAGGAGTGT TTTCACCATT   
  
  
- TCGAACAGTA ATGTTCGCAA ATATTTAAAA AAGATATAAA AATTGTGGAG ATATAATTAA TTTTTTATAA   
  
  
- TCTAAAACCC GTTTGATTCA CCGCATTTTG TTTCACTGTT AATGGTAACC GTATGGTATT GTTGGTTTGA   
  
  
- TAAACCATAT AACTATAAAT CATATGGCTT TCAAACCATG CCATTACTAT TATCTAAAAC GGCATAGTTT   
  
  
- ATAAACCATA CCATTAACCA TCTCTCCAAA ACCATGCCAT TACATGGTTT TGGTGGGGAC ATACGTATCG   
  
  
- ATTTTTGTTT TTATTACTTT ATGTTCATCG ATCACTATAA CAAGAGGATA ATAATCTGTT TCTTGGTGGT   
  
  
- TTGATTGACT CGGAAGGTAA CGAGAAGGGA GGAAGGTGTG TGTAAACTGG GAAAGTCGAA ACAGAACAGA   
  
  
- GGTGTTGGGA TTGAGGCGGG AAGAAAGAGA TAAAAGGTGT AGGTAGAAAG GGAAGTGGTA CTGTACATTT   
  
  
- AAAGTCTCCA TTTTTTTAAA GAACCACCG

+     TATA-box

| Site Name | Organism | Position | Strand | Matrix score. | sequence | function |
| --- | --- | --- | --- | --- | --- | --- |
| TATA-box | Arabidopsis thaliana | 1014 | - | 4 | TATA | core promoter element around -30 of transcription start |
| TATA-box | Arabidopsis thaliana | 1001 | - | 4 | TATA | core promoter element around -30 of transcription start |
| TATA-box | Helianthus annuus | 999 | - | 6 | TATAAA | core promoter element around -30 of transcription start |
| TATA-box | Arabidopsis thaliana | 1000 | - | 5 | TATAA | core promoter element around -30 of transcription start |
| TATA-box | Arabidopsis thaliana | 937 | - | 4 | TATA | core promoter element around -30 of transcription start |
| TATA-box | Arabidopsis thaliana | 936 | - | 5 | TATAA | core promoter element around -30 of transcription start |
| TATA-box | Brassica napus | 935 | + | 6 | ATTATA | core promoter element around -30 of transcription start |
| TATA-box | Arabidopsis thaliana | 929 | - | 4 | TATA | core promoter element around -30 of transcription start |
| TATA-box | Arabidopsis thaliana | 928 | - | 5 | TATAA | core promoter element around -30 of transcription start |
| TATA-box | Helianthus annuus | 927 | - | 6 | TATAAA | core promoter element around -30 of transcription start |
| TATA-box | Brassica juncea | 926 | - | 7 | TATAAAT | core promoter element around -30 of transcription start |
| TATA-box | Arabidopsis thaliana | 925 | - | 9 | taTATAAAtc | core promoter element around -30 of transcription start |
| TATA-box | Arabidopsis thaliana | 734 | - | 5 | TATAA | core promoter element around -30 of transcription start |
| TATA-box | Arabidopsis thaliana | 546 | + | 4 | TATA | core promoter element around -30 of transcription start |
| TATA-box | Arabidopsis thaliana | 545 | - | 5 | TATAA | core promoter element around -30 of transcription start |
| TATA-box | Arabidopsis thaliana | 1031 | - | 4 | TATA | core promoter element around -30 of transcription start |
| TATA-box | Helianthus annuus | 544 | - | 6 | TATAAA | core promoter element around -30 of transcription start |
| TATA-box | Avena sativa | 540 | + | 12 | TATATTTATATTT | core promoter element around -30 of transcription start |
| TATA-box | Arabidopsis thaliana | 1142 | - | 4 | TATA | core promoter element around -30 of transcription start |
| TATA-box | Arabidopsis thaliana | 717 | + | 4 | TATA | core promoter element around -30 of transcription start |
| TATA-box | Helianthus annuus | 733 | - | 6 | TATAAA | core promoter element around -30 of transcription start |
| TATA-box | Avena sativa | 1127 | + | 12 | TATATTTATATTT | core promoter element around -30 of transcription start |
| TATA-box | Arabidopsis thaliana | 735 | + | 4 | TATA | core promoter element around -30 of transcription start |
| TATA-box | Brassica juncea | 543 | - | 7 | TATAAAT | core promoter element around -30 of transcription start |
| TATA-box | Arabidopsis thaliana | 524 | + | 4 | TATA | core promoter element around -30 of transcription start |
| TATA-box | Arabidopsis thaliana | 458 | + | 4 | TATA | core promoter element around -30 of transcription start |
| TATA-box | Arabidopsis thaliana | 385 | + | 4 | TATA | core promoter element around -30 of transcription start |
| TATA-box | Brassica oleracea | 384 | + | 6 | ATATAA | core promoter element around -30 of transcription start |
| TATA-box | Arabidopsis thaliana | 366 | + | 4 | TATA | core promoter element around -30 of transcription start |
| TATA-box | Arabidopsis thaliana | 173 | + | 4 | TATA | core promoter element around -30 of transcription start |
| TATA-box | Brassica napus | 364 | + | 6 | ATTATA | core promoter element around -30 of transcription start |
| TATA-box | Arabidopsis thaliana | 337 | + | 4 | TATA | core promoter element around -30 of transcription start |
| TATA-box | Arabidopsis thaliana | 365 | - | 5 | TATAA | core promoter element around -30 of transcription start |

>023445   
+ TGTAAGTGGT GAATTACCCT GTAAATAAGG CTTTCTTCTT CAACCTACGG TGTCCCGGGC TCAAACCTCC   
  
  
+ GCTCTCCATA CTTGTAGGAT AAATGAATTT AGCGTAAAAA TAAAAAGAGA AAACAAAATT GTTAAAAAAC   
  
  
+ AACTTAATAT CTAAAATACC AAAATAAATA ACTATACTGG GGTGGAAATT TTTACCAAAT TACCGTACCA   
  
  
+ ACCGCCTTAC CAATCATACC ATACCAAATT ATTACCAAAA ATTTGTACCA ATCGGTAATG GTACGGTATT   
  
  
+ TGTACCATAC CATACTATTT TGATACGGTA CTGGTATCCA AAACCAGTAC CGGTGGTATA CCATACCTAC   
  
  
+ CAATATTATT AATATTATAT TATGTATTTA TTCATATAAA TATTTGAGGG AAAAATTAAT AAAATTAATA   
  
  
+ ATAATTATTG TAGAAGTGTA AAAAATGTAA AAAAAAGTAT ACATTCACTC ATAGTGACAA GTTTTACAAC   
  
  
+ TTTCATGAAA ATGTCAGCTT CAAAATCCAA CGCTATAATC CATAAACCTT AAATTTATAT TTAACCGTCG   
  
  
+ ATTGTCATTT ACGCTTTATT CTTCTTATTG AATTTCATTT TTAAAATTTT CTTTTTTGAA AACATGATCA   
  
  
+ TTTGAAGGAT GTAAGAAGAT GAATGGTTTA GATCTTTGAT ATCATGTTTC GATATTTACG GTTAACTGAA   
  
  
+ ATATGAGTCG ATGTCATATA GTTTGTAGAA ACTTTATAAA CATCAAGCAA TATTTTTATT AACCATAAAA   
  
  
+ CTTTGAACAT AATATCAACG ATCCAAATCA TTCATCTTCG TACATCCTCT AATAGATCAT GTTTTCAAAA   
  
  
+ AAGAAAATCT GAAAAAAAAC AAAATTTGAT GAAAAAAATG AAACGTAAAT GTAAACAACA ATCAACGATT   
  
  
+ AAATTTTGAT CTATGATTTA TATGATTATA ATGGCGAATT TCAAAGTTAA GCTCCTCACA AAAGTGGTAA   
  
  
+ AGCTTGTCAT TACAAGCGTT TATAAATTTT TTCTATATTT TTAACACCTC TATATTAATT AAAAAATATT   
  
  
+ AGATTTTGGG CAAACTAAGT GGCGTAAAAC AAAGTGACAA TTACCATTGG CATACCATAA CAACCAAACT   
  
  
+ ATTTGGTATA TTGATATTTA GTATACCGAA AGTTTGGTAC GGTAATGATA ATAGATTTTG CCGTATCAAA   
  
  
+ TATTTGGTAT GGTAATTGGT AGAGAGGTTT TGGTACGGTA ATGTACCAAA ACCACCCCTG TATGCATAGC   
  
  
+ TAAAAACAAA AATAATGAAA TACAAGTAGC TAGTGATATT GTTCTCCTAT TATTAGACAA AGAACCACCA   
  
  
+ AACTAACTGA GCCTTCCATT GCTCTTCCCT CCTTCCACAC ACATTTGACC CTTTCAGCTT TGTCTTGTCT   
  
  
+ CCACAACCCT AACTCCGCCC TTCTTTCTCT ATTTTCCACA TCCATCTTTC CCTTCACCAT GACATGTAAA   
  
  
+ TTTCAGAGGT AAAAAAATTT CTTGGTGGC  

- ACATTCACCA CTTAATGGGA CATTTATTCC GAAAGAAGAA GTTGGATGCC ACAGGGCCCG AGTTTGGAGG   
  
  
- CGAGAGGTAT GAACATCCTA TTTACTTAAA TCGCATTTTT ATTTTTCTCT TTTGTTTTAA CAATTTTTTG   
  
  
- TTGAATTATA GATTTTATGG TTTTATTTAT TGATATGACC CCACCTTTAA AAATGGTTTA ATGGCATGGT   
  
  
- TGGCGGAATG GTTAGTATGG TATGGTTTAA TAATGGTTTT TAAACATGGT TAGCCATTAC CATGCCATAA   
  
  
- ACATGGTATG GTATGATAAA ACTATGCCAT GACCATAGGT TTTGGTCATG GCCACCATAT GGTATGGATG   
  
  
- GTTATAATAA TTATAATATA ATACATAAAT AAGTATATTT ATAAACTCCC TTTTTAATTA TTTTAATTAT   
  
  
- TATTAATAAC ATCTTCACAT TTTTTACATT TTTTTTCATA TGTAAGTGAG TATCACTGTT CAAAATGTTG   
  
  
- AAAGTACTTT TACAGTCGAA GTTTTAGGTT GCGATATTAG GTATTTGGAA TTTAAATATA AATTGGCAGC   
  
  
- TAACAGTAAA TGCGAAATAA GAAGAATAAC TTAAAGTAAA AATTTTAAAA GAAAAAACTT TTGTACTAGT   
  
  
- AAACTTCCTA CATTCTTCTA CTTACCAAAT CTAGAAACTA TAGTACAAAG CTATAAATGC CAATTGACTT   
  
  
- TATACTCAGC TACAGTATAT CAAACATCTT TGAAATATTT GTAGTTCGTT ATAAAAATAA TTGGTATTTT   
  
  
- GAAACTTGTA TTATAGTTGC TAGGTTTAGT AAGTAGAAGC ATGTAGGAGA TTATCTAGTA CAAAAGTTTT   
  
  
- TTCTTTTAGA CTTTTTTTTG TTTTAAACTA CTTTTTTTAC TTTGCATTTA CATTTGTTGT TAGTTGCTAA   
  
  
- TTTAAAACTA GATACTAAAT ATACTAATAT TACCGCTTAA AGTTTCAATT CGAGGAGTGT TTTCACCATT   
  
  
- TCGAACAGTA ATGTTCGCAA ATATTTAAAA AAGATATAAA AATTGTGGAG ATATAATTAA TTTTTTATAA   
  
  
- TCTAAAACCC GTTTGATTCA CCGCATTTTG TTTCACTGTT AATGGTAACC GTATGGTATT GTTGGTTTGA   
  
  
- TAAACCATAT AACTATAAAT CATATGGCTT TCAAACCATG CCATTACTAT TATCTAAAAC GGCATAGTTT   
  
  
- ATAAACCATA CCATTAACCA TCTCTCCAAA ACCATGCCAT TACATGGTTT TGGTGGGGAC ATACGTATCG   
  
  
- ATTTTTGTTT TTATTACTTT ATGTTCATCG ATCACTATAA CAAGAGGATA ATAATCTGTT TCTTGGTGGT   
  
  
- TTGATTGACT CGGAAGGTAA CGAGAAGGGA GGAAGGTGTG TGTAAACTGG GAAAGTCGAA ACAGAACAGA   
  
  
- GGTGTTGGGA TTGAGGCGGG AAGAAAGAGA TAAAAGGTGT AGGTAGAAAG GGAAGTGGTA CTGTACATTT   
  
  
- AAAGTCTCCA TTTTTTTAAA GAACCACCG

+     TCA

| Site Name | Organism | Position | Strand | Matrix score. | sequence | function |
| --- | --- | --- | --- | --- | --- | --- |
| TCA | Pisum sativum | 802 | + | 9 | TCATCTTCAT |  |
| TCA | Pisum sativum | 643 | - | 9 | TCATCTTCAT |  |

>023445   
+ TGTAAGTGGT GAATTACCCT GTAAATAAGG CTTTCTTCTT CAACCTACGG TGTCCCGGGC TCAAACCTCC   
  
  
+ GCTCTCCATA CTTGTAGGAT AAATGAATTT AGCGTAAAAA TAAAAAGAGA AAACAAAATT GTTAAAAAAC   
  
  
+ AACTTAATAT CTAAAATACC AAAATAAATA ACTATACTGG GGTGGAAATT TTTACCAAAT TACCGTACCA   
  
  
+ ACCGCCTTAC CAATCATACC ATACCAAATT ATTACCAAAA ATTTGTACCA ATCGGTAATG GTACGGTATT   
  
  
+ TGTACCATAC CATACTATTT TGATACGGTA CTGGTATCCA AAACCAGTAC CGGTGGTATA CCATACCTAC   
  
  
+ CAATATTATT AATATTATAT TATGTATTTA TTCATATAAA TATTTGAGGG AAAAATTAAT AAAATTAATA   
  
  
+ ATAATTATTG TAGAAGTGTA AAAAATGTAA AAAAAAGTAT ACATTCACTC ATAGTGACAA GTTTTACAAC   
  
  
+ TTTCATGAAA ATGTCAGCTT CAAAATCCAA CGCTATAATC CATAAACCTT AAATTTATAT TTAACCGTCG   
  
  
+ ATTGTCATTT ACGCTTTATT CTTCTTATTG AATTTCATTT TTAAAATTTT CTTTTTTGAA AACATGATCA   
  
  
+ TTTGAAGGAT GTAAGAAGAT GAATGGTTTA GATCTTTGAT ATCATGTTTC GATATTTACG GTTAACTGAA   
  
  
+ ATATGAGTCG ATGTCATATA GTTTGTAGAA ACTTTATAAA CATCAAGCAA TATTTTTATT AACCATAAAA   
  
  
+ CTTTGAACAT AATATCAACG ATCCAAATCA TTCATCTTCG TACATCCTCT AATAGATCAT GTTTTCAAAA   
  
  
+ AAGAAAATCT GAAAAAAAAC AAAATTTGAT GAAAAAAATG AAACGTAAAT GTAAACAACA ATCAACGATT   
  
  
+ AAATTTTGAT CTATGATTTA TATGATTATA ATGGCGAATT TCAAAGTTAA GCTCCTCACA AAAGTGGTAA   
  
  
+ AGCTTGTCAT TACAAGCGTT TATAAATTTT TTCTATATTT TTAACACCTC TATATTAATT AAAAAATATT   
  
  
+ AGATTTTGGG CAAACTAAGT GGCGTAAAAC AAAGTGACAA TTACCATTGG CATACCATAA CAACCAAACT   
  
  
+ ATTTGGTATA TTGATATTTA GTATACCGAA AGTTTGGTAC GGTAATGATA ATAGATTTTG CCGTATCAAA   
  
  
+ TATTTGGTAT GGTAATTGGT AGAGAGGTTT TGGTACGGTA ATGTACCAAA ACCACCCCTG TATGCATAGC   
  
  
+ TAAAAACAAA AATAATGAAA TACAAGTAGC TAGTGATATT GTTCTCCTAT TATTAGACAA AGAACCACCA   
  
  
+ AACTAACTGA GCCTTCCATT GCTCTTCCCT CCTTCCACAC ACATTTGACC CTTTCAGCTT TGTCTTGTCT   
  
  
+ CCACAACCCT AACTCCGCCC TTCTTTCTCT ATTTTCCACA TCCATCTTTC CCTTCACCAT GACATGTAAA   
  
  
+ TTTCAGAGGT AAAAAAATTT CTTGGTGGC  

- ACATTCACCA CTTAATGGGA CATTTATTCC GAAAGAAGAA GTTGGATGCC ACAGGGCCCG AGTTTGGAGG   
  
  
- CGAGAGGTAT GAACATCCTA TTTACTTAAA TCGCATTTTT ATTTTTCTCT TTTGTTTTAA CAATTTTTTG   
  
  
- TTGAATTATA GATTTTATGG TTTTATTTAT TGATATGACC CCACCTTTAA AAATGGTTTA ATGGCATGGT   
  
  
- TGGCGGAATG GTTAGTATGG TATGGTTTAA TAATGGTTTT TAAACATGGT TAGCCATTAC CATGCCATAA   
  
  
- ACATGGTATG GTATGATAAA ACTATGCCAT GACCATAGGT TTTGGTCATG GCCACCATAT GGTATGGATG   
  
  
- GTTATAATAA TTATAATATA ATACATAAAT AAGTATATTT ATAAACTCCC TTTTTAATTA TTTTAATTAT   
  
  
- TATTAATAAC ATCTTCACAT TTTTTACATT TTTTTTCATA TGTAAGTGAG TATCACTGTT CAAAATGTTG   
  
  
- AAAGTACTTT TACAGTCGAA GTTTTAGGTT GCGATATTAG GTATTTGGAA TTTAAATATA AATTGGCAGC   
  
  
- TAACAGTAAA TGCGAAATAA GAAGAATAAC TTAAAGTAAA AATTTTAAAA GAAAAAACTT TTGTACTAGT   
  
  
- AAACTTCCTA CATTCTTCTA CTTACCAAAT CTAGAAACTA TAGTACAAAG CTATAAATGC CAATTGACTT   
  
  
- TATACTCAGC TACAGTATAT CAAACATCTT TGAAATATTT GTAGTTCGTT ATAAAAATAA TTGGTATTTT   
  
  
- GAAACTTGTA TTATAGTTGC TAGGTTTAGT AAGTAGAAGC ATGTAGGAGA TTATCTAGTA CAAAAGTTTT   
  
  
- TTCTTTTAGA CTTTTTTTTG TTTTAAACTA CTTTTTTTAC TTTGCATTTA CATTTGTTGT TAGTTGCTAA   
  
  
- TTTAAAACTA GATACTAAAT ATACTAATAT TACCGCTTAA AGTTTCAATT CGAGGAGTGT TTTCACCATT   
  
  
- TCGAACAGTA ATGTTCGCAA ATATTTAAAA AAGATATAAA AATTGTGGAG ATATAATTAA TTTTTTATAA   
  
  
- TCTAAAACCC GTTTGATTCA CCGCATTTTG TTTCACTGTT AATGGTAACC GTATGGTATT GTTGGTTTGA   
  
  
- TAAACCATAT AACTATAAAT CATATGGCTT TCAAACCATG CCATTACTAT TATCTAAAAC GGCATAGTTT   
  
  
- ATAAACCATA CCATTAACCA TCTCTCCAAA ACCATGCCAT TACATGGTTT TGGTGGGGAC ATACGTATCG   
  
  
- ATTTTTGTTT TTATTACTTT ATGTTCATCG ATCACTATAA CAAGAGGATA ATAATCTGTT TCTTGGTGGT   
  
  
- TTGATTGACT CGGAAGGTAA CGAGAAGGGA GGAAGGTGTG TGTAAACTGG GAAAGTCGAA ACAGAACAGA   
  
  
- GGTGTTGGGA TTGAGGCGGG AAGAAAGAGA TAAAAGGTGT AGGTAGAAAG GGAAGTGGTA CTGTACATTT   
  
  
- AAAGTCTCCA TTTTTTTAAA GAACCACCG

+     TCT-motif

| Site Name | Organism | Position | Strand | Matrix score. | sequence | function |
| --- | --- | --- | --- | --- | --- | --- |
| TCT-motif | Arabidopsis thaliana | 641 | - | 6 | TCTTAC | part of a light responsive element |

>023445   
+ TGTAAGTGGT GAATTACCCT GTAAATAAGG CTTTCTTCTT CAACCTACGG TGTCCCGGGC TCAAACCTCC   
  
  
+ GCTCTCCATA CTTGTAGGAT AAATGAATTT AGCGTAAAAA TAAAAAGAGA AAACAAAATT GTTAAAAAAC   
  
  
+ AACTTAATAT CTAAAATACC AAAATAAATA ACTATACTGG GGTGGAAATT TTTACCAAAT TACCGTACCA   
  
  
+ ACCGCCTTAC CAATCATACC ATACCAAATT ATTACCAAAA ATTTGTACCA ATCGGTAATG GTACGGTATT   
  
  
+ TGTACCATAC CATACTATTT TGATACGGTA CTGGTATCCA AAACCAGTAC CGGTGGTATA CCATACCTAC   
  
  
+ CAATATTATT AATATTATAT TATGTATTTA TTCATATAAA TATTTGAGGG AAAAATTAAT AAAATTAATA   
  
  
+ ATAATTATTG TAGAAGTGTA AAAAATGTAA AAAAAAGTAT ACATTCACTC ATAGTGACAA GTTTTACAAC   
  
  
+ TTTCATGAAA ATGTCAGCTT CAAAATCCAA CGCTATAATC CATAAACCTT AAATTTATAT TTAACCGTCG   
  
  
+ ATTGTCATTT ACGCTTTATT CTTCTTATTG AATTTCATTT TTAAAATTTT CTTTTTTGAA AACATGATCA   
  
  
+ TTTGAAGGAT GTAAGAAGAT GAATGGTTTA GATCTTTGAT ATCATGTTTC GATATTTACG GTTAACTGAA   
  
  
+ ATATGAGTCG ATGTCATATA GTTTGTAGAA ACTTTATAAA CATCAAGCAA TATTTTTATT AACCATAAAA   
  
  
+ CTTTGAACAT AATATCAACG ATCCAAATCA TTCATCTTCG TACATCCTCT AATAGATCAT GTTTTCAAAA   
  
  
+ AAGAAAATCT GAAAAAAAAC AAAATTTGAT GAAAAAAATG AAACGTAAAT GTAAACAACA ATCAACGATT   
  
  
+ AAATTTTGAT CTATGATTTA TATGATTATA ATGGCGAATT TCAAAGTTAA GCTCCTCACA AAAGTGGTAA   
  
  
+ AGCTTGTCAT TACAAGCGTT TATAAATTTT TTCTATATTT TTAACACCTC TATATTAATT AAAAAATATT   
  
  
+ AGATTTTGGG CAAACTAAGT GGCGTAAAAC AAAGTGACAA TTACCATTGG CATACCATAA CAACCAAACT   
  
  
+ ATTTGGTATA TTGATATTTA GTATACCGAA AGTTTGGTAC GGTAATGATA ATAGATTTTG CCGTATCAAA   
  
  
+ TATTTGGTAT GGTAATTGGT AGAGAGGTTT TGGTACGGTA ATGTACCAAA ACCACCCCTG TATGCATAGC   
  
  
+ TAAAAACAAA AATAATGAAA TACAAGTAGC TAGTGATATT GTTCTCCTAT TATTAGACAA AGAACCACCA   
  
  
+ AACTAACTGA GCCTTCCATT GCTCTTCCCT CCTTCCACAC ACATTTGACC CTTTCAGCTT TGTCTTGTCT   
  
  
+ CCACAACCCT AACTCCGCCC TTCTTTCTCT ATTTTCCACA TCCATCTTTC CCTTCACCAT GACATGTAAA   
  
  
+ TTTCAGAGGT AAAAAAATTT CTTGGTGGC  

- ACATTCACCA CTTAATGGGA CATTTATTCC GAAAGAAGAA GTTGGATGCC ACAGGGCCCG AGTTTGGAGG   
  
  
- CGAGAGGTAT GAACATCCTA TTTACTTAAA TCGCATTTTT ATTTTTCTCT TTTGTTTTAA CAATTTTTTG   
  
  
- TTGAATTATA GATTTTATGG TTTTATTTAT TGATATGACC CCACCTTTAA AAATGGTTTA ATGGCATGGT   
  
  
- TGGCGGAATG GTTAGTATGG TATGGTTTAA TAATGGTTTT TAAACATGGT TAGCCATTAC CATGCCATAA   
  
  
- ACATGGTATG GTATGATAAA ACTATGCCAT GACCATAGGT TTTGGTCATG GCCACCATAT GGTATGGATG   
  
  
- GTTATAATAA TTATAATATA ATACATAAAT AAGTATATTT ATAAACTCCC TTTTTAATTA TTTTAATTAT   
  
  
- TATTAATAAC ATCTTCACAT TTTTTACATT TTTTTTCATA TGTAAGTGAG TATCACTGTT CAAAATGTTG   
  
  
- AAAGTACTTT TACAGTCGAA GTTTTAGGTT GCGATATTAG GTATTTGGAA TTTAAATATA AATTGGCAGC   
  
  
- TAACAGTAAA TGCGAAATAA GAAGAATAAC TTAAAGTAAA AATTTTAAAA GAAAAAACTT TTGTACTAGT   
  
  
- AAACTTCCTA CATTCTTCTA CTTACCAAAT CTAGAAACTA TAGTACAAAG CTATAAATGC CAATTGACTT   
  
  
- TATACTCAGC TACAGTATAT CAAACATCTT TGAAATATTT GTAGTTCGTT ATAAAAATAA TTGGTATTTT   
  
  
- GAAACTTGTA TTATAGTTGC TAGGTTTAGT AAGTAGAAGC ATGTAGGAGA TTATCTAGTA CAAAAGTTTT   
  
  
- TTCTTTTAGA CTTTTTTTTG TTTTAAACTA CTTTTTTTAC TTTGCATTTA CATTTGTTGT TAGTTGCTAA   
  
  
- TTTAAAACTA GATACTAAAT ATACTAATAT TACCGCTTAA AGTTTCAATT CGAGGAGTGT TTTCACCATT   
  
  
- TCGAACAGTA ATGTTCGCAA ATATTTAAAA AAGATATAAA AATTGTGGAG ATATAATTAA TTTTTTATAA   
  
  
- TCTAAAACCC GTTTGATTCA CCGCATTTTG TTTCACTGTT AATGGTAACC GTATGGTATT GTTGGTTTGA   
  
  
- TAAACCATAT AACTATAAAT CATATGGCTT TCAAACCATG CCATTACTAT TATCTAAAAC GGCATAGTTT   
  
  
- ATAAACCATA CCATTAACCA TCTCTCCAAA ACCATGCCAT TACATGGTTT TGGTGGGGAC ATACGTATCG   
  
  
- ATTTTTGTTT TTATTACTTT ATGTTCATCG ATCACTATAA CAAGAGGATA ATAATCTGTT TCTTGGTGGT   
  
  
- TTGATTGACT CGGAAGGTAA CGAGAAGGGA GGAAGGTGTG TGTAAACTGG GAAAGTCGAA ACAGAACAGA   
  
  
- GGTGTTGGGA TTGAGGCGGG AAGAAAGAGA TAAAAGGTGT AGGTAGAAAG GGAAGTGGTA CTGTACATTT   
  
  
- AAAGTCTCCA TTTTTTTAAA GAACCACCG

+     Unnamed\_\_4

| Site Name | Organism | Position | Strand | Matrix score. | sequence | function |
| --- | --- | --- | --- | --- | --- | --- |
| Unnamed\_\_4 | Petroselinum hortense | 67 | + | 4 | CTCC |  |
| Unnamed\_\_4 | Petroselinum hortense | 1359 | + | 4 | CTCC |  |
| Unnamed\_\_4 | Petroselinum hortense | 962 | + | 4 | CTCC |  |
| Unnamed\_\_4 | Petroselinum hortense | 1413 | + | 4 | CTCC |  |
| Unnamed\_\_4 | Petroselinum hortense | 74 | + | 4 | CTCC |  |
| Unnamed\_\_4 | Petroselinum hortense | 1399 | + | 4 | CTCC |  |
| Unnamed\_\_4 | Petroselinum hortense | 1304 | + | 4 | CTCC |  |

>023445   
+ TGTAAGTGGT GAATTACCCT GTAAATAAGG CTTTCTTCTT CAACCTACGG TGTCCCGGGC TCAAACCTCC   
  
  
+ GCTCTCCATA CTTGTAGGAT AAATGAATTT AGCGTAAAAA TAAAAAGAGA AAACAAAATT GTTAAAAAAC   
  
  
+ AACTTAATAT CTAAAATACC AAAATAAATA ACTATACTGG GGTGGAAATT TTTACCAAAT TACCGTACCA   
  
  
+ ACCGCCTTAC CAATCATACC ATACCAAATT ATTACCAAAA ATTTGTACCA ATCGGTAATG GTACGGTATT   
  
  
+ TGTACCATAC CATACTATTT TGATACGGTA CTGGTATCCA AAACCAGTAC CGGTGGTATA CCATACCTAC   
  
  
+ CAATATTATT AATATTATAT TATGTATTTA TTCATATAAA TATTTGAGGG AAAAATTAAT AAAATTAATA   
  
  
+ ATAATTATTG TAGAAGTGTA AAAAATGTAA AAAAAAGTAT ACATTCACTC ATAGTGACAA GTTTTACAAC   
  
  
+ TTTCATGAAA ATGTCAGCTT CAAAATCCAA CGCTATAATC CATAAACCTT AAATTTATAT TTAACCGTCG   
  
  
+ ATTGTCATTT ACGCTTTATT CTTCTTATTG AATTTCATTT TTAAAATTTT CTTTTTTGAA AACATGATCA   
  
  
+ TTTGAAGGAT GTAAGAAGAT GAATGGTTTA GATCTTTGAT ATCATGTTTC GATATTTACG GTTAACTGAA   
  
  
+ ATATGAGTCG ATGTCATATA GTTTGTAGAA ACTTTATAAA CATCAAGCAA TATTTTTATT AACCATAAAA   
  
  
+ CTTTGAACAT AATATCAACG ATCCAAATCA TTCATCTTCG TACATCCTCT AATAGATCAT GTTTTCAAAA   
  
  
+ AAGAAAATCT GAAAAAAAAC AAAATTTGAT GAAAAAAATG AAACGTAAAT GTAAACAACA ATCAACGATT   
  
  
+ AAATTTTGAT CTATGATTTA TATGATTATA ATGGCGAATT TCAAAGTTAA GCTCCTCACA AAAGTGGTAA   
  
  
+ AGCTTGTCAT TACAAGCGTT TATAAATTTT TTCTATATTT TTAACACCTC TATATTAATT AAAAAATATT   
  
  
+ AGATTTTGGG CAAACTAAGT GGCGTAAAAC AAAGTGACAA TTACCATTGG CATACCATAA CAACCAAACT   
  
  
+ ATTTGGTATA TTGATATTTA GTATACCGAA AGTTTGGTAC GGTAATGATA ATAGATTTTG CCGTATCAAA   
  
  
+ TATTTGGTAT GGTAATTGGT AGAGAGGTTT TGGTACGGTA ATGTACCAAA ACCACCCCTG TATGCATAGC   
  
  
+ TAAAAACAAA AATAATGAAA TACAAGTAGC TAGTGATATT GTTCTCCTAT TATTAGACAA AGAACCACCA   
  
  
+ AACTAACTGA GCCTTCCATT GCTCTTCCCT CCTTCCACAC ACATTTGACC CTTTCAGCTT TGTCTTGTCT   
  
  
+ CCACAACCCT AACTCCGCCC TTCTTTCTCT ATTTTCCACA TCCATCTTTC CCTTCACCAT GACATGTAAA   
  
  
+ TTTCAGAGGT AAAAAAATTT CTTGGTGGC  

- ACATTCACCA CTTAATGGGA CATTTATTCC GAAAGAAGAA GTTGGATGCC ACAGGGCCCG AGTTTGGAGG   
  
  
- CGAGAGGTAT GAACATCCTA TTTACTTAAA TCGCATTTTT ATTTTTCTCT TTTGTTTTAA CAATTTTTTG   
  
  
- TTGAATTATA GATTTTATGG TTTTATTTAT TGATATGACC CCACCTTTAA AAATGGTTTA ATGGCATGGT   
  
  
- TGGCGGAATG GTTAGTATGG TATGGTTTAA TAATGGTTTT TAAACATGGT TAGCCATTAC CATGCCATAA   
  
  
- ACATGGTATG GTATGATAAA ACTATGCCAT GACCATAGGT TTTGGTCATG GCCACCATAT GGTATGGATG   
  
  
- GTTATAATAA TTATAATATA ATACATAAAT AAGTATATTT ATAAACTCCC TTTTTAATTA TTTTAATTAT   
  
  
- TATTAATAAC ATCTTCACAT TTTTTACATT TTTTTTCATA TGTAAGTGAG TATCACTGTT CAAAATGTTG   
  
  
- AAAGTACTTT TACAGTCGAA GTTTTAGGTT GCGATATTAG GTATTTGGAA TTTAAATATA AATTGGCAGC   
  
  
- TAACAGTAAA TGCGAAATAA GAAGAATAAC TTAAAGTAAA AATTTTAAAA GAAAAAACTT TTGTACTAGT   
  
  
- AAACTTCCTA CATTCTTCTA CTTACCAAAT CTAGAAACTA TAGTACAAAG CTATAAATGC CAATTGACTT   
  
  
- TATACTCAGC TACAGTATAT CAAACATCTT TGAAATATTT GTAGTTCGTT ATAAAAATAA TTGGTATTTT   
  
  
- GAAACTTGTA TTATAGTTGC TAGGTTTAGT AAGTAGAAGC ATGTAGGAGA TTATCTAGTA CAAAAGTTTT   
  
  
- TTCTTTTAGA CTTTTTTTTG TTTTAAACTA CTTTTTTTAC TTTGCATTTA CATTTGTTGT TAGTTGCTAA   
  
  
- TTTAAAACTA GATACTAAAT ATACTAATAT TACCGCTTAA AGTTTCAATT CGAGGAGTGT TTTCACCATT   
  
  
- TCGAACAGTA ATGTTCGCAA ATATTTAAAA AAGATATAAA AATTGTGGAG ATATAATTAA TTTTTTATAA   
  
  
- TCTAAAACCC GTTTGATTCA CCGCATTTTG TTTCACTGTT AATGGTAACC GTATGGTATT GTTGGTTTGA   
  
  
- TAAACCATAT AACTATAAAT CATATGGCTT TCAAACCATG CCATTACTAT TATCTAAAAC GGCATAGTTT   
  
  
- ATAAACCATA CCATTAACCA TCTCTCCAAA ACCATGCCAT TACATGGTTT TGGTGGGGAC ATACGTATCG   
  
  
- ATTTTTGTTT TTATTACTTT ATGTTCATCG ATCACTATAA CAAGAGGATA ATAATCTGTT TCTTGGTGGT   
  
  
- TTGATTGACT CGGAAGGTAA CGAGAAGGGA GGAAGGTGTG TGTAAACTGG GAAAGTCGAA ACAGAACAGA   
  
  
- GGTGTTGGGA TTGAGGCGGG AAGAAAGAGA TAAAAGGTGT AGGTAGAAAG GGAAGTGGTA CTGTACATTT   
  
  
- AAAGTCTCCA TTTTTTTAAA GAACCACCG

+     Unnamed\_\_6

| Site Name | Organism | Position | Strand | Matrix score. | sequence | function |
| --- | --- | --- | --- | --- | --- | --- |
| Unnamed\_\_6 | Zea mays | 385 | + | 10 | taTAAATATct |  |

>023445   
+ TGTAAGTGGT GAATTACCCT GTAAATAAGG CTTTCTTCTT CAACCTACGG TGTCCCGGGC TCAAACCTCC   
  
  
+ GCTCTCCATA CTTGTAGGAT AAATGAATTT AGCGTAAAAA TAAAAAGAGA AAACAAAATT GTTAAAAAAC   
  
  
+ AACTTAATAT CTAAAATACC AAAATAAATA ACTATACTGG GGTGGAAATT TTTACCAAAT TACCGTACCA   
  
  
+ ACCGCCTTAC CAATCATACC ATACCAAATT ATTACCAAAA ATTTGTACCA ATCGGTAATG GTACGGTATT   
  
  
+ TGTACCATAC CATACTATTT TGATACGGTA CTGGTATCCA AAACCAGTAC CGGTGGTATA CCATACCTAC   
  
  
+ CAATATTATT AATATTATAT TATGTATTTA TTCATATAAA TATTTGAGGG AAAAATTAAT AAAATTAATA   
  
  
+ ATAATTATTG TAGAAGTGTA AAAAATGTAA AAAAAAGTAT ACATTCACTC ATAGTGACAA GTTTTACAAC   
  
  
+ TTTCATGAAA ATGTCAGCTT CAAAATCCAA CGCTATAATC CATAAACCTT AAATTTATAT TTAACCGTCG   
  
  
+ ATTGTCATTT ACGCTTTATT CTTCTTATTG AATTTCATTT TTAAAATTTT CTTTTTTGAA AACATGATCA   
  
  
+ TTTGAAGGAT GTAAGAAGAT GAATGGTTTA GATCTTTGAT ATCATGTTTC GATATTTACG GTTAACTGAA   
  
  
+ ATATGAGTCG ATGTCATATA GTTTGTAGAA ACTTTATAAA CATCAAGCAA TATTTTTATT AACCATAAAA   
  
  
+ CTTTGAACAT AATATCAACG ATCCAAATCA TTCATCTTCG TACATCCTCT AATAGATCAT GTTTTCAAAA   
  
  
+ AAGAAAATCT GAAAAAAAAC AAAATTTGAT GAAAAAAATG AAACGTAAAT GTAAACAACA ATCAACGATT   
  
  
+ AAATTTTGAT CTATGATTTA TATGATTATA ATGGCGAATT TCAAAGTTAA GCTCCTCACA AAAGTGGTAA   
  
  
+ AGCTTGTCAT TACAAGCGTT TATAAATTTT TTCTATATTT TTAACACCTC TATATTAATT AAAAAATATT   
  
  
+ AGATTTTGGG CAAACTAAGT GGCGTAAAAC AAAGTGACAA TTACCATTGG CATACCATAA CAACCAAACT   
  
  
+ ATTTGGTATA TTGATATTTA GTATACCGAA AGTTTGGTAC GGTAATGATA ATAGATTTTG CCGTATCAAA   
  
  
+ TATTTGGTAT GGTAATTGGT AGAGAGGTTT TGGTACGGTA ATGTACCAAA ACCACCCCTG TATGCATAGC   
  
  
+ TAAAAACAAA AATAATGAAA TACAAGTAGC TAGTGATATT GTTCTCCTAT TATTAGACAA AGAACCACCA   
  
  
+ AACTAACTGA GCCTTCCATT GCTCTTCCCT CCTTCCACAC ACATTTGACC CTTTCAGCTT TGTCTTGTCT   
  
  
+ CCACAACCCT AACTCCGCCC TTCTTTCTCT ATTTTCCACA TCCATCTTTC CCTTCACCAT GACATGTAAA   
  
  
+ TTTCAGAGGT AAAAAAATTT CTTGGTGGC  

- ACATTCACCA CTTAATGGGA CATTTATTCC GAAAGAAGAA GTTGGATGCC ACAGGGCCCG AGTTTGGAGG   
  
  
- CGAGAGGTAT GAACATCCTA TTTACTTAAA TCGCATTTTT ATTTTTCTCT TTTGTTTTAA CAATTTTTTG   
  
  
- TTGAATTATA GATTTTATGG TTTTATTTAT TGATATGACC CCACCTTTAA AAATGGTTTA ATGGCATGGT   
  
  
- TGGCGGAATG GTTAGTATGG TATGGTTTAA TAATGGTTTT TAAACATGGT TAGCCATTAC CATGCCATAA   
  
  
- ACATGGTATG GTATGATAAA ACTATGCCAT GACCATAGGT TTTGGTCATG GCCACCATAT GGTATGGATG   
  
  
- GTTATAATAA TTATAATATA ATACATAAAT AAGTATATTT ATAAACTCCC TTTTTAATTA TTTTAATTAT   
  
  
- TATTAATAAC ATCTTCACAT TTTTTACATT TTTTTTCATA TGTAAGTGAG TATCACTGTT CAAAATGTTG   
  
  
- AAAGTACTTT TACAGTCGAA GTTTTAGGTT GCGATATTAG GTATTTGGAA TTTAAATATA AATTGGCAGC   
  
  
- TAACAGTAAA TGCGAAATAA GAAGAATAAC TTAAAGTAAA AATTTTAAAA GAAAAAACTT TTGTACTAGT   
  
  
- AAACTTCCTA CATTCTTCTA CTTACCAAAT CTAGAAACTA TAGTACAAAG CTATAAATGC CAATTGACTT   
  
  
- TATACTCAGC TACAGTATAT CAAACATCTT TGAAATATTT GTAGTTCGTT ATAAAAATAA TTGGTATTTT   
  
  
- GAAACTTGTA TTATAGTTGC TAGGTTTAGT AAGTAGAAGC ATGTAGGAGA TTATCTAGTA CAAAAGTTTT   
  
  
- TTCTTTTAGA CTTTTTTTTG TTTTAAACTA CTTTTTTTAC TTTGCATTTA CATTTGTTGT TAGTTGCTAA   
  
  
- TTTAAAACTA GATACTAAAT ATACTAATAT TACCGCTTAA AGTTTCAATT CGAGGAGTGT TTTCACCATT   
  
  
- TCGAACAGTA ATGTTCGCAA ATATTTAAAA AAGATATAAA AATTGTGGAG ATATAATTAA TTTTTTATAA   
  
  
- TCTAAAACCC GTTTGATTCA CCGCATTTTG TTTCACTGTT AATGGTAACC GTATGGTATT GTTGGTTTGA   
  
  
- TAAACCATAT AACTATAAAT CATATGGCTT TCAAACCATG CCATTACTAT TATCTAAAAC GGCATAGTTT   
  
  
- ATAAACCATA CCATTAACCA TCTCTCCAAA ACCATGCCAT TACATGGTTT TGGTGGGGAC ATACGTATCG   
  
  
- ATTTTTGTTT TTATTACTTT ATGTTCATCG ATCACTATAA CAAGAGGATA ATAATCTGTT TCTTGGTGGT   
  
  
- TTGATTGACT CGGAAGGTAA CGAGAAGGGA GGAAGGTGTG TGTAAACTGG GAAAGTCGAA ACAGAACAGA   
  
  
- GGTGTTGGGA TTGAGGCGGG AAGAAAGAGA TAAAAGGTGT AGGTAGAAAG GGAAGTGGTA CTGTACATTT   
  
  
- AAAGTCTCCA TTTTTTTAAA GAACCACCG

+     W box

| Site Name | Organism | Position | Strand | Matrix score. | sequence | function |
| --- | --- | --- | --- | --- | --- | --- |
| W box | Arabidopsis thaliana | 1375 | + | 6 | TTGACC |  |

>023445   
+ TGTAAGTGGT GAATTACCCT GTAAATAAGG CTTTCTTCTT CAACCTACGG TGTCCCGGGC TCAAACCTCC   
  
  
+ GCTCTCCATA CTTGTAGGAT AAATGAATTT AGCGTAAAAA TAAAAAGAGA AAACAAAATT GTTAAAAAAC   
  
  
+ AACTTAATAT CTAAAATACC AAAATAAATA ACTATACTGG GGTGGAAATT TTTACCAAAT TACCGTACCA   
  
  
+ ACCGCCTTAC CAATCATACC ATACCAAATT ATTACCAAAA ATTTGTACCA ATCGGTAATG GTACGGTATT   
  
  
+ TGTACCATAC CATACTATTT TGATACGGTA CTGGTATCCA AAACCAGTAC CGGTGGTATA CCATACCTAC   
  
  
+ CAATATTATT AATATTATAT TATGTATTTA TTCATATAAA TATTTGAGGG AAAAATTAAT AAAATTAATA   
  
  
+ ATAATTATTG TAGAAGTGTA AAAAATGTAA AAAAAAGTAT ACATTCACTC ATAGTGACAA GTTTTACAAC   
  
  
+ TTTCATGAAA ATGTCAGCTT CAAAATCCAA CGCTATAATC CATAAACCTT AAATTTATAT TTAACCGTCG   
  
  
+ ATTGTCATTT ACGCTTTATT CTTCTTATTG AATTTCATTT TTAAAATTTT CTTTTTTGAA AACATGATCA   
  
  
+ TTTGAAGGAT GTAAGAAGAT GAATGGTTTA GATCTTTGAT ATCATGTTTC GATATTTACG GTTAACTGAA   
  
  
+ ATATGAGTCG ATGTCATATA GTTTGTAGAA ACTTTATAAA CATCAAGCAA TATTTTTATT AACCATAAAA   
  
  
+ CTTTGAACAT AATATCAACG ATCCAAATCA TTCATCTTCG TACATCCTCT AATAGATCAT GTTTTCAAAA   
  
  
+ AAGAAAATCT GAAAAAAAAC AAAATTTGAT GAAAAAAATG AAACGTAAAT GTAAACAACA ATCAACGATT   
  
  
+ AAATTTTGAT CTATGATTTA TATGATTATA ATGGCGAATT TCAAAGTTAA GCTCCTCACA AAAGTGGTAA   
  
  
+ AGCTTGTCAT TACAAGCGTT TATAAATTTT TTCTATATTT TTAACACCTC TATATTAATT AAAAAATATT   
  
  
+ AGATTTTGGG CAAACTAAGT GGCGTAAAAC AAAGTGACAA TTACCATTGG CATACCATAA CAACCAAACT   
  
  
+ ATTTGGTATA TTGATATTTA GTATACCGAA AGTTTGGTAC GGTAATGATA ATAGATTTTG CCGTATCAAA   
  
  
+ TATTTGGTAT GGTAATTGGT AGAGAGGTTT TGGTACGGTA ATGTACCAAA ACCACCCCTG TATGCATAGC   
  
  
+ TAAAAACAAA AATAATGAAA TACAAGTAGC TAGTGATATT GTTCTCCTAT TATTAGACAA AGAACCACCA   
  
  
+ AACTAACTGA GCCTTCCATT GCTCTTCCCT CCTTCCACAC ACATTTGACC CTTTCAGCTT TGTCTTGTCT   
  
  
+ CCACAACCCT AACTCCGCCC TTCTTTCTCT ATTTTCCACA TCCATCTTTC CCTTCACCAT GACATGTAAA   
  
  
+ TTTCAGAGGT AAAAAAATTT CTTGGTGGC  

- ACATTCACCA CTTAATGGGA CATTTATTCC GAAAGAAGAA GTTGGATGCC ACAGGGCCCG AGTTTGGAGG   
  
  
- CGAGAGGTAT GAACATCCTA TTTACTTAAA TCGCATTTTT ATTTTTCTCT TTTGTTTTAA CAATTTTTTG   
  
  
- TTGAATTATA GATTTTATGG TTTTATTTAT TGATATGACC CCACCTTTAA AAATGGTTTA ATGGCATGGT   
  
  
- TGGCGGAATG GTTAGTATGG TATGGTTTAA TAATGGTTTT TAAACATGGT TAGCCATTAC CATGCCATAA   
  
  
- ACATGGTATG GTATGATAAA ACTATGCCAT GACCATAGGT TTTGGTCATG GCCACCATAT GGTATGGATG   
  
  
- GTTATAATAA TTATAATATA ATACATAAAT AAGTATATTT ATAAACTCCC TTTTTAATTA TTTTAATTAT   
  
  
- TATTAATAAC ATCTTCACAT TTTTTACATT TTTTTTCATA TGTAAGTGAG TATCACTGTT CAAAATGTTG   
  
  
- AAAGTACTTT TACAGTCGAA GTTTTAGGTT GCGATATTAG GTATTTGGAA TTTAAATATA AATTGGCAGC   
  
  
- TAACAGTAAA TGCGAAATAA GAAGAATAAC TTAAAGTAAA AATTTTAAAA GAAAAAACTT TTGTACTAGT   
  
  
- AAACTTCCTA CATTCTTCTA CTTACCAAAT CTAGAAACTA TAGTACAAAG CTATAAATGC CAATTGACTT   
  
  
- TATACTCAGC TACAGTATAT CAAACATCTT TGAAATATTT GTAGTTCGTT ATAAAAATAA TTGGTATTTT   
  
  
- GAAACTTGTA TTATAGTTGC TAGGTTTAGT AAGTAGAAGC ATGTAGGAGA TTATCTAGTA CAAAAGTTTT   
  
  
- TTCTTTTAGA CTTTTTTTTG TTTTAAACTA CTTTTTTTAC TTTGCATTTA CATTTGTTGT TAGTTGCTAA   
  
  
- TTTAAAACTA GATACTAAAT ATACTAATAT TACCGCTTAA AGTTTCAATT CGAGGAGTGT TTTCACCATT   
  
  
- TCGAACAGTA ATGTTCGCAA ATATTTAAAA AAGATATAAA AATTGTGGAG ATATAATTAA TTTTTTATAA   
  
  
- TCTAAAACCC GTTTGATTCA CCGCATTTTG TTTCACTGTT AATGGTAACC GTATGGTATT GTTGGTTTGA   
  
  
- TAAACCATAT AACTATAAAT CATATGGCTT TCAAACCATG CCATTACTAT TATCTAAAAC GGCATAGTTT   
  
  
- ATAAACCATA CCATTAACCA TCTCTCCAAA ACCATGCCAT TACATGGTTT TGGTGGGGAC ATACGTATCG   
  
  
- ATTTTTGTTT TTATTACTTT ATGTTCATCG ATCACTATAA CAAGAGGATA ATAATCTGTT TCTTGGTGGT   
  
  
- TTGATTGACT CGGAAGGTAA CGAGAAGGGA GGAAGGTGTG TGTAAACTGG GAAAGTCGAA ACAGAACAGA   
  
  
- GGTGTTGGGA TTGAGGCGGG AAGAAAGAGA TAAAAGGTGT AGGTAGAAAG GGAAGTGGTA CTGTACATTT   
  
  
- AAAGTCTCCA TTTTTTTAAA GAACCACCG

+     WUN-motif

| Site Name | Organism | Position | Strand | Matrix score. | sequence | function |
| --- | --- | --- | --- | --- | --- | --- |
| WUN-motif | Nicotiana glutinosa | 1485 | + | 9 | AAATTTCTT |  |

>023445   
+ TGTAAGTGGT GAATTACCCT GTAAATAAGG CTTTCTTCTT CAACCTACGG TGTCCCGGGC TCAAACCTCC   
  
  
+ GCTCTCCATA CTTGTAGGAT AAATGAATTT AGCGTAAAAA TAAAAAGAGA AAACAAAATT GTTAAAAAAC   
  
  
+ AACTTAATAT CTAAAATACC AAAATAAATA ACTATACTGG GGTGGAAATT TTTACCAAAT TACCGTACCA   
  
  
+ ACCGCCTTAC CAATCATACC ATACCAAATT ATTACCAAAA ATTTGTACCA ATCGGTAATG GTACGGTATT   
  
  
+ TGTACCATAC CATACTATTT TGATACGGTA CTGGTATCCA AAACCAGTAC CGGTGGTATA CCATACCTAC   
  
  
+ CAATATTATT AATATTATAT TATGTATTTA TTCATATAAA TATTTGAGGG AAAAATTAAT AAAATTAATA   
  
  
+ ATAATTATTG TAGAAGTGTA AAAAATGTAA AAAAAAGTAT ACATTCACTC ATAGTGACAA GTTTTACAAC   
  
  
+ TTTCATGAAA ATGTCAGCTT CAAAATCCAA CGCTATAATC CATAAACCTT AAATTTATAT TTAACCGTCG   
  
  
+ ATTGTCATTT ACGCTTTATT CTTCTTATTG AATTTCATTT TTAAAATTTT CTTTTTTGAA AACATGATCA   
  
  
+ TTTGAAGGAT GTAAGAAGAT GAATGGTTTA GATCTTTGAT ATCATGTTTC GATATTTACG GTTAACTGAA   
  
  
+ ATATGAGTCG ATGTCATATA GTTTGTAGAA ACTTTATAAA CATCAAGCAA TATTTTTATT AACCATAAAA   
  
  
+ CTTTGAACAT AATATCAACG ATCCAAATCA TTCATCTTCG TACATCCTCT AATAGATCAT GTTTTCAAAA   
  
  
+ AAGAAAATCT GAAAAAAAAC AAAATTTGAT GAAAAAAATG AAACGTAAAT GTAAACAACA ATCAACGATT   
  
  
+ AAATTTTGAT CTATGATTTA TATGATTATA ATGGCGAATT TCAAAGTTAA GCTCCTCACA AAAGTGGTAA   
  
  
+ AGCTTGTCAT TACAAGCGTT TATAAATTTT TTCTATATTT TTAACACCTC TATATTAATT AAAAAATATT   
  
  
+ AGATTTTGGG CAAACTAAGT GGCGTAAAAC AAAGTGACAA TTACCATTGG CATACCATAA CAACCAAACT   
  
  
+ ATTTGGTATA TTGATATTTA GTATACCGAA AGTTTGGTAC GGTAATGATA ATAGATTTTG CCGTATCAAA   
  
  
+ TATTTGGTAT GGTAATTGGT AGAGAGGTTT TGGTACGGTA ATGTACCAAA ACCACCCCTG TATGCATAGC   
  
  
+ TAAAAACAAA AATAATGAAA TACAAGTAGC TAGTGATATT GTTCTCCTAT TATTAGACAA AGAACCACCA   
  
  
+ AACTAACTGA GCCTTCCATT GCTCTTCCCT CCTTCCACAC ACATTTGACC CTTTCAGCTT TGTCTTGTCT   
  
  
+ CCACAACCCT AACTCCGCCC TTCTTTCTCT ATTTTCCACA TCCATCTTTC CCTTCACCAT GACATGTAAA   
  
  
+ TTTCAGAGGT AAAAAAATTT CTTGGTGGC  

- ACATTCACCA CTTAATGGGA CATTTATTCC GAAAGAAGAA GTTGGATGCC ACAGGGCCCG AGTTTGGAGG   
  
  
- CGAGAGGTAT GAACATCCTA TTTACTTAAA TCGCATTTTT ATTTTTCTCT TTTGTTTTAA CAATTTTTTG   
  
  
- TTGAATTATA GATTTTATGG TTTTATTTAT TGATATGACC CCACCTTTAA AAATGGTTTA ATGGCATGGT   
  
  
- TGGCGGAATG GTTAGTATGG TATGGTTTAA TAATGGTTTT TAAACATGGT TAGCCATTAC CATGCCATAA   
  
  
- ACATGGTATG GTATGATAAA ACTATGCCAT GACCATAGGT TTTGGTCATG GCCACCATAT GGTATGGATG   
  
  
- GTTATAATAA TTATAATATA ATACATAAAT AAGTATATTT ATAAACTCCC TTTTTAATTA TTTTAATTAT   
  
  
- TATTAATAAC ATCTTCACAT TTTTTACATT TTTTTTCATA TGTAAGTGAG TATCACTGTT CAAAATGTTG   
  
  
- AAAGTACTTT TACAGTCGAA GTTTTAGGTT GCGATATTAG GTATTTGGAA TTTAAATATA AATTGGCAGC   
  
  
- TAACAGTAAA TGCGAAATAA GAAGAATAAC TTAAAGTAAA AATTTTAAAA GAAAAAACTT TTGTACTAGT   
  
  
- AAACTTCCTA CATTCTTCTA CTTACCAAAT CTAGAAACTA TAGTACAAAG CTATAAATGC CAATTGACTT   
  
  
- TATACTCAGC TACAGTATAT CAAACATCTT TGAAATATTT GTAGTTCGTT ATAAAAATAA TTGGTATTTT   
  
  
- GAAACTTGTA TTATAGTTGC TAGGTTTAGT AAGTAGAAGC ATGTAGGAGA TTATCTAGTA CAAAAGTTTT   
  
  
- TTCTTTTAGA CTTTTTTTTG TTTTAAACTA CTTTTTTTAC TTTGCATTTA CATTTGTTGT TAGTTGCTAA   
  
  
- TTTAAAACTA GATACTAAAT ATACTAATAT TACCGCTTAA AGTTTCAATT CGAGGAGTGT TTTCACCATT   
  
  
- TCGAACAGTA ATGTTCGCAA ATATTTAAAA AAGATATAAA AATTGTGGAG ATATAATTAA TTTTTTATAA   
  
  
- TCTAAAACCC GTTTGATTCA CCGCATTTTG TTTCACTGTT AATGGTAACC GTATGGTATT GTTGGTTTGA   
  
  
- TAAACCATAT AACTATAAAT CATATGGCTT TCAAACCATG CCATTACTAT TATCTAAAAC GGCATAGTTT   
  
  
- ATAAACCATA CCATTAACCA TCTCTCCAAA ACCATGCCAT TACATGGTTT TGGTGGGGAC ATACGTATCG   
  
  
- ATTTTTGTTT TTATTACTTT ATGTTCATCG ATCACTATAA CAAGAGGATA ATAATCTGTT TCTTGGTGGT   
  
  
- TTGATTGACT CGGAAGGTAA CGAGAAGGGA GGAAGGTGTG TGTAAACTGG GAAAGTCGAA ACAGAACAGA   
  
  
- GGTGTTGGGA TTGAGGCGGG AAGAAAGAGA TAAAAGGTGT AGGTAGAAAG GGAAGTGGTA CTGTACATTT   
  
  
- AAAGTCTCCA TTTTTTTAAA GAACCACCG
