# Supplementary material for: In Silico Genome-Wide Analysis of Respiratory Burst Oxidase Homolog (RBOH) Family Genes in Five Fruit-Producing Trees, and Potential Functional Analysis on Lignification of Stone Cells in Chinese White Pear
Source: Cells. 2019 May 29;8(6):520. doi: 10.3390/cells8060520 (PMC6627160; doi:10.3390/cells8060520)
Supplement: Supplementary file 1 [file cells-08-00520-s001.zip › Supplementary Material/Spreadsheets S2. Numbers of cis-elements in promoter region of PbRBOHs.tar.zip!/PbRBOHH.html]

Content-Type: text/html; charset=ISO-8859-1


PlantCARE


Webmaster Firefox specific output  
To save the result:
click on the frame with the right mouse button and save the source code as a text file with extension .html  
REFERENCE:PlantCARE: a database of plant cis-acting regulatory elements and a portal to tools for in silico analysis of promoter sequences.  
Lescot, M., Déhais, P., Moreau, Y., De Moor, B., Rouzé ,P.,and Rombauts, S.  
Nucleic Acids Res., Database issue(2002), 30(1):325-327.   


---

>033955   
+ GAAACTTTTT TACCTCTGAA ATTTACATGT CTTTGTGAAG GGAAAGATAG ATGTGGAAAA TAGAGAAGCA   
  
  
+ AGGGCGCAGT TAAGGTTGTG GAGAGAAGAC AAAGCTGAAA GGGTCAAATG TGTGGAAAGG AGGGAAGAGT   
  
  
+ AATGGAAGGC TCAATTAGTT TGGTGGTTCT TTGTCTAATA ATAGGAGAAC CATATCACTA TTTTGTTTTA   
  
  
+ TTTTGATATC TTATGTATAA TGATGTTTGT AACAAAATAT TTTCTCTTTT GATTTTTATT TTTACAACGG   
  
  
+ ATAGGATAAT TGTCTAAACG ATATTAGGGA GCAAGATTTG AACTCAAGAT GCCGTAAGTT GAAGGAAAGA   
  
  
+ CCTATCCACA AAGCAATACA CCGCTTGCTA ATAAAAACAC GCTTGTTAAA CATATAATAA TTATGTTGCC   
  
  
+ ACTCATCTTA CGATTTTAAC GGTAATATTT TTGTTCACTT TTAAGGGCAA AGACAAAAAC TTTCAATGGG   
  
  
+ ATAGCTAAAC ATGTTATACA TTTAGCATAA GCAACCCTAA TTAAGCACTA GCAACCTTAT ACATGTTATG   
  
  
+ CATTTAAGGC AGGTCTTATT GGCAATGGTG AGCTAGAGGG GGGCACGGGC ACGGTATTGT GCATAATGAA   
  
  
+ GAAATTTTTT TTGTTTTTTT AAGATTAAAA AAAAATTCAT TTATAAATCA AGAGGTTGGG ACTAGTATTT   
  
  
+ AAGACAACCA CAGTTTGACC CTCGATATAA ATTTATGTAA ATATATTTAT TTAATACAAT TATCATTTAA   
  
  
+ ACGTCGAGAT AGCGATTGTA TCAAATACAT TTAAGTATTG TAATCAAATG ACAAAAAGTT CACCGTGAAC   
  
  
+ AATAGGAACC AAATTAAACA TAAATACTAA GACGTCTTAT ACAATGGTAG TACATGGCAA TTCCAAATAA   
  
  
+ ATTGTCTTCC ACCCCCAGAA AAAAAATTAT GCTTTTAAAA AAAGATTTTG AGAAATTTCA TTCTTATACA   
  
  
+ GTTAAATTTA TCTAAAGGAA TAACTTCTTT AAAGTCATAA AACATTCCGG TCACAACTGG GACCATTTAC   
  
  
+ GTATGGTAAA AAACTCCGCA TTTTAACGAG TCATAACTTT TCTTGATCTC ATCTTAAGGA AACACCGACT   
  
  
+ TTTTTTCATG CCTGCGTCAT CATACATGTT GCCCGAGGAC ACAGCCCATT GACGACTCTA GGACAATTAG   
  
  
+ GTACATGAGA AGGCCTTCGA GGAGTTAGCG GGAGCAATAC TGGTGGATTC AACAAGTATT CTTTCATCTT   
  
  
+ GTCAAAGGCA TGCTGACATT CATCTGTCCA TTCGGTGGAA ACATTCTTCT TCAGCAGCTT GAATAATGGT   
  
  
+ TCACATGTGG CAGTTAGTTG AGATAGTTCA CGTAGTAATA ATTGTCAATG ATTACAAAAA TATGCAGTAT   
  
  
+ CACAAGTAAA AATTTAAGAT GAACTAAAAT ATCTCTTGTT TGAGAAATGT TTCAGCTTTT AGAGTTGATG   
  
  
+ TCACGTCTTT GAGCTTGCAT GTATGTAAT  

- CTTTGAAAAA ATGGAGACTT TAAATGTACA GAAACACTTC CCTTTCTATC TACACCTTTT ATCTCTTCGT   
  
  
- TCCCGCGTCA ATTCCAACAC CTCTCTTCTG TTTCGACTTT CCCAGTTTAC ACACCTTTCC TCCCTTCTCA   
  
  
- TTACCTTCCG AGTTAATCAA ACCACCAAGA AACAGATTAT TATCCTCTTG GTATAGTGAT AAAACAAAAT   
  
  
- AAAACTATAG AATACATATT ACTACAAACA TTGTTTTATA AAAGAGAAAA CTAAAAATAA AAATGTTGCC   
  
  
- TATCCTATTA ACAGATTTGC TATAATCCCT CGTTCTAAAC TTGAGTTCTA CGGCATTCAA CTTCCTTTCT   
  
  
- GGATAGGTGT TTCGTTATGT GGCGAACGAT TATTTTTGTG CGAACAATTT GTATATTATT AATACAACGG   
  
  
- TGAGTAGAAT GCTAAAATTG CCATTATAAA AACAAGTGAA AATTCCCGTT TCTGTTTTTG AAAGTTACCC   
  
  
- TATCGATTTG TACAATATGT AAATCGTATT CGTTGGGATT AATTCGTGAT CGTTGGAATA TGTACAATAC   
  
  
- GTAAATTCCG TCCAGAATAA CCGTTACCAC TCGATCTCCC CCCGTGCCCG TGCCATAACA CGTATTACTT   
  
  
- CTTTAAAAAA AACAAAAAAA TTCTAATTTT TTTTTAAGTA AATATTTAGT TCTCCAACCC TGATCATAAA   
  
  
- TTCTGTTGGT GTCAAACTGG GAGCTATATT TAAATACATT TATATAAATA AATTATGTTA ATAGTAAATT   
  
  
- TGCAGCTCTA TCGCTAACAT AGTTTATGTA AATTCATAAC ATTAGTTTAC TGTTTTTCAA GTGGCACTTG   
  
  
- TTATCCTTGG TTTAATTTGT ATTTATGATT CTGCAGAATA TGTTACCATC ATGTACCGTT AAGGTTTATT   
  
  
- TAACAGAAGG TGGGGGTCTT TTTTTTAATA CGAAAATTTT TTTCTAAAAC TCTTTAAAGT AAGAATATGT   
  
  
- CAATTTAAAT AGATTTCCTT ATTGAAGAAA TTTCAGTATT TTGTAAGGCC AGTGTTGACC CTGGTAAATG   
  
  
- CATACCATTT TTTGAGGCGT AAAATTGCTC AGTATTGAAA AGAACTAGAG TAGAATTCCT TTGTGGCTGA   
  
  
- AAAAAAGTAC GGACGCAGTA GTATGTACAA CGGGCTCCTG TGTCGGGTAA CTGCTGAGAT CCTGTTAATC   
  
  
- CATGTACTCT TCCGGAAGCT CCTCAATCGC CCTCGTTATG ACCACCTAAG TTGTTCATAA GAAAGTAGAA   
  
  
- CAGTTTCCGT ACGACTGTAA GTAGACAGGT AAGCCACCTT TGTAAGAAGA AGTCGTCGAA CTTATTACCA   
  
  
- AGTGTACACC GTCAATCAAC TCTATCAAGT GCATCATTAT TAACAGTTAC TAATGTTTTT ATACGTCATA   
  
  
- GTGTTCATTT TTAAATTCTA CTTGATTTTA TAGAGAACAA ACTCTTTACA AAGTCGAAAA TCTCAACTAC   
  
  
- AGTGCAGAAA CTCGAACGTA CATACATTA

  
  
Motifs Found  

+   

| Site Name | Organism | Position | Strand | Matrix score. | sequence | function |
| --- | --- | --- | --- | --- | --- | --- |
|  | organism | 890 | - | 4 | motif\_sequence | short\_function |
|  | organism | 1284 | + | 4 | motif\_sequence | short\_function |
|  | organism | 90 | - | 4 | motif\_sequence | short\_function |
|  | organism | 1191 | - | 4 | motif\_sequence | short\_function |
|  | organism | 134 | - | 4 | motif\_sequence | short\_function |

>033955   
+ GAAACTTTTT TACCTCTGAA ATTTACATGT CTTTGTGAAG GGAAAGATAG ATGTGGAAAA TAGAGAAGCA   
  
  
+ AGGGCGCAGT TAAGGTTGTG GAGAGAAGAC AAAGCTGAAA GGGTCAAATG TGTGGAAAGG AGGGAAGAGT   
  
  
+ AATGGAAGGC TCAATTAGTT TGGTGGTTCT TTGTCTAATA ATAGGAGAAC CATATCACTA TTTTGTTTTA   
  
  
+ TTTTGATATC TTATGTATAA TGATGTTTGT AACAAAATAT TTTCTCTTTT GATTTTTATT TTTACAACGG   
  
  
+ ATAGGATAAT TGTCTAAACG ATATTAGGGA GCAAGATTTG AACTCAAGAT GCCGTAAGTT GAAGGAAAGA   
  
  
+ CCTATCCACA AAGCAATACA CCGCTTGCTA ATAAAAACAC GCTTGTTAAA CATATAATAA TTATGTTGCC   
  
  
+ ACTCATCTTA CGATTTTAAC GGTAATATTT TTGTTCACTT TTAAGGGCAA AGACAAAAAC TTTCAATGGG   
  
  
+ ATAGCTAAAC ATGTTATACA TTTAGCATAA GCAACCCTAA TTAAGCACTA GCAACCTTAT ACATGTTATG   
  
  
+ CATTTAAGGC AGGTCTTATT GGCAATGGTG AGCTAGAGGG GGGCACGGGC ACGGTATTGT GCATAATGAA   
  
  
+ GAAATTTTTT TTGTTTTTTT AAGATTAAAA AAAAATTCAT TTATAAATCA AGAGGTTGGG ACTAGTATTT   
  
  
+ AAGACAACCA CAGTTTGACC CTCGATATAA ATTTATGTAA ATATATTTAT TTAATACAAT TATCATTTAA   
  
  
+ ACGTCGAGAT AGCGATTGTA TCAAATACAT TTAAGTATTG TAATCAAATG ACAAAAAGTT CACCGTGAAC   
  
  
+ AATAGGAACC AAATTAAACA TAAATACTAA GACGTCTTAT ACAATGGTAG TACATGGCAA TTCCAAATAA   
  
  
+ ATTGTCTTCC ACCCCCAGAA AAAAAATTAT GCTTTTAAAA AAAGATTTTG AGAAATTTCA TTCTTATACA   
  
  
+ GTTAAATTTA TCTAAAGGAA TAACTTCTTT AAAGTCATAA AACATTCCGG TCACAACTGG GACCATTTAC   
  
  
+ GTATGGTAAA AAACTCCGCA TTTTAACGAG TCATAACTTT TCTTGATCTC ATCTTAAGGA AACACCGACT   
  
  
+ TTTTTTCATG CCTGCGTCAT CATACATGTT GCCCGAGGAC ACAGCCCATT GACGACTCTA GGACAATTAG   
  
  
+ GTACATGAGA AGGCCTTCGA GGAGTTAGCG GGAGCAATAC TGGTGGATTC AACAAGTATT CTTTCATCTT   
  
  
+ GTCAAAGGCA TGCTGACATT CATCTGTCCA TTCGGTGGAA ACATTCTTCT TCAGCAGCTT GAATAATGGT   
  
  
+ TCACATGTGG CAGTTAGTTG AGATAGTTCA CGTAGTAATA ATTGTCAATG ATTACAAAAA TATGCAGTAT   
  
  
+ CACAAGTAAA AATTTAAGAT GAACTAAAAT ATCTCTTGTT TGAGAAATGT TTCAGCTTTT AGAGTTGATG   
  
  
+ TCACGTCTTT GAGCTTGCAT GTATGTAAT  

- CTTTGAAAAA ATGGAGACTT TAAATGTACA GAAACACTTC CCTTTCTATC TACACCTTTT ATCTCTTCGT   
  
  
- TCCCGCGTCA ATTCCAACAC CTCTCTTCTG TTTCGACTTT CCCAGTTTAC ACACCTTTCC TCCCTTCTCA   
  
  
- TTACCTTCCG AGTTAATCAA ACCACCAAGA AACAGATTAT TATCCTCTTG GTATAGTGAT AAAACAAAAT   
  
  
- AAAACTATAG AATACATATT ACTACAAACA TTGTTTTATA AAAGAGAAAA CTAAAAATAA AAATGTTGCC   
  
  
- TATCCTATTA ACAGATTTGC TATAATCCCT CGTTCTAAAC TTGAGTTCTA CGGCATTCAA CTTCCTTTCT   
  
  
- GGATAGGTGT TTCGTTATGT GGCGAACGAT TATTTTTGTG CGAACAATTT GTATATTATT AATACAACGG   
  
  
- TGAGTAGAAT GCTAAAATTG CCATTATAAA AACAAGTGAA AATTCCCGTT TCTGTTTTTG AAAGTTACCC   
  
  
- TATCGATTTG TACAATATGT AAATCGTATT CGTTGGGATT AATTCGTGAT CGTTGGAATA TGTACAATAC   
  
  
- GTAAATTCCG TCCAGAATAA CCGTTACCAC TCGATCTCCC CCCGTGCCCG TGCCATAACA CGTATTACTT   
  
  
- CTTTAAAAAA AACAAAAAAA TTCTAATTTT TTTTTAAGTA AATATTTAGT TCTCCAACCC TGATCATAAA   
  
  
- TTCTGTTGGT GTCAAACTGG GAGCTATATT TAAATACATT TATATAAATA AATTATGTTA ATAGTAAATT   
  
  
- TGCAGCTCTA TCGCTAACAT AGTTTATGTA AATTCATAAC ATTAGTTTAC TGTTTTTCAA GTGGCACTTG   
  
  
- TTATCCTTGG TTTAATTTGT ATTTATGATT CTGCAGAATA TGTTACCATC ATGTACCGTT AAGGTTTATT   
  
  
- TAACAGAAGG TGGGGGTCTT TTTTTTAATA CGAAAATTTT TTTCTAAAAC TCTTTAAAGT AAGAATATGT   
  
  
- CAATTTAAAT AGATTTCCTT ATTGAAGAAA TTTCAGTATT TTGTAAGGCC AGTGTTGACC CTGGTAAATG   
  
  
- CATACCATTT TTTGAGGCGT AAAATTGCTC AGTATTGAAA AGAACTAGAG TAGAATTCCT TTGTGGCTGA   
  
  
- AAAAAAGTAC GGACGCAGTA GTATGTACAA CGGGCTCCTG TGTCGGGTAA CTGCTGAGAT CCTGTTAATC   
  
  
- CATGTACTCT TCCGGAAGCT CCTCAATCGC CCTCGTTATG ACCACCTAAG TTGTTCATAA GAAAGTAGAA   
  
  
- CAGTTTCCGT ACGACTGTAA GTAGACAGGT AAGCCACCTT TGTAAGAAGA AGTCGTCGAA CTTATTACCA   
  
  
- AGTGTACACC GTCAATCAAC TCTATCAAGT GCATCATTAT TAACAGTTAC TAATGTTTTT ATACGTCATA   
  
  
- GTGTTCATTT TTAAATTCTA CTTGATTTTA TAGAGAACAA ACTCTTTACA AAGTCGAAAA TCTCAACTAC   
  
  
- AGTGCAGAAA CTCGAACGTA CATACATTA

+     AAGAA-motif

| Site Name | Organism | Position | Strand | Matrix score. | sequence | function |
| --- | --- | --- | --- | --- | --- | --- |
| AAGAA-motif | Avena sativa | 1249 | - | 7 | GAAAGAA |  |

>033955   
+ GAAACTTTTT TACCTCTGAA ATTTACATGT CTTTGTGAAG GGAAAGATAG ATGTGGAAAA TAGAGAAGCA   
  
  
+ AGGGCGCAGT TAAGGTTGTG GAGAGAAGAC AAAGCTGAAA GGGTCAAATG TGTGGAAAGG AGGGAAGAGT   
  
  
+ AATGGAAGGC TCAATTAGTT TGGTGGTTCT TTGTCTAATA ATAGGAGAAC CATATCACTA TTTTGTTTTA   
  
  
+ TTTTGATATC TTATGTATAA TGATGTTTGT AACAAAATAT TTTCTCTTTT GATTTTTATT TTTACAACGG   
  
  
+ ATAGGATAAT TGTCTAAACG ATATTAGGGA GCAAGATTTG AACTCAAGAT GCCGTAAGTT GAAGGAAAGA   
  
  
+ CCTATCCACA AAGCAATACA CCGCTTGCTA ATAAAAACAC GCTTGTTAAA CATATAATAA TTATGTTGCC   
  
  
+ ACTCATCTTA CGATTTTAAC GGTAATATTT TTGTTCACTT TTAAGGGCAA AGACAAAAAC TTTCAATGGG   
  
  
+ ATAGCTAAAC ATGTTATACA TTTAGCATAA GCAACCCTAA TTAAGCACTA GCAACCTTAT ACATGTTATG   
  
  
+ CATTTAAGGC AGGTCTTATT GGCAATGGTG AGCTAGAGGG GGGCACGGGC ACGGTATTGT GCATAATGAA   
  
  
+ GAAATTTTTT TTGTTTTTTT AAGATTAAAA AAAAATTCAT TTATAAATCA AGAGGTTGGG ACTAGTATTT   
  
  
+ AAGACAACCA CAGTTTGACC CTCGATATAA ATTTATGTAA ATATATTTAT TTAATACAAT TATCATTTAA   
  
  
+ ACGTCGAGAT AGCGATTGTA TCAAATACAT TTAAGTATTG TAATCAAATG ACAAAAAGTT CACCGTGAAC   
  
  
+ AATAGGAACC AAATTAAACA TAAATACTAA GACGTCTTAT ACAATGGTAG TACATGGCAA TTCCAAATAA   
  
  
+ ATTGTCTTCC ACCCCCAGAA AAAAAATTAT GCTTTTAAAA AAAGATTTTG AGAAATTTCA TTCTTATACA   
  
  
+ GTTAAATTTA TCTAAAGGAA TAACTTCTTT AAAGTCATAA AACATTCCGG TCACAACTGG GACCATTTAC   
  
  
+ GTATGGTAAA AAACTCCGCA TTTTAACGAG TCATAACTTT TCTTGATCTC ATCTTAAGGA AACACCGACT   
  
  
+ TTTTTTCATG CCTGCGTCAT CATACATGTT GCCCGAGGAC ACAGCCCATT GACGACTCTA GGACAATTAG   
  
  
+ GTACATGAGA AGGCCTTCGA GGAGTTAGCG GGAGCAATAC TGGTGGATTC AACAAGTATT CTTTCATCTT   
  
  
+ GTCAAAGGCA TGCTGACATT CATCTGTCCA TTCGGTGGAA ACATTCTTCT TCAGCAGCTT GAATAATGGT   
  
  
+ TCACATGTGG CAGTTAGTTG AGATAGTTCA CGTAGTAATA ATTGTCAATG ATTACAAAAA TATGCAGTAT   
  
  
+ CACAAGTAAA AATTTAAGAT GAACTAAAAT ATCTCTTGTT TGAGAAATGT TTCAGCTTTT AGAGTTGATG   
  
  
+ TCACGTCTTT GAGCTTGCAT GTATGTAAT  

- CTTTGAAAAA ATGGAGACTT TAAATGTACA GAAACACTTC CCTTTCTATC TACACCTTTT ATCTCTTCGT   
  
  
- TCCCGCGTCA ATTCCAACAC CTCTCTTCTG TTTCGACTTT CCCAGTTTAC ACACCTTTCC TCCCTTCTCA   
  
  
- TTACCTTCCG AGTTAATCAA ACCACCAAGA AACAGATTAT TATCCTCTTG GTATAGTGAT AAAACAAAAT   
  
  
- AAAACTATAG AATACATATT ACTACAAACA TTGTTTTATA AAAGAGAAAA CTAAAAATAA AAATGTTGCC   
  
  
- TATCCTATTA ACAGATTTGC TATAATCCCT CGTTCTAAAC TTGAGTTCTA CGGCATTCAA CTTCCTTTCT   
  
  
- GGATAGGTGT TTCGTTATGT GGCGAACGAT TATTTTTGTG CGAACAATTT GTATATTATT AATACAACGG   
  
  
- TGAGTAGAAT GCTAAAATTG CCATTATAAA AACAAGTGAA AATTCCCGTT TCTGTTTTTG AAAGTTACCC   
  
  
- TATCGATTTG TACAATATGT AAATCGTATT CGTTGGGATT AATTCGTGAT CGTTGGAATA TGTACAATAC   
  
  
- GTAAATTCCG TCCAGAATAA CCGTTACCAC TCGATCTCCC CCCGTGCCCG TGCCATAACA CGTATTACTT   
  
  
- CTTTAAAAAA AACAAAAAAA TTCTAATTTT TTTTTAAGTA AATATTTAGT TCTCCAACCC TGATCATAAA   
  
  
- TTCTGTTGGT GTCAAACTGG GAGCTATATT TAAATACATT TATATAAATA AATTATGTTA ATAGTAAATT   
  
  
- TGCAGCTCTA TCGCTAACAT AGTTTATGTA AATTCATAAC ATTAGTTTAC TGTTTTTCAA GTGGCACTTG   
  
  
- TTATCCTTGG TTTAATTTGT ATTTATGATT CTGCAGAATA TGTTACCATC ATGTACCGTT AAGGTTTATT   
  
  
- TAACAGAAGG TGGGGGTCTT TTTTTTAATA CGAAAATTTT TTTCTAAAAC TCTTTAAAGT AAGAATATGT   
  
  
- CAATTTAAAT AGATTTCCTT ATTGAAGAAA TTTCAGTATT TTGTAAGGCC AGTGTTGACC CTGGTAAATG   
  
  
- CATACCATTT TTTGAGGCGT AAAATTGCTC AGTATTGAAA AGAACTAGAG TAGAATTCCT TTGTGGCTGA   
  
  
- AAAAAAGTAC GGACGCAGTA GTATGTACAA CGGGCTCCTG TGTCGGGTAA CTGCTGAGAT CCTGTTAATC   
  
  
- CATGTACTCT TCCGGAAGCT CCTCAATCGC CCTCGTTATG ACCACCTAAG TTGTTCATAA GAAAGTAGAA   
  
  
- CAGTTTCCGT ACGACTGTAA GTAGACAGGT AAGCCACCTT TGTAAGAAGA AGTCGTCGAA CTTATTACCA   
  
  
- AGTGTACACC GTCAATCAAC TCTATCAAGT GCATCATTAT TAACAGTTAC TAATGTTTTT ATACGTCATA   
  
  
- GTGTTCATTT TTAAATTCTA CTTGATTTTA TAGAGAACAA ACTCTTTACA AAGTCGAAAA TCTCAACTAC   
  
  
- AGTGCAGAAA CTCGAACGTA CATACATTA

+     ABRE

| Site Name | Organism | Position | Strand | Matrix score. | sequence | function |
| --- | --- | --- | --- | --- | --- | --- |
| ABRE | Arabidopsis thaliana | 1472 | - | 5 | ACGTG | cis-acting element involved in the abscisic acid responsiveness |
| ABRE | Arabidopsis thaliana | 1359 | - | 5 | ACGTG | cis-acting element involved in the abscisic acid responsiveness |

>033955   
+ GAAACTTTTT TACCTCTGAA ATTTACATGT CTTTGTGAAG GGAAAGATAG ATGTGGAAAA TAGAGAAGCA   
  
  
+ AGGGCGCAGT TAAGGTTGTG GAGAGAAGAC AAAGCTGAAA GGGTCAAATG TGTGGAAAGG AGGGAAGAGT   
  
  
+ AATGGAAGGC TCAATTAGTT TGGTGGTTCT TTGTCTAATA ATAGGAGAAC CATATCACTA TTTTGTTTTA   
  
  
+ TTTTGATATC TTATGTATAA TGATGTTTGT AACAAAATAT TTTCTCTTTT GATTTTTATT TTTACAACGG   
  
  
+ ATAGGATAAT TGTCTAAACG ATATTAGGGA GCAAGATTTG AACTCAAGAT GCCGTAAGTT GAAGGAAAGA   
  
  
+ CCTATCCACA AAGCAATACA CCGCTTGCTA ATAAAAACAC GCTTGTTAAA CATATAATAA TTATGTTGCC   
  
  
+ ACTCATCTTA CGATTTTAAC GGTAATATTT TTGTTCACTT TTAAGGGCAA AGACAAAAAC TTTCAATGGG   
  
  
+ ATAGCTAAAC ATGTTATACA TTTAGCATAA GCAACCCTAA TTAAGCACTA GCAACCTTAT ACATGTTATG   
  
  
+ CATTTAAGGC AGGTCTTATT GGCAATGGTG AGCTAGAGGG GGGCACGGGC ACGGTATTGT GCATAATGAA   
  
  
+ GAAATTTTTT TTGTTTTTTT AAGATTAAAA AAAAATTCAT TTATAAATCA AGAGGTTGGG ACTAGTATTT   
  
  
+ AAGACAACCA CAGTTTGACC CTCGATATAA ATTTATGTAA ATATATTTAT TTAATACAAT TATCATTTAA   
  
  
+ ACGTCGAGAT AGCGATTGTA TCAAATACAT TTAAGTATTG TAATCAAATG ACAAAAAGTT CACCGTGAAC   
  
  
+ AATAGGAACC AAATTAAACA TAAATACTAA GACGTCTTAT ACAATGGTAG TACATGGCAA TTCCAAATAA   
  
  
+ ATTGTCTTCC ACCCCCAGAA AAAAAATTAT GCTTTTAAAA AAAGATTTTG AGAAATTTCA TTCTTATACA   
  
  
+ GTTAAATTTA TCTAAAGGAA TAACTTCTTT AAAGTCATAA AACATTCCGG TCACAACTGG GACCATTTAC   
  
  
+ GTATGGTAAA AAACTCCGCA TTTTAACGAG TCATAACTTT TCTTGATCTC ATCTTAAGGA AACACCGACT   
  
  
+ TTTTTTCATG CCTGCGTCAT CATACATGTT GCCCGAGGAC ACAGCCCATT GACGACTCTA GGACAATTAG   
  
  
+ GTACATGAGA AGGCCTTCGA GGAGTTAGCG GGAGCAATAC TGGTGGATTC AACAAGTATT CTTTCATCTT   
  
  
+ GTCAAAGGCA TGCTGACATT CATCTGTCCA TTCGGTGGAA ACATTCTTCT TCAGCAGCTT GAATAATGGT   
  
  
+ TCACATGTGG CAGTTAGTTG AGATAGTTCA CGTAGTAATA ATTGTCAATG ATTACAAAAA TATGCAGTAT   
  
  
+ CACAAGTAAA AATTTAAGAT GAACTAAAAT ATCTCTTGTT TGAGAAATGT TTCAGCTTTT AGAGTTGATG   
  
  
+ TCACGTCTTT GAGCTTGCAT GTATGTAAT  

- CTTTGAAAAA ATGGAGACTT TAAATGTACA GAAACACTTC CCTTTCTATC TACACCTTTT ATCTCTTCGT   
  
  
- TCCCGCGTCA ATTCCAACAC CTCTCTTCTG TTTCGACTTT CCCAGTTTAC ACACCTTTCC TCCCTTCTCA   
  
  
- TTACCTTCCG AGTTAATCAA ACCACCAAGA AACAGATTAT TATCCTCTTG GTATAGTGAT AAAACAAAAT   
  
  
- AAAACTATAG AATACATATT ACTACAAACA TTGTTTTATA AAAGAGAAAA CTAAAAATAA AAATGTTGCC   
  
  
- TATCCTATTA ACAGATTTGC TATAATCCCT CGTTCTAAAC TTGAGTTCTA CGGCATTCAA CTTCCTTTCT   
  
  
- GGATAGGTGT TTCGTTATGT GGCGAACGAT TATTTTTGTG CGAACAATTT GTATATTATT AATACAACGG   
  
  
- TGAGTAGAAT GCTAAAATTG CCATTATAAA AACAAGTGAA AATTCCCGTT TCTGTTTTTG AAAGTTACCC   
  
  
- TATCGATTTG TACAATATGT AAATCGTATT CGTTGGGATT AATTCGTGAT CGTTGGAATA TGTACAATAC   
  
  
- GTAAATTCCG TCCAGAATAA CCGTTACCAC TCGATCTCCC CCCGTGCCCG TGCCATAACA CGTATTACTT   
  
  
- CTTTAAAAAA AACAAAAAAA TTCTAATTTT TTTTTAAGTA AATATTTAGT TCTCCAACCC TGATCATAAA   
  
  
- TTCTGTTGGT GTCAAACTGG GAGCTATATT TAAATACATT TATATAAATA AATTATGTTA ATAGTAAATT   
  
  
- TGCAGCTCTA TCGCTAACAT AGTTTATGTA AATTCATAAC ATTAGTTTAC TGTTTTTCAA GTGGCACTTG   
  
  
- TTATCCTTGG TTTAATTTGT ATTTATGATT CTGCAGAATA TGTTACCATC ATGTACCGTT AAGGTTTATT   
  
  
- TAACAGAAGG TGGGGGTCTT TTTTTTAATA CGAAAATTTT TTTCTAAAAC TCTTTAAAGT AAGAATATGT   
  
  
- CAATTTAAAT AGATTTCCTT ATTGAAGAAA TTTCAGTATT TTGTAAGGCC AGTGTTGACC CTGGTAAATG   
  
  
- CATACCATTT TTTGAGGCGT AAAATTGCTC AGTATTGAAA AGAACTAGAG TAGAATTCCT TTGTGGCTGA   
  
  
- AAAAAAGTAC GGACGCAGTA GTATGTACAA CGGGCTCCTG TGTCGGGTAA CTGCTGAGAT CCTGTTAATC   
  
  
- CATGTACTCT TCCGGAAGCT CCTCAATCGC CCTCGTTATG ACCACCTAAG TTGTTCATAA GAAAGTAGAA   
  
  
- CAGTTTCCGT ACGACTGTAA GTAGACAGGT AAGCCACCTT TGTAAGAAGA AGTCGTCGAA CTTATTACCA   
  
  
- AGTGTACACC GTCAATCAAC TCTATCAAGT GCATCATTAT TAACAGTTAC TAATGTTTTT ATACGTCATA   
  
  
- GTGTTCATTT TTAAATTCTA CTTGATTTTA TAGAGAACAA ACTCTTTACA AAGTCGAAAA TCTCAACTAC   
  
  
- AGTGCAGAAA CTCGAACGTA CATACATTA

+     ABRE3a

| Site Name | Organism | Position | Strand | Matrix score. | sequence | function |
| --- | --- | --- | --- | --- | --- | --- |
| ABRE3a | Zea mays | 1359 | - | 6 | TACGTG |  |

>033955   
+ GAAACTTTTT TACCTCTGAA ATTTACATGT CTTTGTGAAG GGAAAGATAG ATGTGGAAAA TAGAGAAGCA   
  
  
+ AGGGCGCAGT TAAGGTTGTG GAGAGAAGAC AAAGCTGAAA GGGTCAAATG TGTGGAAAGG AGGGAAGAGT   
  
  
+ AATGGAAGGC TCAATTAGTT TGGTGGTTCT TTGTCTAATA ATAGGAGAAC CATATCACTA TTTTGTTTTA   
  
  
+ TTTTGATATC TTATGTATAA TGATGTTTGT AACAAAATAT TTTCTCTTTT GATTTTTATT TTTACAACGG   
  
  
+ ATAGGATAAT TGTCTAAACG ATATTAGGGA GCAAGATTTG AACTCAAGAT GCCGTAAGTT GAAGGAAAGA   
  
  
+ CCTATCCACA AAGCAATACA CCGCTTGCTA ATAAAAACAC GCTTGTTAAA CATATAATAA TTATGTTGCC   
  
  
+ ACTCATCTTA CGATTTTAAC GGTAATATTT TTGTTCACTT TTAAGGGCAA AGACAAAAAC TTTCAATGGG   
  
  
+ ATAGCTAAAC ATGTTATACA TTTAGCATAA GCAACCCTAA TTAAGCACTA GCAACCTTAT ACATGTTATG   
  
  
+ CATTTAAGGC AGGTCTTATT GGCAATGGTG AGCTAGAGGG GGGCACGGGC ACGGTATTGT GCATAATGAA   
  
  
+ GAAATTTTTT TTGTTTTTTT AAGATTAAAA AAAAATTCAT TTATAAATCA AGAGGTTGGG ACTAGTATTT   
  
  
+ AAGACAACCA CAGTTTGACC CTCGATATAA ATTTATGTAA ATATATTTAT TTAATACAAT TATCATTTAA   
  
  
+ ACGTCGAGAT AGCGATTGTA TCAAATACAT TTAAGTATTG TAATCAAATG ACAAAAAGTT CACCGTGAAC   
  
  
+ AATAGGAACC AAATTAAACA TAAATACTAA GACGTCTTAT ACAATGGTAG TACATGGCAA TTCCAAATAA   
  
  
+ ATTGTCTTCC ACCCCCAGAA AAAAAATTAT GCTTTTAAAA AAAGATTTTG AGAAATTTCA TTCTTATACA   
  
  
+ GTTAAATTTA TCTAAAGGAA TAACTTCTTT AAAGTCATAA AACATTCCGG TCACAACTGG GACCATTTAC   
  
  
+ GTATGGTAAA AAACTCCGCA TTTTAACGAG TCATAACTTT TCTTGATCTC ATCTTAAGGA AACACCGACT   
  
  
+ TTTTTTCATG CCTGCGTCAT CATACATGTT GCCCGAGGAC ACAGCCCATT GACGACTCTA GGACAATTAG   
  
  
+ GTACATGAGA AGGCCTTCGA GGAGTTAGCG GGAGCAATAC TGGTGGATTC AACAAGTATT CTTTCATCTT   
  
  
+ GTCAAAGGCA TGCTGACATT CATCTGTCCA TTCGGTGGAA ACATTCTTCT TCAGCAGCTT GAATAATGGT   
  
  
+ TCACATGTGG CAGTTAGTTG AGATAGTTCA CGTAGTAATA ATTGTCAATG ATTACAAAAA TATGCAGTAT   
  
  
+ CACAAGTAAA AATTTAAGAT GAACTAAAAT ATCTCTTGTT TGAGAAATGT TTCAGCTTTT AGAGTTGATG   
  
  
+ TCACGTCTTT GAGCTTGCAT GTATGTAAT  

- CTTTGAAAAA ATGGAGACTT TAAATGTACA GAAACACTTC CCTTTCTATC TACACCTTTT ATCTCTTCGT   
  
  
- TCCCGCGTCA ATTCCAACAC CTCTCTTCTG TTTCGACTTT CCCAGTTTAC ACACCTTTCC TCCCTTCTCA   
  
  
- TTACCTTCCG AGTTAATCAA ACCACCAAGA AACAGATTAT TATCCTCTTG GTATAGTGAT AAAACAAAAT   
  
  
- AAAACTATAG AATACATATT ACTACAAACA TTGTTTTATA AAAGAGAAAA CTAAAAATAA AAATGTTGCC   
  
  
- TATCCTATTA ACAGATTTGC TATAATCCCT CGTTCTAAAC TTGAGTTCTA CGGCATTCAA CTTCCTTTCT   
  
  
- GGATAGGTGT TTCGTTATGT GGCGAACGAT TATTTTTGTG CGAACAATTT GTATATTATT AATACAACGG   
  
  
- TGAGTAGAAT GCTAAAATTG CCATTATAAA AACAAGTGAA AATTCCCGTT TCTGTTTTTG AAAGTTACCC   
  
  
- TATCGATTTG TACAATATGT AAATCGTATT CGTTGGGATT AATTCGTGAT CGTTGGAATA TGTACAATAC   
  
  
- GTAAATTCCG TCCAGAATAA CCGTTACCAC TCGATCTCCC CCCGTGCCCG TGCCATAACA CGTATTACTT   
  
  
- CTTTAAAAAA AACAAAAAAA TTCTAATTTT TTTTTAAGTA AATATTTAGT TCTCCAACCC TGATCATAAA   
  
  
- TTCTGTTGGT GTCAAACTGG GAGCTATATT TAAATACATT TATATAAATA AATTATGTTA ATAGTAAATT   
  
  
- TGCAGCTCTA TCGCTAACAT AGTTTATGTA AATTCATAAC ATTAGTTTAC TGTTTTTCAA GTGGCACTTG   
  
  
- TTATCCTTGG TTTAATTTGT ATTTATGATT CTGCAGAATA TGTTACCATC ATGTACCGTT AAGGTTTATT   
  
  
- TAACAGAAGG TGGGGGTCTT TTTTTTAATA CGAAAATTTT TTTCTAAAAC TCTTTAAAGT AAGAATATGT   
  
  
- CAATTTAAAT AGATTTCCTT ATTGAAGAAA TTTCAGTATT TTGTAAGGCC AGTGTTGACC CTGGTAAATG   
  
  
- CATACCATTT TTTGAGGCGT AAAATTGCTC AGTATTGAAA AGAACTAGAG TAGAATTCCT TTGTGGCTGA   
  
  
- AAAAAAGTAC GGACGCAGTA GTATGTACAA CGGGCTCCTG TGTCGGGTAA CTGCTGAGAT CCTGTTAATC   
  
  
- CATGTACTCT TCCGGAAGCT CCTCAATCGC CCTCGTTATG ACCACCTAAG TTGTTCATAA GAAAGTAGAA   
  
  
- CAGTTTCCGT ACGACTGTAA GTAGACAGGT AAGCCACCTT TGTAAGAAGA AGTCGTCGAA CTTATTACCA   
  
  
- AGTGTACACC GTCAATCAAC TCTATCAAGT GCATCATTAT TAACAGTTAC TAATGTTTTT ATACGTCATA   
  
  
- GTGTTCATTT TTAAATTCTA CTTGATTTTA TAGAGAACAA ACTCTTTACA AAGTCGAAAA TCTCAACTAC   
  
  
- AGTGCAGAAA CTCGAACGTA CATACATTA

+     ABRE4

| Site Name | Organism | Position | Strand | Matrix score. | sequence | function |
| --- | --- | --- | --- | --- | --- | --- |
| ABRE4 | Zea mays | 1359 | + | 6 | CACGTA |  |

>033955   
+ GAAACTTTTT TACCTCTGAA ATTTACATGT CTTTGTGAAG GGAAAGATAG ATGTGGAAAA TAGAGAAGCA   
  
  
+ AGGGCGCAGT TAAGGTTGTG GAGAGAAGAC AAAGCTGAAA GGGTCAAATG TGTGGAAAGG AGGGAAGAGT   
  
  
+ AATGGAAGGC TCAATTAGTT TGGTGGTTCT TTGTCTAATA ATAGGAGAAC CATATCACTA TTTTGTTTTA   
  
  
+ TTTTGATATC TTATGTATAA TGATGTTTGT AACAAAATAT TTTCTCTTTT GATTTTTATT TTTACAACGG   
  
  
+ ATAGGATAAT TGTCTAAACG ATATTAGGGA GCAAGATTTG AACTCAAGAT GCCGTAAGTT GAAGGAAAGA   
  
  
+ CCTATCCACA AAGCAATACA CCGCTTGCTA ATAAAAACAC GCTTGTTAAA CATATAATAA TTATGTTGCC   
  
  
+ ACTCATCTTA CGATTTTAAC GGTAATATTT TTGTTCACTT TTAAGGGCAA AGACAAAAAC TTTCAATGGG   
  
  
+ ATAGCTAAAC ATGTTATACA TTTAGCATAA GCAACCCTAA TTAAGCACTA GCAACCTTAT ACATGTTATG   
  
  
+ CATTTAAGGC AGGTCTTATT GGCAATGGTG AGCTAGAGGG GGGCACGGGC ACGGTATTGT GCATAATGAA   
  
  
+ GAAATTTTTT TTGTTTTTTT AAGATTAAAA AAAAATTCAT TTATAAATCA AGAGGTTGGG ACTAGTATTT   
  
  
+ AAGACAACCA CAGTTTGACC CTCGATATAA ATTTATGTAA ATATATTTAT TTAATACAAT TATCATTTAA   
  
  
+ ACGTCGAGAT AGCGATTGTA TCAAATACAT TTAAGTATTG TAATCAAATG ACAAAAAGTT CACCGTGAAC   
  
  
+ AATAGGAACC AAATTAAACA TAAATACTAA GACGTCTTAT ACAATGGTAG TACATGGCAA TTCCAAATAA   
  
  
+ ATTGTCTTCC ACCCCCAGAA AAAAAATTAT GCTTTTAAAA AAAGATTTTG AGAAATTTCA TTCTTATACA   
  
  
+ GTTAAATTTA TCTAAAGGAA TAACTTCTTT AAAGTCATAA AACATTCCGG TCACAACTGG GACCATTTAC   
  
  
+ GTATGGTAAA AAACTCCGCA TTTTAACGAG TCATAACTTT TCTTGATCTC ATCTTAAGGA AACACCGACT   
  
  
+ TTTTTTCATG CCTGCGTCAT CATACATGTT GCCCGAGGAC ACAGCCCATT GACGACTCTA GGACAATTAG   
  
  
+ GTACATGAGA AGGCCTTCGA GGAGTTAGCG GGAGCAATAC TGGTGGATTC AACAAGTATT CTTTCATCTT   
  
  
+ GTCAAAGGCA TGCTGACATT CATCTGTCCA TTCGGTGGAA ACATTCTTCT TCAGCAGCTT GAATAATGGT   
  
  
+ TCACATGTGG CAGTTAGTTG AGATAGTTCA CGTAGTAATA ATTGTCAATG ATTACAAAAA TATGCAGTAT   
  
  
+ CACAAGTAAA AATTTAAGAT GAACTAAAAT ATCTCTTGTT TGAGAAATGT TTCAGCTTTT AGAGTTGATG   
  
  
+ TCACGTCTTT GAGCTTGCAT GTATGTAAT  

- CTTTGAAAAA ATGGAGACTT TAAATGTACA GAAACACTTC CCTTTCTATC TACACCTTTT ATCTCTTCGT   
  
  
- TCCCGCGTCA ATTCCAACAC CTCTCTTCTG TTTCGACTTT CCCAGTTTAC ACACCTTTCC TCCCTTCTCA   
  
  
- TTACCTTCCG AGTTAATCAA ACCACCAAGA AACAGATTAT TATCCTCTTG GTATAGTGAT AAAACAAAAT   
  
  
- AAAACTATAG AATACATATT ACTACAAACA TTGTTTTATA AAAGAGAAAA CTAAAAATAA AAATGTTGCC   
  
  
- TATCCTATTA ACAGATTTGC TATAATCCCT CGTTCTAAAC TTGAGTTCTA CGGCATTCAA CTTCCTTTCT   
  
  
- GGATAGGTGT TTCGTTATGT GGCGAACGAT TATTTTTGTG CGAACAATTT GTATATTATT AATACAACGG   
  
  
- TGAGTAGAAT GCTAAAATTG CCATTATAAA AACAAGTGAA AATTCCCGTT TCTGTTTTTG AAAGTTACCC   
  
  
- TATCGATTTG TACAATATGT AAATCGTATT CGTTGGGATT AATTCGTGAT CGTTGGAATA TGTACAATAC   
  
  
- GTAAATTCCG TCCAGAATAA CCGTTACCAC TCGATCTCCC CCCGTGCCCG TGCCATAACA CGTATTACTT   
  
  
- CTTTAAAAAA AACAAAAAAA TTCTAATTTT TTTTTAAGTA AATATTTAGT TCTCCAACCC TGATCATAAA   
  
  
- TTCTGTTGGT GTCAAACTGG GAGCTATATT TAAATACATT TATATAAATA AATTATGTTA ATAGTAAATT   
  
  
- TGCAGCTCTA TCGCTAACAT AGTTTATGTA AATTCATAAC ATTAGTTTAC TGTTTTTCAA GTGGCACTTG   
  
  
- TTATCCTTGG TTTAATTTGT ATTTATGATT CTGCAGAATA TGTTACCATC ATGTACCGTT AAGGTTTATT   
  
  
- TAACAGAAGG TGGGGGTCTT TTTTTTAATA CGAAAATTTT TTTCTAAAAC TCTTTAAAGT AAGAATATGT   
  
  
- CAATTTAAAT AGATTTCCTT ATTGAAGAAA TTTCAGTATT TTGTAAGGCC AGTGTTGACC CTGGTAAATG   
  
  
- CATACCATTT TTTGAGGCGT AAAATTGCTC AGTATTGAAA AGAACTAGAG TAGAATTCCT TTGTGGCTGA   
  
  
- AAAAAAGTAC GGACGCAGTA GTATGTACAA CGGGCTCCTG TGTCGGGTAA CTGCTGAGAT CCTGTTAATC   
  
  
- CATGTACTCT TCCGGAAGCT CCTCAATCGC CCTCGTTATG ACCACCTAAG TTGTTCATAA GAAAGTAGAA   
  
  
- CAGTTTCCGT ACGACTGTAA GTAGACAGGT AAGCCACCTT TGTAAGAAGA AGTCGTCGAA CTTATTACCA   
  
  
- AGTGTACACC GTCAATCAAC TCTATCAAGT GCATCATTAT TAACAGTTAC TAATGTTTTT ATACGTCATA   
  
  
- GTGTTCATTT TTAAATTCTA CTTGATTTTA TAGAGAACAA ACTCTTTACA AAGTCGAAAA TCTCAACTAC   
  
  
- AGTGCAGAAA CTCGAACGTA CATACATTA

+     AT-rich element

| Site Name | Organism | Position | Strand | Matrix score. | sequence | function |
| --- | --- | --- | --- | --- | --- | --- |
| AT-rich element | Glycine max | 259 | - | 10 | ATAGAAATCAA | binding site of AT-rich DNA binding protein (ATBP-1) |

>033955   
+ GAAACTTTTT TACCTCTGAA ATTTACATGT CTTTGTGAAG GGAAAGATAG ATGTGGAAAA TAGAGAAGCA   
  
  
+ AGGGCGCAGT TAAGGTTGTG GAGAGAAGAC AAAGCTGAAA GGGTCAAATG TGTGGAAAGG AGGGAAGAGT   
  
  
+ AATGGAAGGC TCAATTAGTT TGGTGGTTCT TTGTCTAATA ATAGGAGAAC CATATCACTA TTTTGTTTTA   
  
  
+ TTTTGATATC TTATGTATAA TGATGTTTGT AACAAAATAT TTTCTCTTTT GATTTTTATT TTTACAACGG   
  
  
+ ATAGGATAAT TGTCTAAACG ATATTAGGGA GCAAGATTTG AACTCAAGAT GCCGTAAGTT GAAGGAAAGA   
  
  
+ CCTATCCACA AAGCAATACA CCGCTTGCTA ATAAAAACAC GCTTGTTAAA CATATAATAA TTATGTTGCC   
  
  
+ ACTCATCTTA CGATTTTAAC GGTAATATTT TTGTTCACTT TTAAGGGCAA AGACAAAAAC TTTCAATGGG   
  
  
+ ATAGCTAAAC ATGTTATACA TTTAGCATAA GCAACCCTAA TTAAGCACTA GCAACCTTAT ACATGTTATG   
  
  
+ CATTTAAGGC AGGTCTTATT GGCAATGGTG AGCTAGAGGG GGGCACGGGC ACGGTATTGT GCATAATGAA   
  
  
+ GAAATTTTTT TTGTTTTTTT AAGATTAAAA AAAAATTCAT TTATAAATCA AGAGGTTGGG ACTAGTATTT   
  
  
+ AAGACAACCA CAGTTTGACC CTCGATATAA ATTTATGTAA ATATATTTAT TTAATACAAT TATCATTTAA   
  
  
+ ACGTCGAGAT AGCGATTGTA TCAAATACAT TTAAGTATTG TAATCAAATG ACAAAAAGTT CACCGTGAAC   
  
  
+ AATAGGAACC AAATTAAACA TAAATACTAA GACGTCTTAT ACAATGGTAG TACATGGCAA TTCCAAATAA   
  
  
+ ATTGTCTTCC ACCCCCAGAA AAAAAATTAT GCTTTTAAAA AAAGATTTTG AGAAATTTCA TTCTTATACA   
  
  
+ GTTAAATTTA TCTAAAGGAA TAACTTCTTT AAAGTCATAA AACATTCCGG TCACAACTGG GACCATTTAC   
  
  
+ GTATGGTAAA AAACTCCGCA TTTTAACGAG TCATAACTTT TCTTGATCTC ATCTTAAGGA AACACCGACT   
  
  
+ TTTTTTCATG CCTGCGTCAT CATACATGTT GCCCGAGGAC ACAGCCCATT GACGACTCTA GGACAATTAG   
  
  
+ GTACATGAGA AGGCCTTCGA GGAGTTAGCG GGAGCAATAC TGGTGGATTC AACAAGTATT CTTTCATCTT   
  
  
+ GTCAAAGGCA TGCTGACATT CATCTGTCCA TTCGGTGGAA ACATTCTTCT TCAGCAGCTT GAATAATGGT   
  
  
+ TCACATGTGG CAGTTAGTTG AGATAGTTCA CGTAGTAATA ATTGTCAATG ATTACAAAAA TATGCAGTAT   
  
  
+ CACAAGTAAA AATTTAAGAT GAACTAAAAT ATCTCTTGTT TGAGAAATGT TTCAGCTTTT AGAGTTGATG   
  
  
+ TCACGTCTTT GAGCTTGCAT GTATGTAAT  

- CTTTGAAAAA ATGGAGACTT TAAATGTACA GAAACACTTC CCTTTCTATC TACACCTTTT ATCTCTTCGT   
  
  
- TCCCGCGTCA ATTCCAACAC CTCTCTTCTG TTTCGACTTT CCCAGTTTAC ACACCTTTCC TCCCTTCTCA   
  
  
- TTACCTTCCG AGTTAATCAA ACCACCAAGA AACAGATTAT TATCCTCTTG GTATAGTGAT AAAACAAAAT   
  
  
- AAAACTATAG AATACATATT ACTACAAACA TTGTTTTATA AAAGAGAAAA CTAAAAATAA AAATGTTGCC   
  
  
- TATCCTATTA ACAGATTTGC TATAATCCCT CGTTCTAAAC TTGAGTTCTA CGGCATTCAA CTTCCTTTCT   
  
  
- GGATAGGTGT TTCGTTATGT GGCGAACGAT TATTTTTGTG CGAACAATTT GTATATTATT AATACAACGG   
  
  
- TGAGTAGAAT GCTAAAATTG CCATTATAAA AACAAGTGAA AATTCCCGTT TCTGTTTTTG AAAGTTACCC   
  
  
- TATCGATTTG TACAATATGT AAATCGTATT CGTTGGGATT AATTCGTGAT CGTTGGAATA TGTACAATAC   
  
  
- GTAAATTCCG TCCAGAATAA CCGTTACCAC TCGATCTCCC CCCGTGCCCG TGCCATAACA CGTATTACTT   
  
  
- CTTTAAAAAA AACAAAAAAA TTCTAATTTT TTTTTAAGTA AATATTTAGT TCTCCAACCC TGATCATAAA   
  
  
- TTCTGTTGGT GTCAAACTGG GAGCTATATT TAAATACATT TATATAAATA AATTATGTTA ATAGTAAATT   
  
  
- TGCAGCTCTA TCGCTAACAT AGTTTATGTA AATTCATAAC ATTAGTTTAC TGTTTTTCAA GTGGCACTTG   
  
  
- TTATCCTTGG TTTAATTTGT ATTTATGATT CTGCAGAATA TGTTACCATC ATGTACCGTT AAGGTTTATT   
  
  
- TAACAGAAGG TGGGGGTCTT TTTTTTAATA CGAAAATTTT TTTCTAAAAC TCTTTAAAGT AAGAATATGT   
  
  
- CAATTTAAAT AGATTTCCTT ATTGAAGAAA TTTCAGTATT TTGTAAGGCC AGTGTTGACC CTGGTAAATG   
  
  
- CATACCATTT TTTGAGGCGT AAAATTGCTC AGTATTGAAA AGAACTAGAG TAGAATTCCT TTGTGGCTGA   
  
  
- AAAAAAGTAC GGACGCAGTA GTATGTACAA CGGGCTCCTG TGTCGGGTAA CTGCTGAGAT CCTGTTAATC   
  
  
- CATGTACTCT TCCGGAAGCT CCTCAATCGC CCTCGTTATG ACCACCTAAG TTGTTCATAA GAAAGTAGAA   
  
  
- CAGTTTCCGT ACGACTGTAA GTAGACAGGT AAGCCACCTT TGTAAGAAGA AGTCGTCGAA CTTATTACCA   
  
  
- AGTGTACACC GTCAATCAAC TCTATCAAGT GCATCATTAT TAACAGTTAC TAATGTTTTT ATACGTCATA   
  
  
- GTGTTCATTT TTAAATTCTA CTTGATTTTA TAGAGAACAA ACTCTTTACA AAGTCGAAAA TCTCAACTAC   
  
  
- AGTGCAGAAA CTCGAACGTA CATACATTA

+     CAAT-box

| Site Name | Organism | Position | Strand | Matrix score. | sequence | function |
| --- | --- | --- | --- | --- | --- | --- |
| CAAT-box | Nicotiana glutinosa | 289 | - | 4 | CAAT |  |
| CAAT-box | Nicotiana glutinosa | 840 | + | 4 | CAAT |  |
| CAAT-box | Nicotiana glutinosa | 757 | + | 4 | CAAT |  |
| CAAT-box | Nicotiana glutinosa | 1184 | + | 4 | CAAT |  |
| CAAT-box | Nicotiana glutinosa | 807 | - | 4 | CAAT |  |
| CAAT-box | Pisum sativum | 904 | + | 5 | CAAAT | common cis-acting element in promoter and enhancer regions |
| CAAT-box | Nicotiana glutinosa | 583 | + | 4 | CAAT |  |
| CAAT-box | Nicotiana glutinosa | 1376 | + | 4 | CAAT |  |
| CAAT-box | Pisum sativum | 316 | - | 5 | CAAAT | common cis-acting element in promoter and enhancer regions |
| CAAT-box | Nicotiana glutinosa | 882 | + | 4 | CAAT |  |
| CAAT-box | Nicotiana glutinosa | 785 | - | 4 | CAAT |  |
| CAAT-box | Nicotiana glutinosa | 911 | - | 4 | CAAT |  |
| CAAT-box | Arabidopsis thaliana | 578 | - | 5 | CCAAT | common cis-acting element in promoter and enhancer regions |
| CAAT-box | Nicotiana glutinosa | 1371 | - | 4 | CAAT |  |
| CAAT-box | Pisum sativum | 815 | + | 5 | CAAAT | common cis-acting element in promoter and enhancer regions |
| CAAT-box | Pisum sativum | 850 | + | 5 | CAAAT | common cis-acting element in promoter and enhancer regions |
| CAAT-box | Pisum sativum | 115 | + | 5 | CAAAT | common cis-acting element in promoter and enhancer regions |
| CAAT-box | Nicotiana glutinosa | 1168 | - | 4 | CAAT |  |
| CAAT-box | Nicotiana glutinosa | 616 | - | 4 | CAAT |  |
| CAAT-box | Nicotiana glutinosa | 898 | + | 4 | CAAT |  |
| CAAT-box | Nicotiana glutinosa | 1225 | + | 4 | CAAT |  |
| CAAT-box | Nicotiana glutinosa | 364 | + | 4 | CAAT |  |
| CAAT-box | Pisum sativum | 792 | + | 5 | CAAAT | common cis-acting element in promoter and enhancer regions |
| CAAT-box | Nicotiana glutinosa | 152 | + | 4 | CAAT |  |
| CAAT-box | Nicotiana glutinosa | 484 | + | 4 | CAAT |  |

>033955   
+ GAAACTTTTT TACCTCTGAA ATTTACATGT CTTTGTGAAG GGAAAGATAG ATGTGGAAAA TAGAGAAGCA   
  
  
+ AGGGCGCAGT TAAGGTTGTG GAGAGAAGAC AAAGCTGAAA GGGTCAAATG TGTGGAAAGG AGGGAAGAGT   
  
  
+ AATGGAAGGC TCAATTAGTT TGGTGGTTCT TTGTCTAATA ATAGGAGAAC CATATCACTA TTTTGTTTTA   
  
  
+ TTTTGATATC TTATGTATAA TGATGTTTGT AACAAAATAT TTTCTCTTTT GATTTTTATT TTTACAACGG   
  
  
+ ATAGGATAAT TGTCTAAACG ATATTAGGGA GCAAGATTTG AACTCAAGAT GCCGTAAGTT GAAGGAAAGA   
  
  
+ CCTATCCACA AAGCAATACA CCGCTTGCTA ATAAAAACAC GCTTGTTAAA CATATAATAA TTATGTTGCC   
  
  
+ ACTCATCTTA CGATTTTAAC GGTAATATTT TTGTTCACTT TTAAGGGCAA AGACAAAAAC TTTCAATGGG   
  
  
+ ATAGCTAAAC ATGTTATACA TTTAGCATAA GCAACCCTAA TTAAGCACTA GCAACCTTAT ACATGTTATG   
  
  
+ CATTTAAGGC AGGTCTTATT GGCAATGGTG AGCTAGAGGG GGGCACGGGC ACGGTATTGT GCATAATGAA   
  
  
+ GAAATTTTTT TTGTTTTTTT AAGATTAAAA AAAAATTCAT TTATAAATCA AGAGGTTGGG ACTAGTATTT   
  
  
+ AAGACAACCA CAGTTTGACC CTCGATATAA ATTTATGTAA ATATATTTAT TTAATACAAT TATCATTTAA   
  
  
+ ACGTCGAGAT AGCGATTGTA TCAAATACAT TTAAGTATTG TAATCAAATG ACAAAAAGTT CACCGTGAAC   
  
  
+ AATAGGAACC AAATTAAACA TAAATACTAA GACGTCTTAT ACAATGGTAG TACATGGCAA TTCCAAATAA   
  
  
+ ATTGTCTTCC ACCCCCAGAA AAAAAATTAT GCTTTTAAAA AAAGATTTTG AGAAATTTCA TTCTTATACA   
  
  
+ GTTAAATTTA TCTAAAGGAA TAACTTCTTT AAAGTCATAA AACATTCCGG TCACAACTGG GACCATTTAC   
  
  
+ GTATGGTAAA AAACTCCGCA TTTTAACGAG TCATAACTTT TCTTGATCTC ATCTTAAGGA AACACCGACT   
  
  
+ TTTTTTCATG CCTGCGTCAT CATACATGTT GCCCGAGGAC ACAGCCCATT GACGACTCTA GGACAATTAG   
  
  
+ GTACATGAGA AGGCCTTCGA GGAGTTAGCG GGAGCAATAC TGGTGGATTC AACAAGTATT CTTTCATCTT   
  
  
+ GTCAAAGGCA TGCTGACATT CATCTGTCCA TTCGGTGGAA ACATTCTTCT TCAGCAGCTT GAATAATGGT   
  
  
+ TCACATGTGG CAGTTAGTTG AGATAGTTCA CGTAGTAATA ATTGTCAATG ATTACAAAAA TATGCAGTAT   
  
  
+ CACAAGTAAA AATTTAAGAT GAACTAAAAT ATCTCTTGTT TGAGAAATGT TTCAGCTTTT AGAGTTGATG   
  
  
+ TCACGTCTTT GAGCTTGCAT GTATGTAAT  

- CTTTGAAAAA ATGGAGACTT TAAATGTACA GAAACACTTC CCTTTCTATC TACACCTTTT ATCTCTTCGT   
  
  
- TCCCGCGTCA ATTCCAACAC CTCTCTTCTG TTTCGACTTT CCCAGTTTAC ACACCTTTCC TCCCTTCTCA   
  
  
- TTACCTTCCG AGTTAATCAA ACCACCAAGA AACAGATTAT TATCCTCTTG GTATAGTGAT AAAACAAAAT   
  
  
- AAAACTATAG AATACATATT ACTACAAACA TTGTTTTATA AAAGAGAAAA CTAAAAATAA AAATGTTGCC   
  
  
- TATCCTATTA ACAGATTTGC TATAATCCCT CGTTCTAAAC TTGAGTTCTA CGGCATTCAA CTTCCTTTCT   
  
  
- GGATAGGTGT TTCGTTATGT GGCGAACGAT TATTTTTGTG CGAACAATTT GTATATTATT AATACAACGG   
  
  
- TGAGTAGAAT GCTAAAATTG CCATTATAAA AACAAGTGAA AATTCCCGTT TCTGTTTTTG AAAGTTACCC   
  
  
- TATCGATTTG TACAATATGT AAATCGTATT CGTTGGGATT AATTCGTGAT CGTTGGAATA TGTACAATAC   
  
  
- GTAAATTCCG TCCAGAATAA CCGTTACCAC TCGATCTCCC CCCGTGCCCG TGCCATAACA CGTATTACTT   
  
  
- CTTTAAAAAA AACAAAAAAA TTCTAATTTT TTTTTAAGTA AATATTTAGT TCTCCAACCC TGATCATAAA   
  
  
- TTCTGTTGGT GTCAAACTGG GAGCTATATT TAAATACATT TATATAAATA AATTATGTTA ATAGTAAATT   
  
  
- TGCAGCTCTA TCGCTAACAT AGTTTATGTA AATTCATAAC ATTAGTTTAC TGTTTTTCAA GTGGCACTTG   
  
  
- TTATCCTTGG TTTAATTTGT ATTTATGATT CTGCAGAATA TGTTACCATC ATGTACCGTT AAGGTTTATT   
  
  
- TAACAGAAGG TGGGGGTCTT TTTTTTAATA CGAAAATTTT TTTCTAAAAC TCTTTAAAGT AAGAATATGT   
  
  
- CAATTTAAAT AGATTTCCTT ATTGAAGAAA TTTCAGTATT TTGTAAGGCC AGTGTTGACC CTGGTAAATG   
  
  
- CATACCATTT TTTGAGGCGT AAAATTGCTC AGTATTGAAA AGAACTAGAG TAGAATTCCT TTGTGGCTGA   
  
  
- AAAAAAGTAC GGACGCAGTA GTATGTACAA CGGGCTCCTG TGTCGGGTAA CTGCTGAGAT CCTGTTAATC   
  
  
- CATGTACTCT TCCGGAAGCT CCTCAATCGC CCTCGTTATG ACCACCTAAG TTGTTCATAA GAAAGTAGAA   
  
  
- CAGTTTCCGT ACGACTGTAA GTAGACAGGT AAGCCACCTT TGTAAGAAGA AGTCGTCGAA CTTATTACCA   
  
  
- AGTGTACACC GTCAATCAAC TCTATCAAGT GCATCATTAT TAACAGTTAC TAATGTTTTT ATACGTCATA   
  
  
- GTGTTCATTT TTAAATTCTA CTTGATTTTA TAGAGAACAA ACTCTTTACA AAGTCGAAAA TCTCAACTAC   
  
  
- AGTGCAGAAA CTCGAACGTA CATACATTA

+     CAT-box

| Site Name | Organism | Position | Strand | Matrix score. | sequence | function |
| --- | --- | --- | --- | --- | --- | --- |
| CAT-box | Arabidopsis thaliana | 418 | + | 6 | GCCACT | cis-acting regulatory element related to meristem expression |

>033955   
+ GAAACTTTTT TACCTCTGAA ATTTACATGT CTTTGTGAAG GGAAAGATAG ATGTGGAAAA TAGAGAAGCA   
  
  
+ AGGGCGCAGT TAAGGTTGTG GAGAGAAGAC AAAGCTGAAA GGGTCAAATG TGTGGAAAGG AGGGAAGAGT   
  
  
+ AATGGAAGGC TCAATTAGTT TGGTGGTTCT TTGTCTAATA ATAGGAGAAC CATATCACTA TTTTGTTTTA   
  
  
+ TTTTGATATC TTATGTATAA TGATGTTTGT AACAAAATAT TTTCTCTTTT GATTTTTATT TTTACAACGG   
  
  
+ ATAGGATAAT TGTCTAAACG ATATTAGGGA GCAAGATTTG AACTCAAGAT GCCGTAAGTT GAAGGAAAGA   
  
  
+ CCTATCCACA AAGCAATACA CCGCTTGCTA ATAAAAACAC GCTTGTTAAA CATATAATAA TTATGTTGCC   
  
  
+ ACTCATCTTA CGATTTTAAC GGTAATATTT TTGTTCACTT TTAAGGGCAA AGACAAAAAC TTTCAATGGG   
  
  
+ ATAGCTAAAC ATGTTATACA TTTAGCATAA GCAACCCTAA TTAAGCACTA GCAACCTTAT ACATGTTATG   
  
  
+ CATTTAAGGC AGGTCTTATT GGCAATGGTG AGCTAGAGGG GGGCACGGGC ACGGTATTGT GCATAATGAA   
  
  
+ GAAATTTTTT TTGTTTTTTT AAGATTAAAA AAAAATTCAT TTATAAATCA AGAGGTTGGG ACTAGTATTT   
  
  
+ AAGACAACCA CAGTTTGACC CTCGATATAA ATTTATGTAA ATATATTTAT TTAATACAAT TATCATTTAA   
  
  
+ ACGTCGAGAT AGCGATTGTA TCAAATACAT TTAAGTATTG TAATCAAATG ACAAAAAGTT CACCGTGAAC   
  
  
+ AATAGGAACC AAATTAAACA TAAATACTAA GACGTCTTAT ACAATGGTAG TACATGGCAA TTCCAAATAA   
  
  
+ ATTGTCTTCC ACCCCCAGAA AAAAAATTAT GCTTTTAAAA AAAGATTTTG AGAAATTTCA TTCTTATACA   
  
  
+ GTTAAATTTA TCTAAAGGAA TAACTTCTTT AAAGTCATAA AACATTCCGG TCACAACTGG GACCATTTAC   
  
  
+ GTATGGTAAA AAACTCCGCA TTTTAACGAG TCATAACTTT TCTTGATCTC ATCTTAAGGA AACACCGACT   
  
  
+ TTTTTTCATG CCTGCGTCAT CATACATGTT GCCCGAGGAC ACAGCCCATT GACGACTCTA GGACAATTAG   
  
  
+ GTACATGAGA AGGCCTTCGA GGAGTTAGCG GGAGCAATAC TGGTGGATTC AACAAGTATT CTTTCATCTT   
  
  
+ GTCAAAGGCA TGCTGACATT CATCTGTCCA TTCGGTGGAA ACATTCTTCT TCAGCAGCTT GAATAATGGT   
  
  
+ TCACATGTGG CAGTTAGTTG AGATAGTTCA CGTAGTAATA ATTGTCAATG ATTACAAAAA TATGCAGTAT   
  
  
+ CACAAGTAAA AATTTAAGAT GAACTAAAAT ATCTCTTGTT TGAGAAATGT TTCAGCTTTT AGAGTTGATG   
  
  
+ TCACGTCTTT GAGCTTGCAT GTATGTAAT  

- CTTTGAAAAA ATGGAGACTT TAAATGTACA GAAACACTTC CCTTTCTATC TACACCTTTT ATCTCTTCGT   
  
  
- TCCCGCGTCA ATTCCAACAC CTCTCTTCTG TTTCGACTTT CCCAGTTTAC ACACCTTTCC TCCCTTCTCA   
  
  
- TTACCTTCCG AGTTAATCAA ACCACCAAGA AACAGATTAT TATCCTCTTG GTATAGTGAT AAAACAAAAT   
  
  
- AAAACTATAG AATACATATT ACTACAAACA TTGTTTTATA AAAGAGAAAA CTAAAAATAA AAATGTTGCC   
  
  
- TATCCTATTA ACAGATTTGC TATAATCCCT CGTTCTAAAC TTGAGTTCTA CGGCATTCAA CTTCCTTTCT   
  
  
- GGATAGGTGT TTCGTTATGT GGCGAACGAT TATTTTTGTG CGAACAATTT GTATATTATT AATACAACGG   
  
  
- TGAGTAGAAT GCTAAAATTG CCATTATAAA AACAAGTGAA AATTCCCGTT TCTGTTTTTG AAAGTTACCC   
  
  
- TATCGATTTG TACAATATGT AAATCGTATT CGTTGGGATT AATTCGTGAT CGTTGGAATA TGTACAATAC   
  
  
- GTAAATTCCG TCCAGAATAA CCGTTACCAC TCGATCTCCC CCCGTGCCCG TGCCATAACA CGTATTACTT   
  
  
- CTTTAAAAAA AACAAAAAAA TTCTAATTTT TTTTTAAGTA AATATTTAGT TCTCCAACCC TGATCATAAA   
  
  
- TTCTGTTGGT GTCAAACTGG GAGCTATATT TAAATACATT TATATAAATA AATTATGTTA ATAGTAAATT   
  
  
- TGCAGCTCTA TCGCTAACAT AGTTTATGTA AATTCATAAC ATTAGTTTAC TGTTTTTCAA GTGGCACTTG   
  
  
- TTATCCTTGG TTTAATTTGT ATTTATGATT CTGCAGAATA TGTTACCATC ATGTACCGTT AAGGTTTATT   
  
  
- TAACAGAAGG TGGGGGTCTT TTTTTTAATA CGAAAATTTT TTTCTAAAAC TCTTTAAAGT AAGAATATGT   
  
  
- CAATTTAAAT AGATTTCCTT ATTGAAGAAA TTTCAGTATT TTGTAAGGCC AGTGTTGACC CTGGTAAATG   
  
  
- CATACCATTT TTTGAGGCGT AAAATTGCTC AGTATTGAAA AGAACTAGAG TAGAATTCCT TTGTGGCTGA   
  
  
- AAAAAAGTAC GGACGCAGTA GTATGTACAA CGGGCTCCTG TGTCGGGTAA CTGCTGAGAT CCTGTTAATC   
  
  
- CATGTACTCT TCCGGAAGCT CCTCAATCGC CCTCGTTATG ACCACCTAAG TTGTTCATAA GAAAGTAGAA   
  
  
- CAGTTTCCGT ACGACTGTAA GTAGACAGGT AAGCCACCTT TGTAAGAAGA AGTCGTCGAA CTTATTACCA   
  
  
- AGTGTACACC GTCAATCAAC TCTATCAAGT GCATCATTAT TAACAGTTAC TAATGTTTTT ATACGTCATA   
  
  
- GTGTTCATTT TTAAATTCTA CTTGATTTTA TAGAGAACAA ACTCTTTACA AAGTCGAAAA TCTCAACTAC   
  
  
- AGTGCAGAAA CTCGAACGTA CATACATTA

+     CCAAT-box

| Site Name | Organism | Position | Strand | Matrix score. | sequence | function |
| --- | --- | --- | --- | --- | --- | --- |
| CCAAT-box | Hordeum vulgare | 275 | + | 6 | CAACGG | MYBHv1 binding site |

>033955   
+ GAAACTTTTT TACCTCTGAA ATTTACATGT CTTTGTGAAG GGAAAGATAG ATGTGGAAAA TAGAGAAGCA   
  
  
+ AGGGCGCAGT TAAGGTTGTG GAGAGAAGAC AAAGCTGAAA GGGTCAAATG TGTGGAAAGG AGGGAAGAGT   
  
  
+ AATGGAAGGC TCAATTAGTT TGGTGGTTCT TTGTCTAATA ATAGGAGAAC CATATCACTA TTTTGTTTTA   
  
  
+ TTTTGATATC TTATGTATAA TGATGTTTGT AACAAAATAT TTTCTCTTTT GATTTTTATT TTTACAACGG   
  
  
+ ATAGGATAAT TGTCTAAACG ATATTAGGGA GCAAGATTTG AACTCAAGAT GCCGTAAGTT GAAGGAAAGA   
  
  
+ CCTATCCACA AAGCAATACA CCGCTTGCTA ATAAAAACAC GCTTGTTAAA CATATAATAA TTATGTTGCC   
  
  
+ ACTCATCTTA CGATTTTAAC GGTAATATTT TTGTTCACTT TTAAGGGCAA AGACAAAAAC TTTCAATGGG   
  
  
+ ATAGCTAAAC ATGTTATACA TTTAGCATAA GCAACCCTAA TTAAGCACTA GCAACCTTAT ACATGTTATG   
  
  
+ CATTTAAGGC AGGTCTTATT GGCAATGGTG AGCTAGAGGG GGGCACGGGC ACGGTATTGT GCATAATGAA   
  
  
+ GAAATTTTTT TTGTTTTTTT AAGATTAAAA AAAAATTCAT TTATAAATCA AGAGGTTGGG ACTAGTATTT   
  
  
+ AAGACAACCA CAGTTTGACC CTCGATATAA ATTTATGTAA ATATATTTAT TTAATACAAT TATCATTTAA   
  
  
+ ACGTCGAGAT AGCGATTGTA TCAAATACAT TTAAGTATTG TAATCAAATG ACAAAAAGTT CACCGTGAAC   
  
  
+ AATAGGAACC AAATTAAACA TAAATACTAA GACGTCTTAT ACAATGGTAG TACATGGCAA TTCCAAATAA   
  
  
+ ATTGTCTTCC ACCCCCAGAA AAAAAATTAT GCTTTTAAAA AAAGATTTTG AGAAATTTCA TTCTTATACA   
  
  
+ GTTAAATTTA TCTAAAGGAA TAACTTCTTT AAAGTCATAA AACATTCCGG TCACAACTGG GACCATTTAC   
  
  
+ GTATGGTAAA AAACTCCGCA TTTTAACGAG TCATAACTTT TCTTGATCTC ATCTTAAGGA AACACCGACT   
  
  
+ TTTTTTCATG CCTGCGTCAT CATACATGTT GCCCGAGGAC ACAGCCCATT GACGACTCTA GGACAATTAG   
  
  
+ GTACATGAGA AGGCCTTCGA GGAGTTAGCG GGAGCAATAC TGGTGGATTC AACAAGTATT CTTTCATCTT   
  
  
+ GTCAAAGGCA TGCTGACATT CATCTGTCCA TTCGGTGGAA ACATTCTTCT TCAGCAGCTT GAATAATGGT   
  
  
+ TCACATGTGG CAGTTAGTTG AGATAGTTCA CGTAGTAATA ATTGTCAATG ATTACAAAAA TATGCAGTAT   
  
  
+ CACAAGTAAA AATTTAAGAT GAACTAAAAT ATCTCTTGTT TGAGAAATGT TTCAGCTTTT AGAGTTGATG   
  
  
+ TCACGTCTTT GAGCTTGCAT GTATGTAAT  

- CTTTGAAAAA ATGGAGACTT TAAATGTACA GAAACACTTC CCTTTCTATC TACACCTTTT ATCTCTTCGT   
  
  
- TCCCGCGTCA ATTCCAACAC CTCTCTTCTG TTTCGACTTT CCCAGTTTAC ACACCTTTCC TCCCTTCTCA   
  
  
- TTACCTTCCG AGTTAATCAA ACCACCAAGA AACAGATTAT TATCCTCTTG GTATAGTGAT AAAACAAAAT   
  
  
- AAAACTATAG AATACATATT ACTACAAACA TTGTTTTATA AAAGAGAAAA CTAAAAATAA AAATGTTGCC   
  
  
- TATCCTATTA ACAGATTTGC TATAATCCCT CGTTCTAAAC TTGAGTTCTA CGGCATTCAA CTTCCTTTCT   
  
  
- GGATAGGTGT TTCGTTATGT GGCGAACGAT TATTTTTGTG CGAACAATTT GTATATTATT AATACAACGG   
  
  
- TGAGTAGAAT GCTAAAATTG CCATTATAAA AACAAGTGAA AATTCCCGTT TCTGTTTTTG AAAGTTACCC   
  
  
- TATCGATTTG TACAATATGT AAATCGTATT CGTTGGGATT AATTCGTGAT CGTTGGAATA TGTACAATAC   
  
  
- GTAAATTCCG TCCAGAATAA CCGTTACCAC TCGATCTCCC CCCGTGCCCG TGCCATAACA CGTATTACTT   
  
  
- CTTTAAAAAA AACAAAAAAA TTCTAATTTT TTTTTAAGTA AATATTTAGT TCTCCAACCC TGATCATAAA   
  
  
- TTCTGTTGGT GTCAAACTGG GAGCTATATT TAAATACATT TATATAAATA AATTATGTTA ATAGTAAATT   
  
  
- TGCAGCTCTA TCGCTAACAT AGTTTATGTA AATTCATAAC ATTAGTTTAC TGTTTTTCAA GTGGCACTTG   
  
  
- TTATCCTTGG TTTAATTTGT ATTTATGATT CTGCAGAATA TGTTACCATC ATGTACCGTT AAGGTTTATT   
  
  
- TAACAGAAGG TGGGGGTCTT TTTTTTAATA CGAAAATTTT TTTCTAAAAC TCTTTAAAGT AAGAATATGT   
  
  
- CAATTTAAAT AGATTTCCTT ATTGAAGAAA TTTCAGTATT TTGTAAGGCC AGTGTTGACC CTGGTAAATG   
  
  
- CATACCATTT TTTGAGGCGT AAAATTGCTC AGTATTGAAA AGAACTAGAG TAGAATTCCT TTGTGGCTGA   
  
  
- AAAAAAGTAC GGACGCAGTA GTATGTACAA CGGGCTCCTG TGTCGGGTAA CTGCTGAGAT CCTGTTAATC   
  
  
- CATGTACTCT TCCGGAAGCT CCTCAATCGC CCTCGTTATG ACCACCTAAG TTGTTCATAA GAAAGTAGAA   
  
  
- CAGTTTCCGT ACGACTGTAA GTAGACAGGT AAGCCACCTT TGTAAGAAGA AGTCGTCGAA CTTATTACCA   
  
  
- AGTGTACACC GTCAATCAAC TCTATCAAGT GCATCATTAT TAACAGTTAC TAATGTTTTT ATACGTCATA   
  
  
- GTGTTCATTT TTAAATTCTA CTTGATTTTA TAGAGAACAA ACTCTTTACA AAGTCGAAAA TCTCAACTAC   
  
  
- AGTGCAGAAA CTCGAACGTA CATACATTA

+     CGTCA-motif

| Site Name | Organism | Position | Strand | Matrix score. | sequence | function |
| --- | --- | --- | --- | --- | --- | --- |
| CGTCA-motif | Hordeum vulgare | 1170 | - | 5 | CGTCA | cis-acting regulatory element involved in the MeJA-responsiveness |
| CGTCA-motif | Hordeum vulgare | 1135 | + | 5 | CGTCA | cis-acting regulatory element involved in the MeJA-responsiveness |

>033955   
+ GAAACTTTTT TACCTCTGAA ATTTACATGT CTTTGTGAAG GGAAAGATAG ATGTGGAAAA TAGAGAAGCA   
  
  
+ AGGGCGCAGT TAAGGTTGTG GAGAGAAGAC AAAGCTGAAA GGGTCAAATG TGTGGAAAGG AGGGAAGAGT   
  
  
+ AATGGAAGGC TCAATTAGTT TGGTGGTTCT TTGTCTAATA ATAGGAGAAC CATATCACTA TTTTGTTTTA   
  
  
+ TTTTGATATC TTATGTATAA TGATGTTTGT AACAAAATAT TTTCTCTTTT GATTTTTATT TTTACAACGG   
  
  
+ ATAGGATAAT TGTCTAAACG ATATTAGGGA GCAAGATTTG AACTCAAGAT GCCGTAAGTT GAAGGAAAGA   
  
  
+ CCTATCCACA AAGCAATACA CCGCTTGCTA ATAAAAACAC GCTTGTTAAA CATATAATAA TTATGTTGCC   
  
  
+ ACTCATCTTA CGATTTTAAC GGTAATATTT TTGTTCACTT TTAAGGGCAA AGACAAAAAC TTTCAATGGG   
  
  
+ ATAGCTAAAC ATGTTATACA TTTAGCATAA GCAACCCTAA TTAAGCACTA GCAACCTTAT ACATGTTATG   
  
  
+ CATTTAAGGC AGGTCTTATT GGCAATGGTG AGCTAGAGGG GGGCACGGGC ACGGTATTGT GCATAATGAA   
  
  
+ GAAATTTTTT TTGTTTTTTT AAGATTAAAA AAAAATTCAT TTATAAATCA AGAGGTTGGG ACTAGTATTT   
  
  
+ AAGACAACCA CAGTTTGACC CTCGATATAA ATTTATGTAA ATATATTTAT TTAATACAAT TATCATTTAA   
  
  
+ ACGTCGAGAT AGCGATTGTA TCAAATACAT TTAAGTATTG TAATCAAATG ACAAAAAGTT CACCGTGAAC   
  
  
+ AATAGGAACC AAATTAAACA TAAATACTAA GACGTCTTAT ACAATGGTAG TACATGGCAA TTCCAAATAA   
  
  
+ ATTGTCTTCC ACCCCCAGAA AAAAAATTAT GCTTTTAAAA AAAGATTTTG AGAAATTTCA TTCTTATACA   
  
  
+ GTTAAATTTA TCTAAAGGAA TAACTTCTTT AAAGTCATAA AACATTCCGG TCACAACTGG GACCATTTAC   
  
  
+ GTATGGTAAA AAACTCCGCA TTTTAACGAG TCATAACTTT TCTTGATCTC ATCTTAAGGA AACACCGACT   
  
  
+ TTTTTTCATG CCTGCGTCAT CATACATGTT GCCCGAGGAC ACAGCCCATT GACGACTCTA GGACAATTAG   
  
  
+ GTACATGAGA AGGCCTTCGA GGAGTTAGCG GGAGCAATAC TGGTGGATTC AACAAGTATT CTTTCATCTT   
  
  
+ GTCAAAGGCA TGCTGACATT CATCTGTCCA TTCGGTGGAA ACATTCTTCT TCAGCAGCTT GAATAATGGT   
  
  
+ TCACATGTGG CAGTTAGTTG AGATAGTTCA CGTAGTAATA ATTGTCAATG ATTACAAAAA TATGCAGTAT   
  
  
+ CACAAGTAAA AATTTAAGAT GAACTAAAAT ATCTCTTGTT TGAGAAATGT TTCAGCTTTT AGAGTTGATG   
  
  
+ TCACGTCTTT GAGCTTGCAT GTATGTAAT  

- CTTTGAAAAA ATGGAGACTT TAAATGTACA GAAACACTTC CCTTTCTATC TACACCTTTT ATCTCTTCGT   
  
  
- TCCCGCGTCA ATTCCAACAC CTCTCTTCTG TTTCGACTTT CCCAGTTTAC ACACCTTTCC TCCCTTCTCA   
  
  
- TTACCTTCCG AGTTAATCAA ACCACCAAGA AACAGATTAT TATCCTCTTG GTATAGTGAT AAAACAAAAT   
  
  
- AAAACTATAG AATACATATT ACTACAAACA TTGTTTTATA AAAGAGAAAA CTAAAAATAA AAATGTTGCC   
  
  
- TATCCTATTA ACAGATTTGC TATAATCCCT CGTTCTAAAC TTGAGTTCTA CGGCATTCAA CTTCCTTTCT   
  
  
- GGATAGGTGT TTCGTTATGT GGCGAACGAT TATTTTTGTG CGAACAATTT GTATATTATT AATACAACGG   
  
  
- TGAGTAGAAT GCTAAAATTG CCATTATAAA AACAAGTGAA AATTCCCGTT TCTGTTTTTG AAAGTTACCC   
  
  
- TATCGATTTG TACAATATGT AAATCGTATT CGTTGGGATT AATTCGTGAT CGTTGGAATA TGTACAATAC   
  
  
- GTAAATTCCG TCCAGAATAA CCGTTACCAC TCGATCTCCC CCCGTGCCCG TGCCATAACA CGTATTACTT   
  
  
- CTTTAAAAAA AACAAAAAAA TTCTAATTTT TTTTTAAGTA AATATTTAGT TCTCCAACCC TGATCATAAA   
  
  
- TTCTGTTGGT GTCAAACTGG GAGCTATATT TAAATACATT TATATAAATA AATTATGTTA ATAGTAAATT   
  
  
- TGCAGCTCTA TCGCTAACAT AGTTTATGTA AATTCATAAC ATTAGTTTAC TGTTTTTCAA GTGGCACTTG   
  
  
- TTATCCTTGG TTTAATTTGT ATTTATGATT CTGCAGAATA TGTTACCATC ATGTACCGTT AAGGTTTATT   
  
  
- TAACAGAAGG TGGGGGTCTT TTTTTTAATA CGAAAATTTT TTTCTAAAAC TCTTTAAAGT AAGAATATGT   
  
  
- CAATTTAAAT AGATTTCCTT ATTGAAGAAA TTTCAGTATT TTGTAAGGCC AGTGTTGACC CTGGTAAATG   
  
  
- CATACCATTT TTTGAGGCGT AAAATTGCTC AGTATTGAAA AGAACTAGAG TAGAATTCCT TTGTGGCTGA   
  
  
- AAAAAAGTAC GGACGCAGTA GTATGTACAA CGGGCTCCTG TGTCGGGTAA CTGCTGAGAT CCTGTTAATC   
  
  
- CATGTACTCT TCCGGAAGCT CCTCAATCGC CCTCGTTATG ACCACCTAAG TTGTTCATAA GAAAGTAGAA   
  
  
- CAGTTTCCGT ACGACTGTAA GTAGACAGGT AAGCCACCTT TGTAAGAAGA AGTCGTCGAA CTTATTACCA   
  
  
- AGTGTACACC GTCAATCAAC TCTATCAAGT GCATCATTAT TAACAGTTAC TAATGTTTTT ATACGTCATA   
  
  
- GTGTTCATTT TTAAATTCTA CTTGATTTTA TAGAGAACAA ACTCTTTACA AAGTCGAAAA TCTCAACTAC   
  
  
- AGTGCAGAAA CTCGAACGTA CATACATTA

+     G-box

| Site Name | Organism | Position | Strand | Matrix score. | sequence | function |
| --- | --- | --- | --- | --- | --- | --- |
| G-box | Zea mays | 1472 | + | 6 | CACGTC | cis-acting regulatory element involved in light responsiveness |
| G-box | Arabidopsis thaliana | 1359 | - | 6 | TACGTG | cis-acting regulatory element involved in light responsiveness |

>033955   
+ GAAACTTTTT TACCTCTGAA ATTTACATGT CTTTGTGAAG GGAAAGATAG ATGTGGAAAA TAGAGAAGCA   
  
  
+ AGGGCGCAGT TAAGGTTGTG GAGAGAAGAC AAAGCTGAAA GGGTCAAATG TGTGGAAAGG AGGGAAGAGT   
  
  
+ AATGGAAGGC TCAATTAGTT TGGTGGTTCT TTGTCTAATA ATAGGAGAAC CATATCACTA TTTTGTTTTA   
  
  
+ TTTTGATATC TTATGTATAA TGATGTTTGT AACAAAATAT TTTCTCTTTT GATTTTTATT TTTACAACGG   
  
  
+ ATAGGATAAT TGTCTAAACG ATATTAGGGA GCAAGATTTG AACTCAAGAT GCCGTAAGTT GAAGGAAAGA   
  
  
+ CCTATCCACA AAGCAATACA CCGCTTGCTA ATAAAAACAC GCTTGTTAAA CATATAATAA TTATGTTGCC   
  
  
+ ACTCATCTTA CGATTTTAAC GGTAATATTT TTGTTCACTT TTAAGGGCAA AGACAAAAAC TTTCAATGGG   
  
  
+ ATAGCTAAAC ATGTTATACA TTTAGCATAA GCAACCCTAA TTAAGCACTA GCAACCTTAT ACATGTTATG   
  
  
+ CATTTAAGGC AGGTCTTATT GGCAATGGTG AGCTAGAGGG GGGCACGGGC ACGGTATTGT GCATAATGAA   
  
  
+ GAAATTTTTT TTGTTTTTTT AAGATTAAAA AAAAATTCAT TTATAAATCA AGAGGTTGGG ACTAGTATTT   
  
  
+ AAGACAACCA CAGTTTGACC CTCGATATAA ATTTATGTAA ATATATTTAT TTAATACAAT TATCATTTAA   
  
  
+ ACGTCGAGAT AGCGATTGTA TCAAATACAT TTAAGTATTG TAATCAAATG ACAAAAAGTT CACCGTGAAC   
  
  
+ AATAGGAACC AAATTAAACA TAAATACTAA GACGTCTTAT ACAATGGTAG TACATGGCAA TTCCAAATAA   
  
  
+ ATTGTCTTCC ACCCCCAGAA AAAAAATTAT GCTTTTAAAA AAAGATTTTG AGAAATTTCA TTCTTATACA   
  
  
+ GTTAAATTTA TCTAAAGGAA TAACTTCTTT AAAGTCATAA AACATTCCGG TCACAACTGG GACCATTTAC   
  
  
+ GTATGGTAAA AAACTCCGCA TTTTAACGAG TCATAACTTT TCTTGATCTC ATCTTAAGGA AACACCGACT   
  
  
+ TTTTTTCATG CCTGCGTCAT CATACATGTT GCCCGAGGAC ACAGCCCATT GACGACTCTA GGACAATTAG   
  
  
+ GTACATGAGA AGGCCTTCGA GGAGTTAGCG GGAGCAATAC TGGTGGATTC AACAAGTATT CTTTCATCTT   
  
  
+ GTCAAAGGCA TGCTGACATT CATCTGTCCA TTCGGTGGAA ACATTCTTCT TCAGCAGCTT GAATAATGGT   
  
  
+ TCACATGTGG CAGTTAGTTG AGATAGTTCA CGTAGTAATA ATTGTCAATG ATTACAAAAA TATGCAGTAT   
  
  
+ CACAAGTAAA AATTTAAGAT GAACTAAAAT ATCTCTTGTT TGAGAAATGT TTCAGCTTTT AGAGTTGATG   
  
  
+ TCACGTCTTT GAGCTTGCAT GTATGTAAT  

- CTTTGAAAAA ATGGAGACTT TAAATGTACA GAAACACTTC CCTTTCTATC TACACCTTTT ATCTCTTCGT   
  
  
- TCCCGCGTCA ATTCCAACAC CTCTCTTCTG TTTCGACTTT CCCAGTTTAC ACACCTTTCC TCCCTTCTCA   
  
  
- TTACCTTCCG AGTTAATCAA ACCACCAAGA AACAGATTAT TATCCTCTTG GTATAGTGAT AAAACAAAAT   
  
  
- AAAACTATAG AATACATATT ACTACAAACA TTGTTTTATA AAAGAGAAAA CTAAAAATAA AAATGTTGCC   
  
  
- TATCCTATTA ACAGATTTGC TATAATCCCT CGTTCTAAAC TTGAGTTCTA CGGCATTCAA CTTCCTTTCT   
  
  
- GGATAGGTGT TTCGTTATGT GGCGAACGAT TATTTTTGTG CGAACAATTT GTATATTATT AATACAACGG   
  
  
- TGAGTAGAAT GCTAAAATTG CCATTATAAA AACAAGTGAA AATTCCCGTT TCTGTTTTTG AAAGTTACCC   
  
  
- TATCGATTTG TACAATATGT AAATCGTATT CGTTGGGATT AATTCGTGAT CGTTGGAATA TGTACAATAC   
  
  
- GTAAATTCCG TCCAGAATAA CCGTTACCAC TCGATCTCCC CCCGTGCCCG TGCCATAACA CGTATTACTT   
  
  
- CTTTAAAAAA AACAAAAAAA TTCTAATTTT TTTTTAAGTA AATATTTAGT TCTCCAACCC TGATCATAAA   
  
  
- TTCTGTTGGT GTCAAACTGG GAGCTATATT TAAATACATT TATATAAATA AATTATGTTA ATAGTAAATT   
  
  
- TGCAGCTCTA TCGCTAACAT AGTTTATGTA AATTCATAAC ATTAGTTTAC TGTTTTTCAA GTGGCACTTG   
  
  
- TTATCCTTGG TTTAATTTGT ATTTATGATT CTGCAGAATA TGTTACCATC ATGTACCGTT AAGGTTTATT   
  
  
- TAACAGAAGG TGGGGGTCTT TTTTTTAATA CGAAAATTTT TTTCTAAAAC TCTTTAAAGT AAGAATATGT   
  
  
- CAATTTAAAT AGATTTCCTT ATTGAAGAAA TTTCAGTATT TTGTAAGGCC AGTGTTGACC CTGGTAAATG   
  
  
- CATACCATTT TTTGAGGCGT AAAATTGCTC AGTATTGAAA AGAACTAGAG TAGAATTCCT TTGTGGCTGA   
  
  
- AAAAAAGTAC GGACGCAGTA GTATGTACAA CGGGCTCCTG TGTCGGGTAA CTGCTGAGAT CCTGTTAATC   
  
  
- CATGTACTCT TCCGGAAGCT CCTCAATCGC CCTCGTTATG ACCACCTAAG TTGTTCATAA GAAAGTAGAA   
  
  
- CAGTTTCCGT ACGACTGTAA GTAGACAGGT AAGCCACCTT TGTAAGAAGA AGTCGTCGAA CTTATTACCA   
  
  
- AGTGTACACC GTCAATCAAC TCTATCAAGT GCATCATTAT TAACAGTTAC TAATGTTTTT ATACGTCATA   
  
  
- GTGTTCATTT TTAAATTCTA CTTGATTTTA TAGAGAACAA ACTCTTTACA AAGTCGAAAA TCTCAACTAC   
  
  
- AGTGCAGAAA CTCGAACGTA CATACATTA

+     GATA-motif

| Site Name | Organism | Position | Strand | Matrix score. | sequence | function |
| --- | --- | --- | --- | --- | --- | --- |
| GATA-motif | Arabidopsis thaliana | 280 | + | 7 | GATAGGA | part of a light responsive element |

>033955   
+ GAAACTTTTT TACCTCTGAA ATTTACATGT CTTTGTGAAG GGAAAGATAG ATGTGGAAAA TAGAGAAGCA   
  
  
+ AGGGCGCAGT TAAGGTTGTG GAGAGAAGAC AAAGCTGAAA GGGTCAAATG TGTGGAAAGG AGGGAAGAGT   
  
  
+ AATGGAAGGC TCAATTAGTT TGGTGGTTCT TTGTCTAATA ATAGGAGAAC CATATCACTA TTTTGTTTTA   
  
  
+ TTTTGATATC TTATGTATAA TGATGTTTGT AACAAAATAT TTTCTCTTTT GATTTTTATT TTTACAACGG   
  
  
+ ATAGGATAAT TGTCTAAACG ATATTAGGGA GCAAGATTTG AACTCAAGAT GCCGTAAGTT GAAGGAAAGA   
  
  
+ CCTATCCACA AAGCAATACA CCGCTTGCTA ATAAAAACAC GCTTGTTAAA CATATAATAA TTATGTTGCC   
  
  
+ ACTCATCTTA CGATTTTAAC GGTAATATTT TTGTTCACTT TTAAGGGCAA AGACAAAAAC TTTCAATGGG   
  
  
+ ATAGCTAAAC ATGTTATACA TTTAGCATAA GCAACCCTAA TTAAGCACTA GCAACCTTAT ACATGTTATG   
  
  
+ CATTTAAGGC AGGTCTTATT GGCAATGGTG AGCTAGAGGG GGGCACGGGC ACGGTATTGT GCATAATGAA   
  
  
+ GAAATTTTTT TTGTTTTTTT AAGATTAAAA AAAAATTCAT TTATAAATCA AGAGGTTGGG ACTAGTATTT   
  
  
+ AAGACAACCA CAGTTTGACC CTCGATATAA ATTTATGTAA ATATATTTAT TTAATACAAT TATCATTTAA   
  
  
+ ACGTCGAGAT AGCGATTGTA TCAAATACAT TTAAGTATTG TAATCAAATG ACAAAAAGTT CACCGTGAAC   
  
  
+ AATAGGAACC AAATTAAACA TAAATACTAA GACGTCTTAT ACAATGGTAG TACATGGCAA TTCCAAATAA   
  
  
+ ATTGTCTTCC ACCCCCAGAA AAAAAATTAT GCTTTTAAAA AAAGATTTTG AGAAATTTCA TTCTTATACA   
  
  
+ GTTAAATTTA TCTAAAGGAA TAACTTCTTT AAAGTCATAA AACATTCCGG TCACAACTGG GACCATTTAC   
  
  
+ GTATGGTAAA AAACTCCGCA TTTTAACGAG TCATAACTTT TCTTGATCTC ATCTTAAGGA AACACCGACT   
  
  
+ TTTTTTCATG CCTGCGTCAT CATACATGTT GCCCGAGGAC ACAGCCCATT GACGACTCTA GGACAATTAG   
  
  
+ GTACATGAGA AGGCCTTCGA GGAGTTAGCG GGAGCAATAC TGGTGGATTC AACAAGTATT CTTTCATCTT   
  
  
+ GTCAAAGGCA TGCTGACATT CATCTGTCCA TTCGGTGGAA ACATTCTTCT TCAGCAGCTT GAATAATGGT   
  
  
+ TCACATGTGG CAGTTAGTTG AGATAGTTCA CGTAGTAATA ATTGTCAATG ATTACAAAAA TATGCAGTAT   
  
  
+ CACAAGTAAA AATTTAAGAT GAACTAAAAT ATCTCTTGTT TGAGAAATGT TTCAGCTTTT AGAGTTGATG   
  
  
+ TCACGTCTTT GAGCTTGCAT GTATGTAAT  

- CTTTGAAAAA ATGGAGACTT TAAATGTACA GAAACACTTC CCTTTCTATC TACACCTTTT ATCTCTTCGT   
  
  
- TCCCGCGTCA ATTCCAACAC CTCTCTTCTG TTTCGACTTT CCCAGTTTAC ACACCTTTCC TCCCTTCTCA   
  
  
- TTACCTTCCG AGTTAATCAA ACCACCAAGA AACAGATTAT TATCCTCTTG GTATAGTGAT AAAACAAAAT   
  
  
- AAAACTATAG AATACATATT ACTACAAACA TTGTTTTATA AAAGAGAAAA CTAAAAATAA AAATGTTGCC   
  
  
- TATCCTATTA ACAGATTTGC TATAATCCCT CGTTCTAAAC TTGAGTTCTA CGGCATTCAA CTTCCTTTCT   
  
  
- GGATAGGTGT TTCGTTATGT GGCGAACGAT TATTTTTGTG CGAACAATTT GTATATTATT AATACAACGG   
  
  
- TGAGTAGAAT GCTAAAATTG CCATTATAAA AACAAGTGAA AATTCCCGTT TCTGTTTTTG AAAGTTACCC   
  
  
- TATCGATTTG TACAATATGT AAATCGTATT CGTTGGGATT AATTCGTGAT CGTTGGAATA TGTACAATAC   
  
  
- GTAAATTCCG TCCAGAATAA CCGTTACCAC TCGATCTCCC CCCGTGCCCG TGCCATAACA CGTATTACTT   
  
  
- CTTTAAAAAA AACAAAAAAA TTCTAATTTT TTTTTAAGTA AATATTTAGT TCTCCAACCC TGATCATAAA   
  
  
- TTCTGTTGGT GTCAAACTGG GAGCTATATT TAAATACATT TATATAAATA AATTATGTTA ATAGTAAATT   
  
  
- TGCAGCTCTA TCGCTAACAT AGTTTATGTA AATTCATAAC ATTAGTTTAC TGTTTTTCAA GTGGCACTTG   
  
  
- TTATCCTTGG TTTAATTTGT ATTTATGATT CTGCAGAATA TGTTACCATC ATGTACCGTT AAGGTTTATT   
  
  
- TAACAGAAGG TGGGGGTCTT TTTTTTAATA CGAAAATTTT TTTCTAAAAC TCTTTAAAGT AAGAATATGT   
  
  
- CAATTTAAAT AGATTTCCTT ATTGAAGAAA TTTCAGTATT TTGTAAGGCC AGTGTTGACC CTGGTAAATG   
  
  
- CATACCATTT TTTGAGGCGT AAAATTGCTC AGTATTGAAA AGAACTAGAG TAGAATTCCT TTGTGGCTGA   
  
  
- AAAAAAGTAC GGACGCAGTA GTATGTACAA CGGGCTCCTG TGTCGGGTAA CTGCTGAGAT CCTGTTAATC   
  
  
- CATGTACTCT TCCGGAAGCT CCTCAATCGC CCTCGTTATG ACCACCTAAG TTGTTCATAA GAAAGTAGAA   
  
  
- CAGTTTCCGT ACGACTGTAA GTAGACAGGT AAGCCACCTT TGTAAGAAGA AGTCGTCGAA CTTATTACCA   
  
  
- AGTGTACACC GTCAATCAAC TCTATCAAGT GCATCATTAT TAACAGTTAC TAATGTTTTT ATACGTCATA   
  
  
- GTGTTCATTT TTAAATTCTA CTTGATTTTA TAGAGAACAA ACTCTTTACA AAGTCGAAAA TCTCAACTAC   
  
  
- AGTGCAGAAA CTCGAACGTA CATACATTA

+     MBS

| Site Name | Organism | Position | Strand | Matrix score. | sequence | function |
| --- | --- | --- | --- | --- | --- | --- |
| MBS | Arabidopsis thaliana | 1034 | + | 6 | CAACTG | MYB binding site involved in drought-inducibility |

>033955   
+ GAAACTTTTT TACCTCTGAA ATTTACATGT CTTTGTGAAG GGAAAGATAG ATGTGGAAAA TAGAGAAGCA   
  
  
+ AGGGCGCAGT TAAGGTTGTG GAGAGAAGAC AAAGCTGAAA GGGTCAAATG TGTGGAAAGG AGGGAAGAGT   
  
  
+ AATGGAAGGC TCAATTAGTT TGGTGGTTCT TTGTCTAATA ATAGGAGAAC CATATCACTA TTTTGTTTTA   
  
  
+ TTTTGATATC TTATGTATAA TGATGTTTGT AACAAAATAT TTTCTCTTTT GATTTTTATT TTTACAACGG   
  
  
+ ATAGGATAAT TGTCTAAACG ATATTAGGGA GCAAGATTTG AACTCAAGAT GCCGTAAGTT GAAGGAAAGA   
  
  
+ CCTATCCACA AAGCAATACA CCGCTTGCTA ATAAAAACAC GCTTGTTAAA CATATAATAA TTATGTTGCC   
  
  
+ ACTCATCTTA CGATTTTAAC GGTAATATTT TTGTTCACTT TTAAGGGCAA AGACAAAAAC TTTCAATGGG   
  
  
+ ATAGCTAAAC ATGTTATACA TTTAGCATAA GCAACCCTAA TTAAGCACTA GCAACCTTAT ACATGTTATG   
  
  
+ CATTTAAGGC AGGTCTTATT GGCAATGGTG AGCTAGAGGG GGGCACGGGC ACGGTATTGT GCATAATGAA   
  
  
+ GAAATTTTTT TTGTTTTTTT AAGATTAAAA AAAAATTCAT TTATAAATCA AGAGGTTGGG ACTAGTATTT   
  
  
+ AAGACAACCA CAGTTTGACC CTCGATATAA ATTTATGTAA ATATATTTAT TTAATACAAT TATCATTTAA   
  
  
+ ACGTCGAGAT AGCGATTGTA TCAAATACAT TTAAGTATTG TAATCAAATG ACAAAAAGTT CACCGTGAAC   
  
  
+ AATAGGAACC AAATTAAACA TAAATACTAA GACGTCTTAT ACAATGGTAG TACATGGCAA TTCCAAATAA   
  
  
+ ATTGTCTTCC ACCCCCAGAA AAAAAATTAT GCTTTTAAAA AAAGATTTTG AGAAATTTCA TTCTTATACA   
  
  
+ GTTAAATTTA TCTAAAGGAA TAACTTCTTT AAAGTCATAA AACATTCCGG TCACAACTGG GACCATTTAC   
  
  
+ GTATGGTAAA AAACTCCGCA TTTTAACGAG TCATAACTTT TCTTGATCTC ATCTTAAGGA AACACCGACT   
  
  
+ TTTTTTCATG CCTGCGTCAT CATACATGTT GCCCGAGGAC ACAGCCCATT GACGACTCTA GGACAATTAG   
  
  
+ GTACATGAGA AGGCCTTCGA GGAGTTAGCG GGAGCAATAC TGGTGGATTC AACAAGTATT CTTTCATCTT   
  
  
+ GTCAAAGGCA TGCTGACATT CATCTGTCCA TTCGGTGGAA ACATTCTTCT TCAGCAGCTT GAATAATGGT   
  
  
+ TCACATGTGG CAGTTAGTTG AGATAGTTCA CGTAGTAATA ATTGTCAATG ATTACAAAAA TATGCAGTAT   
  
  
+ CACAAGTAAA AATTTAAGAT GAACTAAAAT ATCTCTTGTT TGAGAAATGT TTCAGCTTTT AGAGTTGATG   
  
  
+ TCACGTCTTT GAGCTTGCAT GTATGTAAT  

- CTTTGAAAAA ATGGAGACTT TAAATGTACA GAAACACTTC CCTTTCTATC TACACCTTTT ATCTCTTCGT   
  
  
- TCCCGCGTCA ATTCCAACAC CTCTCTTCTG TTTCGACTTT CCCAGTTTAC ACACCTTTCC TCCCTTCTCA   
  
  
- TTACCTTCCG AGTTAATCAA ACCACCAAGA AACAGATTAT TATCCTCTTG GTATAGTGAT AAAACAAAAT   
  
  
- AAAACTATAG AATACATATT ACTACAAACA TTGTTTTATA AAAGAGAAAA CTAAAAATAA AAATGTTGCC   
  
  
- TATCCTATTA ACAGATTTGC TATAATCCCT CGTTCTAAAC TTGAGTTCTA CGGCATTCAA CTTCCTTTCT   
  
  
- GGATAGGTGT TTCGTTATGT GGCGAACGAT TATTTTTGTG CGAACAATTT GTATATTATT AATACAACGG   
  
  
- TGAGTAGAAT GCTAAAATTG CCATTATAAA AACAAGTGAA AATTCCCGTT TCTGTTTTTG AAAGTTACCC   
  
  
- TATCGATTTG TACAATATGT AAATCGTATT CGTTGGGATT AATTCGTGAT CGTTGGAATA TGTACAATAC   
  
  
- GTAAATTCCG TCCAGAATAA CCGTTACCAC TCGATCTCCC CCCGTGCCCG TGCCATAACA CGTATTACTT   
  
  
- CTTTAAAAAA AACAAAAAAA TTCTAATTTT TTTTTAAGTA AATATTTAGT TCTCCAACCC TGATCATAAA   
  
  
- TTCTGTTGGT GTCAAACTGG GAGCTATATT TAAATACATT TATATAAATA AATTATGTTA ATAGTAAATT   
  
  
- TGCAGCTCTA TCGCTAACAT AGTTTATGTA AATTCATAAC ATTAGTTTAC TGTTTTTCAA GTGGCACTTG   
  
  
- TTATCCTTGG TTTAATTTGT ATTTATGATT CTGCAGAATA TGTTACCATC ATGTACCGTT AAGGTTTATT   
  
  
- TAACAGAAGG TGGGGGTCTT TTTTTTAATA CGAAAATTTT TTTCTAAAAC TCTTTAAAGT AAGAATATGT   
  
  
- CAATTTAAAT AGATTTCCTT ATTGAAGAAA TTTCAGTATT TTGTAAGGCC AGTGTTGACC CTGGTAAATG   
  
  
- CATACCATTT TTTGAGGCGT AAAATTGCTC AGTATTGAAA AGAACTAGAG TAGAATTCCT TTGTGGCTGA   
  
  
- AAAAAAGTAC GGACGCAGTA GTATGTACAA CGGGCTCCTG TGTCGGGTAA CTGCTGAGAT CCTGTTAATC   
  
  
- CATGTACTCT TCCGGAAGCT CCTCAATCGC CCTCGTTATG ACCACCTAAG TTGTTCATAA GAAAGTAGAA   
  
  
- CAGTTTCCGT ACGACTGTAA GTAGACAGGT AAGCCACCTT TGTAAGAAGA AGTCGTCGAA CTTATTACCA   
  
  
- AGTGTACACC GTCAATCAAC TCTATCAAGT GCATCATTAT TAACAGTTAC TAATGTTTTT ATACGTCATA   
  
  
- GTGTTCATTT TTAAATTCTA CTTGATTTTA TAGAGAACAA ACTCTTTACA AAGTCGAAAA TCTCAACTAC   
  
  
- AGTGCAGAAA CTCGAACGTA CATACATTA

+     MYB

| Site Name | Organism | Position | Strand | Matrix score. | sequence | function |
| --- | --- | --- | --- | --- | --- | --- |
| MYB | Arabidopsis thaliana | 705 | + | 6 | CAACCA |  |

>033955   
+ GAAACTTTTT TACCTCTGAA ATTTACATGT CTTTGTGAAG GGAAAGATAG ATGTGGAAAA TAGAGAAGCA   
  
  
+ AGGGCGCAGT TAAGGTTGTG GAGAGAAGAC AAAGCTGAAA GGGTCAAATG TGTGGAAAGG AGGGAAGAGT   
  
  
+ AATGGAAGGC TCAATTAGTT TGGTGGTTCT TTGTCTAATA ATAGGAGAAC CATATCACTA TTTTGTTTTA   
  
  
+ TTTTGATATC TTATGTATAA TGATGTTTGT AACAAAATAT TTTCTCTTTT GATTTTTATT TTTACAACGG   
  
  
+ ATAGGATAAT TGTCTAAACG ATATTAGGGA GCAAGATTTG AACTCAAGAT GCCGTAAGTT GAAGGAAAGA   
  
  
+ CCTATCCACA AAGCAATACA CCGCTTGCTA ATAAAAACAC GCTTGTTAAA CATATAATAA TTATGTTGCC   
  
  
+ ACTCATCTTA CGATTTTAAC GGTAATATTT TTGTTCACTT TTAAGGGCAA AGACAAAAAC TTTCAATGGG   
  
  
+ ATAGCTAAAC ATGTTATACA TTTAGCATAA GCAACCCTAA TTAAGCACTA GCAACCTTAT ACATGTTATG   
  
  
+ CATTTAAGGC AGGTCTTATT GGCAATGGTG AGCTAGAGGG GGGCACGGGC ACGGTATTGT GCATAATGAA   
  
  
+ GAAATTTTTT TTGTTTTTTT AAGATTAAAA AAAAATTCAT TTATAAATCA AGAGGTTGGG ACTAGTATTT   
  
  
+ AAGACAACCA CAGTTTGACC CTCGATATAA ATTTATGTAA ATATATTTAT TTAATACAAT TATCATTTAA   
  
  
+ ACGTCGAGAT AGCGATTGTA TCAAATACAT TTAAGTATTG TAATCAAATG ACAAAAAGTT CACCGTGAAC   
  
  
+ AATAGGAACC AAATTAAACA TAAATACTAA GACGTCTTAT ACAATGGTAG TACATGGCAA TTCCAAATAA   
  
  
+ ATTGTCTTCC ACCCCCAGAA AAAAAATTAT GCTTTTAAAA AAAGATTTTG AGAAATTTCA TTCTTATACA   
  
  
+ GTTAAATTTA TCTAAAGGAA TAACTTCTTT AAAGTCATAA AACATTCCGG TCACAACTGG GACCATTTAC   
  
  
+ GTATGGTAAA AAACTCCGCA TTTTAACGAG TCATAACTTT TCTTGATCTC ATCTTAAGGA AACACCGACT   
  
  
+ TTTTTTCATG CCTGCGTCAT CATACATGTT GCCCGAGGAC ACAGCCCATT GACGACTCTA GGACAATTAG   
  
  
+ GTACATGAGA AGGCCTTCGA GGAGTTAGCG GGAGCAATAC TGGTGGATTC AACAAGTATT CTTTCATCTT   
  
  
+ GTCAAAGGCA TGCTGACATT CATCTGTCCA TTCGGTGGAA ACATTCTTCT TCAGCAGCTT GAATAATGGT   
  
  
+ TCACATGTGG CAGTTAGTTG AGATAGTTCA CGTAGTAATA ATTGTCAATG ATTACAAAAA TATGCAGTAT   
  
  
+ CACAAGTAAA AATTTAAGAT GAACTAAAAT ATCTCTTGTT TGAGAAATGT TTCAGCTTTT AGAGTTGATG   
  
  
+ TCACGTCTTT GAGCTTGCAT GTATGTAAT  

- CTTTGAAAAA ATGGAGACTT TAAATGTACA GAAACACTTC CCTTTCTATC TACACCTTTT ATCTCTTCGT   
  
  
- TCCCGCGTCA ATTCCAACAC CTCTCTTCTG TTTCGACTTT CCCAGTTTAC ACACCTTTCC TCCCTTCTCA   
  
  
- TTACCTTCCG AGTTAATCAA ACCACCAAGA AACAGATTAT TATCCTCTTG GTATAGTGAT AAAACAAAAT   
  
  
- AAAACTATAG AATACATATT ACTACAAACA TTGTTTTATA AAAGAGAAAA CTAAAAATAA AAATGTTGCC   
  
  
- TATCCTATTA ACAGATTTGC TATAATCCCT CGTTCTAAAC TTGAGTTCTA CGGCATTCAA CTTCCTTTCT   
  
  
- GGATAGGTGT TTCGTTATGT GGCGAACGAT TATTTTTGTG CGAACAATTT GTATATTATT AATACAACGG   
  
  
- TGAGTAGAAT GCTAAAATTG CCATTATAAA AACAAGTGAA AATTCCCGTT TCTGTTTTTG AAAGTTACCC   
  
  
- TATCGATTTG TACAATATGT AAATCGTATT CGTTGGGATT AATTCGTGAT CGTTGGAATA TGTACAATAC   
  
  
- GTAAATTCCG TCCAGAATAA CCGTTACCAC TCGATCTCCC CCCGTGCCCG TGCCATAACA CGTATTACTT   
  
  
- CTTTAAAAAA AACAAAAAAA TTCTAATTTT TTTTTAAGTA AATATTTAGT TCTCCAACCC TGATCATAAA   
  
  
- TTCTGTTGGT GTCAAACTGG GAGCTATATT TAAATACATT TATATAAATA AATTATGTTA ATAGTAAATT   
  
  
- TGCAGCTCTA TCGCTAACAT AGTTTATGTA AATTCATAAC ATTAGTTTAC TGTTTTTCAA GTGGCACTTG   
  
  
- TTATCCTTGG TTTAATTTGT ATTTATGATT CTGCAGAATA TGTTACCATC ATGTACCGTT AAGGTTTATT   
  
  
- TAACAGAAGG TGGGGGTCTT TTTTTTAATA CGAAAATTTT TTTCTAAAAC TCTTTAAAGT AAGAATATGT   
  
  
- CAATTTAAAT AGATTTCCTT ATTGAAGAAA TTTCAGTATT TTGTAAGGCC AGTGTTGACC CTGGTAAATG   
  
  
- CATACCATTT TTTGAGGCGT AAAATTGCTC AGTATTGAAA AGAACTAGAG TAGAATTCCT TTGTGGCTGA   
  
  
- AAAAAAGTAC GGACGCAGTA GTATGTACAA CGGGCTCCTG TGTCGGGTAA CTGCTGAGAT CCTGTTAATC   
  
  
- CATGTACTCT TCCGGAAGCT CCTCAATCGC CCTCGTTATG ACCACCTAAG TTGTTCATAA GAAAGTAGAA   
  
  
- CAGTTTCCGT ACGACTGTAA GTAGACAGGT AAGCCACCTT TGTAAGAAGA AGTCGTCGAA CTTATTACCA   
  
  
- AGTGTACACC GTCAATCAAC TCTATCAAGT GCATCATTAT TAACAGTTAC TAATGTTTTT ATACGTCATA   
  
  
- GTGTTCATTT TTAAATTCTA CTTGATTTTA TAGAGAACAA ACTCTTTACA AAGTCGAAAA TCTCAACTAC   
  
  
- AGTGCAGAAA CTCGAACGTA CATACATTA

+     MYB recognition site

| Site Name | Organism | Position | Strand | Matrix score. | sequence | function |
| --- | --- | --- | --- | --- | --- | --- |
| MYB recognition site | Arabidopsis thaliana | 275 | - | 6 | CCGTTG |  |

>033955   
+ GAAACTTTTT TACCTCTGAA ATTTACATGT CTTTGTGAAG GGAAAGATAG ATGTGGAAAA TAGAGAAGCA   
  
  
+ AGGGCGCAGT TAAGGTTGTG GAGAGAAGAC AAAGCTGAAA GGGTCAAATG TGTGGAAAGG AGGGAAGAGT   
  
  
+ AATGGAAGGC TCAATTAGTT TGGTGGTTCT TTGTCTAATA ATAGGAGAAC CATATCACTA TTTTGTTTTA   
  
  
+ TTTTGATATC TTATGTATAA TGATGTTTGT AACAAAATAT TTTCTCTTTT GATTTTTATT TTTACAACGG   
  
  
+ ATAGGATAAT TGTCTAAACG ATATTAGGGA GCAAGATTTG AACTCAAGAT GCCGTAAGTT GAAGGAAAGA   
  
  
+ CCTATCCACA AAGCAATACA CCGCTTGCTA ATAAAAACAC GCTTGTTAAA CATATAATAA TTATGTTGCC   
  
  
+ ACTCATCTTA CGATTTTAAC GGTAATATTT TTGTTCACTT TTAAGGGCAA AGACAAAAAC TTTCAATGGG   
  
  
+ ATAGCTAAAC ATGTTATACA TTTAGCATAA GCAACCCTAA TTAAGCACTA GCAACCTTAT ACATGTTATG   
  
  
+ CATTTAAGGC AGGTCTTATT GGCAATGGTG AGCTAGAGGG GGGCACGGGC ACGGTATTGT GCATAATGAA   
  
  
+ GAAATTTTTT TTGTTTTTTT AAGATTAAAA AAAAATTCAT TTATAAATCA AGAGGTTGGG ACTAGTATTT   
  
  
+ AAGACAACCA CAGTTTGACC CTCGATATAA ATTTATGTAA ATATATTTAT TTAATACAAT TATCATTTAA   
  
  
+ ACGTCGAGAT AGCGATTGTA TCAAATACAT TTAAGTATTG TAATCAAATG ACAAAAAGTT CACCGTGAAC   
  
  
+ AATAGGAACC AAATTAAACA TAAATACTAA GACGTCTTAT ACAATGGTAG TACATGGCAA TTCCAAATAA   
  
  
+ ATTGTCTTCC ACCCCCAGAA AAAAAATTAT GCTTTTAAAA AAAGATTTTG AGAAATTTCA TTCTTATACA   
  
  
+ GTTAAATTTA TCTAAAGGAA TAACTTCTTT AAAGTCATAA AACATTCCGG TCACAACTGG GACCATTTAC   
  
  
+ GTATGGTAAA AAACTCCGCA TTTTAACGAG TCATAACTTT TCTTGATCTC ATCTTAAGGA AACACCGACT   
  
  
+ TTTTTTCATG CCTGCGTCAT CATACATGTT GCCCGAGGAC ACAGCCCATT GACGACTCTA GGACAATTAG   
  
  
+ GTACATGAGA AGGCCTTCGA GGAGTTAGCG GGAGCAATAC TGGTGGATTC AACAAGTATT CTTTCATCTT   
  
  
+ GTCAAAGGCA TGCTGACATT CATCTGTCCA TTCGGTGGAA ACATTCTTCT TCAGCAGCTT GAATAATGGT   
  
  
+ TCACATGTGG CAGTTAGTTG AGATAGTTCA CGTAGTAATA ATTGTCAATG ATTACAAAAA TATGCAGTAT   
  
  
+ CACAAGTAAA AATTTAAGAT GAACTAAAAT ATCTCTTGTT TGAGAAATGT TTCAGCTTTT AGAGTTGATG   
  
  
+ TCACGTCTTT GAGCTTGCAT GTATGTAAT  

- CTTTGAAAAA ATGGAGACTT TAAATGTACA GAAACACTTC CCTTTCTATC TACACCTTTT ATCTCTTCGT   
  
  
- TCCCGCGTCA ATTCCAACAC CTCTCTTCTG TTTCGACTTT CCCAGTTTAC ACACCTTTCC TCCCTTCTCA   
  
  
- TTACCTTCCG AGTTAATCAA ACCACCAAGA AACAGATTAT TATCCTCTTG GTATAGTGAT AAAACAAAAT   
  
  
- AAAACTATAG AATACATATT ACTACAAACA TTGTTTTATA AAAGAGAAAA CTAAAAATAA AAATGTTGCC   
  
  
- TATCCTATTA ACAGATTTGC TATAATCCCT CGTTCTAAAC TTGAGTTCTA CGGCATTCAA CTTCCTTTCT   
  
  
- GGATAGGTGT TTCGTTATGT GGCGAACGAT TATTTTTGTG CGAACAATTT GTATATTATT AATACAACGG   
  
  
- TGAGTAGAAT GCTAAAATTG CCATTATAAA AACAAGTGAA AATTCCCGTT TCTGTTTTTG AAAGTTACCC   
  
  
- TATCGATTTG TACAATATGT AAATCGTATT CGTTGGGATT AATTCGTGAT CGTTGGAATA TGTACAATAC   
  
  
- GTAAATTCCG TCCAGAATAA CCGTTACCAC TCGATCTCCC CCCGTGCCCG TGCCATAACA CGTATTACTT   
  
  
- CTTTAAAAAA AACAAAAAAA TTCTAATTTT TTTTTAAGTA AATATTTAGT TCTCCAACCC TGATCATAAA   
  
  
- TTCTGTTGGT GTCAAACTGG GAGCTATATT TAAATACATT TATATAAATA AATTATGTTA ATAGTAAATT   
  
  
- TGCAGCTCTA TCGCTAACAT AGTTTATGTA AATTCATAAC ATTAGTTTAC TGTTTTTCAA GTGGCACTTG   
  
  
- TTATCCTTGG TTTAATTTGT ATTTATGATT CTGCAGAATA TGTTACCATC ATGTACCGTT AAGGTTTATT   
  
  
- TAACAGAAGG TGGGGGTCTT TTTTTTAATA CGAAAATTTT TTTCTAAAAC TCTTTAAAGT AAGAATATGT   
  
  
- CAATTTAAAT AGATTTCCTT ATTGAAGAAA TTTCAGTATT TTGTAAGGCC AGTGTTGACC CTGGTAAATG   
  
  
- CATACCATTT TTTGAGGCGT AAAATTGCTC AGTATTGAAA AGAACTAGAG TAGAATTCCT TTGTGGCTGA   
  
  
- AAAAAAGTAC GGACGCAGTA GTATGTACAA CGGGCTCCTG TGTCGGGTAA CTGCTGAGAT CCTGTTAATC   
  
  
- CATGTACTCT TCCGGAAGCT CCTCAATCGC CCTCGTTATG ACCACCTAAG TTGTTCATAA GAAAGTAGAA   
  
  
- CAGTTTCCGT ACGACTGTAA GTAGACAGGT AAGCCACCTT TGTAAGAAGA AGTCGTCGAA CTTATTACCA   
  
  
- AGTGTACACC GTCAATCAAC TCTATCAAGT GCATCATTAT TAACAGTTAC TAATGTTTTT ATACGTCATA   
  
  
- GTGTTCATTT TTAAATTCTA CTTGATTTTA TAGAGAACAA ACTCTTTACA AAGTCGAAAA TCTCAACTAC   
  
  
- AGTGCAGAAA CTCGAACGTA CATACATTA

+     MYC

| Site Name | Organism | Position | Strand | Matrix score. | sequence | function |
| --- | --- | --- | --- | --- | --- | --- |
| MYC | Arabidopsis thaliana | 815 | - | 6 | CATTTG |  |
| MYC | Arabidopsis thaliana | 115 | - | 6 | CATTTG |  |
| MYC | Arabidopsis thaliana | 1332 | - | 6 | CATGTG |  |
| MYC | Arabidopsis thaliana | 1334 | + | 6 | CATGTG |  |

>033955   
+ GAAACTTTTT TACCTCTGAA ATTTACATGT CTTTGTGAAG GGAAAGATAG ATGTGGAAAA TAGAGAAGCA   
  
  
+ AGGGCGCAGT TAAGGTTGTG GAGAGAAGAC AAAGCTGAAA GGGTCAAATG TGTGGAAAGG AGGGAAGAGT   
  
  
+ AATGGAAGGC TCAATTAGTT TGGTGGTTCT TTGTCTAATA ATAGGAGAAC CATATCACTA TTTTGTTTTA   
  
  
+ TTTTGATATC TTATGTATAA TGATGTTTGT AACAAAATAT TTTCTCTTTT GATTTTTATT TTTACAACGG   
  
  
+ ATAGGATAAT TGTCTAAACG ATATTAGGGA GCAAGATTTG AACTCAAGAT GCCGTAAGTT GAAGGAAAGA   
  
  
+ CCTATCCACA AAGCAATACA CCGCTTGCTA ATAAAAACAC GCTTGTTAAA CATATAATAA TTATGTTGCC   
  
  
+ ACTCATCTTA CGATTTTAAC GGTAATATTT TTGTTCACTT TTAAGGGCAA AGACAAAAAC TTTCAATGGG   
  
  
+ ATAGCTAAAC ATGTTATACA TTTAGCATAA GCAACCCTAA TTAAGCACTA GCAACCTTAT ACATGTTATG   
  
  
+ CATTTAAGGC AGGTCTTATT GGCAATGGTG AGCTAGAGGG GGGCACGGGC ACGGTATTGT GCATAATGAA   
  
  
+ GAAATTTTTT TTGTTTTTTT AAGATTAAAA AAAAATTCAT TTATAAATCA AGAGGTTGGG ACTAGTATTT   
  
  
+ AAGACAACCA CAGTTTGACC CTCGATATAA ATTTATGTAA ATATATTTAT TTAATACAAT TATCATTTAA   
  
  
+ ACGTCGAGAT AGCGATTGTA TCAAATACAT TTAAGTATTG TAATCAAATG ACAAAAAGTT CACCGTGAAC   
  
  
+ AATAGGAACC AAATTAAACA TAAATACTAA GACGTCTTAT ACAATGGTAG TACATGGCAA TTCCAAATAA   
  
  
+ ATTGTCTTCC ACCCCCAGAA AAAAAATTAT GCTTTTAAAA AAAGATTTTG AGAAATTTCA TTCTTATACA   
  
  
+ GTTAAATTTA TCTAAAGGAA TAACTTCTTT AAAGTCATAA AACATTCCGG TCACAACTGG GACCATTTAC   
  
  
+ GTATGGTAAA AAACTCCGCA TTTTAACGAG TCATAACTTT TCTTGATCTC ATCTTAAGGA AACACCGACT   
  
  
+ TTTTTTCATG CCTGCGTCAT CATACATGTT GCCCGAGGAC ACAGCCCATT GACGACTCTA GGACAATTAG   
  
  
+ GTACATGAGA AGGCCTTCGA GGAGTTAGCG GGAGCAATAC TGGTGGATTC AACAAGTATT CTTTCATCTT   
  
  
+ GTCAAAGGCA TGCTGACATT CATCTGTCCA TTCGGTGGAA ACATTCTTCT TCAGCAGCTT GAATAATGGT   
  
  
+ TCACATGTGG CAGTTAGTTG AGATAGTTCA CGTAGTAATA ATTGTCAATG ATTACAAAAA TATGCAGTAT   
  
  
+ CACAAGTAAA AATTTAAGAT GAACTAAAAT ATCTCTTGTT TGAGAAATGT TTCAGCTTTT AGAGTTGATG   
  
  
+ TCACGTCTTT GAGCTTGCAT GTATGTAAT  

- CTTTGAAAAA ATGGAGACTT TAAATGTACA GAAACACTTC CCTTTCTATC TACACCTTTT ATCTCTTCGT   
  
  
- TCCCGCGTCA ATTCCAACAC CTCTCTTCTG TTTCGACTTT CCCAGTTTAC ACACCTTTCC TCCCTTCTCA   
  
  
- TTACCTTCCG AGTTAATCAA ACCACCAAGA AACAGATTAT TATCCTCTTG GTATAGTGAT AAAACAAAAT   
  
  
- AAAACTATAG AATACATATT ACTACAAACA TTGTTTTATA AAAGAGAAAA CTAAAAATAA AAATGTTGCC   
  
  
- TATCCTATTA ACAGATTTGC TATAATCCCT CGTTCTAAAC TTGAGTTCTA CGGCATTCAA CTTCCTTTCT   
  
  
- GGATAGGTGT TTCGTTATGT GGCGAACGAT TATTTTTGTG CGAACAATTT GTATATTATT AATACAACGG   
  
  
- TGAGTAGAAT GCTAAAATTG CCATTATAAA AACAAGTGAA AATTCCCGTT TCTGTTTTTG AAAGTTACCC   
  
  
- TATCGATTTG TACAATATGT AAATCGTATT CGTTGGGATT AATTCGTGAT CGTTGGAATA TGTACAATAC   
  
  
- GTAAATTCCG TCCAGAATAA CCGTTACCAC TCGATCTCCC CCCGTGCCCG TGCCATAACA CGTATTACTT   
  
  
- CTTTAAAAAA AACAAAAAAA TTCTAATTTT TTTTTAAGTA AATATTTAGT TCTCCAACCC TGATCATAAA   
  
  
- TTCTGTTGGT GTCAAACTGG GAGCTATATT TAAATACATT TATATAAATA AATTATGTTA ATAGTAAATT   
  
  
- TGCAGCTCTA TCGCTAACAT AGTTTATGTA AATTCATAAC ATTAGTTTAC TGTTTTTCAA GTGGCACTTG   
  
  
- TTATCCTTGG TTTAATTTGT ATTTATGATT CTGCAGAATA TGTTACCATC ATGTACCGTT AAGGTTTATT   
  
  
- TAACAGAAGG TGGGGGTCTT TTTTTTAATA CGAAAATTTT TTTCTAAAAC TCTTTAAAGT AAGAATATGT   
  
  
- CAATTTAAAT AGATTTCCTT ATTGAAGAAA TTTCAGTATT TTGTAAGGCC AGTGTTGACC CTGGTAAATG   
  
  
- CATACCATTT TTTGAGGCGT AAAATTGCTC AGTATTGAAA AGAACTAGAG TAGAATTCCT TTGTGGCTGA   
  
  
- AAAAAAGTAC GGACGCAGTA GTATGTACAA CGGGCTCCTG TGTCGGGTAA CTGCTGAGAT CCTGTTAATC   
  
  
- CATGTACTCT TCCGGAAGCT CCTCAATCGC CCTCGTTATG ACCACCTAAG TTGTTCATAA GAAAGTAGAA   
  
  
- CAGTTTCCGT ACGACTGTAA GTAGACAGGT AAGCCACCTT TGTAAGAAGA AGTCGTCGAA CTTATTACCA   
  
  
- AGTGTACACC GTCAATCAAC TCTATCAAGT GCATCATTAT TAACAGTTAC TAATGTTTTT ATACGTCATA   
  
  
- GTGTTCATTT TTAAATTCTA CTTGATTTTA TAGAGAACAA ACTCTTTACA AAGTCGAAAA TCTCAACTAC   
  
  
- AGTGCAGAAA CTCGAACGTA CATACATTA

+     Myb

| Site Name | Organism | Position | Strand | Matrix score. | sequence | function |
| --- | --- | --- | --- | --- | --- | --- |
| Myb | Arabidopsis thaliana | 1034 | + | 6 | CAACTG |  |
| Myb | Arabidopsis thaliana | 979 | - | 6 | TAACTG |  |
| Myb | Arabidopsis thaliana | 1341 | - | 6 | TAACTG |  |
| Myb | Arabidopsis thaliana | 77 | - | 6 | TAACTG |  |

>033955   
+ GAAACTTTTT TACCTCTGAA ATTTACATGT CTTTGTGAAG GGAAAGATAG ATGTGGAAAA TAGAGAAGCA   
  
  
+ AGGGCGCAGT TAAGGTTGTG GAGAGAAGAC AAAGCTGAAA GGGTCAAATG TGTGGAAAGG AGGGAAGAGT   
  
  
+ AATGGAAGGC TCAATTAGTT TGGTGGTTCT TTGTCTAATA ATAGGAGAAC CATATCACTA TTTTGTTTTA   
  
  
+ TTTTGATATC TTATGTATAA TGATGTTTGT AACAAAATAT TTTCTCTTTT GATTTTTATT TTTACAACGG   
  
  
+ ATAGGATAAT TGTCTAAACG ATATTAGGGA GCAAGATTTG AACTCAAGAT GCCGTAAGTT GAAGGAAAGA   
  
  
+ CCTATCCACA AAGCAATACA CCGCTTGCTA ATAAAAACAC GCTTGTTAAA CATATAATAA TTATGTTGCC   
  
  
+ ACTCATCTTA CGATTTTAAC GGTAATATTT TTGTTCACTT TTAAGGGCAA AGACAAAAAC TTTCAATGGG   
  
  
+ ATAGCTAAAC ATGTTATACA TTTAGCATAA GCAACCCTAA TTAAGCACTA GCAACCTTAT ACATGTTATG   
  
  
+ CATTTAAGGC AGGTCTTATT GGCAATGGTG AGCTAGAGGG GGGCACGGGC ACGGTATTGT GCATAATGAA   
  
  
+ GAAATTTTTT TTGTTTTTTT AAGATTAAAA AAAAATTCAT TTATAAATCA AGAGGTTGGG ACTAGTATTT   
  
  
+ AAGACAACCA CAGTTTGACC CTCGATATAA ATTTATGTAA ATATATTTAT TTAATACAAT TATCATTTAA   
  
  
+ ACGTCGAGAT AGCGATTGTA TCAAATACAT TTAAGTATTG TAATCAAATG ACAAAAAGTT CACCGTGAAC   
  
  
+ AATAGGAACC AAATTAAACA TAAATACTAA GACGTCTTAT ACAATGGTAG TACATGGCAA TTCCAAATAA   
  
  
+ ATTGTCTTCC ACCCCCAGAA AAAAAATTAT GCTTTTAAAA AAAGATTTTG AGAAATTTCA TTCTTATACA   
  
  
+ GTTAAATTTA TCTAAAGGAA TAACTTCTTT AAAGTCATAA AACATTCCGG TCACAACTGG GACCATTTAC   
  
  
+ GTATGGTAAA AAACTCCGCA TTTTAACGAG TCATAACTTT TCTTGATCTC ATCTTAAGGA AACACCGACT   
  
  
+ TTTTTTCATG CCTGCGTCAT CATACATGTT GCCCGAGGAC ACAGCCCATT GACGACTCTA GGACAATTAG   
  
  
+ GTACATGAGA AGGCCTTCGA GGAGTTAGCG GGAGCAATAC TGGTGGATTC AACAAGTATT CTTTCATCTT   
  
  
+ GTCAAAGGCA TGCTGACATT CATCTGTCCA TTCGGTGGAA ACATTCTTCT TCAGCAGCTT GAATAATGGT   
  
  
+ TCACATGTGG CAGTTAGTTG AGATAGTTCA CGTAGTAATA ATTGTCAATG ATTACAAAAA TATGCAGTAT   
  
  
+ CACAAGTAAA AATTTAAGAT GAACTAAAAT ATCTCTTGTT TGAGAAATGT TTCAGCTTTT AGAGTTGATG   
  
  
+ TCACGTCTTT GAGCTTGCAT GTATGTAAT  

- CTTTGAAAAA ATGGAGACTT TAAATGTACA GAAACACTTC CCTTTCTATC TACACCTTTT ATCTCTTCGT   
  
  
- TCCCGCGTCA ATTCCAACAC CTCTCTTCTG TTTCGACTTT CCCAGTTTAC ACACCTTTCC TCCCTTCTCA   
  
  
- TTACCTTCCG AGTTAATCAA ACCACCAAGA AACAGATTAT TATCCTCTTG GTATAGTGAT AAAACAAAAT   
  
  
- AAAACTATAG AATACATATT ACTACAAACA TTGTTTTATA AAAGAGAAAA CTAAAAATAA AAATGTTGCC   
  
  
- TATCCTATTA ACAGATTTGC TATAATCCCT CGTTCTAAAC TTGAGTTCTA CGGCATTCAA CTTCCTTTCT   
  
  
- GGATAGGTGT TTCGTTATGT GGCGAACGAT TATTTTTGTG CGAACAATTT GTATATTATT AATACAACGG   
  
  
- TGAGTAGAAT GCTAAAATTG CCATTATAAA AACAAGTGAA AATTCCCGTT TCTGTTTTTG AAAGTTACCC   
  
  
- TATCGATTTG TACAATATGT AAATCGTATT CGTTGGGATT AATTCGTGAT CGTTGGAATA TGTACAATAC   
  
  
- GTAAATTCCG TCCAGAATAA CCGTTACCAC TCGATCTCCC CCCGTGCCCG TGCCATAACA CGTATTACTT   
  
  
- CTTTAAAAAA AACAAAAAAA TTCTAATTTT TTTTTAAGTA AATATTTAGT TCTCCAACCC TGATCATAAA   
  
  
- TTCTGTTGGT GTCAAACTGG GAGCTATATT TAAATACATT TATATAAATA AATTATGTTA ATAGTAAATT   
  
  
- TGCAGCTCTA TCGCTAACAT AGTTTATGTA AATTCATAAC ATTAGTTTAC TGTTTTTCAA GTGGCACTTG   
  
  
- TTATCCTTGG TTTAATTTGT ATTTATGATT CTGCAGAATA TGTTACCATC ATGTACCGTT AAGGTTTATT   
  
  
- TAACAGAAGG TGGGGGTCTT TTTTTTAATA CGAAAATTTT TTTCTAAAAC TCTTTAAAGT AAGAATATGT   
  
  
- CAATTTAAAT AGATTTCCTT ATTGAAGAAA TTTCAGTATT TTGTAAGGCC AGTGTTGACC CTGGTAAATG   
  
  
- CATACCATTT TTTGAGGCGT AAAATTGCTC AGTATTGAAA AGAACTAGAG TAGAATTCCT TTGTGGCTGA   
  
  
- AAAAAAGTAC GGACGCAGTA GTATGTACAA CGGGCTCCTG TGTCGGGTAA CTGCTGAGAT CCTGTTAATC   
  
  
- CATGTACTCT TCCGGAAGCT CCTCAATCGC CCTCGTTATG ACCACCTAAG TTGTTCATAA GAAAGTAGAA   
  
  
- CAGTTTCCGT ACGACTGTAA GTAGACAGGT AAGCCACCTT TGTAAGAAGA AGTCGTCGAA CTTATTACCA   
  
  
- AGTGTACACC GTCAATCAAC TCTATCAAGT GCATCATTAT TAACAGTTAC TAATGTTTTT ATACGTCATA   
  
  
- GTGTTCATTT TTAAATTCTA CTTGATTTTA TAGAGAACAA ACTCTTTACA AAGTCGAAAA TCTCAACTAC   
  
  
- AGTGCAGAAA CTCGAACGTA CATACATTA

+     STRE

| Site Name | Organism | Position | Strand | Matrix score. | sequence | function |
| --- | --- | --- | --- | --- | --- | --- |
| STRE | Arabidopsis thaliana | 597 | + | 5 | AGGGG |  |

>033955   
+ GAAACTTTTT TACCTCTGAA ATTTACATGT CTTTGTGAAG GGAAAGATAG ATGTGGAAAA TAGAGAAGCA   
  
  
+ AGGGCGCAGT TAAGGTTGTG GAGAGAAGAC AAAGCTGAAA GGGTCAAATG TGTGGAAAGG AGGGAAGAGT   
  
  
+ AATGGAAGGC TCAATTAGTT TGGTGGTTCT TTGTCTAATA ATAGGAGAAC CATATCACTA TTTTGTTTTA   
  
  
+ TTTTGATATC TTATGTATAA TGATGTTTGT AACAAAATAT TTTCTCTTTT GATTTTTATT TTTACAACGG   
  
  
+ ATAGGATAAT TGTCTAAACG ATATTAGGGA GCAAGATTTG AACTCAAGAT GCCGTAAGTT GAAGGAAAGA   
  
  
+ CCTATCCACA AAGCAATACA CCGCTTGCTA ATAAAAACAC GCTTGTTAAA CATATAATAA TTATGTTGCC   
  
  
+ ACTCATCTTA CGATTTTAAC GGTAATATTT TTGTTCACTT TTAAGGGCAA AGACAAAAAC TTTCAATGGG   
  
  
+ ATAGCTAAAC ATGTTATACA TTTAGCATAA GCAACCCTAA TTAAGCACTA GCAACCTTAT ACATGTTATG   
  
  
+ CATTTAAGGC AGGTCTTATT GGCAATGGTG AGCTAGAGGG GGGCACGGGC ACGGTATTGT GCATAATGAA   
  
  
+ GAAATTTTTT TTGTTTTTTT AAGATTAAAA AAAAATTCAT TTATAAATCA AGAGGTTGGG ACTAGTATTT   
  
  
+ AAGACAACCA CAGTTTGACC CTCGATATAA ATTTATGTAA ATATATTTAT TTAATACAAT TATCATTTAA   
  
  
+ ACGTCGAGAT AGCGATTGTA TCAAATACAT TTAAGTATTG TAATCAAATG ACAAAAAGTT CACCGTGAAC   
  
  
+ AATAGGAACC AAATTAAACA TAAATACTAA GACGTCTTAT ACAATGGTAG TACATGGCAA TTCCAAATAA   
  
  
+ ATTGTCTTCC ACCCCCAGAA AAAAAATTAT GCTTTTAAAA AAAGATTTTG AGAAATTTCA TTCTTATACA   
  
  
+ GTTAAATTTA TCTAAAGGAA TAACTTCTTT AAAGTCATAA AACATTCCGG TCACAACTGG GACCATTTAC   
  
  
+ GTATGGTAAA AAACTCCGCA TTTTAACGAG TCATAACTTT TCTTGATCTC ATCTTAAGGA AACACCGACT   
  
  
+ TTTTTTCATG CCTGCGTCAT CATACATGTT GCCCGAGGAC ACAGCCCATT GACGACTCTA GGACAATTAG   
  
  
+ GTACATGAGA AGGCCTTCGA GGAGTTAGCG GGAGCAATAC TGGTGGATTC AACAAGTATT CTTTCATCTT   
  
  
+ GTCAAAGGCA TGCTGACATT CATCTGTCCA TTCGGTGGAA ACATTCTTCT TCAGCAGCTT GAATAATGGT   
  
  
+ TCACATGTGG CAGTTAGTTG AGATAGTTCA CGTAGTAATA ATTGTCAATG ATTACAAAAA TATGCAGTAT   
  
  
+ CACAAGTAAA AATTTAAGAT GAACTAAAAT ATCTCTTGTT TGAGAAATGT TTCAGCTTTT AGAGTTGATG   
  
  
+ TCACGTCTTT GAGCTTGCAT GTATGTAAT  

- CTTTGAAAAA ATGGAGACTT TAAATGTACA GAAACACTTC CCTTTCTATC TACACCTTTT ATCTCTTCGT   
  
  
- TCCCGCGTCA ATTCCAACAC CTCTCTTCTG TTTCGACTTT CCCAGTTTAC ACACCTTTCC TCCCTTCTCA   
  
  
- TTACCTTCCG AGTTAATCAA ACCACCAAGA AACAGATTAT TATCCTCTTG GTATAGTGAT AAAACAAAAT   
  
  
- AAAACTATAG AATACATATT ACTACAAACA TTGTTTTATA AAAGAGAAAA CTAAAAATAA AAATGTTGCC   
  
  
- TATCCTATTA ACAGATTTGC TATAATCCCT CGTTCTAAAC TTGAGTTCTA CGGCATTCAA CTTCCTTTCT   
  
  
- GGATAGGTGT TTCGTTATGT GGCGAACGAT TATTTTTGTG CGAACAATTT GTATATTATT AATACAACGG   
  
  
- TGAGTAGAAT GCTAAAATTG CCATTATAAA AACAAGTGAA AATTCCCGTT TCTGTTTTTG AAAGTTACCC   
  
  
- TATCGATTTG TACAATATGT AAATCGTATT CGTTGGGATT AATTCGTGAT CGTTGGAATA TGTACAATAC   
  
  
- GTAAATTCCG TCCAGAATAA CCGTTACCAC TCGATCTCCC CCCGTGCCCG TGCCATAACA CGTATTACTT   
  
  
- CTTTAAAAAA AACAAAAAAA TTCTAATTTT TTTTTAAGTA AATATTTAGT TCTCCAACCC TGATCATAAA   
  
  
- TTCTGTTGGT GTCAAACTGG GAGCTATATT TAAATACATT TATATAAATA AATTATGTTA ATAGTAAATT   
  
  
- TGCAGCTCTA TCGCTAACAT AGTTTATGTA AATTCATAAC ATTAGTTTAC TGTTTTTCAA GTGGCACTTG   
  
  
- TTATCCTTGG TTTAATTTGT ATTTATGATT CTGCAGAATA TGTTACCATC ATGTACCGTT AAGGTTTATT   
  
  
- TAACAGAAGG TGGGGGTCTT TTTTTTAATA CGAAAATTTT TTTCTAAAAC TCTTTAAAGT AAGAATATGT   
  
  
- CAATTTAAAT AGATTTCCTT ATTGAAGAAA TTTCAGTATT TTGTAAGGCC AGTGTTGACC CTGGTAAATG   
  
  
- CATACCATTT TTTGAGGCGT AAAATTGCTC AGTATTGAAA AGAACTAGAG TAGAATTCCT TTGTGGCTGA   
  
  
- AAAAAAGTAC GGACGCAGTA GTATGTACAA CGGGCTCCTG TGTCGGGTAA CTGCTGAGAT CCTGTTAATC   
  
  
- CATGTACTCT TCCGGAAGCT CCTCAATCGC CCTCGTTATG ACCACCTAAG TTGTTCATAA GAAAGTAGAA   
  
  
- CAGTTTCCGT ACGACTGTAA GTAGACAGGT AAGCCACCTT TGTAAGAAGA AGTCGTCGAA CTTATTACCA   
  
  
- AGTGTACACC GTCAATCAAC TCTATCAAGT GCATCATTAT TAACAGTTAC TAATGTTTTT ATACGTCATA   
  
  
- GTGTTCATTT TTAAATTCTA CTTGATTTTA TAGAGAACAA ACTCTTTACA AAGTCGAAAA TCTCAACTAC   
  
  
- AGTGCAGAAA CTCGAACGTA CATACATTA

+     TATA-box

| Site Name | Organism | Position | Strand | Matrix score. | sequence | function |
| --- | --- | --- | --- | --- | --- | --- |
| TATA-box | Arabidopsis thaliana | 726 | + | 4 | TATA | core promoter element around -30 of transcription start |
| TATA-box | Arabidopsis thaliana | 672 | + | 4 | TATA | core promoter element around -30 of transcription start |
| TATA-box | Arabidopsis thaliana | 671 | - | 5 | TATAA | core promoter element around -30 of transcription start |
| TATA-box | Arabidopsis thaliana | 670 | + | 9 | taTATAAAtc | core promoter element around -30 of transcription start |
| TATA-box | Arabidopsis thaliana | 226 | + | 4 | TATA | core promoter element around -30 of transcription start |
| TATA-box | Arabidopsis thaliana | 504 | - | 5 | TATAA | core promoter element around -30 of transcription start |
| TATA-box | Zea mays | 971 | - | 8 | TATAAGAA | core promoter element around -30 of transcription start |
| TATA-box | Helianthus annuus | 224 | - | 6 | TATACA | core promoter element around -30 of transcription start |
| TATA-box | Arabidopsis thaliana | 742 | + | 4 | TATA | core promoter element around -30 of transcription start |
| TATA-box | Brassica juncea | 669 | - | 7 | TATAAAT | core promoter element around -30 of transcription start |
| TATA-box | Oryza sativa | 1383 | + | 7 | TACAAAA | core promoter element around -30 of transcription start |
| TATA-box | Arabidopsis thaliana | 403 | + | 4 | TATA | core promoter element around -30 of transcription start |
| TATA-box | Oryza sativa | 732 | - | 8 | TACATAAA | core promoter element around -30 of transcription start |
| TATA-box | Brassica oleracea | 402 | + | 6 | ATATAA | core promoter element around -30 of transcription start |
| TATA-box | Arabidopsis thaliana | 975 | - | 4 | TATA | core promoter element around -30 of transcription start |
| TATA-box | Arabidopsis thaliana | 548 | + | 4 | TATA | core promoter element around -30 of transcription start |
| TATA-box | Arabidopsis thaliana | 547 | - | 5 | TATAA | core promoter element around -30 of transcription start |
| TATA-box | Arabidopsis thaliana | 878 | - | 4 | TATA | core promoter element around -30 of transcription start |
| TATA-box | Arabidopsis thaliana | 505 | + | 4 | TATA | core promoter element around -30 of transcription start |
| TATA-box | Arabidopsis thaliana | 877 | - | 5 | TATAA | core promoter element around -30 of transcription start |
| TATA-box | Brassica oleracea | 725 | + | 6 | ATATAA | core promoter element around -30 of transcription start |
| TATA-box | Arabidopsis thaliana | 974 | - | 5 | TATAA | core promoter element around -30 of transcription start |
| TATA-box | Brassica napus | 741 | + | 6 | ATATAT | core promoter element around -30 of transcription start |

>033955   
+ GAAACTTTTT TACCTCTGAA ATTTACATGT CTTTGTGAAG GGAAAGATAG ATGTGGAAAA TAGAGAAGCA   
  
  
+ AGGGCGCAGT TAAGGTTGTG GAGAGAAGAC AAAGCTGAAA GGGTCAAATG TGTGGAAAGG AGGGAAGAGT   
  
  
+ AATGGAAGGC TCAATTAGTT TGGTGGTTCT TTGTCTAATA ATAGGAGAAC CATATCACTA TTTTGTTTTA   
  
  
+ TTTTGATATC TTATGTATAA TGATGTTTGT AACAAAATAT TTTCTCTTTT GATTTTTATT TTTACAACGG   
  
  
+ ATAGGATAAT TGTCTAAACG ATATTAGGGA GCAAGATTTG AACTCAAGAT GCCGTAAGTT GAAGGAAAGA   
  
  
+ CCTATCCACA AAGCAATACA CCGCTTGCTA ATAAAAACAC GCTTGTTAAA CATATAATAA TTATGTTGCC   
  
  
+ ACTCATCTTA CGATTTTAAC GGTAATATTT TTGTTCACTT TTAAGGGCAA AGACAAAAAC TTTCAATGGG   
  
  
+ ATAGCTAAAC ATGTTATACA TTTAGCATAA GCAACCCTAA TTAAGCACTA GCAACCTTAT ACATGTTATG   
  
  
+ CATTTAAGGC AGGTCTTATT GGCAATGGTG AGCTAGAGGG GGGCACGGGC ACGGTATTGT GCATAATGAA   
  
  
+ GAAATTTTTT TTGTTTTTTT AAGATTAAAA AAAAATTCAT TTATAAATCA AGAGGTTGGG ACTAGTATTT   
  
  
+ AAGACAACCA CAGTTTGACC CTCGATATAA ATTTATGTAA ATATATTTAT TTAATACAAT TATCATTTAA   
  
  
+ ACGTCGAGAT AGCGATTGTA TCAAATACAT TTAAGTATTG TAATCAAATG ACAAAAAGTT CACCGTGAAC   
  
  
+ AATAGGAACC AAATTAAACA TAAATACTAA GACGTCTTAT ACAATGGTAG TACATGGCAA TTCCAAATAA   
  
  
+ ATTGTCTTCC ACCCCCAGAA AAAAAATTAT GCTTTTAAAA AAAGATTTTG AGAAATTTCA TTCTTATACA   
  
  
+ GTTAAATTTA TCTAAAGGAA TAACTTCTTT AAAGTCATAA AACATTCCGG TCACAACTGG GACCATTTAC   
  
  
+ GTATGGTAAA AAACTCCGCA TTTTAACGAG TCATAACTTT TCTTGATCTC ATCTTAAGGA AACACCGACT   
  
  
+ TTTTTTCATG CCTGCGTCAT CATACATGTT GCCCGAGGAC ACAGCCCATT GACGACTCTA GGACAATTAG   
  
  
+ GTACATGAGA AGGCCTTCGA GGAGTTAGCG GGAGCAATAC TGGTGGATTC AACAAGTATT CTTTCATCTT   
  
  
+ GTCAAAGGCA TGCTGACATT CATCTGTCCA TTCGGTGGAA ACATTCTTCT TCAGCAGCTT GAATAATGGT   
  
  
+ TCACATGTGG CAGTTAGTTG AGATAGTTCA CGTAGTAATA ATTGTCAATG ATTACAAAAA TATGCAGTAT   
  
  
+ CACAAGTAAA AATTTAAGAT GAACTAAAAT ATCTCTTGTT TGAGAAATGT TTCAGCTTTT AGAGTTGATG   
  
  
+ TCACGTCTTT GAGCTTGCAT GTATGTAAT  

- CTTTGAAAAA ATGGAGACTT TAAATGTACA GAAACACTTC CCTTTCTATC TACACCTTTT ATCTCTTCGT   
  
  
- TCCCGCGTCA ATTCCAACAC CTCTCTTCTG TTTCGACTTT CCCAGTTTAC ACACCTTTCC TCCCTTCTCA   
  
  
- TTACCTTCCG AGTTAATCAA ACCACCAAGA AACAGATTAT TATCCTCTTG GTATAGTGAT AAAACAAAAT   
  
  
- AAAACTATAG AATACATATT ACTACAAACA TTGTTTTATA AAAGAGAAAA CTAAAAATAA AAATGTTGCC   
  
  
- TATCCTATTA ACAGATTTGC TATAATCCCT CGTTCTAAAC TTGAGTTCTA CGGCATTCAA CTTCCTTTCT   
  
  
- GGATAGGTGT TTCGTTATGT GGCGAACGAT TATTTTTGTG CGAACAATTT GTATATTATT AATACAACGG   
  
  
- TGAGTAGAAT GCTAAAATTG CCATTATAAA AACAAGTGAA AATTCCCGTT TCTGTTTTTG AAAGTTACCC   
  
  
- TATCGATTTG TACAATATGT AAATCGTATT CGTTGGGATT AATTCGTGAT CGTTGGAATA TGTACAATAC   
  
  
- GTAAATTCCG TCCAGAATAA CCGTTACCAC TCGATCTCCC CCCGTGCCCG TGCCATAACA CGTATTACTT   
  
  
- CTTTAAAAAA AACAAAAAAA TTCTAATTTT TTTTTAAGTA AATATTTAGT TCTCCAACCC TGATCATAAA   
  
  
- TTCTGTTGGT GTCAAACTGG GAGCTATATT TAAATACATT TATATAAATA AATTATGTTA ATAGTAAATT   
  
  
- TGCAGCTCTA TCGCTAACAT AGTTTATGTA AATTCATAAC ATTAGTTTAC TGTTTTTCAA GTGGCACTTG   
  
  
- TTATCCTTGG TTTAATTTGT ATTTATGATT CTGCAGAATA TGTTACCATC ATGTACCGTT AAGGTTTATT   
  
  
- TAACAGAAGG TGGGGGTCTT TTTTTTAATA CGAAAATTTT TTTCTAAAAC TCTTTAAAGT AAGAATATGT   
  
  
- CAATTTAAAT AGATTTCCTT ATTGAAGAAA TTTCAGTATT TTGTAAGGCC AGTGTTGACC CTGGTAAATG   
  
  
- CATACCATTT TTTGAGGCGT AAAATTGCTC AGTATTGAAA AGAACTAGAG TAGAATTCCT TTGTGGCTGA   
  
  
- AAAAAAGTAC GGACGCAGTA GTATGTACAA CGGGCTCCTG TGTCGGGTAA CTGCTGAGAT CCTGTTAATC   
  
  
- CATGTACTCT TCCGGAAGCT CCTCAATCGC CCTCGTTATG ACCACCTAAG TTGTTCATAA GAAAGTAGAA   
  
  
- CAGTTTCCGT ACGACTGTAA GTAGACAGGT AAGCCACCTT TGTAAGAAGA AGTCGTCGAA CTTATTACCA   
  
  
- AGTGTACACC GTCAATCAAC TCTATCAAGT GCATCATTAT TAACAGTTAC TAATGTTTTT ATACGTCATA   
  
  
- GTGTTCATTT TTAAATTCTA CTTGATTTTA TAGAGAACAA ACTCTTTACA AAGTCGAAAA TCTCAACTAC   
  
  
- AGTGCAGAAA CTCGAACGTA CATACATTA

+     TATC-box

| Site Name | Organism | Position | Strand | Matrix score. | sequence | function |
| --- | --- | --- | --- | --- | --- | --- |
| TATC-box | Oryza sativa | 487 | - | 7 | TATCCCA | cis-acting element involved in gibberellin-responsiveness |

>033955   
+ GAAACTTTTT TACCTCTGAA ATTTACATGT CTTTGTGAAG GGAAAGATAG ATGTGGAAAA TAGAGAAGCA   
  
  
+ AGGGCGCAGT TAAGGTTGTG GAGAGAAGAC AAAGCTGAAA GGGTCAAATG TGTGGAAAGG AGGGAAGAGT   
  
  
+ AATGGAAGGC TCAATTAGTT TGGTGGTTCT TTGTCTAATA ATAGGAGAAC CATATCACTA TTTTGTTTTA   
  
  
+ TTTTGATATC TTATGTATAA TGATGTTTGT AACAAAATAT TTTCTCTTTT GATTTTTATT TTTACAACGG   
  
  
+ ATAGGATAAT TGTCTAAACG ATATTAGGGA GCAAGATTTG AACTCAAGAT GCCGTAAGTT GAAGGAAAGA   
  
  
+ CCTATCCACA AAGCAATACA CCGCTTGCTA ATAAAAACAC GCTTGTTAAA CATATAATAA TTATGTTGCC   
  
  
+ ACTCATCTTA CGATTTTAAC GGTAATATTT TTGTTCACTT TTAAGGGCAA AGACAAAAAC TTTCAATGGG   
  
  
+ ATAGCTAAAC ATGTTATACA TTTAGCATAA GCAACCCTAA TTAAGCACTA GCAACCTTAT ACATGTTATG   
  
  
+ CATTTAAGGC AGGTCTTATT GGCAATGGTG AGCTAGAGGG GGGCACGGGC ACGGTATTGT GCATAATGAA   
  
  
+ GAAATTTTTT TTGTTTTTTT AAGATTAAAA AAAAATTCAT TTATAAATCA AGAGGTTGGG ACTAGTATTT   
  
  
+ AAGACAACCA CAGTTTGACC CTCGATATAA ATTTATGTAA ATATATTTAT TTAATACAAT TATCATTTAA   
  
  
+ ACGTCGAGAT AGCGATTGTA TCAAATACAT TTAAGTATTG TAATCAAATG ACAAAAAGTT CACCGTGAAC   
  
  
+ AATAGGAACC AAATTAAACA TAAATACTAA GACGTCTTAT ACAATGGTAG TACATGGCAA TTCCAAATAA   
  
  
+ ATTGTCTTCC ACCCCCAGAA AAAAAATTAT GCTTTTAAAA AAAGATTTTG AGAAATTTCA TTCTTATACA   
  
  
+ GTTAAATTTA TCTAAAGGAA TAACTTCTTT AAAGTCATAA AACATTCCGG TCACAACTGG GACCATTTAC   
  
  
+ GTATGGTAAA AAACTCCGCA TTTTAACGAG TCATAACTTT TCTTGATCTC ATCTTAAGGA AACACCGACT   
  
  
+ TTTTTTCATG CCTGCGTCAT CATACATGTT GCCCGAGGAC ACAGCCCATT GACGACTCTA GGACAATTAG   
  
  
+ GTACATGAGA AGGCCTTCGA GGAGTTAGCG GGAGCAATAC TGGTGGATTC AACAAGTATT CTTTCATCTT   
  
  
+ GTCAAAGGCA TGCTGACATT CATCTGTCCA TTCGGTGGAA ACATTCTTCT TCAGCAGCTT GAATAATGGT   
  
  
+ TCACATGTGG CAGTTAGTTG AGATAGTTCA CGTAGTAATA ATTGTCAATG ATTACAAAAA TATGCAGTAT   
  
  
+ CACAAGTAAA AATTTAAGAT GAACTAAAAT ATCTCTTGTT TGAGAAATGT TTCAGCTTTT AGAGTTGATG   
  
  
+ TCACGTCTTT GAGCTTGCAT GTATGTAAT  

- CTTTGAAAAA ATGGAGACTT TAAATGTACA GAAACACTTC CCTTTCTATC TACACCTTTT ATCTCTTCGT   
  
  
- TCCCGCGTCA ATTCCAACAC CTCTCTTCTG TTTCGACTTT CCCAGTTTAC ACACCTTTCC TCCCTTCTCA   
  
  
- TTACCTTCCG AGTTAATCAA ACCACCAAGA AACAGATTAT TATCCTCTTG GTATAGTGAT AAAACAAAAT   
  
  
- AAAACTATAG AATACATATT ACTACAAACA TTGTTTTATA AAAGAGAAAA CTAAAAATAA AAATGTTGCC   
  
  
- TATCCTATTA ACAGATTTGC TATAATCCCT CGTTCTAAAC TTGAGTTCTA CGGCATTCAA CTTCCTTTCT   
  
  
- GGATAGGTGT TTCGTTATGT GGCGAACGAT TATTTTTGTG CGAACAATTT GTATATTATT AATACAACGG   
  
  
- TGAGTAGAAT GCTAAAATTG CCATTATAAA AACAAGTGAA AATTCCCGTT TCTGTTTTTG AAAGTTACCC   
  
  
- TATCGATTTG TACAATATGT AAATCGTATT CGTTGGGATT AATTCGTGAT CGTTGGAATA TGTACAATAC   
  
  
- GTAAATTCCG TCCAGAATAA CCGTTACCAC TCGATCTCCC CCCGTGCCCG TGCCATAACA CGTATTACTT   
  
  
- CTTTAAAAAA AACAAAAAAA TTCTAATTTT TTTTTAAGTA AATATTTAGT TCTCCAACCC TGATCATAAA   
  
  
- TTCTGTTGGT GTCAAACTGG GAGCTATATT TAAATACATT TATATAAATA AATTATGTTA ATAGTAAATT   
  
  
- TGCAGCTCTA TCGCTAACAT AGTTTATGTA AATTCATAAC ATTAGTTTAC TGTTTTTCAA GTGGCACTTG   
  
  
- TTATCCTTGG TTTAATTTGT ATTTATGATT CTGCAGAATA TGTTACCATC ATGTACCGTT AAGGTTTATT   
  
  
- TAACAGAAGG TGGGGGTCTT TTTTTTAATA CGAAAATTTT TTTCTAAAAC TCTTTAAAGT AAGAATATGT   
  
  
- CAATTTAAAT AGATTTCCTT ATTGAAGAAA TTTCAGTATT TTGTAAGGCC AGTGTTGACC CTGGTAAATG   
  
  
- CATACCATTT TTTGAGGCGT AAAATTGCTC AGTATTGAAA AGAACTAGAG TAGAATTCCT TTGTGGCTGA   
  
  
- AAAAAAGTAC GGACGCAGTA GTATGTACAA CGGGCTCCTG TGTCGGGTAA CTGCTGAGAT CCTGTTAATC   
  
  
- CATGTACTCT TCCGGAAGCT CCTCAATCGC CCTCGTTATG ACCACCTAAG TTGTTCATAA GAAAGTAGAA   
  
  
- CAGTTTCCGT ACGACTGTAA GTAGACAGGT AAGCCACCTT TGTAAGAAGA AGTCGTCGAA CTTATTACCA   
  
  
- AGTGTACACC GTCAATCAAC TCTATCAAGT GCATCATTAT TAACAGTTAC TAATGTTTTT ATACGTCATA   
  
  
- GTGTTCATTT TTAAATTCTA CTTGATTTTA TAGAGAACAA ACTCTTTACA AAGTCGAAAA TCTCAACTAC   
  
  
- AGTGCAGAAA CTCGAACGTA CATACATTA

+     TCT-motif

| Site Name | Organism | Position | Strand | Matrix score. | sequence | function |
| --- | --- | --- | --- | --- | --- | --- |
| TCT-motif | Arabidopsis thaliana | 426 | + | 6 | TCTTAC | part of a light responsive element |

>033955   
+ GAAACTTTTT TACCTCTGAA ATTTACATGT CTTTGTGAAG GGAAAGATAG ATGTGGAAAA TAGAGAAGCA   
  
  
+ AGGGCGCAGT TAAGGTTGTG GAGAGAAGAC AAAGCTGAAA GGGTCAAATG TGTGGAAAGG AGGGAAGAGT   
  
  
+ AATGGAAGGC TCAATTAGTT TGGTGGTTCT TTGTCTAATA ATAGGAGAAC CATATCACTA TTTTGTTTTA   
  
  
+ TTTTGATATC TTATGTATAA TGATGTTTGT AACAAAATAT TTTCTCTTTT GATTTTTATT TTTACAACGG   
  
  
+ ATAGGATAAT TGTCTAAACG ATATTAGGGA GCAAGATTTG AACTCAAGAT GCCGTAAGTT GAAGGAAAGA   
  
  
+ CCTATCCACA AAGCAATACA CCGCTTGCTA ATAAAAACAC GCTTGTTAAA CATATAATAA TTATGTTGCC   
  
  
+ ACTCATCTTA CGATTTTAAC GGTAATATTT TTGTTCACTT TTAAGGGCAA AGACAAAAAC TTTCAATGGG   
  
  
+ ATAGCTAAAC ATGTTATACA TTTAGCATAA GCAACCCTAA TTAAGCACTA GCAACCTTAT ACATGTTATG   
  
  
+ CATTTAAGGC AGGTCTTATT GGCAATGGTG AGCTAGAGGG GGGCACGGGC ACGGTATTGT GCATAATGAA   
  
  
+ GAAATTTTTT TTGTTTTTTT AAGATTAAAA AAAAATTCAT TTATAAATCA AGAGGTTGGG ACTAGTATTT   
  
  
+ AAGACAACCA CAGTTTGACC CTCGATATAA ATTTATGTAA ATATATTTAT TTAATACAAT TATCATTTAA   
  
  
+ ACGTCGAGAT AGCGATTGTA TCAAATACAT TTAAGTATTG TAATCAAATG ACAAAAAGTT CACCGTGAAC   
  
  
+ AATAGGAACC AAATTAAACA TAAATACTAA GACGTCTTAT ACAATGGTAG TACATGGCAA TTCCAAATAA   
  
  
+ ATTGTCTTCC ACCCCCAGAA AAAAAATTAT GCTTTTAAAA AAAGATTTTG AGAAATTTCA TTCTTATACA   
  
  
+ GTTAAATTTA TCTAAAGGAA TAACTTCTTT AAAGTCATAA AACATTCCGG TCACAACTGG GACCATTTAC   
  
  
+ GTATGGTAAA AAACTCCGCA TTTTAACGAG TCATAACTTT TCTTGATCTC ATCTTAAGGA AACACCGACT   
  
  
+ TTTTTTCATG CCTGCGTCAT CATACATGTT GCCCGAGGAC ACAGCCCATT GACGACTCTA GGACAATTAG   
  
  
+ GTACATGAGA AGGCCTTCGA GGAGTTAGCG GGAGCAATAC TGGTGGATTC AACAAGTATT CTTTCATCTT   
  
  
+ GTCAAAGGCA TGCTGACATT CATCTGTCCA TTCGGTGGAA ACATTCTTCT TCAGCAGCTT GAATAATGGT   
  
  
+ TCACATGTGG CAGTTAGTTG AGATAGTTCA CGTAGTAATA ATTGTCAATG ATTACAAAAA TATGCAGTAT   
  
  
+ CACAAGTAAA AATTTAAGAT GAACTAAAAT ATCTCTTGTT TGAGAAATGT TTCAGCTTTT AGAGTTGATG   
  
  
+ TCACGTCTTT GAGCTTGCAT GTATGTAAT  

- CTTTGAAAAA ATGGAGACTT TAAATGTACA GAAACACTTC CCTTTCTATC TACACCTTTT ATCTCTTCGT   
  
  
- TCCCGCGTCA ATTCCAACAC CTCTCTTCTG TTTCGACTTT CCCAGTTTAC ACACCTTTCC TCCCTTCTCA   
  
  
- TTACCTTCCG AGTTAATCAA ACCACCAAGA AACAGATTAT TATCCTCTTG GTATAGTGAT AAAACAAAAT   
  
  
- AAAACTATAG AATACATATT ACTACAAACA TTGTTTTATA AAAGAGAAAA CTAAAAATAA AAATGTTGCC   
  
  
- TATCCTATTA ACAGATTTGC TATAATCCCT CGTTCTAAAC TTGAGTTCTA CGGCATTCAA CTTCCTTTCT   
  
  
- GGATAGGTGT TTCGTTATGT GGCGAACGAT TATTTTTGTG CGAACAATTT GTATATTATT AATACAACGG   
  
  
- TGAGTAGAAT GCTAAAATTG CCATTATAAA AACAAGTGAA AATTCCCGTT TCTGTTTTTG AAAGTTACCC   
  
  
- TATCGATTTG TACAATATGT AAATCGTATT CGTTGGGATT AATTCGTGAT CGTTGGAATA TGTACAATAC   
  
  
- GTAAATTCCG TCCAGAATAA CCGTTACCAC TCGATCTCCC CCCGTGCCCG TGCCATAACA CGTATTACTT   
  
  
- CTTTAAAAAA AACAAAAAAA TTCTAATTTT TTTTTAAGTA AATATTTAGT TCTCCAACCC TGATCATAAA   
  
  
- TTCTGTTGGT GTCAAACTGG GAGCTATATT TAAATACATT TATATAAATA AATTATGTTA ATAGTAAATT   
  
  
- TGCAGCTCTA TCGCTAACAT AGTTTATGTA AATTCATAAC ATTAGTTTAC TGTTTTTCAA GTGGCACTTG   
  
  
- TTATCCTTGG TTTAATTTGT ATTTATGATT CTGCAGAATA TGTTACCATC ATGTACCGTT AAGGTTTATT   
  
  
- TAACAGAAGG TGGGGGTCTT TTTTTTAATA CGAAAATTTT TTTCTAAAAC TCTTTAAAGT AAGAATATGT   
  
  
- CAATTTAAAT AGATTTCCTT ATTGAAGAAA TTTCAGTATT TTGTAAGGCC AGTGTTGACC CTGGTAAATG   
  
  
- CATACCATTT TTTGAGGCGT AAAATTGCTC AGTATTGAAA AGAACTAGAG TAGAATTCCT TTGTGGCTGA   
  
  
- AAAAAAGTAC GGACGCAGTA GTATGTACAA CGGGCTCCTG TGTCGGGTAA CTGCTGAGAT CCTGTTAATC   
  
  
- CATGTACTCT TCCGGAAGCT CCTCAATCGC CCTCGTTATG ACCACCTAAG TTGTTCATAA GAAAGTAGAA   
  
  
- CAGTTTCCGT ACGACTGTAA GTAGACAGGT AAGCCACCTT TGTAAGAAGA AGTCGTCGAA CTTATTACCA   
  
  
- AGTGTACACC GTCAATCAAC TCTATCAAGT GCATCATTAT TAACAGTTAC TAATGTTTTT ATACGTCATA   
  
  
- GTGTTCATTT TTAAATTCTA CTTGATTTTA TAGAGAACAA ACTCTTTACA AAGTCGAAAA TCTCAACTAC   
  
  
- AGTGCAGAAA CTCGAACGTA CATACATTA

+     TGACG-motif

| Site Name | Organism | Position | Strand | Matrix score. | sequence | function |
| --- | --- | --- | --- | --- | --- | --- |
| TGACG-motif | Hordeum vulgare | 1135 | - | 5 | TGACG | cis-acting regulatory element involved in the MeJA-responsiveness |
| TGACG-motif | Hordeum vulgare | 1170 | + | 5 | TGACG | cis-acting regulatory element involved in the MeJA-responsiveness |

>033955   
+ GAAACTTTTT TACCTCTGAA ATTTACATGT CTTTGTGAAG GGAAAGATAG ATGTGGAAAA TAGAGAAGCA   
  
  
+ AGGGCGCAGT TAAGGTTGTG GAGAGAAGAC AAAGCTGAAA GGGTCAAATG TGTGGAAAGG AGGGAAGAGT   
  
  
+ AATGGAAGGC TCAATTAGTT TGGTGGTTCT TTGTCTAATA ATAGGAGAAC CATATCACTA TTTTGTTTTA   
  
  
+ TTTTGATATC TTATGTATAA TGATGTTTGT AACAAAATAT TTTCTCTTTT GATTTTTATT TTTACAACGG   
  
  
+ ATAGGATAAT TGTCTAAACG ATATTAGGGA GCAAGATTTG AACTCAAGAT GCCGTAAGTT GAAGGAAAGA   
  
  
+ CCTATCCACA AAGCAATACA CCGCTTGCTA ATAAAAACAC GCTTGTTAAA CATATAATAA TTATGTTGCC   
  
  
+ ACTCATCTTA CGATTTTAAC GGTAATATTT TTGTTCACTT TTAAGGGCAA AGACAAAAAC TTTCAATGGG   
  
  
+ ATAGCTAAAC ATGTTATACA TTTAGCATAA GCAACCCTAA TTAAGCACTA GCAACCTTAT ACATGTTATG   
  
  
+ CATTTAAGGC AGGTCTTATT GGCAATGGTG AGCTAGAGGG GGGCACGGGC ACGGTATTGT GCATAATGAA   
  
  
+ GAAATTTTTT TTGTTTTTTT AAGATTAAAA AAAAATTCAT TTATAAATCA AGAGGTTGGG ACTAGTATTT   
  
  
+ AAGACAACCA CAGTTTGACC CTCGATATAA ATTTATGTAA ATATATTTAT TTAATACAAT TATCATTTAA   
  
  
+ ACGTCGAGAT AGCGATTGTA TCAAATACAT TTAAGTATTG TAATCAAATG ACAAAAAGTT CACCGTGAAC   
  
  
+ AATAGGAACC AAATTAAACA TAAATACTAA GACGTCTTAT ACAATGGTAG TACATGGCAA TTCCAAATAA   
  
  
+ ATTGTCTTCC ACCCCCAGAA AAAAAATTAT GCTTTTAAAA AAAGATTTTG AGAAATTTCA TTCTTATACA   
  
  
+ GTTAAATTTA TCTAAAGGAA TAACTTCTTT AAAGTCATAA AACATTCCGG TCACAACTGG GACCATTTAC   
  
  
+ GTATGGTAAA AAACTCCGCA TTTTAACGAG TCATAACTTT TCTTGATCTC ATCTTAAGGA AACACCGACT   
  
  
+ TTTTTTCATG CCTGCGTCAT CATACATGTT GCCCGAGGAC ACAGCCCATT GACGACTCTA GGACAATTAG   
  
  
+ GTACATGAGA AGGCCTTCGA GGAGTTAGCG GGAGCAATAC TGGTGGATTC AACAAGTATT CTTTCATCTT   
  
  
+ GTCAAAGGCA TGCTGACATT CATCTGTCCA TTCGGTGGAA ACATTCTTCT TCAGCAGCTT GAATAATGGT   
  
  
+ TCACATGTGG CAGTTAGTTG AGATAGTTCA CGTAGTAATA ATTGTCAATG ATTACAAAAA TATGCAGTAT   
  
  
+ CACAAGTAAA AATTTAAGAT GAACTAAAAT ATCTCTTGTT TGAGAAATGT TTCAGCTTTT AGAGTTGATG   
  
  
+ TCACGTCTTT GAGCTTGCAT GTATGTAAT  

- CTTTGAAAAA ATGGAGACTT TAAATGTACA GAAACACTTC CCTTTCTATC TACACCTTTT ATCTCTTCGT   
  
  
- TCCCGCGTCA ATTCCAACAC CTCTCTTCTG TTTCGACTTT CCCAGTTTAC ACACCTTTCC TCCCTTCTCA   
  
  
- TTACCTTCCG AGTTAATCAA ACCACCAAGA AACAGATTAT TATCCTCTTG GTATAGTGAT AAAACAAAAT   
  
  
- AAAACTATAG AATACATATT ACTACAAACA TTGTTTTATA AAAGAGAAAA CTAAAAATAA AAATGTTGCC   
  
  
- TATCCTATTA ACAGATTTGC TATAATCCCT CGTTCTAAAC TTGAGTTCTA CGGCATTCAA CTTCCTTTCT   
  
  
- GGATAGGTGT TTCGTTATGT GGCGAACGAT TATTTTTGTG CGAACAATTT GTATATTATT AATACAACGG   
  
  
- TGAGTAGAAT GCTAAAATTG CCATTATAAA AACAAGTGAA AATTCCCGTT TCTGTTTTTG AAAGTTACCC   
  
  
- TATCGATTTG TACAATATGT AAATCGTATT CGTTGGGATT AATTCGTGAT CGTTGGAATA TGTACAATAC   
  
  
- GTAAATTCCG TCCAGAATAA CCGTTACCAC TCGATCTCCC CCCGTGCCCG TGCCATAACA CGTATTACTT   
  
  
- CTTTAAAAAA AACAAAAAAA TTCTAATTTT TTTTTAAGTA AATATTTAGT TCTCCAACCC TGATCATAAA   
  
  
- TTCTGTTGGT GTCAAACTGG GAGCTATATT TAAATACATT TATATAAATA AATTATGTTA ATAGTAAATT   
  
  
- TGCAGCTCTA TCGCTAACAT AGTTTATGTA AATTCATAAC ATTAGTTTAC TGTTTTTCAA GTGGCACTTG   
  
  
- TTATCCTTGG TTTAATTTGT ATTTATGATT CTGCAGAATA TGTTACCATC ATGTACCGTT AAGGTTTATT   
  
  
- TAACAGAAGG TGGGGGTCTT TTTTTTAATA CGAAAATTTT TTTCTAAAAC TCTTTAAAGT AAGAATATGT   
  
  
- CAATTTAAAT AGATTTCCTT ATTGAAGAAA TTTCAGTATT TTGTAAGGCC AGTGTTGACC CTGGTAAATG   
  
  
- CATACCATTT TTTGAGGCGT AAAATTGCTC AGTATTGAAA AGAACTAGAG TAGAATTCCT TTGTGGCTGA   
  
  
- AAAAAAGTAC GGACGCAGTA GTATGTACAA CGGGCTCCTG TGTCGGGTAA CTGCTGAGAT CCTGTTAATC   
  
  
- CATGTACTCT TCCGGAAGCT CCTCAATCGC CCTCGTTATG ACCACCTAAG TTGTTCATAA GAAAGTAGAA   
  
  
- CAGTTTCCGT ACGACTGTAA GTAGACAGGT AAGCCACCTT TGTAAGAAGA AGTCGTCGAA CTTATTACCA   
  
  
- AGTGTACACC GTCAATCAAC TCTATCAAGT GCATCATTAT TAACAGTTAC TAATGTTTTT ATACGTCATA   
  
  
- GTGTTCATTT TTAAATTCTA CTTGATTTTA TAGAGAACAA ACTCTTTACA AAGTCGAAAA TCTCAACTAC   
  
  
- AGTGCAGAAA CTCGAACGTA CATACATTA

+     Unnamed\_\_4

| Site Name | Organism | Position | Strand | Matrix score. | sequence | function |
| --- | --- | --- | --- | --- | --- | --- |
| Unnamed\_\_4 | Petroselinum hortense | 1221 | - | 4 | CTCC |  |
| Unnamed\_\_4 | Petroselinum hortense | 1211 | - | 4 | CTCC |  |
| Unnamed\_\_4 | Petroselinum hortense | 1064 | + | 4 | CTCC |  |
| Unnamed\_\_4 | Petroselinum hortense | 308 | - | 4 | CTCC |  |
| Unnamed\_\_4 | Petroselinum hortense | 184 | - | 4 | CTCC |  |
| Unnamed\_\_4 | Petroselinum hortense | 90 | - | 4 | CTCC |  |
| Unnamed\_\_4 | Petroselinum hortense | 129 | - | 4 | CTCC |  |

>033955   
+ GAAACTTTTT TACCTCTGAA ATTTACATGT CTTTGTGAAG GGAAAGATAG ATGTGGAAAA TAGAGAAGCA   
  
  
+ AGGGCGCAGT TAAGGTTGTG GAGAGAAGAC AAAGCTGAAA GGGTCAAATG TGTGGAAAGG AGGGAAGAGT   
  
  
+ AATGGAAGGC TCAATTAGTT TGGTGGTTCT TTGTCTAATA ATAGGAGAAC CATATCACTA TTTTGTTTTA   
  
  
+ TTTTGATATC TTATGTATAA TGATGTTTGT AACAAAATAT TTTCTCTTTT GATTTTTATT TTTACAACGG   
  
  
+ ATAGGATAAT TGTCTAAACG ATATTAGGGA GCAAGATTTG AACTCAAGAT GCCGTAAGTT GAAGGAAAGA   
  
  
+ CCTATCCACA AAGCAATACA CCGCTTGCTA ATAAAAACAC GCTTGTTAAA CATATAATAA TTATGTTGCC   
  
  
+ ACTCATCTTA CGATTTTAAC GGTAATATTT TTGTTCACTT TTAAGGGCAA AGACAAAAAC TTTCAATGGG   
  
  
+ ATAGCTAAAC ATGTTATACA TTTAGCATAA GCAACCCTAA TTAAGCACTA GCAACCTTAT ACATGTTATG   
  
  
+ CATTTAAGGC AGGTCTTATT GGCAATGGTG AGCTAGAGGG GGGCACGGGC ACGGTATTGT GCATAATGAA   
  
  
+ GAAATTTTTT TTGTTTTTTT AAGATTAAAA AAAAATTCAT TTATAAATCA AGAGGTTGGG ACTAGTATTT   
  
  
+ AAGACAACCA CAGTTTGACC CTCGATATAA ATTTATGTAA ATATATTTAT TTAATACAAT TATCATTTAA   
  
  
+ ACGTCGAGAT AGCGATTGTA TCAAATACAT TTAAGTATTG TAATCAAATG ACAAAAAGTT CACCGTGAAC   
  
  
+ AATAGGAACC AAATTAAACA TAAATACTAA GACGTCTTAT ACAATGGTAG TACATGGCAA TTCCAAATAA   
  
  
+ ATTGTCTTCC ACCCCCAGAA AAAAAATTAT GCTTTTAAAA AAAGATTTTG AGAAATTTCA TTCTTATACA   
  
  
+ GTTAAATTTA TCTAAAGGAA TAACTTCTTT AAAGTCATAA AACATTCCGG TCACAACTGG GACCATTTAC   
  
  
+ GTATGGTAAA AAACTCCGCA TTTTAACGAG TCATAACTTT TCTTGATCTC ATCTTAAGGA AACACCGACT   
  
  
+ TTTTTTCATG CCTGCGTCAT CATACATGTT GCCCGAGGAC ACAGCCCATT GACGACTCTA GGACAATTAG   
  
  
+ GTACATGAGA AGGCCTTCGA GGAGTTAGCG GGAGCAATAC TGGTGGATTC AACAAGTATT CTTTCATCTT   
  
  
+ GTCAAAGGCA TGCTGACATT CATCTGTCCA TTCGGTGGAA ACATTCTTCT TCAGCAGCTT GAATAATGGT   
  
  
+ TCACATGTGG CAGTTAGTTG AGATAGTTCA CGTAGTAATA ATTGTCAATG ATTACAAAAA TATGCAGTAT   
  
  
+ CACAAGTAAA AATTTAAGAT GAACTAAAAT ATCTCTTGTT TGAGAAATGT TTCAGCTTTT AGAGTTGATG   
  
  
+ TCACGTCTTT GAGCTTGCAT GTATGTAAT  

- CTTTGAAAAA ATGGAGACTT TAAATGTACA GAAACACTTC CCTTTCTATC TACACCTTTT ATCTCTTCGT   
  
  
- TCCCGCGTCA ATTCCAACAC CTCTCTTCTG TTTCGACTTT CCCAGTTTAC ACACCTTTCC TCCCTTCTCA   
  
  
- TTACCTTCCG AGTTAATCAA ACCACCAAGA AACAGATTAT TATCCTCTTG GTATAGTGAT AAAACAAAAT   
  
  
- AAAACTATAG AATACATATT ACTACAAACA TTGTTTTATA AAAGAGAAAA CTAAAAATAA AAATGTTGCC   
  
  
- TATCCTATTA ACAGATTTGC TATAATCCCT CGTTCTAAAC TTGAGTTCTA CGGCATTCAA CTTCCTTTCT   
  
  
- GGATAGGTGT TTCGTTATGT GGCGAACGAT TATTTTTGTG CGAACAATTT GTATATTATT AATACAACGG   
  
  
- TGAGTAGAAT GCTAAAATTG CCATTATAAA AACAAGTGAA AATTCCCGTT TCTGTTTTTG AAAGTTACCC   
  
  
- TATCGATTTG TACAATATGT AAATCGTATT CGTTGGGATT AATTCGTGAT CGTTGGAATA TGTACAATAC   
  
  
- GTAAATTCCG TCCAGAATAA CCGTTACCAC TCGATCTCCC CCCGTGCCCG TGCCATAACA CGTATTACTT   
  
  
- CTTTAAAAAA AACAAAAAAA TTCTAATTTT TTTTTAAGTA AATATTTAGT TCTCCAACCC TGATCATAAA   
  
  
- TTCTGTTGGT GTCAAACTGG GAGCTATATT TAAATACATT TATATAAATA AATTATGTTA ATAGTAAATT   
  
  
- TGCAGCTCTA TCGCTAACAT AGTTTATGTA AATTCATAAC ATTAGTTTAC TGTTTTTCAA GTGGCACTTG   
  
  
- TTATCCTTGG TTTAATTTGT ATTTATGATT CTGCAGAATA TGTTACCATC ATGTACCGTT AAGGTTTATT   
  
  
- TAACAGAAGG TGGGGGTCTT TTTTTTAATA CGAAAATTTT TTTCTAAAAC TCTTTAAAGT AAGAATATGT   
  
  
- CAATTTAAAT AGATTTCCTT ATTGAAGAAA TTTCAGTATT TTGTAAGGCC AGTGTTGACC CTGGTAAATG   
  
  
- CATACCATTT TTTGAGGCGT AAAATTGCTC AGTATTGAAA AGAACTAGAG TAGAATTCCT TTGTGGCTGA   
  
  
- AAAAAAGTAC GGACGCAGTA GTATGTACAA CGGGCTCCTG TGTCGGGTAA CTGCTGAGAT CCTGTTAATC   
  
  
- CATGTACTCT TCCGGAAGCT CCTCAATCGC CCTCGTTATG ACCACCTAAG TTGTTCATAA GAAAGTAGAA   
  
  
- CAGTTTCCGT ACGACTGTAA GTAGACAGGT AAGCCACCTT TGTAAGAAGA AGTCGTCGAA CTTATTACCA   
  
  
- AGTGTACACC GTCAATCAAC TCTATCAAGT GCATCATTAT TAACAGTTAC TAATGTTTTT ATACGTCATA   
  
  
- GTGTTCATTT TTAAATTCTA CTTGATTTTA TAGAGAACAA ACTCTTTACA AAGTCGAAAA TCTCAACTAC   
  
  
- AGTGCAGAAA CTCGAACGTA CATACATTA

+     W box

| Site Name | Organism | Position | Strand | Matrix score. | sequence | function |
| --- | --- | --- | --- | --- | --- | --- |
| W box | Arabidopsis thaliana | 112 | - | 6 | TTGACC |  |
| W box | Arabidopsis thaliana | 715 | + | 6 | TTGACC |  |

>033955   
+ GAAACTTTTT TACCTCTGAA ATTTACATGT CTTTGTGAAG GGAAAGATAG ATGTGGAAAA TAGAGAAGCA   
  
  
+ AGGGCGCAGT TAAGGTTGTG GAGAGAAGAC AAAGCTGAAA GGGTCAAATG TGTGGAAAGG AGGGAAGAGT   
  
  
+ AATGGAAGGC TCAATTAGTT TGGTGGTTCT TTGTCTAATA ATAGGAGAAC CATATCACTA TTTTGTTTTA   
  
  
+ TTTTGATATC TTATGTATAA TGATGTTTGT AACAAAATAT TTTCTCTTTT GATTTTTATT TTTACAACGG   
  
  
+ ATAGGATAAT TGTCTAAACG ATATTAGGGA GCAAGATTTG AACTCAAGAT GCCGTAAGTT GAAGGAAAGA   
  
  
+ CCTATCCACA AAGCAATACA CCGCTTGCTA ATAAAAACAC GCTTGTTAAA CATATAATAA TTATGTTGCC   
  
  
+ ACTCATCTTA CGATTTTAAC GGTAATATTT TTGTTCACTT TTAAGGGCAA AGACAAAAAC TTTCAATGGG   
  
  
+ ATAGCTAAAC ATGTTATACA TTTAGCATAA GCAACCCTAA TTAAGCACTA GCAACCTTAT ACATGTTATG   
  
  
+ CATTTAAGGC AGGTCTTATT GGCAATGGTG AGCTAGAGGG GGGCACGGGC ACGGTATTGT GCATAATGAA   
  
  
+ GAAATTTTTT TTGTTTTTTT AAGATTAAAA AAAAATTCAT TTATAAATCA AGAGGTTGGG ACTAGTATTT   
  
  
+ AAGACAACCA CAGTTTGACC CTCGATATAA ATTTATGTAA ATATATTTAT TTAATACAAT TATCATTTAA   
  
  
+ ACGTCGAGAT AGCGATTGTA TCAAATACAT TTAAGTATTG TAATCAAATG ACAAAAAGTT CACCGTGAAC   
  
  
+ AATAGGAACC AAATTAAACA TAAATACTAA GACGTCTTAT ACAATGGTAG TACATGGCAA TTCCAAATAA   
  
  
+ ATTGTCTTCC ACCCCCAGAA AAAAAATTAT GCTTTTAAAA AAAGATTTTG AGAAATTTCA TTCTTATACA   
  
  
+ GTTAAATTTA TCTAAAGGAA TAACTTCTTT AAAGTCATAA AACATTCCGG TCACAACTGG GACCATTTAC   
  
  
+ GTATGGTAAA AAACTCCGCA TTTTAACGAG TCATAACTTT TCTTGATCTC ATCTTAAGGA AACACCGACT   
  
  
+ TTTTTTCATG CCTGCGTCAT CATACATGTT GCCCGAGGAC ACAGCCCATT GACGACTCTA GGACAATTAG   
  
  
+ GTACATGAGA AGGCCTTCGA GGAGTTAGCG GGAGCAATAC TGGTGGATTC AACAAGTATT CTTTCATCTT   
  
  
+ GTCAAAGGCA TGCTGACATT CATCTGTCCA TTCGGTGGAA ACATTCTTCT TCAGCAGCTT GAATAATGGT   
  
  
+ TCACATGTGG CAGTTAGTTG AGATAGTTCA CGTAGTAATA ATTGTCAATG ATTACAAAAA TATGCAGTAT   
  
  
+ CACAAGTAAA AATTTAAGAT GAACTAAAAT ATCTCTTGTT TGAGAAATGT TTCAGCTTTT AGAGTTGATG   
  
  
+ TCACGTCTTT GAGCTTGCAT GTATGTAAT  

- CTTTGAAAAA ATGGAGACTT TAAATGTACA GAAACACTTC CCTTTCTATC TACACCTTTT ATCTCTTCGT   
  
  
- TCCCGCGTCA ATTCCAACAC CTCTCTTCTG TTTCGACTTT CCCAGTTTAC ACACCTTTCC TCCCTTCTCA   
  
  
- TTACCTTCCG AGTTAATCAA ACCACCAAGA AACAGATTAT TATCCTCTTG GTATAGTGAT AAAACAAAAT   
  
  
- AAAACTATAG AATACATATT ACTACAAACA TTGTTTTATA AAAGAGAAAA CTAAAAATAA AAATGTTGCC   
  
  
- TATCCTATTA ACAGATTTGC TATAATCCCT CGTTCTAAAC TTGAGTTCTA CGGCATTCAA CTTCCTTTCT   
  
  
- GGATAGGTGT TTCGTTATGT GGCGAACGAT TATTTTTGTG CGAACAATTT GTATATTATT AATACAACGG   
  
  
- TGAGTAGAAT GCTAAAATTG CCATTATAAA AACAAGTGAA AATTCCCGTT TCTGTTTTTG AAAGTTACCC   
  
  
- TATCGATTTG TACAATATGT AAATCGTATT CGTTGGGATT AATTCGTGAT CGTTGGAATA TGTACAATAC   
  
  
- GTAAATTCCG TCCAGAATAA CCGTTACCAC TCGATCTCCC CCCGTGCCCG TGCCATAACA CGTATTACTT   
  
  
- CTTTAAAAAA AACAAAAAAA TTCTAATTTT TTTTTAAGTA AATATTTAGT TCTCCAACCC TGATCATAAA   
  
  
- TTCTGTTGGT GTCAAACTGG GAGCTATATT TAAATACATT TATATAAATA AATTATGTTA ATAGTAAATT   
  
  
- TGCAGCTCTA TCGCTAACAT AGTTTATGTA AATTCATAAC ATTAGTTTAC TGTTTTTCAA GTGGCACTTG   
  
  
- TTATCCTTGG TTTAATTTGT ATTTATGATT CTGCAGAATA TGTTACCATC ATGTACCGTT AAGGTTTATT   
  
  
- TAACAGAAGG TGGGGGTCTT TTTTTTAATA CGAAAATTTT TTTCTAAAAC TCTTTAAAGT AAGAATATGT   
  
  
- CAATTTAAAT AGATTTCCTT ATTGAAGAAA TTTCAGTATT TTGTAAGGCC AGTGTTGACC CTGGTAAATG   
  
  
- CATACCATTT TTTGAGGCGT AAAATTGCTC AGTATTGAAA AGAACTAGAG TAGAATTCCT TTGTGGCTGA   
  
  
- AAAAAAGTAC GGACGCAGTA GTATGTACAA CGGGCTCCTG TGTCGGGTAA CTGCTGAGAT CCTGTTAATC   
  
  
- CATGTACTCT TCCGGAAGCT CCTCAATCGC CCTCGTTATG ACCACCTAAG TTGTTCATAA GAAAGTAGAA   
  
  
- CAGTTTCCGT ACGACTGTAA GTAGACAGGT AAGCCACCTT TGTAAGAAGA AGTCGTCGAA CTTATTACCA   
  
  
- AGTGTACACC GTCAATCAAC TCTATCAAGT GCATCATTAT TAACAGTTAC TAATGTTTTT ATACGTCATA   
  
  
- GTGTTCATTT TTAAATTCTA CTTGATTTTA TAGAGAACAA ACTCTTTACA AAGTCGAAAA TCTCAACTAC   
  
  
- AGTGCAGAAA CTCGAACGTA CATACATTA

+     WUN-motif

| Site Name | Organism | Position | Strand | Matrix score. | sequence | function |
| --- | --- | --- | --- | --- | --- | --- |
| WUN-motif | Nicotiana glutinosa | 629 | - | 9 | AAATTTCTT |  |

>033955   
+ GAAACTTTTT TACCTCTGAA ATTTACATGT CTTTGTGAAG GGAAAGATAG ATGTGGAAAA TAGAGAAGCA   
  
  
+ AGGGCGCAGT TAAGGTTGTG GAGAGAAGAC AAAGCTGAAA GGGTCAAATG TGTGGAAAGG AGGGAAGAGT   
  
  
+ AATGGAAGGC TCAATTAGTT TGGTGGTTCT TTGTCTAATA ATAGGAGAAC CATATCACTA TTTTGTTTTA   
  
  
+ TTTTGATATC TTATGTATAA TGATGTTTGT AACAAAATAT TTTCTCTTTT GATTTTTATT TTTACAACGG   
  
  
+ ATAGGATAAT TGTCTAAACG ATATTAGGGA GCAAGATTTG AACTCAAGAT GCCGTAAGTT GAAGGAAAGA   
  
  
+ CCTATCCACA AAGCAATACA CCGCTTGCTA ATAAAAACAC GCTTGTTAAA CATATAATAA TTATGTTGCC   
  
  
+ ACTCATCTTA CGATTTTAAC GGTAATATTT TTGTTCACTT TTAAGGGCAA AGACAAAAAC TTTCAATGGG   
  
  
+ ATAGCTAAAC ATGTTATACA TTTAGCATAA GCAACCCTAA TTAAGCACTA GCAACCTTAT ACATGTTATG   
  
  
+ CATTTAAGGC AGGTCTTATT GGCAATGGTG AGCTAGAGGG GGGCACGGGC ACGGTATTGT GCATAATGAA   
  
  
+ GAAATTTTTT TTGTTTTTTT AAGATTAAAA AAAAATTCAT TTATAAATCA AGAGGTTGGG ACTAGTATTT   
  
  
+ AAGACAACCA CAGTTTGACC CTCGATATAA ATTTATGTAA ATATATTTAT TTAATACAAT TATCATTTAA   
  
  
+ ACGTCGAGAT AGCGATTGTA TCAAATACAT TTAAGTATTG TAATCAAATG ACAAAAAGTT CACCGTGAAC   
  
  
+ AATAGGAACC AAATTAAACA TAAATACTAA GACGTCTTAT ACAATGGTAG TACATGGCAA TTCCAAATAA   
  
  
+ ATTGTCTTCC ACCCCCAGAA AAAAAATTAT GCTTTTAAAA AAAGATTTTG AGAAATTTCA TTCTTATACA   
  
  
+ GTTAAATTTA TCTAAAGGAA TAACTTCTTT AAAGTCATAA AACATTCCGG TCACAACTGG GACCATTTAC   
  
  
+ GTATGGTAAA AAACTCCGCA TTTTAACGAG TCATAACTTT TCTTGATCTC ATCTTAAGGA AACACCGACT   
  
  
+ TTTTTTCATG CCTGCGTCAT CATACATGTT GCCCGAGGAC ACAGCCCATT GACGACTCTA GGACAATTAG   
  
  
+ GTACATGAGA AGGCCTTCGA GGAGTTAGCG GGAGCAATAC TGGTGGATTC AACAAGTATT CTTTCATCTT   
  
  
+ GTCAAAGGCA TGCTGACATT CATCTGTCCA TTCGGTGGAA ACATTCTTCT TCAGCAGCTT GAATAATGGT   
  
  
+ TCACATGTGG CAGTTAGTTG AGATAGTTCA CGTAGTAATA ATTGTCAATG ATTACAAAAA TATGCAGTAT   
  
  
+ CACAAGTAAA AATTTAAGAT GAACTAAAAT ATCTCTTGTT TGAGAAATGT TTCAGCTTTT AGAGTTGATG   
  
  
+ TCACGTCTTT GAGCTTGCAT GTATGTAAT  

- CTTTGAAAAA ATGGAGACTT TAAATGTACA GAAACACTTC CCTTTCTATC TACACCTTTT ATCTCTTCGT   
  
  
- TCCCGCGTCA ATTCCAACAC CTCTCTTCTG TTTCGACTTT CCCAGTTTAC ACACCTTTCC TCCCTTCTCA   
  
  
- TTACCTTCCG AGTTAATCAA ACCACCAAGA AACAGATTAT TATCCTCTTG GTATAGTGAT AAAACAAAAT   
  
  
- AAAACTATAG AATACATATT ACTACAAACA TTGTTTTATA AAAGAGAAAA CTAAAAATAA AAATGTTGCC   
  
  
- TATCCTATTA ACAGATTTGC TATAATCCCT CGTTCTAAAC TTGAGTTCTA CGGCATTCAA CTTCCTTTCT   
  
  
- GGATAGGTGT TTCGTTATGT GGCGAACGAT TATTTTTGTG CGAACAATTT GTATATTATT AATACAACGG   
  
  
- TGAGTAGAAT GCTAAAATTG CCATTATAAA AACAAGTGAA AATTCCCGTT TCTGTTTTTG AAAGTTACCC   
  
  
- TATCGATTTG TACAATATGT AAATCGTATT CGTTGGGATT AATTCGTGAT CGTTGGAATA TGTACAATAC   
  
  
- GTAAATTCCG TCCAGAATAA CCGTTACCAC TCGATCTCCC CCCGTGCCCG TGCCATAACA CGTATTACTT   
  
  
- CTTTAAAAAA AACAAAAAAA TTCTAATTTT TTTTTAAGTA AATATTTAGT TCTCCAACCC TGATCATAAA   
  
  
- TTCTGTTGGT GTCAAACTGG GAGCTATATT TAAATACATT TATATAAATA AATTATGTTA ATAGTAAATT   
  
  
- TGCAGCTCTA TCGCTAACAT AGTTTATGTA AATTCATAAC ATTAGTTTAC TGTTTTTCAA GTGGCACTTG   
  
  
- TTATCCTTGG TTTAATTTGT ATTTATGATT CTGCAGAATA TGTTACCATC ATGTACCGTT AAGGTTTATT   
  
  
- TAACAGAAGG TGGGGGTCTT TTTTTTAATA CGAAAATTTT TTTCTAAAAC TCTTTAAAGT AAGAATATGT   
  
  
- CAATTTAAAT AGATTTCCTT ATTGAAGAAA TTTCAGTATT TTGTAAGGCC AGTGTTGACC CTGGTAAATG   
  
  
- CATACCATTT TTTGAGGCGT AAAATTGCTC AGTATTGAAA AGAACTAGAG TAGAATTCCT TTGTGGCTGA   
  
  
- AAAAAAGTAC GGACGCAGTA GTATGTACAA CGGGCTCCTG TGTCGGGTAA CTGCTGAGAT CCTGTTAATC   
  
  
- CATGTACTCT TCCGGAAGCT CCTCAATCGC CCTCGTTATG ACCACCTAAG TTGTTCATAA GAAAGTAGAA   
  
  
- CAGTTTCCGT ACGACTGTAA GTAGACAGGT AAGCCACCTT TGTAAGAAGA AGTCGTCGAA CTTATTACCA   
  
  
- AGTGTACACC GTCAATCAAC TCTATCAAGT GCATCATTAT TAACAGTTAC TAATGTTTTT ATACGTCATA   
  
  
- GTGTTCATTT TTAAATTCTA CTTGATTTTA TAGAGAACAA ACTCTTTACA AAGTCGAAAA TCTCAACTAC   
  
  
- AGTGCAGAAA CTCGAACGTA CATACATTA

+     as-1

| Site Name | Organism | Position | Strand | Matrix score. | sequence | function |
| --- | --- | --- | --- | --- | --- | --- |
| as-1 | Arabidopsis thaliana | 1170 | + | 5 | TGACG |  |
| as-1 | Arabidopsis thaliana | 1135 | - | 5 | TGACG |  |

>033955   
+ GAAACTTTTT TACCTCTGAA ATTTACATGT CTTTGTGAAG GGAAAGATAG ATGTGGAAAA TAGAGAAGCA   
  
  
+ AGGGCGCAGT TAAGGTTGTG GAGAGAAGAC AAAGCTGAAA GGGTCAAATG TGTGGAAAGG AGGGAAGAGT   
  
  
+ AATGGAAGGC TCAATTAGTT TGGTGGTTCT TTGTCTAATA ATAGGAGAAC CATATCACTA TTTTGTTTTA   
  
  
+ TTTTGATATC TTATGTATAA TGATGTTTGT AACAAAATAT TTTCTCTTTT GATTTTTATT TTTACAACGG   
  
  
+ ATAGGATAAT TGTCTAAACG ATATTAGGGA GCAAGATTTG AACTCAAGAT GCCGTAAGTT GAAGGAAAGA   
  
  
+ CCTATCCACA AAGCAATACA CCGCTTGCTA ATAAAAACAC GCTTGTTAAA CATATAATAA TTATGTTGCC   
  
  
+ ACTCATCTTA CGATTTTAAC GGTAATATTT TTGTTCACTT TTAAGGGCAA AGACAAAAAC TTTCAATGGG   
  
  
+ ATAGCTAAAC ATGTTATACA TTTAGCATAA GCAACCCTAA TTAAGCACTA GCAACCTTAT ACATGTTATG   
  
  
+ CATTTAAGGC AGGTCTTATT GGCAATGGTG AGCTAGAGGG GGGCACGGGC ACGGTATTGT GCATAATGAA   
  
  
+ GAAATTTTTT TTGTTTTTTT AAGATTAAAA AAAAATTCAT TTATAAATCA AGAGGTTGGG ACTAGTATTT   
  
  
+ AAGACAACCA CAGTTTGACC CTCGATATAA ATTTATGTAA ATATATTTAT TTAATACAAT TATCATTTAA   
  
  
+ ACGTCGAGAT AGCGATTGTA TCAAATACAT TTAAGTATTG TAATCAAATG ACAAAAAGTT CACCGTGAAC   
  
  
+ AATAGGAACC AAATTAAACA TAAATACTAA GACGTCTTAT ACAATGGTAG TACATGGCAA TTCCAAATAA   
  
  
+ ATTGTCTTCC ACCCCCAGAA AAAAAATTAT GCTTTTAAAA AAAGATTTTG AGAAATTTCA TTCTTATACA   
  
  
+ GTTAAATTTA TCTAAAGGAA TAACTTCTTT AAAGTCATAA AACATTCCGG TCACAACTGG GACCATTTAC   
  
  
+ GTATGGTAAA AAACTCCGCA TTTTAACGAG TCATAACTTT TCTTGATCTC ATCTTAAGGA AACACCGACT   
  
  
+ TTTTTTCATG CCTGCGTCAT CATACATGTT GCCCGAGGAC ACAGCCCATT GACGACTCTA GGACAATTAG   
  
  
+ GTACATGAGA AGGCCTTCGA GGAGTTAGCG GGAGCAATAC TGGTGGATTC AACAAGTATT CTTTCATCTT   
  
  
+ GTCAAAGGCA TGCTGACATT CATCTGTCCA TTCGGTGGAA ACATTCTTCT TCAGCAGCTT GAATAATGGT   
  
  
+ TCACATGTGG CAGTTAGTTG AGATAGTTCA CGTAGTAATA ATTGTCAATG ATTACAAAAA TATGCAGTAT   
  
  
+ CACAAGTAAA AATTTAAGAT GAACTAAAAT ATCTCTTGTT TGAGAAATGT TTCAGCTTTT AGAGTTGATG   
  
  
+ TCACGTCTTT GAGCTTGCAT GTATGTAAT  

- CTTTGAAAAA ATGGAGACTT TAAATGTACA GAAACACTTC CCTTTCTATC TACACCTTTT ATCTCTTCGT   
  
  
- TCCCGCGTCA ATTCCAACAC CTCTCTTCTG TTTCGACTTT CCCAGTTTAC ACACCTTTCC TCCCTTCTCA   
  
  
- TTACCTTCCG AGTTAATCAA ACCACCAAGA AACAGATTAT TATCCTCTTG GTATAGTGAT AAAACAAAAT   
  
  
- AAAACTATAG AATACATATT ACTACAAACA TTGTTTTATA AAAGAGAAAA CTAAAAATAA AAATGTTGCC   
  
  
- TATCCTATTA ACAGATTTGC TATAATCCCT CGTTCTAAAC TTGAGTTCTA CGGCATTCAA CTTCCTTTCT   
  
  
- GGATAGGTGT TTCGTTATGT GGCGAACGAT TATTTTTGTG CGAACAATTT GTATATTATT AATACAACGG   
  
  
- TGAGTAGAAT GCTAAAATTG CCATTATAAA AACAAGTGAA AATTCCCGTT TCTGTTTTTG AAAGTTACCC   
  
  
- TATCGATTTG TACAATATGT AAATCGTATT CGTTGGGATT AATTCGTGAT CGTTGGAATA TGTACAATAC   
  
  
- GTAAATTCCG TCCAGAATAA CCGTTACCAC TCGATCTCCC CCCGTGCCCG TGCCATAACA CGTATTACTT   
  
  
- CTTTAAAAAA AACAAAAAAA TTCTAATTTT TTTTTAAGTA AATATTTAGT TCTCCAACCC TGATCATAAA   
  
  
- TTCTGTTGGT GTCAAACTGG GAGCTATATT TAAATACATT TATATAAATA AATTATGTTA ATAGTAAATT   
  
  
- TGCAGCTCTA TCGCTAACAT AGTTTATGTA AATTCATAAC ATTAGTTTAC TGTTTTTCAA GTGGCACTTG   
  
  
- TTATCCTTGG TTTAATTTGT ATTTATGATT CTGCAGAATA TGTTACCATC ATGTACCGTT AAGGTTTATT   
  
  
- TAACAGAAGG TGGGGGTCTT TTTTTTAATA CGAAAATTTT TTTCTAAAAC TCTTTAAAGT AAGAATATGT   
  
  
- CAATTTAAAT AGATTTCCTT ATTGAAGAAA TTTCAGTATT TTGTAAGGCC AGTGTTGACC CTGGTAAATG   
  
  
- CATACCATTT TTTGAGGCGT AAAATTGCTC AGTATTGAAA AGAACTAGAG TAGAATTCCT TTGTGGCTGA   
  
  
- AAAAAAGTAC GGACGCAGTA GTATGTACAA CGGGCTCCTG TGTCGGGTAA CTGCTGAGAT CCTGTTAATC   
  
  
- CATGTACTCT TCCGGAAGCT CCTCAATCGC CCTCGTTATG ACCACCTAAG TTGTTCATAA GAAAGTAGAA   
  
  
- CAGTTTCCGT ACGACTGTAA GTAGACAGGT AAGCCACCTT TGTAAGAAGA AGTCGTCGAA CTTATTACCA   
  
  
- AGTGTACACC GTCAATCAAC TCTATCAAGT GCATCATTAT TAACAGTTAC TAATGTTTTT ATACGTCATA   
  
  
- GTGTTCATTT TTAAATTCTA CTTGATTTTA TAGAGAACAA ACTCTTTACA AAGTCGAAAA TCTCAACTAC   
  
  
- AGTGCAGAAA CTCGAACGTA CATACATTA
