# Supplementary material for: In Silico Genome-Wide Analysis of Respiratory Burst Oxidase Homolog (RBOH) Family Genes in Five Fruit-Producing Trees, and Potential Functional Analysis on Lignification of Stone Cells in Chinese White Pear
Source: Cells. 2019 May 29;8(6):520. doi: 10.3390/cells8060520 (PMC6627160; doi:10.3390/cells8060520)
Supplement: Supplementary file 1 [file cells-08-00520-s001.zip › Supplementary Material/Spreadsheets S2. Numbers of cis-elements in promoter region of PbRBOHs.tar.zip!/PbRBOHE.html]

Content-Type: text/html; charset=ISO-8859-1


PlantCARE


Webmaster Firefox specific output  
To save the result:
click on the frame with the right mouse button and save the source code as a text file with extension .html  
REFERENCE:PlantCARE: a database of plant cis-acting regulatory elements and a portal to tools for in silico analysis of promoter sequences.  
Lescot, M., Déhais, P., Moreau, Y., De Moor, B., Rouzé ,P.,and Rombauts, S.  
Nucleic Acids Res., Database issue(2002), 30(1):325-327.   


---

>036006   
+ GTATACTTAA AACTAGAGAA GCCATCGATG GGCAATTGTT AGGCATAAAA ATAGTGAAAG AACCACCGGC   
  
  
+ CGGATAAACA AGAGTAAAGC TGTCTGAAAA TAAACCCTAC TCACACACAT GGTATTTCTC ATATTTTATT   
  
  
+ TTATAGTCAA TTATTAAGAT AATAAGCATT TTAACCATTT ATGTATACAT GTTCATACCA CGAGCGTTGC   
  
  
+ TTGGAGGAAC ACATTTTGGT ATCAAGTGAT GTTGCAAAAT GATGCATACA ATCAGTACAC ATCACTCTAG   
  
  
+ CTACTTACTC CATCTTGTAG TATTGCAATG GAGTAAAATA TAGCACTAGT CCCAGATTAA TTATTATGGT   
  
  
+ TCTCCTTTCA AACACATAGT TAATGGTTAT AATGATATAC TTAAGGGCAA AATATATTAC TTATACAAAA   
  
  
+ TAACTCGATG GGTTACGTAA AATACATGAA GAATTCTGTT TAATAAGTTT AGTATATGCT TGCATGAGTA   
  
  
+ TGATACAATA TATATGTGAC AAAATAAAAT ACAAGTCTAA ATTTACGACA CCACCACCCC CTTATATAGA   
  
  
+ GTTAAAACGT GAGATTCAAG AGTGTAGTTG GTGGAAAAAG ATAAATTCTA GGGTTTTTGT CTTGTAGAAA   
  
  
+ TACATCATAT ACATAAATAT TTATCTGTAC AACATGTGCG TATGTACAAT AATCATTGAC AATTAACCTA   
  
  
+ AACATTGCAA TTAACATAAA CTTTGTTTCT TCTTAAATTT TAGATGGTAA GTTCCTCCTA TCAAATCATT   
  
  
+ ACAAAATCAT AAAATCCCAA TAACACACTT TATCTATACG CAAAAAAATT TGCCGGGGAA AATATAGTAC   
  
  
+ GTGGAGGTAT ATATAGTACG TACTTTCTCC TTTGCTTTCA AAGATCATCA AATTTTAATT AGTTTATATT   
  
  
+ ATTTTATATA TATACACACA CACACTAGCA TATAGGCACA CACAAAGTGT GTGAGACATT TTTTTATTTT   
  
  
+ TGTTTTTGAA ATAGAAAGAG AGAGGAAGAG AGGAAGAGAG CGATAGGGAA TGTGAGAATC AGAAGTTTTT   
  
  
+ TTTTTTTTTT TAATTAAAGA TCTGTTAAGA TTACATGTAG TTGAAATTTT GAAAAAAAAA CAGCCAAATT   
  
  
+ TAGTTATGTG AAATTACATT TTTGTCCCGT ATTTGTTATT CATATTTTGT TTTAATTAGA GAGTTAAAGT   
  
  
+ GGTAATTTGA TAGAATTTTG GTTGACAATG AATGTTTTAT TAATTAGTAG ATATATATAT ATATATAATT   
  
  
+ GTTATAAGTA AGTGGAAAAG TATTGTTGTC GTGGAGACAA ATTTAGGTTC TTGAGCAAAA ACTCAACTAC   
  
  
+ TCTCTAGAGA GAGAGAGAGG TAGCAGTAAC ACGACTTGTG TAGAAATTGT GAGGGACGAA TCGGTTAAGA   
  
  
+ AGTGCATGTC TTGTATCTCT CTCTAGAGCT AGAAAAATCC TAAGTATTTC ATTTTTTTGG AACAAACAGA   
  
  
+ GAAAACCCCA AGAATTATAA TAATTAAAC  

- CATATGAATT TTGATCTCTT CGGTAGCTAC CCGTTAACAA TCCGTATTTT TATCACTTTC TTGGTGGCCG   
  
  
- GCCTATTTGT TCTCATTTCG ACAGACTTTT ATTTGGGATG AGTGTGTGTA CCATAAAGAG TATAAAATAA   
  
  
- AATATCAGTT AATAATTCTA TTATTCGTAA AATTGGTAAA TACATATGTA CAAGTATGGT GCTCGCAACG   
  
  
- AACCTCCTTG TGTAAAACCA TAGTTCACTA CAACGTTTTA CTACGTATGT TAGTCATGTG TAGTGAGATC   
  
  
- GATGAATGAG GTAGAACATC ATAACGTTAC CTCATTTTAT ATCGTGATCA GGGTCTAATT AATAATACCA   
  
  
- AGAGGAAAGT TTGTGTATCA ATTACCAATA TTACTATATG AATTCCCGTT TTATATAATG AATATGTTTT   
  
  
- ATTGAGCTAC CCAATGCATT TTATGTACTT CTTAAGACAA ATTATTCAAA TCATATACGA ACGTACTCAT   
  
  
- ACTATGTTAT ATATACACTG TTTTATTTTA TGTTCAGATT TAAATGCTGT GGTGGTGGGG GAATATATCT   
  
  
- CAATTTTGCA CTCTAAGTTC TCACATCAAC CACCTTTTTC TATTTAAGAT CCCAAAAACA GAACATCTTT   
  
  
- ATGTAGTATA TGTATTTATA AATAGACATG TTGTACACGC ATACATGTTA TTAGTAACTG TTAATTGGAT   
  
  
- TTGTAACGTT AATTGTATTT GAAACAAAGA AGAATTTAAA ATCTACCATT CAAGGAGGAT AGTTTAGTAA   
  
  
- TGTTTTAGTA TTTTAGGGTT ATTGTGTGAA ATAGATATGC GTTTTTTTAA ACGGCCCCTT TTATATCATG   
  
  
- CACCTCCATA TATATCATGC ATGAAAGAGG AAACGAAAGT TTCTAGTAGT TTAAAATTAA TCAAATATAA   
  
  
- TAAAATATAT ATATGTGTGT GTGTGATCGT ATATCCGTGT GTGTTTCACA CACTCTGTAA AAAAATAAAA   
  
  
- ACAAAAACTT TATCTTTCTC TCTCCTTCTC TCCTTCTCTC GCTATCCCTT ACACTCTTAG TCTTCAAAAA   
  
  
- AAAAAAAAAA ATTAATTTCT AGACAATTCT AATGTACATC AACTTTAAAA CTTTTTTTTT GTCGGTTTAA   
  
  
- ATCAATACAC TTTAATGTAA AAACAGGGCA TAAACAATAA GTATAAAACA AAATTAATCT CTCAATTTCA   
  
  
- CCATTAAACT ATCTTAAAAC CAACTGTTAC TTACAAAATA ATTAATCATC TATATATATA TATATATTAA   
  
  
- CAATATTCAT TCACCTTTTC ATAACAACAG CACCTCTGTT TAAATCCAAG AACTCGTTTT TGAGTTGATG   
  
  
- AGAGATCTCT CTCTCTCTCC ATCGTCATTG TGCTGAACAC ATCTTTAACA CTCCCTGCTT AGCCAATTCT   
  
  
- TCACGTACAG AACATAGAGA GAGATCTCGA TCTTTTTAGG ATTCATAAAG TAAAAAAACC TTGTTTGTCT   
  
  
- CTTTTGGGGT TCTTAATATT ATTAATTTG

  
  
Motifs Found  

+   

| Site Name | Organism | Position | Strand | Matrix score. | sequence | function |
| --- | --- | --- | --- | --- | --- | --- |
|  | organism | 189 | + | 4 | motif\_sequence | short\_function |
|  | organism | 1005 | - | 4 | motif\_sequence | short\_function |
|  | organism | 664 | + | 4 | motif\_sequence | short\_function |
|  | organism | 1237 | - | 4 | motif\_sequence | short\_function |
|  | organism | 655 | + | 4 | motif\_sequence | short\_function |
|  | organism | 1013 | - | 4 | motif\_sequence | short\_function |
|  | organism | 672 | + | 4 | motif\_sequence | short\_function |

>036006   
+ GTATACTTAA AACTAGAGAA GCCATCGATG GGCAATTGTT AGGCATAAAA ATAGTGAAAG AACCACCGGC   
  
  
+ CGGATAAACA AGAGTAAAGC TGTCTGAAAA TAAACCCTAC TCACACACAT GGTATTTCTC ATATTTTATT   
  
  
+ TTATAGTCAA TTATTAAGAT AATAAGCATT TTAACCATTT ATGTATACAT GTTCATACCA CGAGCGTTGC   
  
  
+ TTGGAGGAAC ACATTTTGGT ATCAAGTGAT GTTGCAAAAT GATGCATACA ATCAGTACAC ATCACTCTAG   
  
  
+ CTACTTACTC CATCTTGTAG TATTGCAATG GAGTAAAATA TAGCACTAGT CCCAGATTAA TTATTATGGT   
  
  
+ TCTCCTTTCA AACACATAGT TAATGGTTAT AATGATATAC TTAAGGGCAA AATATATTAC TTATACAAAA   
  
  
+ TAACTCGATG GGTTACGTAA AATACATGAA GAATTCTGTT TAATAAGTTT AGTATATGCT TGCATGAGTA   
  
  
+ TGATACAATA TATATGTGAC AAAATAAAAT ACAAGTCTAA ATTTACGACA CCACCACCCC CTTATATAGA   
  
  
+ GTTAAAACGT GAGATTCAAG AGTGTAGTTG GTGGAAAAAG ATAAATTCTA GGGTTTTTGT CTTGTAGAAA   
  
  
+ TACATCATAT ACATAAATAT TTATCTGTAC AACATGTGCG TATGTACAAT AATCATTGAC AATTAACCTA   
  
  
+ AACATTGCAA TTAACATAAA CTTTGTTTCT TCTTAAATTT TAGATGGTAA GTTCCTCCTA TCAAATCATT   
  
  
+ ACAAAATCAT AAAATCCCAA TAACACACTT TATCTATACG CAAAAAAATT TGCCGGGGAA AATATAGTAC   
  
  
+ GTGGAGGTAT ATATAGTACG TACTTTCTCC TTTGCTTTCA AAGATCATCA AATTTTAATT AGTTTATATT   
  
  
+ ATTTTATATA TATACACACA CACACTAGCA TATAGGCACA CACAAAGTGT GTGAGACATT TTTTTATTTT   
  
  
+ TGTTTTTGAA ATAGAAAGAG AGAGGAAGAG AGGAAGAGAG CGATAGGGAA TGTGAGAATC AGAAGTTTTT   
  
  
+ TTTTTTTTTT TAATTAAAGA TCTGTTAAGA TTACATGTAG TTGAAATTTT GAAAAAAAAA CAGCCAAATT   
  
  
+ TAGTTATGTG AAATTACATT TTTGTCCCGT ATTTGTTATT CATATTTTGT TTTAATTAGA GAGTTAAAGT   
  
  
+ GGTAATTTGA TAGAATTTTG GTTGACAATG AATGTTTTAT TAATTAGTAG ATATATATAT ATATATAATT   
  
  
+ GTTATAAGTA AGTGGAAAAG TATTGTTGTC GTGGAGACAA ATTTAGGTTC TTGAGCAAAA ACTCAACTAC   
  
  
+ TCTCTAGAGA GAGAGAGAGG TAGCAGTAAC ACGACTTGTG TAGAAATTGT GAGGGACGAA TCGGTTAAGA   
  
  
+ AGTGCATGTC TTGTATCTCT CTCTAGAGCT AGAAAAATCC TAAGTATTTC ATTTTTTTGG AACAAACAGA   
  
  
+ GAAAACCCCA AGAATTATAA TAATTAAAC  

- CATATGAATT TTGATCTCTT CGGTAGCTAC CCGTTAACAA TCCGTATTTT TATCACTTTC TTGGTGGCCG   
  
  
- GCCTATTTGT TCTCATTTCG ACAGACTTTT ATTTGGGATG AGTGTGTGTA CCATAAAGAG TATAAAATAA   
  
  
- AATATCAGTT AATAATTCTA TTATTCGTAA AATTGGTAAA TACATATGTA CAAGTATGGT GCTCGCAACG   
  
  
- AACCTCCTTG TGTAAAACCA TAGTTCACTA CAACGTTTTA CTACGTATGT TAGTCATGTG TAGTGAGATC   
  
  
- GATGAATGAG GTAGAACATC ATAACGTTAC CTCATTTTAT ATCGTGATCA GGGTCTAATT AATAATACCA   
  
  
- AGAGGAAAGT TTGTGTATCA ATTACCAATA TTACTATATG AATTCCCGTT TTATATAATG AATATGTTTT   
  
  
- ATTGAGCTAC CCAATGCATT TTATGTACTT CTTAAGACAA ATTATTCAAA TCATATACGA ACGTACTCAT   
  
  
- ACTATGTTAT ATATACACTG TTTTATTTTA TGTTCAGATT TAAATGCTGT GGTGGTGGGG GAATATATCT   
  
  
- CAATTTTGCA CTCTAAGTTC TCACATCAAC CACCTTTTTC TATTTAAGAT CCCAAAAACA GAACATCTTT   
  
  
- ATGTAGTATA TGTATTTATA AATAGACATG TTGTACACGC ATACATGTTA TTAGTAACTG TTAATTGGAT   
  
  
- TTGTAACGTT AATTGTATTT GAAACAAAGA AGAATTTAAA ATCTACCATT CAAGGAGGAT AGTTTAGTAA   
  
  
- TGTTTTAGTA TTTTAGGGTT ATTGTGTGAA ATAGATATGC GTTTTTTTAA ACGGCCCCTT TTATATCATG   
  
  
- CACCTCCATA TATATCATGC ATGAAAGAGG AAACGAAAGT TTCTAGTAGT TTAAAATTAA TCAAATATAA   
  
  
- TAAAATATAT ATATGTGTGT GTGTGATCGT ATATCCGTGT GTGTTTCACA CACTCTGTAA AAAAATAAAA   
  
  
- ACAAAAACTT TATCTTTCTC TCTCCTTCTC TCCTTCTCTC GCTATCCCTT ACACTCTTAG TCTTCAAAAA   
  
  
- AAAAAAAAAA ATTAATTTCT AGACAATTCT AATGTACATC AACTTTAAAA CTTTTTTTTT GTCGGTTTAA   
  
  
- ATCAATACAC TTTAATGTAA AAACAGGGCA TAAACAATAA GTATAAAACA AAATTAATCT CTCAATTTCA   
  
  
- CCATTAAACT ATCTTAAAAC CAACTGTTAC TTACAAAATA ATTAATCATC TATATATATA TATATATTAA   
  
  
- CAATATTCAT TCACCTTTTC ATAACAACAG CACCTCTGTT TAAATCCAAG AACTCGTTTT TGAGTTGATG   
  
  
- AGAGATCTCT CTCTCTCTCC ATCGTCATTG TGCTGAACAC ATCTTTAACA CTCCCTGCTT AGCCAATTCT   
  
  
- TCACGTACAG AACATAGAGA GAGATCTCGA TCTTTTTAGG ATTCATAAAG TAAAAAAACC TTGTTTGTCT   
  
  
- CTTTTGGGGT TCTTAATATT ATTAATTTG

+     3-AF1 binding site

| Site Name | Organism | Position | Strand | Matrix score. | sequence | function |
| --- | --- | --- | --- | --- | --- | --- |
| 3-AF1 binding site | Solanum tuberosum | 1005 | + | 10 | TAAGAGAGGAA | light responsive element |

>036006   
+ GTATACTTAA AACTAGAGAA GCCATCGATG GGCAATTGTT AGGCATAAAA ATAGTGAAAG AACCACCGGC   
  
  
+ CGGATAAACA AGAGTAAAGC TGTCTGAAAA TAAACCCTAC TCACACACAT GGTATTTCTC ATATTTTATT   
  
  
+ TTATAGTCAA TTATTAAGAT AATAAGCATT TTAACCATTT ATGTATACAT GTTCATACCA CGAGCGTTGC   
  
  
+ TTGGAGGAAC ACATTTTGGT ATCAAGTGAT GTTGCAAAAT GATGCATACA ATCAGTACAC ATCACTCTAG   
  
  
+ CTACTTACTC CATCTTGTAG TATTGCAATG GAGTAAAATA TAGCACTAGT CCCAGATTAA TTATTATGGT   
  
  
+ TCTCCTTTCA AACACATAGT TAATGGTTAT AATGATATAC TTAAGGGCAA AATATATTAC TTATACAAAA   
  
  
+ TAACTCGATG GGTTACGTAA AATACATGAA GAATTCTGTT TAATAAGTTT AGTATATGCT TGCATGAGTA   
  
  
+ TGATACAATA TATATGTGAC AAAATAAAAT ACAAGTCTAA ATTTACGACA CCACCACCCC CTTATATAGA   
  
  
+ GTTAAAACGT GAGATTCAAG AGTGTAGTTG GTGGAAAAAG ATAAATTCTA GGGTTTTTGT CTTGTAGAAA   
  
  
+ TACATCATAT ACATAAATAT TTATCTGTAC AACATGTGCG TATGTACAAT AATCATTGAC AATTAACCTA   
  
  
+ AACATTGCAA TTAACATAAA CTTTGTTTCT TCTTAAATTT TAGATGGTAA GTTCCTCCTA TCAAATCATT   
  
  
+ ACAAAATCAT AAAATCCCAA TAACACACTT TATCTATACG CAAAAAAATT TGCCGGGGAA AATATAGTAC   
  
  
+ GTGGAGGTAT ATATAGTACG TACTTTCTCC TTTGCTTTCA AAGATCATCA AATTTTAATT AGTTTATATT   
  
  
+ ATTTTATATA TATACACACA CACACTAGCA TATAGGCACA CACAAAGTGT GTGAGACATT TTTTTATTTT   
  
  
+ TGTTTTTGAA ATAGAAAGAG AGAGGAAGAG AGGAAGAGAG CGATAGGGAA TGTGAGAATC AGAAGTTTTT   
  
  
+ TTTTTTTTTT TAATTAAAGA TCTGTTAAGA TTACATGTAG TTGAAATTTT GAAAAAAAAA CAGCCAAATT   
  
  
+ TAGTTATGTG AAATTACATT TTTGTCCCGT ATTTGTTATT CATATTTTGT TTTAATTAGA GAGTTAAAGT   
  
  
+ GGTAATTTGA TAGAATTTTG GTTGACAATG AATGTTTTAT TAATTAGTAG ATATATATAT ATATATAATT   
  
  
+ GTTATAAGTA AGTGGAAAAG TATTGTTGTC GTGGAGACAA ATTTAGGTTC TTGAGCAAAA ACTCAACTAC   
  
  
+ TCTCTAGAGA GAGAGAGAGG TAGCAGTAAC ACGACTTGTG TAGAAATTGT GAGGGACGAA TCGGTTAAGA   
  
  
+ AGTGCATGTC TTGTATCTCT CTCTAGAGCT AGAAAAATCC TAAGTATTTC ATTTTTTTGG AACAAACAGA   
  
  
+ GAAAACCCCA AGAATTATAA TAATTAAAC  

- CATATGAATT TTGATCTCTT CGGTAGCTAC CCGTTAACAA TCCGTATTTT TATCACTTTC TTGGTGGCCG   
  
  
- GCCTATTTGT TCTCATTTCG ACAGACTTTT ATTTGGGATG AGTGTGTGTA CCATAAAGAG TATAAAATAA   
  
  
- AATATCAGTT AATAATTCTA TTATTCGTAA AATTGGTAAA TACATATGTA CAAGTATGGT GCTCGCAACG   
  
  
- AACCTCCTTG TGTAAAACCA TAGTTCACTA CAACGTTTTA CTACGTATGT TAGTCATGTG TAGTGAGATC   
  
  
- GATGAATGAG GTAGAACATC ATAACGTTAC CTCATTTTAT ATCGTGATCA GGGTCTAATT AATAATACCA   
  
  
- AGAGGAAAGT TTGTGTATCA ATTACCAATA TTACTATATG AATTCCCGTT TTATATAATG AATATGTTTT   
  
  
- ATTGAGCTAC CCAATGCATT TTATGTACTT CTTAAGACAA ATTATTCAAA TCATATACGA ACGTACTCAT   
  
  
- ACTATGTTAT ATATACACTG TTTTATTTTA TGTTCAGATT TAAATGCTGT GGTGGTGGGG GAATATATCT   
  
  
- CAATTTTGCA CTCTAAGTTC TCACATCAAC CACCTTTTTC TATTTAAGAT CCCAAAAACA GAACATCTTT   
  
  
- ATGTAGTATA TGTATTTATA AATAGACATG TTGTACACGC ATACATGTTA TTAGTAACTG TTAATTGGAT   
  
  
- TTGTAACGTT AATTGTATTT GAAACAAAGA AGAATTTAAA ATCTACCATT CAAGGAGGAT AGTTTAGTAA   
  
  
- TGTTTTAGTA TTTTAGGGTT ATTGTGTGAA ATAGATATGC GTTTTTTTAA ACGGCCCCTT TTATATCATG   
  
  
- CACCTCCATA TATATCATGC ATGAAAGAGG AAACGAAAGT TTCTAGTAGT TTAAAATTAA TCAAATATAA   
  
  
- TAAAATATAT ATATGTGTGT GTGTGATCGT ATATCCGTGT GTGTTTCACA CACTCTGTAA AAAAATAAAA   
  
  
- ACAAAAACTT TATCTTTCTC TCTCCTTCTC TCCTTCTCTC GCTATCCCTT ACACTCTTAG TCTTCAAAAA   
  
  
- AAAAAAAAAA ATTAATTTCT AGACAATTCT AATGTACATC AACTTTAAAA CTTTTTTTTT GTCGGTTTAA   
  
  
- ATCAATACAC TTTAATGTAA AAACAGGGCA TAAACAATAA GTATAAAACA AAATTAATCT CTCAATTTCA   
  
  
- CCATTAAACT ATCTTAAAAC CAACTGTTAC TTACAAAATA ATTAATCATC TATATATATA TATATATTAA   
  
  
- CAATATTCAT TCACCTTTTC ATAACAACAG CACCTCTGTT TAAATCCAAG AACTCGTTTT TGAGTTGATG   
  
  
- AGAGATCTCT CTCTCTCTCC ATCGTCATTG TGCTGAACAC ATCTTTAACA CTCCCTGCTT AGCCAATTCT   
  
  
- TCACGTACAG AACATAGAGA GAGATCTCGA TCTTTTTAGG ATTCATAAAG TAAAAAAACC TTGTTTGTCT   
  
  
- CTTTTGGGGT TCTTAATATT ATTAATTTG

+     AAGAA-motif

| Site Name | Organism | Position | Strand | Matrix score. | sequence | function |
| --- | --- | --- | --- | --- | --- | --- |
| AAGAA-motif | Avena sativa | 56 | + | 7 | GAAAGAA |  |

>036006   
+ GTATACTTAA AACTAGAGAA GCCATCGATG GGCAATTGTT AGGCATAAAA ATAGTGAAAG AACCACCGGC   
  
  
+ CGGATAAACA AGAGTAAAGC TGTCTGAAAA TAAACCCTAC TCACACACAT GGTATTTCTC ATATTTTATT   
  
  
+ TTATAGTCAA TTATTAAGAT AATAAGCATT TTAACCATTT ATGTATACAT GTTCATACCA CGAGCGTTGC   
  
  
+ TTGGAGGAAC ACATTTTGGT ATCAAGTGAT GTTGCAAAAT GATGCATACA ATCAGTACAC ATCACTCTAG   
  
  
+ CTACTTACTC CATCTTGTAG TATTGCAATG GAGTAAAATA TAGCACTAGT CCCAGATTAA TTATTATGGT   
  
  
+ TCTCCTTTCA AACACATAGT TAATGGTTAT AATGATATAC TTAAGGGCAA AATATATTAC TTATACAAAA   
  
  
+ TAACTCGATG GGTTACGTAA AATACATGAA GAATTCTGTT TAATAAGTTT AGTATATGCT TGCATGAGTA   
  
  
+ TGATACAATA TATATGTGAC AAAATAAAAT ACAAGTCTAA ATTTACGACA CCACCACCCC CTTATATAGA   
  
  
+ GTTAAAACGT GAGATTCAAG AGTGTAGTTG GTGGAAAAAG ATAAATTCTA GGGTTTTTGT CTTGTAGAAA   
  
  
+ TACATCATAT ACATAAATAT TTATCTGTAC AACATGTGCG TATGTACAAT AATCATTGAC AATTAACCTA   
  
  
+ AACATTGCAA TTAACATAAA CTTTGTTTCT TCTTAAATTT TAGATGGTAA GTTCCTCCTA TCAAATCATT   
  
  
+ ACAAAATCAT AAAATCCCAA TAACACACTT TATCTATACG CAAAAAAATT TGCCGGGGAA AATATAGTAC   
  
  
+ GTGGAGGTAT ATATAGTACG TACTTTCTCC TTTGCTTTCA AAGATCATCA AATTTTAATT AGTTTATATT   
  
  
+ ATTTTATATA TATACACACA CACACTAGCA TATAGGCACA CACAAAGTGT GTGAGACATT TTTTTATTTT   
  
  
+ TGTTTTTGAA ATAGAAAGAG AGAGGAAGAG AGGAAGAGAG CGATAGGGAA TGTGAGAATC AGAAGTTTTT   
  
  
+ TTTTTTTTTT TAATTAAAGA TCTGTTAAGA TTACATGTAG TTGAAATTTT GAAAAAAAAA CAGCCAAATT   
  
  
+ TAGTTATGTG AAATTACATT TTTGTCCCGT ATTTGTTATT CATATTTTGT TTTAATTAGA GAGTTAAAGT   
  
  
+ GGTAATTTGA TAGAATTTTG GTTGACAATG AATGTTTTAT TAATTAGTAG ATATATATAT ATATATAATT   
  
  
+ GTTATAAGTA AGTGGAAAAG TATTGTTGTC GTGGAGACAA ATTTAGGTTC TTGAGCAAAA ACTCAACTAC   
  
  
+ TCTCTAGAGA GAGAGAGAGG TAGCAGTAAC ACGACTTGTG TAGAAATTGT GAGGGACGAA TCGGTTAAGA   
  
  
+ AGTGCATGTC TTGTATCTCT CTCTAGAGCT AGAAAAATCC TAAGTATTTC ATTTTTTTGG AACAAACAGA   
  
  
+ GAAAACCCCA AGAATTATAA TAATTAAAC  

- CATATGAATT TTGATCTCTT CGGTAGCTAC CCGTTAACAA TCCGTATTTT TATCACTTTC TTGGTGGCCG   
  
  
- GCCTATTTGT TCTCATTTCG ACAGACTTTT ATTTGGGATG AGTGTGTGTA CCATAAAGAG TATAAAATAA   
  
  
- AATATCAGTT AATAATTCTA TTATTCGTAA AATTGGTAAA TACATATGTA CAAGTATGGT GCTCGCAACG   
  
  
- AACCTCCTTG TGTAAAACCA TAGTTCACTA CAACGTTTTA CTACGTATGT TAGTCATGTG TAGTGAGATC   
  
  
- GATGAATGAG GTAGAACATC ATAACGTTAC CTCATTTTAT ATCGTGATCA GGGTCTAATT AATAATACCA   
  
  
- AGAGGAAAGT TTGTGTATCA ATTACCAATA TTACTATATG AATTCCCGTT TTATATAATG AATATGTTTT   
  
  
- ATTGAGCTAC CCAATGCATT TTATGTACTT CTTAAGACAA ATTATTCAAA TCATATACGA ACGTACTCAT   
  
  
- ACTATGTTAT ATATACACTG TTTTATTTTA TGTTCAGATT TAAATGCTGT GGTGGTGGGG GAATATATCT   
  
  
- CAATTTTGCA CTCTAAGTTC TCACATCAAC CACCTTTTTC TATTTAAGAT CCCAAAAACA GAACATCTTT   
  
  
- ATGTAGTATA TGTATTTATA AATAGACATG TTGTACACGC ATACATGTTA TTAGTAACTG TTAATTGGAT   
  
  
- TTGTAACGTT AATTGTATTT GAAACAAAGA AGAATTTAAA ATCTACCATT CAAGGAGGAT AGTTTAGTAA   
  
  
- TGTTTTAGTA TTTTAGGGTT ATTGTGTGAA ATAGATATGC GTTTTTTTAA ACGGCCCCTT TTATATCATG   
  
  
- CACCTCCATA TATATCATGC ATGAAAGAGG AAACGAAAGT TTCTAGTAGT TTAAAATTAA TCAAATATAA   
  
  
- TAAAATATAT ATATGTGTGT GTGTGATCGT ATATCCGTGT GTGTTTCACA CACTCTGTAA AAAAATAAAA   
  
  
- ACAAAAACTT TATCTTTCTC TCTCCTTCTC TCCTTCTCTC GCTATCCCTT ACACTCTTAG TCTTCAAAAA   
  
  
- AAAAAAAAAA ATTAATTTCT AGACAATTCT AATGTACATC AACTTTAAAA CTTTTTTTTT GTCGGTTTAA   
  
  
- ATCAATACAC TTTAATGTAA AAACAGGGCA TAAACAATAA GTATAAAACA AAATTAATCT CTCAATTTCA   
  
  
- CCATTAAACT ATCTTAAAAC CAACTGTTAC TTACAAAATA ATTAATCATC TATATATATA TATATATTAA   
  
  
- CAATATTCAT TCACCTTTTC ATAACAACAG CACCTCTGTT TAAATCCAAG AACTCGTTTT TGAGTTGATG   
  
  
- AGAGATCTCT CTCTCTCTCC ATCGTCATTG TGCTGAACAC ATCTTTAACA CTCCCTGCTT AGCCAATTCT   
  
  
- TCACGTACAG AACATAGAGA GAGATCTCGA TCTTTTTAGG ATTCATAAAG TAAAAAAACC TTGTTTGTCT   
  
  
- CTTTTGGGGT TCTTAATATT ATTAATTTG

+     ABRE

| Site Name | Organism | Position | Strand | Matrix score. | sequence | function |
| --- | --- | --- | --- | --- | --- | --- |
| ABRE | Arabidopsis thaliana | 839 | + | 5 | ACGTG | cis-acting element involved in the abscisic acid responsiveness |
| ABRE | Arabidopsis thaliana | 567 | + | 5 | ACGTG | cis-acting element involved in the abscisic acid responsiveness |

>036006   
+ GTATACTTAA AACTAGAGAA GCCATCGATG GGCAATTGTT AGGCATAAAA ATAGTGAAAG AACCACCGGC   
  
  
+ CGGATAAACA AGAGTAAAGC TGTCTGAAAA TAAACCCTAC TCACACACAT GGTATTTCTC ATATTTTATT   
  
  
+ TTATAGTCAA TTATTAAGAT AATAAGCATT TTAACCATTT ATGTATACAT GTTCATACCA CGAGCGTTGC   
  
  
+ TTGGAGGAAC ACATTTTGGT ATCAAGTGAT GTTGCAAAAT GATGCATACA ATCAGTACAC ATCACTCTAG   
  
  
+ CTACTTACTC CATCTTGTAG TATTGCAATG GAGTAAAATA TAGCACTAGT CCCAGATTAA TTATTATGGT   
  
  
+ TCTCCTTTCA AACACATAGT TAATGGTTAT AATGATATAC TTAAGGGCAA AATATATTAC TTATACAAAA   
  
  
+ TAACTCGATG GGTTACGTAA AATACATGAA GAATTCTGTT TAATAAGTTT AGTATATGCT TGCATGAGTA   
  
  
+ TGATACAATA TATATGTGAC AAAATAAAAT ACAAGTCTAA ATTTACGACA CCACCACCCC CTTATATAGA   
  
  
+ GTTAAAACGT GAGATTCAAG AGTGTAGTTG GTGGAAAAAG ATAAATTCTA GGGTTTTTGT CTTGTAGAAA   
  
  
+ TACATCATAT ACATAAATAT TTATCTGTAC AACATGTGCG TATGTACAAT AATCATTGAC AATTAACCTA   
  
  
+ AACATTGCAA TTAACATAAA CTTTGTTTCT TCTTAAATTT TAGATGGTAA GTTCCTCCTA TCAAATCATT   
  
  
+ ACAAAATCAT AAAATCCCAA TAACACACTT TATCTATACG CAAAAAAATT TGCCGGGGAA AATATAGTAC   
  
  
+ GTGGAGGTAT ATATAGTACG TACTTTCTCC TTTGCTTTCA AAGATCATCA AATTTTAATT AGTTTATATT   
  
  
+ ATTTTATATA TATACACACA CACACTAGCA TATAGGCACA CACAAAGTGT GTGAGACATT TTTTTATTTT   
  
  
+ TGTTTTTGAA ATAGAAAGAG AGAGGAAGAG AGGAAGAGAG CGATAGGGAA TGTGAGAATC AGAAGTTTTT   
  
  
+ TTTTTTTTTT TAATTAAAGA TCTGTTAAGA TTACATGTAG TTGAAATTTT GAAAAAAAAA CAGCCAAATT   
  
  
+ TAGTTATGTG AAATTACATT TTTGTCCCGT ATTTGTTATT CATATTTTGT TTTAATTAGA GAGTTAAAGT   
  
  
+ GGTAATTTGA TAGAATTTTG GTTGACAATG AATGTTTTAT TAATTAGTAG ATATATATAT ATATATAATT   
  
  
+ GTTATAAGTA AGTGGAAAAG TATTGTTGTC GTGGAGACAA ATTTAGGTTC TTGAGCAAAA ACTCAACTAC   
  
  
+ TCTCTAGAGA GAGAGAGAGG TAGCAGTAAC ACGACTTGTG TAGAAATTGT GAGGGACGAA TCGGTTAAGA   
  
  
+ AGTGCATGTC TTGTATCTCT CTCTAGAGCT AGAAAAATCC TAAGTATTTC ATTTTTTTGG AACAAACAGA   
  
  
+ GAAAACCCCA AGAATTATAA TAATTAAAC  

- CATATGAATT TTGATCTCTT CGGTAGCTAC CCGTTAACAA TCCGTATTTT TATCACTTTC TTGGTGGCCG   
  
  
- GCCTATTTGT TCTCATTTCG ACAGACTTTT ATTTGGGATG AGTGTGTGTA CCATAAAGAG TATAAAATAA   
  
  
- AATATCAGTT AATAATTCTA TTATTCGTAA AATTGGTAAA TACATATGTA CAAGTATGGT GCTCGCAACG   
  
  
- AACCTCCTTG TGTAAAACCA TAGTTCACTA CAACGTTTTA CTACGTATGT TAGTCATGTG TAGTGAGATC   
  
  
- GATGAATGAG GTAGAACATC ATAACGTTAC CTCATTTTAT ATCGTGATCA GGGTCTAATT AATAATACCA   
  
  
- AGAGGAAAGT TTGTGTATCA ATTACCAATA TTACTATATG AATTCCCGTT TTATATAATG AATATGTTTT   
  
  
- ATTGAGCTAC CCAATGCATT TTATGTACTT CTTAAGACAA ATTATTCAAA TCATATACGA ACGTACTCAT   
  
  
- ACTATGTTAT ATATACACTG TTTTATTTTA TGTTCAGATT TAAATGCTGT GGTGGTGGGG GAATATATCT   
  
  
- CAATTTTGCA CTCTAAGTTC TCACATCAAC CACCTTTTTC TATTTAAGAT CCCAAAAACA GAACATCTTT   
  
  
- ATGTAGTATA TGTATTTATA AATAGACATG TTGTACACGC ATACATGTTA TTAGTAACTG TTAATTGGAT   
  
  
- TTGTAACGTT AATTGTATTT GAAACAAAGA AGAATTTAAA ATCTACCATT CAAGGAGGAT AGTTTAGTAA   
  
  
- TGTTTTAGTA TTTTAGGGTT ATTGTGTGAA ATAGATATGC GTTTTTTTAA ACGGCCCCTT TTATATCATG   
  
  
- CACCTCCATA TATATCATGC ATGAAAGAGG AAACGAAAGT TTCTAGTAGT TTAAAATTAA TCAAATATAA   
  
  
- TAAAATATAT ATATGTGTGT GTGTGATCGT ATATCCGTGT GTGTTTCACA CACTCTGTAA AAAAATAAAA   
  
  
- ACAAAAACTT TATCTTTCTC TCTCCTTCTC TCCTTCTCTC GCTATCCCTT ACACTCTTAG TCTTCAAAAA   
  
  
- AAAAAAAAAA ATTAATTTCT AGACAATTCT AATGTACATC AACTTTAAAA CTTTTTTTTT GTCGGTTTAA   
  
  
- ATCAATACAC TTTAATGTAA AAACAGGGCA TAAACAATAA GTATAAAACA AAATTAATCT CTCAATTTCA   
  
  
- CCATTAAACT ATCTTAAAAC CAACTGTTAC TTACAAAATA ATTAATCATC TATATATATA TATATATTAA   
  
  
- CAATATTCAT TCACCTTTTC ATAACAACAG CACCTCTGTT TAAATCCAAG AACTCGTTTT TGAGTTGATG   
  
  
- AGAGATCTCT CTCTCTCTCC ATCGTCATTG TGCTGAACAC ATCTTTAACA CTCCCTGCTT AGCCAATTCT   
  
  
- TCACGTACAG AACATAGAGA GAGATCTCGA TCTTTTTAGG ATTCATAAAG TAAAAAAACC TTGTTTGTCT   
  
  
- CTTTTGGGGT TCTTAATATT ATTAATTTG

+     ABRE3a

| Site Name | Organism | Position | Strand | Matrix score. | sequence | function |
| --- | --- | --- | --- | --- | --- | --- |
| ABRE3a | Zea mays | 838 | + | 6 | TACGTG |  |

>036006   
+ GTATACTTAA AACTAGAGAA GCCATCGATG GGCAATTGTT AGGCATAAAA ATAGTGAAAG AACCACCGGC   
  
  
+ CGGATAAACA AGAGTAAAGC TGTCTGAAAA TAAACCCTAC TCACACACAT GGTATTTCTC ATATTTTATT   
  
  
+ TTATAGTCAA TTATTAAGAT AATAAGCATT TTAACCATTT ATGTATACAT GTTCATACCA CGAGCGTTGC   
  
  
+ TTGGAGGAAC ACATTTTGGT ATCAAGTGAT GTTGCAAAAT GATGCATACA ATCAGTACAC ATCACTCTAG   
  
  
+ CTACTTACTC CATCTTGTAG TATTGCAATG GAGTAAAATA TAGCACTAGT CCCAGATTAA TTATTATGGT   
  
  
+ TCTCCTTTCA AACACATAGT TAATGGTTAT AATGATATAC TTAAGGGCAA AATATATTAC TTATACAAAA   
  
  
+ TAACTCGATG GGTTACGTAA AATACATGAA GAATTCTGTT TAATAAGTTT AGTATATGCT TGCATGAGTA   
  
  
+ TGATACAATA TATATGTGAC AAAATAAAAT ACAAGTCTAA ATTTACGACA CCACCACCCC CTTATATAGA   
  
  
+ GTTAAAACGT GAGATTCAAG AGTGTAGTTG GTGGAAAAAG ATAAATTCTA GGGTTTTTGT CTTGTAGAAA   
  
  
+ TACATCATAT ACATAAATAT TTATCTGTAC AACATGTGCG TATGTACAAT AATCATTGAC AATTAACCTA   
  
  
+ AACATTGCAA TTAACATAAA CTTTGTTTCT TCTTAAATTT TAGATGGTAA GTTCCTCCTA TCAAATCATT   
  
  
+ ACAAAATCAT AAAATCCCAA TAACACACTT TATCTATACG CAAAAAAATT TGCCGGGGAA AATATAGTAC   
  
  
+ GTGGAGGTAT ATATAGTACG TACTTTCTCC TTTGCTTTCA AAGATCATCA AATTTTAATT AGTTTATATT   
  
  
+ ATTTTATATA TATACACACA CACACTAGCA TATAGGCACA CACAAAGTGT GTGAGACATT TTTTTATTTT   
  
  
+ TGTTTTTGAA ATAGAAAGAG AGAGGAAGAG AGGAAGAGAG CGATAGGGAA TGTGAGAATC AGAAGTTTTT   
  
  
+ TTTTTTTTTT TAATTAAAGA TCTGTTAAGA TTACATGTAG TTGAAATTTT GAAAAAAAAA CAGCCAAATT   
  
  
+ TAGTTATGTG AAATTACATT TTTGTCCCGT ATTTGTTATT CATATTTTGT TTTAATTAGA GAGTTAAAGT   
  
  
+ GGTAATTTGA TAGAATTTTG GTTGACAATG AATGTTTTAT TAATTAGTAG ATATATATAT ATATATAATT   
  
  
+ GTTATAAGTA AGTGGAAAAG TATTGTTGTC GTGGAGACAA ATTTAGGTTC TTGAGCAAAA ACTCAACTAC   
  
  
+ TCTCTAGAGA GAGAGAGAGG TAGCAGTAAC ACGACTTGTG TAGAAATTGT GAGGGACGAA TCGGTTAAGA   
  
  
+ AGTGCATGTC TTGTATCTCT CTCTAGAGCT AGAAAAATCC TAAGTATTTC ATTTTTTTGG AACAAACAGA   
  
  
+ GAAAACCCCA AGAATTATAA TAATTAAAC  

- CATATGAATT TTGATCTCTT CGGTAGCTAC CCGTTAACAA TCCGTATTTT TATCACTTTC TTGGTGGCCG   
  
  
- GCCTATTTGT TCTCATTTCG ACAGACTTTT ATTTGGGATG AGTGTGTGTA CCATAAAGAG TATAAAATAA   
  
  
- AATATCAGTT AATAATTCTA TTATTCGTAA AATTGGTAAA TACATATGTA CAAGTATGGT GCTCGCAACG   
  
  
- AACCTCCTTG TGTAAAACCA TAGTTCACTA CAACGTTTTA CTACGTATGT TAGTCATGTG TAGTGAGATC   
  
  
- GATGAATGAG GTAGAACATC ATAACGTTAC CTCATTTTAT ATCGTGATCA GGGTCTAATT AATAATACCA   
  
  
- AGAGGAAAGT TTGTGTATCA ATTACCAATA TTACTATATG AATTCCCGTT TTATATAATG AATATGTTTT   
  
  
- ATTGAGCTAC CCAATGCATT TTATGTACTT CTTAAGACAA ATTATTCAAA TCATATACGA ACGTACTCAT   
  
  
- ACTATGTTAT ATATACACTG TTTTATTTTA TGTTCAGATT TAAATGCTGT GGTGGTGGGG GAATATATCT   
  
  
- CAATTTTGCA CTCTAAGTTC TCACATCAAC CACCTTTTTC TATTTAAGAT CCCAAAAACA GAACATCTTT   
  
  
- ATGTAGTATA TGTATTTATA AATAGACATG TTGTACACGC ATACATGTTA TTAGTAACTG TTAATTGGAT   
  
  
- TTGTAACGTT AATTGTATTT GAAACAAAGA AGAATTTAAA ATCTACCATT CAAGGAGGAT AGTTTAGTAA   
  
  
- TGTTTTAGTA TTTTAGGGTT ATTGTGTGAA ATAGATATGC GTTTTTTTAA ACGGCCCCTT TTATATCATG   
  
  
- CACCTCCATA TATATCATGC ATGAAAGAGG AAACGAAAGT TTCTAGTAGT TTAAAATTAA TCAAATATAA   
  
  
- TAAAATATAT ATATGTGTGT GTGTGATCGT ATATCCGTGT GTGTTTCACA CACTCTGTAA AAAAATAAAA   
  
  
- ACAAAAACTT TATCTTTCTC TCTCCTTCTC TCCTTCTCTC GCTATCCCTT ACACTCTTAG TCTTCAAAAA   
  
  
- AAAAAAAAAA ATTAATTTCT AGACAATTCT AATGTACATC AACTTTAAAA CTTTTTTTTT GTCGGTTTAA   
  
  
- ATCAATACAC TTTAATGTAA AAACAGGGCA TAAACAATAA GTATAAAACA AAATTAATCT CTCAATTTCA   
  
  
- CCATTAAACT ATCTTAAAAC CAACTGTTAC TTACAAAATA ATTAATCATC TATATATATA TATATATTAA   
  
  
- CAATATTCAT TCACCTTTTC ATAACAACAG CACCTCTGTT TAAATCCAAG AACTCGTTTT TGAGTTGATG   
  
  
- AGAGATCTCT CTCTCTCTCC ATCGTCATTG TGCTGAACAC ATCTTTAACA CTCCCTGCTT AGCCAATTCT   
  
  
- TCACGTACAG AACATAGAGA GAGATCTCGA TCTTTTTAGG ATTCATAAAG TAAAAAAACC TTGTTTGTCT   
  
  
- CTTTTGGGGT TCTTAATATT ATTAATTTG

+     ABRE4

| Site Name | Organism | Position | Strand | Matrix score. | sequence | function |
| --- | --- | --- | --- | --- | --- | --- |
| ABRE4 | Zea mays | 838 | - | 6 | CACGTA |  |

>036006   
+ GTATACTTAA AACTAGAGAA GCCATCGATG GGCAATTGTT AGGCATAAAA ATAGTGAAAG AACCACCGGC   
  
  
+ CGGATAAACA AGAGTAAAGC TGTCTGAAAA TAAACCCTAC TCACACACAT GGTATTTCTC ATATTTTATT   
  
  
+ TTATAGTCAA TTATTAAGAT AATAAGCATT TTAACCATTT ATGTATACAT GTTCATACCA CGAGCGTTGC   
  
  
+ TTGGAGGAAC ACATTTTGGT ATCAAGTGAT GTTGCAAAAT GATGCATACA ATCAGTACAC ATCACTCTAG   
  
  
+ CTACTTACTC CATCTTGTAG TATTGCAATG GAGTAAAATA TAGCACTAGT CCCAGATTAA TTATTATGGT   
  
  
+ TCTCCTTTCA AACACATAGT TAATGGTTAT AATGATATAC TTAAGGGCAA AATATATTAC TTATACAAAA   
  
  
+ TAACTCGATG GGTTACGTAA AATACATGAA GAATTCTGTT TAATAAGTTT AGTATATGCT TGCATGAGTA   
  
  
+ TGATACAATA TATATGTGAC AAAATAAAAT ACAAGTCTAA ATTTACGACA CCACCACCCC CTTATATAGA   
  
  
+ GTTAAAACGT GAGATTCAAG AGTGTAGTTG GTGGAAAAAG ATAAATTCTA GGGTTTTTGT CTTGTAGAAA   
  
  
+ TACATCATAT ACATAAATAT TTATCTGTAC AACATGTGCG TATGTACAAT AATCATTGAC AATTAACCTA   
  
  
+ AACATTGCAA TTAACATAAA CTTTGTTTCT TCTTAAATTT TAGATGGTAA GTTCCTCCTA TCAAATCATT   
  
  
+ ACAAAATCAT AAAATCCCAA TAACACACTT TATCTATACG CAAAAAAATT TGCCGGGGAA AATATAGTAC   
  
  
+ GTGGAGGTAT ATATAGTACG TACTTTCTCC TTTGCTTTCA AAGATCATCA AATTTTAATT AGTTTATATT   
  
  
+ ATTTTATATA TATACACACA CACACTAGCA TATAGGCACA CACAAAGTGT GTGAGACATT TTTTTATTTT   
  
  
+ TGTTTTTGAA ATAGAAAGAG AGAGGAAGAG AGGAAGAGAG CGATAGGGAA TGTGAGAATC AGAAGTTTTT   
  
  
+ TTTTTTTTTT TAATTAAAGA TCTGTTAAGA TTACATGTAG TTGAAATTTT GAAAAAAAAA CAGCCAAATT   
  
  
+ TAGTTATGTG AAATTACATT TTTGTCCCGT ATTTGTTATT CATATTTTGT TTTAATTAGA GAGTTAAAGT   
  
  
+ GGTAATTTGA TAGAATTTTG GTTGACAATG AATGTTTTAT TAATTAGTAG ATATATATAT ATATATAATT   
  
  
+ GTTATAAGTA AGTGGAAAAG TATTGTTGTC GTGGAGACAA ATTTAGGTTC TTGAGCAAAA ACTCAACTAC   
  
  
+ TCTCTAGAGA GAGAGAGAGG TAGCAGTAAC ACGACTTGTG TAGAAATTGT GAGGGACGAA TCGGTTAAGA   
  
  
+ AGTGCATGTC TTGTATCTCT CTCTAGAGCT AGAAAAATCC TAAGTATTTC ATTTTTTTGG AACAAACAGA   
  
  
+ GAAAACCCCA AGAATTATAA TAATTAAAC  

- CATATGAATT TTGATCTCTT CGGTAGCTAC CCGTTAACAA TCCGTATTTT TATCACTTTC TTGGTGGCCG   
  
  
- GCCTATTTGT TCTCATTTCG ACAGACTTTT ATTTGGGATG AGTGTGTGTA CCATAAAGAG TATAAAATAA   
  
  
- AATATCAGTT AATAATTCTA TTATTCGTAA AATTGGTAAA TACATATGTA CAAGTATGGT GCTCGCAACG   
  
  
- AACCTCCTTG TGTAAAACCA TAGTTCACTA CAACGTTTTA CTACGTATGT TAGTCATGTG TAGTGAGATC   
  
  
- GATGAATGAG GTAGAACATC ATAACGTTAC CTCATTTTAT ATCGTGATCA GGGTCTAATT AATAATACCA   
  
  
- AGAGGAAAGT TTGTGTATCA ATTACCAATA TTACTATATG AATTCCCGTT TTATATAATG AATATGTTTT   
  
  
- ATTGAGCTAC CCAATGCATT TTATGTACTT CTTAAGACAA ATTATTCAAA TCATATACGA ACGTACTCAT   
  
  
- ACTATGTTAT ATATACACTG TTTTATTTTA TGTTCAGATT TAAATGCTGT GGTGGTGGGG GAATATATCT   
  
  
- CAATTTTGCA CTCTAAGTTC TCACATCAAC CACCTTTTTC TATTTAAGAT CCCAAAAACA GAACATCTTT   
  
  
- ATGTAGTATA TGTATTTATA AATAGACATG TTGTACACGC ATACATGTTA TTAGTAACTG TTAATTGGAT   
  
  
- TTGTAACGTT AATTGTATTT GAAACAAAGA AGAATTTAAA ATCTACCATT CAAGGAGGAT AGTTTAGTAA   
  
  
- TGTTTTAGTA TTTTAGGGTT ATTGTGTGAA ATAGATATGC GTTTTTTTAA ACGGCCCCTT TTATATCATG   
  
  
- CACCTCCATA TATATCATGC ATGAAAGAGG AAACGAAAGT TTCTAGTAGT TTAAAATTAA TCAAATATAA   
  
  
- TAAAATATAT ATATGTGTGT GTGTGATCGT ATATCCGTGT GTGTTTCACA CACTCTGTAA AAAAATAAAA   
  
  
- ACAAAAACTT TATCTTTCTC TCTCCTTCTC TCCTTCTCTC GCTATCCCTT ACACTCTTAG TCTTCAAAAA   
  
  
- AAAAAAAAAA ATTAATTTCT AGACAATTCT AATGTACATC AACTTTAAAA CTTTTTTTTT GTCGGTTTAA   
  
  
- ATCAATACAC TTTAATGTAA AAACAGGGCA TAAACAATAA GTATAAAACA AAATTAATCT CTCAATTTCA   
  
  
- CCATTAAACT ATCTTAAAAC CAACTGTTAC TTACAAAATA ATTAATCATC TATATATATA TATATATTAA   
  
  
- CAATATTCAT TCACCTTTTC ATAACAACAG CACCTCTGTT TAAATCCAAG AACTCGTTTT TGAGTTGATG   
  
  
- AGAGATCTCT CTCTCTCTCC ATCGTCATTG TGCTGAACAC ATCTTTAACA CTCCCTGCTT AGCCAATTCT   
  
  
- TCACGTACAG AACATAGAGA GAGATCTCGA TCTTTTTAGG ATTCATAAAG TAAAAAAACC TTGTTTGTCT   
  
  
- CTTTTGGGGT TCTTAATATT ATTAATTTG

+     AE-box

| Site Name | Organism | Position | Strand | Matrix score. | sequence | function |
| --- | --- | --- | --- | --- | --- | --- |
| AE-box | Arabidopsis thaliana | 723 | - | 8 | AGAAACAA | part of a module for light response |

>036006   
+ GTATACTTAA AACTAGAGAA GCCATCGATG GGCAATTGTT AGGCATAAAA ATAGTGAAAG AACCACCGGC   
  
  
+ CGGATAAACA AGAGTAAAGC TGTCTGAAAA TAAACCCTAC TCACACACAT GGTATTTCTC ATATTTTATT   
  
  
+ TTATAGTCAA TTATTAAGAT AATAAGCATT TTAACCATTT ATGTATACAT GTTCATACCA CGAGCGTTGC   
  
  
+ TTGGAGGAAC ACATTTTGGT ATCAAGTGAT GTTGCAAAAT GATGCATACA ATCAGTACAC ATCACTCTAG   
  
  
+ CTACTTACTC CATCTTGTAG TATTGCAATG GAGTAAAATA TAGCACTAGT CCCAGATTAA TTATTATGGT   
  
  
+ TCTCCTTTCA AACACATAGT TAATGGTTAT AATGATATAC TTAAGGGCAA AATATATTAC TTATACAAAA   
  
  
+ TAACTCGATG GGTTACGTAA AATACATGAA GAATTCTGTT TAATAAGTTT AGTATATGCT TGCATGAGTA   
  
  
+ TGATACAATA TATATGTGAC AAAATAAAAT ACAAGTCTAA ATTTACGACA CCACCACCCC CTTATATAGA   
  
  
+ GTTAAAACGT GAGATTCAAG AGTGTAGTTG GTGGAAAAAG ATAAATTCTA GGGTTTTTGT CTTGTAGAAA   
  
  
+ TACATCATAT ACATAAATAT TTATCTGTAC AACATGTGCG TATGTACAAT AATCATTGAC AATTAACCTA   
  
  
+ AACATTGCAA TTAACATAAA CTTTGTTTCT TCTTAAATTT TAGATGGTAA GTTCCTCCTA TCAAATCATT   
  
  
+ ACAAAATCAT AAAATCCCAA TAACACACTT TATCTATACG CAAAAAAATT TGCCGGGGAA AATATAGTAC   
  
  
+ GTGGAGGTAT ATATAGTACG TACTTTCTCC TTTGCTTTCA AAGATCATCA AATTTTAATT AGTTTATATT   
  
  
+ ATTTTATATA TATACACACA CACACTAGCA TATAGGCACA CACAAAGTGT GTGAGACATT TTTTTATTTT   
  
  
+ TGTTTTTGAA ATAGAAAGAG AGAGGAAGAG AGGAAGAGAG CGATAGGGAA TGTGAGAATC AGAAGTTTTT   
  
  
+ TTTTTTTTTT TAATTAAAGA TCTGTTAAGA TTACATGTAG TTGAAATTTT GAAAAAAAAA CAGCCAAATT   
  
  
+ TAGTTATGTG AAATTACATT TTTGTCCCGT ATTTGTTATT CATATTTTGT TTTAATTAGA GAGTTAAAGT   
  
  
+ GGTAATTTGA TAGAATTTTG GTTGACAATG AATGTTTTAT TAATTAGTAG ATATATATAT ATATATAATT   
  
  
+ GTTATAAGTA AGTGGAAAAG TATTGTTGTC GTGGAGACAA ATTTAGGTTC TTGAGCAAAA ACTCAACTAC   
  
  
+ TCTCTAGAGA GAGAGAGAGG TAGCAGTAAC ACGACTTGTG TAGAAATTGT GAGGGACGAA TCGGTTAAGA   
  
  
+ AGTGCATGTC TTGTATCTCT CTCTAGAGCT AGAAAAATCC TAAGTATTTC ATTTTTTTGG AACAAACAGA   
  
  
+ GAAAACCCCA AGAATTATAA TAATTAAAC  

- CATATGAATT TTGATCTCTT CGGTAGCTAC CCGTTAACAA TCCGTATTTT TATCACTTTC TTGGTGGCCG   
  
  
- GCCTATTTGT TCTCATTTCG ACAGACTTTT ATTTGGGATG AGTGTGTGTA CCATAAAGAG TATAAAATAA   
  
  
- AATATCAGTT AATAATTCTA TTATTCGTAA AATTGGTAAA TACATATGTA CAAGTATGGT GCTCGCAACG   
  
  
- AACCTCCTTG TGTAAAACCA TAGTTCACTA CAACGTTTTA CTACGTATGT TAGTCATGTG TAGTGAGATC   
  
  
- GATGAATGAG GTAGAACATC ATAACGTTAC CTCATTTTAT ATCGTGATCA GGGTCTAATT AATAATACCA   
  
  
- AGAGGAAAGT TTGTGTATCA ATTACCAATA TTACTATATG AATTCCCGTT TTATATAATG AATATGTTTT   
  
  
- ATTGAGCTAC CCAATGCATT TTATGTACTT CTTAAGACAA ATTATTCAAA TCATATACGA ACGTACTCAT   
  
  
- ACTATGTTAT ATATACACTG TTTTATTTTA TGTTCAGATT TAAATGCTGT GGTGGTGGGG GAATATATCT   
  
  
- CAATTTTGCA CTCTAAGTTC TCACATCAAC CACCTTTTTC TATTTAAGAT CCCAAAAACA GAACATCTTT   
  
  
- ATGTAGTATA TGTATTTATA AATAGACATG TTGTACACGC ATACATGTTA TTAGTAACTG TTAATTGGAT   
  
  
- TTGTAACGTT AATTGTATTT GAAACAAAGA AGAATTTAAA ATCTACCATT CAAGGAGGAT AGTTTAGTAA   
  
  
- TGTTTTAGTA TTTTAGGGTT ATTGTGTGAA ATAGATATGC GTTTTTTTAA ACGGCCCCTT TTATATCATG   
  
  
- CACCTCCATA TATATCATGC ATGAAAGAGG AAACGAAAGT TTCTAGTAGT TTAAAATTAA TCAAATATAA   
  
  
- TAAAATATAT ATATGTGTGT GTGTGATCGT ATATCCGTGT GTGTTTCACA CACTCTGTAA AAAAATAAAA   
  
  
- ACAAAAACTT TATCTTTCTC TCTCCTTCTC TCCTTCTCTC GCTATCCCTT ACACTCTTAG TCTTCAAAAA   
  
  
- AAAAAAAAAA ATTAATTTCT AGACAATTCT AATGTACATC AACTTTAAAA CTTTTTTTTT GTCGGTTTAA   
  
  
- ATCAATACAC TTTAATGTAA AAACAGGGCA TAAACAATAA GTATAAAACA AAATTAATCT CTCAATTTCA   
  
  
- CCATTAAACT ATCTTAAAAC CAACTGTTAC TTACAAAATA ATTAATCATC TATATATATA TATATATTAA   
  
  
- CAATATTCAT TCACCTTTTC ATAACAACAG CACCTCTGTT TAAATCCAAG AACTCGTTTT TGAGTTGATG   
  
  
- AGAGATCTCT CTCTCTCTCC ATCGTCATTG TGCTGAACAC ATCTTTAACA CTCCCTGCTT AGCCAATTCT   
  
  
- TCACGTACAG AACATAGAGA GAGATCTCGA TCTTTTTAGG ATTCATAAAG TAAAAAAACC TTGTTTGTCT   
  
  
- CTTTTGGGGT TCTTAATATT ATTAATTTG

+     AT~TATA-box

| Site Name | Organism | Position | Strand | Matrix score. | sequence | function |
| --- | --- | --- | --- | --- | --- | --- |
| AT~TATA-box | Arabidopsis thaliana | 1252 | - | 6 | TATATA |  |
| AT~TATA-box | Arabidopsis thaliana | 1250 | - | 6 | TATATA |  |
| AT~TATA-box | Arabidopsis thaliana | 1246 | - | 6 | TATATA |  |
| AT~TATA-box | Arabidopsis thaliana | 919 | - | 6 | TATATA |  |
| AT~TATA-box | Arabidopsis thaliana | 915 | - | 6 | TATATA |  |
| AT~TATA-box | Arabidopsis thaliana | 1248 | - | 6 | TATATA |  |
| AT~TATA-box | Arabidopsis thaliana | 1244 | - | 6 | TATATA |  |
| AT~TATA-box | Arabidopsis thaliana | 1242 | - | 6 | TATATA |  |
| AT~TATA-box | Arabidopsis thaliana | 917 | - | 6 | TATATA |  |
| AT~TATA-box | Arabidopsis thaliana | 913 | - | 8 | TATATAAA |  |
| AT~TATA-box | Arabidopsis thaliana | 499 | + | 6 | TATATA |  |
| AT~TATA-box | Arabidopsis thaliana | 850 | - | 6 | TATATA |  |
| AT~TATA-box | Arabidopsis thaliana | 553 | + | 6 | TATATA |  |
| AT~TATA-box | Arabidopsis thaliana | 848 | - | 6 | TATATA |  |

>036006   
+ GTATACTTAA AACTAGAGAA GCCATCGATG GGCAATTGTT AGGCATAAAA ATAGTGAAAG AACCACCGGC   
  
  
+ CGGATAAACA AGAGTAAAGC TGTCTGAAAA TAAACCCTAC TCACACACAT GGTATTTCTC ATATTTTATT   
  
  
+ TTATAGTCAA TTATTAAGAT AATAAGCATT TTAACCATTT ATGTATACAT GTTCATACCA CGAGCGTTGC   
  
  
+ TTGGAGGAAC ACATTTTGGT ATCAAGTGAT GTTGCAAAAT GATGCATACA ATCAGTACAC ATCACTCTAG   
  
  
+ CTACTTACTC CATCTTGTAG TATTGCAATG GAGTAAAATA TAGCACTAGT CCCAGATTAA TTATTATGGT   
  
  
+ TCTCCTTTCA AACACATAGT TAATGGTTAT AATGATATAC TTAAGGGCAA AATATATTAC TTATACAAAA   
  
  
+ TAACTCGATG GGTTACGTAA AATACATGAA GAATTCTGTT TAATAAGTTT AGTATATGCT TGCATGAGTA   
  
  
+ TGATACAATA TATATGTGAC AAAATAAAAT ACAAGTCTAA ATTTACGACA CCACCACCCC CTTATATAGA   
  
  
+ GTTAAAACGT GAGATTCAAG AGTGTAGTTG GTGGAAAAAG ATAAATTCTA GGGTTTTTGT CTTGTAGAAA   
  
  
+ TACATCATAT ACATAAATAT TTATCTGTAC AACATGTGCG TATGTACAAT AATCATTGAC AATTAACCTA   
  
  
+ AACATTGCAA TTAACATAAA CTTTGTTTCT TCTTAAATTT TAGATGGTAA GTTCCTCCTA TCAAATCATT   
  
  
+ ACAAAATCAT AAAATCCCAA TAACACACTT TATCTATACG CAAAAAAATT TGCCGGGGAA AATATAGTAC   
  
  
+ GTGGAGGTAT ATATAGTACG TACTTTCTCC TTTGCTTTCA AAGATCATCA AATTTTAATT AGTTTATATT   
  
  
+ ATTTTATATA TATACACACA CACACTAGCA TATAGGCACA CACAAAGTGT GTGAGACATT TTTTTATTTT   
  
  
+ TGTTTTTGAA ATAGAAAGAG AGAGGAAGAG AGGAAGAGAG CGATAGGGAA TGTGAGAATC AGAAGTTTTT   
  
  
+ TTTTTTTTTT TAATTAAAGA TCTGTTAAGA TTACATGTAG TTGAAATTTT GAAAAAAAAA CAGCCAAATT   
  
  
+ TAGTTATGTG AAATTACATT TTTGTCCCGT ATTTGTTATT CATATTTTGT TTTAATTAGA GAGTTAAAGT   
  
  
+ GGTAATTTGA TAGAATTTTG GTTGACAATG AATGTTTTAT TAATTAGTAG ATATATATAT ATATATAATT   
  
  
+ GTTATAAGTA AGTGGAAAAG TATTGTTGTC GTGGAGACAA ATTTAGGTTC TTGAGCAAAA ACTCAACTAC   
  
  
+ TCTCTAGAGA GAGAGAGAGG TAGCAGTAAC ACGACTTGTG TAGAAATTGT GAGGGACGAA TCGGTTAAGA   
  
  
+ AGTGCATGTC TTGTATCTCT CTCTAGAGCT AGAAAAATCC TAAGTATTTC ATTTTTTTGG AACAAACAGA   
  
  
+ GAAAACCCCA AGAATTATAA TAATTAAAC  

- CATATGAATT TTGATCTCTT CGGTAGCTAC CCGTTAACAA TCCGTATTTT TATCACTTTC TTGGTGGCCG   
  
  
- GCCTATTTGT TCTCATTTCG ACAGACTTTT ATTTGGGATG AGTGTGTGTA CCATAAAGAG TATAAAATAA   
  
  
- AATATCAGTT AATAATTCTA TTATTCGTAA AATTGGTAAA TACATATGTA CAAGTATGGT GCTCGCAACG   
  
  
- AACCTCCTTG TGTAAAACCA TAGTTCACTA CAACGTTTTA CTACGTATGT TAGTCATGTG TAGTGAGATC   
  
  
- GATGAATGAG GTAGAACATC ATAACGTTAC CTCATTTTAT ATCGTGATCA GGGTCTAATT AATAATACCA   
  
  
- AGAGGAAAGT TTGTGTATCA ATTACCAATA TTACTATATG AATTCCCGTT TTATATAATG AATATGTTTT   
  
  
- ATTGAGCTAC CCAATGCATT TTATGTACTT CTTAAGACAA ATTATTCAAA TCATATACGA ACGTACTCAT   
  
  
- ACTATGTTAT ATATACACTG TTTTATTTTA TGTTCAGATT TAAATGCTGT GGTGGTGGGG GAATATATCT   
  
  
- CAATTTTGCA CTCTAAGTTC TCACATCAAC CACCTTTTTC TATTTAAGAT CCCAAAAACA GAACATCTTT   
  
  
- ATGTAGTATA TGTATTTATA AATAGACATG TTGTACACGC ATACATGTTA TTAGTAACTG TTAATTGGAT   
  
  
- TTGTAACGTT AATTGTATTT GAAACAAAGA AGAATTTAAA ATCTACCATT CAAGGAGGAT AGTTTAGTAA   
  
  
- TGTTTTAGTA TTTTAGGGTT ATTGTGTGAA ATAGATATGC GTTTTTTTAA ACGGCCCCTT TTATATCATG   
  
  
- CACCTCCATA TATATCATGC ATGAAAGAGG AAACGAAAGT TTCTAGTAGT TTAAAATTAA TCAAATATAA   
  
  
- TAAAATATAT ATATGTGTGT GTGTGATCGT ATATCCGTGT GTGTTTCACA CACTCTGTAA AAAAATAAAA   
  
  
- ACAAAAACTT TATCTTTCTC TCTCCTTCTC TCCTTCTCTC GCTATCCCTT ACACTCTTAG TCTTCAAAAA   
  
  
- AAAAAAAAAA ATTAATTTCT AGACAATTCT AATGTACATC AACTTTAAAA CTTTTTTTTT GTCGGTTTAA   
  
  
- ATCAATACAC TTTAATGTAA AAACAGGGCA TAAACAATAA GTATAAAACA AAATTAATCT CTCAATTTCA   
  
  
- CCATTAAACT ATCTTAAAAC CAACTGTTAC TTACAAAATA ATTAATCATC TATATATATA TATATATTAA   
  
  
- CAATATTCAT TCACCTTTTC ATAACAACAG CACCTCTGTT TAAATCCAAG AACTCGTTTT TGAGTTGATG   
  
  
- AGAGATCTCT CTCTCTCTCC ATCGTCATTG TGCTGAACAC ATCTTTAACA CTCCCTGCTT AGCCAATTCT   
  
  
- TCACGTACAG AACATAGAGA GAGATCTCGA TCTTTTTAGG ATTCATAAAG TAAAAAAACC TTGTTTGTCT   
  
  
- CTTTTGGGGT TCTTAATATT ATTAATTTG

+     Box 4

| Site Name | Organism | Position | Strand | Matrix score. | sequence | function |
| --- | --- | --- | --- | --- | --- | --- |
| Box 4 | Petroselinum crispum | 336 | + | 6 | ATTAAT | part of a conserved DNA module involved in light responsiveness |
| Box 4 | Petroselinum crispum | 1229 | - | 6 | ATTAAT | part of a conserved DNA module involved in light responsiveness |

>036006   
+ GTATACTTAA AACTAGAGAA GCCATCGATG GGCAATTGTT AGGCATAAAA ATAGTGAAAG AACCACCGGC   
  
  
+ CGGATAAACA AGAGTAAAGC TGTCTGAAAA TAAACCCTAC TCACACACAT GGTATTTCTC ATATTTTATT   
  
  
+ TTATAGTCAA TTATTAAGAT AATAAGCATT TTAACCATTT ATGTATACAT GTTCATACCA CGAGCGTTGC   
  
  
+ TTGGAGGAAC ACATTTTGGT ATCAAGTGAT GTTGCAAAAT GATGCATACA ATCAGTACAC ATCACTCTAG   
  
  
+ CTACTTACTC CATCTTGTAG TATTGCAATG GAGTAAAATA TAGCACTAGT CCCAGATTAA TTATTATGGT   
  
  
+ TCTCCTTTCA AACACATAGT TAATGGTTAT AATGATATAC TTAAGGGCAA AATATATTAC TTATACAAAA   
  
  
+ TAACTCGATG GGTTACGTAA AATACATGAA GAATTCTGTT TAATAAGTTT AGTATATGCT TGCATGAGTA   
  
  
+ TGATACAATA TATATGTGAC AAAATAAAAT ACAAGTCTAA ATTTACGACA CCACCACCCC CTTATATAGA   
  
  
+ GTTAAAACGT GAGATTCAAG AGTGTAGTTG GTGGAAAAAG ATAAATTCTA GGGTTTTTGT CTTGTAGAAA   
  
  
+ TACATCATAT ACATAAATAT TTATCTGTAC AACATGTGCG TATGTACAAT AATCATTGAC AATTAACCTA   
  
  
+ AACATTGCAA TTAACATAAA CTTTGTTTCT TCTTAAATTT TAGATGGTAA GTTCCTCCTA TCAAATCATT   
  
  
+ ACAAAATCAT AAAATCCCAA TAACACACTT TATCTATACG CAAAAAAATT TGCCGGGGAA AATATAGTAC   
  
  
+ GTGGAGGTAT ATATAGTACG TACTTTCTCC TTTGCTTTCA AAGATCATCA AATTTTAATT AGTTTATATT   
  
  
+ ATTTTATATA TATACACACA CACACTAGCA TATAGGCACA CACAAAGTGT GTGAGACATT TTTTTATTTT   
  
  
+ TGTTTTTGAA ATAGAAAGAG AGAGGAAGAG AGGAAGAGAG CGATAGGGAA TGTGAGAATC AGAAGTTTTT   
  
  
+ TTTTTTTTTT TAATTAAAGA TCTGTTAAGA TTACATGTAG TTGAAATTTT GAAAAAAAAA CAGCCAAATT   
  
  
+ TAGTTATGTG AAATTACATT TTTGTCCCGT ATTTGTTATT CATATTTTGT TTTAATTAGA GAGTTAAAGT   
  
  
+ GGTAATTTGA TAGAATTTTG GTTGACAATG AATGTTTTAT TAATTAGTAG ATATATATAT ATATATAATT   
  
  
+ GTTATAAGTA AGTGGAAAAG TATTGTTGTC GTGGAGACAA ATTTAGGTTC TTGAGCAAAA ACTCAACTAC   
  
  
+ TCTCTAGAGA GAGAGAGAGG TAGCAGTAAC ACGACTTGTG TAGAAATTGT GAGGGACGAA TCGGTTAAGA   
  
  
+ AGTGCATGTC TTGTATCTCT CTCTAGAGCT AGAAAAATCC TAAGTATTTC ATTTTTTTGG AACAAACAGA   
  
  
+ GAAAACCCCA AGAATTATAA TAATTAAAC  

- CATATGAATT TTGATCTCTT CGGTAGCTAC CCGTTAACAA TCCGTATTTT TATCACTTTC TTGGTGGCCG   
  
  
- GCCTATTTGT TCTCATTTCG ACAGACTTTT ATTTGGGATG AGTGTGTGTA CCATAAAGAG TATAAAATAA   
  
  
- AATATCAGTT AATAATTCTA TTATTCGTAA AATTGGTAAA TACATATGTA CAAGTATGGT GCTCGCAACG   
  
  
- AACCTCCTTG TGTAAAACCA TAGTTCACTA CAACGTTTTA CTACGTATGT TAGTCATGTG TAGTGAGATC   
  
  
- GATGAATGAG GTAGAACATC ATAACGTTAC CTCATTTTAT ATCGTGATCA GGGTCTAATT AATAATACCA   
  
  
- AGAGGAAAGT TTGTGTATCA ATTACCAATA TTACTATATG AATTCCCGTT TTATATAATG AATATGTTTT   
  
  
- ATTGAGCTAC CCAATGCATT TTATGTACTT CTTAAGACAA ATTATTCAAA TCATATACGA ACGTACTCAT   
  
  
- ACTATGTTAT ATATACACTG TTTTATTTTA TGTTCAGATT TAAATGCTGT GGTGGTGGGG GAATATATCT   
  
  
- CAATTTTGCA CTCTAAGTTC TCACATCAAC CACCTTTTTC TATTTAAGAT CCCAAAAACA GAACATCTTT   
  
  
- ATGTAGTATA TGTATTTATA AATAGACATG TTGTACACGC ATACATGTTA TTAGTAACTG TTAATTGGAT   
  
  
- TTGTAACGTT AATTGTATTT GAAACAAAGA AGAATTTAAA ATCTACCATT CAAGGAGGAT AGTTTAGTAA   
  
  
- TGTTTTAGTA TTTTAGGGTT ATTGTGTGAA ATAGATATGC GTTTTTTTAA ACGGCCCCTT TTATATCATG   
  
  
- CACCTCCATA TATATCATGC ATGAAAGAGG AAACGAAAGT TTCTAGTAGT TTAAAATTAA TCAAATATAA   
  
  
- TAAAATATAT ATATGTGTGT GTGTGATCGT ATATCCGTGT GTGTTTCACA CACTCTGTAA AAAAATAAAA   
  
  
- ACAAAAACTT TATCTTTCTC TCTCCTTCTC TCCTTCTCTC GCTATCCCTT ACACTCTTAG TCTTCAAAAA   
  
  
- AAAAAAAAAA ATTAATTTCT AGACAATTCT AATGTACATC AACTTTAAAA CTTTTTTTTT GTCGGTTTAA   
  
  
- ATCAATACAC TTTAATGTAA AAACAGGGCA TAAACAATAA GTATAAAACA AAATTAATCT CTCAATTTCA   
  
  
- CCATTAAACT ATCTTAAAAC CAACTGTTAC TTACAAAATA ATTAATCATC TATATATATA TATATATTAA   
  
  
- CAATATTCAT TCACCTTTTC ATAACAACAG CACCTCTGTT TAAATCCAAG AACTCGTTTT TGAGTTGATG   
  
  
- AGAGATCTCT CTCTCTCTCC ATCGTCATTG TGCTGAACAC ATCTTTAACA CTCCCTGCTT AGCCAATTCT   
  
  
- TCACGTACAG AACATAGAGA GAGATCTCGA TCTTTTTAGG ATTCATAAAG TAAAAAAACC TTGTTTGTCT   
  
  
- CTTTTGGGGT TCTTAATATT ATTAATTTG

+     CAAT-box

| Site Name | Organism | Position | Strand | Matrix score. | sequence | function |
| --- | --- | --- | --- | --- | --- | --- |
| CAAT-box | Pisum sativum | 1195 | - | 5 | CAAAT | common cis-acting element in promoter and enhancer regions |
| CAAT-box | Nicotiana glutinosa | 1282 | - | 4 | CAAT |  |
| CAAT-box | Pisum sativum | 1151 | - | 5 | CAAAT | common cis-acting element in promoter and enhancer regions |
| CAAT-box | Nicotiana glutinosa | 1376 | - | 4 | CAAT |  |
| CAAT-box | Nicotiana glutinosa | 33 | + | 4 | CAAT |  |
| CAAT-box | Pisum sativum | 1298 | + | 5 | CAAAT | common cis-acting element in promoter and enhancer regions |
| CAAT-box | Nicotiana glutinosa | 35 | - | 4 | CAAT |  |
| CAAT-box | Nicotiana glutinosa | 1216 | + | 4 | CAAT |  |
| CAAT-box | Nicotiana glutinosa | 148 | + | 4 | CAAT |  |
| CAAT-box | Nicotiana glutinosa | 1258 | - | 4 | CAAT |  |
| CAAT-box | Nicotiana glutinosa | 259 | + | 4 | CAAT |  |
| CAAT-box | Nicotiana glutinosa | 302 | - | 4 | CAAT |  |
| CAAT-box | Nicotiana glutinosa | 306 | + | 4 | CAAT |  |
| CAAT-box | Nicotiana glutinosa | 496 | + | 4 | CAAT |  |
| CAAT-box | Nicotiana glutinosa | 677 | + | 4 | CAAT |  |
| CAAT-box | Nicotiana glutinosa | 685 | - | 4 | CAAT |  |
| CAAT-box | Nicotiana glutinosa | 690 | + | 4 | CAAT |  |
| CAAT-box | Nicotiana glutinosa | 704 | - | 4 | CAAT |  |
| CAAT-box | Nicotiana glutinosa | 708 | + | 4 | CAAT |  |
| CAAT-box | Pisum sativum | 762 | + | 5 | CAAAT | common cis-acting element in promoter and enhancer regions |
| CAAT-box | Arabidopsis thaliana | 787 | + | 5 | CCAAT | common cis-acting element in promoter and enhancer regions |
| CAAT-box | Nicotiana glutinosa | 788 | + | 4 | CAAT |  |
| CAAT-box | Pisum sativum | 818 | - | 5 | CAAAT | common cis-acting element in promoter and enhancer regions |
| CAAT-box | Pisum sativum | 889 | + | 5 | CAAAT | common cis-acting element in promoter and enhancer regions |
| CAAT-box | Pisum sativum | 1115 | + | 5 | CAAAT | common cis-acting element in promoter and enhancer regions |

>036006   
+ GTATACTTAA AACTAGAGAA GCCATCGATG GGCAATTGTT AGGCATAAAA ATAGTGAAAG AACCACCGGC   
  
  
+ CGGATAAACA AGAGTAAAGC TGTCTGAAAA TAAACCCTAC TCACACACAT GGTATTTCTC ATATTTTATT   
  
  
+ TTATAGTCAA TTATTAAGAT AATAAGCATT TTAACCATTT ATGTATACAT GTTCATACCA CGAGCGTTGC   
  
  
+ TTGGAGGAAC ACATTTTGGT ATCAAGTGAT GTTGCAAAAT GATGCATACA ATCAGTACAC ATCACTCTAG   
  
  
+ CTACTTACTC CATCTTGTAG TATTGCAATG GAGTAAAATA TAGCACTAGT CCCAGATTAA TTATTATGGT   
  
  
+ TCTCCTTTCA AACACATAGT TAATGGTTAT AATGATATAC TTAAGGGCAA AATATATTAC TTATACAAAA   
  
  
+ TAACTCGATG GGTTACGTAA AATACATGAA GAATTCTGTT TAATAAGTTT AGTATATGCT TGCATGAGTA   
  
  
+ TGATACAATA TATATGTGAC AAAATAAAAT ACAAGTCTAA ATTTACGACA CCACCACCCC CTTATATAGA   
  
  
+ GTTAAAACGT GAGATTCAAG AGTGTAGTTG GTGGAAAAAG ATAAATTCTA GGGTTTTTGT CTTGTAGAAA   
  
  
+ TACATCATAT ACATAAATAT TTATCTGTAC AACATGTGCG TATGTACAAT AATCATTGAC AATTAACCTA   
  
  
+ AACATTGCAA TTAACATAAA CTTTGTTTCT TCTTAAATTT TAGATGGTAA GTTCCTCCTA TCAAATCATT   
  
  
+ ACAAAATCAT AAAATCCCAA TAACACACTT TATCTATACG CAAAAAAATT TGCCGGGGAA AATATAGTAC   
  
  
+ GTGGAGGTAT ATATAGTACG TACTTTCTCC TTTGCTTTCA AAGATCATCA AATTTTAATT AGTTTATATT   
  
  
+ ATTTTATATA TATACACACA CACACTAGCA TATAGGCACA CACAAAGTGT GTGAGACATT TTTTTATTTT   
  
  
+ TGTTTTTGAA ATAGAAAGAG AGAGGAAGAG AGGAAGAGAG CGATAGGGAA TGTGAGAATC AGAAGTTTTT   
  
  
+ TTTTTTTTTT TAATTAAAGA TCTGTTAAGA TTACATGTAG TTGAAATTTT GAAAAAAAAA CAGCCAAATT   
  
  
+ TAGTTATGTG AAATTACATT TTTGTCCCGT ATTTGTTATT CATATTTTGT TTTAATTAGA GAGTTAAAGT   
  
  
+ GGTAATTTGA TAGAATTTTG GTTGACAATG AATGTTTTAT TAATTAGTAG ATATATATAT ATATATAATT   
  
  
+ GTTATAAGTA AGTGGAAAAG TATTGTTGTC GTGGAGACAA ATTTAGGTTC TTGAGCAAAA ACTCAACTAC   
  
  
+ TCTCTAGAGA GAGAGAGAGG TAGCAGTAAC ACGACTTGTG TAGAAATTGT GAGGGACGAA TCGGTTAAGA   
  
  
+ AGTGCATGTC TTGTATCTCT CTCTAGAGCT AGAAAAATCC TAAGTATTTC ATTTTTTTGG AACAAACAGA   
  
  
+ GAAAACCCCA AGAATTATAA TAATTAAAC  

- CATATGAATT TTGATCTCTT CGGTAGCTAC CCGTTAACAA TCCGTATTTT TATCACTTTC TTGGTGGCCG   
  
  
- GCCTATTTGT TCTCATTTCG ACAGACTTTT ATTTGGGATG AGTGTGTGTA CCATAAAGAG TATAAAATAA   
  
  
- AATATCAGTT AATAATTCTA TTATTCGTAA AATTGGTAAA TACATATGTA CAAGTATGGT GCTCGCAACG   
  
  
- AACCTCCTTG TGTAAAACCA TAGTTCACTA CAACGTTTTA CTACGTATGT TAGTCATGTG TAGTGAGATC   
  
  
- GATGAATGAG GTAGAACATC ATAACGTTAC CTCATTTTAT ATCGTGATCA GGGTCTAATT AATAATACCA   
  
  
- AGAGGAAAGT TTGTGTATCA ATTACCAATA TTACTATATG AATTCCCGTT TTATATAATG AATATGTTTT   
  
  
- ATTGAGCTAC CCAATGCATT TTATGTACTT CTTAAGACAA ATTATTCAAA TCATATACGA ACGTACTCAT   
  
  
- ACTATGTTAT ATATACACTG TTTTATTTTA TGTTCAGATT TAAATGCTGT GGTGGTGGGG GAATATATCT   
  
  
- CAATTTTGCA CTCTAAGTTC TCACATCAAC CACCTTTTTC TATTTAAGAT CCCAAAAACA GAACATCTTT   
  
  
- ATGTAGTATA TGTATTTATA AATAGACATG TTGTACACGC ATACATGTTA TTAGTAACTG TTAATTGGAT   
  
  
- TTGTAACGTT AATTGTATTT GAAACAAAGA AGAATTTAAA ATCTACCATT CAAGGAGGAT AGTTTAGTAA   
  
  
- TGTTTTAGTA TTTTAGGGTT ATTGTGTGAA ATAGATATGC GTTTTTTTAA ACGGCCCCTT TTATATCATG   
  
  
- CACCTCCATA TATATCATGC ATGAAAGAGG AAACGAAAGT TTCTAGTAGT TTAAAATTAA TCAAATATAA   
  
  
- TAAAATATAT ATATGTGTGT GTGTGATCGT ATATCCGTGT GTGTTTCACA CACTCTGTAA AAAAATAAAA   
  
  
- ACAAAAACTT TATCTTTCTC TCTCCTTCTC TCCTTCTCTC GCTATCCCTT ACACTCTTAG TCTTCAAAAA   
  
  
- AAAAAAAAAA ATTAATTTCT AGACAATTCT AATGTACATC AACTTTAAAA CTTTTTTTTT GTCGGTTTAA   
  
  
- ATCAATACAC TTTAATGTAA AAACAGGGCA TAAACAATAA GTATAAAACA AAATTAATCT CTCAATTTCA   
  
  
- CCATTAAACT ATCTTAAAAC CAACTGTTAC TTACAAAATA ATTAATCATC TATATATATA TATATATTAA   
  
  
- CAATATTCAT TCACCTTTTC ATAACAACAG CACCTCTGTT TAAATCCAAG AACTCGTTTT TGAGTTGATG   
  
  
- AGAGATCTCT CTCTCTCTCC ATCGTCATTG TGCTGAACAC ATCTTTAACA CTCCCTGCTT AGCCAATTCT   
  
  
- TCACGTACAG AACATAGAGA GAGATCTCGA TCTTTTTAGG ATTCATAAAG TAAAAAAACC TTGTTTGTCT   
  
  
- CTTTTGGGGT TCTTAATATT ATTAATTTG

+     G-Box

| Site Name | Organism | Position | Strand | Matrix score. | sequence | function |
| --- | --- | --- | --- | --- | --- | --- |
| G-Box | Pisum sativum | 566 | - | 6 | CACGTT | cis-acting regulatory element involved in light responsiveness |

>036006   
+ GTATACTTAA AACTAGAGAA GCCATCGATG GGCAATTGTT AGGCATAAAA ATAGTGAAAG AACCACCGGC   
  
  
+ CGGATAAACA AGAGTAAAGC TGTCTGAAAA TAAACCCTAC TCACACACAT GGTATTTCTC ATATTTTATT   
  
  
+ TTATAGTCAA TTATTAAGAT AATAAGCATT TTAACCATTT ATGTATACAT GTTCATACCA CGAGCGTTGC   
  
  
+ TTGGAGGAAC ACATTTTGGT ATCAAGTGAT GTTGCAAAAT GATGCATACA ATCAGTACAC ATCACTCTAG   
  
  
+ CTACTTACTC CATCTTGTAG TATTGCAATG GAGTAAAATA TAGCACTAGT CCCAGATTAA TTATTATGGT   
  
  
+ TCTCCTTTCA AACACATAGT TAATGGTTAT AATGATATAC TTAAGGGCAA AATATATTAC TTATACAAAA   
  
  
+ TAACTCGATG GGTTACGTAA AATACATGAA GAATTCTGTT TAATAAGTTT AGTATATGCT TGCATGAGTA   
  
  
+ TGATACAATA TATATGTGAC AAAATAAAAT ACAAGTCTAA ATTTACGACA CCACCACCCC CTTATATAGA   
  
  
+ GTTAAAACGT GAGATTCAAG AGTGTAGTTG GTGGAAAAAG ATAAATTCTA GGGTTTTTGT CTTGTAGAAA   
  
  
+ TACATCATAT ACATAAATAT TTATCTGTAC AACATGTGCG TATGTACAAT AATCATTGAC AATTAACCTA   
  
  
+ AACATTGCAA TTAACATAAA CTTTGTTTCT TCTTAAATTT TAGATGGTAA GTTCCTCCTA TCAAATCATT   
  
  
+ ACAAAATCAT AAAATCCCAA TAACACACTT TATCTATACG CAAAAAAATT TGCCGGGGAA AATATAGTAC   
  
  
+ GTGGAGGTAT ATATAGTACG TACTTTCTCC TTTGCTTTCA AAGATCATCA AATTTTAATT AGTTTATATT   
  
  
+ ATTTTATATA TATACACACA CACACTAGCA TATAGGCACA CACAAAGTGT GTGAGACATT TTTTTATTTT   
  
  
+ TGTTTTTGAA ATAGAAAGAG AGAGGAAGAG AGGAAGAGAG CGATAGGGAA TGTGAGAATC AGAAGTTTTT   
  
  
+ TTTTTTTTTT TAATTAAAGA TCTGTTAAGA TTACATGTAG TTGAAATTTT GAAAAAAAAA CAGCCAAATT   
  
  
+ TAGTTATGTG AAATTACATT TTTGTCCCGT ATTTGTTATT CATATTTTGT TTTAATTAGA GAGTTAAAGT   
  
  
+ GGTAATTTGA TAGAATTTTG GTTGACAATG AATGTTTTAT TAATTAGTAG ATATATATAT ATATATAATT   
  
  
+ GTTATAAGTA AGTGGAAAAG TATTGTTGTC GTGGAGACAA ATTTAGGTTC TTGAGCAAAA ACTCAACTAC   
  
  
+ TCTCTAGAGA GAGAGAGAGG TAGCAGTAAC ACGACTTGTG TAGAAATTGT GAGGGACGAA TCGGTTAAGA   
  
  
+ AGTGCATGTC TTGTATCTCT CTCTAGAGCT AGAAAAATCC TAAGTATTTC ATTTTTTTGG AACAAACAGA   
  
  
+ GAAAACCCCA AGAATTATAA TAATTAAAC  

- CATATGAATT TTGATCTCTT CGGTAGCTAC CCGTTAACAA TCCGTATTTT TATCACTTTC TTGGTGGCCG   
  
  
- GCCTATTTGT TCTCATTTCG ACAGACTTTT ATTTGGGATG AGTGTGTGTA CCATAAAGAG TATAAAATAA   
  
  
- AATATCAGTT AATAATTCTA TTATTCGTAA AATTGGTAAA TACATATGTA CAAGTATGGT GCTCGCAACG   
  
  
- AACCTCCTTG TGTAAAACCA TAGTTCACTA CAACGTTTTA CTACGTATGT TAGTCATGTG TAGTGAGATC   
  
  
- GATGAATGAG GTAGAACATC ATAACGTTAC CTCATTTTAT ATCGTGATCA GGGTCTAATT AATAATACCA   
  
  
- AGAGGAAAGT TTGTGTATCA ATTACCAATA TTACTATATG AATTCCCGTT TTATATAATG AATATGTTTT   
  
  
- ATTGAGCTAC CCAATGCATT TTATGTACTT CTTAAGACAA ATTATTCAAA TCATATACGA ACGTACTCAT   
  
  
- ACTATGTTAT ATATACACTG TTTTATTTTA TGTTCAGATT TAAATGCTGT GGTGGTGGGG GAATATATCT   
  
  
- CAATTTTGCA CTCTAAGTTC TCACATCAAC CACCTTTTTC TATTTAAGAT CCCAAAAACA GAACATCTTT   
  
  
- ATGTAGTATA TGTATTTATA AATAGACATG TTGTACACGC ATACATGTTA TTAGTAACTG TTAATTGGAT   
  
  
- TTGTAACGTT AATTGTATTT GAAACAAAGA AGAATTTAAA ATCTACCATT CAAGGAGGAT AGTTTAGTAA   
  
  
- TGTTTTAGTA TTTTAGGGTT ATTGTGTGAA ATAGATATGC GTTTTTTTAA ACGGCCCCTT TTATATCATG   
  
  
- CACCTCCATA TATATCATGC ATGAAAGAGG AAACGAAAGT TTCTAGTAGT TTAAAATTAA TCAAATATAA   
  
  
- TAAAATATAT ATATGTGTGT GTGTGATCGT ATATCCGTGT GTGTTTCACA CACTCTGTAA AAAAATAAAA   
  
  
- ACAAAAACTT TATCTTTCTC TCTCCTTCTC TCCTTCTCTC GCTATCCCTT ACACTCTTAG TCTTCAAAAA   
  
  
- AAAAAAAAAA ATTAATTTCT AGACAATTCT AATGTACATC AACTTTAAAA CTTTTTTTTT GTCGGTTTAA   
  
  
- ATCAATACAC TTTAATGTAA AAACAGGGCA TAAACAATAA GTATAAAACA AAATTAATCT CTCAATTTCA   
  
  
- CCATTAAACT ATCTTAAAAC CAACTGTTAC TTACAAAATA ATTAATCATC TATATATATA TATATATTAA   
  
  
- CAATATTCAT TCACCTTTTC ATAACAACAG CACCTCTGTT TAAATCCAAG AACTCGTTTT TGAGTTGATG   
  
  
- AGAGATCTCT CTCTCTCTCC ATCGTCATTG TGCTGAACAC ATCTTTAACA CTCCCTGCTT AGCCAATTCT   
  
  
- TCACGTACAG AACATAGAGA GAGATCTCGA TCTTTTTAGG ATTCATAAAG TAAAAAAACC TTGTTTGTCT   
  
  
- CTTTTGGGGT TCTTAATATT ATTAATTTG

+     G-box

| Site Name | Organism | Position | Strand | Matrix score. | sequence | function |
| --- | --- | --- | --- | --- | --- | --- |
| G-box | Arabidopsis thaliana | 838 | + | 6 | TACGTG | cis-acting regulatory element involved in light responsiveness |
| G-box | Zea mays | 1360 | + | 6 | CACGAC | cis-acting regulatory element involved in light responsiveness |
| G-box | Zea mays | 1288 | - | 6 | CACGAC | cis-acting regulatory element involved in light responsiveness |

>036006   
+ GTATACTTAA AACTAGAGAA GCCATCGATG GGCAATTGTT AGGCATAAAA ATAGTGAAAG AACCACCGGC   
  
  
+ CGGATAAACA AGAGTAAAGC TGTCTGAAAA TAAACCCTAC TCACACACAT GGTATTTCTC ATATTTTATT   
  
  
+ TTATAGTCAA TTATTAAGAT AATAAGCATT TTAACCATTT ATGTATACAT GTTCATACCA CGAGCGTTGC   
  
  
+ TTGGAGGAAC ACATTTTGGT ATCAAGTGAT GTTGCAAAAT GATGCATACA ATCAGTACAC ATCACTCTAG   
  
  
+ CTACTTACTC CATCTTGTAG TATTGCAATG GAGTAAAATA TAGCACTAGT CCCAGATTAA TTATTATGGT   
  
  
+ TCTCCTTTCA AACACATAGT TAATGGTTAT AATGATATAC TTAAGGGCAA AATATATTAC TTATACAAAA   
  
  
+ TAACTCGATG GGTTACGTAA AATACATGAA GAATTCTGTT TAATAAGTTT AGTATATGCT TGCATGAGTA   
  
  
+ TGATACAATA TATATGTGAC AAAATAAAAT ACAAGTCTAA ATTTACGACA CCACCACCCC CTTATATAGA   
  
  
+ GTTAAAACGT GAGATTCAAG AGTGTAGTTG GTGGAAAAAG ATAAATTCTA GGGTTTTTGT CTTGTAGAAA   
  
  
+ TACATCATAT ACATAAATAT TTATCTGTAC AACATGTGCG TATGTACAAT AATCATTGAC AATTAACCTA   
  
  
+ AACATTGCAA TTAACATAAA CTTTGTTTCT TCTTAAATTT TAGATGGTAA GTTCCTCCTA TCAAATCATT   
  
  
+ ACAAAATCAT AAAATCCCAA TAACACACTT TATCTATACG CAAAAAAATT TGCCGGGGAA AATATAGTAC   
  
  
+ GTGGAGGTAT ATATAGTACG TACTTTCTCC TTTGCTTTCA AAGATCATCA AATTTTAATT AGTTTATATT   
  
  
+ ATTTTATATA TATACACACA CACACTAGCA TATAGGCACA CACAAAGTGT GTGAGACATT TTTTTATTTT   
  
  
+ TGTTTTTGAA ATAGAAAGAG AGAGGAAGAG AGGAAGAGAG CGATAGGGAA TGTGAGAATC AGAAGTTTTT   
  
  
+ TTTTTTTTTT TAATTAAAGA TCTGTTAAGA TTACATGTAG TTGAAATTTT GAAAAAAAAA CAGCCAAATT   
  
  
+ TAGTTATGTG AAATTACATT TTTGTCCCGT ATTTGTTATT CATATTTTGT TTTAATTAGA GAGTTAAAGT   
  
  
+ GGTAATTTGA TAGAATTTTG GTTGACAATG AATGTTTTAT TAATTAGTAG ATATATATAT ATATATAATT   
  
  
+ GTTATAAGTA AGTGGAAAAG TATTGTTGTC GTGGAGACAA ATTTAGGTTC TTGAGCAAAA ACTCAACTAC   
  
  
+ TCTCTAGAGA GAGAGAGAGG TAGCAGTAAC ACGACTTGTG TAGAAATTGT GAGGGACGAA TCGGTTAAGA   
  
  
+ AGTGCATGTC TTGTATCTCT CTCTAGAGCT AGAAAAATCC TAAGTATTTC ATTTTTTTGG AACAAACAGA   
  
  
+ GAAAACCCCA AGAATTATAA TAATTAAAC  

- CATATGAATT TTGATCTCTT CGGTAGCTAC CCGTTAACAA TCCGTATTTT TATCACTTTC TTGGTGGCCG   
  
  
- GCCTATTTGT TCTCATTTCG ACAGACTTTT ATTTGGGATG AGTGTGTGTA CCATAAAGAG TATAAAATAA   
  
  
- AATATCAGTT AATAATTCTA TTATTCGTAA AATTGGTAAA TACATATGTA CAAGTATGGT GCTCGCAACG   
  
  
- AACCTCCTTG TGTAAAACCA TAGTTCACTA CAACGTTTTA CTACGTATGT TAGTCATGTG TAGTGAGATC   
  
  
- GATGAATGAG GTAGAACATC ATAACGTTAC CTCATTTTAT ATCGTGATCA GGGTCTAATT AATAATACCA   
  
  
- AGAGGAAAGT TTGTGTATCA ATTACCAATA TTACTATATG AATTCCCGTT TTATATAATG AATATGTTTT   
  
  
- ATTGAGCTAC CCAATGCATT TTATGTACTT CTTAAGACAA ATTATTCAAA TCATATACGA ACGTACTCAT   
  
  
- ACTATGTTAT ATATACACTG TTTTATTTTA TGTTCAGATT TAAATGCTGT GGTGGTGGGG GAATATATCT   
  
  
- CAATTTTGCA CTCTAAGTTC TCACATCAAC CACCTTTTTC TATTTAAGAT CCCAAAAACA GAACATCTTT   
  
  
- ATGTAGTATA TGTATTTATA AATAGACATG TTGTACACGC ATACATGTTA TTAGTAACTG TTAATTGGAT   
  
  
- TTGTAACGTT AATTGTATTT GAAACAAAGA AGAATTTAAA ATCTACCATT CAAGGAGGAT AGTTTAGTAA   
  
  
- TGTTTTAGTA TTTTAGGGTT ATTGTGTGAA ATAGATATGC GTTTTTTTAA ACGGCCCCTT TTATATCATG   
  
  
- CACCTCCATA TATATCATGC ATGAAAGAGG AAACGAAAGT TTCTAGTAGT TTAAAATTAA TCAAATATAA   
  
  
- TAAAATATAT ATATGTGTGT GTGTGATCGT ATATCCGTGT GTGTTTCACA CACTCTGTAA AAAAATAAAA   
  
  
- ACAAAAACTT TATCTTTCTC TCTCCTTCTC TCCTTCTCTC GCTATCCCTT ACACTCTTAG TCTTCAAAAA   
  
  
- AAAAAAAAAA ATTAATTTCT AGACAATTCT AATGTACATC AACTTTAAAA CTTTTTTTTT GTCGGTTTAA   
  
  
- ATCAATACAC TTTAATGTAA AAACAGGGCA TAAACAATAA GTATAAAACA AAATTAATCT CTCAATTTCA   
  
  
- CCATTAAACT ATCTTAAAAC CAACTGTTAC TTACAAAATA ATTAATCATC TATATATATA TATATATTAA   
  
  
- CAATATTCAT TCACCTTTTC ATAACAACAG CACCTCTGTT TAAATCCAAG AACTCGTTTT TGAGTTGATG   
  
  
- AGAGATCTCT CTCTCTCTCC ATCGTCATTG TGCTGAACAC ATCTTTAACA CTCCCTGCTT AGCCAATTCT   
  
  
- TCACGTACAG AACATAGAGA GAGATCTCGA TCTTTTTAGG ATTCATAAAG TAAAAAAACC TTGTTTGTCT   
  
  
- CTTTTGGGGT TCTTAATATT ATTAATTTG

+     GA-motif

| Site Name | Organism | Position | Strand | Matrix score. | sequence | function |
| --- | --- | --- | --- | --- | --- | --- |
| GA-motif | Arabidopsis thaliana | 800 | - | 8 | ATAGATAA | part of a light responsive element |

>036006   
+ GTATACTTAA AACTAGAGAA GCCATCGATG GGCAATTGTT AGGCATAAAA ATAGTGAAAG AACCACCGGC   
  
  
+ CGGATAAACA AGAGTAAAGC TGTCTGAAAA TAAACCCTAC TCACACACAT GGTATTTCTC ATATTTTATT   
  
  
+ TTATAGTCAA TTATTAAGAT AATAAGCATT TTAACCATTT ATGTATACAT GTTCATACCA CGAGCGTTGC   
  
  
+ TTGGAGGAAC ACATTTTGGT ATCAAGTGAT GTTGCAAAAT GATGCATACA ATCAGTACAC ATCACTCTAG   
  
  
+ CTACTTACTC CATCTTGTAG TATTGCAATG GAGTAAAATA TAGCACTAGT CCCAGATTAA TTATTATGGT   
  
  
+ TCTCCTTTCA AACACATAGT TAATGGTTAT AATGATATAC TTAAGGGCAA AATATATTAC TTATACAAAA   
  
  
+ TAACTCGATG GGTTACGTAA AATACATGAA GAATTCTGTT TAATAAGTTT AGTATATGCT TGCATGAGTA   
  
  
+ TGATACAATA TATATGTGAC AAAATAAAAT ACAAGTCTAA ATTTACGACA CCACCACCCC CTTATATAGA   
  
  
+ GTTAAAACGT GAGATTCAAG AGTGTAGTTG GTGGAAAAAG ATAAATTCTA GGGTTTTTGT CTTGTAGAAA   
  
  
+ TACATCATAT ACATAAATAT TTATCTGTAC AACATGTGCG TATGTACAAT AATCATTGAC AATTAACCTA   
  
  
+ AACATTGCAA TTAACATAAA CTTTGTTTCT TCTTAAATTT TAGATGGTAA GTTCCTCCTA TCAAATCATT   
  
  
+ ACAAAATCAT AAAATCCCAA TAACACACTT TATCTATACG CAAAAAAATT TGCCGGGGAA AATATAGTAC   
  
  
+ GTGGAGGTAT ATATAGTACG TACTTTCTCC TTTGCTTTCA AAGATCATCA AATTTTAATT AGTTTATATT   
  
  
+ ATTTTATATA TATACACACA CACACTAGCA TATAGGCACA CACAAAGTGT GTGAGACATT TTTTTATTTT   
  
  
+ TGTTTTTGAA ATAGAAAGAG AGAGGAAGAG AGGAAGAGAG CGATAGGGAA TGTGAGAATC AGAAGTTTTT   
  
  
+ TTTTTTTTTT TAATTAAAGA TCTGTTAAGA TTACATGTAG TTGAAATTTT GAAAAAAAAA CAGCCAAATT   
  
  
+ TAGTTATGTG AAATTACATT TTTGTCCCGT ATTTGTTATT CATATTTTGT TTTAATTAGA GAGTTAAAGT   
  
  
+ GGTAATTTGA TAGAATTTTG GTTGACAATG AATGTTTTAT TAATTAGTAG ATATATATAT ATATATAATT   
  
  
+ GTTATAAGTA AGTGGAAAAG TATTGTTGTC GTGGAGACAA ATTTAGGTTC TTGAGCAAAA ACTCAACTAC   
  
  
+ TCTCTAGAGA GAGAGAGAGG TAGCAGTAAC ACGACTTGTG TAGAAATTGT GAGGGACGAA TCGGTTAAGA   
  
  
+ AGTGCATGTC TTGTATCTCT CTCTAGAGCT AGAAAAATCC TAAGTATTTC ATTTTTTTGG AACAAACAGA   
  
  
+ GAAAACCCCA AGAATTATAA TAATTAAAC  

- CATATGAATT TTGATCTCTT CGGTAGCTAC CCGTTAACAA TCCGTATTTT TATCACTTTC TTGGTGGCCG   
  
  
- GCCTATTTGT TCTCATTTCG ACAGACTTTT ATTTGGGATG AGTGTGTGTA CCATAAAGAG TATAAAATAA   
  
  
- AATATCAGTT AATAATTCTA TTATTCGTAA AATTGGTAAA TACATATGTA CAAGTATGGT GCTCGCAACG   
  
  
- AACCTCCTTG TGTAAAACCA TAGTTCACTA CAACGTTTTA CTACGTATGT TAGTCATGTG TAGTGAGATC   
  
  
- GATGAATGAG GTAGAACATC ATAACGTTAC CTCATTTTAT ATCGTGATCA GGGTCTAATT AATAATACCA   
  
  
- AGAGGAAAGT TTGTGTATCA ATTACCAATA TTACTATATG AATTCCCGTT TTATATAATG AATATGTTTT   
  
  
- ATTGAGCTAC CCAATGCATT TTATGTACTT CTTAAGACAA ATTATTCAAA TCATATACGA ACGTACTCAT   
  
  
- ACTATGTTAT ATATACACTG TTTTATTTTA TGTTCAGATT TAAATGCTGT GGTGGTGGGG GAATATATCT   
  
  
- CAATTTTGCA CTCTAAGTTC TCACATCAAC CACCTTTTTC TATTTAAGAT CCCAAAAACA GAACATCTTT   
  
  
- ATGTAGTATA TGTATTTATA AATAGACATG TTGTACACGC ATACATGTTA TTAGTAACTG TTAATTGGAT   
  
  
- TTGTAACGTT AATTGTATTT GAAACAAAGA AGAATTTAAA ATCTACCATT CAAGGAGGAT AGTTTAGTAA   
  
  
- TGTTTTAGTA TTTTAGGGTT ATTGTGTGAA ATAGATATGC GTTTTTTTAA ACGGCCCCTT TTATATCATG   
  
  
- CACCTCCATA TATATCATGC ATGAAAGAGG AAACGAAAGT TTCTAGTAGT TTAAAATTAA TCAAATATAA   
  
  
- TAAAATATAT ATATGTGTGT GTGTGATCGT ATATCCGTGT GTGTTTCACA CACTCTGTAA AAAAATAAAA   
  
  
- ACAAAAACTT TATCTTTCTC TCTCCTTCTC TCCTTCTCTC GCTATCCCTT ACACTCTTAG TCTTCAAAAA   
  
  
- AAAAAAAAAA ATTAATTTCT AGACAATTCT AATGTACATC AACTTTAAAA CTTTTTTTTT GTCGGTTTAA   
  
  
- ATCAATACAC TTTAATGTAA AAACAGGGCA TAAACAATAA GTATAAAACA AAATTAATCT CTCAATTTCA   
  
  
- CCATTAAACT ATCTTAAAAC CAACTGTTAC TTACAAAATA ATTAATCATC TATATATATA TATATATTAA   
  
  
- CAATATTCAT TCACCTTTTC ATAACAACAG CACCTCTGTT TAAATCCAAG AACTCGTTTT TGAGTTGATG   
  
  
- AGAGATCTCT CTCTCTCTCC ATCGTCATTG TGCTGAACAC ATCTTTAACA CTCCCTGCTT AGCCAATTCT   
  
  
- TCACGTACAG AACATAGAGA GAGATCTCGA TCTTTTTAGG ATTCATAAAG TAAAAAAACC TTGTTTGTCT   
  
  
- CTTTTGGGGT TCTTAATATT ATTAATTTG

+     GATA-motif

| Site Name | Organism | Position | Strand | Matrix score. | sequence | function |
| --- | --- | --- | --- | --- | --- | --- |
| GATA-motif | Arabidopsis thaliana | 756 | - | 7 | GATAGGA | part of a light responsive element |
| GATA-motif | Pisum sativum | 1022 | + | 7 | GATAGGG | part of a light responsive element |

>036006   
+ GTATACTTAA AACTAGAGAA GCCATCGATG GGCAATTGTT AGGCATAAAA ATAGTGAAAG AACCACCGGC   
  
  
+ CGGATAAACA AGAGTAAAGC TGTCTGAAAA TAAACCCTAC TCACACACAT GGTATTTCTC ATATTTTATT   
  
  
+ TTATAGTCAA TTATTAAGAT AATAAGCATT TTAACCATTT ATGTATACAT GTTCATACCA CGAGCGTTGC   
  
  
+ TTGGAGGAAC ACATTTTGGT ATCAAGTGAT GTTGCAAAAT GATGCATACA ATCAGTACAC ATCACTCTAG   
  
  
+ CTACTTACTC CATCTTGTAG TATTGCAATG GAGTAAAATA TAGCACTAGT CCCAGATTAA TTATTATGGT   
  
  
+ TCTCCTTTCA AACACATAGT TAATGGTTAT AATGATATAC TTAAGGGCAA AATATATTAC TTATACAAAA   
  
  
+ TAACTCGATG GGTTACGTAA AATACATGAA GAATTCTGTT TAATAAGTTT AGTATATGCT TGCATGAGTA   
  
  
+ TGATACAATA TATATGTGAC AAAATAAAAT ACAAGTCTAA ATTTACGACA CCACCACCCC CTTATATAGA   
  
  
+ GTTAAAACGT GAGATTCAAG AGTGTAGTTG GTGGAAAAAG ATAAATTCTA GGGTTTTTGT CTTGTAGAAA   
  
  
+ TACATCATAT ACATAAATAT TTATCTGTAC AACATGTGCG TATGTACAAT AATCATTGAC AATTAACCTA   
  
  
+ AACATTGCAA TTAACATAAA CTTTGTTTCT TCTTAAATTT TAGATGGTAA GTTCCTCCTA TCAAATCATT   
  
  
+ ACAAAATCAT AAAATCCCAA TAACACACTT TATCTATACG CAAAAAAATT TGCCGGGGAA AATATAGTAC   
  
  
+ GTGGAGGTAT ATATAGTACG TACTTTCTCC TTTGCTTTCA AAGATCATCA AATTTTAATT AGTTTATATT   
  
  
+ ATTTTATATA TATACACACA CACACTAGCA TATAGGCACA CACAAAGTGT GTGAGACATT TTTTTATTTT   
  
  
+ TGTTTTTGAA ATAGAAAGAG AGAGGAAGAG AGGAAGAGAG CGATAGGGAA TGTGAGAATC AGAAGTTTTT   
  
  
+ TTTTTTTTTT TAATTAAAGA TCTGTTAAGA TTACATGTAG TTGAAATTTT GAAAAAAAAA CAGCCAAATT   
  
  
+ TAGTTATGTG AAATTACATT TTTGTCCCGT ATTTGTTATT CATATTTTGT TTTAATTAGA GAGTTAAAGT   
  
  
+ GGTAATTTGA TAGAATTTTG GTTGACAATG AATGTTTTAT TAATTAGTAG ATATATATAT ATATATAATT   
  
  
+ GTTATAAGTA AGTGGAAAAG TATTGTTGTC GTGGAGACAA ATTTAGGTTC TTGAGCAAAA ACTCAACTAC   
  
  
+ TCTCTAGAGA GAGAGAGAGG TAGCAGTAAC ACGACTTGTG TAGAAATTGT GAGGGACGAA TCGGTTAAGA   
  
  
+ AGTGCATGTC TTGTATCTCT CTCTAGAGCT AGAAAAATCC TAAGTATTTC ATTTTTTTGG AACAAACAGA   
  
  
+ GAAAACCCCA AGAATTATAA TAATTAAAC  

- CATATGAATT TTGATCTCTT CGGTAGCTAC CCGTTAACAA TCCGTATTTT TATCACTTTC TTGGTGGCCG   
  
  
- GCCTATTTGT TCTCATTTCG ACAGACTTTT ATTTGGGATG AGTGTGTGTA CCATAAAGAG TATAAAATAA   
  
  
- AATATCAGTT AATAATTCTA TTATTCGTAA AATTGGTAAA TACATATGTA CAAGTATGGT GCTCGCAACG   
  
  
- AACCTCCTTG TGTAAAACCA TAGTTCACTA CAACGTTTTA CTACGTATGT TAGTCATGTG TAGTGAGATC   
  
  
- GATGAATGAG GTAGAACATC ATAACGTTAC CTCATTTTAT ATCGTGATCA GGGTCTAATT AATAATACCA   
  
  
- AGAGGAAAGT TTGTGTATCA ATTACCAATA TTACTATATG AATTCCCGTT TTATATAATG AATATGTTTT   
  
  
- ATTGAGCTAC CCAATGCATT TTATGTACTT CTTAAGACAA ATTATTCAAA TCATATACGA ACGTACTCAT   
  
  
- ACTATGTTAT ATATACACTG TTTTATTTTA TGTTCAGATT TAAATGCTGT GGTGGTGGGG GAATATATCT   
  
  
- CAATTTTGCA CTCTAAGTTC TCACATCAAC CACCTTTTTC TATTTAAGAT CCCAAAAACA GAACATCTTT   
  
  
- ATGTAGTATA TGTATTTATA AATAGACATG TTGTACACGC ATACATGTTA TTAGTAACTG TTAATTGGAT   
  
  
- TTGTAACGTT AATTGTATTT GAAACAAAGA AGAATTTAAA ATCTACCATT CAAGGAGGAT AGTTTAGTAA   
  
  
- TGTTTTAGTA TTTTAGGGTT ATTGTGTGAA ATAGATATGC GTTTTTTTAA ACGGCCCCTT TTATATCATG   
  
  
- CACCTCCATA TATATCATGC ATGAAAGAGG AAACGAAAGT TTCTAGTAGT TTAAAATTAA TCAAATATAA   
  
  
- TAAAATATAT ATATGTGTGT GTGTGATCGT ATATCCGTGT GTGTTTCACA CACTCTGTAA AAAAATAAAA   
  
  
- ACAAAAACTT TATCTTTCTC TCTCCTTCTC TCCTTCTCTC GCTATCCCTT ACACTCTTAG TCTTCAAAAA   
  
  
- AAAAAAAAAA ATTAATTTCT AGACAATTCT AATGTACATC AACTTTAAAA CTTTTTTTTT GTCGGTTTAA   
  
  
- ATCAATACAC TTTAATGTAA AAACAGGGCA TAAACAATAA GTATAAAACA AAATTAATCT CTCAATTTCA   
  
  
- CCATTAAACT ATCTTAAAAC CAACTGTTAC TTACAAAATA ATTAATCATC TATATATATA TATATATTAA   
  
  
- CAATATTCAT TCACCTTTTC ATAACAACAG CACCTCTGTT TAAATCCAAG AACTCGTTTT TGAGTTGATG   
  
  
- AGAGATCTCT CTCTCTCTCC ATCGTCATTG TGCTGAACAC ATCTTTAACA CTCCCTGCTT AGCCAATTCT   
  
  
- TCACGTACAG AACATAGAGA GAGATCTCGA TCTTTTTAGG ATTCATAAAG TAAAAAAACC TTGTTTGTCT   
  
  
- CTTTTGGGGT TCTTAATATT ATTAATTTG

+     GT1-motif

| Site Name | Organism | Position | Strand | Matrix score. | sequence | function |
| --- | --- | --- | --- | --- | --- | --- |
| GT1-motif | Avena sativa | 692 | - | 7 | GGTTAAT | light responsive element |
| GT1-motif | Arabidopsis thaliana | 171 | - | 6 | GGTTAA | light responsive element |
| GT1-motif | Arabidopsis thaliana | 693 | - | 6 | GGTTAA | light responsive element |
| GT1-motif | Arabidopsis thaliana | 1393 | + | 6 | GGTTAA | light responsive element |

>036006   
+ GTATACTTAA AACTAGAGAA GCCATCGATG GGCAATTGTT AGGCATAAAA ATAGTGAAAG AACCACCGGC   
  
  
+ CGGATAAACA AGAGTAAAGC TGTCTGAAAA TAAACCCTAC TCACACACAT GGTATTTCTC ATATTTTATT   
  
  
+ TTATAGTCAA TTATTAAGAT AATAAGCATT TTAACCATTT ATGTATACAT GTTCATACCA CGAGCGTTGC   
  
  
+ TTGGAGGAAC ACATTTTGGT ATCAAGTGAT GTTGCAAAAT GATGCATACA ATCAGTACAC ATCACTCTAG   
  
  
+ CTACTTACTC CATCTTGTAG TATTGCAATG GAGTAAAATA TAGCACTAGT CCCAGATTAA TTATTATGGT   
  
  
+ TCTCCTTTCA AACACATAGT TAATGGTTAT AATGATATAC TTAAGGGCAA AATATATTAC TTATACAAAA   
  
  
+ TAACTCGATG GGTTACGTAA AATACATGAA GAATTCTGTT TAATAAGTTT AGTATATGCT TGCATGAGTA   
  
  
+ TGATACAATA TATATGTGAC AAAATAAAAT ACAAGTCTAA ATTTACGACA CCACCACCCC CTTATATAGA   
  
  
+ GTTAAAACGT GAGATTCAAG AGTGTAGTTG GTGGAAAAAG ATAAATTCTA GGGTTTTTGT CTTGTAGAAA   
  
  
+ TACATCATAT ACATAAATAT TTATCTGTAC AACATGTGCG TATGTACAAT AATCATTGAC AATTAACCTA   
  
  
+ AACATTGCAA TTAACATAAA CTTTGTTTCT TCTTAAATTT TAGATGGTAA GTTCCTCCTA TCAAATCATT   
  
  
+ ACAAAATCAT AAAATCCCAA TAACACACTT TATCTATACG CAAAAAAATT TGCCGGGGAA AATATAGTAC   
  
  
+ GTGGAGGTAT ATATAGTACG TACTTTCTCC TTTGCTTTCA AAGATCATCA AATTTTAATT AGTTTATATT   
  
  
+ ATTTTATATA TATACACACA CACACTAGCA TATAGGCACA CACAAAGTGT GTGAGACATT TTTTTATTTT   
  
  
+ TGTTTTTGAA ATAGAAAGAG AGAGGAAGAG AGGAAGAGAG CGATAGGGAA TGTGAGAATC AGAAGTTTTT   
  
  
+ TTTTTTTTTT TAATTAAAGA TCTGTTAAGA TTACATGTAG TTGAAATTTT GAAAAAAAAA CAGCCAAATT   
  
  
+ TAGTTATGTG AAATTACATT TTTGTCCCGT ATTTGTTATT CATATTTTGT TTTAATTAGA GAGTTAAAGT   
  
  
+ GGTAATTTGA TAGAATTTTG GTTGACAATG AATGTTTTAT TAATTAGTAG ATATATATAT ATATATAATT   
  
  
+ GTTATAAGTA AGTGGAAAAG TATTGTTGTC GTGGAGACAA ATTTAGGTTC TTGAGCAAAA ACTCAACTAC   
  
  
+ TCTCTAGAGA GAGAGAGAGG TAGCAGTAAC ACGACTTGTG TAGAAATTGT GAGGGACGAA TCGGTTAAGA   
  
  
+ AGTGCATGTC TTGTATCTCT CTCTAGAGCT AGAAAAATCC TAAGTATTTC ATTTTTTTGG AACAAACAGA   
  
  
+ GAAAACCCCA AGAATTATAA TAATTAAAC  

- CATATGAATT TTGATCTCTT CGGTAGCTAC CCGTTAACAA TCCGTATTTT TATCACTTTC TTGGTGGCCG   
  
  
- GCCTATTTGT TCTCATTTCG ACAGACTTTT ATTTGGGATG AGTGTGTGTA CCATAAAGAG TATAAAATAA   
  
  
- AATATCAGTT AATAATTCTA TTATTCGTAA AATTGGTAAA TACATATGTA CAAGTATGGT GCTCGCAACG   
  
  
- AACCTCCTTG TGTAAAACCA TAGTTCACTA CAACGTTTTA CTACGTATGT TAGTCATGTG TAGTGAGATC   
  
  
- GATGAATGAG GTAGAACATC ATAACGTTAC CTCATTTTAT ATCGTGATCA GGGTCTAATT AATAATACCA   
  
  
- AGAGGAAAGT TTGTGTATCA ATTACCAATA TTACTATATG AATTCCCGTT TTATATAATG AATATGTTTT   
  
  
- ATTGAGCTAC CCAATGCATT TTATGTACTT CTTAAGACAA ATTATTCAAA TCATATACGA ACGTACTCAT   
  
  
- ACTATGTTAT ATATACACTG TTTTATTTTA TGTTCAGATT TAAATGCTGT GGTGGTGGGG GAATATATCT   
  
  
- CAATTTTGCA CTCTAAGTTC TCACATCAAC CACCTTTTTC TATTTAAGAT CCCAAAAACA GAACATCTTT   
  
  
- ATGTAGTATA TGTATTTATA AATAGACATG TTGTACACGC ATACATGTTA TTAGTAACTG TTAATTGGAT   
  
  
- TTGTAACGTT AATTGTATTT GAAACAAAGA AGAATTTAAA ATCTACCATT CAAGGAGGAT AGTTTAGTAA   
  
  
- TGTTTTAGTA TTTTAGGGTT ATTGTGTGAA ATAGATATGC GTTTTTTTAA ACGGCCCCTT TTATATCATG   
  
  
- CACCTCCATA TATATCATGC ATGAAAGAGG AAACGAAAGT TTCTAGTAGT TTAAAATTAA TCAAATATAA   
  
  
- TAAAATATAT ATATGTGTGT GTGTGATCGT ATATCCGTGT GTGTTTCACA CACTCTGTAA AAAAATAAAA   
  
  
- ACAAAAACTT TATCTTTCTC TCTCCTTCTC TCCTTCTCTC GCTATCCCTT ACACTCTTAG TCTTCAAAAA   
  
  
- AAAAAAAAAA ATTAATTTCT AGACAATTCT AATGTACATC AACTTTAAAA CTTTTTTTTT GTCGGTTTAA   
  
  
- ATCAATACAC TTTAATGTAA AAACAGGGCA TAAACAATAA GTATAAAACA AAATTAATCT CTCAATTTCA   
  
  
- CCATTAAACT ATCTTAAAAC CAACTGTTAC TTACAAAATA ATTAATCATC TATATATATA TATATATTAA   
  
  
- CAATATTCAT TCACCTTTTC ATAACAACAG CACCTCTGTT TAAATCCAAG AACTCGTTTT TGAGTTGATG   
  
  
- AGAGATCTCT CTCTCTCTCC ATCGTCATTG TGCTGAACAC ATCTTTAACA CTCCCTGCTT AGCCAATTCT   
  
  
- TCACGTACAG AACATAGAGA GAGATCTCGA TCTTTTTAGG ATTCATAAAG TAAAAAAACC TTGTTTGTCT   
  
  
- CTTTTGGGGT TCTTAATATT ATTAATTTG

+     MRE

| Site Name | Organism | Position | Strand | Matrix score. | sequence | function |
| --- | --- | --- | --- | --- | --- | --- |
| MRE | Petroselinum crispum | 695 | + | 7 | AACCTAA | MYB binding site involved in light responsiveness |
| MRE | Petroselinum crispum | 1303 | - | 7 | AACCTAA | MYB binding site involved in light responsiveness |

>036006   
+ GTATACTTAA AACTAGAGAA GCCATCGATG GGCAATTGTT AGGCATAAAA ATAGTGAAAG AACCACCGGC   
  
  
+ CGGATAAACA AGAGTAAAGC TGTCTGAAAA TAAACCCTAC TCACACACAT GGTATTTCTC ATATTTTATT   
  
  
+ TTATAGTCAA TTATTAAGAT AATAAGCATT TTAACCATTT ATGTATACAT GTTCATACCA CGAGCGTTGC   
  
  
+ TTGGAGGAAC ACATTTTGGT ATCAAGTGAT GTTGCAAAAT GATGCATACA ATCAGTACAC ATCACTCTAG   
  
  
+ CTACTTACTC CATCTTGTAG TATTGCAATG GAGTAAAATA TAGCACTAGT CCCAGATTAA TTATTATGGT   
  
  
+ TCTCCTTTCA AACACATAGT TAATGGTTAT AATGATATAC TTAAGGGCAA AATATATTAC TTATACAAAA   
  
  
+ TAACTCGATG GGTTACGTAA AATACATGAA GAATTCTGTT TAATAAGTTT AGTATATGCT TGCATGAGTA   
  
  
+ TGATACAATA TATATGTGAC AAAATAAAAT ACAAGTCTAA ATTTACGACA CCACCACCCC CTTATATAGA   
  
  
+ GTTAAAACGT GAGATTCAAG AGTGTAGTTG GTGGAAAAAG ATAAATTCTA GGGTTTTTGT CTTGTAGAAA   
  
  
+ TACATCATAT ACATAAATAT TTATCTGTAC AACATGTGCG TATGTACAAT AATCATTGAC AATTAACCTA   
  
  
+ AACATTGCAA TTAACATAAA CTTTGTTTCT TCTTAAATTT TAGATGGTAA GTTCCTCCTA TCAAATCATT   
  
  
+ ACAAAATCAT AAAATCCCAA TAACACACTT TATCTATACG CAAAAAAATT TGCCGGGGAA AATATAGTAC   
  
  
+ GTGGAGGTAT ATATAGTACG TACTTTCTCC TTTGCTTTCA AAGATCATCA AATTTTAATT AGTTTATATT   
  
  
+ ATTTTATATA TATACACACA CACACTAGCA TATAGGCACA CACAAAGTGT GTGAGACATT TTTTTATTTT   
  
  
+ TGTTTTTGAA ATAGAAAGAG AGAGGAAGAG AGGAAGAGAG CGATAGGGAA TGTGAGAATC AGAAGTTTTT   
  
  
+ TTTTTTTTTT TAATTAAAGA TCTGTTAAGA TTACATGTAG TTGAAATTTT GAAAAAAAAA CAGCCAAATT   
  
  
+ TAGTTATGTG AAATTACATT TTTGTCCCGT ATTTGTTATT CATATTTTGT TTTAATTAGA GAGTTAAAGT   
  
  
+ GGTAATTTGA TAGAATTTTG GTTGACAATG AATGTTTTAT TAATTAGTAG ATATATATAT ATATATAATT   
  
  
+ GTTATAAGTA AGTGGAAAAG TATTGTTGTC GTGGAGACAA ATTTAGGTTC TTGAGCAAAA ACTCAACTAC   
  
  
+ TCTCTAGAGA GAGAGAGAGG TAGCAGTAAC ACGACTTGTG TAGAAATTGT GAGGGACGAA TCGGTTAAGA   
  
  
+ AGTGCATGTC TTGTATCTCT CTCTAGAGCT AGAAAAATCC TAAGTATTTC ATTTTTTTGG AACAAACAGA   
  
  
+ GAAAACCCCA AGAATTATAA TAATTAAAC  

- CATATGAATT TTGATCTCTT CGGTAGCTAC CCGTTAACAA TCCGTATTTT TATCACTTTC TTGGTGGCCG   
  
  
- GCCTATTTGT TCTCATTTCG ACAGACTTTT ATTTGGGATG AGTGTGTGTA CCATAAAGAG TATAAAATAA   
  
  
- AATATCAGTT AATAATTCTA TTATTCGTAA AATTGGTAAA TACATATGTA CAAGTATGGT GCTCGCAACG   
  
  
- AACCTCCTTG TGTAAAACCA TAGTTCACTA CAACGTTTTA CTACGTATGT TAGTCATGTG TAGTGAGATC   
  
  
- GATGAATGAG GTAGAACATC ATAACGTTAC CTCATTTTAT ATCGTGATCA GGGTCTAATT AATAATACCA   
  
  
- AGAGGAAAGT TTGTGTATCA ATTACCAATA TTACTATATG AATTCCCGTT TTATATAATG AATATGTTTT   
  
  
- ATTGAGCTAC CCAATGCATT TTATGTACTT CTTAAGACAA ATTATTCAAA TCATATACGA ACGTACTCAT   
  
  
- ACTATGTTAT ATATACACTG TTTTATTTTA TGTTCAGATT TAAATGCTGT GGTGGTGGGG GAATATATCT   
  
  
- CAATTTTGCA CTCTAAGTTC TCACATCAAC CACCTTTTTC TATTTAAGAT CCCAAAAACA GAACATCTTT   
  
  
- ATGTAGTATA TGTATTTATA AATAGACATG TTGTACACGC ATACATGTTA TTAGTAACTG TTAATTGGAT   
  
  
- TTGTAACGTT AATTGTATTT GAAACAAAGA AGAATTTAAA ATCTACCATT CAAGGAGGAT AGTTTAGTAA   
  
  
- TGTTTTAGTA TTTTAGGGTT ATTGTGTGAA ATAGATATGC GTTTTTTTAA ACGGCCCCTT TTATATCATG   
  
  
- CACCTCCATA TATATCATGC ATGAAAGAGG AAACGAAAGT TTCTAGTAGT TTAAAATTAA TCAAATATAA   
  
  
- TAAAATATAT ATATGTGTGT GTGTGATCGT ATATCCGTGT GTGTTTCACA CACTCTGTAA AAAAATAAAA   
  
  
- ACAAAAACTT TATCTTTCTC TCTCCTTCTC TCCTTCTCTC GCTATCCCTT ACACTCTTAG TCTTCAAAAA   
  
  
- AAAAAAAAAA ATTAATTTCT AGACAATTCT AATGTACATC AACTTTAAAA CTTTTTTTTT GTCGGTTTAA   
  
  
- ATCAATACAC TTTAATGTAA AAACAGGGCA TAAACAATAA GTATAAAACA AAATTAATCT CTCAATTTCA   
  
  
- CCATTAAACT ATCTTAAAAC CAACTGTTAC TTACAAAATA ATTAATCATC TATATATATA TATATATTAA   
  
  
- CAATATTCAT TCACCTTTTC ATAACAACAG CACCTCTGTT TAAATCCAAG AACTCGTTTT TGAGTTGATG   
  
  
- AGAGATCTCT CTCTCTCTCC ATCGTCATTG TGCTGAACAC ATCTTTAACA CTCCCTGCTT AGCCAATTCT   
  
  
- TCACGTACAG AACATAGAGA GAGATCTCGA TCTTTTTAGG ATTCATAAAG TAAAAAAACC TTGTTTGTCT   
  
  
- CTTTTGGGGT TCTTAATATT ATTAATTTG

+     MYB

| Site Name | Organism | Position | Strand | Matrix score. | sequence | function |
| --- | --- | --- | --- | --- | --- | --- |
| MYB | Arabidopsis thaliana | 1209 | - | 6 | CAACCA |  |
| MYB | Arabidopsis thaliana | 374 | - | 6 | TAACCA |  |
| MYB | Arabidopsis thaliana | 172 | + | 6 | TAACCA |  |

>036006   
+ GTATACTTAA AACTAGAGAA GCCATCGATG GGCAATTGTT AGGCATAAAA ATAGTGAAAG AACCACCGGC   
  
  
+ CGGATAAACA AGAGTAAAGC TGTCTGAAAA TAAACCCTAC TCACACACAT GGTATTTCTC ATATTTTATT   
  
  
+ TTATAGTCAA TTATTAAGAT AATAAGCATT TTAACCATTT ATGTATACAT GTTCATACCA CGAGCGTTGC   
  
  
+ TTGGAGGAAC ACATTTTGGT ATCAAGTGAT GTTGCAAAAT GATGCATACA ATCAGTACAC ATCACTCTAG   
  
  
+ CTACTTACTC CATCTTGTAG TATTGCAATG GAGTAAAATA TAGCACTAGT CCCAGATTAA TTATTATGGT   
  
  
+ TCTCCTTTCA AACACATAGT TAATGGTTAT AATGATATAC TTAAGGGCAA AATATATTAC TTATACAAAA   
  
  
+ TAACTCGATG GGTTACGTAA AATACATGAA GAATTCTGTT TAATAAGTTT AGTATATGCT TGCATGAGTA   
  
  
+ TGATACAATA TATATGTGAC AAAATAAAAT ACAAGTCTAA ATTTACGACA CCACCACCCC CTTATATAGA   
  
  
+ GTTAAAACGT GAGATTCAAG AGTGTAGTTG GTGGAAAAAG ATAAATTCTA GGGTTTTTGT CTTGTAGAAA   
  
  
+ TACATCATAT ACATAAATAT TTATCTGTAC AACATGTGCG TATGTACAAT AATCATTGAC AATTAACCTA   
  
  
+ AACATTGCAA TTAACATAAA CTTTGTTTCT TCTTAAATTT TAGATGGTAA GTTCCTCCTA TCAAATCATT   
  
  
+ ACAAAATCAT AAAATCCCAA TAACACACTT TATCTATACG CAAAAAAATT TGCCGGGGAA AATATAGTAC   
  
  
+ GTGGAGGTAT ATATAGTACG TACTTTCTCC TTTGCTTTCA AAGATCATCA AATTTTAATT AGTTTATATT   
  
  
+ ATTTTATATA TATACACACA CACACTAGCA TATAGGCACA CACAAAGTGT GTGAGACATT TTTTTATTTT   
  
  
+ TGTTTTTGAA ATAGAAAGAG AGAGGAAGAG AGGAAGAGAG CGATAGGGAA TGTGAGAATC AGAAGTTTTT   
  
  
+ TTTTTTTTTT TAATTAAAGA TCTGTTAAGA TTACATGTAG TTGAAATTTT GAAAAAAAAA CAGCCAAATT   
  
  
+ TAGTTATGTG AAATTACATT TTTGTCCCGT ATTTGTTATT CATATTTTGT TTTAATTAGA GAGTTAAAGT   
  
  
+ GGTAATTTGA TAGAATTTTG GTTGACAATG AATGTTTTAT TAATTAGTAG ATATATATAT ATATATAATT   
  
  
+ GTTATAAGTA AGTGGAAAAG TATTGTTGTC GTGGAGACAA ATTTAGGTTC TTGAGCAAAA ACTCAACTAC   
  
  
+ TCTCTAGAGA GAGAGAGAGG TAGCAGTAAC ACGACTTGTG TAGAAATTGT GAGGGACGAA TCGGTTAAGA   
  
  
+ AGTGCATGTC TTGTATCTCT CTCTAGAGCT AGAAAAATCC TAAGTATTTC ATTTTTTTGG AACAAACAGA   
  
  
+ GAAAACCCCA AGAATTATAA TAATTAAAC  

- CATATGAATT TTGATCTCTT CGGTAGCTAC CCGTTAACAA TCCGTATTTT TATCACTTTC TTGGTGGCCG   
  
  
- GCCTATTTGT TCTCATTTCG ACAGACTTTT ATTTGGGATG AGTGTGTGTA CCATAAAGAG TATAAAATAA   
  
  
- AATATCAGTT AATAATTCTA TTATTCGTAA AATTGGTAAA TACATATGTA CAAGTATGGT GCTCGCAACG   
  
  
- AACCTCCTTG TGTAAAACCA TAGTTCACTA CAACGTTTTA CTACGTATGT TAGTCATGTG TAGTGAGATC   
  
  
- GATGAATGAG GTAGAACATC ATAACGTTAC CTCATTTTAT ATCGTGATCA GGGTCTAATT AATAATACCA   
  
  
- AGAGGAAAGT TTGTGTATCA ATTACCAATA TTACTATATG AATTCCCGTT TTATATAATG AATATGTTTT   
  
  
- ATTGAGCTAC CCAATGCATT TTATGTACTT CTTAAGACAA ATTATTCAAA TCATATACGA ACGTACTCAT   
  
  
- ACTATGTTAT ATATACACTG TTTTATTTTA TGTTCAGATT TAAATGCTGT GGTGGTGGGG GAATATATCT   
  
  
- CAATTTTGCA CTCTAAGTTC TCACATCAAC CACCTTTTTC TATTTAAGAT CCCAAAAACA GAACATCTTT   
  
  
- ATGTAGTATA TGTATTTATA AATAGACATG TTGTACACGC ATACATGTTA TTAGTAACTG TTAATTGGAT   
  
  
- TTGTAACGTT AATTGTATTT GAAACAAAGA AGAATTTAAA ATCTACCATT CAAGGAGGAT AGTTTAGTAA   
  
  
- TGTTTTAGTA TTTTAGGGTT ATTGTGTGAA ATAGATATGC GTTTTTTTAA ACGGCCCCTT TTATATCATG   
  
  
- CACCTCCATA TATATCATGC ATGAAAGAGG AAACGAAAGT TTCTAGTAGT TTAAAATTAA TCAAATATAA   
  
  
- TAAAATATAT ATATGTGTGT GTGTGATCGT ATATCCGTGT GTGTTTCACA CACTCTGTAA AAAAATAAAA   
  
  
- ACAAAAACTT TATCTTTCTC TCTCCTTCTC TCCTTCTCTC GCTATCCCTT ACACTCTTAG TCTTCAAAAA   
  
  
- AAAAAAAAAA ATTAATTTCT AGACAATTCT AATGTACATC AACTTTAAAA CTTTTTTTTT GTCGGTTTAA   
  
  
- ATCAATACAC TTTAATGTAA AAACAGGGCA TAAACAATAA GTATAAAACA AAATTAATCT CTCAATTTCA   
  
  
- CCATTAAACT ATCTTAAAAC CAACTGTTAC TTACAAAATA ATTAATCATC TATATATATA TATATATTAA   
  
  
- CAATATTCAT TCACCTTTTC ATAACAACAG CACCTCTGTT TAAATCCAAG AACTCGTTTT TGAGTTGATG   
  
  
- AGAGATCTCT CTCTCTCTCC ATCGTCATTG TGCTGAACAC ATCTTTAACA CTCCCTGCTT AGCCAATTCT   
  
  
- TCACGTACAG AACATAGAGA GAGATCTCGA TCTTTTTAGG ATTCATAAAG TAAAAAAACC TTGTTTGTCT   
  
  
- CTTTTGGGGT TCTTAATATT ATTAATTTG

+     MYB-like sequence

| Site Name | Organism | Position | Strand | Matrix score. | sequence | function |
| --- | --- | --- | --- | --- | --- | --- |
| MYB-like sequence | Arabidopsis thaliana | 374 | - | 6 | TAACCA |  |
| MYB-like sequence | Arabidopsis thaliana | 172 | + | 6 | TAACCA |  |

>036006   
+ GTATACTTAA AACTAGAGAA GCCATCGATG GGCAATTGTT AGGCATAAAA ATAGTGAAAG AACCACCGGC   
  
  
+ CGGATAAACA AGAGTAAAGC TGTCTGAAAA TAAACCCTAC TCACACACAT GGTATTTCTC ATATTTTATT   
  
  
+ TTATAGTCAA TTATTAAGAT AATAAGCATT TTAACCATTT ATGTATACAT GTTCATACCA CGAGCGTTGC   
  
  
+ TTGGAGGAAC ACATTTTGGT ATCAAGTGAT GTTGCAAAAT GATGCATACA ATCAGTACAC ATCACTCTAG   
  
  
+ CTACTTACTC CATCTTGTAG TATTGCAATG GAGTAAAATA TAGCACTAGT CCCAGATTAA TTATTATGGT   
  
  
+ TCTCCTTTCA AACACATAGT TAATGGTTAT AATGATATAC TTAAGGGCAA AATATATTAC TTATACAAAA   
  
  
+ TAACTCGATG GGTTACGTAA AATACATGAA GAATTCTGTT TAATAAGTTT AGTATATGCT TGCATGAGTA   
  
  
+ TGATACAATA TATATGTGAC AAAATAAAAT ACAAGTCTAA ATTTACGACA CCACCACCCC CTTATATAGA   
  
  
+ GTTAAAACGT GAGATTCAAG AGTGTAGTTG GTGGAAAAAG ATAAATTCTA GGGTTTTTGT CTTGTAGAAA   
  
  
+ TACATCATAT ACATAAATAT TTATCTGTAC AACATGTGCG TATGTACAAT AATCATTGAC AATTAACCTA   
  
  
+ AACATTGCAA TTAACATAAA CTTTGTTTCT TCTTAAATTT TAGATGGTAA GTTCCTCCTA TCAAATCATT   
  
  
+ ACAAAATCAT AAAATCCCAA TAACACACTT TATCTATACG CAAAAAAATT TGCCGGGGAA AATATAGTAC   
  
  
+ GTGGAGGTAT ATATAGTACG TACTTTCTCC TTTGCTTTCA AAGATCATCA AATTTTAATT AGTTTATATT   
  
  
+ ATTTTATATA TATACACACA CACACTAGCA TATAGGCACA CACAAAGTGT GTGAGACATT TTTTTATTTT   
  
  
+ TGTTTTTGAA ATAGAAAGAG AGAGGAAGAG AGGAAGAGAG CGATAGGGAA TGTGAGAATC AGAAGTTTTT   
  
  
+ TTTTTTTTTT TAATTAAAGA TCTGTTAAGA TTACATGTAG TTGAAATTTT GAAAAAAAAA CAGCCAAATT   
  
  
+ TAGTTATGTG AAATTACATT TTTGTCCCGT ATTTGTTATT CATATTTTGT TTTAATTAGA GAGTTAAAGT   
  
  
+ GGTAATTTGA TAGAATTTTG GTTGACAATG AATGTTTTAT TAATTAGTAG ATATATATAT ATATATAATT   
  
  
+ GTTATAAGTA AGTGGAAAAG TATTGTTGTC GTGGAGACAA ATTTAGGTTC TTGAGCAAAA ACTCAACTAC   
  
  
+ TCTCTAGAGA GAGAGAGAGG TAGCAGTAAC ACGACTTGTG TAGAAATTGT GAGGGACGAA TCGGTTAAGA   
  
  
+ AGTGCATGTC TTGTATCTCT CTCTAGAGCT AGAAAAATCC TAAGTATTTC ATTTTTTTGG AACAAACAGA   
  
  
+ GAAAACCCCA AGAATTATAA TAATTAAAC  

- CATATGAATT TTGATCTCTT CGGTAGCTAC CCGTTAACAA TCCGTATTTT TATCACTTTC TTGGTGGCCG   
  
  
- GCCTATTTGT TCTCATTTCG ACAGACTTTT ATTTGGGATG AGTGTGTGTA CCATAAAGAG TATAAAATAA   
  
  
- AATATCAGTT AATAATTCTA TTATTCGTAA AATTGGTAAA TACATATGTA CAAGTATGGT GCTCGCAACG   
  
  
- AACCTCCTTG TGTAAAACCA TAGTTCACTA CAACGTTTTA CTACGTATGT TAGTCATGTG TAGTGAGATC   
  
  
- GATGAATGAG GTAGAACATC ATAACGTTAC CTCATTTTAT ATCGTGATCA GGGTCTAATT AATAATACCA   
  
  
- AGAGGAAAGT TTGTGTATCA ATTACCAATA TTACTATATG AATTCCCGTT TTATATAATG AATATGTTTT   
  
  
- ATTGAGCTAC CCAATGCATT TTATGTACTT CTTAAGACAA ATTATTCAAA TCATATACGA ACGTACTCAT   
  
  
- ACTATGTTAT ATATACACTG TTTTATTTTA TGTTCAGATT TAAATGCTGT GGTGGTGGGG GAATATATCT   
  
  
- CAATTTTGCA CTCTAAGTTC TCACATCAAC CACCTTTTTC TATTTAAGAT CCCAAAAACA GAACATCTTT   
  
  
- ATGTAGTATA TGTATTTATA AATAGACATG TTGTACACGC ATACATGTTA TTAGTAACTG TTAATTGGAT   
  
  
- TTGTAACGTT AATTGTATTT GAAACAAAGA AGAATTTAAA ATCTACCATT CAAGGAGGAT AGTTTAGTAA   
  
  
- TGTTTTAGTA TTTTAGGGTT ATTGTGTGAA ATAGATATGC GTTTTTTTAA ACGGCCCCTT TTATATCATG   
  
  
- CACCTCCATA TATATCATGC ATGAAAGAGG AAACGAAAGT TTCTAGTAGT TTAAAATTAA TCAAATATAA   
  
  
- TAAAATATAT ATATGTGTGT GTGTGATCGT ATATCCGTGT GTGTTTCACA CACTCTGTAA AAAAATAAAA   
  
  
- ACAAAAACTT TATCTTTCTC TCTCCTTCTC TCCTTCTCTC GCTATCCCTT ACACTCTTAG TCTTCAAAAA   
  
  
- AAAAAAAAAA ATTAATTTCT AGACAATTCT AATGTACATC AACTTTAAAA CTTTTTTTTT GTCGGTTTAA   
  
  
- ATCAATACAC TTTAATGTAA AAACAGGGCA TAAACAATAA GTATAAAACA AAATTAATCT CTCAATTTCA   
  
  
- CCATTAAACT ATCTTAAAAC CAACTGTTAC TTACAAAATA ATTAATCATC TATATATATA TATATATTAA   
  
  
- CAATATTCAT TCACCTTTTC ATAACAACAG CACCTCTGTT TAAATCCAAG AACTCGTTTT TGAGTTGATG   
  
  
- AGAGATCTCT CTCTCTCTCC ATCGTCATTG TGCTGAACAC ATCTTTAACA CTCCCTGCTT AGCCAATTCT   
  
  
- TCACGTACAG AACATAGAGA GAGATCTCGA TCTTTTTAGG ATTCATAAAG TAAAAAAACC TTGTTTGTCT   
  
  
- CTTTTGGGGT TCTTAATATT ATTAATTTG

+     MYC

| Site Name | Organism | Position | Strand | Matrix score. | sequence | function |
| --- | --- | --- | --- | --- | --- | --- |
| MYC | Arabidopsis thaliana | 116 | - | 6 | CATGTG |  |
| MYC | Arabidopsis thaliana | 33 | + | 6 | CAATTG |  |
| MYC | Arabidopsis thaliana | 663 | + | 6 | CATGTG |  |

>036006   
+ GTATACTTAA AACTAGAGAA GCCATCGATG GGCAATTGTT AGGCATAAAA ATAGTGAAAG AACCACCGGC   
  
  
+ CGGATAAACA AGAGTAAAGC TGTCTGAAAA TAAACCCTAC TCACACACAT GGTATTTCTC ATATTTTATT   
  
  
+ TTATAGTCAA TTATTAAGAT AATAAGCATT TTAACCATTT ATGTATACAT GTTCATACCA CGAGCGTTGC   
  
  
+ TTGGAGGAAC ACATTTTGGT ATCAAGTGAT GTTGCAAAAT GATGCATACA ATCAGTACAC ATCACTCTAG   
  
  
+ CTACTTACTC CATCTTGTAG TATTGCAATG GAGTAAAATA TAGCACTAGT CCCAGATTAA TTATTATGGT   
  
  
+ TCTCCTTTCA AACACATAGT TAATGGTTAT AATGATATAC TTAAGGGCAA AATATATTAC TTATACAAAA   
  
  
+ TAACTCGATG GGTTACGTAA AATACATGAA GAATTCTGTT TAATAAGTTT AGTATATGCT TGCATGAGTA   
  
  
+ TGATACAATA TATATGTGAC AAAATAAAAT ACAAGTCTAA ATTTACGACA CCACCACCCC CTTATATAGA   
  
  
+ GTTAAAACGT GAGATTCAAG AGTGTAGTTG GTGGAAAAAG ATAAATTCTA GGGTTTTTGT CTTGTAGAAA   
  
  
+ TACATCATAT ACATAAATAT TTATCTGTAC AACATGTGCG TATGTACAAT AATCATTGAC AATTAACCTA   
  
  
+ AACATTGCAA TTAACATAAA CTTTGTTTCT TCTTAAATTT TAGATGGTAA GTTCCTCCTA TCAAATCATT   
  
  
+ ACAAAATCAT AAAATCCCAA TAACACACTT TATCTATACG CAAAAAAATT TGCCGGGGAA AATATAGTAC   
  
  
+ GTGGAGGTAT ATATAGTACG TACTTTCTCC TTTGCTTTCA AAGATCATCA AATTTTAATT AGTTTATATT   
  
  
+ ATTTTATATA TATACACACA CACACTAGCA TATAGGCACA CACAAAGTGT GTGAGACATT TTTTTATTTT   
  
  
+ TGTTTTTGAA ATAGAAAGAG AGAGGAAGAG AGGAAGAGAG CGATAGGGAA TGTGAGAATC AGAAGTTTTT   
  
  
+ TTTTTTTTTT TAATTAAAGA TCTGTTAAGA TTACATGTAG TTGAAATTTT GAAAAAAAAA CAGCCAAATT   
  
  
+ TAGTTATGTG AAATTACATT TTTGTCCCGT ATTTGTTATT CATATTTTGT TTTAATTAGA GAGTTAAAGT   
  
  
+ GGTAATTTGA TAGAATTTTG GTTGACAATG AATGTTTTAT TAATTAGTAG ATATATATAT ATATATAATT   
  
  
+ GTTATAAGTA AGTGGAAAAG TATTGTTGTC GTGGAGACAA ATTTAGGTTC TTGAGCAAAA ACTCAACTAC   
  
  
+ TCTCTAGAGA GAGAGAGAGG TAGCAGTAAC ACGACTTGTG TAGAAATTGT GAGGGACGAA TCGGTTAAGA   
  
  
+ AGTGCATGTC TTGTATCTCT CTCTAGAGCT AGAAAAATCC TAAGTATTTC ATTTTTTTGG AACAAACAGA   
  
  
+ GAAAACCCCA AGAATTATAA TAATTAAAC  

- CATATGAATT TTGATCTCTT CGGTAGCTAC CCGTTAACAA TCCGTATTTT TATCACTTTC TTGGTGGCCG   
  
  
- GCCTATTTGT TCTCATTTCG ACAGACTTTT ATTTGGGATG AGTGTGTGTA CCATAAAGAG TATAAAATAA   
  
  
- AATATCAGTT AATAATTCTA TTATTCGTAA AATTGGTAAA TACATATGTA CAAGTATGGT GCTCGCAACG   
  
  
- AACCTCCTTG TGTAAAACCA TAGTTCACTA CAACGTTTTA CTACGTATGT TAGTCATGTG TAGTGAGATC   
  
  
- GATGAATGAG GTAGAACATC ATAACGTTAC CTCATTTTAT ATCGTGATCA GGGTCTAATT AATAATACCA   
  
  
- AGAGGAAAGT TTGTGTATCA ATTACCAATA TTACTATATG AATTCCCGTT TTATATAATG AATATGTTTT   
  
  
- ATTGAGCTAC CCAATGCATT TTATGTACTT CTTAAGACAA ATTATTCAAA TCATATACGA ACGTACTCAT   
  
  
- ACTATGTTAT ATATACACTG TTTTATTTTA TGTTCAGATT TAAATGCTGT GGTGGTGGGG GAATATATCT   
  
  
- CAATTTTGCA CTCTAAGTTC TCACATCAAC CACCTTTTTC TATTTAAGAT CCCAAAAACA GAACATCTTT   
  
  
- ATGTAGTATA TGTATTTATA AATAGACATG TTGTACACGC ATACATGTTA TTAGTAACTG TTAATTGGAT   
  
  
- TTGTAACGTT AATTGTATTT GAAACAAAGA AGAATTTAAA ATCTACCATT CAAGGAGGAT AGTTTAGTAA   
  
  
- TGTTTTAGTA TTTTAGGGTT ATTGTGTGAA ATAGATATGC GTTTTTTTAA ACGGCCCCTT TTATATCATG   
  
  
- CACCTCCATA TATATCATGC ATGAAAGAGG AAACGAAAGT TTCTAGTAGT TTAAAATTAA TCAAATATAA   
  
  
- TAAAATATAT ATATGTGTGT GTGTGATCGT ATATCCGTGT GTGTTTCACA CACTCTGTAA AAAAATAAAA   
  
  
- ACAAAAACTT TATCTTTCTC TCTCCTTCTC TCCTTCTCTC GCTATCCCTT ACACTCTTAG TCTTCAAAAA   
  
  
- AAAAAAAAAA ATTAATTTCT AGACAATTCT AATGTACATC AACTTTAAAA CTTTTTTTTT GTCGGTTTAA   
  
  
- ATCAATACAC TTTAATGTAA AAACAGGGCA TAAACAATAA GTATAAAACA AAATTAATCT CTCAATTTCA   
  
  
- CCATTAAACT ATCTTAAAAC CAACTGTTAC TTACAAAATA ATTAATCATC TATATATATA TATATATTAA   
  
  
- CAATATTCAT TCACCTTTTC ATAACAACAG CACCTCTGTT TAAATCCAAG AACTCGTTTT TGAGTTGATG   
  
  
- AGAGATCTCT CTCTCTCTCC ATCGTCATTG TGCTGAACAC ATCTTTAACA CTCCCTGCTT AGCCAATTCT   
  
  
- TCACGTACAG AACATAGAGA GAGATCTCGA TCTTTTTAGG ATTCATAAAG TAAAAAAACC TTGTTTGTCT   
  
  
- CTTTTGGGGT TCTTAATATT ATTAATTTG

+     STRE

| Site Name | Organism | Position | Strand | Matrix score. | sequence | function |
| --- | --- | --- | --- | --- | --- | --- |
| STRE | Arabidopsis thaliana | 548 | - | 5 | AGGGG |  |

>036006   
+ GTATACTTAA AACTAGAGAA GCCATCGATG GGCAATTGTT AGGCATAAAA ATAGTGAAAG AACCACCGGC   
  
  
+ CGGATAAACA AGAGTAAAGC TGTCTGAAAA TAAACCCTAC TCACACACAT GGTATTTCTC ATATTTTATT   
  
  
+ TTATAGTCAA TTATTAAGAT AATAAGCATT TTAACCATTT ATGTATACAT GTTCATACCA CGAGCGTTGC   
  
  
+ TTGGAGGAAC ACATTTTGGT ATCAAGTGAT GTTGCAAAAT GATGCATACA ATCAGTACAC ATCACTCTAG   
  
  
+ CTACTTACTC CATCTTGTAG TATTGCAATG GAGTAAAATA TAGCACTAGT CCCAGATTAA TTATTATGGT   
  
  
+ TCTCCTTTCA AACACATAGT TAATGGTTAT AATGATATAC TTAAGGGCAA AATATATTAC TTATACAAAA   
  
  
+ TAACTCGATG GGTTACGTAA AATACATGAA GAATTCTGTT TAATAAGTTT AGTATATGCT TGCATGAGTA   
  
  
+ TGATACAATA TATATGTGAC AAAATAAAAT ACAAGTCTAA ATTTACGACA CCACCACCCC CTTATATAGA   
  
  
+ GTTAAAACGT GAGATTCAAG AGTGTAGTTG GTGGAAAAAG ATAAATTCTA GGGTTTTTGT CTTGTAGAAA   
  
  
+ TACATCATAT ACATAAATAT TTATCTGTAC AACATGTGCG TATGTACAAT AATCATTGAC AATTAACCTA   
  
  
+ AACATTGCAA TTAACATAAA CTTTGTTTCT TCTTAAATTT TAGATGGTAA GTTCCTCCTA TCAAATCATT   
  
  
+ ACAAAATCAT AAAATCCCAA TAACACACTT TATCTATACG CAAAAAAATT TGCCGGGGAA AATATAGTAC   
  
  
+ GTGGAGGTAT ATATAGTACG TACTTTCTCC TTTGCTTTCA AAGATCATCA AATTTTAATT AGTTTATATT   
  
  
+ ATTTTATATA TATACACACA CACACTAGCA TATAGGCACA CACAAAGTGT GTGAGACATT TTTTTATTTT   
  
  
+ TGTTTTTGAA ATAGAAAGAG AGAGGAAGAG AGGAAGAGAG CGATAGGGAA TGTGAGAATC AGAAGTTTTT   
  
  
+ TTTTTTTTTT TAATTAAAGA TCTGTTAAGA TTACATGTAG TTGAAATTTT GAAAAAAAAA CAGCCAAATT   
  
  
+ TAGTTATGTG AAATTACATT TTTGTCCCGT ATTTGTTATT CATATTTTGT TTTAATTAGA GAGTTAAAGT   
  
  
+ GGTAATTTGA TAGAATTTTG GTTGACAATG AATGTTTTAT TAATTAGTAG ATATATATAT ATATATAATT   
  
  
+ GTTATAAGTA AGTGGAAAAG TATTGTTGTC GTGGAGACAA ATTTAGGTTC TTGAGCAAAA ACTCAACTAC   
  
  
+ TCTCTAGAGA GAGAGAGAGG TAGCAGTAAC ACGACTTGTG TAGAAATTGT GAGGGACGAA TCGGTTAAGA   
  
  
+ AGTGCATGTC TTGTATCTCT CTCTAGAGCT AGAAAAATCC TAAGTATTTC ATTTTTTTGG AACAAACAGA   
  
  
+ GAAAACCCCA AGAATTATAA TAATTAAAC  

- CATATGAATT TTGATCTCTT CGGTAGCTAC CCGTTAACAA TCCGTATTTT TATCACTTTC TTGGTGGCCG   
  
  
- GCCTATTTGT TCTCATTTCG ACAGACTTTT ATTTGGGATG AGTGTGTGTA CCATAAAGAG TATAAAATAA   
  
  
- AATATCAGTT AATAATTCTA TTATTCGTAA AATTGGTAAA TACATATGTA CAAGTATGGT GCTCGCAACG   
  
  
- AACCTCCTTG TGTAAAACCA TAGTTCACTA CAACGTTTTA CTACGTATGT TAGTCATGTG TAGTGAGATC   
  
  
- GATGAATGAG GTAGAACATC ATAACGTTAC CTCATTTTAT ATCGTGATCA GGGTCTAATT AATAATACCA   
  
  
- AGAGGAAAGT TTGTGTATCA ATTACCAATA TTACTATATG AATTCCCGTT TTATATAATG AATATGTTTT   
  
  
- ATTGAGCTAC CCAATGCATT TTATGTACTT CTTAAGACAA ATTATTCAAA TCATATACGA ACGTACTCAT   
  
  
- ACTATGTTAT ATATACACTG TTTTATTTTA TGTTCAGATT TAAATGCTGT GGTGGTGGGG GAATATATCT   
  
  
- CAATTTTGCA CTCTAAGTTC TCACATCAAC CACCTTTTTC TATTTAAGAT CCCAAAAACA GAACATCTTT   
  
  
- ATGTAGTATA TGTATTTATA AATAGACATG TTGTACACGC ATACATGTTA TTAGTAACTG TTAATTGGAT   
  
  
- TTGTAACGTT AATTGTATTT GAAACAAAGA AGAATTTAAA ATCTACCATT CAAGGAGGAT AGTTTAGTAA   
  
  
- TGTTTTAGTA TTTTAGGGTT ATTGTGTGAA ATAGATATGC GTTTTTTTAA ACGGCCCCTT TTATATCATG   
  
  
- CACCTCCATA TATATCATGC ATGAAAGAGG AAACGAAAGT TTCTAGTAGT TTAAAATTAA TCAAATATAA   
  
  
- TAAAATATAT ATATGTGTGT GTGTGATCGT ATATCCGTGT GTGTTTCACA CACTCTGTAA AAAAATAAAA   
  
  
- ACAAAAACTT TATCTTTCTC TCTCCTTCTC TCCTTCTCTC GCTATCCCTT ACACTCTTAG TCTTCAAAAA   
  
  
- AAAAAAAAAA ATTAATTTCT AGACAATTCT AATGTACATC AACTTTAAAA CTTTTTTTTT GTCGGTTTAA   
  
  
- ATCAATACAC TTTAATGTAA AAACAGGGCA TAAACAATAA GTATAAAACA AAATTAATCT CTCAATTTCA   
  
  
- CCATTAAACT ATCTTAAAAC CAACTGTTAC TTACAAAATA ATTAATCATC TATATATATA TATATATTAA   
  
  
- CAATATTCAT TCACCTTTTC ATAACAACAG CACCTCTGTT TAAATCCAAG AACTCGTTTT TGAGTTGATG   
  
  
- AGAGATCTCT CTCTCTCTCC ATCGTCATTG TGCTGAACAC ATCTTTAACA CTCCCTGCTT AGCCAATTCT   
  
  
- TCACGTACAG AACATAGAGA GAGATCTCGA TCTTTTTAGG ATTCATAAAG TAAAAAAACC TTGTTTGTCT   
  
  
- CTTTTGGGGT TCTTAATATT ATTAATTTG

+     TATA

| Site Name | Organism | Position | Strand | Matrix score. | sequence | function |
| --- | --- | --- | --- | --- | --- | --- |
| TATA | Arabidopsis thaliana | 911 | - | 8 | TATAAAAT |  |
| TATA | Arabidopsis thaliana | 138 | - | 8 | TATAAAAT |  |

>036006   
+ GTATACTTAA AACTAGAGAA GCCATCGATG GGCAATTGTT AGGCATAAAA ATAGTGAAAG AACCACCGGC   
  
  
+ CGGATAAACA AGAGTAAAGC TGTCTGAAAA TAAACCCTAC TCACACACAT GGTATTTCTC ATATTTTATT   
  
  
+ TTATAGTCAA TTATTAAGAT AATAAGCATT TTAACCATTT ATGTATACAT GTTCATACCA CGAGCGTTGC   
  
  
+ TTGGAGGAAC ACATTTTGGT ATCAAGTGAT GTTGCAAAAT GATGCATACA ATCAGTACAC ATCACTCTAG   
  
  
+ CTACTTACTC CATCTTGTAG TATTGCAATG GAGTAAAATA TAGCACTAGT CCCAGATTAA TTATTATGGT   
  
  
+ TCTCCTTTCA AACACATAGT TAATGGTTAT AATGATATAC TTAAGGGCAA AATATATTAC TTATACAAAA   
  
  
+ TAACTCGATG GGTTACGTAA AATACATGAA GAATTCTGTT TAATAAGTTT AGTATATGCT TGCATGAGTA   
  
  
+ TGATACAATA TATATGTGAC AAAATAAAAT ACAAGTCTAA ATTTACGACA CCACCACCCC CTTATATAGA   
  
  
+ GTTAAAACGT GAGATTCAAG AGTGTAGTTG GTGGAAAAAG ATAAATTCTA GGGTTTTTGT CTTGTAGAAA   
  
  
+ TACATCATAT ACATAAATAT TTATCTGTAC AACATGTGCG TATGTACAAT AATCATTGAC AATTAACCTA   
  
  
+ AACATTGCAA TTAACATAAA CTTTGTTTCT TCTTAAATTT TAGATGGTAA GTTCCTCCTA TCAAATCATT   
  
  
+ ACAAAATCAT AAAATCCCAA TAACACACTT TATCTATACG CAAAAAAATT TGCCGGGGAA AATATAGTAC   
  
  
+ GTGGAGGTAT ATATAGTACG TACTTTCTCC TTTGCTTTCA AAGATCATCA AATTTTAATT AGTTTATATT   
  
  
+ ATTTTATATA TATACACACA CACACTAGCA TATAGGCACA CACAAAGTGT GTGAGACATT TTTTTATTTT   
  
  
+ TGTTTTTGAA ATAGAAAGAG AGAGGAAGAG AGGAAGAGAG CGATAGGGAA TGTGAGAATC AGAAGTTTTT   
  
  
+ TTTTTTTTTT TAATTAAAGA TCTGTTAAGA TTACATGTAG TTGAAATTTT GAAAAAAAAA CAGCCAAATT   
  
  
+ TAGTTATGTG AAATTACATT TTTGTCCCGT ATTTGTTATT CATATTTTGT TTTAATTAGA GAGTTAAAGT   
  
  
+ GGTAATTTGA TAGAATTTTG GTTGACAATG AATGTTTTAT TAATTAGTAG ATATATATAT ATATATAATT   
  
  
+ GTTATAAGTA AGTGGAAAAG TATTGTTGTC GTGGAGACAA ATTTAGGTTC TTGAGCAAAA ACTCAACTAC   
  
  
+ TCTCTAGAGA GAGAGAGAGG TAGCAGTAAC ACGACTTGTG TAGAAATTGT GAGGGACGAA TCGGTTAAGA   
  
  
+ AGTGCATGTC TTGTATCTCT CTCTAGAGCT AGAAAAATCC TAAGTATTTC ATTTTTTTGG AACAAACAGA   
  
  
+ GAAAACCCCA AGAATTATAA TAATTAAAC  

- CATATGAATT TTGATCTCTT CGGTAGCTAC CCGTTAACAA TCCGTATTTT TATCACTTTC TTGGTGGCCG   
  
  
- GCCTATTTGT TCTCATTTCG ACAGACTTTT ATTTGGGATG AGTGTGTGTA CCATAAAGAG TATAAAATAA   
  
  
- AATATCAGTT AATAATTCTA TTATTCGTAA AATTGGTAAA TACATATGTA CAAGTATGGT GCTCGCAACG   
  
  
- AACCTCCTTG TGTAAAACCA TAGTTCACTA CAACGTTTTA CTACGTATGT TAGTCATGTG TAGTGAGATC   
  
  
- GATGAATGAG GTAGAACATC ATAACGTTAC CTCATTTTAT ATCGTGATCA GGGTCTAATT AATAATACCA   
  
  
- AGAGGAAAGT TTGTGTATCA ATTACCAATA TTACTATATG AATTCCCGTT TTATATAATG AATATGTTTT   
  
  
- ATTGAGCTAC CCAATGCATT TTATGTACTT CTTAAGACAA ATTATTCAAA TCATATACGA ACGTACTCAT   
  
  
- ACTATGTTAT ATATACACTG TTTTATTTTA TGTTCAGATT TAAATGCTGT GGTGGTGGGG GAATATATCT   
  
  
- CAATTTTGCA CTCTAAGTTC TCACATCAAC CACCTTTTTC TATTTAAGAT CCCAAAAACA GAACATCTTT   
  
  
- ATGTAGTATA TGTATTTATA AATAGACATG TTGTACACGC ATACATGTTA TTAGTAACTG TTAATTGGAT   
  
  
- TTGTAACGTT AATTGTATTT GAAACAAAGA AGAATTTAAA ATCTACCATT CAAGGAGGAT AGTTTAGTAA   
  
  
- TGTTTTAGTA TTTTAGGGTT ATTGTGTGAA ATAGATATGC GTTTTTTTAA ACGGCCCCTT TTATATCATG   
  
  
- CACCTCCATA TATATCATGC ATGAAAGAGG AAACGAAAGT TTCTAGTAGT TTAAAATTAA TCAAATATAA   
  
  
- TAAAATATAT ATATGTGTGT GTGTGATCGT ATATCCGTGT GTGTTTCACA CACTCTGTAA AAAAATAAAA   
  
  
- ACAAAAACTT TATCTTTCTC TCTCCTTCTC TCCTTCTCTC GCTATCCCTT ACACTCTTAG TCTTCAAAAA   
  
  
- AAAAAAAAAA ATTAATTTCT AGACAATTCT AATGTACATC AACTTTAAAA CTTTTTTTTT GTCGGTTTAA   
  
  
- ATCAATACAC TTTAATGTAA AAACAGGGCA TAAACAATAA GTATAAAACA AAATTAATCT CTCAATTTCA   
  
  
- CCATTAAACT ATCTTAAAAC CAACTGTTAC TTACAAAATA ATTAATCATC TATATATATA TATATATTAA   
  
  
- CAATATTCAT TCACCTTTTC ATAACAACAG CACCTCTGTT TAAATCCAAG AACTCGTTTT TGAGTTGATG   
  
  
- AGAGATCTCT CTCTCTCTCC ATCGTCATTG TGCTGAACAC ATCTTTAACA CTCCCTGCTT AGCCAATTCT   
  
  
- TCACGTACAG AACATAGAGA GAGATCTCGA TCTTTTTAGG ATTCATAAAG TAAAAAAACC TTGTTTGTCT   
  
  
- CTTTTGGGGT TCTTAATATT ATTAATTTG

+     TATA-box

| Site Name | Organism | Position | Strand | Matrix score. | sequence | function |
| --- | --- | --- | --- | --- | --- | --- |
| TATA-box | Arabidopsis thaliana | 1262 | - | 5 | TATAA | core promoter element around -30 of transcription start |
| TATA-box | Arabidopsis thaliana | 1240 | - | 9 | taTATAAAtc | core promoter element around -30 of transcription start |
| TATA-box | Arabidopsis thaliana | 917 | - | 6 | TATATA | core promoter element around -30 of transcription start |
| TATA-box | Arabidopsis thaliana | 1252 | - | 6 | TATATA | core promoter element around -30 of transcription start |
| TATA-box | Brassica napus | 1247 | - | 6 | ATATAT | core promoter element around -30 of transcription start |
| TATA-box | Oryza sativa | 640 | + | 8 | TACATAAA | core promoter element around -30 of transcription start |
| TATA-box | Brassica napus | 1251 | - | 6 | ATATAT | core promoter element around -30 of transcription start |
| TATA-box | Brassica napus | 849 | - | 6 | ATATAT | core promoter element around -30 of transcription start |
| TATA-box | Arabidopsis thaliana | 553 | + | 6 | TATATA | core promoter element around -30 of transcription start |
| TATA-box | Arabidopsis thaliana | 377 | - | 5 | TATAA | core promoter element around -30 of transcription start |
| TATA-box | Brassica napus | 918 | - | 6 | ATATAT | core promoter element around -30 of transcription start |
| TATA-box | Brassica napus | 916 | - | 6 | ATATAT | core promoter element around -30 of transcription start |
| TATA-box | Arabidopsis thaliana | 850 | - | 6 | TATATA | core promoter element around -30 of transcription start |
| TATA-box | Arabidopsis thaliana | 184 | + | 4 | TATA | core promoter element around -30 of transcription start |
| TATA-box | Arabidopsis thaliana | 833 | - | 4 | TATA | core promoter element around -30 of transcription start |
| TATA-box | Arabidopsis thaliana | 848 | - | 6 | TATATA | core promoter element around -30 of transcription start |
| TATA-box | Brassica napus | 500 | + | 6 | ATATAT | core promoter element around -30 of transcription start |
| TATA-box | Oryza sativa | 770 | + | 7 | TACAAAA | core promoter element around -30 of transcription start |
| TATA-box | Brassica napus | 1484 | + | 6 | ATTATA | core promoter element around -30 of transcription start |
| TATA-box | Arabidopsis thaliana | 501 | + | 4 | TATA | core promoter element around -30 of transcription start |
| TATA-box | Arabidopsis thaliana | 499 | + | 6 | TATATA | core promoter element around -30 of transcription start |
| TATA-box | Arabidopsis thaliana | 141 | - | 5 | TATAA | core promoter element around -30 of transcription start |
| TATA-box | Arabidopsis thaliana | 805 | - | 4 | TATA | core promoter element around -30 of transcription start |
| TATA-box | Arabidopsis thaliana | 552 | - | 7 | TATATAA | core promoter element around -30 of transcription start |
| TATA-box | Brassica napus | 498 | + | 6 | ATATAT | core promoter element around -30 of transcription start |
| TATA-box | Arabidopsis thaliana | 852 | - | 4 | TATA | core promoter element around -30 of transcription start |
| TATA-box | Arabidopsis thaliana | 2 | + | 4 | TATA | core promoter element around -30 of transcription start |
| TATA-box | Arabidopsis thaliana | 1244 | - | 6 | TATATA | core promoter element around -30 of transcription start |
| TATA-box | Brassica napus | 1243 | - | 6 | ATATAT | core promoter element around -30 of transcription start |
| TATA-box | Arabidopsis thaliana | 1248 | - | 6 | TATATA | core promoter element around -30 of transcription start |
| TATA-box | Arabidopsis thaliana | 915 | - | 6 | TATATA | core promoter element around -30 of transcription start |
| TATA-box | Brassica napus | 1241 | - | 6 | ATATAT | core promoter element around -30 of transcription start |
| TATA-box | Arabidopsis thaliana | 638 | + | 4 | TATA | core promoter element around -30 of transcription start |
| TATA-box | Arabidopsis thaliana | 555 | + | 4 | TATA | core promoter element around -30 of transcription start |
| TATA-box | Arabidopsis thaliana | 1254 | - | 4 | TATA | core promoter element around -30 of transcription start |
| TATA-box | Brassica oleracea | 1253 | + | 6 | ATATAA | core promoter element around -30 of transcription start |
| TATA-box | Arabidopsis thaliana | 411 | - | 5 | TATAA | core promoter element around -30 of transcription start |
| TATA-box | Oryza sativa | 178 | - | 8 | TACATAAA | core promoter element around -30 of transcription start |
| TATA-box | Helianthus annuus | 903 | - | 6 | TATAAA | core promoter element around -30 of transcription start |
| TATA-box | Arabidopsis thaliana | 386 | + | 4 | TATA | core promoter element around -30 of transcription start |
| TATA-box | Arabidopsis thaliana | 914 | - | 7 | TATATAA | core promoter element around -30 of transcription start |
| TATA-box | Arabidopsis thaliana | 378 | + | 4 | TATA | core promoter element around -30 of transcription start |
| TATA-box | Arabidopsis thaliana | 1246 | - | 6 | TATATA | core promoter element around -30 of transcription start |
| TATA-box | Brassica napus | 1245 | - | 6 | ATATAT | core promoter element around -30 of transcription start |
| TATA-box | Arabidopsis thaliana | 403 | + | 4 | TATA | core promoter element around -30 of transcription start |
| TATA-box | Brassica napus | 402 | + | 6 | ATATAT | core promoter element around -30 of transcription start |
| TATA-box | Arabidopsis thaliana | 919 | - | 6 | TATATA | core promoter element around -30 of transcription start |
| TATA-box | Helianthus annuus | 182 | - | 6 | TATACA | core promoter element around -30 of transcription start |
| TATA-box | Pisum sativum | 912 | - | 7 | TATAAAA | core promoter element around -30 of transcription start |
| TATA-box | Pisum sativum | 139 | - | 7 | TATAAAA | core promoter element around -30 of transcription start |
| TATA-box | Arabidopsis thaliana | 1486 | - | 4 | TATA | core promoter element around -30 of transcription start |
| TATA-box | Oryza sativa | 414 | + | 7 | TACAAAA | core promoter element around -30 of transcription start |
| TATA-box | Arabidopsis thaliana | 1250 | - | 6 | TATATA | core promoter element around -30 of transcription start |
| TATA-box | Arabidopsis thaliana | 1242 | - | 6 | TATATA | core promoter element around -30 of transcription start |
| TATA-box | Arabidopsis thaliana | 473 | + | 4 | TATA | core promoter element around -30 of transcription start |
| TATA-box | Arabidopsis thaliana | 319 | + | 4 | TATA | core promoter element around -30 of transcription start |
| TATA-box | Arabidopsis thaliana | 921 | - | 4 | TATA | core promoter element around -30 of transcription start |
| TATA-box | Arabidopsis thaliana | 412 | + | 4 | TATA | core promoter element around -30 of transcription start |
| TATA-box | Arabidopsis thaliana | 1263 | - | 4 | TATA | core promoter element around -30 of transcription start |
| TATA-box | Arabidopsis thaliana | 142 | + | 4 | TATA | core promoter element around -30 of transcription start |
| TATA-box | Arabidopsis thaliana | 941 | - | 4 | TATA | core promoter element around -30 of transcription start |
| TATA-box | Helianthus annuus | 140 | - | 6 | TATAAA | core promoter element around -30 of transcription start |
| TATA-box | Brassica napus | 1249 | - | 6 | ATATAT | core promoter element around -30 of transcription start |
| TATA-box | Helianthus annuus | 913 | - | 6 | TATAAA | core promoter element around -30 of transcription start |
| TATA-box | Arabidopsis thaliana | 1485 | - | 5 | TATAA | core promoter element around -30 of transcription start |
| TATA-box | Arabidopsis thaliana | 904 | - | 5 | TATAA | core promoter element around -30 of transcription start |
| TATA-box | Arabidopsis thaliana | 905 | - | 4 | TATA | core promoter element around -30 of transcription start |

>036006   
+ GTATACTTAA AACTAGAGAA GCCATCGATG GGCAATTGTT AGGCATAAAA ATAGTGAAAG AACCACCGGC   
  
  
+ CGGATAAACA AGAGTAAAGC TGTCTGAAAA TAAACCCTAC TCACACACAT GGTATTTCTC ATATTTTATT   
  
  
+ TTATAGTCAA TTATTAAGAT AATAAGCATT TTAACCATTT ATGTATACAT GTTCATACCA CGAGCGTTGC   
  
  
+ TTGGAGGAAC ACATTTTGGT ATCAAGTGAT GTTGCAAAAT GATGCATACA ATCAGTACAC ATCACTCTAG   
  
  
+ CTACTTACTC CATCTTGTAG TATTGCAATG GAGTAAAATA TAGCACTAGT CCCAGATTAA TTATTATGGT   
  
  
+ TCTCCTTTCA AACACATAGT TAATGGTTAT AATGATATAC TTAAGGGCAA AATATATTAC TTATACAAAA   
  
  
+ TAACTCGATG GGTTACGTAA AATACATGAA GAATTCTGTT TAATAAGTTT AGTATATGCT TGCATGAGTA   
  
  
+ TGATACAATA TATATGTGAC AAAATAAAAT ACAAGTCTAA ATTTACGACA CCACCACCCC CTTATATAGA   
  
  
+ GTTAAAACGT GAGATTCAAG AGTGTAGTTG GTGGAAAAAG ATAAATTCTA GGGTTTTTGT CTTGTAGAAA   
  
  
+ TACATCATAT ACATAAATAT TTATCTGTAC AACATGTGCG TATGTACAAT AATCATTGAC AATTAACCTA   
  
  
+ AACATTGCAA TTAACATAAA CTTTGTTTCT TCTTAAATTT TAGATGGTAA GTTCCTCCTA TCAAATCATT   
  
  
+ ACAAAATCAT AAAATCCCAA TAACACACTT TATCTATACG CAAAAAAATT TGCCGGGGAA AATATAGTAC   
  
  
+ GTGGAGGTAT ATATAGTACG TACTTTCTCC TTTGCTTTCA AAGATCATCA AATTTTAATT AGTTTATATT   
  
  
+ ATTTTATATA TATACACACA CACACTAGCA TATAGGCACA CACAAAGTGT GTGAGACATT TTTTTATTTT   
  
  
+ TGTTTTTGAA ATAGAAAGAG AGAGGAAGAG AGGAAGAGAG CGATAGGGAA TGTGAGAATC AGAAGTTTTT   
  
  
+ TTTTTTTTTT TAATTAAAGA TCTGTTAAGA TTACATGTAG TTGAAATTTT GAAAAAAAAA CAGCCAAATT   
  
  
+ TAGTTATGTG AAATTACATT TTTGTCCCGT ATTTGTTATT CATATTTTGT TTTAATTAGA GAGTTAAAGT   
  
  
+ GGTAATTTGA TAGAATTTTG GTTGACAATG AATGTTTTAT TAATTAGTAG ATATATATAT ATATATAATT   
  
  
+ GTTATAAGTA AGTGGAAAAG TATTGTTGTC GTGGAGACAA ATTTAGGTTC TTGAGCAAAA ACTCAACTAC   
  
  
+ TCTCTAGAGA GAGAGAGAGG TAGCAGTAAC ACGACTTGTG TAGAAATTGT GAGGGACGAA TCGGTTAAGA   
  
  
+ AGTGCATGTC TTGTATCTCT CTCTAGAGCT AGAAAAATCC TAAGTATTTC ATTTTTTTGG AACAAACAGA   
  
  
+ GAAAACCCCA AGAATTATAA TAATTAAAC  

- CATATGAATT TTGATCTCTT CGGTAGCTAC CCGTTAACAA TCCGTATTTT TATCACTTTC TTGGTGGCCG   
  
  
- GCCTATTTGT TCTCATTTCG ACAGACTTTT ATTTGGGATG AGTGTGTGTA CCATAAAGAG TATAAAATAA   
  
  
- AATATCAGTT AATAATTCTA TTATTCGTAA AATTGGTAAA TACATATGTA CAAGTATGGT GCTCGCAACG   
  
  
- AACCTCCTTG TGTAAAACCA TAGTTCACTA CAACGTTTTA CTACGTATGT TAGTCATGTG TAGTGAGATC   
  
  
- GATGAATGAG GTAGAACATC ATAACGTTAC CTCATTTTAT ATCGTGATCA GGGTCTAATT AATAATACCA   
  
  
- AGAGGAAAGT TTGTGTATCA ATTACCAATA TTACTATATG AATTCCCGTT TTATATAATG AATATGTTTT   
  
  
- ATTGAGCTAC CCAATGCATT TTATGTACTT CTTAAGACAA ATTATTCAAA TCATATACGA ACGTACTCAT   
  
  
- ACTATGTTAT ATATACACTG TTTTATTTTA TGTTCAGATT TAAATGCTGT GGTGGTGGGG GAATATATCT   
  
  
- CAATTTTGCA CTCTAAGTTC TCACATCAAC CACCTTTTTC TATTTAAGAT CCCAAAAACA GAACATCTTT   
  
  
- ATGTAGTATA TGTATTTATA AATAGACATG TTGTACACGC ATACATGTTA TTAGTAACTG TTAATTGGAT   
  
  
- TTGTAACGTT AATTGTATTT GAAACAAAGA AGAATTTAAA ATCTACCATT CAAGGAGGAT AGTTTAGTAA   
  
  
- TGTTTTAGTA TTTTAGGGTT ATTGTGTGAA ATAGATATGC GTTTTTTTAA ACGGCCCCTT TTATATCATG   
  
  
- CACCTCCATA TATATCATGC ATGAAAGAGG AAACGAAAGT TTCTAGTAGT TTAAAATTAA TCAAATATAA   
  
  
- TAAAATATAT ATATGTGTGT GTGTGATCGT ATATCCGTGT GTGTTTCACA CACTCTGTAA AAAAATAAAA   
  
  
- ACAAAAACTT TATCTTTCTC TCTCCTTCTC TCCTTCTCTC GCTATCCCTT ACACTCTTAG TCTTCAAAAA   
  
  
- AAAAAAAAAA ATTAATTTCT AGACAATTCT AATGTACATC AACTTTAAAA CTTTTTTTTT GTCGGTTTAA   
  
  
- ATCAATACAC TTTAATGTAA AAACAGGGCA TAAACAATAA GTATAAAACA AAATTAATCT CTCAATTTCA   
  
  
- CCATTAAACT ATCTTAAAAC CAACTGTTAC TTACAAAATA ATTAATCATC TATATATATA TATATATTAA   
  
  
- CAATATTCAT TCACCTTTTC ATAACAACAG CACCTCTGTT TAAATCCAAG AACTCGTTTT TGAGTTGATG   
  
  
- AGAGATCTCT CTCTCTCTCC ATCGTCATTG TGCTGAACAC ATCTTTAACA CTCCCTGCTT AGCCAATTCT   
  
  
- TCACGTACAG AACATAGAGA GAGATCTCGA TCTTTTTAGG ATTCATAAAG TAAAAAAACC TTGTTTGTCT   
  
  
- CTTTTGGGGT TCTTAATATT ATTAATTTG

+     Unnamed\_\_1

| Site Name | Organism | Position | Strand | Matrix score. | sequence | function |
| --- | --- | --- | --- | --- | --- | --- |
| Unnamed\_\_1 | Zea mays | 198 | - | 5 | CGTGG |  |
| Unnamed\_\_1 | Zea mays | 1290 | + | 5 | CGTGG |  |
| Unnamed\_\_1 | Zea mays | 840 | + | 5 | CGTGG |  |

>036006   
+ GTATACTTAA AACTAGAGAA GCCATCGATG GGCAATTGTT AGGCATAAAA ATAGTGAAAG AACCACCGGC   
  
  
+ CGGATAAACA AGAGTAAAGC TGTCTGAAAA TAAACCCTAC TCACACACAT GGTATTTCTC ATATTTTATT   
  
  
+ TTATAGTCAA TTATTAAGAT AATAAGCATT TTAACCATTT ATGTATACAT GTTCATACCA CGAGCGTTGC   
  
  
+ TTGGAGGAAC ACATTTTGGT ATCAAGTGAT GTTGCAAAAT GATGCATACA ATCAGTACAC ATCACTCTAG   
  
  
+ CTACTTACTC CATCTTGTAG TATTGCAATG GAGTAAAATA TAGCACTAGT CCCAGATTAA TTATTATGGT   
  
  
+ TCTCCTTTCA AACACATAGT TAATGGTTAT AATGATATAC TTAAGGGCAA AATATATTAC TTATACAAAA   
  
  
+ TAACTCGATG GGTTACGTAA AATACATGAA GAATTCTGTT TAATAAGTTT AGTATATGCT TGCATGAGTA   
  
  
+ TGATACAATA TATATGTGAC AAAATAAAAT ACAAGTCTAA ATTTACGACA CCACCACCCC CTTATATAGA   
  
  
+ GTTAAAACGT GAGATTCAAG AGTGTAGTTG GTGGAAAAAG ATAAATTCTA GGGTTTTTGT CTTGTAGAAA   
  
  
+ TACATCATAT ACATAAATAT TTATCTGTAC AACATGTGCG TATGTACAAT AATCATTGAC AATTAACCTA   
  
  
+ AACATTGCAA TTAACATAAA CTTTGTTTCT TCTTAAATTT TAGATGGTAA GTTCCTCCTA TCAAATCATT   
  
  
+ ACAAAATCAT AAAATCCCAA TAACACACTT TATCTATACG CAAAAAAATT TGCCGGGGAA AATATAGTAC   
  
  
+ GTGGAGGTAT ATATAGTACG TACTTTCTCC TTTGCTTTCA AAGATCATCA AATTTTAATT AGTTTATATT   
  
  
+ ATTTTATATA TATACACACA CACACTAGCA TATAGGCACA CACAAAGTGT GTGAGACATT TTTTTATTTT   
  
  
+ TGTTTTTGAA ATAGAAAGAG AGAGGAAGAG AGGAAGAGAG CGATAGGGAA TGTGAGAATC AGAAGTTTTT   
  
  
+ TTTTTTTTTT TAATTAAAGA TCTGTTAAGA TTACATGTAG TTGAAATTTT GAAAAAAAAA CAGCCAAATT   
  
  
+ TAGTTATGTG AAATTACATT TTTGTCCCGT ATTTGTTATT CATATTTTGT TTTAATTAGA GAGTTAAAGT   
  
  
+ GGTAATTTGA TAGAATTTTG GTTGACAATG AATGTTTTAT TAATTAGTAG ATATATATAT ATATATAATT   
  
  
+ GTTATAAGTA AGTGGAAAAG TATTGTTGTC GTGGAGACAA ATTTAGGTTC TTGAGCAAAA ACTCAACTAC   
  
  
+ TCTCTAGAGA GAGAGAGAGG TAGCAGTAAC ACGACTTGTG TAGAAATTGT GAGGGACGAA TCGGTTAAGA   
  
  
+ AGTGCATGTC TTGTATCTCT CTCTAGAGCT AGAAAAATCC TAAGTATTTC ATTTTTTTGG AACAAACAGA   
  
  
+ GAAAACCCCA AGAATTATAA TAATTAAAC  

- CATATGAATT TTGATCTCTT CGGTAGCTAC CCGTTAACAA TCCGTATTTT TATCACTTTC TTGGTGGCCG   
  
  
- GCCTATTTGT TCTCATTTCG ACAGACTTTT ATTTGGGATG AGTGTGTGTA CCATAAAGAG TATAAAATAA   
  
  
- AATATCAGTT AATAATTCTA TTATTCGTAA AATTGGTAAA TACATATGTA CAAGTATGGT GCTCGCAACG   
  
  
- AACCTCCTTG TGTAAAACCA TAGTTCACTA CAACGTTTTA CTACGTATGT TAGTCATGTG TAGTGAGATC   
  
  
- GATGAATGAG GTAGAACATC ATAACGTTAC CTCATTTTAT ATCGTGATCA GGGTCTAATT AATAATACCA   
  
  
- AGAGGAAAGT TTGTGTATCA ATTACCAATA TTACTATATG AATTCCCGTT TTATATAATG AATATGTTTT   
  
  
- ATTGAGCTAC CCAATGCATT TTATGTACTT CTTAAGACAA ATTATTCAAA TCATATACGA ACGTACTCAT   
  
  
- ACTATGTTAT ATATACACTG TTTTATTTTA TGTTCAGATT TAAATGCTGT GGTGGTGGGG GAATATATCT   
  
  
- CAATTTTGCA CTCTAAGTTC TCACATCAAC CACCTTTTTC TATTTAAGAT CCCAAAAACA GAACATCTTT   
  
  
- ATGTAGTATA TGTATTTATA AATAGACATG TTGTACACGC ATACATGTTA TTAGTAACTG TTAATTGGAT   
  
  
- TTGTAACGTT AATTGTATTT GAAACAAAGA AGAATTTAAA ATCTACCATT CAAGGAGGAT AGTTTAGTAA   
  
  
- TGTTTTAGTA TTTTAGGGTT ATTGTGTGAA ATAGATATGC GTTTTTTTAA ACGGCCCCTT TTATATCATG   
  
  
- CACCTCCATA TATATCATGC ATGAAAGAGG AAACGAAAGT TTCTAGTAGT TTAAAATTAA TCAAATATAA   
  
  
- TAAAATATAT ATATGTGTGT GTGTGATCGT ATATCCGTGT GTGTTTCACA CACTCTGTAA AAAAATAAAA   
  
  
- ACAAAAACTT TATCTTTCTC TCTCCTTCTC TCCTTCTCTC GCTATCCCTT ACACTCTTAG TCTTCAAAAA   
  
  
- AAAAAAAAAA ATTAATTTCT AGACAATTCT AATGTACATC AACTTTAAAA CTTTTTTTTT GTCGGTTTAA   
  
  
- ATCAATACAC TTTAATGTAA AAACAGGGCA TAAACAATAA GTATAAAACA AAATTAATCT CTCAATTTCA   
  
  
- CCATTAAACT ATCTTAAAAC CAACTGTTAC TTACAAAATA ATTAATCATC TATATATATA TATATATTAA   
  
  
- CAATATTCAT TCACCTTTTC ATAACAACAG CACCTCTGTT TAAATCCAAG AACTCGTTTT TGAGTTGATG   
  
  
- AGAGATCTCT CTCTCTCTCC ATCGTCATTG TGCTGAACAC ATCTTTAACA CTCCCTGCTT AGCCAATTCT   
  
  
- TCACGTACAG AACATAGAGA GAGATCTCGA TCTTTTTAGG ATTCATAAAG TAAAAAAACC TTGTTTGTCT   
  
  
- CTTTTGGGGT TCTTAATATT ATTAATTTG

+     Unnamed\_\_2

| Site Name | Organism | Position | Strand | Matrix score. | sequence | function |
| --- | --- | --- | --- | --- | --- | --- |
| Unnamed\_\_2 | Zea mays | 823 | - | 6 | CCCCGG |  |

>036006   
+ GTATACTTAA AACTAGAGAA GCCATCGATG GGCAATTGTT AGGCATAAAA ATAGTGAAAG AACCACCGGC   
  
  
+ CGGATAAACA AGAGTAAAGC TGTCTGAAAA TAAACCCTAC TCACACACAT GGTATTTCTC ATATTTTATT   
  
  
+ TTATAGTCAA TTATTAAGAT AATAAGCATT TTAACCATTT ATGTATACAT GTTCATACCA CGAGCGTTGC   
  
  
+ TTGGAGGAAC ACATTTTGGT ATCAAGTGAT GTTGCAAAAT GATGCATACA ATCAGTACAC ATCACTCTAG   
  
  
+ CTACTTACTC CATCTTGTAG TATTGCAATG GAGTAAAATA TAGCACTAGT CCCAGATTAA TTATTATGGT   
  
  
+ TCTCCTTTCA AACACATAGT TAATGGTTAT AATGATATAC TTAAGGGCAA AATATATTAC TTATACAAAA   
  
  
+ TAACTCGATG GGTTACGTAA AATACATGAA GAATTCTGTT TAATAAGTTT AGTATATGCT TGCATGAGTA   
  
  
+ TGATACAATA TATATGTGAC AAAATAAAAT ACAAGTCTAA ATTTACGACA CCACCACCCC CTTATATAGA   
  
  
+ GTTAAAACGT GAGATTCAAG AGTGTAGTTG GTGGAAAAAG ATAAATTCTA GGGTTTTTGT CTTGTAGAAA   
  
  
+ TACATCATAT ACATAAATAT TTATCTGTAC AACATGTGCG TATGTACAAT AATCATTGAC AATTAACCTA   
  
  
+ AACATTGCAA TTAACATAAA CTTTGTTTCT TCTTAAATTT TAGATGGTAA GTTCCTCCTA TCAAATCATT   
  
  
+ ACAAAATCAT AAAATCCCAA TAACACACTT TATCTATACG CAAAAAAATT TGCCGGGGAA AATATAGTAC   
  
  
+ GTGGAGGTAT ATATAGTACG TACTTTCTCC TTTGCTTTCA AAGATCATCA AATTTTAATT AGTTTATATT   
  
  
+ ATTTTATATA TATACACACA CACACTAGCA TATAGGCACA CACAAAGTGT GTGAGACATT TTTTTATTTT   
  
  
+ TGTTTTTGAA ATAGAAAGAG AGAGGAAGAG AGGAAGAGAG CGATAGGGAA TGTGAGAATC AGAAGTTTTT   
  
  
+ TTTTTTTTTT TAATTAAAGA TCTGTTAAGA TTACATGTAG TTGAAATTTT GAAAAAAAAA CAGCCAAATT   
  
  
+ TAGTTATGTG AAATTACATT TTTGTCCCGT ATTTGTTATT CATATTTTGT TTTAATTAGA GAGTTAAAGT   
  
  
+ GGTAATTTGA TAGAATTTTG GTTGACAATG AATGTTTTAT TAATTAGTAG ATATATATAT ATATATAATT   
  
  
+ GTTATAAGTA AGTGGAAAAG TATTGTTGTC GTGGAGACAA ATTTAGGTTC TTGAGCAAAA ACTCAACTAC   
  
  
+ TCTCTAGAGA GAGAGAGAGG TAGCAGTAAC ACGACTTGTG TAGAAATTGT GAGGGACGAA TCGGTTAAGA   
  
  
+ AGTGCATGTC TTGTATCTCT CTCTAGAGCT AGAAAAATCC TAAGTATTTC ATTTTTTTGG AACAAACAGA   
  
  
+ GAAAACCCCA AGAATTATAA TAATTAAAC  

- CATATGAATT TTGATCTCTT CGGTAGCTAC CCGTTAACAA TCCGTATTTT TATCACTTTC TTGGTGGCCG   
  
  
- GCCTATTTGT TCTCATTTCG ACAGACTTTT ATTTGGGATG AGTGTGTGTA CCATAAAGAG TATAAAATAA   
  
  
- AATATCAGTT AATAATTCTA TTATTCGTAA AATTGGTAAA TACATATGTA CAAGTATGGT GCTCGCAACG   
  
  
- AACCTCCTTG TGTAAAACCA TAGTTCACTA CAACGTTTTA CTACGTATGT TAGTCATGTG TAGTGAGATC   
  
  
- GATGAATGAG GTAGAACATC ATAACGTTAC CTCATTTTAT ATCGTGATCA GGGTCTAATT AATAATACCA   
  
  
- AGAGGAAAGT TTGTGTATCA ATTACCAATA TTACTATATG AATTCCCGTT TTATATAATG AATATGTTTT   
  
  
- ATTGAGCTAC CCAATGCATT TTATGTACTT CTTAAGACAA ATTATTCAAA TCATATACGA ACGTACTCAT   
  
  
- ACTATGTTAT ATATACACTG TTTTATTTTA TGTTCAGATT TAAATGCTGT GGTGGTGGGG GAATATATCT   
  
  
- CAATTTTGCA CTCTAAGTTC TCACATCAAC CACCTTTTTC TATTTAAGAT CCCAAAAACA GAACATCTTT   
  
  
- ATGTAGTATA TGTATTTATA AATAGACATG TTGTACACGC ATACATGTTA TTAGTAACTG TTAATTGGAT   
  
  
- TTGTAACGTT AATTGTATTT GAAACAAAGA AGAATTTAAA ATCTACCATT CAAGGAGGAT AGTTTAGTAA   
  
  
- TGTTTTAGTA TTTTAGGGTT ATTGTGTGAA ATAGATATGC GTTTTTTTAA ACGGCCCCTT TTATATCATG   
  
  
- CACCTCCATA TATATCATGC ATGAAAGAGG AAACGAAAGT TTCTAGTAGT TTAAAATTAA TCAAATATAA   
  
  
- TAAAATATAT ATATGTGTGT GTGTGATCGT ATATCCGTGT GTGTTTCACA CACTCTGTAA AAAAATAAAA   
  
  
- ACAAAAACTT TATCTTTCTC TCTCCTTCTC TCCTTCTCTC GCTATCCCTT ACACTCTTAG TCTTCAAAAA   
  
  
- AAAAAAAAAA ATTAATTTCT AGACAATTCT AATGTACATC AACTTTAAAA CTTTTTTTTT GTCGGTTTAA   
  
  
- ATCAATACAC TTTAATGTAA AAACAGGGCA TAAACAATAA GTATAAAACA AAATTAATCT CTCAATTTCA   
  
  
- CCATTAAACT ATCTTAAAAC CAACTGTTAC TTACAAAATA ATTAATCATC TATATATATA TATATATTAA   
  
  
- CAATATTCAT TCACCTTTTC ATAACAACAG CACCTCTGTT TAAATCCAAG AACTCGTTTT TGAGTTGATG   
  
  
- AGAGATCTCT CTCTCTCTCC ATCGTCATTG TGCTGAACAC ATCTTTAACA CTCCCTGCTT AGCCAATTCT   
  
  
- TCACGTACAG AACATAGAGA GAGATCTCGA TCTTTTTAGG ATTCATAAAG TAAAAAAACC TTGTTTGTCT   
  
  
- CTTTTGGGGT TCTTAATATT ATTAATTTG

+     Unnamed\_\_4

| Site Name | Organism | Position | Strand | Matrix score. | sequence | function |
| --- | --- | --- | --- | --- | --- | --- |
| Unnamed\_\_4 | Petroselinum hortense | 288 | + | 4 | CTCC |  |
| Unnamed\_\_4 | Petroselinum hortense | 755 | + | 4 | CTCC |  |
| Unnamed\_\_4 | Petroselinum hortense | 310 | - | 4 | CTCC |  |
| Unnamed\_\_4 | Petroselinum hortense | 1293 | - | 4 | CTCC |  |
| Unnamed\_\_4 | Petroselinum hortense | 843 | - | 4 | CTCC |  |
| Unnamed\_\_4 | Petroselinum hortense | 867 | + | 4 | CTCC |  |
| Unnamed\_\_4 | Petroselinum hortense | 213 | - | 4 | CTCC |  |
| Unnamed\_\_4 | Petroselinum hortense | 352 | + | 4 | CTCC |  |

>036006   
+ GTATACTTAA AACTAGAGAA GCCATCGATG GGCAATTGTT AGGCATAAAA ATAGTGAAAG AACCACCGGC   
  
  
+ CGGATAAACA AGAGTAAAGC TGTCTGAAAA TAAACCCTAC TCACACACAT GGTATTTCTC ATATTTTATT   
  
  
+ TTATAGTCAA TTATTAAGAT AATAAGCATT TTAACCATTT ATGTATACAT GTTCATACCA CGAGCGTTGC   
  
  
+ TTGGAGGAAC ACATTTTGGT ATCAAGTGAT GTTGCAAAAT GATGCATACA ATCAGTACAC ATCACTCTAG   
  
  
+ CTACTTACTC CATCTTGTAG TATTGCAATG GAGTAAAATA TAGCACTAGT CCCAGATTAA TTATTATGGT   
  
  
+ TCTCCTTTCA AACACATAGT TAATGGTTAT AATGATATAC TTAAGGGCAA AATATATTAC TTATACAAAA   
  
  
+ TAACTCGATG GGTTACGTAA AATACATGAA GAATTCTGTT TAATAAGTTT AGTATATGCT TGCATGAGTA   
  
  
+ TGATACAATA TATATGTGAC AAAATAAAAT ACAAGTCTAA ATTTACGACA CCACCACCCC CTTATATAGA   
  
  
+ GTTAAAACGT GAGATTCAAG AGTGTAGTTG GTGGAAAAAG ATAAATTCTA GGGTTTTTGT CTTGTAGAAA   
  
  
+ TACATCATAT ACATAAATAT TTATCTGTAC AACATGTGCG TATGTACAAT AATCATTGAC AATTAACCTA   
  
  
+ AACATTGCAA TTAACATAAA CTTTGTTTCT TCTTAAATTT TAGATGGTAA GTTCCTCCTA TCAAATCATT   
  
  
+ ACAAAATCAT AAAATCCCAA TAACACACTT TATCTATACG CAAAAAAATT TGCCGGGGAA AATATAGTAC   
  
  
+ GTGGAGGTAT ATATAGTACG TACTTTCTCC TTTGCTTTCA AAGATCATCA AATTTTAATT AGTTTATATT   
  
  
+ ATTTTATATA TATACACACA CACACTAGCA TATAGGCACA CACAAAGTGT GTGAGACATT TTTTTATTTT   
  
  
+ TGTTTTTGAA ATAGAAAGAG AGAGGAAGAG AGGAAGAGAG CGATAGGGAA TGTGAGAATC AGAAGTTTTT   
  
  
+ TTTTTTTTTT TAATTAAAGA TCTGTTAAGA TTACATGTAG TTGAAATTTT GAAAAAAAAA CAGCCAAATT   
  
  
+ TAGTTATGTG AAATTACATT TTTGTCCCGT ATTTGTTATT CATATTTTGT TTTAATTAGA GAGTTAAAGT   
  
  
+ GGTAATTTGA TAGAATTTTG GTTGACAATG AATGTTTTAT TAATTAGTAG ATATATATAT ATATATAATT   
  
  
+ GTTATAAGTA AGTGGAAAAG TATTGTTGTC GTGGAGACAA ATTTAGGTTC TTGAGCAAAA ACTCAACTAC   
  
  
+ TCTCTAGAGA GAGAGAGAGG TAGCAGTAAC ACGACTTGTG TAGAAATTGT GAGGGACGAA TCGGTTAAGA   
  
  
+ AGTGCATGTC TTGTATCTCT CTCTAGAGCT AGAAAAATCC TAAGTATTTC ATTTTTTTGG AACAAACAGA   
  
  
+ GAAAACCCCA AGAATTATAA TAATTAAAC  

- CATATGAATT TTGATCTCTT CGGTAGCTAC CCGTTAACAA TCCGTATTTT TATCACTTTC TTGGTGGCCG   
  
  
- GCCTATTTGT TCTCATTTCG ACAGACTTTT ATTTGGGATG AGTGTGTGTA CCATAAAGAG TATAAAATAA   
  
  
- AATATCAGTT AATAATTCTA TTATTCGTAA AATTGGTAAA TACATATGTA CAAGTATGGT GCTCGCAACG   
  
  
- AACCTCCTTG TGTAAAACCA TAGTTCACTA CAACGTTTTA CTACGTATGT TAGTCATGTG TAGTGAGATC   
  
  
- GATGAATGAG GTAGAACATC ATAACGTTAC CTCATTTTAT ATCGTGATCA GGGTCTAATT AATAATACCA   
  
  
- AGAGGAAAGT TTGTGTATCA ATTACCAATA TTACTATATG AATTCCCGTT TTATATAATG AATATGTTTT   
  
  
- ATTGAGCTAC CCAATGCATT TTATGTACTT CTTAAGACAA ATTATTCAAA TCATATACGA ACGTACTCAT   
  
  
- ACTATGTTAT ATATACACTG TTTTATTTTA TGTTCAGATT TAAATGCTGT GGTGGTGGGG GAATATATCT   
  
  
- CAATTTTGCA CTCTAAGTTC TCACATCAAC CACCTTTTTC TATTTAAGAT CCCAAAAACA GAACATCTTT   
  
  
- ATGTAGTATA TGTATTTATA AATAGACATG TTGTACACGC ATACATGTTA TTAGTAACTG TTAATTGGAT   
  
  
- TTGTAACGTT AATTGTATTT GAAACAAAGA AGAATTTAAA ATCTACCATT CAAGGAGGAT AGTTTAGTAA   
  
  
- TGTTTTAGTA TTTTAGGGTT ATTGTGTGAA ATAGATATGC GTTTTTTTAA ACGGCCCCTT TTATATCATG   
  
  
- CACCTCCATA TATATCATGC ATGAAAGAGG AAACGAAAGT TTCTAGTAGT TTAAAATTAA TCAAATATAA   
  
  
- TAAAATATAT ATATGTGTGT GTGTGATCGT ATATCCGTGT GTGTTTCACA CACTCTGTAA AAAAATAAAA   
  
  
- ACAAAAACTT TATCTTTCTC TCTCCTTCTC TCCTTCTCTC GCTATCCCTT ACACTCTTAG TCTTCAAAAA   
  
  
- AAAAAAAAAA ATTAATTTCT AGACAATTCT AATGTACATC AACTTTAAAA CTTTTTTTTT GTCGGTTTAA   
  
  
- ATCAATACAC TTTAATGTAA AAACAGGGCA TAAACAATAA GTATAAAACA AAATTAATCT CTCAATTTCA   
  
  
- CCATTAAACT ATCTTAAAAC CAACTGTTAC TTACAAAATA ATTAATCATC TATATATATA TATATATTAA   
  
  
- CAATATTCAT TCACCTTTTC ATAACAACAG CACCTCTGTT TAAATCCAAG AACTCGTTTT TGAGTTGATG   
  
  
- AGAGATCTCT CTCTCTCTCC ATCGTCATTG TGCTGAACAC ATCTTTAACA CTCCCTGCTT AGCCAATTCT   
  
  
- TCACGTACAG AACATAGAGA GAGATCTCGA TCTTTTTAGG ATTCATAAAG TAAAAAAACC TTGTTTGTCT   
  
  
- CTTTTGGGGT TCTTAATATT ATTAATTTG

+     Unnamed\_\_6

| Site Name | Organism | Position | Strand | Matrix score. | sequence | function |
| --- | --- | --- | --- | --- | --- | --- |
| Unnamed\_\_6 | Zea mays | 1239 | - | 10 | taTAAATATct |  |

>036006   
+ GTATACTTAA AACTAGAGAA GCCATCGATG GGCAATTGTT AGGCATAAAA ATAGTGAAAG AACCACCGGC   
  
  
+ CGGATAAACA AGAGTAAAGC TGTCTGAAAA TAAACCCTAC TCACACACAT GGTATTTCTC ATATTTTATT   
  
  
+ TTATAGTCAA TTATTAAGAT AATAAGCATT TTAACCATTT ATGTATACAT GTTCATACCA CGAGCGTTGC   
  
  
+ TTGGAGGAAC ACATTTTGGT ATCAAGTGAT GTTGCAAAAT GATGCATACA ATCAGTACAC ATCACTCTAG   
  
  
+ CTACTTACTC CATCTTGTAG TATTGCAATG GAGTAAAATA TAGCACTAGT CCCAGATTAA TTATTATGGT   
  
  
+ TCTCCTTTCA AACACATAGT TAATGGTTAT AATGATATAC TTAAGGGCAA AATATATTAC TTATACAAAA   
  
  
+ TAACTCGATG GGTTACGTAA AATACATGAA GAATTCTGTT TAATAAGTTT AGTATATGCT TGCATGAGTA   
  
  
+ TGATACAATA TATATGTGAC AAAATAAAAT ACAAGTCTAA ATTTACGACA CCACCACCCC CTTATATAGA   
  
  
+ GTTAAAACGT GAGATTCAAG AGTGTAGTTG GTGGAAAAAG ATAAATTCTA GGGTTTTTGT CTTGTAGAAA   
  
  
+ TACATCATAT ACATAAATAT TTATCTGTAC AACATGTGCG TATGTACAAT AATCATTGAC AATTAACCTA   
  
  
+ AACATTGCAA TTAACATAAA CTTTGTTTCT TCTTAAATTT TAGATGGTAA GTTCCTCCTA TCAAATCATT   
  
  
+ ACAAAATCAT AAAATCCCAA TAACACACTT TATCTATACG CAAAAAAATT TGCCGGGGAA AATATAGTAC   
  
  
+ GTGGAGGTAT ATATAGTACG TACTTTCTCC TTTGCTTTCA AAGATCATCA AATTTTAATT AGTTTATATT   
  
  
+ ATTTTATATA TATACACACA CACACTAGCA TATAGGCACA CACAAAGTGT GTGAGACATT TTTTTATTTT   
  
  
+ TGTTTTTGAA ATAGAAAGAG AGAGGAAGAG AGGAAGAGAG CGATAGGGAA TGTGAGAATC AGAAGTTTTT   
  
  
+ TTTTTTTTTT TAATTAAAGA TCTGTTAAGA TTACATGTAG TTGAAATTTT GAAAAAAAAA CAGCCAAATT   
  
  
+ TAGTTATGTG AAATTACATT TTTGTCCCGT ATTTGTTATT CATATTTTGT TTTAATTAGA GAGTTAAAGT   
  
  
+ GGTAATTTGA TAGAATTTTG GTTGACAATG AATGTTTTAT TAATTAGTAG ATATATATAT ATATATAATT   
  
  
+ GTTATAAGTA AGTGGAAAAG TATTGTTGTC GTGGAGACAA ATTTAGGTTC TTGAGCAAAA ACTCAACTAC   
  
  
+ TCTCTAGAGA GAGAGAGAGG TAGCAGTAAC ACGACTTGTG TAGAAATTGT GAGGGACGAA TCGGTTAAGA   
  
  
+ AGTGCATGTC TTGTATCTCT CTCTAGAGCT AGAAAAATCC TAAGTATTTC ATTTTTTTGG AACAAACAGA   
  
  
+ GAAAACCCCA AGAATTATAA TAATTAAAC  

- CATATGAATT TTGATCTCTT CGGTAGCTAC CCGTTAACAA TCCGTATTTT TATCACTTTC TTGGTGGCCG   
  
  
- GCCTATTTGT TCTCATTTCG ACAGACTTTT ATTTGGGATG AGTGTGTGTA CCATAAAGAG TATAAAATAA   
  
  
- AATATCAGTT AATAATTCTA TTATTCGTAA AATTGGTAAA TACATATGTA CAAGTATGGT GCTCGCAACG   
  
  
- AACCTCCTTG TGTAAAACCA TAGTTCACTA CAACGTTTTA CTACGTATGT TAGTCATGTG TAGTGAGATC   
  
  
- GATGAATGAG GTAGAACATC ATAACGTTAC CTCATTTTAT ATCGTGATCA GGGTCTAATT AATAATACCA   
  
  
- AGAGGAAAGT TTGTGTATCA ATTACCAATA TTACTATATG AATTCCCGTT TTATATAATG AATATGTTTT   
  
  
- ATTGAGCTAC CCAATGCATT TTATGTACTT CTTAAGACAA ATTATTCAAA TCATATACGA ACGTACTCAT   
  
  
- ACTATGTTAT ATATACACTG TTTTATTTTA TGTTCAGATT TAAATGCTGT GGTGGTGGGG GAATATATCT   
  
  
- CAATTTTGCA CTCTAAGTTC TCACATCAAC CACCTTTTTC TATTTAAGAT CCCAAAAACA GAACATCTTT   
  
  
- ATGTAGTATA TGTATTTATA AATAGACATG TTGTACACGC ATACATGTTA TTAGTAACTG TTAATTGGAT   
  
  
- TTGTAACGTT AATTGTATTT GAAACAAAGA AGAATTTAAA ATCTACCATT CAAGGAGGAT AGTTTAGTAA   
  
  
- TGTTTTAGTA TTTTAGGGTT ATTGTGTGAA ATAGATATGC GTTTTTTTAA ACGGCCCCTT TTATATCATG   
  
  
- CACCTCCATA TATATCATGC ATGAAAGAGG AAACGAAAGT TTCTAGTAGT TTAAAATTAA TCAAATATAA   
  
  
- TAAAATATAT ATATGTGTGT GTGTGATCGT ATATCCGTGT GTGTTTCACA CACTCTGTAA AAAAATAAAA   
  
  
- ACAAAAACTT TATCTTTCTC TCTCCTTCTC TCCTTCTCTC GCTATCCCTT ACACTCTTAG TCTTCAAAAA   
  
  
- AAAAAAAAAA ATTAATTTCT AGACAATTCT AATGTACATC AACTTTAAAA CTTTTTTTTT GTCGGTTTAA   
  
  
- ATCAATACAC TTTAATGTAA AAACAGGGCA TAAACAATAA GTATAAAACA AAATTAATCT CTCAATTTCA   
  
  
- CCATTAAACT ATCTTAAAAC CAACTGTTAC TTACAAAATA ATTAATCATC TATATATATA TATATATTAA   
  
  
- CAATATTCAT TCACCTTTTC ATAACAACAG CACCTCTGTT TAAATCCAAG AACTCGTTTT TGAGTTGATG   
  
  
- AGAGATCTCT CTCTCTCTCC ATCGTCATTG TGCTGAACAC ATCTTTAACA CTCCCTGCTT AGCCAATTCT   
  
  
- TCACGTACAG AACATAGAGA GAGATCTCGA TCTTTTTAGG ATTCATAAAG TAAAAAAACC TTGTTTGTCT   
  
  
- CTTTTGGGGT TCTTAATATT ATTAATTTG

+     chs-CMA2a

| Site Name | Organism | Position | Strand | Matrix score. | sequence | function |
| --- | --- | --- | --- | --- | --- | --- |
| chs-CMA2a | Petroselinum crispum | 232 | - | 8 | TCACTTGA | part of a light responsive element |

>036006   
+ GTATACTTAA AACTAGAGAA GCCATCGATG GGCAATTGTT AGGCATAAAA ATAGTGAAAG AACCACCGGC   
  
  
+ CGGATAAACA AGAGTAAAGC TGTCTGAAAA TAAACCCTAC TCACACACAT GGTATTTCTC ATATTTTATT   
  
  
+ TTATAGTCAA TTATTAAGAT AATAAGCATT TTAACCATTT ATGTATACAT GTTCATACCA CGAGCGTTGC   
  
  
+ TTGGAGGAAC ACATTTTGGT ATCAAGTGAT GTTGCAAAAT GATGCATACA ATCAGTACAC ATCACTCTAG   
  
  
+ CTACTTACTC CATCTTGTAG TATTGCAATG GAGTAAAATA TAGCACTAGT CCCAGATTAA TTATTATGGT   
  
  
+ TCTCCTTTCA AACACATAGT TAATGGTTAT AATGATATAC TTAAGGGCAA AATATATTAC TTATACAAAA   
  
  
+ TAACTCGATG GGTTACGTAA AATACATGAA GAATTCTGTT TAATAAGTTT AGTATATGCT TGCATGAGTA   
  
  
+ TGATACAATA TATATGTGAC AAAATAAAAT ACAAGTCTAA ATTTACGACA CCACCACCCC CTTATATAGA   
  
  
+ GTTAAAACGT GAGATTCAAG AGTGTAGTTG GTGGAAAAAG ATAAATTCTA GGGTTTTTGT CTTGTAGAAA   
  
  
+ TACATCATAT ACATAAATAT TTATCTGTAC AACATGTGCG TATGTACAAT AATCATTGAC AATTAACCTA   
  
  
+ AACATTGCAA TTAACATAAA CTTTGTTTCT TCTTAAATTT TAGATGGTAA GTTCCTCCTA TCAAATCATT   
  
  
+ ACAAAATCAT AAAATCCCAA TAACACACTT TATCTATACG CAAAAAAATT TGCCGGGGAA AATATAGTAC   
  
  
+ GTGGAGGTAT ATATAGTACG TACTTTCTCC TTTGCTTTCA AAGATCATCA AATTTTAATT AGTTTATATT   
  
  
+ ATTTTATATA TATACACACA CACACTAGCA TATAGGCACA CACAAAGTGT GTGAGACATT TTTTTATTTT   
  
  
+ TGTTTTTGAA ATAGAAAGAG AGAGGAAGAG AGGAAGAGAG CGATAGGGAA TGTGAGAATC AGAAGTTTTT   
  
  
+ TTTTTTTTTT TAATTAAAGA TCTGTTAAGA TTACATGTAG TTGAAATTTT GAAAAAAAAA CAGCCAAATT   
  
  
+ TAGTTATGTG AAATTACATT TTTGTCCCGT ATTTGTTATT CATATTTTGT TTTAATTAGA GAGTTAAAGT   
  
  
+ GGTAATTTGA TAGAATTTTG GTTGACAATG AATGTTTTAT TAATTAGTAG ATATATATAT ATATATAATT   
  
  
+ GTTATAAGTA AGTGGAAAAG TATTGTTGTC GTGGAGACAA ATTTAGGTTC TTGAGCAAAA ACTCAACTAC   
  
  
+ TCTCTAGAGA GAGAGAGAGG TAGCAGTAAC ACGACTTGTG TAGAAATTGT GAGGGACGAA TCGGTTAAGA   
  
  
+ AGTGCATGTC TTGTATCTCT CTCTAGAGCT AGAAAAATCC TAAGTATTTC ATTTTTTTGG AACAAACAGA   
  
  
+ GAAAACCCCA AGAATTATAA TAATTAAAC  

- CATATGAATT TTGATCTCTT CGGTAGCTAC CCGTTAACAA TCCGTATTTT TATCACTTTC TTGGTGGCCG   
  
  
- GCCTATTTGT TCTCATTTCG ACAGACTTTT ATTTGGGATG AGTGTGTGTA CCATAAAGAG TATAAAATAA   
  
  
- AATATCAGTT AATAATTCTA TTATTCGTAA AATTGGTAAA TACATATGTA CAAGTATGGT GCTCGCAACG   
  
  
- AACCTCCTTG TGTAAAACCA TAGTTCACTA CAACGTTTTA CTACGTATGT TAGTCATGTG TAGTGAGATC   
  
  
- GATGAATGAG GTAGAACATC ATAACGTTAC CTCATTTTAT ATCGTGATCA GGGTCTAATT AATAATACCA   
  
  
- AGAGGAAAGT TTGTGTATCA ATTACCAATA TTACTATATG AATTCCCGTT TTATATAATG AATATGTTTT   
  
  
- ATTGAGCTAC CCAATGCATT TTATGTACTT CTTAAGACAA ATTATTCAAA TCATATACGA ACGTACTCAT   
  
  
- ACTATGTTAT ATATACACTG TTTTATTTTA TGTTCAGATT TAAATGCTGT GGTGGTGGGG GAATATATCT   
  
  
- CAATTTTGCA CTCTAAGTTC TCACATCAAC CACCTTTTTC TATTTAAGAT CCCAAAAACA GAACATCTTT   
  
  
- ATGTAGTATA TGTATTTATA AATAGACATG TTGTACACGC ATACATGTTA TTAGTAACTG TTAATTGGAT   
  
  
- TTGTAACGTT AATTGTATTT GAAACAAAGA AGAATTTAAA ATCTACCATT CAAGGAGGAT AGTTTAGTAA   
  
  
- TGTTTTAGTA TTTTAGGGTT ATTGTGTGAA ATAGATATGC GTTTTTTTAA ACGGCCCCTT TTATATCATG   
  
  
- CACCTCCATA TATATCATGC ATGAAAGAGG AAACGAAAGT TTCTAGTAGT TTAAAATTAA TCAAATATAA   
  
  
- TAAAATATAT ATATGTGTGT GTGTGATCGT ATATCCGTGT GTGTTTCACA CACTCTGTAA AAAAATAAAA   
  
  
- ACAAAAACTT TATCTTTCTC TCTCCTTCTC TCCTTCTCTC GCTATCCCTT ACACTCTTAG TCTTCAAAAA   
  
  
- AAAAAAAAAA ATTAATTTCT AGACAATTCT AATGTACATC AACTTTAAAA CTTTTTTTTT GTCGGTTTAA   
  
  
- ATCAATACAC TTTAATGTAA AAACAGGGCA TAAACAATAA GTATAAAACA AAATTAATCT CTCAATTTCA   
  
  
- CCATTAAACT ATCTTAAAAC CAACTGTTAC TTACAAAATA ATTAATCATC TATATATATA TATATATTAA   
  
  
- CAATATTCAT TCACCTTTTC ATAACAACAG CACCTCTGTT TAAATCCAAG AACTCGTTTT TGAGTTGATG   
  
  
- AGAGATCTCT CTCTCTCTCC ATCGTCATTG TGCTGAACAC ATCTTTAACA CTCCCTGCTT AGCCAATTCT   
  
  
- TCACGTACAG AACATAGAGA GAGATCTCGA TCTTTTTAGG ATTCATAAAG TAAAAAAACC TTGTTTGTCT   
  
  
- CTTTTGGGGT TCTTAATATT ATTAATTTG
