# Supplementary material for: In Silico Genome-Wide Analysis of Respiratory Burst Oxidase Homolog (RBOH) Family Genes in Five Fruit-Producing Trees, and Potential Functional Analysis on Lignification of Stone Cells in Chinese White Pear
Source: Cells. 2019 May 29;8(6):520. doi: 10.3390/cells8060520 (PMC6627160; doi:10.3390/cells8060520)
Supplement: Supplementary file 1 [file cells-08-00520-s001.zip › Supplementary Material/Spreadsheets S2. Numbers of cis-elements in promoter region of PbRBOHs.tar.zip!/PbRBOHJ.html]

Content-Type: text/html; charset=ISO-8859-1


PlantCARE


Webmaster Firefox specific output  
To save the result:
click on the frame with the right mouse button and save the source code as a text file with extension .html  
REFERENCE:PlantCARE: a database of plant cis-acting regulatory elements and a portal to tools for in silico analysis of promoter sequences.  
Lescot, M., Déhais, P., Moreau, Y., De Moor, B., Rouzé ,P.,and Rombauts, S.  
Nucleic Acids Res., Database issue(2002), 30(1):325-327.   


---

>037399   
+ TGTTTAATTA TTATAATTCT TGAGGTTTTG TGTTTGAAAT GCTGAGGATT TTTCTAGTTC TAGAGAGAGA   
  
  
+ GACAGAGACA GAGAGAAACA AGACATGCAC CTCTCAACCA AATCGTCCCC CACAATTTCA ACGCAAGTCG   
  
  
+ TGCTACCTCT CTCTCTAACT CTCTTTCTCT CTCTAAAGAA TAGTTGAGTT TTTGCTCAAG AAGCAAATTT   
  
  
+ GTCCCACAAC AACAATAGAT TTCCACTTAC TTATAACAAT TATATGATCT TTGAAGGGAA CGGAGAAGGT   
  
  
+ ACGTACGTAC TATATATTAC TTCCCAGTAC TATATTTTTC CCGTCAATTT TTTTGCGTAC ATATAAAGTG   
  
  
+ TGTTATTGAG ATTTGATGAT TTTGTAATGA TTTTATAGGA GGAGCTTACC GTCTACAATT TAAGAAGAAA   
  
  
+ CAATGATTAC GTTAATTGGA ATGTTTAGGC TAATAGTACA TGATTATTGT ACATACGCAC ATGTTGTATA   
  
  
+ GATAAATATT TATATTTCTC CAAGACAAAA ACCCTAAAAT TTATCTTTTT CCACCAACTA CACTCTTGAG   
  
  
+ TTTCACATTT TAACTCTAAT GTTGTTGTCG TAAATTTAGA CTTGTATTTT ATATTCTTTT GTTACATATA   
  
  
+ CATTGTATCA TACTCATGCA TGCATATATA GTAAACTTAT TAAACTCAAT TCTTCATGTA TTTTACGTAA   
  
  
+ ACCAATCGAG TTATTTTGTA TGAGTAATAT ATTTTGCCCT TATGGTATCA TTACGACCAT CAACTAGCTA   
  
  
+ TGTGATTGAA ATGAGAACCA TGAAGAACTA ATTTCGGATT AATGCTAGGA AGAACATATG TAGTAAGTTG   
  
  
+ AGTGATGTGT ACTAATTGTA TGCATCATTT TGTAACATCA CGTGATCCAA AAAAATGTGT TATACGGTTA   
  
  
+ CTCCAAGCAA TGCTCATAAT ATGAATATGT ATACATAAAT GGTTAAAATG CGTATTATCC TAATAATTGA   
  
  
+ CTATAAAATA AAATATGAGA ATACTATATA TATGTATGTG TGTGTGTGTG TGTATGTGTA GGGTTAGTGA   
  
  
+ TGCTTCATGG CATGTGTAGG ATTAGTGATG CTTTATGGCC ACAAACTAGG GCAAGGATTT GGTCTACATA   
  
  
+ ATCACCTATT CAAAAGGAAA TATTGTTCTA TATAATTTTC AGACAGTTTT ATATTCTCTT TATTTATGTA   
  
  
+ TTATTGATGA ATAGTTATGC ACAGGGCATA TCCGGCCGGT GGTTCTTTCA CTATTTTTAT GCATAACAAT   
  
  
+ TGGCCATCAA TGGCTTCTCT TATTTTATAC ATAACAATTG GACATATTGG AAGTGGTCAT CACTATTGTT   
  
  
+ GAGCAGTCAG TCTCAGACTC GATTTTTTAT ACAAACGTAA CCACATAGAG ATTACCCAGC ATAGCTTAAT   
  
  
+ TACCTGCTGG AACCAAACAT GCATGCATAC CTCTTTTTAT TTACGTGTTG TCATTGTCTT TGACTGCTTA   
  
  
+ AGATTCTAAA CAAGTTTAAT AGGTGTTTG  

- ACAAATTAAT AATATTAAGA ACTCCAAAAC ACAAACTTTA CGACTCCTAA AAAGATCAAG ATCTCTCTCT   
  
  
- CTGTCTCTGT CTCTCTTTGT TCTGTACGTG GAGAGTTGGT TTAGCAGGGG GTGTTAAAGT TGCGTTCAGC   
  
  
- ACGATGGAGA GAGAGATTGA GAGAAAGAGA GAGATTTCTT ATCAACTCAA AAACGAGTTC TTCGTTTAAA   
  
  
- CAGGGTGTTG TTGTTATCTA AAGGTGAATG AATATTGTTA ATATACTAGA AACTTCCCTT GCCTCTTCCA   
  
  
- TGCATGCATG ATATATAATG AAGGGTCATG ATATAAAAAG GGCAGTTAAA AAAACGCATG TATATTTCAC   
  
  
- ACAATAACTC TAAACTACTA AAACATTACT AAAATATCCT CCTCGAATGG CAGATGTTAA ATTCTTCTTT   
  
  
- GTTACTAATG CAATTAACCT TACAAATCCG ATTATCATGT ACTAATAACA TGTATGCGTG TACAACATAT   
  
  
- CTATTTATAA ATATAAAGAG GTTCTGTTTT TGGGATTTTA AATAGAAAAA GGTGGTTGAT GTGAGAACTC   
  
  
- AAAGTGTAAA ATTGAGATTA CAACAACAGC ATTTAAATCT GAACATAAAA TATAAGAAAA CAATGTATAT   
  
  
- GTAACATAGT ATGAGTACGT ACGTATATAT CATTTGAATA ATTTGAGTTA AGAAGTACAT AAAATGCATT   
  
  
- TGGTTAGCTC AATAAAACAT ACTCATTATA TAAAACGGGA ATACCATAGT AATGCTGGTA GTTGATCGAT   
  
  
- ACACTAACTT TACTCTTGGT ACTTCTTGAT TAAAGCCTAA TTACGATCCT TCTTGTATAC ATCATTCAAC   
  
  
- TCACTACACA TGATTAACAT ACGTAGTAAA ACATTGTAGT GCACTAGGTT TTTTTACACA ATATGCCAAT   
  
  
- GAGGTTCGTT ACGAGTATTA TACTTATACA TATGTATTTA CCAATTTTAC GCATAATAGG ATTATTAACT   
  
  
- GATATTTTAT TTTATACTCT TATGATATAT ATACATACAC ACACACACAC ACATACACAT CCCAATCACT   
  
  
- ACGAAGTACC GTACACATCC TAATCACTAC GAAATACCGG TGTTTGATCC CGTTCCTAAA CCAGATGTAT   
  
  
- TAGTGGATAA GTTTTCCTTT ATAACAAGAT ATATTAAAAG TCTGTCAAAA TATAAGAGAA ATAAATACAT   
  
  
- AATAACTACT TATCAATACG TGTCCCGTAT AGGCCGGCCA CCAAGAAAGT GATAAAAATA CGTATTGTTA   
  
  
- ACCGGTAGTT ACCGAAGAGA ATAAAATATG TATTGTTAAC CTGTATAACC TTCACCAGTA GTGATAACAA   
  
  
- CTCGTCAGTC AGAGTCTGAG CTAAAAAATA TGTTTGCATT GGTGTATCTC TAATGGGTCG TATCGAATTA   
  
  
- ATGGACGACC TTGGTTTGTA CGTACGTATG GAGAAAAATA AATGCACAAC AGTAACAGAA ACTGACGAAT   
  
  
- TCTAAGATTT GTTCAAATTA TCCACAAAC

  
  
Motifs Found  

+   

| Site Name | Organism | Position | Strand | Matrix score. | sequence | function |
| --- | --- | --- | --- | --- | --- | --- |
|  | organism | 1209 | - | 4 | motif\_sequence | short\_function |
|  | organism | 822 | - | 4 | motif\_sequence | short\_function |
|  | organism | 477 | - | 4 | motif\_sequence | short\_function |
|  | organism | 456 | - | 4 | motif\_sequence | short\_function |
|  | organism | 337 | - | 4 | motif\_sequence | short\_function |
|  | organism | 1300 | - | 4 | motif\_sequence | short\_function |
|  | organism | 469 | - | 4 | motif\_sequence | short\_function |

>037399   
+ TGTTTAATTA TTATAATTCT TGAGGTTTTG TGTTTGAAAT GCTGAGGATT TTTCTAGTTC TAGAGAGAGA   
  
  
+ GACAGAGACA GAGAGAAACA AGACATGCAC CTCTCAACCA AATCGTCCCC CACAATTTCA ACGCAAGTCG   
  
  
+ TGCTACCTCT CTCTCTAACT CTCTTTCTCT CTCTAAAGAA TAGTTGAGTT TTTGCTCAAG AAGCAAATTT   
  
  
+ GTCCCACAAC AACAATAGAT TTCCACTTAC TTATAACAAT TATATGATCT TTGAAGGGAA CGGAGAAGGT   
  
  
+ ACGTACGTAC TATATATTAC TTCCCAGTAC TATATTTTTC CCGTCAATTT TTTTGCGTAC ATATAAAGTG   
  
  
+ TGTTATTGAG ATTTGATGAT TTTGTAATGA TTTTATAGGA GGAGCTTACC GTCTACAATT TAAGAAGAAA   
  
  
+ CAATGATTAC GTTAATTGGA ATGTTTAGGC TAATAGTACA TGATTATTGT ACATACGCAC ATGTTGTATA   
  
  
+ GATAAATATT TATATTTCTC CAAGACAAAA ACCCTAAAAT TTATCTTTTT CCACCAACTA CACTCTTGAG   
  
  
+ TTTCACATTT TAACTCTAAT GTTGTTGTCG TAAATTTAGA CTTGTATTTT ATATTCTTTT GTTACATATA   
  
  
+ CATTGTATCA TACTCATGCA TGCATATATA GTAAACTTAT TAAACTCAAT TCTTCATGTA TTTTACGTAA   
  
  
+ ACCAATCGAG TTATTTTGTA TGAGTAATAT ATTTTGCCCT TATGGTATCA TTACGACCAT CAACTAGCTA   
  
  
+ TGTGATTGAA ATGAGAACCA TGAAGAACTA ATTTCGGATT AATGCTAGGA AGAACATATG TAGTAAGTTG   
  
  
+ AGTGATGTGT ACTAATTGTA TGCATCATTT TGTAACATCA CGTGATCCAA AAAAATGTGT TATACGGTTA   
  
  
+ CTCCAAGCAA TGCTCATAAT ATGAATATGT ATACATAAAT GGTTAAAATG CGTATTATCC TAATAATTGA   
  
  
+ CTATAAAATA AAATATGAGA ATACTATATA TATGTATGTG TGTGTGTGTG TGTATGTGTA GGGTTAGTGA   
  
  
+ TGCTTCATGG CATGTGTAGG ATTAGTGATG CTTTATGGCC ACAAACTAGG GCAAGGATTT GGTCTACATA   
  
  
+ ATCACCTATT CAAAAGGAAA TATTGTTCTA TATAATTTTC AGACAGTTTT ATATTCTCTT TATTTATGTA   
  
  
+ TTATTGATGA ATAGTTATGC ACAGGGCATA TCCGGCCGGT GGTTCTTTCA CTATTTTTAT GCATAACAAT   
  
  
+ TGGCCATCAA TGGCTTCTCT TATTTTATAC ATAACAATTG GACATATTGG AAGTGGTCAT CACTATTGTT   
  
  
+ GAGCAGTCAG TCTCAGACTC GATTTTTTAT ACAAACGTAA CCACATAGAG ATTACCCAGC ATAGCTTAAT   
  
  
+ TACCTGCTGG AACCAAACAT GCATGCATAC CTCTTTTTAT TTACGTGTTG TCATTGTCTT TGACTGCTTA   
  
  
+ AGATTCTAAA CAAGTTTAAT AGGTGTTTG  

- ACAAATTAAT AATATTAAGA ACTCCAAAAC ACAAACTTTA CGACTCCTAA AAAGATCAAG ATCTCTCTCT   
  
  
- CTGTCTCTGT CTCTCTTTGT TCTGTACGTG GAGAGTTGGT TTAGCAGGGG GTGTTAAAGT TGCGTTCAGC   
  
  
- ACGATGGAGA GAGAGATTGA GAGAAAGAGA GAGATTTCTT ATCAACTCAA AAACGAGTTC TTCGTTTAAA   
  
  
- CAGGGTGTTG TTGTTATCTA AAGGTGAATG AATATTGTTA ATATACTAGA AACTTCCCTT GCCTCTTCCA   
  
  
- TGCATGCATG ATATATAATG AAGGGTCATG ATATAAAAAG GGCAGTTAAA AAAACGCATG TATATTTCAC   
  
  
- ACAATAACTC TAAACTACTA AAACATTACT AAAATATCCT CCTCGAATGG CAGATGTTAA ATTCTTCTTT   
  
  
- GTTACTAATG CAATTAACCT TACAAATCCG ATTATCATGT ACTAATAACA TGTATGCGTG TACAACATAT   
  
  
- CTATTTATAA ATATAAAGAG GTTCTGTTTT TGGGATTTTA AATAGAAAAA GGTGGTTGAT GTGAGAACTC   
  
  
- AAAGTGTAAA ATTGAGATTA CAACAACAGC ATTTAAATCT GAACATAAAA TATAAGAAAA CAATGTATAT   
  
  
- GTAACATAGT ATGAGTACGT ACGTATATAT CATTTGAATA ATTTGAGTTA AGAAGTACAT AAAATGCATT   
  
  
- TGGTTAGCTC AATAAAACAT ACTCATTATA TAAAACGGGA ATACCATAGT AATGCTGGTA GTTGATCGAT   
  
  
- ACACTAACTT TACTCTTGGT ACTTCTTGAT TAAAGCCTAA TTACGATCCT TCTTGTATAC ATCATTCAAC   
  
  
- TCACTACACA TGATTAACAT ACGTAGTAAA ACATTGTAGT GCACTAGGTT TTTTTACACA ATATGCCAAT   
  
  
- GAGGTTCGTT ACGAGTATTA TACTTATACA TATGTATTTA CCAATTTTAC GCATAATAGG ATTATTAACT   
  
  
- GATATTTTAT TTTATACTCT TATGATATAT ATACATACAC ACACACACAC ACATACACAT CCCAATCACT   
  
  
- ACGAAGTACC GTACACATCC TAATCACTAC GAAATACCGG TGTTTGATCC CGTTCCTAAA CCAGATGTAT   
  
  
- TAGTGGATAA GTTTTCCTTT ATAACAAGAT ATATTAAAAG TCTGTCAAAA TATAAGAGAA ATAAATACAT   
  
  
- AATAACTACT TATCAATACG TGTCCCGTAT AGGCCGGCCA CCAAGAAAGT GATAAAAATA CGTATTGTTA   
  
  
- ACCGGTAGTT ACCGAAGAGA ATAAAATATG TATTGTTAAC CTGTATAACC TTCACCAGTA GTGATAACAA   
  
  
- CTCGTCAGTC AGAGTCTGAG CTAAAAAATA TGTTTGCATT GGTGTATCTC TAATGGGTCG TATCGAATTA   
  
  
- ATGGACGACC TTGGTTTGTA CGTACGTATG GAGAAAAATA AATGCACAAC AGTAACAGAA ACTGACGAAT   
  
  
- TCTAAGATTT GTTCAAATTA TCCACAAAC

+     AAGAA-motif

| Site Name | Organism | Position | Strand | Matrix score. | sequence | function |
| --- | --- | --- | --- | --- | --- | --- |
| AAGAA-motif | Avena sativa | 1233 | - | 7 | GAAAGAA |  |

>037399   
+ TGTTTAATTA TTATAATTCT TGAGGTTTTG TGTTTGAAAT GCTGAGGATT TTTCTAGTTC TAGAGAGAGA   
  
  
+ GACAGAGACA GAGAGAAACA AGACATGCAC CTCTCAACCA AATCGTCCCC CACAATTTCA ACGCAAGTCG   
  
  
+ TGCTACCTCT CTCTCTAACT CTCTTTCTCT CTCTAAAGAA TAGTTGAGTT TTTGCTCAAG AAGCAAATTT   
  
  
+ GTCCCACAAC AACAATAGAT TTCCACTTAC TTATAACAAT TATATGATCT TTGAAGGGAA CGGAGAAGGT   
  
  
+ ACGTACGTAC TATATATTAC TTCCCAGTAC TATATTTTTC CCGTCAATTT TTTTGCGTAC ATATAAAGTG   
  
  
+ TGTTATTGAG ATTTGATGAT TTTGTAATGA TTTTATAGGA GGAGCTTACC GTCTACAATT TAAGAAGAAA   
  
  
+ CAATGATTAC GTTAATTGGA ATGTTTAGGC TAATAGTACA TGATTATTGT ACATACGCAC ATGTTGTATA   
  
  
+ GATAAATATT TATATTTCTC CAAGACAAAA ACCCTAAAAT TTATCTTTTT CCACCAACTA CACTCTTGAG   
  
  
+ TTTCACATTT TAACTCTAAT GTTGTTGTCG TAAATTTAGA CTTGTATTTT ATATTCTTTT GTTACATATA   
  
  
+ CATTGTATCA TACTCATGCA TGCATATATA GTAAACTTAT TAAACTCAAT TCTTCATGTA TTTTACGTAA   
  
  
+ ACCAATCGAG TTATTTTGTA TGAGTAATAT ATTTTGCCCT TATGGTATCA TTACGACCAT CAACTAGCTA   
  
  
+ TGTGATTGAA ATGAGAACCA TGAAGAACTA ATTTCGGATT AATGCTAGGA AGAACATATG TAGTAAGTTG   
  
  
+ AGTGATGTGT ACTAATTGTA TGCATCATTT TGTAACATCA CGTGATCCAA AAAAATGTGT TATACGGTTA   
  
  
+ CTCCAAGCAA TGCTCATAAT ATGAATATGT ATACATAAAT GGTTAAAATG CGTATTATCC TAATAATTGA   
  
  
+ CTATAAAATA AAATATGAGA ATACTATATA TATGTATGTG TGTGTGTGTG TGTATGTGTA GGGTTAGTGA   
  
  
+ TGCTTCATGG CATGTGTAGG ATTAGTGATG CTTTATGGCC ACAAACTAGG GCAAGGATTT GGTCTACATA   
  
  
+ ATCACCTATT CAAAAGGAAA TATTGTTCTA TATAATTTTC AGACAGTTTT ATATTCTCTT TATTTATGTA   
  
  
+ TTATTGATGA ATAGTTATGC ACAGGGCATA TCCGGCCGGT GGTTCTTTCA CTATTTTTAT GCATAACAAT   
  
  
+ TGGCCATCAA TGGCTTCTCT TATTTTATAC ATAACAATTG GACATATTGG AAGTGGTCAT CACTATTGTT   
  
  
+ GAGCAGTCAG TCTCAGACTC GATTTTTTAT ACAAACGTAA CCACATAGAG ATTACCCAGC ATAGCTTAAT   
  
  
+ TACCTGCTGG AACCAAACAT GCATGCATAC CTCTTTTTAT TTACGTGTTG TCATTGTCTT TGACTGCTTA   
  
  
+ AGATTCTAAA CAAGTTTAAT AGGTGTTTG  

- ACAAATTAAT AATATTAAGA ACTCCAAAAC ACAAACTTTA CGACTCCTAA AAAGATCAAG ATCTCTCTCT   
  
  
- CTGTCTCTGT CTCTCTTTGT TCTGTACGTG GAGAGTTGGT TTAGCAGGGG GTGTTAAAGT TGCGTTCAGC   
  
  
- ACGATGGAGA GAGAGATTGA GAGAAAGAGA GAGATTTCTT ATCAACTCAA AAACGAGTTC TTCGTTTAAA   
  
  
- CAGGGTGTTG TTGTTATCTA AAGGTGAATG AATATTGTTA ATATACTAGA AACTTCCCTT GCCTCTTCCA   
  
  
- TGCATGCATG ATATATAATG AAGGGTCATG ATATAAAAAG GGCAGTTAAA AAAACGCATG TATATTTCAC   
  
  
- ACAATAACTC TAAACTACTA AAACATTACT AAAATATCCT CCTCGAATGG CAGATGTTAA ATTCTTCTTT   
  
  
- GTTACTAATG CAATTAACCT TACAAATCCG ATTATCATGT ACTAATAACA TGTATGCGTG TACAACATAT   
  
  
- CTATTTATAA ATATAAAGAG GTTCTGTTTT TGGGATTTTA AATAGAAAAA GGTGGTTGAT GTGAGAACTC   
  
  
- AAAGTGTAAA ATTGAGATTA CAACAACAGC ATTTAAATCT GAACATAAAA TATAAGAAAA CAATGTATAT   
  
  
- GTAACATAGT ATGAGTACGT ACGTATATAT CATTTGAATA ATTTGAGTTA AGAAGTACAT AAAATGCATT   
  
  
- TGGTTAGCTC AATAAAACAT ACTCATTATA TAAAACGGGA ATACCATAGT AATGCTGGTA GTTGATCGAT   
  
  
- ACACTAACTT TACTCTTGGT ACTTCTTGAT TAAAGCCTAA TTACGATCCT TCTTGTATAC ATCATTCAAC   
  
  
- TCACTACACA TGATTAACAT ACGTAGTAAA ACATTGTAGT GCACTAGGTT TTTTTACACA ATATGCCAAT   
  
  
- GAGGTTCGTT ACGAGTATTA TACTTATACA TATGTATTTA CCAATTTTAC GCATAATAGG ATTATTAACT   
  
  
- GATATTTTAT TTTATACTCT TATGATATAT ATACATACAC ACACACACAC ACATACACAT CCCAATCACT   
  
  
- ACGAAGTACC GTACACATCC TAATCACTAC GAAATACCGG TGTTTGATCC CGTTCCTAAA CCAGATGTAT   
  
  
- TAGTGGATAA GTTTTCCTTT ATAACAAGAT ATATTAAAAG TCTGTCAAAA TATAAGAGAA ATAAATACAT   
  
  
- AATAACTACT TATCAATACG TGTCCCGTAT AGGCCGGCCA CCAAGAAAGT GATAAAAATA CGTATTGTTA   
  
  
- ACCGGTAGTT ACCGAAGAGA ATAAAATATG TATTGTTAAC CTGTATAACC TTCACCAGTA GTGATAACAA   
  
  
- CTCGTCAGTC AGAGTCTGAG CTAAAAAATA TGTTTGCATT GGTGTATCTC TAATGGGTCG TATCGAATTA   
  
  
- ATGGACGACC TTGGTTTGTA CGTACGTATG GAGAAAAATA AATGCACAAC AGTAACAGAA ACTGACGAAT   
  
  
- TCTAAGATTT GTTCAAATTA TCCACAAAC

+     ABRE

| Site Name | Organism | Position | Strand | Matrix score. | sequence | function |
| --- | --- | --- | --- | --- | --- | --- |
| ABRE | Arabidopsis thaliana | 1443 | + | 5 | ACGTG | cis-acting element involved in the abscisic acid responsiveness |
| ABRE | Arabidopsis thaliana | 880 | + | 5 | ACGTG | cis-acting element involved in the abscisic acid responsiveness |
| ABRE | Arabidopsis thaliana | 879 | - | 6 | CACGTG | cis-acting element involved in the abscisic acid responsiveness |

>037399   
+ TGTTTAATTA TTATAATTCT TGAGGTTTTG TGTTTGAAAT GCTGAGGATT TTTCTAGTTC TAGAGAGAGA   
  
  
+ GACAGAGACA GAGAGAAACA AGACATGCAC CTCTCAACCA AATCGTCCCC CACAATTTCA ACGCAAGTCG   
  
  
+ TGCTACCTCT CTCTCTAACT CTCTTTCTCT CTCTAAAGAA TAGTTGAGTT TTTGCTCAAG AAGCAAATTT   
  
  
+ GTCCCACAAC AACAATAGAT TTCCACTTAC TTATAACAAT TATATGATCT TTGAAGGGAA CGGAGAAGGT   
  
  
+ ACGTACGTAC TATATATTAC TTCCCAGTAC TATATTTTTC CCGTCAATTT TTTTGCGTAC ATATAAAGTG   
  
  
+ TGTTATTGAG ATTTGATGAT TTTGTAATGA TTTTATAGGA GGAGCTTACC GTCTACAATT TAAGAAGAAA   
  
  
+ CAATGATTAC GTTAATTGGA ATGTTTAGGC TAATAGTACA TGATTATTGT ACATACGCAC ATGTTGTATA   
  
  
+ GATAAATATT TATATTTCTC CAAGACAAAA ACCCTAAAAT TTATCTTTTT CCACCAACTA CACTCTTGAG   
  
  
+ TTTCACATTT TAACTCTAAT GTTGTTGTCG TAAATTTAGA CTTGTATTTT ATATTCTTTT GTTACATATA   
  
  
+ CATTGTATCA TACTCATGCA TGCATATATA GTAAACTTAT TAAACTCAAT TCTTCATGTA TTTTACGTAA   
  
  
+ ACCAATCGAG TTATTTTGTA TGAGTAATAT ATTTTGCCCT TATGGTATCA TTACGACCAT CAACTAGCTA   
  
  
+ TGTGATTGAA ATGAGAACCA TGAAGAACTA ATTTCGGATT AATGCTAGGA AGAACATATG TAGTAAGTTG   
  
  
+ AGTGATGTGT ACTAATTGTA TGCATCATTT TGTAACATCA CGTGATCCAA AAAAATGTGT TATACGGTTA   
  
  
+ CTCCAAGCAA TGCTCATAAT ATGAATATGT ATACATAAAT GGTTAAAATG CGTATTATCC TAATAATTGA   
  
  
+ CTATAAAATA AAATATGAGA ATACTATATA TATGTATGTG TGTGTGTGTG TGTATGTGTA GGGTTAGTGA   
  
  
+ TGCTTCATGG CATGTGTAGG ATTAGTGATG CTTTATGGCC ACAAACTAGG GCAAGGATTT GGTCTACATA   
  
  
+ ATCACCTATT CAAAAGGAAA TATTGTTCTA TATAATTTTC AGACAGTTTT ATATTCTCTT TATTTATGTA   
  
  
+ TTATTGATGA ATAGTTATGC ACAGGGCATA TCCGGCCGGT GGTTCTTTCA CTATTTTTAT GCATAACAAT   
  
  
+ TGGCCATCAA TGGCTTCTCT TATTTTATAC ATAACAATTG GACATATTGG AAGTGGTCAT CACTATTGTT   
  
  
+ GAGCAGTCAG TCTCAGACTC GATTTTTTAT ACAAACGTAA CCACATAGAG ATTACCCAGC ATAGCTTAAT   
  
  
+ TACCTGCTGG AACCAAACAT GCATGCATAC CTCTTTTTAT TTACGTGTTG TCATTGTCTT TGACTGCTTA   
  
  
+ AGATTCTAAA CAAGTTTAAT AGGTGTTTG  

- ACAAATTAAT AATATTAAGA ACTCCAAAAC ACAAACTTTA CGACTCCTAA AAAGATCAAG ATCTCTCTCT   
  
  
- CTGTCTCTGT CTCTCTTTGT TCTGTACGTG GAGAGTTGGT TTAGCAGGGG GTGTTAAAGT TGCGTTCAGC   
  
  
- ACGATGGAGA GAGAGATTGA GAGAAAGAGA GAGATTTCTT ATCAACTCAA AAACGAGTTC TTCGTTTAAA   
  
  
- CAGGGTGTTG TTGTTATCTA AAGGTGAATG AATATTGTTA ATATACTAGA AACTTCCCTT GCCTCTTCCA   
  
  
- TGCATGCATG ATATATAATG AAGGGTCATG ATATAAAAAG GGCAGTTAAA AAAACGCATG TATATTTCAC   
  
  
- ACAATAACTC TAAACTACTA AAACATTACT AAAATATCCT CCTCGAATGG CAGATGTTAA ATTCTTCTTT   
  
  
- GTTACTAATG CAATTAACCT TACAAATCCG ATTATCATGT ACTAATAACA TGTATGCGTG TACAACATAT   
  
  
- CTATTTATAA ATATAAAGAG GTTCTGTTTT TGGGATTTTA AATAGAAAAA GGTGGTTGAT GTGAGAACTC   
  
  
- AAAGTGTAAA ATTGAGATTA CAACAACAGC ATTTAAATCT GAACATAAAA TATAAGAAAA CAATGTATAT   
  
  
- GTAACATAGT ATGAGTACGT ACGTATATAT CATTTGAATA ATTTGAGTTA AGAAGTACAT AAAATGCATT   
  
  
- TGGTTAGCTC AATAAAACAT ACTCATTATA TAAAACGGGA ATACCATAGT AATGCTGGTA GTTGATCGAT   
  
  
- ACACTAACTT TACTCTTGGT ACTTCTTGAT TAAAGCCTAA TTACGATCCT TCTTGTATAC ATCATTCAAC   
  
  
- TCACTACACA TGATTAACAT ACGTAGTAAA ACATTGTAGT GCACTAGGTT TTTTTACACA ATATGCCAAT   
  
  
- GAGGTTCGTT ACGAGTATTA TACTTATACA TATGTATTTA CCAATTTTAC GCATAATAGG ATTATTAACT   
  
  
- GATATTTTAT TTTATACTCT TATGATATAT ATACATACAC ACACACACAC ACATACACAT CCCAATCACT   
  
  
- ACGAAGTACC GTACACATCC TAATCACTAC GAAATACCGG TGTTTGATCC CGTTCCTAAA CCAGATGTAT   
  
  
- TAGTGGATAA GTTTTCCTTT ATAACAAGAT ATATTAAAAG TCTGTCAAAA TATAAGAGAA ATAAATACAT   
  
  
- AATAACTACT TATCAATACG TGTCCCGTAT AGGCCGGCCA CCAAGAAAGT GATAAAAATA CGTATTGTTA   
  
  
- ACCGGTAGTT ACCGAAGAGA ATAAAATATG TATTGTTAAC CTGTATAACC TTCACCAGTA GTGATAACAA   
  
  
- CTCGTCAGTC AGAGTCTGAG CTAAAAAATA TGTTTGCATT GGTGTATCTC TAATGGGTCG TATCGAATTA   
  
  
- ATGGACGACC TTGGTTTGTA CGTACGTATG GAGAAAAATA AATGCACAAC AGTAACAGAA ACTGACGAAT   
  
  
- TCTAAGATTT GTTCAAATTA TCCACAAAC

+     ABRE3a

| Site Name | Organism | Position | Strand | Matrix score. | sequence | function |
| --- | --- | --- | --- | --- | --- | --- |
| ABRE3a | Zea mays | 1442 | + | 6 | TACGTG |  |

>037399   
+ TGTTTAATTA TTATAATTCT TGAGGTTTTG TGTTTGAAAT GCTGAGGATT TTTCTAGTTC TAGAGAGAGA   
  
  
+ GACAGAGACA GAGAGAAACA AGACATGCAC CTCTCAACCA AATCGTCCCC CACAATTTCA ACGCAAGTCG   
  
  
+ TGCTACCTCT CTCTCTAACT CTCTTTCTCT CTCTAAAGAA TAGTTGAGTT TTTGCTCAAG AAGCAAATTT   
  
  
+ GTCCCACAAC AACAATAGAT TTCCACTTAC TTATAACAAT TATATGATCT TTGAAGGGAA CGGAGAAGGT   
  
  
+ ACGTACGTAC TATATATTAC TTCCCAGTAC TATATTTTTC CCGTCAATTT TTTTGCGTAC ATATAAAGTG   
  
  
+ TGTTATTGAG ATTTGATGAT TTTGTAATGA TTTTATAGGA GGAGCTTACC GTCTACAATT TAAGAAGAAA   
  
  
+ CAATGATTAC GTTAATTGGA ATGTTTAGGC TAATAGTACA TGATTATTGT ACATACGCAC ATGTTGTATA   
  
  
+ GATAAATATT TATATTTCTC CAAGACAAAA ACCCTAAAAT TTATCTTTTT CCACCAACTA CACTCTTGAG   
  
  
+ TTTCACATTT TAACTCTAAT GTTGTTGTCG TAAATTTAGA CTTGTATTTT ATATTCTTTT GTTACATATA   
  
  
+ CATTGTATCA TACTCATGCA TGCATATATA GTAAACTTAT TAAACTCAAT TCTTCATGTA TTTTACGTAA   
  
  
+ ACCAATCGAG TTATTTTGTA TGAGTAATAT ATTTTGCCCT TATGGTATCA TTACGACCAT CAACTAGCTA   
  
  
+ TGTGATTGAA ATGAGAACCA TGAAGAACTA ATTTCGGATT AATGCTAGGA AGAACATATG TAGTAAGTTG   
  
  
+ AGTGATGTGT ACTAATTGTA TGCATCATTT TGTAACATCA CGTGATCCAA AAAAATGTGT TATACGGTTA   
  
  
+ CTCCAAGCAA TGCTCATAAT ATGAATATGT ATACATAAAT GGTTAAAATG CGTATTATCC TAATAATTGA   
  
  
+ CTATAAAATA AAATATGAGA ATACTATATA TATGTATGTG TGTGTGTGTG TGTATGTGTA GGGTTAGTGA   
  
  
+ TGCTTCATGG CATGTGTAGG ATTAGTGATG CTTTATGGCC ACAAACTAGG GCAAGGATTT GGTCTACATA   
  
  
+ ATCACCTATT CAAAAGGAAA TATTGTTCTA TATAATTTTC AGACAGTTTT ATATTCTCTT TATTTATGTA   
  
  
+ TTATTGATGA ATAGTTATGC ACAGGGCATA TCCGGCCGGT GGTTCTTTCA CTATTTTTAT GCATAACAAT   
  
  
+ TGGCCATCAA TGGCTTCTCT TATTTTATAC ATAACAATTG GACATATTGG AAGTGGTCAT CACTATTGTT   
  
  
+ GAGCAGTCAG TCTCAGACTC GATTTTTTAT ACAAACGTAA CCACATAGAG ATTACCCAGC ATAGCTTAAT   
  
  
+ TACCTGCTGG AACCAAACAT GCATGCATAC CTCTTTTTAT TTACGTGTTG TCATTGTCTT TGACTGCTTA   
  
  
+ AGATTCTAAA CAAGTTTAAT AGGTGTTTG  

- ACAAATTAAT AATATTAAGA ACTCCAAAAC ACAAACTTTA CGACTCCTAA AAAGATCAAG ATCTCTCTCT   
  
  
- CTGTCTCTGT CTCTCTTTGT TCTGTACGTG GAGAGTTGGT TTAGCAGGGG GTGTTAAAGT TGCGTTCAGC   
  
  
- ACGATGGAGA GAGAGATTGA GAGAAAGAGA GAGATTTCTT ATCAACTCAA AAACGAGTTC TTCGTTTAAA   
  
  
- CAGGGTGTTG TTGTTATCTA AAGGTGAATG AATATTGTTA ATATACTAGA AACTTCCCTT GCCTCTTCCA   
  
  
- TGCATGCATG ATATATAATG AAGGGTCATG ATATAAAAAG GGCAGTTAAA AAAACGCATG TATATTTCAC   
  
  
- ACAATAACTC TAAACTACTA AAACATTACT AAAATATCCT CCTCGAATGG CAGATGTTAA ATTCTTCTTT   
  
  
- GTTACTAATG CAATTAACCT TACAAATCCG ATTATCATGT ACTAATAACA TGTATGCGTG TACAACATAT   
  
  
- CTATTTATAA ATATAAAGAG GTTCTGTTTT TGGGATTTTA AATAGAAAAA GGTGGTTGAT GTGAGAACTC   
  
  
- AAAGTGTAAA ATTGAGATTA CAACAACAGC ATTTAAATCT GAACATAAAA TATAAGAAAA CAATGTATAT   
  
  
- GTAACATAGT ATGAGTACGT ACGTATATAT CATTTGAATA ATTTGAGTTA AGAAGTACAT AAAATGCATT   
  
  
- TGGTTAGCTC AATAAAACAT ACTCATTATA TAAAACGGGA ATACCATAGT AATGCTGGTA GTTGATCGAT   
  
  
- ACACTAACTT TACTCTTGGT ACTTCTTGAT TAAAGCCTAA TTACGATCCT TCTTGTATAC ATCATTCAAC   
  
  
- TCACTACACA TGATTAACAT ACGTAGTAAA ACATTGTAGT GCACTAGGTT TTTTTACACA ATATGCCAAT   
  
  
- GAGGTTCGTT ACGAGTATTA TACTTATACA TATGTATTTA CCAATTTTAC GCATAATAGG ATTATTAACT   
  
  
- GATATTTTAT TTTATACTCT TATGATATAT ATACATACAC ACACACACAC ACATACACAT CCCAATCACT   
  
  
- ACGAAGTACC GTACACATCC TAATCACTAC GAAATACCGG TGTTTGATCC CGTTCCTAAA CCAGATGTAT   
  
  
- TAGTGGATAA GTTTTCCTTT ATAACAAGAT ATATTAAAAG TCTGTCAAAA TATAAGAGAA ATAAATACAT   
  
  
- AATAACTACT TATCAATACG TGTCCCGTAT AGGCCGGCCA CCAAGAAAGT GATAAAAATA CGTATTGTTA   
  
  
- ACCGGTAGTT ACCGAAGAGA ATAAAATATG TATTGTTAAC CTGTATAACC TTCACCAGTA GTGATAACAA   
  
  
- CTCGTCAGTC AGAGTCTGAG CTAAAAAATA TGTTTGCATT GGTGTATCTC TAATGGGTCG TATCGAATTA   
  
  
- ATGGACGACC TTGGTTTGTA CGTACGTATG GAGAAAAATA AATGCACAAC AGTAACAGAA ACTGACGAAT   
  
  
- TCTAAGATTT GTTCAAATTA TCCACAAAC

+     ABRE4

| Site Name | Organism | Position | Strand | Matrix score. | sequence | function |
| --- | --- | --- | --- | --- | --- | --- |
| ABRE4 | Zea mays | 1442 | - | 6 | CACGTA |  |

>037399   
+ TGTTTAATTA TTATAATTCT TGAGGTTTTG TGTTTGAAAT GCTGAGGATT TTTCTAGTTC TAGAGAGAGA   
  
  
+ GACAGAGACA GAGAGAAACA AGACATGCAC CTCTCAACCA AATCGTCCCC CACAATTTCA ACGCAAGTCG   
  
  
+ TGCTACCTCT CTCTCTAACT CTCTTTCTCT CTCTAAAGAA TAGTTGAGTT TTTGCTCAAG AAGCAAATTT   
  
  
+ GTCCCACAAC AACAATAGAT TTCCACTTAC TTATAACAAT TATATGATCT TTGAAGGGAA CGGAGAAGGT   
  
  
+ ACGTACGTAC TATATATTAC TTCCCAGTAC TATATTTTTC CCGTCAATTT TTTTGCGTAC ATATAAAGTG   
  
  
+ TGTTATTGAG ATTTGATGAT TTTGTAATGA TTTTATAGGA GGAGCTTACC GTCTACAATT TAAGAAGAAA   
  
  
+ CAATGATTAC GTTAATTGGA ATGTTTAGGC TAATAGTACA TGATTATTGT ACATACGCAC ATGTTGTATA   
  
  
+ GATAAATATT TATATTTCTC CAAGACAAAA ACCCTAAAAT TTATCTTTTT CCACCAACTA CACTCTTGAG   
  
  
+ TTTCACATTT TAACTCTAAT GTTGTTGTCG TAAATTTAGA CTTGTATTTT ATATTCTTTT GTTACATATA   
  
  
+ CATTGTATCA TACTCATGCA TGCATATATA GTAAACTTAT TAAACTCAAT TCTTCATGTA TTTTACGTAA   
  
  
+ ACCAATCGAG TTATTTTGTA TGAGTAATAT ATTTTGCCCT TATGGTATCA TTACGACCAT CAACTAGCTA   
  
  
+ TGTGATTGAA ATGAGAACCA TGAAGAACTA ATTTCGGATT AATGCTAGGA AGAACATATG TAGTAAGTTG   
  
  
+ AGTGATGTGT ACTAATTGTA TGCATCATTT TGTAACATCA CGTGATCCAA AAAAATGTGT TATACGGTTA   
  
  
+ CTCCAAGCAA TGCTCATAAT ATGAATATGT ATACATAAAT GGTTAAAATG CGTATTATCC TAATAATTGA   
  
  
+ CTATAAAATA AAATATGAGA ATACTATATA TATGTATGTG TGTGTGTGTG TGTATGTGTA GGGTTAGTGA   
  
  
+ TGCTTCATGG CATGTGTAGG ATTAGTGATG CTTTATGGCC ACAAACTAGG GCAAGGATTT GGTCTACATA   
  
  
+ ATCACCTATT CAAAAGGAAA TATTGTTCTA TATAATTTTC AGACAGTTTT ATATTCTCTT TATTTATGTA   
  
  
+ TTATTGATGA ATAGTTATGC ACAGGGCATA TCCGGCCGGT GGTTCTTTCA CTATTTTTAT GCATAACAAT   
  
  
+ TGGCCATCAA TGGCTTCTCT TATTTTATAC ATAACAATTG GACATATTGG AAGTGGTCAT CACTATTGTT   
  
  
+ GAGCAGTCAG TCTCAGACTC GATTTTTTAT ACAAACGTAA CCACATAGAG ATTACCCAGC ATAGCTTAAT   
  
  
+ TACCTGCTGG AACCAAACAT GCATGCATAC CTCTTTTTAT TTACGTGTTG TCATTGTCTT TGACTGCTTA   
  
  
+ AGATTCTAAA CAAGTTTAAT AGGTGTTTG  

- ACAAATTAAT AATATTAAGA ACTCCAAAAC ACAAACTTTA CGACTCCTAA AAAGATCAAG ATCTCTCTCT   
  
  
- CTGTCTCTGT CTCTCTTTGT TCTGTACGTG GAGAGTTGGT TTAGCAGGGG GTGTTAAAGT TGCGTTCAGC   
  
  
- ACGATGGAGA GAGAGATTGA GAGAAAGAGA GAGATTTCTT ATCAACTCAA AAACGAGTTC TTCGTTTAAA   
  
  
- CAGGGTGTTG TTGTTATCTA AAGGTGAATG AATATTGTTA ATATACTAGA AACTTCCCTT GCCTCTTCCA   
  
  
- TGCATGCATG ATATATAATG AAGGGTCATG ATATAAAAAG GGCAGTTAAA AAAACGCATG TATATTTCAC   
  
  
- ACAATAACTC TAAACTACTA AAACATTACT AAAATATCCT CCTCGAATGG CAGATGTTAA ATTCTTCTTT   
  
  
- GTTACTAATG CAATTAACCT TACAAATCCG ATTATCATGT ACTAATAACA TGTATGCGTG TACAACATAT   
  
  
- CTATTTATAA ATATAAAGAG GTTCTGTTTT TGGGATTTTA AATAGAAAAA GGTGGTTGAT GTGAGAACTC   
  
  
- AAAGTGTAAA ATTGAGATTA CAACAACAGC ATTTAAATCT GAACATAAAA TATAAGAAAA CAATGTATAT   
  
  
- GTAACATAGT ATGAGTACGT ACGTATATAT CATTTGAATA ATTTGAGTTA AGAAGTACAT AAAATGCATT   
  
  
- TGGTTAGCTC AATAAAACAT ACTCATTATA TAAAACGGGA ATACCATAGT AATGCTGGTA GTTGATCGAT   
  
  
- ACACTAACTT TACTCTTGGT ACTTCTTGAT TAAAGCCTAA TTACGATCCT TCTTGTATAC ATCATTCAAC   
  
  
- TCACTACACA TGATTAACAT ACGTAGTAAA ACATTGTAGT GCACTAGGTT TTTTTACACA ATATGCCAAT   
  
  
- GAGGTTCGTT ACGAGTATTA TACTTATACA TATGTATTTA CCAATTTTAC GCATAATAGG ATTATTAACT   
  
  
- GATATTTTAT TTTATACTCT TATGATATAT ATACATACAC ACACACACAC ACATACACAT CCCAATCACT   
  
  
- ACGAAGTACC GTACACATCC TAATCACTAC GAAATACCGG TGTTTGATCC CGTTCCTAAA CCAGATGTAT   
  
  
- TAGTGGATAA GTTTTCCTTT ATAACAAGAT ATATTAAAAG TCTGTCAAAA TATAAGAGAA ATAAATACAT   
  
  
- AATAACTACT TATCAATACG TGTCCCGTAT AGGCCGGCCA CCAAGAAAGT GATAAAAATA CGTATTGTTA   
  
  
- ACCGGTAGTT ACCGAAGAGA ATAAAATATG TATTGTTAAC CTGTATAACC TTCACCAGTA GTGATAACAA   
  
  
- CTCGTCAGTC AGAGTCTGAG CTAAAAAATA TGTTTGCATT GGTGTATCTC TAATGGGTCG TATCGAATTA   
  
  
- ATGGACGACC TTGGTTTGTA CGTACGTATG GAGAAAAATA AATGCACAAC AGTAACAGAA ACTGACGAAT   
  
  
- TCTAAGATTT GTTCAAATTA TCCACAAAC

+     AE-box

| Site Name | Organism | Position | Strand | Matrix score. | sequence | function |
| --- | --- | --- | --- | --- | --- | --- |
| AE-box | Arabidopsis thaliana | 416 | + | 8 | AGAAACAA | part of a module for light response |
| AE-box | Arabidopsis thaliana | 84 | + | 8 | AGAAACAA | part of a module for light response |

>037399   
+ TGTTTAATTA TTATAATTCT TGAGGTTTTG TGTTTGAAAT GCTGAGGATT TTTCTAGTTC TAGAGAGAGA   
  
  
+ GACAGAGACA GAGAGAAACA AGACATGCAC CTCTCAACCA AATCGTCCCC CACAATTTCA ACGCAAGTCG   
  
  
+ TGCTACCTCT CTCTCTAACT CTCTTTCTCT CTCTAAAGAA TAGTTGAGTT TTTGCTCAAG AAGCAAATTT   
  
  
+ GTCCCACAAC AACAATAGAT TTCCACTTAC TTATAACAAT TATATGATCT TTGAAGGGAA CGGAGAAGGT   
  
  
+ ACGTACGTAC TATATATTAC TTCCCAGTAC TATATTTTTC CCGTCAATTT TTTTGCGTAC ATATAAAGTG   
  
  
+ TGTTATTGAG ATTTGATGAT TTTGTAATGA TTTTATAGGA GGAGCTTACC GTCTACAATT TAAGAAGAAA   
  
  
+ CAATGATTAC GTTAATTGGA ATGTTTAGGC TAATAGTACA TGATTATTGT ACATACGCAC ATGTTGTATA   
  
  
+ GATAAATATT TATATTTCTC CAAGACAAAA ACCCTAAAAT TTATCTTTTT CCACCAACTA CACTCTTGAG   
  
  
+ TTTCACATTT TAACTCTAAT GTTGTTGTCG TAAATTTAGA CTTGTATTTT ATATTCTTTT GTTACATATA   
  
  
+ CATTGTATCA TACTCATGCA TGCATATATA GTAAACTTAT TAAACTCAAT TCTTCATGTA TTTTACGTAA   
  
  
+ ACCAATCGAG TTATTTTGTA TGAGTAATAT ATTTTGCCCT TATGGTATCA TTACGACCAT CAACTAGCTA   
  
  
+ TGTGATTGAA ATGAGAACCA TGAAGAACTA ATTTCGGATT AATGCTAGGA AGAACATATG TAGTAAGTTG   
  
  
+ AGTGATGTGT ACTAATTGTA TGCATCATTT TGTAACATCA CGTGATCCAA AAAAATGTGT TATACGGTTA   
  
  
+ CTCCAAGCAA TGCTCATAAT ATGAATATGT ATACATAAAT GGTTAAAATG CGTATTATCC TAATAATTGA   
  
  
+ CTATAAAATA AAATATGAGA ATACTATATA TATGTATGTG TGTGTGTGTG TGTATGTGTA GGGTTAGTGA   
  
  
+ TGCTTCATGG CATGTGTAGG ATTAGTGATG CTTTATGGCC ACAAACTAGG GCAAGGATTT GGTCTACATA   
  
  
+ ATCACCTATT CAAAAGGAAA TATTGTTCTA TATAATTTTC AGACAGTTTT ATATTCTCTT TATTTATGTA   
  
  
+ TTATTGATGA ATAGTTATGC ACAGGGCATA TCCGGCCGGT GGTTCTTTCA CTATTTTTAT GCATAACAAT   
  
  
+ TGGCCATCAA TGGCTTCTCT TATTTTATAC ATAACAATTG GACATATTGG AAGTGGTCAT CACTATTGTT   
  
  
+ GAGCAGTCAG TCTCAGACTC GATTTTTTAT ACAAACGTAA CCACATAGAG ATTACCCAGC ATAGCTTAAT   
  
  
+ TACCTGCTGG AACCAAACAT GCATGCATAC CTCTTTTTAT TTACGTGTTG TCATTGTCTT TGACTGCTTA   
  
  
+ AGATTCTAAA CAAGTTTAAT AGGTGTTTG  

- ACAAATTAAT AATATTAAGA ACTCCAAAAC ACAAACTTTA CGACTCCTAA AAAGATCAAG ATCTCTCTCT   
  
  
- CTGTCTCTGT CTCTCTTTGT TCTGTACGTG GAGAGTTGGT TTAGCAGGGG GTGTTAAAGT TGCGTTCAGC   
  
  
- ACGATGGAGA GAGAGATTGA GAGAAAGAGA GAGATTTCTT ATCAACTCAA AAACGAGTTC TTCGTTTAAA   
  
  
- CAGGGTGTTG TTGTTATCTA AAGGTGAATG AATATTGTTA ATATACTAGA AACTTCCCTT GCCTCTTCCA   
  
  
- TGCATGCATG ATATATAATG AAGGGTCATG ATATAAAAAG GGCAGTTAAA AAAACGCATG TATATTTCAC   
  
  
- ACAATAACTC TAAACTACTA AAACATTACT AAAATATCCT CCTCGAATGG CAGATGTTAA ATTCTTCTTT   
  
  
- GTTACTAATG CAATTAACCT TACAAATCCG ATTATCATGT ACTAATAACA TGTATGCGTG TACAACATAT   
  
  
- CTATTTATAA ATATAAAGAG GTTCTGTTTT TGGGATTTTA AATAGAAAAA GGTGGTTGAT GTGAGAACTC   
  
  
- AAAGTGTAAA ATTGAGATTA CAACAACAGC ATTTAAATCT GAACATAAAA TATAAGAAAA CAATGTATAT   
  
  
- GTAACATAGT ATGAGTACGT ACGTATATAT CATTTGAATA ATTTGAGTTA AGAAGTACAT AAAATGCATT   
  
  
- TGGTTAGCTC AATAAAACAT ACTCATTATA TAAAACGGGA ATACCATAGT AATGCTGGTA GTTGATCGAT   
  
  
- ACACTAACTT TACTCTTGGT ACTTCTTGAT TAAAGCCTAA TTACGATCCT TCTTGTATAC ATCATTCAAC   
  
  
- TCACTACACA TGATTAACAT ACGTAGTAAA ACATTGTAGT GCACTAGGTT TTTTTACACA ATATGCCAAT   
  
  
- GAGGTTCGTT ACGAGTATTA TACTTATACA TATGTATTTA CCAATTTTAC GCATAATAGG ATTATTAACT   
  
  
- GATATTTTAT TTTATACTCT TATGATATAT ATACATACAC ACACACACAC ACATACACAT CCCAATCACT   
  
  
- ACGAAGTACC GTACACATCC TAATCACTAC GAAATACCGG TGTTTGATCC CGTTCCTAAA CCAGATGTAT   
  
  
- TAGTGGATAA GTTTTCCTTT ATAACAAGAT ATATTAAAAG TCTGTCAAAA TATAAGAGAA ATAAATACAT   
  
  
- AATAACTACT TATCAATACG TGTCCCGTAT AGGCCGGCCA CCAAGAAAGT GATAAAAATA CGTATTGTTA   
  
  
- ACCGGTAGTT ACCGAAGAGA ATAAAATATG TATTGTTAAC CTGTATAACC TTCACCAGTA GTGATAACAA   
  
  
- CTCGTCAGTC AGAGTCTGAG CTAAAAAATA TGTTTGCATT GGTGTATCTC TAATGGGTCG TATCGAATTA   
  
  
- ATGGACGACC TTGGTTTGTA CGTACGTATG GAGAAAAATA AATGCACAAC AGTAACAGAA ACTGACGAAT   
  
  
- TCTAAGATTT GTTCAAATTA TCCACAAAC

+     ARE

| Site Name | Organism | Position | Strand | Matrix score. | sequence | function |
| --- | --- | --- | --- | --- | --- | --- |
| ARE | Zea mays | 699 | + | 6 | AAACCA | cis-acting regulatory element essential for the anaerobic induction |

>037399   
+ TGTTTAATTA TTATAATTCT TGAGGTTTTG TGTTTGAAAT GCTGAGGATT TTTCTAGTTC TAGAGAGAGA   
  
  
+ GACAGAGACA GAGAGAAACA AGACATGCAC CTCTCAACCA AATCGTCCCC CACAATTTCA ACGCAAGTCG   
  
  
+ TGCTACCTCT CTCTCTAACT CTCTTTCTCT CTCTAAAGAA TAGTTGAGTT TTTGCTCAAG AAGCAAATTT   
  
  
+ GTCCCACAAC AACAATAGAT TTCCACTTAC TTATAACAAT TATATGATCT TTGAAGGGAA CGGAGAAGGT   
  
  
+ ACGTACGTAC TATATATTAC TTCCCAGTAC TATATTTTTC CCGTCAATTT TTTTGCGTAC ATATAAAGTG   
  
  
+ TGTTATTGAG ATTTGATGAT TTTGTAATGA TTTTATAGGA GGAGCTTACC GTCTACAATT TAAGAAGAAA   
  
  
+ CAATGATTAC GTTAATTGGA ATGTTTAGGC TAATAGTACA TGATTATTGT ACATACGCAC ATGTTGTATA   
  
  
+ GATAAATATT TATATTTCTC CAAGACAAAA ACCCTAAAAT TTATCTTTTT CCACCAACTA CACTCTTGAG   
  
  
+ TTTCACATTT TAACTCTAAT GTTGTTGTCG TAAATTTAGA CTTGTATTTT ATATTCTTTT GTTACATATA   
  
  
+ CATTGTATCA TACTCATGCA TGCATATATA GTAAACTTAT TAAACTCAAT TCTTCATGTA TTTTACGTAA   
  
  
+ ACCAATCGAG TTATTTTGTA TGAGTAATAT ATTTTGCCCT TATGGTATCA TTACGACCAT CAACTAGCTA   
  
  
+ TGTGATTGAA ATGAGAACCA TGAAGAACTA ATTTCGGATT AATGCTAGGA AGAACATATG TAGTAAGTTG   
  
  
+ AGTGATGTGT ACTAATTGTA TGCATCATTT TGTAACATCA CGTGATCCAA AAAAATGTGT TATACGGTTA   
  
  
+ CTCCAAGCAA TGCTCATAAT ATGAATATGT ATACATAAAT GGTTAAAATG CGTATTATCC TAATAATTGA   
  
  
+ CTATAAAATA AAATATGAGA ATACTATATA TATGTATGTG TGTGTGTGTG TGTATGTGTA GGGTTAGTGA   
  
  
+ TGCTTCATGG CATGTGTAGG ATTAGTGATG CTTTATGGCC ACAAACTAGG GCAAGGATTT GGTCTACATA   
  
  
+ ATCACCTATT CAAAAGGAAA TATTGTTCTA TATAATTTTC AGACAGTTTT ATATTCTCTT TATTTATGTA   
  
  
+ TTATTGATGA ATAGTTATGC ACAGGGCATA TCCGGCCGGT GGTTCTTTCA CTATTTTTAT GCATAACAAT   
  
  
+ TGGCCATCAA TGGCTTCTCT TATTTTATAC ATAACAATTG GACATATTGG AAGTGGTCAT CACTATTGTT   
  
  
+ GAGCAGTCAG TCTCAGACTC GATTTTTTAT ACAAACGTAA CCACATAGAG ATTACCCAGC ATAGCTTAAT   
  
  
+ TACCTGCTGG AACCAAACAT GCATGCATAC CTCTTTTTAT TTACGTGTTG TCATTGTCTT TGACTGCTTA   
  
  
+ AGATTCTAAA CAAGTTTAAT AGGTGTTTG  

- ACAAATTAAT AATATTAAGA ACTCCAAAAC ACAAACTTTA CGACTCCTAA AAAGATCAAG ATCTCTCTCT   
  
  
- CTGTCTCTGT CTCTCTTTGT TCTGTACGTG GAGAGTTGGT TTAGCAGGGG GTGTTAAAGT TGCGTTCAGC   
  
  
- ACGATGGAGA GAGAGATTGA GAGAAAGAGA GAGATTTCTT ATCAACTCAA AAACGAGTTC TTCGTTTAAA   
  
  
- CAGGGTGTTG TTGTTATCTA AAGGTGAATG AATATTGTTA ATATACTAGA AACTTCCCTT GCCTCTTCCA   
  
  
- TGCATGCATG ATATATAATG AAGGGTCATG ATATAAAAAG GGCAGTTAAA AAAACGCATG TATATTTCAC   
  
  
- ACAATAACTC TAAACTACTA AAACATTACT AAAATATCCT CCTCGAATGG CAGATGTTAA ATTCTTCTTT   
  
  
- GTTACTAATG CAATTAACCT TACAAATCCG ATTATCATGT ACTAATAACA TGTATGCGTG TACAACATAT   
  
  
- CTATTTATAA ATATAAAGAG GTTCTGTTTT TGGGATTTTA AATAGAAAAA GGTGGTTGAT GTGAGAACTC   
  
  
- AAAGTGTAAA ATTGAGATTA CAACAACAGC ATTTAAATCT GAACATAAAA TATAAGAAAA CAATGTATAT   
  
  
- GTAACATAGT ATGAGTACGT ACGTATATAT CATTTGAATA ATTTGAGTTA AGAAGTACAT AAAATGCATT   
  
  
- TGGTTAGCTC AATAAAACAT ACTCATTATA TAAAACGGGA ATACCATAGT AATGCTGGTA GTTGATCGAT   
  
  
- ACACTAACTT TACTCTTGGT ACTTCTTGAT TAAAGCCTAA TTACGATCCT TCTTGTATAC ATCATTCAAC   
  
  
- TCACTACACA TGATTAACAT ACGTAGTAAA ACATTGTAGT GCACTAGGTT TTTTTACACA ATATGCCAAT   
  
  
- GAGGTTCGTT ACGAGTATTA TACTTATACA TATGTATTTA CCAATTTTAC GCATAATAGG ATTATTAACT   
  
  
- GATATTTTAT TTTATACTCT TATGATATAT ATACATACAC ACACACACAC ACATACACAT CCCAATCACT   
  
  
- ACGAAGTACC GTACACATCC TAATCACTAC GAAATACCGG TGTTTGATCC CGTTCCTAAA CCAGATGTAT   
  
  
- TAGTGGATAA GTTTTCCTTT ATAACAAGAT ATATTAAAAG TCTGTCAAAA TATAAGAGAA ATAAATACAT   
  
  
- AATAACTACT TATCAATACG TGTCCCGTAT AGGCCGGCCA CCAAGAAAGT GATAAAAATA CGTATTGTTA   
  
  
- ACCGGTAGTT ACCGAAGAGA ATAAAATATG TATTGTTAAC CTGTATAACC TTCACCAGTA GTGATAACAA   
  
  
- CTCGTCAGTC AGAGTCTGAG CTAAAAAATA TGTTTGCATT GGTGTATCTC TAATGGGTCG TATCGAATTA   
  
  
- ATGGACGACC TTGGTTTGTA CGTACGTATG GAGAAAAATA AATGCACAAC AGTAACAGAA ACTGACGAAT   
  
  
- TCTAAGATTT GTTCAAATTA TCCACAAAC

+     AT~TATA-box

| Site Name | Organism | Position | Strand | Matrix score. | sequence | function |
| --- | --- | --- | --- | --- | --- | --- |
| AT~TATA-box | Arabidopsis thaliana | 1149 | - | 6 | TATATA |  |
| AT~TATA-box | Arabidopsis thaliana | 1005 | - | 6 | TATATA |  |
| AT~TATA-box | Arabidopsis thaliana | 291 | + | 6 | TATATA |  |
| AT~TATA-box | Arabidopsis thaliana | 1007 | - | 6 | TATATA |  |
| AT~TATA-box | Arabidopsis thaliana | 655 | + | 6 | TATATA |  |

>037399   
+ TGTTTAATTA TTATAATTCT TGAGGTTTTG TGTTTGAAAT GCTGAGGATT TTTCTAGTTC TAGAGAGAGA   
  
  
+ GACAGAGACA GAGAGAAACA AGACATGCAC CTCTCAACCA AATCGTCCCC CACAATTTCA ACGCAAGTCG   
  
  
+ TGCTACCTCT CTCTCTAACT CTCTTTCTCT CTCTAAAGAA TAGTTGAGTT TTTGCTCAAG AAGCAAATTT   
  
  
+ GTCCCACAAC AACAATAGAT TTCCACTTAC TTATAACAAT TATATGATCT TTGAAGGGAA CGGAGAAGGT   
  
  
+ ACGTACGTAC TATATATTAC TTCCCAGTAC TATATTTTTC CCGTCAATTT TTTTGCGTAC ATATAAAGTG   
  
  
+ TGTTATTGAG ATTTGATGAT TTTGTAATGA TTTTATAGGA GGAGCTTACC GTCTACAATT TAAGAAGAAA   
  
  
+ CAATGATTAC GTTAATTGGA ATGTTTAGGC TAATAGTACA TGATTATTGT ACATACGCAC ATGTTGTATA   
  
  
+ GATAAATATT TATATTTCTC CAAGACAAAA ACCCTAAAAT TTATCTTTTT CCACCAACTA CACTCTTGAG   
  
  
+ TTTCACATTT TAACTCTAAT GTTGTTGTCG TAAATTTAGA CTTGTATTTT ATATTCTTTT GTTACATATA   
  
  
+ CATTGTATCA TACTCATGCA TGCATATATA GTAAACTTAT TAAACTCAAT TCTTCATGTA TTTTACGTAA   
  
  
+ ACCAATCGAG TTATTTTGTA TGAGTAATAT ATTTTGCCCT TATGGTATCA TTACGACCAT CAACTAGCTA   
  
  
+ TGTGATTGAA ATGAGAACCA TGAAGAACTA ATTTCGGATT AATGCTAGGA AGAACATATG TAGTAAGTTG   
  
  
+ AGTGATGTGT ACTAATTGTA TGCATCATTT TGTAACATCA CGTGATCCAA AAAAATGTGT TATACGGTTA   
  
  
+ CTCCAAGCAA TGCTCATAAT ATGAATATGT ATACATAAAT GGTTAAAATG CGTATTATCC TAATAATTGA   
  
  
+ CTATAAAATA AAATATGAGA ATACTATATA TATGTATGTG TGTGTGTGTG TGTATGTGTA GGGTTAGTGA   
  
  
+ TGCTTCATGG CATGTGTAGG ATTAGTGATG CTTTATGGCC ACAAACTAGG GCAAGGATTT GGTCTACATA   
  
  
+ ATCACCTATT CAAAAGGAAA TATTGTTCTA TATAATTTTC AGACAGTTTT ATATTCTCTT TATTTATGTA   
  
  
+ TTATTGATGA ATAGTTATGC ACAGGGCATA TCCGGCCGGT GGTTCTTTCA CTATTTTTAT GCATAACAAT   
  
  
+ TGGCCATCAA TGGCTTCTCT TATTTTATAC ATAACAATTG GACATATTGG AAGTGGTCAT CACTATTGTT   
  
  
+ GAGCAGTCAG TCTCAGACTC GATTTTTTAT ACAAACGTAA CCACATAGAG ATTACCCAGC ATAGCTTAAT   
  
  
+ TACCTGCTGG AACCAAACAT GCATGCATAC CTCTTTTTAT TTACGTGTTG TCATTGTCTT TGACTGCTTA   
  
  
+ AGATTCTAAA CAAGTTTAAT AGGTGTTTG  

- ACAAATTAAT AATATTAAGA ACTCCAAAAC ACAAACTTTA CGACTCCTAA AAAGATCAAG ATCTCTCTCT   
  
  
- CTGTCTCTGT CTCTCTTTGT TCTGTACGTG GAGAGTTGGT TTAGCAGGGG GTGTTAAAGT TGCGTTCAGC   
  
  
- ACGATGGAGA GAGAGATTGA GAGAAAGAGA GAGATTTCTT ATCAACTCAA AAACGAGTTC TTCGTTTAAA   
  
  
- CAGGGTGTTG TTGTTATCTA AAGGTGAATG AATATTGTTA ATATACTAGA AACTTCCCTT GCCTCTTCCA   
  
  
- TGCATGCATG ATATATAATG AAGGGTCATG ATATAAAAAG GGCAGTTAAA AAAACGCATG TATATTTCAC   
  
  
- ACAATAACTC TAAACTACTA AAACATTACT AAAATATCCT CCTCGAATGG CAGATGTTAA ATTCTTCTTT   
  
  
- GTTACTAATG CAATTAACCT TACAAATCCG ATTATCATGT ACTAATAACA TGTATGCGTG TACAACATAT   
  
  
- CTATTTATAA ATATAAAGAG GTTCTGTTTT TGGGATTTTA AATAGAAAAA GGTGGTTGAT GTGAGAACTC   
  
  
- AAAGTGTAAA ATTGAGATTA CAACAACAGC ATTTAAATCT GAACATAAAA TATAAGAAAA CAATGTATAT   
  
  
- GTAACATAGT ATGAGTACGT ACGTATATAT CATTTGAATA ATTTGAGTTA AGAAGTACAT AAAATGCATT   
  
  
- TGGTTAGCTC AATAAAACAT ACTCATTATA TAAAACGGGA ATACCATAGT AATGCTGGTA GTTGATCGAT   
  
  
- ACACTAACTT TACTCTTGGT ACTTCTTGAT TAAAGCCTAA TTACGATCCT TCTTGTATAC ATCATTCAAC   
  
  
- TCACTACACA TGATTAACAT ACGTAGTAAA ACATTGTAGT GCACTAGGTT TTTTTACACA ATATGCCAAT   
  
  
- GAGGTTCGTT ACGAGTATTA TACTTATACA TATGTATTTA CCAATTTTAC GCATAATAGG ATTATTAACT   
  
  
- GATATTTTAT TTTATACTCT TATGATATAT ATACATACAC ACACACACAC ACATACACAT CCCAATCACT   
  
  
- ACGAAGTACC GTACACATCC TAATCACTAC GAAATACCGG TGTTTGATCC CGTTCCTAAA CCAGATGTAT   
  
  
- TAGTGGATAA GTTTTCCTTT ATAACAAGAT ATATTAAAAG TCTGTCAAAA TATAAGAGAA ATAAATACAT   
  
  
- AATAACTACT TATCAATACG TGTCCCGTAT AGGCCGGCCA CCAAGAAAGT GATAAAAATA CGTATTGTTA   
  
  
- ACCGGTAGTT ACCGAAGAGA ATAAAATATG TATTGTTAAC CTGTATAACC TTCACCAGTA GTGATAACAA   
  
  
- CTCGTCAGTC AGAGTCTGAG CTAAAAAATA TGTTTGCATT GGTGTATCTC TAATGGGTCG TATCGAATTA   
  
  
- ATGGACGACC TTGGTTTGTA CGTACGTATG GAGAAAAATA AATGCACAAC AGTAACAGAA ACTGACGAAT   
  
  
- TCTAAGATTT GTTCAAATTA TCCACAAAC

+     Box 4

| Site Name | Organism | Position | Strand | Matrix score. | sequence | function |
| --- | --- | --- | --- | --- | --- | --- |
| Box 4 | Petroselinum crispum | 808 | - | 6 | ATTAAT | part of a conserved DNA module involved in light responsiveness |

>037399   
+ TGTTTAATTA TTATAATTCT TGAGGTTTTG TGTTTGAAAT GCTGAGGATT TTTCTAGTTC TAGAGAGAGA   
  
  
+ GACAGAGACA GAGAGAAACA AGACATGCAC CTCTCAACCA AATCGTCCCC CACAATTTCA ACGCAAGTCG   
  
  
+ TGCTACCTCT CTCTCTAACT CTCTTTCTCT CTCTAAAGAA TAGTTGAGTT TTTGCTCAAG AAGCAAATTT   
  
  
+ GTCCCACAAC AACAATAGAT TTCCACTTAC TTATAACAAT TATATGATCT TTGAAGGGAA CGGAGAAGGT   
  
  
+ ACGTACGTAC TATATATTAC TTCCCAGTAC TATATTTTTC CCGTCAATTT TTTTGCGTAC ATATAAAGTG   
  
  
+ TGTTATTGAG ATTTGATGAT TTTGTAATGA TTTTATAGGA GGAGCTTACC GTCTACAATT TAAGAAGAAA   
  
  
+ CAATGATTAC GTTAATTGGA ATGTTTAGGC TAATAGTACA TGATTATTGT ACATACGCAC ATGTTGTATA   
  
  
+ GATAAATATT TATATTTCTC CAAGACAAAA ACCCTAAAAT TTATCTTTTT CCACCAACTA CACTCTTGAG   
  
  
+ TTTCACATTT TAACTCTAAT GTTGTTGTCG TAAATTTAGA CTTGTATTTT ATATTCTTTT GTTACATATA   
  
  
+ CATTGTATCA TACTCATGCA TGCATATATA GTAAACTTAT TAAACTCAAT TCTTCATGTA TTTTACGTAA   
  
  
+ ACCAATCGAG TTATTTTGTA TGAGTAATAT ATTTTGCCCT TATGGTATCA TTACGACCAT CAACTAGCTA   
  
  
+ TGTGATTGAA ATGAGAACCA TGAAGAACTA ATTTCGGATT AATGCTAGGA AGAACATATG TAGTAAGTTG   
  
  
+ AGTGATGTGT ACTAATTGTA TGCATCATTT TGTAACATCA CGTGATCCAA AAAAATGTGT TATACGGTTA   
  
  
+ CTCCAAGCAA TGCTCATAAT ATGAATATGT ATACATAAAT GGTTAAAATG CGTATTATCC TAATAATTGA   
  
  
+ CTATAAAATA AAATATGAGA ATACTATATA TATGTATGTG TGTGTGTGTG TGTATGTGTA GGGTTAGTGA   
  
  
+ TGCTTCATGG CATGTGTAGG ATTAGTGATG CTTTATGGCC ACAAACTAGG GCAAGGATTT GGTCTACATA   
  
  
+ ATCACCTATT CAAAAGGAAA TATTGTTCTA TATAATTTTC AGACAGTTTT ATATTCTCTT TATTTATGTA   
  
  
+ TTATTGATGA ATAGTTATGC ACAGGGCATA TCCGGCCGGT GGTTCTTTCA CTATTTTTAT GCATAACAAT   
  
  
+ TGGCCATCAA TGGCTTCTCT TATTTTATAC ATAACAATTG GACATATTGG AAGTGGTCAT CACTATTGTT   
  
  
+ GAGCAGTCAG TCTCAGACTC GATTTTTTAT ACAAACGTAA CCACATAGAG ATTACCCAGC ATAGCTTAAT   
  
  
+ TACCTGCTGG AACCAAACAT GCATGCATAC CTCTTTTTAT TTACGTGTTG TCATTGTCTT TGACTGCTTA   
  
  
+ AGATTCTAAA CAAGTTTAAT AGGTGTTTG  

- ACAAATTAAT AATATTAAGA ACTCCAAAAC ACAAACTTTA CGACTCCTAA AAAGATCAAG ATCTCTCTCT   
  
  
- CTGTCTCTGT CTCTCTTTGT TCTGTACGTG GAGAGTTGGT TTAGCAGGGG GTGTTAAAGT TGCGTTCAGC   
  
  
- ACGATGGAGA GAGAGATTGA GAGAAAGAGA GAGATTTCTT ATCAACTCAA AAACGAGTTC TTCGTTTAAA   
  
  
- CAGGGTGTTG TTGTTATCTA AAGGTGAATG AATATTGTTA ATATACTAGA AACTTCCCTT GCCTCTTCCA   
  
  
- TGCATGCATG ATATATAATG AAGGGTCATG ATATAAAAAG GGCAGTTAAA AAAACGCATG TATATTTCAC   
  
  
- ACAATAACTC TAAACTACTA AAACATTACT AAAATATCCT CCTCGAATGG CAGATGTTAA ATTCTTCTTT   
  
  
- GTTACTAATG CAATTAACCT TACAAATCCG ATTATCATGT ACTAATAACA TGTATGCGTG TACAACATAT   
  
  
- CTATTTATAA ATATAAAGAG GTTCTGTTTT TGGGATTTTA AATAGAAAAA GGTGGTTGAT GTGAGAACTC   
  
  
- AAAGTGTAAA ATTGAGATTA CAACAACAGC ATTTAAATCT GAACATAAAA TATAAGAAAA CAATGTATAT   
  
  
- GTAACATAGT ATGAGTACGT ACGTATATAT CATTTGAATA ATTTGAGTTA AGAAGTACAT AAAATGCATT   
  
  
- TGGTTAGCTC AATAAAACAT ACTCATTATA TAAAACGGGA ATACCATAGT AATGCTGGTA GTTGATCGAT   
  
  
- ACACTAACTT TACTCTTGGT ACTTCTTGAT TAAAGCCTAA TTACGATCCT TCTTGTATAC ATCATTCAAC   
  
  
- TCACTACACA TGATTAACAT ACGTAGTAAA ACATTGTAGT GCACTAGGTT TTTTTACACA ATATGCCAAT   
  
  
- GAGGTTCGTT ACGAGTATTA TACTTATACA TATGTATTTA CCAATTTTAC GCATAATAGG ATTATTAACT   
  
  
- GATATTTTAT TTTATACTCT TATGATATAT ATACATACAC ACACACACAC ACATACACAT CCCAATCACT   
  
  
- ACGAAGTACC GTACACATCC TAATCACTAC GAAATACCGG TGTTTGATCC CGTTCCTAAA CCAGATGTAT   
  
  
- TAGTGGATAA GTTTTCCTTT ATAACAAGAT ATATTAAAAG TCTGTCAAAA TATAAGAGAA ATAAATACAT   
  
  
- AATAACTACT TATCAATACG TGTCCCGTAT AGGCCGGCCA CCAAGAAAGT GATAAAAATA CGTATTGTTA   
  
  
- ACCGGTAGTT ACCGAAGAGA ATAAAATATG TATTGTTAAC CTGTATAACC TTCACCAGTA GTGATAACAA   
  
  
- CTCGTCAGTC AGAGTCTGAG CTAAAAAATA TGTTTGCATT GGTGTATCTC TAATGGGTCG TATCGAATTA   
  
  
- ATGGACGACC TTGGTTTGTA CGTACGTATG GAGAAAAATA AATGCACAAC AGTAACAGAA ACTGACGAAT   
  
  
- TCTAAGATTT GTTCAAATTA TCCACAAAC

+     CAAT-box

| Site Name | Organism | Position | Strand | Matrix score. | sequence | function |
| --- | --- | --- | --- | --- | --- | --- |
| CAAT-box | Arabidopsis thaliana | 1306 | - | 5 | CCAAT | common cis-acting element in promoter and enhancer regions |
| CAAT-box | Arabidopsis thaliana | 1297 | - | 5 | CCAAT | common cis-acting element in promoter and enhancer regions |
| CAAT-box | Nicotiana glutinosa | 1325 | - | 4 | CAAT |  |
| CAAT-box | Nicotiana glutinosa | 1453 | - | 4 | CAAT |  |
| CAAT-box | Nicotiana glutinosa | 1268 | + | 4 | CAAT |  |
| CAAT-box | Nicotiana glutinosa | 1295 | + | 4 | CAAT |  |
| CAAT-box | Nicotiana glutinosa | 1257 | + | 4 | CAAT |  |
| CAAT-box | Arabidopsis thaliana | 1259 | - | 5 | CCAAT | common cis-acting element in promoter and enhancer regions |
| CAAT-box | Nicotiana glutinosa | 976 | - | 4 | CAAT |  |
| CAAT-box | Pisum sativum | 1107 | - | 5 | CAAAT | common cis-acting element in promoter and enhancer regions |
| CAAT-box | Nicotiana glutinosa | 1142 | - | 4 | CAAT |  |
| CAAT-box | Nicotiana glutinosa | 1193 | - | 4 | CAAT |  |
| CAAT-box | Nicotiana glutinosa | 775 | - | 4 | CAAT |  |
| CAAT-box | Nicotiana glutinosa | 703 | + | 4 | CAAT |  |
| CAAT-box | Nicotiana glutinosa | 855 | - | 4 | CAAT |  |
| CAAT-box | Arabidopsis thaliana | 435 | - | 5 | CCAAT | common cis-acting element in promoter and enhancer regions |
| CAAT-box | Nicotiana glutinosa | 406 | + | 4 | CAAT |  |
| CAAT-box | Pisum sativum | 361 | - | 5 | CAAAT | common cis-acting element in promoter and enhancer regions |
| CAAT-box | Nicotiana glutinosa | 466 | - | 4 | CAAT |  |
| CAAT-box | Nicotiana glutinosa | 632 | - | 4 | CAAT |  |
| CAAT-box | Arabidopsis thaliana | 702 | + | 5 | CCAAT | common cis-acting element in promoter and enhancer regions |
| CAAT-box | Nicotiana glutinosa | 677 | + | 4 | CAAT |  |
| CAAT-box | Nicotiana glutinosa | 123 | + | 4 | CAAT |  |
| CAAT-box | Nicotiana glutinosa | 355 | - | 4 | CAAT |  |
| CAAT-box | Pisum sativum | 109 | + | 5 | CAAAT | common cis-acting element in promoter and enhancer regions |
| CAAT-box | Nicotiana glutinosa | 325 | + | 4 | CAAT |  |
| CAAT-box | Nicotiana glutinosa | 421 | + | 4 | CAAT |  |
| CAAT-box | Nicotiana glutinosa | 223 | + | 4 | CAAT |  |
| CAAT-box | Pisum sativum | 207 | - | 5 | CAAAT | common cis-acting element in promoter and enhancer regions |
| CAAT-box | Pisum sativum | 204 | + | 5 | CAAAT | common cis-acting element in promoter and enhancer regions |
| CAAT-box | Nicotiana glutinosa | 247 | + | 4 | CAAT |  |
| CAAT-box | Nicotiana glutinosa | 918 | + | 4 | CAAT |  |

>037399   
+ TGTTTAATTA TTATAATTCT TGAGGTTTTG TGTTTGAAAT GCTGAGGATT TTTCTAGTTC TAGAGAGAGA   
  
  
+ GACAGAGACA GAGAGAAACA AGACATGCAC CTCTCAACCA AATCGTCCCC CACAATTTCA ACGCAAGTCG   
  
  
+ TGCTACCTCT CTCTCTAACT CTCTTTCTCT CTCTAAAGAA TAGTTGAGTT TTTGCTCAAG AAGCAAATTT   
  
  
+ GTCCCACAAC AACAATAGAT TTCCACTTAC TTATAACAAT TATATGATCT TTGAAGGGAA CGGAGAAGGT   
  
  
+ ACGTACGTAC TATATATTAC TTCCCAGTAC TATATTTTTC CCGTCAATTT TTTTGCGTAC ATATAAAGTG   
  
  
+ TGTTATTGAG ATTTGATGAT TTTGTAATGA TTTTATAGGA GGAGCTTACC GTCTACAATT TAAGAAGAAA   
  
  
+ CAATGATTAC GTTAATTGGA ATGTTTAGGC TAATAGTACA TGATTATTGT ACATACGCAC ATGTTGTATA   
  
  
+ GATAAATATT TATATTTCTC CAAGACAAAA ACCCTAAAAT TTATCTTTTT CCACCAACTA CACTCTTGAG   
  
  
+ TTTCACATTT TAACTCTAAT GTTGTTGTCG TAAATTTAGA CTTGTATTTT ATATTCTTTT GTTACATATA   
  
  
+ CATTGTATCA TACTCATGCA TGCATATATA GTAAACTTAT TAAACTCAAT TCTTCATGTA TTTTACGTAA   
  
  
+ ACCAATCGAG TTATTTTGTA TGAGTAATAT ATTTTGCCCT TATGGTATCA TTACGACCAT CAACTAGCTA   
  
  
+ TGTGATTGAA ATGAGAACCA TGAAGAACTA ATTTCGGATT AATGCTAGGA AGAACATATG TAGTAAGTTG   
  
  
+ AGTGATGTGT ACTAATTGTA TGCATCATTT TGTAACATCA CGTGATCCAA AAAAATGTGT TATACGGTTA   
  
  
+ CTCCAAGCAA TGCTCATAAT ATGAATATGT ATACATAAAT GGTTAAAATG CGTATTATCC TAATAATTGA   
  
  
+ CTATAAAATA AAATATGAGA ATACTATATA TATGTATGTG TGTGTGTGTG TGTATGTGTA GGGTTAGTGA   
  
  
+ TGCTTCATGG CATGTGTAGG ATTAGTGATG CTTTATGGCC ACAAACTAGG GCAAGGATTT GGTCTACATA   
  
  
+ ATCACCTATT CAAAAGGAAA TATTGTTCTA TATAATTTTC AGACAGTTTT ATATTCTCTT TATTTATGTA   
  
  
+ TTATTGATGA ATAGTTATGC ACAGGGCATA TCCGGCCGGT GGTTCTTTCA CTATTTTTAT GCATAACAAT   
  
  
+ TGGCCATCAA TGGCTTCTCT TATTTTATAC ATAACAATTG GACATATTGG AAGTGGTCAT CACTATTGTT   
  
  
+ GAGCAGTCAG TCTCAGACTC GATTTTTTAT ACAAACGTAA CCACATAGAG ATTACCCAGC ATAGCTTAAT   
  
  
+ TACCTGCTGG AACCAAACAT GCATGCATAC CTCTTTTTAT TTACGTGTTG TCATTGTCTT TGACTGCTTA   
  
  
+ AGATTCTAAA CAAGTTTAAT AGGTGTTTG  

- ACAAATTAAT AATATTAAGA ACTCCAAAAC ACAAACTTTA CGACTCCTAA AAAGATCAAG ATCTCTCTCT   
  
  
- CTGTCTCTGT CTCTCTTTGT TCTGTACGTG GAGAGTTGGT TTAGCAGGGG GTGTTAAAGT TGCGTTCAGC   
  
  
- ACGATGGAGA GAGAGATTGA GAGAAAGAGA GAGATTTCTT ATCAACTCAA AAACGAGTTC TTCGTTTAAA   
  
  
- CAGGGTGTTG TTGTTATCTA AAGGTGAATG AATATTGTTA ATATACTAGA AACTTCCCTT GCCTCTTCCA   
  
  
- TGCATGCATG ATATATAATG AAGGGTCATG ATATAAAAAG GGCAGTTAAA AAAACGCATG TATATTTCAC   
  
  
- ACAATAACTC TAAACTACTA AAACATTACT AAAATATCCT CCTCGAATGG CAGATGTTAA ATTCTTCTTT   
  
  
- GTTACTAATG CAATTAACCT TACAAATCCG ATTATCATGT ACTAATAACA TGTATGCGTG TACAACATAT   
  
  
- CTATTTATAA ATATAAAGAG GTTCTGTTTT TGGGATTTTA AATAGAAAAA GGTGGTTGAT GTGAGAACTC   
  
  
- AAAGTGTAAA ATTGAGATTA CAACAACAGC ATTTAAATCT GAACATAAAA TATAAGAAAA CAATGTATAT   
  
  
- GTAACATAGT ATGAGTACGT ACGTATATAT CATTTGAATA ATTTGAGTTA AGAAGTACAT AAAATGCATT   
  
  
- TGGTTAGCTC AATAAAACAT ACTCATTATA TAAAACGGGA ATACCATAGT AATGCTGGTA GTTGATCGAT   
  
  
- ACACTAACTT TACTCTTGGT ACTTCTTGAT TAAAGCCTAA TTACGATCCT TCTTGTATAC ATCATTCAAC   
  
  
- TCACTACACA TGATTAACAT ACGTAGTAAA ACATTGTAGT GCACTAGGTT TTTTTACACA ATATGCCAAT   
  
  
- GAGGTTCGTT ACGAGTATTA TACTTATACA TATGTATTTA CCAATTTTAC GCATAATAGG ATTATTAACT   
  
  
- GATATTTTAT TTTATACTCT TATGATATAT ATACATACAC ACACACACAC ACATACACAT CCCAATCACT   
  
  
- ACGAAGTACC GTACACATCC TAATCACTAC GAAATACCGG TGTTTGATCC CGTTCCTAAA CCAGATGTAT   
  
  
- TAGTGGATAA GTTTTCCTTT ATAACAAGAT ATATTAAAAG TCTGTCAAAA TATAAGAGAA ATAAATACAT   
  
  
- AATAACTACT TATCAATACG TGTCCCGTAT AGGCCGGCCA CCAAGAAAGT GATAAAAATA CGTATTGTTA   
  
  
- ACCGGTAGTT ACCGAAGAGA ATAAAATATG TATTGTTAAC CTGTATAACC TTCACCAGTA GTGATAACAA   
  
  
- CTCGTCAGTC AGAGTCTGAG CTAAAAAATA TGTTTGCATT GGTGTATCTC TAATGGGTCG TATCGAATTA   
  
  
- ATGGACGACC TTGGTTTGTA CGTACGTATG GAGAAAAATA AATGCACAAC AGTAACAGAA ACTGACGAAT   
  
  
- TCTAAGATTT GTTCAAATTA TCCACAAAC

+     CGTCA-motif

| Site Name | Organism | Position | Strand | Matrix score. | sequence | function |
| --- | --- | --- | --- | --- | --- | --- |
| CGTCA-motif | Hordeum vulgare | 322 | + | 5 | CGTCA | cis-acting regulatory element involved in the MeJA-responsiveness |

>037399   
+ TGTTTAATTA TTATAATTCT TGAGGTTTTG TGTTTGAAAT GCTGAGGATT TTTCTAGTTC TAGAGAGAGA   
  
  
+ GACAGAGACA GAGAGAAACA AGACATGCAC CTCTCAACCA AATCGTCCCC CACAATTTCA ACGCAAGTCG   
  
  
+ TGCTACCTCT CTCTCTAACT CTCTTTCTCT CTCTAAAGAA TAGTTGAGTT TTTGCTCAAG AAGCAAATTT   
  
  
+ GTCCCACAAC AACAATAGAT TTCCACTTAC TTATAACAAT TATATGATCT TTGAAGGGAA CGGAGAAGGT   
  
  
+ ACGTACGTAC TATATATTAC TTCCCAGTAC TATATTTTTC CCGTCAATTT TTTTGCGTAC ATATAAAGTG   
  
  
+ TGTTATTGAG ATTTGATGAT TTTGTAATGA TTTTATAGGA GGAGCTTACC GTCTACAATT TAAGAAGAAA   
  
  
+ CAATGATTAC GTTAATTGGA ATGTTTAGGC TAATAGTACA TGATTATTGT ACATACGCAC ATGTTGTATA   
  
  
+ GATAAATATT TATATTTCTC CAAGACAAAA ACCCTAAAAT TTATCTTTTT CCACCAACTA CACTCTTGAG   
  
  
+ TTTCACATTT TAACTCTAAT GTTGTTGTCG TAAATTTAGA CTTGTATTTT ATATTCTTTT GTTACATATA   
  
  
+ CATTGTATCA TACTCATGCA TGCATATATA GTAAACTTAT TAAACTCAAT TCTTCATGTA TTTTACGTAA   
  
  
+ ACCAATCGAG TTATTTTGTA TGAGTAATAT ATTTTGCCCT TATGGTATCA TTACGACCAT CAACTAGCTA   
  
  
+ TGTGATTGAA ATGAGAACCA TGAAGAACTA ATTTCGGATT AATGCTAGGA AGAACATATG TAGTAAGTTG   
  
  
+ AGTGATGTGT ACTAATTGTA TGCATCATTT TGTAACATCA CGTGATCCAA AAAAATGTGT TATACGGTTA   
  
  
+ CTCCAAGCAA TGCTCATAAT ATGAATATGT ATACATAAAT GGTTAAAATG CGTATTATCC TAATAATTGA   
  
  
+ CTATAAAATA AAATATGAGA ATACTATATA TATGTATGTG TGTGTGTGTG TGTATGTGTA GGGTTAGTGA   
  
  
+ TGCTTCATGG CATGTGTAGG ATTAGTGATG CTTTATGGCC ACAAACTAGG GCAAGGATTT GGTCTACATA   
  
  
+ ATCACCTATT CAAAAGGAAA TATTGTTCTA TATAATTTTC AGACAGTTTT ATATTCTCTT TATTTATGTA   
  
  
+ TTATTGATGA ATAGTTATGC ACAGGGCATA TCCGGCCGGT GGTTCTTTCA CTATTTTTAT GCATAACAAT   
  
  
+ TGGCCATCAA TGGCTTCTCT TATTTTATAC ATAACAATTG GACATATTGG AAGTGGTCAT CACTATTGTT   
  
  
+ GAGCAGTCAG TCTCAGACTC GATTTTTTAT ACAAACGTAA CCACATAGAG ATTACCCAGC ATAGCTTAAT   
  
  
+ TACCTGCTGG AACCAAACAT GCATGCATAC CTCTTTTTAT TTACGTGTTG TCATTGTCTT TGACTGCTTA   
  
  
+ AGATTCTAAA CAAGTTTAAT AGGTGTTTG  

- ACAAATTAAT AATATTAAGA ACTCCAAAAC ACAAACTTTA CGACTCCTAA AAAGATCAAG ATCTCTCTCT   
  
  
- CTGTCTCTGT CTCTCTTTGT TCTGTACGTG GAGAGTTGGT TTAGCAGGGG GTGTTAAAGT TGCGTTCAGC   
  
  
- ACGATGGAGA GAGAGATTGA GAGAAAGAGA GAGATTTCTT ATCAACTCAA AAACGAGTTC TTCGTTTAAA   
  
  
- CAGGGTGTTG TTGTTATCTA AAGGTGAATG AATATTGTTA ATATACTAGA AACTTCCCTT GCCTCTTCCA   
  
  
- TGCATGCATG ATATATAATG AAGGGTCATG ATATAAAAAG GGCAGTTAAA AAAACGCATG TATATTTCAC   
  
  
- ACAATAACTC TAAACTACTA AAACATTACT AAAATATCCT CCTCGAATGG CAGATGTTAA ATTCTTCTTT   
  
  
- GTTACTAATG CAATTAACCT TACAAATCCG ATTATCATGT ACTAATAACA TGTATGCGTG TACAACATAT   
  
  
- CTATTTATAA ATATAAAGAG GTTCTGTTTT TGGGATTTTA AATAGAAAAA GGTGGTTGAT GTGAGAACTC   
  
  
- AAAGTGTAAA ATTGAGATTA CAACAACAGC ATTTAAATCT GAACATAAAA TATAAGAAAA CAATGTATAT   
  
  
- GTAACATAGT ATGAGTACGT ACGTATATAT CATTTGAATA ATTTGAGTTA AGAAGTACAT AAAATGCATT   
  
  
- TGGTTAGCTC AATAAAACAT ACTCATTATA TAAAACGGGA ATACCATAGT AATGCTGGTA GTTGATCGAT   
  
  
- ACACTAACTT TACTCTTGGT ACTTCTTGAT TAAAGCCTAA TTACGATCCT TCTTGTATAC ATCATTCAAC   
  
  
- TCACTACACA TGATTAACAT ACGTAGTAAA ACATTGTAGT GCACTAGGTT TTTTTACACA ATATGCCAAT   
  
  
- GAGGTTCGTT ACGAGTATTA TACTTATACA TATGTATTTA CCAATTTTAC GCATAATAGG ATTATTAACT   
  
  
- GATATTTTAT TTTATACTCT TATGATATAT ATACATACAC ACACACACAC ACATACACAT CCCAATCACT   
  
  
- ACGAAGTACC GTACACATCC TAATCACTAC GAAATACCGG TGTTTGATCC CGTTCCTAAA CCAGATGTAT   
  
  
- TAGTGGATAA GTTTTCCTTT ATAACAAGAT ATATTAAAAG TCTGTCAAAA TATAAGAGAA ATAAATACAT   
  
  
- AATAACTACT TATCAATACG TGTCCCGTAT AGGCCGGCCA CCAAGAAAGT GATAAAAATA CGTATTGTTA   
  
  
- ACCGGTAGTT ACCGAAGAGA ATAAAATATG TATTGTTAAC CTGTATAACC TTCACCAGTA GTGATAACAA   
  
  
- CTCGTCAGTC AGAGTCTGAG CTAAAAAATA TGTTTGCATT GGTGTATCTC TAATGGGTCG TATCGAATTA   
  
  
- ATGGACGACC TTGGTTTGTA CGTACGTATG GAGAAAAATA AATGCACAAC AGTAACAGAA ACTGACGAAT   
  
  
- TCTAAGATTT GTTCAAATTA TCCACAAAC

+     G-Box

| Site Name | Organism | Position | Strand | Matrix score. | sequence | function |
| --- | --- | --- | --- | --- | --- | --- |
| G-Box | Pisum sativum | 879 | - | 6 | CACGTG | cis-acting regulatory element involved in light responsiveness |

>037399   
+ TGTTTAATTA TTATAATTCT TGAGGTTTTG TGTTTGAAAT GCTGAGGATT TTTCTAGTTC TAGAGAGAGA   
  
  
+ GACAGAGACA GAGAGAAACA AGACATGCAC CTCTCAACCA AATCGTCCCC CACAATTTCA ACGCAAGTCG   
  
  
+ TGCTACCTCT CTCTCTAACT CTCTTTCTCT CTCTAAAGAA TAGTTGAGTT TTTGCTCAAG AAGCAAATTT   
  
  
+ GTCCCACAAC AACAATAGAT TTCCACTTAC TTATAACAAT TATATGATCT TTGAAGGGAA CGGAGAAGGT   
  
  
+ ACGTACGTAC TATATATTAC TTCCCAGTAC TATATTTTTC CCGTCAATTT TTTTGCGTAC ATATAAAGTG   
  
  
+ TGTTATTGAG ATTTGATGAT TTTGTAATGA TTTTATAGGA GGAGCTTACC GTCTACAATT TAAGAAGAAA   
  
  
+ CAATGATTAC GTTAATTGGA ATGTTTAGGC TAATAGTACA TGATTATTGT ACATACGCAC ATGTTGTATA   
  
  
+ GATAAATATT TATATTTCTC CAAGACAAAA ACCCTAAAAT TTATCTTTTT CCACCAACTA CACTCTTGAG   
  
  
+ TTTCACATTT TAACTCTAAT GTTGTTGTCG TAAATTTAGA CTTGTATTTT ATATTCTTTT GTTACATATA   
  
  
+ CATTGTATCA TACTCATGCA TGCATATATA GTAAACTTAT TAAACTCAAT TCTTCATGTA TTTTACGTAA   
  
  
+ ACCAATCGAG TTATTTTGTA TGAGTAATAT ATTTTGCCCT TATGGTATCA TTACGACCAT CAACTAGCTA   
  
  
+ TGTGATTGAA ATGAGAACCA TGAAGAACTA ATTTCGGATT AATGCTAGGA AGAACATATG TAGTAAGTTG   
  
  
+ AGTGATGTGT ACTAATTGTA TGCATCATTT TGTAACATCA CGTGATCCAA AAAAATGTGT TATACGGTTA   
  
  
+ CTCCAAGCAA TGCTCATAAT ATGAATATGT ATACATAAAT GGTTAAAATG CGTATTATCC TAATAATTGA   
  
  
+ CTATAAAATA AAATATGAGA ATACTATATA TATGTATGTG TGTGTGTGTG TGTATGTGTA GGGTTAGTGA   
  
  
+ TGCTTCATGG CATGTGTAGG ATTAGTGATG CTTTATGGCC ACAAACTAGG GCAAGGATTT GGTCTACATA   
  
  
+ ATCACCTATT CAAAAGGAAA TATTGTTCTA TATAATTTTC AGACAGTTTT ATATTCTCTT TATTTATGTA   
  
  
+ TTATTGATGA ATAGTTATGC ACAGGGCATA TCCGGCCGGT GGTTCTTTCA CTATTTTTAT GCATAACAAT   
  
  
+ TGGCCATCAA TGGCTTCTCT TATTTTATAC ATAACAATTG GACATATTGG AAGTGGTCAT CACTATTGTT   
  
  
+ GAGCAGTCAG TCTCAGACTC GATTTTTTAT ACAAACGTAA CCACATAGAG ATTACCCAGC ATAGCTTAAT   
  
  
+ TACCTGCTGG AACCAAACAT GCATGCATAC CTCTTTTTAT TTACGTGTTG TCATTGTCTT TGACTGCTTA   
  
  
+ AGATTCTAAA CAAGTTTAAT AGGTGTTTG  

- ACAAATTAAT AATATTAAGA ACTCCAAAAC ACAAACTTTA CGACTCCTAA AAAGATCAAG ATCTCTCTCT   
  
  
- CTGTCTCTGT CTCTCTTTGT TCTGTACGTG GAGAGTTGGT TTAGCAGGGG GTGTTAAAGT TGCGTTCAGC   
  
  
- ACGATGGAGA GAGAGATTGA GAGAAAGAGA GAGATTTCTT ATCAACTCAA AAACGAGTTC TTCGTTTAAA   
  
  
- CAGGGTGTTG TTGTTATCTA AAGGTGAATG AATATTGTTA ATATACTAGA AACTTCCCTT GCCTCTTCCA   
  
  
- TGCATGCATG ATATATAATG AAGGGTCATG ATATAAAAAG GGCAGTTAAA AAAACGCATG TATATTTCAC   
  
  
- ACAATAACTC TAAACTACTA AAACATTACT AAAATATCCT CCTCGAATGG CAGATGTTAA ATTCTTCTTT   
  
  
- GTTACTAATG CAATTAACCT TACAAATCCG ATTATCATGT ACTAATAACA TGTATGCGTG TACAACATAT   
  
  
- CTATTTATAA ATATAAAGAG GTTCTGTTTT TGGGATTTTA AATAGAAAAA GGTGGTTGAT GTGAGAACTC   
  
  
- AAAGTGTAAA ATTGAGATTA CAACAACAGC ATTTAAATCT GAACATAAAA TATAAGAAAA CAATGTATAT   
  
  
- GTAACATAGT ATGAGTACGT ACGTATATAT CATTTGAATA ATTTGAGTTA AGAAGTACAT AAAATGCATT   
  
  
- TGGTTAGCTC AATAAAACAT ACTCATTATA TAAAACGGGA ATACCATAGT AATGCTGGTA GTTGATCGAT   
  
  
- ACACTAACTT TACTCTTGGT ACTTCTTGAT TAAAGCCTAA TTACGATCCT TCTTGTATAC ATCATTCAAC   
  
  
- TCACTACACA TGATTAACAT ACGTAGTAAA ACATTGTAGT GCACTAGGTT TTTTTACACA ATATGCCAAT   
  
  
- GAGGTTCGTT ACGAGTATTA TACTTATACA TATGTATTTA CCAATTTTAC GCATAATAGG ATTATTAACT   
  
  
- GATATTTTAT TTTATACTCT TATGATATAT ATACATACAC ACACACACAC ACATACACAT CCCAATCACT   
  
  
- ACGAAGTACC GTACACATCC TAATCACTAC GAAATACCGG TGTTTGATCC CGTTCCTAAA CCAGATGTAT   
  
  
- TAGTGGATAA GTTTTCCTTT ATAACAAGAT ATATTAAAAG TCTGTCAAAA TATAAGAGAA ATAAATACAT   
  
  
- AATAACTACT TATCAATACG TGTCCCGTAT AGGCCGGCCA CCAAGAAAGT GATAAAAATA CGTATTGTTA   
  
  
- ACCGGTAGTT ACCGAAGAGA ATAAAATATG TATTGTTAAC CTGTATAACC TTCACCAGTA GTGATAACAA   
  
  
- CTCGTCAGTC AGAGTCTGAG CTAAAAAATA TGTTTGCATT GGTGTATCTC TAATGGGTCG TATCGAATTA   
  
  
- ATGGACGACC TTGGTTTGTA CGTACGTATG GAGAAAAATA AATGCACAAC AGTAACAGAA ACTGACGAAT   
  
  
- TCTAAGATTT GTTCAAATTA TCCACAAAC

+     G-box

| Site Name | Organism | Position | Strand | Matrix score. | sequence | function |
| --- | --- | --- | --- | --- | --- | --- |
| G-box | Arabidopsis thaliana | 1442 | + | 6 | TACGTG | cis-acting regulatory element involved in light responsiveness |
| G-box | Arabidopsis thaliana | 879 | - | 6 | CACGTG | cis-acting regulatory element involved in light responsiveness |
| G-box | Zea mays | 137 | - | 6 | CACGAC | cis-acting regulatory element involved in light responsiveness |

>037399   
+ TGTTTAATTA TTATAATTCT TGAGGTTTTG TGTTTGAAAT GCTGAGGATT TTTCTAGTTC TAGAGAGAGA   
  
  
+ GACAGAGACA GAGAGAAACA AGACATGCAC CTCTCAACCA AATCGTCCCC CACAATTTCA ACGCAAGTCG   
  
  
+ TGCTACCTCT CTCTCTAACT CTCTTTCTCT CTCTAAAGAA TAGTTGAGTT TTTGCTCAAG AAGCAAATTT   
  
  
+ GTCCCACAAC AACAATAGAT TTCCACTTAC TTATAACAAT TATATGATCT TTGAAGGGAA CGGAGAAGGT   
  
  
+ ACGTACGTAC TATATATTAC TTCCCAGTAC TATATTTTTC CCGTCAATTT TTTTGCGTAC ATATAAAGTG   
  
  
+ TGTTATTGAG ATTTGATGAT TTTGTAATGA TTTTATAGGA GGAGCTTACC GTCTACAATT TAAGAAGAAA   
  
  
+ CAATGATTAC GTTAATTGGA ATGTTTAGGC TAATAGTACA TGATTATTGT ACATACGCAC ATGTTGTATA   
  
  
+ GATAAATATT TATATTTCTC CAAGACAAAA ACCCTAAAAT TTATCTTTTT CCACCAACTA CACTCTTGAG   
  
  
+ TTTCACATTT TAACTCTAAT GTTGTTGTCG TAAATTTAGA CTTGTATTTT ATATTCTTTT GTTACATATA   
  
  
+ CATTGTATCA TACTCATGCA TGCATATATA GTAAACTTAT TAAACTCAAT TCTTCATGTA TTTTACGTAA   
  
  
+ ACCAATCGAG TTATTTTGTA TGAGTAATAT ATTTTGCCCT TATGGTATCA TTACGACCAT CAACTAGCTA   
  
  
+ TGTGATTGAA ATGAGAACCA TGAAGAACTA ATTTCGGATT AATGCTAGGA AGAACATATG TAGTAAGTTG   
  
  
+ AGTGATGTGT ACTAATTGTA TGCATCATTT TGTAACATCA CGTGATCCAA AAAAATGTGT TATACGGTTA   
  
  
+ CTCCAAGCAA TGCTCATAAT ATGAATATGT ATACATAAAT GGTTAAAATG CGTATTATCC TAATAATTGA   
  
  
+ CTATAAAATA AAATATGAGA ATACTATATA TATGTATGTG TGTGTGTGTG TGTATGTGTA GGGTTAGTGA   
  
  
+ TGCTTCATGG CATGTGTAGG ATTAGTGATG CTTTATGGCC ACAAACTAGG GCAAGGATTT GGTCTACATA   
  
  
+ ATCACCTATT CAAAAGGAAA TATTGTTCTA TATAATTTTC AGACAGTTTT ATATTCTCTT TATTTATGTA   
  
  
+ TTATTGATGA ATAGTTATGC ACAGGGCATA TCCGGCCGGT GGTTCTTTCA CTATTTTTAT GCATAACAAT   
  
  
+ TGGCCATCAA TGGCTTCTCT TATTTTATAC ATAACAATTG GACATATTGG AAGTGGTCAT CACTATTGTT   
  
  
+ GAGCAGTCAG TCTCAGACTC GATTTTTTAT ACAAACGTAA CCACATAGAG ATTACCCAGC ATAGCTTAAT   
  
  
+ TACCTGCTGG AACCAAACAT GCATGCATAC CTCTTTTTAT TTACGTGTTG TCATTGTCTT TGACTGCTTA   
  
  
+ AGATTCTAAA CAAGTTTAAT AGGTGTTTG  

- ACAAATTAAT AATATTAAGA ACTCCAAAAC ACAAACTTTA CGACTCCTAA AAAGATCAAG ATCTCTCTCT   
  
  
- CTGTCTCTGT CTCTCTTTGT TCTGTACGTG GAGAGTTGGT TTAGCAGGGG GTGTTAAAGT TGCGTTCAGC   
  
  
- ACGATGGAGA GAGAGATTGA GAGAAAGAGA GAGATTTCTT ATCAACTCAA AAACGAGTTC TTCGTTTAAA   
  
  
- CAGGGTGTTG TTGTTATCTA AAGGTGAATG AATATTGTTA ATATACTAGA AACTTCCCTT GCCTCTTCCA   
  
  
- TGCATGCATG ATATATAATG AAGGGTCATG ATATAAAAAG GGCAGTTAAA AAAACGCATG TATATTTCAC   
  
  
- ACAATAACTC TAAACTACTA AAACATTACT AAAATATCCT CCTCGAATGG CAGATGTTAA ATTCTTCTTT   
  
  
- GTTACTAATG CAATTAACCT TACAAATCCG ATTATCATGT ACTAATAACA TGTATGCGTG TACAACATAT   
  
  
- CTATTTATAA ATATAAAGAG GTTCTGTTTT TGGGATTTTA AATAGAAAAA GGTGGTTGAT GTGAGAACTC   
  
  
- AAAGTGTAAA ATTGAGATTA CAACAACAGC ATTTAAATCT GAACATAAAA TATAAGAAAA CAATGTATAT   
  
  
- GTAACATAGT ATGAGTACGT ACGTATATAT CATTTGAATA ATTTGAGTTA AGAAGTACAT AAAATGCATT   
  
  
- TGGTTAGCTC AATAAAACAT ACTCATTATA TAAAACGGGA ATACCATAGT AATGCTGGTA GTTGATCGAT   
  
  
- ACACTAACTT TACTCTTGGT ACTTCTTGAT TAAAGCCTAA TTACGATCCT TCTTGTATAC ATCATTCAAC   
  
  
- TCACTACACA TGATTAACAT ACGTAGTAAA ACATTGTAGT GCACTAGGTT TTTTTACACA ATATGCCAAT   
  
  
- GAGGTTCGTT ACGAGTATTA TACTTATACA TATGTATTTA CCAATTTTAC GCATAATAGG ATTATTAACT   
  
  
- GATATTTTAT TTTATACTCT TATGATATAT ATACATACAC ACACACACAC ACATACACAT CCCAATCACT   
  
  
- ACGAAGTACC GTACACATCC TAATCACTAC GAAATACCGG TGTTTGATCC CGTTCCTAAA CCAGATGTAT   
  
  
- TAGTGGATAA GTTTTCCTTT ATAACAAGAT ATATTAAAAG TCTGTCAAAA TATAAGAGAA ATAAATACAT   
  
  
- AATAACTACT TATCAATACG TGTCCCGTAT AGGCCGGCCA CCAAGAAAGT GATAAAAATA CGTATTGTTA   
  
  
- ACCGGTAGTT ACCGAAGAGA ATAAAATATG TATTGTTAAC CTGTATAACC TTCACCAGTA GTGATAACAA   
  
  
- CTCGTCAGTC AGAGTCTGAG CTAAAAAATA TGTTTGCATT GGTGTATCTC TAATGGGTCG TATCGAATTA   
  
  
- ATGGACGACC TTGGTTTGTA CGTACGTATG GAGAAAAATA AATGCACAAC AGTAACAGAA ACTGACGAAT   
  
  
- TCTAAGATTT GTTCAAATTA TCCACAAAC

+     GA-motif

| Site Name | Organism | Position | Strand | Matrix score. | sequence | function |
| --- | --- | --- | --- | --- | --- | --- |
| GA-motif | Arabidopsis thaliana | 488 | + | 8 | ATAGATAA | part of a light responsive element |

>037399   
+ TGTTTAATTA TTATAATTCT TGAGGTTTTG TGTTTGAAAT GCTGAGGATT TTTCTAGTTC TAGAGAGAGA   
  
  
+ GACAGAGACA GAGAGAAACA AGACATGCAC CTCTCAACCA AATCGTCCCC CACAATTTCA ACGCAAGTCG   
  
  
+ TGCTACCTCT CTCTCTAACT CTCTTTCTCT CTCTAAAGAA TAGTTGAGTT TTTGCTCAAG AAGCAAATTT   
  
  
+ GTCCCACAAC AACAATAGAT TTCCACTTAC TTATAACAAT TATATGATCT TTGAAGGGAA CGGAGAAGGT   
  
  
+ ACGTACGTAC TATATATTAC TTCCCAGTAC TATATTTTTC CCGTCAATTT TTTTGCGTAC ATATAAAGTG   
  
  
+ TGTTATTGAG ATTTGATGAT TTTGTAATGA TTTTATAGGA GGAGCTTACC GTCTACAATT TAAGAAGAAA   
  
  
+ CAATGATTAC GTTAATTGGA ATGTTTAGGC TAATAGTACA TGATTATTGT ACATACGCAC ATGTTGTATA   
  
  
+ GATAAATATT TATATTTCTC CAAGACAAAA ACCCTAAAAT TTATCTTTTT CCACCAACTA CACTCTTGAG   
  
  
+ TTTCACATTT TAACTCTAAT GTTGTTGTCG TAAATTTAGA CTTGTATTTT ATATTCTTTT GTTACATATA   
  
  
+ CATTGTATCA TACTCATGCA TGCATATATA GTAAACTTAT TAAACTCAAT TCTTCATGTA TTTTACGTAA   
  
  
+ ACCAATCGAG TTATTTTGTA TGAGTAATAT ATTTTGCCCT TATGGTATCA TTACGACCAT CAACTAGCTA   
  
  
+ TGTGATTGAA ATGAGAACCA TGAAGAACTA ATTTCGGATT AATGCTAGGA AGAACATATG TAGTAAGTTG   
  
  
+ AGTGATGTGT ACTAATTGTA TGCATCATTT TGTAACATCA CGTGATCCAA AAAAATGTGT TATACGGTTA   
  
  
+ CTCCAAGCAA TGCTCATAAT ATGAATATGT ATACATAAAT GGTTAAAATG CGTATTATCC TAATAATTGA   
  
  
+ CTATAAAATA AAATATGAGA ATACTATATA TATGTATGTG TGTGTGTGTG TGTATGTGTA GGGTTAGTGA   
  
  
+ TGCTTCATGG CATGTGTAGG ATTAGTGATG CTTTATGGCC ACAAACTAGG GCAAGGATTT GGTCTACATA   
  
  
+ ATCACCTATT CAAAAGGAAA TATTGTTCTA TATAATTTTC AGACAGTTTT ATATTCTCTT TATTTATGTA   
  
  
+ TTATTGATGA ATAGTTATGC ACAGGGCATA TCCGGCCGGT GGTTCTTTCA CTATTTTTAT GCATAACAAT   
  
  
+ TGGCCATCAA TGGCTTCTCT TATTTTATAC ATAACAATTG GACATATTGG AAGTGGTCAT CACTATTGTT   
  
  
+ GAGCAGTCAG TCTCAGACTC GATTTTTTAT ACAAACGTAA CCACATAGAG ATTACCCAGC ATAGCTTAAT   
  
  
+ TACCTGCTGG AACCAAACAT GCATGCATAC CTCTTTTTAT TTACGTGTTG TCATTGTCTT TGACTGCTTA   
  
  
+ AGATTCTAAA CAAGTTTAAT AGGTGTTTG  

- ACAAATTAAT AATATTAAGA ACTCCAAAAC ACAAACTTTA CGACTCCTAA AAAGATCAAG ATCTCTCTCT   
  
  
- CTGTCTCTGT CTCTCTTTGT TCTGTACGTG GAGAGTTGGT TTAGCAGGGG GTGTTAAAGT TGCGTTCAGC   
  
  
- ACGATGGAGA GAGAGATTGA GAGAAAGAGA GAGATTTCTT ATCAACTCAA AAACGAGTTC TTCGTTTAAA   
  
  
- CAGGGTGTTG TTGTTATCTA AAGGTGAATG AATATTGTTA ATATACTAGA AACTTCCCTT GCCTCTTCCA   
  
  
- TGCATGCATG ATATATAATG AAGGGTCATG ATATAAAAAG GGCAGTTAAA AAAACGCATG TATATTTCAC   
  
  
- ACAATAACTC TAAACTACTA AAACATTACT AAAATATCCT CCTCGAATGG CAGATGTTAA ATTCTTCTTT   
  
  
- GTTACTAATG CAATTAACCT TACAAATCCG ATTATCATGT ACTAATAACA TGTATGCGTG TACAACATAT   
  
  
- CTATTTATAA ATATAAAGAG GTTCTGTTTT TGGGATTTTA AATAGAAAAA GGTGGTTGAT GTGAGAACTC   
  
  
- AAAGTGTAAA ATTGAGATTA CAACAACAGC ATTTAAATCT GAACATAAAA TATAAGAAAA CAATGTATAT   
  
  
- GTAACATAGT ATGAGTACGT ACGTATATAT CATTTGAATA ATTTGAGTTA AGAAGTACAT AAAATGCATT   
  
  
- TGGTTAGCTC AATAAAACAT ACTCATTATA TAAAACGGGA ATACCATAGT AATGCTGGTA GTTGATCGAT   
  
  
- ACACTAACTT TACTCTTGGT ACTTCTTGAT TAAAGCCTAA TTACGATCCT TCTTGTATAC ATCATTCAAC   
  
  
- TCACTACACA TGATTAACAT ACGTAGTAAA ACATTGTAGT GCACTAGGTT TTTTTACACA ATATGCCAAT   
  
  
- GAGGTTCGTT ACGAGTATTA TACTTATACA TATGTATTTA CCAATTTTAC GCATAATAGG ATTATTAACT   
  
  
- GATATTTTAT TTTATACTCT TATGATATAT ATACATACAC ACACACACAC ACATACACAT CCCAATCACT   
  
  
- ACGAAGTACC GTACACATCC TAATCACTAC GAAATACCGG TGTTTGATCC CGTTCCTAAA CCAGATGTAT   
  
  
- TAGTGGATAA GTTTTCCTTT ATAACAAGAT ATATTAAAAG TCTGTCAAAA TATAAGAGAA ATAAATACAT   
  
  
- AATAACTACT TATCAATACG TGTCCCGTAT AGGCCGGCCA CCAAGAAAGT GATAAAAATA CGTATTGTTA   
  
  
- ACCGGTAGTT ACCGAAGAGA ATAAAATATG TATTGTTAAC CTGTATAACC TTCACCAGTA GTGATAACAA   
  
  
- CTCGTCAGTC AGAGTCTGAG CTAAAAAATA TGTTTGCATT GGTGTATCTC TAATGGGTCG TATCGAATTA   
  
  
- ATGGACGACC TTGGTTTGTA CGTACGTATG GAGAAAAATA AATGCACAAC AGTAACAGAA ACTGACGAAT   
  
  
- TCTAAGATTT GTTCAAATTA TCCACAAAC

+     GT1-motif

| Site Name | Organism | Position | Strand | Matrix score. | sequence | function |
| --- | --- | --- | --- | --- | --- | --- |
| GT1-motif | Arabidopsis thaliana | 951 | + | 6 | GGTTAA | light responsive element |

>037399   
+ TGTTTAATTA TTATAATTCT TGAGGTTTTG TGTTTGAAAT GCTGAGGATT TTTCTAGTTC TAGAGAGAGA   
  
  
+ GACAGAGACA GAGAGAAACA AGACATGCAC CTCTCAACCA AATCGTCCCC CACAATTTCA ACGCAAGTCG   
  
  
+ TGCTACCTCT CTCTCTAACT CTCTTTCTCT CTCTAAAGAA TAGTTGAGTT TTTGCTCAAG AAGCAAATTT   
  
  
+ GTCCCACAAC AACAATAGAT TTCCACTTAC TTATAACAAT TATATGATCT TTGAAGGGAA CGGAGAAGGT   
  
  
+ ACGTACGTAC TATATATTAC TTCCCAGTAC TATATTTTTC CCGTCAATTT TTTTGCGTAC ATATAAAGTG   
  
  
+ TGTTATTGAG ATTTGATGAT TTTGTAATGA TTTTATAGGA GGAGCTTACC GTCTACAATT TAAGAAGAAA   
  
  
+ CAATGATTAC GTTAATTGGA ATGTTTAGGC TAATAGTACA TGATTATTGT ACATACGCAC ATGTTGTATA   
  
  
+ GATAAATATT TATATTTCTC CAAGACAAAA ACCCTAAAAT TTATCTTTTT CCACCAACTA CACTCTTGAG   
  
  
+ TTTCACATTT TAACTCTAAT GTTGTTGTCG TAAATTTAGA CTTGTATTTT ATATTCTTTT GTTACATATA   
  
  
+ CATTGTATCA TACTCATGCA TGCATATATA GTAAACTTAT TAAACTCAAT TCTTCATGTA TTTTACGTAA   
  
  
+ ACCAATCGAG TTATTTTGTA TGAGTAATAT ATTTTGCCCT TATGGTATCA TTACGACCAT CAACTAGCTA   
  
  
+ TGTGATTGAA ATGAGAACCA TGAAGAACTA ATTTCGGATT AATGCTAGGA AGAACATATG TAGTAAGTTG   
  
  
+ AGTGATGTGT ACTAATTGTA TGCATCATTT TGTAACATCA CGTGATCCAA AAAAATGTGT TATACGGTTA   
  
  
+ CTCCAAGCAA TGCTCATAAT ATGAATATGT ATACATAAAT GGTTAAAATG CGTATTATCC TAATAATTGA   
  
  
+ CTATAAAATA AAATATGAGA ATACTATATA TATGTATGTG TGTGTGTGTG TGTATGTGTA GGGTTAGTGA   
  
  
+ TGCTTCATGG CATGTGTAGG ATTAGTGATG CTTTATGGCC ACAAACTAGG GCAAGGATTT GGTCTACATA   
  
  
+ ATCACCTATT CAAAAGGAAA TATTGTTCTA TATAATTTTC AGACAGTTTT ATATTCTCTT TATTTATGTA   
  
  
+ TTATTGATGA ATAGTTATGC ACAGGGCATA TCCGGCCGGT GGTTCTTTCA CTATTTTTAT GCATAACAAT   
  
  
+ TGGCCATCAA TGGCTTCTCT TATTTTATAC ATAACAATTG GACATATTGG AAGTGGTCAT CACTATTGTT   
  
  
+ GAGCAGTCAG TCTCAGACTC GATTTTTTAT ACAAACGTAA CCACATAGAG ATTACCCAGC ATAGCTTAAT   
  
  
+ TACCTGCTGG AACCAAACAT GCATGCATAC CTCTTTTTAT TTACGTGTTG TCATTGTCTT TGACTGCTTA   
  
  
+ AGATTCTAAA CAAGTTTAAT AGGTGTTTG  

- ACAAATTAAT AATATTAAGA ACTCCAAAAC ACAAACTTTA CGACTCCTAA AAAGATCAAG ATCTCTCTCT   
  
  
- CTGTCTCTGT CTCTCTTTGT TCTGTACGTG GAGAGTTGGT TTAGCAGGGG GTGTTAAAGT TGCGTTCAGC   
  
  
- ACGATGGAGA GAGAGATTGA GAGAAAGAGA GAGATTTCTT ATCAACTCAA AAACGAGTTC TTCGTTTAAA   
  
  
- CAGGGTGTTG TTGTTATCTA AAGGTGAATG AATATTGTTA ATATACTAGA AACTTCCCTT GCCTCTTCCA   
  
  
- TGCATGCATG ATATATAATG AAGGGTCATG ATATAAAAAG GGCAGTTAAA AAAACGCATG TATATTTCAC   
  
  
- ACAATAACTC TAAACTACTA AAACATTACT AAAATATCCT CCTCGAATGG CAGATGTTAA ATTCTTCTTT   
  
  
- GTTACTAATG CAATTAACCT TACAAATCCG ATTATCATGT ACTAATAACA TGTATGCGTG TACAACATAT   
  
  
- CTATTTATAA ATATAAAGAG GTTCTGTTTT TGGGATTTTA AATAGAAAAA GGTGGTTGAT GTGAGAACTC   
  
  
- AAAGTGTAAA ATTGAGATTA CAACAACAGC ATTTAAATCT GAACATAAAA TATAAGAAAA CAATGTATAT   
  
  
- GTAACATAGT ATGAGTACGT ACGTATATAT CATTTGAATA ATTTGAGTTA AGAAGTACAT AAAATGCATT   
  
  
- TGGTTAGCTC AATAAAACAT ACTCATTATA TAAAACGGGA ATACCATAGT AATGCTGGTA GTTGATCGAT   
  
  
- ACACTAACTT TACTCTTGGT ACTTCTTGAT TAAAGCCTAA TTACGATCCT TCTTGTATAC ATCATTCAAC   
  
  
- TCACTACACA TGATTAACAT ACGTAGTAAA ACATTGTAGT GCACTAGGTT TTTTTACACA ATATGCCAAT   
  
  
- GAGGTTCGTT ACGAGTATTA TACTTATACA TATGTATTTA CCAATTTTAC GCATAATAGG ATTATTAACT   
  
  
- GATATTTTAT TTTATACTCT TATGATATAT ATACATACAC ACACACACAC ACATACACAT CCCAATCACT   
  
  
- ACGAAGTACC GTACACATCC TAATCACTAC GAAATACCGG TGTTTGATCC CGTTCCTAAA CCAGATGTAT   
  
  
- TAGTGGATAA GTTTTCCTTT ATAACAAGAT ATATTAAAAG TCTGTCAAAA TATAAGAGAA ATAAATACAT   
  
  
- AATAACTACT TATCAATACG TGTCCCGTAT AGGCCGGCCA CCAAGAAAGT GATAAAAATA CGTATTGTTA   
  
  
- ACCGGTAGTT ACCGAAGAGA ATAAAATATG TATTGTTAAC CTGTATAACC TTCACCAGTA GTGATAACAA   
  
  
- CTCGTCAGTC AGAGTCTGAG CTAAAAAATA TGTTTGCATT GGTGTATCTC TAATGGGTCG TATCGAATTA   
  
  
- ATGGACGACC TTGGTTTGTA CGTACGTATG GAGAAAAATA AATGCACAAC AGTAACAGAA ACTGACGAAT   
  
  
- TCTAAGATTT GTTCAAATTA TCCACAAAC

+     LTR

| Site Name | Organism | Position | Strand | Matrix score. | sequence | function |
| --- | --- | --- | --- | --- | --- | --- |
| LTR | Hordeum vulgare | 802 | - | 6 | CCGAAA | cis-acting element involved in low-temperature responsiveness |

>037399   
+ TGTTTAATTA TTATAATTCT TGAGGTTTTG TGTTTGAAAT GCTGAGGATT TTTCTAGTTC TAGAGAGAGA   
  
  
+ GACAGAGACA GAGAGAAACA AGACATGCAC CTCTCAACCA AATCGTCCCC CACAATTTCA ACGCAAGTCG   
  
  
+ TGCTACCTCT CTCTCTAACT CTCTTTCTCT CTCTAAAGAA TAGTTGAGTT TTTGCTCAAG AAGCAAATTT   
  
  
+ GTCCCACAAC AACAATAGAT TTCCACTTAC TTATAACAAT TATATGATCT TTGAAGGGAA CGGAGAAGGT   
  
  
+ ACGTACGTAC TATATATTAC TTCCCAGTAC TATATTTTTC CCGTCAATTT TTTTGCGTAC ATATAAAGTG   
  
  
+ TGTTATTGAG ATTTGATGAT TTTGTAATGA TTTTATAGGA GGAGCTTACC GTCTACAATT TAAGAAGAAA   
  
  
+ CAATGATTAC GTTAATTGGA ATGTTTAGGC TAATAGTACA TGATTATTGT ACATACGCAC ATGTTGTATA   
  
  
+ GATAAATATT TATATTTCTC CAAGACAAAA ACCCTAAAAT TTATCTTTTT CCACCAACTA CACTCTTGAG   
  
  
+ TTTCACATTT TAACTCTAAT GTTGTTGTCG TAAATTTAGA CTTGTATTTT ATATTCTTTT GTTACATATA   
  
  
+ CATTGTATCA TACTCATGCA TGCATATATA GTAAACTTAT TAAACTCAAT TCTTCATGTA TTTTACGTAA   
  
  
+ ACCAATCGAG TTATTTTGTA TGAGTAATAT ATTTTGCCCT TATGGTATCA TTACGACCAT CAACTAGCTA   
  
  
+ TGTGATTGAA ATGAGAACCA TGAAGAACTA ATTTCGGATT AATGCTAGGA AGAACATATG TAGTAAGTTG   
  
  
+ AGTGATGTGT ACTAATTGTA TGCATCATTT TGTAACATCA CGTGATCCAA AAAAATGTGT TATACGGTTA   
  
  
+ CTCCAAGCAA TGCTCATAAT ATGAATATGT ATACATAAAT GGTTAAAATG CGTATTATCC TAATAATTGA   
  
  
+ CTATAAAATA AAATATGAGA ATACTATATA TATGTATGTG TGTGTGTGTG TGTATGTGTA GGGTTAGTGA   
  
  
+ TGCTTCATGG CATGTGTAGG ATTAGTGATG CTTTATGGCC ACAAACTAGG GCAAGGATTT GGTCTACATA   
  
  
+ ATCACCTATT CAAAAGGAAA TATTGTTCTA TATAATTTTC AGACAGTTTT ATATTCTCTT TATTTATGTA   
  
  
+ TTATTGATGA ATAGTTATGC ACAGGGCATA TCCGGCCGGT GGTTCTTTCA CTATTTTTAT GCATAACAAT   
  
  
+ TGGCCATCAA TGGCTTCTCT TATTTTATAC ATAACAATTG GACATATTGG AAGTGGTCAT CACTATTGTT   
  
  
+ GAGCAGTCAG TCTCAGACTC GATTTTTTAT ACAAACGTAA CCACATAGAG ATTACCCAGC ATAGCTTAAT   
  
  
+ TACCTGCTGG AACCAAACAT GCATGCATAC CTCTTTTTAT TTACGTGTTG TCATTGTCTT TGACTGCTTA   
  
  
+ AGATTCTAAA CAAGTTTAAT AGGTGTTTG  

- ACAAATTAAT AATATTAAGA ACTCCAAAAC ACAAACTTTA CGACTCCTAA AAAGATCAAG ATCTCTCTCT   
  
  
- CTGTCTCTGT CTCTCTTTGT TCTGTACGTG GAGAGTTGGT TTAGCAGGGG GTGTTAAAGT TGCGTTCAGC   
  
  
- ACGATGGAGA GAGAGATTGA GAGAAAGAGA GAGATTTCTT ATCAACTCAA AAACGAGTTC TTCGTTTAAA   
  
  
- CAGGGTGTTG TTGTTATCTA AAGGTGAATG AATATTGTTA ATATACTAGA AACTTCCCTT GCCTCTTCCA   
  
  
- TGCATGCATG ATATATAATG AAGGGTCATG ATATAAAAAG GGCAGTTAAA AAAACGCATG TATATTTCAC   
  
  
- ACAATAACTC TAAACTACTA AAACATTACT AAAATATCCT CCTCGAATGG CAGATGTTAA ATTCTTCTTT   
  
  
- GTTACTAATG CAATTAACCT TACAAATCCG ATTATCATGT ACTAATAACA TGTATGCGTG TACAACATAT   
  
  
- CTATTTATAA ATATAAAGAG GTTCTGTTTT TGGGATTTTA AATAGAAAAA GGTGGTTGAT GTGAGAACTC   
  
  
- AAAGTGTAAA ATTGAGATTA CAACAACAGC ATTTAAATCT GAACATAAAA TATAAGAAAA CAATGTATAT   
  
  
- GTAACATAGT ATGAGTACGT ACGTATATAT CATTTGAATA ATTTGAGTTA AGAAGTACAT AAAATGCATT   
  
  
- TGGTTAGCTC AATAAAACAT ACTCATTATA TAAAACGGGA ATACCATAGT AATGCTGGTA GTTGATCGAT   
  
  
- ACACTAACTT TACTCTTGGT ACTTCTTGAT TAAAGCCTAA TTACGATCCT TCTTGTATAC ATCATTCAAC   
  
  
- TCACTACACA TGATTAACAT ACGTAGTAAA ACATTGTAGT GCACTAGGTT TTTTTACACA ATATGCCAAT   
  
  
- GAGGTTCGTT ACGAGTATTA TACTTATACA TATGTATTTA CCAATTTTAC GCATAATAGG ATTATTAACT   
  
  
- GATATTTTAT TTTATACTCT TATGATATAT ATACATACAC ACACACACAC ACATACACAT CCCAATCACT   
  
  
- ACGAAGTACC GTACACATCC TAATCACTAC GAAATACCGG TGTTTGATCC CGTTCCTAAA CCAGATGTAT   
  
  
- TAGTGGATAA GTTTTCCTTT ATAACAAGAT ATATTAAAAG TCTGTCAAAA TATAAGAGAA ATAAATACAT   
  
  
- AATAACTACT TATCAATACG TGTCCCGTAT AGGCCGGCCA CCAAGAAAGT GATAAAAATA CGTATTGTTA   
  
  
- ACCGGTAGTT ACCGAAGAGA ATAAAATATG TATTGTTAAC CTGTATAACC TTCACCAGTA GTGATAACAA   
  
  
- CTCGTCAGTC AGAGTCTGAG CTAAAAAATA TGTTTGCATT GGTGTATCTC TAATGGGTCG TATCGAATTA   
  
  
- ATGGACGACC TTGGTTTGTA CGTACGTATG GAGAAAAATA AATGCACAAC AGTAACAGAA ACTGACGAAT   
  
  
- TCTAAGATTT GTTCAAATTA TCCACAAAC

+     MYB

| Site Name | Organism | Position | Strand | Matrix score. | sequence | function |
| --- | --- | --- | --- | --- | --- | --- |
| MYB | Arabidopsis thaliana | 1368 | + | 6 | TAACCA |  |
| MYB | Arabidopsis thaliana | 950 | - | 6 | TAACCA |  |
| MYB | Arabidopsis thaliana | 105 | + | 6 | CAACCA |  |

>037399   
+ TGTTTAATTA TTATAATTCT TGAGGTTTTG TGTTTGAAAT GCTGAGGATT TTTCTAGTTC TAGAGAGAGA   
  
  
+ GACAGAGACA GAGAGAAACA AGACATGCAC CTCTCAACCA AATCGTCCCC CACAATTTCA ACGCAAGTCG   
  
  
+ TGCTACCTCT CTCTCTAACT CTCTTTCTCT CTCTAAAGAA TAGTTGAGTT TTTGCTCAAG AAGCAAATTT   
  
  
+ GTCCCACAAC AACAATAGAT TTCCACTTAC TTATAACAAT TATATGATCT TTGAAGGGAA CGGAGAAGGT   
  
  
+ ACGTACGTAC TATATATTAC TTCCCAGTAC TATATTTTTC CCGTCAATTT TTTTGCGTAC ATATAAAGTG   
  
  
+ TGTTATTGAG ATTTGATGAT TTTGTAATGA TTTTATAGGA GGAGCTTACC GTCTACAATT TAAGAAGAAA   
  
  
+ CAATGATTAC GTTAATTGGA ATGTTTAGGC TAATAGTACA TGATTATTGT ACATACGCAC ATGTTGTATA   
  
  
+ GATAAATATT TATATTTCTC CAAGACAAAA ACCCTAAAAT TTATCTTTTT CCACCAACTA CACTCTTGAG   
  
  
+ TTTCACATTT TAACTCTAAT GTTGTTGTCG TAAATTTAGA CTTGTATTTT ATATTCTTTT GTTACATATA   
  
  
+ CATTGTATCA TACTCATGCA TGCATATATA GTAAACTTAT TAAACTCAAT TCTTCATGTA TTTTACGTAA   
  
  
+ ACCAATCGAG TTATTTTGTA TGAGTAATAT ATTTTGCCCT TATGGTATCA TTACGACCAT CAACTAGCTA   
  
  
+ TGTGATTGAA ATGAGAACCA TGAAGAACTA ATTTCGGATT AATGCTAGGA AGAACATATG TAGTAAGTTG   
  
  
+ AGTGATGTGT ACTAATTGTA TGCATCATTT TGTAACATCA CGTGATCCAA AAAAATGTGT TATACGGTTA   
  
  
+ CTCCAAGCAA TGCTCATAAT ATGAATATGT ATACATAAAT GGTTAAAATG CGTATTATCC TAATAATTGA   
  
  
+ CTATAAAATA AAATATGAGA ATACTATATA TATGTATGTG TGTGTGTGTG TGTATGTGTA GGGTTAGTGA   
  
  
+ TGCTTCATGG CATGTGTAGG ATTAGTGATG CTTTATGGCC ACAAACTAGG GCAAGGATTT GGTCTACATA   
  
  
+ ATCACCTATT CAAAAGGAAA TATTGTTCTA TATAATTTTC AGACAGTTTT ATATTCTCTT TATTTATGTA   
  
  
+ TTATTGATGA ATAGTTATGC ACAGGGCATA TCCGGCCGGT GGTTCTTTCA CTATTTTTAT GCATAACAAT   
  
  
+ TGGCCATCAA TGGCTTCTCT TATTTTATAC ATAACAATTG GACATATTGG AAGTGGTCAT CACTATTGTT   
  
  
+ GAGCAGTCAG TCTCAGACTC GATTTTTTAT ACAAACGTAA CCACATAGAG ATTACCCAGC ATAGCTTAAT   
  
  
+ TACCTGCTGG AACCAAACAT GCATGCATAC CTCTTTTTAT TTACGTGTTG TCATTGTCTT TGACTGCTTA   
  
  
+ AGATTCTAAA CAAGTTTAAT AGGTGTTTG  

- ACAAATTAAT AATATTAAGA ACTCCAAAAC ACAAACTTTA CGACTCCTAA AAAGATCAAG ATCTCTCTCT   
  
  
- CTGTCTCTGT CTCTCTTTGT TCTGTACGTG GAGAGTTGGT TTAGCAGGGG GTGTTAAAGT TGCGTTCAGC   
  
  
- ACGATGGAGA GAGAGATTGA GAGAAAGAGA GAGATTTCTT ATCAACTCAA AAACGAGTTC TTCGTTTAAA   
  
  
- CAGGGTGTTG TTGTTATCTA AAGGTGAATG AATATTGTTA ATATACTAGA AACTTCCCTT GCCTCTTCCA   
  
  
- TGCATGCATG ATATATAATG AAGGGTCATG ATATAAAAAG GGCAGTTAAA AAAACGCATG TATATTTCAC   
  
  
- ACAATAACTC TAAACTACTA AAACATTACT AAAATATCCT CCTCGAATGG CAGATGTTAA ATTCTTCTTT   
  
  
- GTTACTAATG CAATTAACCT TACAAATCCG ATTATCATGT ACTAATAACA TGTATGCGTG TACAACATAT   
  
  
- CTATTTATAA ATATAAAGAG GTTCTGTTTT TGGGATTTTA AATAGAAAAA GGTGGTTGAT GTGAGAACTC   
  
  
- AAAGTGTAAA ATTGAGATTA CAACAACAGC ATTTAAATCT GAACATAAAA TATAAGAAAA CAATGTATAT   
  
  
- GTAACATAGT ATGAGTACGT ACGTATATAT CATTTGAATA ATTTGAGTTA AGAAGTACAT AAAATGCATT   
  
  
- TGGTTAGCTC AATAAAACAT ACTCATTATA TAAAACGGGA ATACCATAGT AATGCTGGTA GTTGATCGAT   
  
  
- ACACTAACTT TACTCTTGGT ACTTCTTGAT TAAAGCCTAA TTACGATCCT TCTTGTATAC ATCATTCAAC   
  
  
- TCACTACACA TGATTAACAT ACGTAGTAAA ACATTGTAGT GCACTAGGTT TTTTTACACA ATATGCCAAT   
  
  
- GAGGTTCGTT ACGAGTATTA TACTTATACA TATGTATTTA CCAATTTTAC GCATAATAGG ATTATTAACT   
  
  
- GATATTTTAT TTTATACTCT TATGATATAT ATACATACAC ACACACACAC ACATACACAT CCCAATCACT   
  
  
- ACGAAGTACC GTACACATCC TAATCACTAC GAAATACCGG TGTTTGATCC CGTTCCTAAA CCAGATGTAT   
  
  
- TAGTGGATAA GTTTTCCTTT ATAACAAGAT ATATTAAAAG TCTGTCAAAA TATAAGAGAA ATAAATACAT   
  
  
- AATAACTACT TATCAATACG TGTCCCGTAT AGGCCGGCCA CCAAGAAAGT GATAAAAATA CGTATTGTTA   
  
  
- ACCGGTAGTT ACCGAAGAGA ATAAAATATG TATTGTTAAC CTGTATAACC TTCACCAGTA GTGATAACAA   
  
  
- CTCGTCAGTC AGAGTCTGAG CTAAAAAATA TGTTTGCATT GGTGTATCTC TAATGGGTCG TATCGAATTA   
  
  
- ATGGACGACC TTGGTTTGTA CGTACGTATG GAGAAAAATA AATGCACAAC AGTAACAGAA ACTGACGAAT   
  
  
- TCTAAGATTT GTTCAAATTA TCCACAAAC

+     MYB-like sequence

| Site Name | Organism | Position | Strand | Matrix score. | sequence | function |
| --- | --- | --- | --- | --- | --- | --- |
| MYB-like sequence | Arabidopsis thaliana | 1368 | + | 6 | TAACCA |  |
| MYB-like sequence | Arabidopsis thaliana | 950 | - | 6 | TAACCA |  |

>037399   
+ TGTTTAATTA TTATAATTCT TGAGGTTTTG TGTTTGAAAT GCTGAGGATT TTTCTAGTTC TAGAGAGAGA   
  
  
+ GACAGAGACA GAGAGAAACA AGACATGCAC CTCTCAACCA AATCGTCCCC CACAATTTCA ACGCAAGTCG   
  
  
+ TGCTACCTCT CTCTCTAACT CTCTTTCTCT CTCTAAAGAA TAGTTGAGTT TTTGCTCAAG AAGCAAATTT   
  
  
+ GTCCCACAAC AACAATAGAT TTCCACTTAC TTATAACAAT TATATGATCT TTGAAGGGAA CGGAGAAGGT   
  
  
+ ACGTACGTAC TATATATTAC TTCCCAGTAC TATATTTTTC CCGTCAATTT TTTTGCGTAC ATATAAAGTG   
  
  
+ TGTTATTGAG ATTTGATGAT TTTGTAATGA TTTTATAGGA GGAGCTTACC GTCTACAATT TAAGAAGAAA   
  
  
+ CAATGATTAC GTTAATTGGA ATGTTTAGGC TAATAGTACA TGATTATTGT ACATACGCAC ATGTTGTATA   
  
  
+ GATAAATATT TATATTTCTC CAAGACAAAA ACCCTAAAAT TTATCTTTTT CCACCAACTA CACTCTTGAG   
  
  
+ TTTCACATTT TAACTCTAAT GTTGTTGTCG TAAATTTAGA CTTGTATTTT ATATTCTTTT GTTACATATA   
  
  
+ CATTGTATCA TACTCATGCA TGCATATATA GTAAACTTAT TAAACTCAAT TCTTCATGTA TTTTACGTAA   
  
  
+ ACCAATCGAG TTATTTTGTA TGAGTAATAT ATTTTGCCCT TATGGTATCA TTACGACCAT CAACTAGCTA   
  
  
+ TGTGATTGAA ATGAGAACCA TGAAGAACTA ATTTCGGATT AATGCTAGGA AGAACATATG TAGTAAGTTG   
  
  
+ AGTGATGTGT ACTAATTGTA TGCATCATTT TGTAACATCA CGTGATCCAA AAAAATGTGT TATACGGTTA   
  
  
+ CTCCAAGCAA TGCTCATAAT ATGAATATGT ATACATAAAT GGTTAAAATG CGTATTATCC TAATAATTGA   
  
  
+ CTATAAAATA AAATATGAGA ATACTATATA TATGTATGTG TGTGTGTGTG TGTATGTGTA GGGTTAGTGA   
  
  
+ TGCTTCATGG CATGTGTAGG ATTAGTGATG CTTTATGGCC ACAAACTAGG GCAAGGATTT GGTCTACATA   
  
  
+ ATCACCTATT CAAAAGGAAA TATTGTTCTA TATAATTTTC AGACAGTTTT ATATTCTCTT TATTTATGTA   
  
  
+ TTATTGATGA ATAGTTATGC ACAGGGCATA TCCGGCCGGT GGTTCTTTCA CTATTTTTAT GCATAACAAT   
  
  
+ TGGCCATCAA TGGCTTCTCT TATTTTATAC ATAACAATTG GACATATTGG AAGTGGTCAT CACTATTGTT   
  
  
+ GAGCAGTCAG TCTCAGACTC GATTTTTTAT ACAAACGTAA CCACATAGAG ATTACCCAGC ATAGCTTAAT   
  
  
+ TACCTGCTGG AACCAAACAT GCATGCATAC CTCTTTTTAT TTACGTGTTG TCATTGTCTT TGACTGCTTA   
  
  
+ AGATTCTAAA CAAGTTTAAT AGGTGTTTG  

- ACAAATTAAT AATATTAAGA ACTCCAAAAC ACAAACTTTA CGACTCCTAA AAAGATCAAG ATCTCTCTCT   
  
  
- CTGTCTCTGT CTCTCTTTGT TCTGTACGTG GAGAGTTGGT TTAGCAGGGG GTGTTAAAGT TGCGTTCAGC   
  
  
- ACGATGGAGA GAGAGATTGA GAGAAAGAGA GAGATTTCTT ATCAACTCAA AAACGAGTTC TTCGTTTAAA   
  
  
- CAGGGTGTTG TTGTTATCTA AAGGTGAATG AATATTGTTA ATATACTAGA AACTTCCCTT GCCTCTTCCA   
  
  
- TGCATGCATG ATATATAATG AAGGGTCATG ATATAAAAAG GGCAGTTAAA AAAACGCATG TATATTTCAC   
  
  
- ACAATAACTC TAAACTACTA AAACATTACT AAAATATCCT CCTCGAATGG CAGATGTTAA ATTCTTCTTT   
  
  
- GTTACTAATG CAATTAACCT TACAAATCCG ATTATCATGT ACTAATAACA TGTATGCGTG TACAACATAT   
  
  
- CTATTTATAA ATATAAAGAG GTTCTGTTTT TGGGATTTTA AATAGAAAAA GGTGGTTGAT GTGAGAACTC   
  
  
- AAAGTGTAAA ATTGAGATTA CAACAACAGC ATTTAAATCT GAACATAAAA TATAAGAAAA CAATGTATAT   
  
  
- GTAACATAGT ATGAGTACGT ACGTATATAT CATTTGAATA ATTTGAGTTA AGAAGTACAT AAAATGCATT   
  
  
- TGGTTAGCTC AATAAAACAT ACTCATTATA TAAAACGGGA ATACCATAGT AATGCTGGTA GTTGATCGAT   
  
  
- ACACTAACTT TACTCTTGGT ACTTCTTGAT TAAAGCCTAA TTACGATCCT TCTTGTATAC ATCATTCAAC   
  
  
- TCACTACACA TGATTAACAT ACGTAGTAAA ACATTGTAGT GCACTAGGTT TTTTTACACA ATATGCCAAT   
  
  
- GAGGTTCGTT ACGAGTATTA TACTTATACA TATGTATTTA CCAATTTTAC GCATAATAGG ATTATTAACT   
  
  
- GATATTTTAT TTTATACTCT TATGATATAT ATACATACAC ACACACACAC ACATACACAT CCCAATCACT   
  
  
- ACGAAGTACC GTACACATCC TAATCACTAC GAAATACCGG TGTTTGATCC CGTTCCTAAA CCAGATGTAT   
  
  
- TAGTGGATAA GTTTTCCTTT ATAACAAGAT ATATTAAAAG TCTGTCAAAA TATAAGAGAA ATAAATACAT   
  
  
- AATAACTACT TATCAATACG TGTCCCGTAT AGGCCGGCCA CCAAGAAAGT GATAAAAATA CGTATTGTTA   
  
  
- ACCGGTAGTT ACCGAAGAGA ATAAAATATG TATTGTTAAC CTGTATAACC TTCACCAGTA GTGATAACAA   
  
  
- CTCGTCAGTC AGAGTCTGAG CTAAAAAATA TGTTTGCATT GGTGTATCTC TAATGGGTCG TATCGAATTA   
  
  
- ATGGACGACC TTGGTTTGTA CGTACGTATG GAGAAAAATA AATGCACAAC AGTAACAGAA ACTGACGAAT   
  
  
- TCTAAGATTT GTTCAAATTA TCCACAAAC

+     MYC

| Site Name | Organism | Position | Strand | Matrix score. | sequence | function |
| --- | --- | --- | --- | --- | --- | --- |
| MYC | Arabidopsis thaliana | 1295 | - | 6 | CAATTG |  |
| MYC | Arabidopsis thaliana | 1061 | + | 6 | CATGTG |  |
| MYC | Arabidopsis thaliana | 1257 | - | 6 | CAATTG |  |
| MYC | Arabidopsis thaliana | 478 | - | 6 | CATGTG |  |

>037399   
+ TGTTTAATTA TTATAATTCT TGAGGTTTTG TGTTTGAAAT GCTGAGGATT TTTCTAGTTC TAGAGAGAGA   
  
  
+ GACAGAGACA GAGAGAAACA AGACATGCAC CTCTCAACCA AATCGTCCCC CACAATTTCA ACGCAAGTCG   
  
  
+ TGCTACCTCT CTCTCTAACT CTCTTTCTCT CTCTAAAGAA TAGTTGAGTT TTTGCTCAAG AAGCAAATTT   
  
  
+ GTCCCACAAC AACAATAGAT TTCCACTTAC TTATAACAAT TATATGATCT TTGAAGGGAA CGGAGAAGGT   
  
  
+ ACGTACGTAC TATATATTAC TTCCCAGTAC TATATTTTTC CCGTCAATTT TTTTGCGTAC ATATAAAGTG   
  
  
+ TGTTATTGAG ATTTGATGAT TTTGTAATGA TTTTATAGGA GGAGCTTACC GTCTACAATT TAAGAAGAAA   
  
  
+ CAATGATTAC GTTAATTGGA ATGTTTAGGC TAATAGTACA TGATTATTGT ACATACGCAC ATGTTGTATA   
  
  
+ GATAAATATT TATATTTCTC CAAGACAAAA ACCCTAAAAT TTATCTTTTT CCACCAACTA CACTCTTGAG   
  
  
+ TTTCACATTT TAACTCTAAT GTTGTTGTCG TAAATTTAGA CTTGTATTTT ATATTCTTTT GTTACATATA   
  
  
+ CATTGTATCA TACTCATGCA TGCATATATA GTAAACTTAT TAAACTCAAT TCTTCATGTA TTTTACGTAA   
  
  
+ ACCAATCGAG TTATTTTGTA TGAGTAATAT ATTTTGCCCT TATGGTATCA TTACGACCAT CAACTAGCTA   
  
  
+ TGTGATTGAA ATGAGAACCA TGAAGAACTA ATTTCGGATT AATGCTAGGA AGAACATATG TAGTAAGTTG   
  
  
+ AGTGATGTGT ACTAATTGTA TGCATCATTT TGTAACATCA CGTGATCCAA AAAAATGTGT TATACGGTTA   
  
  
+ CTCCAAGCAA TGCTCATAAT ATGAATATGT ATACATAAAT GGTTAAAATG CGTATTATCC TAATAATTGA   
  
  
+ CTATAAAATA AAATATGAGA ATACTATATA TATGTATGTG TGTGTGTGTG TGTATGTGTA GGGTTAGTGA   
  
  
+ TGCTTCATGG CATGTGTAGG ATTAGTGATG CTTTATGGCC ACAAACTAGG GCAAGGATTT GGTCTACATA   
  
  
+ ATCACCTATT CAAAAGGAAA TATTGTTCTA TATAATTTTC AGACAGTTTT ATATTCTCTT TATTTATGTA   
  
  
+ TTATTGATGA ATAGTTATGC ACAGGGCATA TCCGGCCGGT GGTTCTTTCA CTATTTTTAT GCATAACAAT   
  
  
+ TGGCCATCAA TGGCTTCTCT TATTTTATAC ATAACAATTG GACATATTGG AAGTGGTCAT CACTATTGTT   
  
  
+ GAGCAGTCAG TCTCAGACTC GATTTTTTAT ACAAACGTAA CCACATAGAG ATTACCCAGC ATAGCTTAAT   
  
  
+ TACCTGCTGG AACCAAACAT GCATGCATAC CTCTTTTTAT TTACGTGTTG TCATTGTCTT TGACTGCTTA   
  
  
+ AGATTCTAAA CAAGTTTAAT AGGTGTTTG  

- ACAAATTAAT AATATTAAGA ACTCCAAAAC ACAAACTTTA CGACTCCTAA AAAGATCAAG ATCTCTCTCT   
  
  
- CTGTCTCTGT CTCTCTTTGT TCTGTACGTG GAGAGTTGGT TTAGCAGGGG GTGTTAAAGT TGCGTTCAGC   
  
  
- ACGATGGAGA GAGAGATTGA GAGAAAGAGA GAGATTTCTT ATCAACTCAA AAACGAGTTC TTCGTTTAAA   
  
  
- CAGGGTGTTG TTGTTATCTA AAGGTGAATG AATATTGTTA ATATACTAGA AACTTCCCTT GCCTCTTCCA   
  
  
- TGCATGCATG ATATATAATG AAGGGTCATG ATATAAAAAG GGCAGTTAAA AAAACGCATG TATATTTCAC   
  
  
- ACAATAACTC TAAACTACTA AAACATTACT AAAATATCCT CCTCGAATGG CAGATGTTAA ATTCTTCTTT   
  
  
- GTTACTAATG CAATTAACCT TACAAATCCG ATTATCATGT ACTAATAACA TGTATGCGTG TACAACATAT   
  
  
- CTATTTATAA ATATAAAGAG GTTCTGTTTT TGGGATTTTA AATAGAAAAA GGTGGTTGAT GTGAGAACTC   
  
  
- AAAGTGTAAA ATTGAGATTA CAACAACAGC ATTTAAATCT GAACATAAAA TATAAGAAAA CAATGTATAT   
  
  
- GTAACATAGT ATGAGTACGT ACGTATATAT CATTTGAATA ATTTGAGTTA AGAAGTACAT AAAATGCATT   
  
  
- TGGTTAGCTC AATAAAACAT ACTCATTATA TAAAACGGGA ATACCATAGT AATGCTGGTA GTTGATCGAT   
  
  
- ACACTAACTT TACTCTTGGT ACTTCTTGAT TAAAGCCTAA TTACGATCCT TCTTGTATAC ATCATTCAAC   
  
  
- TCACTACACA TGATTAACAT ACGTAGTAAA ACATTGTAGT GCACTAGGTT TTTTTACACA ATATGCCAAT   
  
  
- GAGGTTCGTT ACGAGTATTA TACTTATACA TATGTATTTA CCAATTTTAC GCATAATAGG ATTATTAACT   
  
  
- GATATTTTAT TTTATACTCT TATGATATAT ATACATACAC ACACACACAC ACATACACAT CCCAATCACT   
  
  
- ACGAAGTACC GTACACATCC TAATCACTAC GAAATACCGG TGTTTGATCC CGTTCCTAAA CCAGATGTAT   
  
  
- TAGTGGATAA GTTTTCCTTT ATAACAAGAT ATATTAAAAG TCTGTCAAAA TATAAGAGAA ATAAATACAT   
  
  
- AATAACTACT TATCAATACG TGTCCCGTAT AGGCCGGCCA CCAAGAAAGT GATAAAAATA CGTATTGTTA   
  
  
- ACCGGTAGTT ACCGAAGAGA ATAAAATATG TATTGTTAAC CTGTATAACC TTCACCAGTA GTGATAACAA   
  
  
- CTCGTCAGTC AGAGTCTGAG CTAAAAAATA TGTTTGCATT GGTGTATCTC TAATGGGTCG TATCGAATTA   
  
  
- ATGGACGACC TTGGTTTGTA CGTACGTATG GAGAAAAATA AATGCACAAC AGTAACAGAA ACTGACGAAT   
  
  
- TCTAAGATTT GTTCAAATTA TCCACAAAC

+     Myc

| Site Name | Organism | Position | Strand | Matrix score. | sequence | function |
| --- | --- | --- | --- | --- | --- | --- |
| Myc | Arabidopsis thaliana | 1276 | + | 7 | TCTCTTA |  |

>037399   
+ TGTTTAATTA TTATAATTCT TGAGGTTTTG TGTTTGAAAT GCTGAGGATT TTTCTAGTTC TAGAGAGAGA   
  
  
+ GACAGAGACA GAGAGAAACA AGACATGCAC CTCTCAACCA AATCGTCCCC CACAATTTCA ACGCAAGTCG   
  
  
+ TGCTACCTCT CTCTCTAACT CTCTTTCTCT CTCTAAAGAA TAGTTGAGTT TTTGCTCAAG AAGCAAATTT   
  
  
+ GTCCCACAAC AACAATAGAT TTCCACTTAC TTATAACAAT TATATGATCT TTGAAGGGAA CGGAGAAGGT   
  
  
+ ACGTACGTAC TATATATTAC TTCCCAGTAC TATATTTTTC CCGTCAATTT TTTTGCGTAC ATATAAAGTG   
  
  
+ TGTTATTGAG ATTTGATGAT TTTGTAATGA TTTTATAGGA GGAGCTTACC GTCTACAATT TAAGAAGAAA   
  
  
+ CAATGATTAC GTTAATTGGA ATGTTTAGGC TAATAGTACA TGATTATTGT ACATACGCAC ATGTTGTATA   
  
  
+ GATAAATATT TATATTTCTC CAAGACAAAA ACCCTAAAAT TTATCTTTTT CCACCAACTA CACTCTTGAG   
  
  
+ TTTCACATTT TAACTCTAAT GTTGTTGTCG TAAATTTAGA CTTGTATTTT ATATTCTTTT GTTACATATA   
  
  
+ CATTGTATCA TACTCATGCA TGCATATATA GTAAACTTAT TAAACTCAAT TCTTCATGTA TTTTACGTAA   
  
  
+ ACCAATCGAG TTATTTTGTA TGAGTAATAT ATTTTGCCCT TATGGTATCA TTACGACCAT CAACTAGCTA   
  
  
+ TGTGATTGAA ATGAGAACCA TGAAGAACTA ATTTCGGATT AATGCTAGGA AGAACATATG TAGTAAGTTG   
  
  
+ AGTGATGTGT ACTAATTGTA TGCATCATTT TGTAACATCA CGTGATCCAA AAAAATGTGT TATACGGTTA   
  
  
+ CTCCAAGCAA TGCTCATAAT ATGAATATGT ATACATAAAT GGTTAAAATG CGTATTATCC TAATAATTGA   
  
  
+ CTATAAAATA AAATATGAGA ATACTATATA TATGTATGTG TGTGTGTGTG TGTATGTGTA GGGTTAGTGA   
  
  
+ TGCTTCATGG CATGTGTAGG ATTAGTGATG CTTTATGGCC ACAAACTAGG GCAAGGATTT GGTCTACATA   
  
  
+ ATCACCTATT CAAAAGGAAA TATTGTTCTA TATAATTTTC AGACAGTTTT ATATTCTCTT TATTTATGTA   
  
  
+ TTATTGATGA ATAGTTATGC ACAGGGCATA TCCGGCCGGT GGTTCTTTCA CTATTTTTAT GCATAACAAT   
  
  
+ TGGCCATCAA TGGCTTCTCT TATTTTATAC ATAACAATTG GACATATTGG AAGTGGTCAT CACTATTGTT   
  
  
+ GAGCAGTCAG TCTCAGACTC GATTTTTTAT ACAAACGTAA CCACATAGAG ATTACCCAGC ATAGCTTAAT   
  
  
+ TACCTGCTGG AACCAAACAT GCATGCATAC CTCTTTTTAT TTACGTGTTG TCATTGTCTT TGACTGCTTA   
  
  
+ AGATTCTAAA CAAGTTTAAT AGGTGTTTG  

- ACAAATTAAT AATATTAAGA ACTCCAAAAC ACAAACTTTA CGACTCCTAA AAAGATCAAG ATCTCTCTCT   
  
  
- CTGTCTCTGT CTCTCTTTGT TCTGTACGTG GAGAGTTGGT TTAGCAGGGG GTGTTAAAGT TGCGTTCAGC   
  
  
- ACGATGGAGA GAGAGATTGA GAGAAAGAGA GAGATTTCTT ATCAACTCAA AAACGAGTTC TTCGTTTAAA   
  
  
- CAGGGTGTTG TTGTTATCTA AAGGTGAATG AATATTGTTA ATATACTAGA AACTTCCCTT GCCTCTTCCA   
  
  
- TGCATGCATG ATATATAATG AAGGGTCATG ATATAAAAAG GGCAGTTAAA AAAACGCATG TATATTTCAC   
  
  
- ACAATAACTC TAAACTACTA AAACATTACT AAAATATCCT CCTCGAATGG CAGATGTTAA ATTCTTCTTT   
  
  
- GTTACTAATG CAATTAACCT TACAAATCCG ATTATCATGT ACTAATAACA TGTATGCGTG TACAACATAT   
  
  
- CTATTTATAA ATATAAAGAG GTTCTGTTTT TGGGATTTTA AATAGAAAAA GGTGGTTGAT GTGAGAACTC   
  
  
- AAAGTGTAAA ATTGAGATTA CAACAACAGC ATTTAAATCT GAACATAAAA TATAAGAAAA CAATGTATAT   
  
  
- GTAACATAGT ATGAGTACGT ACGTATATAT CATTTGAATA ATTTGAGTTA AGAAGTACAT AAAATGCATT   
  
  
- TGGTTAGCTC AATAAAACAT ACTCATTATA TAAAACGGGA ATACCATAGT AATGCTGGTA GTTGATCGAT   
  
  
- ACACTAACTT TACTCTTGGT ACTTCTTGAT TAAAGCCTAA TTACGATCCT TCTTGTATAC ATCATTCAAC   
  
  
- TCACTACACA TGATTAACAT ACGTAGTAAA ACATTGTAGT GCACTAGGTT TTTTTACACA ATATGCCAAT   
  
  
- GAGGTTCGTT ACGAGTATTA TACTTATACA TATGTATTTA CCAATTTTAC GCATAATAGG ATTATTAACT   
  
  
- GATATTTTAT TTTATACTCT TATGATATAT ATACATACAC ACACACACAC ACATACACAT CCCAATCACT   
  
  
- ACGAAGTACC GTACACATCC TAATCACTAC GAAATACCGG TGTTTGATCC CGTTCCTAAA CCAGATGTAT   
  
  
- TAGTGGATAA GTTTTCCTTT ATAACAAGAT ATATTAAAAG TCTGTCAAAA TATAAGAGAA ATAAATACAT   
  
  
- AATAACTACT TATCAATACG TGTCCCGTAT AGGCCGGCCA CCAAGAAAGT GATAAAAATA CGTATTGTTA   
  
  
- ACCGGTAGTT ACCGAAGAGA ATAAAATATG TATTGTTAAC CTGTATAACC TTCACCAGTA GTGATAACAA   
  
  
- CTCGTCAGTC AGAGTCTGAG CTAAAAAATA TGTTTGCATT GGTGTATCTC TAATGGGTCG TATCGAATTA   
  
  
- ATGGACGACC TTGGTTTGTA CGTACGTATG GAGAAAAATA AATGCACAAC AGTAACAGAA ACTGACGAAT   
  
  
- TCTAAGATTT GTTCAAATTA TCCACAAAC

+     P-box

| Site Name | Organism | Position | Strand | Matrix score. | sequence | function |
| --- | --- | --- | --- | --- | --- | --- |
| P-box | Oryza sativa | 1131 | - | 7 | CCTTTTG | gibberellin-responsive element |

>037399   
+ TGTTTAATTA TTATAATTCT TGAGGTTTTG TGTTTGAAAT GCTGAGGATT TTTCTAGTTC TAGAGAGAGA   
  
  
+ GACAGAGACA GAGAGAAACA AGACATGCAC CTCTCAACCA AATCGTCCCC CACAATTTCA ACGCAAGTCG   
  
  
+ TGCTACCTCT CTCTCTAACT CTCTTTCTCT CTCTAAAGAA TAGTTGAGTT TTTGCTCAAG AAGCAAATTT   
  
  
+ GTCCCACAAC AACAATAGAT TTCCACTTAC TTATAACAAT TATATGATCT TTGAAGGGAA CGGAGAAGGT   
  
  
+ ACGTACGTAC TATATATTAC TTCCCAGTAC TATATTTTTC CCGTCAATTT TTTTGCGTAC ATATAAAGTG   
  
  
+ TGTTATTGAG ATTTGATGAT TTTGTAATGA TTTTATAGGA GGAGCTTACC GTCTACAATT TAAGAAGAAA   
  
  
+ CAATGATTAC GTTAATTGGA ATGTTTAGGC TAATAGTACA TGATTATTGT ACATACGCAC ATGTTGTATA   
  
  
+ GATAAATATT TATATTTCTC CAAGACAAAA ACCCTAAAAT TTATCTTTTT CCACCAACTA CACTCTTGAG   
  
  
+ TTTCACATTT TAACTCTAAT GTTGTTGTCG TAAATTTAGA CTTGTATTTT ATATTCTTTT GTTACATATA   
  
  
+ CATTGTATCA TACTCATGCA TGCATATATA GTAAACTTAT TAAACTCAAT TCTTCATGTA TTTTACGTAA   
  
  
+ ACCAATCGAG TTATTTTGTA TGAGTAATAT ATTTTGCCCT TATGGTATCA TTACGACCAT CAACTAGCTA   
  
  
+ TGTGATTGAA ATGAGAACCA TGAAGAACTA ATTTCGGATT AATGCTAGGA AGAACATATG TAGTAAGTTG   
  
  
+ AGTGATGTGT ACTAATTGTA TGCATCATTT TGTAACATCA CGTGATCCAA AAAAATGTGT TATACGGTTA   
  
  
+ CTCCAAGCAA TGCTCATAAT ATGAATATGT ATACATAAAT GGTTAAAATG CGTATTATCC TAATAATTGA   
  
  
+ CTATAAAATA AAATATGAGA ATACTATATA TATGTATGTG TGTGTGTGTG TGTATGTGTA GGGTTAGTGA   
  
  
+ TGCTTCATGG CATGTGTAGG ATTAGTGATG CTTTATGGCC ACAAACTAGG GCAAGGATTT GGTCTACATA   
  
  
+ ATCACCTATT CAAAAGGAAA TATTGTTCTA TATAATTTTC AGACAGTTTT ATATTCTCTT TATTTATGTA   
  
  
+ TTATTGATGA ATAGTTATGC ACAGGGCATA TCCGGCCGGT GGTTCTTTCA CTATTTTTAT GCATAACAAT   
  
  
+ TGGCCATCAA TGGCTTCTCT TATTTTATAC ATAACAATTG GACATATTGG AAGTGGTCAT CACTATTGTT   
  
  
+ GAGCAGTCAG TCTCAGACTC GATTTTTTAT ACAAACGTAA CCACATAGAG ATTACCCAGC ATAGCTTAAT   
  
  
+ TACCTGCTGG AACCAAACAT GCATGCATAC CTCTTTTTAT TTACGTGTTG TCATTGTCTT TGACTGCTTA   
  
  
+ AGATTCTAAA CAAGTTTAAT AGGTGTTTG  

- ACAAATTAAT AATATTAAGA ACTCCAAAAC ACAAACTTTA CGACTCCTAA AAAGATCAAG ATCTCTCTCT   
  
  
- CTGTCTCTGT CTCTCTTTGT TCTGTACGTG GAGAGTTGGT TTAGCAGGGG GTGTTAAAGT TGCGTTCAGC   
  
  
- ACGATGGAGA GAGAGATTGA GAGAAAGAGA GAGATTTCTT ATCAACTCAA AAACGAGTTC TTCGTTTAAA   
  
  
- CAGGGTGTTG TTGTTATCTA AAGGTGAATG AATATTGTTA ATATACTAGA AACTTCCCTT GCCTCTTCCA   
  
  
- TGCATGCATG ATATATAATG AAGGGTCATG ATATAAAAAG GGCAGTTAAA AAAACGCATG TATATTTCAC   
  
  
- ACAATAACTC TAAACTACTA AAACATTACT AAAATATCCT CCTCGAATGG CAGATGTTAA ATTCTTCTTT   
  
  
- GTTACTAATG CAATTAACCT TACAAATCCG ATTATCATGT ACTAATAACA TGTATGCGTG TACAACATAT   
  
  
- CTATTTATAA ATATAAAGAG GTTCTGTTTT TGGGATTTTA AATAGAAAAA GGTGGTTGAT GTGAGAACTC   
  
  
- AAAGTGTAAA ATTGAGATTA CAACAACAGC ATTTAAATCT GAACATAAAA TATAAGAAAA CAATGTATAT   
  
  
- GTAACATAGT ATGAGTACGT ACGTATATAT CATTTGAATA ATTTGAGTTA AGAAGTACAT AAAATGCATT   
  
  
- TGGTTAGCTC AATAAAACAT ACTCATTATA TAAAACGGGA ATACCATAGT AATGCTGGTA GTTGATCGAT   
  
  
- ACACTAACTT TACTCTTGGT ACTTCTTGAT TAAAGCCTAA TTACGATCCT TCTTGTATAC ATCATTCAAC   
  
  
- TCACTACACA TGATTAACAT ACGTAGTAAA ACATTGTAGT GCACTAGGTT TTTTTACACA ATATGCCAAT   
  
  
- GAGGTTCGTT ACGAGTATTA TACTTATACA TATGTATTTA CCAATTTTAC GCATAATAGG ATTATTAACT   
  
  
- GATATTTTAT TTTATACTCT TATGATATAT ATACATACAC ACACACACAC ACATACACAT CCCAATCACT   
  
  
- ACGAAGTACC GTACACATCC TAATCACTAC GAAATACCGG TGTTTGATCC CGTTCCTAAA CCAGATGTAT   
  
  
- TAGTGGATAA GTTTTCCTTT ATAACAAGAT ATATTAAAAG TCTGTCAAAA TATAAGAGAA ATAAATACAT   
  
  
- AATAACTACT TATCAATACG TGTCCCGTAT AGGCCGGCCA CCAAGAAAGT GATAAAAATA CGTATTGTTA   
  
  
- ACCGGTAGTT ACCGAAGAGA ATAAAATATG TATTGTTAAC CTGTATAACC TTCACCAGTA GTGATAACAA   
  
  
- CTCGTCAGTC AGAGTCTGAG CTAAAAAATA TGTTTGCATT GGTGTATCTC TAATGGGTCG TATCGAATTA   
  
  
- ATGGACGACC TTGGTTTGTA CGTACGTATG GAGAAAAATA AATGCACAAC AGTAACAGAA ACTGACGAAT   
  
  
- TCTAAGATTT GTTCAAATTA TCCACAAAC

+     RY-element

| Site Name | Organism | Position | Strand | Matrix score. | sequence | function |
| --- | --- | --- | --- | --- | --- | --- |
| RY-element | Helianthus annuus | 1418 | - | 8 | CATGCATG | cis-acting regulatory element involved in seed-specific regulation |
| RY-element | Helianthus annuus | 645 | + | 8 | CATGCATG | cis-acting regulatory element involved in seed-specific regulation |

>037399   
+ TGTTTAATTA TTATAATTCT TGAGGTTTTG TGTTTGAAAT GCTGAGGATT TTTCTAGTTC TAGAGAGAGA   
  
  
+ GACAGAGACA GAGAGAAACA AGACATGCAC CTCTCAACCA AATCGTCCCC CACAATTTCA ACGCAAGTCG   
  
  
+ TGCTACCTCT CTCTCTAACT CTCTTTCTCT CTCTAAAGAA TAGTTGAGTT TTTGCTCAAG AAGCAAATTT   
  
  
+ GTCCCACAAC AACAATAGAT TTCCACTTAC TTATAACAAT TATATGATCT TTGAAGGGAA CGGAGAAGGT   
  
  
+ ACGTACGTAC TATATATTAC TTCCCAGTAC TATATTTTTC CCGTCAATTT TTTTGCGTAC ATATAAAGTG   
  
  
+ TGTTATTGAG ATTTGATGAT TTTGTAATGA TTTTATAGGA GGAGCTTACC GTCTACAATT TAAGAAGAAA   
  
  
+ CAATGATTAC GTTAATTGGA ATGTTTAGGC TAATAGTACA TGATTATTGT ACATACGCAC ATGTTGTATA   
  
  
+ GATAAATATT TATATTTCTC CAAGACAAAA ACCCTAAAAT TTATCTTTTT CCACCAACTA CACTCTTGAG   
  
  
+ TTTCACATTT TAACTCTAAT GTTGTTGTCG TAAATTTAGA CTTGTATTTT ATATTCTTTT GTTACATATA   
  
  
+ CATTGTATCA TACTCATGCA TGCATATATA GTAAACTTAT TAAACTCAAT TCTTCATGTA TTTTACGTAA   
  
  
+ ACCAATCGAG TTATTTTGTA TGAGTAATAT ATTTTGCCCT TATGGTATCA TTACGACCAT CAACTAGCTA   
  
  
+ TGTGATTGAA ATGAGAACCA TGAAGAACTA ATTTCGGATT AATGCTAGGA AGAACATATG TAGTAAGTTG   
  
  
+ AGTGATGTGT ACTAATTGTA TGCATCATTT TGTAACATCA CGTGATCCAA AAAAATGTGT TATACGGTTA   
  
  
+ CTCCAAGCAA TGCTCATAAT ATGAATATGT ATACATAAAT GGTTAAAATG CGTATTATCC TAATAATTGA   
  
  
+ CTATAAAATA AAATATGAGA ATACTATATA TATGTATGTG TGTGTGTGTG TGTATGTGTA GGGTTAGTGA   
  
  
+ TGCTTCATGG CATGTGTAGG ATTAGTGATG CTTTATGGCC ACAAACTAGG GCAAGGATTT GGTCTACATA   
  
  
+ ATCACCTATT CAAAAGGAAA TATTGTTCTA TATAATTTTC AGACAGTTTT ATATTCTCTT TATTTATGTA   
  
  
+ TTATTGATGA ATAGTTATGC ACAGGGCATA TCCGGCCGGT GGTTCTTTCA CTATTTTTAT GCATAACAAT   
  
  
+ TGGCCATCAA TGGCTTCTCT TATTTTATAC ATAACAATTG GACATATTGG AAGTGGTCAT CACTATTGTT   
  
  
+ GAGCAGTCAG TCTCAGACTC GATTTTTTAT ACAAACGTAA CCACATAGAG ATTACCCAGC ATAGCTTAAT   
  
  
+ TACCTGCTGG AACCAAACAT GCATGCATAC CTCTTTTTAT TTACGTGTTG TCATTGTCTT TGACTGCTTA   
  
  
+ AGATTCTAAA CAAGTTTAAT AGGTGTTTG  

- ACAAATTAAT AATATTAAGA ACTCCAAAAC ACAAACTTTA CGACTCCTAA AAAGATCAAG ATCTCTCTCT   
  
  
- CTGTCTCTGT CTCTCTTTGT TCTGTACGTG GAGAGTTGGT TTAGCAGGGG GTGTTAAAGT TGCGTTCAGC   
  
  
- ACGATGGAGA GAGAGATTGA GAGAAAGAGA GAGATTTCTT ATCAACTCAA AAACGAGTTC TTCGTTTAAA   
  
  
- CAGGGTGTTG TTGTTATCTA AAGGTGAATG AATATTGTTA ATATACTAGA AACTTCCCTT GCCTCTTCCA   
  
  
- TGCATGCATG ATATATAATG AAGGGTCATG ATATAAAAAG GGCAGTTAAA AAAACGCATG TATATTTCAC   
  
  
- ACAATAACTC TAAACTACTA AAACATTACT AAAATATCCT CCTCGAATGG CAGATGTTAA ATTCTTCTTT   
  
  
- GTTACTAATG CAATTAACCT TACAAATCCG ATTATCATGT ACTAATAACA TGTATGCGTG TACAACATAT   
  
  
- CTATTTATAA ATATAAAGAG GTTCTGTTTT TGGGATTTTA AATAGAAAAA GGTGGTTGAT GTGAGAACTC   
  
  
- AAAGTGTAAA ATTGAGATTA CAACAACAGC ATTTAAATCT GAACATAAAA TATAAGAAAA CAATGTATAT   
  
  
- GTAACATAGT ATGAGTACGT ACGTATATAT CATTTGAATA ATTTGAGTTA AGAAGTACAT AAAATGCATT   
  
  
- TGGTTAGCTC AATAAAACAT ACTCATTATA TAAAACGGGA ATACCATAGT AATGCTGGTA GTTGATCGAT   
  
  
- ACACTAACTT TACTCTTGGT ACTTCTTGAT TAAAGCCTAA TTACGATCCT TCTTGTATAC ATCATTCAAC   
  
  
- TCACTACACA TGATTAACAT ACGTAGTAAA ACATTGTAGT GCACTAGGTT TTTTTACACA ATATGCCAAT   
  
  
- GAGGTTCGTT ACGAGTATTA TACTTATACA TATGTATTTA CCAATTTTAC GCATAATAGG ATTATTAACT   
  
  
- GATATTTTAT TTTATACTCT TATGATATAT ATACATACAC ACACACACAC ACATACACAT CCCAATCACT   
  
  
- ACGAAGTACC GTACACATCC TAATCACTAC GAAATACCGG TGTTTGATCC CGTTCCTAAA CCAGATGTAT   
  
  
- TAGTGGATAA GTTTTCCTTT ATAACAAGAT ATATTAAAAG TCTGTCAAAA TATAAGAGAA ATAAATACAT   
  
  
- AATAACTACT TATCAATACG TGTCCCGTAT AGGCCGGCCA CCAAGAAAGT GATAAAAATA CGTATTGTTA   
  
  
- ACCGGTAGTT ACCGAAGAGA ATAAAATATG TATTGTTAAC CTGTATAACC TTCACCAGTA GTGATAACAA   
  
  
- CTCGTCAGTC AGAGTCTGAG CTAAAAAATA TGTTTGCATT GGTGTATCTC TAATGGGTCG TATCGAATTA   
  
  
- ATGGACGACC TTGGTTTGTA CGTACGTATG GAGAAAAATA AATGCACAAC AGTAACAGAA ACTGACGAAT   
  
  
- TCTAAGATTT GTTCAAATTA TCCACAAAC

+     TATA

| Site Name | Organism | Position | Strand | Matrix score. | sequence | function |
| --- | --- | --- | --- | --- | --- | --- |
| TATA | Arabidopsis thaliana | 1282 | - | 8 | TATAAAAT |  |
| TATA | Arabidopsis thaliana | 982 | + | 8 | TATAAAAT |  |
| TATA | Arabidopsis thaliana | 380 | - | 8 | TATAAAAT |  |
| TATA | Arabidopsis thaliana | 606 | - | 8 | TATAAAAT |  |

>037399   
+ TGTTTAATTA TTATAATTCT TGAGGTTTTG TGTTTGAAAT GCTGAGGATT TTTCTAGTTC TAGAGAGAGA   
  
  
+ GACAGAGACA GAGAGAAACA AGACATGCAC CTCTCAACCA AATCGTCCCC CACAATTTCA ACGCAAGTCG   
  
  
+ TGCTACCTCT CTCTCTAACT CTCTTTCTCT CTCTAAAGAA TAGTTGAGTT TTTGCTCAAG AAGCAAATTT   
  
  
+ GTCCCACAAC AACAATAGAT TTCCACTTAC TTATAACAAT TATATGATCT TTGAAGGGAA CGGAGAAGGT   
  
  
+ ACGTACGTAC TATATATTAC TTCCCAGTAC TATATTTTTC CCGTCAATTT TTTTGCGTAC ATATAAAGTG   
  
  
+ TGTTATTGAG ATTTGATGAT TTTGTAATGA TTTTATAGGA GGAGCTTACC GTCTACAATT TAAGAAGAAA   
  
  
+ CAATGATTAC GTTAATTGGA ATGTTTAGGC TAATAGTACA TGATTATTGT ACATACGCAC ATGTTGTATA   
  
  
+ GATAAATATT TATATTTCTC CAAGACAAAA ACCCTAAAAT TTATCTTTTT CCACCAACTA CACTCTTGAG   
  
  
+ TTTCACATTT TAACTCTAAT GTTGTTGTCG TAAATTTAGA CTTGTATTTT ATATTCTTTT GTTACATATA   
  
  
+ CATTGTATCA TACTCATGCA TGCATATATA GTAAACTTAT TAAACTCAAT TCTTCATGTA TTTTACGTAA   
  
  
+ ACCAATCGAG TTATTTTGTA TGAGTAATAT ATTTTGCCCT TATGGTATCA TTACGACCAT CAACTAGCTA   
  
  
+ TGTGATTGAA ATGAGAACCA TGAAGAACTA ATTTCGGATT AATGCTAGGA AGAACATATG TAGTAAGTTG   
  
  
+ AGTGATGTGT ACTAATTGTA TGCATCATTT TGTAACATCA CGTGATCCAA AAAAATGTGT TATACGGTTA   
  
  
+ CTCCAAGCAA TGCTCATAAT ATGAATATGT ATACATAAAT GGTTAAAATG CGTATTATCC TAATAATTGA   
  
  
+ CTATAAAATA AAATATGAGA ATACTATATA TATGTATGTG TGTGTGTGTG TGTATGTGTA GGGTTAGTGA   
  
  
+ TGCTTCATGG CATGTGTAGG ATTAGTGATG CTTTATGGCC ACAAACTAGG GCAAGGATTT GGTCTACATA   
  
  
+ ATCACCTATT CAAAAGGAAA TATTGTTCTA TATAATTTTC AGACAGTTTT ATATTCTCTT TATTTATGTA   
  
  
+ TTATTGATGA ATAGTTATGC ACAGGGCATA TCCGGCCGGT GGTTCTTTCA CTATTTTTAT GCATAACAAT   
  
  
+ TGGCCATCAA TGGCTTCTCT TATTTTATAC ATAACAATTG GACATATTGG AAGTGGTCAT CACTATTGTT   
  
  
+ GAGCAGTCAG TCTCAGACTC GATTTTTTAT ACAAACGTAA CCACATAGAG ATTACCCAGC ATAGCTTAAT   
  
  
+ TACCTGCTGG AACCAAACAT GCATGCATAC CTCTTTTTAT TTACGTGTTG TCATTGTCTT TGACTGCTTA   
  
  
+ AGATTCTAAA CAAGTTTAAT AGGTGTTTG  

- ACAAATTAAT AATATTAAGA ACTCCAAAAC ACAAACTTTA CGACTCCTAA AAAGATCAAG ATCTCTCTCT   
  
  
- CTGTCTCTGT CTCTCTTTGT TCTGTACGTG GAGAGTTGGT TTAGCAGGGG GTGTTAAAGT TGCGTTCAGC   
  
  
- ACGATGGAGA GAGAGATTGA GAGAAAGAGA GAGATTTCTT ATCAACTCAA AAACGAGTTC TTCGTTTAAA   
  
  
- CAGGGTGTTG TTGTTATCTA AAGGTGAATG AATATTGTTA ATATACTAGA AACTTCCCTT GCCTCTTCCA   
  
  
- TGCATGCATG ATATATAATG AAGGGTCATG ATATAAAAAG GGCAGTTAAA AAAACGCATG TATATTTCAC   
  
  
- ACAATAACTC TAAACTACTA AAACATTACT AAAATATCCT CCTCGAATGG CAGATGTTAA ATTCTTCTTT   
  
  
- GTTACTAATG CAATTAACCT TACAAATCCG ATTATCATGT ACTAATAACA TGTATGCGTG TACAACATAT   
  
  
- CTATTTATAA ATATAAAGAG GTTCTGTTTT TGGGATTTTA AATAGAAAAA GGTGGTTGAT GTGAGAACTC   
  
  
- AAAGTGTAAA ATTGAGATTA CAACAACAGC ATTTAAATCT GAACATAAAA TATAAGAAAA CAATGTATAT   
  
  
- GTAACATAGT ATGAGTACGT ACGTATATAT CATTTGAATA ATTTGAGTTA AGAAGTACAT AAAATGCATT   
  
  
- TGGTTAGCTC AATAAAACAT ACTCATTATA TAAAACGGGA ATACCATAGT AATGCTGGTA GTTGATCGAT   
  
  
- ACACTAACTT TACTCTTGGT ACTTCTTGAT TAAAGCCTAA TTACGATCCT TCTTGTATAC ATCATTCAAC   
  
  
- TCACTACACA TGATTAACAT ACGTAGTAAA ACATTGTAGT GCACTAGGTT TTTTTACACA ATATGCCAAT   
  
  
- GAGGTTCGTT ACGAGTATTA TACTTATACA TATGTATTTA CCAATTTTAC GCATAATAGG ATTATTAACT   
  
  
- GATATTTTAT TTTATACTCT TATGATATAT ATACATACAC ACACACACAC ACATACACAT CCCAATCACT   
  
  
- ACGAAGTACC GTACACATCC TAATCACTAC GAAATACCGG TGTTTGATCC CGTTCCTAAA CCAGATGTAT   
  
  
- TAGTGGATAA GTTTTCCTTT ATAACAAGAT ATATTAAAAG TCTGTCAAAA TATAAGAGAA ATAAATACAT   
  
  
- AATAACTACT TATCAATACG TGTCCCGTAT AGGCCGGCCA CCAAGAAAGT GATAAAAATA CGTATTGTTA   
  
  
- ACCGGTAGTT ACCGAAGAGA ATAAAATATG TATTGTTAAC CTGTATAACC TTCACCAGTA GTGATAACAA   
  
  
- CTCGTCAGTC AGAGTCTGAG CTAAAAAATA TGTTTGCATT GGTGTATCTC TAATGGGTCG TATCGAATTA   
  
  
- ATGGACGACC TTGGTTTGTA CGTACGTATG GAGAAAAATA AATGCACAAC AGTAACAGAA ACTGACGAAT   
  
  
- TCTAAGATTT GTTCAAATTA TCCACAAAC

+     TATA-box

| Site Name | Organism | Position | Strand | Matrix score. | sequence | function |
| --- | --- | --- | --- | --- | --- | --- |
| TATA-box | Helianthus annuus | 1168 | - | 6 | TATAAA | core promoter element around -30 of transcription start |
| TATA-box | Arabidopsis thaliana | 609 | - | 5 | TATAA | core promoter element around -30 of transcription start |
| TATA-box | Helianthus annuus | 1356 | - | 6 | TATAAA | core promoter element around -30 of transcription start |
| TATA-box | Arabidopsis thaliana | 1357 | - | 5 | TATAA | core promoter element around -30 of transcription start |
| TATA-box | Arabidopsis thaliana | 1286 | - | 4 | TATA | core promoter element around -30 of transcription start |
| TATA-box | Arabidopsis thaliana | 1358 | - | 4 | TATA | core promoter element around -30 of transcription start |
| TATA-box | Arabidopsis thaliana | 610 | + | 4 | TATA | core promoter element around -30 of transcription start |
| TATA-box | Brassica napus | 654 | + | 6 | ATATAT | core promoter element around -30 of transcription start |
| TATA-box | Helianthus annuus | 382 | - | 6 | TATAAA | core promoter element around -30 of transcription start |
| TATA-box | Pisum sativum | 1167 | - | 7 | TATAAAA | core promoter element around -30 of transcription start |
| TATA-box | Arabidopsis thaliana | 1151 | - | 4 | TATA | core promoter element around -30 of transcription start |
| TATA-box | Arabidopsis thaliana | 627 | + | 4 | TATA | core promoter element around -30 of transcription start |
| TATA-box | Helianthus annuus | 608 | - | 6 | TATAAA | core promoter element around -30 of transcription start |
| TATA-box | Pisum sativum | 1355 | - | 7 | TATAAAA | core promoter element around -30 of transcription start |
| TATA-box | Helianthus annuus | 1284 | - | 6 | TATAAA | core promoter element around -30 of transcription start |
| TATA-box | Arabidopsis thaliana | 1285 | - | 5 | TATAA | core promoter element around -30 of transcription start |
| TATA-box | Brassica oleracea | 1150 | + | 6 | ATATAA | core promoter element around -30 of transcription start |
| TATA-box | Helianthus annuus | 938 | - | 6 | TATACA | core promoter element around -30 of transcription start |
| TATA-box | Arabidopsis thaliana | 1170 | - | 4 | TATA | core promoter element around -30 of transcription start |
| TATA-box | Oryza sativa | 1183 | - | 8 | TACATAAA | core promoter element around -30 of transcription start |
| TATA-box | Pisum sativum | 607 | - | 7 | TATAAAA | core promoter element around -30 of transcription start |
| TATA-box | Helianthus annuus | 499 | - | 6 | TATAAA | core promoter element around -30 of transcription start |
| TATA-box | Arabidopsis thaliana | 500 | - | 5 | TATAA | core promoter element around -30 of transcription start |
| TATA-box | Arabidopsis thaliana | 1169 | - | 5 | TATAA | core promoter element around -30 of transcription start |
| TATA-box | Pisum sativum | 381 | - | 7 | TATAAAA | core promoter element around -30 of transcription start |
| TATA-box | Arabidopsis thaliana | 501 | + | 4 | TATA | core promoter element around -30 of transcription start |
| TATA-box | Brassica juncea | 498 | - | 7 | TATAAAT | core promoter element around -30 of transcription start |
| TATA-box | Pisum sativum | 1283 | - | 7 | TATAAAA | core promoter element around -30 of transcription start |
| TATA-box | Arabidopsis thaliana | 940 | - | 4 | TATA | core promoter element around -30 of transcription start |
| TATA-box | Arabidopsis thaliana | 1149 | - | 6 | TATATA | core promoter element around -30 of transcription start |
| TATA-box | Daucus carota | 497 | - | 8 | TATAAATA | core promoter element around -30 of transcription start |
| TATA-box | Arabidopsis thaliana | 1007 | - | 6 | TATATA | core promoter element around -30 of transcription start |
| TATA-box | Arabidopsis thaliana | 1009 | - | 4 | TATA | core promoter element around -30 of transcription start |
| TATA-box | Avena sativa | 495 | + | 12 | TATATTTATATTT | core promoter element around -30 of transcription start |
| TATA-box | Brassica napus | 727 | + | 6 | ATATAT | core promoter element around -30 of transcription start |
| TATA-box | Arabidopsis thaliana | 982 | - | 4 | TATA | core promoter element around -30 of transcription start |
| TATA-box | Arabidopsis thaliana | 487 | + | 4 | TATA | core promoter element around -30 of transcription start |
| TATA-box | Helianthus annuus | 485 | - | 6 | TATACA | core promoter element around -30 of transcription start |
| TATA-box | Arabidopsis thaliana | 383 | - | 5 | TATAA | core promoter element around -30 of transcription start |
| TATA-box | Arabidopsis thaliana | 900 | - | 5 | TATAA | core promoter element around -30 of transcription start |
| TATA-box | Arabidopsis thaliana | 384 | + | 4 | TATA | core promoter element around -30 of transcription start |
| TATA-box | Oryza sativa | 370 | - | 7 | TACAAAA | core promoter element around -30 of transcription start |
| TATA-box | Arabidopsis thaliana | 901 | - | 4 | TATA | core promoter element around -30 of transcription start |
| TATA-box | Oryza sativa | 714 | - | 7 | TACAAAA | core promoter element around -30 of transcription start |
| TATA-box | Arabidopsis thaliana | 380 | - | 9 | ccTATAAAaa | core promoter element around -30 of transcription start |
| TATA-box | Oryza sativa | 942 | + | 8 | TACATAAA | core promoter element around -30 of transcription start |
| TATA-box | Brassica napus | 292 | + | 6 | ATATAT | core promoter element around -30 of transcription start |
| TATA-box | Brassica napus | 1008 | - | 6 | ATATAT | core promoter element around -30 of transcription start |
| TATA-box | Arabidopsis thaliana | 291 | + | 6 | TATATA | core promoter element around -30 of transcription start |
| TATA-box | Brassica napus | 1006 | - | 6 | ATATAT | core promoter element around -30 of transcription start |
| TATA-box | Arabidopsis thaliana | 251 | + | 4 | TATA | core promoter element around -30 of transcription start |
| TATA-box | Arabidopsis thaliana | 250 | - | 5 | TATAA | core promoter element around -30 of transcription start |
| TATA-box | Brassica napus | 10 | + | 6 | ATTATA | core promoter element around -30 of transcription start |
| TATA-box | Arabidopsis thaliana | 241 | - | 5 | TATAA | core promoter element around -30 of transcription start |
| TATA-box | Arabidopsis thaliana | 12 | + | 4 | TATA | core promoter element around -30 of transcription start |
| TATA-box | Arabidopsis thaliana | 11 | - | 5 | TATAA | core promoter element around -30 of transcription start |
| TATA-box | Arabidopsis thaliana | 1005 | - | 6 | TATATA | core promoter element around -30 of transcription start |
| TATA-box | Arabidopsis thaliana | 657 | + | 4 | TATA | core promoter element around -30 of transcription start |
| TATA-box | Arabidopsis thaliana | 728 | + | 4 | TATA | core promoter element around -30 of transcription start |
| TATA-box | Brassica napus | 249 | + | 6 | ATTATA | core promoter element around -30 of transcription start |
| TATA-box | Arabidopsis thaliana | 242 | + | 4 | TATA | core promoter element around -30 of transcription start |
| TATA-box | Arabidopsis thaliana | 342 | + | 4 | TATA | core promoter element around -30 of transcription start |
| TATA-box | Brassica oleracea | 341 | + | 6 | ATATAA | core promoter element around -30 of transcription start |
| TATA-box | Oryza sativa | 868 | - | 7 | TACAAAA | core promoter element around -30 of transcription start |
| TATA-box | Arabidopsis thaliana | 655 | + | 6 | TATATA | core promoter element around -30 of transcription start |
| TATA-box | Arabidopsis thaliana | 293 | + | 4 | TATA | core promoter element around -30 of transcription start |
| TATA-box | Arabidopsis thaliana | 311 | + | 4 | TATA | core promoter element around -30 of transcription start |

>037399   
+ TGTTTAATTA TTATAATTCT TGAGGTTTTG TGTTTGAAAT GCTGAGGATT TTTCTAGTTC TAGAGAGAGA   
  
  
+ GACAGAGACA GAGAGAAACA AGACATGCAC CTCTCAACCA AATCGTCCCC CACAATTTCA ACGCAAGTCG   
  
  
+ TGCTACCTCT CTCTCTAACT CTCTTTCTCT CTCTAAAGAA TAGTTGAGTT TTTGCTCAAG AAGCAAATTT   
  
  
+ GTCCCACAAC AACAATAGAT TTCCACTTAC TTATAACAAT TATATGATCT TTGAAGGGAA CGGAGAAGGT   
  
  
+ ACGTACGTAC TATATATTAC TTCCCAGTAC TATATTTTTC CCGTCAATTT TTTTGCGTAC ATATAAAGTG   
  
  
+ TGTTATTGAG ATTTGATGAT TTTGTAATGA TTTTATAGGA GGAGCTTACC GTCTACAATT TAAGAAGAAA   
  
  
+ CAATGATTAC GTTAATTGGA ATGTTTAGGC TAATAGTACA TGATTATTGT ACATACGCAC ATGTTGTATA   
  
  
+ GATAAATATT TATATTTCTC CAAGACAAAA ACCCTAAAAT TTATCTTTTT CCACCAACTA CACTCTTGAG   
  
  
+ TTTCACATTT TAACTCTAAT GTTGTTGTCG TAAATTTAGA CTTGTATTTT ATATTCTTTT GTTACATATA   
  
  
+ CATTGTATCA TACTCATGCA TGCATATATA GTAAACTTAT TAAACTCAAT TCTTCATGTA TTTTACGTAA   
  
  
+ ACCAATCGAG TTATTTTGTA TGAGTAATAT ATTTTGCCCT TATGGTATCA TTACGACCAT CAACTAGCTA   
  
  
+ TGTGATTGAA ATGAGAACCA TGAAGAACTA ATTTCGGATT AATGCTAGGA AGAACATATG TAGTAAGTTG   
  
  
+ AGTGATGTGT ACTAATTGTA TGCATCATTT TGTAACATCA CGTGATCCAA AAAAATGTGT TATACGGTTA   
  
  
+ CTCCAAGCAA TGCTCATAAT ATGAATATGT ATACATAAAT GGTTAAAATG CGTATTATCC TAATAATTGA   
  
  
+ CTATAAAATA AAATATGAGA ATACTATATA TATGTATGTG TGTGTGTGTG TGTATGTGTA GGGTTAGTGA   
  
  
+ TGCTTCATGG CATGTGTAGG ATTAGTGATG CTTTATGGCC ACAAACTAGG GCAAGGATTT GGTCTACATA   
  
  
+ ATCACCTATT CAAAAGGAAA TATTGTTCTA TATAATTTTC AGACAGTTTT ATATTCTCTT TATTTATGTA   
  
  
+ TTATTGATGA ATAGTTATGC ACAGGGCATA TCCGGCCGGT GGTTCTTTCA CTATTTTTAT GCATAACAAT   
  
  
+ TGGCCATCAA TGGCTTCTCT TATTTTATAC ATAACAATTG GACATATTGG AAGTGGTCAT CACTATTGTT   
  
  
+ GAGCAGTCAG TCTCAGACTC GATTTTTTAT ACAAACGTAA CCACATAGAG ATTACCCAGC ATAGCTTAAT   
  
  
+ TACCTGCTGG AACCAAACAT GCATGCATAC CTCTTTTTAT TTACGTGTTG TCATTGTCTT TGACTGCTTA   
  
  
+ AGATTCTAAA CAAGTTTAAT AGGTGTTTG  

- ACAAATTAAT AATATTAAGA ACTCCAAAAC ACAAACTTTA CGACTCCTAA AAAGATCAAG ATCTCTCTCT   
  
  
- CTGTCTCTGT CTCTCTTTGT TCTGTACGTG GAGAGTTGGT TTAGCAGGGG GTGTTAAAGT TGCGTTCAGC   
  
  
- ACGATGGAGA GAGAGATTGA GAGAAAGAGA GAGATTTCTT ATCAACTCAA AAACGAGTTC TTCGTTTAAA   
  
  
- CAGGGTGTTG TTGTTATCTA AAGGTGAATG AATATTGTTA ATATACTAGA AACTTCCCTT GCCTCTTCCA   
  
  
- TGCATGCATG ATATATAATG AAGGGTCATG ATATAAAAAG GGCAGTTAAA AAAACGCATG TATATTTCAC   
  
  
- ACAATAACTC TAAACTACTA AAACATTACT AAAATATCCT CCTCGAATGG CAGATGTTAA ATTCTTCTTT   
  
  
- GTTACTAATG CAATTAACCT TACAAATCCG ATTATCATGT ACTAATAACA TGTATGCGTG TACAACATAT   
  
  
- CTATTTATAA ATATAAAGAG GTTCTGTTTT TGGGATTTTA AATAGAAAAA GGTGGTTGAT GTGAGAACTC   
  
  
- AAAGTGTAAA ATTGAGATTA CAACAACAGC ATTTAAATCT GAACATAAAA TATAAGAAAA CAATGTATAT   
  
  
- GTAACATAGT ATGAGTACGT ACGTATATAT CATTTGAATA ATTTGAGTTA AGAAGTACAT AAAATGCATT   
  
  
- TGGTTAGCTC AATAAAACAT ACTCATTATA TAAAACGGGA ATACCATAGT AATGCTGGTA GTTGATCGAT   
  
  
- ACACTAACTT TACTCTTGGT ACTTCTTGAT TAAAGCCTAA TTACGATCCT TCTTGTATAC ATCATTCAAC   
  
  
- TCACTACACA TGATTAACAT ACGTAGTAAA ACATTGTAGT GCACTAGGTT TTTTTACACA ATATGCCAAT   
  
  
- GAGGTTCGTT ACGAGTATTA TACTTATACA TATGTATTTA CCAATTTTAC GCATAATAGG ATTATTAACT   
  
  
- GATATTTTAT TTTATACTCT TATGATATAT ATACATACAC ACACACACAC ACATACACAT CCCAATCACT   
  
  
- ACGAAGTACC GTACACATCC TAATCACTAC GAAATACCGG TGTTTGATCC CGTTCCTAAA CCAGATGTAT   
  
  
- TAGTGGATAA GTTTTCCTTT ATAACAAGAT ATATTAAAAG TCTGTCAAAA TATAAGAGAA ATAAATACAT   
  
  
- AATAACTACT TATCAATACG TGTCCCGTAT AGGCCGGCCA CCAAGAAAGT GATAAAAATA CGTATTGTTA   
  
  
- ACCGGTAGTT ACCGAAGAGA ATAAAATATG TATTGTTAAC CTGTATAACC TTCACCAGTA GTGATAACAA   
  
  
- CTCGTCAGTC AGAGTCTGAG CTAAAAAATA TGTTTGCATT GGTGTATCTC TAATGGGTCG TATCGAATTA   
  
  
- ATGGACGACC TTGGTTTGTA CGTACGTATG GAGAAAAATA AATGCACAAC AGTAACAGAA ACTGACGAAT   
  
  
- TCTAAGATTT GTTCAAATTA TCCACAAAC

+     TGACG-motif

| Site Name | Organism | Position | Strand | Matrix score. | sequence | function |
| --- | --- | --- | --- | --- | --- | --- |
| TGACG-motif | Hordeum vulgare | 322 | - | 5 | TGACG | cis-acting regulatory element involved in the MeJA-responsiveness |

>037399   
+ TGTTTAATTA TTATAATTCT TGAGGTTTTG TGTTTGAAAT GCTGAGGATT TTTCTAGTTC TAGAGAGAGA   
  
  
+ GACAGAGACA GAGAGAAACA AGACATGCAC CTCTCAACCA AATCGTCCCC CACAATTTCA ACGCAAGTCG   
  
  
+ TGCTACCTCT CTCTCTAACT CTCTTTCTCT CTCTAAAGAA TAGTTGAGTT TTTGCTCAAG AAGCAAATTT   
  
  
+ GTCCCACAAC AACAATAGAT TTCCACTTAC TTATAACAAT TATATGATCT TTGAAGGGAA CGGAGAAGGT   
  
  
+ ACGTACGTAC TATATATTAC TTCCCAGTAC TATATTTTTC CCGTCAATTT TTTTGCGTAC ATATAAAGTG   
  
  
+ TGTTATTGAG ATTTGATGAT TTTGTAATGA TTTTATAGGA GGAGCTTACC GTCTACAATT TAAGAAGAAA   
  
  
+ CAATGATTAC GTTAATTGGA ATGTTTAGGC TAATAGTACA TGATTATTGT ACATACGCAC ATGTTGTATA   
  
  
+ GATAAATATT TATATTTCTC CAAGACAAAA ACCCTAAAAT TTATCTTTTT CCACCAACTA CACTCTTGAG   
  
  
+ TTTCACATTT TAACTCTAAT GTTGTTGTCG TAAATTTAGA CTTGTATTTT ATATTCTTTT GTTACATATA   
  
  
+ CATTGTATCA TACTCATGCA TGCATATATA GTAAACTTAT TAAACTCAAT TCTTCATGTA TTTTACGTAA   
  
  
+ ACCAATCGAG TTATTTTGTA TGAGTAATAT ATTTTGCCCT TATGGTATCA TTACGACCAT CAACTAGCTA   
  
  
+ TGTGATTGAA ATGAGAACCA TGAAGAACTA ATTTCGGATT AATGCTAGGA AGAACATATG TAGTAAGTTG   
  
  
+ AGTGATGTGT ACTAATTGTA TGCATCATTT TGTAACATCA CGTGATCCAA AAAAATGTGT TATACGGTTA   
  
  
+ CTCCAAGCAA TGCTCATAAT ATGAATATGT ATACATAAAT GGTTAAAATG CGTATTATCC TAATAATTGA   
  
  
+ CTATAAAATA AAATATGAGA ATACTATATA TATGTATGTG TGTGTGTGTG TGTATGTGTA GGGTTAGTGA   
  
  
+ TGCTTCATGG CATGTGTAGG ATTAGTGATG CTTTATGGCC ACAAACTAGG GCAAGGATTT GGTCTACATA   
  
  
+ ATCACCTATT CAAAAGGAAA TATTGTTCTA TATAATTTTC AGACAGTTTT ATATTCTCTT TATTTATGTA   
  
  
+ TTATTGATGA ATAGTTATGC ACAGGGCATA TCCGGCCGGT GGTTCTTTCA CTATTTTTAT GCATAACAAT   
  
  
+ TGGCCATCAA TGGCTTCTCT TATTTTATAC ATAACAATTG GACATATTGG AAGTGGTCAT CACTATTGTT   
  
  
+ GAGCAGTCAG TCTCAGACTC GATTTTTTAT ACAAACGTAA CCACATAGAG ATTACCCAGC ATAGCTTAAT   
  
  
+ TACCTGCTGG AACCAAACAT GCATGCATAC CTCTTTTTAT TTACGTGTTG TCATTGTCTT TGACTGCTTA   
  
  
+ AGATTCTAAA CAAGTTTAAT AGGTGTTTG  

- ACAAATTAAT AATATTAAGA ACTCCAAAAC ACAAACTTTA CGACTCCTAA AAAGATCAAG ATCTCTCTCT   
  
  
- CTGTCTCTGT CTCTCTTTGT TCTGTACGTG GAGAGTTGGT TTAGCAGGGG GTGTTAAAGT TGCGTTCAGC   
  
  
- ACGATGGAGA GAGAGATTGA GAGAAAGAGA GAGATTTCTT ATCAACTCAA AAACGAGTTC TTCGTTTAAA   
  
  
- CAGGGTGTTG TTGTTATCTA AAGGTGAATG AATATTGTTA ATATACTAGA AACTTCCCTT GCCTCTTCCA   
  
  
- TGCATGCATG ATATATAATG AAGGGTCATG ATATAAAAAG GGCAGTTAAA AAAACGCATG TATATTTCAC   
  
  
- ACAATAACTC TAAACTACTA AAACATTACT AAAATATCCT CCTCGAATGG CAGATGTTAA ATTCTTCTTT   
  
  
- GTTACTAATG CAATTAACCT TACAAATCCG ATTATCATGT ACTAATAACA TGTATGCGTG TACAACATAT   
  
  
- CTATTTATAA ATATAAAGAG GTTCTGTTTT TGGGATTTTA AATAGAAAAA GGTGGTTGAT GTGAGAACTC   
  
  
- AAAGTGTAAA ATTGAGATTA CAACAACAGC ATTTAAATCT GAACATAAAA TATAAGAAAA CAATGTATAT   
  
  
- GTAACATAGT ATGAGTACGT ACGTATATAT CATTTGAATA ATTTGAGTTA AGAAGTACAT AAAATGCATT   
  
  
- TGGTTAGCTC AATAAAACAT ACTCATTATA TAAAACGGGA ATACCATAGT AATGCTGGTA GTTGATCGAT   
  
  
- ACACTAACTT TACTCTTGGT ACTTCTTGAT TAAAGCCTAA TTACGATCCT TCTTGTATAC ATCATTCAAC   
  
  
- TCACTACACA TGATTAACAT ACGTAGTAAA ACATTGTAGT GCACTAGGTT TTTTTACACA ATATGCCAAT   
  
  
- GAGGTTCGTT ACGAGTATTA TACTTATACA TATGTATTTA CCAATTTTAC GCATAATAGG ATTATTAACT   
  
  
- GATATTTTAT TTTATACTCT TATGATATAT ATACATACAC ACACACACAC ACATACACAT CCCAATCACT   
  
  
- ACGAAGTACC GTACACATCC TAATCACTAC GAAATACCGG TGTTTGATCC CGTTCCTAAA CCAGATGTAT   
  
  
- TAGTGGATAA GTTTTCCTTT ATAACAAGAT ATATTAAAAG TCTGTCAAAA TATAAGAGAA ATAAATACAT   
  
  
- AATAACTACT TATCAATACG TGTCCCGTAT AGGCCGGCCA CCAAGAAAGT GATAAAAATA CGTATTGTTA   
  
  
- ACCGGTAGTT ACCGAAGAGA ATAAAATATG TATTGTTAAC CTGTATAACC TTCACCAGTA GTGATAACAA   
  
  
- CTCGTCAGTC AGAGTCTGAG CTAAAAAATA TGTTTGCATT GGTGTATCTC TAATGGGTCG TATCGAATTA   
  
  
- ATGGACGACC TTGGTTTGTA CGTACGTATG GAGAAAAATA AATGCACAAC AGTAACAGAA ACTGACGAAT   
  
  
- TCTAAGATTT GTTCAAATTA TCCACAAAC

+     Unnamed\_\_4

| Site Name | Organism | Position | Strand | Matrix score. | sequence | function |
| --- | --- | --- | --- | --- | --- | --- |
| Unnamed\_\_4 | Petroselinum hortense | 911 | + | 4 | CTCC |  |
| Unnamed\_\_4 | Petroselinum hortense | 391 | - | 4 | CTCC |  |
| Unnamed\_\_4 | Petroselinum hortense | 508 | + | 4 | CTCC |  |
| Unnamed\_\_4 | Petroselinum hortense | 272 | - | 4 | CTCC |  |
| Unnamed\_\_4 | Petroselinum hortense | 388 | - | 4 | CTCC |  |

>037399   
+ TGTTTAATTA TTATAATTCT TGAGGTTTTG TGTTTGAAAT GCTGAGGATT TTTCTAGTTC TAGAGAGAGA   
  
  
+ GACAGAGACA GAGAGAAACA AGACATGCAC CTCTCAACCA AATCGTCCCC CACAATTTCA ACGCAAGTCG   
  
  
+ TGCTACCTCT CTCTCTAACT CTCTTTCTCT CTCTAAAGAA TAGTTGAGTT TTTGCTCAAG AAGCAAATTT   
  
  
+ GTCCCACAAC AACAATAGAT TTCCACTTAC TTATAACAAT TATATGATCT TTGAAGGGAA CGGAGAAGGT   
  
  
+ ACGTACGTAC TATATATTAC TTCCCAGTAC TATATTTTTC CCGTCAATTT TTTTGCGTAC ATATAAAGTG   
  
  
+ TGTTATTGAG ATTTGATGAT TTTGTAATGA TTTTATAGGA GGAGCTTACC GTCTACAATT TAAGAAGAAA   
  
  
+ CAATGATTAC GTTAATTGGA ATGTTTAGGC TAATAGTACA TGATTATTGT ACATACGCAC ATGTTGTATA   
  
  
+ GATAAATATT TATATTTCTC CAAGACAAAA ACCCTAAAAT TTATCTTTTT CCACCAACTA CACTCTTGAG   
  
  
+ TTTCACATTT TAACTCTAAT GTTGTTGTCG TAAATTTAGA CTTGTATTTT ATATTCTTTT GTTACATATA   
  
  
+ CATTGTATCA TACTCATGCA TGCATATATA GTAAACTTAT TAAACTCAAT TCTTCATGTA TTTTACGTAA   
  
  
+ ACCAATCGAG TTATTTTGTA TGAGTAATAT ATTTTGCCCT TATGGTATCA TTACGACCAT CAACTAGCTA   
  
  
+ TGTGATTGAA ATGAGAACCA TGAAGAACTA ATTTCGGATT AATGCTAGGA AGAACATATG TAGTAAGTTG   
  
  
+ AGTGATGTGT ACTAATTGTA TGCATCATTT TGTAACATCA CGTGATCCAA AAAAATGTGT TATACGGTTA   
  
  
+ CTCCAAGCAA TGCTCATAAT ATGAATATGT ATACATAAAT GGTTAAAATG CGTATTATCC TAATAATTGA   
  
  
+ CTATAAAATA AAATATGAGA ATACTATATA TATGTATGTG TGTGTGTGTG TGTATGTGTA GGGTTAGTGA   
  
  
+ TGCTTCATGG CATGTGTAGG ATTAGTGATG CTTTATGGCC ACAAACTAGG GCAAGGATTT GGTCTACATA   
  
  
+ ATCACCTATT CAAAAGGAAA TATTGTTCTA TATAATTTTC AGACAGTTTT ATATTCTCTT TATTTATGTA   
  
  
+ TTATTGATGA ATAGTTATGC ACAGGGCATA TCCGGCCGGT GGTTCTTTCA CTATTTTTAT GCATAACAAT   
  
  
+ TGGCCATCAA TGGCTTCTCT TATTTTATAC ATAACAATTG GACATATTGG AAGTGGTCAT CACTATTGTT   
  
  
+ GAGCAGTCAG TCTCAGACTC GATTTTTTAT ACAAACGTAA CCACATAGAG ATTACCCAGC ATAGCTTAAT   
  
  
+ TACCTGCTGG AACCAAACAT GCATGCATAC CTCTTTTTAT TTACGTGTTG TCATTGTCTT TGACTGCTTA   
  
  
+ AGATTCTAAA CAAGTTTAAT AGGTGTTTG  

- ACAAATTAAT AATATTAAGA ACTCCAAAAC ACAAACTTTA CGACTCCTAA AAAGATCAAG ATCTCTCTCT   
  
  
- CTGTCTCTGT CTCTCTTTGT TCTGTACGTG GAGAGTTGGT TTAGCAGGGG GTGTTAAAGT TGCGTTCAGC   
  
  
- ACGATGGAGA GAGAGATTGA GAGAAAGAGA GAGATTTCTT ATCAACTCAA AAACGAGTTC TTCGTTTAAA   
  
  
- CAGGGTGTTG TTGTTATCTA AAGGTGAATG AATATTGTTA ATATACTAGA AACTTCCCTT GCCTCTTCCA   
  
  
- TGCATGCATG ATATATAATG AAGGGTCATG ATATAAAAAG GGCAGTTAAA AAAACGCATG TATATTTCAC   
  
  
- ACAATAACTC TAAACTACTA AAACATTACT AAAATATCCT CCTCGAATGG CAGATGTTAA ATTCTTCTTT   
  
  
- GTTACTAATG CAATTAACCT TACAAATCCG ATTATCATGT ACTAATAACA TGTATGCGTG TACAACATAT   
  
  
- CTATTTATAA ATATAAAGAG GTTCTGTTTT TGGGATTTTA AATAGAAAAA GGTGGTTGAT GTGAGAACTC   
  
  
- AAAGTGTAAA ATTGAGATTA CAACAACAGC ATTTAAATCT GAACATAAAA TATAAGAAAA CAATGTATAT   
  
  
- GTAACATAGT ATGAGTACGT ACGTATATAT CATTTGAATA ATTTGAGTTA AGAAGTACAT AAAATGCATT   
  
  
- TGGTTAGCTC AATAAAACAT ACTCATTATA TAAAACGGGA ATACCATAGT AATGCTGGTA GTTGATCGAT   
  
  
- ACACTAACTT TACTCTTGGT ACTTCTTGAT TAAAGCCTAA TTACGATCCT TCTTGTATAC ATCATTCAAC   
  
  
- TCACTACACA TGATTAACAT ACGTAGTAAA ACATTGTAGT GCACTAGGTT TTTTTACACA ATATGCCAAT   
  
  
- GAGGTTCGTT ACGAGTATTA TACTTATACA TATGTATTTA CCAATTTTAC GCATAATAGG ATTATTAACT   
  
  
- GATATTTTAT TTTATACTCT TATGATATAT ATACATACAC ACACACACAC ACATACACAT CCCAATCACT   
  
  
- ACGAAGTACC GTACACATCC TAATCACTAC GAAATACCGG TGTTTGATCC CGTTCCTAAA CCAGATGTAT   
  
  
- TAGTGGATAA GTTTTCCTTT ATAACAAGAT ATATTAAAAG TCTGTCAAAA TATAAGAGAA ATAAATACAT   
  
  
- AATAACTACT TATCAATACG TGTCCCGTAT AGGCCGGCCA CCAAGAAAGT GATAAAAATA CGTATTGTTA   
  
  
- ACCGGTAGTT ACCGAAGAGA ATAAAATATG TATTGTTAAC CTGTATAACC TTCACCAGTA GTGATAACAA   
  
  
- CTCGTCAGTC AGAGTCTGAG CTAAAAAATA TGTTTGCATT GGTGTATCTC TAATGGGTCG TATCGAATTA   
  
  
- ATGGACGACC TTGGTTTGTA CGTACGTATG GAGAAAAATA AATGCACAAC AGTAACAGAA ACTGACGAAT   
  
  
- TCTAAGATTT GTTCAAATTA TCCACAAAC

+     Unnamed\_\_6

| Site Name | Organism | Position | Strand | Matrix score. | sequence | function |
| --- | --- | --- | --- | --- | --- | --- |
| Unnamed\_\_6 | Zea mays | 494 | - | 10 | taTAAATATct |  |

>037399   
+ TGTTTAATTA TTATAATTCT TGAGGTTTTG TGTTTGAAAT GCTGAGGATT TTTCTAGTTC TAGAGAGAGA   
  
  
+ GACAGAGACA GAGAGAAACA AGACATGCAC CTCTCAACCA AATCGTCCCC CACAATTTCA ACGCAAGTCG   
  
  
+ TGCTACCTCT CTCTCTAACT CTCTTTCTCT CTCTAAAGAA TAGTTGAGTT TTTGCTCAAG AAGCAAATTT   
  
  
+ GTCCCACAAC AACAATAGAT TTCCACTTAC TTATAACAAT TATATGATCT TTGAAGGGAA CGGAGAAGGT   
  
  
+ ACGTACGTAC TATATATTAC TTCCCAGTAC TATATTTTTC CCGTCAATTT TTTTGCGTAC ATATAAAGTG   
  
  
+ TGTTATTGAG ATTTGATGAT TTTGTAATGA TTTTATAGGA GGAGCTTACC GTCTACAATT TAAGAAGAAA   
  
  
+ CAATGATTAC GTTAATTGGA ATGTTTAGGC TAATAGTACA TGATTATTGT ACATACGCAC ATGTTGTATA   
  
  
+ GATAAATATT TATATTTCTC CAAGACAAAA ACCCTAAAAT TTATCTTTTT CCACCAACTA CACTCTTGAG   
  
  
+ TTTCACATTT TAACTCTAAT GTTGTTGTCG TAAATTTAGA CTTGTATTTT ATATTCTTTT GTTACATATA   
  
  
+ CATTGTATCA TACTCATGCA TGCATATATA GTAAACTTAT TAAACTCAAT TCTTCATGTA TTTTACGTAA   
  
  
+ ACCAATCGAG TTATTTTGTA TGAGTAATAT ATTTTGCCCT TATGGTATCA TTACGACCAT CAACTAGCTA   
  
  
+ TGTGATTGAA ATGAGAACCA TGAAGAACTA ATTTCGGATT AATGCTAGGA AGAACATATG TAGTAAGTTG   
  
  
+ AGTGATGTGT ACTAATTGTA TGCATCATTT TGTAACATCA CGTGATCCAA AAAAATGTGT TATACGGTTA   
  
  
+ CTCCAAGCAA TGCTCATAAT ATGAATATGT ATACATAAAT GGTTAAAATG CGTATTATCC TAATAATTGA   
  
  
+ CTATAAAATA AAATATGAGA ATACTATATA TATGTATGTG TGTGTGTGTG TGTATGTGTA GGGTTAGTGA   
  
  
+ TGCTTCATGG CATGTGTAGG ATTAGTGATG CTTTATGGCC ACAAACTAGG GCAAGGATTT GGTCTACATA   
  
  
+ ATCACCTATT CAAAAGGAAA TATTGTTCTA TATAATTTTC AGACAGTTTT ATATTCTCTT TATTTATGTA   
  
  
+ TTATTGATGA ATAGTTATGC ACAGGGCATA TCCGGCCGGT GGTTCTTTCA CTATTTTTAT GCATAACAAT   
  
  
+ TGGCCATCAA TGGCTTCTCT TATTTTATAC ATAACAATTG GACATATTGG AAGTGGTCAT CACTATTGTT   
  
  
+ GAGCAGTCAG TCTCAGACTC GATTTTTTAT ACAAACGTAA CCACATAGAG ATTACCCAGC ATAGCTTAAT   
  
  
+ TACCTGCTGG AACCAAACAT GCATGCATAC CTCTTTTTAT TTACGTGTTG TCATTGTCTT TGACTGCTTA   
  
  
+ AGATTCTAAA CAAGTTTAAT AGGTGTTTG  

- ACAAATTAAT AATATTAAGA ACTCCAAAAC ACAAACTTTA CGACTCCTAA AAAGATCAAG ATCTCTCTCT   
  
  
- CTGTCTCTGT CTCTCTTTGT TCTGTACGTG GAGAGTTGGT TTAGCAGGGG GTGTTAAAGT TGCGTTCAGC   
  
  
- ACGATGGAGA GAGAGATTGA GAGAAAGAGA GAGATTTCTT ATCAACTCAA AAACGAGTTC TTCGTTTAAA   
  
  
- CAGGGTGTTG TTGTTATCTA AAGGTGAATG AATATTGTTA ATATACTAGA AACTTCCCTT GCCTCTTCCA   
  
  
- TGCATGCATG ATATATAATG AAGGGTCATG ATATAAAAAG GGCAGTTAAA AAAACGCATG TATATTTCAC   
  
  
- ACAATAACTC TAAACTACTA AAACATTACT AAAATATCCT CCTCGAATGG CAGATGTTAA ATTCTTCTTT   
  
  
- GTTACTAATG CAATTAACCT TACAAATCCG ATTATCATGT ACTAATAACA TGTATGCGTG TACAACATAT   
  
  
- CTATTTATAA ATATAAAGAG GTTCTGTTTT TGGGATTTTA AATAGAAAAA GGTGGTTGAT GTGAGAACTC   
  
  
- AAAGTGTAAA ATTGAGATTA CAACAACAGC ATTTAAATCT GAACATAAAA TATAAGAAAA CAATGTATAT   
  
  
- GTAACATAGT ATGAGTACGT ACGTATATAT CATTTGAATA ATTTGAGTTA AGAAGTACAT AAAATGCATT   
  
  
- TGGTTAGCTC AATAAAACAT ACTCATTATA TAAAACGGGA ATACCATAGT AATGCTGGTA GTTGATCGAT   
  
  
- ACACTAACTT TACTCTTGGT ACTTCTTGAT TAAAGCCTAA TTACGATCCT TCTTGTATAC ATCATTCAAC   
  
  
- TCACTACACA TGATTAACAT ACGTAGTAAA ACATTGTAGT GCACTAGGTT TTTTTACACA ATATGCCAAT   
  
  
- GAGGTTCGTT ACGAGTATTA TACTTATACA TATGTATTTA CCAATTTTAC GCATAATAGG ATTATTAACT   
  
  
- GATATTTTAT TTTATACTCT TATGATATAT ATACATACAC ACACACACAC ACATACACAT CCCAATCACT   
  
  
- ACGAAGTACC GTACACATCC TAATCACTAC GAAATACCGG TGTTTGATCC CGTTCCTAAA CCAGATGTAT   
  
  
- TAGTGGATAA GTTTTCCTTT ATAACAAGAT ATATTAAAAG TCTGTCAAAA TATAAGAGAA ATAAATACAT   
  
  
- AATAACTACT TATCAATACG TGTCCCGTAT AGGCCGGCCA CCAAGAAAGT GATAAAAATA CGTATTGTTA   
  
  
- ACCGGTAGTT ACCGAAGAGA ATAAAATATG TATTGTTAAC CTGTATAACC TTCACCAGTA GTGATAACAA   
  
  
- CTCGTCAGTC AGAGTCTGAG CTAAAAAATA TGTTTGCATT GGTGTATCTC TAATGGGTCG TATCGAATTA   
  
  
- ATGGACGACC TTGGTTTGTA CGTACGTATG GAGAAAAATA AATGCACAAC AGTAACAGAA ACTGACGAAT   
  
  
- TCTAAGATTT GTTCAAATTA TCCACAAAC

+     as-1

| Site Name | Organism | Position | Strand | Matrix score. | sequence | function |
| --- | --- | --- | --- | --- | --- | --- |
| as-1 | Arabidopsis thaliana | 322 | - | 5 | TGACG |  |

>037399   
+ TGTTTAATTA TTATAATTCT TGAGGTTTTG TGTTTGAAAT GCTGAGGATT TTTCTAGTTC TAGAGAGAGA   
  
  
+ GACAGAGACA GAGAGAAACA AGACATGCAC CTCTCAACCA AATCGTCCCC CACAATTTCA ACGCAAGTCG   
  
  
+ TGCTACCTCT CTCTCTAACT CTCTTTCTCT CTCTAAAGAA TAGTTGAGTT TTTGCTCAAG AAGCAAATTT   
  
  
+ GTCCCACAAC AACAATAGAT TTCCACTTAC TTATAACAAT TATATGATCT TTGAAGGGAA CGGAGAAGGT   
  
  
+ ACGTACGTAC TATATATTAC TTCCCAGTAC TATATTTTTC CCGTCAATTT TTTTGCGTAC ATATAAAGTG   
  
  
+ TGTTATTGAG ATTTGATGAT TTTGTAATGA TTTTATAGGA GGAGCTTACC GTCTACAATT TAAGAAGAAA   
  
  
+ CAATGATTAC GTTAATTGGA ATGTTTAGGC TAATAGTACA TGATTATTGT ACATACGCAC ATGTTGTATA   
  
  
+ GATAAATATT TATATTTCTC CAAGACAAAA ACCCTAAAAT TTATCTTTTT CCACCAACTA CACTCTTGAG   
  
  
+ TTTCACATTT TAACTCTAAT GTTGTTGTCG TAAATTTAGA CTTGTATTTT ATATTCTTTT GTTACATATA   
  
  
+ CATTGTATCA TACTCATGCA TGCATATATA GTAAACTTAT TAAACTCAAT TCTTCATGTA TTTTACGTAA   
  
  
+ ACCAATCGAG TTATTTTGTA TGAGTAATAT ATTTTGCCCT TATGGTATCA TTACGACCAT CAACTAGCTA   
  
  
+ TGTGATTGAA ATGAGAACCA TGAAGAACTA ATTTCGGATT AATGCTAGGA AGAACATATG TAGTAAGTTG   
  
  
+ AGTGATGTGT ACTAATTGTA TGCATCATTT TGTAACATCA CGTGATCCAA AAAAATGTGT TATACGGTTA   
  
  
+ CTCCAAGCAA TGCTCATAAT ATGAATATGT ATACATAAAT GGTTAAAATG CGTATTATCC TAATAATTGA   
  
  
+ CTATAAAATA AAATATGAGA ATACTATATA TATGTATGTG TGTGTGTGTG TGTATGTGTA GGGTTAGTGA   
  
  
+ TGCTTCATGG CATGTGTAGG ATTAGTGATG CTTTATGGCC ACAAACTAGG GCAAGGATTT GGTCTACATA   
  
  
+ ATCACCTATT CAAAAGGAAA TATTGTTCTA TATAATTTTC AGACAGTTTT ATATTCTCTT TATTTATGTA   
  
  
+ TTATTGATGA ATAGTTATGC ACAGGGCATA TCCGGCCGGT GGTTCTTTCA CTATTTTTAT GCATAACAAT   
  
  
+ TGGCCATCAA TGGCTTCTCT TATTTTATAC ATAACAATTG GACATATTGG AAGTGGTCAT CACTATTGTT   
  
  
+ GAGCAGTCAG TCTCAGACTC GATTTTTTAT ACAAACGTAA CCACATAGAG ATTACCCAGC ATAGCTTAAT   
  
  
+ TACCTGCTGG AACCAAACAT GCATGCATAC CTCTTTTTAT TTACGTGTTG TCATTGTCTT TGACTGCTTA   
  
  
+ AGATTCTAAA CAAGTTTAAT AGGTGTTTG  

- ACAAATTAAT AATATTAAGA ACTCCAAAAC ACAAACTTTA CGACTCCTAA AAAGATCAAG ATCTCTCTCT   
  
  
- CTGTCTCTGT CTCTCTTTGT TCTGTACGTG GAGAGTTGGT TTAGCAGGGG GTGTTAAAGT TGCGTTCAGC   
  
  
- ACGATGGAGA GAGAGATTGA GAGAAAGAGA GAGATTTCTT ATCAACTCAA AAACGAGTTC TTCGTTTAAA   
  
  
- CAGGGTGTTG TTGTTATCTA AAGGTGAATG AATATTGTTA ATATACTAGA AACTTCCCTT GCCTCTTCCA   
  
  
- TGCATGCATG ATATATAATG AAGGGTCATG ATATAAAAAG GGCAGTTAAA AAAACGCATG TATATTTCAC   
  
  
- ACAATAACTC TAAACTACTA AAACATTACT AAAATATCCT CCTCGAATGG CAGATGTTAA ATTCTTCTTT   
  
  
- GTTACTAATG CAATTAACCT TACAAATCCG ATTATCATGT ACTAATAACA TGTATGCGTG TACAACATAT   
  
  
- CTATTTATAA ATATAAAGAG GTTCTGTTTT TGGGATTTTA AATAGAAAAA GGTGGTTGAT GTGAGAACTC   
  
  
- AAAGTGTAAA ATTGAGATTA CAACAACAGC ATTTAAATCT GAACATAAAA TATAAGAAAA CAATGTATAT   
  
  
- GTAACATAGT ATGAGTACGT ACGTATATAT CATTTGAATA ATTTGAGTTA AGAAGTACAT AAAATGCATT   
  
  
- TGGTTAGCTC AATAAAACAT ACTCATTATA TAAAACGGGA ATACCATAGT AATGCTGGTA GTTGATCGAT   
  
  
- ACACTAACTT TACTCTTGGT ACTTCTTGAT TAAAGCCTAA TTACGATCCT TCTTGTATAC ATCATTCAAC   
  
  
- TCACTACACA TGATTAACAT ACGTAGTAAA ACATTGTAGT GCACTAGGTT TTTTTACACA ATATGCCAAT   
  
  
- GAGGTTCGTT ACGAGTATTA TACTTATACA TATGTATTTA CCAATTTTAC GCATAATAGG ATTATTAACT   
  
  
- GATATTTTAT TTTATACTCT TATGATATAT ATACATACAC ACACACACAC ACATACACAT CCCAATCACT   
  
  
- ACGAAGTACC GTACACATCC TAATCACTAC GAAATACCGG TGTTTGATCC CGTTCCTAAA CCAGATGTAT   
  
  
- TAGTGGATAA GTTTTCCTTT ATAACAAGAT ATATTAAAAG TCTGTCAAAA TATAAGAGAA ATAAATACAT   
  
  
- AATAACTACT TATCAATACG TGTCCCGTAT AGGCCGGCCA CCAAGAAAGT GATAAAAATA CGTATTGTTA   
  
  
- ACCGGTAGTT ACCGAAGAGA ATAAAATATG TATTGTTAAC CTGTATAACC TTCACCAGTA GTGATAACAA   
  
  
- CTCGTCAGTC AGAGTCTGAG CTAAAAAATA TGTTTGCATT GGTGTATCTC TAATGGGTCG TATCGAATTA   
  
  
- ATGGACGACC TTGGTTTGTA CGTACGTATG GAGAAAAATA AATGCACAAC AGTAACAGAA ACTGACGAAT   
  
  
- TCTAAGATTT GTTCAAATTA TCCACAAAC
