# Supplementary material for: In Silico Genome-Wide Analysis of Respiratory Burst Oxidase Homolog (RBOH) Family Genes in Five Fruit-Producing Trees, and Potential Functional Analysis on Lignification of Stone Cells in Chinese White Pear
Source: Cells. 2019 May 29;8(6):520. doi: 10.3390/cells8060520 (PMC6627160; doi:10.3390/cells8060520)
Supplement: Supplementary file 1 [file cells-08-00520-s001.zip › Supplementary Material/Spreadsheets S2. Numbers of cis-elements in promoter region of PbRBOHs.tar.zip!/PbRBOHB.html]

Content-Type: text/html; charset=ISO-8859-1


PlantCARE


Webmaster Firefox specific output  
To save the result:
click on the frame with the right mouse button and save the source code as a text file with extension .html  
REFERENCE:PlantCARE: a database of plant cis-acting regulatory elements and a portal to tools for in silico analysis of promoter sequences.  
Lescot, M., Déhais, P., Moreau, Y., De Moor, B., Rouzé ,P.,and Rombauts, S.  
Nucleic Acids Res., Database issue(2002), 30(1):325-327.   


---

>037815   
+ TTTCAGATGC TTCTCACAGA TATTCAAAGT TTGGTTGATT CAACTATTCA ACAAGTAGGA AACTAATTAG   
  
  
+ CACAAAGAAA AACAAATGGG GGGTCTGAAG ATATTTTGGG ATTAAAATAC GAGGGGTGGA TGGGTATATG   
  
  
+ TAATGTAAGA GAGGAGGAAG GTAGCATGCA GTAGCAGAAG CGATAAAAAA ACACGACAGT GAAAAGGAAC   
  
  
+ ACGAAGACGA CTTGGAATAC GGTTGGGAAG GGAATATGAG TGTTACATCG CATGTTTTTA TACAGTGGTG   
  
  
+ AGATATTTTA TTTTTAAGTT ATTAACTTTT TAACCCACAT ATCTTACCAG TAATATAATG ATACGTAGTG   
  
  
+ TACTATCTCA TATGTCGGTC ACATTAAAAA AAATTTCTCA ATTATCATTT GATGTTATAT TTGACACGTC   
  
  
+ AATTCGTCTC TTTGATTAAT AATTATATAA ATTATTTATC ACATCATATA ATACACTGTA CACACCAATA   
  
  
+ TAATATAAAC ATAAACATGT GATTTTCCTG ATATTACTAT GAATGAAATT ATATACAATA TTATTTATAA   
  
  
+ TGTTGTCCTC AAGGTGGAGA TTTCGAGTTC AAATTATACA TATACAAGAG ATTCATGCAA GTATTCAATA   
  
  
+ TTAAATTGTG ATCACCCCTT AATCATTTGT GAAAAAGAAA AAGTTAACTT GCCAATCTCA TTCCTCTTAA   
  
  
+ TAATTAACAA TTTAGCTTAT TAATGCTATC ATTTGTTAAA AATTTAAAAT ATGAGAACTT TTATGATACT   
  
  
+ TTTTTATAGA AGTTAATAGT ATCTCGTTTT AATAAAATTA TTTGCGGACG TTATGTAGTT ACTCATCATC   
  
  
+ ATTATTTGCA TTCGGAAATC CTATCTTCAC GTTTAACGAT TTTACAACAT GATGTATGAA TTAGAAGAAA   
  
  
+ AATAATGTTA TTACGCAAGA ATTCTTCTCT CTCCAAACCT CTATAACTCT CCCTCCCCTC TCTTTCATTT   
  
  
+ GGTGCAGGTG GTGGCGCGAA GATGTCCTTT TAGCAGAGGT GGGGTGGCTA AAAGAAATAG GGGTACTGCG   
  
  
+ TTTTGGATTC TGGGTTGTGA GTTCTGGGTT CTAAATTTAT TCGTTAACTC CAGCTTCTCG TCCTACTTCG   
  
  
+ GGTTCAGATC TTGCTGTTTC GTCTTCATCC TCTGTCATTG CCTGTCAAAG TCGACGATTG CGTTGGTCCT   
  
  
+ CGTCAGGTTC AGATCTCACT CCTCATTTTC CCTTCGGATT CCTATTTTTT CAACTTTCTG AAATTTATGC   
  
  
+ CAATCTTTTT CGATATTTTT CTGGGTTTTG TATGAACATA TGTGGGTTGA AGTTGAAGAG GGGAGGGAGA   
  
  
+ GTTATAGAGG TTTGGAGAGA GAAGAAGATG AAAGAGAGTG GGGAAGAACG AGGAGGGGGA AAAGAAAGAT   
  
  
+ ACTTTCATGG TTTAGGGAGT GTGTGAAAGT TTGAGAAGGT GTTGGGTGAG AGTGAGAGGG AGAGAGTGTT   
  
  
+ GCGGCAGCTA TGGGTGGATC TGGGTTCTT  

- AAAGTCTACG AAGAGTGTCT ATAAGTTTCA AACCAACTAA GTTGATAAGT TGTTCATCCT TTGATTAATC   
  
  
- GTGTTTCTTT TTGTTTACCC CCCAGACTTC TATAAAACCC TAATTTTATG CTCCCCACCT ACCCATATAC   
  
  
- ATTACATTCT CTCCTCCTTC CATCGTACGT CATCGTCTTC GCTATTTTTT TGTGCTGTCA CTTTTCCTTG   
  
  
- TGCTTCTGCT GAACCTTATG CCAACCCTTC CCTTATACTC ACAATGTAGC GTACAAAAAT ATGTCACCAC   
  
  
- TCTATAAAAT AAAAATTCAA TAATTGAAAA ATTGGGTGTA TAGAATGGTC ATTATATTAC TATGCATCAC   
  
  
- ATGATAGAGT ATACAGCCAG TGTAATTTTT TTTAAAGAGT TAATAGTAAA CTACAATATA AACTGTGCAG   
  
  
- TTAAGCAGAG AAACTAATTA TTAATATATT TAATAAATAG TGTAGTATAT TATGTGACAT GTGTGGTTAT   
  
  
- ATTATATTTG TATTTGTACA CTAAAAGGAC TATAATGATA CTTACTTTAA TATATGTTAT AATAAATATT   
  
  
- ACAACAGGAG TTCCACCTCT AAAGCTCAAG TTTAATATGT ATATGTTCTC TAAGTACGTT CATAAGTTAT   
  
  
- AATTTAACAC TAGTGGGGAA TTAGTAAACA CTTTTTCTTT TTCAATTGAA CGGTTAGAGT AAGGAGAATT   
  
  
- ATTAATTGTT AAATCGAATA ATTACGATAG TAAACAATTT TTAAATTTTA TACTCTTGAA AATACTATGA   
  
  
- AAAAATATCT TCAATTATCA TAGAGCAAAA TTATTTTAAT AAACGCCTGC AATACATCAA TGAGTAGTAG   
  
  
- TAATAAACGT AAGCCTTTAG GATAGAAGTG CAAATTGCTA AAATGTTGTA CTACATACTT AATCTTCTTT   
  
  
- TTATTACAAT AATGCGTTCT TAAGAAGAGA GAGGTTTGGA GATATTGAGA GGGAGGGGAG AGAAAGTAAA   
  
  
- CCACGTCCAC CACCGCGCTT CTACAGGAAA ATCGTCTCCA CCCCACCGAT TTTCTTTATC CCCATGACGC   
  
  
- AAAACCTAAG ACCCAACACT CAAGACCCAA GATTTAAATA AGCAATTGAG GTCGAAGAGC AGGATGAAGC   
  
  
- CCAAGTCTAG AACGACAAAG CAGAAGTAGG AGACAGTAAC GGACAGTTTC AGCTGCTAAC GCAACCAGGA   
  
  
- GCAGTCCAAG TCTAGAGTGA GGAGTAAAAG GGAAGCCTAA GGATAAAAAA GTTGAAAGAC TTTAAATACG   
  
  
- GTTAGAAAAA GCTATAAAAA GACCCAAAAC ATACTTGTAT ACACCCAACT TCAACTTCTC CCCTCCCTCT   
  
  
- CAATATCTCC AAACCTCTCT CTTCTTCTAC TTTCTCTCAC CCCTTCTTGC TCCTCCCCCT TTTCTTTCTA   
  
  
- TGAAAGTACC AAATCCCTCA CACACTTTCA AACTCTTCCA CAACCCACTC TCACTCTCCC TCTCTCACAA   
  
  
- CGCCGTCGAT ACCCACCTAG ACCCAAGAA

  
  
Motifs Found  

+   

| Site Name | Organism | Position | Strand | Matrix score. | sequence | function |
| --- | --- | --- | --- | --- | --- | --- |
|  | organism | 1354 | - | 4 | motif\_sequence | short\_function |
|  | organism | 1294 | - | 4 | motif\_sequence | short\_function |
|  | organism | 97 | - | 4 | motif\_sequence | short\_function |
|  | organism | 957 | + | 4 | motif\_sequence | short\_function |
|  | organism | 998 | - | 4 | motif\_sequence | short\_function |
|  | organism | 1002 | + | 4 | motif\_sequence | short\_function |
|  | organism | 939 | + | 4 | motif\_sequence | short\_function |
|  | organism | 576 | - | 4 | motif\_sequence | short\_function |
|  | organism | 476 | + | 4 | motif\_sequence | short\_function |
|  | organism | 1459 | - | 4 | motif\_sequence | short\_function |
|  | organism | 863 | + | 4 | motif\_sequence | short\_function |
|  | organism | 1315 | - | 4 | motif\_sequence | short\_function |
|  | organism | 1344 | - | 4 | motif\_sequence | short\_function |
|  | organism | 1013 | - | 4 | motif\_sequence | short\_function |
|  | organism | 1326 | - | 4 | motif\_sequence | short\_function |

>037815   
+ TTTCAGATGC TTCTCACAGA TATTCAAAGT TTGGTTGATT CAACTATTCA ACAAGTAGGA AACTAATTAG   
  
  
+ CACAAAGAAA AACAAATGGG GGGTCTGAAG ATATTTTGGG ATTAAAATAC GAGGGGTGGA TGGGTATATG   
  
  
+ TAATGTAAGA GAGGAGGAAG GTAGCATGCA GTAGCAGAAG CGATAAAAAA ACACGACAGT GAAAAGGAAC   
  
  
+ ACGAAGACGA CTTGGAATAC GGTTGGGAAG GGAATATGAG TGTTACATCG CATGTTTTTA TACAGTGGTG   
  
  
+ AGATATTTTA TTTTTAAGTT ATTAACTTTT TAACCCACAT ATCTTACCAG TAATATAATG ATACGTAGTG   
  
  
+ TACTATCTCA TATGTCGGTC ACATTAAAAA AAATTTCTCA ATTATCATTT GATGTTATAT TTGACACGTC   
  
  
+ AATTCGTCTC TTTGATTAAT AATTATATAA ATTATTTATC ACATCATATA ATACACTGTA CACACCAATA   
  
  
+ TAATATAAAC ATAAACATGT GATTTTCCTG ATATTACTAT GAATGAAATT ATATACAATA TTATTTATAA   
  
  
+ TGTTGTCCTC AAGGTGGAGA TTTCGAGTTC AAATTATACA TATACAAGAG ATTCATGCAA GTATTCAATA   
  
  
+ TTAAATTGTG ATCACCCCTT AATCATTTGT GAAAAAGAAA AAGTTAACTT GCCAATCTCA TTCCTCTTAA   
  
  
+ TAATTAACAA TTTAGCTTAT TAATGCTATC ATTTGTTAAA AATTTAAAAT ATGAGAACTT TTATGATACT   
  
  
+ TTTTTATAGA AGTTAATAGT ATCTCGTTTT AATAAAATTA TTTGCGGACG TTATGTAGTT ACTCATCATC   
  
  
+ ATTATTTGCA TTCGGAAATC CTATCTTCAC GTTTAACGAT TTTACAACAT GATGTATGAA TTAGAAGAAA   
  
  
+ AATAATGTTA TTACGCAAGA ATTCTTCTCT CTCCAAACCT CTATAACTCT CCCTCCCCTC TCTTTCATTT   
  
  
+ GGTGCAGGTG GTGGCGCGAA GATGTCCTTT TAGCAGAGGT GGGGTGGCTA AAAGAAATAG GGGTACTGCG   
  
  
+ TTTTGGATTC TGGGTTGTGA GTTCTGGGTT CTAAATTTAT TCGTTAACTC CAGCTTCTCG TCCTACTTCG   
  
  
+ GGTTCAGATC TTGCTGTTTC GTCTTCATCC TCTGTCATTG CCTGTCAAAG TCGACGATTG CGTTGGTCCT   
  
  
+ CGTCAGGTTC AGATCTCACT CCTCATTTTC CCTTCGGATT CCTATTTTTT CAACTTTCTG AAATTTATGC   
  
  
+ CAATCTTTTT CGATATTTTT CTGGGTTTTG TATGAACATA TGTGGGTTGA AGTTGAAGAG GGGAGGGAGA   
  
  
+ GTTATAGAGG TTTGGAGAGA GAAGAAGATG AAAGAGAGTG GGGAAGAACG AGGAGGGGGA AAAGAAAGAT   
  
  
+ ACTTTCATGG TTTAGGGAGT GTGTGAAAGT TTGAGAAGGT GTTGGGTGAG AGTGAGAGGG AGAGAGTGTT   
  
  
+ GCGGCAGCTA TGGGTGGATC TGGGTTCTT  

- AAAGTCTACG AAGAGTGTCT ATAAGTTTCA AACCAACTAA GTTGATAAGT TGTTCATCCT TTGATTAATC   
  
  
- GTGTTTCTTT TTGTTTACCC CCCAGACTTC TATAAAACCC TAATTTTATG CTCCCCACCT ACCCATATAC   
  
  
- ATTACATTCT CTCCTCCTTC CATCGTACGT CATCGTCTTC GCTATTTTTT TGTGCTGTCA CTTTTCCTTG   
  
  
- TGCTTCTGCT GAACCTTATG CCAACCCTTC CCTTATACTC ACAATGTAGC GTACAAAAAT ATGTCACCAC   
  
  
- TCTATAAAAT AAAAATTCAA TAATTGAAAA ATTGGGTGTA TAGAATGGTC ATTATATTAC TATGCATCAC   
  
  
- ATGATAGAGT ATACAGCCAG TGTAATTTTT TTTAAAGAGT TAATAGTAAA CTACAATATA AACTGTGCAG   
  
  
- TTAAGCAGAG AAACTAATTA TTAATATATT TAATAAATAG TGTAGTATAT TATGTGACAT GTGTGGTTAT   
  
  
- ATTATATTTG TATTTGTACA CTAAAAGGAC TATAATGATA CTTACTTTAA TATATGTTAT AATAAATATT   
  
  
- ACAACAGGAG TTCCACCTCT AAAGCTCAAG TTTAATATGT ATATGTTCTC TAAGTACGTT CATAAGTTAT   
  
  
- AATTTAACAC TAGTGGGGAA TTAGTAAACA CTTTTTCTTT TTCAATTGAA CGGTTAGAGT AAGGAGAATT   
  
  
- ATTAATTGTT AAATCGAATA ATTACGATAG TAAACAATTT TTAAATTTTA TACTCTTGAA AATACTATGA   
  
  
- AAAAATATCT TCAATTATCA TAGAGCAAAA TTATTTTAAT AAACGCCTGC AATACATCAA TGAGTAGTAG   
  
  
- TAATAAACGT AAGCCTTTAG GATAGAAGTG CAAATTGCTA AAATGTTGTA CTACATACTT AATCTTCTTT   
  
  
- TTATTACAAT AATGCGTTCT TAAGAAGAGA GAGGTTTGGA GATATTGAGA GGGAGGGGAG AGAAAGTAAA   
  
  
- CCACGTCCAC CACCGCGCTT CTACAGGAAA ATCGTCTCCA CCCCACCGAT TTTCTTTATC CCCATGACGC   
  
  
- AAAACCTAAG ACCCAACACT CAAGACCCAA GATTTAAATA AGCAATTGAG GTCGAAGAGC AGGATGAAGC   
  
  
- CCAAGTCTAG AACGACAAAG CAGAAGTAGG AGACAGTAAC GGACAGTTTC AGCTGCTAAC GCAACCAGGA   
  
  
- GCAGTCCAAG TCTAGAGTGA GGAGTAAAAG GGAAGCCTAA GGATAAAAAA GTTGAAAGAC TTTAAATACG   
  
  
- GTTAGAAAAA GCTATAAAAA GACCCAAAAC ATACTTGTAT ACACCCAACT TCAACTTCTC CCCTCCCTCT   
  
  
- CAATATCTCC AAACCTCTCT CTTCTTCTAC TTTCTCTCAC CCCTTCTTGC TCCTCCCCCT TTTCTTTCTA   
  
  
- TGAAAGTACC AAATCCCTCA CACACTTTCA AACTCTTCCA CAACCCACTC TCACTCTCCC TCTCTCACAA   
  
  
- CGCCGTCGAT ACCCACCTAG ACCCAAGAA

+     3-AF1 binding site

| Site Name | Organism | Position | Strand | Matrix score. | sequence | function |
| --- | --- | --- | --- | --- | --- | --- |
| 3-AF1 binding site | Solanum tuberosum | 146 | + | 10 | TAAGAGAGGAA | light responsive element |

>037815   
+ TTTCAGATGC TTCTCACAGA TATTCAAAGT TTGGTTGATT CAACTATTCA ACAAGTAGGA AACTAATTAG   
  
  
+ CACAAAGAAA AACAAATGGG GGGTCTGAAG ATATTTTGGG ATTAAAATAC GAGGGGTGGA TGGGTATATG   
  
  
+ TAATGTAAGA GAGGAGGAAG GTAGCATGCA GTAGCAGAAG CGATAAAAAA ACACGACAGT GAAAAGGAAC   
  
  
+ ACGAAGACGA CTTGGAATAC GGTTGGGAAG GGAATATGAG TGTTACATCG CATGTTTTTA TACAGTGGTG   
  
  
+ AGATATTTTA TTTTTAAGTT ATTAACTTTT TAACCCACAT ATCTTACCAG TAATATAATG ATACGTAGTG   
  
  
+ TACTATCTCA TATGTCGGTC ACATTAAAAA AAATTTCTCA ATTATCATTT GATGTTATAT TTGACACGTC   
  
  
+ AATTCGTCTC TTTGATTAAT AATTATATAA ATTATTTATC ACATCATATA ATACACTGTA CACACCAATA   
  
  
+ TAATATAAAC ATAAACATGT GATTTTCCTG ATATTACTAT GAATGAAATT ATATACAATA TTATTTATAA   
  
  
+ TGTTGTCCTC AAGGTGGAGA TTTCGAGTTC AAATTATACA TATACAAGAG ATTCATGCAA GTATTCAATA   
  
  
+ TTAAATTGTG ATCACCCCTT AATCATTTGT GAAAAAGAAA AAGTTAACTT GCCAATCTCA TTCCTCTTAA   
  
  
+ TAATTAACAA TTTAGCTTAT TAATGCTATC ATTTGTTAAA AATTTAAAAT ATGAGAACTT TTATGATACT   
  
  
+ TTTTTATAGA AGTTAATAGT ATCTCGTTTT AATAAAATTA TTTGCGGACG TTATGTAGTT ACTCATCATC   
  
  
+ ATTATTTGCA TTCGGAAATC CTATCTTCAC GTTTAACGAT TTTACAACAT GATGTATGAA TTAGAAGAAA   
  
  
+ AATAATGTTA TTACGCAAGA ATTCTTCTCT CTCCAAACCT CTATAACTCT CCCTCCCCTC TCTTTCATTT   
  
  
+ GGTGCAGGTG GTGGCGCGAA GATGTCCTTT TAGCAGAGGT GGGGTGGCTA AAAGAAATAG GGGTACTGCG   
  
  
+ TTTTGGATTC TGGGTTGTGA GTTCTGGGTT CTAAATTTAT TCGTTAACTC CAGCTTCTCG TCCTACTTCG   
  
  
+ GGTTCAGATC TTGCTGTTTC GTCTTCATCC TCTGTCATTG CCTGTCAAAG TCGACGATTG CGTTGGTCCT   
  
  
+ CGTCAGGTTC AGATCTCACT CCTCATTTTC CCTTCGGATT CCTATTTTTT CAACTTTCTG AAATTTATGC   
  
  
+ CAATCTTTTT CGATATTTTT CTGGGTTTTG TATGAACATA TGTGGGTTGA AGTTGAAGAG GGGAGGGAGA   
  
  
+ GTTATAGAGG TTTGGAGAGA GAAGAAGATG AAAGAGAGTG GGGAAGAACG AGGAGGGGGA AAAGAAAGAT   
  
  
+ ACTTTCATGG TTTAGGGAGT GTGTGAAAGT TTGAGAAGGT GTTGGGTGAG AGTGAGAGGG AGAGAGTGTT   
  
  
+ GCGGCAGCTA TGGGTGGATC TGGGTTCTT  

- AAAGTCTACG AAGAGTGTCT ATAAGTTTCA AACCAACTAA GTTGATAAGT TGTTCATCCT TTGATTAATC   
  
  
- GTGTTTCTTT TTGTTTACCC CCCAGACTTC TATAAAACCC TAATTTTATG CTCCCCACCT ACCCATATAC   
  
  
- ATTACATTCT CTCCTCCTTC CATCGTACGT CATCGTCTTC GCTATTTTTT TGTGCTGTCA CTTTTCCTTG   
  
  
- TGCTTCTGCT GAACCTTATG CCAACCCTTC CCTTATACTC ACAATGTAGC GTACAAAAAT ATGTCACCAC   
  
  
- TCTATAAAAT AAAAATTCAA TAATTGAAAA ATTGGGTGTA TAGAATGGTC ATTATATTAC TATGCATCAC   
  
  
- ATGATAGAGT ATACAGCCAG TGTAATTTTT TTTAAAGAGT TAATAGTAAA CTACAATATA AACTGTGCAG   
  
  
- TTAAGCAGAG AAACTAATTA TTAATATATT TAATAAATAG TGTAGTATAT TATGTGACAT GTGTGGTTAT   
  
  
- ATTATATTTG TATTTGTACA CTAAAAGGAC TATAATGATA CTTACTTTAA TATATGTTAT AATAAATATT   
  
  
- ACAACAGGAG TTCCACCTCT AAAGCTCAAG TTTAATATGT ATATGTTCTC TAAGTACGTT CATAAGTTAT   
  
  
- AATTTAACAC TAGTGGGGAA TTAGTAAACA CTTTTTCTTT TTCAATTGAA CGGTTAGAGT AAGGAGAATT   
  
  
- ATTAATTGTT AAATCGAATA ATTACGATAG TAAACAATTT TTAAATTTTA TACTCTTGAA AATACTATGA   
  
  
- AAAAATATCT TCAATTATCA TAGAGCAAAA TTATTTTAAT AAACGCCTGC AATACATCAA TGAGTAGTAG   
  
  
- TAATAAACGT AAGCCTTTAG GATAGAAGTG CAAATTGCTA AAATGTTGTA CTACATACTT AATCTTCTTT   
  
  
- TTATTACAAT AATGCGTTCT TAAGAAGAGA GAGGTTTGGA GATATTGAGA GGGAGGGGAG AGAAAGTAAA   
  
  
- CCACGTCCAC CACCGCGCTT CTACAGGAAA ATCGTCTCCA CCCCACCGAT TTTCTTTATC CCCATGACGC   
  
  
- AAAACCTAAG ACCCAACACT CAAGACCCAA GATTTAAATA AGCAATTGAG GTCGAAGAGC AGGATGAAGC   
  
  
- CCAAGTCTAG AACGACAAAG CAGAAGTAGG AGACAGTAAC GGACAGTTTC AGCTGCTAAC GCAACCAGGA   
  
  
- GCAGTCCAAG TCTAGAGTGA GGAGTAAAAG GGAAGCCTAA GGATAAAAAA GTTGAAAGAC TTTAAATACG   
  
  
- GTTAGAAAAA GCTATAAAAA GACCCAAAAC ATACTTGTAT ACACCCAACT TCAACTTCTC CCCTCCCTCT   
  
  
- CAATATCTCC AAACCTCTCT CTTCTTCTAC TTTCTCTCAC CCCTTCTTGC TCCTCCCCCT TTTCTTTCTA   
  
  
- TGAAAGTACC AAATCCCTCA CACACTTTCA AACTCTTCCA CAACCCACTC TCACTCTCCC TCTCTCACAA   
  
  
- CGCCGTCGAT ACCCACCTAG ACCCAAGAA

+     AAGAA-motif

| Site Name | Organism | Position | Strand | Matrix score. | sequence | function |
| --- | --- | --- | --- | --- | --- | --- |
| AAGAA-motif | Avena sativa | 1388 | + | 9 | gGTAAAGAAA |  |

>037815   
+ TTTCAGATGC TTCTCACAGA TATTCAAAGT TTGGTTGATT CAACTATTCA ACAAGTAGGA AACTAATTAG   
  
  
+ CACAAAGAAA AACAAATGGG GGGTCTGAAG ATATTTTGGG ATTAAAATAC GAGGGGTGGA TGGGTATATG   
  
  
+ TAATGTAAGA GAGGAGGAAG GTAGCATGCA GTAGCAGAAG CGATAAAAAA ACACGACAGT GAAAAGGAAC   
  
  
+ ACGAAGACGA CTTGGAATAC GGTTGGGAAG GGAATATGAG TGTTACATCG CATGTTTTTA TACAGTGGTG   
  
  
+ AGATATTTTA TTTTTAAGTT ATTAACTTTT TAACCCACAT ATCTTACCAG TAATATAATG ATACGTAGTG   
  
  
+ TACTATCTCA TATGTCGGTC ACATTAAAAA AAATTTCTCA ATTATCATTT GATGTTATAT TTGACACGTC   
  
  
+ AATTCGTCTC TTTGATTAAT AATTATATAA ATTATTTATC ACATCATATA ATACACTGTA CACACCAATA   
  
  
+ TAATATAAAC ATAAACATGT GATTTTCCTG ATATTACTAT GAATGAAATT ATATACAATA TTATTTATAA   
  
  
+ TGTTGTCCTC AAGGTGGAGA TTTCGAGTTC AAATTATACA TATACAAGAG ATTCATGCAA GTATTCAATA   
  
  
+ TTAAATTGTG ATCACCCCTT AATCATTTGT GAAAAAGAAA AAGTTAACTT GCCAATCTCA TTCCTCTTAA   
  
  
+ TAATTAACAA TTTAGCTTAT TAATGCTATC ATTTGTTAAA AATTTAAAAT ATGAGAACTT TTATGATACT   
  
  
+ TTTTTATAGA AGTTAATAGT ATCTCGTTTT AATAAAATTA TTTGCGGACG TTATGTAGTT ACTCATCATC   
  
  
+ ATTATTTGCA TTCGGAAATC CTATCTTCAC GTTTAACGAT TTTACAACAT GATGTATGAA TTAGAAGAAA   
  
  
+ AATAATGTTA TTACGCAAGA ATTCTTCTCT CTCCAAACCT CTATAACTCT CCCTCCCCTC TCTTTCATTT   
  
  
+ GGTGCAGGTG GTGGCGCGAA GATGTCCTTT TAGCAGAGGT GGGGTGGCTA AAAGAAATAG GGGTACTGCG   
  
  
+ TTTTGGATTC TGGGTTGTGA GTTCTGGGTT CTAAATTTAT TCGTTAACTC CAGCTTCTCG TCCTACTTCG   
  
  
+ GGTTCAGATC TTGCTGTTTC GTCTTCATCC TCTGTCATTG CCTGTCAAAG TCGACGATTG CGTTGGTCCT   
  
  
+ CGTCAGGTTC AGATCTCACT CCTCATTTTC CCTTCGGATT CCTATTTTTT CAACTTTCTG AAATTTATGC   
  
  
+ CAATCTTTTT CGATATTTTT CTGGGTTTTG TATGAACATA TGTGGGTTGA AGTTGAAGAG GGGAGGGAGA   
  
  
+ GTTATAGAGG TTTGGAGAGA GAAGAAGATG AAAGAGAGTG GGGAAGAACG AGGAGGGGGA AAAGAAAGAT   
  
  
+ ACTTTCATGG TTTAGGGAGT GTGTGAAAGT TTGAGAAGGT GTTGGGTGAG AGTGAGAGGG AGAGAGTGTT   
  
  
+ GCGGCAGCTA TGGGTGGATC TGGGTTCTT  

- AAAGTCTACG AAGAGTGTCT ATAAGTTTCA AACCAACTAA GTTGATAAGT TGTTCATCCT TTGATTAATC   
  
  
- GTGTTTCTTT TTGTTTACCC CCCAGACTTC TATAAAACCC TAATTTTATG CTCCCCACCT ACCCATATAC   
  
  
- ATTACATTCT CTCCTCCTTC CATCGTACGT CATCGTCTTC GCTATTTTTT TGTGCTGTCA CTTTTCCTTG   
  
  
- TGCTTCTGCT GAACCTTATG CCAACCCTTC CCTTATACTC ACAATGTAGC GTACAAAAAT ATGTCACCAC   
  
  
- TCTATAAAAT AAAAATTCAA TAATTGAAAA ATTGGGTGTA TAGAATGGTC ATTATATTAC TATGCATCAC   
  
  
- ATGATAGAGT ATACAGCCAG TGTAATTTTT TTTAAAGAGT TAATAGTAAA CTACAATATA AACTGTGCAG   
  
  
- TTAAGCAGAG AAACTAATTA TTAATATATT TAATAAATAG TGTAGTATAT TATGTGACAT GTGTGGTTAT   
  
  
- ATTATATTTG TATTTGTACA CTAAAAGGAC TATAATGATA CTTACTTTAA TATATGTTAT AATAAATATT   
  
  
- ACAACAGGAG TTCCACCTCT AAAGCTCAAG TTTAATATGT ATATGTTCTC TAAGTACGTT CATAAGTTAT   
  
  
- AATTTAACAC TAGTGGGGAA TTAGTAAACA CTTTTTCTTT TTCAATTGAA CGGTTAGAGT AAGGAGAATT   
  
  
- ATTAATTGTT AAATCGAATA ATTACGATAG TAAACAATTT TTAAATTTTA TACTCTTGAA AATACTATGA   
  
  
- AAAAATATCT TCAATTATCA TAGAGCAAAA TTATTTTAAT AAACGCCTGC AATACATCAA TGAGTAGTAG   
  
  
- TAATAAACGT AAGCCTTTAG GATAGAAGTG CAAATTGCTA AAATGTTGTA CTACATACTT AATCTTCTTT   
  
  
- TTATTACAAT AATGCGTTCT TAAGAAGAGA GAGGTTTGGA GATATTGAGA GGGAGGGGAG AGAAAGTAAA   
  
  
- CCACGTCCAC CACCGCGCTT CTACAGGAAA ATCGTCTCCA CCCCACCGAT TTTCTTTATC CCCATGACGC   
  
  
- AAAACCTAAG ACCCAACACT CAAGACCCAA GATTTAAATA AGCAATTGAG GTCGAAGAGC AGGATGAAGC   
  
  
- CCAAGTCTAG AACGACAAAG CAGAAGTAGG AGACAGTAAC GGACAGTTTC AGCTGCTAAC GCAACCAGGA   
  
  
- GCAGTCCAAG TCTAGAGTGA GGAGTAAAAG GGAAGCCTAA GGATAAAAAA GTTGAAAGAC TTTAAATACG   
  
  
- GTTAGAAAAA GCTATAAAAA GACCCAAAAC ATACTTGTAT ACACCCAACT TCAACTTCTC CCCTCCCTCT   
  
  
- CAATATCTCC AAACCTCTCT CTTCTTCTAC TTTCTCTCAC CCCTTCTTGC TCCTCCCCCT TTTCTTTCTA   
  
  
- TGAAAGTACC AAATCCCTCA CACACTTTCA AACTCTTCCA CAACCCACTC TCACTCTCCC TCTCTCACAA   
  
  
- CGCCGTCGAT ACCCACCTAG ACCCAAGAA

+     ABRE

| Site Name | Organism | Position | Strand | Matrix score. | sequence | function |
| --- | --- | --- | --- | --- | --- | --- |
| ABRE | Arabidopsis thaliana | 415 | - | 5 | ACGTG | cis-acting element involved in the abscisic acid responsiveness |
| ABRE | Arabidopsis thaliana | 868 | - | 5 | ACGTG | cis-acting element involved in the abscisic acid responsiveness |

>037815   
+ TTTCAGATGC TTCTCACAGA TATTCAAAGT TTGGTTGATT CAACTATTCA ACAAGTAGGA AACTAATTAG   
  
  
+ CACAAAGAAA AACAAATGGG GGGTCTGAAG ATATTTTGGG ATTAAAATAC GAGGGGTGGA TGGGTATATG   
  
  
+ TAATGTAAGA GAGGAGGAAG GTAGCATGCA GTAGCAGAAG CGATAAAAAA ACACGACAGT GAAAAGGAAC   
  
  
+ ACGAAGACGA CTTGGAATAC GGTTGGGAAG GGAATATGAG TGTTACATCG CATGTTTTTA TACAGTGGTG   
  
  
+ AGATATTTTA TTTTTAAGTT ATTAACTTTT TAACCCACAT ATCTTACCAG TAATATAATG ATACGTAGTG   
  
  
+ TACTATCTCA TATGTCGGTC ACATTAAAAA AAATTTCTCA ATTATCATTT GATGTTATAT TTGACACGTC   
  
  
+ AATTCGTCTC TTTGATTAAT AATTATATAA ATTATTTATC ACATCATATA ATACACTGTA CACACCAATA   
  
  
+ TAATATAAAC ATAAACATGT GATTTTCCTG ATATTACTAT GAATGAAATT ATATACAATA TTATTTATAA   
  
  
+ TGTTGTCCTC AAGGTGGAGA TTTCGAGTTC AAATTATACA TATACAAGAG ATTCATGCAA GTATTCAATA   
  
  
+ TTAAATTGTG ATCACCCCTT AATCATTTGT GAAAAAGAAA AAGTTAACTT GCCAATCTCA TTCCTCTTAA   
  
  
+ TAATTAACAA TTTAGCTTAT TAATGCTATC ATTTGTTAAA AATTTAAAAT ATGAGAACTT TTATGATACT   
  
  
+ TTTTTATAGA AGTTAATAGT ATCTCGTTTT AATAAAATTA TTTGCGGACG TTATGTAGTT ACTCATCATC   
  
  
+ ATTATTTGCA TTCGGAAATC CTATCTTCAC GTTTAACGAT TTTACAACAT GATGTATGAA TTAGAAGAAA   
  
  
+ AATAATGTTA TTACGCAAGA ATTCTTCTCT CTCCAAACCT CTATAACTCT CCCTCCCCTC TCTTTCATTT   
  
  
+ GGTGCAGGTG GTGGCGCGAA GATGTCCTTT TAGCAGAGGT GGGGTGGCTA AAAGAAATAG GGGTACTGCG   
  
  
+ TTTTGGATTC TGGGTTGTGA GTTCTGGGTT CTAAATTTAT TCGTTAACTC CAGCTTCTCG TCCTACTTCG   
  
  
+ GGTTCAGATC TTGCTGTTTC GTCTTCATCC TCTGTCATTG CCTGTCAAAG TCGACGATTG CGTTGGTCCT   
  
  
+ CGTCAGGTTC AGATCTCACT CCTCATTTTC CCTTCGGATT CCTATTTTTT CAACTTTCTG AAATTTATGC   
  
  
+ CAATCTTTTT CGATATTTTT CTGGGTTTTG TATGAACATA TGTGGGTTGA AGTTGAAGAG GGGAGGGAGA   
  
  
+ GTTATAGAGG TTTGGAGAGA GAAGAAGATG AAAGAGAGTG GGGAAGAACG AGGAGGGGGA AAAGAAAGAT   
  
  
+ ACTTTCATGG TTTAGGGAGT GTGTGAAAGT TTGAGAAGGT GTTGGGTGAG AGTGAGAGGG AGAGAGTGTT   
  
  
+ GCGGCAGCTA TGGGTGGATC TGGGTTCTT  

- AAAGTCTACG AAGAGTGTCT ATAAGTTTCA AACCAACTAA GTTGATAAGT TGTTCATCCT TTGATTAATC   
  
  
- GTGTTTCTTT TTGTTTACCC CCCAGACTTC TATAAAACCC TAATTTTATG CTCCCCACCT ACCCATATAC   
  
  
- ATTACATTCT CTCCTCCTTC CATCGTACGT CATCGTCTTC GCTATTTTTT TGTGCTGTCA CTTTTCCTTG   
  
  
- TGCTTCTGCT GAACCTTATG CCAACCCTTC CCTTATACTC ACAATGTAGC GTACAAAAAT ATGTCACCAC   
  
  
- TCTATAAAAT AAAAATTCAA TAATTGAAAA ATTGGGTGTA TAGAATGGTC ATTATATTAC TATGCATCAC   
  
  
- ATGATAGAGT ATACAGCCAG TGTAATTTTT TTTAAAGAGT TAATAGTAAA CTACAATATA AACTGTGCAG   
  
  
- TTAAGCAGAG AAACTAATTA TTAATATATT TAATAAATAG TGTAGTATAT TATGTGACAT GTGTGGTTAT   
  
  
- ATTATATTTG TATTTGTACA CTAAAAGGAC TATAATGATA CTTACTTTAA TATATGTTAT AATAAATATT   
  
  
- ACAACAGGAG TTCCACCTCT AAAGCTCAAG TTTAATATGT ATATGTTCTC TAAGTACGTT CATAAGTTAT   
  
  
- AATTTAACAC TAGTGGGGAA TTAGTAAACA CTTTTTCTTT TTCAATTGAA CGGTTAGAGT AAGGAGAATT   
  
  
- ATTAATTGTT AAATCGAATA ATTACGATAG TAAACAATTT TTAAATTTTA TACTCTTGAA AATACTATGA   
  
  
- AAAAATATCT TCAATTATCA TAGAGCAAAA TTATTTTAAT AAACGCCTGC AATACATCAA TGAGTAGTAG   
  
  
- TAATAAACGT AAGCCTTTAG GATAGAAGTG CAAATTGCTA AAATGTTGTA CTACATACTT AATCTTCTTT   
  
  
- TTATTACAAT AATGCGTTCT TAAGAAGAGA GAGGTTTGGA GATATTGAGA GGGAGGGGAG AGAAAGTAAA   
  
  
- CCACGTCCAC CACCGCGCTT CTACAGGAAA ATCGTCTCCA CCCCACCGAT TTTCTTTATC CCCATGACGC   
  
  
- AAAACCTAAG ACCCAACACT CAAGACCCAA GATTTAAATA AGCAATTGAG GTCGAAGAGC AGGATGAAGC   
  
  
- CCAAGTCTAG AACGACAAAG CAGAAGTAGG AGACAGTAAC GGACAGTTTC AGCTGCTAAC GCAACCAGGA   
  
  
- GCAGTCCAAG TCTAGAGTGA GGAGTAAAAG GGAAGCCTAA GGATAAAAAA GTTGAAAGAC TTTAAATACG   
  
  
- GTTAGAAAAA GCTATAAAAA GACCCAAAAC ATACTTGTAT ACACCCAACT TCAACTTCTC CCCTCCCTCT   
  
  
- CAATATCTCC AAACCTCTCT CTTCTTCTAC TTTCTCTCAC CCCTTCTTGC TCCTCCCCCT TTTCTTTCTA   
  
  
- TGAAAGTACC AAATCCCTCA CACACTTTCA AACTCTTCCA CAACCCACTC TCACTCTCCC TCTCTCACAA   
  
  
- CGCCGTCGAT ACCCACCTAG ACCCAAGAA

+     ARE

| Site Name | Organism | Position | Strand | Matrix score. | sequence | function |
| --- | --- | --- | --- | --- | --- | --- |
| ARE | Zea mays | 1408 | - | 6 | AAACCA | cis-acting regulatory element essential for the anaerobic induction |

>037815   
+ TTTCAGATGC TTCTCACAGA TATTCAAAGT TTGGTTGATT CAACTATTCA ACAAGTAGGA AACTAATTAG   
  
  
+ CACAAAGAAA AACAAATGGG GGGTCTGAAG ATATTTTGGG ATTAAAATAC GAGGGGTGGA TGGGTATATG   
  
  
+ TAATGTAAGA GAGGAGGAAG GTAGCATGCA GTAGCAGAAG CGATAAAAAA ACACGACAGT GAAAAGGAAC   
  
  
+ ACGAAGACGA CTTGGAATAC GGTTGGGAAG GGAATATGAG TGTTACATCG CATGTTTTTA TACAGTGGTG   
  
  
+ AGATATTTTA TTTTTAAGTT ATTAACTTTT TAACCCACAT ATCTTACCAG TAATATAATG ATACGTAGTG   
  
  
+ TACTATCTCA TATGTCGGTC ACATTAAAAA AAATTTCTCA ATTATCATTT GATGTTATAT TTGACACGTC   
  
  
+ AATTCGTCTC TTTGATTAAT AATTATATAA ATTATTTATC ACATCATATA ATACACTGTA CACACCAATA   
  
  
+ TAATATAAAC ATAAACATGT GATTTTCCTG ATATTACTAT GAATGAAATT ATATACAATA TTATTTATAA   
  
  
+ TGTTGTCCTC AAGGTGGAGA TTTCGAGTTC AAATTATACA TATACAAGAG ATTCATGCAA GTATTCAATA   
  
  
+ TTAAATTGTG ATCACCCCTT AATCATTTGT GAAAAAGAAA AAGTTAACTT GCCAATCTCA TTCCTCTTAA   
  
  
+ TAATTAACAA TTTAGCTTAT TAATGCTATC ATTTGTTAAA AATTTAAAAT ATGAGAACTT TTATGATACT   
  
  
+ TTTTTATAGA AGTTAATAGT ATCTCGTTTT AATAAAATTA TTTGCGGACG TTATGTAGTT ACTCATCATC   
  
  
+ ATTATTTGCA TTCGGAAATC CTATCTTCAC GTTTAACGAT TTTACAACAT GATGTATGAA TTAGAAGAAA   
  
  
+ AATAATGTTA TTACGCAAGA ATTCTTCTCT CTCCAAACCT CTATAACTCT CCCTCCCCTC TCTTTCATTT   
  
  
+ GGTGCAGGTG GTGGCGCGAA GATGTCCTTT TAGCAGAGGT GGGGTGGCTA AAAGAAATAG GGGTACTGCG   
  
  
+ TTTTGGATTC TGGGTTGTGA GTTCTGGGTT CTAAATTTAT TCGTTAACTC CAGCTTCTCG TCCTACTTCG   
  
  
+ GGTTCAGATC TTGCTGTTTC GTCTTCATCC TCTGTCATTG CCTGTCAAAG TCGACGATTG CGTTGGTCCT   
  
  
+ CGTCAGGTTC AGATCTCACT CCTCATTTTC CCTTCGGATT CCTATTTTTT CAACTTTCTG AAATTTATGC   
  
  
+ CAATCTTTTT CGATATTTTT CTGGGTTTTG TATGAACATA TGTGGGTTGA AGTTGAAGAG GGGAGGGAGA   
  
  
+ GTTATAGAGG TTTGGAGAGA GAAGAAGATG AAAGAGAGTG GGGAAGAACG AGGAGGGGGA AAAGAAAGAT   
  
  
+ ACTTTCATGG TTTAGGGAGT GTGTGAAAGT TTGAGAAGGT GTTGGGTGAG AGTGAGAGGG AGAGAGTGTT   
  
  
+ GCGGCAGCTA TGGGTGGATC TGGGTTCTT  

- AAAGTCTACG AAGAGTGTCT ATAAGTTTCA AACCAACTAA GTTGATAAGT TGTTCATCCT TTGATTAATC   
  
  
- GTGTTTCTTT TTGTTTACCC CCCAGACTTC TATAAAACCC TAATTTTATG CTCCCCACCT ACCCATATAC   
  
  
- ATTACATTCT CTCCTCCTTC CATCGTACGT CATCGTCTTC GCTATTTTTT TGTGCTGTCA CTTTTCCTTG   
  
  
- TGCTTCTGCT GAACCTTATG CCAACCCTTC CCTTATACTC ACAATGTAGC GTACAAAAAT ATGTCACCAC   
  
  
- TCTATAAAAT AAAAATTCAA TAATTGAAAA ATTGGGTGTA TAGAATGGTC ATTATATTAC TATGCATCAC   
  
  
- ATGATAGAGT ATACAGCCAG TGTAATTTTT TTTAAAGAGT TAATAGTAAA CTACAATATA AACTGTGCAG   
  
  
- TTAAGCAGAG AAACTAATTA TTAATATATT TAATAAATAG TGTAGTATAT TATGTGACAT GTGTGGTTAT   
  
  
- ATTATATTTG TATTTGTACA CTAAAAGGAC TATAATGATA CTTACTTTAA TATATGTTAT AATAAATATT   
  
  
- ACAACAGGAG TTCCACCTCT AAAGCTCAAG TTTAATATGT ATATGTTCTC TAAGTACGTT CATAAGTTAT   
  
  
- AATTTAACAC TAGTGGGGAA TTAGTAAACA CTTTTTCTTT TTCAATTGAA CGGTTAGAGT AAGGAGAATT   
  
  
- ATTAATTGTT AAATCGAATA ATTACGATAG TAAACAATTT TTAAATTTTA TACTCTTGAA AATACTATGA   
  
  
- AAAAATATCT TCAATTATCA TAGAGCAAAA TTATTTTAAT AAACGCCTGC AATACATCAA TGAGTAGTAG   
  
  
- TAATAAACGT AAGCCTTTAG GATAGAAGTG CAAATTGCTA AAATGTTGTA CTACATACTT AATCTTCTTT   
  
  
- TTATTACAAT AATGCGTTCT TAAGAAGAGA GAGGTTTGGA GATATTGAGA GGGAGGGGAG AGAAAGTAAA   
  
  
- CCACGTCCAC CACCGCGCTT CTACAGGAAA ATCGTCTCCA CCCCACCGAT TTTCTTTATC CCCATGACGC   
  
  
- AAAACCTAAG ACCCAACACT CAAGACCCAA GATTTAAATA AGCAATTGAG GTCGAAGAGC AGGATGAAGC   
  
  
- CCAAGTCTAG AACGACAAAG CAGAAGTAGG AGACAGTAAC GGACAGTTTC AGCTGCTAAC GCAACCAGGA   
  
  
- GCAGTCCAAG TCTAGAGTGA GGAGTAAAAG GGAAGCCTAA GGATAAAAAA GTTGAAAGAC TTTAAATACG   
  
  
- GTTAGAAAAA GCTATAAAAA GACCCAAAAC ATACTTGTAT ACACCCAACT TCAACTTCTC CCCTCCCTCT   
  
  
- CAATATCTCC AAACCTCTCT CTTCTTCTAC TTTCTCTCAC CCCTTCTTGC TCCTCCCCCT TTTCTTTCTA   
  
  
- TGAAAGTACC AAATCCCTCA CACACTTTCA AACTCTTCCA CAACCCACTC TCACTCTCCC TCTCTCACAA   
  
  
- CGCCGTCGAT ACCCACCTAG ACCCAAGAA

+     AT~TATA-box

| Site Name | Organism | Position | Strand | Matrix score. | sequence | function |
| --- | --- | --- | --- | --- | --- | --- |
| AT~TATA-box | Arabidopsis thaliana | 540 | + | 6 | TATATA |  |
| AT~TATA-box | Arabidopsis thaliana | 444 | + | 6 | TATATA |  |

>037815   
+ TTTCAGATGC TTCTCACAGA TATTCAAAGT TTGGTTGATT CAACTATTCA ACAAGTAGGA AACTAATTAG   
  
  
+ CACAAAGAAA AACAAATGGG GGGTCTGAAG ATATTTTGGG ATTAAAATAC GAGGGGTGGA TGGGTATATG   
  
  
+ TAATGTAAGA GAGGAGGAAG GTAGCATGCA GTAGCAGAAG CGATAAAAAA ACACGACAGT GAAAAGGAAC   
  
  
+ ACGAAGACGA CTTGGAATAC GGTTGGGAAG GGAATATGAG TGTTACATCG CATGTTTTTA TACAGTGGTG   
  
  
+ AGATATTTTA TTTTTAAGTT ATTAACTTTT TAACCCACAT ATCTTACCAG TAATATAATG ATACGTAGTG   
  
  
+ TACTATCTCA TATGTCGGTC ACATTAAAAA AAATTTCTCA ATTATCATTT GATGTTATAT TTGACACGTC   
  
  
+ AATTCGTCTC TTTGATTAAT AATTATATAA ATTATTTATC ACATCATATA ATACACTGTA CACACCAATA   
  
  
+ TAATATAAAC ATAAACATGT GATTTTCCTG ATATTACTAT GAATGAAATT ATATACAATA TTATTTATAA   
  
  
+ TGTTGTCCTC AAGGTGGAGA TTTCGAGTTC AAATTATACA TATACAAGAG ATTCATGCAA GTATTCAATA   
  
  
+ TTAAATTGTG ATCACCCCTT AATCATTTGT GAAAAAGAAA AAGTTAACTT GCCAATCTCA TTCCTCTTAA   
  
  
+ TAATTAACAA TTTAGCTTAT TAATGCTATC ATTTGTTAAA AATTTAAAAT ATGAGAACTT TTATGATACT   
  
  
+ TTTTTATAGA AGTTAATAGT ATCTCGTTTT AATAAAATTA TTTGCGGACG TTATGTAGTT ACTCATCATC   
  
  
+ ATTATTTGCA TTCGGAAATC CTATCTTCAC GTTTAACGAT TTTACAACAT GATGTATGAA TTAGAAGAAA   
  
  
+ AATAATGTTA TTACGCAAGA ATTCTTCTCT CTCCAAACCT CTATAACTCT CCCTCCCCTC TCTTTCATTT   
  
  
+ GGTGCAGGTG GTGGCGCGAA GATGTCCTTT TAGCAGAGGT GGGGTGGCTA AAAGAAATAG GGGTACTGCG   
  
  
+ TTTTGGATTC TGGGTTGTGA GTTCTGGGTT CTAAATTTAT TCGTTAACTC CAGCTTCTCG TCCTACTTCG   
  
  
+ GGTTCAGATC TTGCTGTTTC GTCTTCATCC TCTGTCATTG CCTGTCAAAG TCGACGATTG CGTTGGTCCT   
  
  
+ CGTCAGGTTC AGATCTCACT CCTCATTTTC CCTTCGGATT CCTATTTTTT CAACTTTCTG AAATTTATGC   
  
  
+ CAATCTTTTT CGATATTTTT CTGGGTTTTG TATGAACATA TGTGGGTTGA AGTTGAAGAG GGGAGGGAGA   
  
  
+ GTTATAGAGG TTTGGAGAGA GAAGAAGATG AAAGAGAGTG GGGAAGAACG AGGAGGGGGA AAAGAAAGAT   
  
  
+ ACTTTCATGG TTTAGGGAGT GTGTGAAAGT TTGAGAAGGT GTTGGGTGAG AGTGAGAGGG AGAGAGTGTT   
  
  
+ GCGGCAGCTA TGGGTGGATC TGGGTTCTT  

- AAAGTCTACG AAGAGTGTCT ATAAGTTTCA AACCAACTAA GTTGATAAGT TGTTCATCCT TTGATTAATC   
  
  
- GTGTTTCTTT TTGTTTACCC CCCAGACTTC TATAAAACCC TAATTTTATG CTCCCCACCT ACCCATATAC   
  
  
- ATTACATTCT CTCCTCCTTC CATCGTACGT CATCGTCTTC GCTATTTTTT TGTGCTGTCA CTTTTCCTTG   
  
  
- TGCTTCTGCT GAACCTTATG CCAACCCTTC CCTTATACTC ACAATGTAGC GTACAAAAAT ATGTCACCAC   
  
  
- TCTATAAAAT AAAAATTCAA TAATTGAAAA ATTGGGTGTA TAGAATGGTC ATTATATTAC TATGCATCAC   
  
  
- ATGATAGAGT ATACAGCCAG TGTAATTTTT TTTAAAGAGT TAATAGTAAA CTACAATATA AACTGTGCAG   
  
  
- TTAAGCAGAG AAACTAATTA TTAATATATT TAATAAATAG TGTAGTATAT TATGTGACAT GTGTGGTTAT   
  
  
- ATTATATTTG TATTTGTACA CTAAAAGGAC TATAATGATA CTTACTTTAA TATATGTTAT AATAAATATT   
  
  
- ACAACAGGAG TTCCACCTCT AAAGCTCAAG TTTAATATGT ATATGTTCTC TAAGTACGTT CATAAGTTAT   
  
  
- AATTTAACAC TAGTGGGGAA TTAGTAAACA CTTTTTCTTT TTCAATTGAA CGGTTAGAGT AAGGAGAATT   
  
  
- ATTAATTGTT AAATCGAATA ATTACGATAG TAAACAATTT TTAAATTTTA TACTCTTGAA AATACTATGA   
  
  
- AAAAATATCT TCAATTATCA TAGAGCAAAA TTATTTTAAT AAACGCCTGC AATACATCAA TGAGTAGTAG   
  
  
- TAATAAACGT AAGCCTTTAG GATAGAAGTG CAAATTGCTA AAATGTTGTA CTACATACTT AATCTTCTTT   
  
  
- TTATTACAAT AATGCGTTCT TAAGAAGAGA GAGGTTTGGA GATATTGAGA GGGAGGGGAG AGAAAGTAAA   
  
  
- CCACGTCCAC CACCGCGCTT CTACAGGAAA ATCGTCTCCA CCCCACCGAT TTTCTTTATC CCCATGACGC   
  
  
- AAAACCTAAG ACCCAACACT CAAGACCCAA GATTTAAATA AGCAATTGAG GTCGAAGAGC AGGATGAAGC   
  
  
- CCAAGTCTAG AACGACAAAG CAGAAGTAGG AGACAGTAAC GGACAGTTTC AGCTGCTAAC GCAACCAGGA   
  
  
- GCAGTCCAAG TCTAGAGTGA GGAGTAAAAG GGAAGCCTAA GGATAAAAAA GTTGAAAGAC TTTAAATACG   
  
  
- GTTAGAAAAA GCTATAAAAA GACCCAAAAC ATACTTGTAT ACACCCAACT TCAACTTCTC CCCTCCCTCT   
  
  
- CAATATCTCC AAACCTCTCT CTTCTTCTAC TTTCTCTCAC CCCTTCTTGC TCCTCCCCCT TTTCTTTCTA   
  
  
- TGAAAGTACC AAATCCCTCA CACACTTTCA AACTCTTCCA CAACCCACTC TCACTCTCCC TCTCTCACAA   
  
  
- CGCCGTCGAT ACCCACCTAG ACCCAAGAA

+     Box 4

| Site Name | Organism | Position | Strand | Matrix score. | sequence | function |
| --- | --- | --- | --- | --- | --- | --- |
| Box 4 | Petroselinum crispum | 719 | + | 6 | ATTAAT | part of a conserved DNA module involved in light responsiveness |
| Box 4 | Petroselinum crispum | 435 | + | 6 | ATTAAT | part of a conserved DNA module involved in light responsiveness |

>037815   
+ TTTCAGATGC TTCTCACAGA TATTCAAAGT TTGGTTGATT CAACTATTCA ACAAGTAGGA AACTAATTAG   
  
  
+ CACAAAGAAA AACAAATGGG GGGTCTGAAG ATATTTTGGG ATTAAAATAC GAGGGGTGGA TGGGTATATG   
  
  
+ TAATGTAAGA GAGGAGGAAG GTAGCATGCA GTAGCAGAAG CGATAAAAAA ACACGACAGT GAAAAGGAAC   
  
  
+ ACGAAGACGA CTTGGAATAC GGTTGGGAAG GGAATATGAG TGTTACATCG CATGTTTTTA TACAGTGGTG   
  
  
+ AGATATTTTA TTTTTAAGTT ATTAACTTTT TAACCCACAT ATCTTACCAG TAATATAATG ATACGTAGTG   
  
  
+ TACTATCTCA TATGTCGGTC ACATTAAAAA AAATTTCTCA ATTATCATTT GATGTTATAT TTGACACGTC   
  
  
+ AATTCGTCTC TTTGATTAAT AATTATATAA ATTATTTATC ACATCATATA ATACACTGTA CACACCAATA   
  
  
+ TAATATAAAC ATAAACATGT GATTTTCCTG ATATTACTAT GAATGAAATT ATATACAATA TTATTTATAA   
  
  
+ TGTTGTCCTC AAGGTGGAGA TTTCGAGTTC AAATTATACA TATACAAGAG ATTCATGCAA GTATTCAATA   
  
  
+ TTAAATTGTG ATCACCCCTT AATCATTTGT GAAAAAGAAA AAGTTAACTT GCCAATCTCA TTCCTCTTAA   
  
  
+ TAATTAACAA TTTAGCTTAT TAATGCTATC ATTTGTTAAA AATTTAAAAT ATGAGAACTT TTATGATACT   
  
  
+ TTTTTATAGA AGTTAATAGT ATCTCGTTTT AATAAAATTA TTTGCGGACG TTATGTAGTT ACTCATCATC   
  
  
+ ATTATTTGCA TTCGGAAATC CTATCTTCAC GTTTAACGAT TTTACAACAT GATGTATGAA TTAGAAGAAA   
  
  
+ AATAATGTTA TTACGCAAGA ATTCTTCTCT CTCCAAACCT CTATAACTCT CCCTCCCCTC TCTTTCATTT   
  
  
+ GGTGCAGGTG GTGGCGCGAA GATGTCCTTT TAGCAGAGGT GGGGTGGCTA AAAGAAATAG GGGTACTGCG   
  
  
+ TTTTGGATTC TGGGTTGTGA GTTCTGGGTT CTAAATTTAT TCGTTAACTC CAGCTTCTCG TCCTACTTCG   
  
  
+ GGTTCAGATC TTGCTGTTTC GTCTTCATCC TCTGTCATTG CCTGTCAAAG TCGACGATTG CGTTGGTCCT   
  
  
+ CGTCAGGTTC AGATCTCACT CCTCATTTTC CCTTCGGATT CCTATTTTTT CAACTTTCTG AAATTTATGC   
  
  
+ CAATCTTTTT CGATATTTTT CTGGGTTTTG TATGAACATA TGTGGGTTGA AGTTGAAGAG GGGAGGGAGA   
  
  
+ GTTATAGAGG TTTGGAGAGA GAAGAAGATG AAAGAGAGTG GGGAAGAACG AGGAGGGGGA AAAGAAAGAT   
  
  
+ ACTTTCATGG TTTAGGGAGT GTGTGAAAGT TTGAGAAGGT GTTGGGTGAG AGTGAGAGGG AGAGAGTGTT   
  
  
+ GCGGCAGCTA TGGGTGGATC TGGGTTCTT  

- AAAGTCTACG AAGAGTGTCT ATAAGTTTCA AACCAACTAA GTTGATAAGT TGTTCATCCT TTGATTAATC   
  
  
- GTGTTTCTTT TTGTTTACCC CCCAGACTTC TATAAAACCC TAATTTTATG CTCCCCACCT ACCCATATAC   
  
  
- ATTACATTCT CTCCTCCTTC CATCGTACGT CATCGTCTTC GCTATTTTTT TGTGCTGTCA CTTTTCCTTG   
  
  
- TGCTTCTGCT GAACCTTATG CCAACCCTTC CCTTATACTC ACAATGTAGC GTACAAAAAT ATGTCACCAC   
  
  
- TCTATAAAAT AAAAATTCAA TAATTGAAAA ATTGGGTGTA TAGAATGGTC ATTATATTAC TATGCATCAC   
  
  
- ATGATAGAGT ATACAGCCAG TGTAATTTTT TTTAAAGAGT TAATAGTAAA CTACAATATA AACTGTGCAG   
  
  
- TTAAGCAGAG AAACTAATTA TTAATATATT TAATAAATAG TGTAGTATAT TATGTGACAT GTGTGGTTAT   
  
  
- ATTATATTTG TATTTGTACA CTAAAAGGAC TATAATGATA CTTACTTTAA TATATGTTAT AATAAATATT   
  
  
- ACAACAGGAG TTCCACCTCT AAAGCTCAAG TTTAATATGT ATATGTTCTC TAAGTACGTT CATAAGTTAT   
  
  
- AATTTAACAC TAGTGGGGAA TTAGTAAACA CTTTTTCTTT TTCAATTGAA CGGTTAGAGT AAGGAGAATT   
  
  
- ATTAATTGTT AAATCGAATA ATTACGATAG TAAACAATTT TTAAATTTTA TACTCTTGAA AATACTATGA   
  
  
- AAAAATATCT TCAATTATCA TAGAGCAAAA TTATTTTAAT AAACGCCTGC AATACATCAA TGAGTAGTAG   
  
  
- TAATAAACGT AAGCCTTTAG GATAGAAGTG CAAATTGCTA AAATGTTGTA CTACATACTT AATCTTCTTT   
  
  
- TTATTACAAT AATGCGTTCT TAAGAAGAGA GAGGTTTGGA GATATTGAGA GGGAGGGGAG AGAAAGTAAA   
  
  
- CCACGTCCAC CACCGCGCTT CTACAGGAAA ATCGTCTCCA CCCCACCGAT TTTCTTTATC CCCATGACGC   
  
  
- AAAACCTAAG ACCCAACACT CAAGACCCAA GATTTAAATA AGCAATTGAG GTCGAAGAGC AGGATGAAGC   
  
  
- CCAAGTCTAG AACGACAAAG CAGAAGTAGG AGACAGTAAC GGACAGTTTC AGCTGCTAAC GCAACCAGGA   
  
  
- GCAGTCCAAG TCTAGAGTGA GGAGTAAAAG GGAAGCCTAA GGATAAAAAA GTTGAAAGAC TTTAAATACG   
  
  
- GTTAGAAAAA GCTATAAAAA GACCCAAAAC ATACTTGTAT ACACCCAACT TCAACTTCTC CCCTCCCTCT   
  
  
- CAATATCTCC AAACCTCTCT CTTCTTCTAC TTTCTCTCAC CCCTTCTTGC TCCTCCCCCT TTTCTTTCTA   
  
  
- TGAAAGTACC AAATCCCTCA CACACTTTCA AACTCTTCCA CAACCCACTC TCACTCTCCC TCTCTCACAA   
  
  
- CGCCGTCGAT ACCCACCTAG ACCCAAGAA

+     CAAT-box

| Site Name | Organism | Position | Strand | Matrix score. | sequence | function |
| --- | --- | --- | --- | --- | --- | --- |
| CAAT-box | Pisum sativum | 810 | - | 5 | CAAAT | common cis-acting element in promoter and enhancer regions |
| CAAT-box | Nicotiana glutinosa | 626 | + | 4 | CAAT |  |
| CAAT-box | Pisum sativum | 844 | - | 5 | CAAAT | common cis-acting element in promoter and enhancer regions |
| CAAT-box | Pisum sativum | 590 | + | 5 | CAAAT | common cis-acting element in promoter and enhancer regions |
| CAAT-box | Arabidopsis thaliana | 485 | + | 5 | CCAAT | common cis-acting element in promoter and enhancer regions |
| CAAT-box | Arabidopsis thaliana | 682 | + | 5 | CCAAT | common cis-acting element in promoter and enhancer regions |
| CAAT-box | Nicotiana glutinosa | 683 | + | 4 | CAAT |  |
| CAAT-box | Nicotiana glutinosa | 420 | + | 4 | CAAT |  |
| CAAT-box | Nicotiana glutinosa | 1261 | + | 4 | CAAT |  |
| CAAT-box | Pisum sativum | 409 | - | 5 | CAAAT | common cis-acting element in promoter and enhancer regions |
| CAAT-box | Arabidopsis thaliana | 1260 | + | 5 | CCAAT | common cis-acting element in promoter and enhancer regions |
| CAAT-box | Nicotiana glutinosa | 635 | - | 4 | CAAT |  |
| CAAT-box | Nicotiana glutinosa | 1177 | - | 4 | CAAT |  |
| CAAT-box | Nicotiana glutinosa | 1157 | - | 4 | CAAT |  |
| CAAT-box | Pisum sativum | 977 | - | 5 | CAAAT | common cis-acting element in promoter and enhancer regions |
| CAAT-box | Nicotiana glutinosa | 708 | + | 4 | CAAT |  |
| CAAT-box | Nicotiana glutinosa | 546 | + | 4 | CAAT |  |
| CAAT-box | Pisum sativum | 655 | - | 5 | CAAAT | common cis-acting element in promoter and enhancer regions |
| CAAT-box | Pisum sativum | 731 | - | 5 | CAAAT | common cis-acting element in promoter and enhancer regions |
| CAAT-box | Pisum sativum | 83 | + | 5 | CAAAT | common cis-acting element in promoter and enhancer regions |
| CAAT-box | Pisum sativum | 397 | - | 5 | CAAAT | common cis-acting element in promoter and enhancer regions |
| CAAT-box | Nicotiana glutinosa | 486 | + | 4 | CAAT |  |
| CAAT-box | Nicotiana glutinosa | 389 | + | 4 | CAAT |  |

>037815   
+ TTTCAGATGC TTCTCACAGA TATTCAAAGT TTGGTTGATT CAACTATTCA ACAAGTAGGA AACTAATTAG   
  
  
+ CACAAAGAAA AACAAATGGG GGGTCTGAAG ATATTTTGGG ATTAAAATAC GAGGGGTGGA TGGGTATATG   
  
  
+ TAATGTAAGA GAGGAGGAAG GTAGCATGCA GTAGCAGAAG CGATAAAAAA ACACGACAGT GAAAAGGAAC   
  
  
+ ACGAAGACGA CTTGGAATAC GGTTGGGAAG GGAATATGAG TGTTACATCG CATGTTTTTA TACAGTGGTG   
  
  
+ AGATATTTTA TTTTTAAGTT ATTAACTTTT TAACCCACAT ATCTTACCAG TAATATAATG ATACGTAGTG   
  
  
+ TACTATCTCA TATGTCGGTC ACATTAAAAA AAATTTCTCA ATTATCATTT GATGTTATAT TTGACACGTC   
  
  
+ AATTCGTCTC TTTGATTAAT AATTATATAA ATTATTTATC ACATCATATA ATACACTGTA CACACCAATA   
  
  
+ TAATATAAAC ATAAACATGT GATTTTCCTG ATATTACTAT GAATGAAATT ATATACAATA TTATTTATAA   
  
  
+ TGTTGTCCTC AAGGTGGAGA TTTCGAGTTC AAATTATACA TATACAAGAG ATTCATGCAA GTATTCAATA   
  
  
+ TTAAATTGTG ATCACCCCTT AATCATTTGT GAAAAAGAAA AAGTTAACTT GCCAATCTCA TTCCTCTTAA   
  
  
+ TAATTAACAA TTTAGCTTAT TAATGCTATC ATTTGTTAAA AATTTAAAAT ATGAGAACTT TTATGATACT   
  
  
+ TTTTTATAGA AGTTAATAGT ATCTCGTTTT AATAAAATTA TTTGCGGACG TTATGTAGTT ACTCATCATC   
  
  
+ ATTATTTGCA TTCGGAAATC CTATCTTCAC GTTTAACGAT TTTACAACAT GATGTATGAA TTAGAAGAAA   
  
  
+ AATAATGTTA TTACGCAAGA ATTCTTCTCT CTCCAAACCT CTATAACTCT CCCTCCCCTC TCTTTCATTT   
  
  
+ GGTGCAGGTG GTGGCGCGAA GATGTCCTTT TAGCAGAGGT GGGGTGGCTA AAAGAAATAG GGGTACTGCG   
  
  
+ TTTTGGATTC TGGGTTGTGA GTTCTGGGTT CTAAATTTAT TCGTTAACTC CAGCTTCTCG TCCTACTTCG   
  
  
+ GGTTCAGATC TTGCTGTTTC GTCTTCATCC TCTGTCATTG CCTGTCAAAG TCGACGATTG CGTTGGTCCT   
  
  
+ CGTCAGGTTC AGATCTCACT CCTCATTTTC CCTTCGGATT CCTATTTTTT CAACTTTCTG AAATTTATGC   
  
  
+ CAATCTTTTT CGATATTTTT CTGGGTTTTG TATGAACATA TGTGGGTTGA AGTTGAAGAG GGGAGGGAGA   
  
  
+ GTTATAGAGG TTTGGAGAGA GAAGAAGATG AAAGAGAGTG GGGAAGAACG AGGAGGGGGA AAAGAAAGAT   
  
  
+ ACTTTCATGG TTTAGGGAGT GTGTGAAAGT TTGAGAAGGT GTTGGGTGAG AGTGAGAGGG AGAGAGTGTT   
  
  
+ GCGGCAGCTA TGGGTGGATC TGGGTTCTT  

- AAAGTCTACG AAGAGTGTCT ATAAGTTTCA AACCAACTAA GTTGATAAGT TGTTCATCCT TTGATTAATC   
  
  
- GTGTTTCTTT TTGTTTACCC CCCAGACTTC TATAAAACCC TAATTTTATG CTCCCCACCT ACCCATATAC   
  
  
- ATTACATTCT CTCCTCCTTC CATCGTACGT CATCGTCTTC GCTATTTTTT TGTGCTGTCA CTTTTCCTTG   
  
  
- TGCTTCTGCT GAACCTTATG CCAACCCTTC CCTTATACTC ACAATGTAGC GTACAAAAAT ATGTCACCAC   
  
  
- TCTATAAAAT AAAAATTCAA TAATTGAAAA ATTGGGTGTA TAGAATGGTC ATTATATTAC TATGCATCAC   
  
  
- ATGATAGAGT ATACAGCCAG TGTAATTTTT TTTAAAGAGT TAATAGTAAA CTACAATATA AACTGTGCAG   
  
  
- TTAAGCAGAG AAACTAATTA TTAATATATT TAATAAATAG TGTAGTATAT TATGTGACAT GTGTGGTTAT   
  
  
- ATTATATTTG TATTTGTACA CTAAAAGGAC TATAATGATA CTTACTTTAA TATATGTTAT AATAAATATT   
  
  
- ACAACAGGAG TTCCACCTCT AAAGCTCAAG TTTAATATGT ATATGTTCTC TAAGTACGTT CATAAGTTAT   
  
  
- AATTTAACAC TAGTGGGGAA TTAGTAAACA CTTTTTCTTT TTCAATTGAA CGGTTAGAGT AAGGAGAATT   
  
  
- ATTAATTGTT AAATCGAATA ATTACGATAG TAAACAATTT TTAAATTTTA TACTCTTGAA AATACTATGA   
  
  
- AAAAATATCT TCAATTATCA TAGAGCAAAA TTATTTTAAT AAACGCCTGC AATACATCAA TGAGTAGTAG   
  
  
- TAATAAACGT AAGCCTTTAG GATAGAAGTG CAAATTGCTA AAATGTTGTA CTACATACTT AATCTTCTTT   
  
  
- TTATTACAAT AATGCGTTCT TAAGAAGAGA GAGGTTTGGA GATATTGAGA GGGAGGGGAG AGAAAGTAAA   
  
  
- CCACGTCCAC CACCGCGCTT CTACAGGAAA ATCGTCTCCA CCCCACCGAT TTTCTTTATC CCCATGACGC   
  
  
- AAAACCTAAG ACCCAACACT CAAGACCCAA GATTTAAATA AGCAATTGAG GTCGAAGAGC AGGATGAAGC   
  
  
- CCAAGTCTAG AACGACAAAG CAGAAGTAGG AGACAGTAAC GGACAGTTTC AGCTGCTAAC GCAACCAGGA   
  
  
- GCAGTCCAAG TCTAGAGTGA GGAGTAAAAG GGAAGCCTAA GGATAAAAAA GTTGAAAGAC TTTAAATACG   
  
  
- GTTAGAAAAA GCTATAAAAA GACCCAAAAC ATACTTGTAT ACACCCAACT TCAACTTCTC CCCTCCCTCT   
  
  
- CAATATCTCC AAACCTCTCT CTTCTTCTAC TTTCTCTCAC CCCTTCTTGC TCCTCCCCCT TTTCTTTCTA   
  
  
- TGAAAGTACC AAATCCCTCA CACACTTTCA AACTCTTCCA CAACCCACTC TCACTCTCCC TCTCTCACAA   
  
  
- CGCCGTCGAT ACCCACCTAG ACCCAAGAA

+     CGTCA-motif

| Site Name | Organism | Position | Strand | Matrix score. | sequence | function |
| --- | --- | --- | --- | --- | --- | --- |
| CGTCA-motif | Hordeum vulgare | 417 | + | 5 | CGTCA | cis-acting regulatory element involved in the MeJA-responsiveness |
| CGTCA-motif | Hordeum vulgare | 1191 | + | 5 | CGTCA | cis-acting regulatory element involved in the MeJA-responsiveness |

>037815   
+ TTTCAGATGC TTCTCACAGA TATTCAAAGT TTGGTTGATT CAACTATTCA ACAAGTAGGA AACTAATTAG   
  
  
+ CACAAAGAAA AACAAATGGG GGGTCTGAAG ATATTTTGGG ATTAAAATAC GAGGGGTGGA TGGGTATATG   
  
  
+ TAATGTAAGA GAGGAGGAAG GTAGCATGCA GTAGCAGAAG CGATAAAAAA ACACGACAGT GAAAAGGAAC   
  
  
+ ACGAAGACGA CTTGGAATAC GGTTGGGAAG GGAATATGAG TGTTACATCG CATGTTTTTA TACAGTGGTG   
  
  
+ AGATATTTTA TTTTTAAGTT ATTAACTTTT TAACCCACAT ATCTTACCAG TAATATAATG ATACGTAGTG   
  
  
+ TACTATCTCA TATGTCGGTC ACATTAAAAA AAATTTCTCA ATTATCATTT GATGTTATAT TTGACACGTC   
  
  
+ AATTCGTCTC TTTGATTAAT AATTATATAA ATTATTTATC ACATCATATA ATACACTGTA CACACCAATA   
  
  
+ TAATATAAAC ATAAACATGT GATTTTCCTG ATATTACTAT GAATGAAATT ATATACAATA TTATTTATAA   
  
  
+ TGTTGTCCTC AAGGTGGAGA TTTCGAGTTC AAATTATACA TATACAAGAG ATTCATGCAA GTATTCAATA   
  
  
+ TTAAATTGTG ATCACCCCTT AATCATTTGT GAAAAAGAAA AAGTTAACTT GCCAATCTCA TTCCTCTTAA   
  
  
+ TAATTAACAA TTTAGCTTAT TAATGCTATC ATTTGTTAAA AATTTAAAAT ATGAGAACTT TTATGATACT   
  
  
+ TTTTTATAGA AGTTAATAGT ATCTCGTTTT AATAAAATTA TTTGCGGACG TTATGTAGTT ACTCATCATC   
  
  
+ ATTATTTGCA TTCGGAAATC CTATCTTCAC GTTTAACGAT TTTACAACAT GATGTATGAA TTAGAAGAAA   
  
  
+ AATAATGTTA TTACGCAAGA ATTCTTCTCT CTCCAAACCT CTATAACTCT CCCTCCCCTC TCTTTCATTT   
  
  
+ GGTGCAGGTG GTGGCGCGAA GATGTCCTTT TAGCAGAGGT GGGGTGGCTA AAAGAAATAG GGGTACTGCG   
  
  
+ TTTTGGATTC TGGGTTGTGA GTTCTGGGTT CTAAATTTAT TCGTTAACTC CAGCTTCTCG TCCTACTTCG   
  
  
+ GGTTCAGATC TTGCTGTTTC GTCTTCATCC TCTGTCATTG CCTGTCAAAG TCGACGATTG CGTTGGTCCT   
  
  
+ CGTCAGGTTC AGATCTCACT CCTCATTTTC CCTTCGGATT CCTATTTTTT CAACTTTCTG AAATTTATGC   
  
  
+ CAATCTTTTT CGATATTTTT CTGGGTTTTG TATGAACATA TGTGGGTTGA AGTTGAAGAG GGGAGGGAGA   
  
  
+ GTTATAGAGG TTTGGAGAGA GAAGAAGATG AAAGAGAGTG GGGAAGAACG AGGAGGGGGA AAAGAAAGAT   
  
  
+ ACTTTCATGG TTTAGGGAGT GTGTGAAAGT TTGAGAAGGT GTTGGGTGAG AGTGAGAGGG AGAGAGTGTT   
  
  
+ GCGGCAGCTA TGGGTGGATC TGGGTTCTT  

- AAAGTCTACG AAGAGTGTCT ATAAGTTTCA AACCAACTAA GTTGATAAGT TGTTCATCCT TTGATTAATC   
  
  
- GTGTTTCTTT TTGTTTACCC CCCAGACTTC TATAAAACCC TAATTTTATG CTCCCCACCT ACCCATATAC   
  
  
- ATTACATTCT CTCCTCCTTC CATCGTACGT CATCGTCTTC GCTATTTTTT TGTGCTGTCA CTTTTCCTTG   
  
  
- TGCTTCTGCT GAACCTTATG CCAACCCTTC CCTTATACTC ACAATGTAGC GTACAAAAAT ATGTCACCAC   
  
  
- TCTATAAAAT AAAAATTCAA TAATTGAAAA ATTGGGTGTA TAGAATGGTC ATTATATTAC TATGCATCAC   
  
  
- ATGATAGAGT ATACAGCCAG TGTAATTTTT TTTAAAGAGT TAATAGTAAA CTACAATATA AACTGTGCAG   
  
  
- TTAAGCAGAG AAACTAATTA TTAATATATT TAATAAATAG TGTAGTATAT TATGTGACAT GTGTGGTTAT   
  
  
- ATTATATTTG TATTTGTACA CTAAAAGGAC TATAATGATA CTTACTTTAA TATATGTTAT AATAAATATT   
  
  
- ACAACAGGAG TTCCACCTCT AAAGCTCAAG TTTAATATGT ATATGTTCTC TAAGTACGTT CATAAGTTAT   
  
  
- AATTTAACAC TAGTGGGGAA TTAGTAAACA CTTTTTCTTT TTCAATTGAA CGGTTAGAGT AAGGAGAATT   
  
  
- ATTAATTGTT AAATCGAATA ATTACGATAG TAAACAATTT TTAAATTTTA TACTCTTGAA AATACTATGA   
  
  
- AAAAATATCT TCAATTATCA TAGAGCAAAA TTATTTTAAT AAACGCCTGC AATACATCAA TGAGTAGTAG   
  
  
- TAATAAACGT AAGCCTTTAG GATAGAAGTG CAAATTGCTA AAATGTTGTA CTACATACTT AATCTTCTTT   
  
  
- TTATTACAAT AATGCGTTCT TAAGAAGAGA GAGGTTTGGA GATATTGAGA GGGAGGGGAG AGAAAGTAAA   
  
  
- CCACGTCCAC CACCGCGCTT CTACAGGAAA ATCGTCTCCA CCCCACCGAT TTTCTTTATC CCCATGACGC   
  
  
- AAAACCTAAG ACCCAACACT CAAGACCCAA GATTTAAATA AGCAATTGAG GTCGAAGAGC AGGATGAAGC   
  
  
- CCAAGTCTAG AACGACAAAG CAGAAGTAGG AGACAGTAAC GGACAGTTTC AGCTGCTAAC GCAACCAGGA   
  
  
- GCAGTCCAAG TCTAGAGTGA GGAGTAAAAG GGAAGCCTAA GGATAAAAAA GTTGAAAGAC TTTAAATACG   
  
  
- GTTAGAAAAA GCTATAAAAA GACCCAAAAC ATACTTGTAT ACACCCAACT TCAACTTCTC CCCTCCCTCT   
  
  
- CAATATCTCC AAACCTCTCT CTTCTTCTAC TTTCTCTCAC CCCTTCTTGC TCCTCCCCCT TTTCTTTCTA   
  
  
- TGAAAGTACC AAATCCCTCA CACACTTTCA AACTCTTCCA CAACCCACTC TCACTCTCCC TCTCTCACAA   
  
  
- CGCCGTCGAT ACCCACCTAG ACCCAAGAA

+     CTAG-motif

| Site Name | Organism | Position | Strand | Matrix score. | sequence | function |
| --- | --- | --- | --- | --- | --- | --- |
| CTAG-motif | Avena sativa | 170 | + | 9 | ACTAGCAGAA |  |

>037815   
+ TTTCAGATGC TTCTCACAGA TATTCAAAGT TTGGTTGATT CAACTATTCA ACAAGTAGGA AACTAATTAG   
  
  
+ CACAAAGAAA AACAAATGGG GGGTCTGAAG ATATTTTGGG ATTAAAATAC GAGGGGTGGA TGGGTATATG   
  
  
+ TAATGTAAGA GAGGAGGAAG GTAGCATGCA GTAGCAGAAG CGATAAAAAA ACACGACAGT GAAAAGGAAC   
  
  
+ ACGAAGACGA CTTGGAATAC GGTTGGGAAG GGAATATGAG TGTTACATCG CATGTTTTTA TACAGTGGTG   
  
  
+ AGATATTTTA TTTTTAAGTT ATTAACTTTT TAACCCACAT ATCTTACCAG TAATATAATG ATACGTAGTG   
  
  
+ TACTATCTCA TATGTCGGTC ACATTAAAAA AAATTTCTCA ATTATCATTT GATGTTATAT TTGACACGTC   
  
  
+ AATTCGTCTC TTTGATTAAT AATTATATAA ATTATTTATC ACATCATATA ATACACTGTA CACACCAATA   
  
  
+ TAATATAAAC ATAAACATGT GATTTTCCTG ATATTACTAT GAATGAAATT ATATACAATA TTATTTATAA   
  
  
+ TGTTGTCCTC AAGGTGGAGA TTTCGAGTTC AAATTATACA TATACAAGAG ATTCATGCAA GTATTCAATA   
  
  
+ TTAAATTGTG ATCACCCCTT AATCATTTGT GAAAAAGAAA AAGTTAACTT GCCAATCTCA TTCCTCTTAA   
  
  
+ TAATTAACAA TTTAGCTTAT TAATGCTATC ATTTGTTAAA AATTTAAAAT ATGAGAACTT TTATGATACT   
  
  
+ TTTTTATAGA AGTTAATAGT ATCTCGTTTT AATAAAATTA TTTGCGGACG TTATGTAGTT ACTCATCATC   
  
  
+ ATTATTTGCA TTCGGAAATC CTATCTTCAC GTTTAACGAT TTTACAACAT GATGTATGAA TTAGAAGAAA   
  
  
+ AATAATGTTA TTACGCAAGA ATTCTTCTCT CTCCAAACCT CTATAACTCT CCCTCCCCTC TCTTTCATTT   
  
  
+ GGTGCAGGTG GTGGCGCGAA GATGTCCTTT TAGCAGAGGT GGGGTGGCTA AAAGAAATAG GGGTACTGCG   
  
  
+ TTTTGGATTC TGGGTTGTGA GTTCTGGGTT CTAAATTTAT TCGTTAACTC CAGCTTCTCG TCCTACTTCG   
  
  
+ GGTTCAGATC TTGCTGTTTC GTCTTCATCC TCTGTCATTG CCTGTCAAAG TCGACGATTG CGTTGGTCCT   
  
  
+ CGTCAGGTTC AGATCTCACT CCTCATTTTC CCTTCGGATT CCTATTTTTT CAACTTTCTG AAATTTATGC   
  
  
+ CAATCTTTTT CGATATTTTT CTGGGTTTTG TATGAACATA TGTGGGTTGA AGTTGAAGAG GGGAGGGAGA   
  
  
+ GTTATAGAGG TTTGGAGAGA GAAGAAGATG AAAGAGAGTG GGGAAGAACG AGGAGGGGGA AAAGAAAGAT   
  
  
+ ACTTTCATGG TTTAGGGAGT GTGTGAAAGT TTGAGAAGGT GTTGGGTGAG AGTGAGAGGG AGAGAGTGTT   
  
  
+ GCGGCAGCTA TGGGTGGATC TGGGTTCTT  

- AAAGTCTACG AAGAGTGTCT ATAAGTTTCA AACCAACTAA GTTGATAAGT TGTTCATCCT TTGATTAATC   
  
  
- GTGTTTCTTT TTGTTTACCC CCCAGACTTC TATAAAACCC TAATTTTATG CTCCCCACCT ACCCATATAC   
  
  
- ATTACATTCT CTCCTCCTTC CATCGTACGT CATCGTCTTC GCTATTTTTT TGTGCTGTCA CTTTTCCTTG   
  
  
- TGCTTCTGCT GAACCTTATG CCAACCCTTC CCTTATACTC ACAATGTAGC GTACAAAAAT ATGTCACCAC   
  
  
- TCTATAAAAT AAAAATTCAA TAATTGAAAA ATTGGGTGTA TAGAATGGTC ATTATATTAC TATGCATCAC   
  
  
- ATGATAGAGT ATACAGCCAG TGTAATTTTT TTTAAAGAGT TAATAGTAAA CTACAATATA AACTGTGCAG   
  
  
- TTAAGCAGAG AAACTAATTA TTAATATATT TAATAAATAG TGTAGTATAT TATGTGACAT GTGTGGTTAT   
  
  
- ATTATATTTG TATTTGTACA CTAAAAGGAC TATAATGATA CTTACTTTAA TATATGTTAT AATAAATATT   
  
  
- ACAACAGGAG TTCCACCTCT AAAGCTCAAG TTTAATATGT ATATGTTCTC TAAGTACGTT CATAAGTTAT   
  
  
- AATTTAACAC TAGTGGGGAA TTAGTAAACA CTTTTTCTTT TTCAATTGAA CGGTTAGAGT AAGGAGAATT   
  
  
- ATTAATTGTT AAATCGAATA ATTACGATAG TAAACAATTT TTAAATTTTA TACTCTTGAA AATACTATGA   
  
  
- AAAAATATCT TCAATTATCA TAGAGCAAAA TTATTTTAAT AAACGCCTGC AATACATCAA TGAGTAGTAG   
  
  
- TAATAAACGT AAGCCTTTAG GATAGAAGTG CAAATTGCTA AAATGTTGTA CTACATACTT AATCTTCTTT   
  
  
- TTATTACAAT AATGCGTTCT TAAGAAGAGA GAGGTTTGGA GATATTGAGA GGGAGGGGAG AGAAAGTAAA   
  
  
- CCACGTCCAC CACCGCGCTT CTACAGGAAA ATCGTCTCCA CCCCACCGAT TTTCTTTATC CCCATGACGC   
  
  
- AAAACCTAAG ACCCAACACT CAAGACCCAA GATTTAAATA AGCAATTGAG GTCGAAGAGC AGGATGAAGC   
  
  
- CCAAGTCTAG AACGACAAAG CAGAAGTAGG AGACAGTAAC GGACAGTTTC AGCTGCTAAC GCAACCAGGA   
  
  
- GCAGTCCAAG TCTAGAGTGA GGAGTAAAAG GGAAGCCTAA GGATAAAAAA GTTGAAAGAC TTTAAATACG   
  
  
- GTTAGAAAAA GCTATAAAAA GACCCAAAAC ATACTTGTAT ACACCCAACT TCAACTTCTC CCCTCCCTCT   
  
  
- CAATATCTCC AAACCTCTCT CTTCTTCTAC TTTCTCTCAC CCCTTCTTGC TCCTCCCCCT TTTCTTTCTA   
  
  
- TGAAAGTACC AAATCCCTCA CACACTTTCA AACTCTTCCA CAACCCACTC TCACTCTCCC TCTCTCACAA   
  
  
- CGCCGTCGAT ACCCACCTAG ACCCAAGAA

+     ERE

| Site Name | Organism | Position | Strand | Matrix score. | sequence | function |
| --- | --- | --- | --- | --- | --- | --- |
| ERE | Nicotiana glutinos | 743 | - | 8 | ATTTTAAA |  |

>037815   
+ TTTCAGATGC TTCTCACAGA TATTCAAAGT TTGGTTGATT CAACTATTCA ACAAGTAGGA AACTAATTAG   
  
  
+ CACAAAGAAA AACAAATGGG GGGTCTGAAG ATATTTTGGG ATTAAAATAC GAGGGGTGGA TGGGTATATG   
  
  
+ TAATGTAAGA GAGGAGGAAG GTAGCATGCA GTAGCAGAAG CGATAAAAAA ACACGACAGT GAAAAGGAAC   
  
  
+ ACGAAGACGA CTTGGAATAC GGTTGGGAAG GGAATATGAG TGTTACATCG CATGTTTTTA TACAGTGGTG   
  
  
+ AGATATTTTA TTTTTAAGTT ATTAACTTTT TAACCCACAT ATCTTACCAG TAATATAATG ATACGTAGTG   
  
  
+ TACTATCTCA TATGTCGGTC ACATTAAAAA AAATTTCTCA ATTATCATTT GATGTTATAT TTGACACGTC   
  
  
+ AATTCGTCTC TTTGATTAAT AATTATATAA ATTATTTATC ACATCATATA ATACACTGTA CACACCAATA   
  
  
+ TAATATAAAC ATAAACATGT GATTTTCCTG ATATTACTAT GAATGAAATT ATATACAATA TTATTTATAA   
  
  
+ TGTTGTCCTC AAGGTGGAGA TTTCGAGTTC AAATTATACA TATACAAGAG ATTCATGCAA GTATTCAATA   
  
  
+ TTAAATTGTG ATCACCCCTT AATCATTTGT GAAAAAGAAA AAGTTAACTT GCCAATCTCA TTCCTCTTAA   
  
  
+ TAATTAACAA TTTAGCTTAT TAATGCTATC ATTTGTTAAA AATTTAAAAT ATGAGAACTT TTATGATACT   
  
  
+ TTTTTATAGA AGTTAATAGT ATCTCGTTTT AATAAAATTA TTTGCGGACG TTATGTAGTT ACTCATCATC   
  
  
+ ATTATTTGCA TTCGGAAATC CTATCTTCAC GTTTAACGAT TTTACAACAT GATGTATGAA TTAGAAGAAA   
  
  
+ AATAATGTTA TTACGCAAGA ATTCTTCTCT CTCCAAACCT CTATAACTCT CCCTCCCCTC TCTTTCATTT   
  
  
+ GGTGCAGGTG GTGGCGCGAA GATGTCCTTT TAGCAGAGGT GGGGTGGCTA AAAGAAATAG GGGTACTGCG   
  
  
+ TTTTGGATTC TGGGTTGTGA GTTCTGGGTT CTAAATTTAT TCGTTAACTC CAGCTTCTCG TCCTACTTCG   
  
  
+ GGTTCAGATC TTGCTGTTTC GTCTTCATCC TCTGTCATTG CCTGTCAAAG TCGACGATTG CGTTGGTCCT   
  
  
+ CGTCAGGTTC AGATCTCACT CCTCATTTTC CCTTCGGATT CCTATTTTTT CAACTTTCTG AAATTTATGC   
  
  
+ CAATCTTTTT CGATATTTTT CTGGGTTTTG TATGAACATA TGTGGGTTGA AGTTGAAGAG GGGAGGGAGA   
  
  
+ GTTATAGAGG TTTGGAGAGA GAAGAAGATG AAAGAGAGTG GGGAAGAACG AGGAGGGGGA AAAGAAAGAT   
  
  
+ ACTTTCATGG TTTAGGGAGT GTGTGAAAGT TTGAGAAGGT GTTGGGTGAG AGTGAGAGGG AGAGAGTGTT   
  
  
+ GCGGCAGCTA TGGGTGGATC TGGGTTCTT  

- AAAGTCTACG AAGAGTGTCT ATAAGTTTCA AACCAACTAA GTTGATAAGT TGTTCATCCT TTGATTAATC   
  
  
- GTGTTTCTTT TTGTTTACCC CCCAGACTTC TATAAAACCC TAATTTTATG CTCCCCACCT ACCCATATAC   
  
  
- ATTACATTCT CTCCTCCTTC CATCGTACGT CATCGTCTTC GCTATTTTTT TGTGCTGTCA CTTTTCCTTG   
  
  
- TGCTTCTGCT GAACCTTATG CCAACCCTTC CCTTATACTC ACAATGTAGC GTACAAAAAT ATGTCACCAC   
  
  
- TCTATAAAAT AAAAATTCAA TAATTGAAAA ATTGGGTGTA TAGAATGGTC ATTATATTAC TATGCATCAC   
  
  
- ATGATAGAGT ATACAGCCAG TGTAATTTTT TTTAAAGAGT TAATAGTAAA CTACAATATA AACTGTGCAG   
  
  
- TTAAGCAGAG AAACTAATTA TTAATATATT TAATAAATAG TGTAGTATAT TATGTGACAT GTGTGGTTAT   
  
  
- ATTATATTTG TATTTGTACA CTAAAAGGAC TATAATGATA CTTACTTTAA TATATGTTAT AATAAATATT   
  
  
- ACAACAGGAG TTCCACCTCT AAAGCTCAAG TTTAATATGT ATATGTTCTC TAAGTACGTT CATAAGTTAT   
  
  
- AATTTAACAC TAGTGGGGAA TTAGTAAACA CTTTTTCTTT TTCAATTGAA CGGTTAGAGT AAGGAGAATT   
  
  
- ATTAATTGTT AAATCGAATA ATTACGATAG TAAACAATTT TTAAATTTTA TACTCTTGAA AATACTATGA   
  
  
- AAAAATATCT TCAATTATCA TAGAGCAAAA TTATTTTAAT AAACGCCTGC AATACATCAA TGAGTAGTAG   
  
  
- TAATAAACGT AAGCCTTTAG GATAGAAGTG CAAATTGCTA AAATGTTGTA CTACATACTT AATCTTCTTT   
  
  
- TTATTACAAT AATGCGTTCT TAAGAAGAGA GAGGTTTGGA GATATTGAGA GGGAGGGGAG AGAAAGTAAA   
  
  
- CCACGTCCAC CACCGCGCTT CTACAGGAAA ATCGTCTCCA CCCCACCGAT TTTCTTTATC CCCATGACGC   
  
  
- AAAACCTAAG ACCCAACACT CAAGACCCAA GATTTAAATA AGCAATTGAG GTCGAAGAGC AGGATGAAGC   
  
  
- CCAAGTCTAG AACGACAAAG CAGAAGTAGG AGACAGTAAC GGACAGTTTC AGCTGCTAAC GCAACCAGGA   
  
  
- GCAGTCCAAG TCTAGAGTGA GGAGTAAAAG GGAAGCCTAA GGATAAAAAA GTTGAAAGAC TTTAAATACG   
  
  
- GTTAGAAAAA GCTATAAAAA GACCCAAAAC ATACTTGTAT ACACCCAACT TCAACTTCTC CCCTCCCTCT   
  
  
- CAATATCTCC AAACCTCTCT CTTCTTCTAC TTTCTCTCAC CCCTTCTTGC TCCTCCCCCT TTTCTTTCTA   
  
  
- TGAAAGTACC AAATCCCTCA CACACTTTCA AACTCTTCCA CAACCCACTC TCACTCTCCC TCTCTCACAA   
  
  
- CGCCGTCGAT ACCCACCTAG ACCCAAGAA

+     G-Box

| Site Name | Organism | Position | Strand | Matrix score. | sequence | function |
| --- | --- | --- | --- | --- | --- | --- |
| G-Box | Pisum sativum | 868 | + | 6 | CACGTT | cis-acting regulatory element involved in light responsiveness |

>037815   
+ TTTCAGATGC TTCTCACAGA TATTCAAAGT TTGGTTGATT CAACTATTCA ACAAGTAGGA AACTAATTAG   
  
  
+ CACAAAGAAA AACAAATGGG GGGTCTGAAG ATATTTTGGG ATTAAAATAC GAGGGGTGGA TGGGTATATG   
  
  
+ TAATGTAAGA GAGGAGGAAG GTAGCATGCA GTAGCAGAAG CGATAAAAAA ACACGACAGT GAAAAGGAAC   
  
  
+ ACGAAGACGA CTTGGAATAC GGTTGGGAAG GGAATATGAG TGTTACATCG CATGTTTTTA TACAGTGGTG   
  
  
+ AGATATTTTA TTTTTAAGTT ATTAACTTTT TAACCCACAT ATCTTACCAG TAATATAATG ATACGTAGTG   
  
  
+ TACTATCTCA TATGTCGGTC ACATTAAAAA AAATTTCTCA ATTATCATTT GATGTTATAT TTGACACGTC   
  
  
+ AATTCGTCTC TTTGATTAAT AATTATATAA ATTATTTATC ACATCATATA ATACACTGTA CACACCAATA   
  
  
+ TAATATAAAC ATAAACATGT GATTTTCCTG ATATTACTAT GAATGAAATT ATATACAATA TTATTTATAA   
  
  
+ TGTTGTCCTC AAGGTGGAGA TTTCGAGTTC AAATTATACA TATACAAGAG ATTCATGCAA GTATTCAATA   
  
  
+ TTAAATTGTG ATCACCCCTT AATCATTTGT GAAAAAGAAA AAGTTAACTT GCCAATCTCA TTCCTCTTAA   
  
  
+ TAATTAACAA TTTAGCTTAT TAATGCTATC ATTTGTTAAA AATTTAAAAT ATGAGAACTT TTATGATACT   
  
  
+ TTTTTATAGA AGTTAATAGT ATCTCGTTTT AATAAAATTA TTTGCGGACG TTATGTAGTT ACTCATCATC   
  
  
+ ATTATTTGCA TTCGGAAATC CTATCTTCAC GTTTAACGAT TTTACAACAT GATGTATGAA TTAGAAGAAA   
  
  
+ AATAATGTTA TTACGCAAGA ATTCTTCTCT CTCCAAACCT CTATAACTCT CCCTCCCCTC TCTTTCATTT   
  
  
+ GGTGCAGGTG GTGGCGCGAA GATGTCCTTT TAGCAGAGGT GGGGTGGCTA AAAGAAATAG GGGTACTGCG   
  
  
+ TTTTGGATTC TGGGTTGTGA GTTCTGGGTT CTAAATTTAT TCGTTAACTC CAGCTTCTCG TCCTACTTCG   
  
  
+ GGTTCAGATC TTGCTGTTTC GTCTTCATCC TCTGTCATTG CCTGTCAAAG TCGACGATTG CGTTGGTCCT   
  
  
+ CGTCAGGTTC AGATCTCACT CCTCATTTTC CCTTCGGATT CCTATTTTTT CAACTTTCTG AAATTTATGC   
  
  
+ CAATCTTTTT CGATATTTTT CTGGGTTTTG TATGAACATA TGTGGGTTGA AGTTGAAGAG GGGAGGGAGA   
  
  
+ GTTATAGAGG TTTGGAGAGA GAAGAAGATG AAAGAGAGTG GGGAAGAACG AGGAGGGGGA AAAGAAAGAT   
  
  
+ ACTTTCATGG TTTAGGGAGT GTGTGAAAGT TTGAGAAGGT GTTGGGTGAG AGTGAGAGGG AGAGAGTGTT   
  
  
+ GCGGCAGCTA TGGGTGGATC TGGGTTCTT  

- AAAGTCTACG AAGAGTGTCT ATAAGTTTCA AACCAACTAA GTTGATAAGT TGTTCATCCT TTGATTAATC   
  
  
- GTGTTTCTTT TTGTTTACCC CCCAGACTTC TATAAAACCC TAATTTTATG CTCCCCACCT ACCCATATAC   
  
  
- ATTACATTCT CTCCTCCTTC CATCGTACGT CATCGTCTTC GCTATTTTTT TGTGCTGTCA CTTTTCCTTG   
  
  
- TGCTTCTGCT GAACCTTATG CCAACCCTTC CCTTATACTC ACAATGTAGC GTACAAAAAT ATGTCACCAC   
  
  
- TCTATAAAAT AAAAATTCAA TAATTGAAAA ATTGGGTGTA TAGAATGGTC ATTATATTAC TATGCATCAC   
  
  
- ATGATAGAGT ATACAGCCAG TGTAATTTTT TTTAAAGAGT TAATAGTAAA CTACAATATA AACTGTGCAG   
  
  
- TTAAGCAGAG AAACTAATTA TTAATATATT TAATAAATAG TGTAGTATAT TATGTGACAT GTGTGGTTAT   
  
  
- ATTATATTTG TATTTGTACA CTAAAAGGAC TATAATGATA CTTACTTTAA TATATGTTAT AATAAATATT   
  
  
- ACAACAGGAG TTCCACCTCT AAAGCTCAAG TTTAATATGT ATATGTTCTC TAAGTACGTT CATAAGTTAT   
  
  
- AATTTAACAC TAGTGGGGAA TTAGTAAACA CTTTTTCTTT TTCAATTGAA CGGTTAGAGT AAGGAGAATT   
  
  
- ATTAATTGTT AAATCGAATA ATTACGATAG TAAACAATTT TTAAATTTTA TACTCTTGAA AATACTATGA   
  
  
- AAAAATATCT TCAATTATCA TAGAGCAAAA TTATTTTAAT AAACGCCTGC AATACATCAA TGAGTAGTAG   
  
  
- TAATAAACGT AAGCCTTTAG GATAGAAGTG CAAATTGCTA AAATGTTGTA CTACATACTT AATCTTCTTT   
  
  
- TTATTACAAT AATGCGTTCT TAAGAAGAGA GAGGTTTGGA GATATTGAGA GGGAGGGGAG AGAAAGTAAA   
  
  
- CCACGTCCAC CACCGCGCTT CTACAGGAAA ATCGTCTCCA CCCCACCGAT TTTCTTTATC CCCATGACGC   
  
  
- AAAACCTAAG ACCCAACACT CAAGACCCAA GATTTAAATA AGCAATTGAG GTCGAAGAGC AGGATGAAGC   
  
  
- CCAAGTCTAG AACGACAAAG CAGAAGTAGG AGACAGTAAC GGACAGTTTC AGCTGCTAAC GCAACCAGGA   
  
  
- GCAGTCCAAG TCTAGAGTGA GGAGTAAAAG GGAAGCCTAA GGATAAAAAA GTTGAAAGAC TTTAAATACG   
  
  
- GTTAGAAAAA GCTATAAAAA GACCCAAAAC ATACTTGTAT ACACCCAACT TCAACTTCTC CCCTCCCTCT   
  
  
- CAATATCTCC AAACCTCTCT CTTCTTCTAC TTTCTCTCAC CCCTTCTTGC TCCTCCCCCT TTTCTTTCTA   
  
  
- TGAAAGTACC AAATCCCTCA CACACTTTCA AACTCTTCCA CAACCCACTC TCACTCTCCC TCTCTCACAA   
  
  
- CGCCGTCGAT ACCCACCTAG ACCCAAGAA

+     G-box

| Site Name | Organism | Position | Strand | Matrix score. | sequence | function |
| --- | --- | --- | --- | --- | --- | --- |
| G-box | Brassica oleracea | 868 | - | 8 | TAAACGTG | cis-acting regulatory element involved in light responsiveness |
| G-box | Zea mays | 192 | + | 6 | CACGAC | cis-acting regulatory element involved in light responsiveness |
| G-box | Zea mays | 415 | + | 6 | CACGTC | cis-acting regulatory element involved in light responsiveness |

>037815   
+ TTTCAGATGC TTCTCACAGA TATTCAAAGT TTGGTTGATT CAACTATTCA ACAAGTAGGA AACTAATTAG   
  
  
+ CACAAAGAAA AACAAATGGG GGGTCTGAAG ATATTTTGGG ATTAAAATAC GAGGGGTGGA TGGGTATATG   
  
  
+ TAATGTAAGA GAGGAGGAAG GTAGCATGCA GTAGCAGAAG CGATAAAAAA ACACGACAGT GAAAAGGAAC   
  
  
+ ACGAAGACGA CTTGGAATAC GGTTGGGAAG GGAATATGAG TGTTACATCG CATGTTTTTA TACAGTGGTG   
  
  
+ AGATATTTTA TTTTTAAGTT ATTAACTTTT TAACCCACAT ATCTTACCAG TAATATAATG ATACGTAGTG   
  
  
+ TACTATCTCA TATGTCGGTC ACATTAAAAA AAATTTCTCA ATTATCATTT GATGTTATAT TTGACACGTC   
  
  
+ AATTCGTCTC TTTGATTAAT AATTATATAA ATTATTTATC ACATCATATA ATACACTGTA CACACCAATA   
  
  
+ TAATATAAAC ATAAACATGT GATTTTCCTG ATATTACTAT GAATGAAATT ATATACAATA TTATTTATAA   
  
  
+ TGTTGTCCTC AAGGTGGAGA TTTCGAGTTC AAATTATACA TATACAAGAG ATTCATGCAA GTATTCAATA   
  
  
+ TTAAATTGTG ATCACCCCTT AATCATTTGT GAAAAAGAAA AAGTTAACTT GCCAATCTCA TTCCTCTTAA   
  
  
+ TAATTAACAA TTTAGCTTAT TAATGCTATC ATTTGTTAAA AATTTAAAAT ATGAGAACTT TTATGATACT   
  
  
+ TTTTTATAGA AGTTAATAGT ATCTCGTTTT AATAAAATTA TTTGCGGACG TTATGTAGTT ACTCATCATC   
  
  
+ ATTATTTGCA TTCGGAAATC CTATCTTCAC GTTTAACGAT TTTACAACAT GATGTATGAA TTAGAAGAAA   
  
  
+ AATAATGTTA TTACGCAAGA ATTCTTCTCT CTCCAAACCT CTATAACTCT CCCTCCCCTC TCTTTCATTT   
  
  
+ GGTGCAGGTG GTGGCGCGAA GATGTCCTTT TAGCAGAGGT GGGGTGGCTA AAAGAAATAG GGGTACTGCG   
  
  
+ TTTTGGATTC TGGGTTGTGA GTTCTGGGTT CTAAATTTAT TCGTTAACTC CAGCTTCTCG TCCTACTTCG   
  
  
+ GGTTCAGATC TTGCTGTTTC GTCTTCATCC TCTGTCATTG CCTGTCAAAG TCGACGATTG CGTTGGTCCT   
  
  
+ CGTCAGGTTC AGATCTCACT CCTCATTTTC CCTTCGGATT CCTATTTTTT CAACTTTCTG AAATTTATGC   
  
  
+ CAATCTTTTT CGATATTTTT CTGGGTTTTG TATGAACATA TGTGGGTTGA AGTTGAAGAG GGGAGGGAGA   
  
  
+ GTTATAGAGG TTTGGAGAGA GAAGAAGATG AAAGAGAGTG GGGAAGAACG AGGAGGGGGA AAAGAAAGAT   
  
  
+ ACTTTCATGG TTTAGGGAGT GTGTGAAAGT TTGAGAAGGT GTTGGGTGAG AGTGAGAGGG AGAGAGTGTT   
  
  
+ GCGGCAGCTA TGGGTGGATC TGGGTTCTT  

- AAAGTCTACG AAGAGTGTCT ATAAGTTTCA AACCAACTAA GTTGATAAGT TGTTCATCCT TTGATTAATC   
  
  
- GTGTTTCTTT TTGTTTACCC CCCAGACTTC TATAAAACCC TAATTTTATG CTCCCCACCT ACCCATATAC   
  
  
- ATTACATTCT CTCCTCCTTC CATCGTACGT CATCGTCTTC GCTATTTTTT TGTGCTGTCA CTTTTCCTTG   
  
  
- TGCTTCTGCT GAACCTTATG CCAACCCTTC CCTTATACTC ACAATGTAGC GTACAAAAAT ATGTCACCAC   
  
  
- TCTATAAAAT AAAAATTCAA TAATTGAAAA ATTGGGTGTA TAGAATGGTC ATTATATTAC TATGCATCAC   
  
  
- ATGATAGAGT ATACAGCCAG TGTAATTTTT TTTAAAGAGT TAATAGTAAA CTACAATATA AACTGTGCAG   
  
  
- TTAAGCAGAG AAACTAATTA TTAATATATT TAATAAATAG TGTAGTATAT TATGTGACAT GTGTGGTTAT   
  
  
- ATTATATTTG TATTTGTACA CTAAAAGGAC TATAATGATA CTTACTTTAA TATATGTTAT AATAAATATT   
  
  
- ACAACAGGAG TTCCACCTCT AAAGCTCAAG TTTAATATGT ATATGTTCTC TAAGTACGTT CATAAGTTAT   
  
  
- AATTTAACAC TAGTGGGGAA TTAGTAAACA CTTTTTCTTT TTCAATTGAA CGGTTAGAGT AAGGAGAATT   
  
  
- ATTAATTGTT AAATCGAATA ATTACGATAG TAAACAATTT TTAAATTTTA TACTCTTGAA AATACTATGA   
  
  
- AAAAATATCT TCAATTATCA TAGAGCAAAA TTATTTTAAT AAACGCCTGC AATACATCAA TGAGTAGTAG   
  
  
- TAATAAACGT AAGCCTTTAG GATAGAAGTG CAAATTGCTA AAATGTTGTA CTACATACTT AATCTTCTTT   
  
  
- TTATTACAAT AATGCGTTCT TAAGAAGAGA GAGGTTTGGA GATATTGAGA GGGAGGGGAG AGAAAGTAAA   
  
  
- CCACGTCCAC CACCGCGCTT CTACAGGAAA ATCGTCTCCA CCCCACCGAT TTTCTTTATC CCCATGACGC   
  
  
- AAAACCTAAG ACCCAACACT CAAGACCCAA GATTTAAATA AGCAATTGAG GTCGAAGAGC AGGATGAAGC   
  
  
- CCAAGTCTAG AACGACAAAG CAGAAGTAGG AGACAGTAAC GGACAGTTTC AGCTGCTAAC GCAACCAGGA   
  
  
- GCAGTCCAAG TCTAGAGTGA GGAGTAAAAG GGAAGCCTAA GGATAAAAAA GTTGAAAGAC TTTAAATACG   
  
  
- GTTAGAAAAA GCTATAAAAA GACCCAAAAC ATACTTGTAT ACACCCAACT TCAACTTCTC CCCTCCCTCT   
  
  
- CAATATCTCC AAACCTCTCT CTTCTTCTAC TTTCTCTCAC CCCTTCTTGC TCCTCCCCCT TTTCTTTCTA   
  
  
- TGAAAGTACC AAATCCCTCA CACACTTTCA AACTCTTCCA CAACCCACTC TCACTCTCCC TCTCTCACAA   
  
  
- CGCCGTCGAT ACCCACCTAG ACCCAAGAA

+     GATA-motif

| Site Name | Organism | Position | Strand | Matrix score. | sequence | function |
| --- | --- | --- | --- | --- | --- | --- |
| GATA-motif | Arabidopsis thaliana | 859 | - | 7 | GATAGGA | part of a light responsive element |
| GATA-motif | Arabidopsis thaliana | 857 | - | 10 | AAGATAAGATT | part of a light responsive element |

>037815   
+ TTTCAGATGC TTCTCACAGA TATTCAAAGT TTGGTTGATT CAACTATTCA ACAAGTAGGA AACTAATTAG   
  
  
+ CACAAAGAAA AACAAATGGG GGGTCTGAAG ATATTTTGGG ATTAAAATAC GAGGGGTGGA TGGGTATATG   
  
  
+ TAATGTAAGA GAGGAGGAAG GTAGCATGCA GTAGCAGAAG CGATAAAAAA ACACGACAGT GAAAAGGAAC   
  
  
+ ACGAAGACGA CTTGGAATAC GGTTGGGAAG GGAATATGAG TGTTACATCG CATGTTTTTA TACAGTGGTG   
  
  
+ AGATATTTTA TTTTTAAGTT ATTAACTTTT TAACCCACAT ATCTTACCAG TAATATAATG ATACGTAGTG   
  
  
+ TACTATCTCA TATGTCGGTC ACATTAAAAA AAATTTCTCA ATTATCATTT GATGTTATAT TTGACACGTC   
  
  
+ AATTCGTCTC TTTGATTAAT AATTATATAA ATTATTTATC ACATCATATA ATACACTGTA CACACCAATA   
  
  
+ TAATATAAAC ATAAACATGT GATTTTCCTG ATATTACTAT GAATGAAATT ATATACAATA TTATTTATAA   
  
  
+ TGTTGTCCTC AAGGTGGAGA TTTCGAGTTC AAATTATACA TATACAAGAG ATTCATGCAA GTATTCAATA   
  
  
+ TTAAATTGTG ATCACCCCTT AATCATTTGT GAAAAAGAAA AAGTTAACTT GCCAATCTCA TTCCTCTTAA   
  
  
+ TAATTAACAA TTTAGCTTAT TAATGCTATC ATTTGTTAAA AATTTAAAAT ATGAGAACTT TTATGATACT   
  
  
+ TTTTTATAGA AGTTAATAGT ATCTCGTTTT AATAAAATTA TTTGCGGACG TTATGTAGTT ACTCATCATC   
  
  
+ ATTATTTGCA TTCGGAAATC CTATCTTCAC GTTTAACGAT TTTACAACAT GATGTATGAA TTAGAAGAAA   
  
  
+ AATAATGTTA TTACGCAAGA ATTCTTCTCT CTCCAAACCT CTATAACTCT CCCTCCCCTC TCTTTCATTT   
  
  
+ GGTGCAGGTG GTGGCGCGAA GATGTCCTTT TAGCAGAGGT GGGGTGGCTA AAAGAAATAG GGGTACTGCG   
  
  
+ TTTTGGATTC TGGGTTGTGA GTTCTGGGTT CTAAATTTAT TCGTTAACTC CAGCTTCTCG TCCTACTTCG   
  
  
+ GGTTCAGATC TTGCTGTTTC GTCTTCATCC TCTGTCATTG CCTGTCAAAG TCGACGATTG CGTTGGTCCT   
  
  
+ CGTCAGGTTC AGATCTCACT CCTCATTTTC CCTTCGGATT CCTATTTTTT CAACTTTCTG AAATTTATGC   
  
  
+ CAATCTTTTT CGATATTTTT CTGGGTTTTG TATGAACATA TGTGGGTTGA AGTTGAAGAG GGGAGGGAGA   
  
  
+ GTTATAGAGG TTTGGAGAGA GAAGAAGATG AAAGAGAGTG GGGAAGAACG AGGAGGGGGA AAAGAAAGAT   
  
  
+ ACTTTCATGG TTTAGGGAGT GTGTGAAAGT TTGAGAAGGT GTTGGGTGAG AGTGAGAGGG AGAGAGTGTT   
  
  
+ GCGGCAGCTA TGGGTGGATC TGGGTTCTT  

- AAAGTCTACG AAGAGTGTCT ATAAGTTTCA AACCAACTAA GTTGATAAGT TGTTCATCCT TTGATTAATC   
  
  
- GTGTTTCTTT TTGTTTACCC CCCAGACTTC TATAAAACCC TAATTTTATG CTCCCCACCT ACCCATATAC   
  
  
- ATTACATTCT CTCCTCCTTC CATCGTACGT CATCGTCTTC GCTATTTTTT TGTGCTGTCA CTTTTCCTTG   
  
  
- TGCTTCTGCT GAACCTTATG CCAACCCTTC CCTTATACTC ACAATGTAGC GTACAAAAAT ATGTCACCAC   
  
  
- TCTATAAAAT AAAAATTCAA TAATTGAAAA ATTGGGTGTA TAGAATGGTC ATTATATTAC TATGCATCAC   
  
  
- ATGATAGAGT ATACAGCCAG TGTAATTTTT TTTAAAGAGT TAATAGTAAA CTACAATATA AACTGTGCAG   
  
  
- TTAAGCAGAG AAACTAATTA TTAATATATT TAATAAATAG TGTAGTATAT TATGTGACAT GTGTGGTTAT   
  
  
- ATTATATTTG TATTTGTACA CTAAAAGGAC TATAATGATA CTTACTTTAA TATATGTTAT AATAAATATT   
  
  
- ACAACAGGAG TTCCACCTCT AAAGCTCAAG TTTAATATGT ATATGTTCTC TAAGTACGTT CATAAGTTAT   
  
  
- AATTTAACAC TAGTGGGGAA TTAGTAAACA CTTTTTCTTT TTCAATTGAA CGGTTAGAGT AAGGAGAATT   
  
  
- ATTAATTGTT AAATCGAATA ATTACGATAG TAAACAATTT TTAAATTTTA TACTCTTGAA AATACTATGA   
  
  
- AAAAATATCT TCAATTATCA TAGAGCAAAA TTATTTTAAT AAACGCCTGC AATACATCAA TGAGTAGTAG   
  
  
- TAATAAACGT AAGCCTTTAG GATAGAAGTG CAAATTGCTA AAATGTTGTA CTACATACTT AATCTTCTTT   
  
  
- TTATTACAAT AATGCGTTCT TAAGAAGAGA GAGGTTTGGA GATATTGAGA GGGAGGGGAG AGAAAGTAAA   
  
  
- CCACGTCCAC CACCGCGCTT CTACAGGAAA ATCGTCTCCA CCCCACCGAT TTTCTTTATC CCCATGACGC   
  
  
- AAAACCTAAG ACCCAACACT CAAGACCCAA GATTTAAATA AGCAATTGAG GTCGAAGAGC AGGATGAAGC   
  
  
- CCAAGTCTAG AACGACAAAG CAGAAGTAGG AGACAGTAAC GGACAGTTTC AGCTGCTAAC GCAACCAGGA   
  
  
- GCAGTCCAAG TCTAGAGTGA GGAGTAAAAG GGAAGCCTAA GGATAAAAAA GTTGAAAGAC TTTAAATACG   
  
  
- GTTAGAAAAA GCTATAAAAA GACCCAAAAC ATACTTGTAT ACACCCAACT TCAACTTCTC CCCTCCCTCT   
  
  
- CAATATCTCC AAACCTCTCT CTTCTTCTAC TTTCTCTCAC CCCTTCTTGC TCCTCCCCCT TTTCTTTCTA   
  
  
- TGAAAGTACC AAATCCCTCA CACACTTTCA AACTCTTCCA CAACCCACTC TCACTCTCCC TCTCTCACAA   
  
  
- CGCCGTCGAT ACCCACCTAG ACCCAAGAA

+     GT1-motif

| Site Name | Organism | Position | Strand | Matrix score. | sequence | function |
| --- | --- | --- | --- | --- | --- | --- |
| GT1-motif | Arabidopsis thaliana | 310 | - | 6 | GGTTAA | light responsive element |
| GT1-motif | Solanum tuberosum | 1419 | + | 9 | GTGTGTGAA | light responsive element |

>037815   
+ TTTCAGATGC TTCTCACAGA TATTCAAAGT TTGGTTGATT CAACTATTCA ACAAGTAGGA AACTAATTAG   
  
  
+ CACAAAGAAA AACAAATGGG GGGTCTGAAG ATATTTTGGG ATTAAAATAC GAGGGGTGGA TGGGTATATG   
  
  
+ TAATGTAAGA GAGGAGGAAG GTAGCATGCA GTAGCAGAAG CGATAAAAAA ACACGACAGT GAAAAGGAAC   
  
  
+ ACGAAGACGA CTTGGAATAC GGTTGGGAAG GGAATATGAG TGTTACATCG CATGTTTTTA TACAGTGGTG   
  
  
+ AGATATTTTA TTTTTAAGTT ATTAACTTTT TAACCCACAT ATCTTACCAG TAATATAATG ATACGTAGTG   
  
  
+ TACTATCTCA TATGTCGGTC ACATTAAAAA AAATTTCTCA ATTATCATTT GATGTTATAT TTGACACGTC   
  
  
+ AATTCGTCTC TTTGATTAAT AATTATATAA ATTATTTATC ACATCATATA ATACACTGTA CACACCAATA   
  
  
+ TAATATAAAC ATAAACATGT GATTTTCCTG ATATTACTAT GAATGAAATT ATATACAATA TTATTTATAA   
  
  
+ TGTTGTCCTC AAGGTGGAGA TTTCGAGTTC AAATTATACA TATACAAGAG ATTCATGCAA GTATTCAATA   
  
  
+ TTAAATTGTG ATCACCCCTT AATCATTTGT GAAAAAGAAA AAGTTAACTT GCCAATCTCA TTCCTCTTAA   
  
  
+ TAATTAACAA TTTAGCTTAT TAATGCTATC ATTTGTTAAA AATTTAAAAT ATGAGAACTT TTATGATACT   
  
  
+ TTTTTATAGA AGTTAATAGT ATCTCGTTTT AATAAAATTA TTTGCGGACG TTATGTAGTT ACTCATCATC   
  
  
+ ATTATTTGCA TTCGGAAATC CTATCTTCAC GTTTAACGAT TTTACAACAT GATGTATGAA TTAGAAGAAA   
  
  
+ AATAATGTTA TTACGCAAGA ATTCTTCTCT CTCCAAACCT CTATAACTCT CCCTCCCCTC TCTTTCATTT   
  
  
+ GGTGCAGGTG GTGGCGCGAA GATGTCCTTT TAGCAGAGGT GGGGTGGCTA AAAGAAATAG GGGTACTGCG   
  
  
+ TTTTGGATTC TGGGTTGTGA GTTCTGGGTT CTAAATTTAT TCGTTAACTC CAGCTTCTCG TCCTACTTCG   
  
  
+ GGTTCAGATC TTGCTGTTTC GTCTTCATCC TCTGTCATTG CCTGTCAAAG TCGACGATTG CGTTGGTCCT   
  
  
+ CGTCAGGTTC AGATCTCACT CCTCATTTTC CCTTCGGATT CCTATTTTTT CAACTTTCTG AAATTTATGC   
  
  
+ CAATCTTTTT CGATATTTTT CTGGGTTTTG TATGAACATA TGTGGGTTGA AGTTGAAGAG GGGAGGGAGA   
  
  
+ GTTATAGAGG TTTGGAGAGA GAAGAAGATG AAAGAGAGTG GGGAAGAACG AGGAGGGGGA AAAGAAAGAT   
  
  
+ ACTTTCATGG TTTAGGGAGT GTGTGAAAGT TTGAGAAGGT GTTGGGTGAG AGTGAGAGGG AGAGAGTGTT   
  
  
+ GCGGCAGCTA TGGGTGGATC TGGGTTCTT  

- AAAGTCTACG AAGAGTGTCT ATAAGTTTCA AACCAACTAA GTTGATAAGT TGTTCATCCT TTGATTAATC   
  
  
- GTGTTTCTTT TTGTTTACCC CCCAGACTTC TATAAAACCC TAATTTTATG CTCCCCACCT ACCCATATAC   
  
  
- ATTACATTCT CTCCTCCTTC CATCGTACGT CATCGTCTTC GCTATTTTTT TGTGCTGTCA CTTTTCCTTG   
  
  
- TGCTTCTGCT GAACCTTATG CCAACCCTTC CCTTATACTC ACAATGTAGC GTACAAAAAT ATGTCACCAC   
  
  
- TCTATAAAAT AAAAATTCAA TAATTGAAAA ATTGGGTGTA TAGAATGGTC ATTATATTAC TATGCATCAC   
  
  
- ATGATAGAGT ATACAGCCAG TGTAATTTTT TTTAAAGAGT TAATAGTAAA CTACAATATA AACTGTGCAG   
  
  
- TTAAGCAGAG AAACTAATTA TTAATATATT TAATAAATAG TGTAGTATAT TATGTGACAT GTGTGGTTAT   
  
  
- ATTATATTTG TATTTGTACA CTAAAAGGAC TATAATGATA CTTACTTTAA TATATGTTAT AATAAATATT   
  
  
- ACAACAGGAG TTCCACCTCT AAAGCTCAAG TTTAATATGT ATATGTTCTC TAAGTACGTT CATAAGTTAT   
  
  
- AATTTAACAC TAGTGGGGAA TTAGTAAACA CTTTTTCTTT TTCAATTGAA CGGTTAGAGT AAGGAGAATT   
  
  
- ATTAATTGTT AAATCGAATA ATTACGATAG TAAACAATTT TTAAATTTTA TACTCTTGAA AATACTATGA   
  
  
- AAAAATATCT TCAATTATCA TAGAGCAAAA TTATTTTAAT AAACGCCTGC AATACATCAA TGAGTAGTAG   
  
  
- TAATAAACGT AAGCCTTTAG GATAGAAGTG CAAATTGCTA AAATGTTGTA CTACATACTT AATCTTCTTT   
  
  
- TTATTACAAT AATGCGTTCT TAAGAAGAGA GAGGTTTGGA GATATTGAGA GGGAGGGGAG AGAAAGTAAA   
  
  
- CCACGTCCAC CACCGCGCTT CTACAGGAAA ATCGTCTCCA CCCCACCGAT TTTCTTTATC CCCATGACGC   
  
  
- AAAACCTAAG ACCCAACACT CAAGACCCAA GATTTAAATA AGCAATTGAG GTCGAAGAGC AGGATGAAGC   
  
  
- CCAAGTCTAG AACGACAAAG CAGAAGTAGG AGACAGTAAC GGACAGTTTC AGCTGCTAAC GCAACCAGGA   
  
  
- GCAGTCCAAG TCTAGAGTGA GGAGTAAAAG GGAAGCCTAA GGATAAAAAA GTTGAAAGAC TTTAAATACG   
  
  
- GTTAGAAAAA GCTATAAAAA GACCCAAAAC ATACTTGTAT ACACCCAACT TCAACTTCTC CCCTCCCTCT   
  
  
- CAATATCTCC AAACCTCTCT CTTCTTCTAC TTTCTCTCAC CCCTTCTTGC TCCTCCCCCT TTTCTTTCTA   
  
  
- TGAAAGTACC AAATCCCTCA CACACTTTCA AACTCTTCCA CAACCCACTC TCACTCTCCC TCTCTCACAA   
  
  
- CGCCGTCGAT ACCCACCTAG ACCCAAGAA

+     MYB

| Site Name | Organism | Position | Strand | Matrix score. | sequence | function |
| --- | --- | --- | --- | --- | --- | --- |
| MYB | Arabidopsis thaliana | 32 | - | 6 | CAACCA |  |

>037815   
+ TTTCAGATGC TTCTCACAGA TATTCAAAGT TTGGTTGATT CAACTATTCA ACAAGTAGGA AACTAATTAG   
  
  
+ CACAAAGAAA AACAAATGGG GGGTCTGAAG ATATTTTGGG ATTAAAATAC GAGGGGTGGA TGGGTATATG   
  
  
+ TAATGTAAGA GAGGAGGAAG GTAGCATGCA GTAGCAGAAG CGATAAAAAA ACACGACAGT GAAAAGGAAC   
  
  
+ ACGAAGACGA CTTGGAATAC GGTTGGGAAG GGAATATGAG TGTTACATCG CATGTTTTTA TACAGTGGTG   
  
  
+ AGATATTTTA TTTTTAAGTT ATTAACTTTT TAACCCACAT ATCTTACCAG TAATATAATG ATACGTAGTG   
  
  
+ TACTATCTCA TATGTCGGTC ACATTAAAAA AAATTTCTCA ATTATCATTT GATGTTATAT TTGACACGTC   
  
  
+ AATTCGTCTC TTTGATTAAT AATTATATAA ATTATTTATC ACATCATATA ATACACTGTA CACACCAATA   
  
  
+ TAATATAAAC ATAAACATGT GATTTTCCTG ATATTACTAT GAATGAAATT ATATACAATA TTATTTATAA   
  
  
+ TGTTGTCCTC AAGGTGGAGA TTTCGAGTTC AAATTATACA TATACAAGAG ATTCATGCAA GTATTCAATA   
  
  
+ TTAAATTGTG ATCACCCCTT AATCATTTGT GAAAAAGAAA AAGTTAACTT GCCAATCTCA TTCCTCTTAA   
  
  
+ TAATTAACAA TTTAGCTTAT TAATGCTATC ATTTGTTAAA AATTTAAAAT ATGAGAACTT TTATGATACT   
  
  
+ TTTTTATAGA AGTTAATAGT ATCTCGTTTT AATAAAATTA TTTGCGGACG TTATGTAGTT ACTCATCATC   
  
  
+ ATTATTTGCA TTCGGAAATC CTATCTTCAC GTTTAACGAT TTTACAACAT GATGTATGAA TTAGAAGAAA   
  
  
+ AATAATGTTA TTACGCAAGA ATTCTTCTCT CTCCAAACCT CTATAACTCT CCCTCCCCTC TCTTTCATTT   
  
  
+ GGTGCAGGTG GTGGCGCGAA GATGTCCTTT TAGCAGAGGT GGGGTGGCTA AAAGAAATAG GGGTACTGCG   
  
  
+ TTTTGGATTC TGGGTTGTGA GTTCTGGGTT CTAAATTTAT TCGTTAACTC CAGCTTCTCG TCCTACTTCG   
  
  
+ GGTTCAGATC TTGCTGTTTC GTCTTCATCC TCTGTCATTG CCTGTCAAAG TCGACGATTG CGTTGGTCCT   
  
  
+ CGTCAGGTTC AGATCTCACT CCTCATTTTC CCTTCGGATT CCTATTTTTT CAACTTTCTG AAATTTATGC   
  
  
+ CAATCTTTTT CGATATTTTT CTGGGTTTTG TATGAACATA TGTGGGTTGA AGTTGAAGAG GGGAGGGAGA   
  
  
+ GTTATAGAGG TTTGGAGAGA GAAGAAGATG AAAGAGAGTG GGGAAGAACG AGGAGGGGGA AAAGAAAGAT   
  
  
+ ACTTTCATGG TTTAGGGAGT GTGTGAAAGT TTGAGAAGGT GTTGGGTGAG AGTGAGAGGG AGAGAGTGTT   
  
  
+ GCGGCAGCTA TGGGTGGATC TGGGTTCTT  

- AAAGTCTACG AAGAGTGTCT ATAAGTTTCA AACCAACTAA GTTGATAAGT TGTTCATCCT TTGATTAATC   
  
  
- GTGTTTCTTT TTGTTTACCC CCCAGACTTC TATAAAACCC TAATTTTATG CTCCCCACCT ACCCATATAC   
  
  
- ATTACATTCT CTCCTCCTTC CATCGTACGT CATCGTCTTC GCTATTTTTT TGTGCTGTCA CTTTTCCTTG   
  
  
- TGCTTCTGCT GAACCTTATG CCAACCCTTC CCTTATACTC ACAATGTAGC GTACAAAAAT ATGTCACCAC   
  
  
- TCTATAAAAT AAAAATTCAA TAATTGAAAA ATTGGGTGTA TAGAATGGTC ATTATATTAC TATGCATCAC   
  
  
- ATGATAGAGT ATACAGCCAG TGTAATTTTT TTTAAAGAGT TAATAGTAAA CTACAATATA AACTGTGCAG   
  
  
- TTAAGCAGAG AAACTAATTA TTAATATATT TAATAAATAG TGTAGTATAT TATGTGACAT GTGTGGTTAT   
  
  
- ATTATATTTG TATTTGTACA CTAAAAGGAC TATAATGATA CTTACTTTAA TATATGTTAT AATAAATATT   
  
  
- ACAACAGGAG TTCCACCTCT AAAGCTCAAG TTTAATATGT ATATGTTCTC TAAGTACGTT CATAAGTTAT   
  
  
- AATTTAACAC TAGTGGGGAA TTAGTAAACA CTTTTTCTTT TTCAATTGAA CGGTTAGAGT AAGGAGAATT   
  
  
- ATTAATTGTT AAATCGAATA ATTACGATAG TAAACAATTT TTAAATTTTA TACTCTTGAA AATACTATGA   
  
  
- AAAAATATCT TCAATTATCA TAGAGCAAAA TTATTTTAAT AAACGCCTGC AATACATCAA TGAGTAGTAG   
  
  
- TAATAAACGT AAGCCTTTAG GATAGAAGTG CAAATTGCTA AAATGTTGTA CTACATACTT AATCTTCTTT   
  
  
- TTATTACAAT AATGCGTTCT TAAGAAGAGA GAGGTTTGGA GATATTGAGA GGGAGGGGAG AGAAAGTAAA   
  
  
- CCACGTCCAC CACCGCGCTT CTACAGGAAA ATCGTCTCCA CCCCACCGAT TTTCTTTATC CCCATGACGC   
  
  
- AAAACCTAAG ACCCAACACT CAAGACCCAA GATTTAAATA AGCAATTGAG GTCGAAGAGC AGGATGAAGC   
  
  
- CCAAGTCTAG AACGACAAAG CAGAAGTAGG AGACAGTAAC GGACAGTTTC AGCTGCTAAC GCAACCAGGA   
  
  
- GCAGTCCAAG TCTAGAGTGA GGAGTAAAAG GGAAGCCTAA GGATAAAAAA GTTGAAAGAC TTTAAATACG   
  
  
- GTTAGAAAAA GCTATAAAAA GACCCAAAAC ATACTTGTAT ACACCCAACT TCAACTTCTC CCCTCCCTCT   
  
  
- CAATATCTCC AAACCTCTCT CTTCTTCTAC TTTCTCTCAC CCCTTCTTGC TCCTCCCCCT TTTCTTTCTA   
  
  
- TGAAAGTACC AAATCCCTCA CACACTTTCA AACTCTTCCA CAACCCACTC TCACTCTCCC TCTCTCACAA   
  
  
- CGCCGTCGAT ACCCACCTAG ACCCAAGAA

+     MYC

| Site Name | Organism | Position | Strand | Matrix score. | sequence | function |
| --- | --- | --- | --- | --- | --- | --- |
| MYC | Arabidopsis thaliana | 730 | + | 6 | CATTTG |  |
| MYC | Arabidopsis thaliana | 654 | + | 6 | CATTTG |  |
| MYC | Arabidopsis thaliana | 976 | + | 6 | CATTTG |  |
| MYC | Arabidopsis thaliana | 396 | + | 6 | CATTTG |  |
| MYC | Arabidopsis thaliana | 83 | - | 6 | CATTTG |  |
| MYC | Arabidopsis thaliana | 506 | + | 6 | CATGTG |  |

>037815   
+ TTTCAGATGC TTCTCACAGA TATTCAAAGT TTGGTTGATT CAACTATTCA ACAAGTAGGA AACTAATTAG   
  
  
+ CACAAAGAAA AACAAATGGG GGGTCTGAAG ATATTTTGGG ATTAAAATAC GAGGGGTGGA TGGGTATATG   
  
  
+ TAATGTAAGA GAGGAGGAAG GTAGCATGCA GTAGCAGAAG CGATAAAAAA ACACGACAGT GAAAAGGAAC   
  
  
+ ACGAAGACGA CTTGGAATAC GGTTGGGAAG GGAATATGAG TGTTACATCG CATGTTTTTA TACAGTGGTG   
  
  
+ AGATATTTTA TTTTTAAGTT ATTAACTTTT TAACCCACAT ATCTTACCAG TAATATAATG ATACGTAGTG   
  
  
+ TACTATCTCA TATGTCGGTC ACATTAAAAA AAATTTCTCA ATTATCATTT GATGTTATAT TTGACACGTC   
  
  
+ AATTCGTCTC TTTGATTAAT AATTATATAA ATTATTTATC ACATCATATA ATACACTGTA CACACCAATA   
  
  
+ TAATATAAAC ATAAACATGT GATTTTCCTG ATATTACTAT GAATGAAATT ATATACAATA TTATTTATAA   
  
  
+ TGTTGTCCTC AAGGTGGAGA TTTCGAGTTC AAATTATACA TATACAAGAG ATTCATGCAA GTATTCAATA   
  
  
+ TTAAATTGTG ATCACCCCTT AATCATTTGT GAAAAAGAAA AAGTTAACTT GCCAATCTCA TTCCTCTTAA   
  
  
+ TAATTAACAA TTTAGCTTAT TAATGCTATC ATTTGTTAAA AATTTAAAAT ATGAGAACTT TTATGATACT   
  
  
+ TTTTTATAGA AGTTAATAGT ATCTCGTTTT AATAAAATTA TTTGCGGACG TTATGTAGTT ACTCATCATC   
  
  
+ ATTATTTGCA TTCGGAAATC CTATCTTCAC GTTTAACGAT TTTACAACAT GATGTATGAA TTAGAAGAAA   
  
  
+ AATAATGTTA TTACGCAAGA ATTCTTCTCT CTCCAAACCT CTATAACTCT CCCTCCCCTC TCTTTCATTT   
  
  
+ GGTGCAGGTG GTGGCGCGAA GATGTCCTTT TAGCAGAGGT GGGGTGGCTA AAAGAAATAG GGGTACTGCG   
  
  
+ TTTTGGATTC TGGGTTGTGA GTTCTGGGTT CTAAATTTAT TCGTTAACTC CAGCTTCTCG TCCTACTTCG   
  
  
+ GGTTCAGATC TTGCTGTTTC GTCTTCATCC TCTGTCATTG CCTGTCAAAG TCGACGATTG CGTTGGTCCT   
  
  
+ CGTCAGGTTC AGATCTCACT CCTCATTTTC CCTTCGGATT CCTATTTTTT CAACTTTCTG AAATTTATGC   
  
  
+ CAATCTTTTT CGATATTTTT CTGGGTTTTG TATGAACATA TGTGGGTTGA AGTTGAAGAG GGGAGGGAGA   
  
  
+ GTTATAGAGG TTTGGAGAGA GAAGAAGATG AAAGAGAGTG GGGAAGAACG AGGAGGGGGA AAAGAAAGAT   
  
  
+ ACTTTCATGG TTTAGGGAGT GTGTGAAAGT TTGAGAAGGT GTTGGGTGAG AGTGAGAGGG AGAGAGTGTT   
  
  
+ GCGGCAGCTA TGGGTGGATC TGGGTTCTT  

- AAAGTCTACG AAGAGTGTCT ATAAGTTTCA AACCAACTAA GTTGATAAGT TGTTCATCCT TTGATTAATC   
  
  
- GTGTTTCTTT TTGTTTACCC CCCAGACTTC TATAAAACCC TAATTTTATG CTCCCCACCT ACCCATATAC   
  
  
- ATTACATTCT CTCCTCCTTC CATCGTACGT CATCGTCTTC GCTATTTTTT TGTGCTGTCA CTTTTCCTTG   
  
  
- TGCTTCTGCT GAACCTTATG CCAACCCTTC CCTTATACTC ACAATGTAGC GTACAAAAAT ATGTCACCAC   
  
  
- TCTATAAAAT AAAAATTCAA TAATTGAAAA ATTGGGTGTA TAGAATGGTC ATTATATTAC TATGCATCAC   
  
  
- ATGATAGAGT ATACAGCCAG TGTAATTTTT TTTAAAGAGT TAATAGTAAA CTACAATATA AACTGTGCAG   
  
  
- TTAAGCAGAG AAACTAATTA TTAATATATT TAATAAATAG TGTAGTATAT TATGTGACAT GTGTGGTTAT   
  
  
- ATTATATTTG TATTTGTACA CTAAAAGGAC TATAATGATA CTTACTTTAA TATATGTTAT AATAAATATT   
  
  
- ACAACAGGAG TTCCACCTCT AAAGCTCAAG TTTAATATGT ATATGTTCTC TAAGTACGTT CATAAGTTAT   
  
  
- AATTTAACAC TAGTGGGGAA TTAGTAAACA CTTTTTCTTT TTCAATTGAA CGGTTAGAGT AAGGAGAATT   
  
  
- ATTAATTGTT AAATCGAATA ATTACGATAG TAAACAATTT TTAAATTTTA TACTCTTGAA AATACTATGA   
  
  
- AAAAATATCT TCAATTATCA TAGAGCAAAA TTATTTTAAT AAACGCCTGC AATACATCAA TGAGTAGTAG   
  
  
- TAATAAACGT AAGCCTTTAG GATAGAAGTG CAAATTGCTA AAATGTTGTA CTACATACTT AATCTTCTTT   
  
  
- TTATTACAAT AATGCGTTCT TAAGAAGAGA GAGGTTTGGA GATATTGAGA GGGAGGGGAG AGAAAGTAAA   
  
  
- CCACGTCCAC CACCGCGCTT CTACAGGAAA ATCGTCTCCA CCCCACCGAT TTTCTTTATC CCCATGACGC   
  
  
- AAAACCTAAG ACCCAACACT CAAGACCCAA GATTTAAATA AGCAATTGAG GTCGAAGAGC AGGATGAAGC   
  
  
- CCAAGTCTAG AACGACAAAG CAGAAGTAGG AGACAGTAAC GGACAGTTTC AGCTGCTAAC GCAACCAGGA   
  
  
- GCAGTCCAAG TCTAGAGTGA GGAGTAAAAG GGAAGCCTAA GGATAAAAAA GTTGAAAGAC TTTAAATACG   
  
  
- GTTAGAAAAA GCTATAAAAA GACCCAAAAC ATACTTGTAT ACACCCAACT TCAACTTCTC CCCTCCCTCT   
  
  
- CAATATCTCC AAACCTCTCT CTTCTTCTAC TTTCTCTCAC CCCTTCTTGC TCCTCCCCCT TTTCTTTCTA   
  
  
- TGAAAGTACC AAATCCCTCA CACACTTTCA AACTCTTCCA CAACCCACTC TCACTCTCCC TCTCTCACAA   
  
  
- CGCCGTCGAT ACCCACCTAG ACCCAAGAA

+     Myc

| Site Name | Organism | Position | Strand | Matrix score. | sequence | function |
| --- | --- | --- | --- | --- | --- | --- |
| Myc | Arabidopsis thaliana | 146 | - | 7 | TCTCTTA |  |

>037815   
+ TTTCAGATGC TTCTCACAGA TATTCAAAGT TTGGTTGATT CAACTATTCA ACAAGTAGGA AACTAATTAG   
  
  
+ CACAAAGAAA AACAAATGGG GGGTCTGAAG ATATTTTGGG ATTAAAATAC GAGGGGTGGA TGGGTATATG   
  
  
+ TAATGTAAGA GAGGAGGAAG GTAGCATGCA GTAGCAGAAG CGATAAAAAA ACACGACAGT GAAAAGGAAC   
  
  
+ ACGAAGACGA CTTGGAATAC GGTTGGGAAG GGAATATGAG TGTTACATCG CATGTTTTTA TACAGTGGTG   
  
  
+ AGATATTTTA TTTTTAAGTT ATTAACTTTT TAACCCACAT ATCTTACCAG TAATATAATG ATACGTAGTG   
  
  
+ TACTATCTCA TATGTCGGTC ACATTAAAAA AAATTTCTCA ATTATCATTT GATGTTATAT TTGACACGTC   
  
  
+ AATTCGTCTC TTTGATTAAT AATTATATAA ATTATTTATC ACATCATATA ATACACTGTA CACACCAATA   
  
  
+ TAATATAAAC ATAAACATGT GATTTTCCTG ATATTACTAT GAATGAAATT ATATACAATA TTATTTATAA   
  
  
+ TGTTGTCCTC AAGGTGGAGA TTTCGAGTTC AAATTATACA TATACAAGAG ATTCATGCAA GTATTCAATA   
  
  
+ TTAAATTGTG ATCACCCCTT AATCATTTGT GAAAAAGAAA AAGTTAACTT GCCAATCTCA TTCCTCTTAA   
  
  
+ TAATTAACAA TTTAGCTTAT TAATGCTATC ATTTGTTAAA AATTTAAAAT ATGAGAACTT TTATGATACT   
  
  
+ TTTTTATAGA AGTTAATAGT ATCTCGTTTT AATAAAATTA TTTGCGGACG TTATGTAGTT ACTCATCATC   
  
  
+ ATTATTTGCA TTCGGAAATC CTATCTTCAC GTTTAACGAT TTTACAACAT GATGTATGAA TTAGAAGAAA   
  
  
+ AATAATGTTA TTACGCAAGA ATTCTTCTCT CTCCAAACCT CTATAACTCT CCCTCCCCTC TCTTTCATTT   
  
  
+ GGTGCAGGTG GTGGCGCGAA GATGTCCTTT TAGCAGAGGT GGGGTGGCTA AAAGAAATAG GGGTACTGCG   
  
  
+ TTTTGGATTC TGGGTTGTGA GTTCTGGGTT CTAAATTTAT TCGTTAACTC CAGCTTCTCG TCCTACTTCG   
  
  
+ GGTTCAGATC TTGCTGTTTC GTCTTCATCC TCTGTCATTG CCTGTCAAAG TCGACGATTG CGTTGGTCCT   
  
  
+ CGTCAGGTTC AGATCTCACT CCTCATTTTC CCTTCGGATT CCTATTTTTT CAACTTTCTG AAATTTATGC   
  
  
+ CAATCTTTTT CGATATTTTT CTGGGTTTTG TATGAACATA TGTGGGTTGA AGTTGAAGAG GGGAGGGAGA   
  
  
+ GTTATAGAGG TTTGGAGAGA GAAGAAGATG AAAGAGAGTG GGGAAGAACG AGGAGGGGGA AAAGAAAGAT   
  
  
+ ACTTTCATGG TTTAGGGAGT GTGTGAAAGT TTGAGAAGGT GTTGGGTGAG AGTGAGAGGG AGAGAGTGTT   
  
  
+ GCGGCAGCTA TGGGTGGATC TGGGTTCTT  

- AAAGTCTACG AAGAGTGTCT ATAAGTTTCA AACCAACTAA GTTGATAAGT TGTTCATCCT TTGATTAATC   
  
  
- GTGTTTCTTT TTGTTTACCC CCCAGACTTC TATAAAACCC TAATTTTATG CTCCCCACCT ACCCATATAC   
  
  
- ATTACATTCT CTCCTCCTTC CATCGTACGT CATCGTCTTC GCTATTTTTT TGTGCTGTCA CTTTTCCTTG   
  
  
- TGCTTCTGCT GAACCTTATG CCAACCCTTC CCTTATACTC ACAATGTAGC GTACAAAAAT ATGTCACCAC   
  
  
- TCTATAAAAT AAAAATTCAA TAATTGAAAA ATTGGGTGTA TAGAATGGTC ATTATATTAC TATGCATCAC   
  
  
- ATGATAGAGT ATACAGCCAG TGTAATTTTT TTTAAAGAGT TAATAGTAAA CTACAATATA AACTGTGCAG   
  
  
- TTAAGCAGAG AAACTAATTA TTAATATATT TAATAAATAG TGTAGTATAT TATGTGACAT GTGTGGTTAT   
  
  
- ATTATATTTG TATTTGTACA CTAAAAGGAC TATAATGATA CTTACTTTAA TATATGTTAT AATAAATATT   
  
  
- ACAACAGGAG TTCCACCTCT AAAGCTCAAG TTTAATATGT ATATGTTCTC TAAGTACGTT CATAAGTTAT   
  
  
- AATTTAACAC TAGTGGGGAA TTAGTAAACA CTTTTTCTTT TTCAATTGAA CGGTTAGAGT AAGGAGAATT   
  
  
- ATTAATTGTT AAATCGAATA ATTACGATAG TAAACAATTT TTAAATTTTA TACTCTTGAA AATACTATGA   
  
  
- AAAAATATCT TCAATTATCA TAGAGCAAAA TTATTTTAAT AAACGCCTGC AATACATCAA TGAGTAGTAG   
  
  
- TAATAAACGT AAGCCTTTAG GATAGAAGTG CAAATTGCTA AAATGTTGTA CTACATACTT AATCTTCTTT   
  
  
- TTATTACAAT AATGCGTTCT TAAGAAGAGA GAGGTTTGGA GATATTGAGA GGGAGGGGAG AGAAAGTAAA   
  
  
- CCACGTCCAC CACCGCGCTT CTACAGGAAA ATCGTCTCCA CCCCACCGAT TTTCTTTATC CCCATGACGC   
  
  
- AAAACCTAAG ACCCAACACT CAAGACCCAA GATTTAAATA AGCAATTGAG GTCGAAGAGC AGGATGAAGC   
  
  
- CCAAGTCTAG AACGACAAAG CAGAAGTAGG AGACAGTAAC GGACAGTTTC AGCTGCTAAC GCAACCAGGA   
  
  
- GCAGTCCAAG TCTAGAGTGA GGAGTAAAAG GGAAGCCTAA GGATAAAAAA GTTGAAAGAC TTTAAATACG   
  
  
- GTTAGAAAAA GCTATAAAAA GACCCAAAAC ATACTTGTAT ACACCCAACT TCAACTTCTC CCCTCCCTCT   
  
  
- CAATATCTCC AAACCTCTCT CTTCTTCTAC TTTCTCTCAC CCCTTCTTGC TCCTCCCCCT TTTCTTTCTA   
  
  
- TGAAAGTACC AAATCCCTCA CACACTTTCA AACTCTTCCA CAACCCACTC TCACTCTCCC TCTCTCACAA   
  
  
- CGCCGTCGAT ACCCACCTAG ACCCAAGAA

+     STRE

| Site Name | Organism | Position | Strand | Matrix score. | sequence | function |
| --- | --- | --- | --- | --- | --- | --- |
| STRE | Arabidopsis thaliana | 1384 | + | 5 | AGGGG |  |
| STRE | Arabidopsis thaliana | 1319 | + | 5 | AGGGG |  |
| STRE | Arabidopsis thaliana | 965 | - | 5 | AGGGG |  |
| STRE | Arabidopsis thaliana | 1039 | + | 5 | AGGGG |  |
| STRE | Arabidopsis thaliana | 645 | - | 5 | AGGGG |  |
| STRE | Arabidopsis thaliana | 122 | + | 5 | AGGGG |  |

>037815   
+ TTTCAGATGC TTCTCACAGA TATTCAAAGT TTGGTTGATT CAACTATTCA ACAAGTAGGA AACTAATTAG   
  
  
+ CACAAAGAAA AACAAATGGG GGGTCTGAAG ATATTTTGGG ATTAAAATAC GAGGGGTGGA TGGGTATATG   
  
  
+ TAATGTAAGA GAGGAGGAAG GTAGCATGCA GTAGCAGAAG CGATAAAAAA ACACGACAGT GAAAAGGAAC   
  
  
+ ACGAAGACGA CTTGGAATAC GGTTGGGAAG GGAATATGAG TGTTACATCG CATGTTTTTA TACAGTGGTG   
  
  
+ AGATATTTTA TTTTTAAGTT ATTAACTTTT TAACCCACAT ATCTTACCAG TAATATAATG ATACGTAGTG   
  
  
+ TACTATCTCA TATGTCGGTC ACATTAAAAA AAATTTCTCA ATTATCATTT GATGTTATAT TTGACACGTC   
  
  
+ AATTCGTCTC TTTGATTAAT AATTATATAA ATTATTTATC ACATCATATA ATACACTGTA CACACCAATA   
  
  
+ TAATATAAAC ATAAACATGT GATTTTCCTG ATATTACTAT GAATGAAATT ATATACAATA TTATTTATAA   
  
  
+ TGTTGTCCTC AAGGTGGAGA TTTCGAGTTC AAATTATACA TATACAAGAG ATTCATGCAA GTATTCAATA   
  
  
+ TTAAATTGTG ATCACCCCTT AATCATTTGT GAAAAAGAAA AAGTTAACTT GCCAATCTCA TTCCTCTTAA   
  
  
+ TAATTAACAA TTTAGCTTAT TAATGCTATC ATTTGTTAAA AATTTAAAAT ATGAGAACTT TTATGATACT   
  
  
+ TTTTTATAGA AGTTAATAGT ATCTCGTTTT AATAAAATTA TTTGCGGACG TTATGTAGTT ACTCATCATC   
  
  
+ ATTATTTGCA TTCGGAAATC CTATCTTCAC GTTTAACGAT TTTACAACAT GATGTATGAA TTAGAAGAAA   
  
  
+ AATAATGTTA TTACGCAAGA ATTCTTCTCT CTCCAAACCT CTATAACTCT CCCTCCCCTC TCTTTCATTT   
  
  
+ GGTGCAGGTG GTGGCGCGAA GATGTCCTTT TAGCAGAGGT GGGGTGGCTA AAAGAAATAG GGGTACTGCG   
  
  
+ TTTTGGATTC TGGGTTGTGA GTTCTGGGTT CTAAATTTAT TCGTTAACTC CAGCTTCTCG TCCTACTTCG   
  
  
+ GGTTCAGATC TTGCTGTTTC GTCTTCATCC TCTGTCATTG CCTGTCAAAG TCGACGATTG CGTTGGTCCT   
  
  
+ CGTCAGGTTC AGATCTCACT CCTCATTTTC CCTTCGGATT CCTATTTTTT CAACTTTCTG AAATTTATGC   
  
  
+ CAATCTTTTT CGATATTTTT CTGGGTTTTG TATGAACATA TGTGGGTTGA AGTTGAAGAG GGGAGGGAGA   
  
  
+ GTTATAGAGG TTTGGAGAGA GAAGAAGATG AAAGAGAGTG GGGAAGAACG AGGAGGGGGA AAAGAAAGAT   
  
  
+ ACTTTCATGG TTTAGGGAGT GTGTGAAAGT TTGAGAAGGT GTTGGGTGAG AGTGAGAGGG AGAGAGTGTT   
  
  
+ GCGGCAGCTA TGGGTGGATC TGGGTTCTT  

- AAAGTCTACG AAGAGTGTCT ATAAGTTTCA AACCAACTAA GTTGATAAGT TGTTCATCCT TTGATTAATC   
  
  
- GTGTTTCTTT TTGTTTACCC CCCAGACTTC TATAAAACCC TAATTTTATG CTCCCCACCT ACCCATATAC   
  
  
- ATTACATTCT CTCCTCCTTC CATCGTACGT CATCGTCTTC GCTATTTTTT TGTGCTGTCA CTTTTCCTTG   
  
  
- TGCTTCTGCT GAACCTTATG CCAACCCTTC CCTTATACTC ACAATGTAGC GTACAAAAAT ATGTCACCAC   
  
  
- TCTATAAAAT AAAAATTCAA TAATTGAAAA ATTGGGTGTA TAGAATGGTC ATTATATTAC TATGCATCAC   
  
  
- ATGATAGAGT ATACAGCCAG TGTAATTTTT TTTAAAGAGT TAATAGTAAA CTACAATATA AACTGTGCAG   
  
  
- TTAAGCAGAG AAACTAATTA TTAATATATT TAATAAATAG TGTAGTATAT TATGTGACAT GTGTGGTTAT   
  
  
- ATTATATTTG TATTTGTACA CTAAAAGGAC TATAATGATA CTTACTTTAA TATATGTTAT AATAAATATT   
  
  
- ACAACAGGAG TTCCACCTCT AAAGCTCAAG TTTAATATGT ATATGTTCTC TAAGTACGTT CATAAGTTAT   
  
  
- AATTTAACAC TAGTGGGGAA TTAGTAAACA CTTTTTCTTT TTCAATTGAA CGGTTAGAGT AAGGAGAATT   
  
  
- ATTAATTGTT AAATCGAATA ATTACGATAG TAAACAATTT TTAAATTTTA TACTCTTGAA AATACTATGA   
  
  
- AAAAATATCT TCAATTATCA TAGAGCAAAA TTATTTTAAT AAACGCCTGC AATACATCAA TGAGTAGTAG   
  
  
- TAATAAACGT AAGCCTTTAG GATAGAAGTG CAAATTGCTA AAATGTTGTA CTACATACTT AATCTTCTTT   
  
  
- TTATTACAAT AATGCGTTCT TAAGAAGAGA GAGGTTTGGA GATATTGAGA GGGAGGGGAG AGAAAGTAAA   
  
  
- CCACGTCCAC CACCGCGCTT CTACAGGAAA ATCGTCTCCA CCCCACCGAT TTTCTTTATC CCCATGACGC   
  
  
- AAAACCTAAG ACCCAACACT CAAGACCCAA GATTTAAATA AGCAATTGAG GTCGAAGAGC AGGATGAAGC   
  
  
- CCAAGTCTAG AACGACAAAG CAGAAGTAGG AGACAGTAAC GGACAGTTTC AGCTGCTAAC GCAACCAGGA   
  
  
- GCAGTCCAAG TCTAGAGTGA GGAGTAAAAG GGAAGCCTAA GGATAAAAAA GTTGAAAGAC TTTAAATACG   
  
  
- GTTAGAAAAA GCTATAAAAA GACCCAAAAC ATACTTGTAT ACACCCAACT TCAACTTCTC CCCTCCCTCT   
  
  
- CAATATCTCC AAACCTCTCT CTTCTTCTAC TTTCTCTCAC CCCTTCTTGC TCCTCCCCCT TTTCTTTCTA   
  
  
- TGAAAGTACC AAATCCCTCA CACACTTTCA AACTCTTCCA CAACCCACTC TCACTCTCCC TCTCTCACAA   
  
  
- CGCCGTCGAT ACCCACCTAG ACCCAAGAA

+     TATA-box

| Site Name | Organism | Position | Strand | Matrix score. | sequence | function |
| --- | --- | --- | --- | --- | --- | --- |
| TATA-box | Arabidopsis thaliana | 1333 | - | 4 | TATA | core promoter element around -30 of transcription start |
| TATA-box | Arabidopsis thaliana | 594 | - | 5 | TATAA | core promoter element around -30 of transcription start |
| TATA-box | Arabidopsis thaliana | 489 | + | 4 | TATA | core promoter element around -30 of transcription start |
| TATA-box | Arabidopsis thaliana | 952 | - | 4 | TATA | core promoter element around -30 of transcription start |
| TATA-box | Arabidopsis thaliana | 775 | - | 4 | TATA | core promoter element around -30 of transcription start |
| TATA-box | Brassica oleracea | 488 | + | 6 | ATATAA | core promoter element around -30 of transcription start |
| TATA-box | Brassica oleracea | 333 | + | 6 | ATATAA | core promoter element around -30 of transcription start |
| TATA-box | Arabidopsis thaliana | 334 | + | 4 | TATA | core promoter element around -30 of transcription start |
| TATA-box | Arabidopsis thaliana | 443 | - | 7 | TATATAA | core promoter element around -30 of transcription start |
| TATA-box | Arabidopsis thaliana | 269 | + | 4 | TATA | core promoter element around -30 of transcription start |
| TATA-box | Oryza sativa | 1286 | - | 7 | TACAAAA | core promoter element around -30 of transcription start |
| TATA-box | Arabidopsis thaliana | 405 | - | 5 | TATAA | core promoter element around -30 of transcription start |
| TATA-box | Pisum sativum | 266 | - | 7 | TATAAAA | core promoter element around -30 of transcription start |
| TATA-box | Arabidopsis thaliana | 494 | + | 4 | TATA | core promoter element around -30 of transcription start |
| TATA-box | Brassica oleracea | 493 | + | 6 | ATATAA | core promoter element around -30 of transcription start |
| TATA-box | Arabidopsis thaliana | 774 | - | 5 | TATAA | core promoter element around -30 of transcription start |
| TATA-box | Arabidopsis thaliana | 1332 | - | 5 | TATAA | core promoter element around -30 of transcription start |
| TATA-box | Arabidopsis thaliana | 601 | + | 4 | TATA | core promoter element around -30 of transcription start |
| TATA-box | Arabidopsis thaliana | 595 | + | 4 | TATA | core promoter element around -30 of transcription start |
| TATA-box | Arabidopsis thaliana | 444 | + | 6 | TATATA | core promoter element around -30 of transcription start |
| TATA-box | Arabidopsis thaliana | 556 | + | 4 | TATA | core promoter element around -30 of transcription start |
| TATA-box | Brassica oleracea | 466 | + | 6 | ATATAA | core promoter element around -30 of transcription start |
| TATA-box | Arabidopsis thaliana | 135 | + | 4 | TATA | core promoter element around -30 of transcription start |
| TATA-box | Arabidopsis thaliana | 467 | + | 4 | TATA | core promoter element around -30 of transcription start |
| TATA-box | Pisum sativum | 772 | - | 7 | TATAAAA | core promoter element around -30 of transcription start |
| TATA-box | Arabidopsis thaliana | 446 | + | 4 | TATA | core promoter element around -30 of transcription start |
| TATA-box | Brassica oleracea | 445 | + | 6 | ATATAA | core promoter element around -30 of transcription start |
| TATA-box | Brassica napus | 593 | + | 6 | ATTATA | core promoter element around -30 of transcription start |
| TATA-box | Arabidopsis thaliana | 555 | - | 5 | TATAA | core promoter element around -30 of transcription start |
| TATA-box | Arabidopsis thaliana | 268 | - | 5 | TATAA | core promoter element around -30 of transcription start |
| TATA-box | Helianthus annuus | 554 | - | 6 | TATAAA | core promoter element around -30 of transcription start |
| TATA-box | Brassica juncea | 553 | - | 7 | TATAAAT | core promoter element around -30 of transcription start |
| TATA-box | Arabidopsis thaliana | 771 | - | 9 | ccTATAAAaa | core promoter element around -30 of transcription start |
| TATA-box | Arabidopsis thaliana | 406 | + | 4 | TATA | core promoter element around -30 of transcription start |
| TATA-box | Arabidopsis thaliana | 540 | + | 6 | TATATA | core promoter element around -30 of transcription start |
| TATA-box | Brassica napus | 442 | + | 6 | ATTATA | core promoter element around -30 of transcription start |
| TATA-box | Brassica napus | 538 | + | 6 | ATTATA | core promoter element around -30 of transcription start |
| TATA-box | Helianthus annuus | 773 | - | 6 | TATAAA | core promoter element around -30 of transcription start |
| TATA-box | Helianthus annuus | 267 | - | 6 | TATAAA | core promoter element around -30 of transcription start |
| TATA-box | Daucus carota | 552 | - | 8 | TATAAATA | core promoter element around -30 of transcription start |
| TATA-box | Arabidopsis thaliana | 542 | + | 4 | TATA | core promoter element around -30 of transcription start |
| TATA-box | Arabidopsis thaliana | 539 | - | 7 | TATATAA | core promoter element around -30 of transcription start |

>037815   
+ TTTCAGATGC TTCTCACAGA TATTCAAAGT TTGGTTGATT CAACTATTCA ACAAGTAGGA AACTAATTAG   
  
  
+ CACAAAGAAA AACAAATGGG GGGTCTGAAG ATATTTTGGG ATTAAAATAC GAGGGGTGGA TGGGTATATG   
  
  
+ TAATGTAAGA GAGGAGGAAG GTAGCATGCA GTAGCAGAAG CGATAAAAAA ACACGACAGT GAAAAGGAAC   
  
  
+ ACGAAGACGA CTTGGAATAC GGTTGGGAAG GGAATATGAG TGTTACATCG CATGTTTTTA TACAGTGGTG   
  
  
+ AGATATTTTA TTTTTAAGTT ATTAACTTTT TAACCCACAT ATCTTACCAG TAATATAATG ATACGTAGTG   
  
  
+ TACTATCTCA TATGTCGGTC ACATTAAAAA AAATTTCTCA ATTATCATTT GATGTTATAT TTGACACGTC   
  
  
+ AATTCGTCTC TTTGATTAAT AATTATATAA ATTATTTATC ACATCATATA ATACACTGTA CACACCAATA   
  
  
+ TAATATAAAC ATAAACATGT GATTTTCCTG ATATTACTAT GAATGAAATT ATATACAATA TTATTTATAA   
  
  
+ TGTTGTCCTC AAGGTGGAGA TTTCGAGTTC AAATTATACA TATACAAGAG ATTCATGCAA GTATTCAATA   
  
  
+ TTAAATTGTG ATCACCCCTT AATCATTTGT GAAAAAGAAA AAGTTAACTT GCCAATCTCA TTCCTCTTAA   
  
  
+ TAATTAACAA TTTAGCTTAT TAATGCTATC ATTTGTTAAA AATTTAAAAT ATGAGAACTT TTATGATACT   
  
  
+ TTTTTATAGA AGTTAATAGT ATCTCGTTTT AATAAAATTA TTTGCGGACG TTATGTAGTT ACTCATCATC   
  
  
+ ATTATTTGCA TTCGGAAATC CTATCTTCAC GTTTAACGAT TTTACAACAT GATGTATGAA TTAGAAGAAA   
  
  
+ AATAATGTTA TTACGCAAGA ATTCTTCTCT CTCCAAACCT CTATAACTCT CCCTCCCCTC TCTTTCATTT   
  
  
+ GGTGCAGGTG GTGGCGCGAA GATGTCCTTT TAGCAGAGGT GGGGTGGCTA AAAGAAATAG GGGTACTGCG   
  
  
+ TTTTGGATTC TGGGTTGTGA GTTCTGGGTT CTAAATTTAT TCGTTAACTC CAGCTTCTCG TCCTACTTCG   
  
  
+ GGTTCAGATC TTGCTGTTTC GTCTTCATCC TCTGTCATTG CCTGTCAAAG TCGACGATTG CGTTGGTCCT   
  
  
+ CGTCAGGTTC AGATCTCACT CCTCATTTTC CCTTCGGATT CCTATTTTTT CAACTTTCTG AAATTTATGC   
  
  
+ CAATCTTTTT CGATATTTTT CTGGGTTTTG TATGAACATA TGTGGGTTGA AGTTGAAGAG GGGAGGGAGA   
  
  
+ GTTATAGAGG TTTGGAGAGA GAAGAAGATG AAAGAGAGTG GGGAAGAACG AGGAGGGGGA AAAGAAAGAT   
  
  
+ ACTTTCATGG TTTAGGGAGT GTGTGAAAGT TTGAGAAGGT GTTGGGTGAG AGTGAGAGGG AGAGAGTGTT   
  
  
+ GCGGCAGCTA TGGGTGGATC TGGGTTCTT  

- AAAGTCTACG AAGAGTGTCT ATAAGTTTCA AACCAACTAA GTTGATAAGT TGTTCATCCT TTGATTAATC   
  
  
- GTGTTTCTTT TTGTTTACCC CCCAGACTTC TATAAAACCC TAATTTTATG CTCCCCACCT ACCCATATAC   
  
  
- ATTACATTCT CTCCTCCTTC CATCGTACGT CATCGTCTTC GCTATTTTTT TGTGCTGTCA CTTTTCCTTG   
  
  
- TGCTTCTGCT GAACCTTATG CCAACCCTTC CCTTATACTC ACAATGTAGC GTACAAAAAT ATGTCACCAC   
  
  
- TCTATAAAAT AAAAATTCAA TAATTGAAAA ATTGGGTGTA TAGAATGGTC ATTATATTAC TATGCATCAC   
  
  
- ATGATAGAGT ATACAGCCAG TGTAATTTTT TTTAAAGAGT TAATAGTAAA CTACAATATA AACTGTGCAG   
  
  
- TTAAGCAGAG AAACTAATTA TTAATATATT TAATAAATAG TGTAGTATAT TATGTGACAT GTGTGGTTAT   
  
  
- ATTATATTTG TATTTGTACA CTAAAAGGAC TATAATGATA CTTACTTTAA TATATGTTAT AATAAATATT   
  
  
- ACAACAGGAG TTCCACCTCT AAAGCTCAAG TTTAATATGT ATATGTTCTC TAAGTACGTT CATAAGTTAT   
  
  
- AATTTAACAC TAGTGGGGAA TTAGTAAACA CTTTTTCTTT TTCAATTGAA CGGTTAGAGT AAGGAGAATT   
  
  
- ATTAATTGTT AAATCGAATA ATTACGATAG TAAACAATTT TTAAATTTTA TACTCTTGAA AATACTATGA   
  
  
- AAAAATATCT TCAATTATCA TAGAGCAAAA TTATTTTAAT AAACGCCTGC AATACATCAA TGAGTAGTAG   
  
  
- TAATAAACGT AAGCCTTTAG GATAGAAGTG CAAATTGCTA AAATGTTGTA CTACATACTT AATCTTCTTT   
  
  
- TTATTACAAT AATGCGTTCT TAAGAAGAGA GAGGTTTGGA GATATTGAGA GGGAGGGGAG AGAAAGTAAA   
  
  
- CCACGTCCAC CACCGCGCTT CTACAGGAAA ATCGTCTCCA CCCCACCGAT TTTCTTTATC CCCATGACGC   
  
  
- AAAACCTAAG ACCCAACACT CAAGACCCAA GATTTAAATA AGCAATTGAG GTCGAAGAGC AGGATGAAGC   
  
  
- CCAAGTCTAG AACGACAAAG CAGAAGTAGG AGACAGTAAC GGACAGTTTC AGCTGCTAAC GCAACCAGGA   
  
  
- GCAGTCCAAG TCTAGAGTGA GGAGTAAAAG GGAAGCCTAA GGATAAAAAA GTTGAAAGAC TTTAAATACG   
  
  
- GTTAGAAAAA GCTATAAAAA GACCCAAAAC ATACTTGTAT ACACCCAACT TCAACTTCTC CCCTCCCTCT   
  
  
- CAATATCTCC AAACCTCTCT CTTCTTCTAC TTTCTCTCAC CCCTTCTTGC TCCTCCCCCT TTTCTTTCTA   
  
  
- TGAAAGTACC AAATCCCTCA CACACTTTCA AACTCTTCCA CAACCCACTC TCACTCTCCC TCTCTCACAA   
  
  
- CGCCGTCGAT ACCCACCTAG ACCCAAGAA

+     TCA

| Site Name | Organism | Position | Strand | Matrix score. | sequence | function |
| --- | --- | --- | --- | --- | --- | --- |
| TCA | Pisum sativum | 1352 | - | 9 | TCATCTTCAT |  |
| TCA | Pisum sativum | 1139 | + | 9 | TCATCTTCAT |  |
| TCA | Pisum sativum | 833 | + | 9 | TCATCTTCAT |  |

>037815   
+ TTTCAGATGC TTCTCACAGA TATTCAAAGT TTGGTTGATT CAACTATTCA ACAAGTAGGA AACTAATTAG   
  
  
+ CACAAAGAAA AACAAATGGG GGGTCTGAAG ATATTTTGGG ATTAAAATAC GAGGGGTGGA TGGGTATATG   
  
  
+ TAATGTAAGA GAGGAGGAAG GTAGCATGCA GTAGCAGAAG CGATAAAAAA ACACGACAGT GAAAAGGAAC   
  
  
+ ACGAAGACGA CTTGGAATAC GGTTGGGAAG GGAATATGAG TGTTACATCG CATGTTTTTA TACAGTGGTG   
  
  
+ AGATATTTTA TTTTTAAGTT ATTAACTTTT TAACCCACAT ATCTTACCAG TAATATAATG ATACGTAGTG   
  
  
+ TACTATCTCA TATGTCGGTC ACATTAAAAA AAATTTCTCA ATTATCATTT GATGTTATAT TTGACACGTC   
  
  
+ AATTCGTCTC TTTGATTAAT AATTATATAA ATTATTTATC ACATCATATA ATACACTGTA CACACCAATA   
  
  
+ TAATATAAAC ATAAACATGT GATTTTCCTG ATATTACTAT GAATGAAATT ATATACAATA TTATTTATAA   
  
  
+ TGTTGTCCTC AAGGTGGAGA TTTCGAGTTC AAATTATACA TATACAAGAG ATTCATGCAA GTATTCAATA   
  
  
+ TTAAATTGTG ATCACCCCTT AATCATTTGT GAAAAAGAAA AAGTTAACTT GCCAATCTCA TTCCTCTTAA   
  
  
+ TAATTAACAA TTTAGCTTAT TAATGCTATC ATTTGTTAAA AATTTAAAAT ATGAGAACTT TTATGATACT   
  
  
+ TTTTTATAGA AGTTAATAGT ATCTCGTTTT AATAAAATTA TTTGCGGACG TTATGTAGTT ACTCATCATC   
  
  
+ ATTATTTGCA TTCGGAAATC CTATCTTCAC GTTTAACGAT TTTACAACAT GATGTATGAA TTAGAAGAAA   
  
  
+ AATAATGTTA TTACGCAAGA ATTCTTCTCT CTCCAAACCT CTATAACTCT CCCTCCCCTC TCTTTCATTT   
  
  
+ GGTGCAGGTG GTGGCGCGAA GATGTCCTTT TAGCAGAGGT GGGGTGGCTA AAAGAAATAG GGGTACTGCG   
  
  
+ TTTTGGATTC TGGGTTGTGA GTTCTGGGTT CTAAATTTAT TCGTTAACTC CAGCTTCTCG TCCTACTTCG   
  
  
+ GGTTCAGATC TTGCTGTTTC GTCTTCATCC TCTGTCATTG CCTGTCAAAG TCGACGATTG CGTTGGTCCT   
  
  
+ CGTCAGGTTC AGATCTCACT CCTCATTTTC CCTTCGGATT CCTATTTTTT CAACTTTCTG AAATTTATGC   
  
  
+ CAATCTTTTT CGATATTTTT CTGGGTTTTG TATGAACATA TGTGGGTTGA AGTTGAAGAG GGGAGGGAGA   
  
  
+ GTTATAGAGG TTTGGAGAGA GAAGAAGATG AAAGAGAGTG GGGAAGAACG AGGAGGGGGA AAAGAAAGAT   
  
  
+ ACTTTCATGG TTTAGGGAGT GTGTGAAAGT TTGAGAAGGT GTTGGGTGAG AGTGAGAGGG AGAGAGTGTT   
  
  
+ GCGGCAGCTA TGGGTGGATC TGGGTTCTT  

- AAAGTCTACG AAGAGTGTCT ATAAGTTTCA AACCAACTAA GTTGATAAGT TGTTCATCCT TTGATTAATC   
  
  
- GTGTTTCTTT TTGTTTACCC CCCAGACTTC TATAAAACCC TAATTTTATG CTCCCCACCT ACCCATATAC   
  
  
- ATTACATTCT CTCCTCCTTC CATCGTACGT CATCGTCTTC GCTATTTTTT TGTGCTGTCA CTTTTCCTTG   
  
  
- TGCTTCTGCT GAACCTTATG CCAACCCTTC CCTTATACTC ACAATGTAGC GTACAAAAAT ATGTCACCAC   
  
  
- TCTATAAAAT AAAAATTCAA TAATTGAAAA ATTGGGTGTA TAGAATGGTC ATTATATTAC TATGCATCAC   
  
  
- ATGATAGAGT ATACAGCCAG TGTAATTTTT TTTAAAGAGT TAATAGTAAA CTACAATATA AACTGTGCAG   
  
  
- TTAAGCAGAG AAACTAATTA TTAATATATT TAATAAATAG TGTAGTATAT TATGTGACAT GTGTGGTTAT   
  
  
- ATTATATTTG TATTTGTACA CTAAAAGGAC TATAATGATA CTTACTTTAA TATATGTTAT AATAAATATT   
  
  
- ACAACAGGAG TTCCACCTCT AAAGCTCAAG TTTAATATGT ATATGTTCTC TAAGTACGTT CATAAGTTAT   
  
  
- AATTTAACAC TAGTGGGGAA TTAGTAAACA CTTTTTCTTT TTCAATTGAA CGGTTAGAGT AAGGAGAATT   
  
  
- ATTAATTGTT AAATCGAATA ATTACGATAG TAAACAATTT TTAAATTTTA TACTCTTGAA AATACTATGA   
  
  
- AAAAATATCT TCAATTATCA TAGAGCAAAA TTATTTTAAT AAACGCCTGC AATACATCAA TGAGTAGTAG   
  
  
- TAATAAACGT AAGCCTTTAG GATAGAAGTG CAAATTGCTA AAATGTTGTA CTACATACTT AATCTTCTTT   
  
  
- TTATTACAAT AATGCGTTCT TAAGAAGAGA GAGGTTTGGA GATATTGAGA GGGAGGGGAG AGAAAGTAAA   
  
  
- CCACGTCCAC CACCGCGCTT CTACAGGAAA ATCGTCTCCA CCCCACCGAT TTTCTTTATC CCCATGACGC   
  
  
- AAAACCTAAG ACCCAACACT CAAGACCCAA GATTTAAATA AGCAATTGAG GTCGAAGAGC AGGATGAAGC   
  
  
- CCAAGTCTAG AACGACAAAG CAGAAGTAGG AGACAGTAAC GGACAGTTTC AGCTGCTAAC GCAACCAGGA   
  
  
- GCAGTCCAAG TCTAGAGTGA GGAGTAAAAG GGAAGCCTAA GGATAAAAAA GTTGAAAGAC TTTAAATACG   
  
  
- GTTAGAAAAA GCTATAAAAA GACCCAAAAC ATACTTGTAT ACACCCAACT TCAACTTCTC CCCTCCCTCT   
  
  
- CAATATCTCC AAACCTCTCT CTTCTTCTAC TTTCTCTCAC CCCTTCTTGC TCCTCCCCCT TTTCTTTCTA   
  
  
- TGAAAGTACC AAATCCCTCA CACACTTTCA AACTCTTCCA CAACCCACTC TCACTCTCCC TCTCTCACAA   
  
  
- CGCCGTCGAT ACCCACCTAG ACCCAAGAA

+     TCA-element

| Site Name | Organism | Position | Strand | Matrix score. | sequence | function |
| --- | --- | --- | --- | --- | --- | --- |
| TCA-element | Nicotiana tabacum | 1261 | + | 9 | CCATCTTTTT | cis-acting element involved in salicylic acid responsiveness |

>037815   
+ TTTCAGATGC TTCTCACAGA TATTCAAAGT TTGGTTGATT CAACTATTCA ACAAGTAGGA AACTAATTAG   
  
  
+ CACAAAGAAA AACAAATGGG GGGTCTGAAG ATATTTTGGG ATTAAAATAC GAGGGGTGGA TGGGTATATG   
  
  
+ TAATGTAAGA GAGGAGGAAG GTAGCATGCA GTAGCAGAAG CGATAAAAAA ACACGACAGT GAAAAGGAAC   
  
  
+ ACGAAGACGA CTTGGAATAC GGTTGGGAAG GGAATATGAG TGTTACATCG CATGTTTTTA TACAGTGGTG   
  
  
+ AGATATTTTA TTTTTAAGTT ATTAACTTTT TAACCCACAT ATCTTACCAG TAATATAATG ATACGTAGTG   
  
  
+ TACTATCTCA TATGTCGGTC ACATTAAAAA AAATTTCTCA ATTATCATTT GATGTTATAT TTGACACGTC   
  
  
+ AATTCGTCTC TTTGATTAAT AATTATATAA ATTATTTATC ACATCATATA ATACACTGTA CACACCAATA   
  
  
+ TAATATAAAC ATAAACATGT GATTTTCCTG ATATTACTAT GAATGAAATT ATATACAATA TTATTTATAA   
  
  
+ TGTTGTCCTC AAGGTGGAGA TTTCGAGTTC AAATTATACA TATACAAGAG ATTCATGCAA GTATTCAATA   
  
  
+ TTAAATTGTG ATCACCCCTT AATCATTTGT GAAAAAGAAA AAGTTAACTT GCCAATCTCA TTCCTCTTAA   
  
  
+ TAATTAACAA TTTAGCTTAT TAATGCTATC ATTTGTTAAA AATTTAAAAT ATGAGAACTT TTATGATACT   
  
  
+ TTTTTATAGA AGTTAATAGT ATCTCGTTTT AATAAAATTA TTTGCGGACG TTATGTAGTT ACTCATCATC   
  
  
+ ATTATTTGCA TTCGGAAATC CTATCTTCAC GTTTAACGAT TTTACAACAT GATGTATGAA TTAGAAGAAA   
  
  
+ AATAATGTTA TTACGCAAGA ATTCTTCTCT CTCCAAACCT CTATAACTCT CCCTCCCCTC TCTTTCATTT   
  
  
+ GGTGCAGGTG GTGGCGCGAA GATGTCCTTT TAGCAGAGGT GGGGTGGCTA AAAGAAATAG GGGTACTGCG   
  
  
+ TTTTGGATTC TGGGTTGTGA GTTCTGGGTT CTAAATTTAT TCGTTAACTC CAGCTTCTCG TCCTACTTCG   
  
  
+ GGTTCAGATC TTGCTGTTTC GTCTTCATCC TCTGTCATTG CCTGTCAAAG TCGACGATTG CGTTGGTCCT   
  
  
+ CGTCAGGTTC AGATCTCACT CCTCATTTTC CCTTCGGATT CCTATTTTTT CAACTTTCTG AAATTTATGC   
  
  
+ CAATCTTTTT CGATATTTTT CTGGGTTTTG TATGAACATA TGTGGGTTGA AGTTGAAGAG GGGAGGGAGA   
  
  
+ GTTATAGAGG TTTGGAGAGA GAAGAAGATG AAAGAGAGTG GGGAAGAACG AGGAGGGGGA AAAGAAAGAT   
  
  
+ ACTTTCATGG TTTAGGGAGT GTGTGAAAGT TTGAGAAGGT GTTGGGTGAG AGTGAGAGGG AGAGAGTGTT   
  
  
+ GCGGCAGCTA TGGGTGGATC TGGGTTCTT  

- AAAGTCTACG AAGAGTGTCT ATAAGTTTCA AACCAACTAA GTTGATAAGT TGTTCATCCT TTGATTAATC   
  
  
- GTGTTTCTTT TTGTTTACCC CCCAGACTTC TATAAAACCC TAATTTTATG CTCCCCACCT ACCCATATAC   
  
  
- ATTACATTCT CTCCTCCTTC CATCGTACGT CATCGTCTTC GCTATTTTTT TGTGCTGTCA CTTTTCCTTG   
  
  
- TGCTTCTGCT GAACCTTATG CCAACCCTTC CCTTATACTC ACAATGTAGC GTACAAAAAT ATGTCACCAC   
  
  
- TCTATAAAAT AAAAATTCAA TAATTGAAAA ATTGGGTGTA TAGAATGGTC ATTATATTAC TATGCATCAC   
  
  
- ATGATAGAGT ATACAGCCAG TGTAATTTTT TTTAAAGAGT TAATAGTAAA CTACAATATA AACTGTGCAG   
  
  
- TTAAGCAGAG AAACTAATTA TTAATATATT TAATAAATAG TGTAGTATAT TATGTGACAT GTGTGGTTAT   
  
  
- ATTATATTTG TATTTGTACA CTAAAAGGAC TATAATGATA CTTACTTTAA TATATGTTAT AATAAATATT   
  
  
- ACAACAGGAG TTCCACCTCT AAAGCTCAAG TTTAATATGT ATATGTTCTC TAAGTACGTT CATAAGTTAT   
  
  
- AATTTAACAC TAGTGGGGAA TTAGTAAACA CTTTTTCTTT TTCAATTGAA CGGTTAGAGT AAGGAGAATT   
  
  
- ATTAATTGTT AAATCGAATA ATTACGATAG TAAACAATTT TTAAATTTTA TACTCTTGAA AATACTATGA   
  
  
- AAAAATATCT TCAATTATCA TAGAGCAAAA TTATTTTAAT AAACGCCTGC AATACATCAA TGAGTAGTAG   
  
  
- TAATAAACGT AAGCCTTTAG GATAGAAGTG CAAATTGCTA AAATGTTGTA CTACATACTT AATCTTCTTT   
  
  
- TTATTACAAT AATGCGTTCT TAAGAAGAGA GAGGTTTGGA GATATTGAGA GGGAGGGGAG AGAAAGTAAA   
  
  
- CCACGTCCAC CACCGCGCTT CTACAGGAAA ATCGTCTCCA CCCCACCGAT TTTCTTTATC CCCATGACGC   
  
  
- AAAACCTAAG ACCCAACACT CAAGACCCAA GATTTAAATA AGCAATTGAG GTCGAAGAGC AGGATGAAGC   
  
  
- CCAAGTCTAG AACGACAAAG CAGAAGTAGG AGACAGTAAC GGACAGTTTC AGCTGCTAAC GCAACCAGGA   
  
  
- GCAGTCCAAG TCTAGAGTGA GGAGTAAAAG GGAAGCCTAA GGATAAAAAA GTTGAAAGAC TTTAAATACG   
  
  
- GTTAGAAAAA GCTATAAAAA GACCCAAAAC ATACTTGTAT ACACCCAACT TCAACTTCTC CCCTCCCTCT   
  
  
- CAATATCTCC AAACCTCTCT CTTCTTCTAC TTTCTCTCAC CCCTTCTTGC TCCTCCCCCT TTTCTTTCTA   
  
  
- TGAAAGTACC AAATCCCTCA CACACTTTCA AACTCTTCCA CAACCCACTC TCACTCTCCC TCTCTCACAA   
  
  
- CGCCGTCGAT ACCCACCTAG ACCCAAGAA

+     TCCC-motif

| Site Name | Organism | Position | Strand | Matrix score. | sequence | function |
| --- | --- | --- | --- | --- | --- | --- |
| TCCC-motif | Spinacia oleracea | 1457 | - | 7 | TCTCCCT | part of a light responsive element |
| TCCC-motif | Spinacia oleracea | 958 | + | 7 | TCTCCCT | part of a light responsive element |
| TCCC-motif | Spinacia oleracea | 1324 | - | 7 | TCTCCCT | part of a light responsive element |

>037815   
+ TTTCAGATGC TTCTCACAGA TATTCAAAGT TTGGTTGATT CAACTATTCA ACAAGTAGGA AACTAATTAG   
  
  
+ CACAAAGAAA AACAAATGGG GGGTCTGAAG ATATTTTGGG ATTAAAATAC GAGGGGTGGA TGGGTATATG   
  
  
+ TAATGTAAGA GAGGAGGAAG GTAGCATGCA GTAGCAGAAG CGATAAAAAA ACACGACAGT GAAAAGGAAC   
  
  
+ ACGAAGACGA CTTGGAATAC GGTTGGGAAG GGAATATGAG TGTTACATCG CATGTTTTTA TACAGTGGTG   
  
  
+ AGATATTTTA TTTTTAAGTT ATTAACTTTT TAACCCACAT ATCTTACCAG TAATATAATG ATACGTAGTG   
  
  
+ TACTATCTCA TATGTCGGTC ACATTAAAAA AAATTTCTCA ATTATCATTT GATGTTATAT TTGACACGTC   
  
  
+ AATTCGTCTC TTTGATTAAT AATTATATAA ATTATTTATC ACATCATATA ATACACTGTA CACACCAATA   
  
  
+ TAATATAAAC ATAAACATGT GATTTTCCTG ATATTACTAT GAATGAAATT ATATACAATA TTATTTATAA   
  
  
+ TGTTGTCCTC AAGGTGGAGA TTTCGAGTTC AAATTATACA TATACAAGAG ATTCATGCAA GTATTCAATA   
  
  
+ TTAAATTGTG ATCACCCCTT AATCATTTGT GAAAAAGAAA AAGTTAACTT GCCAATCTCA TTCCTCTTAA   
  
  
+ TAATTAACAA TTTAGCTTAT TAATGCTATC ATTTGTTAAA AATTTAAAAT ATGAGAACTT TTATGATACT   
  
  
+ TTTTTATAGA AGTTAATAGT ATCTCGTTTT AATAAAATTA TTTGCGGACG TTATGTAGTT ACTCATCATC   
  
  
+ ATTATTTGCA TTCGGAAATC CTATCTTCAC GTTTAACGAT TTTACAACAT GATGTATGAA TTAGAAGAAA   
  
  
+ AATAATGTTA TTACGCAAGA ATTCTTCTCT CTCCAAACCT CTATAACTCT CCCTCCCCTC TCTTTCATTT   
  
  
+ GGTGCAGGTG GTGGCGCGAA GATGTCCTTT TAGCAGAGGT GGGGTGGCTA AAAGAAATAG GGGTACTGCG   
  
  
+ TTTTGGATTC TGGGTTGTGA GTTCTGGGTT CTAAATTTAT TCGTTAACTC CAGCTTCTCG TCCTACTTCG   
  
  
+ GGTTCAGATC TTGCTGTTTC GTCTTCATCC TCTGTCATTG CCTGTCAAAG TCGACGATTG CGTTGGTCCT   
  
  
+ CGTCAGGTTC AGATCTCACT CCTCATTTTC CCTTCGGATT CCTATTTTTT CAACTTTCTG AAATTTATGC   
  
  
+ CAATCTTTTT CGATATTTTT CTGGGTTTTG TATGAACATA TGTGGGTTGA AGTTGAAGAG GGGAGGGAGA   
  
  
+ GTTATAGAGG TTTGGAGAGA GAAGAAGATG AAAGAGAGTG GGGAAGAACG AGGAGGGGGA AAAGAAAGAT   
  
  
+ ACTTTCATGG TTTAGGGAGT GTGTGAAAGT TTGAGAAGGT GTTGGGTGAG AGTGAGAGGG AGAGAGTGTT   
  
  
+ GCGGCAGCTA TGGGTGGATC TGGGTTCTT  

- AAAGTCTACG AAGAGTGTCT ATAAGTTTCA AACCAACTAA GTTGATAAGT TGTTCATCCT TTGATTAATC   
  
  
- GTGTTTCTTT TTGTTTACCC CCCAGACTTC TATAAAACCC TAATTTTATG CTCCCCACCT ACCCATATAC   
  
  
- ATTACATTCT CTCCTCCTTC CATCGTACGT CATCGTCTTC GCTATTTTTT TGTGCTGTCA CTTTTCCTTG   
  
  
- TGCTTCTGCT GAACCTTATG CCAACCCTTC CCTTATACTC ACAATGTAGC GTACAAAAAT ATGTCACCAC   
  
  
- TCTATAAAAT AAAAATTCAA TAATTGAAAA ATTGGGTGTA TAGAATGGTC ATTATATTAC TATGCATCAC   
  
  
- ATGATAGAGT ATACAGCCAG TGTAATTTTT TTTAAAGAGT TAATAGTAAA CTACAATATA AACTGTGCAG   
  
  
- TTAAGCAGAG AAACTAATTA TTAATATATT TAATAAATAG TGTAGTATAT TATGTGACAT GTGTGGTTAT   
  
  
- ATTATATTTG TATTTGTACA CTAAAAGGAC TATAATGATA CTTACTTTAA TATATGTTAT AATAAATATT   
  
  
- ACAACAGGAG TTCCACCTCT AAAGCTCAAG TTTAATATGT ATATGTTCTC TAAGTACGTT CATAAGTTAT   
  
  
- AATTTAACAC TAGTGGGGAA TTAGTAAACA CTTTTTCTTT TTCAATTGAA CGGTTAGAGT AAGGAGAATT   
  
  
- ATTAATTGTT AAATCGAATA ATTACGATAG TAAACAATTT TTAAATTTTA TACTCTTGAA AATACTATGA   
  
  
- AAAAATATCT TCAATTATCA TAGAGCAAAA TTATTTTAAT AAACGCCTGC AATACATCAA TGAGTAGTAG   
  
  
- TAATAAACGT AAGCCTTTAG GATAGAAGTG CAAATTGCTA AAATGTTGTA CTACATACTT AATCTTCTTT   
  
  
- TTATTACAAT AATGCGTTCT TAAGAAGAGA GAGGTTTGGA GATATTGAGA GGGAGGGGAG AGAAAGTAAA   
  
  
- CCACGTCCAC CACCGCGCTT CTACAGGAAA ATCGTCTCCA CCCCACCGAT TTTCTTTATC CCCATGACGC   
  
  
- AAAACCTAAG ACCCAACACT CAAGACCCAA GATTTAAATA AGCAATTGAG GTCGAAGAGC AGGATGAAGC   
  
  
- CCAAGTCTAG AACGACAAAG CAGAAGTAGG AGACAGTAAC GGACAGTTTC AGCTGCTAAC GCAACCAGGA   
  
  
- GCAGTCCAAG TCTAGAGTGA GGAGTAAAAG GGAAGCCTAA GGATAAAAAA GTTGAAAGAC TTTAAATACG   
  
  
- GTTAGAAAAA GCTATAAAAA GACCCAAAAC ATACTTGTAT ACACCCAACT TCAACTTCTC CCCTCCCTCT   
  
  
- CAATATCTCC AAACCTCTCT CTTCTTCTAC TTTCTCTCAC CCCTTCTTGC TCCTCCCCCT TTTCTTTCTA   
  
  
- TGAAAGTACC AAATCCCTCA CACACTTTCA AACTCTTCCA CAACCCACTC TCACTCTCCC TCTCTCACAA   
  
  
- CGCCGTCGAT ACCCACCTAG ACCCAAGAA

+     TCT-motif

| Site Name | Organism | Position | Strand | Matrix score. | sequence | function |
| --- | --- | --- | --- | --- | --- | --- |
| TCT-motif | Arabidopsis thaliana | 322 | + | 6 | TCTTAC | part of a light responsive element |
| TCT-motif | Arabidopsis thaliana | 145 | - | 6 | TCTTAC | part of a light responsive element |

>037815   
+ TTTCAGATGC TTCTCACAGA TATTCAAAGT TTGGTTGATT CAACTATTCA ACAAGTAGGA AACTAATTAG   
  
  
+ CACAAAGAAA AACAAATGGG GGGTCTGAAG ATATTTTGGG ATTAAAATAC GAGGGGTGGA TGGGTATATG   
  
  
+ TAATGTAAGA GAGGAGGAAG GTAGCATGCA GTAGCAGAAG CGATAAAAAA ACACGACAGT GAAAAGGAAC   
  
  
+ ACGAAGACGA CTTGGAATAC GGTTGGGAAG GGAATATGAG TGTTACATCG CATGTTTTTA TACAGTGGTG   
  
  
+ AGATATTTTA TTTTTAAGTT ATTAACTTTT TAACCCACAT ATCTTACCAG TAATATAATG ATACGTAGTG   
  
  
+ TACTATCTCA TATGTCGGTC ACATTAAAAA AAATTTCTCA ATTATCATTT GATGTTATAT TTGACACGTC   
  
  
+ AATTCGTCTC TTTGATTAAT AATTATATAA ATTATTTATC ACATCATATA ATACACTGTA CACACCAATA   
  
  
+ TAATATAAAC ATAAACATGT GATTTTCCTG ATATTACTAT GAATGAAATT ATATACAATA TTATTTATAA   
  
  
+ TGTTGTCCTC AAGGTGGAGA TTTCGAGTTC AAATTATACA TATACAAGAG ATTCATGCAA GTATTCAATA   
  
  
+ TTAAATTGTG ATCACCCCTT AATCATTTGT GAAAAAGAAA AAGTTAACTT GCCAATCTCA TTCCTCTTAA   
  
  
+ TAATTAACAA TTTAGCTTAT TAATGCTATC ATTTGTTAAA AATTTAAAAT ATGAGAACTT TTATGATACT   
  
  
+ TTTTTATAGA AGTTAATAGT ATCTCGTTTT AATAAAATTA TTTGCGGACG TTATGTAGTT ACTCATCATC   
  
  
+ ATTATTTGCA TTCGGAAATC CTATCTTCAC GTTTAACGAT TTTACAACAT GATGTATGAA TTAGAAGAAA   
  
  
+ AATAATGTTA TTACGCAAGA ATTCTTCTCT CTCCAAACCT CTATAACTCT CCCTCCCCTC TCTTTCATTT   
  
  
+ GGTGCAGGTG GTGGCGCGAA GATGTCCTTT TAGCAGAGGT GGGGTGGCTA AAAGAAATAG GGGTACTGCG   
  
  
+ TTTTGGATTC TGGGTTGTGA GTTCTGGGTT CTAAATTTAT TCGTTAACTC CAGCTTCTCG TCCTACTTCG   
  
  
+ GGTTCAGATC TTGCTGTTTC GTCTTCATCC TCTGTCATTG CCTGTCAAAG TCGACGATTG CGTTGGTCCT   
  
  
+ CGTCAGGTTC AGATCTCACT CCTCATTTTC CCTTCGGATT CCTATTTTTT CAACTTTCTG AAATTTATGC   
  
  
+ CAATCTTTTT CGATATTTTT CTGGGTTTTG TATGAACATA TGTGGGTTGA AGTTGAAGAG GGGAGGGAGA   
  
  
+ GTTATAGAGG TTTGGAGAGA GAAGAAGATG AAAGAGAGTG GGGAAGAACG AGGAGGGGGA AAAGAAAGAT   
  
  
+ ACTTTCATGG TTTAGGGAGT GTGTGAAAGT TTGAGAAGGT GTTGGGTGAG AGTGAGAGGG AGAGAGTGTT   
  
  
+ GCGGCAGCTA TGGGTGGATC TGGGTTCTT  

- AAAGTCTACG AAGAGTGTCT ATAAGTTTCA AACCAACTAA GTTGATAAGT TGTTCATCCT TTGATTAATC   
  
  
- GTGTTTCTTT TTGTTTACCC CCCAGACTTC TATAAAACCC TAATTTTATG CTCCCCACCT ACCCATATAC   
  
  
- ATTACATTCT CTCCTCCTTC CATCGTACGT CATCGTCTTC GCTATTTTTT TGTGCTGTCA CTTTTCCTTG   
  
  
- TGCTTCTGCT GAACCTTATG CCAACCCTTC CCTTATACTC ACAATGTAGC GTACAAAAAT ATGTCACCAC   
  
  
- TCTATAAAAT AAAAATTCAA TAATTGAAAA ATTGGGTGTA TAGAATGGTC ATTATATTAC TATGCATCAC   
  
  
- ATGATAGAGT ATACAGCCAG TGTAATTTTT TTTAAAGAGT TAATAGTAAA CTACAATATA AACTGTGCAG   
  
  
- TTAAGCAGAG AAACTAATTA TTAATATATT TAATAAATAG TGTAGTATAT TATGTGACAT GTGTGGTTAT   
  
  
- ATTATATTTG TATTTGTACA CTAAAAGGAC TATAATGATA CTTACTTTAA TATATGTTAT AATAAATATT   
  
  
- ACAACAGGAG TTCCACCTCT AAAGCTCAAG TTTAATATGT ATATGTTCTC TAAGTACGTT CATAAGTTAT   
  
  
- AATTTAACAC TAGTGGGGAA TTAGTAAACA CTTTTTCTTT TTCAATTGAA CGGTTAGAGT AAGGAGAATT   
  
  
- ATTAATTGTT AAATCGAATA ATTACGATAG TAAACAATTT TTAAATTTTA TACTCTTGAA AATACTATGA   
  
  
- AAAAATATCT TCAATTATCA TAGAGCAAAA TTATTTTAAT AAACGCCTGC AATACATCAA TGAGTAGTAG   
  
  
- TAATAAACGT AAGCCTTTAG GATAGAAGTG CAAATTGCTA AAATGTTGTA CTACATACTT AATCTTCTTT   
  
  
- TTATTACAAT AATGCGTTCT TAAGAAGAGA GAGGTTTGGA GATATTGAGA GGGAGGGGAG AGAAAGTAAA   
  
  
- CCACGTCCAC CACCGCGCTT CTACAGGAAA ATCGTCTCCA CCCCACCGAT TTTCTTTATC CCCATGACGC   
  
  
- AAAACCTAAG ACCCAACACT CAAGACCCAA GATTTAAATA AGCAATTGAG GTCGAAGAGC AGGATGAAGC   
  
  
- CCAAGTCTAG AACGACAAAG CAGAAGTAGG AGACAGTAAC GGACAGTTTC AGCTGCTAAC GCAACCAGGA   
  
  
- GCAGTCCAAG TCTAGAGTGA GGAGTAAAAG GGAAGCCTAA GGATAAAAAA GTTGAAAGAC TTTAAATACG   
  
  
- GTTAGAAAAA GCTATAAAAA GACCCAAAAC ATACTTGTAT ACACCCAACT TCAACTTCTC CCCTCCCTCT   
  
  
- CAATATCTCC AAACCTCTCT CTTCTTCTAC TTTCTCTCAC CCCTTCTTGC TCCTCCCCCT TTTCTTTCTA   
  
  
- TGAAAGTACC AAATCCCTCA CACACTTTCA AACTCTTCCA CAACCCACTC TCACTCTCCC TCTCTCACAA   
  
  
- CGCCGTCGAT ACCCACCTAG ACCCAAGAA

+     TGACG-motif

| Site Name | Organism | Position | Strand | Matrix score. | sequence | function |
| --- | --- | --- | --- | --- | --- | --- |
| TGACG-motif | Hordeum vulgare | 417 | - | 5 | TGACG | cis-acting regulatory element involved in the MeJA-responsiveness |
| TGACG-motif | Hordeum vulgare | 1191 | - | 5 | TGACG | cis-acting regulatory element involved in the MeJA-responsiveness |

>037815   
+ TTTCAGATGC TTCTCACAGA TATTCAAAGT TTGGTTGATT CAACTATTCA ACAAGTAGGA AACTAATTAG   
  
  
+ CACAAAGAAA AACAAATGGG GGGTCTGAAG ATATTTTGGG ATTAAAATAC GAGGGGTGGA TGGGTATATG   
  
  
+ TAATGTAAGA GAGGAGGAAG GTAGCATGCA GTAGCAGAAG CGATAAAAAA ACACGACAGT GAAAAGGAAC   
  
  
+ ACGAAGACGA CTTGGAATAC GGTTGGGAAG GGAATATGAG TGTTACATCG CATGTTTTTA TACAGTGGTG   
  
  
+ AGATATTTTA TTTTTAAGTT ATTAACTTTT TAACCCACAT ATCTTACCAG TAATATAATG ATACGTAGTG   
  
  
+ TACTATCTCA TATGTCGGTC ACATTAAAAA AAATTTCTCA ATTATCATTT GATGTTATAT TTGACACGTC   
  
  
+ AATTCGTCTC TTTGATTAAT AATTATATAA ATTATTTATC ACATCATATA ATACACTGTA CACACCAATA   
  
  
+ TAATATAAAC ATAAACATGT GATTTTCCTG ATATTACTAT GAATGAAATT ATATACAATA TTATTTATAA   
  
  
+ TGTTGTCCTC AAGGTGGAGA TTTCGAGTTC AAATTATACA TATACAAGAG ATTCATGCAA GTATTCAATA   
  
  
+ TTAAATTGTG ATCACCCCTT AATCATTTGT GAAAAAGAAA AAGTTAACTT GCCAATCTCA TTCCTCTTAA   
  
  
+ TAATTAACAA TTTAGCTTAT TAATGCTATC ATTTGTTAAA AATTTAAAAT ATGAGAACTT TTATGATACT   
  
  
+ TTTTTATAGA AGTTAATAGT ATCTCGTTTT AATAAAATTA TTTGCGGACG TTATGTAGTT ACTCATCATC   
  
  
+ ATTATTTGCA TTCGGAAATC CTATCTTCAC GTTTAACGAT TTTACAACAT GATGTATGAA TTAGAAGAAA   
  
  
+ AATAATGTTA TTACGCAAGA ATTCTTCTCT CTCCAAACCT CTATAACTCT CCCTCCCCTC TCTTTCATTT   
  
  
+ GGTGCAGGTG GTGGCGCGAA GATGTCCTTT TAGCAGAGGT GGGGTGGCTA AAAGAAATAG GGGTACTGCG   
  
  
+ TTTTGGATTC TGGGTTGTGA GTTCTGGGTT CTAAATTTAT TCGTTAACTC CAGCTTCTCG TCCTACTTCG   
  
  
+ GGTTCAGATC TTGCTGTTTC GTCTTCATCC TCTGTCATTG CCTGTCAAAG TCGACGATTG CGTTGGTCCT   
  
  
+ CGTCAGGTTC AGATCTCACT CCTCATTTTC CCTTCGGATT CCTATTTTTT CAACTTTCTG AAATTTATGC   
  
  
+ CAATCTTTTT CGATATTTTT CTGGGTTTTG TATGAACATA TGTGGGTTGA AGTTGAAGAG GGGAGGGAGA   
  
  
+ GTTATAGAGG TTTGGAGAGA GAAGAAGATG AAAGAGAGTG GGGAAGAACG AGGAGGGGGA AAAGAAAGAT   
  
  
+ ACTTTCATGG TTTAGGGAGT GTGTGAAAGT TTGAGAAGGT GTTGGGTGAG AGTGAGAGGG AGAGAGTGTT   
  
  
+ GCGGCAGCTA TGGGTGGATC TGGGTTCTT  

- AAAGTCTACG AAGAGTGTCT ATAAGTTTCA AACCAACTAA GTTGATAAGT TGTTCATCCT TTGATTAATC   
  
  
- GTGTTTCTTT TTGTTTACCC CCCAGACTTC TATAAAACCC TAATTTTATG CTCCCCACCT ACCCATATAC   
  
  
- ATTACATTCT CTCCTCCTTC CATCGTACGT CATCGTCTTC GCTATTTTTT TGTGCTGTCA CTTTTCCTTG   
  
  
- TGCTTCTGCT GAACCTTATG CCAACCCTTC CCTTATACTC ACAATGTAGC GTACAAAAAT ATGTCACCAC   
  
  
- TCTATAAAAT AAAAATTCAA TAATTGAAAA ATTGGGTGTA TAGAATGGTC ATTATATTAC TATGCATCAC   
  
  
- ATGATAGAGT ATACAGCCAG TGTAATTTTT TTTAAAGAGT TAATAGTAAA CTACAATATA AACTGTGCAG   
  
  
- TTAAGCAGAG AAACTAATTA TTAATATATT TAATAAATAG TGTAGTATAT TATGTGACAT GTGTGGTTAT   
  
  
- ATTATATTTG TATTTGTACA CTAAAAGGAC TATAATGATA CTTACTTTAA TATATGTTAT AATAAATATT   
  
  
- ACAACAGGAG TTCCACCTCT AAAGCTCAAG TTTAATATGT ATATGTTCTC TAAGTACGTT CATAAGTTAT   
  
  
- AATTTAACAC TAGTGGGGAA TTAGTAAACA CTTTTTCTTT TTCAATTGAA CGGTTAGAGT AAGGAGAATT   
  
  
- ATTAATTGTT AAATCGAATA ATTACGATAG TAAACAATTT TTAAATTTTA TACTCTTGAA AATACTATGA   
  
  
- AAAAATATCT TCAATTATCA TAGAGCAAAA TTATTTTAAT AAACGCCTGC AATACATCAA TGAGTAGTAG   
  
  
- TAATAAACGT AAGCCTTTAG GATAGAAGTG CAAATTGCTA AAATGTTGTA CTACATACTT AATCTTCTTT   
  
  
- TTATTACAAT AATGCGTTCT TAAGAAGAGA GAGGTTTGGA GATATTGAGA GGGAGGGGAG AGAAAGTAAA   
  
  
- CCACGTCCAC CACCGCGCTT CTACAGGAAA ATCGTCTCCA CCCCACCGAT TTTCTTTATC CCCATGACGC   
  
  
- AAAACCTAAG ACCCAACACT CAAGACCCAA GATTTAAATA AGCAATTGAG GTCGAAGAGC AGGATGAAGC   
  
  
- CCAAGTCTAG AACGACAAAG CAGAAGTAGG AGACAGTAAC GGACAGTTTC AGCTGCTAAC GCAACCAGGA   
  
  
- GCAGTCCAAG TCTAGAGTGA GGAGTAAAAG GGAAGCCTAA GGATAAAAAA GTTGAAAGAC TTTAAATACG   
  
  
- GTTAGAAAAA GCTATAAAAA GACCCAAAAC ATACTTGTAT ACACCCAACT TCAACTTCTC CCCTCCCTCT   
  
  
- CAATATCTCC AAACCTCTCT CTTCTTCTAC TTTCTCTCAC CCCTTCTTGC TCCTCCCCCT TTTCTTTCTA   
  
  
- TGAAAGTACC AAATCCCTCA CACACTTTCA AACTCTTCCA CAACCCACTC TCACTCTCCC TCTCTCACAA   
  
  
- CGCCGTCGAT ACCCACCTAG ACCCAAGAA

+     Unnamed\_\_4

| Site Name | Organism | Position | Strand | Matrix score. | sequence | function |
| --- | --- | --- | --- | --- | --- | --- |
| Unnamed\_\_4 | Petroselinum hortense | 1459 | - | 4 | CTCC |  |
| Unnamed\_\_4 | Petroselinum hortense | 1416 | - | 4 | CTCC |  |
| Unnamed\_\_4 | Petroselinum hortense | 1382 | - | 4 | CTCC |  |
| Unnamed\_\_4 | Petroselinum hortense | 1344 | - | 4 | CTCC |  |
| Unnamed\_\_4 | Petroselinum hortense | 1322 | - | 4 | CTCC |  |
| Unnamed\_\_4 | Petroselinum hortense | 1098 | + | 4 | CTCC |  |
| Unnamed\_\_4 | Petroselinum hortense | 963 | + | 4 | CTCC |  |
| Unnamed\_\_4 | Petroselinum hortense | 1326 | - | 4 | CTCC |  |
| Unnamed\_\_4 | Petroselinum hortense | 959 | + | 4 | CTCC |  |
| Unnamed\_\_4 | Petroselinum hortense | 941 | + | 4 | CTCC |  |
| Unnamed\_\_4 | Petroselinum hortense | 153 | - | 4 | CTCC |  |
| Unnamed\_\_4 | Petroselinum hortense | 1209 | + | 4 | CTCC |  |
| Unnamed\_\_4 | Petroselinum hortense | 576 | - | 4 | CTCC |  |

>037815   
+ TTTCAGATGC TTCTCACAGA TATTCAAAGT TTGGTTGATT CAACTATTCA ACAAGTAGGA AACTAATTAG   
  
  
+ CACAAAGAAA AACAAATGGG GGGTCTGAAG ATATTTTGGG ATTAAAATAC GAGGGGTGGA TGGGTATATG   
  
  
+ TAATGTAAGA GAGGAGGAAG GTAGCATGCA GTAGCAGAAG CGATAAAAAA ACACGACAGT GAAAAGGAAC   
  
  
+ ACGAAGACGA CTTGGAATAC GGTTGGGAAG GGAATATGAG TGTTACATCG CATGTTTTTA TACAGTGGTG   
  
  
+ AGATATTTTA TTTTTAAGTT ATTAACTTTT TAACCCACAT ATCTTACCAG TAATATAATG ATACGTAGTG   
  
  
+ TACTATCTCA TATGTCGGTC ACATTAAAAA AAATTTCTCA ATTATCATTT GATGTTATAT TTGACACGTC   
  
  
+ AATTCGTCTC TTTGATTAAT AATTATATAA ATTATTTATC ACATCATATA ATACACTGTA CACACCAATA   
  
  
+ TAATATAAAC ATAAACATGT GATTTTCCTG ATATTACTAT GAATGAAATT ATATACAATA TTATTTATAA   
  
  
+ TGTTGTCCTC AAGGTGGAGA TTTCGAGTTC AAATTATACA TATACAAGAG ATTCATGCAA GTATTCAATA   
  
  
+ TTAAATTGTG ATCACCCCTT AATCATTTGT GAAAAAGAAA AAGTTAACTT GCCAATCTCA TTCCTCTTAA   
  
  
+ TAATTAACAA TTTAGCTTAT TAATGCTATC ATTTGTTAAA AATTTAAAAT ATGAGAACTT TTATGATACT   
  
  
+ TTTTTATAGA AGTTAATAGT ATCTCGTTTT AATAAAATTA TTTGCGGACG TTATGTAGTT ACTCATCATC   
  
  
+ ATTATTTGCA TTCGGAAATC CTATCTTCAC GTTTAACGAT TTTACAACAT GATGTATGAA TTAGAAGAAA   
  
  
+ AATAATGTTA TTACGCAAGA ATTCTTCTCT CTCCAAACCT CTATAACTCT CCCTCCCCTC TCTTTCATTT   
  
  
+ GGTGCAGGTG GTGGCGCGAA GATGTCCTTT TAGCAGAGGT GGGGTGGCTA AAAGAAATAG GGGTACTGCG   
  
  
+ TTTTGGATTC TGGGTTGTGA GTTCTGGGTT CTAAATTTAT TCGTTAACTC CAGCTTCTCG TCCTACTTCG   
  
  
+ GGTTCAGATC TTGCTGTTTC GTCTTCATCC TCTGTCATTG CCTGTCAAAG TCGACGATTG CGTTGGTCCT   
  
  
+ CGTCAGGTTC AGATCTCACT CCTCATTTTC CCTTCGGATT CCTATTTTTT CAACTTTCTG AAATTTATGC   
  
  
+ CAATCTTTTT CGATATTTTT CTGGGTTTTG TATGAACATA TGTGGGTTGA AGTTGAAGAG GGGAGGGAGA   
  
  
+ GTTATAGAGG TTTGGAGAGA GAAGAAGATG AAAGAGAGTG GGGAAGAACG AGGAGGGGGA AAAGAAAGAT   
  
  
+ ACTTTCATGG TTTAGGGAGT GTGTGAAAGT TTGAGAAGGT GTTGGGTGAG AGTGAGAGGG AGAGAGTGTT   
  
  
+ GCGGCAGCTA TGGGTGGATC TGGGTTCTT  

- AAAGTCTACG AAGAGTGTCT ATAAGTTTCA AACCAACTAA GTTGATAAGT TGTTCATCCT TTGATTAATC   
  
  
- GTGTTTCTTT TTGTTTACCC CCCAGACTTC TATAAAACCC TAATTTTATG CTCCCCACCT ACCCATATAC   
  
  
- ATTACATTCT CTCCTCCTTC CATCGTACGT CATCGTCTTC GCTATTTTTT TGTGCTGTCA CTTTTCCTTG   
  
  
- TGCTTCTGCT GAACCTTATG CCAACCCTTC CCTTATACTC ACAATGTAGC GTACAAAAAT ATGTCACCAC   
  
  
- TCTATAAAAT AAAAATTCAA TAATTGAAAA ATTGGGTGTA TAGAATGGTC ATTATATTAC TATGCATCAC   
  
  
- ATGATAGAGT ATACAGCCAG TGTAATTTTT TTTAAAGAGT TAATAGTAAA CTACAATATA AACTGTGCAG   
  
  
- TTAAGCAGAG AAACTAATTA TTAATATATT TAATAAATAG TGTAGTATAT TATGTGACAT GTGTGGTTAT   
  
  
- ATTATATTTG TATTTGTACA CTAAAAGGAC TATAATGATA CTTACTTTAA TATATGTTAT AATAAATATT   
  
  
- ACAACAGGAG TTCCACCTCT AAAGCTCAAG TTTAATATGT ATATGTTCTC TAAGTACGTT CATAAGTTAT   
  
  
- AATTTAACAC TAGTGGGGAA TTAGTAAACA CTTTTTCTTT TTCAATTGAA CGGTTAGAGT AAGGAGAATT   
  
  
- ATTAATTGTT AAATCGAATA ATTACGATAG TAAACAATTT TTAAATTTTA TACTCTTGAA AATACTATGA   
  
  
- AAAAATATCT TCAATTATCA TAGAGCAAAA TTATTTTAAT AAACGCCTGC AATACATCAA TGAGTAGTAG   
  
  
- TAATAAACGT AAGCCTTTAG GATAGAAGTG CAAATTGCTA AAATGTTGTA CTACATACTT AATCTTCTTT   
  
  
- TTATTACAAT AATGCGTTCT TAAGAAGAGA GAGGTTTGGA GATATTGAGA GGGAGGGGAG AGAAAGTAAA   
  
  
- CCACGTCCAC CACCGCGCTT CTACAGGAAA ATCGTCTCCA CCCCACCGAT TTTCTTTATC CCCATGACGC   
  
  
- AAAACCTAAG ACCCAACACT CAAGACCCAA GATTTAAATA AGCAATTGAG GTCGAAGAGC AGGATGAAGC   
  
  
- CCAAGTCTAG AACGACAAAG CAGAAGTAGG AGACAGTAAC GGACAGTTTC AGCTGCTAAC GCAACCAGGA   
  
  
- GCAGTCCAAG TCTAGAGTGA GGAGTAAAAG GGAAGCCTAA GGATAAAAAA GTTGAAAGAC TTTAAATACG   
  
  
- GTTAGAAAAA GCTATAAAAA GACCCAAAAC ATACTTGTAT ACACCCAACT TCAACTTCTC CCCTCCCTCT   
  
  
- CAATATCTCC AAACCTCTCT CTTCTTCTAC TTTCTCTCAC CCCTTCTTGC TCCTCCCCCT TTTCTTTCTA   
  
  
- TGAAAGTACC AAATCCCTCA CACACTTTCA AACTCTTCCA CAACCCACTC TCACTCTCCC TCTCTCACAA   
  
  
- CGCCGTCGAT ACCCACCTAG ACCCAAGAA

+     WRE3

| Site Name | Organism | Position | Strand | Matrix score. | sequence | function |
| --- | --- | --- | --- | --- | --- | --- |
| WRE3 | Pisum sativum | 986 | - | 6 | CCACCT |  |
| WRE3 | Pisum sativum | 572 | - | 6 | CCACCT |  |
| WRE3 | Pisum sativum | 1017 | - | 6 | CCACCT |  |

>037815   
+ TTTCAGATGC TTCTCACAGA TATTCAAAGT TTGGTTGATT CAACTATTCA ACAAGTAGGA AACTAATTAG   
  
  
+ CACAAAGAAA AACAAATGGG GGGTCTGAAG ATATTTTGGG ATTAAAATAC GAGGGGTGGA TGGGTATATG   
  
  
+ TAATGTAAGA GAGGAGGAAG GTAGCATGCA GTAGCAGAAG CGATAAAAAA ACACGACAGT GAAAAGGAAC   
  
  
+ ACGAAGACGA CTTGGAATAC GGTTGGGAAG GGAATATGAG TGTTACATCG CATGTTTTTA TACAGTGGTG   
  
  
+ AGATATTTTA TTTTTAAGTT ATTAACTTTT TAACCCACAT ATCTTACCAG TAATATAATG ATACGTAGTG   
  
  
+ TACTATCTCA TATGTCGGTC ACATTAAAAA AAATTTCTCA ATTATCATTT GATGTTATAT TTGACACGTC   
  
  
+ AATTCGTCTC TTTGATTAAT AATTATATAA ATTATTTATC ACATCATATA ATACACTGTA CACACCAATA   
  
  
+ TAATATAAAC ATAAACATGT GATTTTCCTG ATATTACTAT GAATGAAATT ATATACAATA TTATTTATAA   
  
  
+ TGTTGTCCTC AAGGTGGAGA TTTCGAGTTC AAATTATACA TATACAAGAG ATTCATGCAA GTATTCAATA   
  
  
+ TTAAATTGTG ATCACCCCTT AATCATTTGT GAAAAAGAAA AAGTTAACTT GCCAATCTCA TTCCTCTTAA   
  
  
+ TAATTAACAA TTTAGCTTAT TAATGCTATC ATTTGTTAAA AATTTAAAAT ATGAGAACTT TTATGATACT   
  
  
+ TTTTTATAGA AGTTAATAGT ATCTCGTTTT AATAAAATTA TTTGCGGACG TTATGTAGTT ACTCATCATC   
  
  
+ ATTATTTGCA TTCGGAAATC CTATCTTCAC GTTTAACGAT TTTACAACAT GATGTATGAA TTAGAAGAAA   
  
  
+ AATAATGTTA TTACGCAAGA ATTCTTCTCT CTCCAAACCT CTATAACTCT CCCTCCCCTC TCTTTCATTT   
  
  
+ GGTGCAGGTG GTGGCGCGAA GATGTCCTTT TAGCAGAGGT GGGGTGGCTA AAAGAAATAG GGGTACTGCG   
  
  
+ TTTTGGATTC TGGGTTGTGA GTTCTGGGTT CTAAATTTAT TCGTTAACTC CAGCTTCTCG TCCTACTTCG   
  
  
+ GGTTCAGATC TTGCTGTTTC GTCTTCATCC TCTGTCATTG CCTGTCAAAG TCGACGATTG CGTTGGTCCT   
  
  
+ CGTCAGGTTC AGATCTCACT CCTCATTTTC CCTTCGGATT CCTATTTTTT CAACTTTCTG AAATTTATGC   
  
  
+ CAATCTTTTT CGATATTTTT CTGGGTTTTG TATGAACATA TGTGGGTTGA AGTTGAAGAG GGGAGGGAGA   
  
  
+ GTTATAGAGG TTTGGAGAGA GAAGAAGATG AAAGAGAGTG GGGAAGAACG AGGAGGGGGA AAAGAAAGAT   
  
  
+ ACTTTCATGG TTTAGGGAGT GTGTGAAAGT TTGAGAAGGT GTTGGGTGAG AGTGAGAGGG AGAGAGTGTT   
  
  
+ GCGGCAGCTA TGGGTGGATC TGGGTTCTT  

- AAAGTCTACG AAGAGTGTCT ATAAGTTTCA AACCAACTAA GTTGATAAGT TGTTCATCCT TTGATTAATC   
  
  
- GTGTTTCTTT TTGTTTACCC CCCAGACTTC TATAAAACCC TAATTTTATG CTCCCCACCT ACCCATATAC   
  
  
- ATTACATTCT CTCCTCCTTC CATCGTACGT CATCGTCTTC GCTATTTTTT TGTGCTGTCA CTTTTCCTTG   
  
  
- TGCTTCTGCT GAACCTTATG CCAACCCTTC CCTTATACTC ACAATGTAGC GTACAAAAAT ATGTCACCAC   
  
  
- TCTATAAAAT AAAAATTCAA TAATTGAAAA ATTGGGTGTA TAGAATGGTC ATTATATTAC TATGCATCAC   
  
  
- ATGATAGAGT ATACAGCCAG TGTAATTTTT TTTAAAGAGT TAATAGTAAA CTACAATATA AACTGTGCAG   
  
  
- TTAAGCAGAG AAACTAATTA TTAATATATT TAATAAATAG TGTAGTATAT TATGTGACAT GTGTGGTTAT   
  
  
- ATTATATTTG TATTTGTACA CTAAAAGGAC TATAATGATA CTTACTTTAA TATATGTTAT AATAAATATT   
  
  
- ACAACAGGAG TTCCACCTCT AAAGCTCAAG TTTAATATGT ATATGTTCTC TAAGTACGTT CATAAGTTAT   
  
  
- AATTTAACAC TAGTGGGGAA TTAGTAAACA CTTTTTCTTT TTCAATTGAA CGGTTAGAGT AAGGAGAATT   
  
  
- ATTAATTGTT AAATCGAATA ATTACGATAG TAAACAATTT TTAAATTTTA TACTCTTGAA AATACTATGA   
  
  
- AAAAATATCT TCAATTATCA TAGAGCAAAA TTATTTTAAT AAACGCCTGC AATACATCAA TGAGTAGTAG   
  
  
- TAATAAACGT AAGCCTTTAG GATAGAAGTG CAAATTGCTA AAATGTTGTA CTACATACTT AATCTTCTTT   
  
  
- TTATTACAAT AATGCGTTCT TAAGAAGAGA GAGGTTTGGA GATATTGAGA GGGAGGGGAG AGAAAGTAAA   
  
  
- CCACGTCCAC CACCGCGCTT CTACAGGAAA ATCGTCTCCA CCCCACCGAT TTTCTTTATC CCCATGACGC   
  
  
- AAAACCTAAG ACCCAACACT CAAGACCCAA GATTTAAATA AGCAATTGAG GTCGAAGAGC AGGATGAAGC   
  
  
- CCAAGTCTAG AACGACAAAG CAGAAGTAGG AGACAGTAAC GGACAGTTTC AGCTGCTAAC GCAACCAGGA   
  
  
- GCAGTCCAAG TCTAGAGTGA GGAGTAAAAG GGAAGCCTAA GGATAAAAAA GTTGAAAGAC TTTAAATACG   
  
  
- GTTAGAAAAA GCTATAAAAA GACCCAAAAC ATACTTGTAT ACACCCAACT TCAACTTCTC CCCTCCCTCT   
  
  
- CAATATCTCC AAACCTCTCT CTTCTTCTAC TTTCTCTCAC CCCTTCTTGC TCCTCCCCCT TTTCTTTCTA   
  
  
- TGAAAGTACC AAATCCCTCA CACACTTTCA AACTCTTCCA CAACCCACTC TCACTCTCCC TCTCTCACAA   
  
  
- CGCCGTCGAT ACCCACCTAG ACCCAAGAA

+     as-1

| Site Name | Organism | Position | Strand | Matrix score. | sequence | function |
| --- | --- | --- | --- | --- | --- | --- |
| as-1 | Arabidopsis thaliana | 417 | - | 5 | TGACG |  |
| as-1 | Arabidopsis thaliana | 1191 | - | 5 | TGACG |  |

>037815   
+ TTTCAGATGC TTCTCACAGA TATTCAAAGT TTGGTTGATT CAACTATTCA ACAAGTAGGA AACTAATTAG   
  
  
+ CACAAAGAAA AACAAATGGG GGGTCTGAAG ATATTTTGGG ATTAAAATAC GAGGGGTGGA TGGGTATATG   
  
  
+ TAATGTAAGA GAGGAGGAAG GTAGCATGCA GTAGCAGAAG CGATAAAAAA ACACGACAGT GAAAAGGAAC   
  
  
+ ACGAAGACGA CTTGGAATAC GGTTGGGAAG GGAATATGAG TGTTACATCG CATGTTTTTA TACAGTGGTG   
  
  
+ AGATATTTTA TTTTTAAGTT ATTAACTTTT TAACCCACAT ATCTTACCAG TAATATAATG ATACGTAGTG   
  
  
+ TACTATCTCA TATGTCGGTC ACATTAAAAA AAATTTCTCA ATTATCATTT GATGTTATAT TTGACACGTC   
  
  
+ AATTCGTCTC TTTGATTAAT AATTATATAA ATTATTTATC ACATCATATA ATACACTGTA CACACCAATA   
  
  
+ TAATATAAAC ATAAACATGT GATTTTCCTG ATATTACTAT GAATGAAATT ATATACAATA TTATTTATAA   
  
  
+ TGTTGTCCTC AAGGTGGAGA TTTCGAGTTC AAATTATACA TATACAAGAG ATTCATGCAA GTATTCAATA   
  
  
+ TTAAATTGTG ATCACCCCTT AATCATTTGT GAAAAAGAAA AAGTTAACTT GCCAATCTCA TTCCTCTTAA   
  
  
+ TAATTAACAA TTTAGCTTAT TAATGCTATC ATTTGTTAAA AATTTAAAAT ATGAGAACTT TTATGATACT   
  
  
+ TTTTTATAGA AGTTAATAGT ATCTCGTTTT AATAAAATTA TTTGCGGACG TTATGTAGTT ACTCATCATC   
  
  
+ ATTATTTGCA TTCGGAAATC CTATCTTCAC GTTTAACGAT TTTACAACAT GATGTATGAA TTAGAAGAAA   
  
  
+ AATAATGTTA TTACGCAAGA ATTCTTCTCT CTCCAAACCT CTATAACTCT CCCTCCCCTC TCTTTCATTT   
  
  
+ GGTGCAGGTG GTGGCGCGAA GATGTCCTTT TAGCAGAGGT GGGGTGGCTA AAAGAAATAG GGGTACTGCG   
  
  
+ TTTTGGATTC TGGGTTGTGA GTTCTGGGTT CTAAATTTAT TCGTTAACTC CAGCTTCTCG TCCTACTTCG   
  
  
+ GGTTCAGATC TTGCTGTTTC GTCTTCATCC TCTGTCATTG CCTGTCAAAG TCGACGATTG CGTTGGTCCT   
  
  
+ CGTCAGGTTC AGATCTCACT CCTCATTTTC CCTTCGGATT CCTATTTTTT CAACTTTCTG AAATTTATGC   
  
  
+ CAATCTTTTT CGATATTTTT CTGGGTTTTG TATGAACATA TGTGGGTTGA AGTTGAAGAG GGGAGGGAGA   
  
  
+ GTTATAGAGG TTTGGAGAGA GAAGAAGATG AAAGAGAGTG GGGAAGAACG AGGAGGGGGA AAAGAAAGAT   
  
  
+ ACTTTCATGG TTTAGGGAGT GTGTGAAAGT TTGAGAAGGT GTTGGGTGAG AGTGAGAGGG AGAGAGTGTT   
  
  
+ GCGGCAGCTA TGGGTGGATC TGGGTTCTT  

- AAAGTCTACG AAGAGTGTCT ATAAGTTTCA AACCAACTAA GTTGATAAGT TGTTCATCCT TTGATTAATC   
  
  
- GTGTTTCTTT TTGTTTACCC CCCAGACTTC TATAAAACCC TAATTTTATG CTCCCCACCT ACCCATATAC   
  
  
- ATTACATTCT CTCCTCCTTC CATCGTACGT CATCGTCTTC GCTATTTTTT TGTGCTGTCA CTTTTCCTTG   
  
  
- TGCTTCTGCT GAACCTTATG CCAACCCTTC CCTTATACTC ACAATGTAGC GTACAAAAAT ATGTCACCAC   
  
  
- TCTATAAAAT AAAAATTCAA TAATTGAAAA ATTGGGTGTA TAGAATGGTC ATTATATTAC TATGCATCAC   
  
  
- ATGATAGAGT ATACAGCCAG TGTAATTTTT TTTAAAGAGT TAATAGTAAA CTACAATATA AACTGTGCAG   
  
  
- TTAAGCAGAG AAACTAATTA TTAATATATT TAATAAATAG TGTAGTATAT TATGTGACAT GTGTGGTTAT   
  
  
- ATTATATTTG TATTTGTACA CTAAAAGGAC TATAATGATA CTTACTTTAA TATATGTTAT AATAAATATT   
  
  
- ACAACAGGAG TTCCACCTCT AAAGCTCAAG TTTAATATGT ATATGTTCTC TAAGTACGTT CATAAGTTAT   
  
  
- AATTTAACAC TAGTGGGGAA TTAGTAAACA CTTTTTCTTT TTCAATTGAA CGGTTAGAGT AAGGAGAATT   
  
  
- ATTAATTGTT AAATCGAATA ATTACGATAG TAAACAATTT TTAAATTTTA TACTCTTGAA AATACTATGA   
  
  
- AAAAATATCT TCAATTATCA TAGAGCAAAA TTATTTTAAT AAACGCCTGC AATACATCAA TGAGTAGTAG   
  
  
- TAATAAACGT AAGCCTTTAG GATAGAAGTG CAAATTGCTA AAATGTTGTA CTACATACTT AATCTTCTTT   
  
  
- TTATTACAAT AATGCGTTCT TAAGAAGAGA GAGGTTTGGA GATATTGAGA GGGAGGGGAG AGAAAGTAAA   
  
  
- CCACGTCCAC CACCGCGCTT CTACAGGAAA ATCGTCTCCA CCCCACCGAT TTTCTTTATC CCCATGACGC   
  
  
- AAAACCTAAG ACCCAACACT CAAGACCCAA GATTTAAATA AGCAATTGAG GTCGAAGAGC AGGATGAAGC   
  
  
- CCAAGTCTAG AACGACAAAG CAGAAGTAGG AGACAGTAAC GGACAGTTTC AGCTGCTAAC GCAACCAGGA   
  
  
- GCAGTCCAAG TCTAGAGTGA GGAGTAAAAG GGAAGCCTAA GGATAAAAAA GTTGAAAGAC TTTAAATACG   
  
  
- GTTAGAAAAA GCTATAAAAA GACCCAAAAC ATACTTGTAT ACACCCAACT TCAACTTCTC CCCTCCCTCT   
  
  
- CAATATCTCC AAACCTCTCT CTTCTTCTAC TTTCTCTCAC CCCTTCTTGC TCCTCCCCCT TTTCTTTCTA   
  
  
- TGAAAGTACC AAATCCCTCA CACACTTTCA AACTCTTCCA CAACCCACTC TCACTCTCCC TCTCTCACAA   
  
  
- CGCCGTCGAT ACCCACCTAG ACCCAAGAA

+     box S

| Site Name | Organism | Position | Strand | Matrix score. | sequence | function |
| --- | --- | --- | --- | --- | --- | --- |
| box S | Arabidopsis thaliana | 1023 | - | 7 | AGCCACC |  |

>037815   
+ TTTCAGATGC TTCTCACAGA TATTCAAAGT TTGGTTGATT CAACTATTCA ACAAGTAGGA AACTAATTAG   
  
  
+ CACAAAGAAA AACAAATGGG GGGTCTGAAG ATATTTTGGG ATTAAAATAC GAGGGGTGGA TGGGTATATG   
  
  
+ TAATGTAAGA GAGGAGGAAG GTAGCATGCA GTAGCAGAAG CGATAAAAAA ACACGACAGT GAAAAGGAAC   
  
  
+ ACGAAGACGA CTTGGAATAC GGTTGGGAAG GGAATATGAG TGTTACATCG CATGTTTTTA TACAGTGGTG   
  
  
+ AGATATTTTA TTTTTAAGTT ATTAACTTTT TAACCCACAT ATCTTACCAG TAATATAATG ATACGTAGTG   
  
  
+ TACTATCTCA TATGTCGGTC ACATTAAAAA AAATTTCTCA ATTATCATTT GATGTTATAT TTGACACGTC   
  
  
+ AATTCGTCTC TTTGATTAAT AATTATATAA ATTATTTATC ACATCATATA ATACACTGTA CACACCAATA   
  
  
+ TAATATAAAC ATAAACATGT GATTTTCCTG ATATTACTAT GAATGAAATT ATATACAATA TTATTTATAA   
  
  
+ TGTTGTCCTC AAGGTGGAGA TTTCGAGTTC AAATTATACA TATACAAGAG ATTCATGCAA GTATTCAATA   
  
  
+ TTAAATTGTG ATCACCCCTT AATCATTTGT GAAAAAGAAA AAGTTAACTT GCCAATCTCA TTCCTCTTAA   
  
  
+ TAATTAACAA TTTAGCTTAT TAATGCTATC ATTTGTTAAA AATTTAAAAT ATGAGAACTT TTATGATACT   
  
  
+ TTTTTATAGA AGTTAATAGT ATCTCGTTTT AATAAAATTA TTTGCGGACG TTATGTAGTT ACTCATCATC   
  
  
+ ATTATTTGCA TTCGGAAATC CTATCTTCAC GTTTAACGAT TTTACAACAT GATGTATGAA TTAGAAGAAA   
  
  
+ AATAATGTTA TTACGCAAGA ATTCTTCTCT CTCCAAACCT CTATAACTCT CCCTCCCCTC TCTTTCATTT   
  
  
+ GGTGCAGGTG GTGGCGCGAA GATGTCCTTT TAGCAGAGGT GGGGTGGCTA AAAGAAATAG GGGTACTGCG   
  
  
+ TTTTGGATTC TGGGTTGTGA GTTCTGGGTT CTAAATTTAT TCGTTAACTC CAGCTTCTCG TCCTACTTCG   
  
  
+ GGTTCAGATC TTGCTGTTTC GTCTTCATCC TCTGTCATTG CCTGTCAAAG TCGACGATTG CGTTGGTCCT   
  
  
+ CGTCAGGTTC AGATCTCACT CCTCATTTTC CCTTCGGATT CCTATTTTTT CAACTTTCTG AAATTTATGC   
  
  
+ CAATCTTTTT CGATATTTTT CTGGGTTTTG TATGAACATA TGTGGGTTGA AGTTGAAGAG GGGAGGGAGA   
  
  
+ GTTATAGAGG TTTGGAGAGA GAAGAAGATG AAAGAGAGTG GGGAAGAACG AGGAGGGGGA AAAGAAAGAT   
  
  
+ ACTTTCATGG TTTAGGGAGT GTGTGAAAGT TTGAGAAGGT GTTGGGTGAG AGTGAGAGGG AGAGAGTGTT   
  
  
+ GCGGCAGCTA TGGGTGGATC TGGGTTCTT  

- AAAGTCTACG AAGAGTGTCT ATAAGTTTCA AACCAACTAA GTTGATAAGT TGTTCATCCT TTGATTAATC   
  
  
- GTGTTTCTTT TTGTTTACCC CCCAGACTTC TATAAAACCC TAATTTTATG CTCCCCACCT ACCCATATAC   
  
  
- ATTACATTCT CTCCTCCTTC CATCGTACGT CATCGTCTTC GCTATTTTTT TGTGCTGTCA CTTTTCCTTG   
  
  
- TGCTTCTGCT GAACCTTATG CCAACCCTTC CCTTATACTC ACAATGTAGC GTACAAAAAT ATGTCACCAC   
  
  
- TCTATAAAAT AAAAATTCAA TAATTGAAAA ATTGGGTGTA TAGAATGGTC ATTATATTAC TATGCATCAC   
  
  
- ATGATAGAGT ATACAGCCAG TGTAATTTTT TTTAAAGAGT TAATAGTAAA CTACAATATA AACTGTGCAG   
  
  
- TTAAGCAGAG AAACTAATTA TTAATATATT TAATAAATAG TGTAGTATAT TATGTGACAT GTGTGGTTAT   
  
  
- ATTATATTTG TATTTGTACA CTAAAAGGAC TATAATGATA CTTACTTTAA TATATGTTAT AATAAATATT   
  
  
- ACAACAGGAG TTCCACCTCT AAAGCTCAAG TTTAATATGT ATATGTTCTC TAAGTACGTT CATAAGTTAT   
  
  
- AATTTAACAC TAGTGGGGAA TTAGTAAACA CTTTTTCTTT TTCAATTGAA CGGTTAGAGT AAGGAGAATT   
  
  
- ATTAATTGTT AAATCGAATA ATTACGATAG TAAACAATTT TTAAATTTTA TACTCTTGAA AATACTATGA   
  
  
- AAAAATATCT TCAATTATCA TAGAGCAAAA TTATTTTAAT AAACGCCTGC AATACATCAA TGAGTAGTAG   
  
  
- TAATAAACGT AAGCCTTTAG GATAGAAGTG CAAATTGCTA AAATGTTGTA CTACATACTT AATCTTCTTT   
  
  
- TTATTACAAT AATGCGTTCT TAAGAAGAGA GAGGTTTGGA GATATTGAGA GGGAGGGGAG AGAAAGTAAA   
  
  
- CCACGTCCAC CACCGCGCTT CTACAGGAAA ATCGTCTCCA CCCCACCGAT TTTCTTTATC CCCATGACGC   
  
  
- AAAACCTAAG ACCCAACACT CAAGACCCAA GATTTAAATA AGCAATTGAG GTCGAAGAGC AGGATGAAGC   
  
  
- CCAAGTCTAG AACGACAAAG CAGAAGTAGG AGACAGTAAC GGACAGTTTC AGCTGCTAAC GCAACCAGGA   
  
  
- GCAGTCCAAG TCTAGAGTGA GGAGTAAAAG GGAAGCCTAA GGATAAAAAA GTTGAAAGAC TTTAAATACG   
  
  
- GTTAGAAAAA GCTATAAAAA GACCCAAAAC ATACTTGTAT ACACCCAACT TCAACTTCTC CCCTCCCTCT   
  
  
- CAATATCTCC AAACCTCTCT CTTCTTCTAC TTTCTCTCAC CCCTTCTTGC TCCTCCCCCT TTTCTTTCTA   
  
  
- TGAAAGTACC AAATCCCTCA CACACTTTCA AACTCTTCCA CAACCCACTC TCACTCTCCC TCTCTCACAA   
  
  
- CGCCGTCGAT ACCCACCTAG ACCCAAGAA
