# Supplementary material for: In Silico Genome-Wide Analysis of Respiratory Burst Oxidase Homolog (RBOH) Family Genes in Five Fruit-Producing Trees, and Potential Functional Analysis on Lignification of Stone Cells in Chinese White Pear
Source: Cells. 2019 May 29;8(6):520. doi: 10.3390/cells8060520 (PMC6627160; doi:10.3390/cells8060520)
Supplement: Supplementary file 1 [file cells-08-00520-s001.zip › Supplementary Material/Spreadsheets S2. Numbers of cis-elements in promoter region of PbRBOHs.tar.zip!/PbRBOHC.html]

Content-Type: text/html; charset=ISO-8859-1


PlantCARE


Webmaster Firefox specific output  
To save the result:
click on the frame with the right mouse button and save the source code as a text file with extension .html  
REFERENCE:PlantCARE: a database of plant cis-acting regulatory elements and a portal to tools for in silico analysis of promoter sequences.  
Lescot, M., Déhais, P., Moreau, Y., De Moor, B., Rouzé ,P.,and Rombauts, S.  
Nucleic Acids Res., Database issue(2002), 30(1):325-327.   


---

>038667   
+ ACGAAAACCG AAGCAATAAC ATTCTATTTA TAGGAGAGGA AAGTCGGCAC AGAGGAAGGT GAGATTAGAC   
  
  
+ CAAGCAAACT TACACCGCCT AAAAGCTATT GATAAGAATG ATGTAAAAGC CCTTTAATTG GCTATGGTTT   
  
  
+ TGGTCCAATT AGAATAGAAC AAACAACTTA ATAAAATAAT GTCAAAAGGA AAGAAAATGA ATAATATGAA   
  
  
+ TATATAGTTC AAATACTACT GTAATGGTGT ACTGAACTTA TTTGGTTCAA AATAGTAGTA ATTAGAGTAT   
  
  
+ AGAAAAATGA AATAAAAGAA TTAACGATTA AATAAGAAAT AATACAAAAC GATATGAAAT TAATAAGTAT   
  
  
+ ATACAACACA TAATTATATA TAATTATTTT GGAAAATATA GGTCCTCACA AGACGCCTAA GTGCTAGCAC   
  
  
+ CTACTTGCTG CTGGCTAGCG CTTAGGTCCT GCTTTTTCTT ATATTATACT CTAAATTATT GTAACTTGGG   
  
  
+ TATAGAAGTG CAGGCAAACT AACCTATCCC ATATTCACCA GATTCACGTT TGCTTTGAAA AGAAGTTTAT   
  
  
+ AGATGAAGAA AATGTTAAAA AGAAAAAAGA GTGATTAGAG ATAACTTGCT TGGATGTGGA TTTTGCTCGG   
  
  
+ GGTAAAGAAG GGCGGGCACA CTGACCTATC CCAGATATAC TATTTTCACA TTTTCTTTGA AAAGAGGTTT   
  
  
+ ACATATAAAG AAAATGTGTA AAAAAAAATA AAAAATAAAA TAAAATAAAG TGATTAAAGA TAACTTTGGA   
  
  
+ TGAGGATTTG GCTCGGGGTA TAGAAGAGTG GATACTTTGA CCTATCTCAA ATTCACCAGA CTCATGTTTG   
  
  
+ CTTTGAAAAG AGGTTTATAG ATAAAGAAAA CGTAAAAAAA AAAAAAATAA ATAAAAAAAA ATGAGTGACT   
  
  
+ AAAGATAACT TACGTGGATG AGAATTTGAG TTGGAGTATA AAAGGACGGA CACATTGACC TATCCCAGAT   
  
  
+ TCATGAGATT CACGTTTACC ATAGAGGTTT ATAGATAAAG AGAAAGTAAA AAAAAGAAAA AACAATGATT   
  
  
+ AAAGATTGTA ACTTTCTCAG CACCATCCAG ACTATTCTAA TAGACATCAC ACAAGTCATA ATATCAGAAT   
  
  
+ TTGTAGTGTT AGTTGTTTAC GCACACAAAT GATAGGCAGC CATCGACTGT TACAAAATTT ATAAAAGTCT   
  
  
+ CACACTAATA GATTATTAAA AGATTATTTC GTACTTGCGC GACTGATGGT AACAAAACTC GTAGATAGAA   
  
  
+ TATACTTTCT TTGAAGTGGG AATGATTTGA CCTACTCTAT AAATAATATA AACATCACAT GCATACACTC   
  
  
+ CAACCACTAA GCCACAGAAA GAAATGAAAG AAACAGAAAA TAATTAACAA CCCTATTGGA CTCTTCTCTA   
  
  
+ CACTGCCAGC CTTTCCTTCT TTGTTCTGAG CAAAGAAACA CACCAAAATT GTCAGCCATA AAATTTCTTC   
  
  
+ CTCACAGAAA CTCGTTTGGT TACTGAGAA  

- TGCTTTTGGC TTCGTTATTG TAAGATAAAT ATCCTCTCCT TTCAGCCGTG TCTCCTTCCA CTCTAATCTG   
  
  
- GTTCGTTTGA ATGTGGCGGA TTTTCGATAA CTATTCTTAC TACATTTTCG GGAAATTAAC CGATACCAAA   
  
  
- ACCAGGTTAA TCTTATCTTG TTTGTTGAAT TATTTTATTA CAGTTTTCCT TTCTTTTACT TATTATACTT   
  
  
- ATATATCAAG TTTATGATGA CATTACCACA TGACTTGAAT AAACCAAGTT TTATCATCAT TAATCTCATA   
  
  
- TCTTTTTACT TTATTTTCTT AATTGCTAAT TTATTCTTTA TTATGTTTTG CTATACTTTA ATTATTCATA   
  
  
- TATGTTGTGT ATTAATATAT ATTAATAAAA CCTTTTATAT CCAGGAGTGT TCTGCGGATT CACGATCGTG   
  
  
- GATGAACGAC GACCGATCGC GAATCCAGGA CGAAAAAGAA TATAATATGA GATTTAATAA CATTGAACCC   
  
  
- ATATCTTCAC GTCCGTTTGA TTGGATAGGG TATAAGTGGT CTAAGTGCAA ACGAAACTTT TCTTCAAATA   
  
  
- TCTACTTCTT TTACAATTTT TCTTTTTTCT CACTAATCTC TATTGAACGA ACCTACACCT AAAACGAGCC   
  
  
- CCATTTCTTC CCGCCCGTGT GACTGGATAG GGTCTATATG ATAAAAGTGT AAAAGAAACT TTTCTCCAAA   
  
  
- TGTATATTTC TTTTACACAT TTTTTTTTAT TTTTTATTTT ATTTTATTTC ACTAATTTCT ATTGAAACCT   
  
  
- ACTCCTAAAC CGAGCCCCAT ATCTTCTCAC CTATGAAACT GGATAGAGTT TAAGTGGTCT GAGTACAAAC   
  
  
- GAAACTTTTC TCCAAATATC TATTTCTTTT GCATTTTTTT TTTTTTTATT TATTTTTTTT TACTCACTGA   
  
  
- TTTCTATTGA ATGCACCTAC TCTTAAACTC AACCTCATAT TTTCCTGCCT GTGTAACTGG ATAGGGTCTA   
  
  
- AGTACTCTAA GTGCAAATGG TATCTCCAAA TATCTATTTC TCTTTCATTT TTTTTCTTTT TTGTTACTAA   
  
  
- TTTCTAACAT TGAAAGAGTC GTGGTAGGTC TGATAAGATT ATCTGTAGTG TGTTCAGTAT TATAGTCTTA   
  
  
- AACATCACAA TCAACAAATG CGTGTGTTTA CTATCCGTCG GTAGCTGACA ATGTTTTAAA TATTTTCAGA   
  
  
- GTGTGATTAT CTAATAATTT TCTAATAAAG CATGAACGCG CTGACTACCA TTGTTTTGAG CATCTATCTT   
  
  
- ATATGAAAGA AACTTCACCC TTACTAAACT GGATGAGATA TTTATTATAT TTGTAGTGTA CGTATGTGAG   
  
  
- GTTGGTGATT CGGTGTCTTT CTTTACTTTC TTTGTCTTTT ATTAATTGTT GGGATAACCT GAGAAGAGAT   
  
  
- GTGACGGTCG GAAAGGAAGA AACAAGACTC GTTTCTTTGT GTGGTTTTAA CAGTCGGTAT TTTAAAGAAG   
  
  
- GAGTGTCTTT GAGCAAACCA ATGACTCTT

  
  
Motifs Found  

+   

| Site Name | Organism | Position | Strand | Matrix score. | sequence | function |
| --- | --- | --- | --- | --- | --- | --- |
|  | organism | 1391 | + | 4 | motif\_sequence | short\_function |
|  | organism | 33 | - | 4 | motif\_sequence | short\_function |
|  | organism | 793 | - | 4 | motif\_sequence | short\_function |
|  | organism | 1251 | - | 4 | motif\_sequence | short\_function |
|  | organism | 1396 | + | 4 | motif\_sequence | short\_function |
|  | organism | 47 | - | 4 | motif\_sequence | short\_function |

>038667   
+ ACGAAAACCG AAGCAATAAC ATTCTATTTA TAGGAGAGGA AAGTCGGCAC AGAGGAAGGT GAGATTAGAC   
  
  
+ CAAGCAAACT TACACCGCCT AAAAGCTATT GATAAGAATG ATGTAAAAGC CCTTTAATTG GCTATGGTTT   
  
  
+ TGGTCCAATT AGAATAGAAC AAACAACTTA ATAAAATAAT GTCAAAAGGA AAGAAAATGA ATAATATGAA   
  
  
+ TATATAGTTC AAATACTACT GTAATGGTGT ACTGAACTTA TTTGGTTCAA AATAGTAGTA ATTAGAGTAT   
  
  
+ AGAAAAATGA AATAAAAGAA TTAACGATTA AATAAGAAAT AATACAAAAC GATATGAAAT TAATAAGTAT   
  
  
+ ATACAACACA TAATTATATA TAATTATTTT GGAAAATATA GGTCCTCACA AGACGCCTAA GTGCTAGCAC   
  
  
+ CTACTTGCTG CTGGCTAGCG CTTAGGTCCT GCTTTTTCTT ATATTATACT CTAAATTATT GTAACTTGGG   
  
  
+ TATAGAAGTG CAGGCAAACT AACCTATCCC ATATTCACCA GATTCACGTT TGCTTTGAAA AGAAGTTTAT   
  
  
+ AGATGAAGAA AATGTTAAAA AGAAAAAAGA GTGATTAGAG ATAACTTGCT TGGATGTGGA TTTTGCTCGG   
  
  
+ GGTAAAGAAG GGCGGGCACA CTGACCTATC CCAGATATAC TATTTTCACA TTTTCTTTGA AAAGAGGTTT   
  
  
+ ACATATAAAG AAAATGTGTA AAAAAAAATA AAAAATAAAA TAAAATAAAG TGATTAAAGA TAACTTTGGA   
  
  
+ TGAGGATTTG GCTCGGGGTA TAGAAGAGTG GATACTTTGA CCTATCTCAA ATTCACCAGA CTCATGTTTG   
  
  
+ CTTTGAAAAG AGGTTTATAG ATAAAGAAAA CGTAAAAAAA AAAAAAATAA ATAAAAAAAA ATGAGTGACT   
  
  
+ AAAGATAACT TACGTGGATG AGAATTTGAG TTGGAGTATA AAAGGACGGA CACATTGACC TATCCCAGAT   
  
  
+ TCATGAGATT CACGTTTACC ATAGAGGTTT ATAGATAAAG AGAAAGTAAA AAAAAGAAAA AACAATGATT   
  
  
+ AAAGATTGTA ACTTTCTCAG CACCATCCAG ACTATTCTAA TAGACATCAC ACAAGTCATA ATATCAGAAT   
  
  
+ TTGTAGTGTT AGTTGTTTAC GCACACAAAT GATAGGCAGC CATCGACTGT TACAAAATTT ATAAAAGTCT   
  
  
+ CACACTAATA GATTATTAAA AGATTATTTC GTACTTGCGC GACTGATGGT AACAAAACTC GTAGATAGAA   
  
  
+ TATACTTTCT TTGAAGTGGG AATGATTTGA CCTACTCTAT AAATAATATA AACATCACAT GCATACACTC   
  
  
+ CAACCACTAA GCCACAGAAA GAAATGAAAG AAACAGAAAA TAATTAACAA CCCTATTGGA CTCTTCTCTA   
  
  
+ CACTGCCAGC CTTTCCTTCT TTGTTCTGAG CAAAGAAACA CACCAAAATT GTCAGCCATA AAATTTCTTC   
  
  
+ CTCACAGAAA CTCGTTTGGT TACTGAGAA  

- TGCTTTTGGC TTCGTTATTG TAAGATAAAT ATCCTCTCCT TTCAGCCGTG TCTCCTTCCA CTCTAATCTG   
  
  
- GTTCGTTTGA ATGTGGCGGA TTTTCGATAA CTATTCTTAC TACATTTTCG GGAAATTAAC CGATACCAAA   
  
  
- ACCAGGTTAA TCTTATCTTG TTTGTTGAAT TATTTTATTA CAGTTTTCCT TTCTTTTACT TATTATACTT   
  
  
- ATATATCAAG TTTATGATGA CATTACCACA TGACTTGAAT AAACCAAGTT TTATCATCAT TAATCTCATA   
  
  
- TCTTTTTACT TTATTTTCTT AATTGCTAAT TTATTCTTTA TTATGTTTTG CTATACTTTA ATTATTCATA   
  
  
- TATGTTGTGT ATTAATATAT ATTAATAAAA CCTTTTATAT CCAGGAGTGT TCTGCGGATT CACGATCGTG   
  
  
- GATGAACGAC GACCGATCGC GAATCCAGGA CGAAAAAGAA TATAATATGA GATTTAATAA CATTGAACCC   
  
  
- ATATCTTCAC GTCCGTTTGA TTGGATAGGG TATAAGTGGT CTAAGTGCAA ACGAAACTTT TCTTCAAATA   
  
  
- TCTACTTCTT TTACAATTTT TCTTTTTTCT CACTAATCTC TATTGAACGA ACCTACACCT AAAACGAGCC   
  
  
- CCATTTCTTC CCGCCCGTGT GACTGGATAG GGTCTATATG ATAAAAGTGT AAAAGAAACT TTTCTCCAAA   
  
  
- TGTATATTTC TTTTACACAT TTTTTTTTAT TTTTTATTTT ATTTTATTTC ACTAATTTCT ATTGAAACCT   
  
  
- ACTCCTAAAC CGAGCCCCAT ATCTTCTCAC CTATGAAACT GGATAGAGTT TAAGTGGTCT GAGTACAAAC   
  
  
- GAAACTTTTC TCCAAATATC TATTTCTTTT GCATTTTTTT TTTTTTTATT TATTTTTTTT TACTCACTGA   
  
  
- TTTCTATTGA ATGCACCTAC TCTTAAACTC AACCTCATAT TTTCCTGCCT GTGTAACTGG ATAGGGTCTA   
  
  
- AGTACTCTAA GTGCAAATGG TATCTCCAAA TATCTATTTC TCTTTCATTT TTTTTCTTTT TTGTTACTAA   
  
  
- TTTCTAACAT TGAAAGAGTC GTGGTAGGTC TGATAAGATT ATCTGTAGTG TGTTCAGTAT TATAGTCTTA   
  
  
- AACATCACAA TCAACAAATG CGTGTGTTTA CTATCCGTCG GTAGCTGACA ATGTTTTAAA TATTTTCAGA   
  
  
- GTGTGATTAT CTAATAATTT TCTAATAAAG CATGAACGCG CTGACTACCA TTGTTTTGAG CATCTATCTT   
  
  
- ATATGAAAGA AACTTCACCC TTACTAAACT GGATGAGATA TTTATTATAT TTGTAGTGTA CGTATGTGAG   
  
  
- GTTGGTGATT CGGTGTCTTT CTTTACTTTC TTTGTCTTTT ATTAATTGTT GGGATAACCT GAGAAGAGAT   
  
  
- GTGACGGTCG GAAAGGAAGA AACAAGACTC GTTTCTTTGT GTGGTTTTAA CAGTCGGTAT TTTAAAGAAG   
  
  
- GAGTGTCTTT GAGCAAACCA ATGACTCTT

+     3-AF1 binding site

| Site Name | Organism | Position | Strand | Matrix score. | sequence | function |
| --- | --- | --- | --- | --- | --- | --- |
| 3-AF1 binding site | Solanum tuberosum | 31 | + | 10 | TAAGAGAGGAA | light responsive element |

>038667   
+ ACGAAAACCG AAGCAATAAC ATTCTATTTA TAGGAGAGGA AAGTCGGCAC AGAGGAAGGT GAGATTAGAC   
  
  
+ CAAGCAAACT TACACCGCCT AAAAGCTATT GATAAGAATG ATGTAAAAGC CCTTTAATTG GCTATGGTTT   
  
  
+ TGGTCCAATT AGAATAGAAC AAACAACTTA ATAAAATAAT GTCAAAAGGA AAGAAAATGA ATAATATGAA   
  
  
+ TATATAGTTC AAATACTACT GTAATGGTGT ACTGAACTTA TTTGGTTCAA AATAGTAGTA ATTAGAGTAT   
  
  
+ AGAAAAATGA AATAAAAGAA TTAACGATTA AATAAGAAAT AATACAAAAC GATATGAAAT TAATAAGTAT   
  
  
+ ATACAACACA TAATTATATA TAATTATTTT GGAAAATATA GGTCCTCACA AGACGCCTAA GTGCTAGCAC   
  
  
+ CTACTTGCTG CTGGCTAGCG CTTAGGTCCT GCTTTTTCTT ATATTATACT CTAAATTATT GTAACTTGGG   
  
  
+ TATAGAAGTG CAGGCAAACT AACCTATCCC ATATTCACCA GATTCACGTT TGCTTTGAAA AGAAGTTTAT   
  
  
+ AGATGAAGAA AATGTTAAAA AGAAAAAAGA GTGATTAGAG ATAACTTGCT TGGATGTGGA TTTTGCTCGG   
  
  
+ GGTAAAGAAG GGCGGGCACA CTGACCTATC CCAGATATAC TATTTTCACA TTTTCTTTGA AAAGAGGTTT   
  
  
+ ACATATAAAG AAAATGTGTA AAAAAAAATA AAAAATAAAA TAAAATAAAG TGATTAAAGA TAACTTTGGA   
  
  
+ TGAGGATTTG GCTCGGGGTA TAGAAGAGTG GATACTTTGA CCTATCTCAA ATTCACCAGA CTCATGTTTG   
  
  
+ CTTTGAAAAG AGGTTTATAG ATAAAGAAAA CGTAAAAAAA AAAAAAATAA ATAAAAAAAA ATGAGTGACT   
  
  
+ AAAGATAACT TACGTGGATG AGAATTTGAG TTGGAGTATA AAAGGACGGA CACATTGACC TATCCCAGAT   
  
  
+ TCATGAGATT CACGTTTACC ATAGAGGTTT ATAGATAAAG AGAAAGTAAA AAAAAGAAAA AACAATGATT   
  
  
+ AAAGATTGTA ACTTTCTCAG CACCATCCAG ACTATTCTAA TAGACATCAC ACAAGTCATA ATATCAGAAT   
  
  
+ TTGTAGTGTT AGTTGTTTAC GCACACAAAT GATAGGCAGC CATCGACTGT TACAAAATTT ATAAAAGTCT   
  
  
+ CACACTAATA GATTATTAAA AGATTATTTC GTACTTGCGC GACTGATGGT AACAAAACTC GTAGATAGAA   
  
  
+ TATACTTTCT TTGAAGTGGG AATGATTTGA CCTACTCTAT AAATAATATA AACATCACAT GCATACACTC   
  
  
+ CAACCACTAA GCCACAGAAA GAAATGAAAG AAACAGAAAA TAATTAACAA CCCTATTGGA CTCTTCTCTA   
  
  
+ CACTGCCAGC CTTTCCTTCT TTGTTCTGAG CAAAGAAACA CACCAAAATT GTCAGCCATA AAATTTCTTC   
  
  
+ CTCACAGAAA CTCGTTTGGT TACTGAGAA  

- TGCTTTTGGC TTCGTTATTG TAAGATAAAT ATCCTCTCCT TTCAGCCGTG TCTCCTTCCA CTCTAATCTG   
  
  
- GTTCGTTTGA ATGTGGCGGA TTTTCGATAA CTATTCTTAC TACATTTTCG GGAAATTAAC CGATACCAAA   
  
  
- ACCAGGTTAA TCTTATCTTG TTTGTTGAAT TATTTTATTA CAGTTTTCCT TTCTTTTACT TATTATACTT   
  
  
- ATATATCAAG TTTATGATGA CATTACCACA TGACTTGAAT AAACCAAGTT TTATCATCAT TAATCTCATA   
  
  
- TCTTTTTACT TTATTTTCTT AATTGCTAAT TTATTCTTTA TTATGTTTTG CTATACTTTA ATTATTCATA   
  
  
- TATGTTGTGT ATTAATATAT ATTAATAAAA CCTTTTATAT CCAGGAGTGT TCTGCGGATT CACGATCGTG   
  
  
- GATGAACGAC GACCGATCGC GAATCCAGGA CGAAAAAGAA TATAATATGA GATTTAATAA CATTGAACCC   
  
  
- ATATCTTCAC GTCCGTTTGA TTGGATAGGG TATAAGTGGT CTAAGTGCAA ACGAAACTTT TCTTCAAATA   
  
  
- TCTACTTCTT TTACAATTTT TCTTTTTTCT CACTAATCTC TATTGAACGA ACCTACACCT AAAACGAGCC   
  
  
- CCATTTCTTC CCGCCCGTGT GACTGGATAG GGTCTATATG ATAAAAGTGT AAAAGAAACT TTTCTCCAAA   
  
  
- TGTATATTTC TTTTACACAT TTTTTTTTAT TTTTTATTTT ATTTTATTTC ACTAATTTCT ATTGAAACCT   
  
  
- ACTCCTAAAC CGAGCCCCAT ATCTTCTCAC CTATGAAACT GGATAGAGTT TAAGTGGTCT GAGTACAAAC   
  
  
- GAAACTTTTC TCCAAATATC TATTTCTTTT GCATTTTTTT TTTTTTTATT TATTTTTTTT TACTCACTGA   
  
  
- TTTCTATTGA ATGCACCTAC TCTTAAACTC AACCTCATAT TTTCCTGCCT GTGTAACTGG ATAGGGTCTA   
  
  
- AGTACTCTAA GTGCAAATGG TATCTCCAAA TATCTATTTC TCTTTCATTT TTTTTCTTTT TTGTTACTAA   
  
  
- TTTCTAACAT TGAAAGAGTC GTGGTAGGTC TGATAAGATT ATCTGTAGTG TGTTCAGTAT TATAGTCTTA   
  
  
- AACATCACAA TCAACAAATG CGTGTGTTTA CTATCCGTCG GTAGCTGACA ATGTTTTAAA TATTTTCAGA   
  
  
- GTGTGATTAT CTAATAATTT TCTAATAAAG CATGAACGCG CTGACTACCA TTGTTTTGAG CATCTATCTT   
  
  
- ATATGAAAGA AACTTCACCC TTACTAAACT GGATGAGATA TTTATTATAT TTGTAGTGTA CGTATGTGAG   
  
  
- GTTGGTGATT CGGTGTCTTT CTTTACTTTC TTTGTCTTTT ATTAATTGTT GGGATAACCT GAGAAGAGAT   
  
  
- GTGACGGTCG GAAAGGAAGA AACAAGACTC GTTTCTTTGT GTGGTTTTAA CAGTCGGTAT TTTAAAGAAG   
  
  
- GAGTGTCTTT GAGCAAACCA ATGACTCTT

+     A-box

| Site Name | Organism | Position | Strand | Matrix score. | sequence | function |
| --- | --- | --- | --- | --- | --- | --- |
| A-box | Petroselinum crispum | 954 | - | 6 | CCGTCC | cis-acting regulatory element |

>038667   
+ ACGAAAACCG AAGCAATAAC ATTCTATTTA TAGGAGAGGA AAGTCGGCAC AGAGGAAGGT GAGATTAGAC   
  
  
+ CAAGCAAACT TACACCGCCT AAAAGCTATT GATAAGAATG ATGTAAAAGC CCTTTAATTG GCTATGGTTT   
  
  
+ TGGTCCAATT AGAATAGAAC AAACAACTTA ATAAAATAAT GTCAAAAGGA AAGAAAATGA ATAATATGAA   
  
  
+ TATATAGTTC AAATACTACT GTAATGGTGT ACTGAACTTA TTTGGTTCAA AATAGTAGTA ATTAGAGTAT   
  
  
+ AGAAAAATGA AATAAAAGAA TTAACGATTA AATAAGAAAT AATACAAAAC GATATGAAAT TAATAAGTAT   
  
  
+ ATACAACACA TAATTATATA TAATTATTTT GGAAAATATA GGTCCTCACA AGACGCCTAA GTGCTAGCAC   
  
  
+ CTACTTGCTG CTGGCTAGCG CTTAGGTCCT GCTTTTTCTT ATATTATACT CTAAATTATT GTAACTTGGG   
  
  
+ TATAGAAGTG CAGGCAAACT AACCTATCCC ATATTCACCA GATTCACGTT TGCTTTGAAA AGAAGTTTAT   
  
  
+ AGATGAAGAA AATGTTAAAA AGAAAAAAGA GTGATTAGAG ATAACTTGCT TGGATGTGGA TTTTGCTCGG   
  
  
+ GGTAAAGAAG GGCGGGCACA CTGACCTATC CCAGATATAC TATTTTCACA TTTTCTTTGA AAAGAGGTTT   
  
  
+ ACATATAAAG AAAATGTGTA AAAAAAAATA AAAAATAAAA TAAAATAAAG TGATTAAAGA TAACTTTGGA   
  
  
+ TGAGGATTTG GCTCGGGGTA TAGAAGAGTG GATACTTTGA CCTATCTCAA ATTCACCAGA CTCATGTTTG   
  
  
+ CTTTGAAAAG AGGTTTATAG ATAAAGAAAA CGTAAAAAAA AAAAAAATAA ATAAAAAAAA ATGAGTGACT   
  
  
+ AAAGATAACT TACGTGGATG AGAATTTGAG TTGGAGTATA AAAGGACGGA CACATTGACC TATCCCAGAT   
  
  
+ TCATGAGATT CACGTTTACC ATAGAGGTTT ATAGATAAAG AGAAAGTAAA AAAAAGAAAA AACAATGATT   
  
  
+ AAAGATTGTA ACTTTCTCAG CACCATCCAG ACTATTCTAA TAGACATCAC ACAAGTCATA ATATCAGAAT   
  
  
+ TTGTAGTGTT AGTTGTTTAC GCACACAAAT GATAGGCAGC CATCGACTGT TACAAAATTT ATAAAAGTCT   
  
  
+ CACACTAATA GATTATTAAA AGATTATTTC GTACTTGCGC GACTGATGGT AACAAAACTC GTAGATAGAA   
  
  
+ TATACTTTCT TTGAAGTGGG AATGATTTGA CCTACTCTAT AAATAATATA AACATCACAT GCATACACTC   
  
  
+ CAACCACTAA GCCACAGAAA GAAATGAAAG AAACAGAAAA TAATTAACAA CCCTATTGGA CTCTTCTCTA   
  
  
+ CACTGCCAGC CTTTCCTTCT TTGTTCTGAG CAAAGAAACA CACCAAAATT GTCAGCCATA AAATTTCTTC   
  
  
+ CTCACAGAAA CTCGTTTGGT TACTGAGAA  

- TGCTTTTGGC TTCGTTATTG TAAGATAAAT ATCCTCTCCT TTCAGCCGTG TCTCCTTCCA CTCTAATCTG   
  
  
- GTTCGTTTGA ATGTGGCGGA TTTTCGATAA CTATTCTTAC TACATTTTCG GGAAATTAAC CGATACCAAA   
  
  
- ACCAGGTTAA TCTTATCTTG TTTGTTGAAT TATTTTATTA CAGTTTTCCT TTCTTTTACT TATTATACTT   
  
  
- ATATATCAAG TTTATGATGA CATTACCACA TGACTTGAAT AAACCAAGTT TTATCATCAT TAATCTCATA   
  
  
- TCTTTTTACT TTATTTTCTT AATTGCTAAT TTATTCTTTA TTATGTTTTG CTATACTTTA ATTATTCATA   
  
  
- TATGTTGTGT ATTAATATAT ATTAATAAAA CCTTTTATAT CCAGGAGTGT TCTGCGGATT CACGATCGTG   
  
  
- GATGAACGAC GACCGATCGC GAATCCAGGA CGAAAAAGAA TATAATATGA GATTTAATAA CATTGAACCC   
  
  
- ATATCTTCAC GTCCGTTTGA TTGGATAGGG TATAAGTGGT CTAAGTGCAA ACGAAACTTT TCTTCAAATA   
  
  
- TCTACTTCTT TTACAATTTT TCTTTTTTCT CACTAATCTC TATTGAACGA ACCTACACCT AAAACGAGCC   
  
  
- CCATTTCTTC CCGCCCGTGT GACTGGATAG GGTCTATATG ATAAAAGTGT AAAAGAAACT TTTCTCCAAA   
  
  
- TGTATATTTC TTTTACACAT TTTTTTTTAT TTTTTATTTT ATTTTATTTC ACTAATTTCT ATTGAAACCT   
  
  
- ACTCCTAAAC CGAGCCCCAT ATCTTCTCAC CTATGAAACT GGATAGAGTT TAAGTGGTCT GAGTACAAAC   
  
  
- GAAACTTTTC TCCAAATATC TATTTCTTTT GCATTTTTTT TTTTTTTATT TATTTTTTTT TACTCACTGA   
  
  
- TTTCTATTGA ATGCACCTAC TCTTAAACTC AACCTCATAT TTTCCTGCCT GTGTAACTGG ATAGGGTCTA   
  
  
- AGTACTCTAA GTGCAAATGG TATCTCCAAA TATCTATTTC TCTTTCATTT TTTTTCTTTT TTGTTACTAA   
  
  
- TTTCTAACAT TGAAAGAGTC GTGGTAGGTC TGATAAGATT ATCTGTAGTG TGTTCAGTAT TATAGTCTTA   
  
  
- AACATCACAA TCAACAAATG CGTGTGTTTA CTATCCGTCG GTAGCTGACA ATGTTTTAAA TATTTTCAGA   
  
  
- GTGTGATTAT CTAATAATTT TCTAATAAAG CATGAACGCG CTGACTACCA TTGTTTTGAG CATCTATCTT   
  
  
- ATATGAAAGA AACTTCACCC TTACTAAACT GGATGAGATA TTTATTATAT TTGTAGTGTA CGTATGTGAG   
  
  
- GTTGGTGATT CGGTGTCTTT CTTTACTTTC TTTGTCTTTT ATTAATTGTT GGGATAACCT GAGAAGAGAT   
  
  
- GTGACGGTCG GAAAGGAAGA AACAAGACTC GTTTCTTTGT GTGGTTTTAA CAGTCGGTAT TTTAAAGAAG   
  
  
- GAGTGTCTTT GAGCAAACCA ATGACTCTT

+     AAGAA-motif

| Site Name | Organism | Position | Strand | Matrix score. | sequence | function |
| --- | --- | --- | --- | --- | --- | --- |
| AAGAA-motif | Avena sativa | 1356 | + | 7 | GAAAGAA |  |
| AAGAA-motif | Avena sativa | 1347 | + | 7 | GAAAGAA |  |
| AAGAA-motif | Avena sativa | 860 | + | 9 | gGTAAAGAAA |  |
| AAGAA-motif | Avena sativa | 189 | + | 7 | GAAAGAA |  |
| AAGAA-motif | Avena sativa | 631 | + | 9 | gGTAAAGAAA |  |

>038667   
+ ACGAAAACCG AAGCAATAAC ATTCTATTTA TAGGAGAGGA AAGTCGGCAC AGAGGAAGGT GAGATTAGAC   
  
  
+ CAAGCAAACT TACACCGCCT AAAAGCTATT GATAAGAATG ATGTAAAAGC CCTTTAATTG GCTATGGTTT   
  
  
+ TGGTCCAATT AGAATAGAAC AAACAACTTA ATAAAATAAT GTCAAAAGGA AAGAAAATGA ATAATATGAA   
  
  
+ TATATAGTTC AAATACTACT GTAATGGTGT ACTGAACTTA TTTGGTTCAA AATAGTAGTA ATTAGAGTAT   
  
  
+ AGAAAAATGA AATAAAAGAA TTAACGATTA AATAAGAAAT AATACAAAAC GATATGAAAT TAATAAGTAT   
  
  
+ ATACAACACA TAATTATATA TAATTATTTT GGAAAATATA GGTCCTCACA AGACGCCTAA GTGCTAGCAC   
  
  
+ CTACTTGCTG CTGGCTAGCG CTTAGGTCCT GCTTTTTCTT ATATTATACT CTAAATTATT GTAACTTGGG   
  
  
+ TATAGAAGTG CAGGCAAACT AACCTATCCC ATATTCACCA GATTCACGTT TGCTTTGAAA AGAAGTTTAT   
  
  
+ AGATGAAGAA AATGTTAAAA AGAAAAAAGA GTGATTAGAG ATAACTTGCT TGGATGTGGA TTTTGCTCGG   
  
  
+ GGTAAAGAAG GGCGGGCACA CTGACCTATC CCAGATATAC TATTTTCACA TTTTCTTTGA AAAGAGGTTT   
  
  
+ ACATATAAAG AAAATGTGTA AAAAAAAATA AAAAATAAAA TAAAATAAAG TGATTAAAGA TAACTTTGGA   
  
  
+ TGAGGATTTG GCTCGGGGTA TAGAAGAGTG GATACTTTGA CCTATCTCAA ATTCACCAGA CTCATGTTTG   
  
  
+ CTTTGAAAAG AGGTTTATAG ATAAAGAAAA CGTAAAAAAA AAAAAAATAA ATAAAAAAAA ATGAGTGACT   
  
  
+ AAAGATAACT TACGTGGATG AGAATTTGAG TTGGAGTATA AAAGGACGGA CACATTGACC TATCCCAGAT   
  
  
+ TCATGAGATT CACGTTTACC ATAGAGGTTT ATAGATAAAG AGAAAGTAAA AAAAAGAAAA AACAATGATT   
  
  
+ AAAGATTGTA ACTTTCTCAG CACCATCCAG ACTATTCTAA TAGACATCAC ACAAGTCATA ATATCAGAAT   
  
  
+ TTGTAGTGTT AGTTGTTTAC GCACACAAAT GATAGGCAGC CATCGACTGT TACAAAATTT ATAAAAGTCT   
  
  
+ CACACTAATA GATTATTAAA AGATTATTTC GTACTTGCGC GACTGATGGT AACAAAACTC GTAGATAGAA   
  
  
+ TATACTTTCT TTGAAGTGGG AATGATTTGA CCTACTCTAT AAATAATATA AACATCACAT GCATACACTC   
  
  
+ CAACCACTAA GCCACAGAAA GAAATGAAAG AAACAGAAAA TAATTAACAA CCCTATTGGA CTCTTCTCTA   
  
  
+ CACTGCCAGC CTTTCCTTCT TTGTTCTGAG CAAAGAAACA CACCAAAATT GTCAGCCATA AAATTTCTTC   
  
  
+ CTCACAGAAA CTCGTTTGGT TACTGAGAA  

- TGCTTTTGGC TTCGTTATTG TAAGATAAAT ATCCTCTCCT TTCAGCCGTG TCTCCTTCCA CTCTAATCTG   
  
  
- GTTCGTTTGA ATGTGGCGGA TTTTCGATAA CTATTCTTAC TACATTTTCG GGAAATTAAC CGATACCAAA   
  
  
- ACCAGGTTAA TCTTATCTTG TTTGTTGAAT TATTTTATTA CAGTTTTCCT TTCTTTTACT TATTATACTT   
  
  
- ATATATCAAG TTTATGATGA CATTACCACA TGACTTGAAT AAACCAAGTT TTATCATCAT TAATCTCATA   
  
  
- TCTTTTTACT TTATTTTCTT AATTGCTAAT TTATTCTTTA TTATGTTTTG CTATACTTTA ATTATTCATA   
  
  
- TATGTTGTGT ATTAATATAT ATTAATAAAA CCTTTTATAT CCAGGAGTGT TCTGCGGATT CACGATCGTG   
  
  
- GATGAACGAC GACCGATCGC GAATCCAGGA CGAAAAAGAA TATAATATGA GATTTAATAA CATTGAACCC   
  
  
- ATATCTTCAC GTCCGTTTGA TTGGATAGGG TATAAGTGGT CTAAGTGCAA ACGAAACTTT TCTTCAAATA   
  
  
- TCTACTTCTT TTACAATTTT TCTTTTTTCT CACTAATCTC TATTGAACGA ACCTACACCT AAAACGAGCC   
  
  
- CCATTTCTTC CCGCCCGTGT GACTGGATAG GGTCTATATG ATAAAAGTGT AAAAGAAACT TTTCTCCAAA   
  
  
- TGTATATTTC TTTTACACAT TTTTTTTTAT TTTTTATTTT ATTTTATTTC ACTAATTTCT ATTGAAACCT   
  
  
- ACTCCTAAAC CGAGCCCCAT ATCTTCTCAC CTATGAAACT GGATAGAGTT TAAGTGGTCT GAGTACAAAC   
  
  
- GAAACTTTTC TCCAAATATC TATTTCTTTT GCATTTTTTT TTTTTTTATT TATTTTTTTT TACTCACTGA   
  
  
- TTTCTATTGA ATGCACCTAC TCTTAAACTC AACCTCATAT TTTCCTGCCT GTGTAACTGG ATAGGGTCTA   
  
  
- AGTACTCTAA GTGCAAATGG TATCTCCAAA TATCTATTTC TCTTTCATTT TTTTTCTTTT TTGTTACTAA   
  
  
- TTTCTAACAT TGAAAGAGTC GTGGTAGGTC TGATAAGATT ATCTGTAGTG TGTTCAGTAT TATAGTCTTA   
  
  
- AACATCACAA TCAACAAATG CGTGTGTTTA CTATCCGTCG GTAGCTGACA ATGTTTTAAA TATTTTCAGA   
  
  
- GTGTGATTAT CTAATAATTT TCTAATAAAG CATGAACGCG CTGACTACCA TTGTTTTGAG CATCTATCTT   
  
  
- ATATGAAAGA AACTTCACCC TTACTAAACT GGATGAGATA TTTATTATAT TTGTAGTGTA CGTATGTGAG   
  
  
- GTTGGTGATT CGGTGTCTTT CTTTACTTTC TTTGTCTTTT ATTAATTGTT GGGATAACCT GAGAAGAGAT   
  
  
- GTGACGGTCG GAAAGGAAGA AACAAGACTC GTTTCTTTGT GTGGTTTTAA CAGTCGGTAT TTTAAAGAAG   
  
  
- GAGTGTCTTT GAGCAAACCA ATGACTCTT

+     ABRE

| Site Name | Organism | Position | Strand | Matrix score. | sequence | function |
| --- | --- | --- | --- | --- | --- | --- |
| ABRE | Arabidopsis thaliana | 535 | - | 5 | ACGTG | cis-acting element involved in the abscisic acid responsiveness |
| ABRE | Arabidopsis thaliana | 922 | + | 5 | ACGTG | cis-acting element involved in the abscisic acid responsiveness |
| ABRE | Arabidopsis thaliana | 991 | - | 5 | ACGTG | cis-acting element involved in the abscisic acid responsiveness |

>038667   
+ ACGAAAACCG AAGCAATAAC ATTCTATTTA TAGGAGAGGA AAGTCGGCAC AGAGGAAGGT GAGATTAGAC   
  
  
+ CAAGCAAACT TACACCGCCT AAAAGCTATT GATAAGAATG ATGTAAAAGC CCTTTAATTG GCTATGGTTT   
  
  
+ TGGTCCAATT AGAATAGAAC AAACAACTTA ATAAAATAAT GTCAAAAGGA AAGAAAATGA ATAATATGAA   
  
  
+ TATATAGTTC AAATACTACT GTAATGGTGT ACTGAACTTA TTTGGTTCAA AATAGTAGTA ATTAGAGTAT   
  
  
+ AGAAAAATGA AATAAAAGAA TTAACGATTA AATAAGAAAT AATACAAAAC GATATGAAAT TAATAAGTAT   
  
  
+ ATACAACACA TAATTATATA TAATTATTTT GGAAAATATA GGTCCTCACA AGACGCCTAA GTGCTAGCAC   
  
  
+ CTACTTGCTG CTGGCTAGCG CTTAGGTCCT GCTTTTTCTT ATATTATACT CTAAATTATT GTAACTTGGG   
  
  
+ TATAGAAGTG CAGGCAAACT AACCTATCCC ATATTCACCA GATTCACGTT TGCTTTGAAA AGAAGTTTAT   
  
  
+ AGATGAAGAA AATGTTAAAA AGAAAAAAGA GTGATTAGAG ATAACTTGCT TGGATGTGGA TTTTGCTCGG   
  
  
+ GGTAAAGAAG GGCGGGCACA CTGACCTATC CCAGATATAC TATTTTCACA TTTTCTTTGA AAAGAGGTTT   
  
  
+ ACATATAAAG AAAATGTGTA AAAAAAAATA AAAAATAAAA TAAAATAAAG TGATTAAAGA TAACTTTGGA   
  
  
+ TGAGGATTTG GCTCGGGGTA TAGAAGAGTG GATACTTTGA CCTATCTCAA ATTCACCAGA CTCATGTTTG   
  
  
+ CTTTGAAAAG AGGTTTATAG ATAAAGAAAA CGTAAAAAAA AAAAAAATAA ATAAAAAAAA ATGAGTGACT   
  
  
+ AAAGATAACT TACGTGGATG AGAATTTGAG TTGGAGTATA AAAGGACGGA CACATTGACC TATCCCAGAT   
  
  
+ TCATGAGATT CACGTTTACC ATAGAGGTTT ATAGATAAAG AGAAAGTAAA AAAAAGAAAA AACAATGATT   
  
  
+ AAAGATTGTA ACTTTCTCAG CACCATCCAG ACTATTCTAA TAGACATCAC ACAAGTCATA ATATCAGAAT   
  
  
+ TTGTAGTGTT AGTTGTTTAC GCACACAAAT GATAGGCAGC CATCGACTGT TACAAAATTT ATAAAAGTCT   
  
  
+ CACACTAATA GATTATTAAA AGATTATTTC GTACTTGCGC GACTGATGGT AACAAAACTC GTAGATAGAA   
  
  
+ TATACTTTCT TTGAAGTGGG AATGATTTGA CCTACTCTAT AAATAATATA AACATCACAT GCATACACTC   
  
  
+ CAACCACTAA GCCACAGAAA GAAATGAAAG AAACAGAAAA TAATTAACAA CCCTATTGGA CTCTTCTCTA   
  
  
+ CACTGCCAGC CTTTCCTTCT TTGTTCTGAG CAAAGAAACA CACCAAAATT GTCAGCCATA AAATTTCTTC   
  
  
+ CTCACAGAAA CTCGTTTGGT TACTGAGAA  

- TGCTTTTGGC TTCGTTATTG TAAGATAAAT ATCCTCTCCT TTCAGCCGTG TCTCCTTCCA CTCTAATCTG   
  
  
- GTTCGTTTGA ATGTGGCGGA TTTTCGATAA CTATTCTTAC TACATTTTCG GGAAATTAAC CGATACCAAA   
  
  
- ACCAGGTTAA TCTTATCTTG TTTGTTGAAT TATTTTATTA CAGTTTTCCT TTCTTTTACT TATTATACTT   
  
  
- ATATATCAAG TTTATGATGA CATTACCACA TGACTTGAAT AAACCAAGTT TTATCATCAT TAATCTCATA   
  
  
- TCTTTTTACT TTATTTTCTT AATTGCTAAT TTATTCTTTA TTATGTTTTG CTATACTTTA ATTATTCATA   
  
  
- TATGTTGTGT ATTAATATAT ATTAATAAAA CCTTTTATAT CCAGGAGTGT TCTGCGGATT CACGATCGTG   
  
  
- GATGAACGAC GACCGATCGC GAATCCAGGA CGAAAAAGAA TATAATATGA GATTTAATAA CATTGAACCC   
  
  
- ATATCTTCAC GTCCGTTTGA TTGGATAGGG TATAAGTGGT CTAAGTGCAA ACGAAACTTT TCTTCAAATA   
  
  
- TCTACTTCTT TTACAATTTT TCTTTTTTCT CACTAATCTC TATTGAACGA ACCTACACCT AAAACGAGCC   
  
  
- CCATTTCTTC CCGCCCGTGT GACTGGATAG GGTCTATATG ATAAAAGTGT AAAAGAAACT TTTCTCCAAA   
  
  
- TGTATATTTC TTTTACACAT TTTTTTTTAT TTTTTATTTT ATTTTATTTC ACTAATTTCT ATTGAAACCT   
  
  
- ACTCCTAAAC CGAGCCCCAT ATCTTCTCAC CTATGAAACT GGATAGAGTT TAAGTGGTCT GAGTACAAAC   
  
  
- GAAACTTTTC TCCAAATATC TATTTCTTTT GCATTTTTTT TTTTTTTATT TATTTTTTTT TACTCACTGA   
  
  
- TTTCTATTGA ATGCACCTAC TCTTAAACTC AACCTCATAT TTTCCTGCCT GTGTAACTGG ATAGGGTCTA   
  
  
- AGTACTCTAA GTGCAAATGG TATCTCCAAA TATCTATTTC TCTTTCATTT TTTTTCTTTT TTGTTACTAA   
  
  
- TTTCTAACAT TGAAAGAGTC GTGGTAGGTC TGATAAGATT ATCTGTAGTG TGTTCAGTAT TATAGTCTTA   
  
  
- AACATCACAA TCAACAAATG CGTGTGTTTA CTATCCGTCG GTAGCTGACA ATGTTTTAAA TATTTTCAGA   
  
  
- GTGTGATTAT CTAATAATTT TCTAATAAAG CATGAACGCG CTGACTACCA TTGTTTTGAG CATCTATCTT   
  
  
- ATATGAAAGA AACTTCACCC TTACTAAACT GGATGAGATA TTTATTATAT TTGTAGTGTA CGTATGTGAG   
  
  
- GTTGGTGATT CGGTGTCTTT CTTTACTTTC TTTGTCTTTT ATTAATTGTT GGGATAACCT GAGAAGAGAT   
  
  
- GTGACGGTCG GAAAGGAAGA AACAAGACTC GTTTCTTTGT GTGGTTTTAA CAGTCGGTAT TTTAAAGAAG   
  
  
- GAGTGTCTTT GAGCAAACCA ATGACTCTT

+     ABRE3a

| Site Name | Organism | Position | Strand | Matrix score. | sequence | function |
| --- | --- | --- | --- | --- | --- | --- |
| ABRE3a | Zea mays | 921 | + | 6 | TACGTG |  |

>038667   
+ ACGAAAACCG AAGCAATAAC ATTCTATTTA TAGGAGAGGA AAGTCGGCAC AGAGGAAGGT GAGATTAGAC   
  
  
+ CAAGCAAACT TACACCGCCT AAAAGCTATT GATAAGAATG ATGTAAAAGC CCTTTAATTG GCTATGGTTT   
  
  
+ TGGTCCAATT AGAATAGAAC AAACAACTTA ATAAAATAAT GTCAAAAGGA AAGAAAATGA ATAATATGAA   
  
  
+ TATATAGTTC AAATACTACT GTAATGGTGT ACTGAACTTA TTTGGTTCAA AATAGTAGTA ATTAGAGTAT   
  
  
+ AGAAAAATGA AATAAAAGAA TTAACGATTA AATAAGAAAT AATACAAAAC GATATGAAAT TAATAAGTAT   
  
  
+ ATACAACACA TAATTATATA TAATTATTTT GGAAAATATA GGTCCTCACA AGACGCCTAA GTGCTAGCAC   
  
  
+ CTACTTGCTG CTGGCTAGCG CTTAGGTCCT GCTTTTTCTT ATATTATACT CTAAATTATT GTAACTTGGG   
  
  
+ TATAGAAGTG CAGGCAAACT AACCTATCCC ATATTCACCA GATTCACGTT TGCTTTGAAA AGAAGTTTAT   
  
  
+ AGATGAAGAA AATGTTAAAA AGAAAAAAGA GTGATTAGAG ATAACTTGCT TGGATGTGGA TTTTGCTCGG   
  
  
+ GGTAAAGAAG GGCGGGCACA CTGACCTATC CCAGATATAC TATTTTCACA TTTTCTTTGA AAAGAGGTTT   
  
  
+ ACATATAAAG AAAATGTGTA AAAAAAAATA AAAAATAAAA TAAAATAAAG TGATTAAAGA TAACTTTGGA   
  
  
+ TGAGGATTTG GCTCGGGGTA TAGAAGAGTG GATACTTTGA CCTATCTCAA ATTCACCAGA CTCATGTTTG   
  
  
+ CTTTGAAAAG AGGTTTATAG ATAAAGAAAA CGTAAAAAAA AAAAAAATAA ATAAAAAAAA ATGAGTGACT   
  
  
+ AAAGATAACT TACGTGGATG AGAATTTGAG TTGGAGTATA AAAGGACGGA CACATTGACC TATCCCAGAT   
  
  
+ TCATGAGATT CACGTTTACC ATAGAGGTTT ATAGATAAAG AGAAAGTAAA AAAAAGAAAA AACAATGATT   
  
  
+ AAAGATTGTA ACTTTCTCAG CACCATCCAG ACTATTCTAA TAGACATCAC ACAAGTCATA ATATCAGAAT   
  
  
+ TTGTAGTGTT AGTTGTTTAC GCACACAAAT GATAGGCAGC CATCGACTGT TACAAAATTT ATAAAAGTCT   
  
  
+ CACACTAATA GATTATTAAA AGATTATTTC GTACTTGCGC GACTGATGGT AACAAAACTC GTAGATAGAA   
  
  
+ TATACTTTCT TTGAAGTGGG AATGATTTGA CCTACTCTAT AAATAATATA AACATCACAT GCATACACTC   
  
  
+ CAACCACTAA GCCACAGAAA GAAATGAAAG AAACAGAAAA TAATTAACAA CCCTATTGGA CTCTTCTCTA   
  
  
+ CACTGCCAGC CTTTCCTTCT TTGTTCTGAG CAAAGAAACA CACCAAAATT GTCAGCCATA AAATTTCTTC   
  
  
+ CTCACAGAAA CTCGTTTGGT TACTGAGAA  

- TGCTTTTGGC TTCGTTATTG TAAGATAAAT ATCCTCTCCT TTCAGCCGTG TCTCCTTCCA CTCTAATCTG   
  
  
- GTTCGTTTGA ATGTGGCGGA TTTTCGATAA CTATTCTTAC TACATTTTCG GGAAATTAAC CGATACCAAA   
  
  
- ACCAGGTTAA TCTTATCTTG TTTGTTGAAT TATTTTATTA CAGTTTTCCT TTCTTTTACT TATTATACTT   
  
  
- ATATATCAAG TTTATGATGA CATTACCACA TGACTTGAAT AAACCAAGTT TTATCATCAT TAATCTCATA   
  
  
- TCTTTTTACT TTATTTTCTT AATTGCTAAT TTATTCTTTA TTATGTTTTG CTATACTTTA ATTATTCATA   
  
  
- TATGTTGTGT ATTAATATAT ATTAATAAAA CCTTTTATAT CCAGGAGTGT TCTGCGGATT CACGATCGTG   
  
  
- GATGAACGAC GACCGATCGC GAATCCAGGA CGAAAAAGAA TATAATATGA GATTTAATAA CATTGAACCC   
  
  
- ATATCTTCAC GTCCGTTTGA TTGGATAGGG TATAAGTGGT CTAAGTGCAA ACGAAACTTT TCTTCAAATA   
  
  
- TCTACTTCTT TTACAATTTT TCTTTTTTCT CACTAATCTC TATTGAACGA ACCTACACCT AAAACGAGCC   
  
  
- CCATTTCTTC CCGCCCGTGT GACTGGATAG GGTCTATATG ATAAAAGTGT AAAAGAAACT TTTCTCCAAA   
  
  
- TGTATATTTC TTTTACACAT TTTTTTTTAT TTTTTATTTT ATTTTATTTC ACTAATTTCT ATTGAAACCT   
  
  
- ACTCCTAAAC CGAGCCCCAT ATCTTCTCAC CTATGAAACT GGATAGAGTT TAAGTGGTCT GAGTACAAAC   
  
  
- GAAACTTTTC TCCAAATATC TATTTCTTTT GCATTTTTTT TTTTTTTATT TATTTTTTTT TACTCACTGA   
  
  
- TTTCTATTGA ATGCACCTAC TCTTAAACTC AACCTCATAT TTTCCTGCCT GTGTAACTGG ATAGGGTCTA   
  
  
- AGTACTCTAA GTGCAAATGG TATCTCCAAA TATCTATTTC TCTTTCATTT TTTTTCTTTT TTGTTACTAA   
  
  
- TTTCTAACAT TGAAAGAGTC GTGGTAGGTC TGATAAGATT ATCTGTAGTG TGTTCAGTAT TATAGTCTTA   
  
  
- AACATCACAA TCAACAAATG CGTGTGTTTA CTATCCGTCG GTAGCTGACA ATGTTTTAAA TATTTTCAGA   
  
  
- GTGTGATTAT CTAATAATTT TCTAATAAAG CATGAACGCG CTGACTACCA TTGTTTTGAG CATCTATCTT   
  
  
- ATATGAAAGA AACTTCACCC TTACTAAACT GGATGAGATA TTTATTATAT TTGTAGTGTA CGTATGTGAG   
  
  
- GTTGGTGATT CGGTGTCTTT CTTTACTTTC TTTGTCTTTT ATTAATTGTT GGGATAACCT GAGAAGAGAT   
  
  
- GTGACGGTCG GAAAGGAAGA AACAAGACTC GTTTCTTTGT GTGGTTTTAA CAGTCGGTAT TTTAAAGAAG   
  
  
- GAGTGTCTTT GAGCAAACCA ATGACTCTT

+     ABRE4

| Site Name | Organism | Position | Strand | Matrix score. | sequence | function |
| --- | --- | --- | --- | --- | --- | --- |
| ABRE4 | Zea mays | 921 | - | 6 | CACGTA |  |

>038667   
+ ACGAAAACCG AAGCAATAAC ATTCTATTTA TAGGAGAGGA AAGTCGGCAC AGAGGAAGGT GAGATTAGAC   
  
  
+ CAAGCAAACT TACACCGCCT AAAAGCTATT GATAAGAATG ATGTAAAAGC CCTTTAATTG GCTATGGTTT   
  
  
+ TGGTCCAATT AGAATAGAAC AAACAACTTA ATAAAATAAT GTCAAAAGGA AAGAAAATGA ATAATATGAA   
  
  
+ TATATAGTTC AAATACTACT GTAATGGTGT ACTGAACTTA TTTGGTTCAA AATAGTAGTA ATTAGAGTAT   
  
  
+ AGAAAAATGA AATAAAAGAA TTAACGATTA AATAAGAAAT AATACAAAAC GATATGAAAT TAATAAGTAT   
  
  
+ ATACAACACA TAATTATATA TAATTATTTT GGAAAATATA GGTCCTCACA AGACGCCTAA GTGCTAGCAC   
  
  
+ CTACTTGCTG CTGGCTAGCG CTTAGGTCCT GCTTTTTCTT ATATTATACT CTAAATTATT GTAACTTGGG   
  
  
+ TATAGAAGTG CAGGCAAACT AACCTATCCC ATATTCACCA GATTCACGTT TGCTTTGAAA AGAAGTTTAT   
  
  
+ AGATGAAGAA AATGTTAAAA AGAAAAAAGA GTGATTAGAG ATAACTTGCT TGGATGTGGA TTTTGCTCGG   
  
  
+ GGTAAAGAAG GGCGGGCACA CTGACCTATC CCAGATATAC TATTTTCACA TTTTCTTTGA AAAGAGGTTT   
  
  
+ ACATATAAAG AAAATGTGTA AAAAAAAATA AAAAATAAAA TAAAATAAAG TGATTAAAGA TAACTTTGGA   
  
  
+ TGAGGATTTG GCTCGGGGTA TAGAAGAGTG GATACTTTGA CCTATCTCAA ATTCACCAGA CTCATGTTTG   
  
  
+ CTTTGAAAAG AGGTTTATAG ATAAAGAAAA CGTAAAAAAA AAAAAAATAA ATAAAAAAAA ATGAGTGACT   
  
  
+ AAAGATAACT TACGTGGATG AGAATTTGAG TTGGAGTATA AAAGGACGGA CACATTGACC TATCCCAGAT   
  
  
+ TCATGAGATT CACGTTTACC ATAGAGGTTT ATAGATAAAG AGAAAGTAAA AAAAAGAAAA AACAATGATT   
  
  
+ AAAGATTGTA ACTTTCTCAG CACCATCCAG ACTATTCTAA TAGACATCAC ACAAGTCATA ATATCAGAAT   
  
  
+ TTGTAGTGTT AGTTGTTTAC GCACACAAAT GATAGGCAGC CATCGACTGT TACAAAATTT ATAAAAGTCT   
  
  
+ CACACTAATA GATTATTAAA AGATTATTTC GTACTTGCGC GACTGATGGT AACAAAACTC GTAGATAGAA   
  
  
+ TATACTTTCT TTGAAGTGGG AATGATTTGA CCTACTCTAT AAATAATATA AACATCACAT GCATACACTC   
  
  
+ CAACCACTAA GCCACAGAAA GAAATGAAAG AAACAGAAAA TAATTAACAA CCCTATTGGA CTCTTCTCTA   
  
  
+ CACTGCCAGC CTTTCCTTCT TTGTTCTGAG CAAAGAAACA CACCAAAATT GTCAGCCATA AAATTTCTTC   
  
  
+ CTCACAGAAA CTCGTTTGGT TACTGAGAA  

- TGCTTTTGGC TTCGTTATTG TAAGATAAAT ATCCTCTCCT TTCAGCCGTG TCTCCTTCCA CTCTAATCTG   
  
  
- GTTCGTTTGA ATGTGGCGGA TTTTCGATAA CTATTCTTAC TACATTTTCG GGAAATTAAC CGATACCAAA   
  
  
- ACCAGGTTAA TCTTATCTTG TTTGTTGAAT TATTTTATTA CAGTTTTCCT TTCTTTTACT TATTATACTT   
  
  
- ATATATCAAG TTTATGATGA CATTACCACA TGACTTGAAT AAACCAAGTT TTATCATCAT TAATCTCATA   
  
  
- TCTTTTTACT TTATTTTCTT AATTGCTAAT TTATTCTTTA TTATGTTTTG CTATACTTTA ATTATTCATA   
  
  
- TATGTTGTGT ATTAATATAT ATTAATAAAA CCTTTTATAT CCAGGAGTGT TCTGCGGATT CACGATCGTG   
  
  
- GATGAACGAC GACCGATCGC GAATCCAGGA CGAAAAAGAA TATAATATGA GATTTAATAA CATTGAACCC   
  
  
- ATATCTTCAC GTCCGTTTGA TTGGATAGGG TATAAGTGGT CTAAGTGCAA ACGAAACTTT TCTTCAAATA   
  
  
- TCTACTTCTT TTACAATTTT TCTTTTTTCT CACTAATCTC TATTGAACGA ACCTACACCT AAAACGAGCC   
  
  
- CCATTTCTTC CCGCCCGTGT GACTGGATAG GGTCTATATG ATAAAAGTGT AAAAGAAACT TTTCTCCAAA   
  
  
- TGTATATTTC TTTTACACAT TTTTTTTTAT TTTTTATTTT ATTTTATTTC ACTAATTTCT ATTGAAACCT   
  
  
- ACTCCTAAAC CGAGCCCCAT ATCTTCTCAC CTATGAAACT GGATAGAGTT TAAGTGGTCT GAGTACAAAC   
  
  
- GAAACTTTTC TCCAAATATC TATTTCTTTT GCATTTTTTT TTTTTTTATT TATTTTTTTT TACTCACTGA   
  
  
- TTTCTATTGA ATGCACCTAC TCTTAAACTC AACCTCATAT TTTCCTGCCT GTGTAACTGG ATAGGGTCTA   
  
  
- AGTACTCTAA GTGCAAATGG TATCTCCAAA TATCTATTTC TCTTTCATTT TTTTTCTTTT TTGTTACTAA   
  
  
- TTTCTAACAT TGAAAGAGTC GTGGTAGGTC TGATAAGATT ATCTGTAGTG TGTTCAGTAT TATAGTCTTA   
  
  
- AACATCACAA TCAACAAATG CGTGTGTTTA CTATCCGTCG GTAGCTGACA ATGTTTTAAA TATTTTCAGA   
  
  
- GTGTGATTAT CTAATAATTT TCTAATAAAG CATGAACGCG CTGACTACCA TTGTTTTGAG CATCTATCTT   
  
  
- ATATGAAAGA AACTTCACCC TTACTAAACT GGATGAGATA TTTATTATAT TTGTAGTGTA CGTATGTGAG   
  
  
- GTTGGTGATT CGGTGTCTTT CTTTACTTTC TTTGTCTTTT ATTAATTGTT GGGATAACCT GAGAAGAGAT   
  
  
- GTGACGGTCG GAAAGGAAGA AACAAGACTC GTTTCTTTGT GTGGTTTTAA CAGTCGGTAT TTTAAAGAAG   
  
  
- GAGTGTCTTT GAGCAAACCA ATGACTCTT

+     ARE

| Site Name | Organism | Position | Strand | Matrix score. | sequence | function |
| --- | --- | --- | --- | --- | --- | --- |
| ARE | Zea mays | 135 | - | 6 | AAACCA | cis-acting regulatory element essential for the anaerobic induction |

>038667   
+ ACGAAAACCG AAGCAATAAC ATTCTATTTA TAGGAGAGGA AAGTCGGCAC AGAGGAAGGT GAGATTAGAC   
  
  
+ CAAGCAAACT TACACCGCCT AAAAGCTATT GATAAGAATG ATGTAAAAGC CCTTTAATTG GCTATGGTTT   
  
  
+ TGGTCCAATT AGAATAGAAC AAACAACTTA ATAAAATAAT GTCAAAAGGA AAGAAAATGA ATAATATGAA   
  
  
+ TATATAGTTC AAATACTACT GTAATGGTGT ACTGAACTTA TTTGGTTCAA AATAGTAGTA ATTAGAGTAT   
  
  
+ AGAAAAATGA AATAAAAGAA TTAACGATTA AATAAGAAAT AATACAAAAC GATATGAAAT TAATAAGTAT   
  
  
+ ATACAACACA TAATTATATA TAATTATTTT GGAAAATATA GGTCCTCACA AGACGCCTAA GTGCTAGCAC   
  
  
+ CTACTTGCTG CTGGCTAGCG CTTAGGTCCT GCTTTTTCTT ATATTATACT CTAAATTATT GTAACTTGGG   
  
  
+ TATAGAAGTG CAGGCAAACT AACCTATCCC ATATTCACCA GATTCACGTT TGCTTTGAAA AGAAGTTTAT   
  
  
+ AGATGAAGAA AATGTTAAAA AGAAAAAAGA GTGATTAGAG ATAACTTGCT TGGATGTGGA TTTTGCTCGG   
  
  
+ GGTAAAGAAG GGCGGGCACA CTGACCTATC CCAGATATAC TATTTTCACA TTTTCTTTGA AAAGAGGTTT   
  
  
+ ACATATAAAG AAAATGTGTA AAAAAAAATA AAAAATAAAA TAAAATAAAG TGATTAAAGA TAACTTTGGA   
  
  
+ TGAGGATTTG GCTCGGGGTA TAGAAGAGTG GATACTTTGA CCTATCTCAA ATTCACCAGA CTCATGTTTG   
  
  
+ CTTTGAAAAG AGGTTTATAG ATAAAGAAAA CGTAAAAAAA AAAAAAATAA ATAAAAAAAA ATGAGTGACT   
  
  
+ AAAGATAACT TACGTGGATG AGAATTTGAG TTGGAGTATA AAAGGACGGA CACATTGACC TATCCCAGAT   
  
  
+ TCATGAGATT CACGTTTACC ATAGAGGTTT ATAGATAAAG AGAAAGTAAA AAAAAGAAAA AACAATGATT   
  
  
+ AAAGATTGTA ACTTTCTCAG CACCATCCAG ACTATTCTAA TAGACATCAC ACAAGTCATA ATATCAGAAT   
  
  
+ TTGTAGTGTT AGTTGTTTAC GCACACAAAT GATAGGCAGC CATCGACTGT TACAAAATTT ATAAAAGTCT   
  
  
+ CACACTAATA GATTATTAAA AGATTATTTC GTACTTGCGC GACTGATGGT AACAAAACTC GTAGATAGAA   
  
  
+ TATACTTTCT TTGAAGTGGG AATGATTTGA CCTACTCTAT AAATAATATA AACATCACAT GCATACACTC   
  
  
+ CAACCACTAA GCCACAGAAA GAAATGAAAG AAACAGAAAA TAATTAACAA CCCTATTGGA CTCTTCTCTA   
  
  
+ CACTGCCAGC CTTTCCTTCT TTGTTCTGAG CAAAGAAACA CACCAAAATT GTCAGCCATA AAATTTCTTC   
  
  
+ CTCACAGAAA CTCGTTTGGT TACTGAGAA  

- TGCTTTTGGC TTCGTTATTG TAAGATAAAT ATCCTCTCCT TTCAGCCGTG TCTCCTTCCA CTCTAATCTG   
  
  
- GTTCGTTTGA ATGTGGCGGA TTTTCGATAA CTATTCTTAC TACATTTTCG GGAAATTAAC CGATACCAAA   
  
  
- ACCAGGTTAA TCTTATCTTG TTTGTTGAAT TATTTTATTA CAGTTTTCCT TTCTTTTACT TATTATACTT   
  
  
- ATATATCAAG TTTATGATGA CATTACCACA TGACTTGAAT AAACCAAGTT TTATCATCAT TAATCTCATA   
  
  
- TCTTTTTACT TTATTTTCTT AATTGCTAAT TTATTCTTTA TTATGTTTTG CTATACTTTA ATTATTCATA   
  
  
- TATGTTGTGT ATTAATATAT ATTAATAAAA CCTTTTATAT CCAGGAGTGT TCTGCGGATT CACGATCGTG   
  
  
- GATGAACGAC GACCGATCGC GAATCCAGGA CGAAAAAGAA TATAATATGA GATTTAATAA CATTGAACCC   
  
  
- ATATCTTCAC GTCCGTTTGA TTGGATAGGG TATAAGTGGT CTAAGTGCAA ACGAAACTTT TCTTCAAATA   
  
  
- TCTACTTCTT TTACAATTTT TCTTTTTTCT CACTAATCTC TATTGAACGA ACCTACACCT AAAACGAGCC   
  
  
- CCATTTCTTC CCGCCCGTGT GACTGGATAG GGTCTATATG ATAAAAGTGT AAAAGAAACT TTTCTCCAAA   
  
  
- TGTATATTTC TTTTACACAT TTTTTTTTAT TTTTTATTTT ATTTTATTTC ACTAATTTCT ATTGAAACCT   
  
  
- ACTCCTAAAC CGAGCCCCAT ATCTTCTCAC CTATGAAACT GGATAGAGTT TAAGTGGTCT GAGTACAAAC   
  
  
- GAAACTTTTC TCCAAATATC TATTTCTTTT GCATTTTTTT TTTTTTTATT TATTTTTTTT TACTCACTGA   
  
  
- TTTCTATTGA ATGCACCTAC TCTTAAACTC AACCTCATAT TTTCCTGCCT GTGTAACTGG ATAGGGTCTA   
  
  
- AGTACTCTAA GTGCAAATGG TATCTCCAAA TATCTATTTC TCTTTCATTT TTTTTCTTTT TTGTTACTAA   
  
  
- TTTCTAACAT TGAAAGAGTC GTGGTAGGTC TGATAAGATT ATCTGTAGTG TGTTCAGTAT TATAGTCTTA   
  
  
- AACATCACAA TCAACAAATG CGTGTGTTTA CTATCCGTCG GTAGCTGACA ATGTTTTAAA TATTTTCAGA   
  
  
- GTGTGATTAT CTAATAATTT TCTAATAAAG CATGAACGCG CTGACTACCA TTGTTTTGAG CATCTATCTT   
  
  
- ATATGAAAGA AACTTCACCC TTACTAAACT GGATGAGATA TTTATTATAT TTGTAGTGTA CGTATGTGAG   
  
  
- GTTGGTGATT CGGTGTCTTT CTTTACTTTC TTTGTCTTTT ATTAATTGTT GGGATAACCT GAGAAGAGAT   
  
  
- GTGACGGTCG GAAAGGAAGA AACAAGACTC GTTTCTTTGT GTGGTTTTAA CAGTCGGTAT TTTAAAGAAG   
  
  
- GAGTGTCTTT GAGCAAACCA ATGACTCTT

+     AT~TATA-box

| Site Name | Organism | Position | Strand | Matrix score. | sequence | function |
| --- | --- | --- | --- | --- | --- | --- |
| AT~TATA-box | Arabidopsis thaliana | 211 | + | 6 | TATATA |  |
| AT~TATA-box | Arabidopsis thaliana | 348 | + | 6 | TATATA |  |
| AT~TATA-box | Arabidopsis thaliana | 365 | + | 6 | TATATA |  |
| AT~TATA-box | Arabidopsis thaliana | 367 | + | 6 | TATATA |  |

>038667   
+ ACGAAAACCG AAGCAATAAC ATTCTATTTA TAGGAGAGGA AAGTCGGCAC AGAGGAAGGT GAGATTAGAC   
  
  
+ CAAGCAAACT TACACCGCCT AAAAGCTATT GATAAGAATG ATGTAAAAGC CCTTTAATTG GCTATGGTTT   
  
  
+ TGGTCCAATT AGAATAGAAC AAACAACTTA ATAAAATAAT GTCAAAAGGA AAGAAAATGA ATAATATGAA   
  
  
+ TATATAGTTC AAATACTACT GTAATGGTGT ACTGAACTTA TTTGGTTCAA AATAGTAGTA ATTAGAGTAT   
  
  
+ AGAAAAATGA AATAAAAGAA TTAACGATTA AATAAGAAAT AATACAAAAC GATATGAAAT TAATAAGTAT   
  
  
+ ATACAACACA TAATTATATA TAATTATTTT GGAAAATATA GGTCCTCACA AGACGCCTAA GTGCTAGCAC   
  
  
+ CTACTTGCTG CTGGCTAGCG CTTAGGTCCT GCTTTTTCTT ATATTATACT CTAAATTATT GTAACTTGGG   
  
  
+ TATAGAAGTG CAGGCAAACT AACCTATCCC ATATTCACCA GATTCACGTT TGCTTTGAAA AGAAGTTTAT   
  
  
+ AGATGAAGAA AATGTTAAAA AGAAAAAAGA GTGATTAGAG ATAACTTGCT TGGATGTGGA TTTTGCTCGG   
  
  
+ GGTAAAGAAG GGCGGGCACA CTGACCTATC CCAGATATAC TATTTTCACA TTTTCTTTGA AAAGAGGTTT   
  
  
+ ACATATAAAG AAAATGTGTA AAAAAAAATA AAAAATAAAA TAAAATAAAG TGATTAAAGA TAACTTTGGA   
  
  
+ TGAGGATTTG GCTCGGGGTA TAGAAGAGTG GATACTTTGA CCTATCTCAA ATTCACCAGA CTCATGTTTG   
  
  
+ CTTTGAAAAG AGGTTTATAG ATAAAGAAAA CGTAAAAAAA AAAAAAATAA ATAAAAAAAA ATGAGTGACT   
  
  
+ AAAGATAACT TACGTGGATG AGAATTTGAG TTGGAGTATA AAAGGACGGA CACATTGACC TATCCCAGAT   
  
  
+ TCATGAGATT CACGTTTACC ATAGAGGTTT ATAGATAAAG AGAAAGTAAA AAAAAGAAAA AACAATGATT   
  
  
+ AAAGATTGTA ACTTTCTCAG CACCATCCAG ACTATTCTAA TAGACATCAC ACAAGTCATA ATATCAGAAT   
  
  
+ TTGTAGTGTT AGTTGTTTAC GCACACAAAT GATAGGCAGC CATCGACTGT TACAAAATTT ATAAAAGTCT   
  
  
+ CACACTAATA GATTATTAAA AGATTATTTC GTACTTGCGC GACTGATGGT AACAAAACTC GTAGATAGAA   
  
  
+ TATACTTTCT TTGAAGTGGG AATGATTTGA CCTACTCTAT AAATAATATA AACATCACAT GCATACACTC   
  
  
+ CAACCACTAA GCCACAGAAA GAAATGAAAG AAACAGAAAA TAATTAACAA CCCTATTGGA CTCTTCTCTA   
  
  
+ CACTGCCAGC CTTTCCTTCT TTGTTCTGAG CAAAGAAACA CACCAAAATT GTCAGCCATA AAATTTCTTC   
  
  
+ CTCACAGAAA CTCGTTTGGT TACTGAGAA  

- TGCTTTTGGC TTCGTTATTG TAAGATAAAT ATCCTCTCCT TTCAGCCGTG TCTCCTTCCA CTCTAATCTG   
  
  
- GTTCGTTTGA ATGTGGCGGA TTTTCGATAA CTATTCTTAC TACATTTTCG GGAAATTAAC CGATACCAAA   
  
  
- ACCAGGTTAA TCTTATCTTG TTTGTTGAAT TATTTTATTA CAGTTTTCCT TTCTTTTACT TATTATACTT   
  
  
- ATATATCAAG TTTATGATGA CATTACCACA TGACTTGAAT AAACCAAGTT TTATCATCAT TAATCTCATA   
  
  
- TCTTTTTACT TTATTTTCTT AATTGCTAAT TTATTCTTTA TTATGTTTTG CTATACTTTA ATTATTCATA   
  
  
- TATGTTGTGT ATTAATATAT ATTAATAAAA CCTTTTATAT CCAGGAGTGT TCTGCGGATT CACGATCGTG   
  
  
- GATGAACGAC GACCGATCGC GAATCCAGGA CGAAAAAGAA TATAATATGA GATTTAATAA CATTGAACCC   
  
  
- ATATCTTCAC GTCCGTTTGA TTGGATAGGG TATAAGTGGT CTAAGTGCAA ACGAAACTTT TCTTCAAATA   
  
  
- TCTACTTCTT TTACAATTTT TCTTTTTTCT CACTAATCTC TATTGAACGA ACCTACACCT AAAACGAGCC   
  
  
- CCATTTCTTC CCGCCCGTGT GACTGGATAG GGTCTATATG ATAAAAGTGT AAAAGAAACT TTTCTCCAAA   
  
  
- TGTATATTTC TTTTACACAT TTTTTTTTAT TTTTTATTTT ATTTTATTTC ACTAATTTCT ATTGAAACCT   
  
  
- ACTCCTAAAC CGAGCCCCAT ATCTTCTCAC CTATGAAACT GGATAGAGTT TAAGTGGTCT GAGTACAAAC   
  
  
- GAAACTTTTC TCCAAATATC TATTTCTTTT GCATTTTTTT TTTTTTTATT TATTTTTTTT TACTCACTGA   
  
  
- TTTCTATTGA ATGCACCTAC TCTTAAACTC AACCTCATAT TTTCCTGCCT GTGTAACTGG ATAGGGTCTA   
  
  
- AGTACTCTAA GTGCAAATGG TATCTCCAAA TATCTATTTC TCTTTCATTT TTTTTCTTTT TTGTTACTAA   
  
  
- TTTCTAACAT TGAAAGAGTC GTGGTAGGTC TGATAAGATT ATCTGTAGTG TGTTCAGTAT TATAGTCTTA   
  
  
- AACATCACAA TCAACAAATG CGTGTGTTTA CTATCCGTCG GTAGCTGACA ATGTTTTAAA TATTTTCAGA   
  
  
- GTGTGATTAT CTAATAATTT TCTAATAAAG CATGAACGCG CTGACTACCA TTGTTTTGAG CATCTATCTT   
  
  
- ATATGAAAGA AACTTCACCC TTACTAAACT GGATGAGATA TTTATTATAT TTGTAGTGTA CGTATGTGAG   
  
  
- GTTGGTGATT CGGTGTCTTT CTTTACTTTC TTTGTCTTTT ATTAATTGTT GGGATAACCT GAGAAGAGAT   
  
  
- GTGACGGTCG GAAAGGAAGA AACAAGACTC GTTTCTTTGT GTGGTTTTAA CAGTCGGTAT TTTAAAGAAG   
  
  
- GAGTGTCTTT GAGCAAACCA ATGACTCTT

+     Box 4

| Site Name | Organism | Position | Strand | Matrix score. | sequence | function |
| --- | --- | --- | --- | --- | --- | --- |
| Box 4 | Petroselinum crispum | 339 | + | 6 | ATTAAT | part of a conserved DNA module involved in light responsiveness |

>038667   
+ ACGAAAACCG AAGCAATAAC ATTCTATTTA TAGGAGAGGA AAGTCGGCAC AGAGGAAGGT GAGATTAGAC   
  
  
+ CAAGCAAACT TACACCGCCT AAAAGCTATT GATAAGAATG ATGTAAAAGC CCTTTAATTG GCTATGGTTT   
  
  
+ TGGTCCAATT AGAATAGAAC AAACAACTTA ATAAAATAAT GTCAAAAGGA AAGAAAATGA ATAATATGAA   
  
  
+ TATATAGTTC AAATACTACT GTAATGGTGT ACTGAACTTA TTTGGTTCAA AATAGTAGTA ATTAGAGTAT   
  
  
+ AGAAAAATGA AATAAAAGAA TTAACGATTA AATAAGAAAT AATACAAAAC GATATGAAAT TAATAAGTAT   
  
  
+ ATACAACACA TAATTATATA TAATTATTTT GGAAAATATA GGTCCTCACA AGACGCCTAA GTGCTAGCAC   
  
  
+ CTACTTGCTG CTGGCTAGCG CTTAGGTCCT GCTTTTTCTT ATATTATACT CTAAATTATT GTAACTTGGG   
  
  
+ TATAGAAGTG CAGGCAAACT AACCTATCCC ATATTCACCA GATTCACGTT TGCTTTGAAA AGAAGTTTAT   
  
  
+ AGATGAAGAA AATGTTAAAA AGAAAAAAGA GTGATTAGAG ATAACTTGCT TGGATGTGGA TTTTGCTCGG   
  
  
+ GGTAAAGAAG GGCGGGCACA CTGACCTATC CCAGATATAC TATTTTCACA TTTTCTTTGA AAAGAGGTTT   
  
  
+ ACATATAAAG AAAATGTGTA AAAAAAAATA AAAAATAAAA TAAAATAAAG TGATTAAAGA TAACTTTGGA   
  
  
+ TGAGGATTTG GCTCGGGGTA TAGAAGAGTG GATACTTTGA CCTATCTCAA ATTCACCAGA CTCATGTTTG   
  
  
+ CTTTGAAAAG AGGTTTATAG ATAAAGAAAA CGTAAAAAAA AAAAAAATAA ATAAAAAAAA ATGAGTGACT   
  
  
+ AAAGATAACT TACGTGGATG AGAATTTGAG TTGGAGTATA AAAGGACGGA CACATTGACC TATCCCAGAT   
  
  
+ TCATGAGATT CACGTTTACC ATAGAGGTTT ATAGATAAAG AGAAAGTAAA AAAAAGAAAA AACAATGATT   
  
  
+ AAAGATTGTA ACTTTCTCAG CACCATCCAG ACTATTCTAA TAGACATCAC ACAAGTCATA ATATCAGAAT   
  
  
+ TTGTAGTGTT AGTTGTTTAC GCACACAAAT GATAGGCAGC CATCGACTGT TACAAAATTT ATAAAAGTCT   
  
  
+ CACACTAATA GATTATTAAA AGATTATTTC GTACTTGCGC GACTGATGGT AACAAAACTC GTAGATAGAA   
  
  
+ TATACTTTCT TTGAAGTGGG AATGATTTGA CCTACTCTAT AAATAATATA AACATCACAT GCATACACTC   
  
  
+ CAACCACTAA GCCACAGAAA GAAATGAAAG AAACAGAAAA TAATTAACAA CCCTATTGGA CTCTTCTCTA   
  
  
+ CACTGCCAGC CTTTCCTTCT TTGTTCTGAG CAAAGAAACA CACCAAAATT GTCAGCCATA AAATTTCTTC   
  
  
+ CTCACAGAAA CTCGTTTGGT TACTGAGAA  

- TGCTTTTGGC TTCGTTATTG TAAGATAAAT ATCCTCTCCT TTCAGCCGTG TCTCCTTCCA CTCTAATCTG   
  
  
- GTTCGTTTGA ATGTGGCGGA TTTTCGATAA CTATTCTTAC TACATTTTCG GGAAATTAAC CGATACCAAA   
  
  
- ACCAGGTTAA TCTTATCTTG TTTGTTGAAT TATTTTATTA CAGTTTTCCT TTCTTTTACT TATTATACTT   
  
  
- ATATATCAAG TTTATGATGA CATTACCACA TGACTTGAAT AAACCAAGTT TTATCATCAT TAATCTCATA   
  
  
- TCTTTTTACT TTATTTTCTT AATTGCTAAT TTATTCTTTA TTATGTTTTG CTATACTTTA ATTATTCATA   
  
  
- TATGTTGTGT ATTAATATAT ATTAATAAAA CCTTTTATAT CCAGGAGTGT TCTGCGGATT CACGATCGTG   
  
  
- GATGAACGAC GACCGATCGC GAATCCAGGA CGAAAAAGAA TATAATATGA GATTTAATAA CATTGAACCC   
  
  
- ATATCTTCAC GTCCGTTTGA TTGGATAGGG TATAAGTGGT CTAAGTGCAA ACGAAACTTT TCTTCAAATA   
  
  
- TCTACTTCTT TTACAATTTT TCTTTTTTCT CACTAATCTC TATTGAACGA ACCTACACCT AAAACGAGCC   
  
  
- CCATTTCTTC CCGCCCGTGT GACTGGATAG GGTCTATATG ATAAAAGTGT AAAAGAAACT TTTCTCCAAA   
  
  
- TGTATATTTC TTTTACACAT TTTTTTTTAT TTTTTATTTT ATTTTATTTC ACTAATTTCT ATTGAAACCT   
  
  
- ACTCCTAAAC CGAGCCCCAT ATCTTCTCAC CTATGAAACT GGATAGAGTT TAAGTGGTCT GAGTACAAAC   
  
  
- GAAACTTTTC TCCAAATATC TATTTCTTTT GCATTTTTTT TTTTTTTATT TATTTTTTTT TACTCACTGA   
  
  
- TTTCTATTGA ATGCACCTAC TCTTAAACTC AACCTCATAT TTTCCTGCCT GTGTAACTGG ATAGGGTCTA   
  
  
- AGTACTCTAA GTGCAAATGG TATCTCCAAA TATCTATTTC TCTTTCATTT TTTTTCTTTT TTGTTACTAA   
  
  
- TTTCTAACAT TGAAAGAGTC GTGGTAGGTC TGATAAGATT ATCTGTAGTG TGTTCAGTAT TATAGTCTTA   
  
  
- AACATCACAA TCAACAAATG CGTGTGTTTA CTATCCGTCG GTAGCTGACA ATGTTTTAAA TATTTTCAGA   
  
  
- GTGTGATTAT CTAATAATTT TCTAATAAAG CATGAACGCG CTGACTACCA TTGTTTTGAG CATCTATCTT   
  
  
- ATATGAAAGA AACTTCACCC TTACTAAACT GGATGAGATA TTTATTATAT TTGTAGTGTA CGTATGTGAG   
  
  
- GTTGGTGATT CGGTGTCTTT CTTTACTTTC TTTGTCTTTT ATTAATTGTT GGGATAACCT GAGAAGAGAT   
  
  
- GTGACGGTCG GAAAGGAAGA AACAAGACTC GTTTCTTTGT GTGGTTTTAA CAGTCGGTAT TTTAAAGAAG   
  
  
- GAGTGTCTTT GAGCAAACCA ATGACTCTT

+     CAAT-box

| Site Name | Organism | Position | Strand | Matrix score. | sequence | function |
| --- | --- | --- | --- | --- | --- | --- |
| CAAT-box | Nicotiana glutinosa | 478 | - | 4 | CAAT |  |
| CAAT-box | Pisum sativum | 220 | + | 5 | CAAAT | common cis-acting element in promoter and enhancer regions |
| CAAT-box | Pisum sativum | 818 | + | 5 | CAAAT | common cis-acting element in promoter and enhancer regions |
| CAAT-box | Pisum sativum | 776 | - | 5 | CAAAT | common cis-acting element in promoter and enhancer regions |
| CAAT-box | Nicotiana glutinosa | 1055 | - | 4 | CAAT |  |
| CAAT-box | Pisum sativum | 250 | - | 5 | CAAAT | common cis-acting element in promoter and enhancer regions |
| CAAT-box | Nicotiana glutinosa | 146 | + | 4 | CAAT |  |
| CAAT-box | Pisum sativum | 1285 | - | 5 | CAAAT | common cis-acting element in promoter and enhancer regions |
| CAAT-box | Arabidopsis thaliana | 127 | - | 5 | CCAAT | common cis-acting element in promoter and enhancer regions |
| CAAT-box | Nicotiana glutinosa | 14 | + | 4 | CAAT |  |
| CAAT-box | Nicotiana glutinosa | 1043 | + | 4 | CAAT |  |
| CAAT-box | Nicotiana glutinosa | 98 | - | 4 | CAAT |  |
| CAAT-box | Pisum sativum | 1119 | - | 5 | CAAAT | common cis-acting element in promoter and enhancer regions |
| CAAT-box | Nicotiana glutinosa | 1448 | - | 4 | CAAT |  |
| CAAT-box | Arabidopsis thaliana | 145 | + | 5 | CCAAT | common cis-acting element in promoter and enhancer regions |
| CAAT-box | Arabidopsis thaliana | 1385 | - | 5 | CCAAT | common cis-acting element in promoter and enhancer regions |
| CAAT-box | Pisum sativum | 934 | - | 5 | CAAAT | common cis-acting element in promoter and enhancer regions |
| CAAT-box | Pisum sativum | 1146 | + | 5 | CAAAT | common cis-acting element in promoter and enhancer regions |
| CAAT-box | Nicotiana glutinosa | 964 | - | 4 | CAAT |  |

>038667   
+ ACGAAAACCG AAGCAATAAC ATTCTATTTA TAGGAGAGGA AAGTCGGCAC AGAGGAAGGT GAGATTAGAC   
  
  
+ CAAGCAAACT TACACCGCCT AAAAGCTATT GATAAGAATG ATGTAAAAGC CCTTTAATTG GCTATGGTTT   
  
  
+ TGGTCCAATT AGAATAGAAC AAACAACTTA ATAAAATAAT GTCAAAAGGA AAGAAAATGA ATAATATGAA   
  
  
+ TATATAGTTC AAATACTACT GTAATGGTGT ACTGAACTTA TTTGGTTCAA AATAGTAGTA ATTAGAGTAT   
  
  
+ AGAAAAATGA AATAAAAGAA TTAACGATTA AATAAGAAAT AATACAAAAC GATATGAAAT TAATAAGTAT   
  
  
+ ATACAACACA TAATTATATA TAATTATTTT GGAAAATATA GGTCCTCACA AGACGCCTAA GTGCTAGCAC   
  
  
+ CTACTTGCTG CTGGCTAGCG CTTAGGTCCT GCTTTTTCTT ATATTATACT CTAAATTATT GTAACTTGGG   
  
  
+ TATAGAAGTG CAGGCAAACT AACCTATCCC ATATTCACCA GATTCACGTT TGCTTTGAAA AGAAGTTTAT   
  
  
+ AGATGAAGAA AATGTTAAAA AGAAAAAAGA GTGATTAGAG ATAACTTGCT TGGATGTGGA TTTTGCTCGG   
  
  
+ GGTAAAGAAG GGCGGGCACA CTGACCTATC CCAGATATAC TATTTTCACA TTTTCTTTGA AAAGAGGTTT   
  
  
+ ACATATAAAG AAAATGTGTA AAAAAAAATA AAAAATAAAA TAAAATAAAG TGATTAAAGA TAACTTTGGA   
  
  
+ TGAGGATTTG GCTCGGGGTA TAGAAGAGTG GATACTTTGA CCTATCTCAA ATTCACCAGA CTCATGTTTG   
  
  
+ CTTTGAAAAG AGGTTTATAG ATAAAGAAAA CGTAAAAAAA AAAAAAATAA ATAAAAAAAA ATGAGTGACT   
  
  
+ AAAGATAACT TACGTGGATG AGAATTTGAG TTGGAGTATA AAAGGACGGA CACATTGACC TATCCCAGAT   
  
  
+ TCATGAGATT CACGTTTACC ATAGAGGTTT ATAGATAAAG AGAAAGTAAA AAAAAGAAAA AACAATGATT   
  
  
+ AAAGATTGTA ACTTTCTCAG CACCATCCAG ACTATTCTAA TAGACATCAC ACAAGTCATA ATATCAGAAT   
  
  
+ TTGTAGTGTT AGTTGTTTAC GCACACAAAT GATAGGCAGC CATCGACTGT TACAAAATTT ATAAAAGTCT   
  
  
+ CACACTAATA GATTATTAAA AGATTATTTC GTACTTGCGC GACTGATGGT AACAAAACTC GTAGATAGAA   
  
  
+ TATACTTTCT TTGAAGTGGG AATGATTTGA CCTACTCTAT AAATAATATA AACATCACAT GCATACACTC   
  
  
+ CAACCACTAA GCCACAGAAA GAAATGAAAG AAACAGAAAA TAATTAACAA CCCTATTGGA CTCTTCTCTA   
  
  
+ CACTGCCAGC CTTTCCTTCT TTGTTCTGAG CAAAGAAACA CACCAAAATT GTCAGCCATA AAATTTCTTC   
  
  
+ CTCACAGAAA CTCGTTTGGT TACTGAGAA  

- TGCTTTTGGC TTCGTTATTG TAAGATAAAT ATCCTCTCCT TTCAGCCGTG TCTCCTTCCA CTCTAATCTG   
  
  
- GTTCGTTTGA ATGTGGCGGA TTTTCGATAA CTATTCTTAC TACATTTTCG GGAAATTAAC CGATACCAAA   
  
  
- ACCAGGTTAA TCTTATCTTG TTTGTTGAAT TATTTTATTA CAGTTTTCCT TTCTTTTACT TATTATACTT   
  
  
- ATATATCAAG TTTATGATGA CATTACCACA TGACTTGAAT AAACCAAGTT TTATCATCAT TAATCTCATA   
  
  
- TCTTTTTACT TTATTTTCTT AATTGCTAAT TTATTCTTTA TTATGTTTTG CTATACTTTA ATTATTCATA   
  
  
- TATGTTGTGT ATTAATATAT ATTAATAAAA CCTTTTATAT CCAGGAGTGT TCTGCGGATT CACGATCGTG   
  
  
- GATGAACGAC GACCGATCGC GAATCCAGGA CGAAAAAGAA TATAATATGA GATTTAATAA CATTGAACCC   
  
  
- ATATCTTCAC GTCCGTTTGA TTGGATAGGG TATAAGTGGT CTAAGTGCAA ACGAAACTTT TCTTCAAATA   
  
  
- TCTACTTCTT TTACAATTTT TCTTTTTTCT CACTAATCTC TATTGAACGA ACCTACACCT AAAACGAGCC   
  
  
- CCATTTCTTC CCGCCCGTGT GACTGGATAG GGTCTATATG ATAAAAGTGT AAAAGAAACT TTTCTCCAAA   
  
  
- TGTATATTTC TTTTACACAT TTTTTTTTAT TTTTTATTTT ATTTTATTTC ACTAATTTCT ATTGAAACCT   
  
  
- ACTCCTAAAC CGAGCCCCAT ATCTTCTCAC CTATGAAACT GGATAGAGTT TAAGTGGTCT GAGTACAAAC   
  
  
- GAAACTTTTC TCCAAATATC TATTTCTTTT GCATTTTTTT TTTTTTTATT TATTTTTTTT TACTCACTGA   
  
  
- TTTCTATTGA ATGCACCTAC TCTTAAACTC AACCTCATAT TTTCCTGCCT GTGTAACTGG ATAGGGTCTA   
  
  
- AGTACTCTAA GTGCAAATGG TATCTCCAAA TATCTATTTC TCTTTCATTT TTTTTCTTTT TTGTTACTAA   
  
  
- TTTCTAACAT TGAAAGAGTC GTGGTAGGTC TGATAAGATT ATCTGTAGTG TGTTCAGTAT TATAGTCTTA   
  
  
- AACATCACAA TCAACAAATG CGTGTGTTTA CTATCCGTCG GTAGCTGACA ATGTTTTAAA TATTTTCAGA   
  
  
- GTGTGATTAT CTAATAATTT TCTAATAAAG CATGAACGCG CTGACTACCA TTGTTTTGAG CATCTATCTT   
  
  
- ATATGAAAGA AACTTCACCC TTACTAAACT GGATGAGATA TTTATTATAT TTGTAGTGTA CGTATGTGAG   
  
  
- GTTGGTGATT CGGTGTCTTT CTTTACTTTC TTTGTCTTTT ATTAATTGTT GGGATAACCT GAGAAGAGAT   
  
  
- GTGACGGTCG GAAAGGAAGA AACAAGACTC GTTTCTTTGT GTGGTTTTAA CAGTCGGTAT TTTAAAGAAG   
  
  
- GAGTGTCTTT GAGCAAACCA ATGACTCTT

+     CCGTCC motif

| Site Name | Organism | Position | Strand | Matrix score. | sequence | function |
| --- | --- | --- | --- | --- | --- | --- |
| CCGTCC motif | Nicotiana tabacum | 954 | - | 6 | CCGTCC |  |

>038667   
+ ACGAAAACCG AAGCAATAAC ATTCTATTTA TAGGAGAGGA AAGTCGGCAC AGAGGAAGGT GAGATTAGAC   
  
  
+ CAAGCAAACT TACACCGCCT AAAAGCTATT GATAAGAATG ATGTAAAAGC CCTTTAATTG GCTATGGTTT   
  
  
+ TGGTCCAATT AGAATAGAAC AAACAACTTA ATAAAATAAT GTCAAAAGGA AAGAAAATGA ATAATATGAA   
  
  
+ TATATAGTTC AAATACTACT GTAATGGTGT ACTGAACTTA TTTGGTTCAA AATAGTAGTA ATTAGAGTAT   
  
  
+ AGAAAAATGA AATAAAAGAA TTAACGATTA AATAAGAAAT AATACAAAAC GATATGAAAT TAATAAGTAT   
  
  
+ ATACAACACA TAATTATATA TAATTATTTT GGAAAATATA GGTCCTCACA AGACGCCTAA GTGCTAGCAC   
  
  
+ CTACTTGCTG CTGGCTAGCG CTTAGGTCCT GCTTTTTCTT ATATTATACT CTAAATTATT GTAACTTGGG   
  
  
+ TATAGAAGTG CAGGCAAACT AACCTATCCC ATATTCACCA GATTCACGTT TGCTTTGAAA AGAAGTTTAT   
  
  
+ AGATGAAGAA AATGTTAAAA AGAAAAAAGA GTGATTAGAG ATAACTTGCT TGGATGTGGA TTTTGCTCGG   
  
  
+ GGTAAAGAAG GGCGGGCACA CTGACCTATC CCAGATATAC TATTTTCACA TTTTCTTTGA AAAGAGGTTT   
  
  
+ ACATATAAAG AAAATGTGTA AAAAAAAATA AAAAATAAAA TAAAATAAAG TGATTAAAGA TAACTTTGGA   
  
  
+ TGAGGATTTG GCTCGGGGTA TAGAAGAGTG GATACTTTGA CCTATCTCAA ATTCACCAGA CTCATGTTTG   
  
  
+ CTTTGAAAAG AGGTTTATAG ATAAAGAAAA CGTAAAAAAA AAAAAAATAA ATAAAAAAAA ATGAGTGACT   
  
  
+ AAAGATAACT TACGTGGATG AGAATTTGAG TTGGAGTATA AAAGGACGGA CACATTGACC TATCCCAGAT   
  
  
+ TCATGAGATT CACGTTTACC ATAGAGGTTT ATAGATAAAG AGAAAGTAAA AAAAAGAAAA AACAATGATT   
  
  
+ AAAGATTGTA ACTTTCTCAG CACCATCCAG ACTATTCTAA TAGACATCAC ACAAGTCATA ATATCAGAAT   
  
  
+ TTGTAGTGTT AGTTGTTTAC GCACACAAAT GATAGGCAGC CATCGACTGT TACAAAATTT ATAAAAGTCT   
  
  
+ CACACTAATA GATTATTAAA AGATTATTTC GTACTTGCGC GACTGATGGT AACAAAACTC GTAGATAGAA   
  
  
+ TATACTTTCT TTGAAGTGGG AATGATTTGA CCTACTCTAT AAATAATATA AACATCACAT GCATACACTC   
  
  
+ CAACCACTAA GCCACAGAAA GAAATGAAAG AAACAGAAAA TAATTAACAA CCCTATTGGA CTCTTCTCTA   
  
  
+ CACTGCCAGC CTTTCCTTCT TTGTTCTGAG CAAAGAAACA CACCAAAATT GTCAGCCATA AAATTTCTTC   
  
  
+ CTCACAGAAA CTCGTTTGGT TACTGAGAA  

- TGCTTTTGGC TTCGTTATTG TAAGATAAAT ATCCTCTCCT TTCAGCCGTG TCTCCTTCCA CTCTAATCTG   
  
  
- GTTCGTTTGA ATGTGGCGGA TTTTCGATAA CTATTCTTAC TACATTTTCG GGAAATTAAC CGATACCAAA   
  
  
- ACCAGGTTAA TCTTATCTTG TTTGTTGAAT TATTTTATTA CAGTTTTCCT TTCTTTTACT TATTATACTT   
  
  
- ATATATCAAG TTTATGATGA CATTACCACA TGACTTGAAT AAACCAAGTT TTATCATCAT TAATCTCATA   
  
  
- TCTTTTTACT TTATTTTCTT AATTGCTAAT TTATTCTTTA TTATGTTTTG CTATACTTTA ATTATTCATA   
  
  
- TATGTTGTGT ATTAATATAT ATTAATAAAA CCTTTTATAT CCAGGAGTGT TCTGCGGATT CACGATCGTG   
  
  
- GATGAACGAC GACCGATCGC GAATCCAGGA CGAAAAAGAA TATAATATGA GATTTAATAA CATTGAACCC   
  
  
- ATATCTTCAC GTCCGTTTGA TTGGATAGGG TATAAGTGGT CTAAGTGCAA ACGAAACTTT TCTTCAAATA   
  
  
- TCTACTTCTT TTACAATTTT TCTTTTTTCT CACTAATCTC TATTGAACGA ACCTACACCT AAAACGAGCC   
  
  
- CCATTTCTTC CCGCCCGTGT GACTGGATAG GGTCTATATG ATAAAAGTGT AAAAGAAACT TTTCTCCAAA   
  
  
- TGTATATTTC TTTTACACAT TTTTTTTTAT TTTTTATTTT ATTTTATTTC ACTAATTTCT ATTGAAACCT   
  
  
- ACTCCTAAAC CGAGCCCCAT ATCTTCTCAC CTATGAAACT GGATAGAGTT TAAGTGGTCT GAGTACAAAC   
  
  
- GAAACTTTTC TCCAAATATC TATTTCTTTT GCATTTTTTT TTTTTTTATT TATTTTTTTT TACTCACTGA   
  
  
- TTTCTATTGA ATGCACCTAC TCTTAAACTC AACCTCATAT TTTCCTGCCT GTGTAACTGG ATAGGGTCTA   
  
  
- AGTACTCTAA GTGCAAATGG TATCTCCAAA TATCTATTTC TCTTTCATTT TTTTTCTTTT TTGTTACTAA   
  
  
- TTTCTAACAT TGAAAGAGTC GTGGTAGGTC TGATAAGATT ATCTGTAGTG TGTTCAGTAT TATAGTCTTA   
  
  
- AACATCACAA TCAACAAATG CGTGTGTTTA CTATCCGTCG GTAGCTGACA ATGTTTTAAA TATTTTCAGA   
  
  
- GTGTGATTAT CTAATAATTT TCTAATAAAG CATGAACGCG CTGACTACCA TTGTTTTGAG CATCTATCTT   
  
  
- ATATGAAAGA AACTTCACCC TTACTAAACT GGATGAGATA TTTATTATAT TTGTAGTGTA CGTATGTGAG   
  
  
- GTTGGTGATT CGGTGTCTTT CTTTACTTTC TTTGTCTTTT ATTAATTGTT GGGATAACCT GAGAAGAGAT   
  
  
- GTGACGGTCG GAAAGGAAGA AACAAGACTC GTTTCTTTGT GTGGTTTTAA CAGTCGGTAT TTTAAAGAAG   
  
  
- GAGTGTCTTT GAGCAAACCA ATGACTCTT

+     CCGTCC-box

| Site Name | Organism | Position | Strand | Matrix score. | sequence | function |
| --- | --- | --- | --- | --- | --- | --- |
| CCGTCC-box | Petroselinum hortense | 954 | - | 6 | CCGTCC |  |

>038667   
+ ACGAAAACCG AAGCAATAAC ATTCTATTTA TAGGAGAGGA AAGTCGGCAC AGAGGAAGGT GAGATTAGAC   
  
  
+ CAAGCAAACT TACACCGCCT AAAAGCTATT GATAAGAATG ATGTAAAAGC CCTTTAATTG GCTATGGTTT   
  
  
+ TGGTCCAATT AGAATAGAAC AAACAACTTA ATAAAATAAT GTCAAAAGGA AAGAAAATGA ATAATATGAA   
  
  
+ TATATAGTTC AAATACTACT GTAATGGTGT ACTGAACTTA TTTGGTTCAA AATAGTAGTA ATTAGAGTAT   
  
  
+ AGAAAAATGA AATAAAAGAA TTAACGATTA AATAAGAAAT AATACAAAAC GATATGAAAT TAATAAGTAT   
  
  
+ ATACAACACA TAATTATATA TAATTATTTT GGAAAATATA GGTCCTCACA AGACGCCTAA GTGCTAGCAC   
  
  
+ CTACTTGCTG CTGGCTAGCG CTTAGGTCCT GCTTTTTCTT ATATTATACT CTAAATTATT GTAACTTGGG   
  
  
+ TATAGAAGTG CAGGCAAACT AACCTATCCC ATATTCACCA GATTCACGTT TGCTTTGAAA AGAAGTTTAT   
  
  
+ AGATGAAGAA AATGTTAAAA AGAAAAAAGA GTGATTAGAG ATAACTTGCT TGGATGTGGA TTTTGCTCGG   
  
  
+ GGTAAAGAAG GGCGGGCACA CTGACCTATC CCAGATATAC TATTTTCACA TTTTCTTTGA AAAGAGGTTT   
  
  
+ ACATATAAAG AAAATGTGTA AAAAAAAATA AAAAATAAAA TAAAATAAAG TGATTAAAGA TAACTTTGGA   
  
  
+ TGAGGATTTG GCTCGGGGTA TAGAAGAGTG GATACTTTGA CCTATCTCAA ATTCACCAGA CTCATGTTTG   
  
  
+ CTTTGAAAAG AGGTTTATAG ATAAAGAAAA CGTAAAAAAA AAAAAAATAA ATAAAAAAAA ATGAGTGACT   
  
  
+ AAAGATAACT TACGTGGATG AGAATTTGAG TTGGAGTATA AAAGGACGGA CACATTGACC TATCCCAGAT   
  
  
+ TCATGAGATT CACGTTTACC ATAGAGGTTT ATAGATAAAG AGAAAGTAAA AAAAAGAAAA AACAATGATT   
  
  
+ AAAGATTGTA ACTTTCTCAG CACCATCCAG ACTATTCTAA TAGACATCAC ACAAGTCATA ATATCAGAAT   
  
  
+ TTGTAGTGTT AGTTGTTTAC GCACACAAAT GATAGGCAGC CATCGACTGT TACAAAATTT ATAAAAGTCT   
  
  
+ CACACTAATA GATTATTAAA AGATTATTTC GTACTTGCGC GACTGATGGT AACAAAACTC GTAGATAGAA   
  
  
+ TATACTTTCT TTGAAGTGGG AATGATTTGA CCTACTCTAT AAATAATATA AACATCACAT GCATACACTC   
  
  
+ CAACCACTAA GCCACAGAAA GAAATGAAAG AAACAGAAAA TAATTAACAA CCCTATTGGA CTCTTCTCTA   
  
  
+ CACTGCCAGC CTTTCCTTCT TTGTTCTGAG CAAAGAAACA CACCAAAATT GTCAGCCATA AAATTTCTTC   
  
  
+ CTCACAGAAA CTCGTTTGGT TACTGAGAA  

- TGCTTTTGGC TTCGTTATTG TAAGATAAAT ATCCTCTCCT TTCAGCCGTG TCTCCTTCCA CTCTAATCTG   
  
  
- GTTCGTTTGA ATGTGGCGGA TTTTCGATAA CTATTCTTAC TACATTTTCG GGAAATTAAC CGATACCAAA   
  
  
- ACCAGGTTAA TCTTATCTTG TTTGTTGAAT TATTTTATTA CAGTTTTCCT TTCTTTTACT TATTATACTT   
  
  
- ATATATCAAG TTTATGATGA CATTACCACA TGACTTGAAT AAACCAAGTT TTATCATCAT TAATCTCATA   
  
  
- TCTTTTTACT TTATTTTCTT AATTGCTAAT TTATTCTTTA TTATGTTTTG CTATACTTTA ATTATTCATA   
  
  
- TATGTTGTGT ATTAATATAT ATTAATAAAA CCTTTTATAT CCAGGAGTGT TCTGCGGATT CACGATCGTG   
  
  
- GATGAACGAC GACCGATCGC GAATCCAGGA CGAAAAAGAA TATAATATGA GATTTAATAA CATTGAACCC   
  
  
- ATATCTTCAC GTCCGTTTGA TTGGATAGGG TATAAGTGGT CTAAGTGCAA ACGAAACTTT TCTTCAAATA   
  
  
- TCTACTTCTT TTACAATTTT TCTTTTTTCT CACTAATCTC TATTGAACGA ACCTACACCT AAAACGAGCC   
  
  
- CCATTTCTTC CCGCCCGTGT GACTGGATAG GGTCTATATG ATAAAAGTGT AAAAGAAACT TTTCTCCAAA   
  
  
- TGTATATTTC TTTTACACAT TTTTTTTTAT TTTTTATTTT ATTTTATTTC ACTAATTTCT ATTGAAACCT   
  
  
- ACTCCTAAAC CGAGCCCCAT ATCTTCTCAC CTATGAAACT GGATAGAGTT TAAGTGGTCT GAGTACAAAC   
  
  
- GAAACTTTTC TCCAAATATC TATTTCTTTT GCATTTTTTT TTTTTTTATT TATTTTTTTT TACTCACTGA   
  
  
- TTTCTATTGA ATGCACCTAC TCTTAAACTC AACCTCATAT TTTCCTGCCT GTGTAACTGG ATAGGGTCTA   
  
  
- AGTACTCTAA GTGCAAATGG TATCTCCAAA TATCTATTTC TCTTTCATTT TTTTTCTTTT TTGTTACTAA   
  
  
- TTTCTAACAT TGAAAGAGTC GTGGTAGGTC TGATAAGATT ATCTGTAGTG TGTTCAGTAT TATAGTCTTA   
  
  
- AACATCACAA TCAACAAATG CGTGTGTTTA CTATCCGTCG GTAGCTGACA ATGTTTTAAA TATTTTCAGA   
  
  
- GTGTGATTAT CTAATAATTT TCTAATAAAG CATGAACGCG CTGACTACCA TTGTTTTGAG CATCTATCTT   
  
  
- ATATGAAAGA AACTTCACCC TTACTAAACT GGATGAGATA TTTATTATAT TTGTAGTGTA CGTATGTGAG   
  
  
- GTTGGTGATT CGGTGTCTTT CTTTACTTTC TTTGTCTTTT ATTAATTGTT GGGATAACCT GAGAAGAGAT   
  
  
- GTGACGGTCG GAAAGGAAGA AACAAGACTC GTTTCTTTGT GTGGTTTTAA CAGTCGGTAT TTTAAAGAAG   
  
  
- GAGTGTCTTT GAGCAAACCA ATGACTCTT

+     DRE core

| Site Name | Organism | Position | Strand | Matrix score. | sequence | function |
| --- | --- | --- | --- | --- | --- | --- |
| DRE core | Arabidopsis thaliana | 43 | - | 6 | GCCGAC |  |

>038667   
+ ACGAAAACCG AAGCAATAAC ATTCTATTTA TAGGAGAGGA AAGTCGGCAC AGAGGAAGGT GAGATTAGAC   
  
  
+ CAAGCAAACT TACACCGCCT AAAAGCTATT GATAAGAATG ATGTAAAAGC CCTTTAATTG GCTATGGTTT   
  
  
+ TGGTCCAATT AGAATAGAAC AAACAACTTA ATAAAATAAT GTCAAAAGGA AAGAAAATGA ATAATATGAA   
  
  
+ TATATAGTTC AAATACTACT GTAATGGTGT ACTGAACTTA TTTGGTTCAA AATAGTAGTA ATTAGAGTAT   
  
  
+ AGAAAAATGA AATAAAAGAA TTAACGATTA AATAAGAAAT AATACAAAAC GATATGAAAT TAATAAGTAT   
  
  
+ ATACAACACA TAATTATATA TAATTATTTT GGAAAATATA GGTCCTCACA AGACGCCTAA GTGCTAGCAC   
  
  
+ CTACTTGCTG CTGGCTAGCG CTTAGGTCCT GCTTTTTCTT ATATTATACT CTAAATTATT GTAACTTGGG   
  
  
+ TATAGAAGTG CAGGCAAACT AACCTATCCC ATATTCACCA GATTCACGTT TGCTTTGAAA AGAAGTTTAT   
  
  
+ AGATGAAGAA AATGTTAAAA AGAAAAAAGA GTGATTAGAG ATAACTTGCT TGGATGTGGA TTTTGCTCGG   
  
  
+ GGTAAAGAAG GGCGGGCACA CTGACCTATC CCAGATATAC TATTTTCACA TTTTCTTTGA AAAGAGGTTT   
  
  
+ ACATATAAAG AAAATGTGTA AAAAAAAATA AAAAATAAAA TAAAATAAAG TGATTAAAGA TAACTTTGGA   
  
  
+ TGAGGATTTG GCTCGGGGTA TAGAAGAGTG GATACTTTGA CCTATCTCAA ATTCACCAGA CTCATGTTTG   
  
  
+ CTTTGAAAAG AGGTTTATAG ATAAAGAAAA CGTAAAAAAA AAAAAAATAA ATAAAAAAAA ATGAGTGACT   
  
  
+ AAAGATAACT TACGTGGATG AGAATTTGAG TTGGAGTATA AAAGGACGGA CACATTGACC TATCCCAGAT   
  
  
+ TCATGAGATT CACGTTTACC ATAGAGGTTT ATAGATAAAG AGAAAGTAAA AAAAAGAAAA AACAATGATT   
  
  
+ AAAGATTGTA ACTTTCTCAG CACCATCCAG ACTATTCTAA TAGACATCAC ACAAGTCATA ATATCAGAAT   
  
  
+ TTGTAGTGTT AGTTGTTTAC GCACACAAAT GATAGGCAGC CATCGACTGT TACAAAATTT ATAAAAGTCT   
  
  
+ CACACTAATA GATTATTAAA AGATTATTTC GTACTTGCGC GACTGATGGT AACAAAACTC GTAGATAGAA   
  
  
+ TATACTTTCT TTGAAGTGGG AATGATTTGA CCTACTCTAT AAATAATATA AACATCACAT GCATACACTC   
  
  
+ CAACCACTAA GCCACAGAAA GAAATGAAAG AAACAGAAAA TAATTAACAA CCCTATTGGA CTCTTCTCTA   
  
  
+ CACTGCCAGC CTTTCCTTCT TTGTTCTGAG CAAAGAAACA CACCAAAATT GTCAGCCATA AAATTTCTTC   
  
  
+ CTCACAGAAA CTCGTTTGGT TACTGAGAA  

- TGCTTTTGGC TTCGTTATTG TAAGATAAAT ATCCTCTCCT TTCAGCCGTG TCTCCTTCCA CTCTAATCTG   
  
  
- GTTCGTTTGA ATGTGGCGGA TTTTCGATAA CTATTCTTAC TACATTTTCG GGAAATTAAC CGATACCAAA   
  
  
- ACCAGGTTAA TCTTATCTTG TTTGTTGAAT TATTTTATTA CAGTTTTCCT TTCTTTTACT TATTATACTT   
  
  
- ATATATCAAG TTTATGATGA CATTACCACA TGACTTGAAT AAACCAAGTT TTATCATCAT TAATCTCATA   
  
  
- TCTTTTTACT TTATTTTCTT AATTGCTAAT TTATTCTTTA TTATGTTTTG CTATACTTTA ATTATTCATA   
  
  
- TATGTTGTGT ATTAATATAT ATTAATAAAA CCTTTTATAT CCAGGAGTGT TCTGCGGATT CACGATCGTG   
  
  
- GATGAACGAC GACCGATCGC GAATCCAGGA CGAAAAAGAA TATAATATGA GATTTAATAA CATTGAACCC   
  
  
- ATATCTTCAC GTCCGTTTGA TTGGATAGGG TATAAGTGGT CTAAGTGCAA ACGAAACTTT TCTTCAAATA   
  
  
- TCTACTTCTT TTACAATTTT TCTTTTTTCT CACTAATCTC TATTGAACGA ACCTACACCT AAAACGAGCC   
  
  
- CCATTTCTTC CCGCCCGTGT GACTGGATAG GGTCTATATG ATAAAAGTGT AAAAGAAACT TTTCTCCAAA   
  
  
- TGTATATTTC TTTTACACAT TTTTTTTTAT TTTTTATTTT ATTTTATTTC ACTAATTTCT ATTGAAACCT   
  
  
- ACTCCTAAAC CGAGCCCCAT ATCTTCTCAC CTATGAAACT GGATAGAGTT TAAGTGGTCT GAGTACAAAC   
  
  
- GAAACTTTTC TCCAAATATC TATTTCTTTT GCATTTTTTT TTTTTTTATT TATTTTTTTT TACTCACTGA   
  
  
- TTTCTATTGA ATGCACCTAC TCTTAAACTC AACCTCATAT TTTCCTGCCT GTGTAACTGG ATAGGGTCTA   
  
  
- AGTACTCTAA GTGCAAATGG TATCTCCAAA TATCTATTTC TCTTTCATTT TTTTTCTTTT TTGTTACTAA   
  
  
- TTTCTAACAT TGAAAGAGTC GTGGTAGGTC TGATAAGATT ATCTGTAGTG TGTTCAGTAT TATAGTCTTA   
  
  
- AACATCACAA TCAACAAATG CGTGTGTTTA CTATCCGTCG GTAGCTGACA ATGTTTTAAA TATTTTCAGA   
  
  
- GTGTGATTAT CTAATAATTT TCTAATAAAG CATGAACGCG CTGACTACCA TTGTTTTGAG CATCTATCTT   
  
  
- ATATGAAAGA AACTTCACCC TTACTAAACT GGATGAGATA TTTATTATAT TTGTAGTGTA CGTATGTGAG   
  
  
- GTTGGTGATT CGGTGTCTTT CTTTACTTTC TTTGTCTTTT ATTAATTGTT GGGATAACCT GAGAAGAGAT   
  
  
- GTGACGGTCG GAAAGGAAGA AACAAGACTC GTTTCTTTGT GTGGTTTTAA CAGTCGGTAT TTTAAAGAAG   
  
  
- GAGTGTCTTT GAGCAAACCA ATGACTCTT

+     ERE

| Site Name | Organism | Position | Strand | Matrix score. | sequence | function |
| --- | --- | --- | --- | --- | --- | --- |
| ERE | Nicotiana glutinos | 333 | - | 8 | ATTTCATA |  |

>038667   
+ ACGAAAACCG AAGCAATAAC ATTCTATTTA TAGGAGAGGA AAGTCGGCAC AGAGGAAGGT GAGATTAGAC   
  
  
+ CAAGCAAACT TACACCGCCT AAAAGCTATT GATAAGAATG ATGTAAAAGC CCTTTAATTG GCTATGGTTT   
  
  
+ TGGTCCAATT AGAATAGAAC AAACAACTTA ATAAAATAAT GTCAAAAGGA AAGAAAATGA ATAATATGAA   
  
  
+ TATATAGTTC AAATACTACT GTAATGGTGT ACTGAACTTA TTTGGTTCAA AATAGTAGTA ATTAGAGTAT   
  
  
+ AGAAAAATGA AATAAAAGAA TTAACGATTA AATAAGAAAT AATACAAAAC GATATGAAAT TAATAAGTAT   
  
  
+ ATACAACACA TAATTATATA TAATTATTTT GGAAAATATA GGTCCTCACA AGACGCCTAA GTGCTAGCAC   
  
  
+ CTACTTGCTG CTGGCTAGCG CTTAGGTCCT GCTTTTTCTT ATATTATACT CTAAATTATT GTAACTTGGG   
  
  
+ TATAGAAGTG CAGGCAAACT AACCTATCCC ATATTCACCA GATTCACGTT TGCTTTGAAA AGAAGTTTAT   
  
  
+ AGATGAAGAA AATGTTAAAA AGAAAAAAGA GTGATTAGAG ATAACTTGCT TGGATGTGGA TTTTGCTCGG   
  
  
+ GGTAAAGAAG GGCGGGCACA CTGACCTATC CCAGATATAC TATTTTCACA TTTTCTTTGA AAAGAGGTTT   
  
  
+ ACATATAAAG AAAATGTGTA AAAAAAAATA AAAAATAAAA TAAAATAAAG TGATTAAAGA TAACTTTGGA   
  
  
+ TGAGGATTTG GCTCGGGGTA TAGAAGAGTG GATACTTTGA CCTATCTCAA ATTCACCAGA CTCATGTTTG   
  
  
+ CTTTGAAAAG AGGTTTATAG ATAAAGAAAA CGTAAAAAAA AAAAAAATAA ATAAAAAAAA ATGAGTGACT   
  
  
+ AAAGATAACT TACGTGGATG AGAATTTGAG TTGGAGTATA AAAGGACGGA CACATTGACC TATCCCAGAT   
  
  
+ TCATGAGATT CACGTTTACC ATAGAGGTTT ATAGATAAAG AGAAAGTAAA AAAAAGAAAA AACAATGATT   
  
  
+ AAAGATTGTA ACTTTCTCAG CACCATCCAG ACTATTCTAA TAGACATCAC ACAAGTCATA ATATCAGAAT   
  
  
+ TTGTAGTGTT AGTTGTTTAC GCACACAAAT GATAGGCAGC CATCGACTGT TACAAAATTT ATAAAAGTCT   
  
  
+ CACACTAATA GATTATTAAA AGATTATTTC GTACTTGCGC GACTGATGGT AACAAAACTC GTAGATAGAA   
  
  
+ TATACTTTCT TTGAAGTGGG AATGATTTGA CCTACTCTAT AAATAATATA AACATCACAT GCATACACTC   
  
  
+ CAACCACTAA GCCACAGAAA GAAATGAAAG AAACAGAAAA TAATTAACAA CCCTATTGGA CTCTTCTCTA   
  
  
+ CACTGCCAGC CTTTCCTTCT TTGTTCTGAG CAAAGAAACA CACCAAAATT GTCAGCCATA AAATTTCTTC   
  
  
+ CTCACAGAAA CTCGTTTGGT TACTGAGAA  

- TGCTTTTGGC TTCGTTATTG TAAGATAAAT ATCCTCTCCT TTCAGCCGTG TCTCCTTCCA CTCTAATCTG   
  
  
- GTTCGTTTGA ATGTGGCGGA TTTTCGATAA CTATTCTTAC TACATTTTCG GGAAATTAAC CGATACCAAA   
  
  
- ACCAGGTTAA TCTTATCTTG TTTGTTGAAT TATTTTATTA CAGTTTTCCT TTCTTTTACT TATTATACTT   
  
  
- ATATATCAAG TTTATGATGA CATTACCACA TGACTTGAAT AAACCAAGTT TTATCATCAT TAATCTCATA   
  
  
- TCTTTTTACT TTATTTTCTT AATTGCTAAT TTATTCTTTA TTATGTTTTG CTATACTTTA ATTATTCATA   
  
  
- TATGTTGTGT ATTAATATAT ATTAATAAAA CCTTTTATAT CCAGGAGTGT TCTGCGGATT CACGATCGTG   
  
  
- GATGAACGAC GACCGATCGC GAATCCAGGA CGAAAAAGAA TATAATATGA GATTTAATAA CATTGAACCC   
  
  
- ATATCTTCAC GTCCGTTTGA TTGGATAGGG TATAAGTGGT CTAAGTGCAA ACGAAACTTT TCTTCAAATA   
  
  
- TCTACTTCTT TTACAATTTT TCTTTTTTCT CACTAATCTC TATTGAACGA ACCTACACCT AAAACGAGCC   
  
  
- CCATTTCTTC CCGCCCGTGT GACTGGATAG GGTCTATATG ATAAAAGTGT AAAAGAAACT TTTCTCCAAA   
  
  
- TGTATATTTC TTTTACACAT TTTTTTTTAT TTTTTATTTT ATTTTATTTC ACTAATTTCT ATTGAAACCT   
  
  
- ACTCCTAAAC CGAGCCCCAT ATCTTCTCAC CTATGAAACT GGATAGAGTT TAAGTGGTCT GAGTACAAAC   
  
  
- GAAACTTTTC TCCAAATATC TATTTCTTTT GCATTTTTTT TTTTTTTATT TATTTTTTTT TACTCACTGA   
  
  
- TTTCTATTGA ATGCACCTAC TCTTAAACTC AACCTCATAT TTTCCTGCCT GTGTAACTGG ATAGGGTCTA   
  
  
- AGTACTCTAA GTGCAAATGG TATCTCCAAA TATCTATTTC TCTTTCATTT TTTTTCTTTT TTGTTACTAA   
  
  
- TTTCTAACAT TGAAAGAGTC GTGGTAGGTC TGATAAGATT ATCTGTAGTG TGTTCAGTAT TATAGTCTTA   
  
  
- AACATCACAA TCAACAAATG CGTGTGTTTA CTATCCGTCG GTAGCTGACA ATGTTTTAAA TATTTTCAGA   
  
  
- GTGTGATTAT CTAATAATTT TCTAATAAAG CATGAACGCG CTGACTACCA TTGTTTTGAG CATCTATCTT   
  
  
- ATATGAAAGA AACTTCACCC TTACTAAACT GGATGAGATA TTTATTATAT TTGTAGTGTA CGTATGTGAG   
  
  
- GTTGGTGATT CGGTGTCTTT CTTTACTTTC TTTGTCTTTT ATTAATTGTT GGGATAACCT GAGAAGAGAT   
  
  
- GTGACGGTCG GAAAGGAAGA AACAAGACTC GTTTCTTTGT GTGGTTTTAA CAGTCGGTAT TTTAAAGAAG   
  
  
- GAGTGTCTTT GAGCAAACCA ATGACTCTT

+     F-box

| Site Name | Organism | Position | Strand | Matrix score. | sequence | function |
| --- | --- | --- | --- | --- | --- | --- |
| F-box | Lycopersicon esculentum | 147 | - | 10 | CTATTCTCATT |  |

>038667   
+ ACGAAAACCG AAGCAATAAC ATTCTATTTA TAGGAGAGGA AAGTCGGCAC AGAGGAAGGT GAGATTAGAC   
  
  
+ CAAGCAAACT TACACCGCCT AAAAGCTATT GATAAGAATG ATGTAAAAGC CCTTTAATTG GCTATGGTTT   
  
  
+ TGGTCCAATT AGAATAGAAC AAACAACTTA ATAAAATAAT GTCAAAAGGA AAGAAAATGA ATAATATGAA   
  
  
+ TATATAGTTC AAATACTACT GTAATGGTGT ACTGAACTTA TTTGGTTCAA AATAGTAGTA ATTAGAGTAT   
  
  
+ AGAAAAATGA AATAAAAGAA TTAACGATTA AATAAGAAAT AATACAAAAC GATATGAAAT TAATAAGTAT   
  
  
+ ATACAACACA TAATTATATA TAATTATTTT GGAAAATATA GGTCCTCACA AGACGCCTAA GTGCTAGCAC   
  
  
+ CTACTTGCTG CTGGCTAGCG CTTAGGTCCT GCTTTTTCTT ATATTATACT CTAAATTATT GTAACTTGGG   
  
  
+ TATAGAAGTG CAGGCAAACT AACCTATCCC ATATTCACCA GATTCACGTT TGCTTTGAAA AGAAGTTTAT   
  
  
+ AGATGAAGAA AATGTTAAAA AGAAAAAAGA GTGATTAGAG ATAACTTGCT TGGATGTGGA TTTTGCTCGG   
  
  
+ GGTAAAGAAG GGCGGGCACA CTGACCTATC CCAGATATAC TATTTTCACA TTTTCTTTGA AAAGAGGTTT   
  
  
+ ACATATAAAG AAAATGTGTA AAAAAAAATA AAAAATAAAA TAAAATAAAG TGATTAAAGA TAACTTTGGA   
  
  
+ TGAGGATTTG GCTCGGGGTA TAGAAGAGTG GATACTTTGA CCTATCTCAA ATTCACCAGA CTCATGTTTG   
  
  
+ CTTTGAAAAG AGGTTTATAG ATAAAGAAAA CGTAAAAAAA AAAAAAATAA ATAAAAAAAA ATGAGTGACT   
  
  
+ AAAGATAACT TACGTGGATG AGAATTTGAG TTGGAGTATA AAAGGACGGA CACATTGACC TATCCCAGAT   
  
  
+ TCATGAGATT CACGTTTACC ATAGAGGTTT ATAGATAAAG AGAAAGTAAA AAAAAGAAAA AACAATGATT   
  
  
+ AAAGATTGTA ACTTTCTCAG CACCATCCAG ACTATTCTAA TAGACATCAC ACAAGTCATA ATATCAGAAT   
  
  
+ TTGTAGTGTT AGTTGTTTAC GCACACAAAT GATAGGCAGC CATCGACTGT TACAAAATTT ATAAAAGTCT   
  
  
+ CACACTAATA GATTATTAAA AGATTATTTC GTACTTGCGC GACTGATGGT AACAAAACTC GTAGATAGAA   
  
  
+ TATACTTTCT TTGAAGTGGG AATGATTTGA CCTACTCTAT AAATAATATA AACATCACAT GCATACACTC   
  
  
+ CAACCACTAA GCCACAGAAA GAAATGAAAG AAACAGAAAA TAATTAACAA CCCTATTGGA CTCTTCTCTA   
  
  
+ CACTGCCAGC CTTTCCTTCT TTGTTCTGAG CAAAGAAACA CACCAAAATT GTCAGCCATA AAATTTCTTC   
  
  
+ CTCACAGAAA CTCGTTTGGT TACTGAGAA  

- TGCTTTTGGC TTCGTTATTG TAAGATAAAT ATCCTCTCCT TTCAGCCGTG TCTCCTTCCA CTCTAATCTG   
  
  
- GTTCGTTTGA ATGTGGCGGA TTTTCGATAA CTATTCTTAC TACATTTTCG GGAAATTAAC CGATACCAAA   
  
  
- ACCAGGTTAA TCTTATCTTG TTTGTTGAAT TATTTTATTA CAGTTTTCCT TTCTTTTACT TATTATACTT   
  
  
- ATATATCAAG TTTATGATGA CATTACCACA TGACTTGAAT AAACCAAGTT TTATCATCAT TAATCTCATA   
  
  
- TCTTTTTACT TTATTTTCTT AATTGCTAAT TTATTCTTTA TTATGTTTTG CTATACTTTA ATTATTCATA   
  
  
- TATGTTGTGT ATTAATATAT ATTAATAAAA CCTTTTATAT CCAGGAGTGT TCTGCGGATT CACGATCGTG   
  
  
- GATGAACGAC GACCGATCGC GAATCCAGGA CGAAAAAGAA TATAATATGA GATTTAATAA CATTGAACCC   
  
  
- ATATCTTCAC GTCCGTTTGA TTGGATAGGG TATAAGTGGT CTAAGTGCAA ACGAAACTTT TCTTCAAATA   
  
  
- TCTACTTCTT TTACAATTTT TCTTTTTTCT CACTAATCTC TATTGAACGA ACCTACACCT AAAACGAGCC   
  
  
- CCATTTCTTC CCGCCCGTGT GACTGGATAG GGTCTATATG ATAAAAGTGT AAAAGAAACT TTTCTCCAAA   
  
  
- TGTATATTTC TTTTACACAT TTTTTTTTAT TTTTTATTTT ATTTTATTTC ACTAATTTCT ATTGAAACCT   
  
  
- ACTCCTAAAC CGAGCCCCAT ATCTTCTCAC CTATGAAACT GGATAGAGTT TAAGTGGTCT GAGTACAAAC   
  
  
- GAAACTTTTC TCCAAATATC TATTTCTTTT GCATTTTTTT TTTTTTTATT TATTTTTTTT TACTCACTGA   
  
  
- TTTCTATTGA ATGCACCTAC TCTTAAACTC AACCTCATAT TTTCCTGCCT GTGTAACTGG ATAGGGTCTA   
  
  
- AGTACTCTAA GTGCAAATGG TATCTCCAAA TATCTATTTC TCTTTCATTT TTTTTCTTTT TTGTTACTAA   
  
  
- TTTCTAACAT TGAAAGAGTC GTGGTAGGTC TGATAAGATT ATCTGTAGTG TGTTCAGTAT TATAGTCTTA   
  
  
- AACATCACAA TCAACAAATG CGTGTGTTTA CTATCCGTCG GTAGCTGACA ATGTTTTAAA TATTTTCAGA   
  
  
- GTGTGATTAT CTAATAATTT TCTAATAAAG CATGAACGCG CTGACTACCA TTGTTTTGAG CATCTATCTT   
  
  
- ATATGAAAGA AACTTCACCC TTACTAAACT GGATGAGATA TTTATTATAT TTGTAGTGTA CGTATGTGAG   
  
  
- GTTGGTGATT CGGTGTCTTT CTTTACTTTC TTTGTCTTTT ATTAATTGTT GGGATAACCT GAGAAGAGAT   
  
  
- GTGACGGTCG GAAAGGAAGA AACAAGACTC GTTTCTTTGT GTGGTTTTAA CAGTCGGTAT TTTAAAGAAG   
  
  
- GAGTGTCTTT GAGCAAACCA ATGACTCTT

+     G-Box

| Site Name | Organism | Position | Strand | Matrix score. | sequence | function |
| --- | --- | --- | --- | --- | --- | --- |
| G-Box | Pisum sativum | 991 | + | 6 | CACGTT | cis-acting regulatory element involved in light responsiveness |
| G-Box | Pisum sativum | 535 | + | 6 | CACGTT | cis-acting regulatory element involved in light responsiveness |

>038667   
+ ACGAAAACCG AAGCAATAAC ATTCTATTTA TAGGAGAGGA AAGTCGGCAC AGAGGAAGGT GAGATTAGAC   
  
  
+ CAAGCAAACT TACACCGCCT AAAAGCTATT GATAAGAATG ATGTAAAAGC CCTTTAATTG GCTATGGTTT   
  
  
+ TGGTCCAATT AGAATAGAAC AAACAACTTA ATAAAATAAT GTCAAAAGGA AAGAAAATGA ATAATATGAA   
  
  
+ TATATAGTTC AAATACTACT GTAATGGTGT ACTGAACTTA TTTGGTTCAA AATAGTAGTA ATTAGAGTAT   
  
  
+ AGAAAAATGA AATAAAAGAA TTAACGATTA AATAAGAAAT AATACAAAAC GATATGAAAT TAATAAGTAT   
  
  
+ ATACAACACA TAATTATATA TAATTATTTT GGAAAATATA GGTCCTCACA AGACGCCTAA GTGCTAGCAC   
  
  
+ CTACTTGCTG CTGGCTAGCG CTTAGGTCCT GCTTTTTCTT ATATTATACT CTAAATTATT GTAACTTGGG   
  
  
+ TATAGAAGTG CAGGCAAACT AACCTATCCC ATATTCACCA GATTCACGTT TGCTTTGAAA AGAAGTTTAT   
  
  
+ AGATGAAGAA AATGTTAAAA AGAAAAAAGA GTGATTAGAG ATAACTTGCT TGGATGTGGA TTTTGCTCGG   
  
  
+ GGTAAAGAAG GGCGGGCACA CTGACCTATC CCAGATATAC TATTTTCACA TTTTCTTTGA AAAGAGGTTT   
  
  
+ ACATATAAAG AAAATGTGTA AAAAAAAATA AAAAATAAAA TAAAATAAAG TGATTAAAGA TAACTTTGGA   
  
  
+ TGAGGATTTG GCTCGGGGTA TAGAAGAGTG GATACTTTGA CCTATCTCAA ATTCACCAGA CTCATGTTTG   
  
  
+ CTTTGAAAAG AGGTTTATAG ATAAAGAAAA CGTAAAAAAA AAAAAAATAA ATAAAAAAAA ATGAGTGACT   
  
  
+ AAAGATAACT TACGTGGATG AGAATTTGAG TTGGAGTATA AAAGGACGGA CACATTGACC TATCCCAGAT   
  
  
+ TCATGAGATT CACGTTTACC ATAGAGGTTT ATAGATAAAG AGAAAGTAAA AAAAAGAAAA AACAATGATT   
  
  
+ AAAGATTGTA ACTTTCTCAG CACCATCCAG ACTATTCTAA TAGACATCAC ACAAGTCATA ATATCAGAAT   
  
  
+ TTGTAGTGTT AGTTGTTTAC GCACACAAAT GATAGGCAGC CATCGACTGT TACAAAATTT ATAAAAGTCT   
  
  
+ CACACTAATA GATTATTAAA AGATTATTTC GTACTTGCGC GACTGATGGT AACAAAACTC GTAGATAGAA   
  
  
+ TATACTTTCT TTGAAGTGGG AATGATTTGA CCTACTCTAT AAATAATATA AACATCACAT GCATACACTC   
  
  
+ CAACCACTAA GCCACAGAAA GAAATGAAAG AAACAGAAAA TAATTAACAA CCCTATTGGA CTCTTCTCTA   
  
  
+ CACTGCCAGC CTTTCCTTCT TTGTTCTGAG CAAAGAAACA CACCAAAATT GTCAGCCATA AAATTTCTTC   
  
  
+ CTCACAGAAA CTCGTTTGGT TACTGAGAA  

- TGCTTTTGGC TTCGTTATTG TAAGATAAAT ATCCTCTCCT TTCAGCCGTG TCTCCTTCCA CTCTAATCTG   
  
  
- GTTCGTTTGA ATGTGGCGGA TTTTCGATAA CTATTCTTAC TACATTTTCG GGAAATTAAC CGATACCAAA   
  
  
- ACCAGGTTAA TCTTATCTTG TTTGTTGAAT TATTTTATTA CAGTTTTCCT TTCTTTTACT TATTATACTT   
  
  
- ATATATCAAG TTTATGATGA CATTACCACA TGACTTGAAT AAACCAAGTT TTATCATCAT TAATCTCATA   
  
  
- TCTTTTTACT TTATTTTCTT AATTGCTAAT TTATTCTTTA TTATGTTTTG CTATACTTTA ATTATTCATA   
  
  
- TATGTTGTGT ATTAATATAT ATTAATAAAA CCTTTTATAT CCAGGAGTGT TCTGCGGATT CACGATCGTG   
  
  
- GATGAACGAC GACCGATCGC GAATCCAGGA CGAAAAAGAA TATAATATGA GATTTAATAA CATTGAACCC   
  
  
- ATATCTTCAC GTCCGTTTGA TTGGATAGGG TATAAGTGGT CTAAGTGCAA ACGAAACTTT TCTTCAAATA   
  
  
- TCTACTTCTT TTACAATTTT TCTTTTTTCT CACTAATCTC TATTGAACGA ACCTACACCT AAAACGAGCC   
  
  
- CCATTTCTTC CCGCCCGTGT GACTGGATAG GGTCTATATG ATAAAAGTGT AAAAGAAACT TTTCTCCAAA   
  
  
- TGTATATTTC TTTTACACAT TTTTTTTTAT TTTTTATTTT ATTTTATTTC ACTAATTTCT ATTGAAACCT   
  
  
- ACTCCTAAAC CGAGCCCCAT ATCTTCTCAC CTATGAAACT GGATAGAGTT TAAGTGGTCT GAGTACAAAC   
  
  
- GAAACTTTTC TCCAAATATC TATTTCTTTT GCATTTTTTT TTTTTTTATT TATTTTTTTT TACTCACTGA   
  
  
- TTTCTATTGA ATGCACCTAC TCTTAAACTC AACCTCATAT TTTCCTGCCT GTGTAACTGG ATAGGGTCTA   
  
  
- AGTACTCTAA GTGCAAATGG TATCTCCAAA TATCTATTTC TCTTTCATTT TTTTTCTTTT TTGTTACTAA   
  
  
- TTTCTAACAT TGAAAGAGTC GTGGTAGGTC TGATAAGATT ATCTGTAGTG TGTTCAGTAT TATAGTCTTA   
  
  
- AACATCACAA TCAACAAATG CGTGTGTTTA CTATCCGTCG GTAGCTGACA ATGTTTTAAA TATTTTCAGA   
  
  
- GTGTGATTAT CTAATAATTT TCTAATAAAG CATGAACGCG CTGACTACCA TTGTTTTGAG CATCTATCTT   
  
  
- ATATGAAAGA AACTTCACCC TTACTAAACT GGATGAGATA TTTATTATAT TTGTAGTGTA CGTATGTGAG   
  
  
- GTTGGTGATT CGGTGTCTTT CTTTACTTTC TTTGTCTTTT ATTAATTGTT GGGATAACCT GAGAAGAGAT   
  
  
- GTGACGGTCG GAAAGGAAGA AACAAGACTC GTTTCTTTGT GTGGTTTTAA CAGTCGGTAT TTTAAAGAAG   
  
  
- GAGTGTCTTT GAGCAAACCA ATGACTCTT

+     G-box

| Site Name | Organism | Position | Strand | Matrix score. | sequence | function |
| --- | --- | --- | --- | --- | --- | --- |
| G-box | Brassica napus | 920 | - | 8 | CCACGTAA | cis-acting regulatory element involved in light responsiveness |
| G-box | Brassica oleracea | 991 | - | 8 | TAAACGTG | cis-acting regulatory element involved in light responsiveness |
| G-box | Arabidopsis thaliana | 921 | + | 6 | TACGTG | cis-acting regulatory element involved in light responsiveness |

>038667   
+ ACGAAAACCG AAGCAATAAC ATTCTATTTA TAGGAGAGGA AAGTCGGCAC AGAGGAAGGT GAGATTAGAC   
  
  
+ CAAGCAAACT TACACCGCCT AAAAGCTATT GATAAGAATG ATGTAAAAGC CCTTTAATTG GCTATGGTTT   
  
  
+ TGGTCCAATT AGAATAGAAC AAACAACTTA ATAAAATAAT GTCAAAAGGA AAGAAAATGA ATAATATGAA   
  
  
+ TATATAGTTC AAATACTACT GTAATGGTGT ACTGAACTTA TTTGGTTCAA AATAGTAGTA ATTAGAGTAT   
  
  
+ AGAAAAATGA AATAAAAGAA TTAACGATTA AATAAGAAAT AATACAAAAC GATATGAAAT TAATAAGTAT   
  
  
+ ATACAACACA TAATTATATA TAATTATTTT GGAAAATATA GGTCCTCACA AGACGCCTAA GTGCTAGCAC   
  
  
+ CTACTTGCTG CTGGCTAGCG CTTAGGTCCT GCTTTTTCTT ATATTATACT CTAAATTATT GTAACTTGGG   
  
  
+ TATAGAAGTG CAGGCAAACT AACCTATCCC ATATTCACCA GATTCACGTT TGCTTTGAAA AGAAGTTTAT   
  
  
+ AGATGAAGAA AATGTTAAAA AGAAAAAAGA GTGATTAGAG ATAACTTGCT TGGATGTGGA TTTTGCTCGG   
  
  
+ GGTAAAGAAG GGCGGGCACA CTGACCTATC CCAGATATAC TATTTTCACA TTTTCTTTGA AAAGAGGTTT   
  
  
+ ACATATAAAG AAAATGTGTA AAAAAAAATA AAAAATAAAA TAAAATAAAG TGATTAAAGA TAACTTTGGA   
  
  
+ TGAGGATTTG GCTCGGGGTA TAGAAGAGTG GATACTTTGA CCTATCTCAA ATTCACCAGA CTCATGTTTG   
  
  
+ CTTTGAAAAG AGGTTTATAG ATAAAGAAAA CGTAAAAAAA AAAAAAATAA ATAAAAAAAA ATGAGTGACT   
  
  
+ AAAGATAACT TACGTGGATG AGAATTTGAG TTGGAGTATA AAAGGACGGA CACATTGACC TATCCCAGAT   
  
  
+ TCATGAGATT CACGTTTACC ATAGAGGTTT ATAGATAAAG AGAAAGTAAA AAAAAGAAAA AACAATGATT   
  
  
+ AAAGATTGTA ACTTTCTCAG CACCATCCAG ACTATTCTAA TAGACATCAC ACAAGTCATA ATATCAGAAT   
  
  
+ TTGTAGTGTT AGTTGTTTAC GCACACAAAT GATAGGCAGC CATCGACTGT TACAAAATTT ATAAAAGTCT   
  
  
+ CACACTAATA GATTATTAAA AGATTATTTC GTACTTGCGC GACTGATGGT AACAAAACTC GTAGATAGAA   
  
  
+ TATACTTTCT TTGAAGTGGG AATGATTTGA CCTACTCTAT AAATAATATA AACATCACAT GCATACACTC   
  
  
+ CAACCACTAA GCCACAGAAA GAAATGAAAG AAACAGAAAA TAATTAACAA CCCTATTGGA CTCTTCTCTA   
  
  
+ CACTGCCAGC CTTTCCTTCT TTGTTCTGAG CAAAGAAACA CACCAAAATT GTCAGCCATA AAATTTCTTC   
  
  
+ CTCACAGAAA CTCGTTTGGT TACTGAGAA  

- TGCTTTTGGC TTCGTTATTG TAAGATAAAT ATCCTCTCCT TTCAGCCGTG TCTCCTTCCA CTCTAATCTG   
  
  
- GTTCGTTTGA ATGTGGCGGA TTTTCGATAA CTATTCTTAC TACATTTTCG GGAAATTAAC CGATACCAAA   
  
  
- ACCAGGTTAA TCTTATCTTG TTTGTTGAAT TATTTTATTA CAGTTTTCCT TTCTTTTACT TATTATACTT   
  
  
- ATATATCAAG TTTATGATGA CATTACCACA TGACTTGAAT AAACCAAGTT TTATCATCAT TAATCTCATA   
  
  
- TCTTTTTACT TTATTTTCTT AATTGCTAAT TTATTCTTTA TTATGTTTTG CTATACTTTA ATTATTCATA   
  
  
- TATGTTGTGT ATTAATATAT ATTAATAAAA CCTTTTATAT CCAGGAGTGT TCTGCGGATT CACGATCGTG   
  
  
- GATGAACGAC GACCGATCGC GAATCCAGGA CGAAAAAGAA TATAATATGA GATTTAATAA CATTGAACCC   
  
  
- ATATCTTCAC GTCCGTTTGA TTGGATAGGG TATAAGTGGT CTAAGTGCAA ACGAAACTTT TCTTCAAATA   
  
  
- TCTACTTCTT TTACAATTTT TCTTTTTTCT CACTAATCTC TATTGAACGA ACCTACACCT AAAACGAGCC   
  
  
- CCATTTCTTC CCGCCCGTGT GACTGGATAG GGTCTATATG ATAAAAGTGT AAAAGAAACT TTTCTCCAAA   
  
  
- TGTATATTTC TTTTACACAT TTTTTTTTAT TTTTTATTTT ATTTTATTTC ACTAATTTCT ATTGAAACCT   
  
  
- ACTCCTAAAC CGAGCCCCAT ATCTTCTCAC CTATGAAACT GGATAGAGTT TAAGTGGTCT GAGTACAAAC   
  
  
- GAAACTTTTC TCCAAATATC TATTTCTTTT GCATTTTTTT TTTTTTTATT TATTTTTTTT TACTCACTGA   
  
  
- TTTCTATTGA ATGCACCTAC TCTTAAACTC AACCTCATAT TTTCCTGCCT GTGTAACTGG ATAGGGTCTA   
  
  
- AGTACTCTAA GTGCAAATGG TATCTCCAAA TATCTATTTC TCTTTCATTT TTTTTCTTTT TTGTTACTAA   
  
  
- TTTCTAACAT TGAAAGAGTC GTGGTAGGTC TGATAAGATT ATCTGTAGTG TGTTCAGTAT TATAGTCTTA   
  
  
- AACATCACAA TCAACAAATG CGTGTGTTTA CTATCCGTCG GTAGCTGACA ATGTTTTAAA TATTTTCAGA   
  
  
- GTGTGATTAT CTAATAATTT TCTAATAAAG CATGAACGCG CTGACTACCA TTGTTTTGAG CATCTATCTT   
  
  
- ATATGAAAGA AACTTCACCC TTACTAAACT GGATGAGATA TTTATTATAT TTGTAGTGTA CGTATGTGAG   
  
  
- GTTGGTGATT CGGTGTCTTT CTTTACTTTC TTTGTCTTTT ATTAATTGTT GGGATAACCT GAGAAGAGAT   
  
  
- GTGACGGTCG GAAAGGAAGA AACAAGACTC GTTTCTTTGT GTGGTTTTAA CAGTCGGTAT TTTAAAGAAG   
  
  
- GAGTGTCTTT GAGCAAACCA ATGACTCTT

+     GA-motif

| Site Name | Organism | Position | Strand | Matrix score. | sequence | function |
| --- | --- | --- | --- | --- | --- | --- |
| GA-motif | Arabidopsis thaliana | 1011 | + | 8 | ATAGATAA | part of a light responsive element |
| GA-motif | Arabidopsis thaliana | 857 | + | 8 | ATAGATAA | part of a light responsive element |

>038667   
+ ACGAAAACCG AAGCAATAAC ATTCTATTTA TAGGAGAGGA AAGTCGGCAC AGAGGAAGGT GAGATTAGAC   
  
  
+ CAAGCAAACT TACACCGCCT AAAAGCTATT GATAAGAATG ATGTAAAAGC CCTTTAATTG GCTATGGTTT   
  
  
+ TGGTCCAATT AGAATAGAAC AAACAACTTA ATAAAATAAT GTCAAAAGGA AAGAAAATGA ATAATATGAA   
  
  
+ TATATAGTTC AAATACTACT GTAATGGTGT ACTGAACTTA TTTGGTTCAA AATAGTAGTA ATTAGAGTAT   
  
  
+ AGAAAAATGA AATAAAAGAA TTAACGATTA AATAAGAAAT AATACAAAAC GATATGAAAT TAATAAGTAT   
  
  
+ ATACAACACA TAATTATATA TAATTATTTT GGAAAATATA GGTCCTCACA AGACGCCTAA GTGCTAGCAC   
  
  
+ CTACTTGCTG CTGGCTAGCG CTTAGGTCCT GCTTTTTCTT ATATTATACT CTAAATTATT GTAACTTGGG   
  
  
+ TATAGAAGTG CAGGCAAACT AACCTATCCC ATATTCACCA GATTCACGTT TGCTTTGAAA AGAAGTTTAT   
  
  
+ AGATGAAGAA AATGTTAAAA AGAAAAAAGA GTGATTAGAG ATAACTTGCT TGGATGTGGA TTTTGCTCGG   
  
  
+ GGTAAAGAAG GGCGGGCACA CTGACCTATC CCAGATATAC TATTTTCACA TTTTCTTTGA AAAGAGGTTT   
  
  
+ ACATATAAAG AAAATGTGTA AAAAAAAATA AAAAATAAAA TAAAATAAAG TGATTAAAGA TAACTTTGGA   
  
  
+ TGAGGATTTG GCTCGGGGTA TAGAAGAGTG GATACTTTGA CCTATCTCAA ATTCACCAGA CTCATGTTTG   
  
  
+ CTTTGAAAAG AGGTTTATAG ATAAAGAAAA CGTAAAAAAA AAAAAAATAA ATAAAAAAAA ATGAGTGACT   
  
  
+ AAAGATAACT TACGTGGATG AGAATTTGAG TTGGAGTATA AAAGGACGGA CACATTGACC TATCCCAGAT   
  
  
+ TCATGAGATT CACGTTTACC ATAGAGGTTT ATAGATAAAG AGAAAGTAAA AAAAAGAAAA AACAATGATT   
  
  
+ AAAGATTGTA ACTTTCTCAG CACCATCCAG ACTATTCTAA TAGACATCAC ACAAGTCATA ATATCAGAAT   
  
  
+ TTGTAGTGTT AGTTGTTTAC GCACACAAAT GATAGGCAGC CATCGACTGT TACAAAATTT ATAAAAGTCT   
  
  
+ CACACTAATA GATTATTAAA AGATTATTTC GTACTTGCGC GACTGATGGT AACAAAACTC GTAGATAGAA   
  
  
+ TATACTTTCT TTGAAGTGGG AATGATTTGA CCTACTCTAT AAATAATATA AACATCACAT GCATACACTC   
  
  
+ CAACCACTAA GCCACAGAAA GAAATGAAAG AAACAGAAAA TAATTAACAA CCCTATTGGA CTCTTCTCTA   
  
  
+ CACTGCCAGC CTTTCCTTCT TTGTTCTGAG CAAAGAAACA CACCAAAATT GTCAGCCATA AAATTTCTTC   
  
  
+ CTCACAGAAA CTCGTTTGGT TACTGAGAA  

- TGCTTTTGGC TTCGTTATTG TAAGATAAAT ATCCTCTCCT TTCAGCCGTG TCTCCTTCCA CTCTAATCTG   
  
  
- GTTCGTTTGA ATGTGGCGGA TTTTCGATAA CTATTCTTAC TACATTTTCG GGAAATTAAC CGATACCAAA   
  
  
- ACCAGGTTAA TCTTATCTTG TTTGTTGAAT TATTTTATTA CAGTTTTCCT TTCTTTTACT TATTATACTT   
  
  
- ATATATCAAG TTTATGATGA CATTACCACA TGACTTGAAT AAACCAAGTT TTATCATCAT TAATCTCATA   
  
  
- TCTTTTTACT TTATTTTCTT AATTGCTAAT TTATTCTTTA TTATGTTTTG CTATACTTTA ATTATTCATA   
  
  
- TATGTTGTGT ATTAATATAT ATTAATAAAA CCTTTTATAT CCAGGAGTGT TCTGCGGATT CACGATCGTG   
  
  
- GATGAACGAC GACCGATCGC GAATCCAGGA CGAAAAAGAA TATAATATGA GATTTAATAA CATTGAACCC   
  
  
- ATATCTTCAC GTCCGTTTGA TTGGATAGGG TATAAGTGGT CTAAGTGCAA ACGAAACTTT TCTTCAAATA   
  
  
- TCTACTTCTT TTACAATTTT TCTTTTTTCT CACTAATCTC TATTGAACGA ACCTACACCT AAAACGAGCC   
  
  
- CCATTTCTTC CCGCCCGTGT GACTGGATAG GGTCTATATG ATAAAAGTGT AAAAGAAACT TTTCTCCAAA   
  
  
- TGTATATTTC TTTTACACAT TTTTTTTTAT TTTTTATTTT ATTTTATTTC ACTAATTTCT ATTGAAACCT   
  
  
- ACTCCTAAAC CGAGCCCCAT ATCTTCTCAC CTATGAAACT GGATAGAGTT TAAGTGGTCT GAGTACAAAC   
  
  
- GAAACTTTTC TCCAAATATC TATTTCTTTT GCATTTTTTT TTTTTTTATT TATTTTTTTT TACTCACTGA   
  
  
- TTTCTATTGA ATGCACCTAC TCTTAAACTC AACCTCATAT TTTCCTGCCT GTGTAACTGG ATAGGGTCTA   
  
  
- AGTACTCTAA GTGCAAATGG TATCTCCAAA TATCTATTTC TCTTTCATTT TTTTTCTTTT TTGTTACTAA   
  
  
- TTTCTAACAT TGAAAGAGTC GTGGTAGGTC TGATAAGATT ATCTGTAGTG TGTTCAGTAT TATAGTCTTA   
  
  
- AACATCACAA TCAACAAATG CGTGTGTTTA CTATCCGTCG GTAGCTGACA ATGTTTTAAA TATTTTCAGA   
  
  
- GTGTGATTAT CTAATAATTT TCTAATAAAG CATGAACGCG CTGACTACCA TTGTTTTGAG CATCTATCTT   
  
  
- ATATGAAAGA AACTTCACCC TTACTAAACT GGATGAGATA TTTATTATAT TTGTAGTGTA CGTATGTGAG   
  
  
- GTTGGTGATT CGGTGTCTTT CTTTACTTTC TTTGTCTTTT ATTAATTGTT GGGATAACCT GAGAAGAGAT   
  
  
- GTGACGGTCG GAAAGGAAGA AACAAGACTC GTTTCTTTGT GTGGTTTTAA CAGTCGGTAT TTTAAAGAAG   
  
  
- GAGTGTCTTT GAGCAAACCA ATGACTCTT

+     I-box

| Site Name | Organism | Position | Strand | Matrix score. | sequence | function |
| --- | --- | --- | --- | --- | --- | --- |
| I-box | Flaveria trinervia | 127 | - | 10 | cCATATCCAAT | part of a light responsive element |

>038667   
+ ACGAAAACCG AAGCAATAAC ATTCTATTTA TAGGAGAGGA AAGTCGGCAC AGAGGAAGGT GAGATTAGAC   
  
  
+ CAAGCAAACT TACACCGCCT AAAAGCTATT GATAAGAATG ATGTAAAAGC CCTTTAATTG GCTATGGTTT   
  
  
+ TGGTCCAATT AGAATAGAAC AAACAACTTA ATAAAATAAT GTCAAAAGGA AAGAAAATGA ATAATATGAA   
  
  
+ TATATAGTTC AAATACTACT GTAATGGTGT ACTGAACTTA TTTGGTTCAA AATAGTAGTA ATTAGAGTAT   
  
  
+ AGAAAAATGA AATAAAAGAA TTAACGATTA AATAAGAAAT AATACAAAAC GATATGAAAT TAATAAGTAT   
  
  
+ ATACAACACA TAATTATATA TAATTATTTT GGAAAATATA GGTCCTCACA AGACGCCTAA GTGCTAGCAC   
  
  
+ CTACTTGCTG CTGGCTAGCG CTTAGGTCCT GCTTTTTCTT ATATTATACT CTAAATTATT GTAACTTGGG   
  
  
+ TATAGAAGTG CAGGCAAACT AACCTATCCC ATATTCACCA GATTCACGTT TGCTTTGAAA AGAAGTTTAT   
  
  
+ AGATGAAGAA AATGTTAAAA AGAAAAAAGA GTGATTAGAG ATAACTTGCT TGGATGTGGA TTTTGCTCGG   
  
  
+ GGTAAAGAAG GGCGGGCACA CTGACCTATC CCAGATATAC TATTTTCACA TTTTCTTTGA AAAGAGGTTT   
  
  
+ ACATATAAAG AAAATGTGTA AAAAAAAATA AAAAATAAAA TAAAATAAAG TGATTAAAGA TAACTTTGGA   
  
  
+ TGAGGATTTG GCTCGGGGTA TAGAAGAGTG GATACTTTGA CCTATCTCAA ATTCACCAGA CTCATGTTTG   
  
  
+ CTTTGAAAAG AGGTTTATAG ATAAAGAAAA CGTAAAAAAA AAAAAAATAA ATAAAAAAAA ATGAGTGACT   
  
  
+ AAAGATAACT TACGTGGATG AGAATTTGAG TTGGAGTATA AAAGGACGGA CACATTGACC TATCCCAGAT   
  
  
+ TCATGAGATT CACGTTTACC ATAGAGGTTT ATAGATAAAG AGAAAGTAAA AAAAAGAAAA AACAATGATT   
  
  
+ AAAGATTGTA ACTTTCTCAG CACCATCCAG ACTATTCTAA TAGACATCAC ACAAGTCATA ATATCAGAAT   
  
  
+ TTGTAGTGTT AGTTGTTTAC GCACACAAAT GATAGGCAGC CATCGACTGT TACAAAATTT ATAAAAGTCT   
  
  
+ CACACTAATA GATTATTAAA AGATTATTTC GTACTTGCGC GACTGATGGT AACAAAACTC GTAGATAGAA   
  
  
+ TATACTTTCT TTGAAGTGGG AATGATTTGA CCTACTCTAT AAATAATATA AACATCACAT GCATACACTC   
  
  
+ CAACCACTAA GCCACAGAAA GAAATGAAAG AAACAGAAAA TAATTAACAA CCCTATTGGA CTCTTCTCTA   
  
  
+ CACTGCCAGC CTTTCCTTCT TTGTTCTGAG CAAAGAAACA CACCAAAATT GTCAGCCATA AAATTTCTTC   
  
  
+ CTCACAGAAA CTCGTTTGGT TACTGAGAA  

- TGCTTTTGGC TTCGTTATTG TAAGATAAAT ATCCTCTCCT TTCAGCCGTG TCTCCTTCCA CTCTAATCTG   
  
  
- GTTCGTTTGA ATGTGGCGGA TTTTCGATAA CTATTCTTAC TACATTTTCG GGAAATTAAC CGATACCAAA   
  
  
- ACCAGGTTAA TCTTATCTTG TTTGTTGAAT TATTTTATTA CAGTTTTCCT TTCTTTTACT TATTATACTT   
  
  
- ATATATCAAG TTTATGATGA CATTACCACA TGACTTGAAT AAACCAAGTT TTATCATCAT TAATCTCATA   
  
  
- TCTTTTTACT TTATTTTCTT AATTGCTAAT TTATTCTTTA TTATGTTTTG CTATACTTTA ATTATTCATA   
  
  
- TATGTTGTGT ATTAATATAT ATTAATAAAA CCTTTTATAT CCAGGAGTGT TCTGCGGATT CACGATCGTG   
  
  
- GATGAACGAC GACCGATCGC GAATCCAGGA CGAAAAAGAA TATAATATGA GATTTAATAA CATTGAACCC   
  
  
- ATATCTTCAC GTCCGTTTGA TTGGATAGGG TATAAGTGGT CTAAGTGCAA ACGAAACTTT TCTTCAAATA   
  
  
- TCTACTTCTT TTACAATTTT TCTTTTTTCT CACTAATCTC TATTGAACGA ACCTACACCT AAAACGAGCC   
  
  
- CCATTTCTTC CCGCCCGTGT GACTGGATAG GGTCTATATG ATAAAAGTGT AAAAGAAACT TTTCTCCAAA   
  
  
- TGTATATTTC TTTTACACAT TTTTTTTTAT TTTTTATTTT ATTTTATTTC ACTAATTTCT ATTGAAACCT   
  
  
- ACTCCTAAAC CGAGCCCCAT ATCTTCTCAC CTATGAAACT GGATAGAGTT TAAGTGGTCT GAGTACAAAC   
  
  
- GAAACTTTTC TCCAAATATC TATTTCTTTT GCATTTTTTT TTTTTTTATT TATTTTTTTT TACTCACTGA   
  
  
- TTTCTATTGA ATGCACCTAC TCTTAAACTC AACCTCATAT TTTCCTGCCT GTGTAACTGG ATAGGGTCTA   
  
  
- AGTACTCTAA GTGCAAATGG TATCTCCAAA TATCTATTTC TCTTTCATTT TTTTTCTTTT TTGTTACTAA   
  
  
- TTTCTAACAT TGAAAGAGTC GTGGTAGGTC TGATAAGATT ATCTGTAGTG TGTTCAGTAT TATAGTCTTA   
  
  
- AACATCACAA TCAACAAATG CGTGTGTTTA CTATCCGTCG GTAGCTGACA ATGTTTTAAA TATTTTCAGA   
  
  
- GTGTGATTAT CTAATAATTT TCTAATAAAG CATGAACGCG CTGACTACCA TTGTTTTGAG CATCTATCTT   
  
  
- ATATGAAAGA AACTTCACCC TTACTAAACT GGATGAGATA TTTATTATAT TTGTAGTGTA CGTATGTGAG   
  
  
- GTTGGTGATT CGGTGTCTTT CTTTACTTTC TTTGTCTTTT ATTAATTGTT GGGATAACCT GAGAAGAGAT   
  
  
- GTGACGGTCG GAAAGGAAGA AACAAGACTC GTTTCTTTGT GTGGTTTTAA CAGTCGGTAT TTTAAAGAAG   
  
  
- GAGTGTCTTT GAGCAAACCA ATGACTCTT

+     MYB

| Site Name | Organism | Position | Strand | Matrix score. | sequence | function |
| --- | --- | --- | --- | --- | --- | --- |
| MYB | Arabidopsis thaliana | 1331 | + | 6 | CAACCA |  |
| MYB | Arabidopsis thaliana | 1487 | - | 6 | TAACCA |  |

>038667   
+ ACGAAAACCG AAGCAATAAC ATTCTATTTA TAGGAGAGGA AAGTCGGCAC AGAGGAAGGT GAGATTAGAC   
  
  
+ CAAGCAAACT TACACCGCCT AAAAGCTATT GATAAGAATG ATGTAAAAGC CCTTTAATTG GCTATGGTTT   
  
  
+ TGGTCCAATT AGAATAGAAC AAACAACTTA ATAAAATAAT GTCAAAAGGA AAGAAAATGA ATAATATGAA   
  
  
+ TATATAGTTC AAATACTACT GTAATGGTGT ACTGAACTTA TTTGGTTCAA AATAGTAGTA ATTAGAGTAT   
  
  
+ AGAAAAATGA AATAAAAGAA TTAACGATTA AATAAGAAAT AATACAAAAC GATATGAAAT TAATAAGTAT   
  
  
+ ATACAACACA TAATTATATA TAATTATTTT GGAAAATATA GGTCCTCACA AGACGCCTAA GTGCTAGCAC   
  
  
+ CTACTTGCTG CTGGCTAGCG CTTAGGTCCT GCTTTTTCTT ATATTATACT CTAAATTATT GTAACTTGGG   
  
  
+ TATAGAAGTG CAGGCAAACT AACCTATCCC ATATTCACCA GATTCACGTT TGCTTTGAAA AGAAGTTTAT   
  
  
+ AGATGAAGAA AATGTTAAAA AGAAAAAAGA GTGATTAGAG ATAACTTGCT TGGATGTGGA TTTTGCTCGG   
  
  
+ GGTAAAGAAG GGCGGGCACA CTGACCTATC CCAGATATAC TATTTTCACA TTTTCTTTGA AAAGAGGTTT   
  
  
+ ACATATAAAG AAAATGTGTA AAAAAAAATA AAAAATAAAA TAAAATAAAG TGATTAAAGA TAACTTTGGA   
  
  
+ TGAGGATTTG GCTCGGGGTA TAGAAGAGTG GATACTTTGA CCTATCTCAA ATTCACCAGA CTCATGTTTG   
  
  
+ CTTTGAAAAG AGGTTTATAG ATAAAGAAAA CGTAAAAAAA AAAAAAATAA ATAAAAAAAA ATGAGTGACT   
  
  
+ AAAGATAACT TACGTGGATG AGAATTTGAG TTGGAGTATA AAAGGACGGA CACATTGACC TATCCCAGAT   
  
  
+ TCATGAGATT CACGTTTACC ATAGAGGTTT ATAGATAAAG AGAAAGTAAA AAAAAGAAAA AACAATGATT   
  
  
+ AAAGATTGTA ACTTTCTCAG CACCATCCAG ACTATTCTAA TAGACATCAC ACAAGTCATA ATATCAGAAT   
  
  
+ TTGTAGTGTT AGTTGTTTAC GCACACAAAT GATAGGCAGC CATCGACTGT TACAAAATTT ATAAAAGTCT   
  
  
+ CACACTAATA GATTATTAAA AGATTATTTC GTACTTGCGC GACTGATGGT AACAAAACTC GTAGATAGAA   
  
  
+ TATACTTTCT TTGAAGTGGG AATGATTTGA CCTACTCTAT AAATAATATA AACATCACAT GCATACACTC   
  
  
+ CAACCACTAA GCCACAGAAA GAAATGAAAG AAACAGAAAA TAATTAACAA CCCTATTGGA CTCTTCTCTA   
  
  
+ CACTGCCAGC CTTTCCTTCT TTGTTCTGAG CAAAGAAACA CACCAAAATT GTCAGCCATA AAATTTCTTC   
  
  
+ CTCACAGAAA CTCGTTTGGT TACTGAGAA  

- TGCTTTTGGC TTCGTTATTG TAAGATAAAT ATCCTCTCCT TTCAGCCGTG TCTCCTTCCA CTCTAATCTG   
  
  
- GTTCGTTTGA ATGTGGCGGA TTTTCGATAA CTATTCTTAC TACATTTTCG GGAAATTAAC CGATACCAAA   
  
  
- ACCAGGTTAA TCTTATCTTG TTTGTTGAAT TATTTTATTA CAGTTTTCCT TTCTTTTACT TATTATACTT   
  
  
- ATATATCAAG TTTATGATGA CATTACCACA TGACTTGAAT AAACCAAGTT TTATCATCAT TAATCTCATA   
  
  
- TCTTTTTACT TTATTTTCTT AATTGCTAAT TTATTCTTTA TTATGTTTTG CTATACTTTA ATTATTCATA   
  
  
- TATGTTGTGT ATTAATATAT ATTAATAAAA CCTTTTATAT CCAGGAGTGT TCTGCGGATT CACGATCGTG   
  
  
- GATGAACGAC GACCGATCGC GAATCCAGGA CGAAAAAGAA TATAATATGA GATTTAATAA CATTGAACCC   
  
  
- ATATCTTCAC GTCCGTTTGA TTGGATAGGG TATAAGTGGT CTAAGTGCAA ACGAAACTTT TCTTCAAATA   
  
  
- TCTACTTCTT TTACAATTTT TCTTTTTTCT CACTAATCTC TATTGAACGA ACCTACACCT AAAACGAGCC   
  
  
- CCATTTCTTC CCGCCCGTGT GACTGGATAG GGTCTATATG ATAAAAGTGT AAAAGAAACT TTTCTCCAAA   
  
  
- TGTATATTTC TTTTACACAT TTTTTTTTAT TTTTTATTTT ATTTTATTTC ACTAATTTCT ATTGAAACCT   
  
  
- ACTCCTAAAC CGAGCCCCAT ATCTTCTCAC CTATGAAACT GGATAGAGTT TAAGTGGTCT GAGTACAAAC   
  
  
- GAAACTTTTC TCCAAATATC TATTTCTTTT GCATTTTTTT TTTTTTTATT TATTTTTTTT TACTCACTGA   
  
  
- TTTCTATTGA ATGCACCTAC TCTTAAACTC AACCTCATAT TTTCCTGCCT GTGTAACTGG ATAGGGTCTA   
  
  
- AGTACTCTAA GTGCAAATGG TATCTCCAAA TATCTATTTC TCTTTCATTT TTTTTCTTTT TTGTTACTAA   
  
  
- TTTCTAACAT TGAAAGAGTC GTGGTAGGTC TGATAAGATT ATCTGTAGTG TGTTCAGTAT TATAGTCTTA   
  
  
- AACATCACAA TCAACAAATG CGTGTGTTTA CTATCCGTCG GTAGCTGACA ATGTTTTAAA TATTTTCAGA   
  
  
- GTGTGATTAT CTAATAATTT TCTAATAAAG CATGAACGCG CTGACTACCA TTGTTTTGAG CATCTATCTT   
  
  
- ATATGAAAGA AACTTCACCC TTACTAAACT GGATGAGATA TTTATTATAT TTGTAGTGTA CGTATGTGAG   
  
  
- GTTGGTGATT CGGTGTCTTT CTTTACTTTC TTTGTCTTTT ATTAATTGTT GGGATAACCT GAGAAGAGAT   
  
  
- GTGACGGTCG GAAAGGAAGA AACAAGACTC GTTTCTTTGT GTGGTTTTAA CAGTCGGTAT TTTAAAGAAG   
  
  
- GAGTGTCTTT GAGCAAACCA ATGACTCTT

+     MYB-like sequence

| Site Name | Organism | Position | Strand | Matrix score. | sequence | function |
| --- | --- | --- | --- | --- | --- | --- |
| MYB-like sequence | Arabidopsis thaliana | 1487 | - | 6 | TAACCA |  |

>038667   
+ ACGAAAACCG AAGCAATAAC ATTCTATTTA TAGGAGAGGA AAGTCGGCAC AGAGGAAGGT GAGATTAGAC   
  
  
+ CAAGCAAACT TACACCGCCT AAAAGCTATT GATAAGAATG ATGTAAAAGC CCTTTAATTG GCTATGGTTT   
  
  
+ TGGTCCAATT AGAATAGAAC AAACAACTTA ATAAAATAAT GTCAAAAGGA AAGAAAATGA ATAATATGAA   
  
  
+ TATATAGTTC AAATACTACT GTAATGGTGT ACTGAACTTA TTTGGTTCAA AATAGTAGTA ATTAGAGTAT   
  
  
+ AGAAAAATGA AATAAAAGAA TTAACGATTA AATAAGAAAT AATACAAAAC GATATGAAAT TAATAAGTAT   
  
  
+ ATACAACACA TAATTATATA TAATTATTTT GGAAAATATA GGTCCTCACA AGACGCCTAA GTGCTAGCAC   
  
  
+ CTACTTGCTG CTGGCTAGCG CTTAGGTCCT GCTTTTTCTT ATATTATACT CTAAATTATT GTAACTTGGG   
  
  
+ TATAGAAGTG CAGGCAAACT AACCTATCCC ATATTCACCA GATTCACGTT TGCTTTGAAA AGAAGTTTAT   
  
  
+ AGATGAAGAA AATGTTAAAA AGAAAAAAGA GTGATTAGAG ATAACTTGCT TGGATGTGGA TTTTGCTCGG   
  
  
+ GGTAAAGAAG GGCGGGCACA CTGACCTATC CCAGATATAC TATTTTCACA TTTTCTTTGA AAAGAGGTTT   
  
  
+ ACATATAAAG AAAATGTGTA AAAAAAAATA AAAAATAAAA TAAAATAAAG TGATTAAAGA TAACTTTGGA   
  
  
+ TGAGGATTTG GCTCGGGGTA TAGAAGAGTG GATACTTTGA CCTATCTCAA ATTCACCAGA CTCATGTTTG   
  
  
+ CTTTGAAAAG AGGTTTATAG ATAAAGAAAA CGTAAAAAAA AAAAAAATAA ATAAAAAAAA ATGAGTGACT   
  
  
+ AAAGATAACT TACGTGGATG AGAATTTGAG TTGGAGTATA AAAGGACGGA CACATTGACC TATCCCAGAT   
  
  
+ TCATGAGATT CACGTTTACC ATAGAGGTTT ATAGATAAAG AGAAAGTAAA AAAAAGAAAA AACAATGATT   
  
  
+ AAAGATTGTA ACTTTCTCAG CACCATCCAG ACTATTCTAA TAGACATCAC ACAAGTCATA ATATCAGAAT   
  
  
+ TTGTAGTGTT AGTTGTTTAC GCACACAAAT GATAGGCAGC CATCGACTGT TACAAAATTT ATAAAAGTCT   
  
  
+ CACACTAATA GATTATTAAA AGATTATTTC GTACTTGCGC GACTGATGGT AACAAAACTC GTAGATAGAA   
  
  
+ TATACTTTCT TTGAAGTGGG AATGATTTGA CCTACTCTAT AAATAATATA AACATCACAT GCATACACTC   
  
  
+ CAACCACTAA GCCACAGAAA GAAATGAAAG AAACAGAAAA TAATTAACAA CCCTATTGGA CTCTTCTCTA   
  
  
+ CACTGCCAGC CTTTCCTTCT TTGTTCTGAG CAAAGAAACA CACCAAAATT GTCAGCCATA AAATTTCTTC   
  
  
+ CTCACAGAAA CTCGTTTGGT TACTGAGAA  

- TGCTTTTGGC TTCGTTATTG TAAGATAAAT ATCCTCTCCT TTCAGCCGTG TCTCCTTCCA CTCTAATCTG   
  
  
- GTTCGTTTGA ATGTGGCGGA TTTTCGATAA CTATTCTTAC TACATTTTCG GGAAATTAAC CGATACCAAA   
  
  
- ACCAGGTTAA TCTTATCTTG TTTGTTGAAT TATTTTATTA CAGTTTTCCT TTCTTTTACT TATTATACTT   
  
  
- ATATATCAAG TTTATGATGA CATTACCACA TGACTTGAAT AAACCAAGTT TTATCATCAT TAATCTCATA   
  
  
- TCTTTTTACT TTATTTTCTT AATTGCTAAT TTATTCTTTA TTATGTTTTG CTATACTTTA ATTATTCATA   
  
  
- TATGTTGTGT ATTAATATAT ATTAATAAAA CCTTTTATAT CCAGGAGTGT TCTGCGGATT CACGATCGTG   
  
  
- GATGAACGAC GACCGATCGC GAATCCAGGA CGAAAAAGAA TATAATATGA GATTTAATAA CATTGAACCC   
  
  
- ATATCTTCAC GTCCGTTTGA TTGGATAGGG TATAAGTGGT CTAAGTGCAA ACGAAACTTT TCTTCAAATA   
  
  
- TCTACTTCTT TTACAATTTT TCTTTTTTCT CACTAATCTC TATTGAACGA ACCTACACCT AAAACGAGCC   
  
  
- CCATTTCTTC CCGCCCGTGT GACTGGATAG GGTCTATATG ATAAAAGTGT AAAAGAAACT TTTCTCCAAA   
  
  
- TGTATATTTC TTTTACACAT TTTTTTTTAT TTTTTATTTT ATTTTATTTC ACTAATTTCT ATTGAAACCT   
  
  
- ACTCCTAAAC CGAGCCCCAT ATCTTCTCAC CTATGAAACT GGATAGAGTT TAAGTGGTCT GAGTACAAAC   
  
  
- GAAACTTTTC TCCAAATATC TATTTCTTTT GCATTTTTTT TTTTTTTATT TATTTTTTTT TACTCACTGA   
  
  
- TTTCTATTGA ATGCACCTAC TCTTAAACTC AACCTCATAT TTTCCTGCCT GTGTAACTGG ATAGGGTCTA   
  
  
- AGTACTCTAA GTGCAAATGG TATCTCCAAA TATCTATTTC TCTTTCATTT TTTTTCTTTT TTGTTACTAA   
  
  
- TTTCTAACAT TGAAAGAGTC GTGGTAGGTC TGATAAGATT ATCTGTAGTG TGTTCAGTAT TATAGTCTTA   
  
  
- AACATCACAA TCAACAAATG CGTGTGTTTA CTATCCGTCG GTAGCTGACA ATGTTTTAAA TATTTTCAGA   
  
  
- GTGTGATTAT CTAATAATTT TCTAATAAAG CATGAACGCG CTGACTACCA TTGTTTTGAG CATCTATCTT   
  
  
- ATATGAAAGA AACTTCACCC TTACTAAACT GGATGAGATA TTTATTATAT TTGTAGTGTA CGTATGTGAG   
  
  
- GTTGGTGATT CGGTGTCTTT CTTTACTTTC TTTGTCTTTT ATTAATTGTT GGGATAACCT GAGAAGAGAT   
  
  
- GTGACGGTCG GAAAGGAAGA AACAAGACTC GTTTCTTTGT GTGGTTTTAA CAGTCGGTAT TTTAAAGAAG   
  
  
- GAGTGTCTTT GAGCAAACCA ATGACTCTT

+     MYC

| Site Name | Organism | Position | Strand | Matrix score. | sequence | function |
| --- | --- | --- | --- | --- | --- | --- |
| MYC | Arabidopsis thaliana | 1316 | - | 6 | CATGTG |  |
| MYC | Arabidopsis thaliana | 1146 | - | 6 | CATTTG |  |

>038667   
+ ACGAAAACCG AAGCAATAAC ATTCTATTTA TAGGAGAGGA AAGTCGGCAC AGAGGAAGGT GAGATTAGAC   
  
  
+ CAAGCAAACT TACACCGCCT AAAAGCTATT GATAAGAATG ATGTAAAAGC CCTTTAATTG GCTATGGTTT   
  
  
+ TGGTCCAATT AGAATAGAAC AAACAACTTA ATAAAATAAT GTCAAAAGGA AAGAAAATGA ATAATATGAA   
  
  
+ TATATAGTTC AAATACTACT GTAATGGTGT ACTGAACTTA TTTGGTTCAA AATAGTAGTA ATTAGAGTAT   
  
  
+ AGAAAAATGA AATAAAAGAA TTAACGATTA AATAAGAAAT AATACAAAAC GATATGAAAT TAATAAGTAT   
  
  
+ ATACAACACA TAATTATATA TAATTATTTT GGAAAATATA GGTCCTCACA AGACGCCTAA GTGCTAGCAC   
  
  
+ CTACTTGCTG CTGGCTAGCG CTTAGGTCCT GCTTTTTCTT ATATTATACT CTAAATTATT GTAACTTGGG   
  
  
+ TATAGAAGTG CAGGCAAACT AACCTATCCC ATATTCACCA GATTCACGTT TGCTTTGAAA AGAAGTTTAT   
  
  
+ AGATGAAGAA AATGTTAAAA AGAAAAAAGA GTGATTAGAG ATAACTTGCT TGGATGTGGA TTTTGCTCGG   
  
  
+ GGTAAAGAAG GGCGGGCACA CTGACCTATC CCAGATATAC TATTTTCACA TTTTCTTTGA AAAGAGGTTT   
  
  
+ ACATATAAAG AAAATGTGTA AAAAAAAATA AAAAATAAAA TAAAATAAAG TGATTAAAGA TAACTTTGGA   
  
  
+ TGAGGATTTG GCTCGGGGTA TAGAAGAGTG GATACTTTGA CCTATCTCAA ATTCACCAGA CTCATGTTTG   
  
  
+ CTTTGAAAAG AGGTTTATAG ATAAAGAAAA CGTAAAAAAA AAAAAAATAA ATAAAAAAAA ATGAGTGACT   
  
  
+ AAAGATAACT TACGTGGATG AGAATTTGAG TTGGAGTATA AAAGGACGGA CACATTGACC TATCCCAGAT   
  
  
+ TCATGAGATT CACGTTTACC ATAGAGGTTT ATAGATAAAG AGAAAGTAAA AAAAAGAAAA AACAATGATT   
  
  
+ AAAGATTGTA ACTTTCTCAG CACCATCCAG ACTATTCTAA TAGACATCAC ACAAGTCATA ATATCAGAAT   
  
  
+ TTGTAGTGTT AGTTGTTTAC GCACACAAAT GATAGGCAGC CATCGACTGT TACAAAATTT ATAAAAGTCT   
  
  
+ CACACTAATA GATTATTAAA AGATTATTTC GTACTTGCGC GACTGATGGT AACAAAACTC GTAGATAGAA   
  
  
+ TATACTTTCT TTGAAGTGGG AATGATTTGA CCTACTCTAT AAATAATATA AACATCACAT GCATACACTC   
  
  
+ CAACCACTAA GCCACAGAAA GAAATGAAAG AAACAGAAAA TAATTAACAA CCCTATTGGA CTCTTCTCTA   
  
  
+ CACTGCCAGC CTTTCCTTCT TTGTTCTGAG CAAAGAAACA CACCAAAATT GTCAGCCATA AAATTTCTTC   
  
  
+ CTCACAGAAA CTCGTTTGGT TACTGAGAA  

- TGCTTTTGGC TTCGTTATTG TAAGATAAAT ATCCTCTCCT TTCAGCCGTG TCTCCTTCCA CTCTAATCTG   
  
  
- GTTCGTTTGA ATGTGGCGGA TTTTCGATAA CTATTCTTAC TACATTTTCG GGAAATTAAC CGATACCAAA   
  
  
- ACCAGGTTAA TCTTATCTTG TTTGTTGAAT TATTTTATTA CAGTTTTCCT TTCTTTTACT TATTATACTT   
  
  
- ATATATCAAG TTTATGATGA CATTACCACA TGACTTGAAT AAACCAAGTT TTATCATCAT TAATCTCATA   
  
  
- TCTTTTTACT TTATTTTCTT AATTGCTAAT TTATTCTTTA TTATGTTTTG CTATACTTTA ATTATTCATA   
  
  
- TATGTTGTGT ATTAATATAT ATTAATAAAA CCTTTTATAT CCAGGAGTGT TCTGCGGATT CACGATCGTG   
  
  
- GATGAACGAC GACCGATCGC GAATCCAGGA CGAAAAAGAA TATAATATGA GATTTAATAA CATTGAACCC   
  
  
- ATATCTTCAC GTCCGTTTGA TTGGATAGGG TATAAGTGGT CTAAGTGCAA ACGAAACTTT TCTTCAAATA   
  
  
- TCTACTTCTT TTACAATTTT TCTTTTTTCT CACTAATCTC TATTGAACGA ACCTACACCT AAAACGAGCC   
  
  
- CCATTTCTTC CCGCCCGTGT GACTGGATAG GGTCTATATG ATAAAAGTGT AAAAGAAACT TTTCTCCAAA   
  
  
- TGTATATTTC TTTTACACAT TTTTTTTTAT TTTTTATTTT ATTTTATTTC ACTAATTTCT ATTGAAACCT   
  
  
- ACTCCTAAAC CGAGCCCCAT ATCTTCTCAC CTATGAAACT GGATAGAGTT TAAGTGGTCT GAGTACAAAC   
  
  
- GAAACTTTTC TCCAAATATC TATTTCTTTT GCATTTTTTT TTTTTTTATT TATTTTTTTT TACTCACTGA   
  
  
- TTTCTATTGA ATGCACCTAC TCTTAAACTC AACCTCATAT TTTCCTGCCT GTGTAACTGG ATAGGGTCTA   
  
  
- AGTACTCTAA GTGCAAATGG TATCTCCAAA TATCTATTTC TCTTTCATTT TTTTTCTTTT TTGTTACTAA   
  
  
- TTTCTAACAT TGAAAGAGTC GTGGTAGGTC TGATAAGATT ATCTGTAGTG TGTTCAGTAT TATAGTCTTA   
  
  
- AACATCACAA TCAACAAATG CGTGTGTTTA CTATCCGTCG GTAGCTGACA ATGTTTTAAA TATTTTCAGA   
  
  
- GTGTGATTAT CTAATAATTT TCTAATAAAG CATGAACGCG CTGACTACCA TTGTTTTGAG CATCTATCTT   
  
  
- ATATGAAAGA AACTTCACCC TTACTAAACT GGATGAGATA TTTATTATAT TTGTAGTGTA CGTATGTGAG   
  
  
- GTTGGTGATT CGGTGTCTTT CTTTACTTTC TTTGTCTTTT ATTAATTGTT GGGATAACCT GAGAAGAGAT   
  
  
- GTGACGGTCG GAAAGGAAGA AACAAGACTC GTTTCTTTGT GTGGTTTTAA CAGTCGGTAT TTTAAAGAAG   
  
  
- GAGTGTCTTT GAGCAAACCA ATGACTCTT

+     P-box

| Site Name | Organism | Position | Strand | Matrix score. | sequence | function |
| --- | --- | --- | --- | --- | --- | --- |
| P-box | Oryza sativa | 183 | - | 7 | CCTTTTG | gibberellin-responsive element |

>038667   
+ ACGAAAACCG AAGCAATAAC ATTCTATTTA TAGGAGAGGA AAGTCGGCAC AGAGGAAGGT GAGATTAGAC   
  
  
+ CAAGCAAACT TACACCGCCT AAAAGCTATT GATAAGAATG ATGTAAAAGC CCTTTAATTG GCTATGGTTT   
  
  
+ TGGTCCAATT AGAATAGAAC AAACAACTTA ATAAAATAAT GTCAAAAGGA AAGAAAATGA ATAATATGAA   
  
  
+ TATATAGTTC AAATACTACT GTAATGGTGT ACTGAACTTA TTTGGTTCAA AATAGTAGTA ATTAGAGTAT   
  
  
+ AGAAAAATGA AATAAAAGAA TTAACGATTA AATAAGAAAT AATACAAAAC GATATGAAAT TAATAAGTAT   
  
  
+ ATACAACACA TAATTATATA TAATTATTTT GGAAAATATA GGTCCTCACA AGACGCCTAA GTGCTAGCAC   
  
  
+ CTACTTGCTG CTGGCTAGCG CTTAGGTCCT GCTTTTTCTT ATATTATACT CTAAATTATT GTAACTTGGG   
  
  
+ TATAGAAGTG CAGGCAAACT AACCTATCCC ATATTCACCA GATTCACGTT TGCTTTGAAA AGAAGTTTAT   
  
  
+ AGATGAAGAA AATGTTAAAA AGAAAAAAGA GTGATTAGAG ATAACTTGCT TGGATGTGGA TTTTGCTCGG   
  
  
+ GGTAAAGAAG GGCGGGCACA CTGACCTATC CCAGATATAC TATTTTCACA TTTTCTTTGA AAAGAGGTTT   
  
  
+ ACATATAAAG AAAATGTGTA AAAAAAAATA AAAAATAAAA TAAAATAAAG TGATTAAAGA TAACTTTGGA   
  
  
+ TGAGGATTTG GCTCGGGGTA TAGAAGAGTG GATACTTTGA CCTATCTCAA ATTCACCAGA CTCATGTTTG   
  
  
+ CTTTGAAAAG AGGTTTATAG ATAAAGAAAA CGTAAAAAAA AAAAAAATAA ATAAAAAAAA ATGAGTGACT   
  
  
+ AAAGATAACT TACGTGGATG AGAATTTGAG TTGGAGTATA AAAGGACGGA CACATTGACC TATCCCAGAT   
  
  
+ TCATGAGATT CACGTTTACC ATAGAGGTTT ATAGATAAAG AGAAAGTAAA AAAAAGAAAA AACAATGATT   
  
  
+ AAAGATTGTA ACTTTCTCAG CACCATCCAG ACTATTCTAA TAGACATCAC ACAAGTCATA ATATCAGAAT   
  
  
+ TTGTAGTGTT AGTTGTTTAC GCACACAAAT GATAGGCAGC CATCGACTGT TACAAAATTT ATAAAAGTCT   
  
  
+ CACACTAATA GATTATTAAA AGATTATTTC GTACTTGCGC GACTGATGGT AACAAAACTC GTAGATAGAA   
  
  
+ TATACTTTCT TTGAAGTGGG AATGATTTGA CCTACTCTAT AAATAATATA AACATCACAT GCATACACTC   
  
  
+ CAACCACTAA GCCACAGAAA GAAATGAAAG AAACAGAAAA TAATTAACAA CCCTATTGGA CTCTTCTCTA   
  
  
+ CACTGCCAGC CTTTCCTTCT TTGTTCTGAG CAAAGAAACA CACCAAAATT GTCAGCCATA AAATTTCTTC   
  
  
+ CTCACAGAAA CTCGTTTGGT TACTGAGAA  

- TGCTTTTGGC TTCGTTATTG TAAGATAAAT ATCCTCTCCT TTCAGCCGTG TCTCCTTCCA CTCTAATCTG   
  
  
- GTTCGTTTGA ATGTGGCGGA TTTTCGATAA CTATTCTTAC TACATTTTCG GGAAATTAAC CGATACCAAA   
  
  
- ACCAGGTTAA TCTTATCTTG TTTGTTGAAT TATTTTATTA CAGTTTTCCT TTCTTTTACT TATTATACTT   
  
  
- ATATATCAAG TTTATGATGA CATTACCACA TGACTTGAAT AAACCAAGTT TTATCATCAT TAATCTCATA   
  
  
- TCTTTTTACT TTATTTTCTT AATTGCTAAT TTATTCTTTA TTATGTTTTG CTATACTTTA ATTATTCATA   
  
  
- TATGTTGTGT ATTAATATAT ATTAATAAAA CCTTTTATAT CCAGGAGTGT TCTGCGGATT CACGATCGTG   
  
  
- GATGAACGAC GACCGATCGC GAATCCAGGA CGAAAAAGAA TATAATATGA GATTTAATAA CATTGAACCC   
  
  
- ATATCTTCAC GTCCGTTTGA TTGGATAGGG TATAAGTGGT CTAAGTGCAA ACGAAACTTT TCTTCAAATA   
  
  
- TCTACTTCTT TTACAATTTT TCTTTTTTCT CACTAATCTC TATTGAACGA ACCTACACCT AAAACGAGCC   
  
  
- CCATTTCTTC CCGCCCGTGT GACTGGATAG GGTCTATATG ATAAAAGTGT AAAAGAAACT TTTCTCCAAA   
  
  
- TGTATATTTC TTTTACACAT TTTTTTTTAT TTTTTATTTT ATTTTATTTC ACTAATTTCT ATTGAAACCT   
  
  
- ACTCCTAAAC CGAGCCCCAT ATCTTCTCAC CTATGAAACT GGATAGAGTT TAAGTGGTCT GAGTACAAAC   
  
  
- GAAACTTTTC TCCAAATATC TATTTCTTTT GCATTTTTTT TTTTTTTATT TATTTTTTTT TACTCACTGA   
  
  
- TTTCTATTGA ATGCACCTAC TCTTAAACTC AACCTCATAT TTTCCTGCCT GTGTAACTGG ATAGGGTCTA   
  
  
- AGTACTCTAA GTGCAAATGG TATCTCCAAA TATCTATTTC TCTTTCATTT TTTTTCTTTT TTGTTACTAA   
  
  
- TTTCTAACAT TGAAAGAGTC GTGGTAGGTC TGATAAGATT ATCTGTAGTG TGTTCAGTAT TATAGTCTTA   
  
  
- AACATCACAA TCAACAAATG CGTGTGTTTA CTATCCGTCG GTAGCTGACA ATGTTTTAAA TATTTTCAGA   
  
  
- GTGTGATTAT CTAATAATTT TCTAATAAAG CATGAACGCG CTGACTACCA TTGTTTTGAG CATCTATCTT   
  
  
- ATATGAAAGA AACTTCACCC TTACTAAACT GGATGAGATA TTTATTATAT TTGTAGTGTA CGTATGTGAG   
  
  
- GTTGGTGATT CGGTGTCTTT CTTTACTTTC TTTGTCTTTT ATTAATTGTT GGGATAACCT GAGAAGAGAT   
  
  
- GTGACGGTCG GAAAGGAAGA AACAAGACTC GTTTCTTTGT GTGGTTTTAA CAGTCGGTAT TTTAAAGAAG   
  
  
- GAGTGTCTTT GAGCAAACCA ATGACTCTT

+     Sp1

| Site Name | Organism | Position | Strand | Matrix score. | sequence | function |
| --- | --- | --- | --- | --- | --- | --- |
| Sp1 | Oryza sativa | 640 | + | 6 | GGGCGG | light responsive element |

>038667   
+ ACGAAAACCG AAGCAATAAC ATTCTATTTA TAGGAGAGGA AAGTCGGCAC AGAGGAAGGT GAGATTAGAC   
  
  
+ CAAGCAAACT TACACCGCCT AAAAGCTATT GATAAGAATG ATGTAAAAGC CCTTTAATTG GCTATGGTTT   
  
  
+ TGGTCCAATT AGAATAGAAC AAACAACTTA ATAAAATAAT GTCAAAAGGA AAGAAAATGA ATAATATGAA   
  
  
+ TATATAGTTC AAATACTACT GTAATGGTGT ACTGAACTTA TTTGGTTCAA AATAGTAGTA ATTAGAGTAT   
  
  
+ AGAAAAATGA AATAAAAGAA TTAACGATTA AATAAGAAAT AATACAAAAC GATATGAAAT TAATAAGTAT   
  
  
+ ATACAACACA TAATTATATA TAATTATTTT GGAAAATATA GGTCCTCACA AGACGCCTAA GTGCTAGCAC   
  
  
+ CTACTTGCTG CTGGCTAGCG CTTAGGTCCT GCTTTTTCTT ATATTATACT CTAAATTATT GTAACTTGGG   
  
  
+ TATAGAAGTG CAGGCAAACT AACCTATCCC ATATTCACCA GATTCACGTT TGCTTTGAAA AGAAGTTTAT   
  
  
+ AGATGAAGAA AATGTTAAAA AGAAAAAAGA GTGATTAGAG ATAACTTGCT TGGATGTGGA TTTTGCTCGG   
  
  
+ GGTAAAGAAG GGCGGGCACA CTGACCTATC CCAGATATAC TATTTTCACA TTTTCTTTGA AAAGAGGTTT   
  
  
+ ACATATAAAG AAAATGTGTA AAAAAAAATA AAAAATAAAA TAAAATAAAG TGATTAAAGA TAACTTTGGA   
  
  
+ TGAGGATTTG GCTCGGGGTA TAGAAGAGTG GATACTTTGA CCTATCTCAA ATTCACCAGA CTCATGTTTG   
  
  
+ CTTTGAAAAG AGGTTTATAG ATAAAGAAAA CGTAAAAAAA AAAAAAATAA ATAAAAAAAA ATGAGTGACT   
  
  
+ AAAGATAACT TACGTGGATG AGAATTTGAG TTGGAGTATA AAAGGACGGA CACATTGACC TATCCCAGAT   
  
  
+ TCATGAGATT CACGTTTACC ATAGAGGTTT ATAGATAAAG AGAAAGTAAA AAAAAGAAAA AACAATGATT   
  
  
+ AAAGATTGTA ACTTTCTCAG CACCATCCAG ACTATTCTAA TAGACATCAC ACAAGTCATA ATATCAGAAT   
  
  
+ TTGTAGTGTT AGTTGTTTAC GCACACAAAT GATAGGCAGC CATCGACTGT TACAAAATTT ATAAAAGTCT   
  
  
+ CACACTAATA GATTATTAAA AGATTATTTC GTACTTGCGC GACTGATGGT AACAAAACTC GTAGATAGAA   
  
  
+ TATACTTTCT TTGAAGTGGG AATGATTTGA CCTACTCTAT AAATAATATA AACATCACAT GCATACACTC   
  
  
+ CAACCACTAA GCCACAGAAA GAAATGAAAG AAACAGAAAA TAATTAACAA CCCTATTGGA CTCTTCTCTA   
  
  
+ CACTGCCAGC CTTTCCTTCT TTGTTCTGAG CAAAGAAACA CACCAAAATT GTCAGCCATA AAATTTCTTC   
  
  
+ CTCACAGAAA CTCGTTTGGT TACTGAGAA  

- TGCTTTTGGC TTCGTTATTG TAAGATAAAT ATCCTCTCCT TTCAGCCGTG TCTCCTTCCA CTCTAATCTG   
  
  
- GTTCGTTTGA ATGTGGCGGA TTTTCGATAA CTATTCTTAC TACATTTTCG GGAAATTAAC CGATACCAAA   
  
  
- ACCAGGTTAA TCTTATCTTG TTTGTTGAAT TATTTTATTA CAGTTTTCCT TTCTTTTACT TATTATACTT   
  
  
- ATATATCAAG TTTATGATGA CATTACCACA TGACTTGAAT AAACCAAGTT TTATCATCAT TAATCTCATA   
  
  
- TCTTTTTACT TTATTTTCTT AATTGCTAAT TTATTCTTTA TTATGTTTTG CTATACTTTA ATTATTCATA   
  
  
- TATGTTGTGT ATTAATATAT ATTAATAAAA CCTTTTATAT CCAGGAGTGT TCTGCGGATT CACGATCGTG   
  
  
- GATGAACGAC GACCGATCGC GAATCCAGGA CGAAAAAGAA TATAATATGA GATTTAATAA CATTGAACCC   
  
  
- ATATCTTCAC GTCCGTTTGA TTGGATAGGG TATAAGTGGT CTAAGTGCAA ACGAAACTTT TCTTCAAATA   
  
  
- TCTACTTCTT TTACAATTTT TCTTTTTTCT CACTAATCTC TATTGAACGA ACCTACACCT AAAACGAGCC   
  
  
- CCATTTCTTC CCGCCCGTGT GACTGGATAG GGTCTATATG ATAAAAGTGT AAAAGAAACT TTTCTCCAAA   
  
  
- TGTATATTTC TTTTACACAT TTTTTTTTAT TTTTTATTTT ATTTTATTTC ACTAATTTCT ATTGAAACCT   
  
  
- ACTCCTAAAC CGAGCCCCAT ATCTTCTCAC CTATGAAACT GGATAGAGTT TAAGTGGTCT GAGTACAAAC   
  
  
- GAAACTTTTC TCCAAATATC TATTTCTTTT GCATTTTTTT TTTTTTTATT TATTTTTTTT TACTCACTGA   
  
  
- TTTCTATTGA ATGCACCTAC TCTTAAACTC AACCTCATAT TTTCCTGCCT GTGTAACTGG ATAGGGTCTA   
  
  
- AGTACTCTAA GTGCAAATGG TATCTCCAAA TATCTATTTC TCTTTCATTT TTTTTCTTTT TTGTTACTAA   
  
  
- TTTCTAACAT TGAAAGAGTC GTGGTAGGTC TGATAAGATT ATCTGTAGTG TGTTCAGTAT TATAGTCTTA   
  
  
- AACATCACAA TCAACAAATG CGTGTGTTTA CTATCCGTCG GTAGCTGACA ATGTTTTAAA TATTTTCAGA   
  
  
- GTGTGATTAT CTAATAATTT TCTAATAAAG CATGAACGCG CTGACTACCA TTGTTTTGAG CATCTATCTT   
  
  
- ATATGAAAGA AACTTCACCC TTACTAAACT GGATGAGATA TTTATTATAT TTGTAGTGTA CGTATGTGAG   
  
  
- GTTGGTGATT CGGTGTCTTT CTTTACTTTC TTTGTCTTTT ATTAATTGTT GGGATAACCT GAGAAGAGAT   
  
  
- GTGACGGTCG GAAAGGAAGA AACAAGACTC GTTTCTTTGT GTGGTTTTAA CAGTCGGTAT TTTAAAGAAG   
  
  
- GAGTGTCTTT GAGCAAACCA ATGACTCTT

+     TATA-box

| Site Name | Organism | Position | Strand | Matrix score. | sequence | function |
| --- | --- | --- | --- | --- | --- | --- |
| TATA-box | Arabidopsis thaliana | 464 | - | 5 | TATAA | core promoter element around -30 of transcription start |
| TATA-box | Arabidopsis thaliana | 369 | + | 4 | TATA | core promoter element around -30 of transcription start |
| TATA-box | Arabidopsis thaliana | 465 | + | 4 | TATA | core promoter element around -30 of transcription start |
| TATA-box | Arabidopsis thaliana | 460 | + | 4 | TATA | core promoter element around -30 of transcription start |
| TATA-box | Arabidopsis thaliana | 459 | - | 5 | TATAA | core promoter element around -30 of transcription start |
| TATA-box | Arabidopsis thaliana | 348 | + | 6 | TATATA | core promoter element around -30 of transcription start |
| TATA-box | Helianthus annuus | 1178 | - | 6 | TATAAA | core promoter element around -30 of transcription start |
| TATA-box | Oryza sativa | 323 | + | 7 | TACAAAA | core promoter element around -30 of transcription start |
| TATA-box | Oryza sativa | 1171 | + | 7 | TACAAAA | core promoter element around -30 of transcription start |
| TATA-box | Arabidopsis thaliana | 28 | - | 5 | TATAA | core promoter element around -30 of transcription start |
| TATA-box | Arabidopsis thaliana | 1307 | - | 4 | TATA | core promoter element around -30 of transcription start |
| TATA-box | Arabidopsis thaliana | 1010 | - | 4 | TATA | core promoter element around -30 of transcription start |
| TATA-box | Arabidopsis thaliana | 1009 | - | 5 | TATAA | core promoter element around -30 of transcription start |
| TATA-box | Zea mays | 456 | - | 8 | TATAAGAA | core promoter element around -30 of transcription start |
| TATA-box | Arabidopsis thaliana | 789 | - | 4 | TATA | core promoter element around -30 of transcription start |
| TATA-box | Arabidopsis thaliana | 704 | + | 4 | TATA | core promoter element around -30 of transcription start |
| TATA-box | Brassica oleracea | 368 | + | 6 | ATATAA | core promoter element around -30 of transcription start |
| TATA-box | Arabidopsis thaliana | 387 | + | 4 | TATA | core promoter element around -30 of transcription start |
| TATA-box | Brassica napus | 366 | + | 6 | ATATAT | core promoter element around -30 of transcription start |
| TATA-box | Brassica juncea | 1177 | - | 7 | TATAAAT | core promoter element around -30 of transcription start |
| TATA-box | Arabidopsis thaliana | 1050 | + | 8 | TAAAGATT | core promoter element around -30 of transcription start |
| TATA-box | Arabidopsis thaliana | 213 | + | 4 | TATA | core promoter element around -30 of transcription start |
| TATA-box | Brassica napus | 463 | + | 6 | ATTATA | core promoter element around -30 of transcription start |
| TATA-box | Arabidopsis thaliana | 211 | + | 6 | TATATA | core promoter element around -30 of transcription start |
| TATA-box | Arabidopsis thaliana | 1298 | - | 4 | TATA | core promoter element around -30 of transcription start |
| TATA-box | Brassica napus | 363 | + | 6 | ATTATA | core promoter element around -30 of transcription start |
| TATA-box | Helianthus annuus | 27 | - | 6 | TATAAA | core promoter element around -30 of transcription start |
| TATA-box | Helianthus annuus | 1008 | - | 6 | TATAAA | core promoter element around -30 of transcription start |
| TATA-box | Arabidopsis thaliana | 558 | + | 4 | TATA | core promoter element around -30 of transcription start |
| TATA-box | Brassica juncea | 26 | - | 7 | TATAAAT | core promoter element around -30 of transcription start |
| TATA-box | Arabidopsis thaliana | 364 | - | 7 | TATATAA | core promoter element around -30 of transcription start |
| TATA-box | Arabidopsis thaliana | 947 | - | 4 | TATA | core promoter element around -30 of transcription start |
| TATA-box | Arabidopsis thaliana | 856 | - | 4 | TATA | core promoter element around -30 of transcription start |
| TATA-box | Arabidopsis thaliana | 855 | - | 5 | TATAA | core promoter element around -30 of transcription start |
| TATA-box | Arabidopsis thaliana | 666 | + | 4 | TATA | core promoter element around -30 of transcription start |
| TATA-box | Brassica oleracea | 703 | + | 6 | ATATAA | core promoter element around -30 of transcription start |
| TATA-box | Arabidopsis thaliana | 29 | + | 4 | TATA | core promoter element around -30 of transcription start |
| TATA-box | Arabidopsis thaliana | 25 | - | 9 | ccTATAAAaa | core promoter element around -30 of transcription start |
| TATA-box | Arabidopsis thaliana | 1179 | - | 5 | TATAA | core promoter element around -30 of transcription start |
| TATA-box | Brassica oleracea | 1306 | + | 6 | ATATAA | core promoter element around -30 of transcription start |
| TATA-box | Arabidopsis thaliana | 1261 | - | 4 | TATA | core promoter element around -30 of transcription start |
| TATA-box | Arabidopsis thaliana | 365 | + | 6 | TATATA | core promoter element around -30 of transcription start |
| TATA-box | Arabidopsis thaliana | 557 | - | 5 | TATAA | core promoter element around -30 of transcription start |
| TATA-box | Arabidopsis thaliana | 491 | + | 4 | TATA | core promoter element around -30 of transcription start |
| TATA-box | Brassica napus | 210 | + | 6 | ATATAT | core promoter element around -30 of transcription start |
| TATA-box | Arabidopsis thaliana | 1180 | - | 4 | TATA | core promoter element around -30 of transcription start |
| TATA-box | Arabidopsis thaliana | 278 | + | 4 | TATA | core promoter element around -30 of transcription start |
| TATA-box | Arabidopsis thaliana | 367 | + | 6 | TATATA | core promoter element around -30 of transcription start |
| TATA-box | Helianthus annuus | 854 | - | 6 | TATAAA | core promoter element around -30 of transcription start |
| TATA-box | Arabidopsis thaliana | 350 | + | 4 | TATA | core promoter element around -30 of transcription start |
| TATA-box | Helianthus annuus | 556 | - | 6 | TATAAA | core promoter element around -30 of transcription start |

>038667   
+ ACGAAAACCG AAGCAATAAC ATTCTATTTA TAGGAGAGGA AAGTCGGCAC AGAGGAAGGT GAGATTAGAC   
  
  
+ CAAGCAAACT TACACCGCCT AAAAGCTATT GATAAGAATG ATGTAAAAGC CCTTTAATTG GCTATGGTTT   
  
  
+ TGGTCCAATT AGAATAGAAC AAACAACTTA ATAAAATAAT GTCAAAAGGA AAGAAAATGA ATAATATGAA   
  
  
+ TATATAGTTC AAATACTACT GTAATGGTGT ACTGAACTTA TTTGGTTCAA AATAGTAGTA ATTAGAGTAT   
  
  
+ AGAAAAATGA AATAAAAGAA TTAACGATTA AATAAGAAAT AATACAAAAC GATATGAAAT TAATAAGTAT   
  
  
+ ATACAACACA TAATTATATA TAATTATTTT GGAAAATATA GGTCCTCACA AGACGCCTAA GTGCTAGCAC   
  
  
+ CTACTTGCTG CTGGCTAGCG CTTAGGTCCT GCTTTTTCTT ATATTATACT CTAAATTATT GTAACTTGGG   
  
  
+ TATAGAAGTG CAGGCAAACT AACCTATCCC ATATTCACCA GATTCACGTT TGCTTTGAAA AGAAGTTTAT   
  
  
+ AGATGAAGAA AATGTTAAAA AGAAAAAAGA GTGATTAGAG ATAACTTGCT TGGATGTGGA TTTTGCTCGG   
  
  
+ GGTAAAGAAG GGCGGGCACA CTGACCTATC CCAGATATAC TATTTTCACA TTTTCTTTGA AAAGAGGTTT   
  
  
+ ACATATAAAG AAAATGTGTA AAAAAAAATA AAAAATAAAA TAAAATAAAG TGATTAAAGA TAACTTTGGA   
  
  
+ TGAGGATTTG GCTCGGGGTA TAGAAGAGTG GATACTTTGA CCTATCTCAA ATTCACCAGA CTCATGTTTG   
  
  
+ CTTTGAAAAG AGGTTTATAG ATAAAGAAAA CGTAAAAAAA AAAAAAATAA ATAAAAAAAA ATGAGTGACT   
  
  
+ AAAGATAACT TACGTGGATG AGAATTTGAG TTGGAGTATA AAAGGACGGA CACATTGACC TATCCCAGAT   
  
  
+ TCATGAGATT CACGTTTACC ATAGAGGTTT ATAGATAAAG AGAAAGTAAA AAAAAGAAAA AACAATGATT   
  
  
+ AAAGATTGTA ACTTTCTCAG CACCATCCAG ACTATTCTAA TAGACATCAC ACAAGTCATA ATATCAGAAT   
  
  
+ TTGTAGTGTT AGTTGTTTAC GCACACAAAT GATAGGCAGC CATCGACTGT TACAAAATTT ATAAAAGTCT   
  
  
+ CACACTAATA GATTATTAAA AGATTATTTC GTACTTGCGC GACTGATGGT AACAAAACTC GTAGATAGAA   
  
  
+ TATACTTTCT TTGAAGTGGG AATGATTTGA CCTACTCTAT AAATAATATA AACATCACAT GCATACACTC   
  
  
+ CAACCACTAA GCCACAGAAA GAAATGAAAG AAACAGAAAA TAATTAACAA CCCTATTGGA CTCTTCTCTA   
  
  
+ CACTGCCAGC CTTTCCTTCT TTGTTCTGAG CAAAGAAACA CACCAAAATT GTCAGCCATA AAATTTCTTC   
  
  
+ CTCACAGAAA CTCGTTTGGT TACTGAGAA  

- TGCTTTTGGC TTCGTTATTG TAAGATAAAT ATCCTCTCCT TTCAGCCGTG TCTCCTTCCA CTCTAATCTG   
  
  
- GTTCGTTTGA ATGTGGCGGA TTTTCGATAA CTATTCTTAC TACATTTTCG GGAAATTAAC CGATACCAAA   
  
  
- ACCAGGTTAA TCTTATCTTG TTTGTTGAAT TATTTTATTA CAGTTTTCCT TTCTTTTACT TATTATACTT   
  
  
- ATATATCAAG TTTATGATGA CATTACCACA TGACTTGAAT AAACCAAGTT TTATCATCAT TAATCTCATA   
  
  
- TCTTTTTACT TTATTTTCTT AATTGCTAAT TTATTCTTTA TTATGTTTTG CTATACTTTA ATTATTCATA   
  
  
- TATGTTGTGT ATTAATATAT ATTAATAAAA CCTTTTATAT CCAGGAGTGT TCTGCGGATT CACGATCGTG   
  
  
- GATGAACGAC GACCGATCGC GAATCCAGGA CGAAAAAGAA TATAATATGA GATTTAATAA CATTGAACCC   
  
  
- ATATCTTCAC GTCCGTTTGA TTGGATAGGG TATAAGTGGT CTAAGTGCAA ACGAAACTTT TCTTCAAATA   
  
  
- TCTACTTCTT TTACAATTTT TCTTTTTTCT CACTAATCTC TATTGAACGA ACCTACACCT AAAACGAGCC   
  
  
- CCATTTCTTC CCGCCCGTGT GACTGGATAG GGTCTATATG ATAAAAGTGT AAAAGAAACT TTTCTCCAAA   
  
  
- TGTATATTTC TTTTACACAT TTTTTTTTAT TTTTTATTTT ATTTTATTTC ACTAATTTCT ATTGAAACCT   
  
  
- ACTCCTAAAC CGAGCCCCAT ATCTTCTCAC CTATGAAACT GGATAGAGTT TAAGTGGTCT GAGTACAAAC   
  
  
- GAAACTTTTC TCCAAATATC TATTTCTTTT GCATTTTTTT TTTTTTTATT TATTTTTTTT TACTCACTGA   
  
  
- TTTCTATTGA ATGCACCTAC TCTTAAACTC AACCTCATAT TTTCCTGCCT GTGTAACTGG ATAGGGTCTA   
  
  
- AGTACTCTAA GTGCAAATGG TATCTCCAAA TATCTATTTC TCTTTCATTT TTTTTCTTTT TTGTTACTAA   
  
  
- TTTCTAACAT TGAAAGAGTC GTGGTAGGTC TGATAAGATT ATCTGTAGTG TGTTCAGTAT TATAGTCTTA   
  
  
- AACATCACAA TCAACAAATG CGTGTGTTTA CTATCCGTCG GTAGCTGACA ATGTTTTAAA TATTTTCAGA   
  
  
- GTGTGATTAT CTAATAATTT TCTAATAAAG CATGAACGCG CTGACTACCA TTGTTTTGAG CATCTATCTT   
  
  
- ATATGAAAGA AACTTCACCC TTACTAAACT GGATGAGATA TTTATTATAT TTGTAGTGTA CGTATGTGAG   
  
  
- GTTGGTGATT CGGTGTCTTT CTTTACTTTC TTTGTCTTTT ATTAATTGTT GGGATAACCT GAGAAGAGAT   
  
  
- GTGACGGTCG GAAAGGAAGA AACAAGACTC GTTTCTTTGT GTGGTTTTAA CAGTCGGTAT TTTAAAGAAG   
  
  
- GAGTGTCTTT GAGCAAACCA ATGACTCTT

+     TATC-box

| Site Name | Organism | Position | Strand | Matrix score. | sequence | function |
| --- | --- | --- | --- | --- | --- | --- |
| TATC-box | Oryza sativa | 971 | + | 7 | TATCCCA | cis-acting element involved in gibberellin-responsiveness |
| TATC-box | Oryza sativa | 515 | + | 7 | TATCCCA | cis-acting element involved in gibberellin-responsiveness |
| TATC-box | Oryza sativa | 657 | + | 7 | TATCCCA | cis-acting element involved in gibberellin-responsiveness |

>038667   
+ ACGAAAACCG AAGCAATAAC ATTCTATTTA TAGGAGAGGA AAGTCGGCAC AGAGGAAGGT GAGATTAGAC   
  
  
+ CAAGCAAACT TACACCGCCT AAAAGCTATT GATAAGAATG ATGTAAAAGC CCTTTAATTG GCTATGGTTT   
  
  
+ TGGTCCAATT AGAATAGAAC AAACAACTTA ATAAAATAAT GTCAAAAGGA AAGAAAATGA ATAATATGAA   
  
  
+ TATATAGTTC AAATACTACT GTAATGGTGT ACTGAACTTA TTTGGTTCAA AATAGTAGTA ATTAGAGTAT   
  
  
+ AGAAAAATGA AATAAAAGAA TTAACGATTA AATAAGAAAT AATACAAAAC GATATGAAAT TAATAAGTAT   
  
  
+ ATACAACACA TAATTATATA TAATTATTTT GGAAAATATA GGTCCTCACA AGACGCCTAA GTGCTAGCAC   
  
  
+ CTACTTGCTG CTGGCTAGCG CTTAGGTCCT GCTTTTTCTT ATATTATACT CTAAATTATT GTAACTTGGG   
  
  
+ TATAGAAGTG CAGGCAAACT AACCTATCCC ATATTCACCA GATTCACGTT TGCTTTGAAA AGAAGTTTAT   
  
  
+ AGATGAAGAA AATGTTAAAA AGAAAAAAGA GTGATTAGAG ATAACTTGCT TGGATGTGGA TTTTGCTCGG   
  
  
+ GGTAAAGAAG GGCGGGCACA CTGACCTATC CCAGATATAC TATTTTCACA TTTTCTTTGA AAAGAGGTTT   
  
  
+ ACATATAAAG AAAATGTGTA AAAAAAAATA AAAAATAAAA TAAAATAAAG TGATTAAAGA TAACTTTGGA   
  
  
+ TGAGGATTTG GCTCGGGGTA TAGAAGAGTG GATACTTTGA CCTATCTCAA ATTCACCAGA CTCATGTTTG   
  
  
+ CTTTGAAAAG AGGTTTATAG ATAAAGAAAA CGTAAAAAAA AAAAAAATAA ATAAAAAAAA ATGAGTGACT   
  
  
+ AAAGATAACT TACGTGGATG AGAATTTGAG TTGGAGTATA AAAGGACGGA CACATTGACC TATCCCAGAT   
  
  
+ TCATGAGATT CACGTTTACC ATAGAGGTTT ATAGATAAAG AGAAAGTAAA AAAAAGAAAA AACAATGATT   
  
  
+ AAAGATTGTA ACTTTCTCAG CACCATCCAG ACTATTCTAA TAGACATCAC ACAAGTCATA ATATCAGAAT   
  
  
+ TTGTAGTGTT AGTTGTTTAC GCACACAAAT GATAGGCAGC CATCGACTGT TACAAAATTT ATAAAAGTCT   
  
  
+ CACACTAATA GATTATTAAA AGATTATTTC GTACTTGCGC GACTGATGGT AACAAAACTC GTAGATAGAA   
  
  
+ TATACTTTCT TTGAAGTGGG AATGATTTGA CCTACTCTAT AAATAATATA AACATCACAT GCATACACTC   
  
  
+ CAACCACTAA GCCACAGAAA GAAATGAAAG AAACAGAAAA TAATTAACAA CCCTATTGGA CTCTTCTCTA   
  
  
+ CACTGCCAGC CTTTCCTTCT TTGTTCTGAG CAAAGAAACA CACCAAAATT GTCAGCCATA AAATTTCTTC   
  
  
+ CTCACAGAAA CTCGTTTGGT TACTGAGAA  

- TGCTTTTGGC TTCGTTATTG TAAGATAAAT ATCCTCTCCT TTCAGCCGTG TCTCCTTCCA CTCTAATCTG   
  
  
- GTTCGTTTGA ATGTGGCGGA TTTTCGATAA CTATTCTTAC TACATTTTCG GGAAATTAAC CGATACCAAA   
  
  
- ACCAGGTTAA TCTTATCTTG TTTGTTGAAT TATTTTATTA CAGTTTTCCT TTCTTTTACT TATTATACTT   
  
  
- ATATATCAAG TTTATGATGA CATTACCACA TGACTTGAAT AAACCAAGTT TTATCATCAT TAATCTCATA   
  
  
- TCTTTTTACT TTATTTTCTT AATTGCTAAT TTATTCTTTA TTATGTTTTG CTATACTTTA ATTATTCATA   
  
  
- TATGTTGTGT ATTAATATAT ATTAATAAAA CCTTTTATAT CCAGGAGTGT TCTGCGGATT CACGATCGTG   
  
  
- GATGAACGAC GACCGATCGC GAATCCAGGA CGAAAAAGAA TATAATATGA GATTTAATAA CATTGAACCC   
  
  
- ATATCTTCAC GTCCGTTTGA TTGGATAGGG TATAAGTGGT CTAAGTGCAA ACGAAACTTT TCTTCAAATA   
  
  
- TCTACTTCTT TTACAATTTT TCTTTTTTCT CACTAATCTC TATTGAACGA ACCTACACCT AAAACGAGCC   
  
  
- CCATTTCTTC CCGCCCGTGT GACTGGATAG GGTCTATATG ATAAAAGTGT AAAAGAAACT TTTCTCCAAA   
  
  
- TGTATATTTC TTTTACACAT TTTTTTTTAT TTTTTATTTT ATTTTATTTC ACTAATTTCT ATTGAAACCT   
  
  
- ACTCCTAAAC CGAGCCCCAT ATCTTCTCAC CTATGAAACT GGATAGAGTT TAAGTGGTCT GAGTACAAAC   
  
  
- GAAACTTTTC TCCAAATATC TATTTCTTTT GCATTTTTTT TTTTTTTATT TATTTTTTTT TACTCACTGA   
  
  
- TTTCTATTGA ATGCACCTAC TCTTAAACTC AACCTCATAT TTTCCTGCCT GTGTAACTGG ATAGGGTCTA   
  
  
- AGTACTCTAA GTGCAAATGG TATCTCCAAA TATCTATTTC TCTTTCATTT TTTTTCTTTT TTGTTACTAA   
  
  
- TTTCTAACAT TGAAAGAGTC GTGGTAGGTC TGATAAGATT ATCTGTAGTG TGTTCAGTAT TATAGTCTTA   
  
  
- AACATCACAA TCAACAAATG CGTGTGTTTA CTATCCGTCG GTAGCTGACA ATGTTTTAAA TATTTTCAGA   
  
  
- GTGTGATTAT CTAATAATTT TCTAATAAAG CATGAACGCG CTGACTACCA TTGTTTTGAG CATCTATCTT   
  
  
- ATATGAAAGA AACTTCACCC TTACTAAACT GGATGAGATA TTTATTATAT TTGTAGTGTA CGTATGTGAG   
  
  
- GTTGGTGATT CGGTGTCTTT CTTTACTTTC TTTGTCTTTT ATTAATTGTT GGGATAACCT GAGAAGAGAT   
  
  
- GTGACGGTCG GAAAGGAAGA AACAAGACTC GTTTCTTTGT GTGGTTTTAA CAGTCGGTAT TTTAAAGAAG   
  
  
- GAGTGTCTTT GAGCAAACCA ATGACTCTT

+     TC-rich repeats

| Site Name | Organism | Position | Strand | Matrix score. | sequence | function |
| --- | --- | --- | --- | --- | --- | --- |
| TC-rich repeats | Nicotiana tabacum | 1239 | - | 9 | GTTTTCTTAC | cis-acting element involved in defense and stress responsiveness |

>038667   
+ ACGAAAACCG AAGCAATAAC ATTCTATTTA TAGGAGAGGA AAGTCGGCAC AGAGGAAGGT GAGATTAGAC   
  
  
+ CAAGCAAACT TACACCGCCT AAAAGCTATT GATAAGAATG ATGTAAAAGC CCTTTAATTG GCTATGGTTT   
  
  
+ TGGTCCAATT AGAATAGAAC AAACAACTTA ATAAAATAAT GTCAAAAGGA AAGAAAATGA ATAATATGAA   
  
  
+ TATATAGTTC AAATACTACT GTAATGGTGT ACTGAACTTA TTTGGTTCAA AATAGTAGTA ATTAGAGTAT   
  
  
+ AGAAAAATGA AATAAAAGAA TTAACGATTA AATAAGAAAT AATACAAAAC GATATGAAAT TAATAAGTAT   
  
  
+ ATACAACACA TAATTATATA TAATTATTTT GGAAAATATA GGTCCTCACA AGACGCCTAA GTGCTAGCAC   
  
  
+ CTACTTGCTG CTGGCTAGCG CTTAGGTCCT GCTTTTTCTT ATATTATACT CTAAATTATT GTAACTTGGG   
  
  
+ TATAGAAGTG CAGGCAAACT AACCTATCCC ATATTCACCA GATTCACGTT TGCTTTGAAA AGAAGTTTAT   
  
  
+ AGATGAAGAA AATGTTAAAA AGAAAAAAGA GTGATTAGAG ATAACTTGCT TGGATGTGGA TTTTGCTCGG   
  
  
+ GGTAAAGAAG GGCGGGCACA CTGACCTATC CCAGATATAC TATTTTCACA TTTTCTTTGA AAAGAGGTTT   
  
  
+ ACATATAAAG AAAATGTGTA AAAAAAAATA AAAAATAAAA TAAAATAAAG TGATTAAAGA TAACTTTGGA   
  
  
+ TGAGGATTTG GCTCGGGGTA TAGAAGAGTG GATACTTTGA CCTATCTCAA ATTCACCAGA CTCATGTTTG   
  
  
+ CTTTGAAAAG AGGTTTATAG ATAAAGAAAA CGTAAAAAAA AAAAAAATAA ATAAAAAAAA ATGAGTGACT   
  
  
+ AAAGATAACT TACGTGGATG AGAATTTGAG TTGGAGTATA AAAGGACGGA CACATTGACC TATCCCAGAT   
  
  
+ TCATGAGATT CACGTTTACC ATAGAGGTTT ATAGATAAAG AGAAAGTAAA AAAAAGAAAA AACAATGATT   
  
  
+ AAAGATTGTA ACTTTCTCAG CACCATCCAG ACTATTCTAA TAGACATCAC ACAAGTCATA ATATCAGAAT   
  
  
+ TTGTAGTGTT AGTTGTTTAC GCACACAAAT GATAGGCAGC CATCGACTGT TACAAAATTT ATAAAAGTCT   
  
  
+ CACACTAATA GATTATTAAA AGATTATTTC GTACTTGCGC GACTGATGGT AACAAAACTC GTAGATAGAA   
  
  
+ TATACTTTCT TTGAAGTGGG AATGATTTGA CCTACTCTAT AAATAATATA AACATCACAT GCATACACTC   
  
  
+ CAACCACTAA GCCACAGAAA GAAATGAAAG AAACAGAAAA TAATTAACAA CCCTATTGGA CTCTTCTCTA   
  
  
+ CACTGCCAGC CTTTCCTTCT TTGTTCTGAG CAAAGAAACA CACCAAAATT GTCAGCCATA AAATTTCTTC   
  
  
+ CTCACAGAAA CTCGTTTGGT TACTGAGAA  

- TGCTTTTGGC TTCGTTATTG TAAGATAAAT ATCCTCTCCT TTCAGCCGTG TCTCCTTCCA CTCTAATCTG   
  
  
- GTTCGTTTGA ATGTGGCGGA TTTTCGATAA CTATTCTTAC TACATTTTCG GGAAATTAAC CGATACCAAA   
  
  
- ACCAGGTTAA TCTTATCTTG TTTGTTGAAT TATTTTATTA CAGTTTTCCT TTCTTTTACT TATTATACTT   
  
  
- ATATATCAAG TTTATGATGA CATTACCACA TGACTTGAAT AAACCAAGTT TTATCATCAT TAATCTCATA   
  
  
- TCTTTTTACT TTATTTTCTT AATTGCTAAT TTATTCTTTA TTATGTTTTG CTATACTTTA ATTATTCATA   
  
  
- TATGTTGTGT ATTAATATAT ATTAATAAAA CCTTTTATAT CCAGGAGTGT TCTGCGGATT CACGATCGTG   
  
  
- GATGAACGAC GACCGATCGC GAATCCAGGA CGAAAAAGAA TATAATATGA GATTTAATAA CATTGAACCC   
  
  
- ATATCTTCAC GTCCGTTTGA TTGGATAGGG TATAAGTGGT CTAAGTGCAA ACGAAACTTT TCTTCAAATA   
  
  
- TCTACTTCTT TTACAATTTT TCTTTTTTCT CACTAATCTC TATTGAACGA ACCTACACCT AAAACGAGCC   
  
  
- CCATTTCTTC CCGCCCGTGT GACTGGATAG GGTCTATATG ATAAAAGTGT AAAAGAAACT TTTCTCCAAA   
  
  
- TGTATATTTC TTTTACACAT TTTTTTTTAT TTTTTATTTT ATTTTATTTC ACTAATTTCT ATTGAAACCT   
  
  
- ACTCCTAAAC CGAGCCCCAT ATCTTCTCAC CTATGAAACT GGATAGAGTT TAAGTGGTCT GAGTACAAAC   
  
  
- GAAACTTTTC TCCAAATATC TATTTCTTTT GCATTTTTTT TTTTTTTATT TATTTTTTTT TACTCACTGA   
  
  
- TTTCTATTGA ATGCACCTAC TCTTAAACTC AACCTCATAT TTTCCTGCCT GTGTAACTGG ATAGGGTCTA   
  
  
- AGTACTCTAA GTGCAAATGG TATCTCCAAA TATCTATTTC TCTTTCATTT TTTTTCTTTT TTGTTACTAA   
  
  
- TTTCTAACAT TGAAAGAGTC GTGGTAGGTC TGATAAGATT ATCTGTAGTG TGTTCAGTAT TATAGTCTTA   
  
  
- AACATCACAA TCAACAAATG CGTGTGTTTA CTATCCGTCG GTAGCTGACA ATGTTTTAAA TATTTTCAGA   
  
  
- GTGTGATTAT CTAATAATTT TCTAATAAAG CATGAACGCG CTGACTACCA TTGTTTTGAG CATCTATCTT   
  
  
- ATATGAAAGA AACTTCACCC TTACTAAACT GGATGAGATA TTTATTATAT TTGTAGTGTA CGTATGTGAG   
  
  
- GTTGGTGATT CGGTGTCTTT CTTTACTTTC TTTGTCTTTT ATTAATTGTT GGGATAACCT GAGAAGAGAT   
  
  
- GTGACGGTCG GAAAGGAAGA AACAAGACTC GTTTCTTTGT GTGGTTTTAA CAGTCGGTAT TTTAAAGAAG   
  
  
- GAGTGTCTTT GAGCAAACCA ATGACTCTT

+     Unnamed\_\_1

| Site Name | Organism | Position | Strand | Matrix score. | sequence | function |
| --- | --- | --- | --- | --- | --- | --- |
| Unnamed\_\_1 | Zea mays | 923 | + | 5 | CGTGG |  |

>038667   
+ ACGAAAACCG AAGCAATAAC ATTCTATTTA TAGGAGAGGA AAGTCGGCAC AGAGGAAGGT GAGATTAGAC   
  
  
+ CAAGCAAACT TACACCGCCT AAAAGCTATT GATAAGAATG ATGTAAAAGC CCTTTAATTG GCTATGGTTT   
  
  
+ TGGTCCAATT AGAATAGAAC AAACAACTTA ATAAAATAAT GTCAAAAGGA AAGAAAATGA ATAATATGAA   
  
  
+ TATATAGTTC AAATACTACT GTAATGGTGT ACTGAACTTA TTTGGTTCAA AATAGTAGTA ATTAGAGTAT   
  
  
+ AGAAAAATGA AATAAAAGAA TTAACGATTA AATAAGAAAT AATACAAAAC GATATGAAAT TAATAAGTAT   
  
  
+ ATACAACACA TAATTATATA TAATTATTTT GGAAAATATA GGTCCTCACA AGACGCCTAA GTGCTAGCAC   
  
  
+ CTACTTGCTG CTGGCTAGCG CTTAGGTCCT GCTTTTTCTT ATATTATACT CTAAATTATT GTAACTTGGG   
  
  
+ TATAGAAGTG CAGGCAAACT AACCTATCCC ATATTCACCA GATTCACGTT TGCTTTGAAA AGAAGTTTAT   
  
  
+ AGATGAAGAA AATGTTAAAA AGAAAAAAGA GTGATTAGAG ATAACTTGCT TGGATGTGGA TTTTGCTCGG   
  
  
+ GGTAAAGAAG GGCGGGCACA CTGACCTATC CCAGATATAC TATTTTCACA TTTTCTTTGA AAAGAGGTTT   
  
  
+ ACATATAAAG AAAATGTGTA AAAAAAAATA AAAAATAAAA TAAAATAAAG TGATTAAAGA TAACTTTGGA   
  
  
+ TGAGGATTTG GCTCGGGGTA TAGAAGAGTG GATACTTTGA CCTATCTCAA ATTCACCAGA CTCATGTTTG   
  
  
+ CTTTGAAAAG AGGTTTATAG ATAAAGAAAA CGTAAAAAAA AAAAAAATAA ATAAAAAAAA ATGAGTGACT   
  
  
+ AAAGATAACT TACGTGGATG AGAATTTGAG TTGGAGTATA AAAGGACGGA CACATTGACC TATCCCAGAT   
  
  
+ TCATGAGATT CACGTTTACC ATAGAGGTTT ATAGATAAAG AGAAAGTAAA AAAAAGAAAA AACAATGATT   
  
  
+ AAAGATTGTA ACTTTCTCAG CACCATCCAG ACTATTCTAA TAGACATCAC ACAAGTCATA ATATCAGAAT   
  
  
+ TTGTAGTGTT AGTTGTTTAC GCACACAAAT GATAGGCAGC CATCGACTGT TACAAAATTT ATAAAAGTCT   
  
  
+ CACACTAATA GATTATTAAA AGATTATTTC GTACTTGCGC GACTGATGGT AACAAAACTC GTAGATAGAA   
  
  
+ TATACTTTCT TTGAAGTGGG AATGATTTGA CCTACTCTAT AAATAATATA AACATCACAT GCATACACTC   
  
  
+ CAACCACTAA GCCACAGAAA GAAATGAAAG AAACAGAAAA TAATTAACAA CCCTATTGGA CTCTTCTCTA   
  
  
+ CACTGCCAGC CTTTCCTTCT TTGTTCTGAG CAAAGAAACA CACCAAAATT GTCAGCCATA AAATTTCTTC   
  
  
+ CTCACAGAAA CTCGTTTGGT TACTGAGAA  

- TGCTTTTGGC TTCGTTATTG TAAGATAAAT ATCCTCTCCT TTCAGCCGTG TCTCCTTCCA CTCTAATCTG   
  
  
- GTTCGTTTGA ATGTGGCGGA TTTTCGATAA CTATTCTTAC TACATTTTCG GGAAATTAAC CGATACCAAA   
  
  
- ACCAGGTTAA TCTTATCTTG TTTGTTGAAT TATTTTATTA CAGTTTTCCT TTCTTTTACT TATTATACTT   
  
  
- ATATATCAAG TTTATGATGA CATTACCACA TGACTTGAAT AAACCAAGTT TTATCATCAT TAATCTCATA   
  
  
- TCTTTTTACT TTATTTTCTT AATTGCTAAT TTATTCTTTA TTATGTTTTG CTATACTTTA ATTATTCATA   
  
  
- TATGTTGTGT ATTAATATAT ATTAATAAAA CCTTTTATAT CCAGGAGTGT TCTGCGGATT CACGATCGTG   
  
  
- GATGAACGAC GACCGATCGC GAATCCAGGA CGAAAAAGAA TATAATATGA GATTTAATAA CATTGAACCC   
  
  
- ATATCTTCAC GTCCGTTTGA TTGGATAGGG TATAAGTGGT CTAAGTGCAA ACGAAACTTT TCTTCAAATA   
  
  
- TCTACTTCTT TTACAATTTT TCTTTTTTCT CACTAATCTC TATTGAACGA ACCTACACCT AAAACGAGCC   
  
  
- CCATTTCTTC CCGCCCGTGT GACTGGATAG GGTCTATATG ATAAAAGTGT AAAAGAAACT TTTCTCCAAA   
  
  
- TGTATATTTC TTTTACACAT TTTTTTTTAT TTTTTATTTT ATTTTATTTC ACTAATTTCT ATTGAAACCT   
  
  
- ACTCCTAAAC CGAGCCCCAT ATCTTCTCAC CTATGAAACT GGATAGAGTT TAAGTGGTCT GAGTACAAAC   
  
  
- GAAACTTTTC TCCAAATATC TATTTCTTTT GCATTTTTTT TTTTTTTATT TATTTTTTTT TACTCACTGA   
  
  
- TTTCTATTGA ATGCACCTAC TCTTAAACTC AACCTCATAT TTTCCTGCCT GTGTAACTGG ATAGGGTCTA   
  
  
- AGTACTCTAA GTGCAAATGG TATCTCCAAA TATCTATTTC TCTTTCATTT TTTTTCTTTT TTGTTACTAA   
  
  
- TTTCTAACAT TGAAAGAGTC GTGGTAGGTC TGATAAGATT ATCTGTAGTG TGTTCAGTAT TATAGTCTTA   
  
  
- AACATCACAA TCAACAAATG CGTGTGTTTA CTATCCGTCG GTAGCTGACA ATGTTTTAAA TATTTTCAGA   
  
  
- GTGTGATTAT CTAATAATTT TCTAATAAAG CATGAACGCG CTGACTACCA TTGTTTTGAG CATCTATCTT   
  
  
- ATATGAAAGA AACTTCACCC TTACTAAACT GGATGAGATA TTTATTATAT TTGTAGTGTA CGTATGTGAG   
  
  
- GTTGGTGATT CGGTGTCTTT CTTTACTTTC TTTGTCTTTT ATTAATTGTT GGGATAACCT GAGAAGAGAT   
  
  
- GTGACGGTCG GAAAGGAAGA AACAAGACTC GTTTCTTTGT GTGGTTTTAA CAGTCGGTAT TTTAAAGAAG   
  
  
- GAGTGTCTTT GAGCAAACCA ATGACTCTT

+     Unnamed\_\_2

| Site Name | Organism | Position | Strand | Matrix score. | sequence | function |
| --- | --- | --- | --- | --- | --- | --- |
| Unnamed\_\_2 | Petroselinum hortense | 506 | + | 9 | AACCTAACCT |  |

>038667   
+ ACGAAAACCG AAGCAATAAC ATTCTATTTA TAGGAGAGGA AAGTCGGCAC AGAGGAAGGT GAGATTAGAC   
  
  
+ CAAGCAAACT TACACCGCCT AAAAGCTATT GATAAGAATG ATGTAAAAGC CCTTTAATTG GCTATGGTTT   
  
  
+ TGGTCCAATT AGAATAGAAC AAACAACTTA ATAAAATAAT GTCAAAAGGA AAGAAAATGA ATAATATGAA   
  
  
+ TATATAGTTC AAATACTACT GTAATGGTGT ACTGAACTTA TTTGGTTCAA AATAGTAGTA ATTAGAGTAT   
  
  
+ AGAAAAATGA AATAAAAGAA TTAACGATTA AATAAGAAAT AATACAAAAC GATATGAAAT TAATAAGTAT   
  
  
+ ATACAACACA TAATTATATA TAATTATTTT GGAAAATATA GGTCCTCACA AGACGCCTAA GTGCTAGCAC   
  
  
+ CTACTTGCTG CTGGCTAGCG CTTAGGTCCT GCTTTTTCTT ATATTATACT CTAAATTATT GTAACTTGGG   
  
  
+ TATAGAAGTG CAGGCAAACT AACCTATCCC ATATTCACCA GATTCACGTT TGCTTTGAAA AGAAGTTTAT   
  
  
+ AGATGAAGAA AATGTTAAAA AGAAAAAAGA GTGATTAGAG ATAACTTGCT TGGATGTGGA TTTTGCTCGG   
  
  
+ GGTAAAGAAG GGCGGGCACA CTGACCTATC CCAGATATAC TATTTTCACA TTTTCTTTGA AAAGAGGTTT   
  
  
+ ACATATAAAG AAAATGTGTA AAAAAAAATA AAAAATAAAA TAAAATAAAG TGATTAAAGA TAACTTTGGA   
  
  
+ TGAGGATTTG GCTCGGGGTA TAGAAGAGTG GATACTTTGA CCTATCTCAA ATTCACCAGA CTCATGTTTG   
  
  
+ CTTTGAAAAG AGGTTTATAG ATAAAGAAAA CGTAAAAAAA AAAAAAATAA ATAAAAAAAA ATGAGTGACT   
  
  
+ AAAGATAACT TACGTGGATG AGAATTTGAG TTGGAGTATA AAAGGACGGA CACATTGACC TATCCCAGAT   
  
  
+ TCATGAGATT CACGTTTACC ATAGAGGTTT ATAGATAAAG AGAAAGTAAA AAAAAGAAAA AACAATGATT   
  
  
+ AAAGATTGTA ACTTTCTCAG CACCATCCAG ACTATTCTAA TAGACATCAC ACAAGTCATA ATATCAGAAT   
  
  
+ TTGTAGTGTT AGTTGTTTAC GCACACAAAT GATAGGCAGC CATCGACTGT TACAAAATTT ATAAAAGTCT   
  
  
+ CACACTAATA GATTATTAAA AGATTATTTC GTACTTGCGC GACTGATGGT AACAAAACTC GTAGATAGAA   
  
  
+ TATACTTTCT TTGAAGTGGG AATGATTTGA CCTACTCTAT AAATAATATA AACATCACAT GCATACACTC   
  
  
+ CAACCACTAA GCCACAGAAA GAAATGAAAG AAACAGAAAA TAATTAACAA CCCTATTGGA CTCTTCTCTA   
  
  
+ CACTGCCAGC CTTTCCTTCT TTGTTCTGAG CAAAGAAACA CACCAAAATT GTCAGCCATA AAATTTCTTC   
  
  
+ CTCACAGAAA CTCGTTTGGT TACTGAGAA  

- TGCTTTTGGC TTCGTTATTG TAAGATAAAT ATCCTCTCCT TTCAGCCGTG TCTCCTTCCA CTCTAATCTG   
  
  
- GTTCGTTTGA ATGTGGCGGA TTTTCGATAA CTATTCTTAC TACATTTTCG GGAAATTAAC CGATACCAAA   
  
  
- ACCAGGTTAA TCTTATCTTG TTTGTTGAAT TATTTTATTA CAGTTTTCCT TTCTTTTACT TATTATACTT   
  
  
- ATATATCAAG TTTATGATGA CATTACCACA TGACTTGAAT AAACCAAGTT TTATCATCAT TAATCTCATA   
  
  
- TCTTTTTACT TTATTTTCTT AATTGCTAAT TTATTCTTTA TTATGTTTTG CTATACTTTA ATTATTCATA   
  
  
- TATGTTGTGT ATTAATATAT ATTAATAAAA CCTTTTATAT CCAGGAGTGT TCTGCGGATT CACGATCGTG   
  
  
- GATGAACGAC GACCGATCGC GAATCCAGGA CGAAAAAGAA TATAATATGA GATTTAATAA CATTGAACCC   
  
  
- ATATCTTCAC GTCCGTTTGA TTGGATAGGG TATAAGTGGT CTAAGTGCAA ACGAAACTTT TCTTCAAATA   
  
  
- TCTACTTCTT TTACAATTTT TCTTTTTTCT CACTAATCTC TATTGAACGA ACCTACACCT AAAACGAGCC   
  
  
- CCATTTCTTC CCGCCCGTGT GACTGGATAG GGTCTATATG ATAAAAGTGT AAAAGAAACT TTTCTCCAAA   
  
  
- TGTATATTTC TTTTACACAT TTTTTTTTAT TTTTTATTTT ATTTTATTTC ACTAATTTCT ATTGAAACCT   
  
  
- ACTCCTAAAC CGAGCCCCAT ATCTTCTCAC CTATGAAACT GGATAGAGTT TAAGTGGTCT GAGTACAAAC   
  
  
- GAAACTTTTC TCCAAATATC TATTTCTTTT GCATTTTTTT TTTTTTTATT TATTTTTTTT TACTCACTGA   
  
  
- TTTCTATTGA ATGCACCTAC TCTTAAACTC AACCTCATAT TTTCCTGCCT GTGTAACTGG ATAGGGTCTA   
  
  
- AGTACTCTAA GTGCAAATGG TATCTCCAAA TATCTATTTC TCTTTCATTT TTTTTCTTTT TTGTTACTAA   
  
  
- TTTCTAACAT TGAAAGAGTC GTGGTAGGTC TGATAAGATT ATCTGTAGTG TGTTCAGTAT TATAGTCTTA   
  
  
- AACATCACAA TCAACAAATG CGTGTGTTTA CTATCCGTCG GTAGCTGACA ATGTTTTAAA TATTTTCAGA   
  
  
- GTGTGATTAT CTAATAATTT TCTAATAAAG CATGAACGCG CTGACTACCA TTGTTTTGAG CATCTATCTT   
  
  
- ATATGAAAGA AACTTCACCC TTACTAAACT GGATGAGATA TTTATTATAT TTGTAGTGTA CGTATGTGAG   
  
  
- GTTGGTGATT CGGTGTCTTT CTTTACTTTC TTTGTCTTTT ATTAATTGTT GGGATAACCT GAGAAGAGAT   
  
  
- GTGACGGTCG GAAAGGAAGA AACAAGACTC GTTTCTTTGT GTGGTTTTAA CAGTCGGTAT TTTAAAGAAG   
  
  
- GAGTGTCTTT GAGCAAACCA ATGACTCTT

+     Unnamed\_\_4

| Site Name | Organism | Position | Strand | Matrix score. | sequence | function |
| --- | --- | --- | --- | --- | --- | --- |
| Unnamed\_\_4 | Petroselinum hortense | 33 | - | 4 | CTCC |  |
| Unnamed\_\_4 | Petroselinum hortense | 943 | - | 4 | CTCC |  |
| Unnamed\_\_4 | Petroselinum hortense | 1328 | + | 4 | CTCC |  |

>038667   
+ ACGAAAACCG AAGCAATAAC ATTCTATTTA TAGGAGAGGA AAGTCGGCAC AGAGGAAGGT GAGATTAGAC   
  
  
+ CAAGCAAACT TACACCGCCT AAAAGCTATT GATAAGAATG ATGTAAAAGC CCTTTAATTG GCTATGGTTT   
  
  
+ TGGTCCAATT AGAATAGAAC AAACAACTTA ATAAAATAAT GTCAAAAGGA AAGAAAATGA ATAATATGAA   
  
  
+ TATATAGTTC AAATACTACT GTAATGGTGT ACTGAACTTA TTTGGTTCAA AATAGTAGTA ATTAGAGTAT   
  
  
+ AGAAAAATGA AATAAAAGAA TTAACGATTA AATAAGAAAT AATACAAAAC GATATGAAAT TAATAAGTAT   
  
  
+ ATACAACACA TAATTATATA TAATTATTTT GGAAAATATA GGTCCTCACA AGACGCCTAA GTGCTAGCAC   
  
  
+ CTACTTGCTG CTGGCTAGCG CTTAGGTCCT GCTTTTTCTT ATATTATACT CTAAATTATT GTAACTTGGG   
  
  
+ TATAGAAGTG CAGGCAAACT AACCTATCCC ATATTCACCA GATTCACGTT TGCTTTGAAA AGAAGTTTAT   
  
  
+ AGATGAAGAA AATGTTAAAA AGAAAAAAGA GTGATTAGAG ATAACTTGCT TGGATGTGGA TTTTGCTCGG   
  
  
+ GGTAAAGAAG GGCGGGCACA CTGACCTATC CCAGATATAC TATTTTCACA TTTTCTTTGA AAAGAGGTTT   
  
  
+ ACATATAAAG AAAATGTGTA AAAAAAAATA AAAAATAAAA TAAAATAAAG TGATTAAAGA TAACTTTGGA   
  
  
+ TGAGGATTTG GCTCGGGGTA TAGAAGAGTG GATACTTTGA CCTATCTCAA ATTCACCAGA CTCATGTTTG   
  
  
+ CTTTGAAAAG AGGTTTATAG ATAAAGAAAA CGTAAAAAAA AAAAAAATAA ATAAAAAAAA ATGAGTGACT   
  
  
+ AAAGATAACT TACGTGGATG AGAATTTGAG TTGGAGTATA AAAGGACGGA CACATTGACC TATCCCAGAT   
  
  
+ TCATGAGATT CACGTTTACC ATAGAGGTTT ATAGATAAAG AGAAAGTAAA AAAAAGAAAA AACAATGATT   
  
  
+ AAAGATTGTA ACTTTCTCAG CACCATCCAG ACTATTCTAA TAGACATCAC ACAAGTCATA ATATCAGAAT   
  
  
+ TTGTAGTGTT AGTTGTTTAC GCACACAAAT GATAGGCAGC CATCGACTGT TACAAAATTT ATAAAAGTCT   
  
  
+ CACACTAATA GATTATTAAA AGATTATTTC GTACTTGCGC GACTGATGGT AACAAAACTC GTAGATAGAA   
  
  
+ TATACTTTCT TTGAAGTGGG AATGATTTGA CCTACTCTAT AAATAATATA AACATCACAT GCATACACTC   
  
  
+ CAACCACTAA GCCACAGAAA GAAATGAAAG AAACAGAAAA TAATTAACAA CCCTATTGGA CTCTTCTCTA   
  
  
+ CACTGCCAGC CTTTCCTTCT TTGTTCTGAG CAAAGAAACA CACCAAAATT GTCAGCCATA AAATTTCTTC   
  
  
+ CTCACAGAAA CTCGTTTGGT TACTGAGAA  

- TGCTTTTGGC TTCGTTATTG TAAGATAAAT ATCCTCTCCT TTCAGCCGTG TCTCCTTCCA CTCTAATCTG   
  
  
- GTTCGTTTGA ATGTGGCGGA TTTTCGATAA CTATTCTTAC TACATTTTCG GGAAATTAAC CGATACCAAA   
  
  
- ACCAGGTTAA TCTTATCTTG TTTGTTGAAT TATTTTATTA CAGTTTTCCT TTCTTTTACT TATTATACTT   
  
  
- ATATATCAAG TTTATGATGA CATTACCACA TGACTTGAAT AAACCAAGTT TTATCATCAT TAATCTCATA   
  
  
- TCTTTTTACT TTATTTTCTT AATTGCTAAT TTATTCTTTA TTATGTTTTG CTATACTTTA ATTATTCATA   
  
  
- TATGTTGTGT ATTAATATAT ATTAATAAAA CCTTTTATAT CCAGGAGTGT TCTGCGGATT CACGATCGTG   
  
  
- GATGAACGAC GACCGATCGC GAATCCAGGA CGAAAAAGAA TATAATATGA GATTTAATAA CATTGAACCC   
  
  
- ATATCTTCAC GTCCGTTTGA TTGGATAGGG TATAAGTGGT CTAAGTGCAA ACGAAACTTT TCTTCAAATA   
  
  
- TCTACTTCTT TTACAATTTT TCTTTTTTCT CACTAATCTC TATTGAACGA ACCTACACCT AAAACGAGCC   
  
  
- CCATTTCTTC CCGCCCGTGT GACTGGATAG GGTCTATATG ATAAAAGTGT AAAAGAAACT TTTCTCCAAA   
  
  
- TGTATATTTC TTTTACACAT TTTTTTTTAT TTTTTATTTT ATTTTATTTC ACTAATTTCT ATTGAAACCT   
  
  
- ACTCCTAAAC CGAGCCCCAT ATCTTCTCAC CTATGAAACT GGATAGAGTT TAAGTGGTCT GAGTACAAAC   
  
  
- GAAACTTTTC TCCAAATATC TATTTCTTTT GCATTTTTTT TTTTTTTATT TATTTTTTTT TACTCACTGA   
  
  
- TTTCTATTGA ATGCACCTAC TCTTAAACTC AACCTCATAT TTTCCTGCCT GTGTAACTGG ATAGGGTCTA   
  
  
- AGTACTCTAA GTGCAAATGG TATCTCCAAA TATCTATTTC TCTTTCATTT TTTTTCTTTT TTGTTACTAA   
  
  
- TTTCTAACAT TGAAAGAGTC GTGGTAGGTC TGATAAGATT ATCTGTAGTG TGTTCAGTAT TATAGTCTTA   
  
  
- AACATCACAA TCAACAAATG CGTGTGTTTA CTATCCGTCG GTAGCTGACA ATGTTTTAAA TATTTTCAGA   
  
  
- GTGTGATTAT CTAATAATTT TCTAATAAAG CATGAACGCG CTGACTACCA TTGTTTTGAG CATCTATCTT   
  
  
- ATATGAAAGA AACTTCACCC TTACTAAACT GGATGAGATA TTTATTATAT TTGTAGTGTA CGTATGTGAG   
  
  
- GTTGGTGATT CGGTGTCTTT CTTTACTTTC TTTGTCTTTT ATTAATTGTT GGGATAACCT GAGAAGAGAT   
  
  
- GTGACGGTCG GAAAGGAAGA AACAAGACTC GTTTCTTTGT GTGGTTTTAA CAGTCGGTAT TTTAAAGAAG   
  
  
- GAGTGTCTTT GAGCAAACCA ATGACTCTT

+     W box

| Site Name | Organism | Position | Strand | Matrix score. | sequence | function |
| --- | --- | --- | --- | --- | --- | --- |
| W box | Arabidopsis thaliana | 807 | + | 6 | TTGACC |  |
| W box | Arabidopsis thaliana | 1287 | + | 6 | TTGACC |  |
| W box | Arabidopsis thaliana | 965 | + | 6 | TTGACC |  |

>038667   
+ ACGAAAACCG AAGCAATAAC ATTCTATTTA TAGGAGAGGA AAGTCGGCAC AGAGGAAGGT GAGATTAGAC   
  
  
+ CAAGCAAACT TACACCGCCT AAAAGCTATT GATAAGAATG ATGTAAAAGC CCTTTAATTG GCTATGGTTT   
  
  
+ TGGTCCAATT AGAATAGAAC AAACAACTTA ATAAAATAAT GTCAAAAGGA AAGAAAATGA ATAATATGAA   
  
  
+ TATATAGTTC AAATACTACT GTAATGGTGT ACTGAACTTA TTTGGTTCAA AATAGTAGTA ATTAGAGTAT   
  
  
+ AGAAAAATGA AATAAAAGAA TTAACGATTA AATAAGAAAT AATACAAAAC GATATGAAAT TAATAAGTAT   
  
  
+ ATACAACACA TAATTATATA TAATTATTTT GGAAAATATA GGTCCTCACA AGACGCCTAA GTGCTAGCAC   
  
  
+ CTACTTGCTG CTGGCTAGCG CTTAGGTCCT GCTTTTTCTT ATATTATACT CTAAATTATT GTAACTTGGG   
  
  
+ TATAGAAGTG CAGGCAAACT AACCTATCCC ATATTCACCA GATTCACGTT TGCTTTGAAA AGAAGTTTAT   
  
  
+ AGATGAAGAA AATGTTAAAA AGAAAAAAGA GTGATTAGAG ATAACTTGCT TGGATGTGGA TTTTGCTCGG   
  
  
+ GGTAAAGAAG GGCGGGCACA CTGACCTATC CCAGATATAC TATTTTCACA TTTTCTTTGA AAAGAGGTTT   
  
  
+ ACATATAAAG AAAATGTGTA AAAAAAAATA AAAAATAAAA TAAAATAAAG TGATTAAAGA TAACTTTGGA   
  
  
+ TGAGGATTTG GCTCGGGGTA TAGAAGAGTG GATACTTTGA CCTATCTCAA ATTCACCAGA CTCATGTTTG   
  
  
+ CTTTGAAAAG AGGTTTATAG ATAAAGAAAA CGTAAAAAAA AAAAAAATAA ATAAAAAAAA ATGAGTGACT   
  
  
+ AAAGATAACT TACGTGGATG AGAATTTGAG TTGGAGTATA AAAGGACGGA CACATTGACC TATCCCAGAT   
  
  
+ TCATGAGATT CACGTTTACC ATAGAGGTTT ATAGATAAAG AGAAAGTAAA AAAAAGAAAA AACAATGATT   
  
  
+ AAAGATTGTA ACTTTCTCAG CACCATCCAG ACTATTCTAA TAGACATCAC ACAAGTCATA ATATCAGAAT   
  
  
+ TTGTAGTGTT AGTTGTTTAC GCACACAAAT GATAGGCAGC CATCGACTGT TACAAAATTT ATAAAAGTCT   
  
  
+ CACACTAATA GATTATTAAA AGATTATTTC GTACTTGCGC GACTGATGGT AACAAAACTC GTAGATAGAA   
  
  
+ TATACTTTCT TTGAAGTGGG AATGATTTGA CCTACTCTAT AAATAATATA AACATCACAT GCATACACTC   
  
  
+ CAACCACTAA GCCACAGAAA GAAATGAAAG AAACAGAAAA TAATTAACAA CCCTATTGGA CTCTTCTCTA   
  
  
+ CACTGCCAGC CTTTCCTTCT TTGTTCTGAG CAAAGAAACA CACCAAAATT GTCAGCCATA AAATTTCTTC   
  
  
+ CTCACAGAAA CTCGTTTGGT TACTGAGAA  

- TGCTTTTGGC TTCGTTATTG TAAGATAAAT ATCCTCTCCT TTCAGCCGTG TCTCCTTCCA CTCTAATCTG   
  
  
- GTTCGTTTGA ATGTGGCGGA TTTTCGATAA CTATTCTTAC TACATTTTCG GGAAATTAAC CGATACCAAA   
  
  
- ACCAGGTTAA TCTTATCTTG TTTGTTGAAT TATTTTATTA CAGTTTTCCT TTCTTTTACT TATTATACTT   
  
  
- ATATATCAAG TTTATGATGA CATTACCACA TGACTTGAAT AAACCAAGTT TTATCATCAT TAATCTCATA   
  
  
- TCTTTTTACT TTATTTTCTT AATTGCTAAT TTATTCTTTA TTATGTTTTG CTATACTTTA ATTATTCATA   
  
  
- TATGTTGTGT ATTAATATAT ATTAATAAAA CCTTTTATAT CCAGGAGTGT TCTGCGGATT CACGATCGTG   
  
  
- GATGAACGAC GACCGATCGC GAATCCAGGA CGAAAAAGAA TATAATATGA GATTTAATAA CATTGAACCC   
  
  
- ATATCTTCAC GTCCGTTTGA TTGGATAGGG TATAAGTGGT CTAAGTGCAA ACGAAACTTT TCTTCAAATA   
  
  
- TCTACTTCTT TTACAATTTT TCTTTTTTCT CACTAATCTC TATTGAACGA ACCTACACCT AAAACGAGCC   
  
  
- CCATTTCTTC CCGCCCGTGT GACTGGATAG GGTCTATATG ATAAAAGTGT AAAAGAAACT TTTCTCCAAA   
  
  
- TGTATATTTC TTTTACACAT TTTTTTTTAT TTTTTATTTT ATTTTATTTC ACTAATTTCT ATTGAAACCT   
  
  
- ACTCCTAAAC CGAGCCCCAT ATCTTCTCAC CTATGAAACT GGATAGAGTT TAAGTGGTCT GAGTACAAAC   
  
  
- GAAACTTTTC TCCAAATATC TATTTCTTTT GCATTTTTTT TTTTTTTATT TATTTTTTTT TACTCACTGA   
  
  
- TTTCTATTGA ATGCACCTAC TCTTAAACTC AACCTCATAT TTTCCTGCCT GTGTAACTGG ATAGGGTCTA   
  
  
- AGTACTCTAA GTGCAAATGG TATCTCCAAA TATCTATTTC TCTTTCATTT TTTTTCTTTT TTGTTACTAA   
  
  
- TTTCTAACAT TGAAAGAGTC GTGGTAGGTC TGATAAGATT ATCTGTAGTG TGTTCAGTAT TATAGTCTTA   
  
  
- AACATCACAA TCAACAAATG CGTGTGTTTA CTATCCGTCG GTAGCTGACA ATGTTTTAAA TATTTTCAGA   
  
  
- GTGTGATTAT CTAATAATTT TCTAATAAAG CATGAACGCG CTGACTACCA TTGTTTTGAG CATCTATCTT   
  
  
- ATATGAAAGA AACTTCACCC TTACTAAACT GGATGAGATA TTTATTATAT TTGTAGTGTA CGTATGTGAG   
  
  
- GTTGGTGATT CGGTGTCTTT CTTTACTTTC TTTGTCTTTT ATTAATTGTT GGGATAACCT GAGAAGAGAT   
  
  
- GTGACGGTCG GAAAGGAAGA AACAAGACTC GTTTCTTTGT GTGGTTTTAA CAGTCGGTAT TTTAAAGAAG   
  
  
- GAGTGTCTTT GAGCAAACCA ATGACTCTT

+     WUN-motif

| Site Name | Organism | Position | Strand | Matrix score. | sequence | function |
| --- | --- | --- | --- | --- | --- | --- |
| WUN-motif | Nicotiana glutinosa | 1461 | + | 9 | AAATTTCTT |  |

>038667   
+ ACGAAAACCG AAGCAATAAC ATTCTATTTA TAGGAGAGGA AAGTCGGCAC AGAGGAAGGT GAGATTAGAC   
  
  
+ CAAGCAAACT TACACCGCCT AAAAGCTATT GATAAGAATG ATGTAAAAGC CCTTTAATTG GCTATGGTTT   
  
  
+ TGGTCCAATT AGAATAGAAC AAACAACTTA ATAAAATAAT GTCAAAAGGA AAGAAAATGA ATAATATGAA   
  
  
+ TATATAGTTC AAATACTACT GTAATGGTGT ACTGAACTTA TTTGGTTCAA AATAGTAGTA ATTAGAGTAT   
  
  
+ AGAAAAATGA AATAAAAGAA TTAACGATTA AATAAGAAAT AATACAAAAC GATATGAAAT TAATAAGTAT   
  
  
+ ATACAACACA TAATTATATA TAATTATTTT GGAAAATATA GGTCCTCACA AGACGCCTAA GTGCTAGCAC   
  
  
+ CTACTTGCTG CTGGCTAGCG CTTAGGTCCT GCTTTTTCTT ATATTATACT CTAAATTATT GTAACTTGGG   
  
  
+ TATAGAAGTG CAGGCAAACT AACCTATCCC ATATTCACCA GATTCACGTT TGCTTTGAAA AGAAGTTTAT   
  
  
+ AGATGAAGAA AATGTTAAAA AGAAAAAAGA GTGATTAGAG ATAACTTGCT TGGATGTGGA TTTTGCTCGG   
  
  
+ GGTAAAGAAG GGCGGGCACA CTGACCTATC CCAGATATAC TATTTTCACA TTTTCTTTGA AAAGAGGTTT   
  
  
+ ACATATAAAG AAAATGTGTA AAAAAAAATA AAAAATAAAA TAAAATAAAG TGATTAAAGA TAACTTTGGA   
  
  
+ TGAGGATTTG GCTCGGGGTA TAGAAGAGTG GATACTTTGA CCTATCTCAA ATTCACCAGA CTCATGTTTG   
  
  
+ CTTTGAAAAG AGGTTTATAG ATAAAGAAAA CGTAAAAAAA AAAAAAATAA ATAAAAAAAA ATGAGTGACT   
  
  
+ AAAGATAACT TACGTGGATG AGAATTTGAG TTGGAGTATA AAAGGACGGA CACATTGACC TATCCCAGAT   
  
  
+ TCATGAGATT CACGTTTACC ATAGAGGTTT ATAGATAAAG AGAAAGTAAA AAAAAGAAAA AACAATGATT   
  
  
+ AAAGATTGTA ACTTTCTCAG CACCATCCAG ACTATTCTAA TAGACATCAC ACAAGTCATA ATATCAGAAT   
  
  
+ TTGTAGTGTT AGTTGTTTAC GCACACAAAT GATAGGCAGC CATCGACTGT TACAAAATTT ATAAAAGTCT   
  
  
+ CACACTAATA GATTATTAAA AGATTATTTC GTACTTGCGC GACTGATGGT AACAAAACTC GTAGATAGAA   
  
  
+ TATACTTTCT TTGAAGTGGG AATGATTTGA CCTACTCTAT AAATAATATA AACATCACAT GCATACACTC   
  
  
+ CAACCACTAA GCCACAGAAA GAAATGAAAG AAACAGAAAA TAATTAACAA CCCTATTGGA CTCTTCTCTA   
  
  
+ CACTGCCAGC CTTTCCTTCT TTGTTCTGAG CAAAGAAACA CACCAAAATT GTCAGCCATA AAATTTCTTC   
  
  
+ CTCACAGAAA CTCGTTTGGT TACTGAGAA  

- TGCTTTTGGC TTCGTTATTG TAAGATAAAT ATCCTCTCCT TTCAGCCGTG TCTCCTTCCA CTCTAATCTG   
  
  
- GTTCGTTTGA ATGTGGCGGA TTTTCGATAA CTATTCTTAC TACATTTTCG GGAAATTAAC CGATACCAAA   
  
  
- ACCAGGTTAA TCTTATCTTG TTTGTTGAAT TATTTTATTA CAGTTTTCCT TTCTTTTACT TATTATACTT   
  
  
- ATATATCAAG TTTATGATGA CATTACCACA TGACTTGAAT AAACCAAGTT TTATCATCAT TAATCTCATA   
  
  
- TCTTTTTACT TTATTTTCTT AATTGCTAAT TTATTCTTTA TTATGTTTTG CTATACTTTA ATTATTCATA   
  
  
- TATGTTGTGT ATTAATATAT ATTAATAAAA CCTTTTATAT CCAGGAGTGT TCTGCGGATT CACGATCGTG   
  
  
- GATGAACGAC GACCGATCGC GAATCCAGGA CGAAAAAGAA TATAATATGA GATTTAATAA CATTGAACCC   
  
  
- ATATCTTCAC GTCCGTTTGA TTGGATAGGG TATAAGTGGT CTAAGTGCAA ACGAAACTTT TCTTCAAATA   
  
  
- TCTACTTCTT TTACAATTTT TCTTTTTTCT CACTAATCTC TATTGAACGA ACCTACACCT AAAACGAGCC   
  
  
- CCATTTCTTC CCGCCCGTGT GACTGGATAG GGTCTATATG ATAAAAGTGT AAAAGAAACT TTTCTCCAAA   
  
  
- TGTATATTTC TTTTACACAT TTTTTTTTAT TTTTTATTTT ATTTTATTTC ACTAATTTCT ATTGAAACCT   
  
  
- ACTCCTAAAC CGAGCCCCAT ATCTTCTCAC CTATGAAACT GGATAGAGTT TAAGTGGTCT GAGTACAAAC   
  
  
- GAAACTTTTC TCCAAATATC TATTTCTTTT GCATTTTTTT TTTTTTTATT TATTTTTTTT TACTCACTGA   
  
  
- TTTCTATTGA ATGCACCTAC TCTTAAACTC AACCTCATAT TTTCCTGCCT GTGTAACTGG ATAGGGTCTA   
  
  
- AGTACTCTAA GTGCAAATGG TATCTCCAAA TATCTATTTC TCTTTCATTT TTTTTCTTTT TTGTTACTAA   
  
  
- TTTCTAACAT TGAAAGAGTC GTGGTAGGTC TGATAAGATT ATCTGTAGTG TGTTCAGTAT TATAGTCTTA   
  
  
- AACATCACAA TCAACAAATG CGTGTGTTTA CTATCCGTCG GTAGCTGACA ATGTTTTAAA TATTTTCAGA   
  
  
- GTGTGATTAT CTAATAATTT TCTAATAAAG CATGAACGCG CTGACTACCA TTGTTTTGAG CATCTATCTT   
  
  
- ATATGAAAGA AACTTCACCC TTACTAAACT GGATGAGATA TTTATTATAT TTGTAGTGTA CGTATGTGAG   
  
  
- GTTGGTGATT CGGTGTCTTT CTTTACTTTC TTTGTCTTTT ATTAATTGTT GGGATAACCT GAGAAGAGAT   
  
  
- GTGACGGTCG GAAAGGAAGA AACAAGACTC GTTTCTTTGT GTGGTTTTAA CAGTCGGTAT TTTAAAGAAG   
  
  
- GAGTGTCTTT GAGCAAACCA ATGACTCTT

+     circadian

| Site Name | Organism | Position | Strand | Matrix score. | sequence | function |
| --- | --- | --- | --- | --- | --- | --- |
| circadian | Lycopersicon esculentum | 759 | - | 9 | CAAAGATATC | cis-acting regulatory element involved in circadian control |

>038667   
+ ACGAAAACCG AAGCAATAAC ATTCTATTTA TAGGAGAGGA AAGTCGGCAC AGAGGAAGGT GAGATTAGAC   
  
  
+ CAAGCAAACT TACACCGCCT AAAAGCTATT GATAAGAATG ATGTAAAAGC CCTTTAATTG GCTATGGTTT   
  
  
+ TGGTCCAATT AGAATAGAAC AAACAACTTA ATAAAATAAT GTCAAAAGGA AAGAAAATGA ATAATATGAA   
  
  
+ TATATAGTTC AAATACTACT GTAATGGTGT ACTGAACTTA TTTGGTTCAA AATAGTAGTA ATTAGAGTAT   
  
  
+ AGAAAAATGA AATAAAAGAA TTAACGATTA AATAAGAAAT AATACAAAAC GATATGAAAT TAATAAGTAT   
  
  
+ ATACAACACA TAATTATATA TAATTATTTT GGAAAATATA GGTCCTCACA AGACGCCTAA GTGCTAGCAC   
  
  
+ CTACTTGCTG CTGGCTAGCG CTTAGGTCCT GCTTTTTCTT ATATTATACT CTAAATTATT GTAACTTGGG   
  
  
+ TATAGAAGTG CAGGCAAACT AACCTATCCC ATATTCACCA GATTCACGTT TGCTTTGAAA AGAAGTTTAT   
  
  
+ AGATGAAGAA AATGTTAAAA AGAAAAAAGA GTGATTAGAG ATAACTTGCT TGGATGTGGA TTTTGCTCGG   
  
  
+ GGTAAAGAAG GGCGGGCACA CTGACCTATC CCAGATATAC TATTTTCACA TTTTCTTTGA AAAGAGGTTT   
  
  
+ ACATATAAAG AAAATGTGTA AAAAAAAATA AAAAATAAAA TAAAATAAAG TGATTAAAGA TAACTTTGGA   
  
  
+ TGAGGATTTG GCTCGGGGTA TAGAAGAGTG GATACTTTGA CCTATCTCAA ATTCACCAGA CTCATGTTTG   
  
  
+ CTTTGAAAAG AGGTTTATAG ATAAAGAAAA CGTAAAAAAA AAAAAAATAA ATAAAAAAAA ATGAGTGACT   
  
  
+ AAAGATAACT TACGTGGATG AGAATTTGAG TTGGAGTATA AAAGGACGGA CACATTGACC TATCCCAGAT   
  
  
+ TCATGAGATT CACGTTTACC ATAGAGGTTT ATAGATAAAG AGAAAGTAAA AAAAAGAAAA AACAATGATT   
  
  
+ AAAGATTGTA ACTTTCTCAG CACCATCCAG ACTATTCTAA TAGACATCAC ACAAGTCATA ATATCAGAAT   
  
  
+ TTGTAGTGTT AGTTGTTTAC GCACACAAAT GATAGGCAGC CATCGACTGT TACAAAATTT ATAAAAGTCT   
  
  
+ CACACTAATA GATTATTAAA AGATTATTTC GTACTTGCGC GACTGATGGT AACAAAACTC GTAGATAGAA   
  
  
+ TATACTTTCT TTGAAGTGGG AATGATTTGA CCTACTCTAT AAATAATATA AACATCACAT GCATACACTC   
  
  
+ CAACCACTAA GCCACAGAAA GAAATGAAAG AAACAGAAAA TAATTAACAA CCCTATTGGA CTCTTCTCTA   
  
  
+ CACTGCCAGC CTTTCCTTCT TTGTTCTGAG CAAAGAAACA CACCAAAATT GTCAGCCATA AAATTTCTTC   
  
  
+ CTCACAGAAA CTCGTTTGGT TACTGAGAA  

- TGCTTTTGGC TTCGTTATTG TAAGATAAAT ATCCTCTCCT TTCAGCCGTG TCTCCTTCCA CTCTAATCTG   
  
  
- GTTCGTTTGA ATGTGGCGGA TTTTCGATAA CTATTCTTAC TACATTTTCG GGAAATTAAC CGATACCAAA   
  
  
- ACCAGGTTAA TCTTATCTTG TTTGTTGAAT TATTTTATTA CAGTTTTCCT TTCTTTTACT TATTATACTT   
  
  
- ATATATCAAG TTTATGATGA CATTACCACA TGACTTGAAT AAACCAAGTT TTATCATCAT TAATCTCATA   
  
  
- TCTTTTTACT TTATTTTCTT AATTGCTAAT TTATTCTTTA TTATGTTTTG CTATACTTTA ATTATTCATA   
  
  
- TATGTTGTGT ATTAATATAT ATTAATAAAA CCTTTTATAT CCAGGAGTGT TCTGCGGATT CACGATCGTG   
  
  
- GATGAACGAC GACCGATCGC GAATCCAGGA CGAAAAAGAA TATAATATGA GATTTAATAA CATTGAACCC   
  
  
- ATATCTTCAC GTCCGTTTGA TTGGATAGGG TATAAGTGGT CTAAGTGCAA ACGAAACTTT TCTTCAAATA   
  
  
- TCTACTTCTT TTACAATTTT TCTTTTTTCT CACTAATCTC TATTGAACGA ACCTACACCT AAAACGAGCC   
  
  
- CCATTTCTTC CCGCCCGTGT GACTGGATAG GGTCTATATG ATAAAAGTGT AAAAGAAACT TTTCTCCAAA   
  
  
- TGTATATTTC TTTTACACAT TTTTTTTTAT TTTTTATTTT ATTTTATTTC ACTAATTTCT ATTGAAACCT   
  
  
- ACTCCTAAAC CGAGCCCCAT ATCTTCTCAC CTATGAAACT GGATAGAGTT TAAGTGGTCT GAGTACAAAC   
  
  
- GAAACTTTTC TCCAAATATC TATTTCTTTT GCATTTTTTT TTTTTTTATT TATTTTTTTT TACTCACTGA   
  
  
- TTTCTATTGA ATGCACCTAC TCTTAAACTC AACCTCATAT TTTCCTGCCT GTGTAACTGG ATAGGGTCTA   
  
  
- AGTACTCTAA GTGCAAATGG TATCTCCAAA TATCTATTTC TCTTTCATTT TTTTTCTTTT TTGTTACTAA   
  
  
- TTTCTAACAT TGAAAGAGTC GTGGTAGGTC TGATAAGATT ATCTGTAGTG TGTTCAGTAT TATAGTCTTA   
  
  
- AACATCACAA TCAACAAATG CGTGTGTTTA CTATCCGTCG GTAGCTGACA ATGTTTTAAA TATTTTCAGA   
  
  
- GTGTGATTAT CTAATAATTT TCTAATAAAG CATGAACGCG CTGACTACCA TTGTTTTGAG CATCTATCTT   
  
  
- ATATGAAAGA AACTTCACCC TTACTAAACT GGATGAGATA TTTATTATAT TTGTAGTGTA CGTATGTGAG   
  
  
- GTTGGTGATT CGGTGTCTTT CTTTACTTTC TTTGTCTTTT ATTAATTGTT GGGATAACCT GAGAAGAGAT   
  
  
- GTGACGGTCG GAAAGGAAGA AACAAGACTC GTTTCTTTGT GTGGTTTTAA CAGTCGGTAT TTTAAAGAAG   
  
  
- GAGTGTCTTT GAGCAAACCA ATGACTCTT
