# Supplementary material for: In Silico Genome-Wide Analysis of Respiratory Burst Oxidase Homolog (RBOH) Family Genes in Five Fruit-Producing Trees, and Potential Functional Analysis on Lignification of Stone Cells in Chinese White Pear
Source: Cells. 2019 May 29;8(6):520. doi: 10.3390/cells8060520 (PMC6627160; doi:10.3390/cells8060520)
Supplement: Supplementary file 1 [file cells-08-00520-s001.zip › Supplementary Material/Spreadsheets S2. Numbers of cis-elements in promoter region of PbRBOHs.tar.zip!/PbRBOHA.html]

Content-Type: text/html; charset=ISO-8859-1


PlantCARE


Webmaster Firefox specific output  
To save the result:
click on the frame with the right mouse button and save the source code as a text file with extension .html  
REFERENCE:PlantCARE: a database of plant cis-acting regulatory elements and a portal to tools for in silico analysis of promoter sequences.  
Lescot, M., Déhais, P., Moreau, Y., De Moor, B., Rouzé ,P.,and Rombauts, S.  
Nucleic Acids Res., Database issue(2002), 30(1):325-327.   


---

>018609   
+ CTGTCATCCA CGGCGGTGCG TGGCCAGTGG GCCGATTCTG CCATTTTGTG CAAATTTTGA TATTCTAAAT   
  
  
+ ATGATTTGGT ATCCAATCGA TGTGTTTTGA TTATTAAATC CAATGTTGGT TATTTCGGTT ATTTGCATAT   
  
  
+ TATCGGAAAT GGTTTGGAAT ATGAAATTAT GAACTATGAA AGGCCAAGGG GACCCACCCA TCATGTGGCA   
  
  
+ GTACTCGTTC CCTCCGTGAC TCTAATACTA TGTAGAATCA AAGAGACGGG CCAAATACCC ACTTCCAACA   
  
  
+ ACACTGATTT TGTCTCCAAC TTGGTAATTA CCATATGTGC AATTCGTCAG GTGTGAGGTT TTATCACAAA   
  
  
+ AGGGCTCGGT ATTAGTTAGA GTGAAATTAA GATATTTAAA CTCTTCTTTT ATTAGAGATA CGGTTGATGT   
  
  
+ GGGATGTTTA ACAAGCCCCC TCATGTGGGA TCCAATTAGT GGGTCACACA TGGGGGATTC ACACATCGAC   
  
  
+ AACCACATGA AGTCAAGGGG ACTCACCTTA CACACTGGCC CAAGTGACCC ACCTTCTTCG AGCGGGGTCA   
  
  
+ AGGGGACCCG GACCCACCCA TCACATGACA CTACAGTACG AGCCCACTTC TTGGCTTCGA TACCATGTAT   
  
  
+ AATATTCAAA GAGACTGGTC AAAAATCCAC TTCCAACAAT ATTTATATTA TCCCCAACTT GGTAATTATC   
  
  
+ ACCTACACAT TCCATCAAGT GTGGAATTTT ATCATAAAAG ACATCAGTAT TAGTTAGAGT AAGATTATCA   
  
  
+ TATACTATTA CTTTTGTTTT TCTAGGACAA ATGTGGGATT CAACAATACC ACATATAATA TTATTAAATA   
  
  
+ ATTTGGTTAA GAAAAGAATA ACAAGAAAGT CCATCGTATA ATATGTACGG AACGGTACAC CACATATTAT   
  
  
+ TATACAATTG AAAAAAAGTT TTATTTTCAA GTGTTCATTT AATTATATAA TGATATGTAG TGTTTTACTT   
  
  
+ TTGCTCCGGG CAGATTGAAA AAATTTTCAG AAATTTTTCA ATGTTTCTAA AATAGAAAAC GATACACCAA   
  
  
+ ATGTCATTAT GCAATTAAAA TAACATTTTA AATAAAAAAC AAAATTTTCC TCCACTTTCA TAGTAACATA   
  
  
+ TGATTATTAC GATGTGACTT TAATTTCGAG CCAATTGAAA AATCTTATCA TATGTCTGAG TCGTGAGATA   
  
  
+ TTAGACTAAC ACACATCAAT CCCCGATTCC CCGCCGAATG TTGCTCCTTT GCTGGCCATA CTATTCTACG   
  
  
+ CTCTTCTCAC GCTTTTTGAC TTTTCCGTAT TCAAAGTCGT TTTCGTCTCC CTTCTCACTT CGTTCTGCTG   
  
  
+ CATGCTACCC TCTTCCTCCT CCTCCTCTCT TATATATACC CATCTACCCC TCGAATTTTA ATCCCAAAAT   
  
  
+ ATCTCCAAAC CCACATCTCT TTTCTTTCCA CTGTTTTTCA CAACTTGATG AAATAATTGA ATCAACAAAA   
  
  
+ CATTTGGATA TTGTGAGAAA AATCTGAGC  

- GACAGTAGGT GCCGCCACGC ACCGGTCACC CGGCTAAGAC GGTAAAACAC GTTTAAAACT ATAAGATTTA   
  
  
- TACTAAACCA TAGGTTAGCT ACACAAAACT AATAATTTAG GTTACAACCA ATAAAGCCAA TAAACGTATA   
  
  
- ATAGCCTTTA CCAAACCTTA TACTTTAATA CTTGATACTT TCCGGTTCCC CTGGGTGGGT AGTACACCGT   
  
  
- CATGAGCAAG GGAGGCACTG AGATTATGAT ACATCTTAGT TTCTCTGCCC GGTTTATGGG TGAAGGTTGT   
  
  
- TGTGACTAAA ACAGAGGTTG AACCATTAAT GGTATACACG TTAAGCAGTC CACACTCCAA AATAGTGTTT   
  
  
- TCCCGAGCCA TAATCAATCT CACTTTAATT CTATAAATTT GAGAAGAAAA TAATCTCTAT GCCAACTACA   
  
  
- CCCTACAAAT TGTTCGGGGG AGTACACCCT AGGTTAATCA CCCAGTGTGT ACCCCCTAAG TGTGTAGCTG   
  
  
- TTGGTGTACT TCAGTTCCCC TGAGTGGAAT GTGTGACCGG GTTCACTGGG TGGAAGAAGC TCGCCCCAGT   
  
  
- TCCCCTGGGC CTGGGTGGGT AGTGTACTGT GATGTCATGC TCGGGTGAAG AACCGAAGCT ATGGTACATA   
  
  
- TTATAAGTTT CTCTGACCAG TTTTTAGGTG AAGGTTGTTA TAAATATAAT AGGGGTTGAA CCATTAATAG   
  
  
- TGGATGTGTA AGGTAGTTCA CACCTTAAAA TAGTATTTTC TGTAGTCATA ATCAATCTCA TTCTAATAGT   
  
  
- ATATGATAAT GAAAACAAAA AGATCCTGTT TACACCCTAA GTTGTTATGG TGTATATTAT AATAATTTAT   
  
  
- TAAACCAATT CTTTTCTTAT TGTTCTTTCA GGTAGCATAT TATACATGCC TTGCCATGTG GTGTATAATA   
  
  
- ATATGTTAAC TTTTTTTCAA AATAAAAGTT CACAAGTAAA TTAATATATT ACTATACATC ACAAAATGAA   
  
  
- AACGAGGCCC GTCTAACTTT TTTAAAAGTC TTTAAAAAGT TACAAAGATT TTATCTTTTG CTATGTGGTT   
  
  
- TACAGTAATA CGTTAATTTT ATTGTAAAAT TTATTTTTTG TTTTAAAAGG AGGTGAAAGT ATCATTGTAT   
  
  
- ACTAATAATG CTACACTGAA ATTAAAGCTC GGTTAACTTT TTAGAATAGT ATACAGACTC AGCACTCTAT   
  
  
- AATCTGATTG TGTGTAGTTA GGGGCTAAGG GGCGGCTTAC AACGAGGAAA CGACCGGTAT GATAAGATGC   
  
  
- GAGAAGAGTG CGAAAAACTG AAAAGGCATA AGTTTCAGCA AAAGCAGAGG GAAGAGTGAA GCAAGACGAC   
  
  
- GTACGATGGG AGAAGGAGGA GGAGGAGAGA ATATATATGG GTAGATGGGG AGCTTAAAAT TAGGGTTTTA   
  
  
- TAGAGGTTTG GGTGTAGAGA AAAGAAAGGT GACAAAAAGT GTTGAACTAC TTTATTAACT TAGTTGTTTT   
  
  
- GTAAACCTAT AACACTCTTT TTAGACTCG

  
  
Motifs Found  

+   

| Site Name | Organism | Position | Strand | Matrix score. | sequence | function |
| --- | --- | --- | --- | --- | --- | --- |
|  | organism | 990 | - | 4 | motif\_sequence | short\_function |
|  | organism | 1340 | + | 4 | motif\_sequence | short\_function |
|  | organism | 883 | + | 4 | motif\_sequence | short\_function |
|  | organism | 315 | + | 4 | motif\_sequence | short\_function |
|  | organism | 391 | + | 4 | motif\_sequence | short\_function |
|  | organism | 1401 | + | 4 | motif\_sequence | short\_function |
|  | organism | 1261 | + | 4 | motif\_sequence | short\_function |
|  | organism | 1372 | + | 4 | motif\_sequence | short\_function |

>018609   
+ CTGTCATCCA CGGCGGTGCG TGGCCAGTGG GCCGATTCTG CCATTTTGTG CAAATTTTGA TATTCTAAAT   
  
  
+ ATGATTTGGT ATCCAATCGA TGTGTTTTGA TTATTAAATC CAATGTTGGT TATTTCGGTT ATTTGCATAT   
  
  
+ TATCGGAAAT GGTTTGGAAT ATGAAATTAT GAACTATGAA AGGCCAAGGG GACCCACCCA TCATGTGGCA   
  
  
+ GTACTCGTTC CCTCCGTGAC TCTAATACTA TGTAGAATCA AAGAGACGGG CCAAATACCC ACTTCCAACA   
  
  
+ ACACTGATTT TGTCTCCAAC TTGGTAATTA CCATATGTGC AATTCGTCAG GTGTGAGGTT TTATCACAAA   
  
  
+ AGGGCTCGGT ATTAGTTAGA GTGAAATTAA GATATTTAAA CTCTTCTTTT ATTAGAGATA CGGTTGATGT   
  
  
+ GGGATGTTTA ACAAGCCCCC TCATGTGGGA TCCAATTAGT GGGTCACACA TGGGGGATTC ACACATCGAC   
  
  
+ AACCACATGA AGTCAAGGGG ACTCACCTTA CACACTGGCC CAAGTGACCC ACCTTCTTCG AGCGGGGTCA   
  
  
+ AGGGGACCCG GACCCACCCA TCACATGACA CTACAGTACG AGCCCACTTC TTGGCTTCGA TACCATGTAT   
  
  
+ AATATTCAAA GAGACTGGTC AAAAATCCAC TTCCAACAAT ATTTATATTA TCCCCAACTT GGTAATTATC   
  
  
+ ACCTACACAT TCCATCAAGT GTGGAATTTT ATCATAAAAG ACATCAGTAT TAGTTAGAGT AAGATTATCA   
  
  
+ TATACTATTA CTTTTGTTTT TCTAGGACAA ATGTGGGATT CAACAATACC ACATATAATA TTATTAAATA   
  
  
+ ATTTGGTTAA GAAAAGAATA ACAAGAAAGT CCATCGTATA ATATGTACGG AACGGTACAC CACATATTAT   
  
  
+ TATACAATTG AAAAAAAGTT TTATTTTCAA GTGTTCATTT AATTATATAA TGATATGTAG TGTTTTACTT   
  
  
+ TTGCTCCGGG CAGATTGAAA AAATTTTCAG AAATTTTTCA ATGTTTCTAA AATAGAAAAC GATACACCAA   
  
  
+ ATGTCATTAT GCAATTAAAA TAACATTTTA AATAAAAAAC AAAATTTTCC TCCACTTTCA TAGTAACATA   
  
  
+ TGATTATTAC GATGTGACTT TAATTTCGAG CCAATTGAAA AATCTTATCA TATGTCTGAG TCGTGAGATA   
  
  
+ TTAGACTAAC ACACATCAAT CCCCGATTCC CCGCCGAATG TTGCTCCTTT GCTGGCCATA CTATTCTACG   
  
  
+ CTCTTCTCAC GCTTTTTGAC TTTTCCGTAT TCAAAGTCGT TTTCGTCTCC CTTCTCACTT CGTTCTGCTG   
  
  
+ CATGCTACCC TCTTCCTCCT CCTCCTCTCT TATATATACC CATCTACCCC TCGAATTTTA ATCCCAAAAT   
  
  
+ ATCTCCAAAC CCACATCTCT TTTCTTTCCA CTGTTTTTCA CAACTTGATG AAATAATTGA ATCAACAAAA   
  
  
+ CATTTGGATA TTGTGAGAAA AATCTGAGC  

- GACAGTAGGT GCCGCCACGC ACCGGTCACC CGGCTAAGAC GGTAAAACAC GTTTAAAACT ATAAGATTTA   
  
  
- TACTAAACCA TAGGTTAGCT ACACAAAACT AATAATTTAG GTTACAACCA ATAAAGCCAA TAAACGTATA   
  
  
- ATAGCCTTTA CCAAACCTTA TACTTTAATA CTTGATACTT TCCGGTTCCC CTGGGTGGGT AGTACACCGT   
  
  
- CATGAGCAAG GGAGGCACTG AGATTATGAT ACATCTTAGT TTCTCTGCCC GGTTTATGGG TGAAGGTTGT   
  
  
- TGTGACTAAA ACAGAGGTTG AACCATTAAT GGTATACACG TTAAGCAGTC CACACTCCAA AATAGTGTTT   
  
  
- TCCCGAGCCA TAATCAATCT CACTTTAATT CTATAAATTT GAGAAGAAAA TAATCTCTAT GCCAACTACA   
  
  
- CCCTACAAAT TGTTCGGGGG AGTACACCCT AGGTTAATCA CCCAGTGTGT ACCCCCTAAG TGTGTAGCTG   
  
  
- TTGGTGTACT TCAGTTCCCC TGAGTGGAAT GTGTGACCGG GTTCACTGGG TGGAAGAAGC TCGCCCCAGT   
  
  
- TCCCCTGGGC CTGGGTGGGT AGTGTACTGT GATGTCATGC TCGGGTGAAG AACCGAAGCT ATGGTACATA   
  
  
- TTATAAGTTT CTCTGACCAG TTTTTAGGTG AAGGTTGTTA TAAATATAAT AGGGGTTGAA CCATTAATAG   
  
  
- TGGATGTGTA AGGTAGTTCA CACCTTAAAA TAGTATTTTC TGTAGTCATA ATCAATCTCA TTCTAATAGT   
  
  
- ATATGATAAT GAAAACAAAA AGATCCTGTT TACACCCTAA GTTGTTATGG TGTATATTAT AATAATTTAT   
  
  
- TAAACCAATT CTTTTCTTAT TGTTCTTTCA GGTAGCATAT TATACATGCC TTGCCATGTG GTGTATAATA   
  
  
- ATATGTTAAC TTTTTTTCAA AATAAAAGTT CACAAGTAAA TTAATATATT ACTATACATC ACAAAATGAA   
  
  
- AACGAGGCCC GTCTAACTTT TTTAAAAGTC TTTAAAAAGT TACAAAGATT TTATCTTTTG CTATGTGGTT   
  
  
- TACAGTAATA CGTTAATTTT ATTGTAAAAT TTATTTTTTG TTTTAAAAGG AGGTGAAAGT ATCATTGTAT   
  
  
- ACTAATAATG CTACACTGAA ATTAAAGCTC GGTTAACTTT TTAGAATAGT ATACAGACTC AGCACTCTAT   
  
  
- AATCTGATTG TGTGTAGTTA GGGGCTAAGG GGCGGCTTAC AACGAGGAAA CGACCGGTAT GATAAGATGC   
  
  
- GAGAAGAGTG CGAAAAACTG AAAAGGCATA AGTTTCAGCA AAAGCAGAGG GAAGAGTGAA GCAAGACGAC   
  
  
- GTACGATGGG AGAAGGAGGA GGAGGAGAGA ATATATATGG GTAGATGGGG AGCTTAAAAT TAGGGTTTTA   
  
  
- TAGAGGTTTG GGTGTAGAGA AAAGAAAGGT GACAAAAAGT GTTGAACTAC TTTATTAACT TAGTTGTTTT   
  
  
- GTAAACCTAT AACACTCTTT TTAGACTCG

+     3-AF1 binding site

| Site Name | Organism | Position | Strand | Matrix score. | sequence | function |
| --- | --- | --- | --- | --- | --- | --- |
| 3-AF1 binding site | Solanum tuberosum | 1352 | - | 10 | TAAGAGAGGAA | light responsive element |

>018609   
+ CTGTCATCCA CGGCGGTGCG TGGCCAGTGG GCCGATTCTG CCATTTTGTG CAAATTTTGA TATTCTAAAT   
  
  
+ ATGATTTGGT ATCCAATCGA TGTGTTTTGA TTATTAAATC CAATGTTGGT TATTTCGGTT ATTTGCATAT   
  
  
+ TATCGGAAAT GGTTTGGAAT ATGAAATTAT GAACTATGAA AGGCCAAGGG GACCCACCCA TCATGTGGCA   
  
  
+ GTACTCGTTC CCTCCGTGAC TCTAATACTA TGTAGAATCA AAGAGACGGG CCAAATACCC ACTTCCAACA   
  
  
+ ACACTGATTT TGTCTCCAAC TTGGTAATTA CCATATGTGC AATTCGTCAG GTGTGAGGTT TTATCACAAA   
  
  
+ AGGGCTCGGT ATTAGTTAGA GTGAAATTAA GATATTTAAA CTCTTCTTTT ATTAGAGATA CGGTTGATGT   
  
  
+ GGGATGTTTA ACAAGCCCCC TCATGTGGGA TCCAATTAGT GGGTCACACA TGGGGGATTC ACACATCGAC   
  
  
+ AACCACATGA AGTCAAGGGG ACTCACCTTA CACACTGGCC CAAGTGACCC ACCTTCTTCG AGCGGGGTCA   
  
  
+ AGGGGACCCG GACCCACCCA TCACATGACA CTACAGTACG AGCCCACTTC TTGGCTTCGA TACCATGTAT   
  
  
+ AATATTCAAA GAGACTGGTC AAAAATCCAC TTCCAACAAT ATTTATATTA TCCCCAACTT GGTAATTATC   
  
  
+ ACCTACACAT TCCATCAAGT GTGGAATTTT ATCATAAAAG ACATCAGTAT TAGTTAGAGT AAGATTATCA   
  
  
+ TATACTATTA CTTTTGTTTT TCTAGGACAA ATGTGGGATT CAACAATACC ACATATAATA TTATTAAATA   
  
  
+ ATTTGGTTAA GAAAAGAATA ACAAGAAAGT CCATCGTATA ATATGTACGG AACGGTACAC CACATATTAT   
  
  
+ TATACAATTG AAAAAAAGTT TTATTTTCAA GTGTTCATTT AATTATATAA TGATATGTAG TGTTTTACTT   
  
  
+ TTGCTCCGGG CAGATTGAAA AAATTTTCAG AAATTTTTCA ATGTTTCTAA AATAGAAAAC GATACACCAA   
  
  
+ ATGTCATTAT GCAATTAAAA TAACATTTTA AATAAAAAAC AAAATTTTCC TCCACTTTCA TAGTAACATA   
  
  
+ TGATTATTAC GATGTGACTT TAATTTCGAG CCAATTGAAA AATCTTATCA TATGTCTGAG TCGTGAGATA   
  
  
+ TTAGACTAAC ACACATCAAT CCCCGATTCC CCGCCGAATG TTGCTCCTTT GCTGGCCATA CTATTCTACG   
  
  
+ CTCTTCTCAC GCTTTTTGAC TTTTCCGTAT TCAAAGTCGT TTTCGTCTCC CTTCTCACTT CGTTCTGCTG   
  
  
+ CATGCTACCC TCTTCCTCCT CCTCCTCTCT TATATATACC CATCTACCCC TCGAATTTTA ATCCCAAAAT   
  
  
+ ATCTCCAAAC CCACATCTCT TTTCTTTCCA CTGTTTTTCA CAACTTGATG AAATAATTGA ATCAACAAAA   
  
  
+ CATTTGGATA TTGTGAGAAA AATCTGAGC  

- GACAGTAGGT GCCGCCACGC ACCGGTCACC CGGCTAAGAC GGTAAAACAC GTTTAAAACT ATAAGATTTA   
  
  
- TACTAAACCA TAGGTTAGCT ACACAAAACT AATAATTTAG GTTACAACCA ATAAAGCCAA TAAACGTATA   
  
  
- ATAGCCTTTA CCAAACCTTA TACTTTAATA CTTGATACTT TCCGGTTCCC CTGGGTGGGT AGTACACCGT   
  
  
- CATGAGCAAG GGAGGCACTG AGATTATGAT ACATCTTAGT TTCTCTGCCC GGTTTATGGG TGAAGGTTGT   
  
  
- TGTGACTAAA ACAGAGGTTG AACCATTAAT GGTATACACG TTAAGCAGTC CACACTCCAA AATAGTGTTT   
  
  
- TCCCGAGCCA TAATCAATCT CACTTTAATT CTATAAATTT GAGAAGAAAA TAATCTCTAT GCCAACTACA   
  
  
- CCCTACAAAT TGTTCGGGGG AGTACACCCT AGGTTAATCA CCCAGTGTGT ACCCCCTAAG TGTGTAGCTG   
  
  
- TTGGTGTACT TCAGTTCCCC TGAGTGGAAT GTGTGACCGG GTTCACTGGG TGGAAGAAGC TCGCCCCAGT   
  
  
- TCCCCTGGGC CTGGGTGGGT AGTGTACTGT GATGTCATGC TCGGGTGAAG AACCGAAGCT ATGGTACATA   
  
  
- TTATAAGTTT CTCTGACCAG TTTTTAGGTG AAGGTTGTTA TAAATATAAT AGGGGTTGAA CCATTAATAG   
  
  
- TGGATGTGTA AGGTAGTTCA CACCTTAAAA TAGTATTTTC TGTAGTCATA ATCAATCTCA TTCTAATAGT   
  
  
- ATATGATAAT GAAAACAAAA AGATCCTGTT TACACCCTAA GTTGTTATGG TGTATATTAT AATAATTTAT   
  
  
- TAAACCAATT CTTTTCTTAT TGTTCTTTCA GGTAGCATAT TATACATGCC TTGCCATGTG GTGTATAATA   
  
  
- ATATGTTAAC TTTTTTTCAA AATAAAAGTT CACAAGTAAA TTAATATATT ACTATACATC ACAAAATGAA   
  
  
- AACGAGGCCC GTCTAACTTT TTTAAAAGTC TTTAAAAAGT TACAAAGATT TTATCTTTTG CTATGTGGTT   
  
  
- TACAGTAATA CGTTAATTTT ATTGTAAAAT TTATTTTTTG TTTTAAAAGG AGGTGAAAGT ATCATTGTAT   
  
  
- ACTAATAATG CTACACTGAA ATTAAAGCTC GGTTAACTTT TTAGAATAGT ATACAGACTC AGCACTCTAT   
  
  
- AATCTGATTG TGTGTAGTTA GGGGCTAAGG GGCGGCTTAC AACGAGGAAA CGACCGGTAT GATAAGATGC   
  
  
- GAGAAGAGTG CGAAAAACTG AAAAGGCATA AGTTTCAGCA AAAGCAGAGG GAAGAGTGAA GCAAGACGAC   
  
  
- GTACGATGGG AGAAGGAGGA GGAGGAGAGA ATATATATGG GTAGATGGGG AGCTTAAAAT TAGGGTTTTA   
  
  
- TAGAGGTTTG GGTGTAGAGA AAAGAAAGGT GACAAAAAGT GTTGAACTAC TTTATTAACT TAGTTGTTTT   
  
  
- GTAAACCTAT AACACTCTTT TTAGACTCG

+     AAGAA-motif

| Site Name | Organism | Position | Strand | Matrix score. | sequence | function |
| --- | --- | --- | --- | --- | --- | --- |
| AAGAA-motif | Avena sativa | 1422 | - | 7 | GAAAGAA |  |
| AAGAA-motif | Avena sativa | 845 | + | 9 | gGTAAAGAAA |  |

>018609   
+ CTGTCATCCA CGGCGGTGCG TGGCCAGTGG GCCGATTCTG CCATTTTGTG CAAATTTTGA TATTCTAAAT   
  
  
+ ATGATTTGGT ATCCAATCGA TGTGTTTTGA TTATTAAATC CAATGTTGGT TATTTCGGTT ATTTGCATAT   
  
  
+ TATCGGAAAT GGTTTGGAAT ATGAAATTAT GAACTATGAA AGGCCAAGGG GACCCACCCA TCATGTGGCA   
  
  
+ GTACTCGTTC CCTCCGTGAC TCTAATACTA TGTAGAATCA AAGAGACGGG CCAAATACCC ACTTCCAACA   
  
  
+ ACACTGATTT TGTCTCCAAC TTGGTAATTA CCATATGTGC AATTCGTCAG GTGTGAGGTT TTATCACAAA   
  
  
+ AGGGCTCGGT ATTAGTTAGA GTGAAATTAA GATATTTAAA CTCTTCTTTT ATTAGAGATA CGGTTGATGT   
  
  
+ GGGATGTTTA ACAAGCCCCC TCATGTGGGA TCCAATTAGT GGGTCACACA TGGGGGATTC ACACATCGAC   
  
  
+ AACCACATGA AGTCAAGGGG ACTCACCTTA CACACTGGCC CAAGTGACCC ACCTTCTTCG AGCGGGGTCA   
  
  
+ AGGGGACCCG GACCCACCCA TCACATGACA CTACAGTACG AGCCCACTTC TTGGCTTCGA TACCATGTAT   
  
  
+ AATATTCAAA GAGACTGGTC AAAAATCCAC TTCCAACAAT ATTTATATTA TCCCCAACTT GGTAATTATC   
  
  
+ ACCTACACAT TCCATCAAGT GTGGAATTTT ATCATAAAAG ACATCAGTAT TAGTTAGAGT AAGATTATCA   
  
  
+ TATACTATTA CTTTTGTTTT TCTAGGACAA ATGTGGGATT CAACAATACC ACATATAATA TTATTAAATA   
  
  
+ ATTTGGTTAA GAAAAGAATA ACAAGAAAGT CCATCGTATA ATATGTACGG AACGGTACAC CACATATTAT   
  
  
+ TATACAATTG AAAAAAAGTT TTATTTTCAA GTGTTCATTT AATTATATAA TGATATGTAG TGTTTTACTT   
  
  
+ TTGCTCCGGG CAGATTGAAA AAATTTTCAG AAATTTTTCA ATGTTTCTAA AATAGAAAAC GATACACCAA   
  
  
+ ATGTCATTAT GCAATTAAAA TAACATTTTA AATAAAAAAC AAAATTTTCC TCCACTTTCA TAGTAACATA   
  
  
+ TGATTATTAC GATGTGACTT TAATTTCGAG CCAATTGAAA AATCTTATCA TATGTCTGAG TCGTGAGATA   
  
  
+ TTAGACTAAC ACACATCAAT CCCCGATTCC CCGCCGAATG TTGCTCCTTT GCTGGCCATA CTATTCTACG   
  
  
+ CTCTTCTCAC GCTTTTTGAC TTTTCCGTAT TCAAAGTCGT TTTCGTCTCC CTTCTCACTT CGTTCTGCTG   
  
  
+ CATGCTACCC TCTTCCTCCT CCTCCTCTCT TATATATACC CATCTACCCC TCGAATTTTA ATCCCAAAAT   
  
  
+ ATCTCCAAAC CCACATCTCT TTTCTTTCCA CTGTTTTTCA CAACTTGATG AAATAATTGA ATCAACAAAA   
  
  
+ CATTTGGATA TTGTGAGAAA AATCTGAGC  

- GACAGTAGGT GCCGCCACGC ACCGGTCACC CGGCTAAGAC GGTAAAACAC GTTTAAAACT ATAAGATTTA   
  
  
- TACTAAACCA TAGGTTAGCT ACACAAAACT AATAATTTAG GTTACAACCA ATAAAGCCAA TAAACGTATA   
  
  
- ATAGCCTTTA CCAAACCTTA TACTTTAATA CTTGATACTT TCCGGTTCCC CTGGGTGGGT AGTACACCGT   
  
  
- CATGAGCAAG GGAGGCACTG AGATTATGAT ACATCTTAGT TTCTCTGCCC GGTTTATGGG TGAAGGTTGT   
  
  
- TGTGACTAAA ACAGAGGTTG AACCATTAAT GGTATACACG TTAAGCAGTC CACACTCCAA AATAGTGTTT   
  
  
- TCCCGAGCCA TAATCAATCT CACTTTAATT CTATAAATTT GAGAAGAAAA TAATCTCTAT GCCAACTACA   
  
  
- CCCTACAAAT TGTTCGGGGG AGTACACCCT AGGTTAATCA CCCAGTGTGT ACCCCCTAAG TGTGTAGCTG   
  
  
- TTGGTGTACT TCAGTTCCCC TGAGTGGAAT GTGTGACCGG GTTCACTGGG TGGAAGAAGC TCGCCCCAGT   
  
  
- TCCCCTGGGC CTGGGTGGGT AGTGTACTGT GATGTCATGC TCGGGTGAAG AACCGAAGCT ATGGTACATA   
  
  
- TTATAAGTTT CTCTGACCAG TTTTTAGGTG AAGGTTGTTA TAAATATAAT AGGGGTTGAA CCATTAATAG   
  
  
- TGGATGTGTA AGGTAGTTCA CACCTTAAAA TAGTATTTTC TGTAGTCATA ATCAATCTCA TTCTAATAGT   
  
  
- ATATGATAAT GAAAACAAAA AGATCCTGTT TACACCCTAA GTTGTTATGG TGTATATTAT AATAATTTAT   
  
  
- TAAACCAATT CTTTTCTTAT TGTTCTTTCA GGTAGCATAT TATACATGCC TTGCCATGTG GTGTATAATA   
  
  
- ATATGTTAAC TTTTTTTCAA AATAAAAGTT CACAAGTAAA TTAATATATT ACTATACATC ACAAAATGAA   
  
  
- AACGAGGCCC GTCTAACTTT TTTAAAAGTC TTTAAAAAGT TACAAAGATT TTATCTTTTG CTATGTGGTT   
  
  
- TACAGTAATA CGTTAATTTT ATTGTAAAAT TTATTTTTTG TTTTAAAAGG AGGTGAAAGT ATCATTGTAT   
  
  
- ACTAATAATG CTACACTGAA ATTAAAGCTC GGTTAACTTT TTAGAATAGT ATACAGACTC AGCACTCTAT   
  
  
- AATCTGATTG TGTGTAGTTA GGGGCTAAGG GGCGGCTTAC AACGAGGAAA CGACCGGTAT GATAAGATGC   
  
  
- GAGAAGAGTG CGAAAAACTG AAAAGGCATA AGTTTCAGCA AAAGCAGAGG GAAGAGTGAA GCAAGACGAC   
  
  
- GTACGATGGG AGAAGGAGGA GGAGGAGAGA ATATATATGG GTAGATGGGG AGCTTAAAAT TAGGGTTTTA   
  
  
- TAGAGGTTTG GGTGTAGAGA AAAGAAAGGT GACAAAAAGT GTTGAACTAC TTTATTAACT TAGTTGTTTT   
  
  
- GTAAACCTAT AACACTCTTT TTAGACTCG

+     ARE

| Site Name | Organism | Position | Strand | Matrix score. | sequence | function |
| --- | --- | --- | --- | --- | --- | --- |
| ARE | Zea mays | 150 | - | 6 | AAACCA | cis-acting regulatory element essential for the anaerobic induction |

>018609   
+ CTGTCATCCA CGGCGGTGCG TGGCCAGTGG GCCGATTCTG CCATTTTGTG CAAATTTTGA TATTCTAAAT   
  
  
+ ATGATTTGGT ATCCAATCGA TGTGTTTTGA TTATTAAATC CAATGTTGGT TATTTCGGTT ATTTGCATAT   
  
  
+ TATCGGAAAT GGTTTGGAAT ATGAAATTAT GAACTATGAA AGGCCAAGGG GACCCACCCA TCATGTGGCA   
  
  
+ GTACTCGTTC CCTCCGTGAC TCTAATACTA TGTAGAATCA AAGAGACGGG CCAAATACCC ACTTCCAACA   
  
  
+ ACACTGATTT TGTCTCCAAC TTGGTAATTA CCATATGTGC AATTCGTCAG GTGTGAGGTT TTATCACAAA   
  
  
+ AGGGCTCGGT ATTAGTTAGA GTGAAATTAA GATATTTAAA CTCTTCTTTT ATTAGAGATA CGGTTGATGT   
  
  
+ GGGATGTTTA ACAAGCCCCC TCATGTGGGA TCCAATTAGT GGGTCACACA TGGGGGATTC ACACATCGAC   
  
  
+ AACCACATGA AGTCAAGGGG ACTCACCTTA CACACTGGCC CAAGTGACCC ACCTTCTTCG AGCGGGGTCA   
  
  
+ AGGGGACCCG GACCCACCCA TCACATGACA CTACAGTACG AGCCCACTTC TTGGCTTCGA TACCATGTAT   
  
  
+ AATATTCAAA GAGACTGGTC AAAAATCCAC TTCCAACAAT ATTTATATTA TCCCCAACTT GGTAATTATC   
  
  
+ ACCTACACAT TCCATCAAGT GTGGAATTTT ATCATAAAAG ACATCAGTAT TAGTTAGAGT AAGATTATCA   
  
  
+ TATACTATTA CTTTTGTTTT TCTAGGACAA ATGTGGGATT CAACAATACC ACATATAATA TTATTAAATA   
  
  
+ ATTTGGTTAA GAAAAGAATA ACAAGAAAGT CCATCGTATA ATATGTACGG AACGGTACAC CACATATTAT   
  
  
+ TATACAATTG AAAAAAAGTT TTATTTTCAA GTGTTCATTT AATTATATAA TGATATGTAG TGTTTTACTT   
  
  
+ TTGCTCCGGG CAGATTGAAA AAATTTTCAG AAATTTTTCA ATGTTTCTAA AATAGAAAAC GATACACCAA   
  
  
+ ATGTCATTAT GCAATTAAAA TAACATTTTA AATAAAAAAC AAAATTTTCC TCCACTTTCA TAGTAACATA   
  
  
+ TGATTATTAC GATGTGACTT TAATTTCGAG CCAATTGAAA AATCTTATCA TATGTCTGAG TCGTGAGATA   
  
  
+ TTAGACTAAC ACACATCAAT CCCCGATTCC CCGCCGAATG TTGCTCCTTT GCTGGCCATA CTATTCTACG   
  
  
+ CTCTTCTCAC GCTTTTTGAC TTTTCCGTAT TCAAAGTCGT TTTCGTCTCC CTTCTCACTT CGTTCTGCTG   
  
  
+ CATGCTACCC TCTTCCTCCT CCTCCTCTCT TATATATACC CATCTACCCC TCGAATTTTA ATCCCAAAAT   
  
  
+ ATCTCCAAAC CCACATCTCT TTTCTTTCCA CTGTTTTTCA CAACTTGATG AAATAATTGA ATCAACAAAA   
  
  
+ CATTTGGATA TTGTGAGAAA AATCTGAGC  

- GACAGTAGGT GCCGCCACGC ACCGGTCACC CGGCTAAGAC GGTAAAACAC GTTTAAAACT ATAAGATTTA   
  
  
- TACTAAACCA TAGGTTAGCT ACACAAAACT AATAATTTAG GTTACAACCA ATAAAGCCAA TAAACGTATA   
  
  
- ATAGCCTTTA CCAAACCTTA TACTTTAATA CTTGATACTT TCCGGTTCCC CTGGGTGGGT AGTACACCGT   
  
  
- CATGAGCAAG GGAGGCACTG AGATTATGAT ACATCTTAGT TTCTCTGCCC GGTTTATGGG TGAAGGTTGT   
  
  
- TGTGACTAAA ACAGAGGTTG AACCATTAAT GGTATACACG TTAAGCAGTC CACACTCCAA AATAGTGTTT   
  
  
- TCCCGAGCCA TAATCAATCT CACTTTAATT CTATAAATTT GAGAAGAAAA TAATCTCTAT GCCAACTACA   
  
  
- CCCTACAAAT TGTTCGGGGG AGTACACCCT AGGTTAATCA CCCAGTGTGT ACCCCCTAAG TGTGTAGCTG   
  
  
- TTGGTGTACT TCAGTTCCCC TGAGTGGAAT GTGTGACCGG GTTCACTGGG TGGAAGAAGC TCGCCCCAGT   
  
  
- TCCCCTGGGC CTGGGTGGGT AGTGTACTGT GATGTCATGC TCGGGTGAAG AACCGAAGCT ATGGTACATA   
  
  
- TTATAAGTTT CTCTGACCAG TTTTTAGGTG AAGGTTGTTA TAAATATAAT AGGGGTTGAA CCATTAATAG   
  
  
- TGGATGTGTA AGGTAGTTCA CACCTTAAAA TAGTATTTTC TGTAGTCATA ATCAATCTCA TTCTAATAGT   
  
  
- ATATGATAAT GAAAACAAAA AGATCCTGTT TACACCCTAA GTTGTTATGG TGTATATTAT AATAATTTAT   
  
  
- TAAACCAATT CTTTTCTTAT TGTTCTTTCA GGTAGCATAT TATACATGCC TTGCCATGTG GTGTATAATA   
  
  
- ATATGTTAAC TTTTTTTCAA AATAAAAGTT CACAAGTAAA TTAATATATT ACTATACATC ACAAAATGAA   
  
  
- AACGAGGCCC GTCTAACTTT TTTAAAAGTC TTTAAAAAGT TACAAAGATT TTATCTTTTG CTATGTGGTT   
  
  
- TACAGTAATA CGTTAATTTT ATTGTAAAAT TTATTTTTTG TTTTAAAAGG AGGTGAAAGT ATCATTGTAT   
  
  
- ACTAATAATG CTACACTGAA ATTAAAGCTC GGTTAACTTT TTAGAATAGT ATACAGACTC AGCACTCTAT   
  
  
- AATCTGATTG TGTGTAGTTA GGGGCTAAGG GGCGGCTTAC AACGAGGAAA CGACCGGTAT GATAAGATGC   
  
  
- GAGAAGAGTG CGAAAAACTG AAAAGGCATA AGTTTCAGCA AAAGCAGAGG GAAGAGTGAA GCAAGACGAC   
  
  
- GTACGATGGG AGAAGGAGGA GGAGGAGAGA ATATATATGG GTAGATGGGG AGCTTAAAAT TAGGGTTTTA   
  
  
- TAGAGGTTTG GGTGTAGAGA AAAGAAAGGT GACAAAAAGT GTTGAACTAC TTTATTAACT TAGTTGTTTT   
  
  
- GTAAACCTAT AACACTCTTT TTAGACTCG

+     AT~TATA-box

| Site Name | Organism | Position | Strand | Matrix score. | sequence | function |
| --- | --- | --- | --- | --- | --- | --- |
| AT~TATA-box | Arabidopsis thaliana | 1363 | - | 6 | TATATA |  |
| AT~TATA-box | Arabidopsis thaliana | 1361 | - | 6 | TATATA |  |
| AT~TATA-box | Arabidopsis thaliana | 954 | - | 6 | TATATA |  |

>018609   
+ CTGTCATCCA CGGCGGTGCG TGGCCAGTGG GCCGATTCTG CCATTTTGTG CAAATTTTGA TATTCTAAAT   
  
  
+ ATGATTTGGT ATCCAATCGA TGTGTTTTGA TTATTAAATC CAATGTTGGT TATTTCGGTT ATTTGCATAT   
  
  
+ TATCGGAAAT GGTTTGGAAT ATGAAATTAT GAACTATGAA AGGCCAAGGG GACCCACCCA TCATGTGGCA   
  
  
+ GTACTCGTTC CCTCCGTGAC TCTAATACTA TGTAGAATCA AAGAGACGGG CCAAATACCC ACTTCCAACA   
  
  
+ ACACTGATTT TGTCTCCAAC TTGGTAATTA CCATATGTGC AATTCGTCAG GTGTGAGGTT TTATCACAAA   
  
  
+ AGGGCTCGGT ATTAGTTAGA GTGAAATTAA GATATTTAAA CTCTTCTTTT ATTAGAGATA CGGTTGATGT   
  
  
+ GGGATGTTTA ACAAGCCCCC TCATGTGGGA TCCAATTAGT GGGTCACACA TGGGGGATTC ACACATCGAC   
  
  
+ AACCACATGA AGTCAAGGGG ACTCACCTTA CACACTGGCC CAAGTGACCC ACCTTCTTCG AGCGGGGTCA   
  
  
+ AGGGGACCCG GACCCACCCA TCACATGACA CTACAGTACG AGCCCACTTC TTGGCTTCGA TACCATGTAT   
  
  
+ AATATTCAAA GAGACTGGTC AAAAATCCAC TTCCAACAAT ATTTATATTA TCCCCAACTT GGTAATTATC   
  
  
+ ACCTACACAT TCCATCAAGT GTGGAATTTT ATCATAAAAG ACATCAGTAT TAGTTAGAGT AAGATTATCA   
  
  
+ TATACTATTA CTTTTGTTTT TCTAGGACAA ATGTGGGATT CAACAATACC ACATATAATA TTATTAAATA   
  
  
+ ATTTGGTTAA GAAAAGAATA ACAAGAAAGT CCATCGTATA ATATGTACGG AACGGTACAC CACATATTAT   
  
  
+ TATACAATTG AAAAAAAGTT TTATTTTCAA GTGTTCATTT AATTATATAA TGATATGTAG TGTTTTACTT   
  
  
+ TTGCTCCGGG CAGATTGAAA AAATTTTCAG AAATTTTTCA ATGTTTCTAA AATAGAAAAC GATACACCAA   
  
  
+ ATGTCATTAT GCAATTAAAA TAACATTTTA AATAAAAAAC AAAATTTTCC TCCACTTTCA TAGTAACATA   
  
  
+ TGATTATTAC GATGTGACTT TAATTTCGAG CCAATTGAAA AATCTTATCA TATGTCTGAG TCGTGAGATA   
  
  
+ TTAGACTAAC ACACATCAAT CCCCGATTCC CCGCCGAATG TTGCTCCTTT GCTGGCCATA CTATTCTACG   
  
  
+ CTCTTCTCAC GCTTTTTGAC TTTTCCGTAT TCAAAGTCGT TTTCGTCTCC CTTCTCACTT CGTTCTGCTG   
  
  
+ CATGCTACCC TCTTCCTCCT CCTCCTCTCT TATATATACC CATCTACCCC TCGAATTTTA ATCCCAAAAT   
  
  
+ ATCTCCAAAC CCACATCTCT TTTCTTTCCA CTGTTTTTCA CAACTTGATG AAATAATTGA ATCAACAAAA   
  
  
+ CATTTGGATA TTGTGAGAAA AATCTGAGC  

- GACAGTAGGT GCCGCCACGC ACCGGTCACC CGGCTAAGAC GGTAAAACAC GTTTAAAACT ATAAGATTTA   
  
  
- TACTAAACCA TAGGTTAGCT ACACAAAACT AATAATTTAG GTTACAACCA ATAAAGCCAA TAAACGTATA   
  
  
- ATAGCCTTTA CCAAACCTTA TACTTTAATA CTTGATACTT TCCGGTTCCC CTGGGTGGGT AGTACACCGT   
  
  
- CATGAGCAAG GGAGGCACTG AGATTATGAT ACATCTTAGT TTCTCTGCCC GGTTTATGGG TGAAGGTTGT   
  
  
- TGTGACTAAA ACAGAGGTTG AACCATTAAT GGTATACACG TTAAGCAGTC CACACTCCAA AATAGTGTTT   
  
  
- TCCCGAGCCA TAATCAATCT CACTTTAATT CTATAAATTT GAGAAGAAAA TAATCTCTAT GCCAACTACA   
  
  
- CCCTACAAAT TGTTCGGGGG AGTACACCCT AGGTTAATCA CCCAGTGTGT ACCCCCTAAG TGTGTAGCTG   
  
  
- TTGGTGTACT TCAGTTCCCC TGAGTGGAAT GTGTGACCGG GTTCACTGGG TGGAAGAAGC TCGCCCCAGT   
  
  
- TCCCCTGGGC CTGGGTGGGT AGTGTACTGT GATGTCATGC TCGGGTGAAG AACCGAAGCT ATGGTACATA   
  
  
- TTATAAGTTT CTCTGACCAG TTTTTAGGTG AAGGTTGTTA TAAATATAAT AGGGGTTGAA CCATTAATAG   
  
  
- TGGATGTGTA AGGTAGTTCA CACCTTAAAA TAGTATTTTC TGTAGTCATA ATCAATCTCA TTCTAATAGT   
  
  
- ATATGATAAT GAAAACAAAA AGATCCTGTT TACACCCTAA GTTGTTATGG TGTATATTAT AATAATTTAT   
  
  
- TAAACCAATT CTTTTCTTAT TGTTCTTTCA GGTAGCATAT TATACATGCC TTGCCATGTG GTGTATAATA   
  
  
- ATATGTTAAC TTTTTTTCAA AATAAAAGTT CACAAGTAAA TTAATATATT ACTATACATC ACAAAATGAA   
  
  
- AACGAGGCCC GTCTAACTTT TTTAAAAGTC TTTAAAAAGT TACAAAGATT TTATCTTTTG CTATGTGGTT   
  
  
- TACAGTAATA CGTTAATTTT ATTGTAAAAT TTATTTTTTG TTTTAAAAGG AGGTGAAAGT ATCATTGTAT   
  
  
- ACTAATAATG CTACACTGAA ATTAAAGCTC GGTTAACTTT TTAGAATAGT ATACAGACTC AGCACTCTAT   
  
  
- AATCTGATTG TGTGTAGTTA GGGGCTAAGG GGCGGCTTAC AACGAGGAAA CGACCGGTAT GATAAGATGC   
  
  
- GAGAAGAGTG CGAAAAACTG AAAAGGCATA AGTTTCAGCA AAAGCAGAGG GAAGAGTGAA GCAAGACGAC   
  
  
- GTACGATGGG AGAAGGAGGA GGAGGAGAGA ATATATATGG GTAGATGGGG AGCTTAAAAT TAGGGTTTTA   
  
  
- TAGAGGTTTG GGTGTAGAGA AAAGAAAGGT GACAAAAAGT GTTGAACTAC TTTATTAACT TAGTTGTTTT   
  
  
- GTAAACCTAT AACACTCTTT TTAGACTCG

+     CAAT-box

| Site Name | Organism | Position | Strand | Matrix score. | sequence | function |
| --- | --- | --- | --- | --- | --- | --- |
| CAAT-box | Arabidopsis thaliana | 452 | + | 5 | CCAAT | common cis-acting element in promoter and enhancer regions |
| CAAT-box | Nicotiana glutinosa | 1152 | + | 4 | CAAT |  |
| CAAT-box | Arabidopsis thaliana | 1151 | + | 5 | CCAAT | common cis-acting element in promoter and enhancer regions |
| CAAT-box | Arabidopsis thaliana | 110 | + | 5 | CCAAT | common cis-acting element in promoter and enhancer regions |
| CAAT-box | Nicotiana glutinosa | 84 | + | 4 | CAAT |  |
| CAAT-box | Arabidopsis thaliana | 83 | + | 5 | CCAAT | common cis-acting element in promoter and enhancer regions |
| CAAT-box | Nicotiana glutinosa | 453 | + | 4 | CAAT |  |
| CAAT-box | Nicotiana glutinosa | 1062 | + | 4 | CAAT |  |
| CAAT-box | Pisum sativum | 131 | - | 5 | CAAAT | common cis-acting element in promoter and enhancer regions |
| CAAT-box | Nicotiana glutinosa | 1456 | - | 4 | CAAT |  |
| CAAT-box | Pisum sativum | 841 | - | 5 | CAAAT | common cis-acting element in promoter and enhancer regions |
| CAAT-box | Pisum sativum | 798 | + | 5 | CAAAT | common cis-acting element in promoter and enhancer regions |
| CAAT-box | Nicotiana glutinosa | 1480 | - | 4 | CAAT |  |
| CAAT-box | Pisum sativum | 51 | + | 5 | CAAAT | common cis-acting element in promoter and enhancer regions |
| CAAT-box | Nicotiana glutinosa | 814 | + | 4 | CAAT |  |
| CAAT-box | Nicotiana glutinosa | 917 | - | 4 | CAAT |  |
| CAAT-box | Pisum sativum | 262 | + | 5 | CAAAT | common cis-acting element in promoter and enhancer regions |
| CAAT-box | Nicotiana glutinosa | 1154 | - | 4 | CAAT |  |
| CAAT-box | Nicotiana glutinosa | 1019 | + | 4 | CAAT |  |
| CAAT-box | Nicotiana glutinosa | 320 | + | 4 | CAAT |  |
| CAAT-box | Pisum sativum | 74 | - | 5 | CAAAT | common cis-acting element in promoter and enhancer regions |
| CAAT-box | Pisum sativum | 1472 | - | 5 | CAAAT | common cis-acting element in promoter and enhancer regions |
| CAAT-box | Nicotiana glutinosa | 667 | + | 4 | CAAT |  |
| CAAT-box | Nicotiana glutinosa | 994 | - | 4 | CAAT |  |
| CAAT-box | Nicotiana glutinosa | 1207 | + | 4 | CAAT |  |
| CAAT-box | Nicotiana glutinosa | 111 | + | 4 | CAAT |  |
| CAAT-box | Pisum sativum | 1048 | + | 5 | CAAAT | common cis-acting element in promoter and enhancer regions |
| CAAT-box | Nicotiana glutinosa | 915 | + | 4 | CAAT |  |

>018609   
+ CTGTCATCCA CGGCGGTGCG TGGCCAGTGG GCCGATTCTG CCATTTTGTG CAAATTTTGA TATTCTAAAT   
  
  
+ ATGATTTGGT ATCCAATCGA TGTGTTTTGA TTATTAAATC CAATGTTGGT TATTTCGGTT ATTTGCATAT   
  
  
+ TATCGGAAAT GGTTTGGAAT ATGAAATTAT GAACTATGAA AGGCCAAGGG GACCCACCCA TCATGTGGCA   
  
  
+ GTACTCGTTC CCTCCGTGAC TCTAATACTA TGTAGAATCA AAGAGACGGG CCAAATACCC ACTTCCAACA   
  
  
+ ACACTGATTT TGTCTCCAAC TTGGTAATTA CCATATGTGC AATTCGTCAG GTGTGAGGTT TTATCACAAA   
  
  
+ AGGGCTCGGT ATTAGTTAGA GTGAAATTAA GATATTTAAA CTCTTCTTTT ATTAGAGATA CGGTTGATGT   
  
  
+ GGGATGTTTA ACAAGCCCCC TCATGTGGGA TCCAATTAGT GGGTCACACA TGGGGGATTC ACACATCGAC   
  
  
+ AACCACATGA AGTCAAGGGG ACTCACCTTA CACACTGGCC CAAGTGACCC ACCTTCTTCG AGCGGGGTCA   
  
  
+ AGGGGACCCG GACCCACCCA TCACATGACA CTACAGTACG AGCCCACTTC TTGGCTTCGA TACCATGTAT   
  
  
+ AATATTCAAA GAGACTGGTC AAAAATCCAC TTCCAACAAT ATTTATATTA TCCCCAACTT GGTAATTATC   
  
  
+ ACCTACACAT TCCATCAAGT GTGGAATTTT ATCATAAAAG ACATCAGTAT TAGTTAGAGT AAGATTATCA   
  
  
+ TATACTATTA CTTTTGTTTT TCTAGGACAA ATGTGGGATT CAACAATACC ACATATAATA TTATTAAATA   
  
  
+ ATTTGGTTAA GAAAAGAATA ACAAGAAAGT CCATCGTATA ATATGTACGG AACGGTACAC CACATATTAT   
  
  
+ TATACAATTG AAAAAAAGTT TTATTTTCAA GTGTTCATTT AATTATATAA TGATATGTAG TGTTTTACTT   
  
  
+ TTGCTCCGGG CAGATTGAAA AAATTTTCAG AAATTTTTCA ATGTTTCTAA AATAGAAAAC GATACACCAA   
  
  
+ ATGTCATTAT GCAATTAAAA TAACATTTTA AATAAAAAAC AAAATTTTCC TCCACTTTCA TAGTAACATA   
  
  
+ TGATTATTAC GATGTGACTT TAATTTCGAG CCAATTGAAA AATCTTATCA TATGTCTGAG TCGTGAGATA   
  
  
+ TTAGACTAAC ACACATCAAT CCCCGATTCC CCGCCGAATG TTGCTCCTTT GCTGGCCATA CTATTCTACG   
  
  
+ CTCTTCTCAC GCTTTTTGAC TTTTCCGTAT TCAAAGTCGT TTTCGTCTCC CTTCTCACTT CGTTCTGCTG   
  
  
+ CATGCTACCC TCTTCCTCCT CCTCCTCTCT TATATATACC CATCTACCCC TCGAATTTTA ATCCCAAAAT   
  
  
+ ATCTCCAAAC CCACATCTCT TTTCTTTCCA CTGTTTTTCA CAACTTGATG AAATAATTGA ATCAACAAAA   
  
  
+ CATTTGGATA TTGTGAGAAA AATCTGAGC  

- GACAGTAGGT GCCGCCACGC ACCGGTCACC CGGCTAAGAC GGTAAAACAC GTTTAAAACT ATAAGATTTA   
  
  
- TACTAAACCA TAGGTTAGCT ACACAAAACT AATAATTTAG GTTACAACCA ATAAAGCCAA TAAACGTATA   
  
  
- ATAGCCTTTA CCAAACCTTA TACTTTAATA CTTGATACTT TCCGGTTCCC CTGGGTGGGT AGTACACCGT   
  
  
- CATGAGCAAG GGAGGCACTG AGATTATGAT ACATCTTAGT TTCTCTGCCC GGTTTATGGG TGAAGGTTGT   
  
  
- TGTGACTAAA ACAGAGGTTG AACCATTAAT GGTATACACG TTAAGCAGTC CACACTCCAA AATAGTGTTT   
  
  
- TCCCGAGCCA TAATCAATCT CACTTTAATT CTATAAATTT GAGAAGAAAA TAATCTCTAT GCCAACTACA   
  
  
- CCCTACAAAT TGTTCGGGGG AGTACACCCT AGGTTAATCA CCCAGTGTGT ACCCCCTAAG TGTGTAGCTG   
  
  
- TTGGTGTACT TCAGTTCCCC TGAGTGGAAT GTGTGACCGG GTTCACTGGG TGGAAGAAGC TCGCCCCAGT   
  
  
- TCCCCTGGGC CTGGGTGGGT AGTGTACTGT GATGTCATGC TCGGGTGAAG AACCGAAGCT ATGGTACATA   
  
  
- TTATAAGTTT CTCTGACCAG TTTTTAGGTG AAGGTTGTTA TAAATATAAT AGGGGTTGAA CCATTAATAG   
  
  
- TGGATGTGTA AGGTAGTTCA CACCTTAAAA TAGTATTTTC TGTAGTCATA ATCAATCTCA TTCTAATAGT   
  
  
- ATATGATAAT GAAAACAAAA AGATCCTGTT TACACCCTAA GTTGTTATGG TGTATATTAT AATAATTTAT   
  
  
- TAAACCAATT CTTTTCTTAT TGTTCTTTCA GGTAGCATAT TATACATGCC TTGCCATGTG GTGTATAATA   
  
  
- ATATGTTAAC TTTTTTTCAA AATAAAAGTT CACAAGTAAA TTAATATATT ACTATACATC ACAAAATGAA   
  
  
- AACGAGGCCC GTCTAACTTT TTTAAAAGTC TTTAAAAAGT TACAAAGATT TTATCTTTTG CTATGTGGTT   
  
  
- TACAGTAATA CGTTAATTTT ATTGTAAAAT TTATTTTTTG TTTTAAAAGG AGGTGAAAGT ATCATTGTAT   
  
  
- ACTAATAATG CTACACTGAA ATTAAAGCTC GGTTAACTTT TTAGAATAGT ATACAGACTC AGCACTCTAT   
  
  
- AATCTGATTG TGTGTAGTTA GGGGCTAAGG GGCGGCTTAC AACGAGGAAA CGACCGGTAT GATAAGATGC   
  
  
- GAGAAGAGTG CGAAAAACTG AAAAGGCATA AGTTTCAGCA AAAGCAGAGG GAAGAGTGAA GCAAGACGAC   
  
  
- GTACGATGGG AGAAGGAGGA GGAGGAGAGA ATATATATGG GTAGATGGGG AGCTTAAAAT TAGGGTTTTA   
  
  
- TAGAGGTTTG GGTGTAGAGA AAAGAAAGGT GACAAAAAGT GTTGAACTAC TTTATTAACT TAGTTGTTTT   
  
  
- GTAAACCTAT AACACTCTTT TTAGACTCG

+     CGTCA-motif

| Site Name | Organism | Position | Strand | Matrix score. | sequence | function |
| --- | --- | --- | --- | --- | --- | --- |
| CGTCA-motif | Hordeum vulgare | 325 | + | 5 | CGTCA | cis-acting regulatory element involved in the MeJA-responsiveness |

>018609   
+ CTGTCATCCA CGGCGGTGCG TGGCCAGTGG GCCGATTCTG CCATTTTGTG CAAATTTTGA TATTCTAAAT   
  
  
+ ATGATTTGGT ATCCAATCGA TGTGTTTTGA TTATTAAATC CAATGTTGGT TATTTCGGTT ATTTGCATAT   
  
  
+ TATCGGAAAT GGTTTGGAAT ATGAAATTAT GAACTATGAA AGGCCAAGGG GACCCACCCA TCATGTGGCA   
  
  
+ GTACTCGTTC CCTCCGTGAC TCTAATACTA TGTAGAATCA AAGAGACGGG CCAAATACCC ACTTCCAACA   
  
  
+ ACACTGATTT TGTCTCCAAC TTGGTAATTA CCATATGTGC AATTCGTCAG GTGTGAGGTT TTATCACAAA   
  
  
+ AGGGCTCGGT ATTAGTTAGA GTGAAATTAA GATATTTAAA CTCTTCTTTT ATTAGAGATA CGGTTGATGT   
  
  
+ GGGATGTTTA ACAAGCCCCC TCATGTGGGA TCCAATTAGT GGGTCACACA TGGGGGATTC ACACATCGAC   
  
  
+ AACCACATGA AGTCAAGGGG ACTCACCTTA CACACTGGCC CAAGTGACCC ACCTTCTTCG AGCGGGGTCA   
  
  
+ AGGGGACCCG GACCCACCCA TCACATGACA CTACAGTACG AGCCCACTTC TTGGCTTCGA TACCATGTAT   
  
  
+ AATATTCAAA GAGACTGGTC AAAAATCCAC TTCCAACAAT ATTTATATTA TCCCCAACTT GGTAATTATC   
  
  
+ ACCTACACAT TCCATCAAGT GTGGAATTTT ATCATAAAAG ACATCAGTAT TAGTTAGAGT AAGATTATCA   
  
  
+ TATACTATTA CTTTTGTTTT TCTAGGACAA ATGTGGGATT CAACAATACC ACATATAATA TTATTAAATA   
  
  
+ ATTTGGTTAA GAAAAGAATA ACAAGAAAGT CCATCGTATA ATATGTACGG AACGGTACAC CACATATTAT   
  
  
+ TATACAATTG AAAAAAAGTT TTATTTTCAA GTGTTCATTT AATTATATAA TGATATGTAG TGTTTTACTT   
  
  
+ TTGCTCCGGG CAGATTGAAA AAATTTTCAG AAATTTTTCA ATGTTTCTAA AATAGAAAAC GATACACCAA   
  
  
+ ATGTCATTAT GCAATTAAAA TAACATTTTA AATAAAAAAC AAAATTTTCC TCCACTTTCA TAGTAACATA   
  
  
+ TGATTATTAC GATGTGACTT TAATTTCGAG CCAATTGAAA AATCTTATCA TATGTCTGAG TCGTGAGATA   
  
  
+ TTAGACTAAC ACACATCAAT CCCCGATTCC CCGCCGAATG TTGCTCCTTT GCTGGCCATA CTATTCTACG   
  
  
+ CTCTTCTCAC GCTTTTTGAC TTTTCCGTAT TCAAAGTCGT TTTCGTCTCC CTTCTCACTT CGTTCTGCTG   
  
  
+ CATGCTACCC TCTTCCTCCT CCTCCTCTCT TATATATACC CATCTACCCC TCGAATTTTA ATCCCAAAAT   
  
  
+ ATCTCCAAAC CCACATCTCT TTTCTTTCCA CTGTTTTTCA CAACTTGATG AAATAATTGA ATCAACAAAA   
  
  
+ CATTTGGATA TTGTGAGAAA AATCTGAGC  

- GACAGTAGGT GCCGCCACGC ACCGGTCACC CGGCTAAGAC GGTAAAACAC GTTTAAAACT ATAAGATTTA   
  
  
- TACTAAACCA TAGGTTAGCT ACACAAAACT AATAATTTAG GTTACAACCA ATAAAGCCAA TAAACGTATA   
  
  
- ATAGCCTTTA CCAAACCTTA TACTTTAATA CTTGATACTT TCCGGTTCCC CTGGGTGGGT AGTACACCGT   
  
  
- CATGAGCAAG GGAGGCACTG AGATTATGAT ACATCTTAGT TTCTCTGCCC GGTTTATGGG TGAAGGTTGT   
  
  
- TGTGACTAAA ACAGAGGTTG AACCATTAAT GGTATACACG TTAAGCAGTC CACACTCCAA AATAGTGTTT   
  
  
- TCCCGAGCCA TAATCAATCT CACTTTAATT CTATAAATTT GAGAAGAAAA TAATCTCTAT GCCAACTACA   
  
  
- CCCTACAAAT TGTTCGGGGG AGTACACCCT AGGTTAATCA CCCAGTGTGT ACCCCCTAAG TGTGTAGCTG   
  
  
- TTGGTGTACT TCAGTTCCCC TGAGTGGAAT GTGTGACCGG GTTCACTGGG TGGAAGAAGC TCGCCCCAGT   
  
  
- TCCCCTGGGC CTGGGTGGGT AGTGTACTGT GATGTCATGC TCGGGTGAAG AACCGAAGCT ATGGTACATA   
  
  
- TTATAAGTTT CTCTGACCAG TTTTTAGGTG AAGGTTGTTA TAAATATAAT AGGGGTTGAA CCATTAATAG   
  
  
- TGGATGTGTA AGGTAGTTCA CACCTTAAAA TAGTATTTTC TGTAGTCATA ATCAATCTCA TTCTAATAGT   
  
  
- ATATGATAAT GAAAACAAAA AGATCCTGTT TACACCCTAA GTTGTTATGG TGTATATTAT AATAATTTAT   
  
  
- TAAACCAATT CTTTTCTTAT TGTTCTTTCA GGTAGCATAT TATACATGCC TTGCCATGTG GTGTATAATA   
  
  
- ATATGTTAAC TTTTTTTCAA AATAAAAGTT CACAAGTAAA TTAATATATT ACTATACATC ACAAAATGAA   
  
  
- AACGAGGCCC GTCTAACTTT TTTAAAAGTC TTTAAAAAGT TACAAAGATT TTATCTTTTG CTATGTGGTT   
  
  
- TACAGTAATA CGTTAATTTT ATTGTAAAAT TTATTTTTTG TTTTAAAAGG AGGTGAAAGT ATCATTGTAT   
  
  
- ACTAATAATG CTACACTGAA ATTAAAGCTC GGTTAACTTT TTAGAATAGT ATACAGACTC AGCACTCTAT   
  
  
- AATCTGATTG TGTGTAGTTA GGGGCTAAGG GGCGGCTTAC AACGAGGAAA CGACCGGTAT GATAAGATGC   
  
  
- GAGAAGAGTG CGAAAAACTG AAAAGGCATA AGTTTCAGCA AAAGCAGAGG GAAGAGTGAA GCAAGACGAC   
  
  
- GTACGATGGG AGAAGGAGGA GGAGGAGAGA ATATATATGG GTAGATGGGG AGCTTAAAAT TAGGGTTTTA   
  
  
- TAGAGGTTTG GGTGTAGAGA AAAGAAAGGT GACAAAAAGT GTTGAACTAC TTTATTAACT TAGTTGTTTT   
  
  
- GTAAACCTAT AACACTCTTT TTAGACTCG

+     ERE

| Site Name | Organism | Position | Strand | Matrix score. | sequence | function |
| --- | --- | --- | --- | --- | --- | --- |
| ERE | Nicotiana glutinos | 160 | - | 8 | ATTTCATA |  |
| ERE | Nicotiana glutinos | 1075 | + | 8 | ATTTTAAA |  |

>018609   
+ CTGTCATCCA CGGCGGTGCG TGGCCAGTGG GCCGATTCTG CCATTTTGTG CAAATTTTGA TATTCTAAAT   
  
  
+ ATGATTTGGT ATCCAATCGA TGTGTTTTGA TTATTAAATC CAATGTTGGT TATTTCGGTT ATTTGCATAT   
  
  
+ TATCGGAAAT GGTTTGGAAT ATGAAATTAT GAACTATGAA AGGCCAAGGG GACCCACCCA TCATGTGGCA   
  
  
+ GTACTCGTTC CCTCCGTGAC TCTAATACTA TGTAGAATCA AAGAGACGGG CCAAATACCC ACTTCCAACA   
  
  
+ ACACTGATTT TGTCTCCAAC TTGGTAATTA CCATATGTGC AATTCGTCAG GTGTGAGGTT TTATCACAAA   
  
  
+ AGGGCTCGGT ATTAGTTAGA GTGAAATTAA GATATTTAAA CTCTTCTTTT ATTAGAGATA CGGTTGATGT   
  
  
+ GGGATGTTTA ACAAGCCCCC TCATGTGGGA TCCAATTAGT GGGTCACACA TGGGGGATTC ACACATCGAC   
  
  
+ AACCACATGA AGTCAAGGGG ACTCACCTTA CACACTGGCC CAAGTGACCC ACCTTCTTCG AGCGGGGTCA   
  
  
+ AGGGGACCCG GACCCACCCA TCACATGACA CTACAGTACG AGCCCACTTC TTGGCTTCGA TACCATGTAT   
  
  
+ AATATTCAAA GAGACTGGTC AAAAATCCAC TTCCAACAAT ATTTATATTA TCCCCAACTT GGTAATTATC   
  
  
+ ACCTACACAT TCCATCAAGT GTGGAATTTT ATCATAAAAG ACATCAGTAT TAGTTAGAGT AAGATTATCA   
  
  
+ TATACTATTA CTTTTGTTTT TCTAGGACAA ATGTGGGATT CAACAATACC ACATATAATA TTATTAAATA   
  
  
+ ATTTGGTTAA GAAAAGAATA ACAAGAAAGT CCATCGTATA ATATGTACGG AACGGTACAC CACATATTAT   
  
  
+ TATACAATTG AAAAAAAGTT TTATTTTCAA GTGTTCATTT AATTATATAA TGATATGTAG TGTTTTACTT   
  
  
+ TTGCTCCGGG CAGATTGAAA AAATTTTCAG AAATTTTTCA ATGTTTCTAA AATAGAAAAC GATACACCAA   
  
  
+ ATGTCATTAT GCAATTAAAA TAACATTTTA AATAAAAAAC AAAATTTTCC TCCACTTTCA TAGTAACATA   
  
  
+ TGATTATTAC GATGTGACTT TAATTTCGAG CCAATTGAAA AATCTTATCA TATGTCTGAG TCGTGAGATA   
  
  
+ TTAGACTAAC ACACATCAAT CCCCGATTCC CCGCCGAATG TTGCTCCTTT GCTGGCCATA CTATTCTACG   
  
  
+ CTCTTCTCAC GCTTTTTGAC TTTTCCGTAT TCAAAGTCGT TTTCGTCTCC CTTCTCACTT CGTTCTGCTG   
  
  
+ CATGCTACCC TCTTCCTCCT CCTCCTCTCT TATATATACC CATCTACCCC TCGAATTTTA ATCCCAAAAT   
  
  
+ ATCTCCAAAC CCACATCTCT TTTCTTTCCA CTGTTTTTCA CAACTTGATG AAATAATTGA ATCAACAAAA   
  
  
+ CATTTGGATA TTGTGAGAAA AATCTGAGC  

- GACAGTAGGT GCCGCCACGC ACCGGTCACC CGGCTAAGAC GGTAAAACAC GTTTAAAACT ATAAGATTTA   
  
  
- TACTAAACCA TAGGTTAGCT ACACAAAACT AATAATTTAG GTTACAACCA ATAAAGCCAA TAAACGTATA   
  
  
- ATAGCCTTTA CCAAACCTTA TACTTTAATA CTTGATACTT TCCGGTTCCC CTGGGTGGGT AGTACACCGT   
  
  
- CATGAGCAAG GGAGGCACTG AGATTATGAT ACATCTTAGT TTCTCTGCCC GGTTTATGGG TGAAGGTTGT   
  
  
- TGTGACTAAA ACAGAGGTTG AACCATTAAT GGTATACACG TTAAGCAGTC CACACTCCAA AATAGTGTTT   
  
  
- TCCCGAGCCA TAATCAATCT CACTTTAATT CTATAAATTT GAGAAGAAAA TAATCTCTAT GCCAACTACA   
  
  
- CCCTACAAAT TGTTCGGGGG AGTACACCCT AGGTTAATCA CCCAGTGTGT ACCCCCTAAG TGTGTAGCTG   
  
  
- TTGGTGTACT TCAGTTCCCC TGAGTGGAAT GTGTGACCGG GTTCACTGGG TGGAAGAAGC TCGCCCCAGT   
  
  
- TCCCCTGGGC CTGGGTGGGT AGTGTACTGT GATGTCATGC TCGGGTGAAG AACCGAAGCT ATGGTACATA   
  
  
- TTATAAGTTT CTCTGACCAG TTTTTAGGTG AAGGTTGTTA TAAATATAAT AGGGGTTGAA CCATTAATAG   
  
  
- TGGATGTGTA AGGTAGTTCA CACCTTAAAA TAGTATTTTC TGTAGTCATA ATCAATCTCA TTCTAATAGT   
  
  
- ATATGATAAT GAAAACAAAA AGATCCTGTT TACACCCTAA GTTGTTATGG TGTATATTAT AATAATTTAT   
  
  
- TAAACCAATT CTTTTCTTAT TGTTCTTTCA GGTAGCATAT TATACATGCC TTGCCATGTG GTGTATAATA   
  
  
- ATATGTTAAC TTTTTTTCAA AATAAAAGTT CACAAGTAAA TTAATATATT ACTATACATC ACAAAATGAA   
  
  
- AACGAGGCCC GTCTAACTTT TTTAAAAGTC TTTAAAAAGT TACAAAGATT TTATCTTTTG CTATGTGGTT   
  
  
- TACAGTAATA CGTTAATTTT ATTGTAAAAT TTATTTTTTG TTTTAAAAGG AGGTGAAAGT ATCATTGTAT   
  
  
- ACTAATAATG CTACACTGAA ATTAAAGCTC GGTTAACTTT TTAGAATAGT ATACAGACTC AGCACTCTAT   
  
  
- AATCTGATTG TGTGTAGTTA GGGGCTAAGG GGCGGCTTAC AACGAGGAAA CGACCGGTAT GATAAGATGC   
  
  
- GAGAAGAGTG CGAAAAACTG AAAAGGCATA AGTTTCAGCA AAAGCAGAGG GAAGAGTGAA GCAAGACGAC   
  
  
- GTACGATGGG AGAAGGAGGA GGAGGAGAGA ATATATATGG GTAGATGGGG AGCTTAAAAT TAGGGTTTTA   
  
  
- TAGAGGTTTG GGTGTAGAGA AAAGAAAGGT GACAAAAAGT GTTGAACTAC TTTATTAACT TAGTTGTTTT   
  
  
- GTAAACCTAT AACACTCTTT TTAGACTCG

+     G-box

| Site Name | Organism | Position | Strand | Matrix score. | sequence | function |
| --- | --- | --- | --- | --- | --- | --- |
| G-box | Zea mays | 1180 | - | 6 | CACGAC | cis-acting regulatory element involved in light responsiveness |

>018609   
+ CTGTCATCCA CGGCGGTGCG TGGCCAGTGG GCCGATTCTG CCATTTTGTG CAAATTTTGA TATTCTAAAT   
  
  
+ ATGATTTGGT ATCCAATCGA TGTGTTTTGA TTATTAAATC CAATGTTGGT TATTTCGGTT ATTTGCATAT   
  
  
+ TATCGGAAAT GGTTTGGAAT ATGAAATTAT GAACTATGAA AGGCCAAGGG GACCCACCCA TCATGTGGCA   
  
  
+ GTACTCGTTC CCTCCGTGAC TCTAATACTA TGTAGAATCA AAGAGACGGG CCAAATACCC ACTTCCAACA   
  
  
+ ACACTGATTT TGTCTCCAAC TTGGTAATTA CCATATGTGC AATTCGTCAG GTGTGAGGTT TTATCACAAA   
  
  
+ AGGGCTCGGT ATTAGTTAGA GTGAAATTAA GATATTTAAA CTCTTCTTTT ATTAGAGATA CGGTTGATGT   
  
  
+ GGGATGTTTA ACAAGCCCCC TCATGTGGGA TCCAATTAGT GGGTCACACA TGGGGGATTC ACACATCGAC   
  
  
+ AACCACATGA AGTCAAGGGG ACTCACCTTA CACACTGGCC CAAGTGACCC ACCTTCTTCG AGCGGGGTCA   
  
  
+ AGGGGACCCG GACCCACCCA TCACATGACA CTACAGTACG AGCCCACTTC TTGGCTTCGA TACCATGTAT   
  
  
+ AATATTCAAA GAGACTGGTC AAAAATCCAC TTCCAACAAT ATTTATATTA TCCCCAACTT GGTAATTATC   
  
  
+ ACCTACACAT TCCATCAAGT GTGGAATTTT ATCATAAAAG ACATCAGTAT TAGTTAGAGT AAGATTATCA   
  
  
+ TATACTATTA CTTTTGTTTT TCTAGGACAA ATGTGGGATT CAACAATACC ACATATAATA TTATTAAATA   
  
  
+ ATTTGGTTAA GAAAAGAATA ACAAGAAAGT CCATCGTATA ATATGTACGG AACGGTACAC CACATATTAT   
  
  
+ TATACAATTG AAAAAAAGTT TTATTTTCAA GTGTTCATTT AATTATATAA TGATATGTAG TGTTTTACTT   
  
  
+ TTGCTCCGGG CAGATTGAAA AAATTTTCAG AAATTTTTCA ATGTTTCTAA AATAGAAAAC GATACACCAA   
  
  
+ ATGTCATTAT GCAATTAAAA TAACATTTTA AATAAAAAAC AAAATTTTCC TCCACTTTCA TAGTAACATA   
  
  
+ TGATTATTAC GATGTGACTT TAATTTCGAG CCAATTGAAA AATCTTATCA TATGTCTGAG TCGTGAGATA   
  
  
+ TTAGACTAAC ACACATCAAT CCCCGATTCC CCGCCGAATG TTGCTCCTTT GCTGGCCATA CTATTCTACG   
  
  
+ CTCTTCTCAC GCTTTTTGAC TTTTCCGTAT TCAAAGTCGT TTTCGTCTCC CTTCTCACTT CGTTCTGCTG   
  
  
+ CATGCTACCC TCTTCCTCCT CCTCCTCTCT TATATATACC CATCTACCCC TCGAATTTTA ATCCCAAAAT   
  
  
+ ATCTCCAAAC CCACATCTCT TTTCTTTCCA CTGTTTTTCA CAACTTGATG AAATAATTGA ATCAACAAAA   
  
  
+ CATTTGGATA TTGTGAGAAA AATCTGAGC  

- GACAGTAGGT GCCGCCACGC ACCGGTCACC CGGCTAAGAC GGTAAAACAC GTTTAAAACT ATAAGATTTA   
  
  
- TACTAAACCA TAGGTTAGCT ACACAAAACT AATAATTTAG GTTACAACCA ATAAAGCCAA TAAACGTATA   
  
  
- ATAGCCTTTA CCAAACCTTA TACTTTAATA CTTGATACTT TCCGGTTCCC CTGGGTGGGT AGTACACCGT   
  
  
- CATGAGCAAG GGAGGCACTG AGATTATGAT ACATCTTAGT TTCTCTGCCC GGTTTATGGG TGAAGGTTGT   
  
  
- TGTGACTAAA ACAGAGGTTG AACCATTAAT GGTATACACG TTAAGCAGTC CACACTCCAA AATAGTGTTT   
  
  
- TCCCGAGCCA TAATCAATCT CACTTTAATT CTATAAATTT GAGAAGAAAA TAATCTCTAT GCCAACTACA   
  
  
- CCCTACAAAT TGTTCGGGGG AGTACACCCT AGGTTAATCA CCCAGTGTGT ACCCCCTAAG TGTGTAGCTG   
  
  
- TTGGTGTACT TCAGTTCCCC TGAGTGGAAT GTGTGACCGG GTTCACTGGG TGGAAGAAGC TCGCCCCAGT   
  
  
- TCCCCTGGGC CTGGGTGGGT AGTGTACTGT GATGTCATGC TCGGGTGAAG AACCGAAGCT ATGGTACATA   
  
  
- TTATAAGTTT CTCTGACCAG TTTTTAGGTG AAGGTTGTTA TAAATATAAT AGGGGTTGAA CCATTAATAG   
  
  
- TGGATGTGTA AGGTAGTTCA CACCTTAAAA TAGTATTTTC TGTAGTCATA ATCAATCTCA TTCTAATAGT   
  
  
- ATATGATAAT GAAAACAAAA AGATCCTGTT TACACCCTAA GTTGTTATGG TGTATATTAT AATAATTTAT   
  
  
- TAAACCAATT CTTTTCTTAT TGTTCTTTCA GGTAGCATAT TATACATGCC TTGCCATGTG GTGTATAATA   
  
  
- ATATGTTAAC TTTTTTTCAA AATAAAAGTT CACAAGTAAA TTAATATATT ACTATACATC ACAAAATGAA   
  
  
- AACGAGGCCC GTCTAACTTT TTTAAAAGTC TTTAAAAAGT TACAAAGATT TTATCTTTTG CTATGTGGTT   
  
  
- TACAGTAATA CGTTAATTTT ATTGTAAAAT TTATTTTTTG TTTTAAAAGG AGGTGAAAGT ATCATTGTAT   
  
  
- ACTAATAATG CTACACTGAA ATTAAAGCTC GGTTAACTTT TTAGAATAGT ATACAGACTC AGCACTCTAT   
  
  
- AATCTGATTG TGTGTAGTTA GGGGCTAAGG GGCGGCTTAC AACGAGGAAA CGACCGGTAT GATAAGATGC   
  
  
- GAGAAGAGTG CGAAAAACTG AAAAGGCATA AGTTTCAGCA AAAGCAGAGG GAAGAGTGAA GCAAGACGAC   
  
  
- GTACGATGGG AGAAGGAGGA GGAGGAGAGA ATATATATGG GTAGATGGGG AGCTTAAAAT TAGGGTTTTA   
  
  
- TAGAGGTTTG GGTGTAGAGA AAAGAAAGGT GACAAAAAGT GTTGAACTAC TTTATTAACT TAGTTGTTTT   
  
  
- GTAAACCTAT AACACTCTTT TTAGACTCG

+     GATA-motif

| Site Name | Organism | Position | Strand | Matrix score. | sequence | function |
| --- | --- | --- | --- | --- | --- | --- |
| GATA-motif | Arabidopsis thaliana | 1161 | - | 10 | AAGATAAGATT | part of a light responsive element |

>018609   
+ CTGTCATCCA CGGCGGTGCG TGGCCAGTGG GCCGATTCTG CCATTTTGTG CAAATTTTGA TATTCTAAAT   
  
  
+ ATGATTTGGT ATCCAATCGA TGTGTTTTGA TTATTAAATC CAATGTTGGT TATTTCGGTT ATTTGCATAT   
  
  
+ TATCGGAAAT GGTTTGGAAT ATGAAATTAT GAACTATGAA AGGCCAAGGG GACCCACCCA TCATGTGGCA   
  
  
+ GTACTCGTTC CCTCCGTGAC TCTAATACTA TGTAGAATCA AAGAGACGGG CCAAATACCC ACTTCCAACA   
  
  
+ ACACTGATTT TGTCTCCAAC TTGGTAATTA CCATATGTGC AATTCGTCAG GTGTGAGGTT TTATCACAAA   
  
  
+ AGGGCTCGGT ATTAGTTAGA GTGAAATTAA GATATTTAAA CTCTTCTTTT ATTAGAGATA CGGTTGATGT   
  
  
+ GGGATGTTTA ACAAGCCCCC TCATGTGGGA TCCAATTAGT GGGTCACACA TGGGGGATTC ACACATCGAC   
  
  
+ AACCACATGA AGTCAAGGGG ACTCACCTTA CACACTGGCC CAAGTGACCC ACCTTCTTCG AGCGGGGTCA   
  
  
+ AGGGGACCCG GACCCACCCA TCACATGACA CTACAGTACG AGCCCACTTC TTGGCTTCGA TACCATGTAT   
  
  
+ AATATTCAAA GAGACTGGTC AAAAATCCAC TTCCAACAAT ATTTATATTA TCCCCAACTT GGTAATTATC   
  
  
+ ACCTACACAT TCCATCAAGT GTGGAATTTT ATCATAAAAG ACATCAGTAT TAGTTAGAGT AAGATTATCA   
  
  
+ TATACTATTA CTTTTGTTTT TCTAGGACAA ATGTGGGATT CAACAATACC ACATATAATA TTATTAAATA   
  
  
+ ATTTGGTTAA GAAAAGAATA ACAAGAAAGT CCATCGTATA ATATGTACGG AACGGTACAC CACATATTAT   
  
  
+ TATACAATTG AAAAAAAGTT TTATTTTCAA GTGTTCATTT AATTATATAA TGATATGTAG TGTTTTACTT   
  
  
+ TTGCTCCGGG CAGATTGAAA AAATTTTCAG AAATTTTTCA ATGTTTCTAA AATAGAAAAC GATACACCAA   
  
  
+ ATGTCATTAT GCAATTAAAA TAACATTTTA AATAAAAAAC AAAATTTTCC TCCACTTTCA TAGTAACATA   
  
  
+ TGATTATTAC GATGTGACTT TAATTTCGAG CCAATTGAAA AATCTTATCA TATGTCTGAG TCGTGAGATA   
  
  
+ TTAGACTAAC ACACATCAAT CCCCGATTCC CCGCCGAATG TTGCTCCTTT GCTGGCCATA CTATTCTACG   
  
  
+ CTCTTCTCAC GCTTTTTGAC TTTTCCGTAT TCAAAGTCGT TTTCGTCTCC CTTCTCACTT CGTTCTGCTG   
  
  
+ CATGCTACCC TCTTCCTCCT CCTCCTCTCT TATATATACC CATCTACCCC TCGAATTTTA ATCCCAAAAT   
  
  
+ ATCTCCAAAC CCACATCTCT TTTCTTTCCA CTGTTTTTCA CAACTTGATG AAATAATTGA ATCAACAAAA   
  
  
+ CATTTGGATA TTGTGAGAAA AATCTGAGC  

- GACAGTAGGT GCCGCCACGC ACCGGTCACC CGGCTAAGAC GGTAAAACAC GTTTAAAACT ATAAGATTTA   
  
  
- TACTAAACCA TAGGTTAGCT ACACAAAACT AATAATTTAG GTTACAACCA ATAAAGCCAA TAAACGTATA   
  
  
- ATAGCCTTTA CCAAACCTTA TACTTTAATA CTTGATACTT TCCGGTTCCC CTGGGTGGGT AGTACACCGT   
  
  
- CATGAGCAAG GGAGGCACTG AGATTATGAT ACATCTTAGT TTCTCTGCCC GGTTTATGGG TGAAGGTTGT   
  
  
- TGTGACTAAA ACAGAGGTTG AACCATTAAT GGTATACACG TTAAGCAGTC CACACTCCAA AATAGTGTTT   
  
  
- TCCCGAGCCA TAATCAATCT CACTTTAATT CTATAAATTT GAGAAGAAAA TAATCTCTAT GCCAACTACA   
  
  
- CCCTACAAAT TGTTCGGGGG AGTACACCCT AGGTTAATCA CCCAGTGTGT ACCCCCTAAG TGTGTAGCTG   
  
  
- TTGGTGTACT TCAGTTCCCC TGAGTGGAAT GTGTGACCGG GTTCACTGGG TGGAAGAAGC TCGCCCCAGT   
  
  
- TCCCCTGGGC CTGGGTGGGT AGTGTACTGT GATGTCATGC TCGGGTGAAG AACCGAAGCT ATGGTACATA   
  
  
- TTATAAGTTT CTCTGACCAG TTTTTAGGTG AAGGTTGTTA TAAATATAAT AGGGGTTGAA CCATTAATAG   
  
  
- TGGATGTGTA AGGTAGTTCA CACCTTAAAA TAGTATTTTC TGTAGTCATA ATCAATCTCA TTCTAATAGT   
  
  
- ATATGATAAT GAAAACAAAA AGATCCTGTT TACACCCTAA GTTGTTATGG TGTATATTAT AATAATTTAT   
  
  
- TAAACCAATT CTTTTCTTAT TGTTCTTTCA GGTAGCATAT TATACATGCC TTGCCATGTG GTGTATAATA   
  
  
- ATATGTTAAC TTTTTTTCAA AATAAAAGTT CACAAGTAAA TTAATATATT ACTATACATC ACAAAATGAA   
  
  
- AACGAGGCCC GTCTAACTTT TTTAAAAGTC TTTAAAAAGT TACAAAGATT TTATCTTTTG CTATGTGGTT   
  
  
- TACAGTAATA CGTTAATTTT ATTGTAAAAT TTATTTTTTG TTTTAAAAGG AGGTGAAAGT ATCATTGTAT   
  
  
- ACTAATAATG CTACACTGAA ATTAAAGCTC GGTTAACTTT TTAGAATAGT ATACAGACTC AGCACTCTAT   
  
  
- AATCTGATTG TGTGTAGTTA GGGGCTAAGG GGCGGCTTAC AACGAGGAAA CGACCGGTAT GATAAGATGC   
  
  
- GAGAAGAGTG CGAAAAACTG AAAAGGCATA AGTTTCAGCA AAAGCAGAGG GAAGAGTGAA GCAAGACGAC   
  
  
- GTACGATGGG AGAAGGAGGA GGAGGAGAGA ATATATATGG GTAGATGGGG AGCTTAAAAT TAGGGTTTTA   
  
  
- TAGAGGTTTG GGTGTAGAGA AAAGAAAGGT GACAAAAAGT GTTGAACTAC TTTATTAACT TAGTTGTTTT   
  
  
- GTAAACCTAT AACACTCTTT TTAGACTCG

+     GT1-motif

| Site Name | Organism | Position | Strand | Matrix score. | sequence | function |
| --- | --- | --- | --- | --- | --- | --- |
| GT1-motif | Arabidopsis thaliana | 845 | + | 6 | GGTTAA | light responsive element |

>018609   
+ CTGTCATCCA CGGCGGTGCG TGGCCAGTGG GCCGATTCTG CCATTTTGTG CAAATTTTGA TATTCTAAAT   
  
  
+ ATGATTTGGT ATCCAATCGA TGTGTTTTGA TTATTAAATC CAATGTTGGT TATTTCGGTT ATTTGCATAT   
  
  
+ TATCGGAAAT GGTTTGGAAT ATGAAATTAT GAACTATGAA AGGCCAAGGG GACCCACCCA TCATGTGGCA   
  
  
+ GTACTCGTTC CCTCCGTGAC TCTAATACTA TGTAGAATCA AAGAGACGGG CCAAATACCC ACTTCCAACA   
  
  
+ ACACTGATTT TGTCTCCAAC TTGGTAATTA CCATATGTGC AATTCGTCAG GTGTGAGGTT TTATCACAAA   
  
  
+ AGGGCTCGGT ATTAGTTAGA GTGAAATTAA GATATTTAAA CTCTTCTTTT ATTAGAGATA CGGTTGATGT   
  
  
+ GGGATGTTTA ACAAGCCCCC TCATGTGGGA TCCAATTAGT GGGTCACACA TGGGGGATTC ACACATCGAC   
  
  
+ AACCACATGA AGTCAAGGGG ACTCACCTTA CACACTGGCC CAAGTGACCC ACCTTCTTCG AGCGGGGTCA   
  
  
+ AGGGGACCCG GACCCACCCA TCACATGACA CTACAGTACG AGCCCACTTC TTGGCTTCGA TACCATGTAT   
  
  
+ AATATTCAAA GAGACTGGTC AAAAATCCAC TTCCAACAAT ATTTATATTA TCCCCAACTT GGTAATTATC   
  
  
+ ACCTACACAT TCCATCAAGT GTGGAATTTT ATCATAAAAG ACATCAGTAT TAGTTAGAGT AAGATTATCA   
  
  
+ TATACTATTA CTTTTGTTTT TCTAGGACAA ATGTGGGATT CAACAATACC ACATATAATA TTATTAAATA   
  
  
+ ATTTGGTTAA GAAAAGAATA ACAAGAAAGT CCATCGTATA ATATGTACGG AACGGTACAC CACATATTAT   
  
  
+ TATACAATTG AAAAAAAGTT TTATTTTCAA GTGTTCATTT AATTATATAA TGATATGTAG TGTTTTACTT   
  
  
+ TTGCTCCGGG CAGATTGAAA AAATTTTCAG AAATTTTTCA ATGTTTCTAA AATAGAAAAC GATACACCAA   
  
  
+ ATGTCATTAT GCAATTAAAA TAACATTTTA AATAAAAAAC AAAATTTTCC TCCACTTTCA TAGTAACATA   
  
  
+ TGATTATTAC GATGTGACTT TAATTTCGAG CCAATTGAAA AATCTTATCA TATGTCTGAG TCGTGAGATA   
  
  
+ TTAGACTAAC ACACATCAAT CCCCGATTCC CCGCCGAATG TTGCTCCTTT GCTGGCCATA CTATTCTACG   
  
  
+ CTCTTCTCAC GCTTTTTGAC TTTTCCGTAT TCAAAGTCGT TTTCGTCTCC CTTCTCACTT CGTTCTGCTG   
  
  
+ CATGCTACCC TCTTCCTCCT CCTCCTCTCT TATATATACC CATCTACCCC TCGAATTTTA ATCCCAAAAT   
  
  
+ ATCTCCAAAC CCACATCTCT TTTCTTTCCA CTGTTTTTCA CAACTTGATG AAATAATTGA ATCAACAAAA   
  
  
+ CATTTGGATA TTGTGAGAAA AATCTGAGC  

- GACAGTAGGT GCCGCCACGC ACCGGTCACC CGGCTAAGAC GGTAAAACAC GTTTAAAACT ATAAGATTTA   
  
  
- TACTAAACCA TAGGTTAGCT ACACAAAACT AATAATTTAG GTTACAACCA ATAAAGCCAA TAAACGTATA   
  
  
- ATAGCCTTTA CCAAACCTTA TACTTTAATA CTTGATACTT TCCGGTTCCC CTGGGTGGGT AGTACACCGT   
  
  
- CATGAGCAAG GGAGGCACTG AGATTATGAT ACATCTTAGT TTCTCTGCCC GGTTTATGGG TGAAGGTTGT   
  
  
- TGTGACTAAA ACAGAGGTTG AACCATTAAT GGTATACACG TTAAGCAGTC CACACTCCAA AATAGTGTTT   
  
  
- TCCCGAGCCA TAATCAATCT CACTTTAATT CTATAAATTT GAGAAGAAAA TAATCTCTAT GCCAACTACA   
  
  
- CCCTACAAAT TGTTCGGGGG AGTACACCCT AGGTTAATCA CCCAGTGTGT ACCCCCTAAG TGTGTAGCTG   
  
  
- TTGGTGTACT TCAGTTCCCC TGAGTGGAAT GTGTGACCGG GTTCACTGGG TGGAAGAAGC TCGCCCCAGT   
  
  
- TCCCCTGGGC CTGGGTGGGT AGTGTACTGT GATGTCATGC TCGGGTGAAG AACCGAAGCT ATGGTACATA   
  
  
- TTATAAGTTT CTCTGACCAG TTTTTAGGTG AAGGTTGTTA TAAATATAAT AGGGGTTGAA CCATTAATAG   
  
  
- TGGATGTGTA AGGTAGTTCA CACCTTAAAA TAGTATTTTC TGTAGTCATA ATCAATCTCA TTCTAATAGT   
  
  
- ATATGATAAT GAAAACAAAA AGATCCTGTT TACACCCTAA GTTGTTATGG TGTATATTAT AATAATTTAT   
  
  
- TAAACCAATT CTTTTCTTAT TGTTCTTTCA GGTAGCATAT TATACATGCC TTGCCATGTG GTGTATAATA   
  
  
- ATATGTTAAC TTTTTTTCAA AATAAAAGTT CACAAGTAAA TTAATATATT ACTATACATC ACAAAATGAA   
  
  
- AACGAGGCCC GTCTAACTTT TTTAAAAGTC TTTAAAAAGT TACAAAGATT TTATCTTTTG CTATGTGGTT   
  
  
- TACAGTAATA CGTTAATTTT ATTGTAAAAT TTATTTTTTG TTTTAAAAGG AGGTGAAAGT ATCATTGTAT   
  
  
- ACTAATAATG CTACACTGAA ATTAAAGCTC GGTTAACTTT TTAGAATAGT ATACAGACTC AGCACTCTAT   
  
  
- AATCTGATTG TGTGTAGTTA GGGGCTAAGG GGCGGCTTAC AACGAGGAAA CGACCGGTAT GATAAGATGC   
  
  
- GAGAAGAGTG CGAAAAACTG AAAAGGCATA AGTTTCAGCA AAAGCAGAGG GAAGAGTGAA GCAAGACGAC   
  
  
- GTACGATGGG AGAAGGAGGA GGAGGAGAGA ATATATATGG GTAGATGGGG AGCTTAAAAT TAGGGTTTTA   
  
  
- TAGAGGTTTG GGTGTAGAGA AAAGAAAGGT GACAAAAAGT GTTGAACTAC TTTATTAACT TAGTTGTTTT   
  
  
- GTAAACCTAT AACACTCTTT TTAGACTCG

+     GTGGC-motif

| Site Name | Organism | Position | Strand | Matrix score. | sequence | function |
| --- | --- | --- | --- | --- | --- | --- |
| GTGGC-motif | Arabidopsis thaliana | 199 | + | 10 | CATCGTGTGGC | part of a light responsive element |

>018609   
+ CTGTCATCCA CGGCGGTGCG TGGCCAGTGG GCCGATTCTG CCATTTTGTG CAAATTTTGA TATTCTAAAT   
  
  
+ ATGATTTGGT ATCCAATCGA TGTGTTTTGA TTATTAAATC CAATGTTGGT TATTTCGGTT ATTTGCATAT   
  
  
+ TATCGGAAAT GGTTTGGAAT ATGAAATTAT GAACTATGAA AGGCCAAGGG GACCCACCCA TCATGTGGCA   
  
  
+ GTACTCGTTC CCTCCGTGAC TCTAATACTA TGTAGAATCA AAGAGACGGG CCAAATACCC ACTTCCAACA   
  
  
+ ACACTGATTT TGTCTCCAAC TTGGTAATTA CCATATGTGC AATTCGTCAG GTGTGAGGTT TTATCACAAA   
  
  
+ AGGGCTCGGT ATTAGTTAGA GTGAAATTAA GATATTTAAA CTCTTCTTTT ATTAGAGATA CGGTTGATGT   
  
  
+ GGGATGTTTA ACAAGCCCCC TCATGTGGGA TCCAATTAGT GGGTCACACA TGGGGGATTC ACACATCGAC   
  
  
+ AACCACATGA AGTCAAGGGG ACTCACCTTA CACACTGGCC CAAGTGACCC ACCTTCTTCG AGCGGGGTCA   
  
  
+ AGGGGACCCG GACCCACCCA TCACATGACA CTACAGTACG AGCCCACTTC TTGGCTTCGA TACCATGTAT   
  
  
+ AATATTCAAA GAGACTGGTC AAAAATCCAC TTCCAACAAT ATTTATATTA TCCCCAACTT GGTAATTATC   
  
  
+ ACCTACACAT TCCATCAAGT GTGGAATTTT ATCATAAAAG ACATCAGTAT TAGTTAGAGT AAGATTATCA   
  
  
+ TATACTATTA CTTTTGTTTT TCTAGGACAA ATGTGGGATT CAACAATACC ACATATAATA TTATTAAATA   
  
  
+ ATTTGGTTAA GAAAAGAATA ACAAGAAAGT CCATCGTATA ATATGTACGG AACGGTACAC CACATATTAT   
  
  
+ TATACAATTG AAAAAAAGTT TTATTTTCAA GTGTTCATTT AATTATATAA TGATATGTAG TGTTTTACTT   
  
  
+ TTGCTCCGGG CAGATTGAAA AAATTTTCAG AAATTTTTCA ATGTTTCTAA AATAGAAAAC GATACACCAA   
  
  
+ ATGTCATTAT GCAATTAAAA TAACATTTTA AATAAAAAAC AAAATTTTCC TCCACTTTCA TAGTAACATA   
  
  
+ TGATTATTAC GATGTGACTT TAATTTCGAG CCAATTGAAA AATCTTATCA TATGTCTGAG TCGTGAGATA   
  
  
+ TTAGACTAAC ACACATCAAT CCCCGATTCC CCGCCGAATG TTGCTCCTTT GCTGGCCATA CTATTCTACG   
  
  
+ CTCTTCTCAC GCTTTTTGAC TTTTCCGTAT TCAAAGTCGT TTTCGTCTCC CTTCTCACTT CGTTCTGCTG   
  
  
+ CATGCTACCC TCTTCCTCCT CCTCCTCTCT TATATATACC CATCTACCCC TCGAATTTTA ATCCCAAAAT   
  
  
+ ATCTCCAAAC CCACATCTCT TTTCTTTCCA CTGTTTTTCA CAACTTGATG AAATAATTGA ATCAACAAAA   
  
  
+ CATTTGGATA TTGTGAGAAA AATCTGAGC  

- GACAGTAGGT GCCGCCACGC ACCGGTCACC CGGCTAAGAC GGTAAAACAC GTTTAAAACT ATAAGATTTA   
  
  
- TACTAAACCA TAGGTTAGCT ACACAAAACT AATAATTTAG GTTACAACCA ATAAAGCCAA TAAACGTATA   
  
  
- ATAGCCTTTA CCAAACCTTA TACTTTAATA CTTGATACTT TCCGGTTCCC CTGGGTGGGT AGTACACCGT   
  
  
- CATGAGCAAG GGAGGCACTG AGATTATGAT ACATCTTAGT TTCTCTGCCC GGTTTATGGG TGAAGGTTGT   
  
  
- TGTGACTAAA ACAGAGGTTG AACCATTAAT GGTATACACG TTAAGCAGTC CACACTCCAA AATAGTGTTT   
  
  
- TCCCGAGCCA TAATCAATCT CACTTTAATT CTATAAATTT GAGAAGAAAA TAATCTCTAT GCCAACTACA   
  
  
- CCCTACAAAT TGTTCGGGGG AGTACACCCT AGGTTAATCA CCCAGTGTGT ACCCCCTAAG TGTGTAGCTG   
  
  
- TTGGTGTACT TCAGTTCCCC TGAGTGGAAT GTGTGACCGG GTTCACTGGG TGGAAGAAGC TCGCCCCAGT   
  
  
- TCCCCTGGGC CTGGGTGGGT AGTGTACTGT GATGTCATGC TCGGGTGAAG AACCGAAGCT ATGGTACATA   
  
  
- TTATAAGTTT CTCTGACCAG TTTTTAGGTG AAGGTTGTTA TAAATATAAT AGGGGTTGAA CCATTAATAG   
  
  
- TGGATGTGTA AGGTAGTTCA CACCTTAAAA TAGTATTTTC TGTAGTCATA ATCAATCTCA TTCTAATAGT   
  
  
- ATATGATAAT GAAAACAAAA AGATCCTGTT TACACCCTAA GTTGTTATGG TGTATATTAT AATAATTTAT   
  
  
- TAAACCAATT CTTTTCTTAT TGTTCTTTCA GGTAGCATAT TATACATGCC TTGCCATGTG GTGTATAATA   
  
  
- ATATGTTAAC TTTTTTTCAA AATAAAAGTT CACAAGTAAA TTAATATATT ACTATACATC ACAAAATGAA   
  
  
- AACGAGGCCC GTCTAACTTT TTTAAAAGTC TTTAAAAAGT TACAAAGATT TTATCTTTTG CTATGTGGTT   
  
  
- TACAGTAATA CGTTAATTTT ATTGTAAAAT TTATTTTTTG TTTTAAAAGG AGGTGAAAGT ATCATTGTAT   
  
  
- ACTAATAATG CTACACTGAA ATTAAAGCTC GGTTAACTTT TTAGAATAGT ATACAGACTC AGCACTCTAT   
  
  
- AATCTGATTG TGTGTAGTTA GGGGCTAAGG GGCGGCTTAC AACGAGGAAA CGACCGGTAT GATAAGATGC   
  
  
- GAGAAGAGTG CGAAAAACTG AAAAGGCATA AGTTTCAGCA AAAGCAGAGG GAAGAGTGAA GCAAGACGAC   
  
  
- GTACGATGGG AGAAGGAGGA GGAGGAGAGA ATATATATGG GTAGATGGGG AGCTTAAAAT TAGGGTTTTA   
  
  
- TAGAGGTTTG GGTGTAGAGA AAAGAAAGGT GACAAAAAGT GTTGAACTAC TTTATTAACT TAGTTGTTTT   
  
  
- GTAAACCTAT AACACTCTTT TTAGACTCG

+     LTR

| Site Name | Organism | Position | Strand | Matrix score. | sequence | function |
| --- | --- | --- | --- | --- | --- | --- |
| LTR | Hordeum vulgare | 123 | - | 6 | CCGAAA | cis-acting element involved in low-temperature responsiveness |

>018609   
+ CTGTCATCCA CGGCGGTGCG TGGCCAGTGG GCCGATTCTG CCATTTTGTG CAAATTTTGA TATTCTAAAT   
  
  
+ ATGATTTGGT ATCCAATCGA TGTGTTTTGA TTATTAAATC CAATGTTGGT TATTTCGGTT ATTTGCATAT   
  
  
+ TATCGGAAAT GGTTTGGAAT ATGAAATTAT GAACTATGAA AGGCCAAGGG GACCCACCCA TCATGTGGCA   
  
  
+ GTACTCGTTC CCTCCGTGAC TCTAATACTA TGTAGAATCA AAGAGACGGG CCAAATACCC ACTTCCAACA   
  
  
+ ACACTGATTT TGTCTCCAAC TTGGTAATTA CCATATGTGC AATTCGTCAG GTGTGAGGTT TTATCACAAA   
  
  
+ AGGGCTCGGT ATTAGTTAGA GTGAAATTAA GATATTTAAA CTCTTCTTTT ATTAGAGATA CGGTTGATGT   
  
  
+ GGGATGTTTA ACAAGCCCCC TCATGTGGGA TCCAATTAGT GGGTCACACA TGGGGGATTC ACACATCGAC   
  
  
+ AACCACATGA AGTCAAGGGG ACTCACCTTA CACACTGGCC CAAGTGACCC ACCTTCTTCG AGCGGGGTCA   
  
  
+ AGGGGACCCG GACCCACCCA TCACATGACA CTACAGTACG AGCCCACTTC TTGGCTTCGA TACCATGTAT   
  
  
+ AATATTCAAA GAGACTGGTC AAAAATCCAC TTCCAACAAT ATTTATATTA TCCCCAACTT GGTAATTATC   
  
  
+ ACCTACACAT TCCATCAAGT GTGGAATTTT ATCATAAAAG ACATCAGTAT TAGTTAGAGT AAGATTATCA   
  
  
+ TATACTATTA CTTTTGTTTT TCTAGGACAA ATGTGGGATT CAACAATACC ACATATAATA TTATTAAATA   
  
  
+ ATTTGGTTAA GAAAAGAATA ACAAGAAAGT CCATCGTATA ATATGTACGG AACGGTACAC CACATATTAT   
  
  
+ TATACAATTG AAAAAAAGTT TTATTTTCAA GTGTTCATTT AATTATATAA TGATATGTAG TGTTTTACTT   
  
  
+ TTGCTCCGGG CAGATTGAAA AAATTTTCAG AAATTTTTCA ATGTTTCTAA AATAGAAAAC GATACACCAA   
  
  
+ ATGTCATTAT GCAATTAAAA TAACATTTTA AATAAAAAAC AAAATTTTCC TCCACTTTCA TAGTAACATA   
  
  
+ TGATTATTAC GATGTGACTT TAATTTCGAG CCAATTGAAA AATCTTATCA TATGTCTGAG TCGTGAGATA   
  
  
+ TTAGACTAAC ACACATCAAT CCCCGATTCC CCGCCGAATG TTGCTCCTTT GCTGGCCATA CTATTCTACG   
  
  
+ CTCTTCTCAC GCTTTTTGAC TTTTCCGTAT TCAAAGTCGT TTTCGTCTCC CTTCTCACTT CGTTCTGCTG   
  
  
+ CATGCTACCC TCTTCCTCCT CCTCCTCTCT TATATATACC CATCTACCCC TCGAATTTTA ATCCCAAAAT   
  
  
+ ATCTCCAAAC CCACATCTCT TTTCTTTCCA CTGTTTTTCA CAACTTGATG AAATAATTGA ATCAACAAAA   
  
  
+ CATTTGGATA TTGTGAGAAA AATCTGAGC  

- GACAGTAGGT GCCGCCACGC ACCGGTCACC CGGCTAAGAC GGTAAAACAC GTTTAAAACT ATAAGATTTA   
  
  
- TACTAAACCA TAGGTTAGCT ACACAAAACT AATAATTTAG GTTACAACCA ATAAAGCCAA TAAACGTATA   
  
  
- ATAGCCTTTA CCAAACCTTA TACTTTAATA CTTGATACTT TCCGGTTCCC CTGGGTGGGT AGTACACCGT   
  
  
- CATGAGCAAG GGAGGCACTG AGATTATGAT ACATCTTAGT TTCTCTGCCC GGTTTATGGG TGAAGGTTGT   
  
  
- TGTGACTAAA ACAGAGGTTG AACCATTAAT GGTATACACG TTAAGCAGTC CACACTCCAA AATAGTGTTT   
  
  
- TCCCGAGCCA TAATCAATCT CACTTTAATT CTATAAATTT GAGAAGAAAA TAATCTCTAT GCCAACTACA   
  
  
- CCCTACAAAT TGTTCGGGGG AGTACACCCT AGGTTAATCA CCCAGTGTGT ACCCCCTAAG TGTGTAGCTG   
  
  
- TTGGTGTACT TCAGTTCCCC TGAGTGGAAT GTGTGACCGG GTTCACTGGG TGGAAGAAGC TCGCCCCAGT   
  
  
- TCCCCTGGGC CTGGGTGGGT AGTGTACTGT GATGTCATGC TCGGGTGAAG AACCGAAGCT ATGGTACATA   
  
  
- TTATAAGTTT CTCTGACCAG TTTTTAGGTG AAGGTTGTTA TAAATATAAT AGGGGTTGAA CCATTAATAG   
  
  
- TGGATGTGTA AGGTAGTTCA CACCTTAAAA TAGTATTTTC TGTAGTCATA ATCAATCTCA TTCTAATAGT   
  
  
- ATATGATAAT GAAAACAAAA AGATCCTGTT TACACCCTAA GTTGTTATGG TGTATATTAT AATAATTTAT   
  
  
- TAAACCAATT CTTTTCTTAT TGTTCTTTCA GGTAGCATAT TATACATGCC TTGCCATGTG GTGTATAATA   
  
  
- ATATGTTAAC TTTTTTTCAA AATAAAAGTT CACAAGTAAA TTAATATATT ACTATACATC ACAAAATGAA   
  
  
- AACGAGGCCC GTCTAACTTT TTTAAAAGTC TTTAAAAAGT TACAAAGATT TTATCTTTTG CTATGTGGTT   
  
  
- TACAGTAATA CGTTAATTTT ATTGTAAAAT TTATTTTTTG TTTTAAAAGG AGGTGAAAGT ATCATTGTAT   
  
  
- ACTAATAATG CTACACTGAA ATTAAAGCTC GGTTAACTTT TTAGAATAGT ATACAGACTC AGCACTCTAT   
  
  
- AATCTGATTG TGTGTAGTTA GGGGCTAAGG GGCGGCTTAC AACGAGGAAA CGACCGGTAT GATAAGATGC   
  
  
- GAGAAGAGTG CGAAAAACTG AAAAGGCATA AGTTTCAGCA AAAGCAGAGG GAAGAGTGAA GCAAGACGAC   
  
  
- GTACGATGGG AGAAGGAGGA GGAGGAGAGA ATATATATGG GTAGATGGGG AGCTTAAAAT TAGGGTTTTA   
  
  
- TAGAGGTTTG GGTGTAGAGA AAAGAAAGGT GACAAAAAGT GTTGAACTAC TTTATTAACT TAGTTGTTTT   
  
  
- GTAAACCTAT AACACTCTTT TTAGACTCG

+     MYB

| Site Name | Organism | Position | Strand | Matrix score. | sequence | function |
| --- | --- | --- | --- | --- | --- | --- |
| MYB | Arabidopsis thaliana | 844 | - | 6 | TAACCA |  |
| MYB | Arabidopsis thaliana | 490 | + | 6 | CAACCA |  |
| MYB | Arabidopsis thaliana | 117 | - | 6 | TAACCA |  |

>018609   
+ CTGTCATCCA CGGCGGTGCG TGGCCAGTGG GCCGATTCTG CCATTTTGTG CAAATTTTGA TATTCTAAAT   
  
  
+ ATGATTTGGT ATCCAATCGA TGTGTTTTGA TTATTAAATC CAATGTTGGT TATTTCGGTT ATTTGCATAT   
  
  
+ TATCGGAAAT GGTTTGGAAT ATGAAATTAT GAACTATGAA AGGCCAAGGG GACCCACCCA TCATGTGGCA   
  
  
+ GTACTCGTTC CCTCCGTGAC TCTAATACTA TGTAGAATCA AAGAGACGGG CCAAATACCC ACTTCCAACA   
  
  
+ ACACTGATTT TGTCTCCAAC TTGGTAATTA CCATATGTGC AATTCGTCAG GTGTGAGGTT TTATCACAAA   
  
  
+ AGGGCTCGGT ATTAGTTAGA GTGAAATTAA GATATTTAAA CTCTTCTTTT ATTAGAGATA CGGTTGATGT   
  
  
+ GGGATGTTTA ACAAGCCCCC TCATGTGGGA TCCAATTAGT GGGTCACACA TGGGGGATTC ACACATCGAC   
  
  
+ AACCACATGA AGTCAAGGGG ACTCACCTTA CACACTGGCC CAAGTGACCC ACCTTCTTCG AGCGGGGTCA   
  
  
+ AGGGGACCCG GACCCACCCA TCACATGACA CTACAGTACG AGCCCACTTC TTGGCTTCGA TACCATGTAT   
  
  
+ AATATTCAAA GAGACTGGTC AAAAATCCAC TTCCAACAAT ATTTATATTA TCCCCAACTT GGTAATTATC   
  
  
+ ACCTACACAT TCCATCAAGT GTGGAATTTT ATCATAAAAG ACATCAGTAT TAGTTAGAGT AAGATTATCA   
  
  
+ TATACTATTA CTTTTGTTTT TCTAGGACAA ATGTGGGATT CAACAATACC ACATATAATA TTATTAAATA   
  
  
+ ATTTGGTTAA GAAAAGAATA ACAAGAAAGT CCATCGTATA ATATGTACGG AACGGTACAC CACATATTAT   
  
  
+ TATACAATTG AAAAAAAGTT TTATTTTCAA GTGTTCATTT AATTATATAA TGATATGTAG TGTTTTACTT   
  
  
+ TTGCTCCGGG CAGATTGAAA AAATTTTCAG AAATTTTTCA ATGTTTCTAA AATAGAAAAC GATACACCAA   
  
  
+ ATGTCATTAT GCAATTAAAA TAACATTTTA AATAAAAAAC AAAATTTTCC TCCACTTTCA TAGTAACATA   
  
  
+ TGATTATTAC GATGTGACTT TAATTTCGAG CCAATTGAAA AATCTTATCA TATGTCTGAG TCGTGAGATA   
  
  
+ TTAGACTAAC ACACATCAAT CCCCGATTCC CCGCCGAATG TTGCTCCTTT GCTGGCCATA CTATTCTACG   
  
  
+ CTCTTCTCAC GCTTTTTGAC TTTTCCGTAT TCAAAGTCGT TTTCGTCTCC CTTCTCACTT CGTTCTGCTG   
  
  
+ CATGCTACCC TCTTCCTCCT CCTCCTCTCT TATATATACC CATCTACCCC TCGAATTTTA ATCCCAAAAT   
  
  
+ ATCTCCAAAC CCACATCTCT TTTCTTTCCA CTGTTTTTCA CAACTTGATG AAATAATTGA ATCAACAAAA   
  
  
+ CATTTGGATA TTGTGAGAAA AATCTGAGC  

- GACAGTAGGT GCCGCCACGC ACCGGTCACC CGGCTAAGAC GGTAAAACAC GTTTAAAACT ATAAGATTTA   
  
  
- TACTAAACCA TAGGTTAGCT ACACAAAACT AATAATTTAG GTTACAACCA ATAAAGCCAA TAAACGTATA   
  
  
- ATAGCCTTTA CCAAACCTTA TACTTTAATA CTTGATACTT TCCGGTTCCC CTGGGTGGGT AGTACACCGT   
  
  
- CATGAGCAAG GGAGGCACTG AGATTATGAT ACATCTTAGT TTCTCTGCCC GGTTTATGGG TGAAGGTTGT   
  
  
- TGTGACTAAA ACAGAGGTTG AACCATTAAT GGTATACACG TTAAGCAGTC CACACTCCAA AATAGTGTTT   
  
  
- TCCCGAGCCA TAATCAATCT CACTTTAATT CTATAAATTT GAGAAGAAAA TAATCTCTAT GCCAACTACA   
  
  
- CCCTACAAAT TGTTCGGGGG AGTACACCCT AGGTTAATCA CCCAGTGTGT ACCCCCTAAG TGTGTAGCTG   
  
  
- TTGGTGTACT TCAGTTCCCC TGAGTGGAAT GTGTGACCGG GTTCACTGGG TGGAAGAAGC TCGCCCCAGT   
  
  
- TCCCCTGGGC CTGGGTGGGT AGTGTACTGT GATGTCATGC TCGGGTGAAG AACCGAAGCT ATGGTACATA   
  
  
- TTATAAGTTT CTCTGACCAG TTTTTAGGTG AAGGTTGTTA TAAATATAAT AGGGGTTGAA CCATTAATAG   
  
  
- TGGATGTGTA AGGTAGTTCA CACCTTAAAA TAGTATTTTC TGTAGTCATA ATCAATCTCA TTCTAATAGT   
  
  
- ATATGATAAT GAAAACAAAA AGATCCTGTT TACACCCTAA GTTGTTATGG TGTATATTAT AATAATTTAT   
  
  
- TAAACCAATT CTTTTCTTAT TGTTCTTTCA GGTAGCATAT TATACATGCC TTGCCATGTG GTGTATAATA   
  
  
- ATATGTTAAC TTTTTTTCAA AATAAAAGTT CACAAGTAAA TTAATATATT ACTATACATC ACAAAATGAA   
  
  
- AACGAGGCCC GTCTAACTTT TTTAAAAGTC TTTAAAAAGT TACAAAGATT TTATCTTTTG CTATGTGGTT   
  
  
- TACAGTAATA CGTTAATTTT ATTGTAAAAT TTATTTTTTG TTTTAAAAGG AGGTGAAAGT ATCATTGTAT   
  
  
- ACTAATAATG CTACACTGAA ATTAAAGCTC GGTTAACTTT TTAGAATAGT ATACAGACTC AGCACTCTAT   
  
  
- AATCTGATTG TGTGTAGTTA GGGGCTAAGG GGCGGCTTAC AACGAGGAAA CGACCGGTAT GATAAGATGC   
  
  
- GAGAAGAGTG CGAAAAACTG AAAAGGCATA AGTTTCAGCA AAAGCAGAGG GAAGAGTGAA GCAAGACGAC   
  
  
- GTACGATGGG AGAAGGAGGA GGAGGAGAGA ATATATATGG GTAGATGGGG AGCTTAAAAT TAGGGTTTTA   
  
  
- TAGAGGTTTG GGTGTAGAGA AAAGAAAGGT GACAAAAAGT GTTGAACTAC TTTATTAACT TAGTTGTTTT   
  
  
- GTAAACCTAT AACACTCTTT TTAGACTCG

+     MYB-like sequence

| Site Name | Organism | Position | Strand | Matrix score. | sequence | function |
| --- | --- | --- | --- | --- | --- | --- |
| MYB-like sequence | Arabidopsis thaliana | 844 | - | 6 | TAACCA |  |
| MYB-like sequence | Arabidopsis thaliana | 117 | - | 6 | TAACCA |  |

>018609   
+ CTGTCATCCA CGGCGGTGCG TGGCCAGTGG GCCGATTCTG CCATTTTGTG CAAATTTTGA TATTCTAAAT   
  
  
+ ATGATTTGGT ATCCAATCGA TGTGTTTTGA TTATTAAATC CAATGTTGGT TATTTCGGTT ATTTGCATAT   
  
  
+ TATCGGAAAT GGTTTGGAAT ATGAAATTAT GAACTATGAA AGGCCAAGGG GACCCACCCA TCATGTGGCA   
  
  
+ GTACTCGTTC CCTCCGTGAC TCTAATACTA TGTAGAATCA AAGAGACGGG CCAAATACCC ACTTCCAACA   
  
  
+ ACACTGATTT TGTCTCCAAC TTGGTAATTA CCATATGTGC AATTCGTCAG GTGTGAGGTT TTATCACAAA   
  
  
+ AGGGCTCGGT ATTAGTTAGA GTGAAATTAA GATATTTAAA CTCTTCTTTT ATTAGAGATA CGGTTGATGT   
  
  
+ GGGATGTTTA ACAAGCCCCC TCATGTGGGA TCCAATTAGT GGGTCACACA TGGGGGATTC ACACATCGAC   
  
  
+ AACCACATGA AGTCAAGGGG ACTCACCTTA CACACTGGCC CAAGTGACCC ACCTTCTTCG AGCGGGGTCA   
  
  
+ AGGGGACCCG GACCCACCCA TCACATGACA CTACAGTACG AGCCCACTTC TTGGCTTCGA TACCATGTAT   
  
  
+ AATATTCAAA GAGACTGGTC AAAAATCCAC TTCCAACAAT ATTTATATTA TCCCCAACTT GGTAATTATC   
  
  
+ ACCTACACAT TCCATCAAGT GTGGAATTTT ATCATAAAAG ACATCAGTAT TAGTTAGAGT AAGATTATCA   
  
  
+ TATACTATTA CTTTTGTTTT TCTAGGACAA ATGTGGGATT CAACAATACC ACATATAATA TTATTAAATA   
  
  
+ ATTTGGTTAA GAAAAGAATA ACAAGAAAGT CCATCGTATA ATATGTACGG AACGGTACAC CACATATTAT   
  
  
+ TATACAATTG AAAAAAAGTT TTATTTTCAA GTGTTCATTT AATTATATAA TGATATGTAG TGTTTTACTT   
  
  
+ TTGCTCCGGG CAGATTGAAA AAATTTTCAG AAATTTTTCA ATGTTTCTAA AATAGAAAAC GATACACCAA   
  
  
+ ATGTCATTAT GCAATTAAAA TAACATTTTA AATAAAAAAC AAAATTTTCC TCCACTTTCA TAGTAACATA   
  
  
+ TGATTATTAC GATGTGACTT TAATTTCGAG CCAATTGAAA AATCTTATCA TATGTCTGAG TCGTGAGATA   
  
  
+ TTAGACTAAC ACACATCAAT CCCCGATTCC CCGCCGAATG TTGCTCCTTT GCTGGCCATA CTATTCTACG   
  
  
+ CTCTTCTCAC GCTTTTTGAC TTTTCCGTAT TCAAAGTCGT TTTCGTCTCC CTTCTCACTT CGTTCTGCTG   
  
  
+ CATGCTACCC TCTTCCTCCT CCTCCTCTCT TATATATACC CATCTACCCC TCGAATTTTA ATCCCAAAAT   
  
  
+ ATCTCCAAAC CCACATCTCT TTTCTTTCCA CTGTTTTTCA CAACTTGATG AAATAATTGA ATCAACAAAA   
  
  
+ CATTTGGATA TTGTGAGAAA AATCTGAGC  

- GACAGTAGGT GCCGCCACGC ACCGGTCACC CGGCTAAGAC GGTAAAACAC GTTTAAAACT ATAAGATTTA   
  
  
- TACTAAACCA TAGGTTAGCT ACACAAAACT AATAATTTAG GTTACAACCA ATAAAGCCAA TAAACGTATA   
  
  
- ATAGCCTTTA CCAAACCTTA TACTTTAATA CTTGATACTT TCCGGTTCCC CTGGGTGGGT AGTACACCGT   
  
  
- CATGAGCAAG GGAGGCACTG AGATTATGAT ACATCTTAGT TTCTCTGCCC GGTTTATGGG TGAAGGTTGT   
  
  
- TGTGACTAAA ACAGAGGTTG AACCATTAAT GGTATACACG TTAAGCAGTC CACACTCCAA AATAGTGTTT   
  
  
- TCCCGAGCCA TAATCAATCT CACTTTAATT CTATAAATTT GAGAAGAAAA TAATCTCTAT GCCAACTACA   
  
  
- CCCTACAAAT TGTTCGGGGG AGTACACCCT AGGTTAATCA CCCAGTGTGT ACCCCCTAAG TGTGTAGCTG   
  
  
- TTGGTGTACT TCAGTTCCCC TGAGTGGAAT GTGTGACCGG GTTCACTGGG TGGAAGAAGC TCGCCCCAGT   
  
  
- TCCCCTGGGC CTGGGTGGGT AGTGTACTGT GATGTCATGC TCGGGTGAAG AACCGAAGCT ATGGTACATA   
  
  
- TTATAAGTTT CTCTGACCAG TTTTTAGGTG AAGGTTGTTA TAAATATAAT AGGGGTTGAA CCATTAATAG   
  
  
- TGGATGTGTA AGGTAGTTCA CACCTTAAAA TAGTATTTTC TGTAGTCATA ATCAATCTCA TTCTAATAGT   
  
  
- ATATGATAAT GAAAACAAAA AGATCCTGTT TACACCCTAA GTTGTTATGG TGTATATTAT AATAATTTAT   
  
  
- TAAACCAATT CTTTTCTTAT TGTTCTTTCA GGTAGCATAT TATACATGCC TTGCCATGTG GTGTATAATA   
  
  
- ATATGTTAAC TTTTTTTCAA AATAAAAGTT CACAAGTAAA TTAATATATT ACTATACATC ACAAAATGAA   
  
  
- AACGAGGCCC GTCTAACTTT TTTAAAAGTC TTTAAAAAGT TACAAAGATT TTATCTTTTG CTATGTGGTT   
  
  
- TACAGTAATA CGTTAATTTT ATTGTAAAAT TTATTTTTTG TTTTAAAAGG AGGTGAAAGT ATCATTGTAT   
  
  
- ACTAATAATG CTACACTGAA ATTAAAGCTC GGTTAACTTT TTAGAATAGT ATACAGACTC AGCACTCTAT   
  
  
- AATCTGATTG TGTGTAGTTA GGGGCTAAGG GGCGGCTTAC AACGAGGAAA CGACCGGTAT GATAAGATGC   
  
  
- GAGAAGAGTG CGAAAAACTG AAAAGGCATA AGTTTCAGCA AAAGCAGAGG GAAGAGTGAA GCAAGACGAC   
  
  
- GTACGATGGG AGAAGGAGGA GGAGGAGAGA ATATATATGG GTAGATGGGG AGCTTAAAAT TAGGGTTTTA   
  
  
- TAGAGGTTTG GGTGTAGAGA AAAGAAAGGT GACAAAAAGT GTTGAACTAC TTTATTAACT TAGTTGTTTT   
  
  
- GTAAACCTAT AACACTCTTT TTAGACTCG

+     MYC

| Site Name | Organism | Position | Strand | Matrix score. | sequence | function |
| --- | --- | --- | --- | --- | --- | --- |
| MYC | Arabidopsis thaliana | 1471 | + | 6 | CATTTG |  |
| MYC | Arabidopsis thaliana | 1152 | - | 6 | CAATTG |  |
| MYC | Arabidopsis thaliana | 1048 | - | 6 | CATTTG |  |
| MYC | Arabidopsis thaliana | 915 | - | 6 | CAATTG |  |
| MYC | Arabidopsis thaliana | 798 | - | 6 | CATTTG |  |
| MYC | Arabidopsis thaliana | 582 | - | 6 | CATGTG |  |
| MYC | Arabidopsis thaliana | 494 | - | 6 | CATGTG |  |
| MYC | Arabidopsis thaliana | 467 | - | 6 | CATGTG |  |
| MYC | Arabidopsis thaliana | 442 | + | 6 | CATGTG |  |
| MYC | Arabidopsis thaliana | 202 | + | 6 | CATGTG |  |

>018609   
+ CTGTCATCCA CGGCGGTGCG TGGCCAGTGG GCCGATTCTG CCATTTTGTG CAAATTTTGA TATTCTAAAT   
  
  
+ ATGATTTGGT ATCCAATCGA TGTGTTTTGA TTATTAAATC CAATGTTGGT TATTTCGGTT ATTTGCATAT   
  
  
+ TATCGGAAAT GGTTTGGAAT ATGAAATTAT GAACTATGAA AGGCCAAGGG GACCCACCCA TCATGTGGCA   
  
  
+ GTACTCGTTC CCTCCGTGAC TCTAATACTA TGTAGAATCA AAGAGACGGG CCAAATACCC ACTTCCAACA   
  
  
+ ACACTGATTT TGTCTCCAAC TTGGTAATTA CCATATGTGC AATTCGTCAG GTGTGAGGTT TTATCACAAA   
  
  
+ AGGGCTCGGT ATTAGTTAGA GTGAAATTAA GATATTTAAA CTCTTCTTTT ATTAGAGATA CGGTTGATGT   
  
  
+ GGGATGTTTA ACAAGCCCCC TCATGTGGGA TCCAATTAGT GGGTCACACA TGGGGGATTC ACACATCGAC   
  
  
+ AACCACATGA AGTCAAGGGG ACTCACCTTA CACACTGGCC CAAGTGACCC ACCTTCTTCG AGCGGGGTCA   
  
  
+ AGGGGACCCG GACCCACCCA TCACATGACA CTACAGTACG AGCCCACTTC TTGGCTTCGA TACCATGTAT   
  
  
+ AATATTCAAA GAGACTGGTC AAAAATCCAC TTCCAACAAT ATTTATATTA TCCCCAACTT GGTAATTATC   
  
  
+ ACCTACACAT TCCATCAAGT GTGGAATTTT ATCATAAAAG ACATCAGTAT TAGTTAGAGT AAGATTATCA   
  
  
+ TATACTATTA CTTTTGTTTT TCTAGGACAA ATGTGGGATT CAACAATACC ACATATAATA TTATTAAATA   
  
  
+ ATTTGGTTAA GAAAAGAATA ACAAGAAAGT CCATCGTATA ATATGTACGG AACGGTACAC CACATATTAT   
  
  
+ TATACAATTG AAAAAAAGTT TTATTTTCAA GTGTTCATTT AATTATATAA TGATATGTAG TGTTTTACTT   
  
  
+ TTGCTCCGGG CAGATTGAAA AAATTTTCAG AAATTTTTCA ATGTTTCTAA AATAGAAAAC GATACACCAA   
  
  
+ ATGTCATTAT GCAATTAAAA TAACATTTTA AATAAAAAAC AAAATTTTCC TCCACTTTCA TAGTAACATA   
  
  
+ TGATTATTAC GATGTGACTT TAATTTCGAG CCAATTGAAA AATCTTATCA TATGTCTGAG TCGTGAGATA   
  
  
+ TTAGACTAAC ACACATCAAT CCCCGATTCC CCGCCGAATG TTGCTCCTTT GCTGGCCATA CTATTCTACG   
  
  
+ CTCTTCTCAC GCTTTTTGAC TTTTCCGTAT TCAAAGTCGT TTTCGTCTCC CTTCTCACTT CGTTCTGCTG   
  
  
+ CATGCTACCC TCTTCCTCCT CCTCCTCTCT TATATATACC CATCTACCCC TCGAATTTTA ATCCCAAAAT   
  
  
+ ATCTCCAAAC CCACATCTCT TTTCTTTCCA CTGTTTTTCA CAACTTGATG AAATAATTGA ATCAACAAAA   
  
  
+ CATTTGGATA TTGTGAGAAA AATCTGAGC  

- GACAGTAGGT GCCGCCACGC ACCGGTCACC CGGCTAAGAC GGTAAAACAC GTTTAAAACT ATAAGATTTA   
  
  
- TACTAAACCA TAGGTTAGCT ACACAAAACT AATAATTTAG GTTACAACCA ATAAAGCCAA TAAACGTATA   
  
  
- ATAGCCTTTA CCAAACCTTA TACTTTAATA CTTGATACTT TCCGGTTCCC CTGGGTGGGT AGTACACCGT   
  
  
- CATGAGCAAG GGAGGCACTG AGATTATGAT ACATCTTAGT TTCTCTGCCC GGTTTATGGG TGAAGGTTGT   
  
  
- TGTGACTAAA ACAGAGGTTG AACCATTAAT GGTATACACG TTAAGCAGTC CACACTCCAA AATAGTGTTT   
  
  
- TCCCGAGCCA TAATCAATCT CACTTTAATT CTATAAATTT GAGAAGAAAA TAATCTCTAT GCCAACTACA   
  
  
- CCCTACAAAT TGTTCGGGGG AGTACACCCT AGGTTAATCA CCCAGTGTGT ACCCCCTAAG TGTGTAGCTG   
  
  
- TTGGTGTACT TCAGTTCCCC TGAGTGGAAT GTGTGACCGG GTTCACTGGG TGGAAGAAGC TCGCCCCAGT   
  
  
- TCCCCTGGGC CTGGGTGGGT AGTGTACTGT GATGTCATGC TCGGGTGAAG AACCGAAGCT ATGGTACATA   
  
  
- TTATAAGTTT CTCTGACCAG TTTTTAGGTG AAGGTTGTTA TAAATATAAT AGGGGTTGAA CCATTAATAG   
  
  
- TGGATGTGTA AGGTAGTTCA CACCTTAAAA TAGTATTTTC TGTAGTCATA ATCAATCTCA TTCTAATAGT   
  
  
- ATATGATAAT GAAAACAAAA AGATCCTGTT TACACCCTAA GTTGTTATGG TGTATATTAT AATAATTTAT   
  
  
- TAAACCAATT CTTTTCTTAT TGTTCTTTCA GGTAGCATAT TATACATGCC TTGCCATGTG GTGTATAATA   
  
  
- ATATGTTAAC TTTTTTTCAA AATAAAAGTT CACAAGTAAA TTAATATATT ACTATACATC ACAAAATGAA   
  
  
- AACGAGGCCC GTCTAACTTT TTTAAAAGTC TTTAAAAAGT TACAAAGATT TTATCTTTTG CTATGTGGTT   
  
  
- TACAGTAATA CGTTAATTTT ATTGTAAAAT TTATTTTTTG TTTTAAAAGG AGGTGAAAGT ATCATTGTAT   
  
  
- ACTAATAATG CTACACTGAA ATTAAAGCTC GGTTAACTTT TTAGAATAGT ATACAGACTC AGCACTCTAT   
  
  
- AATCTGATTG TGTGTAGTTA GGGGCTAAGG GGCGGCTTAC AACGAGGAAA CGACCGGTAT GATAAGATGC   
  
  
- GAGAAGAGTG CGAAAAACTG AAAAGGCATA AGTTTCAGCA AAAGCAGAGG GAAGAGTGAA GCAAGACGAC   
  
  
- GTACGATGGG AGAAGGAGGA GGAGGAGAGA ATATATATGG GTAGATGGGG AGCTTAAAAT TAGGGTTTTA   
  
  
- TAGAGGTTTG GGTGTAGAGA AAAGAAAGGT GACAAAAAGT GTTGAACTAC TTTATTAACT TAGTTGTTTT   
  
  
- GTAAACCTAT AACACTCTTT TTAGACTCG

+     Myc

| Site Name | Organism | Position | Strand | Matrix score. | sequence | function |
| --- | --- | --- | --- | --- | --- | --- |
| Myc | Arabidopsis thaliana | 1356 | + | 7 | TCTCTTA |  |

>018609   
+ CTGTCATCCA CGGCGGTGCG TGGCCAGTGG GCCGATTCTG CCATTTTGTG CAAATTTTGA TATTCTAAAT   
  
  
+ ATGATTTGGT ATCCAATCGA TGTGTTTTGA TTATTAAATC CAATGTTGGT TATTTCGGTT ATTTGCATAT   
  
  
+ TATCGGAAAT GGTTTGGAAT ATGAAATTAT GAACTATGAA AGGCCAAGGG GACCCACCCA TCATGTGGCA   
  
  
+ GTACTCGTTC CCTCCGTGAC TCTAATACTA TGTAGAATCA AAGAGACGGG CCAAATACCC ACTTCCAACA   
  
  
+ ACACTGATTT TGTCTCCAAC TTGGTAATTA CCATATGTGC AATTCGTCAG GTGTGAGGTT TTATCACAAA   
  
  
+ AGGGCTCGGT ATTAGTTAGA GTGAAATTAA GATATTTAAA CTCTTCTTTT ATTAGAGATA CGGTTGATGT   
  
  
+ GGGATGTTTA ACAAGCCCCC TCATGTGGGA TCCAATTAGT GGGTCACACA TGGGGGATTC ACACATCGAC   
  
  
+ AACCACATGA AGTCAAGGGG ACTCACCTTA CACACTGGCC CAAGTGACCC ACCTTCTTCG AGCGGGGTCA   
  
  
+ AGGGGACCCG GACCCACCCA TCACATGACA CTACAGTACG AGCCCACTTC TTGGCTTCGA TACCATGTAT   
  
  
+ AATATTCAAA GAGACTGGTC AAAAATCCAC TTCCAACAAT ATTTATATTA TCCCCAACTT GGTAATTATC   
  
  
+ ACCTACACAT TCCATCAAGT GTGGAATTTT ATCATAAAAG ACATCAGTAT TAGTTAGAGT AAGATTATCA   
  
  
+ TATACTATTA CTTTTGTTTT TCTAGGACAA ATGTGGGATT CAACAATACC ACATATAATA TTATTAAATA   
  
  
+ ATTTGGTTAA GAAAAGAATA ACAAGAAAGT CCATCGTATA ATATGTACGG AACGGTACAC CACATATTAT   
  
  
+ TATACAATTG AAAAAAAGTT TTATTTTCAA GTGTTCATTT AATTATATAA TGATATGTAG TGTTTTACTT   
  
  
+ TTGCTCCGGG CAGATTGAAA AAATTTTCAG AAATTTTTCA ATGTTTCTAA AATAGAAAAC GATACACCAA   
  
  
+ ATGTCATTAT GCAATTAAAA TAACATTTTA AATAAAAAAC AAAATTTTCC TCCACTTTCA TAGTAACATA   
  
  
+ TGATTATTAC GATGTGACTT TAATTTCGAG CCAATTGAAA AATCTTATCA TATGTCTGAG TCGTGAGATA   
  
  
+ TTAGACTAAC ACACATCAAT CCCCGATTCC CCGCCGAATG TTGCTCCTTT GCTGGCCATA CTATTCTACG   
  
  
+ CTCTTCTCAC GCTTTTTGAC TTTTCCGTAT TCAAAGTCGT TTTCGTCTCC CTTCTCACTT CGTTCTGCTG   
  
  
+ CATGCTACCC TCTTCCTCCT CCTCCTCTCT TATATATACC CATCTACCCC TCGAATTTTA ATCCCAAAAT   
  
  
+ ATCTCCAAAC CCACATCTCT TTTCTTTCCA CTGTTTTTCA CAACTTGATG AAATAATTGA ATCAACAAAA   
  
  
+ CATTTGGATA TTGTGAGAAA AATCTGAGC  

- GACAGTAGGT GCCGCCACGC ACCGGTCACC CGGCTAAGAC GGTAAAACAC GTTTAAAACT ATAAGATTTA   
  
  
- TACTAAACCA TAGGTTAGCT ACACAAAACT AATAATTTAG GTTACAACCA ATAAAGCCAA TAAACGTATA   
  
  
- ATAGCCTTTA CCAAACCTTA TACTTTAATA CTTGATACTT TCCGGTTCCC CTGGGTGGGT AGTACACCGT   
  
  
- CATGAGCAAG GGAGGCACTG AGATTATGAT ACATCTTAGT TTCTCTGCCC GGTTTATGGG TGAAGGTTGT   
  
  
- TGTGACTAAA ACAGAGGTTG AACCATTAAT GGTATACACG TTAAGCAGTC CACACTCCAA AATAGTGTTT   
  
  
- TCCCGAGCCA TAATCAATCT CACTTTAATT CTATAAATTT GAGAAGAAAA TAATCTCTAT GCCAACTACA   
  
  
- CCCTACAAAT TGTTCGGGGG AGTACACCCT AGGTTAATCA CCCAGTGTGT ACCCCCTAAG TGTGTAGCTG   
  
  
- TTGGTGTACT TCAGTTCCCC TGAGTGGAAT GTGTGACCGG GTTCACTGGG TGGAAGAAGC TCGCCCCAGT   
  
  
- TCCCCTGGGC CTGGGTGGGT AGTGTACTGT GATGTCATGC TCGGGTGAAG AACCGAAGCT ATGGTACATA   
  
  
- TTATAAGTTT CTCTGACCAG TTTTTAGGTG AAGGTTGTTA TAAATATAAT AGGGGTTGAA CCATTAATAG   
  
  
- TGGATGTGTA AGGTAGTTCA CACCTTAAAA TAGTATTTTC TGTAGTCATA ATCAATCTCA TTCTAATAGT   
  
  
- ATATGATAAT GAAAACAAAA AGATCCTGTT TACACCCTAA GTTGTTATGG TGTATATTAT AATAATTTAT   
  
  
- TAAACCAATT CTTTTCTTAT TGTTCTTTCA GGTAGCATAT TATACATGCC TTGCCATGTG GTGTATAATA   
  
  
- ATATGTTAAC TTTTTTTCAA AATAAAAGTT CACAAGTAAA TTAATATATT ACTATACATC ACAAAATGAA   
  
  
- AACGAGGCCC GTCTAACTTT TTTAAAAGTC TTTAAAAAGT TACAAAGATT TTATCTTTTG CTATGTGGTT   
  
  
- TACAGTAATA CGTTAATTTT ATTGTAAAAT TTATTTTTTG TTTTAAAAGG AGGTGAAAGT ATCATTGTAT   
  
  
- ACTAATAATG CTACACTGAA ATTAAAGCTC GGTTAACTTT TTAGAATAGT ATACAGACTC AGCACTCTAT   
  
  
- AATCTGATTG TGTGTAGTTA GGGGCTAAGG GGCGGCTTAC AACGAGGAAA CGACCGGTAT GATAAGATGC   
  
  
- GAGAAGAGTG CGAAAAACTG AAAAGGCATA AGTTTCAGCA AAAGCAGAGG GAAGAGTGAA GCAAGACGAC   
  
  
- GTACGATGGG AGAAGGAGGA GGAGGAGAGA ATATATATGG GTAGATGGGG AGCTTAAAAT TAGGGTTTTA   
  
  
- TAGAGGTTTG GGTGTAGAGA AAAGAAAGGT GACAAAAAGT GTTGAACTAC TTTATTAACT TAGTTGTTTT   
  
  
- GTAAACCTAT AACACTCTTT TTAGACTCG

+     O2-site

| Site Name | Organism | Position | Strand | Matrix score. | sequence | function |
| --- | --- | --- | --- | --- | --- | --- |
| O2-site | Zea mays | 413 | + | 9 | GATGATGTGG | cis-acting regulatory element involved in zein metabolism regulation |

>018609   
+ CTGTCATCCA CGGCGGTGCG TGGCCAGTGG GCCGATTCTG CCATTTTGTG CAAATTTTGA TATTCTAAAT   
  
  
+ ATGATTTGGT ATCCAATCGA TGTGTTTTGA TTATTAAATC CAATGTTGGT TATTTCGGTT ATTTGCATAT   
  
  
+ TATCGGAAAT GGTTTGGAAT ATGAAATTAT GAACTATGAA AGGCCAAGGG GACCCACCCA TCATGTGGCA   
  
  
+ GTACTCGTTC CCTCCGTGAC TCTAATACTA TGTAGAATCA AAGAGACGGG CCAAATACCC ACTTCCAACA   
  
  
+ ACACTGATTT TGTCTCCAAC TTGGTAATTA CCATATGTGC AATTCGTCAG GTGTGAGGTT TTATCACAAA   
  
  
+ AGGGCTCGGT ATTAGTTAGA GTGAAATTAA GATATTTAAA CTCTTCTTTT ATTAGAGATA CGGTTGATGT   
  
  
+ GGGATGTTTA ACAAGCCCCC TCATGTGGGA TCCAATTAGT GGGTCACACA TGGGGGATTC ACACATCGAC   
  
  
+ AACCACATGA AGTCAAGGGG ACTCACCTTA CACACTGGCC CAAGTGACCC ACCTTCTTCG AGCGGGGTCA   
  
  
+ AGGGGACCCG GACCCACCCA TCACATGACA CTACAGTACG AGCCCACTTC TTGGCTTCGA TACCATGTAT   
  
  
+ AATATTCAAA GAGACTGGTC AAAAATCCAC TTCCAACAAT ATTTATATTA TCCCCAACTT GGTAATTATC   
  
  
+ ACCTACACAT TCCATCAAGT GTGGAATTTT ATCATAAAAG ACATCAGTAT TAGTTAGAGT AAGATTATCA   
  
  
+ TATACTATTA CTTTTGTTTT TCTAGGACAA ATGTGGGATT CAACAATACC ACATATAATA TTATTAAATA   
  
  
+ ATTTGGTTAA GAAAAGAATA ACAAGAAAGT CCATCGTATA ATATGTACGG AACGGTACAC CACATATTAT   
  
  
+ TATACAATTG AAAAAAAGTT TTATTTTCAA GTGTTCATTT AATTATATAA TGATATGTAG TGTTTTACTT   
  
  
+ TTGCTCCGGG CAGATTGAAA AAATTTTCAG AAATTTTTCA ATGTTTCTAA AATAGAAAAC GATACACCAA   
  
  
+ ATGTCATTAT GCAATTAAAA TAACATTTTA AATAAAAAAC AAAATTTTCC TCCACTTTCA TAGTAACATA   
  
  
+ TGATTATTAC GATGTGACTT TAATTTCGAG CCAATTGAAA AATCTTATCA TATGTCTGAG TCGTGAGATA   
  
  
+ TTAGACTAAC ACACATCAAT CCCCGATTCC CCGCCGAATG TTGCTCCTTT GCTGGCCATA CTATTCTACG   
  
  
+ CTCTTCTCAC GCTTTTTGAC TTTTCCGTAT TCAAAGTCGT TTTCGTCTCC CTTCTCACTT CGTTCTGCTG   
  
  
+ CATGCTACCC TCTTCCTCCT CCTCCTCTCT TATATATACC CATCTACCCC TCGAATTTTA ATCCCAAAAT   
  
  
+ ATCTCCAAAC CCACATCTCT TTTCTTTCCA CTGTTTTTCA CAACTTGATG AAATAATTGA ATCAACAAAA   
  
  
+ CATTTGGATA TTGTGAGAAA AATCTGAGC  

- GACAGTAGGT GCCGCCACGC ACCGGTCACC CGGCTAAGAC GGTAAAACAC GTTTAAAACT ATAAGATTTA   
  
  
- TACTAAACCA TAGGTTAGCT ACACAAAACT AATAATTTAG GTTACAACCA ATAAAGCCAA TAAACGTATA   
  
  
- ATAGCCTTTA CCAAACCTTA TACTTTAATA CTTGATACTT TCCGGTTCCC CTGGGTGGGT AGTACACCGT   
  
  
- CATGAGCAAG GGAGGCACTG AGATTATGAT ACATCTTAGT TTCTCTGCCC GGTTTATGGG TGAAGGTTGT   
  
  
- TGTGACTAAA ACAGAGGTTG AACCATTAAT GGTATACACG TTAAGCAGTC CACACTCCAA AATAGTGTTT   
  
  
- TCCCGAGCCA TAATCAATCT CACTTTAATT CTATAAATTT GAGAAGAAAA TAATCTCTAT GCCAACTACA   
  
  
- CCCTACAAAT TGTTCGGGGG AGTACACCCT AGGTTAATCA CCCAGTGTGT ACCCCCTAAG TGTGTAGCTG   
  
  
- TTGGTGTACT TCAGTTCCCC TGAGTGGAAT GTGTGACCGG GTTCACTGGG TGGAAGAAGC TCGCCCCAGT   
  
  
- TCCCCTGGGC CTGGGTGGGT AGTGTACTGT GATGTCATGC TCGGGTGAAG AACCGAAGCT ATGGTACATA   
  
  
- TTATAAGTTT CTCTGACCAG TTTTTAGGTG AAGGTTGTTA TAAATATAAT AGGGGTTGAA CCATTAATAG   
  
  
- TGGATGTGTA AGGTAGTTCA CACCTTAAAA TAGTATTTTC TGTAGTCATA ATCAATCTCA TTCTAATAGT   
  
  
- ATATGATAAT GAAAACAAAA AGATCCTGTT TACACCCTAA GTTGTTATGG TGTATATTAT AATAATTTAT   
  
  
- TAAACCAATT CTTTTCTTAT TGTTCTTTCA GGTAGCATAT TATACATGCC TTGCCATGTG GTGTATAATA   
  
  
- ATATGTTAAC TTTTTTTCAA AATAAAAGTT CACAAGTAAA TTAATATATT ACTATACATC ACAAAATGAA   
  
  
- AACGAGGCCC GTCTAACTTT TTTAAAAGTC TTTAAAAAGT TACAAAGATT TTATCTTTTG CTATGTGGTT   
  
  
- TACAGTAATA CGTTAATTTT ATTGTAAAAT TTATTTTTTG TTTTAAAAGG AGGTGAAAGT ATCATTGTAT   
  
  
- ACTAATAATG CTACACTGAA ATTAAAGCTC GGTTAACTTT TTAGAATAGT ATACAGACTC AGCACTCTAT   
  
  
- AATCTGATTG TGTGTAGTTA GGGGCTAAGG GGCGGCTTAC AACGAGGAAA CGACCGGTAT GATAAGATGC   
  
  
- GAGAAGAGTG CGAAAAACTG AAAAGGCATA AGTTTCAGCA AAAGCAGAGG GAAGAGTGAA GCAAGACGAC   
  
  
- GTACGATGGG AGAAGGAGGA GGAGGAGAGA ATATATATGG GTAGATGGGG AGCTTAAAAT TAGGGTTTTA   
  
  
- TAGAGGTTTG GGTGTAGAGA AAAGAAAGGT GACAAAAAGT GTTGAACTAC TTTATTAACT TAGTTGTTTT   
  
  
- GTAAACCTAT AACACTCTTT TTAGACTCG

+     P-box

| Site Name | Organism | Position | Strand | Matrix score. | sequence | function |
| --- | --- | --- | --- | --- | --- | --- |
| P-box | Oryza sativa | 347 | - | 7 | CCTTTTG | gibberellin-responsive element |

>018609   
+ CTGTCATCCA CGGCGGTGCG TGGCCAGTGG GCCGATTCTG CCATTTTGTG CAAATTTTGA TATTCTAAAT   
  
  
+ ATGATTTGGT ATCCAATCGA TGTGTTTTGA TTATTAAATC CAATGTTGGT TATTTCGGTT ATTTGCATAT   
  
  
+ TATCGGAAAT GGTTTGGAAT ATGAAATTAT GAACTATGAA AGGCCAAGGG GACCCACCCA TCATGTGGCA   
  
  
+ GTACTCGTTC CCTCCGTGAC TCTAATACTA TGTAGAATCA AAGAGACGGG CCAAATACCC ACTTCCAACA   
  
  
+ ACACTGATTT TGTCTCCAAC TTGGTAATTA CCATATGTGC AATTCGTCAG GTGTGAGGTT TTATCACAAA   
  
  
+ AGGGCTCGGT ATTAGTTAGA GTGAAATTAA GATATTTAAA CTCTTCTTTT ATTAGAGATA CGGTTGATGT   
  
  
+ GGGATGTTTA ACAAGCCCCC TCATGTGGGA TCCAATTAGT GGGTCACACA TGGGGGATTC ACACATCGAC   
  
  
+ AACCACATGA AGTCAAGGGG ACTCACCTTA CACACTGGCC CAAGTGACCC ACCTTCTTCG AGCGGGGTCA   
  
  
+ AGGGGACCCG GACCCACCCA TCACATGACA CTACAGTACG AGCCCACTTC TTGGCTTCGA TACCATGTAT   
  
  
+ AATATTCAAA GAGACTGGTC AAAAATCCAC TTCCAACAAT ATTTATATTA TCCCCAACTT GGTAATTATC   
  
  
+ ACCTACACAT TCCATCAAGT GTGGAATTTT ATCATAAAAG ACATCAGTAT TAGTTAGAGT AAGATTATCA   
  
  
+ TATACTATTA CTTTTGTTTT TCTAGGACAA ATGTGGGATT CAACAATACC ACATATAATA TTATTAAATA   
  
  
+ ATTTGGTTAA GAAAAGAATA ACAAGAAAGT CCATCGTATA ATATGTACGG AACGGTACAC CACATATTAT   
  
  
+ TATACAATTG AAAAAAAGTT TTATTTTCAA GTGTTCATTT AATTATATAA TGATATGTAG TGTTTTACTT   
  
  
+ TTGCTCCGGG CAGATTGAAA AAATTTTCAG AAATTTTTCA ATGTTTCTAA AATAGAAAAC GATACACCAA   
  
  
+ ATGTCATTAT GCAATTAAAA TAACATTTTA AATAAAAAAC AAAATTTTCC TCCACTTTCA TAGTAACATA   
  
  
+ TGATTATTAC GATGTGACTT TAATTTCGAG CCAATTGAAA AATCTTATCA TATGTCTGAG TCGTGAGATA   
  
  
+ TTAGACTAAC ACACATCAAT CCCCGATTCC CCGCCGAATG TTGCTCCTTT GCTGGCCATA CTATTCTACG   
  
  
+ CTCTTCTCAC GCTTTTTGAC TTTTCCGTAT TCAAAGTCGT TTTCGTCTCC CTTCTCACTT CGTTCTGCTG   
  
  
+ CATGCTACCC TCTTCCTCCT CCTCCTCTCT TATATATACC CATCTACCCC TCGAATTTTA ATCCCAAAAT   
  
  
+ ATCTCCAAAC CCACATCTCT TTTCTTTCCA CTGTTTTTCA CAACTTGATG AAATAATTGA ATCAACAAAA   
  
  
+ CATTTGGATA TTGTGAGAAA AATCTGAGC  

- GACAGTAGGT GCCGCCACGC ACCGGTCACC CGGCTAAGAC GGTAAAACAC GTTTAAAACT ATAAGATTTA   
  
  
- TACTAAACCA TAGGTTAGCT ACACAAAACT AATAATTTAG GTTACAACCA ATAAAGCCAA TAAACGTATA   
  
  
- ATAGCCTTTA CCAAACCTTA TACTTTAATA CTTGATACTT TCCGGTTCCC CTGGGTGGGT AGTACACCGT   
  
  
- CATGAGCAAG GGAGGCACTG AGATTATGAT ACATCTTAGT TTCTCTGCCC GGTTTATGGG TGAAGGTTGT   
  
  
- TGTGACTAAA ACAGAGGTTG AACCATTAAT GGTATACACG TTAAGCAGTC CACACTCCAA AATAGTGTTT   
  
  
- TCCCGAGCCA TAATCAATCT CACTTTAATT CTATAAATTT GAGAAGAAAA TAATCTCTAT GCCAACTACA   
  
  
- CCCTACAAAT TGTTCGGGGG AGTACACCCT AGGTTAATCA CCCAGTGTGT ACCCCCTAAG TGTGTAGCTG   
  
  
- TTGGTGTACT TCAGTTCCCC TGAGTGGAAT GTGTGACCGG GTTCACTGGG TGGAAGAAGC TCGCCCCAGT   
  
  
- TCCCCTGGGC CTGGGTGGGT AGTGTACTGT GATGTCATGC TCGGGTGAAG AACCGAAGCT ATGGTACATA   
  
  
- TTATAAGTTT CTCTGACCAG TTTTTAGGTG AAGGTTGTTA TAAATATAAT AGGGGTTGAA CCATTAATAG   
  
  
- TGGATGTGTA AGGTAGTTCA CACCTTAAAA TAGTATTTTC TGTAGTCATA ATCAATCTCA TTCTAATAGT   
  
  
- ATATGATAAT GAAAACAAAA AGATCCTGTT TACACCCTAA GTTGTTATGG TGTATATTAT AATAATTTAT   
  
  
- TAAACCAATT CTTTTCTTAT TGTTCTTTCA GGTAGCATAT TATACATGCC TTGCCATGTG GTGTATAATA   
  
  
- ATATGTTAAC TTTTTTTCAA AATAAAAGTT CACAAGTAAA TTAATATATT ACTATACATC ACAAAATGAA   
  
  
- AACGAGGCCC GTCTAACTTT TTTAAAAGTC TTTAAAAAGT TACAAAGATT TTATCTTTTG CTATGTGGTT   
  
  
- TACAGTAATA CGTTAATTTT ATTGTAAAAT TTATTTTTTG TTTTAAAAGG AGGTGAAAGT ATCATTGTAT   
  
  
- ACTAATAATG CTACACTGAA ATTAAAGCTC GGTTAACTTT TTAGAATAGT ATACAGACTC AGCACTCTAT   
  
  
- AATCTGATTG TGTGTAGTTA GGGGCTAAGG GGCGGCTTAC AACGAGGAAA CGACCGGTAT GATAAGATGC   
  
  
- GAGAAGAGTG CGAAAAACTG AAAAGGCATA AGTTTCAGCA AAAGCAGAGG GAAGAGTGAA GCAAGACGAC   
  
  
- GTACGATGGG AGAAGGAGGA GGAGGAGAGA ATATATATGG GTAGATGGGG AGCTTAAAAT TAGGGTTTTA   
  
  
- TAGAGGTTTG GGTGTAGAGA AAAGAAAGGT GACAAAAAGT GTTGAACTAC TTTATTAACT TAGTTGTTTT   
  
  
- GTAAACCTAT AACACTCTTT TTAGACTCG

+     STRE

| Site Name | Organism | Position | Strand | Matrix score. | sequence | function |
| --- | --- | --- | --- | --- | --- | --- |
| STRE | Arabidopsis thaliana | 1377 | - | 5 | AGGGG |  |
| STRE | Arabidopsis thaliana | 561 | + | 5 | AGGGG |  |
| STRE | Arabidopsis thaliana | 506 | + | 5 | AGGGG |  |
| STRE | Arabidopsis thaliana | 437 | - | 5 | AGGGG |  |
| STRE | Arabidopsis thaliana | 187 | + | 5 | AGGGG |  |

>018609   
+ CTGTCATCCA CGGCGGTGCG TGGCCAGTGG GCCGATTCTG CCATTTTGTG CAAATTTTGA TATTCTAAAT   
  
  
+ ATGATTTGGT ATCCAATCGA TGTGTTTTGA TTATTAAATC CAATGTTGGT TATTTCGGTT ATTTGCATAT   
  
  
+ TATCGGAAAT GGTTTGGAAT ATGAAATTAT GAACTATGAA AGGCCAAGGG GACCCACCCA TCATGTGGCA   
  
  
+ GTACTCGTTC CCTCCGTGAC TCTAATACTA TGTAGAATCA AAGAGACGGG CCAAATACCC ACTTCCAACA   
  
  
+ ACACTGATTT TGTCTCCAAC TTGGTAATTA CCATATGTGC AATTCGTCAG GTGTGAGGTT TTATCACAAA   
  
  
+ AGGGCTCGGT ATTAGTTAGA GTGAAATTAA GATATTTAAA CTCTTCTTTT ATTAGAGATA CGGTTGATGT   
  
  
+ GGGATGTTTA ACAAGCCCCC TCATGTGGGA TCCAATTAGT GGGTCACACA TGGGGGATTC ACACATCGAC   
  
  
+ AACCACATGA AGTCAAGGGG ACTCACCTTA CACACTGGCC CAAGTGACCC ACCTTCTTCG AGCGGGGTCA   
  
  
+ AGGGGACCCG GACCCACCCA TCACATGACA CTACAGTACG AGCCCACTTC TTGGCTTCGA TACCATGTAT   
  
  
+ AATATTCAAA GAGACTGGTC AAAAATCCAC TTCCAACAAT ATTTATATTA TCCCCAACTT GGTAATTATC   
  
  
+ ACCTACACAT TCCATCAAGT GTGGAATTTT ATCATAAAAG ACATCAGTAT TAGTTAGAGT AAGATTATCA   
  
  
+ TATACTATTA CTTTTGTTTT TCTAGGACAA ATGTGGGATT CAACAATACC ACATATAATA TTATTAAATA   
  
  
+ ATTTGGTTAA GAAAAGAATA ACAAGAAAGT CCATCGTATA ATATGTACGG AACGGTACAC CACATATTAT   
  
  
+ TATACAATTG AAAAAAAGTT TTATTTTCAA GTGTTCATTT AATTATATAA TGATATGTAG TGTTTTACTT   
  
  
+ TTGCTCCGGG CAGATTGAAA AAATTTTCAG AAATTTTTCA ATGTTTCTAA AATAGAAAAC GATACACCAA   
  
  
+ ATGTCATTAT GCAATTAAAA TAACATTTTA AATAAAAAAC AAAATTTTCC TCCACTTTCA TAGTAACATA   
  
  
+ TGATTATTAC GATGTGACTT TAATTTCGAG CCAATTGAAA AATCTTATCA TATGTCTGAG TCGTGAGATA   
  
  
+ TTAGACTAAC ACACATCAAT CCCCGATTCC CCGCCGAATG TTGCTCCTTT GCTGGCCATA CTATTCTACG   
  
  
+ CTCTTCTCAC GCTTTTTGAC TTTTCCGTAT TCAAAGTCGT TTTCGTCTCC CTTCTCACTT CGTTCTGCTG   
  
  
+ CATGCTACCC TCTTCCTCCT CCTCCTCTCT TATATATACC CATCTACCCC TCGAATTTTA ATCCCAAAAT   
  
  
+ ATCTCCAAAC CCACATCTCT TTTCTTTCCA CTGTTTTTCA CAACTTGATG AAATAATTGA ATCAACAAAA   
  
  
+ CATTTGGATA TTGTGAGAAA AATCTGAGC  

- GACAGTAGGT GCCGCCACGC ACCGGTCACC CGGCTAAGAC GGTAAAACAC GTTTAAAACT ATAAGATTTA   
  
  
- TACTAAACCA TAGGTTAGCT ACACAAAACT AATAATTTAG GTTACAACCA ATAAAGCCAA TAAACGTATA   
  
  
- ATAGCCTTTA CCAAACCTTA TACTTTAATA CTTGATACTT TCCGGTTCCC CTGGGTGGGT AGTACACCGT   
  
  
- CATGAGCAAG GGAGGCACTG AGATTATGAT ACATCTTAGT TTCTCTGCCC GGTTTATGGG TGAAGGTTGT   
  
  
- TGTGACTAAA ACAGAGGTTG AACCATTAAT GGTATACACG TTAAGCAGTC CACACTCCAA AATAGTGTTT   
  
  
- TCCCGAGCCA TAATCAATCT CACTTTAATT CTATAAATTT GAGAAGAAAA TAATCTCTAT GCCAACTACA   
  
  
- CCCTACAAAT TGTTCGGGGG AGTACACCCT AGGTTAATCA CCCAGTGTGT ACCCCCTAAG TGTGTAGCTG   
  
  
- TTGGTGTACT TCAGTTCCCC TGAGTGGAAT GTGTGACCGG GTTCACTGGG TGGAAGAAGC TCGCCCCAGT   
  
  
- TCCCCTGGGC CTGGGTGGGT AGTGTACTGT GATGTCATGC TCGGGTGAAG AACCGAAGCT ATGGTACATA   
  
  
- TTATAAGTTT CTCTGACCAG TTTTTAGGTG AAGGTTGTTA TAAATATAAT AGGGGTTGAA CCATTAATAG   
  
  
- TGGATGTGTA AGGTAGTTCA CACCTTAAAA TAGTATTTTC TGTAGTCATA ATCAATCTCA TTCTAATAGT   
  
  
- ATATGATAAT GAAAACAAAA AGATCCTGTT TACACCCTAA GTTGTTATGG TGTATATTAT AATAATTTAT   
  
  
- TAAACCAATT CTTTTCTTAT TGTTCTTTCA GGTAGCATAT TATACATGCC TTGCCATGTG GTGTATAATA   
  
  
- ATATGTTAAC TTTTTTTCAA AATAAAAGTT CACAAGTAAA TTAATATATT ACTATACATC ACAAAATGAA   
  
  
- AACGAGGCCC GTCTAACTTT TTTAAAAGTC TTTAAAAAGT TACAAAGATT TTATCTTTTG CTATGTGGTT   
  
  
- TACAGTAATA CGTTAATTTT ATTGTAAAAT TTATTTTTTG TTTTAAAAGG AGGTGAAAGT ATCATTGTAT   
  
  
- ACTAATAATG CTACACTGAA ATTAAAGCTC GGTTAACTTT TTAGAATAGT ATACAGACTC AGCACTCTAT   
  
  
- AATCTGATTG TGTGTAGTTA GGGGCTAAGG GGCGGCTTAC AACGAGGAAA CGACCGGTAT GATAAGATGC   
  
  
- GAGAAGAGTG CGAAAAACTG AAAAGGCATA AGTTTCAGCA AAAGCAGAGG GAAGAGTGAA GCAAGACGAC   
  
  
- GTACGATGGG AGAAGGAGGA GGAGGAGAGA ATATATATGG GTAGATGGGG AGCTTAAAAT TAGGGTTTTA   
  
  
- TAGAGGTTTG GGTGTAGAGA AAAGAAAGGT GACAAAAAGT GTTGAACTAC TTTATTAACT TAGTTGTTTT   
  
  
- GTAAACCTAT AACACTCTTT TTAGACTCG

+     TATA-box

| Site Name | Organism | Position | Strand | Matrix score. | sequence | function |
| --- | --- | --- | --- | --- | --- | --- |
| TATA-box | Helianthus annuus | 672 | - | 6 | TATAAA | core promoter element around -30 of transcription start |
| TATA-box | Brassica juncea | 671 | - | 7 | TATAAAT | core promoter element around -30 of transcription start |
| TATA-box | Arabidopsis thaliana | 824 | - | 4 | TATA | core promoter element around -30 of transcription start |
| TATA-box | Arabidopsis thaliana | 674 | + | 4 | TATA | core promoter element around -30 of transcription start |
| TATA-box | Arabidopsis thaliana | 673 | - | 5 | TATAA | core promoter element around -30 of transcription start |
| TATA-box | Arabidopsis thaliana | 1365 | - | 4 | TATA | core promoter element around -30 of transcription start |
| TATA-box | Arabidopsis thaliana | 787 | - | 9 | ccTATAAAaa | core promoter element around -30 of transcription start |
| TATA-box | Arabidopsis thaliana | 771 | - | 4 | TATA | core promoter element around -30 of transcription start |
| TATA-box | Arabidopsis thaliana | 877 | - | 4 | TATA | core promoter element around -30 of transcription start |
| TATA-box | Brassica napus | 909 | + | 6 | ATTATA | core promoter element around -30 of transcription start |
| TATA-box | Arabidopsis thaliana | 1363 | - | 6 | TATATA | core promoter element around -30 of transcription start |
| TATA-box | Brassica napus | 1362 | - | 6 | ATATAT | core promoter element around -30 of transcription start |
| TATA-box | Arabidopsis thaliana | 911 | - | 4 | TATA | core promoter element around -30 of transcription start |
| TATA-box | Arabidopsis thaliana | 1361 | - | 6 | TATATA | core promoter element around -30 of transcription start |
| TATA-box | Brassica napus | 952 | + | 6 | ATTATA | core promoter element around -30 of transcription start |
| TATA-box | Arabidopsis thaliana | 1360 | - | 7 | TATATAA | core promoter element around -30 of transcription start |
| TATA-box | Arabidopsis thaliana | 910 | - | 5 | TATAA | core promoter element around -30 of transcription start |
| TATA-box | Arabidopsis thaliana | 953 | - | 7 | TATATAA | core promoter element around -30 of transcription start |
| TATA-box | Arabidopsis thaliana | 1077 | - | 8 | TATTTAAA | core promoter element around -30 of transcription start |
| TATA-box | Brassica oleracea | 955 | + | 6 | ATATAA | core promoter element around -30 of transcription start |
| TATA-box | Arabidopsis thaliana | 956 | - | 4 | TATA | core promoter element around -30 of transcription start |
| TATA-box | Arabidopsis thaliana | 954 | - | 6 | TATATA | core promoter element around -30 of transcription start |
| TATA-box | Arabidopsis thaliana | 628 | + | 4 | TATA | core promoter element around -30 of transcription start |
| TATA-box | Daucus carota | 670 | - | 8 | TATAAATA | core promoter element around -30 of transcription start |
| TATA-box | Helianthus annuus | 626 | - | 6 | TATACA | core promoter element around -30 of transcription start |
| TATA-box | Arabidopsis thaliana | 383 | + | 8 | TATTTAAA | core promoter element around -30 of transcription start |
| TATA-box | Brassica oleracea | 823 | + | 6 | ATATAA | core promoter element around -30 of transcription start |

>018609   
+ CTGTCATCCA CGGCGGTGCG TGGCCAGTGG GCCGATTCTG CCATTTTGTG CAAATTTTGA TATTCTAAAT   
  
  
+ ATGATTTGGT ATCCAATCGA TGTGTTTTGA TTATTAAATC CAATGTTGGT TATTTCGGTT ATTTGCATAT   
  
  
+ TATCGGAAAT GGTTTGGAAT ATGAAATTAT GAACTATGAA AGGCCAAGGG GACCCACCCA TCATGTGGCA   
  
  
+ GTACTCGTTC CCTCCGTGAC TCTAATACTA TGTAGAATCA AAGAGACGGG CCAAATACCC ACTTCCAACA   
  
  
+ ACACTGATTT TGTCTCCAAC TTGGTAATTA CCATATGTGC AATTCGTCAG GTGTGAGGTT TTATCACAAA   
  
  
+ AGGGCTCGGT ATTAGTTAGA GTGAAATTAA GATATTTAAA CTCTTCTTTT ATTAGAGATA CGGTTGATGT   
  
  
+ GGGATGTTTA ACAAGCCCCC TCATGTGGGA TCCAATTAGT GGGTCACACA TGGGGGATTC ACACATCGAC   
  
  
+ AACCACATGA AGTCAAGGGG ACTCACCTTA CACACTGGCC CAAGTGACCC ACCTTCTTCG AGCGGGGTCA   
  
  
+ AGGGGACCCG GACCCACCCA TCACATGACA CTACAGTACG AGCCCACTTC TTGGCTTCGA TACCATGTAT   
  
  
+ AATATTCAAA GAGACTGGTC AAAAATCCAC TTCCAACAAT ATTTATATTA TCCCCAACTT GGTAATTATC   
  
  
+ ACCTACACAT TCCATCAAGT GTGGAATTTT ATCATAAAAG ACATCAGTAT TAGTTAGAGT AAGATTATCA   
  
  
+ TATACTATTA CTTTTGTTTT TCTAGGACAA ATGTGGGATT CAACAATACC ACATATAATA TTATTAAATA   
  
  
+ ATTTGGTTAA GAAAAGAATA ACAAGAAAGT CCATCGTATA ATATGTACGG AACGGTACAC CACATATTAT   
  
  
+ TATACAATTG AAAAAAAGTT TTATTTTCAA GTGTTCATTT AATTATATAA TGATATGTAG TGTTTTACTT   
  
  
+ TTGCTCCGGG CAGATTGAAA AAATTTTCAG AAATTTTTCA ATGTTTCTAA AATAGAAAAC GATACACCAA   
  
  
+ ATGTCATTAT GCAATTAAAA TAACATTTTA AATAAAAAAC AAAATTTTCC TCCACTTTCA TAGTAACATA   
  
  
+ TGATTATTAC GATGTGACTT TAATTTCGAG CCAATTGAAA AATCTTATCA TATGTCTGAG TCGTGAGATA   
  
  
+ TTAGACTAAC ACACATCAAT CCCCGATTCC CCGCCGAATG TTGCTCCTTT GCTGGCCATA CTATTCTACG   
  
  
+ CTCTTCTCAC GCTTTTTGAC TTTTCCGTAT TCAAAGTCGT TTTCGTCTCC CTTCTCACTT CGTTCTGCTG   
  
  
+ CATGCTACCC TCTTCCTCCT CCTCCTCTCT TATATATACC CATCTACCCC TCGAATTTTA ATCCCAAAAT   
  
  
+ ATCTCCAAAC CCACATCTCT TTTCTTTCCA CTGTTTTTCA CAACTTGATG AAATAATTGA ATCAACAAAA   
  
  
+ CATTTGGATA TTGTGAGAAA AATCTGAGC  

- GACAGTAGGT GCCGCCACGC ACCGGTCACC CGGCTAAGAC GGTAAAACAC GTTTAAAACT ATAAGATTTA   
  
  
- TACTAAACCA TAGGTTAGCT ACACAAAACT AATAATTTAG GTTACAACCA ATAAAGCCAA TAAACGTATA   
  
  
- ATAGCCTTTA CCAAACCTTA TACTTTAATA CTTGATACTT TCCGGTTCCC CTGGGTGGGT AGTACACCGT   
  
  
- CATGAGCAAG GGAGGCACTG AGATTATGAT ACATCTTAGT TTCTCTGCCC GGTTTATGGG TGAAGGTTGT   
  
  
- TGTGACTAAA ACAGAGGTTG AACCATTAAT GGTATACACG TTAAGCAGTC CACACTCCAA AATAGTGTTT   
  
  
- TCCCGAGCCA TAATCAATCT CACTTTAATT CTATAAATTT GAGAAGAAAA TAATCTCTAT GCCAACTACA   
  
  
- CCCTACAAAT TGTTCGGGGG AGTACACCCT AGGTTAATCA CCCAGTGTGT ACCCCCTAAG TGTGTAGCTG   
  
  
- TTGGTGTACT TCAGTTCCCC TGAGTGGAAT GTGTGACCGG GTTCACTGGG TGGAAGAAGC TCGCCCCAGT   
  
  
- TCCCCTGGGC CTGGGTGGGT AGTGTACTGT GATGTCATGC TCGGGTGAAG AACCGAAGCT ATGGTACATA   
  
  
- TTATAAGTTT CTCTGACCAG TTTTTAGGTG AAGGTTGTTA TAAATATAAT AGGGGTTGAA CCATTAATAG   
  
  
- TGGATGTGTA AGGTAGTTCA CACCTTAAAA TAGTATTTTC TGTAGTCATA ATCAATCTCA TTCTAATAGT   
  
  
- ATATGATAAT GAAAACAAAA AGATCCTGTT TACACCCTAA GTTGTTATGG TGTATATTAT AATAATTTAT   
  
  
- TAAACCAATT CTTTTCTTAT TGTTCTTTCA GGTAGCATAT TATACATGCC TTGCCATGTG GTGTATAATA   
  
  
- ATATGTTAAC TTTTTTTCAA AATAAAAGTT CACAAGTAAA TTAATATATT ACTATACATC ACAAAATGAA   
  
  
- AACGAGGCCC GTCTAACTTT TTTAAAAGTC TTTAAAAAGT TACAAAGATT TTATCTTTTG CTATGTGGTT   
  
  
- TACAGTAATA CGTTAATTTT ATTGTAAAAT TTATTTTTTG TTTTAAAAGG AGGTGAAAGT ATCATTGTAT   
  
  
- ACTAATAATG CTACACTGAA ATTAAAGCTC GGTTAACTTT TTAGAATAGT ATACAGACTC AGCACTCTAT   
  
  
- AATCTGATTG TGTGTAGTTA GGGGCTAAGG GGCGGCTTAC AACGAGGAAA CGACCGGTAT GATAAGATGC   
  
  
- GAGAAGAGTG CGAAAAACTG AAAAGGCATA AGTTTCAGCA AAAGCAGAGG GAAGAGTGAA GCAAGACGAC   
  
  
- GTACGATGGG AGAAGGAGGA GGAGGAGAGA ATATATATGG GTAGATGGGG AGCTTAAAAT TAGGGTTTTA   
  
  
- TAGAGGTTTG GGTGTAGAGA AAAGAAAGGT GACAAAAAGT GTTGAACTAC TTTATTAACT TAGTTGTTTT   
  
  
- GTAAACCTAT AACACTCTTT TTAGACTCG

+     TCCC-motif

| Site Name | Organism | Position | Strand | Matrix score. | sequence | function |
| --- | --- | --- | --- | --- | --- | --- |
| TCCC-motif | Spinacia oleracea | 1306 | + | 7 | TCTCCCT | part of a light responsive element |

>018609   
+ CTGTCATCCA CGGCGGTGCG TGGCCAGTGG GCCGATTCTG CCATTTTGTG CAAATTTTGA TATTCTAAAT   
  
  
+ ATGATTTGGT ATCCAATCGA TGTGTTTTGA TTATTAAATC CAATGTTGGT TATTTCGGTT ATTTGCATAT   
  
  
+ TATCGGAAAT GGTTTGGAAT ATGAAATTAT GAACTATGAA AGGCCAAGGG GACCCACCCA TCATGTGGCA   
  
  
+ GTACTCGTTC CCTCCGTGAC TCTAATACTA TGTAGAATCA AAGAGACGGG CCAAATACCC ACTTCCAACA   
  
  
+ ACACTGATTT TGTCTCCAAC TTGGTAATTA CCATATGTGC AATTCGTCAG GTGTGAGGTT TTATCACAAA   
  
  
+ AGGGCTCGGT ATTAGTTAGA GTGAAATTAA GATATTTAAA CTCTTCTTTT ATTAGAGATA CGGTTGATGT   
  
  
+ GGGATGTTTA ACAAGCCCCC TCATGTGGGA TCCAATTAGT GGGTCACACA TGGGGGATTC ACACATCGAC   
  
  
+ AACCACATGA AGTCAAGGGG ACTCACCTTA CACACTGGCC CAAGTGACCC ACCTTCTTCG AGCGGGGTCA   
  
  
+ AGGGGACCCG GACCCACCCA TCACATGACA CTACAGTACG AGCCCACTTC TTGGCTTCGA TACCATGTAT   
  
  
+ AATATTCAAA GAGACTGGTC AAAAATCCAC TTCCAACAAT ATTTATATTA TCCCCAACTT GGTAATTATC   
  
  
+ ACCTACACAT TCCATCAAGT GTGGAATTTT ATCATAAAAG ACATCAGTAT TAGTTAGAGT AAGATTATCA   
  
  
+ TATACTATTA CTTTTGTTTT TCTAGGACAA ATGTGGGATT CAACAATACC ACATATAATA TTATTAAATA   
  
  
+ ATTTGGTTAA GAAAAGAATA ACAAGAAAGT CCATCGTATA ATATGTACGG AACGGTACAC CACATATTAT   
  
  
+ TATACAATTG AAAAAAAGTT TTATTTTCAA GTGTTCATTT AATTATATAA TGATATGTAG TGTTTTACTT   
  
  
+ TTGCTCCGGG CAGATTGAAA AAATTTTCAG AAATTTTTCA ATGTTTCTAA AATAGAAAAC GATACACCAA   
  
  
+ ATGTCATTAT GCAATTAAAA TAACATTTTA AATAAAAAAC AAAATTTTCC TCCACTTTCA TAGTAACATA   
  
  
+ TGATTATTAC GATGTGACTT TAATTTCGAG CCAATTGAAA AATCTTATCA TATGTCTGAG TCGTGAGATA   
  
  
+ TTAGACTAAC ACACATCAAT CCCCGATTCC CCGCCGAATG TTGCTCCTTT GCTGGCCATA CTATTCTACG   
  
  
+ CTCTTCTCAC GCTTTTTGAC TTTTCCGTAT TCAAAGTCGT TTTCGTCTCC CTTCTCACTT CGTTCTGCTG   
  
  
+ CATGCTACCC TCTTCCTCCT CCTCCTCTCT TATATATACC CATCTACCCC TCGAATTTTA ATCCCAAAAT   
  
  
+ ATCTCCAAAC CCACATCTCT TTTCTTTCCA CTGTTTTTCA CAACTTGATG AAATAATTGA ATCAACAAAA   
  
  
+ CATTTGGATA TTGTGAGAAA AATCTGAGC  

- GACAGTAGGT GCCGCCACGC ACCGGTCACC CGGCTAAGAC GGTAAAACAC GTTTAAAACT ATAAGATTTA   
  
  
- TACTAAACCA TAGGTTAGCT ACACAAAACT AATAATTTAG GTTACAACCA ATAAAGCCAA TAAACGTATA   
  
  
- ATAGCCTTTA CCAAACCTTA TACTTTAATA CTTGATACTT TCCGGTTCCC CTGGGTGGGT AGTACACCGT   
  
  
- CATGAGCAAG GGAGGCACTG AGATTATGAT ACATCTTAGT TTCTCTGCCC GGTTTATGGG TGAAGGTTGT   
  
  
- TGTGACTAAA ACAGAGGTTG AACCATTAAT GGTATACACG TTAAGCAGTC CACACTCCAA AATAGTGTTT   
  
  
- TCCCGAGCCA TAATCAATCT CACTTTAATT CTATAAATTT GAGAAGAAAA TAATCTCTAT GCCAACTACA   
  
  
- CCCTACAAAT TGTTCGGGGG AGTACACCCT AGGTTAATCA CCCAGTGTGT ACCCCCTAAG TGTGTAGCTG   
  
  
- TTGGTGTACT TCAGTTCCCC TGAGTGGAAT GTGTGACCGG GTTCACTGGG TGGAAGAAGC TCGCCCCAGT   
  
  
- TCCCCTGGGC CTGGGTGGGT AGTGTACTGT GATGTCATGC TCGGGTGAAG AACCGAAGCT ATGGTACATA   
  
  
- TTATAAGTTT CTCTGACCAG TTTTTAGGTG AAGGTTGTTA TAAATATAAT AGGGGTTGAA CCATTAATAG   
  
  
- TGGATGTGTA AGGTAGTTCA CACCTTAAAA TAGTATTTTC TGTAGTCATA ATCAATCTCA TTCTAATAGT   
  
  
- ATATGATAAT GAAAACAAAA AGATCCTGTT TACACCCTAA GTTGTTATGG TGTATATTAT AATAATTTAT   
  
  
- TAAACCAATT CTTTTCTTAT TGTTCTTTCA GGTAGCATAT TATACATGCC TTGCCATGTG GTGTATAATA   
  
  
- ATATGTTAAC TTTTTTTCAA AATAAAAGTT CACAAGTAAA TTAATATATT ACTATACATC ACAAAATGAA   
  
  
- AACGAGGCCC GTCTAACTTT TTTAAAAGTC TTTAAAAAGT TACAAAGATT TTATCTTTTG CTATGTGGTT   
  
  
- TACAGTAATA CGTTAATTTT ATTGTAAAAT TTATTTTTTG TTTTAAAAGG AGGTGAAAGT ATCATTGTAT   
  
  
- ACTAATAATG CTACACTGAA ATTAAAGCTC GGTTAACTTT TTAGAATAGT ATACAGACTC AGCACTCTAT   
  
  
- AATCTGATTG TGTGTAGTTA GGGGCTAAGG GGCGGCTTAC AACGAGGAAA CGACCGGTAT GATAAGATGC   
  
  
- GAGAAGAGTG CGAAAAACTG AAAAGGCATA AGTTTCAGCA AAAGCAGAGG GAAGAGTGAA GCAAGACGAC   
  
  
- GTACGATGGG AGAAGGAGGA GGAGGAGAGA ATATATATGG GTAGATGGGG AGCTTAAAAT TAGGGTTTTA   
  
  
- TAGAGGTTTG GGTGTAGAGA AAAGAAAGGT GACAAAAAGT GTTGAACTAC TTTATTAACT TAGTTGTTTT   
  
  
- GTAAACCTAT AACACTCTTT TTAGACTCG

+     TCT-motif

| Site Name | Organism | Position | Strand | Matrix score. | sequence | function |
| --- | --- | --- | --- | --- | --- | --- |
| TCT-motif | Arabidopsis thaliana | 759 | - | 6 | TCTTAC | part of a light responsive element |

>018609   
+ CTGTCATCCA CGGCGGTGCG TGGCCAGTGG GCCGATTCTG CCATTTTGTG CAAATTTTGA TATTCTAAAT   
  
  
+ ATGATTTGGT ATCCAATCGA TGTGTTTTGA TTATTAAATC CAATGTTGGT TATTTCGGTT ATTTGCATAT   
  
  
+ TATCGGAAAT GGTTTGGAAT ATGAAATTAT GAACTATGAA AGGCCAAGGG GACCCACCCA TCATGTGGCA   
  
  
+ GTACTCGTTC CCTCCGTGAC TCTAATACTA TGTAGAATCA AAGAGACGGG CCAAATACCC ACTTCCAACA   
  
  
+ ACACTGATTT TGTCTCCAAC TTGGTAATTA CCATATGTGC AATTCGTCAG GTGTGAGGTT TTATCACAAA   
  
  
+ AGGGCTCGGT ATTAGTTAGA GTGAAATTAA GATATTTAAA CTCTTCTTTT ATTAGAGATA CGGTTGATGT   
  
  
+ GGGATGTTTA ACAAGCCCCC TCATGTGGGA TCCAATTAGT GGGTCACACA TGGGGGATTC ACACATCGAC   
  
  
+ AACCACATGA AGTCAAGGGG ACTCACCTTA CACACTGGCC CAAGTGACCC ACCTTCTTCG AGCGGGGTCA   
  
  
+ AGGGGACCCG GACCCACCCA TCACATGACA CTACAGTACG AGCCCACTTC TTGGCTTCGA TACCATGTAT   
  
  
+ AATATTCAAA GAGACTGGTC AAAAATCCAC TTCCAACAAT ATTTATATTA TCCCCAACTT GGTAATTATC   
  
  
+ ACCTACACAT TCCATCAAGT GTGGAATTTT ATCATAAAAG ACATCAGTAT TAGTTAGAGT AAGATTATCA   
  
  
+ TATACTATTA CTTTTGTTTT TCTAGGACAA ATGTGGGATT CAACAATACC ACATATAATA TTATTAAATA   
  
  
+ ATTTGGTTAA GAAAAGAATA ACAAGAAAGT CCATCGTATA ATATGTACGG AACGGTACAC CACATATTAT   
  
  
+ TATACAATTG AAAAAAAGTT TTATTTTCAA GTGTTCATTT AATTATATAA TGATATGTAG TGTTTTACTT   
  
  
+ TTGCTCCGGG CAGATTGAAA AAATTTTCAG AAATTTTTCA ATGTTTCTAA AATAGAAAAC GATACACCAA   
  
  
+ ATGTCATTAT GCAATTAAAA TAACATTTTA AATAAAAAAC AAAATTTTCC TCCACTTTCA TAGTAACATA   
  
  
+ TGATTATTAC GATGTGACTT TAATTTCGAG CCAATTGAAA AATCTTATCA TATGTCTGAG TCGTGAGATA   
  
  
+ TTAGACTAAC ACACATCAAT CCCCGATTCC CCGCCGAATG TTGCTCCTTT GCTGGCCATA CTATTCTACG   
  
  
+ CTCTTCTCAC GCTTTTTGAC TTTTCCGTAT TCAAAGTCGT TTTCGTCTCC CTTCTCACTT CGTTCTGCTG   
  
  
+ CATGCTACCC TCTTCCTCCT CCTCCTCTCT TATATATACC CATCTACCCC TCGAATTTTA ATCCCAAAAT   
  
  
+ ATCTCCAAAC CCACATCTCT TTTCTTTCCA CTGTTTTTCA CAACTTGATG AAATAATTGA ATCAACAAAA   
  
  
+ CATTTGGATA TTGTGAGAAA AATCTGAGC  

- GACAGTAGGT GCCGCCACGC ACCGGTCACC CGGCTAAGAC GGTAAAACAC GTTTAAAACT ATAAGATTTA   
  
  
- TACTAAACCA TAGGTTAGCT ACACAAAACT AATAATTTAG GTTACAACCA ATAAAGCCAA TAAACGTATA   
  
  
- ATAGCCTTTA CCAAACCTTA TACTTTAATA CTTGATACTT TCCGGTTCCC CTGGGTGGGT AGTACACCGT   
  
  
- CATGAGCAAG GGAGGCACTG AGATTATGAT ACATCTTAGT TTCTCTGCCC GGTTTATGGG TGAAGGTTGT   
  
  
- TGTGACTAAA ACAGAGGTTG AACCATTAAT GGTATACACG TTAAGCAGTC CACACTCCAA AATAGTGTTT   
  
  
- TCCCGAGCCA TAATCAATCT CACTTTAATT CTATAAATTT GAGAAGAAAA TAATCTCTAT GCCAACTACA   
  
  
- CCCTACAAAT TGTTCGGGGG AGTACACCCT AGGTTAATCA CCCAGTGTGT ACCCCCTAAG TGTGTAGCTG   
  
  
- TTGGTGTACT TCAGTTCCCC TGAGTGGAAT GTGTGACCGG GTTCACTGGG TGGAAGAAGC TCGCCCCAGT   
  
  
- TCCCCTGGGC CTGGGTGGGT AGTGTACTGT GATGTCATGC TCGGGTGAAG AACCGAAGCT ATGGTACATA   
  
  
- TTATAAGTTT CTCTGACCAG TTTTTAGGTG AAGGTTGTTA TAAATATAAT AGGGGTTGAA CCATTAATAG   
  
  
- TGGATGTGTA AGGTAGTTCA CACCTTAAAA TAGTATTTTC TGTAGTCATA ATCAATCTCA TTCTAATAGT   
  
  
- ATATGATAAT GAAAACAAAA AGATCCTGTT TACACCCTAA GTTGTTATGG TGTATATTAT AATAATTTAT   
  
  
- TAAACCAATT CTTTTCTTAT TGTTCTTTCA GGTAGCATAT TATACATGCC TTGCCATGTG GTGTATAATA   
  
  
- ATATGTTAAC TTTTTTTCAA AATAAAAGTT CACAAGTAAA TTAATATATT ACTATACATC ACAAAATGAA   
  
  
- AACGAGGCCC GTCTAACTTT TTTAAAAGTC TTTAAAAAGT TACAAAGATT TTATCTTTTG CTATGTGGTT   
  
  
- TACAGTAATA CGTTAATTTT ATTGTAAAAT TTATTTTTTG TTTTAAAAGG AGGTGAAAGT ATCATTGTAT   
  
  
- ACTAATAATG CTACACTGAA ATTAAAGCTC GGTTAACTTT TTAGAATAGT ATACAGACTC AGCACTCTAT   
  
  
- AATCTGATTG TGTGTAGTTA GGGGCTAAGG GGCGGCTTAC AACGAGGAAA CGACCGGTAT GATAAGATGC   
  
  
- GAGAAGAGTG CGAAAAACTG AAAAGGCATA AGTTTCAGCA AAAGCAGAGG GAAGAGTGAA GCAAGACGAC   
  
  
- GTACGATGGG AGAAGGAGGA GGAGGAGAGA ATATATATGG GTAGATGGGG AGCTTAAAAT TAGGGTTTTA   
  
  
- TAGAGGTTTG GGTGTAGAGA AAAGAAAGGT GACAAAAAGT GTTGAACTAC TTTATTAACT TAGTTGTTTT   
  
  
- GTAAACCTAT AACACTCTTT TTAGACTCG

+     TGA-element

| Site Name | Organism | Position | Strand | Matrix score. | sequence | function |
| --- | --- | --- | --- | --- | --- | --- |
| TGA-element | Brassica oleracea | 1296 | - | 6 | AACGAC | auxin-responsive element |

>018609   
+ CTGTCATCCA CGGCGGTGCG TGGCCAGTGG GCCGATTCTG CCATTTTGTG CAAATTTTGA TATTCTAAAT   
  
  
+ ATGATTTGGT ATCCAATCGA TGTGTTTTGA TTATTAAATC CAATGTTGGT TATTTCGGTT ATTTGCATAT   
  
  
+ TATCGGAAAT GGTTTGGAAT ATGAAATTAT GAACTATGAA AGGCCAAGGG GACCCACCCA TCATGTGGCA   
  
  
+ GTACTCGTTC CCTCCGTGAC TCTAATACTA TGTAGAATCA AAGAGACGGG CCAAATACCC ACTTCCAACA   
  
  
+ ACACTGATTT TGTCTCCAAC TTGGTAATTA CCATATGTGC AATTCGTCAG GTGTGAGGTT TTATCACAAA   
  
  
+ AGGGCTCGGT ATTAGTTAGA GTGAAATTAA GATATTTAAA CTCTTCTTTT ATTAGAGATA CGGTTGATGT   
  
  
+ GGGATGTTTA ACAAGCCCCC TCATGTGGGA TCCAATTAGT GGGTCACACA TGGGGGATTC ACACATCGAC   
  
  
+ AACCACATGA AGTCAAGGGG ACTCACCTTA CACACTGGCC CAAGTGACCC ACCTTCTTCG AGCGGGGTCA   
  
  
+ AGGGGACCCG GACCCACCCA TCACATGACA CTACAGTACG AGCCCACTTC TTGGCTTCGA TACCATGTAT   
  
  
+ AATATTCAAA GAGACTGGTC AAAAATCCAC TTCCAACAAT ATTTATATTA TCCCCAACTT GGTAATTATC   
  
  
+ ACCTACACAT TCCATCAAGT GTGGAATTTT ATCATAAAAG ACATCAGTAT TAGTTAGAGT AAGATTATCA   
  
  
+ TATACTATTA CTTTTGTTTT TCTAGGACAA ATGTGGGATT CAACAATACC ACATATAATA TTATTAAATA   
  
  
+ ATTTGGTTAA GAAAAGAATA ACAAGAAAGT CCATCGTATA ATATGTACGG AACGGTACAC CACATATTAT   
  
  
+ TATACAATTG AAAAAAAGTT TTATTTTCAA GTGTTCATTT AATTATATAA TGATATGTAG TGTTTTACTT   
  
  
+ TTGCTCCGGG CAGATTGAAA AAATTTTCAG AAATTTTTCA ATGTTTCTAA AATAGAAAAC GATACACCAA   
  
  
+ ATGTCATTAT GCAATTAAAA TAACATTTTA AATAAAAAAC AAAATTTTCC TCCACTTTCA TAGTAACATA   
  
  
+ TGATTATTAC GATGTGACTT TAATTTCGAG CCAATTGAAA AATCTTATCA TATGTCTGAG TCGTGAGATA   
  
  
+ TTAGACTAAC ACACATCAAT CCCCGATTCC CCGCCGAATG TTGCTCCTTT GCTGGCCATA CTATTCTACG   
  
  
+ CTCTTCTCAC GCTTTTTGAC TTTTCCGTAT TCAAAGTCGT TTTCGTCTCC CTTCTCACTT CGTTCTGCTG   
  
  
+ CATGCTACCC TCTTCCTCCT CCTCCTCTCT TATATATACC CATCTACCCC TCGAATTTTA ATCCCAAAAT   
  
  
+ ATCTCCAAAC CCACATCTCT TTTCTTTCCA CTGTTTTTCA CAACTTGATG AAATAATTGA ATCAACAAAA   
  
  
+ CATTTGGATA TTGTGAGAAA AATCTGAGC  

- GACAGTAGGT GCCGCCACGC ACCGGTCACC CGGCTAAGAC GGTAAAACAC GTTTAAAACT ATAAGATTTA   
  
  
- TACTAAACCA TAGGTTAGCT ACACAAAACT AATAATTTAG GTTACAACCA ATAAAGCCAA TAAACGTATA   
  
  
- ATAGCCTTTA CCAAACCTTA TACTTTAATA CTTGATACTT TCCGGTTCCC CTGGGTGGGT AGTACACCGT   
  
  
- CATGAGCAAG GGAGGCACTG AGATTATGAT ACATCTTAGT TTCTCTGCCC GGTTTATGGG TGAAGGTTGT   
  
  
- TGTGACTAAA ACAGAGGTTG AACCATTAAT GGTATACACG TTAAGCAGTC CACACTCCAA AATAGTGTTT   
  
  
- TCCCGAGCCA TAATCAATCT CACTTTAATT CTATAAATTT GAGAAGAAAA TAATCTCTAT GCCAACTACA   
  
  
- CCCTACAAAT TGTTCGGGGG AGTACACCCT AGGTTAATCA CCCAGTGTGT ACCCCCTAAG TGTGTAGCTG   
  
  
- TTGGTGTACT TCAGTTCCCC TGAGTGGAAT GTGTGACCGG GTTCACTGGG TGGAAGAAGC TCGCCCCAGT   
  
  
- TCCCCTGGGC CTGGGTGGGT AGTGTACTGT GATGTCATGC TCGGGTGAAG AACCGAAGCT ATGGTACATA   
  
  
- TTATAAGTTT CTCTGACCAG TTTTTAGGTG AAGGTTGTTA TAAATATAAT AGGGGTTGAA CCATTAATAG   
  
  
- TGGATGTGTA AGGTAGTTCA CACCTTAAAA TAGTATTTTC TGTAGTCATA ATCAATCTCA TTCTAATAGT   
  
  
- ATATGATAAT GAAAACAAAA AGATCCTGTT TACACCCTAA GTTGTTATGG TGTATATTAT AATAATTTAT   
  
  
- TAAACCAATT CTTTTCTTAT TGTTCTTTCA GGTAGCATAT TATACATGCC TTGCCATGTG GTGTATAATA   
  
  
- ATATGTTAAC TTTTTTTCAA AATAAAAGTT CACAAGTAAA TTAATATATT ACTATACATC ACAAAATGAA   
  
  
- AACGAGGCCC GTCTAACTTT TTTAAAAGTC TTTAAAAAGT TACAAAGATT TTATCTTTTG CTATGTGGTT   
  
  
- TACAGTAATA CGTTAATTTT ATTGTAAAAT TTATTTTTTG TTTTAAAAGG AGGTGAAAGT ATCATTGTAT   
  
  
- ACTAATAATG CTACACTGAA ATTAAAGCTC GGTTAACTTT TTAGAATAGT ATACAGACTC AGCACTCTAT   
  
  
- AATCTGATTG TGTGTAGTTA GGGGCTAAGG GGCGGCTTAC AACGAGGAAA CGACCGGTAT GATAAGATGC   
  
  
- GAGAAGAGTG CGAAAAACTG AAAAGGCATA AGTTTCAGCA AAAGCAGAGG GAAGAGTGAA GCAAGACGAC   
  
  
- GTACGATGGG AGAAGGAGGA GGAGGAGAGA ATATATATGG GTAGATGGGG AGCTTAAAAT TAGGGTTTTA   
  
  
- TAGAGGTTTG GGTGTAGAGA AAAGAAAGGT GACAAAAAGT GTTGAACTAC TTTATTAACT TAGTTGTTTT   
  
  
- GTAAACCTAT AACACTCTTT TTAGACTCG

+     TGACG-motif

| Site Name | Organism | Position | Strand | Matrix score. | sequence | function |
| --- | --- | --- | --- | --- | --- | --- |
| TGACG-motif | Hordeum vulgare | 325 | - | 5 | TGACG | cis-acting regulatory element involved in the MeJA-responsiveness |

>018609   
+ CTGTCATCCA CGGCGGTGCG TGGCCAGTGG GCCGATTCTG CCATTTTGTG CAAATTTTGA TATTCTAAAT   
  
  
+ ATGATTTGGT ATCCAATCGA TGTGTTTTGA TTATTAAATC CAATGTTGGT TATTTCGGTT ATTTGCATAT   
  
  
+ TATCGGAAAT GGTTTGGAAT ATGAAATTAT GAACTATGAA AGGCCAAGGG GACCCACCCA TCATGTGGCA   
  
  
+ GTACTCGTTC CCTCCGTGAC TCTAATACTA TGTAGAATCA AAGAGACGGG CCAAATACCC ACTTCCAACA   
  
  
+ ACACTGATTT TGTCTCCAAC TTGGTAATTA CCATATGTGC AATTCGTCAG GTGTGAGGTT TTATCACAAA   
  
  
+ AGGGCTCGGT ATTAGTTAGA GTGAAATTAA GATATTTAAA CTCTTCTTTT ATTAGAGATA CGGTTGATGT   
  
  
+ GGGATGTTTA ACAAGCCCCC TCATGTGGGA TCCAATTAGT GGGTCACACA TGGGGGATTC ACACATCGAC   
  
  
+ AACCACATGA AGTCAAGGGG ACTCACCTTA CACACTGGCC CAAGTGACCC ACCTTCTTCG AGCGGGGTCA   
  
  
+ AGGGGACCCG GACCCACCCA TCACATGACA CTACAGTACG AGCCCACTTC TTGGCTTCGA TACCATGTAT   
  
  
+ AATATTCAAA GAGACTGGTC AAAAATCCAC TTCCAACAAT ATTTATATTA TCCCCAACTT GGTAATTATC   
  
  
+ ACCTACACAT TCCATCAAGT GTGGAATTTT ATCATAAAAG ACATCAGTAT TAGTTAGAGT AAGATTATCA   
  
  
+ TATACTATTA CTTTTGTTTT TCTAGGACAA ATGTGGGATT CAACAATACC ACATATAATA TTATTAAATA   
  
  
+ ATTTGGTTAA GAAAAGAATA ACAAGAAAGT CCATCGTATA ATATGTACGG AACGGTACAC CACATATTAT   
  
  
+ TATACAATTG AAAAAAAGTT TTATTTTCAA GTGTTCATTT AATTATATAA TGATATGTAG TGTTTTACTT   
  
  
+ TTGCTCCGGG CAGATTGAAA AAATTTTCAG AAATTTTTCA ATGTTTCTAA AATAGAAAAC GATACACCAA   
  
  
+ ATGTCATTAT GCAATTAAAA TAACATTTTA AATAAAAAAC AAAATTTTCC TCCACTTTCA TAGTAACATA   
  
  
+ TGATTATTAC GATGTGACTT TAATTTCGAG CCAATTGAAA AATCTTATCA TATGTCTGAG TCGTGAGATA   
  
  
+ TTAGACTAAC ACACATCAAT CCCCGATTCC CCGCCGAATG TTGCTCCTTT GCTGGCCATA CTATTCTACG   
  
  
+ CTCTTCTCAC GCTTTTTGAC TTTTCCGTAT TCAAAGTCGT TTTCGTCTCC CTTCTCACTT CGTTCTGCTG   
  
  
+ CATGCTACCC TCTTCCTCCT CCTCCTCTCT TATATATACC CATCTACCCC TCGAATTTTA ATCCCAAAAT   
  
  
+ ATCTCCAAAC CCACATCTCT TTTCTTTCCA CTGTTTTTCA CAACTTGATG AAATAATTGA ATCAACAAAA   
  
  
+ CATTTGGATA TTGTGAGAAA AATCTGAGC  

- GACAGTAGGT GCCGCCACGC ACCGGTCACC CGGCTAAGAC GGTAAAACAC GTTTAAAACT ATAAGATTTA   
  
  
- TACTAAACCA TAGGTTAGCT ACACAAAACT AATAATTTAG GTTACAACCA ATAAAGCCAA TAAACGTATA   
  
  
- ATAGCCTTTA CCAAACCTTA TACTTTAATA CTTGATACTT TCCGGTTCCC CTGGGTGGGT AGTACACCGT   
  
  
- CATGAGCAAG GGAGGCACTG AGATTATGAT ACATCTTAGT TTCTCTGCCC GGTTTATGGG TGAAGGTTGT   
  
  
- TGTGACTAAA ACAGAGGTTG AACCATTAAT GGTATACACG TTAAGCAGTC CACACTCCAA AATAGTGTTT   
  
  
- TCCCGAGCCA TAATCAATCT CACTTTAATT CTATAAATTT GAGAAGAAAA TAATCTCTAT GCCAACTACA   
  
  
- CCCTACAAAT TGTTCGGGGG AGTACACCCT AGGTTAATCA CCCAGTGTGT ACCCCCTAAG TGTGTAGCTG   
  
  
- TTGGTGTACT TCAGTTCCCC TGAGTGGAAT GTGTGACCGG GTTCACTGGG TGGAAGAAGC TCGCCCCAGT   
  
  
- TCCCCTGGGC CTGGGTGGGT AGTGTACTGT GATGTCATGC TCGGGTGAAG AACCGAAGCT ATGGTACATA   
  
  
- TTATAAGTTT CTCTGACCAG TTTTTAGGTG AAGGTTGTTA TAAATATAAT AGGGGTTGAA CCATTAATAG   
  
  
- TGGATGTGTA AGGTAGTTCA CACCTTAAAA TAGTATTTTC TGTAGTCATA ATCAATCTCA TTCTAATAGT   
  
  
- ATATGATAAT GAAAACAAAA AGATCCTGTT TACACCCTAA GTTGTTATGG TGTATATTAT AATAATTTAT   
  
  
- TAAACCAATT CTTTTCTTAT TGTTCTTTCA GGTAGCATAT TATACATGCC TTGCCATGTG GTGTATAATA   
  
  
- ATATGTTAAC TTTTTTTCAA AATAAAAGTT CACAAGTAAA TTAATATATT ACTATACATC ACAAAATGAA   
  
  
- AACGAGGCCC GTCTAACTTT TTTAAAAGTC TTTAAAAAGT TACAAAGATT TTATCTTTTG CTATGTGGTT   
  
  
- TACAGTAATA CGTTAATTTT ATTGTAAAAT TTATTTTTTG TTTTAAAAGG AGGTGAAAGT ATCATTGTAT   
  
  
- ACTAATAATG CTACACTGAA ATTAAAGCTC GGTTAACTTT TTAGAATAGT ATACAGACTC AGCACTCTAT   
  
  
- AATCTGATTG TGTGTAGTTA GGGGCTAAGG GGCGGCTTAC AACGAGGAAA CGACCGGTAT GATAAGATGC   
  
  
- GAGAAGAGTG CGAAAAACTG AAAAGGCATA AGTTTCAGCA AAAGCAGAGG GAAGAGTGAA GCAAGACGAC   
  
  
- GTACGATGGG AGAAGGAGGA GGAGGAGAGA ATATATATGG GTAGATGGGG AGCTTAAAAT TAGGGTTTTA   
  
  
- TAGAGGTTTG GGTGTAGAGA AAAGAAAGGT GACAAAAAGT GTTGAACTAC TTTATTAACT TAGTTGTTTT   
  
  
- GTAAACCTAT AACACTCTTT TTAGACTCG

+     Unnamed\_\_1

| Site Name | Organism | Position | Strand | Matrix score. | sequence | function |
| --- | --- | --- | --- | --- | --- | --- |
| Unnamed\_\_1 | Zea mays | 8 | - | 5 | CGTGG |  |
| Unnamed\_\_1 | Zea mays | 19 | + | 5 | CGTGG |  |

>018609   
+ CTGTCATCCA CGGCGGTGCG TGGCCAGTGG GCCGATTCTG CCATTTTGTG CAAATTTTGA TATTCTAAAT   
  
  
+ ATGATTTGGT ATCCAATCGA TGTGTTTTGA TTATTAAATC CAATGTTGGT TATTTCGGTT ATTTGCATAT   
  
  
+ TATCGGAAAT GGTTTGGAAT ATGAAATTAT GAACTATGAA AGGCCAAGGG GACCCACCCA TCATGTGGCA   
  
  
+ GTACTCGTTC CCTCCGTGAC TCTAATACTA TGTAGAATCA AAGAGACGGG CCAAATACCC ACTTCCAACA   
  
  
+ ACACTGATTT TGTCTCCAAC TTGGTAATTA CCATATGTGC AATTCGTCAG GTGTGAGGTT TTATCACAAA   
  
  
+ AGGGCTCGGT ATTAGTTAGA GTGAAATTAA GATATTTAAA CTCTTCTTTT ATTAGAGATA CGGTTGATGT   
  
  
+ GGGATGTTTA ACAAGCCCCC TCATGTGGGA TCCAATTAGT GGGTCACACA TGGGGGATTC ACACATCGAC   
  
  
+ AACCACATGA AGTCAAGGGG ACTCACCTTA CACACTGGCC CAAGTGACCC ACCTTCTTCG AGCGGGGTCA   
  
  
+ AGGGGACCCG GACCCACCCA TCACATGACA CTACAGTACG AGCCCACTTC TTGGCTTCGA TACCATGTAT   
  
  
+ AATATTCAAA GAGACTGGTC AAAAATCCAC TTCCAACAAT ATTTATATTA TCCCCAACTT GGTAATTATC   
  
  
+ ACCTACACAT TCCATCAAGT GTGGAATTTT ATCATAAAAG ACATCAGTAT TAGTTAGAGT AAGATTATCA   
  
  
+ TATACTATTA CTTTTGTTTT TCTAGGACAA ATGTGGGATT CAACAATACC ACATATAATA TTATTAAATA   
  
  
+ ATTTGGTTAA GAAAAGAATA ACAAGAAAGT CCATCGTATA ATATGTACGG AACGGTACAC CACATATTAT   
  
  
+ TATACAATTG AAAAAAAGTT TTATTTTCAA GTGTTCATTT AATTATATAA TGATATGTAG TGTTTTACTT   
  
  
+ TTGCTCCGGG CAGATTGAAA AAATTTTCAG AAATTTTTCA ATGTTTCTAA AATAGAAAAC GATACACCAA   
  
  
+ ATGTCATTAT GCAATTAAAA TAACATTTTA AATAAAAAAC AAAATTTTCC TCCACTTTCA TAGTAACATA   
  
  
+ TGATTATTAC GATGTGACTT TAATTTCGAG CCAATTGAAA AATCTTATCA TATGTCTGAG TCGTGAGATA   
  
  
+ TTAGACTAAC ACACATCAAT CCCCGATTCC CCGCCGAATG TTGCTCCTTT GCTGGCCATA CTATTCTACG   
  
  
+ CTCTTCTCAC GCTTTTTGAC TTTTCCGTAT TCAAAGTCGT TTTCGTCTCC CTTCTCACTT CGTTCTGCTG   
  
  
+ CATGCTACCC TCTTCCTCCT CCTCCTCTCT TATATATACC CATCTACCCC TCGAATTTTA ATCCCAAAAT   
  
  
+ ATCTCCAAAC CCACATCTCT TTTCTTTCCA CTGTTTTTCA CAACTTGATG AAATAATTGA ATCAACAAAA   
  
  
+ CATTTGGATA TTGTGAGAAA AATCTGAGC  

- GACAGTAGGT GCCGCCACGC ACCGGTCACC CGGCTAAGAC GGTAAAACAC GTTTAAAACT ATAAGATTTA   
  
  
- TACTAAACCA TAGGTTAGCT ACACAAAACT AATAATTTAG GTTACAACCA ATAAAGCCAA TAAACGTATA   
  
  
- ATAGCCTTTA CCAAACCTTA TACTTTAATA CTTGATACTT TCCGGTTCCC CTGGGTGGGT AGTACACCGT   
  
  
- CATGAGCAAG GGAGGCACTG AGATTATGAT ACATCTTAGT TTCTCTGCCC GGTTTATGGG TGAAGGTTGT   
  
  
- TGTGACTAAA ACAGAGGTTG AACCATTAAT GGTATACACG TTAAGCAGTC CACACTCCAA AATAGTGTTT   
  
  
- TCCCGAGCCA TAATCAATCT CACTTTAATT CTATAAATTT GAGAAGAAAA TAATCTCTAT GCCAACTACA   
  
  
- CCCTACAAAT TGTTCGGGGG AGTACACCCT AGGTTAATCA CCCAGTGTGT ACCCCCTAAG TGTGTAGCTG   
  
  
- TTGGTGTACT TCAGTTCCCC TGAGTGGAAT GTGTGACCGG GTTCACTGGG TGGAAGAAGC TCGCCCCAGT   
  
  
- TCCCCTGGGC CTGGGTGGGT AGTGTACTGT GATGTCATGC TCGGGTGAAG AACCGAAGCT ATGGTACATA   
  
  
- TTATAAGTTT CTCTGACCAG TTTTTAGGTG AAGGTTGTTA TAAATATAAT AGGGGTTGAA CCATTAATAG   
  
  
- TGGATGTGTA AGGTAGTTCA CACCTTAAAA TAGTATTTTC TGTAGTCATA ATCAATCTCA TTCTAATAGT   
  
  
- ATATGATAAT GAAAACAAAA AGATCCTGTT TACACCCTAA GTTGTTATGG TGTATATTAT AATAATTTAT   
  
  
- TAAACCAATT CTTTTCTTAT TGTTCTTTCA GGTAGCATAT TATACATGCC TTGCCATGTG GTGTATAATA   
  
  
- ATATGTTAAC TTTTTTTCAA AATAAAAGTT CACAAGTAAA TTAATATATT ACTATACATC ACAAAATGAA   
  
  
- AACGAGGCCC GTCTAACTTT TTTAAAAGTC TTTAAAAAGT TACAAAGATT TTATCTTTTG CTATGTGGTT   
  
  
- TACAGTAATA CGTTAATTTT ATTGTAAAAT TTATTTTTTG TTTTAAAAGG AGGTGAAAGT ATCATTGTAT   
  
  
- ACTAATAATG CTACACTGAA ATTAAAGCTC GGTTAACTTT TTAGAATAGT ATACAGACTC AGCACTCTAT   
  
  
- AATCTGATTG TGTGTAGTTA GGGGCTAAGG GGCGGCTTAC AACGAGGAAA CGACCGGTAT GATAAGATGC   
  
  
- GAGAAGAGTG CGAAAAACTG AAAAGGCATA AGTTTCAGCA AAAGCAGAGG GAAGAGTGAA GCAAGACGAC   
  
  
- GTACGATGGG AGAAGGAGGA GGAGGAGAGA ATATATATGG GTAGATGGGG AGCTTAAAAT TAGGGTTTTA   
  
  
- TAGAGGTTTG GGTGTAGAGA AAAGAAAGGT GACAAAAAGT GTTGAACTAC TTTATTAACT TAGTTGTTTT   
  
  
- GTAAACCTAT AACACTCTTT TTAGACTCG

+     Unnamed\_\_4

| Site Name | Organism | Position | Strand | Matrix score. | sequence | function |
| --- | --- | --- | --- | --- | --- | --- |
| Unnamed\_\_4 | Petroselinum hortense | 222 | + | 4 | CTCC |  |
| Unnamed\_\_4 | Petroselinum hortense | 1346 | + | 4 | CTCC |  |
| Unnamed\_\_4 | Petroselinum hortense | 1234 | + | 4 | CTCC |  |
| Unnamed\_\_4 | Petroselinum hortense | 984 | + | 4 | CTCC |  |
| Unnamed\_\_4 | Petroselinum hortense | 1352 | + | 4 | CTCC |  |
| Unnamed\_\_4 | Petroselinum hortense | 1307 | + | 4 | CTCC |  |
| Unnamed\_\_4 | Petroselinum hortense | 294 | + | 4 | CTCC |  |
| Unnamed\_\_4 | Petroselinum hortense | 1349 | + | 4 | CTCC |  |
| Unnamed\_\_4 | Petroselinum hortense | 1100 | + | 4 | CTCC |  |
| Unnamed\_\_4 | Petroselinum hortense | 1403 | + | 4 | CTCC |  |

>018609   
+ CTGTCATCCA CGGCGGTGCG TGGCCAGTGG GCCGATTCTG CCATTTTGTG CAAATTTTGA TATTCTAAAT   
  
  
+ ATGATTTGGT ATCCAATCGA TGTGTTTTGA TTATTAAATC CAATGTTGGT TATTTCGGTT ATTTGCATAT   
  
  
+ TATCGGAAAT GGTTTGGAAT ATGAAATTAT GAACTATGAA AGGCCAAGGG GACCCACCCA TCATGTGGCA   
  
  
+ GTACTCGTTC CCTCCGTGAC TCTAATACTA TGTAGAATCA AAGAGACGGG CCAAATACCC ACTTCCAACA   
  
  
+ ACACTGATTT TGTCTCCAAC TTGGTAATTA CCATATGTGC AATTCGTCAG GTGTGAGGTT TTATCACAAA   
  
  
+ AGGGCTCGGT ATTAGTTAGA GTGAAATTAA GATATTTAAA CTCTTCTTTT ATTAGAGATA CGGTTGATGT   
  
  
+ GGGATGTTTA ACAAGCCCCC TCATGTGGGA TCCAATTAGT GGGTCACACA TGGGGGATTC ACACATCGAC   
  
  
+ AACCACATGA AGTCAAGGGG ACTCACCTTA CACACTGGCC CAAGTGACCC ACCTTCTTCG AGCGGGGTCA   
  
  
+ AGGGGACCCG GACCCACCCA TCACATGACA CTACAGTACG AGCCCACTTC TTGGCTTCGA TACCATGTAT   
  
  
+ AATATTCAAA GAGACTGGTC AAAAATCCAC TTCCAACAAT ATTTATATTA TCCCCAACTT GGTAATTATC   
  
  
+ ACCTACACAT TCCATCAAGT GTGGAATTTT ATCATAAAAG ACATCAGTAT TAGTTAGAGT AAGATTATCA   
  
  
+ TATACTATTA CTTTTGTTTT TCTAGGACAA ATGTGGGATT CAACAATACC ACATATAATA TTATTAAATA   
  
  
+ ATTTGGTTAA GAAAAGAATA ACAAGAAAGT CCATCGTATA ATATGTACGG AACGGTACAC CACATATTAT   
  
  
+ TATACAATTG AAAAAAAGTT TTATTTTCAA GTGTTCATTT AATTATATAA TGATATGTAG TGTTTTACTT   
  
  
+ TTGCTCCGGG CAGATTGAAA AAATTTTCAG AAATTTTTCA ATGTTTCTAA AATAGAAAAC GATACACCAA   
  
  
+ ATGTCATTAT GCAATTAAAA TAACATTTTA AATAAAAAAC AAAATTTTCC TCCACTTTCA TAGTAACATA   
  
  
+ TGATTATTAC GATGTGACTT TAATTTCGAG CCAATTGAAA AATCTTATCA TATGTCTGAG TCGTGAGATA   
  
  
+ TTAGACTAAC ACACATCAAT CCCCGATTCC CCGCCGAATG TTGCTCCTTT GCTGGCCATA CTATTCTACG   
  
  
+ CTCTTCTCAC GCTTTTTGAC TTTTCCGTAT TCAAAGTCGT TTTCGTCTCC CTTCTCACTT CGTTCTGCTG   
  
  
+ CATGCTACCC TCTTCCTCCT CCTCCTCTCT TATATATACC CATCTACCCC TCGAATTTTA ATCCCAAAAT   
  
  
+ ATCTCCAAAC CCACATCTCT TTTCTTTCCA CTGTTTTTCA CAACTTGATG AAATAATTGA ATCAACAAAA   
  
  
+ CATTTGGATA TTGTGAGAAA AATCTGAGC  

- GACAGTAGGT GCCGCCACGC ACCGGTCACC CGGCTAAGAC GGTAAAACAC GTTTAAAACT ATAAGATTTA   
  
  
- TACTAAACCA TAGGTTAGCT ACACAAAACT AATAATTTAG GTTACAACCA ATAAAGCCAA TAAACGTATA   
  
  
- ATAGCCTTTA CCAAACCTTA TACTTTAATA CTTGATACTT TCCGGTTCCC CTGGGTGGGT AGTACACCGT   
  
  
- CATGAGCAAG GGAGGCACTG AGATTATGAT ACATCTTAGT TTCTCTGCCC GGTTTATGGG TGAAGGTTGT   
  
  
- TGTGACTAAA ACAGAGGTTG AACCATTAAT GGTATACACG TTAAGCAGTC CACACTCCAA AATAGTGTTT   
  
  
- TCCCGAGCCA TAATCAATCT CACTTTAATT CTATAAATTT GAGAAGAAAA TAATCTCTAT GCCAACTACA   
  
  
- CCCTACAAAT TGTTCGGGGG AGTACACCCT AGGTTAATCA CCCAGTGTGT ACCCCCTAAG TGTGTAGCTG   
  
  
- TTGGTGTACT TCAGTTCCCC TGAGTGGAAT GTGTGACCGG GTTCACTGGG TGGAAGAAGC TCGCCCCAGT   
  
  
- TCCCCTGGGC CTGGGTGGGT AGTGTACTGT GATGTCATGC TCGGGTGAAG AACCGAAGCT ATGGTACATA   
  
  
- TTATAAGTTT CTCTGACCAG TTTTTAGGTG AAGGTTGTTA TAAATATAAT AGGGGTTGAA CCATTAATAG   
  
  
- TGGATGTGTA AGGTAGTTCA CACCTTAAAA TAGTATTTTC TGTAGTCATA ATCAATCTCA TTCTAATAGT   
  
  
- ATATGATAAT GAAAACAAAA AGATCCTGTT TACACCCTAA GTTGTTATGG TGTATATTAT AATAATTTAT   
  
  
- TAAACCAATT CTTTTCTTAT TGTTCTTTCA GGTAGCATAT TATACATGCC TTGCCATGTG GTGTATAATA   
  
  
- ATATGTTAAC TTTTTTTCAA AATAAAAGTT CACAAGTAAA TTAATATATT ACTATACATC ACAAAATGAA   
  
  
- AACGAGGCCC GTCTAACTTT TTTAAAAGTC TTTAAAAAGT TACAAAGATT TTATCTTTTG CTATGTGGTT   
  
  
- TACAGTAATA CGTTAATTTT ATTGTAAAAT TTATTTTTTG TTTTAAAAGG AGGTGAAAGT ATCATTGTAT   
  
  
- ACTAATAATG CTACACTGAA ATTAAAGCTC GGTTAACTTT TTAGAATAGT ATACAGACTC AGCACTCTAT   
  
  
- AATCTGATTG TGTGTAGTTA GGGGCTAAGG GGCGGCTTAC AACGAGGAAA CGACCGGTAT GATAAGATGC   
  
  
- GAGAAGAGTG CGAAAAACTG AAAAGGCATA AGTTTCAGCA AAAGCAGAGG GAAGAGTGAA GCAAGACGAC   
  
  
- GTACGATGGG AGAAGGAGGA GGAGGAGAGA ATATATATGG GTAGATGGGG AGCTTAAAAT TAGGGTTTTA   
  
  
- TAGAGGTTTG GGTGTAGAGA AAAGAAAGGT GACAAAAAGT GTTGAACTAC TTTATTAACT TAGTTGTTTT   
  
  
- GTAAACCTAT AACACTCTTT TTAGACTCG

+     Unnamed\_\_6

| Site Name | Organism | Position | Strand | Matrix score. | sequence | function |
| --- | --- | --- | --- | --- | --- | --- |
| Unnamed\_\_6 | Zea mays | 380 | - | 10 | taTAAATATct |  |

>018609   
+ CTGTCATCCA CGGCGGTGCG TGGCCAGTGG GCCGATTCTG CCATTTTGTG CAAATTTTGA TATTCTAAAT   
  
  
+ ATGATTTGGT ATCCAATCGA TGTGTTTTGA TTATTAAATC CAATGTTGGT TATTTCGGTT ATTTGCATAT   
  
  
+ TATCGGAAAT GGTTTGGAAT ATGAAATTAT GAACTATGAA AGGCCAAGGG GACCCACCCA TCATGTGGCA   
  
  
+ GTACTCGTTC CCTCCGTGAC TCTAATACTA TGTAGAATCA AAGAGACGGG CCAAATACCC ACTTCCAACA   
  
  
+ ACACTGATTT TGTCTCCAAC TTGGTAATTA CCATATGTGC AATTCGTCAG GTGTGAGGTT TTATCACAAA   
  
  
+ AGGGCTCGGT ATTAGTTAGA GTGAAATTAA GATATTTAAA CTCTTCTTTT ATTAGAGATA CGGTTGATGT   
  
  
+ GGGATGTTTA ACAAGCCCCC TCATGTGGGA TCCAATTAGT GGGTCACACA TGGGGGATTC ACACATCGAC   
  
  
+ AACCACATGA AGTCAAGGGG ACTCACCTTA CACACTGGCC CAAGTGACCC ACCTTCTTCG AGCGGGGTCA   
  
  
+ AGGGGACCCG GACCCACCCA TCACATGACA CTACAGTACG AGCCCACTTC TTGGCTTCGA TACCATGTAT   
  
  
+ AATATTCAAA GAGACTGGTC AAAAATCCAC TTCCAACAAT ATTTATATTA TCCCCAACTT GGTAATTATC   
  
  
+ ACCTACACAT TCCATCAAGT GTGGAATTTT ATCATAAAAG ACATCAGTAT TAGTTAGAGT AAGATTATCA   
  
  
+ TATACTATTA CTTTTGTTTT TCTAGGACAA ATGTGGGATT CAACAATACC ACATATAATA TTATTAAATA   
  
  
+ ATTTGGTTAA GAAAAGAATA ACAAGAAAGT CCATCGTATA ATATGTACGG AACGGTACAC CACATATTAT   
  
  
+ TATACAATTG AAAAAAAGTT TTATTTTCAA GTGTTCATTT AATTATATAA TGATATGTAG TGTTTTACTT   
  
  
+ TTGCTCCGGG CAGATTGAAA AAATTTTCAG AAATTTTTCA ATGTTTCTAA AATAGAAAAC GATACACCAA   
  
  
+ ATGTCATTAT GCAATTAAAA TAACATTTTA AATAAAAAAC AAAATTTTCC TCCACTTTCA TAGTAACATA   
  
  
+ TGATTATTAC GATGTGACTT TAATTTCGAG CCAATTGAAA AATCTTATCA TATGTCTGAG TCGTGAGATA   
  
  
+ TTAGACTAAC ACACATCAAT CCCCGATTCC CCGCCGAATG TTGCTCCTTT GCTGGCCATA CTATTCTACG   
  
  
+ CTCTTCTCAC GCTTTTTGAC TTTTCCGTAT TCAAAGTCGT TTTCGTCTCC CTTCTCACTT CGTTCTGCTG   
  
  
+ CATGCTACCC TCTTCCTCCT CCTCCTCTCT TATATATACC CATCTACCCC TCGAATTTTA ATCCCAAAAT   
  
  
+ ATCTCCAAAC CCACATCTCT TTTCTTTCCA CTGTTTTTCA CAACTTGATG AAATAATTGA ATCAACAAAA   
  
  
+ CATTTGGATA TTGTGAGAAA AATCTGAGC  

- GACAGTAGGT GCCGCCACGC ACCGGTCACC CGGCTAAGAC GGTAAAACAC GTTTAAAACT ATAAGATTTA   
  
  
- TACTAAACCA TAGGTTAGCT ACACAAAACT AATAATTTAG GTTACAACCA ATAAAGCCAA TAAACGTATA   
  
  
- ATAGCCTTTA CCAAACCTTA TACTTTAATA CTTGATACTT TCCGGTTCCC CTGGGTGGGT AGTACACCGT   
  
  
- CATGAGCAAG GGAGGCACTG AGATTATGAT ACATCTTAGT TTCTCTGCCC GGTTTATGGG TGAAGGTTGT   
  
  
- TGTGACTAAA ACAGAGGTTG AACCATTAAT GGTATACACG TTAAGCAGTC CACACTCCAA AATAGTGTTT   
  
  
- TCCCGAGCCA TAATCAATCT CACTTTAATT CTATAAATTT GAGAAGAAAA TAATCTCTAT GCCAACTACA   
  
  
- CCCTACAAAT TGTTCGGGGG AGTACACCCT AGGTTAATCA CCCAGTGTGT ACCCCCTAAG TGTGTAGCTG   
  
  
- TTGGTGTACT TCAGTTCCCC TGAGTGGAAT GTGTGACCGG GTTCACTGGG TGGAAGAAGC TCGCCCCAGT   
  
  
- TCCCCTGGGC CTGGGTGGGT AGTGTACTGT GATGTCATGC TCGGGTGAAG AACCGAAGCT ATGGTACATA   
  
  
- TTATAAGTTT CTCTGACCAG TTTTTAGGTG AAGGTTGTTA TAAATATAAT AGGGGTTGAA CCATTAATAG   
  
  
- TGGATGTGTA AGGTAGTTCA CACCTTAAAA TAGTATTTTC TGTAGTCATA ATCAATCTCA TTCTAATAGT   
  
  
- ATATGATAAT GAAAACAAAA AGATCCTGTT TACACCCTAA GTTGTTATGG TGTATATTAT AATAATTTAT   
  
  
- TAAACCAATT CTTTTCTTAT TGTTCTTTCA GGTAGCATAT TATACATGCC TTGCCATGTG GTGTATAATA   
  
  
- ATATGTTAAC TTTTTTTCAA AATAAAAGTT CACAAGTAAA TTAATATATT ACTATACATC ACAAAATGAA   
  
  
- AACGAGGCCC GTCTAACTTT TTTAAAAGTC TTTAAAAAGT TACAAAGATT TTATCTTTTG CTATGTGGTT   
  
  
- TACAGTAATA CGTTAATTTT ATTGTAAAAT TTATTTTTTG TTTTAAAAGG AGGTGAAAGT ATCATTGTAT   
  
  
- ACTAATAATG CTACACTGAA ATTAAAGCTC GGTTAACTTT TTAGAATAGT ATACAGACTC AGCACTCTAT   
  
  
- AATCTGATTG TGTGTAGTTA GGGGCTAAGG GGCGGCTTAC AACGAGGAAA CGACCGGTAT GATAAGATGC   
  
  
- GAGAAGAGTG CGAAAAACTG AAAAGGCATA AGTTTCAGCA AAAGCAGAGG GAAGAGTGAA GCAAGACGAC   
  
  
- GTACGATGGG AGAAGGAGGA GGAGGAGAGA ATATATATGG GTAGATGGGG AGCTTAAAAT TAGGGTTTTA   
  
  
- TAGAGGTTTG GGTGTAGAGA AAAGAAAGGT GACAAAAAGT GTTGAACTAC TTTATTAACT TAGTTGTTTT   
  
  
- GTAAACCTAT AACACTCTTT TTAGACTCG

+     W box

| Site Name | Organism | Position | Strand | Matrix score. | sequence | function |
| --- | --- | --- | --- | --- | --- | --- |
| W box | Arabidopsis thaliana | 647 | - | 6 | TTGACC |  |
| W box | Arabidopsis thaliana | 556 | - | 6 | TTGACC |  |

>018609   
+ CTGTCATCCA CGGCGGTGCG TGGCCAGTGG GCCGATTCTG CCATTTTGTG CAAATTTTGA TATTCTAAAT   
  
  
+ ATGATTTGGT ATCCAATCGA TGTGTTTTGA TTATTAAATC CAATGTTGGT TATTTCGGTT ATTTGCATAT   
  
  
+ TATCGGAAAT GGTTTGGAAT ATGAAATTAT GAACTATGAA AGGCCAAGGG GACCCACCCA TCATGTGGCA   
  
  
+ GTACTCGTTC CCTCCGTGAC TCTAATACTA TGTAGAATCA AAGAGACGGG CCAAATACCC ACTTCCAACA   
  
  
+ ACACTGATTT TGTCTCCAAC TTGGTAATTA CCATATGTGC AATTCGTCAG GTGTGAGGTT TTATCACAAA   
  
  
+ AGGGCTCGGT ATTAGTTAGA GTGAAATTAA GATATTTAAA CTCTTCTTTT ATTAGAGATA CGGTTGATGT   
  
  
+ GGGATGTTTA ACAAGCCCCC TCATGTGGGA TCCAATTAGT GGGTCACACA TGGGGGATTC ACACATCGAC   
  
  
+ AACCACATGA AGTCAAGGGG ACTCACCTTA CACACTGGCC CAAGTGACCC ACCTTCTTCG AGCGGGGTCA   
  
  
+ AGGGGACCCG GACCCACCCA TCACATGACA CTACAGTACG AGCCCACTTC TTGGCTTCGA TACCATGTAT   
  
  
+ AATATTCAAA GAGACTGGTC AAAAATCCAC TTCCAACAAT ATTTATATTA TCCCCAACTT GGTAATTATC   
  
  
+ ACCTACACAT TCCATCAAGT GTGGAATTTT ATCATAAAAG ACATCAGTAT TAGTTAGAGT AAGATTATCA   
  
  
+ TATACTATTA CTTTTGTTTT TCTAGGACAA ATGTGGGATT CAACAATACC ACATATAATA TTATTAAATA   
  
  
+ ATTTGGTTAA GAAAAGAATA ACAAGAAAGT CCATCGTATA ATATGTACGG AACGGTACAC CACATATTAT   
  
  
+ TATACAATTG AAAAAAAGTT TTATTTTCAA GTGTTCATTT AATTATATAA TGATATGTAG TGTTTTACTT   
  
  
+ TTGCTCCGGG CAGATTGAAA AAATTTTCAG AAATTTTTCA ATGTTTCTAA AATAGAAAAC GATACACCAA   
  
  
+ ATGTCATTAT GCAATTAAAA TAACATTTTA AATAAAAAAC AAAATTTTCC TCCACTTTCA TAGTAACATA   
  
  
+ TGATTATTAC GATGTGACTT TAATTTCGAG CCAATTGAAA AATCTTATCA TATGTCTGAG TCGTGAGATA   
  
  
+ TTAGACTAAC ACACATCAAT CCCCGATTCC CCGCCGAATG TTGCTCCTTT GCTGGCCATA CTATTCTACG   
  
  
+ CTCTTCTCAC GCTTTTTGAC TTTTCCGTAT TCAAAGTCGT TTTCGTCTCC CTTCTCACTT CGTTCTGCTG   
  
  
+ CATGCTACCC TCTTCCTCCT CCTCCTCTCT TATATATACC CATCTACCCC TCGAATTTTA ATCCCAAAAT   
  
  
+ ATCTCCAAAC CCACATCTCT TTTCTTTCCA CTGTTTTTCA CAACTTGATG AAATAATTGA ATCAACAAAA   
  
  
+ CATTTGGATA TTGTGAGAAA AATCTGAGC  

- GACAGTAGGT GCCGCCACGC ACCGGTCACC CGGCTAAGAC GGTAAAACAC GTTTAAAACT ATAAGATTTA   
  
  
- TACTAAACCA TAGGTTAGCT ACACAAAACT AATAATTTAG GTTACAACCA ATAAAGCCAA TAAACGTATA   
  
  
- ATAGCCTTTA CCAAACCTTA TACTTTAATA CTTGATACTT TCCGGTTCCC CTGGGTGGGT AGTACACCGT   
  
  
- CATGAGCAAG GGAGGCACTG AGATTATGAT ACATCTTAGT TTCTCTGCCC GGTTTATGGG TGAAGGTTGT   
  
  
- TGTGACTAAA ACAGAGGTTG AACCATTAAT GGTATACACG TTAAGCAGTC CACACTCCAA AATAGTGTTT   
  
  
- TCCCGAGCCA TAATCAATCT CACTTTAATT CTATAAATTT GAGAAGAAAA TAATCTCTAT GCCAACTACA   
  
  
- CCCTACAAAT TGTTCGGGGG AGTACACCCT AGGTTAATCA CCCAGTGTGT ACCCCCTAAG TGTGTAGCTG   
  
  
- TTGGTGTACT TCAGTTCCCC TGAGTGGAAT GTGTGACCGG GTTCACTGGG TGGAAGAAGC TCGCCCCAGT   
  
  
- TCCCCTGGGC CTGGGTGGGT AGTGTACTGT GATGTCATGC TCGGGTGAAG AACCGAAGCT ATGGTACATA   
  
  
- TTATAAGTTT CTCTGACCAG TTTTTAGGTG AAGGTTGTTA TAAATATAAT AGGGGTTGAA CCATTAATAG   
  
  
- TGGATGTGTA AGGTAGTTCA CACCTTAAAA TAGTATTTTC TGTAGTCATA ATCAATCTCA TTCTAATAGT   
  
  
- ATATGATAAT GAAAACAAAA AGATCCTGTT TACACCCTAA GTTGTTATGG TGTATATTAT AATAATTTAT   
  
  
- TAAACCAATT CTTTTCTTAT TGTTCTTTCA GGTAGCATAT TATACATGCC TTGCCATGTG GTGTATAATA   
  
  
- ATATGTTAAC TTTTTTTCAA AATAAAAGTT CACAAGTAAA TTAATATATT ACTATACATC ACAAAATGAA   
  
  
- AACGAGGCCC GTCTAACTTT TTTAAAAGTC TTTAAAAAGT TACAAAGATT TTATCTTTTG CTATGTGGTT   
  
  
- TACAGTAATA CGTTAATTTT ATTGTAAAAT TTATTTTTTG TTTTAAAAGG AGGTGAAAGT ATCATTGTAT   
  
  
- ACTAATAATG CTACACTGAA ATTAAAGCTC GGTTAACTTT TTAGAATAGT ATACAGACTC AGCACTCTAT   
  
  
- AATCTGATTG TGTGTAGTTA GGGGCTAAGG GGCGGCTTAC AACGAGGAAA CGACCGGTAT GATAAGATGC   
  
  
- GAGAAGAGTG CGAAAAACTG AAAAGGCATA AGTTTCAGCA AAAGCAGAGG GAAGAGTGAA GCAAGACGAC   
  
  
- GTACGATGGG AGAAGGAGGA GGAGGAGAGA ATATATATGG GTAGATGGGG AGCTTAAAAT TAGGGTTTTA   
  
  
- TAGAGGTTTG GGTGTAGAGA AAAGAAAGGT GACAAAAAGT GTTGAACTAC TTTATTAACT TAGTTGTTTT   
  
  
- GTAAACCTAT AACACTCTTT TTAGACTCG

+     WRE3

| Site Name | Organism | Position | Strand | Matrix score. | sequence | function |
| --- | --- | --- | --- | --- | --- | --- |
| WRE3 | Pisum sativum | 539 | + | 6 | CCACCT |  |

>018609   
+ CTGTCATCCA CGGCGGTGCG TGGCCAGTGG GCCGATTCTG CCATTTTGTG CAAATTTTGA TATTCTAAAT   
  
  
+ ATGATTTGGT ATCCAATCGA TGTGTTTTGA TTATTAAATC CAATGTTGGT TATTTCGGTT ATTTGCATAT   
  
  
+ TATCGGAAAT GGTTTGGAAT ATGAAATTAT GAACTATGAA AGGCCAAGGG GACCCACCCA TCATGTGGCA   
  
  
+ GTACTCGTTC CCTCCGTGAC TCTAATACTA TGTAGAATCA AAGAGACGGG CCAAATACCC ACTTCCAACA   
  
  
+ ACACTGATTT TGTCTCCAAC TTGGTAATTA CCATATGTGC AATTCGTCAG GTGTGAGGTT TTATCACAAA   
  
  
+ AGGGCTCGGT ATTAGTTAGA GTGAAATTAA GATATTTAAA CTCTTCTTTT ATTAGAGATA CGGTTGATGT   
  
  
+ GGGATGTTTA ACAAGCCCCC TCATGTGGGA TCCAATTAGT GGGTCACACA TGGGGGATTC ACACATCGAC   
  
  
+ AACCACATGA AGTCAAGGGG ACTCACCTTA CACACTGGCC CAAGTGACCC ACCTTCTTCG AGCGGGGTCA   
  
  
+ AGGGGACCCG GACCCACCCA TCACATGACA CTACAGTACG AGCCCACTTC TTGGCTTCGA TACCATGTAT   
  
  
+ AATATTCAAA GAGACTGGTC AAAAATCCAC TTCCAACAAT ATTTATATTA TCCCCAACTT GGTAATTATC   
  
  
+ ACCTACACAT TCCATCAAGT GTGGAATTTT ATCATAAAAG ACATCAGTAT TAGTTAGAGT AAGATTATCA   
  
  
+ TATACTATTA CTTTTGTTTT TCTAGGACAA ATGTGGGATT CAACAATACC ACATATAATA TTATTAAATA   
  
  
+ ATTTGGTTAA GAAAAGAATA ACAAGAAAGT CCATCGTATA ATATGTACGG AACGGTACAC CACATATTAT   
  
  
+ TATACAATTG AAAAAAAGTT TTATTTTCAA GTGTTCATTT AATTATATAA TGATATGTAG TGTTTTACTT   
  
  
+ TTGCTCCGGG CAGATTGAAA AAATTTTCAG AAATTTTTCA ATGTTTCTAA AATAGAAAAC GATACACCAA   
  
  
+ ATGTCATTAT GCAATTAAAA TAACATTTTA AATAAAAAAC AAAATTTTCC TCCACTTTCA TAGTAACATA   
  
  
+ TGATTATTAC GATGTGACTT TAATTTCGAG CCAATTGAAA AATCTTATCA TATGTCTGAG TCGTGAGATA   
  
  
+ TTAGACTAAC ACACATCAAT CCCCGATTCC CCGCCGAATG TTGCTCCTTT GCTGGCCATA CTATTCTACG   
  
  
+ CTCTTCTCAC GCTTTTTGAC TTTTCCGTAT TCAAAGTCGT TTTCGTCTCC CTTCTCACTT CGTTCTGCTG   
  
  
+ CATGCTACCC TCTTCCTCCT CCTCCTCTCT TATATATACC CATCTACCCC TCGAATTTTA ATCCCAAAAT   
  
  
+ ATCTCCAAAC CCACATCTCT TTTCTTTCCA CTGTTTTTCA CAACTTGATG AAATAATTGA ATCAACAAAA   
  
  
+ CATTTGGATA TTGTGAGAAA AATCTGAGC  

- GACAGTAGGT GCCGCCACGC ACCGGTCACC CGGCTAAGAC GGTAAAACAC GTTTAAAACT ATAAGATTTA   
  
  
- TACTAAACCA TAGGTTAGCT ACACAAAACT AATAATTTAG GTTACAACCA ATAAAGCCAA TAAACGTATA   
  
  
- ATAGCCTTTA CCAAACCTTA TACTTTAATA CTTGATACTT TCCGGTTCCC CTGGGTGGGT AGTACACCGT   
  
  
- CATGAGCAAG GGAGGCACTG AGATTATGAT ACATCTTAGT TTCTCTGCCC GGTTTATGGG TGAAGGTTGT   
  
  
- TGTGACTAAA ACAGAGGTTG AACCATTAAT GGTATACACG TTAAGCAGTC CACACTCCAA AATAGTGTTT   
  
  
- TCCCGAGCCA TAATCAATCT CACTTTAATT CTATAAATTT GAGAAGAAAA TAATCTCTAT GCCAACTACA   
  
  
- CCCTACAAAT TGTTCGGGGG AGTACACCCT AGGTTAATCA CCCAGTGTGT ACCCCCTAAG TGTGTAGCTG   
  
  
- TTGGTGTACT TCAGTTCCCC TGAGTGGAAT GTGTGACCGG GTTCACTGGG TGGAAGAAGC TCGCCCCAGT   
  
  
- TCCCCTGGGC CTGGGTGGGT AGTGTACTGT GATGTCATGC TCGGGTGAAG AACCGAAGCT ATGGTACATA   
  
  
- TTATAAGTTT CTCTGACCAG TTTTTAGGTG AAGGTTGTTA TAAATATAAT AGGGGTTGAA CCATTAATAG   
  
  
- TGGATGTGTA AGGTAGTTCA CACCTTAAAA TAGTATTTTC TGTAGTCATA ATCAATCTCA TTCTAATAGT   
  
  
- ATATGATAAT GAAAACAAAA AGATCCTGTT TACACCCTAA GTTGTTATGG TGTATATTAT AATAATTTAT   
  
  
- TAAACCAATT CTTTTCTTAT TGTTCTTTCA GGTAGCATAT TATACATGCC TTGCCATGTG GTGTATAATA   
  
  
- ATATGTTAAC TTTTTTTCAA AATAAAAGTT CACAAGTAAA TTAATATATT ACTATACATC ACAAAATGAA   
  
  
- AACGAGGCCC GTCTAACTTT TTTAAAAGTC TTTAAAAAGT TACAAAGATT TTATCTTTTG CTATGTGGTT   
  
  
- TACAGTAATA CGTTAATTTT ATTGTAAAAT TTATTTTTTG TTTTAAAAGG AGGTGAAAGT ATCATTGTAT   
  
  
- ACTAATAATG CTACACTGAA ATTAAAGCTC GGTTAACTTT TTAGAATAGT ATACAGACTC AGCACTCTAT   
  
  
- AATCTGATTG TGTGTAGTTA GGGGCTAAGG GGCGGCTTAC AACGAGGAAA CGACCGGTAT GATAAGATGC   
  
  
- GAGAAGAGTG CGAAAAACTG AAAAGGCATA AGTTTCAGCA AAAGCAGAGG GAAGAGTGAA GCAAGACGAC   
  
  
- GTACGATGGG AGAAGGAGGA GGAGGAGAGA ATATATATGG GTAGATGGGG AGCTTAAAAT TAGGGTTTTA   
  
  
- TAGAGGTTTG GGTGTAGAGA AAAGAAAGGT GACAAAAAGT GTTGAACTAC TTTATTAACT TAGTTGTTTT   
  
  
- GTAAACCTAT AACACTCTTT TTAGACTCG

+     as-1

| Site Name | Organism | Position | Strand | Matrix score. | sequence | function |
| --- | --- | --- | --- | --- | --- | --- |
| as-1 | Arabidopsis thaliana | 325 | - | 5 | TGACG |  |

>018609   
+ CTGTCATCCA CGGCGGTGCG TGGCCAGTGG GCCGATTCTG CCATTTTGTG CAAATTTTGA TATTCTAAAT   
  
  
+ ATGATTTGGT ATCCAATCGA TGTGTTTTGA TTATTAAATC CAATGTTGGT TATTTCGGTT ATTTGCATAT   
  
  
+ TATCGGAAAT GGTTTGGAAT ATGAAATTAT GAACTATGAA AGGCCAAGGG GACCCACCCA TCATGTGGCA   
  
  
+ GTACTCGTTC CCTCCGTGAC TCTAATACTA TGTAGAATCA AAGAGACGGG CCAAATACCC ACTTCCAACA   
  
  
+ ACACTGATTT TGTCTCCAAC TTGGTAATTA CCATATGTGC AATTCGTCAG GTGTGAGGTT TTATCACAAA   
  
  
+ AGGGCTCGGT ATTAGTTAGA GTGAAATTAA GATATTTAAA CTCTTCTTTT ATTAGAGATA CGGTTGATGT   
  
  
+ GGGATGTTTA ACAAGCCCCC TCATGTGGGA TCCAATTAGT GGGTCACACA TGGGGGATTC ACACATCGAC   
  
  
+ AACCACATGA AGTCAAGGGG ACTCACCTTA CACACTGGCC CAAGTGACCC ACCTTCTTCG AGCGGGGTCA   
  
  
+ AGGGGACCCG GACCCACCCA TCACATGACA CTACAGTACG AGCCCACTTC TTGGCTTCGA TACCATGTAT   
  
  
+ AATATTCAAA GAGACTGGTC AAAAATCCAC TTCCAACAAT ATTTATATTA TCCCCAACTT GGTAATTATC   
  
  
+ ACCTACACAT TCCATCAAGT GTGGAATTTT ATCATAAAAG ACATCAGTAT TAGTTAGAGT AAGATTATCA   
  
  
+ TATACTATTA CTTTTGTTTT TCTAGGACAA ATGTGGGATT CAACAATACC ACATATAATA TTATTAAATA   
  
  
+ ATTTGGTTAA GAAAAGAATA ACAAGAAAGT CCATCGTATA ATATGTACGG AACGGTACAC CACATATTAT   
  
  
+ TATACAATTG AAAAAAAGTT TTATTTTCAA GTGTTCATTT AATTATATAA TGATATGTAG TGTTTTACTT   
  
  
+ TTGCTCCGGG CAGATTGAAA AAATTTTCAG AAATTTTTCA ATGTTTCTAA AATAGAAAAC GATACACCAA   
  
  
+ ATGTCATTAT GCAATTAAAA TAACATTTTA AATAAAAAAC AAAATTTTCC TCCACTTTCA TAGTAACATA   
  
  
+ TGATTATTAC GATGTGACTT TAATTTCGAG CCAATTGAAA AATCTTATCA TATGTCTGAG TCGTGAGATA   
  
  
+ TTAGACTAAC ACACATCAAT CCCCGATTCC CCGCCGAATG TTGCTCCTTT GCTGGCCATA CTATTCTACG   
  
  
+ CTCTTCTCAC GCTTTTTGAC TTTTCCGTAT TCAAAGTCGT TTTCGTCTCC CTTCTCACTT CGTTCTGCTG   
  
  
+ CATGCTACCC TCTTCCTCCT CCTCCTCTCT TATATATACC CATCTACCCC TCGAATTTTA ATCCCAAAAT   
  
  
+ ATCTCCAAAC CCACATCTCT TTTCTTTCCA CTGTTTTTCA CAACTTGATG AAATAATTGA ATCAACAAAA   
  
  
+ CATTTGGATA TTGTGAGAAA AATCTGAGC  

- GACAGTAGGT GCCGCCACGC ACCGGTCACC CGGCTAAGAC GGTAAAACAC GTTTAAAACT ATAAGATTTA   
  
  
- TACTAAACCA TAGGTTAGCT ACACAAAACT AATAATTTAG GTTACAACCA ATAAAGCCAA TAAACGTATA   
  
  
- ATAGCCTTTA CCAAACCTTA TACTTTAATA CTTGATACTT TCCGGTTCCC CTGGGTGGGT AGTACACCGT   
  
  
- CATGAGCAAG GGAGGCACTG AGATTATGAT ACATCTTAGT TTCTCTGCCC GGTTTATGGG TGAAGGTTGT   
  
  
- TGTGACTAAA ACAGAGGTTG AACCATTAAT GGTATACACG TTAAGCAGTC CACACTCCAA AATAGTGTTT   
  
  
- TCCCGAGCCA TAATCAATCT CACTTTAATT CTATAAATTT GAGAAGAAAA TAATCTCTAT GCCAACTACA   
  
  
- CCCTACAAAT TGTTCGGGGG AGTACACCCT AGGTTAATCA CCCAGTGTGT ACCCCCTAAG TGTGTAGCTG   
  
  
- TTGGTGTACT TCAGTTCCCC TGAGTGGAAT GTGTGACCGG GTTCACTGGG TGGAAGAAGC TCGCCCCAGT   
  
  
- TCCCCTGGGC CTGGGTGGGT AGTGTACTGT GATGTCATGC TCGGGTGAAG AACCGAAGCT ATGGTACATA   
  
  
- TTATAAGTTT CTCTGACCAG TTTTTAGGTG AAGGTTGTTA TAAATATAAT AGGGGTTGAA CCATTAATAG   
  
  
- TGGATGTGTA AGGTAGTTCA CACCTTAAAA TAGTATTTTC TGTAGTCATA ATCAATCTCA TTCTAATAGT   
  
  
- ATATGATAAT GAAAACAAAA AGATCCTGTT TACACCCTAA GTTGTTATGG TGTATATTAT AATAATTTAT   
  
  
- TAAACCAATT CTTTTCTTAT TGTTCTTTCA GGTAGCATAT TATACATGCC TTGCCATGTG GTGTATAATA   
  
  
- ATATGTTAAC TTTTTTTCAA AATAAAAGTT CACAAGTAAA TTAATATATT ACTATACATC ACAAAATGAA   
  
  
- AACGAGGCCC GTCTAACTTT TTTAAAAGTC TTTAAAAAGT TACAAAGATT TTATCTTTTG CTATGTGGTT   
  
  
- TACAGTAATA CGTTAATTTT ATTGTAAAAT TTATTTTTTG TTTTAAAAGG AGGTGAAAGT ATCATTGTAT   
  
  
- ACTAATAATG CTACACTGAA ATTAAAGCTC GGTTAACTTT TTAGAATAGT ATACAGACTC AGCACTCTAT   
  
  
- AATCTGATTG TGTGTAGTTA GGGGCTAAGG GGCGGCTTAC AACGAGGAAA CGACCGGTAT GATAAGATGC   
  
  
- GAGAAGAGTG CGAAAAACTG AAAAGGCATA AGTTTCAGCA AAAGCAGAGG GAAGAGTGAA GCAAGACGAC   
  
  
- GTACGATGGG AGAAGGAGGA GGAGGAGAGA ATATATATGG GTAGATGGGG AGCTTAAAAT TAGGGTTTTA   
  
  
- TAGAGGTTTG GGTGTAGAGA AAAGAAAGGT GACAAAAAGT GTTGAACTAC TTTATTAACT TAGTTGTTTT   
  
  
- GTAAACCTAT AACACTCTTT TTAGACTCG
